# Supplementary material for: Highly pathogenic avian influenza virus of the A/H5N8 subtype, clade 2.3.4.4b, caused outbreaks in Kazakhstan in 2020
Source: PeerJ. 2022 Mar 2;10:e13038. doi: 10.7717/peerj.13038 (PMC8898005; doi:10.7717/peerj.13038)
Supplement: Supplemental Information 1 [file peerj-10-13038-s001.docx]

Supplementary Information to

**Highly pathogenic avian influenza virus of the A/H5N8 subtype, clade 2.3.4.4b, caused outbreaks in Kazakhstan in 2020**

Asylulan Amirgazin^1^, Alexandr Shevtsov^1^, Talgat Karibayev^2^, Maxat Berdikulov^2^, Tamila Kozhakhmetova^2^, Laura Syzdykova^1^, Yerlan Ramankulov^1,3^, Alexandr V. Shustov ^1,*^

^1^ National Center for Biotechnology, Nur-Sultan, Kazakhstan

^2^ National Reference Veterinary Center, Nur-Sultan, Kazakhstan

^3^ National Laboratory Astana, Nazarbayev University, Nur-Sultan, Kazakhstan

***Correspondence**:

Alexandr V. Shustov, [shustov@biocenter.kz](mailto:shustov@biocenter.kz), phone: +87024735305

**A.A.**: [amirgazin@biocenter.kz](mailto:amirgazin@biocenter.kz) ORCID 0000-0001-9418-7758 Phone: +77023847557

**A.S.**: [shevtsov@biocenter.kz](mailto:shevtsov@biocenter.kz) ORCID 0000-0002-0307-1053 Phone: +77476369912

**T.Ka.**: [karibaev.v7@mail.ru](mailto:karibaev.v7@mail.ru) ORCID 0000-0003-4463-127X Phone: +77078205899

**M.B.**: [berdikulov.ma@mail.ru](mailto:berdikulov.ma@mail.ru) ORCID 0000-0003-1304-0354 Phone: +77075877287

**T.Ko.**: [tamilkyun@mail.ru](mailto:tamilkyun@mail.ru) ORCID 0000-0002-5129-8949 Phone: +77073249681

**L.S.**: [syzdykova@biocenter.kz](mailto:syzdykova@biocenter.kz) ORCID 0000-0002-8889-1615 Phone: +77750698825

**Y.R.**: [yerlan.ramankulov@nu.edu.kz](mailto:yerlan.ramankulov@nu.edu.kz) ORCID 0000-0001-6786-3028 Phone: +77770111525

**A.V.S.**: [shustov@biocenter.kz](mailto:shustov@biocenter.kz) ORCID 0000-0001-9880-9382 Phone: +77024735305

**Table S1** GISAID accession numbers for sequences of Kazakhstan A/H5N8 avian influenza viruses sequenced by the authors of this work

| GISAID Isolate ID | Isolate name | GISAID Accessions | Isolate Identifier | Place of Isolation |
| --- | --- | --- | --- | --- |
| EPI_ISL_2932600 | A/goose/Kazakhstan/4-190-20-B-H5N8-1/2020 | PB2 EPI1927648  PB1 EPI1927649  PA EPI1927650  HA EPI1882525  NP EPI1927651  NA EPI1882526  MP EPI1927652  NS EPI1927653 | A/goose/Umtyl/4-190-20-B-H5N8-1/2020 | Umtyl |
| EPI_ISL_2932608 | A/chicken/Kazakhstan/220-B-2-H5N8-4/2020 | PB2 EPI1927654  PB1 EPI1927655  PA EPI1927656  HA EPI1882547  NP EPI1927657  NA EPI1882546  MP EPI1927658  NS EPI1927659 | A/chicken/Umtyl/220-B-2-H5N8-4/2020 | Umtyl |
| EPI_ISL_2932609 | A/duck/Kazakhstan/12-20-B-Talg-11/2020 | PB2 EPI1927660  PB1 EPI1927661  PA EPI1927662  HA EPI1882548  NP EPI1927663  NA EPI1882549  MP EPI1927664  NS EPI1927665 | A/duck/Timiryazevo/12-20-B-Talg-11/2020 | Timiryazevo |
| EPI_ISL_2932612 | A/goose/Kazakhstan/7-20-B-Talg-12/2020 | PB2 EPI1927666  PB1 EPI1927667  PA EPI1927668  HA EPI1882551  NP EPI1927669  NA EPI1882550  MP EPI1927670  NS EPI1927671 | A/goose/Ayyrtausky/7-20-B-Talg-12/2020 | Ayyrtausky |
| EPI_ISL_2932614 | A/swan/Kazakhstan/9-20-B-Talg-39/2020 | PB2 EPI1927694  PB1 EPI1927695  PA EPI1927696  HA EPI1882552  NP EPI1927697  NA EPI1882553  MP EPI1927698  NS EPI1927699 | A/swan/Mamlyutka/9-20-B-Talg-39/2020 | Mamlyutka |
| EPI_ISL_2932616 | A/chicken/Kazakhstan/12-20-B-Talg-45/2020 | PB2 EPI1927700  PB1 EPI1927701  PA EPI1927702  HA EPI1882555  NP EPI1927703  NA EPI1882554  MP EPI1927704  NS EPI1927705 | A/chicken/Akzhar/12-20-B-Talg-45/2020 | Akzhar |
| EPI_ISL_2932617 | A/crow/Kazakhstan/15-20-B-Talg-4/2020 | PB2 EPI1927706  PB1 EPI1927707  PA EPI1927708  HA EPI1882556  NP EPI1927709  NA EPI1882557  MP EPI1927710  NS EPI1927711 | A/crow/Petropavlovsk/15-20-B-Talg-4/2020 | Petropavlovsk |
| EPI_ISL_2932619 | A/swan/Kazakhstan/1-267-20-B-Talg-52/2020 | PB2 EPI1927712  PB1 EPI1927713  PA EPI1927714  HA EPI1882559  NP EPI1927715  NA EPI1882558  MP EPI1927716  NS EPI1927717 | A/swan/Zhylandy/1-267-20-B-Talg-52/2020 | Zhylandy |
| EPI_ISL_2932682 | A/pigeon/Kazakhstan/15-20-B-Talg-5/2020 | PB2 EPI1927718  PB1 EPI1927719  PA EPI1927720  HA EPI1882560  NP EPI1927721  NA EPI1882561  MP EPI1927722  NS EPI1927723 | A/pigeon/Petropavlovsk/15-20-B-Talg-5/2020 | Petropavlovsk |
| EPI_ISL_2932683 | A/chicken/Kazakhstan/1-20-B-Talg-67/2020 | PB2 EPI1927724  PB1 EPI1927725  PA EPI1927726  HA EPI1882563  NP EPI1927727  NA EPI1882562  MP EPI1927728  NS EPI1927729 | A/chicken/Tayinshinsky/1-20-B-Talg-67/2020 | Tayinshinsky |

**Table S2** We gratefully acknowledge the following Authors and the Originating laboratories which submitted data to the GISAID Initiative, which data were used in the study

| Virus name | GISAID Isolate ID | Collected | Originating laboratory | Submitting laboratory | Authors |
| --- | --- | --- | --- | --- | --- |
| A/chicken/Egypt/N15173D/2018 | EPI_ISL_502280 | 2018-02-05 | NA | NA | Kandeil A., Webby R., McKenzie P., Kayali G., Ali M.A. |
| A/chicken/Egypt/ME-2018/2018 | EPI_ISL_697697 | 2018-01-01 | NA | NA | Ibrahim M., Zakaria S., Bazid A. I., Kelany W. H., El Sayed M., Ali A. |
| A/duck/Nigeria/SK28T_19VIR8424-2/2019 | EPI_ISL_503150 | 2019-06-30 | NA | NA | Shittu I., Bianco A., Gado D., Mkpuma N., Sulaiman L., Laleye A., Gobbo F., Bortolami A., Bonfante F., Vakuru C., Meseko C., Fusaro A., Shamaki D., Alabi O., Terregino C., Joannis T., Goobo F., Salviato A., Pastori A. |
| A/teal/Dakahlia/VRLCU/2019 | EPI_ISL_503935 | 2019-06-18 | NA | NA | Rohaim M. A., El Naggar R. F., Madbouly Y., AbdelSabour M. A., Munir M. |
| A/whooper swan/Mongolia/24/2020 | EPI_ISL_707015 | 2020-04-21 | NA | NA | Jeong S., Otgontogtokh N., Lee D. H., Davganyam B., Lee S. H., Cho A. Y., Tseren-Ochir E. O., Song C. S. |
| A/whooper swan/Mongolia/25/2020 | EPI_ISL_707016 | 2020-04-30 | NA | NA | Jeong S., Otgontogtokh N., Lee D. H., Davganyam B., Lee S. H., Cho A. Y., Tseren-Ochir E. O., Song C. S. |
| A/muscovy duck/China/FJFZ21/H5N6/2020 | EPI_ISL_833248 | 2020-08-12 | Institute of animal husbandry and veterinary medicine, Fujian Academy of Agricultural Sciences | Fujian Agriculture and Forestry University | NA |
| A/chicken/Vietnam/RAHO4-CD-20-421/2020 | EPI_ISL_1379443 | 2020-01-12 | Center for Veterinary Diagnostics - Regional Animal Health Office No.6 | Centers for Disease Control and Prevention | NA |
| A/Vietnam/HN31388M1/2007 | EPI_ISL_76680 | 2007-12-14 | NA | NA | Takano R. |
| A/duck/Lao/961/2010(H5N1) | EPI_ISL_95820 | 2010-03 | NA | NA | Sonnberg S., Phommachanh P., Naipospos T. S. P., McKenzie J., Chanthavisouk C., Pathammavong S., Darnell D., Meeduangchanh P., Rubrum A. M., Souriya M., Khambounheuang B., Webby R. J., Douangngeun B., Webster R. G. |
| A/Duck/Lao/567/2010(H5N1) | EPI_ISL_95823 | 2010-03 | NA | NA | Sonnberg S., Phommachanh P., Naipospos T. S. P., McKenzie J., Chanthavisouk C., Pathammavong S., Darnell D., Meeduangchanh P., Rubrum A. M., Souriya M., Khambounheuang B., Webby R. J., Douangngeun B., Webster R. G. |
| A/wigeon/Sakha/1/2014 | EPI_ISL_169427 | 2014-09-25 | State Research Center of Virology and Biotechnology (VECTOR) | State Research Center of Virology and Biotechnology (VECTOR) | Susloparov I., Kolosova N., Goncharova N., Marchenko V., Ryzhikov A. |
| A/broiler duck/Korea/Buan2/2014 | EPI_ISL_157610 | 2014-01-17 | NA | NA | Lee Y. J., Kang H. M., Lee E. K., Song B. M., Jeong J., Kwon Y. K., Kim H. R., Lee K. J., Hong M. S., Jang I., Choi K. S., Kim J. Y., Lee H. J., Kang M. S., Jeong O. M., Baek J. H., Joo Y. S., Park Y. Ho., Lee H. S., Park Y. H. |
| A/goose/Taiwan/TNO15/2015 | EPI_ISL_206484 | 2015-01-17 | NA | NA | Huang P. Y., Lee C. D., Yip C. H., Cheung C. L., Yu G., Lam T. T., Smith D. K., Zhu H., Guan Y., Lee C. C., Lam T. Y. |
| A/Sichuan/26221/2014 | EPI_ISL_163493 | 2014-04-21 | NA | WHO Chinese National Influenza Center | NA |
| A/breeder duck/Korea/Gochang1/2014 | EPI_ISL_157609 | 2014-01-16 | NA | NA | Lee Y. J., Kang H. M., Lee E. K., Song B. M., Jeong J., Kwon Y. K., Kim H. R., Lee K. J., Hong M. S., Jang I., Choi K. S., Kim J. Y., Lee H. J., Kang M. S., Jeong O. M., Baek J. H., Joo Y. S., Park Y. Ho., Lee H. S., Park Y. H. |
| A/Fujian-Sanyuan/21099/2017 | EPI_ISL_304404 | 2017-12-25 | Fujian Provincial Center for Disease Control and Prevention | WHO Chinese National Influenza Center | NA |
| A/chicken/Iraq/1/2020 | EPI_ISL_623074 | 2020-05-12 | Central Veterinary Labs | Animal and Plant Health Agency (APHA) | NA |
| A/domestic_goose/Kazakhstan/1-248_2-20-B/2020 | EPI_ISL_615068 | 2020-09-20 | National Veterinary Reference Center  Ministry of Agriculture of the Republic of Kazakhstan  let Abaya street 22/3, microdistrict Koktal-1  734067 Nur-Sultan city  Kazakhstan | Animal and Plant Health Agency (APHA)  Virology Department  Woodham Lane  New Haw, Addlestone, Surrey KT15 3NB  United Kingdom | NA |
| A/duck/Saratov/29804/2020 | EPI_ISL_654824 | 2020-09-15 | State Research Center of Virology and Biotechnology (VECTOR) | State Research Center of Virology and Biotechnology (VECTOR) | Natalia Goncharova, Ivan Susloparov, Natalia Kolosova, Alexey Danilenko, Juliya Bulanovich, Vasiliy Marchenko, Alexander Ryzhikov. |
| A/chicken/Kazakhstan/Kn-3/2020 | EPI_ISL_739686 | 2020-09-18 | Research Institute of Experimental and Clinical Medicine | WHO National Influenza Centre Russian Federation | Sobolev I., Sharshov K., Dubovitskiy N., Alekseev A., Leonov S., Irza V., Fadeev A., Danilenko D., Komissarov A., Shestopalov A. |
| A/chicken/Kazakhstan/Kn-6/2020 | EPI_ISL_739687 | 2020-09-18 | Research Institute of Experimental and Clinical Medicine | WHO National Influenza Centre Russian Federation | Sobolev I., Sharshov K., Dubovitskiy N., Alekseev A., Leonov S., Irza V., Fadeev A., Danilenko D., Komissarov A., Shestopalov A. |
| A/swan/Tumen/1479-2/2020 | EPI_ISL_661178 | 2020-09-10 | Federal Centre for Animal Health (ARRIAH) OIE Regional Reference Laboratory | Federal Centre for Animal Health (ARRIAH) | Zinyakov N., Akshalova P., Zhestkov P., Kozlov A., Andriyasov A., Ovchinnikova E., Nikonova Z., Sosipatorova V., Scherbakova L., Andreychuk D., Chvala I. |
| A/mute swan/Czech_Republic/1656-1/2021 | EPI_ISL_1180234 | 2021-01-24 | State Veterinary Institute Prague | State Veterinary Institute Prague | Nagy A, Cernikova L, Stara M. |
| A/swan/Lithuania/1298PG1_21VIR2606-3/2021 | EPI_ISL_1719910 | 2021-02-26 | Istituto Zooprofilattico Sperimentale delle Venezie, EU/OIE/Reference Laboratory and FAO Reference Centre for AI and ND | Istituto Zooprofilattico Sperimentale Delle Venezie | Pridotkas G., Jurgelevicius V., Pileviciene S., Zecchin B., Fusaro A., Milani A., Schivo A., Salviato A., Giussani E., Monne I., Terregino C. |
| A/wigeon/Latvia/23903/2021 | EPI_ISL_1420637 | 2021-02-04 | Institute of Food Safety, Animal Health and Environment "BIOR" | Institute of Food Safety, Animal Health and Environment "BIOR" | Kibilds J., Cvetkova S. |
| A/mute swan/Czech_Republic/1410-2/2021 | EPI_ISL_956368 | 2021-01-19 | State Veterinary Institute Prague | State Veterinary Institute Prague | Nagy A., Cernikova L., Stara M. |
| A/mute swan/Poland/MB189/2021 | EPI_ISL_2113167 | 2021-02-22 | National Veterinary Research Institut Poland, PIWet-PIB | National Veterinary Research Institute | Dziadek K., Swieton E., Smietanka K. |
| A/goose/Russian_Federation/Kurgan/1345-25/2020 | EPI_ISL_626651 | 2020-08-20 | Federal Centre for Animal Health (ARRIAH) | Animal and Plant Health Agency (APHA) | NA |
| A/chicken/Rostov-on-Don/308-02/2020 | EPI_ISL_1114746 | 2020-10-25 | State Research Center of Virology and Biotechnology (VECTOR) | State Research Center of Virology and Biotechnology (VECTOR) | Natalia Goncharova, Ivan Susloparov, Natalia Kolosova, Alexey Danilenko, Juliya Bulanovich, Vasiliy Marchenko, Alexander Ryzhikov. |
| A/pheasant/Wales/000252/2021 | EPI_ISL_1123355 | 2021-01-26 | Animal and Plant Health Agency (APHA) | Animal and Plant Health Agency (APHA) | NA |
| A/anser_anser/Spain/297-1_21VIR1230-5/2021 | EPI_ISL_2234812 | 2021-01-27 | Istituto Zooprofilattico Sperimentale delle Venezie, EU/OIE/Reference Laboratory and FAO Reference Centre for AI and ND | Istituto Zooprofilattico Sperimentale Delle Venezie | Ruano Ramos M. J., Zecchin B., Fusaro A., Schivo A., Salviato A., Giussani E., Monne I., Terregino C. |
| A/turkey/Stavropol/320-02/2020 | EPI_ISL_1114750 | 2020-12-11 | State Research Center of Virology and Biotechnology (VECTOR) | State Research Center of Virology and Biotechnology (VECTOR) | Natalia Goncharova, Ivan Susloparov, Natalia Kolosova, Alexey Danilenko, Juliya Bulanovich, Vasiliy Marchenko, Alexander Ryzhikov. |
| A/chicken/Czech_Republic/3531-1/2021 | EPI_ISL_1399231 | 2021-02-18 | State Veterinary Institute Prague | State Veterinary Institute Prague | Nagy A., Cernikova L., Stara M. |
| A/chicken/Krasnodar/334-03/2021 | EPI_ISL_1114763 | 2021-01-05 | State Research Center of Virology and Biotechnology (VECTOR) | State Research Center of Virology and Biotechnology (VECTOR) | Natalia Goncharova, Ivan Susloparov, Natalia Kolosova, Alexey Danilenko, Juliya Bulanovich, Vasiliy Marchenko, Alexander Ryzhikov. |
| A/mute swan/Croatia/14/2021 | EPI_ISL_1279262 | 2021-03-01 | Croatian Veterinary Institute, Poultry Centre | Croatian Veterinary Institute, Poultry Centre | Savić Vladimir |
| A/chicken/Bulgaria/50-1_21VIR1454-9/2021 | EPI_ISL_1719915 | 2021-02-08 | Istituto Zooprofilattico Sperimentale delle Venezie, EU/OIE/Reference Laboratory and FAO Reference Centre for AI and ND | Istituto Zooprofilattico Sperimentale Delle Venezie | Goujgoulova G., Slavcheva I., Zecchin B., Fusaro A., Milani A., Schivo A., Salviato A., Giussani E., Monne I.,Terregino C. |
| A/mute swan/Poland/MB272/2021 | EPI_ISL_2114013 | 2021-03-06 | National Veterinary Research Institut Poland, PIWet-PIB | National Veterinary Research Institute | Dziadek K., Swieton E., Smietanka K. |
| A/ciconia_ciconia/Spain/102-1_21VIR1230-2/2021 | EPI_ISL_2234813 | 2021-01-07 | Istituto Zooprofilattico Sperimentale delle Venezie, EU/OIE/Reference Laboratory and FAO Reference Centre for AI and ND | Istituto Zooprofilattico Sperimentale Delle Venezie | Ruano Ramos M. J., Zecchin B., Fusaro A., Schivo A., Salviato A., Giussani E., Monne I., Terregino C. |
| A/chicken/Czech Republic/4980/2021 | EPI_ISL_1697194 | 2021-03-12 | State Veterinary Institute Prague | State Veterinary Institute Prague | Nagy A., Cernikova L., Stara M. |
| A/swan/Lithuania/1258PG1_21VIR2606-2/2021 | EPI_ISL_1719909 | 2021-02-26 | Istituto Zooprofilattico Sperimentale delle Venezie, EU/OIE/Reference Laboratory and FAO Reference Centre for AI and ND | Istituto Zooprofilattico Sperimentale Delle Venezie | Pridotkas G., Jurgelevicius V., Pileviciene S., Zecchin B., Fusaro A., Milani A., Schivo A., Salviato A., Giussani E., Monne I., Terregino C. |
| A/domestic_duck/Kazakhstan/1-274-20-B/2020 | EPI_ISL_615072 | 2020-09-25 | National Veterinary Reference Center  Ministry of Agriculture of the Republic of Kazakhstan  let Abaya street 22/3, microdistrict Koktal-1  734067 Nur-Sultan city  Kazakhstan | Animal and Plant Health Agency (APHA)  Virology Department  Woodham Lane  New Haw, Addlestone, Surrey KT15 3NB  United Kingdom | NA |
| A/goose/Omsk/01171/2020 | EPI_ISL_644132 | 2020-08-17 | State Research Center of Virology and Biotechnology (VECTOR) | State Research Center of Virology and Biotechnology (VECTOR) | Natalia Goncharova, Ivan Susloparov, Natalia Kolosova, Alexey Danilenko, Juliya Bulanovich, Vasiliy Marchenko, Alexander Ryzhikov. |
| A/chicken/Astrakhan/321-01/2020 | EPI_ISL_1039231 | 2020-12-12 | State Research Center of Virology and Biotechnology (VECTOR) | State Research Center of Virology and Biotechnology (VECTOR) | NA |
| A/Astrakhan/3212/2020 | EPI_ISL_1038924 | 2020-12-12 | Center of Hygiene and Epidemiology in Astrakhan Region | Center of Hygiene and Epidemiology in Astrakhan Region | Pyankova O., Susloparov I., Marchenko V., Ryzhikov A. |
| A/domestic_goose/Kazakhstan/1-242_2-20-B/2020 | EPI_ISL_615073 | 2020-09-19 | National Veterinary Reference Center  Ministry of Agriculture of the Republic of Kazakhstan  let Abaya street 22/3, microdistrict Koktal-1  734067 Nur-Sultan city  Kazakhstan | Animal and Plant Health Agency (APHA)  Virology Department  Woodham Lane  New Haw, Addlestone, Surrey KT15 3NB  United Kingdom | NA |
| A/chicken/Omsk/0112/2020 | EPI_ISL_644150 | 2020-08-17 | State Research Center of Virology and Biotechnology (VECTOR) | State Research Center of Virology and Biotechnology (VECTOR) | Natalia Goncharova, Ivan Susloparov, Natalia Kolosova, Alexey Danilenko, Juliya Bulanovich, Vasiliy Marchenko, Alexander Ryzhikov. |
| A/chicken/Kostroma/304-06/2020 | EPI_ISL_1114741 | 2020-10-17 | State Research Center of Virology and Biotechnology (VECTOR) | State Research Center of Virology and Biotechnology (VECTOR) | Natalia Goncharova, Ivan Susloparov, Natalia Kolosova, Alexey Danilenko, Juliya Bulanovich, Vasiliy Marchenko, Alexander Ryzhikov. |
| A/turkey/Rostov-on-Don/332-09/2021 | EPI_ISL_1114757 | 2021-01-29 | State Research Center of Virology and Biotechnology (VECTOR) | State Research Center of Virology and Biotechnology (VECTOR) | Natalia Goncharova, Ivan Susloparov, Natalia Kolosova, Alexey Danilenko, Juliya Bulanovich, Vasiliy Marchenko, Alexander Ryzhikov. |
| A/whooper swan/Inner_Mongolia/W1-1/2020 | EPI_ISL_625671 | 2020-10-17 | College of Wildlife and Protected Areas, Northeast Forestry University | Northeast Forestry University | NA |
| A/mute swan/Inner_Mongolia/W2-1/2020 | EPI_ISL_625672 | 2020-10-17 | College of Wildlife and Protected Areas, Northeast Forestry University | Northeast Forestry University | NA |
| A/mallard/Korea/WA820/2020 | EPI_ISL_1009698 | 2020-11-25 | Animal and Plant Quarantine Agency (APQA) | Animal and Plant Quarantine Agency (S-2158) | NA |
| A/duck/Korea/H016/2021 | EPI_ISL_1009711 | 2021-01-06 | Animal and Plant Quarantine Agency (APQA) | Animal and Plant Quarantine Agency (S-2158) | NA |
| A/chicken/Korea/H008/2021 | EPI_ISL_1009690 | 2021-01-04 | Animal and Plant Quarantine Agency (APQA) | Animal and Plant Quarantine Agency (S-2158) | NA |
| A/crane/Kagoshima/KU-93/2021 | EPI_ISL_1098830 | 2021-01-19 | Kagoshima University | Kagoshima University | NA |
| A/mallard/Kagoshima/KU-d89/2021 | EPI_ISL_1114725 | 2021-01-16 | National Institute of Infectious Diseases (NIID) | National Institute of Infectious Diseases (NIID) | NA |
| A/chicken/Czech Republic/1566-1/2021 | EPI_ISL_977513 | 2021-01-22 | State Veterinary Institute Prague | State Veterinary Institute Prague | Nagy A., Cernikova L., Stara M. |
| A/mute swan/North_Ossetia-Alania/325-03/2020 | EPI_ISL_1114754 | 2020-12-31 | State Research Center of Virology and Biotechnology (VECTOR) | State Research Center of Virology and Biotechnology (VECTOR) | Natalia Goncharova, Ivan Susloparov, Natalia Kolosova, Alexey Danilenko, Juliya Bulanovich, Vasiliy Marchenko, Alexander Ryzhikov. |
| A/mute_swan/Kazakhstan/1-267-20-B/2020 | EPI_ISL_614401 | 2020-09-23 | National Veterinary Reference Center  Ministry of Agriculture of the Republic of Kazakhstan  let Abaya street 22/3, microdistrict Koktal-1  734067 Nur-Sultan city  Kazakhstan | Animal and Plant Health Agency (APHA)  Virology Department  Woodham Lane  New Haw, Addlestone, Surrey KT15 3NB  United Kingdom | Whittard, Elliot |
| A/goose/Omsk/0002/2020 | EPI_ISL_644122 | 2020-08-13 | State Research Center of Virology and Biotechnology (VECTOR) | State Research Center of Virology and Biotechnology (VECTOR) | Natalia Goncharova, Ivan Susloparov, Natalia Kolosova, Alexey Danilenko, Juliya Bulanovich, Vasiliy Marchenko, Alexander Ryzhikov. |
| A/goose/Omsk/30003/2020 | EPI_ISL_654827 | 2020-09-03 | State Research Center of Virology and Biotechnology (VECTOR) | State Research Center of Virology and Biotechnology (VECTOR) | Natalia Goncharova, Ivan Susloparov, Natalia Kolosova, Alexey Danilenko, Juliya Bulanovich, Vasiliy Marchenko, Alexander Ryzhikov. |
| A/goose/Omsk/30001/2020 | EPI_ISL_654826 | 2020-09-03 | State Research Center of Virology and Biotechnology (VECTOR) | State Research Center of Virology and Biotechnology (VECTOR) | Natalia Goncharova, Ivan Susloparov, Natalia Kolosova, Alexey Danilenko, Juliya Bulanovich, Vasiliy Marchenko, Alexander Ryzhikov. |
| A/duck/Omsk/0004/2020 | EPI_ISL_644149 | 2020-08-13 | State Research Center of Virology and Biotechnology (VECTOR) | State Research Center of Virology and Biotechnology (VECTOR) | Natalia Goncharova, Ivan Susloparov, Natalia Kolosova, Alexey Danilenko, Juliya Bulanovich, Vasiliy Marchenko, Alexander Ryzhikov. |
| A/duck/Chelyabinsk/1207-1/2020 | EPI_ISL_637098 | 2020-07-31 | Federal Centre for Animal Health (ARRIAH) OIE Regional Reference Laboratory | Federal Centre for Animal Health (ARRIAH) | Zinyakov N., Akshalova P., Zhestkov P., Kozlov A., Andriyasov A., Ovchinnikova E., Nikonova Z., Sosipatorova V., Scherbakova L., Andreychuk D., Chvala I. |
| A/duck/Omsk/0075/2020 | EPI_ISL_644142 | 2020-08-17 | State Research Center of Virology and Biotechnology (VECTOR) | State Research Center of Virology and Biotechnology (VECTOR) | Natalia Goncharova, Ivan Susloparov, Natalia Kolosova, Alexey Danilenko, Juliya Bulanovich, Vasiliy Marchenko, Alexander Ryzhikov. |
| A/chicken/Chelyabinsk/201/2020 | EPI_ISL_654833 | 2020-09-08 | State Research Center of Virology and Biotechnology (VECTOR) | State Research Center of Virology and Biotechnology (VECTOR) | Natalia Goncharova, Ivan Susloparov, Natalia Kolosova, Alexey Danilenko, Juliya Bulanovich, Vasiliy Marchenko, Alexander Ryzhikov. |
| A/chicken/Kurgan/1005/2020 | EPI_ISL_654837 | 2020-08-27 | State Research Center of Virology and Biotechnology (VECTOR) | State Research Center of Virology and Biotechnology (VECTOR) | Natalia Goncharova, Ivan Susloparov, Natalia Kolosova, Alexey Danilenko, Juliya Bulanovich, Vasiliy Marchenko, Alexander Ryzhikov. |
| A/chicken/Tyumen/302-02/2020 | EPI_ISL_1114736 | 2020-09-26 | State Research Center of Virology and Biotechnology (VECTOR) | State Research Center of Virology and Biotechnology (VECTOR) | Natalia Goncharova, Ivan Susloparov, Natalia Kolosova, Alexey Danilenko, Juliya Bulanovich, Vasiliy Marchenko, Alexander Ryzhikov. |
| A/muscovy_duck/Slovakia/Pah1_21VIR1086-1/2021 | EPI_ISL_1665256 | 2021-01-08 | Istituto Zooprofilattico Sperimentale delle Venezie, EU/OIE/Reference Laboratory and FAO Reference Centre for AI and ND | Istituto Zooprofilattico Sperimentale Delle Venezie | Zecchin B., Fusaro A., Pastori A., Schivo A., Salviato A., Monne I., Terregino C. |
| A/duck/Sichuan/NCXJ16/2014 | EPI_ISL_179649 | 2014-04-27 | NA | NA | Bi,Y.;  Chen,Q.;  Chen,J. |
| A/Fujian-Sanyuan/21099/2017 x PR8 (CNIC-21099) | EPI_ISL_341294 | 2017-12-25 | WHO Chinese National Influenza Center  Virology Institute, Chinese CDC  155 Changbai Road, Changping District,  Beijing 102206  China | WHO Chinese National Influenza Center  Virology Institute, Chinese CDC  155 Changbai Road, Changping District,  Beijing 102206  China | Liqi, Liu;  Dayan, Wang |
| A/Perigrine falcon/Netherlands/18003274-001/2018 | EPI_ISL_332439 | 2018-03-01 | Wageningen Bioveterinary Research  Houtribweg 39  8221 RA Lelystad  Netherlands | Wageningen Bioveterinary Research  Houtribweg 39  8222 RA Lelystad  Netherlands | Beerens, Nancy;  Heutink, Rene;  Harders, Frank;  Verschuren-Pritz, Sylvia;  Bossers, Alex;  Koch, Guus;  Bergervoet, Saskia |
| A/chicken/Washington/3490-18/2015 | EPI_ISL_179544 | 2015-01-29 | NA | NA | Killian,M.L. |
| A/gyrfalcon/Washington/41088-6/2014 | EPI_ISL_173878 | 2014-12-08 | NA | NA | Ip,H.S.;  Kim-Torchetti,M.;  Crespo,R.;  Kohrs,P.;  DeBruyn,P.;  Mansfield,K.G.;  Baszler,T.;  Badcoe,L.;  Bodenstein,B.;  Shearn-Boschler,V.;  Killian,M.L.;  Pedersen,J.C.;  Hines,N.;  Gidlewski,T.;  DeLiberto,T.;  Sleeman,J.;  Shearn-Bochsler,V.;  Long,R.L.;  Hines,N.L. |
| A/chicken/Hubei/ZYSJF38/2016 | EPI_ISL_244531 | 2016-02-21 | NA | NA | Chen,L.J.;  Tian,J.H.;  Lin,X.D.;  Liao,Y.;  Shi,M.;  Zhang,Y.Z. |
| A/duck/Hyogo/1/2016 | EPI_ISL_239351 | 2016-11-14 | Hyogo Prefectural Institute of Public Health and Consumer Sciences  2-1-29 Arata-cho Hyogo-ku Kobe-shi,  Hyogo 652-0032  Japan | National Institute of Infectious Diseases (NIID)  Gakuen 4-7-1, Musashimurajama-shi,  Tokyo 208-0011  Japan | Nakauchi,Mina;  Saito,Shinji;  Takayama,Ikuyo;  Kageyama,Tsutomu;  Odagiri,Takato |
| A/tundra swan/Niigata/5112007/2016 | EPI_ISL_300663 | 2016-12-06 | NA | NA | Soda,K.;  Usui,T.;  Ito,H.;  Ozaki,H.;  Yamaguchi,T.;  Ito,T. |
| A/chicken/Vietnam/NCVD-15A55/2015 | EPI_ISL_244516 | 2015-08-04 | NA | NA | Davis,T.;  Jang,Y. |
| A/chicken/Vietnam/NCVD-15A59/2015 | EPI_ISL_244518 | 2015-08-18 | NA | NA | Davis,T.;  Jang,Y. |
| A/duck/Wuhan/JXYFB22/2015 | EPI_ISL_205120 | 2015-01 | NA | NA | Chen,L.-J.;  Lin,X.-D.;  Guo,W.-P.;  Tian,J.-H.;  Zhang,Y.-Z. |
| A/goose/Hunan/116/2014 | EPI_ISL_255830 | 2014-11-13 | NA | Wuhan Institute of Virology  Chinese Academy of Sciences  44 Xiaohongshan,  Wuhan 430071  China | NA |
| A/Guangdong/18SF020/2018 | EPI_ISL_337274 | 2018-09-29 | Guangdong Provincial Center for Disease Control and Prevention  176 Xingang West Road, Haizhu District,  Guangzhou 510300  China | WHO Chinese National Influenza Center  Virology Institute, Chinese CDC  155 Changbai Road, Changping District,  Beijing 102206  China | Wang,Dayan;  Zhou,Shumei;  Li,Xiyan;  Liu,Jia;  Zhang,Ye;  Bo,Hong;  Shu,Yuelong |
| A/duck/Bangladesh/43127/2020 | EPI_ISL_4071515 | 2020-01-27 | NA | NA | Barman,S.;  Turner,J.C.;  Hasan,M.;  Akhtar,S.;  Franks,J.;  El-Shesheny,R.;  Walker,D.;  Seiler,P.;  Mukherjee,N.;  Kercher,L.;  McKenzie,P.;  Feeroz,M.;  Webby,R.J. |
| A/turkey/Egypt/AR550/2018 | EPI_ISL_344539 | 2018-03-28 | Poultry Diseases Department ,Faculty of Veterinary Medicine, Beni-Suef University | Friedrich-Loeffler-Institut | Hassan K. E., King J., El-Kady M. F., Abohamra S., Pohlmann A., Harder T. C. |
| A/chicken/Egypt/AR553/2018 | EPI_ISL_344541 | 2018-04-04 | Poultry Diseases Department ,Faculty of Veterinary Medicine, Beni-Suef University | Friedrich-Loeffler-Institut | Hassan K. E., King J., El-Kady M. F., Abohamra S., Pohlmann A., Harder T. C. |
| A/chicken/Egypt/AI20286/2019 | EPI_ISL_400043 | 2019-04-23 | Poultry Diseases Department ,Faculty of Veterinary Medicine, Beni-Suef University | Friedrich-Loeffler-Institut | Hassan K. E., El-Kady M. F., Harder T. |
| A/turkey/Egypt/AI20285/2019 | EPI_ISL_400027 | 2019-04-03 | Poultry Diseases Department ,Faculty of Veterinary Medicine, Beni-Suef University | Friedrich-Loeffler-Institut | Hassan K. E., El-Kady M. F., Harder T. |
| A/duck/Jiangsu/K1203/2010 | EPI_ISL_139385 | 2010-12-05 | NA | NA | Zhao K., Gu M., Zhong L., Duan,Z., Zhang Y., ZhuY., Zhao G., ZhaoM., Chen Z., Hu S., Liu W., Liu X., Peng D. |
| A/broiler duck/Korea/Buan2/2014 | EPI_ISL_157610 | 2014-01-17 | NA | NA | Lee Y. J., Kang H. M., Lee E. K., Song B. M., Jeong J., Kwon Y. K., Kim H. R., Lee K. J., Hong M. S., Jang I., Choi K. S., Kim J. Y., Lee H. J., Kang M. S., Jeong O. M., Baek J. H., Joo Y. S., Park Y. Ho., Lee H. S., Park Y. H. |
| A/goose/Taiwan/TNO15/2015 | EPI_ISL_206484 | 2015-01-17 | NA | NA | Huang P. Y., Lee C. D., Yip C. H., Cheung C. L., Yu G., Lam T. T., Smith D. K., Zhu H., Guan Y., Lee C. C., Lam T. Y. |
| A/breeder duck/Korea/Gochang1/2014 | EPI_ISL_157609 | 2014-01-16 | NA | NA | Lee Y. J., Kang H. M., Lee E. K., Song B. M., Jeong J., Kwon Y. K., Kim H. R., Lee K. J., Hong M. S., Jang I., Choi K. S., Kim J. Y., Lee H. J., Kang M. S., Jeong O. M., Baek J. H., Joo Y. S., Park Y. Ho., Lee H. S., Park Y. H. |
| A/whooper swan/Inner_Mongolia/W1-1/2020 | EPI_ISL_625671 | 2020-10-17 | College of Wildlife and Protected Areas, Northeast Forestry University | Northeast Forestry University | NA |
| A/mute swan/Inner_Mongolia/W2-1/2020 | EPI_ISL_625672 | 2020-10-17 | College of Wildlife and Protected Areas, Northeast Forestry University | Northeast Forestry University | NA |
| A/duck/Northern_China/ZGL/2020 | EPI_ISL_977582 | 2020-12-29 | South China Agricultural University Veterinary Medicine College | South China Agricultural University | Zhang Jiahao |
| A/duck/Southwestern China/B1904/2020 | EPI_ISL_977595 | 2020-12-31 | South China Agricultural University Veterinary Medicine College | South China Agricultural University | Zhang Jiahao |
| A/duck/Korea/H411/2020 | EPI_ISL_985184 | 2020-12-08 | Animal and Plant Quarantine Agency (APQA) | Animal and Plant Quarantine Agency (S-2158) | NA |
| A/duck/Korea/H431/2020 | EPI_ISL_985187 | 2020-12-10 | Animal and Plant Quarantine Agency (APQA) | Animal and Plant Quarantine Agency (S-2158) | NA |
| A/duck/Korea/H538/2020 | EPI_ISL_985211 | 2020-12-28 | Animal and Plant Quarantine Agency (APQA) | Animal and Plant Quarantine Agency (S-2158) | NA |
| A/duck/Korea/H471/2020 | EPI_ISL_985196 | 2020-12-16 | Animal and Plant Quarantine Agency (APQA) | Animal and Plant Quarantine Agency (S-2158) | NA |
| A/chicken/Korea/H510/2020 | EPI_ISL_985199 | 2020-12-22 | Animal and Plant Quarantine Agency (APQA) | Animal and Plant Quarantine Agency (S-2158) | NA |
| A/mallard/Korea/WA820/2020 | EPI_ISL_1009698 | 2020-11-25 | Animal and Plant Quarantine Agency (APQA) | Animal and Plant Quarantine Agency (S-2158) | NA |
| A/domestic_goose/Kazakhstan/1-248_2-20-B/2020 | EPI_ISL_615068 | 2020-09-20 | National Veterinary Reference Center  Ministry of Agriculture of the Republic of Kazakhstan  let Abaya street 22/3, microdistrict Koktal-1  734067 Nur-Sultan city  Kazakhstan | Animal and Plant Health Agency (APHA)  Virology Department  Woodham Lane  New Haw, Addlestone, Surrey KT15 3NB  United Kingdom | NA |
| A/mute_swan/Kazakhstan/1-267-20-B/2020 | EPI_ISL_614401 | 2020-09-23 | National Veterinary Reference Center  Ministry of Agriculture of the Republic of Kazakhstan  let Abaya street 22/3, microdistrict Koktal-1  734067 Nur-Sultan city  Kazakhstan | Animal and Plant Health Agency (APHA)  Virology Department  Woodham Lane  New Haw, Addlestone, Surrey KT15 3NB  United Kingdom | Whittard, Elliot |
| A/goose/Omsk/01161/2020 | EPI_ISL_644152 | 2020-08-17 | State Research Center of Virology and Biotechnology (VECTOR) | State Research Center of Virology and Biotechnology (VECTOR) | Natalia Goncharova, Ivan Susloparov, Natalia Kolosova, Alexey Danilenko, Juliya Bulanovich, Vasiliy Marchenko, Alexander Ryzhikov. |
| A/goose/Omsk/0002/2020 | EPI_ISL_644122 | 2020-08-13 | State Research Center of Virology and Biotechnology (VECTOR) | State Research Center of Virology and Biotechnology (VECTOR) | Natalia Goncharova, Ivan Susloparov, Natalia Kolosova, Alexey Danilenko, Juliya Bulanovich, Vasiliy Marchenko, Alexander Ryzhikov. |
| A/chicken/Omsk/30007/2020 | EPI_ISL_654830 | 2020-09-03 | State Research Center of Virology and Biotechnology (VECTOR) | State Research Center of Virology and Biotechnology (VECTOR) | Natalia Goncharova, Ivan Susloparov, Natalia Kolosova, Alexey Danilenko, Juliya Bulanovich, Vasiliy Marchenko, Alexander Ryzhikov. |
| A/goose/Omsk/30009/2020 | EPI_ISL_654831 | 2020-09-03 | State Research Center of Virology and Biotechnology (VECTOR) | State Research Center of Virology and Biotechnology (VECTOR) | Natalia Goncharova, Ivan Susloparov, Natalia Kolosova, Alexey Danilenko, Juliya Bulanovich, Vasiliy Marchenko, Alexander Ryzhikov. |
| A/domestic_duck/Kazakhstan/1-274-20-B/2020 | EPI_ISL_615072 | 2020-09-25 | National Veterinary Reference Center  Ministry of Agriculture of the Republic of Kazakhstan  let Abaya street 22/3, microdistrict Koktal-1  734067 Nur-Sultan city  Kazakhstan | Animal and Plant Health Agency (APHA)  Virology Department  Woodham Lane  New Haw, Addlestone, Surrey KT15 3NB  United Kingdom | NA |
| A/goose/Omsk/01171/2020 | EPI_ISL_644132 | 2020-08-17 | State Research Center of Virology and Biotechnology (VECTOR) | State Research Center of Virology and Biotechnology (VECTOR) | Natalia Goncharova, Ivan Susloparov, Natalia Kolosova, Alexey Danilenko, Juliya Bulanovich, Vasiliy Marchenko, Alexander Ryzhikov. |
| A/chicken/Omsk/0119/2020 | EPI_ISL_644155 | 2020-08-17 | State Research Center of Virology and Biotechnology (VECTOR) | State Research Center of Virology and Biotechnology (VECTOR) | Natalia Goncharova, Ivan Susloparov, Natalia Kolosova, Alexey Danilenko, Juliya Bulanovich, Vasiliy Marchenko, Alexander Ryzhikov. |
| A/turkey/Poland/464/2020 | EPI_ISL_779129 | 2020-12-01 | National Veterinary Research Institut Poland, PIWet-PIB | National Veterinary Research Institut Poland, PIWet-PIB | Swieton E., Smietanka K. |
| A/chicken/Astrakhan/321-01/2020 | EPI_ISL_1039231 | 2020-12-12 | State Research Center of Virology and Biotechnology (VECTOR) | State Research Center of Virology and Biotechnology (VECTOR) | Natalia Goncharova, Ivan Susloparov, Natalia Kolosova, Alexey Danilenko, Juliya Bulanovich, Vasiliy Marchenko, Alexander Ryzhikov. |
| A/Astrakhan/3212/2020 | EPI_ISL_1038924 | 2020-12-12 | Center of Hygiene and Epidemiology in Astrakhan Region | State Research Center of Virology and Biotechnology (VECTOR) | Pyankova O, Susloparov I, Marchenko V, Ryzhikov A. |
| A/chicken/Omsk/0118/2020 | EPI_ISL_644154 | 2020-08-17 | State Research Center of Virology and Biotechnology (VECTOR) | State Research Center of Virology and Biotechnology (VECTOR) | Natalia Goncharova, Ivan Susloparov, Natalia Kolosova, Alexey Danilenko, Juliya Bulanovich, Vasiliy Marchenko, Alexander Ryzhikov. |
| A/mute_swan/Slovenia/1639-20_21VIR959-1/2020 | EPI_ISL_1665263 | 2020-11-17 | Istituto Zooprofilattico Sperimentale delle Venezie, EU/OIE/Reference Laboratory and FAO Reference Centre for AI and ND | Istituto Zooprofilattico Sperimentale Delle Venezie | Slavec B., Racnik J., Zorman Rojs Olga, Zecchin B., Fusaro A., Pastori A., Schivo A., Salviato A., Monne I., Terregino C. |
| A/domestic goose/Kazakhstan/1-242_2-20-B/2020 | EPI_ISL_615073 | 2020-09-19 | National Veterinary Reference Center  Ministry of Agriculture of the Republic of Kazakhstan  let Abaya street 22/3, microdistrict Koktal-1  734067 Nur-Sultan city  Kazakhstan | Animal and Plant Health Agency (APHA)  Virology Department  Woodham Lane  New Haw, Addlestone, Surrey KT15 3NB  United Kingdom | NA |
| A/goose/Omsk/0111/2020 | EPI_ISL_644125 | 2020-08-17 | State Research Center of Virology and Biotechnology (VECTOR) | State Research Center of Virology and Biotechnology (VECTOR) | Natalia Goncharova, Ivan Susloparov, Natalia Kolosova, Alexey Danilenko, Juliya Bulanovich, Vasiliy Marchenko, Alexander Ryzhikov. |
| A/chicken/Tyumen/302-01/2020 | EPI_ISL_1114735 | 2020-09-26 | State Research Center of Virology and Biotechnology (VECTOR) | State Research Center of Virology and Biotechnology (VECTOR) | Natalia Goncharova, Ivan Susloparov, Natalia Kolosova, Alexey Danilenko, Juliya Bulanovich, Vasiliy Marchenko, Alexander Ryzhikov. |
| A/chicken/Kazakhstan/Kn-3/2020 | EPI_ISL_739686 | 2020-09-18 | Research Institute of Experimental and Clinical Medicine | WHO National Influenza Centre Russian Federation | Sobolev I., Sharshov K., Dubovitskiy N., Alekseev A., Leonov S., Irza, V., Fadeev A., Danilenko D., Komissarov A., Shestopalov A. |
| A/chicken/Kazakhstan/Kn-6/2020 | EPI_ISL_739687 | 2020-09-18 | Research Institute of Experimental and Clinical Medicine | WHO National Influenza Centre Russian Federation | Sobolev I., Sharshov K., Dubovitskiy N., Alekseev A., Leonov S., Irza, V., Fadeev A., Danilenko D., Komissarov A., Shestopalov A. |
| A/chicken/Omsk/0073/2020 | EPI_ISL_644158 | 2020-08-17 | State Research Center of Virology and Biotechnology (VECTOR)  Emerging Zoonotic Diseases and Influenza  Vector Street Build 12  630559 Koltsovo  Russian Federation | State Research Center of Virology and Biotechnology (VECTOR)  Emerging Zoonotic Diseases and Influenza  Vector Street Build 12  630559 Koltsovo  Russian Federation | Natalia,Goncharova;  Ivan,Susloparov;  Natalia,Kolosova;  Alexey,Danilenko;  Juliya,Bulanovich;  Vasiliy,Marchenko;  Alexander,Ryzhikov |
| A/duck/Omsk/0004/2020 | EPI_ISL_644149 | 2020-08-13 | State Research Center of Virology and Biotechnology (VECTOR) | State Research Center of Virology and Biotechnology (VECTOR) | Natalia Goncharova, Ivan Susloparov, Natalia Kolosova, Alexey Danilenko, Juliya Bulanovich, Vasiliy Marchenko, Alexander Ryzhikov. |
| A/chicken/Poland/474/2020 | EPI_ISL_846601 | 2020-12-03 | National Veterinary Research Institut Poland, PIWet-PIB | National Veterinary Research Institut Poland, PIWet-PIB | Swieton E., Smietanka K. |
| A/swan/Poland/MB141/2020 | EPI_ISL_846623 | 2020-12-16 | National Veterinary Research Institut Poland, PIWet-PIB | National Veterinary Research Institut Poland, PIWet-PIB | Swieton E., Smietanka K. |
| A/chicken/Rostov-on-Don/308-02/2020 | EPI_ISL_1114746 | 2020-10-25 | State Research Center of Virology and Biotechnology (VECTOR) | State Research Center of Virology and Biotechnology (VECTOR) | Natalia Goncharova, Ivan Susloparov, Natalia Kolosova, Alexey Danilenko, Juliya Bulanovich, Vasiliy Marchenko, Alexander Ryzhikov. |
