# Supplementary material for: Highly pathogenic avian influenza virus of the A/H5N8 subtype, clade 2.3.4.4b, caused outbreaks in Kazakhstan in 2020
Source: PeerJ. 2022 Mar 2;10:e13038. doi: 10.7717/peerj.13038 (PMC8898005; doi:10.7717/peerj.13038)
Supplement: Figure S1 [file peerj-10-13038-s002.docx]

**Fig.S1** Alignment of the nucleotide sequences for the hemagglutinin (HA) segment used in this study

>A_chicken_Egypt_N15173D_2018_EPI1772327

ATGGAGAACATAGTGCTTCTTCTTGCAATAGTTAGCCTTGTTAAAAGTGATCAGATTTGC

ATTGGTTACCATGCAAACAATTCGACAGAGCAAGTTGACACGATAATGGAAAAGAACGTC

ACTGTTACACATGCCCAAGACATACTGGAAAAAACACACAACGGGAAGCTCTGCGATCTA

AATGGGGTGAAGCCTCTGATTTTAAAGGATTGTAGTGTAGCTGGATGGCTCCTCGGAAAC

CCAATGTGCGACGAATTCATCAGCGTGCCGGAATGGTCTTACATAGTGGAGAGGGCTAAT

CCAGCTAATGACCTCTGTTACCCAGGGAGCCTCAATGACTATGAAGAACTGAAACACCTG

TTGAGCAGAATAAATCATTTTGAGAAGATTCTGATCATCCCCAAGAGTTCTTGGCCCAAT

CATGAAACATCATTAGGGGTGAGCGCAGCTTGTCCATACCAGGGAACACCCTCCTTTTTC

AGAAATGTGGTGTGGCTTATCAAAAAGAACGATGCATACCCAACAATAAAGATAAGCTAC

AATAACACCAATCGGGAAGATCTCTTGATACTGTGGGGGATTCATCATTCCAACAATGCA

GAAGAGCAGACAAATCTCTATAAAAACCCAACCACCTATATTTCAGTTGGAACATCAACT

TTAAACCAGAGATTGGTACCAAAAATAGCTACCAGATCCCAAGTAAACGGGCAACGTGGA

AGAATGGACTTCTTCTGGACAATTTTAAAACCGAATGATGCAATCCATTTCGAGAGTAAT

GGAAATTTCATTGCTCCAGAATATGCATACAAAATTGTCAAGAAAGGGGACTCAACGATT

ATGAAAAGTGGAGTGGAATATGGCCACTGCAACACCAAATGTCAAACCCCAGTAGGAGCG

ATAAACTCTAGTATGCCATTCCACAATATACATCCTCTCACCATCGGGGAATGCCCCAAA

TACGTGAAGTCAAACAAGTTGGTCCTTGCAACTGGGCTCAGAAATAGTCCTCTAAGAGAA

AAGAGAAGAAAAAGAGGGCTGTTTGGGGCTATAGCAGGTTTTATAGAGGGAGGATGGCAG

GGAATGGTTGATGGTTGGTATGGGTACCACCATAGCAATGAGCAGGGGAGTGGGTACGCT

GCAGACAAAGAATCCACCCAAAAGGCAATAGATGGAGTTACCAATAAGGTCAACTCGATC

ATTGACAAAATGAACACTCAATTTGAGGCAGTTGGAAGGGAGTTTAATAACTTAGAAAGG

AGGATAGAGAATTTGAACAAGAAAATGGAAGACGGATTCCTAGATGTCTGGACCTATAAT

GCTGAACTTCTAGTTCTCATGGAAAACGAGAGGACTCTAGATTTCCATGACTCAAATGTC

AAGAACCTTTATGACAAAGTCAGACTGCAGCTTAGGGATAATGCAAAGGAGCTGGGTAAC

GGTTGTTTCGAATTCTATCACAAATGTGATAATGAATGTATGGAAAGTGTGAGAAATGGG

ACGTATGACTACCCTCAGTATTCAGAAGAAGCAAGATTAAAAAGAGAAGAAATAAGCGGA

GTGAAATTAGAATCAATAGGAACTTACCAAATACTGTCAATTTATTCAACAGTGGCGAGT

TCCCTAGCACTGGCAATCATGGTGGCTGGTCTATCTTTATGGATGTGCTCCAATGGGTCG

TTACAGTGCAGAATTTGCATTTAA

>A_chicken_Egypt_ME-2018_2018_EPI1818113

ATGGAGAACATAGTGCTTCTTCTTGCAATAGTTAGCCTTGTTAAAAGTGATCAGATTTGC

ATTGGTTACCATGCAAACAATTCGACAGAGCAAGTTGACACGATAATGGAAAAGAACGTC

ACTGTTACACATGCCCAAGACATACTGGAAAAAACACACAACGGGAAGCTCTGCGATCTA

AATGGGGTGAAGCCTCTGATTTTAAAGGATTGTAGTGTAGCTGGATGGCTCCTCGGGAAC

CCAATGTGCGACGAATTCATCAGAGTGCCGGAATGGTCTTACATAGTGGAGAGGGCTAAT

CCAGCTAATGACCTCTGTTACCCAGGGAGCCTCAATGACTATGAAGAACTGAAACACCTG

TTGAGCAGAATAAATCATTTTGAGAAGATTCTGATCATCCCCAAGAGTTCTTGGCCCAAT

CATGAAACATCATTAGGGGTGAGCGCAGCTTGTCCATACCAGGGAACGCCCTCCTTTTTC

AGAAATGTGGTGTGGCTTATCAAAAAGAACGATGCATACCCAACAATAAAGATAAGCTAC

AATAATACCAATCGGGAAGATCTCTTGATACTGTGGGGGATTCATCATTCCAACAATGCA

GAAGAGCAGACAAATCTCTATAAAAACCCAACCACCTATATTTCAGTTGGAACATCAACT

TTAAACCAGAGATTGGTACCAAAAATAGCTACTAGATCCCAAGTAAACGGGCAACGTGGA

AGAATGGACTTCTTCTGGACAATTTTAAAACCGAATGATGCAATCCATTTCGAGAGTAAT

GGAAATTTCATTGCTCCAGAATATGCATACAAAATTGTCAAGAAAGGGGACTCAACAATT

ATGAAAAGTGAAGTGGAATATGGCCACTGCAACACCAAATGTCAAACCCCAGTAGGAGCG

ATAAACTCTAGTATGCCATTCCACAACATACATCCTCTCACCATCGGGGAATGCCCCAAA

TACGTGAAGTCAAACAAGTTGGTCCTTGCGACTGGGCTCAGAAATAGTCCTCTAATAGAA

ACGA---GA------GGGCTGTTTGGGGCTATAGCAGGTTTTATAGAGGGAGGATGGCAG

GGAATGGTTGATGGTTGGTATGGGTACCACCATAGCAACGAGCAGGGGAGTGGGTACGCC

GCAGACAAAGAATCCACCCAAAAGGCAATAGATGGAGTTACCAATAAGGTCAACTCGATC

ATTGACAAAATGAACACTCAATTTGAGGCAGTTGGAAGGGAGTTTAATAACTTAGAAAGG

AGGATAGAGAATTTGAACAAGAAAATGGAAGACGGATTCCTAGATGTCTGGACCTATAAT

GCTGAACTTCTAGTTCTCATGGAAAACGAGAGGACTCTAGATTTCCATGACTCGAATGTC

AAGAACCTTTACGACAAAGTCAGACTGCAGCTTAGGGATAATGCAAAGGAGCTGGGTAAC

GGTTGTTTCGAGTTCTATCACAAATGTGATAATGAATGTATGGAAAGTGTGAGAAATGGG

ACGTATGACTACCCTCAGTATTCAGAAGAAGCAAGATTAAAAAGAGAAGAAATAAGCGGA

GTGAAATTAGAATCAATAGGAACTTACCAAATACTGTCAATTTATTCAACAGTGGCGAGT

TCCCTAGCACTGGCAATCATGGTGGCTGGTCTGTCTTTATGGATGTGCTCCAATGGGTCG

TTGCAGTGCAGAATTTGCATTTAA

>A_duck_Nigeria_SK28T_19VIR8424-2_2019_EPI1777107

ATGGAGAACATAGTGCTTCTTCTTGCAATAGTTAGCCTTGTTAAAAGTGATCAGATTTGC

ATTGGTTACCATGCAAACAACTCGACAGAGCAAGTTGACACGATAATGGAAAAGAACGTC

ACTGTTACACATGCCCAAGACATACTAGAAAAAACACACAACGGGAAGCTCTGCGATCTA

AATGGAGTGAAGCCTCTGATTTTAAATGATTGTAGTGTAGCTGGATGGCTCCTCGGAAAC

CCAATGTGCGACGAATTCATCAGAGTGCCGGAATGGTCTTACATAGTGGAGAGGGATAAT

CCAGCTGATGACCTCTGTTACCCAGGGAGCCTCAATGATTATGAAGAACTGAAACACCTG

TTGAGCAGAATAAATCATTTTGAGAAGATTCTGATCATCCCCAAGAGTTCTTGGCCCAAT

CATGAAACATCATTAGGGGTGAGTGCAGCTTGTCCATACCAGGGGACGCCCTCCTTTTTC

AGAAACGTGGTATGGCTTATCAAAANGAACGATGCATACCCCACAATAAAGATGAGCTAC

AATAATACCAATCGGGAAGATCTCTTGATACTGTGGGGNATTCATCATTCCAACAATGCA

GAAGAGCAGACAAATCTCTATAAAAACCCAACCACCTATATTTCAGTTGGAACATCAACA

TTAAACCAGAGATTGGTACCAAAAATAGCTACCAGATCCCAAGTAAACGGGCAACGTGGA

AGAATGGACTTNTTCTGGACAATTTTAAAACCGAATGATGCAATCCATTTCGAGAGTAAT

GGAAATTTCATTGCTCCAGAATATGCATACAAAATTGTCAAGAAAGGGGACTCAACAATT

ATGAAAAGTGGAGTGGAATATGGCCACTGCAACACCAAATGTCAAACCCCAGTAGGAGCG

ATAAACTCTAGTATGCCGTTCCACAATATACATCCTCTCACCATTGGGGAATGCCCCAAA

TACGTGAAGTCAAACAAGTTGGTCCTTGCGACTGGGCTTAGAAATAGTCCTCTAAGAGAA

AAGAGAAGAAAAAGAGGGCTGTTTGGGGCGATAGCAGGGTTTATAGAGGGAGGATGGCAG

GGAATGGTTGATGGTTGGTATGGCTACCACCATAGCAATGAGCAGGGGAGTGGGTACGCT

GCAGACAAAGAGTCCACCCAAAAGGCAATAGATGGAGTTACCAATAAGGTCAACTCGATC

ATTGACAAAATGAACACTCAATTTGAGGCAATTGGAAGGGAGTTTAATAACTTAGAGAGG

AGGATAGAGAATTTGAACAAGAAAATGGAAGACGGATTCCTAGATGTCTGGACCTATAAT

GCTGAACTTCTAGTTCTCATGGAAAACGAGAGGACTCTAGATTTCCATGACTCAAATGTC

AAGAACCTTTACGACAAAGTCAGACTGCAGCTTAGGGACAATGCAAAGGAGCTGGGTAAC

GGTTGTTTCGAATTCTATCACAAATGTGATAATGAATGTATGGAAAGTGTGAGAAATGGG

ACGTATGACTACCCTCAGTACTCAGAAGAAGCAAGATTAAAAAGAGAAGAAATAAGCGGA

GTTAAATTAGAATCAATAGGAACTTACCAAATACTGTCAGTTTATTCAACAGTGGCGAGT

TCCCTAGCACTGGCAATCATGGTGGCTGGTCTATCTTTGTGGATGTGCTCCAATGGGTCG

TTACAGTGCAGAATTTGCATTTAA

>A_teal_Dakahlia_VRLCU_2019_EPI1779898

ATGGAGAACATAGTGCTTCTTCTTGCAATAGTTAGCCTTGTTAAAAGTGATCAGATTTGC

ATTGGTTACCATGCAAACAACTCGACAGAGCAAGTTGACACGATAATGGAAAAGAACGTC

ACTGTTACACATGCCCAAGACATACTGGAAAAAACACACAACGGGAAGCTCTGCGATCTA

AATGGGGTGAAACCTCTGATTTTAAAGGATTGTAGTGTAGCTGGATGGCTCCTCGGAAAC

CCAATGTGCGACGAATTCATCAGAGTGCCGGAATGGTCTTACATAGTGGAGAGGGCTAAT

CCAGCTAATGACCTCTGTTACCCAGGGAGCCTCAATGACTATGAAGAACTGAAACACCTT

TTGAGCAGAATAAATCATTTTGAGAAGATTCTGATCATCCCCAAGAGCTCTTGGCCCAAT

CATGAAACATCATTAGGGGTGAGCGCAGCTTGTCCATACCAGGGAACGCCCTCCTTTTTC

AGAAATGTGGTATGGCTTATCAAAAAGAACGATGCATATCCAACAATAAAGATAAGCTAC

AATAATACCAATCGGGAAGATCTCTTGATACTGTGGGGAATCCATCATTCCAACAATGCA

GAAGAGCAGACAAATCTCTATAAAAACCCAACCACCTATATTTCAGTTGGAACATCAACA

TTAAACCAGAGATTGGTACCAAAAATAGCTACTAGATCCCAAGTAAACGGGCAACGTGGA

AGAATGGACTTCTTCTGGACAATTTTAAAACCGAATGATGCAATCCATTTCGAGAGTAAT

GGAAATTTCATTGCTCCAGAATATGCATACAAAATTGTCAAGAAAGGGGACTCAACAATT

ATGAAAAGTGGAGTGGAATATGGCCACTGCAACACCAAATGTCAAACCCCAGTAGGAGCG

ATAAACTCTAGTATGCCATTCCACAATATACATCCTCTCACCATCGGGGAATGCCCCAAA

TACGTGAAGTCAAACAAGTTGGTCCTTGCGACTGGGCTCAGAAATAGTCCTCTAAGAGAG

AAGAGAAGAAAAAGAGGGCTGTTTGGGGCTATAGCAGGTTTTATAGAGGGAGGATGGCAG

GGAATGGTTGATGGTTGGTATGGGTACCACCATAGCAATGAGCAGGGGAGTGGGTACGCT

GCAGACAAAGAATCCACCCAAAAGGCAATAGATGGAGTTACCAATAAGGTCAACTCAATC

ATTGACAAAATGAACACTCAATTTGAGGCAGTTGGAAGGGAGTTTAATAACTTGGAAAGG

AGGATAGAAAATTTGAACAAGAAAATGGAAGACGGATTCCTAGATGTATGGACCTATAAT

GCTGAACTTCTAGTTCTCATGGAAAACGAGAGGACTCTAGATTTCCATGACTCAAATGTC

AAGAACCTTTACGACAAAGTCAGACTGCAGCTTAGGGATAATGCAAAGGAGCTGGGTAAC

GGTTGTTTCGAATTCTATCACAAATGTGATAATGAATGTATGGAAAGTGTGAGAAATGGG

ACGTATGACTACCCTCAGTATTCAGAAGAAGCAAGATTAAAAAGAGGAGAAATAAGCGGA

GTGAAATTAGAATCAATAGGAACTTACCAAATACTGTCAATTTATTCAACAGTGGCGAGT

TCCCTAGCACTGGCAATCATGGTGGCTGGTCTGTCTTTATGGATGTGCTCCAATGGGTCG

TTACAGTGCAGAATTTGCATTTAA

>A_whooper_swan_Mongolia_24_2020_EPI1831864

ATGGAGAAAATAGTACTTCTTTTTTCAGTGGTTAGCCTTGTTAAAAGTGATCAGATTTGC

ATTGGTTACCATGCAAACAACTCGACAGAGCAGGTTGACACAATAATGGAAAAAAACGTC

ACTGTTACGCATGCCCAAGACATACTGGAAAAGACACACAACGGGAAGCTCTGCGATCTG

AATGGAGTAAAACCACTGATTTTAAAGGATTGTAGTGTAGCTGGATGGCTTCTTGGAAAC

CCAATGTGCGACGAGTTCATCAGTGTGCCGGAATGGTCTTATATAGTGGAGAGGGCTAAC

CCAGCCAATGACCTCTGTTACCCGGGGAACCTCAATGACTATGAAGAGCTGAAACACCTA

TTGAGCAGAATAAATCATTTTGAGAAGACTCAGATCATCCCCAAGAGGTCTTGGTCCAAT

CATACATCATC---AGGGGTGAGCGCAGCATGTCCATACCAAGGAGTGGCCTCCTTTTTT

AGAAATGTGGTATGGCTTACCAAGAAGAATGATGCATACCCGACAATAAAGATGAGCTAC

AATAATACCAATAAAGAAGATCTTTTGATACTGTGGGGAATCCATCATTCCAACAGTGCA

GAGGAGCAGATAAGTCTCTACAAGAACCCAACCACCTATGTTTCCGTTGGGACATCAACA

TTAAACCAGAGGTTGGTGCCAAAAATAGCTACTAGATCCCAAGTAAATGGGCAACGTGGA

AGAATGGATTTCTTCTGGACAATTTTAAGACCGAGTGATGCAATCCACTTCGAGAGTAAT

GGAAATTTTATTGCTCCAGAATATGCATACAAAATTATCAAGACAGGAGACTCAACAATT

ATGAAAAGTGAATTAGAATATGGCAACTGCAACACCAAGTGTCAAACTCCAATAGGGGCG

ATAAACTCTAGTATGCCATTCCACAATATACATCCTCTCACCATCGGGGAGTGCCCCAAA

TATGTGAAATCAAACAAATTAGTCCTCGCGACTGGGCTCAGAAATAGTCCTCTAAGAGAA

AGAAGAAGAAAAAGAGGACTGTTTGGAGCCATAGCAGGGTTTATAGAGGGAGGATGGCAA

GGAATGGTAGATGGTTGGTATGGGTACCACCATAGTAATGAGCAGGGGAGTGGATACGCT

GCAGACAGAGAATCCACTCAAAAGGCAATAGATGGAGTCACCAACAAGGTCAATTCAATA

ATTGACAAAATGAACACTCAATTTGAGGCCGTTGGAAGAGAATTTAACAGCTTAGAACGG

AGAATAGAGAATTTAAACAAGAAAATGGAAGACGGATTCCTAGATGTCTGGACTTATAAT

GCTGAACTTTTGGTTCTCATGGAAAATGAGAGAACTCTAGATTTCCATGATTCAAATGTC

AAGAACCTTTATGACAAAGTCCGACTACAGCTTAGGGATAATGCAAAGGAGCTGGGTAAT

GGTTGTTTCGAGTTCTATCACAAATGTGATAATGAATGTATGGAAAGTGTAAGAAATGGA

ACGTATGACTACCCCCAGTACTCAGAAGAAGCAAGATTAAAAAGGGAGGAAATAAGCGGA

GTGAAATTGGAATCAATAGGAACTTACCAAATACTGTCAATTTATTCAACAGTGGCGAGT

TCCCTAGTACTGGCAATCATTGTGGCTGGTCTATCTTTATGGATGTGCTCCAATGGGTCG

TTACAATGCAGAATTTGCATTTAA

>A_whooper_swan_Mongolia_25_2020_EPI1831872

ATGGAGAAAATAGTACTTCTTTTTTCAGTGGTTAGCCTTGTTAAAAGTGATCAGATTTGC

ATTGGTTACCATGCAAACAACTCGACAGAGCAGGTTGACACAATAATGGAAAAAAACGTC

ACTGTTACGCATGCCCAAGACATACTGGAAAAGACACACAACGGGAAGCTCTGCGATCTG

AATGGAGTAAAACCACTGATTTTAAAGGATTGTAGTGTAGCTGGATGGCTTCTTGGAAAC

CCAATGTGCGACGAGTTCATCAGTGTACCGGAATGGTCTTATATAGTGGAGAGGGCTAAC

CCAGCCAATGACCTCTGTTACCCGGGGAACCTCAATGACTATGAAGAGCTGAAACACCTA

TTGAGCAGAATAAATCATTTTGAGAAGACTCAGATCATCCCCAAGAGGTCTTGGTCCAAT

CATACATCATC---AGGGGTGAGCGCAGCATGTCCATACCAAGGAGTGGCCTCCTTTTTT

AGAAATGTGGTATGGCTTACCAAGAAGAATGATGCATACCCGACAATAAAGATGAGCTAC

AATAATACCAATAAAGAAGATCTTTTGATACTGTGGGGAATCCATCATTCCAACAGTGCA

GAGGAGCAGATAAGTCTCTACAAGAACCCAACCACCTATGTTTCCGTTGGGACATCAACA

TTAAACCAGAGGTTGGTGCCAAAAATAGCTACTAGATCCCAAGTAAATGGGCAACGTGGA

AGAATGGATTTCTTCTGGACAATTTTAAGACCGAGTGATGCAATCCACTTCGAGAGTAAT

GGAAATTTTATTGCTCCAGAATATGCATACAAAATTATCAAGACAGGAGACTCAACAATT

ATGAAAAGTGAATTAGAATATGGCAACTGCAACACCAAGTGTCAAACTCCAATAGGGGCG

ATAAACTCTAGTATGCCATTCCACAATATACATCCTCTCACCATCGGGGAGTGCCCCAAA

TATGTAAAATCAAACAAATTAGTCCTTGCGACTGGGCTCAGAAATAGTCCTCTAAGAGAA

AGAAGAAGAAAAAGAGGACTGTTTGGAGCCATAGCAGGGTTTATAGAGGGAGGATGGCAA

GGAATGGTAGATGGTTGGTATGGGTACCACCATAGTAATGAGCAGGGGAGTGGATACGCT

GCAGACAGAGAATCCACCCAAAAGGCAATAGATGGAGTCACCAACAAGGTCAATTCGATA

ATTGACAAAATGAACACTCAATTTGAGGCCGTTGGAAGAGAATTTAACAGCTTAGAACGG

AGAATAGAGAATTTAAACAAGAAAATGGAAGACGGATTCCTAGATGTCTGGACTTATAAT

GCTGAACTTTTGGTTCTCATGGAAAATGAGAGAACTCTAGATTTCCATGACTCAAATGTC

AAGAACCTTTATGACAAAGTCCGACTACAGCTTAGGGATAATGCAAAGGAGCTGGGTAAT

GGTTGTTTCGAGTTCTATCACAAATGTGATAATGAATGTATGGAAAGTGTAAGAAATGGA

ACGTATGACTACCCCCAGTACTCAGAAGAAGCAAGATTAAAAAGGGAGGAAATAAGCGGA

GTGAAATTGGAATCAATAGGAACTTACCAAATACTGTCAATTTATTCAACAGTGGCGAGT

TCCCTAGTACTGGCAATCATTGTGGCTGGTCTATCTTTATGGATGTGCTCCAATGGGTCG

TTACAATGCAGAATTTGCATTTAA

>A_muscovy_duck_China_FJFZ21_H5N6_2020_EPI1841913

ATGGAGAAAATAGTACTTCTTCTCTCAGTGGTTGGCCTTGTTAAAAGTGATCAGATCTGC

ATTGGTTACCATGCAAACAACTCGACAGAGCAGGTTGACACAATAATGGAAAAAAACGTC

ACTGTTACGCATGCCCAAGACATACTGGAAAAGACACACAACGGGAAGCTCTGCGATCTG

AATGGAGTGAAACCTCTGATTTTAAAGAATTGTAGTGTGGCTGGATGGCTTCTTGGAAAC

CCAATGTGCGATGAGTTCATCAGCGTACCGGAATGGTCTTATATAGTGGAGAGGGCCAAC

CCAGCCAATGACCTCTGTTACCCAGGGAACCTCAATGACTATGAAGAACTGAAACACCTA

TTGAGCAGAATAAATCGTTTTGAGAAGACTCAGATCATCCCCAAGGAGTCTTGGTCCAAT

CATACAACATC---AGGAGTGAGCGCAGCATGTCCATACCAAGGAGTGGCCTCCTTTTTT

AGAAATGTGGTATGGCTTACCAAGAAGAATGATGCATACCCGACAATAAAGAAGAGCTAC

AATAATACCAATAAAGAGGACCTTTTGATACTATGGGGAATTCATCATTCCAACAGTGTA

GAGGAGCAGACAGATCTCTACAAGAACCCAACCACCTATGTTTCCGTTGGGACATCAACA

CTAAATCAGAGGTTGGTGCCAAAAATAGCTACCAGATCCCAAGTAAATGGGCAACGTGGA

AGAATGGATTTCTTCTGGACAATTTTAAGACCGAATGATGCAATCCACTTCGAGAGTAAT

GGAAATTTTATCGCTCCAGAATATGCATACAAAATCATCAAGACAGGAGACTCAACAATT

ATGAAAAGTGAAATAGAATATGGCGACTGCAACAGCAAGTGTCAAACTCCGATAGGGGCG

ATAAACTCTAGTATGCCATTCCACAATATACACCCTCTCACTATCGGGGAGTGCCCCAAA

TATGTGAAATCAAACAAATTAGTCCTTGCGACTGGGCTCAGAAATAGTCCTCTAAGGGAA

AGAAGAAGGAAAAGAGGACTGTTTGGAGCTATAGCAGGATTTATAGAGGGAGGATGGCAA

GGAATGGTAGATGGTTGGTATGGGTACCATCATAGTAATGAACAGGGGAGTGGGTACGCT

GCAGACAGAGAATCCACTCAAAAGGCAATAGATGGGGTCACCAACAAGGTCAACTCGATA

ATAGACAAAATGAACACTCAATTTGAGGCCGTTGGAAGAGAATTTAACAGCTTAGAACGG

AGAATAGAGAATTTAAATAAGAAAATGGAAGACGGATTCCTAGATGTCTGGACTTATAAT

GCTGAACTTTTAGTTCTCATGGAAAATGAGAGAACTCTAGATTTCCATGACTCAAATGTC

AAGAACCTTTATGACAAAGTCCGACTACAGCTTAGGGATAATGCAAAGGAGCTGGGTAAT

GGTTGTTTCGAATTCTATCACAAATGTGATAATGAATGTATGGAAAGTGTAAGAAATGGA

ACGTATGACTACCCCCAGTACTCAGAGGAGGCAAGATTAAAAAGGGAAGAAATAAGCGGA

GTGAAATTGGAAACAATAGGAACTTTCCAAATACTGTCAATTTATTCAACAGTGGCGAGT

TCCCTAGTACTGGCAATCATTATGGCTGGTCTATCTTTATGGATGTGCTCCAATGGGTCG

TTACAATGCAGAATTTGCATTTAA

>A_chicken_Vietnam_RAHO4-CD-20-421_2020_EPI1853938

ATGGAGAAAATAGTGCTTCTTCTTGCAGTGGTTAGCCTTGTCAAAAGYGATCAGATTTGC

ATTGGTTACCATGCAAATAACTCGACAGAACAGGTTGACACGATAATGGAAAAAAACGTC

ACTGTTACACATGCCCAAGACATACTAGAAAAGACACACAACGGGAGGCTCTGCGATTTG

AATGGAGTGAAACCTCTGATTTTAAAGGATTGTAGTGTAGCTGGATGGCTCCTTGGAAAC

CCTATGTGCGACGAGTTCATCAGAGTGCCGGAATGGTCCTACATAGTGGAGAGGGCTAAC

CCGCCCCACGACCTCTGTTACCCCGGGAACCTCAACGACTATGAAGAACTGAAACATCTA

TTGAGTAGAATAAATCATTTTGAGAAAACTCTGATCATCCCCAAAAGTTCTTGGCCCAAT

CATGAAACATCGTTAGGAGTGAGCGCTGCATGCCAATACCAGGGAATGCCTTCCTTTTTC

AGAAATGTGGTATGGCTCATCAAGAAGAACGATGCATACCCAACAATAGAGATGAGCTAC

AATAATACCAACAGTGAAGATCTTTTGATACTGTGGGGGATTCATCATTCTAACAACGCA

GCAGAACAAACAAATCTCTATAAAAACCCAACCACCTATGTTTCCGTTGGGACATCAACA

TTAAACCAGAGATTGGTACCCAAAATAGCTACTAGATCCCAAGTAAACGGGCAACGTGGA

AGAATGGATTTCTACTGGACAATTTTAAAACCGAATGATGCAATCCACTTCGAGAGTAAT

GGAAATTTTATTGCTCCAGAATATGCATACAAAATTGTCAAGAAAGGGGACTCAACAATC

ATGAAAAGTGAGATGGAATATGGCCGTTGCAACACCAAATGCCAAACTCCAATAGGGGCG

ATAAACTCTAGTATGCCATTCCACAATATACACCCTCTCACAATCGGGGAATGCCCCAAA

TATGTAAAATCAAACAAATTAGTACTTGCGACTGGGCTCAGAAATAGCCCCCTAAGAGAG

AGGAGAAGAAAAAGAGGACTATTTGGAGCTATAGCAGGATTTATAGAGGGAGGATGGCAA

GGAATGGTAGATGGTTGGTATGGATACCACCATAGCAATGAACAGGGAAGTGGGTACGCT

GCCGACAAAGAATCCACCCAAAAGGCAATAGATGGAGTTACCAATAAGGTCAACTCGATC

ATTGAAAAGATGAACACTCAATTTGAGGCCGTTGGGAGGGAATTTAATAACTTAGAAAGG

AGAATAGAGAATTTAAACAAGAAAATGGAAGACGGATTCCTGGATGTCTGGACTTATAAT

GCGGAACTTCTAGTTCTCATGGAAAATGAGAGAACCCTAGATTTCCATGACTCAAATGTC

AAGAACCTTTATGACAAAGTCCGACTACAGCTTAGGGACAATGCAAAGGAGCTGGGTAAT

GGTTGCTTTGAGTTCTATCACAAATGTGATAATGAATGTATGGAAAGTGTAAGAAATGGA

ACATATAACTACCCTCAGTACTCAGAAGAAGCAAGATTGAAAAGAGAAGAAATAAGCGGA

GTGAAATTGGAATCAATAGGAACTTACCAGATACTGTCAATTTATTCAACAGTGGCGAGT

TCCCTAGCACTGGCAATCATTGTGGCTGGTCTATCTTTATGGATGTGTTCCAATGGGTCA

CTACAATGCAGAATTTGCATCTAA

>A_Vietnam_HN31388M1_2007_EPI270236

ATGGAGAAAATAGTGCTTCTTCTTGCAATAATCAGCCTTGTTAAAAGTGATCAGATTTGC

ATTGGTTACCATGCAAACAACTCGACAGAGCAGGTTGACACAATAATGGAAAAGAACGTT

ACTGTTACACATGCTCAAGATATACTGGAAAAGACACACAACGGGAAGCTCTGCGATCTA

GATGGAGTGAAGCCTCTGATTTTAAGAGATTGTAGTGTAGCTGGATGGCTCCTCGGAAAC

CCGATGTGTGACGAATTCATCAATGTGCCGGAATGGTCTTACATAGTGGAGAAGGCCAAC

CCAGCCAATGACCTCTGTTACCCAGGGAATTTCAACGACTATGAAGAACTGAAGCACCTA

TTGAGCAGGATAAACCATTTTGAGAAAATTCAGATCATCCCCAAAAGTTCTTGGGTCGAT

CATGAAGCCTCATCAGGGGTGAGCTCAGCATGTCCATACCAGGGAGTGCCCTCCTTTTTC

AGAAATGTGGTATGGCTTATCAAAAAGAACAATACATACCCAACAATAAAGAGAAGCTAC

AATAATACCAACCAGGAAGATCTTTTGATACTGTGGGGGATTCATCATTCCAATGATGCA

GCAGAGCAGACAAAGCTCTATCAAAACCCAACCACCTATATTTCCGTTGGGACATCAACA

CTGAACCAGAGATTGGTACCAAAAATAGCTACTAGATCCAAAGTAAACGGGCAAAGTGGA

AGGATGGATTTCTTCTGGACAATTTTAAAACCGAATGATGCAATCAACTTCGAGAGTAAT

GGAAATTTCATTGCTCCAGAATATGCATACAAAATTGTCAAGAAAGGGGACTCGGCAATT

ATGAAAAGTGAGGTGGAGTATGGTAACTGCAACACCAAGTGTCAAACTCCAATAGGGGCG

ATAAACTCTAGTATGCCATTCCACAACATACACCCTCTCACCATTGGGGAATGCCCCAAA

TATGTGAAATCAAACAAATTAGTCCTTGCGACTGGGCTCAGAAATAGTCCTCTAAGAGAA

AGAAGAAGAAAAAGAGGACTATTTGGAGCTATAGCAGGTTTTATAGAGGGCGGATGGCAA

GGAATGGTAGATGGTTGGTATGGGTTTCACCATAGCAATGAGCAGGGGAGTGGGTACGCT

GCAGACAAAGAATCCACTCAAAAGGCAATAGATGGAGTCACCAATAAGGTCAACTCAATC

ATTGACAAAATGAACACTCAGTTTGAGGCCGTTGGAAGGGAATTTAACTACTTAGAGAGG

AGAATAGAGAATTTAAACAAGAAAATGGAAGACGGATTCCTAGATGTCTGGACTTATAAT

GCTGAACTTCTGGTTCTCATGGAAAATGAGAGAACTCTAGACTTCCATGATTCAAATGTC

AAGAACCTTTACGACAAGGTCCGACTACAGCTTAGGGATAATGCAAAGGAGCTGGGTAAC

GGTTGTTTCGAGTTCTATCACAAATGTGATAATGAATGCATGGAAAGTGTAAGAAACGGA

ACGTATGACTACCCGCAGTATTCAGAAGAAGCAAGATTAAAAAGAGAGGAAATAAGTGGA

GTAAAATTGGAATCAATAGGAACTTACCAAATACTGTCAATTTATTCAACAGTTGCGAGT

TCTCTAGCACTGGCAATCATGGTGGCTGGTCTATCTTTATGGATGTGCTCCAATGGGTCG

TTACAATGCAGAATTTGCATTTAA

>A_duck_Lao_961_2010_EPI335155

ATGGAGAAAATAGTGACTCTTCTTGCAATGGTCAGTCTTGTTAAAAGTGATCAGATTTGC

ATTGGTTACCATGCAAACAACTCGACAGAGCAGGTTGACACAATAATGGAAAAGAACGTT

ACTGTCACACATGCCCAAGATATACTGGAAAAGACACACAACGGAAAGCTCTGCGATCTA

GATGGAGTGAAGCCTCTGATTTTAAGAGATTGTAGTGTAGCTGGATGGCTCCTCGGAAAC

CCAATGTGTGACGAATTCATCAATGTGCCGGAATGGTCTTACATAGTGGAGAAAACCAAC

CCAGCCAATGACCTCTGTTACCCAGGGAATCTCAACGACTATGAAGAACTGAAACACCTA

TTGAGCAGAATAAACCATTTTGAGAAAATTCAGATCATCCCCAAAAGTTCTTGGTCCGAT

CATGAAGCCTCATCAGGAGTGAGCTCAGCATGTCCATACCAGGGAACACCCTCCTTTTTC

AGAAATGTGGTATGGCTTATCAAGAAGAACAATACATACCCAACAATAAAGAGAAGCTAC

AACAATACCAACCAGGAAGATCTTTTGATACTGTGGGGGATTCATCATTCTAATGATGAG

GCAGAGCAGATAAAGCTCTATCAAAATCCAACCACCTATATTTCCGTTGGGACATCAACA

CTAAACCAGAGATTGGTTCCAAAAATAGCTACTAGATCCAAAGTAAACGGGCAAAGTGGA

AGGATGGATTTCTTCTGGACAATTTTAAAACCGGACGATGCAATCAACTTCGAGAGTAAT

GGAAATTTCATTGCTCCAGAATATGCATACAAAATTGTCAAGAAAGGAGACTCTGCAATT

ATGAAAAGTGAAGTGGAATACGGTAATTGCAACACCAAGTGCCAAACTCCAATAGGGGCG

ATAAACTCTAGTATGCCATTCCACAACATACACCCTCTCACTATCGGGGAATGCCCCAAA

TATGTGAAATCAAACAAATTAGTCCTTGCGACTGGGCTCAGAAATAGTCCTCTAAGAGAA

AGAAGAAGAAAAAGAGGACTATTTGGAGCTATAGCAGGTTTTATAGAGGGAGGATGGCAG

GGAATGGTAGATGGTTGGTATGGGTACCATCATAGCAATGAGCAGGGGAGTGGGTACGCT

GCAGACAAAGAATCCACTCAAAAGGCAATAGATGGAGTCACCAATAAGGTCAACTCGATT

ATTGACAAAATGAACACTCAGTTTGAGGCCGTTGGAAGGGAATTTAGTAACTTAGAAAGG

AGAATAGAGAATTTAAACAAGAAAATGGAAGACGGGTTCCTAGATGTCTGGACTTATAAT

GCTGAACTTCTGGTTCTCATGGAAAATGAGAGAACTCTAGACTTCCATGACTCAAATGTC

AAGAACCTCTACGACAAGGTCCGATTACAGCTTAGGGATAATGCAAAGGAACTGGGTAAC

GGTTGTTTCGAGTTCTATCACAAATGTGATAATGAATGCATGGAAAGTGTAAGAAACGGA

ACGTATGACTACCCGCAGTATTCAGAAGAAGCAAGATTAAAAAGAGAGGAAATAAGTGGA

GTAAAATTGGAATCAATAGGAACTTACCAAATACTGTCAATTTATTCAACAGTTGCGAGT

TCTCTAGCACTGGCAATCATGATGGCTGGTCTATCTTTATGGATGTGCTCCAATGGGTCG

TTACAATGCAGAATTTGCATTTAA

>A_Duck_Lao_567_2010_EPI335179

ATGGAGAAAATAGTGCTTCTTCTTGCAATAGTCAGCCTTGTTAGAAGTGATCAGATTTGC

ATTGGTTACCATGCAAACAACTCGACAGAGCAGGTTGACACAATAATGGAAAAGAACGTT

ACTGTTACACATGCCCAAGACATACTGGAAAAGACACACAACGGGAAGCTCTGCGATCTA

GATGGAGTGAAGCCTTTGATTTTAAGAGATTGTAGTGTAGCTGGATGGCTCCTCGGAAAC

CCAATGTGTGACGAATTTATCAATGTGCCAGAATGGTCTTACATAGTGGAGAAGGCCAAC

CCAGCCAATGACCTCTGTTACCCAGGGAATTTCAATGACTATGAAGAACTGAAACACCTA

TTGAGCAGAATAAATCATTTTGAGAAAATTCAGATCATCCCCAAAAGTTCTTGGTCCGAT

CATGAAGCCTCATCAGGGGTGAGCTCAGCATGTCCGTATCAGGGAACGCCCTCTTTTTTC

AGGAATGTAGTATGGCTTATCAAAAAGAACAATACATACCCAACAATAAAGAGAAGCTAC

AATAACACCAACCAGGAAGATCTTTTGGTACTGTGGGGGATTCACCATTCTAATGATGCG

ACAGAACAGATAAAGCTCTATCAAAACCCAACCACCTATGTTTCCGTTGGAACATCAACA

CTAAACCAGAGATTGGTACCAAAAATAGCTACTAGATCCAAAGTAAACGGGCAAAGTGGA

AGGATGGATTTCTTCTGGACAATTTTAAAATCGAATGATGCAATCAACTTCGAGAGTAAT

GGGAATTTCATTGCTCCAGAATATGCATACAAAATTGTCAAGAAAGGGGACTCAGCAATT

ATGAAAAGTGAAGTGGAATATGGTAACTGCAGCACCAAGTGTCAAACTCCAATAGGGGCG

ATAAACTCTAGTATGCCATTCCACAATATACACCCTCTCACCATCGGGGAATGCCCCAAA

TATGTGAAATCAAACAAATTAGTCCTTGCGACTGGGCTCAGAAATAGTCCTCTAAGAGAG

AGAAGAAGAAAAAGAGGACTATTTGGAGCTATAGCAGGGTTTATAGAGGGAGGATGGCAG

GGAATGGTAGATGGTTGGTATGGGTACCACCATAGTAATGAGCAGGGGAGTGGGTACGCT

GCAGACAAAGAATCCACCCAAAAGGCAATAGATGGAGTCACCAATAAGGTCAACTCTATC

ATTGACAAAATGAACACTCAGTTTGAGGCCGTTGGAAGGGAATTTAATAACTTAGAAAGG

AGAATAGAGAATTTAAACAAGAAAATGGAAGACGGATTCCTAGATGTCTGGACGTATAAT

GCTGAACTTCTGGTTCTCATGGAAAATGAGAGGACTCTAGACTTCCATGACTCAAATGTT

AAGAACCTTTACGACAAGGTACGACTACAGCTGAGGGATAATGCAAAGGAGCTGGGTAAC

GGTTGCTTCGAGTTCTATCACAGATGTGATAATGAATGTATGGAAAGTGTAAGGAACGGA

ACGTATGACTACCCGCAGTATTCAGAAGAAGCAAGATTAAAAAGAGAGGAAATTAGTGGA

GTAAAATTGGAATCAATAGGAACTTACCAAATACTGTCAATTTATTCAACAGTTGCGAGT

TCACTAGTGCTGGCAATCATGGTGGCTGGTCTATCTTTGTGGATGTGCTCCAATGGGTCG

TTACAATGCAGAATTTGCATTTAA

>A_wigeon_Sakha_1_2014_EPI553349

ATGGAGAAAATAGTGCTTCTTCTTGCAGTGGTTAGCCTTGTTAAAAGTGATCAGATTTGC

ATTGGTTACCATGCAAACAACTCAACAAAACAGGTTGACACAATAATGGAAAAAAACGTC

ACTGTTACACATGCCCAAGACATACTGGAAAAGACACACAACGGGAAGCTCTGCGATCTT

AATGGAGTGAAGCCCCTGATTCTAAAGGATTGTAGCGTAGCTGGGTGGCTCCTTGGAAAT

CCAATGTGCGACGAGTTCATCAGGGTGCCGGAATGGTCTTACATCGTGGAGAGGGCTAAC

CCAGCCAACGACCTCTGTTACCCAGGGACCCTCAATGACTATGAGGAACTGAAACACCTA

CTGAGCAGAATAAATCATTTTGAGAAAACTCTGATCATCCCCAAGAGTTCTTGGCCCAAT

CATGAAACATCATTAGGGGTGAGCGCAGCATGTCCATACCAGGGAGCATCCTCATTTTTC

AGAAATGTGGTATGGCTCATCAAAAAGAACGATGCATACCCGACAATAAAGATAAGCTAC

AATAATACCAATCGGGAAGATCTTTTGATACTGTGGGGGATTCATCATTCCAACAATGCA

GAAGAGCAGACAAATCTCTATAAAAACCCAGACACTTATGTTTCCGTTGGGACATCAACA

TTAAACCAGAGATTGGTGCCAAAAATAGCTACTAGATCCCAAGTAAACGGGCAACGTGGA

AGAATGGATTTCTTCTGGACAATTTTAAAACCGAATGATGCAATCCACTTTGAGAGTAAT

GGAAATTTCATTGCTCCAGAATATGCCTACAAAATTGTCAAGAAAGGGGACTCAACAATT

ATGAAAAGTGAAGTGGAGTATGGCCACTGCAACACCAAATGTCAAACCCCAATAGGGGCG

ATAAACTCTAGCATGCCATTCCACAATATACACCCTCTCACCATCGGGGAATGCCCCAAA

TACGTGAAGTCAAACAAATTAGTCCTTGCGACTGGGCTCAGAAATAGTCCTCTAAGGGAA

AGAAGAAGAAAAAGAGGACTATTTGGAGCTATAGCAGGGTTTATAGAGGGAGGATGGCAG

GGAATGGTAGACGGTTGGTATGGGTACCACCATAGCAATGAGCAGGGGAGTGGGTACGCT

GCAGACAAAGAATCCACCCAAAAGGCAGTAGATGGAGTTACCAATAAGGTCAACTCAATC

ATTGACAAAATGAACACTCAATTTGAGGCCGTTGGAAGGGAATTTAATAACTTAGAAAGG

AGAATAGAGAATTTAAACAAGAAAATGGAAGACGGATTCCTAGATGTCTGGACTTATAAT

GCTGAACTTTTAGTTCTCATGGAAAATGAGAGAACTCTAGATTTCCATGACTCAAATGTC

AAGAACCTTTACGACAAAGTCCGACTACAGCTTAGGGATAATGCAAAAGAGCTGGGTAAT

GGTTGTTTCGAGTTCTATCACAAATGTGATAACGAATGTATGGAAAGCGTAAGAAATGGG

ACGTATGACTACCCTAAGTATTCAGAAGAAGCAAGATTAAAAAGAGAAGAAATAAGCGGA

GTGAAATTAGAATCAATAGGAACTTACCAAATACTGTCAATTTATTCAACAGTGGCGAGT

TCCCTAGCACTGGCAATCATAGTGGCTGGTCTATCTTTATGGATGTGCTCTAATGGGTCG

CTACAATGCAGAATTTGCATCTAA

>A_broiler_duck_Korea_Buan2_2014_EPI509704

ATGGAGAAAATAGTGCTTCTTCTTGCAGTGGTTAGCCTTGTTAAAAGTGATCAGATTTGC

ATTGGTTACCATGCAAACAACTCAACAAAGCAGGTTGACACGATAATGGAAAAAAACGTC

ACTGTTACACATGCCCAAGACATACTGGAAAAGACACACAACGGGAAGCTCTGCGATCTT

AATGGAGTGAAGCCCCTGATTCTAAAGGATTGTAGCGTAGCTGGGTGGCTCCTTGGAAAT

CCAATGTGCGACGAGTTCATCAGGGTGCCGGAATGGTCTTACATCGTGGAGAGGGCTAAC

CCAGCCAACGACCTCTGTTACCCAGGGACCCTCAATGACTATGAGGAACTGAAACACCTA

TTGAGCAGAATAAATCATTTTGAGAAAACTCTGATCATCCCCAAGAGTTCTTGGCCCAAT

CATGAAACATCATTAGGGGTGAGCGCAGCATGTCCATACCAGGGAGCATCCTCATTTTTC

AGAAATGTGGTATGGCTCATCAAAAAGAACGATGCATACCCGACAATAAAGATAAGCTAC

AATAATACCAATCGGGAAGATCTTTTGATACTGTGGGGGATTCATCATTCCAACAATGCA

GCAGAGCAGACAAATCTCTATAAAAACCCAGACACTTATGTTTCCGTTGGGACATCAACA

TTAAACCAGAGATTGGTGCCAAAAATAGCTACTAGATCCCAAGTAAACGGGCAACGTGGA

AGAATGGATTTCTTCTGGACAATTTTAAAACCGAATGATGCAATCCACTTTGAGAGTAAT

GGAAATTTCATTGCTCCAGAATATGCCTACAAAATTGTCAAGAAAGGGGACTCAACAATT

ATGAAAAGTGAAGTGGAGTATGGCCACTGCAACACCAAATGTCAAACTCCAATAGGGGCG

ATAAACTCTAGCATGCCATTCCACAATATACACCCTCTCACCATCGGGGAATGCCCCAAA

TACGTGAAGTCAAACAAATTAGTCCTTGCGACTGGGCTCAGAAATAGTCCTCTAAGAGAA

AGAAGAAGAAAAAGAGGACTATTTGGAGCTATAGCAGGGTTTATAGAGGGAGGATGGCAG

GGAATGGTAGACGGTTGGTATGGGTACCACCATAGCAATGAGCAGGGGAGTGGGTACGCT

GCAGACAAAGAATCCACCCAAAAGGCAATAGATGGAGTTACCAATAAGGTCAACTCAATC

ATTGACAAAATGAACACTCAATTTGAGGCCGTTGGAAGGGAATTTAATAACTTAGAAAGG

AGAATAGAGAATTTAAACAAGAAAATGGAAGACGGATTCCTAGATGTCTGGACTTATAAT

GCTGAACTTTTAGTTCTCATGGAAAATGAGAGAACTCTAGATTTCCATGACTCAAATGTC

AAGAACCTTTACGACAAAGTCCGACTACAGCTTAGGGATAATGCAAAGGAGCTGGGTAAT

GGTTGTTTCGAGTTCTATCACAAATGTGATAACGAATGTATGGAAAGCGTAAGAAATGGG

ACGTATGACTACCCTAAGTATTCAGAAGAAGCAAGATTAAAAAGAGAAGAAATAAGCGGA

GTGAAATTAGAATCAATAGGAACTTACCAAATACTGTCAATTTATTCAACAGTGGCGAGT

TCCCTAGCACTGGCAATCATAGTGGCTGGTCTATCTTTATGGATGTGCTCTAATGGGTCG

CTACAATGCAGAATTTGCATCTAA

>A_goose_Taiwan_TNO15_2015_EPI690744

ATGGAGAAAATAGTGCTTCTTCTTGCAGTGATTAGCCTTGTTAAAAGTGATCAGATTTGC

ATTGGTTACCATGCAAACAACTCAACAAAGCAGGTTGACACGATAATGGAGAAAAACGTC

ACTGTTACACATGCCCAAGACATACTAGAAAAGACACACAACGGGAAGCTCTGCGATCTT

AATGGAGTGAAGCCCCTGATTCTAAAGGATTGTAGCGTAGCTGGGTGGCTCCTTGGAAAT

CCAATGTGCGACGAGTTCATCAGGGTGCCGGAATGGTCTTACATCGTGGAGAGGGCTAAC

CCAGCCAACGACCTCTGTTACCCAGGGACCCTCAATGACTATGAGGAACTGAAACACCTA

TTGAGCCGAATAAATCATTTTGAAAAAACTCTGATCATCCCCAGGAGTTCTTGGCCCAAT

CATGAAACATCATTAGGGGTGAGCGCAGCATGTCCATACCAGGGAGCATCCTCATTTTTC

AGAAATGTGGTATGGCTCATCAAAAAGAACGATGCATACCCGACAATAAAGATAAGCTAC

AATAATACCAATCGGGAAGATCTTTTGATACTGTGGGGGATTCATCATTCCAACAATGCA

GCAGAGCAGACAAATCTCTATAAAAACCCAGACACTTATGTTTCCGTGGGGACATCAACA

TTAAACCAGAGATTGGTGCCAAAAATAGCTACTAGATCCCAAGTAAACGGGCAAAGTGGA

AGAATGGATTTCTTCTGGACAATTTTAAAACCGAATGATGCAATCCACTTTGAGAGTAAT

GGAAATTTCATTGCTCCAGAATATGCATACAAAATTGTCAAGAAAGGGGACTCAACAATT

ATGAAAAGTGAAATGGAGTATGGCCACTGCAACACCAAATGTCAAACTCCAATAGGGGCG

ATAAACTCTAGCATGCCATTCCACAATATACACCCTCTCACCATCGGGGAATGCCCTAAA

TACGTGAAGTCAAACAAATTAGTCCTTGCGACTGGGCTCAGAAATAGTCCTCTAAGAGAA

AGAAGAAGAAAAAGAGGACTTTTTGGAGCTATAGCAGGGTTTATAGAGGGAGGATGGCAG

GGAATGGTAGACGGTTGGTATGGGTATCATCATAGCAATGAGCAGGGGAGTGGGTACGCT

GCAGACAAAGAATCCACCCAAAAGGCAATAGATGGAGTTACCAATAAGGTCAACTCAATC

ATTGACAAAATGAACACTCAATTTGAGGCCGTTGGAAGGGAATTTAATAACTTAGAAAGG

AGAATAGAGAATTTAAACAAGAAAATGGAAGACGGATTCCTAGATGTCTGGACTTATAAT

GCTGAACTTTTAGTTCTCATGGAAAATGAGAGAACTCTAGATTTCCATGACTCAAATGTC

AAGAACCTTTACGACAAAGTCCGGCTACAGCTTAGGGATAATGCAAAAGAGCTGGGTAAT

GGTTGTTTCGAGTTCTATCACAAATGTGATAACGAATGTATGGAGAGCGTAAGAAATGGG

ACGTATGACTACCCTAAGTATTCAGAAGAAGCAATATTAAAAAGAGAAGAAATAAGCGGA

GTGAAATTAGAATCAATAGGAACTTACCAAATACTGTCAATTTATTCAACAGTGGCGAGT

TCCCTAGCACTGGCAATCATAGTGGCTGGTCTATCTTTATGGATGTGCTCTAATGGGTCG

TTACAATGCAGAATTTGCATCTAA

>A_Sichuan_26221_2014_EPI533583_2344a

ATGGAGAAAATAGTGCTTCTTCTTGCAATAGTTAGCCTTGTTAAAGGTGATCAGATTTGC

ATTGGTTACCATGCAAACAACTCGACGGAGCAGGTTGACACGATAATGGAAAAGAACGTC

ACTGTTACACATGCCCAAGACATACTGGAAAAGACACACAATGGAAAGCTCTGCGATTTA

AATGGAGTGAAGCCTCTGATTTTAAAGGATTGTAGTGTAGCTGGATGGCTTCTCGGAAAC

CCAATGTGCGACGAATTCATCAGGGTGCCGGAATGGTCTTACATAGTAGAAAGGGCTAAC

CCAGCCAATGACCTCTGTTACCCAGGGAACCTCAATGATTATGAAGAACTGAAACACCTA

TTGAGCAGAATAAATCATTTTGAGAAAATTCTGATCATCCCCAAGAGTTCTTGGACCAAT

CATGAAACATCATTAGGGGTGAGCGCAGCATGCCCATACCAGGGAACGCCCTCCTTTTTC

AGAAATGTGGTATGGCTTATCAAAAAGAACGATGCATACCCCACAATAAAAATAAGCTAC

AATAATACCAATCAGGAAGATCTGTTGATACTGTGGGGGGTTCACCATTCCAACAATGCA

GCAGAGCAGACGAACCTCTATAAAAACCCAACCACCTATATTTCCGTTGGGACATCAACA

TTAAACCAGAGATTGGTACCAAAAATAGCTACTAGATCCCAAGTGAACGGACAGCGTGGA

AGAATGGATTTCTTCTGGACAATTCTAAAACCGAATGATGCAATCCACTTCGAGAGTAAT

GGAAATTTCATTGCTCCAGAATATGCATACAAAATTGTCAAGAAAGGGGACTCAACAATT

ATGAAAAGTGAAATGGAATATGGCCACTGCAACACCAAATGTCAAACTCCAATAGGGGCG

ATAAACTCTAGTATGCCATTTCACAATATACACCCTCTCACCATCGGGGAATGCCCCAAA

TACGTGAAATCAAACAAATTAGTCCTTGCGACTGGGCTCAGAAATAGTCCTCTAAGAGAA

AAGAGAAGAAAAAGAGGACTATTTGGAGCTATAGCAGGGTTTATAGAGGGAGGATGGCAG

GGAATGGTAGATGGCTGGTATGGGTACCACCATAGCAATGAGCAGGGGAGTGGGTACGCT

GCAGACAAAGAATCCACCCAAAAGGCTATAGATGGAGTTACCAATAAGGTCAACTCGATT

ATTGACAAAATGAACACTCAATTTGAGGCCGTTGGAAGGGAATTTAATAACTTAGAAAGG

AGAATAGAGAATTTAAACAAGAAAATGGAAGACGGATTCCTAGATGTCTGGACTTATAAT

GCTGAACTTCTAGTTCTCATGGAAAATGAGAGAACTCTAGATTTCCATGACTCAAATGTC

AAGAACCTTTACGATAAAGTCCGACTACAGCTTAGGGACAATGCAAAGGAACTGGGTAAT

GGTTGTTTTGAGTTCTATCACAAATGTGATAATAAATGTATGGAAAGTGTAAGAAATGGG

ACATATGACTACCCGCAATATTCAGAAGAAGCAAGATTAAAAAGAGAAGAAATAAGCGGA

GTGAAATTAGAGTCAATAGGAACTTACCAAATACTGTCAATTTATTCAACAGTGGCGAGT

TCCCTAGCACTGGCAATCATAGTGGCTGGTTTATCTTTATGGATGTGCTCCAATGGGTCG

TTGCAGTGCAGAATTTGCATTTAA

>A_breeder_duck_Korea_Gochang1_2014_EPI509698

ATGGAGAAAATAGTGCTTCTTCTTGCAATAGTTAGCCTTGTTAAAAGTGATCAGATTTGC

ATTGGTTACCATGCAAACAACTCGACAGAGCAGGTTGACACGATAATGGAAAAGAACGTC

ACTGTTACACATGCCCAAGACATACTGGAAAAGACACACAACGGGAAGCTCTGCGATCTA

AATGGGGTGAAGCCTCTGATTTTAAAGGATTGTAGTGTAGCTGGATGGCTCCTCGGAAAC

CCAATGTGCGACGAATTCATCAGGGTGCCGGAATGGTCTTACATAGTGGAGAGGGCTAAC

CCAGCCAATGACCTCTGTTACCCAGGGAGCCTCAATGACTATGAAGAACTGAAACACCTA

TTGAGCAGAATAAATCATTTTGAGAAGATTCTGATCATCCCCAAGAGTTCTTGGCCCGAT

CATGAAACATCATTAGGGGTGAGCGCAGCATGTCCATACCAGGGAACGCCCTCCTTTTTC

AGAAATGTGGTATGGCTTATCAAAAAGAACGATGCATACCCAACAATAAAGATAAGCTAC

AATAATACCAATCGGGAAGATCTTTTGATACTGTGGGGGATTCATCATTCCAACAATGCA

GCAGAGCAGACAAATCTCTATAAAAACCCAACCACCTATGTTTCCGTTGGGACATCAACA

TTAAACCAGAGATTGGTACCAAAAATAGCTACTAGATCCCAAGTAAACGGGCAACGTGGA

AGAATGGATTTCTTCTGGACAATTTTAAAACCGAATGATGCAATCCACTTCGAGAGTAAT

GGAAATTTCATTGCTCCAGAATATGCATACAAAATTGTCAAGAAAGGGGACTCAACAATT

ATGAAAAGTGAAGTGGAATATGGCCACTGCAACACCAAATGTCAAACTCCAGTAGGGGCG

ATAAACTCTAGTATGCCATTCCACAATATACATCCTCTCACCATCGGGGAATGCCCCAAA

TACGTGAAGTCAAACAAATTGGTCCTTGCGACTGGGCTCAGAAATAATCCTCTAAGAGAA

AAGAGAAGAAAAAGAGGGCTGTTTGGGGCTATAGCAGGTTTTATAGAGGGAGGATGGCAG

GGAATGGTAGATGGTTGGTATGGGTACCACCATAGCAATGAGCAGGGGAGTGGGTACGCT

GCAGACAAAGAATCCACCCAAAAGGCAATAGATGGAGTTACCAATAAGGTCAACTCGATC

ATTGACAAAATGAACACTCAATTTGAGGCCGTTGGAAGGGAATTTAATAACTTAGAAAGG

AGGCTAGAGAATTTAAACAAGAAAATGGAAGACGGATTCCTAGATGTCTGGACTTATAAT

GCTGAACTTCTAGTTCTCATGGAAAATGAGAGGACTCTAGATTTCCATGACTCAAATGTC

AAGAACCTTTACGACAAAGTCCGACTACAGCTTAGGGATAATGCAAAGGAGCTGGGTAAT

GGTTGTTTCGAGTTCTATCACAAATGTGATAATGAATGTATGGAAAGTGTAAGAAATGGG

ACGTATGACTACCCTCAGTATTCAGAAGAAGCAAGATTAAAAAGAGAAGAAATAAGCGGA

GTGAAATTAGAATCAATAGGAACTTACCAAATACTGTCAATTTATTCAACAGTGGCGAGT

TCCCTAGCACTGGCAATCATGGTGGCTGGTCTATCTTTATGGATGTGCTCCAATGGGTCG

TTACAGTGCAGAATTTGCATTTAA

>A_Fujian-Sanyuan_21099_2017_EPI1202729

ATGGAGGACATAGTGCTTCTTCTTGCAATAGTTAGCCTTGTTAAAAGTGATCAGATTTGC

ATTGGTTACCATGCAAACAACTCGACAGAGCAAGTTGACACGATAATGGAAAAGAACGTC

ACTGTTACACATGCCCAAGACATACTGGAAAAAACACACAATGGGAAGCTCTGCGATCTA

AATGGGGTGAAACCCCTGATTTTAAAGGATTGTAGTGTAGCTGGATGGCTCCTCGGAAAC

CCAATGTGCGACGAATTCATCAGAGTGCCGGAATGGTCTTACATAGTGGAGAGGGCTAAC

CCAGCTAATGACCTCTGTTACCCAGGGAGCCTCAATGACTATGAAGAACTGAAACACCTG

TTGAGCAGAATAAATCATTTTGAGAAGATTCTGATCATCCCCAAGAGTTCTTGGCCCAAT

CATGAAACATCATTAGGGGTGAGTGCAGCTTGTCCATACCAGGGGACGCCCTCCTTTTTC

AGAAATGTGGTATGGCTTATCAAAAAGAACGATGCATACCCAACAATAAAGATAAGCTAC

AATAATACCAATCGGGAAGATCTCTTGATACTGTGGGGGATTCATCATTCCAACAATGCA

GAAGAGCAGACAAATCTCTATAAAAACCCAACCACCTATATTTCAGTTGGAACATCAACA

TTAAACCAGAGATTGGTACCAAAAATAGCTACTAGATCCCAAGTAAACGGGCAACGTGGA

AGAATGGACTTCTTCTGGACAATTTTAAAACCGAATGATGCAATCCATTTCGAGAGTAAT

GGAAATTTCATTGCTCCAGAATATGCATACAAAATTGTCAAGAAAGGGGACTCAACAATT

ATGAAAAGTGAAGTGGAATATGGCCACTGCAACACCAAATGTCAAACCCCAGTAGGAGCG

ATAAACTCTAGTATGCCATTCCACAATATACATCCTCTCACCATCGGGGAATGCCCCAAA

TACGTGAAGTCAAACAAGTTGGTCCTTGCGACTGGGCTCAGAAATAGTCCTCTAAGAGAA

AAGAGAAGAAAAAGAGGGCTGTTTGGGGCTATAGCAGGTTTTATAGAGGGAGGATGGCAG

GGAATGGTTGATGGTTGGTATGGGTACCACCATAGCAATGAGCAGGGGAGTGGGTACGCT

GCAGACAAAGAATCCACCCAAAAGGCAATAGATGGAGTTACCAATAAGGTCAACTCGATC

ATTGACAAAATGAACACTCAATTTGAGGCAGTTGGAAGGGAGTTTAATAACTTAGAAAGG

AGGATAGAGAATTTGAACAAGAAAATGGAAGACGGATTCCTAGATGTCTGGACCTATAAT

GCTGAACTTCTAGTTCTCATGGAAAACGAGAGGACTCTAGATTTCCATGACTCAAATGTC

AAGAACCTTTACGACAAAGTCAGACTGCAGCTTAGGGATAATGCAAAGGAGCTGGGTAAC

GGTTGTTTCGAGTTCTATCACAAATGTGATAATGAATGTATGGAAAGTGTGAAAAATGGG

ACGTATGACTACCCTCAGTATTCAGAAGAAGCAAGATTAAAAAGAGAAGAAATAAGCGGA

GTGAAATTAGAATCAATAGGAACTTACCAAATACTGTCAATTTATTCAACAGTGGCGAGT

TCCCTAGCACTGGCAATCATGGTGGCTGGTCTATCTTTATGGATGTGCTCCAATGGGTCG

TTACAGTGCAGAATTTGCATTTAA

>A_chicken_Iraq_1_2020_EPI1811628

ATGGAGAACATAGTACTTCTTCTTGCAATAGTTAGCCTTGTTAAAAGTGATCAGATTTGC

ATTGGTTACCATGCAAACAATTCGACAGAGCAAGTTGACACGATAATGGAAAAGAACGTC

ACTGTTACACATGCCCAAGACATACTGGAAAAAACACACAACGGGAAGCTCTGTGATCTA

AATGGGGTGAAGCCTCTGATTTTAAAGGATTGTAGTGTAGCTGGATGGCTCCTCGGAAAC

CCAATGTGCGACGAATTCATCAGAGTGCCGGAATGGTCCTACATAGTGGAGAGGGCTAAT

CCAGCTAATGACCTCTGTTACCCAGGGAGCCTCAATGACTATGAAGAACTGAAACACCTG

TTGAGCAGAATAAATCATTTTGAGAAGATTCTGATCATCCCCAAGAGTTCCTGGTCCAAT

CATGAAACATCACTAGGGGTGAGCGCAGCTTGTCCATACCAGGGAGCGCCCTCCTTTTTC

AGAAATGTGGTGTGGCTTATCAAAAAGAACGATGCATACCCAACAATAAAGATAAGCTAC

AATAATACCAATCGGGAAGATCTCTTGATACTGTGGGGGATTCATCATTCCAACAATGCA

GAAGAGCAGACAAATCTCTATAAAAACCCAACCACCTACATTTCAGTTGGAACATCAACT

TTAAACCAGAGGTTGGTACCAAAAATAGCTACTAGATCCCAAGTAAACGGGCAACGTGGA

AGAATGGACTTCTTCTGGACAATTTTAAAACCGGATGATGCAATCCATTTCGAGAGTAAT

GGAAATTTCATTGCTCCAGAATATGCATACAAAATTGTCAAGAAAGGGGACTCAACAATT

ATGAAAAGTGGAGTGGAATATGGCCACTGCAACACCAAATGTCAAACCCCAGTAGGAGCG

ATAAATTCTAGTATGCCATTCCACAACATACATCCTCTCACCATTGGGGAATGCCCCAAA

TACGTGAAGTCAAACAAGTTGGTCCTTGCGACTGGGCTCAGAAATAGTCCTCTTAGAGAA

AAGAGAAGAAAAAGAGGCCTGTTTGGGGCGATAGCAGGGTTTATAGAGGGAGGATGGCAG

GGAATGGTTGATGGTTGGTATGGGTACCACCATAGCAATGAGCAGGGGAGTGGGTACGCT

GCAGACAAAGAATCCACCCAAAAGGCAATAGATGGAGTCACCAATAAGGTCAACTCAATC

ATTGACAAAATGAACACTCAATTTGAGGCAGTTGGAAGGGAGTTTAATAACTTAGAAAGG

AGGATAGAGAATTTGAACAAGAAAATGGAAGACGGATTTCTAGATGTCTGGACCTATAAT

GCTGAACTTCTAGTTCTCATGGAAAACGAGAGAACTCTAGATTTCCATGATTCAAATGTC

AAGAACCTTTACGACAAAGTCAGACTGCAGCTTAGGGATAATGCAAAGGAGCTGGGTAAC

GGCTGTTTCGAATTCTATCACAAATGCGATAATGAATGTATGGAAAGTGTGAGAAATGGG

ACGTATGACTACCCTCAGTATTCAGAAGAAGCAAGATTAAAAAGAGAAGAAATAAGCGGA

GTGAAATTAGAATCAATAGGAACTTACCAGATACTGTCAATTTATTCAACAGCGGCGAGT

TCCCTAGCACTGGCAATCATGATGGCTGGTCTATCTTTATGGATGTGCTCCAATGGGTCG

TTACAGTGCAGAATTTGCATTTAA

>A_domestic_goose_Kazakhstan_1-248_2-20-B_2020_EPI1811601

ATGGAGAACATAGTAYTTCTTCTTGCAATAGTCAGCCTTGTTAAAAGTGATCAGATTTGC

ATTGGTTACCATGCAAACAATTCGACAGAGCAAGTTGACACGATAATGGAAAAGAACGTC

ACTGTTACACATGCCCAAGACATACTGGAAAAAACACACAACGGGAAGCTCTGTGATCTA

AATGGGGTGAAGCCTCTGATTTTAAAGGATTGTAGTGTAGCTGGATGGCTCCTCGGAAAC

CCAATGTGCGACGAATTCATCAGAGTGCCGGAATGGTCCTACATAGTGGAGAGGGCTAAT

CCAGCTAATGACCTCTGTTACCCAGGGAGCCTCAATGACTATGAAGAACTGAAACACCTG

TTGAGCAGAATAAATCATTTTGAGAAGATTCTGATCATCCCCAAGAGTTCCTGGCCAAAT

CATGAAACATCACTAGGGGTGAGCGCAGCTTGTCCATACCAGGGAGCGCCCTCCTTTTTC

AGAAATGTGGTGTGGATTATCAAAAAGAACGATGCATACCCAACAATAAAGATAAGCTAC

AATAATACCAATCGGGAAGATCTCTTGATACTGTGGGGGATTCATCATTCCAACAATGCA

GAAGAGCAGACAACTCTCTATAAAAACCCAACCACCTACATTTCAGTTGGAACATCAACT

TTAAACCAGAGGTTGGTACCAAAAATAGCTACTAGATCCCAAGTAAACGGGCAACGTGGA

AGAATGGACTTCTTCTGGACAATTTTAAAACCGGATGATGCAATCCATTTCGAGAGTAAT

GGAAATTTCATTGCTCCAGAATATGCATACAAAATTGTCAAGAAAGGGGACTCAACAATT

ATGAAAAGTGGAGTGGAATATGGCCACTGCAACACCAAATGTCAAACCCCAGTAGGAGCG

ATAAATTCTAGTATGCCATTCCACAACATACATCCTCTCACCATTGGGGAATGCCCCAAA

TACGTGAAGTCAAACAAGTTGGTCCTTGCGACTGGGCTCAGAAATAGTCCTCTAAGAGAA

AAGAGAAGAAAAAGAGGCCTGTTTGGGGCGATAGCAGGGTTTATAGAGGGAGGATGGCAG

GGAATGGTTGATGGTTGGTATGGGTACCACCATAGCAATGAGCAGGGGAGTGGATACGCT

GCAGACAAAGAATCCACCCAAAAGGCAATAGATGGAGTTACCAATAAGGTCAACTCAATA

ATTGACAAAATGAACACTCAATTTGAGGCAGTTGGAAGGGAGTTTAATAACTTAGAAAGG

AGGATAGAGAATTTGAACAAGAAAATGGAAGACGGATTCCTAGATGTCTGGACCTATAAT

GCTGAACTTCTAGTTCTCATGGAAAACGAGAGGACTCTAGATTTCCATGATTCAAATGTC

AAGAACCTTTACGACAAAGTCAGACTACAGCTTAGGGATAATGCAAAGGAGCTGGGTAAC

GGCTGTTTCGAATTCTATCACAAATGCGATAATGAATGTATGGAAAGTGTGAGAAATGGG

ACGTATGACTACCCTCAGTATTCAGAAGAAGCTAGATTAAAAAGAGAAGAAATAAGCGGA

GTGAAATTAGAATCAATAGGAACTTACCAGATACTGTCAATTTATTCAACAGCGGCGAGT

TCCCTAGCACTGGCAATCATGATGGCTGGTCTATCTTTATGGATGTGCTCCAATGGGTCG

TTACAGTGCAGAATTTGCATTTAA

>A_duck_Saratov_29804_2020_EPI1814265

ATGGAGAACATAGTACTTCTTCTTGCAATAGTTAGCCTTGTTAAAAGTGATCAGATTTGC

ATTGGTTACCATGCAAACAATTCGACAGAGCAAGTTGACACGATAATGGAAAAGAACGTC

ACTGTTACACATGCCCAAGACATACTGGAAAAAACACACAACGGGAAGCTCTGTGATCTA

AATGGGGTGAAGCCTCTGATTTTAAAGGATTGTAGTGTAGCTGGATGGCTCCTCGGAAAC

CCAATGTGCGACGAATTCATCAGAGTGCCGGAATGGTCCTACATAGTGGAGAGGGCTAAT

CCAGCTAATGACCTCTGTTACCCAGGGAGCCTCAATGACTATGAAGAACTGAAACACCTG

TTGAGCAGAATAAATCATTTTGAGAAGATTCTGATCATCCCCAAGAGTTCCTGGCCAAAT

CATGAAACATCACTAGGGGTGAGCGCAGCTTGTCCATACCAGGGAGCGCCCTCCTTTTTC

AGAAATGTGGTGTGGCTTATCAAAAAGAACGATGCATACCCAACAATAAAGATAAGCTAC

AATAATACCAATCGGGAAGATCTCTTGATACTGTGGGGGATTCATCATTCCAACAATGCA

GAAGAGCAGACAAATCTCTATAAAAACCCAACCACCTACATTTCAGTTGGAACATCAACT

TTAAACCAGAGGTTGGTACCAAAAATAGCTACTAGATCCCAAGTAAACGGGCAACGTGGA

AGAATGGACTTCTTCTGGACAATTTTAAAACCGGATGATGCAATCCATTTCGAGAGTAAT

GGAAATTTCATTGCTCCAGAATATGCATACAAAATTGTCAAGAAAGGGGACTCAACAATT

ATGAAAAGTGGAGTGGAATATGGCCACTGCAACACCAAATGTCAAACTCCAGTAGGAGCG

ATAAATTCTAGTATGCCATTCCACAACATACATCCTCTCACCATTGGGGAATGCCCCAAA

TACGTGAAGTCAAACAAGTTGGTCCTTGCGACTGGGCTCAGAAATAGTCCTCTAAGAGAA

AAGAGAAGAAAAAGAGGCCTGTTTGGGGCGATAGCAGGGTTTATAGAGGGAGGATGGCAG

GGAATGGTTGATGGTTGGTATGGGTACCACCATAGCAATGAGCAGGGGAGTGGGTACGCT

GCAGACAAAGAATCCACCCAAAAGGCAATAGATGGAGTTACCAATAAGGTCAACTCAATA

ATTGACAAAATGAACACTCAATTTGAGGCAGTTGGAAGGGAGTTTAATAACTTAGAAAGG

AGGATAGAGAATTTGAACAAGAAAATGGAAGACGGATTCCTAGATGTCTGGACCTATAAT

GCTGAACTTCTAGTTCTCATGGAAAACGAGAGGACTCTAGATTTCCATGATTCAAATGTC

AAGAACCTTTACGACAAAGTCAGACTACAGCTTAGGGATAATGCAAAGGAGCTGGGTAAC

GGCTGTTTCGAATTCTATCACAAATGCGATAATGAATGTATGGAAAGTGTGAGAAATGGG

ACGTATGACTACCCTCAGTATTCAGAAGAAGCAAGATTAAAAAGAGAAGAAATAAGCGGA

GTGAAATTAGAATCAATGGGAACTTACCAGATACTGTCAATTTATTCAACAGCGGCGAGT

TCCCTAGCACTGGCAATCATGATGGCTGGTCTATCTTTATGGATGTGCTCCAATGGGTCG

TTACAGTGCAGAATTTGCATTTAA

>A_chicken_Kazakhstan_Kn-3_2020_EPI1839261

ATGGAGAACATAGTACTTCTTCTTGCAATAGTTAGCCTTGTTAAAAGTGATCAGATTTGC

ATTGGTTACCATGCAAACAATTCGACAGAGCAAGTTGACACGATAATGGAAAAGAACGTC

ACTGTTACACATGCCCAAGACATACTGGAAAAAACACACAACGGGAAGCTCTGTGATCTA

AATGGGGTGAAGCCTCTGATTTTAAAGGATTGTAGTGTAGCTGGATGGCTCCTCGGAAAC

CCAATGTGCGACGAATTCATCAGAGTGCCGGAATGGTCCTACATAGTGGAGAGGGCTAAT

CCAGCTAATGACCTCTGTTACCCAGGGAGCCTCAATGACTATGAAGAACTGAAACACCTG

TTGAGCAGAATAAATCATTTTGAGAAGATTCTGATCATCCCCAAGAGTTCCTGGCCAAAT

CATGAAACATCACTAGGGGTGAGCGCAGCTTGTCCATACCAGGGAGCGCCCTCCTTTTTC

AGAAATGTGGTGTGGCTTATCAAAAAGAACGATGCATACCCAACAATAAAGATAAGCTAC

AATAATACCAATCGGGAAGATCTCTTGATACTGTGGGGGATTCATCATTCCAACAATGCA

GAAGAGCAGACAAATCTCTATAAAAACCCAACCACCTACATTTCAGTTGGAACATCAACT

TTAAACCAGAGGTTGGTACCAAAAATAGCTACTAGATCCCAAGTAAACGGGCAACGTGGA

AGAATGGACTTCTTCTGGACAATTTTAAAACCGGATGATGCAATCCATTTCGAGAGTAAT

GGAAATTTCATTGCTCCAGAATATGCATACAAAATTGTCAAGAAAGGGGACTCAACAATT

ATGAAAAGTGGAGTGGAATATGGCCACTGCAACACCAAATGTCAAACTCCAGTAGGAGCG

ATAAATTCTAGTATGCCATTCCACAACATACATCCTCTCACCATTGGGGAATGCCCCAAA

TACGTGAAGTCAAACAAGTTGGTCCTTGCGACTGGGCTCAGAAATAGTCCTCTAAGAGAA

AAGAGAAGGAAAAGAGGCCTGTTTGGGGCGATAGCAGGGTTTATAGAGGGAGGATGGCAG

GGAATGGTTGATGGTTGGTATGGGTACCACCATAGCAATGAGCAGGGGAGTGGGTACGCT

GCAGACAAAGAATCCACCCAAAAGGCAATAGATGGAGTTACCAATAAGGTCAACTCAATA

ATTGACAAAATGAACACTCAATTTGAGGCAGTTGGAAGGGAGTTTAATAACTTAGAAAGG

AGGATAGAGAATTTGAACAAGAAAATGGAAGACGGATTCCTAGATGTCTGGACCTATAAT

GCTGAACTTCTAGTTCTCATGGAAAACGAGAGGACTCTAGATTTCCATGATTCAAATGTC

AAGAACCTTTACGACAAAGTCAGACTACAGCTTAGGGATAATGCAAAGGAGCTGGGTAAC

GGCTGTTTCGAATTCTATCACAAATGCGATAATGAATGTATGGAAAGTGTGAGAAATGGG

ACGTATGACTACCCTCAGTATTCAGAAGAAGCAAGATTAAAAAGAGAAGAAATAAGCGGA

GTGAAATTAGAATCAATAGGAACTTACCAGATACTGTCAATTTATTCAACAGCGGCGAGT

TCCCTAGCACTGGCAATCATGATGGCTGGTCTATCTTTATGGATGTGCTCCAATGGGTCG

TTACAGTGCAGAATTTGCATTTAA

>A_chicken_Kazakhstan_Kn-6_2020_EPI1839269

ATGGAGAACATAGTACTTCTTCTTGCAATAGTTAGCCTTGTTAAAAGTGATCAGATTTGC

ATTGGTTACCATGCAAACAATTCGACAGAGCAAGTTGACACGATAATGGAAAAGAACGTC

ACTGTTACACATGCCCAAGACATACTGGAAAAAACACACAACGGGAAGCTCTGTGATCTA

AATGGGGTGAAGCCTCTGATTTTAAAGGATTGTAGTGTAGCTGGATGGCTCCTCGGAAAC

CCAATGTGCGACGAATTCATCAGAGTGCCGGAATGGTCCTACATAGTGGAGAGGGCTAAT

CCAGCTAATGACCTCTGTTACCCAGGGAGCCTCAATGACTATGAAGAACTGAAACACCTG

TTGAGCAGAATAAATCATTTTGAGAAGATTCTGATCATCCCCAAGAGTTCCTGGCCAAAT

CATGAAACATCACTAGGGGTGAGCGCAGCTTGTCCATACCAGGGAGCGCCCTCCTTTTTC

AGAAATGTGGTGTGGCTTATCAAAAAGAACGATGCATACCCAACAATAAAGATAAGCTAC

AATAATACCAATCGGGAAGATCTCTTGATACTGTGGGGGATTCATCATTCCAACAATGCA

GAAGAGCAGACAAATCTCTATAAAAACCCAACCACCTACATTTCAGTTGGAACATCAACT

TTAAACCAGAGGTTGGTACCAAAAATAGCTACTAGATCCCAAGTAAACGGGCAACGTGGA

AGAATGGACTTCTTCTGGACAATTTTAAAACCGGATGATGCAATCCATTTCGAGAGTAAT

GGAAATTTCATTGCTCCAGAATATGCATACAAAATTGTCAAGAAAGGGGACTCAACAATT

ATGAAAAGTGGAGTGGAATATGGCCACTGCAACACCAAATGTCAAACTCCAGTAGGAGCG

ATAAATTCTAGTATGCCATTCCACAACATACATCCTCTCACCATTGGGGAATGCCCCAAA

TACGTGAAGTCAAACAAGTTGGTCCTTGCGACTGGGCTCAGAAATAGTCCTCTAAGAGAA

AAGAGAAGGAAAAGAGGCCTGTTTGGGGCGATAGCAGGGTTTATAGAGGGAGGATGGCAG

GGAATGGTTGATGGTTGGTATGGGTACCACCATAGCAATGAGCAGGGGAGTGGGTACGCT

GCAGACAAAGAATCCACCCAAAAGGCAATAGATGGAGTTACCAATAAGGTCAACTCAATA

ATTGACAAAATGAACACTCAATTTGAGGCAGTTGGAAGGGAGTTTAATAACTTAGAAAGG

AGGATAGAGAATTTGAACAAGAAAATGGAAGACGGATTCCTAGATGTCTGGACCTATAAT

GCTGAACTTCTAGTTCTCATGGAAAACGAGAGGACTCTAGATTTCCATGATTCAAATGTC

AAGAACCTTTACGACAAAGTCAGACTACAGCTTAGGGATAATGCAAAGGAGCTGGGTAAC

GGCTGTTTCGAATTCTATCACAAATGCGATAATGAATGTATGGAAAGTGTGAGAAATGGG

ACGTATGACTACCCTCAGTATTCAGAAGAAGCAAGATTAAAAAGAGAAGAAATAAGCGGA

GTGAAATTAGAATCAATAGGAACTTACCAGATACTGTCAATTTATTCAACAGCGGCGAGT

TCCCTAGCACTGGCAATCATGATGGCTGGTCTATCTTTATGGATGTGCTCCAATGGGTCG

TTACAGTGCAGAATTTGCATTTAA

>A_swan_Tumen_1479-2_2020_EPI1814684

ATGGAGAACATAGTACTTCTTCTTGCAATAGTTAGCCTTGTTAAAAGTGATCAGATTTGC

ATTGGTTACCATGCAAACAATTCGACAGAGCAAGTTGACACGATAATGGAAAAGAACGTC

ACTGTTACACATGCCCAAGACATACTGGAAAAAACACACAACGGGAAGCTCTGTGATCTA

AATGGGGTGAAGCCTCTGATTTTAAAGGATTGTAGTGTAGCTGGATGGCTCCTCGGAAAC

CCAATGTGCGACGAATTCATCAGAGTGCCGGAATGGTCCTACATAGTGGAGAGGGCTAAT

CCAGCTAATGACCTCTGTTACCCAGGGAGCCTCAATGACTATGAAGAACTGAAACACCTG

TTGAGCAGAATAAATCATTTTGAGAAGATTCTGATCATCCCCAAGAGTTCCTGGCCAAAT

CATGAAACATCACTAGGGGTGAGCGCAGCTTGTCCATACCAGGGAGCGCCCTCCTTTTTC

AGAAATGTGGTGTGGCTTATCAAAAAGAACGATGCATACCCAACAATAAAGATAAGCTAC

AATAATACCAATCGGGAAGATCTCTTGATACTGTGGGGGATTCATCATTCCAACAATGCA

GAAGAGCAGACAAATCTCTATAAAAACCCAACCACCTACATTTCAGTTGGAACATCAACT

TTAAACCAGAGGTTGGTACCAAAAATAGCTACTAGATCCCAAGTAAACGGGCAACGTGGA

AGAATGGACTTCTTCTGGACAATTTTAAAACCGGATGATGCAATCCATTTCGAGAGTAAT

GGAAATTTCATTGCTCCAGAATATGCATACAAAATTGTCAAGAAAGGGGACTCAACAATT

ATGAAAAGTGGAGTGGAATATGGCCACTGCAACACCAAATGTCAAACTCCAGTAGGAGCG

ATAAATTCTAGTATGCCATTCCACAACATACATCCTCTCACCATTGGGGAATGCCCCAAA

TACGTGAAGTCAAACAAGTTGGTCCTTGCGACTGGGCTCAGAAATAGTCCTCTAAGAGAA

AAGAGAAGAAAAAGAGGCCTGTTTGGGGCGATAGCAGGGTTTATAGAGGGAGGATGGCAG

GGAATGGTTGATGGTTGGTATGGGTACCACCATAGCAATGAGCAGGGGAGTGGGTACGCT

GCAGACAAAGAATCCACCCAAAAGGCAATAGATGGAGTTACCAATAAGGTCAACTCAATA

ATTGACAAAATGAACACTCAATTTGAGGCAGTTGGAAGGGAGTTTAATAACTTAGAAAGG

AGGATAGAGAATTTGAACAAGAAAATGGAAGACGGATTCCTAGATGTCTGGACCTATAAT

GCTGAACTTCTAGTTCTCATGGAAAACGAGAGGACTCTAGATTTCCATGATTCAAATGTC

AAGAACCTTTACGACAAAGTCAGACTACAGCTTAGGGATAATGCAAAGGAGCTGGGTAAC

GGCTGTTTCGAATTCTATCACAAATGCGATAATGAATGTATGGAAAGTGTGAGAAATGGG

ACGTATGACTACCCTCAGTATTCAGAAGAAGCAAGATTAAAAAGAGAAGAAATAAGCGGA

GTGAAATTAGAATCAATAGGAACTTACCAGATACTGTCAATTTATTCAACAGCGGCGAGT

TCCCTAGCACTGGCAATCATGATGGCTGGTCTATCTTTATGGATGTGCTCCAATGGGTCG

TTACAGTGCAGAATTTGCATTTAA

>A_swan_Kazakhstan_9-20-B-Talg-39_2020_EPI1882552

ATGGAGAACATAGTACTTCTTCTTGCAATAGTTAGCCTTGTTAAAAGTGATCAGATTTGC

ATTGGTTACCATGCAAACAATTCGACAGAGCAAGTTGACACGATAATGGAAAAGAACGTC

ACTGTTACACATGCCCAAGACATACTGGAAAAAACACACAACGGGAAGCTCTGTGATCTA

AATGGGGTGAAGCCTCTGATTTTAAAGGATTGTAGTGTAGCTGGATGGCTCCTCGGAAAC

CCAATGTGCGACGAATTCATCAGAGTGCCGGAATGGTCCTACATAGTGGAGAGGGCTAAT

CCAGCTAATGACCTCTGTTACCCAGGGAGCCTCAATGACTATGAAGAACTGAAACACCTG

TTGAGCAGAATAAATCATTTTGAGAAGATTCTGATCATCCCCAAGAGTTCCTGGCCAAAT

CATGAAACATCACTAGGGGTGAGCGCAGCTTGTCCATACCAGGGAGCGCCCTCCTTTTTC

AGAAATGTGGTGTGGCTTATCAAAAAGAACGATGCATACCCAACAATAAAGATAAGCTAC

AATAATACCAATCGGGAAGATCTCTTGATACTGTGGGGGATTCATCATTCCAACAATGCA

GAAGAGCAGACAAATCTCTATAAAAACCCAACCACCTACATTTCAGTTGGAACATCAACT

TTAAACCAGAGGTTGGTACCAAAAATAGCTACTAGATCCCAAGTAAACGGGCAACGTGGA

AGAATGGACTTCTTCTGGACAATTTTAAAACCGGATGATGCAATCCATTTCGAGAGTAAT

GGAAATTTCATTGCTCCAGAATATGCATACAAAATTGTCAAGAAAGGGGACTCAACAATT

ATGAAAAGTGGAGTGGAATATGGCCACTGCAACACCAAATGTCAAACTCCAGTAGGAGCG

ATAAATTCCAGTATGCCATTCCACAACATACATCCTCTCACCATTGGGGAATGCCCCAAA

TACGTGAAGTCAAACAAGTTGGTCCTTGCGACTGGGCTCAGAAATAGTCCTCTAAGAGAA

AAGAGAAGAAAAAGAGGCCTGTTTGGGGCGATAGCAGGGTTTATAGAGGGAGGATGGCAG

GGAATGGTTGATGGTTGGTATGGGTACCACCATAGCAATGAGCAGGGGAGTGGGTACGCT

GCAGACAAAGAATCCACCCAAAAGGCAATAGATGGAGTTACCAATAAGGTCAACTCAATA

ATTGACAAAATGAACACTCAATTTGAGGCAGTTGGAAGGGAGTTTAATAACTTAGAAAGG

AGGATAGAGAATTTGAACAAGAAAATGGAAGACGGATTCCTAGATGTCTGGACCTATAAT

GCTGAACTTCTAGTTCTCATGGAAAACGAGAGGACTCTAGATTTCCATGATTCAAATGTC

AAGAACCTTTACGACAAAGTCAGACTACAGCTTAGGGATAATGCAAAGGAGCTGGGTAAC

GGCTGTTTCGAATTCTATCACAAATGCGATAATGAATGTATGGAAAGTGTGAGAAATGGG

ACGTATGACTACCCTCAGTATTCAGAAGAAGCAAGATTAAAAAGAGAAGAAATAAGCGGA

GTGAAATTAGAATCAATAGGAACTTACCAGATACTGTCAATTTATTCAACAGCGGCGAGT

TCCCTAGCACTGGCAATCATGATGGCTGGTCTATCTTTATGGATGTGCTCCAATGGGTCG

TTACAGTGCAGAATTTGCATTTAA

>A_mute_swan_Czech_Republic_1656-1_2021_EPI1850128

ATGGAGAACATAGTACTTCTTCTTGCAATAGTTAGCCTTGTTAAAAGTGATCAGATTTGC

ATTGGTTACCATGCAAACAATTCGACAGAGCAAGTTGACACGATAATGGAAAAGAACGTC

ACTGTTACACATGCCCAAGACATACTGGAAAAAACACACAACGGGAAGCTCTGTGATCTA

AATGGGGTGAAGCCTCTGATTTTAAAGGATTGTAGTGTAGCTGGATGGCTCCTCGGAAAC

CCAATGTGCGACGAATTCATCAGAGTGCCGGAATGGTCCTACATAGTGGAGAGGGCTAAT

CCAGCTAATGACCTCTGTTACCCAGGGAGCCTCAATGACTATGAAGAACTGAAACACCTG

TTGAGCAGAATAAATCATTTTGAGAAGATTCTGATCATCCCCAAGAGTTCCTGGCCAAAT

CATGAAACATCACTAGGGGTGAGCGCAGCTTGTCCATACCAGGGAGCGCCCTCCTTTTTC

AGAAATGTGGTGTGGCTTATCAAAAAGAACGATGCATACCCAACAATAAAGATAAGCTAC

AATAATACCAATCGGGAAGATCTCTTGATACTGTGGGGGATTCATCATTCCAACAATGCA

GAAGAGCAGACAAATCTCTATAAAAACCCAACCACCTACATTTCAGTTGGAACATCAACT

TTAAACCAGAGGTTGGTACCAAAAATAGCTACTAGATCCCAAGTGAACGGGCAACGTGGA

AGAATGGACTTCTTCTGGACAATTTTAAAACCGGATGATGCAATCCATTTCGAGAGTAAT

GGAAATTTCATTGCTCCAGAATATGCATACAAAATTGTCAAGAAAGGGGACTCAACAATT

ATGAAAAGTGGAGTGGAATATGGCCACTGCAACACCAAATGTCAAACTCCAGTAGGAGCG

ATAAATTCTAGTATGCCATTCCACAACATACATCCTCTCACCATTGGGGAATGCCCCAAA

TACGTGAAGTCAAACAAGTTGGTCCTTGCGACTGGGCTCAGAAATAGTCCTCTAAGAGAA

AAGAGAAGAAAAAGAGGCCTGTTTGGGGCGATAGCAGGGTTTATAGAGGGAGGATGGCAG

GGAATGGTTGATGGTTGGTATGGGTACCACCATAGCAATGAGCAGGGGAGTGGGTACGCT

GCAGACAAAGAATCCACCCAAAAGGCAATAGATGGAGTTACCAATAAGGTCAACTCAATA

ATTGACAAAATGAACACTCAATTTGAGGCAGTTGGAAGGGAGTTTAATAACTTAGAAAGG

AGGATAGAGAATTTGAACAAGAAAATGGAAGACGGATTCCTAGATGTCTGGACCTATAAT

GCTGAACTTCTAGTTCTCATGGAAAACGAGAGGACTCTAGATTTCCATGATTCAAACGTC

AAGAACCTTTACGACAAAGTCAGACTACAGCTCAGGGATAATGCAAAGGAGCTGGGTAAC

GGCTGTTTCGAATTCTATCACAAATGCGATAATGAATGTATGGAAAGTGTGAGAAATGGG

ACGTATGACTACCCTCAGTATTCAGAAGAAGCAAGATTAAAAAGAGAAGAAATAAGCGGA

GTGAAATTAGAATCAATAGGAACTTACCAGATACTGTCAATTTATTCAACAGCGGCGAGT

TCCCTAGCACTGGCAATCATGATGGCTGGTCTATCTTTATGGATGTGCTCCAATGGGTCG

TTACAGTGCAGAATTTGCATTTAA

>A_swan_Lithuania_1298PG1_21VIR2606-3_2021_EPI1858582

ATGGAGAACATAGTACTTCTTCTTGCAATAGTTAGCCTTGTTAAAAGTGATCAGATTTGC

ATTGGTTACCATGCAAACAATTCGACAGAACAAGTTGACACGATAATGGAAAAGAACGTC

ACTGTTACACATGCCCAAGACATACTGGAAAAAACACACAACGGGAAGCTCTGTGATCTA

AATGGGGTGAAGCCTCTGATTTTAAAGGATTGTAGTGTAGCTGGATGGCTCCTCGGAAAC

CCAATGTGCGACGAATTCATCAGAGTGCCGGAATGGTCCTACATAGTGGAGAGGGCTAAT

CCAGCTAATGACCTCTGTTACCCAGGGAGCCTCAATGACTATGAAGAACTGAAACACCTG

TTGAGCAGAATAAATCATTTTGAGAAGATTCTGATCATCCCCAAGAGTTCCTGGCCAAAT

CATGAAACATCACTAGGGGTGAGCGCAGCTTGTCCATACCAGGGAGCGCCCTCCTTTTTC

AGAAATGTGGTGTGGCTTATCAAAAAGAACGATGCGTACCCAACAATAAAGATAAGCTAC

AATAATACCAATCGGGAAGATCTCTTGATACTGTGGGGGATTCATCATTCCAACAATGCA

GAAGAGCAGACAAATCTCTATAAAAACCCAACCACCTACATTTCAGTTGGAACATCAACT

TTAAACCAGAGGTTGGTACCAAAAATAGCTACTAGATCCCAAGTGAACGGGCAACGTGGA

AGAATGGACTTCTTCTGGACAATTTTAAAACCGGATGATGCAATCCATTTCGAGAGTAAT

GGAAATTTCATTGCTCCAGAATATGCATACAAAATTGTCAAGAAAGGGGACTCAACAATT

ATGAAAAGTGGAGTGGAATATGGCCACTGCAACACCAAATGTCAAACTCCAGTAGGAGCG

ATAAATTCTAGTATGCCATTCCACAACATACATCCTCTCACCATTGGGGAATGCCCCAAA

TACGTGAAGTCAAACAAGTTGGTCCTTGCGACTGGGCTCAGAAATAGTCCTCTAAGAGAA

AAGAGAAGAAAAAGAGGCCTGTTTGGGGCGATAGCAGGGTTTATAGAGGGAGGATGGCAG

GGAATGGTTGATGGTTGGTATGGGTACCACCATAGCAATGAGCAGGGGAGTGGGTACGCT

GCAGACAAAGAATCCACCCAAAAGGCAATAGATGGAGTTACCAATAAGGTCAACTCAATA

ATTGACAAAATGAACACTCAATTTGAGGCAGTTGGAAGGGAGTTTAATAACTTAGAAAGG

AGGATAGAGAATTTGAACAAGAAAATGGAAGACGGATTCCTAGATGTCTGGACCTATAAT

GCTGAACTTCTAGTTCTCATGGAAAACGAGAGGACTCTAGATTTCCATGATTCAAACGTC

AAGAACCTTTACGACAAAGTCAGACTACAGCTCAGGGATAATGCAAAGGAGCTGGGTAAC

GGCTGTTTCGAATTCTATCACAAATGCGATAATGAATGTATGGAAAGTGTGAGAAATGGG

ACGTATGACTACCCTCAGTATTCAGAAGAAGCAAGATTAAAAAGAGAAAAAATAAGCGGA

GTGAAATTAGAATCAATAGGAACTTACCAGATACTGTCAATTTATTCAACAGCGGCGAGT

TCCCTAGCACTGGCAATCATGATGGCTGGTCTATCTTTATGGATGTGCTCCAATGGGTCG

TTACAGTGCAGAATTTGCATTTAA

>A_wigeon_Latvia_23903_2021_EPI1855975

ATGGAGAACATAGTACTTCTTCTTGCAATAGTTAGCCTTGTTAAAAGTGATCAGATTTGC

ATTGGTTACCATGCAAACAATTCGACAGAGCAAGTTGACACGATAATGGAAAAGAACGTC

ACTGTCACACATGCCCAAGACATACTGGAAAAAACACACAACGGGAAGCTCTGTGATCTA

AATGGGGTGAAGCCTCTGATTTTAAAGGATTGTAGTGTAGCTGGATGGCTCCTCGGAAAC

CCAATGTGCGACGAATTCATCAGAGTGCCGGAATGGTCCTACATAGTGGAGAGGGCTAAT

CCAGCTAATGACCTCTGTTACCCAGGGAGCCTCAATGACTATGAAGAACTGAAACACCTG

TTGAGCAGAATAAATCATTTTGAGAAGATTCTGATCATCCCCAAGAGTTCCTGGCCAAAT

CATGAAACATCACTAGGGGTGAGCGCAGCTTGTCCATACCAGGGAGCGCCCTCCTTTTTC

AGAAATGTGGTGTGGCTTATCAAAAAGAACGATGCATACCCAACAATAAAGATAAGCTAC

AATAATACCAATCGGGAAGATCTCTTGATACTGTGGGGGATTCATCATTCCAACAATGCA

GAAGAGCAGACAAATCTCTATAAAAACCCAACCACCTACATTTCAGTTGGAACATCAACT

TTAAACCAGAGGTTGGTACCAAAAATAGCTACTAGATCCCAAGTGAACGGGCAACGTGGA

AGAATGGACTTCTTCTGGACAATTTTAAAACCGGATGATGCAATCCATTTCGAGAGTAAT

GGAAATTTCATTGCTCCAGAATATGCATACAAAATTGTCAAGAAAGGGGACTCAACAATT

ATGAAAAGTGGAGTGGAATATGGCCACTGCAACACCAAATGTCAAACTCCAGTAGGAGCG

ATAAATTCTAGTATGCCATTCCACAACATACATCCTCTCACCATTGGGGAATGCCCCAAA

TACGTGAAGTCAAACAAGTTGGTCCTTGCGACTGGGCTCAGAAATAGTCCTCTAAGAGAA

AAGAGAAGAAAACGAGGCCTGTTTGGGGCGATAGCAGGGTTTATAGAGGGAGGATGGCAG

GGAATGGTTGATGGTTGGTATGGGTACCACCATAGCAATGAGCAGGGGAGTGGGTACGCT

GCAGACAAAGAATCCACCCAAAAGGCAATAGATGGAGTTACCAATAAGGTCAACTCAATA

ATTGACAAAATGAACACTCAATTTGAGGCAGTTGGAAGGGAGTTTAATAACTTAGAAAGG

AGGATAGAGAATTTGAACAAGAAAATGGAAGACGGATTCCTAGATGTCTGGACCTATAAT

GCTGAACTTCTAGTTCTCATGGAAAACGAGAGGACTCTAGATTTCCATGATTCAAACGTC

AAGAACCTTTACGACAAAGTCAGACTACAGCTCAGGGATAATGCAAAGGAGCTGGGTAAC

GGCTGTTTCGAATTCTATCACAAATGCGATAATGAATGTATGGAAAGTGTGAGAAATGGG

ACGTATGACTACCCTCAGTATTCAGAAGAAGCAAGATTAAAAAGAGAAGAAATAAGCGGA

GTGAAATTAGAATCAATAGGAACTTACCAGATACTGTCAATTTATTCAACAGCGGCGAGT

TCCCTAGCACTGGCAATCATGATGGCTGGTCTATCTTTATGGATGTGCTCCAATGGGTCG

TTACAGTGCAGAATTTGCATTTAA

>A_mute_swan_Czech_Republic_1410-2_2021_EPI1843606

ATGGAGAACATAGTACTTCTTCTTGCAATAGTTAGCCTTGTTAAAAGTGATCAGATTTGC

ATTGGTTACCATGCAAACAATTCGACAGAGCAAGTTGACACGATAATGGAAAAGAACGTC

ACTGTTACACATGCCCAAGACATACTGGAAAAAACACACAACGGGAAGCTCTGTGATCTA

AATGGGGTGAAGCCTCTGATTTTAAAGGATTGTAGTGTAGCTGGATGGCTCCTCGGAAAC

CCAATGTGCGACGAATTCATCAGAGTGCCGGAATGGTCCTACATAGTGGAGAGGGCTAAT

CCAGCTAATGACCTCTGTTACCCAGGGAGCCTCAATGACTATGAAGAACTGAAACACCTG

TTGAGCAGAATAAATCATTTTGAGAAGATTATGATCATCCCCAAGAGTTCCTGGCCAAAT

CATGAAACATCACTAGGGGTGAGCGCAGCTTGTCCATACCAGGGAGCGCCCTCCTTTTTC

AGAAATGTGGTGTGGCTTATCAAAAAGAACGATGCATACCCAACAATAAAGATAAGCTAC

AATAATACCAATCGGGAAGATCTCTTGATACTGTGGGGGATTCATCATTCCAACAATGCA

GAAGAGCAGACAAATCTCTATAAAAACCCAACCACCTACATTTCAGTTGGAACATCAACT

TTAAACCAGAGGTTGGTACCAAAAATAGCTACTAGATCCCAAGTAAACGGGCAACGTGGG

AGAATGGACTTCTTCTGGACAATTTTAAAACCGGATGATGCAATCCATTTCGAGAGTAAT

GGAAATTTCATTGCTCCAGAATATGCATACAAAATTGTCAAGAAAGGGGACTCAACAATC

ATGAAAAGTGGAGTGGAATATGGCCACTGCAACACCAAATGTCAAACCCCAGTAGGAGCG

ATAAATTCTAGTATGCCATTCCACAACATACATCCTCTCACCATTGGGGAATGCCCCAAA

TACGTGAAGTCAAACAAGTTGGTCCTTGCGACTGGGCTCAGAAATAGCCCTCTAAGAGAA

AAGAGAAGAAAAAGAGGCCTGTTTGGGGCGATAGCAGGGTTTATAGAGGGAGGATGGCAG

GGAATGGTTGATGGTTGGTATGGGTACCACCATAGCAATGAGCAGGGGAGTGGGTACGCT

GCAGACAAAGAATCCACCCAAAAGGCAATAGATGGAGTTACCAATAAGGTCAACTCAATA

ATTGACAAAATGAACACTCAATTTGAGGCAGTTGGAAGGGAGTTTAATAACTTAGAAAGG

AGGATAGAGAATTTGAACAAGAAAATGGAAGACGGATTCCTAGATGTTTGGACCTATAAT

GCTGAACTTCTAGTTCTCATGGAAAACGAGAGGACTCTAGATTTCCATGATTCAAATGTC

AAGAACCTTTACGACAAGGTCAGACTACAGCTTAGGGATAATGCAAAGGAGCTGGGTAAC

GGCTGTTTCGAATTCTATCACAAATGCGATAATGAATGTATGGAAAGTGTGAGAAATGGG

ACGTATGACTACCCTCAGTATTCAGAAGAAGCAAGATTAAAAAGAGAAGAAATAAGCGGA

GTGAAATTAGAATCAATAGGAACTTACCAGATACTGTCAATTTATTCAACAGCGGCGAGT

TCCCTAGCACTGGCAATCATGATGGCTGGTCTATCTTTATGGATGTGCTCCAATGGGTCG

TTACAGTGCAGAATTTGCATTTAA

>A_goose_Kazakhstan_4-190-20-B-H5N8-1_2020_EPI1882525

ATGGAGAACATAGTACTTCTTCTTGCAATAGTTGGCCTTGTTAAAAGTGACCAGATTTGC

ATTGGTTACCATGCAAACAATTCGACAGAGCAAGTTGACACGATAATGGAAAAGAACGTC

ACTGTTACACATGCCCAAGACATACTGGAAAAAACACACAACGGGAAGCTCTGTGATCTA

AATGGGGTGAAGCCTCTGATTTTAAAGGATTGTAGTGTAGCTGGATGGCTCCTCGGAAAC

CCAATGTGCGACGAATTCATCAGAGTGCCGGAATGGTCCTACATAGTGGAGAGGGCTAAT

CCAGCTAATGACCTCTGTTACCCAGGGAGCCTCAATGACTATGAAGAACTGAAACACCTG

TTGAGCAGAATAAATCATTTTGAGAAGATTCTGATCATCCCCAAGAGTTCCTGGCCAAAT

CATGAAACATCACTAGGGGTGAGCGCAGCTTGTCCATACCAGGGAGCGCCCTCCTTTTTC

AGAAACGTGGTGTGGCTTATCAAAAAGAACGATGCATACCCAACAATAAAGATAAGCTAC

AATAATACCAATCGGGAAGATCTCTTGATACTGTGGGGGATTCATCATTCCAACAATGCA

GAAGAGCAGACAAATCTCTATAAAAACCCAACCACCTACATTTCAGTTGGAACATCAACT

TTAAACCAGAGGTTGGTACCAAAAATAGCTACTAGATCCCAAGTAAACGGGCAACGTGGG

AGAATGGACTTCTTCTGGACAATTTTAAAACCGGACGATGCAATCCATTTCGAGAGTAAT

GGAAATTTCATTGCTCCAGAATATGCATACAAAATTGTCAAGAAAGGGGACTCAACAATT

ATGAAAAGTGGAGTGGAATATGGCCACTGCAACACCAAATGTCAAACCCCAGTAGGAGCG

ATAAATTCTAGTATGCCATTCCACAACATACATCCTCTCACCATTGGGGAATGCCCCAAA

TACGTGAAGTCAAACAAGTTGGTCCTTGCGACTGGGCTCAGAAATAGTCCTCTAAGAGAA

AAGAGAAGAAAAAGAGGCCTGTTTGGGGCGATAGCAGGGTTTATAGAGGGAGGATGGCAG

GGAATGGTTGATGGTTGGTATGGGTACCACCATAGCAATGAGCAGGGGAGTGGGTACGCT

GCAGACAAAGAATCCACCCAAAAGGCAATAGATGGAGTTACCAATAAGGTCAACTCAATA

ATTGACAAAATGAACACTCAATTTGAGGCAGTTGGAAGGGAGTTTAATAACTTAGAAAGG

AGGATAGAGAATTTGAACAAGAAAATGGAAGATGGATTTCTAGATGTCTGGACCTATAAT

GCTGAACTTCTAGTTCTCATGGAAAACGAGAGGACTCTAGATTTCCATGATTCAAATGTC

AAGAACCTTTATGACAAGGTCAGACTACAGCTTAGGGATAATGCAAAGGAGCTGGGTAAC

GGCTGTTTCGAATTCTATCACAAATGCGATAATGAATGTATGGAAAGTGTGAGAAATGGG

ACGTATGACTACCCTCAGTATTCAGAAGAAGCAAGATTAAAAAGAGAAGAAATAAGCGGA

GTGAAATTAGAATCAATAGGAACTTACCAGATACTGTCAATTTATTCAACAGCGGCGAGT

TCCCTAGCACTGGCAATCATGATGGCTGGTCTATCTTTATGGATGTGCTCCAATGGGTCG

TTACAGTGCAGAATTTGCATTTAA

>A_chicken_Kazakhstan_220-B-2-H5N8-4_2020_EPI1882547

ATGGAGAACATAGTACTTCTTCTTGCAATAGTTGGCCTTGTTAAAAGTGACCAGATTTGC

ATTGGTTACCATGCAAACAATTCGACAGAGCAAGTTGACACGATAATGGAAAAGAACGTC

ACTGTTACACATGCCCAAGACATACTGGAAAAAACACACAACGGGAAGCTCTGTGATCTA

AATGGGGTGAAGCCTCTGATTTTAAAGGATTGTAGTGTAGCTGGATGGCTCCTCGGAAAC

CCAATGTGCGACGAATTCATCAGAGTGCCGGAATGGTCCTACATAGTGGAGAGGGCTAAT

CCAGCTAATGACCTCTGTTACCCAGGGAGCCTCAATGACTATGAAGAACTGAAACACCTG

TTGAGCAGAATAAATCATTTTGAGAAGATTCTGATCATCCCCAAGAGTTCCTGGCCAAAT

CATGAAACATCACTAGGGGTGAGCGCAGCTTGTCCATACCAGGGAGCGCCCTCCTTTTTC

AGAAACGTGGTGTGGCTTATCAAAAAGAACGATGCATACCCAACAATAAAGATAAGCTAC

AATAATACCAATCGGGAAGATCTCTTGATACTGTGGGGGATTCATCATTCCAACAATGCA

GAAGAGCAGACAAATCTCTATAAAAACCCAACCACCTACATTTCAGTTGGAACATCAACT

TTAAACCAGAGGTTGGTACCAAAAATAGCTACTAGATCCCAAGTAAACGGGCAACGTGGG

AGAATGGACTTCTTCTGGACAATTTTAAAACCGGACGATGCAATCCATTTCGAGAGTAAT

GGAAATTTCATTGCTCCAGAATATGCATACAAAATTGTCAAGAAAGGGGACTCAACAATT

ATGAAAAGTGGAGTGGAATATGGCCACTGCAACACCAAATGTCAAACCCCAGTAGGAGCG

ATAAATTCTAGTATGCCATTCCACAACATACATCCTCTCACCATTGGGGAATGCCCCAAA

TACGTGAAGTCAAACAAGTTGGTCCTTGCGACTGGGCTCAGAAATAGTCCTCTAAGAGAA

AAGAGAAGAAAAAGAGGCCTGTTTGGGGCGATAGCAGGGTTTATAGAGGGAGGATGGCAG

GGAATGGTTGATGGTTGGTATGGGTACCACCATAGCAATGAGCAGGGGAGTGGGTACGCT

GCAGACAAAGAATCCACCCAAAAGGCAATAGATGGAGTTACCAATAAGGTCAACTCAATA

ATTGACAAAATGAACACTCAATTTGAGGCAGTTGGAAGGGAGTTTAATAACTTAGAAAGG

AGGATAGAGAATTTGAACAAGAAAATGGAAGATGGATTTCTAGATGTCTGGACCTATAAT

GCTGAACTTCTAGTTCTCATGGAAAACGAGAGGACTCTAGATTTCCATGATTCAAATGTC

AAGAACCTTTATGACAAGGTCAGACTACAGCTTAGGGATAATGCAAAGGAGCTGGGTAAC

GGCTGTTTCGAATTCTATCACAAATGCGATAATGAATGTATGGAAAGTGTGAGAAATGGG

ACGTATGACTACCCTCAGTATTCAGAAGAAGCAAGATTAAAAAGAGAAGAAATAAGCGGA

GTGAAATTAGAATCAATAGGAACTTACCAGATACTGTCAATTTATTCAACAGCGGCGAGT

TCCCTAGCACTGGCAATCATGATGGCTGGTCTATCTTTATGGATGTGCTCCAATGGGTCG

TTACAGTGCAGAATTTGCATTTAA

>A_mute_swan_Poland_MB189_2021_EPI1859655

ATGGAGAACATAGTACTTCTTCTTGCAATAGTTAGCCTTGTTAAAAGTGATCAGATTTGC

ATTGGTTACCATGCAAACAATTCGACAGAGCAAGTTGACACGATAATGGAAAAGAACGTC

ACTGTTACACATGCCCAAGACATACTGGAAAAAACACACAACGGGAAGCTCTGTGATCTA

AATGGGGTGAAGCCTCTGATTTTAAAGGATTGTAGTGTAGCTGGATGGCTCCTCGGAAAC

CCAATGTGCGACGAATTCATCAGAGTGCCGGAATGGTCCTACATAGTGGAGAGGGCTAAT

CCAGCTAATGACCTCTGTTACCCAGGGAGCCTCAATGACTATGAAGAACTGAAACACCTG

TTGAGCAGAATAAATCATTTTGAGAAGATTCTGATCATCCCCAAGAGTTCCTGGCCAAAT

CATGAAACATCACTAGGGGTGAGCGCAGCTTGTCCATACCAGGGAGCGCCCTCCTTTTTC

AGAAATGTGGTGTGGCTTATCAAAAAGAATGATGCATACCCAACAATAAAGATAAGCTAC

AATAATACCAATCGGGAAGATCTCTTGATACTGTGGGGGATTCATCATTCCAACAATGCA

GAAGAGCAGACAAATCTCTATAAAAACCCAACCACCTACATTTCAGTTGGAACATCAACT

TTAAACCAGAGGTTGGTACCAAAAATAGCTACTAGATCCCAAGTAAACGGGCAACGTGGG

AGAATGGACTTCTTCTGGACAATTTTAAAACCGGATGATGCAATCCATTTCGAGAGTAAT

GGAAATTTCATTGCTCCAGAATATGCATACAAAATTGTCAAGAAAGGGGACTCAACAATT

ATGAAAAGTGGAGTGGAATATGGCCACTGCAACACCAAATGTCAAACCCCAGTAGGAGCG

ATAAATTCTAGTATGCCATTCCACAACATACATCCTCTCACCATTGGGGAATGCCCCAAA

TACGTGAAGTCAAACAAGTTGGTCCTTGCGACTGGGCTCAGAAATAGTCCTCTAAGAGAA

AAGAGAAGAAAAAGAGGCCTGTTTGGGGCGATAGCAGGGTTTATAGAGGGAGGATGGCAG

GGAATGGTTGATGGTTGGTATGGGTACCACCATAGCAATGAGCAGGGGAGTGGGTACGCT

GCAGACAAAGAATCCACCCAAAAGGCAATAGATGGAGTTACCAATAAGGTCAACTCAATA

ATTGACAAAATGAACACTCAATTTGAGGCAGTTGGAAGGGAGTTTAATAACTTAGAAAGG

AGGATAGAGAATTTGAACAAGAAAATGGAAGACGGATTCCTAGATGTCTGGACCTATAAT

GCTGAACTTCTAGTTCTCATGGAAAACGAGAGAACTCTAGATTTCCATGATTCAAATGTC

AAGAACCTTTACGACAAGGTCAGACTACAGCTTAGGGATAATGCAAAGGAGCTGGGTAAC

GGCTGTTTCGAATTCTATCACAAATGCGATAATGAATGTATGGAAAGTGTGAGAAATGGG

ACGTACGACTACCCTCAGTATTCAGAAGAAGCAAGATTAAAAAGAGAAGAAATAAGCGGA

GTGAAATTAGAATCAATAGGAACTTACCAGATACTGTCAATTTATTCAACAGCGGCGAGT

TCCCTAGCACTGGCAATCATGATGGCTGGTCTATCTTTATGGATGTGCTCCAATGGGTCG

TTACAGTGCAGAATTTGCATTTAA

>A_goose_Russian_Federation_Kurgan_1345-25_2020_EPI1811688

ATGGAGAACATAGTACTTCTTCTTGCAATAGTTAGCCTTGTTAAAAGTGATCAGATTTGC

ATTGGTTACCATGCAAACAATTCGACAGAGCAAGTTGACACGATAATGGAAAAGAACGTC

ACTGTTACACATGCCCAAGACATACTGGAAAAAACACACAACGGGAAGCTCTGTGATCTA

AATGGGGTGAAGCCTCTGATTTTAAAGGATTGTAGTGTAGCTGGATGGCTCCTCGGAAAC

CCAATGTGCGACGAATTCATCAGAGTGCCGGAATGGTCCTACATAGTGGAGAGGGCTAAT

CCAGCTAATGACCTCTGTTACCCAGGGAGCCTCAATGACTATGAAGAACTGAAACACCTG

TTGAGCAGAATAAATCATTTTGAGAAGATTCTGATCATCCCCAAGAGTTCCTGGCCAAAT

CATGAAACATCACTAGGGGTGAGCGCAGCTTGTCCATACCAGGGAGCGCCCTCCTTTTTC

AGAAATGTGGTGTGGCTTATCAAAAAGAACGATGCATACCCAACAATAAAGATAAGCTAC

AATAATACCAATCGGGAAGATCTCTTGATACTGTGGGGGATTCATCATTCCAACAATGCA

GAAGAGCAGACAAATCTCTATAAAAACCCAACCACCTACATTTCAGTTGGAACATCAACT

TTAAACCAGAGGTTGGTACCAAAAATAGCTACTAGATCCCAAGTAAACGGGCAACGTGGA

AGAATGGACTTCTTCTGGACAATTTTAAAACCGGATGATGCAATCCATTTCGAGAGTAAT

GGAAATTTCATTGCTCCAGAATATGCATACAAAATTGTCAAGAAAGGGGACTCAACAATT

ATGAAAAGTGGAGTGGAATATGGCCACTGCAACACCAAATGTCAAACCCCAGTAGGAGCG

ATAAATTCTAGTATGCCATTCCACAACATACATCCTCTCACCATTGGGGAATGCCCCAAA

TACGTGAAGTCAAACAAGTTGGTCCTTGCGACTGGGCTCAGAAATAGTCCTCTAAGAGAA

AAGAGAAGAAAAAGAGGCCTGTTTGGGGCGATAGCAGGGTTTATAGAGGGAGGATGGCAG

GGAATGGTTGATGGTTGGTATGGGTACCACCATAGCAATGAGCAGGGGAGTGGGTACGCT

GCAGACAAAGAATCCACCCAAAAGGCAATAGATGGAGTTACCAATAAGGTCAACTCAATC

ATTGACAAAATGAACACTCAATTTGAGGCAGTTGGAAGGGAGTTTAATAACTTAGAAAGG

AGGATAGAGAATTTGAACAAGAAAATGGAAGACGGATTCCTAGATGTCTGGACCTATAAT

GCTGAACTTCTAGTTCTCATGGAAAACGAGAGGACTCTAGATTTCCATGATTCAAATGTC

AAAAACCTTTACGACAAAGTCAGACTACAGCTTAGGGATAATGCAAAGGAGCTGGGTAAC

GGCTGTTTCGAATTCTATCACAAATGCGATAATGAATGTATGGAAAGTGTGAGAAATGGG

ACGTATGACTACCCTCAGTATTCAGAAGAAGCAAGATTAAAAAGAGAAGAAATAAGCGGA

GTGAAATTAGAATCAATAGGAACTTACCAGATACTGTCAATTTATTCAACAGCGGCGAGT

TCCCTAGCACTGGCAATCATGATGGCTGGTCTATCTTTATGGATGTGCTCCAATGGGTCG

TTACAGTGCAGAATTTGCATTTAA

>A_chicken_Rostov-on-Don_308-02_2020_EPI1848670

ATGGAGAACATAGTACTTCTTCTTGCAATAGTTAGCCTTGTTAAAAGTGATCAGATTTGC

ATTGGTTACCATGCAAACAATTCGACAGAGCAAGTTGACACGATAATGGAAAAGAACGTC

ACTGTTACACATGCCCAAGACATACTGGAAAAAACACACAACGGGAAGCTCTGTGATCTA

AATGGGGTGAAGCCTCTGATTTTAAAGGATTGTAGTGTAGCTGGATGGCTCCTCGGAAAC

CCAATGTGCGACGAATTCATCAGAGTGCCGGAATGGTCCTACATAGTGGAGAGGGCTAAT

CCAGCTAATGACCTCTGTTACCCAGGGAGCCTCAATGACTATGAAGAACTGAAACACCTG

TTGAGCAGAATAAATCATTTTGAAAAGATTCTGATCATCCCCAAGAGTTCCTGGCCAAAT

CATGAAACATCACTAGGGGTGAGCGCAGCTTGTCCATACCAGGGAGCGCCCTCCTTTTTC

AGAAATGTGGTGTGGCTTATCAAAAAGAACGATGCATACCCAACAATAAAGATAAGCTAC

AATAATACCAATCGGGAAGATCTCTTGATACTGTGGGGGATTCATCATTCCAACAATGCA

GAAGAGCAGACAAATCTCTATAAAAACCCAACCACCTACATTTCAGTTGGAACATCAACT

TTAAACCAGAGGTTGGTACCAAAAATAGCTACTAGATCCCAAGTAAACGGGCAACGTGGA

AGAATGGACTTCTTCTGGACAATTTTAAAACCGGATGATGCAATCCATTTCGAGAGTAAT

GGAAATTTCATTGCTCCAGAATATGCATACAAAATTGTCAAGAAAGGGGACTCAACAATT

ATGAAAAGTGGAGTGGAATATGGCCACTGTAACACCAAATGTCAAACCCCAGTAGGAGCG

ATAAATTCTAGTATGCCATTCCACAACATACATCCTCTCACCATTGGGGAATGCCCCAAA

TACGTGAAGTCAAACAAGTTGGTCCTTGCGACTGGGCTCAGAAATAGTCCTCTAAGAGAA

AAGAGAAGAAAAAGAGGCCTGTTTGGGGCGATAGCAGGGTTTATAGAGGGAGGATGGCAG

GGAATGGTTGATGGTTGGTATGGGTACCACCATAGCAATGAGCAGGGGAGTGGGTACGCT

GCAGACAAAGAATCCACCCAAAAGGCAATAGATGGAGTTACCAATAAGGTCAACTCAATC

ATTGACAAAATGAACACTCAATTTGAGGCAGTTGGAAGGGAGTTTAATAACTTAGAAAGG

AGGATAGAGAATTTGAACAAGAAAATGGAAGACGGATTCCTAGATGTCTGGACCTATAAT

GCTGAACTTCTAGTTCTCATGGAAAACGAGAGGACTCTAGATTTCCATGATTCAAATGTC

AAAAACCTTTACGACAAAGTCAGACTACAGCTTAGGGATAATGCAAAGGAGCTGGGTAAC

GGCTGTTTCGAATTCTATCACAAATGCGATAATGAATGTATGGAAAGTGTGAGAAATGGG

ACGTATGACTACCCTCAGTATTCAGAAGAAGCAAGATTGAAAAGAGAAGAAATAAGCGGA

GTGAAATTAGAATCAATAGGAACTTACCAGATACTGTCAATTTATTCAACAGCGGCGAGT

TCCCTAGCACTGGCAATCATGATGGCTGGTCTATCTTTATGGATGTGCTCCAATGGGTCG

TTACAGTGCAGAATTTGCATTTAA

>A_pheasant_Wales_000252_2021_EPI1848886

ATGGAGAACATAGTACTTCTTCTTGCAATAGTTAGCCTTGTTAAAAGTGATCAGATTTGC

ATTGGTTACCATGCAAACAATTCGACAGAGCAAGTTGACACGATAATGGAAAAGAACGTC

ACTGTTACACATGCCCAAGACATACTGGAAAAAACACACAACGGGAAGCTCTGTGATCTA

AATGGGGTGAAGCCTCTGATTTTAAAGGATTGTAGTGTAGCTGGATGGCTCCTCGGAAAC

CCAATGTGCGACGAATTCATCAGAGTGCCGGAATGGTCCTACATAGTGGAGAGGGCTAAT

CCAGCTAATGACCTCTGTTACCCAGGGAGCCTCAATGACTATGAAGAACTGAAACACCTG

TTGAGCAGAATAAATCATTTTGAGAAGATTCTGATCATCCCCAAGAGTTCCTGGCCAAAT

CATGAAACATCACAAGGGGTGAGCGCAGCTTGTCCATACCAGGGAGCGCCCTCCTTTTTC

AGAAATGTGGTGTGGCTTATCAAAAAGAACGATGCATACCCAACAATAAAGATAAGCTAC

AATAATACCAATCGGGAAGATCTCTTGATACTGTGGGGGATTCATCATTCCAACAATGCA

GAAGAGCAGACAAATCTCTATAAAAACCCAACCACCTACATTTCAGTTGGAACATCAACT

TTAAACCAGAGGTTGGTACCAAAAATAGCTACTAGATCCCAAGTAAACGGGCAACGTGGA

AGAATGGACTTCTTCTGGACAATTTTAAAACCGGATGATGCAATCCATTTCGAGAGTAAT

GGAAATTTCATTGCTCCAGAATATGCATACAAAATTGTCAAGAAAGGGGACTCAACAATT

ATGAAAAGTGGAGTGGAATATGGCCACTGCAACACCAAATGTCAAACCCCAGTAGGAGCG

ATAAATTCTAGTATGCCATTCCACAACATACATCCTCTCACCATTGGGGAATGCCCCAAA

TACGTGAAGTCAAACAAGTTGGTCCTTGCGACTGGGCTCAGAAATAGTCCTCTAAGAGAA

AAGAGAAGAAAAAGAGGCCTGTTTGGGGCGATAGCAGGGTTTATAGAGGGGGGATGGCAG

GGAATGGTTGATGGTTGGTATGGGTACCACCATAGCAATGAGCAGGGGAGTGGGTACGCT

GCAGACAAAGAATCCACCCAAAAGGCAATAGATGGAGTTACCAATAAGGTCAACTCAATC

ATTGACAAAATGAACACTCAATTTGAGGCAGTTGGAAGGGAGTTTAATAACTTAGAAAGG

AGGATAGAGAATTTGAACAAGAAAATGGAAGACGGATTCCTAGATGTCTGGACCTATAAT

GCTGAACTTCTAGTTCTCATGGAAAACGAGAGGACTCTAGATTTCCATGATTCAAATGTC

AAAAACCTTTACGACAAAGTCAGACTACAGCTTAGGGATAATGCAAAGGAGCTGGGTAAC

GGCTGTTTCGAATTCTATCACAAATGCGATAATGAATGTATGGAAAGTGTGAGAAATGGG

ACGTATGACTACCCTCAGTATTCAGAAGAAGCAAGATTAAAAAGAGAAGAAATAAGCGGA

GTGAAATTAGAATCAATAGGAACTTACCAGATACTGTCAATTTATTCAACAGCGGCGAGT

TCCCTAGCACTGGCAATCATGATGGCTGGTCTATCTTTATGGATGTGCTCCAATGGGTCG

TTACAGTGCAGAATTTGCATTTAA

>A_anser_anser_Spain_297-1_21VIR1230-5_2021_EPI1860071

ATGGAGAACATAGTACTTCTTCTTGCAATAGTTAGCCTTGTTAAAAGTGATCAGATTTGC

ATTGGTTACCATGCAAACAATTCGACAGAGCAAGTTGACACGATAATGGAAAAGAACGTC

ACTGTTACACATGCCCAAGACATACTGGAAAAAACACACAACGGGAAGCTCTGTGATCTA

AATGGGGTGAAGCCTCTGATTTTAAAGGATTGTAGTGTAGCTGGATGGCTCCTCGGAAAC

CCAATGTGCGACGAATTCATCAGAGTGCCGGAATGGTCCTACATAGTGGAGAGGGCTAAT

CCAGCTAATGACCTCTGTTACCCAGGGAGCCTCAATGACTATGAAGAACTGAAACACCTG

TTGAGCAGAATAAATCATTTTGAGAAGATTCTGATCATCCCCAAGAGTTCCTGGCCAAAT

CATGAAACATCACTAGGGGTGAGCGCAGCTTGTCCATACCAGGGAGCGCCCTCCTTTTTC

AGAAATGTGGTGTGGCTTATCAAAAAGAACGATGCATACCCAACAATAAAGATAAGCTAC

AATAATACCAATCGGGAAGATCTCTTGATACTGTGGGGGATTCATCATTCCAACAATGCA

GAAGAGCAGACAAATCTCTATAAAAACCCAACCACCTACATTTCAGTTGGAACATCAACT

TTAAACCAGAGGTTGGTACCAAAAATAGCTACTAGATCCCAAGTAAACGGGCAACGTGGA

AGAATGGACTTCTTCTGGACAATTTTAAAACCGGATGATGCAATCCATTTCGAGAGTAAT

GGAAATTTCATTGCTCCAGAATATGCATACAAAATTGTCAAGAAAGGGGACTCAACAATT

ATGAAAAGTGGAGTGGAATATGGCCACTGCAACACCAAATGTCAAACCCCAGTAGGAGCG

ATAAATTCTAGTATGCCATTCCACAACATACATCCTCTCACCATTGGGGAATGCCCCAAA

TACGTGAAGTCAAACAAGTTGGTCCTTGCGACTGGGCTCAGAAATAGTCCTCTAAGAGAA

AAGAGAAGAAAAAGAGGCCTGTTTGGGGCGATAGCAGGGTTTATAGAGGGAGGATGGCAG

GGAATGGTTGATGGTTGGTATGGGTACCACCATAGCAATGAGCAGGGGAGTGGGTACGCT

GCAGACAAAGAATCCACCCAAAAGGCAATAGATGGAGTTACCAATAAGGTCAACTCAATC

ATTGACAAAATGAACACTCAATTTGAGGCAGTTGGAAGGGAGTTTAATAACTTAGAAAGG

AGGATAGAGAATTTGAACAAGAAAATGGAAGACGGATTCCTAGATGTCTGGACCTATAAT

GCTGAACTTCTAGTTCTCATGGAAAACGAGAGGACTCTAGATTTCCATGATTCAAATGTC

AAAAACCTTTACGACAAAGTCAGACTACAGCTTAGGGATAATGCAAAGGAGCTGGGTAAC

GGCTGTTTCGAATTCTATCACAAATGCGATAATGAATGTATGGAAAGTGTGAGAAATGGG

ACGTATGACTACCCTCAGTATTCAGAAGAAGCAAGATTAAAAAGAGAAGAAATAAGCGGA

GTGAAATTAGAATCAATAGGAACTTACCAGATACTGTCAATTTATTCAACAGCGGCGAGT

TCCCTAGCACTGGCAATCATGATGGCTGGTCTATCTTTATGGATGTGCTCCAATGGGTCG

TTACAGTGCAGAATTTGCATTTAA

>A_turkey_Stavropol_320-02_2020_EPI1848702

ATGGAGAACATAGTACTTCTTCTTGCAATAGTTAGCCTTGTTAAAAGTGATCAGATTTGC

ATTGGTTACCATGCAAACAATTCGACAGAGCAAGTTGACACGATAATGGAAAAGAACGTC

ACTGTTACACATGCCCAAGACATACTGGAAAAAACACACAACGGGAAGCTCTGTGATCTA

AATGGGGTGAAGCCTCTGATTTTAAAGGATTGTAGTGTAGCTGGATGGCTCCTCGGAAAC

CCAATGTGCGACGAATTCATCAGAGTGCCGGAATGGTCTTACATAGTGGAGAGGGCTAAT

CCAGCTAATGACCTCTGTTACCCAGGGAGCCTCAATGACTATGAAGAACTGAAACACCTG

TTGAGCAGAATAAATCATTTTGAGAAGATTCTGATCATCCCCAAGAGTTCCTGGCCAAAT

CATGAAACATCACTAGGGGTGAGCGCAGCTTGTCCATACCAGGGAGCGCCCTCCTTTTTC

AGAAATGTGGTGTGGCTTATCAAAAAGAACGATGCATACCCAACAATAAAGATAAGCTAC

AATAATACCAATCGGGAAGATCTCTTGATACTGTGGGGGATTCATCATTCCAACAATGCA

GAAGAGCAGACAAATCTCTATAAAAACCCAACCACCTACATTTCAGTTGGAACATCAACT

TTAAACCAAAGGTTGGTACCAAAAATAGCTACTAGATCCCAAGTAAACGGGCAACGTGGA

AGAATGGACTTCTTCTGGACAATTTTAAAACCGGATGATGCAATCCATTTCGAGAGTAAT

GGAAATTTCATTGCTCCAGAATATGCATACAAAATTGTCAAGAAAGGGGACTCAACAATT

ATGAAAAGTGGAGTGGAATATGGCCACTGCAACACCAAATGTCAAACCCCAGTAGGAGCG

ATAAATTCTAGTATGCCATTCCACAACATACATCCTCTCACCATTGGGGAATGCCCCAAA

TACGTGAAGTCAAACAAGTTGGTCCTTGCGACTGGGCTCAGAAATAGTCCTCTAAGAGAA

AAGAGAAGAAAAAGAGGCCTGTTTGGGGCGATAGCAGGGTTTATAGAGGGAGGATGGCAG

GGAATGGTTGATGGTTGGTATGGGTACCACCATAGCAATGAGCAGGGGAGTGGGTACGCT

GCAGACAAAGAATCCACCCAAAAGGCAATAGATGGAGTTACCAATAAGGTCAACTCAATC

ATTGACAAAATGAACACTCAATTTGAGGCAGTTGGAAGGGAGTTTAATAACTTAGAAAGG

AGGATAGAGAATTTGAACAAGAAAATGGAAGACGGATTCCTAGATGTCTGGACCTATAAT

GCTGAACTTCTAGTTCTCATGGAAAACGAGAGGACTCTAGATTTCCATGATTCAAATGTC

AAAAACCTTTACGACAAAGTCAGACTACAGCTTAGGGATAATGCAAAGGAGCTGGGTAAC

GGCTGTTTCGAATTCTATCACAAATGCGATAATGAATGTATGGAAAGTGTGAGAAATGGG

ACGTATGACTACCCTCAGTATTCAGAAGAAGCAAGATTAAAAAGAGAAGAAATAAGCGGA

GTGAAATTAGAATCAATAGGAACTTACCAGATACTGTCAATTTATTCAACAGCGGCGAGT

TCCCTAGCACTGGCAATCATGATGGCTGGTCTATCTTTATGGATGTGCTCCAATGGGTCG

TTACAGTGCAGAATTTGCATTTAA

>A_chicken_Czech_Republic_3531-1_2021_EPI1854243

ATGGAGAACATAGTACTTCTTCTTGCAATAGTTAGCCTTGTTAAAAGTGATCAGATTTGC

ATTGGTTACCATGCAAACAATTCGACAGAGCAAGTTGACACGATAATGGAAAAGAACGTC

ACTGTTACACATGCCCAAGACATACTGGAAAAAACACACAACGGGAAGCTCTGTGATCTA

AATGGGGTGAAGCCTCTGATTTTAAAGGATTGTAGTGTAGCTGGATGGCTCCTCGGAAAC

CCAATGTGCGACGAATTCATCAGAGTGCCGGAATGGTCCTACATAGTGGAGAGGGCTAAT

CCAGCTAATGACCTCTGTTACCCAGGGAGCCTCAATGACTATGAAGAACTGAAACACCTG

TTGAGCAGAATAAATCATTTTGAGAAGATTCTGATCATCCCCAAGAGTTCCTGGCCAAAT

CATGAAACATCACTAGGGGTGAGCGCAGCTTGTCCATACCAGGGAGCGCCCTCCTTTTTC

AGAAATGTGGTGTGGCTTATCAAAAAGAACGATGCATACCCAACAATAAAGATAAGCTAC

AATAATACCAATCGGGAAGATCTCTTGATACTGTGGGGGATTCATCATTCCAACAATGCA

GAAGAGCAGACAAATCTCTATAAAAACCCAACCACCTACATTTCAGTTGGAACATCAACT

TTAAACCAGAGGTTGGTACCAAAAATAGCTACTAGATCCCAAGTAAACGGGCAACGTGGA

AGAATGGACTTCTTCTGGACAATTTTAAAACCGGATGATGCAATCCATTTCGAGAGTAAT

GGAAATTTCATTGCTCCAGAATATGCATACAAAATTGTCAAGAAAGGGGACTCAACAATT

ATGAAAAGTGGAGTGGAATATGGCCACTGCAACACCAAATGTCAAACCCCAGTAGGAGCG

ATAAATTCTAGTATGCCATTCCACAACATACATCCTCTCACCATTGGGGAATGCCCCAAA

TACGTGAAGTCAAACAAGTTGGTCCTTGCGACTGGGCTCAGAAATAGTCCTCTAAGAGAA

AAGAGAAGAAAAAGAGGCCTGTTTGGGGCGATAGCAGGGTTTATAGAAGGAGGATGGCAG

GGAATGGTTGATGGTTGGTATGGGTACCACCATAGCAATGAGCAGGGGAGTGGGTACGCT

GCAGACAAAGAATCCACCCAAAAGGCAATAGATGGAGTTACCAATAAGGTCAACTCAATC

ATTGACAAAATGAACACTCAATTTGAGGCAGTTGGAAGGGAGTTTAATAACTTAGAAAGG

AGGATAGAGAATTTGAACAAGAAAATGGAAGACGGATTCCTAGATGTCTGGACCTATAAT

GCTGAACTTCTAGTTCTCATGGAAAACGAGAGGACTCTAGATTTCCATGATTCAAATGTC

AAAAACCTTTACGACAAAGTCAGACTACAGCTTAGGGATAATGCAAAGGAGCTGGGTAAC

GGCTGTTTCGAATTCTATCACAAATGCGATAATGAATGTATGGAAAGTGTGAGAAATGGG

ACGTATGACTACCCTCAGTATTCAGAAGAAGCAAGATTAAAAAGAGAAGAAATAAGCGGA

GTGAAATTAGAATCAATAGGAACTTACCAGATACTGTCAATTTATTCAACGGCGGCGAGT

TCCCTAGCACTGGCAATCATGATGGCTGGTCTATCTTTATGGATGTGCTCCAATGGGTCG

TTACAGTGCAGAATCTGCATTTAA

>A_chicken_Krasnodar_334-03_2021_EPI1848806

ATGGAGAACATAGTACTTCTTCTTGCAATAGTTAGCCTTGTTAAAAGTGATCAGATTTGC

ATTGGTTACCATGCAAACAATTCGACAGAGCAAGTTGACACGATAATGGAAAAGAACGTC

ACTGTTACACATGCCCAAGACATACTGGAAAAAACACACAACGGGAAGCTCTGTGATCTA

AATGGGGTGAAGCCTCTGATTTTAAAGGATTGTAGTGTAGCTGGATGGCTCCTCGGAAAC

CCAATGTGCGACGAATTCATCAGAGTGCCGGAATGGTCCTACATAGTGGAGAGGGCTAAT

CCAGCTAATGACCTCTGTTACCCAGGGAGCCTCAATGACTATGAAGAACTGAAACACCTG

TTGAGCAGAATAAATCATTTTGAGAAGATTCTGATCATCCCCAAGAGTTCCTGGCCAAAT

CATGAAACATCACTAGGGGTGAGCGCAGCTTGTCCATACCAGGGAGCGCCCTCCTTTTTC

AGAAATGTGGTGTGGCTTATCAAAAAGAACGATGCATACCCAACAATAAAGATAAGCTAC

AATAATACCAATCGGGAAGATCTCTTGATACTGTGGGGGATTCATCATTCCAACAATGCA

GAAGAGCAGACAAATCTCTATAAAAACCCAACCACCTACATTTCAGTTGGAACATCAACT

TTAAACCAGAGGTTGGTACCAAAAATAGCTACTAGATCCCAAGTAAACGGGCAACGTGGG

AGAATGGACTTCTTCTGGACAATTTTAAAACCGGATGATGCAATCCATTTCGAGAGTAAT

GGAAATTTCATTGCTCCAGAATATGCATACAAAATTGTCAAGAAAGGGGACTCAACAATT

ATGAAAAGTGGAGTGGAGTATGGCCACTGCAACGCCAAATGTCAAACCCCAGTAGGAGCG

ATAAATTCTAGTATGCCATTCCACAACATACATCCTCTCACCATTGGGGAATGCCCCAAA

TACGTGAAGTCAAACAAGTTGGTCCTTGCGACTGGGCTCAGAAATAGTCCTCTAAGAGAA

AAGAGAAGAAAAAGAGGCCTGTTTGGGGCGATAGCAGGGTTTATAGAGGGAGGATGGCAG

GGAATGGTTGATGGTTGGTATGGGTACCACCATAGCAATGAGCAGGGGAGTGGGTACGCT

GCAGACAAAGAATCCACCCAAAAGGCAATAGATGGAGTTACCAATAAGGTCAACTCAATC

ATTGACAAAATGAACACTCAATTTGAGGCAGTTGGAAGGGAGTTTAATAACTTAGAAAGG

AGGATAGAGAATTTGAACAAGAAAATGGAAGACGGATTCCTAGATGTCTGGACCTATAAT

GCTGAACTTCTAGTTCTCATGGAAAACGAGAGGACTCTAGATTTCCATGATTCAAATGTC

AAAAACCTTTACGACAAAGTCAGACTACAGCTTAGGGATAATGCAAAGGAGCTGGGTAAC

GGCTGTTTCGAATTCTATCACAAATGCGATAATGAATGTATGGAAAGTGTGAGAAATGGG

ACGTATGACTACCCTCAGTATTCAGAAGAAGCAAGATTAAAAAGAGAAGAAATAAGCGGA

GTGAAATTAGAATCAATAGGAACTTACCAGATACTGTCAATTTATTCAACAGCGGCGAGT

TCCCTAGCACTGGCAATCATGATGGCTGGTCTATCTTTATGGATGTGCTCCAATGGGTCG

TTACAGTGCAGAATTTGCATTTAA

>A_mute_swan_Croatia_14_2021_EPI1850962

ATGGAGAACATAGTACTTCTTCTTGCAATAGTTAGCCTTGTTAAAAGTGATCAGATTTGC

ATTGGTTACCATGCAAACAATTCGACAGAGCAAGTTGACACGATAATGGAAAAGAACGTC

ACTGTTACACATGCCCAAGACATACTGGAAAAAACACACAACGGGAAGCTCTGTGATCTA

AATGGGGTGAAGCCTCTGATTTTAAAGGATTGTAGTGTAGCTGGATGGCTCCTCGGAAAC

CCAATGTGCGACGAATTCATCAGAGTGCCGGAATGGTCCTACATAGTGGAGAGGGCTAAT

CCAGCTAATGACCTCTGTTACCCAGGGAGCCTCAATGACTATGAAGAACTGAAACACCTG

TTGAGCAGAATAAATCATTTTGAGAAGATTCTGATCATCCCCAAGAGTTCCTGGCCAAAT

CATGAAACATCACTAGGGGTGAGCGCAGCTTGTCCATACCAGGGAGCGCCCTCCTTTTTC

AGAAATGTGGTGTGGCTTATCAAAAAGAACGATGCATACCCAACAATAAAGATAAGCTAC

AATAATACCAATCGGGAAGATCTCTTGATACTGTGGGGGATTCATCATTCCAACAATGCA

GAAGAGCAGACAAATCTCTATAAAAACCCAACCACCTACATTTCAGTTGGAACATCAACT

TTAAACCAGAGGTTGGTACCAAAAATAGCTACTAGATCCCAAGTAAACGGGCAACGTGGG

AGAATGGACTTCTTCTGGACAATTTTAAAACCGGATGATGCAATCCATTTCGAGAGTAAT

GGAAATTTCATTGCTCCAGAATATGCATACAAAATTGTCAAGAAAGGGGACTCAACAATT

ATGAAAAGTGGAGTGGAGTATGGCCACTGCAACACCAAATGTCAAACCCCAGTAGGAGCG

ATAAATTCTAGTATGCCATTCCACAACATACATCCTCTCACCATTGGGGAATGCCCCAAA

TACGTGAAGTCAAACAAGTTGGTCCTTGCGACTGGGCTCAGAAATAGTCCTCTAAGAGAA

AAGAGAAGAAAAAGAGGCCTGTTTGGGGCGATAGCAGGGTTTATAGAGGGAGGATGGCAG

GGAATGGTTGATGGTTGGTATGGGTACCACCATAGCAATGAGCAGGGGAGTGGGTACGCT

GCAGACAAAGAATCCACCCAAAAGGCAATAGATGGAGTTACCAATAAGGTCAACTCAATC

ATTGACAAAATGAACACTCAATTTGAGGCAGTTGGAAGGGAGTTTAATAACTTAGAAAGG

AGGATAGAGAATTTGAACAAGAAAATGGAAGACGGATTCCTAGATGTCTGGACCTATAAT

GCTGAACTTCTAGTTCTCATGGAAAACGAGAGGACTCTAGATTTCCATGATTCAAATGTC

AAAAACCTTTACGACAAAGTCAGACTACAGCTTAGGGATAATGCAAAGGAGCTGGGTAAC

GGCTGTTTCGAATTCTATCACAAATGCGATAATGAATGTATGGAAAGTGTGAGAAATGGG

ACGTATGACTACCCTCAGTATTCAGAAGAAGCAAGATTAAAAAGAGAAGAAATAAGCGGA

GTGAAATTAGAATCAATAGGAACTTACCAGATACTGTCAATTTATTCAACAGCGGCGAGT

TCCCTAGCACTGGCAATCATGATGGCTGGTCTATCTTTATGGATGTGCTCCAATGGGTCG

TTACAGTGCAGAATTTGCATTTAA

>A_chicken_Bulgaria_50-1_21VIR1454-9_2021_EPI1858622

ATGGAGAACATAGTACTTCTTCTTGCAATAGTTAGCCTTGTTAAAAGTGATCAGATTTGC

ATTGGTTACCATGCAAACAATTCGACAGAGCAAGTTGACACGATAATGGAAAAGAACGTC

ACTGTTACACATGCCCAAGACATACTGGAAAAAACACACAACGGGAAGCTCTGTGATCTA

AATGGGGTGAAGCCTCTGATTTTAAAGGATTGTAGTGTAGCTGGATGGCTCCTCGGAAAC

CCAATGTGCGACGAATTCATCAGAGTGCCGGAATGGTCCTACATAGTGGAGAGGGCTAAT

CCAGCTAATGACCTCTGTTACCCAGGGAGCCTCAATGACTATGAAGAACTGAAACACCTG

TTGAGCAGAATAAATCATTTTGAGAAGATTCTGATCATCCCCAAGAGTTCCTGGCCAAAT

CATGAAACATCACTAGGGGTGAGCGCAGCTTGTCCATACCAGGGAGCGCCCTCCTTTTTC

AGAAATGTGGTGTGGCTTATCAAAAAGAATGATGCATACCCAACAATAAAGATAAGCTAC

AATAATACCAATCGGGAAGATCTCTTGATACTGTGGGGGATTCATCATTCCAACAATGCA

GAAGAGCAGACAAATCTCTATAAAAACCCAACCACCTACATTTCAGTTGGAACATCAACT

TTAAACCAGAGGTTGGTACCAAAAATAGCTACTAGATCCCAAGTAAACGGGCAACGTGGG

AGAATGGACTTCTTCTGGACAATTTTAAAACCGGATGATGCAATCCATTTCGAGAGTAAT

GGAAATTTCATTGCTCCAGAATATGCATACAAAATTGTCAAGAAAGGGGACTCAACAATT

ATGAAAAGTGGAGTGGAGTATGGCCACTGCAACACCAAATGTCAAACCCCAGTAGGAGCG

ATAAATTCTAGTATGCCATTCCACAACATACATCCTCTCACCATTGGGGAATGCCCCAAA

TACGTGAAGTCAAACAAGTTGGTCCTTGCGACTGGGCTCAGAAATAGTCCTCTAAGAGAA

AAGAGAAGAAAAAGAGGCCTGTTTGGGGCGATAGCAGGGTTTATAGAGGGAGGATGGCAG

GGAATGGTTGATGGTTGGTATGGGTACCACCATAGCAATGAGCAGGGGAGTGGGTACGCT

GCAGACAAAGAATCCACCCAAAAGGCAATAGATGGAGTTACCAATAAGGTCAACTCAATC

ATTGACAAAATGAACACTCAATTTGAGGCAGTTGGAAGGGAGTTTAATAACTTAGAAAGG

AGGATAGAGAATTTGAACAAGAAAATGGAAGACGGATTCCTAGATGTCTGGACCTATAAT

GCTGAACTTCTAGTTCTCATGGAAAACGAGAGGACTCTAGATTTCCATGATTCAAATGTC

AAAAACCTTTACGACAAAGTCAGACTACAGCTTAGGGATAATGCAAAGGAGCTGGGTAAC

GGCTGTTTCGAATTCTATCACAAATGCGATAATGAATGTATGGRAAGTGTGAGAAATGGG

ACGTATGACTACCCTCAGTATTCAGAAGAAGCAAGATTAAAAAGAGAAGAAATAAGCGGA

GTGAAATTAGAATCAATAGGAACTTACCAGATACTGTCAATTTATTCAACAGCGGCGAGT

TCCCTAGCACTGGCAATCATGATGGCTGGTCTATCTTTATGGATGTGCTCCAATGGGTCG

TTACAGTGCAGAATTTGCATTTAA

>A_mute_swan_Poland_MB272_2021_EPI1859671

ATGGAGAACATAGTACTTCTTCTTGCAGTAGTTAGCCTTGTTAAAAGTGATCAGATTTGC

ATTGGTTACCATGCAAATAATTCGACAGAGCAAGTTGACACGATAATGGAAAAGAACGTC

ACTGTTACACATGCCCAAGACATACTGGAAAAAACACACAACGGGAAGCTCTGTGATCTA

AATGGGGTGAAGCCTCTGATTTTAAAGGATTGTAGTGTAGCTGGATGGCTCCTCGGAAAC

CCAATGTGCGACGAATTCATCAGAGTGCCGGAATGGTCCTACATAGTGGAGAGGGCTAAT

CCAGCTAATGACCTCTGTTACCCAGGGAGCCTCAATGACTATGAAGAACTGAAACACCTG

TTGAGCAGAATAAATCATTTTGAGAAGATTCTGATCATCCCCAAGAGTTCCTGGCCAAAT

CATGAAACATCACTAGGGGTGAGCGCAGCTTGTCCATACCAGGGAGCGCCCTCCTTTTTC

AGAAATGTGGTGTGGCTTATCAAAAAGAACGATGCATACCCAACAATAAAGATAAGCTAC

AATAATACCAATCGGGAAGATCTCTTGATACTGTGGGGGATTCATCATTCCAACAATGCA

GAAGAGCAGACAAATCTCTATAAAAACCCAACCACCTACATTTCAGTTGGAACATCAACT

TTAAACCAGAGGTTGGTACCAAAAATAGCTACTAGATCCCAAGTAAACGGGCAACGTGGG

AGAATGGACTTCTTCTGGACAATTTTAAAACCGGATGATGCAATCCATTTCGAGAGTAAT

GGAAATTTCATTGCTCCAGAATATGCATACAAAATTGTCAAGAAAGGGGACTCAACAATT

ATGAAAAGTGGAGTGGAATATGGCCACTGCAACACCAAATGTCAAACCCCAGTAGGAGCG

ATAAATTCTAGTATGCCATTCCACAACATACATCCTCTCACCATTGGGGAATGCCCCAAA

TACGTGAAGTCAAACAAGTTGGTCCTTGCGACTGGGCTCAGAAATAGTCCTCTAAGAGAA

AAGAGAAGAAAAAGAGGCCTGTTTGGGGCGATAGCAGGGTTTATAGAGGGAGGATGGCAG

GGAATGGTTGATGGTTGGTATGGGTACCACCATAGCAATGAGCAGGGGAGTGGGTACGCT

GCAGACAAAGAATCCACCCAAAAGGCAATAGATGGAGTTACCAATAAGGTCAACTCAATC

ATTGACAAAATGAACACTCAATTTGAGGCAGTTGGAAGGGAGTTTAATAACTTGGAAAGG

AGGATAGAGAATTTGAACAAGAAAATGGAAGACGGATTCCTAGATGTCTGGACCTATAAT

GCTGAACTTCTAGTTCTCATGGAAAACGAGAGGACTCTAGATTTCCATGATTCAAATGTC

AAAAACCTTTACGACAAAGTCAGACTACAGCTTAGGGATAATGCAAAGGAGCTGGGTAAC

GGCTGTTTCGAATTCTATCACAAATGCGATAATGAATGTATGGAAAGTGTGAGAAATGGG

ACGTATGACTACCCTCAGTATTCAGAAGAAGCAAGATTAAAAAGAGAAGAAATAAGCGGA

GTGAAATTAGAATCAATAGGAACTTACCAGATACTGTCAATTTATTCAACAGCGGCGAGT

TCCCTAGCACTGGCAATCATGATGGCTGGTCTATCTTTATGGATGTGCTCCAATGGGTCG

TTACAGTGCAGAATTTGCATTTAA

>A_ciconia_ciconia_Spain_102-1_21VIR1230-2_2021_EPI1860079

ATGGAGAACATAGTACTTCTTCTTGCAATAGTTAGCCTTGTTAAAAGTGATCAGATTTGC

ATTGGTTACCATGCAAACAATTCGACAGAGCAAGTTGACACGATAATGGAAAAGAACGTC

ACTGTTACACATGCCCAAGACATACTGGAAAAAACACACAACGGGAAGCTCTGTGATCTA

AATGGGGTGAAGCCTCTGATTTTAAAGGATTGTAGTGTAGCTGGATGGCTCCTCGGAAAC

CCAATGTGCGACGAATTCATCAGAGTGCCGGAATGGTCCTACATAGTGGAGAGGGCTAAT

CCAGCTAATGACCTCTGTTACCCAGGGAGCCTCAATGACTATGAAGAACTGAAACACCTG

TTGAGCAGAATAAATCATTTTGAGAAGATTCTGATCATCCCCAAGAGTTCCTGGCCAAAT

CATGAAACATCACTAGGGGTGAGCGCAGCTTGTCCATACCAGGGAGCGCCCTCCTTTTTC

AGAAATGTGGTGTGGCTCATCAAAAAGAACGATGCATACCCAACAATAAAGATAAGCTAC

AATAATACCAATCGGGAAGATCTCTTGATACTGTGGGGGATTCATCATTCCAACAATGCA

GAAGAGCAGACAAATCTCTATAAAAACCCAACCACCTACATTTCAGTTGGAACATCAACT

TTAAACCAGAGATTGGTACCAAAAATAGCTACTAGATCCCAAGTAAACGGGCAACGTGGG

AGAATGGACTTCTTCTGGACAATTTTAAAACCGGATGATGCAATCCATTTCGAGAGTAAT

GGAAATTTCATTGCTCCAGAATATGCATACAAAATTGTCAAGAAAGGGGACTCAACAATT

ATGAAAAGTGGAGTGGAATATGGCCACTGCAACACCAAATGTCAAACCCCAGTAGGAGCG

ATAAATTCTAGTATGCCATTCCACAACATACATCCTCTCACCATTGGGGAATGCCCCAAA

TACGTGAAGTCAAACAAGTTGGTCCTTGCGACTGGGCTCAGAAATAGTCCTCTAAGAGAA

AAGAGAAGAAAAAGAGGCCTGTTTGGGGCGATAGCAGGATTTATAGAGGGAGGATGGCAG

GGAATGGTTGATGGTTGGTATGGGTACCACCATAGCAATGAGCAGGGGAGTGGGTACGCT

GCAGACAAAGAATCCACCCAAAAGGCAATAGATGGAGTTACCAATAAAGTCAACTCAATC

ATTGACAAAATGAACACTCAATTTGAGGCAGTTGGAAGGGAGTTTAATAACTTAGAAAGG

AGGATAGAGAATTTGAACAAGAAAATGGAAGACGGATTCCTAGATGTCTGGACCTATAAT

GCTGAACTTCTAGTTCTCATGGAAAACGAGAGGACTCTAGATTTCCATGATTCAAATGTC

AAAAACCTTTACGACAAAGTCAGACTACAGCTTAGGGATAATGCAAAGGAGCTGGGTAAC

GGCTGTTTCGAATTCTATCACAAATGCGATAATGAATGTATGGAAAGTGTGAGAAATGGG

ACGTATGACTACCCTCAGTATTCAGAAGAAGCAAGATTAAAAAGAGAAGAAATAAGCGGA

GTGAAATTAGAATCAATAGGAACTTACCAGATACTGTCAATTTATTCAACAGCGGCGAGT

TCCCTAGCACTGGCAATCATGATGGCTGGTCTATCTTTATGGATGTGCTCCAATGGGTCG

TTACAGTGCAGAATTTGCATTTAA

>A_chicken_Czech_Republic_4980_2021_EPI1858500

ATGGAGAACATAGTACTTCTTCTTGCAATAGTTAGCCTTGTTAAAAGTGATCAGATTTGC

ATTGGTTACCATGCAAACAATTCGACAGAGCAAGTTGACACGATAATGGAAAAGAACGTC

ACTGTTACACATGCCCAAGACATACTGGAAAAAACACACAACGGGAAGCTCTGTGATCTA

AATGGGGTGAAGCCTCTGATTTTAAAGGATTGTAGTGTAGCTGGATGGCTCCTCGGAAAC

CCAATGTGCGACGAATTCATCAGAGTGCCAGAATGGTCCTACATAGTGGAGAGGGCTAAT

CCAGCTAATGACCTCTGTTACCCAGGGAGCCTCAATGACTATGAAGAACTGAAACACCTG

TTGAGCAGAATAAATCATTTTGAGAAGATTCTGATCATCCCCAAGAGTTCCTGGCCAAAT

CATGAAACATCACTAGGGGTGAGCGCAGCTTGTCCATACCAGGGAGCGCCCTCCTTTTTC

AGAAATGTGGTGTGGCTTATCAAAAAGAACGATGCATACCCAACAATAAAGATAAGCTAC

AATAATACCAATCGGGAAGATCTCTTGATACTGTGGGGGATTCATCATTCCAACAATGCA

GAAGAGCAGACAAATCTCTATAAAAACCCAACCACCTACATTTCAGTTGGAACATCAACT

TTAAACCAGAGGTTGGTACCAAAAATAGCTACTAGATCCCAAGTAAACGGGCAACGTGGG

AGAATGGACTTCTTCTGGACAATTTTAAAACCGGATGATGCAATCCATTTCGAGAGTAAT

GGAAATTTCATTGCTCCAGAATATGCATACAAAATTGTCAAGAAAGGGGACTCAACAATT

ATGAAAAGTGGAGTGGAATATGGCCACTGCAACACCAAATGTCAAACCCCAGTAGGAGCG

ATAAATTCTAGTATGCCATTCCACAACATACATCCTCTCACCATTGGGGAATGCCCCAAA

TACGTGAAGTCAAACAAGTTGGTCCTTGCGACCGGGCTCAGAAATAGTCCTCTAAGAGAA

AAGAGAAGAAAAAGAGGCCTGTTTGGGGCGATTGCAGGGTTTATAGAGGGAGGATGGCAG

GGAATGATTGATGGTTGGTATGGGTACCACCATAGCAATGAGCAGGGGAGTGGGTACGCT

GCAGACAAAGAATCCACCCAAAAGGCAATAGATGGAGTTACCAATAAGGTCAACTCAATC

ATTGACAAAATGAACACTCAATTTGAGGCAGTTGGAAGGGAGTTTAATAACTTAGAAAGG

AGGATAGAGAATTTGAACAAGAAAATGGAAGACGGATTCCTAGATGTCTGGACCTATAAT

GCTGAACTTCTAGTTCTCATGGAAAACGAGAGGACTCTAGATTTCCATGATTCAAATGTC

AAAAACCTTTACGACAAAGTCAGACTACAGCTTAGGGATAATGCAAAGGAGCTGGGTAAC

GGCTGTTTCGAATTCTATCACAAATGCGATAATGAATGTATGGAAAGTGTGAGAAATGGG

ACGTATGACTACCCTCAGTATTCAGAAGAAGCAAGATTAAAAAGAGAAGAAATAAGCGGA

GTGAAATTAGAATCAATAGGAACTTACCAGATACTGTCAATTTATTCAACAGTGGCGAGT

TCCCTAGCACTGGCAATCATGATGGCTGGTCTATCTTTATGGATGTGCTCCAATGGGTCG

TTACAGTGCAGAATTTGCATTTAA

>A_swan_Lithuania_1258PG1_21VIR2606-2_2021_EPI1858574

ATGGAGAACATAGTACTTCTTCTTGCAATAGTTAGCCTTGTTAAAAGTGATCAGATTTGC

ATTGGTTACCATGCAAACAATTCGACAGAGCAAGTTGACACGATAATGGAAAAGAACGTC

ACTGTTACACATGCCCAAGACATACTGGAAAAAACACACAACGGGAAGCTCTGTGATCTA

AATGGGGTGAAGCCTCTGATTTTAAAGGATTGTAGTGTAGCTGGATGGCTCCTCGGAAAC

CCAATGTGCGACGAATTCATCAGAGTGCCGGAATGGTCCTACATAGTGGAGAGGGCTAAT

CCAGCTAATGACCTCTGTTACCCAGGGAGCCTCAATGACTATGAAGAACTGAAACACCTG

TTGAGCAGAATAAATCATTTTGAGAAGATTCTGATCATCCCCAAGAGTTCCTGGCCAAAT

CATGAAACATCACTAGGGGTGAGCGCAGCTTGTCCATACCAGGGAGCGCCCTCCTTTTTC

AGAAATGTGGTGTGGCTTATCAAAAAGAACGATGCATACCCAACAATAAAGATAAGCTAC

AATAATACCAATCGGGAAGATCTCTTGATACTGTGGGGGATTCATCATTCCAACAATGCA

GAAGAGCAGACAAATCTCTATAAAAACCCAAACACCTACATTTCAGTTGGAACATCAACT

TTAAACCAGAGGTTGGTACCAAAAATAGCTACTAGATCCCAAGTAAACGGGCAACGTGGA

AGAATGGACTTCTTCTGGACAATTTTAAAACCGGATGATGCAATCCATTTCGAGAGTAAT

GGAAATTTCATTGCTCCAGAATATGCATACAAAATTGTCAAGAAAGGGGACTCAACAATT

ATGAAAAGTGGAGTGGAATATGGCCACTGCAACACCAAATGTCAAACCCCAGTAGGAGCG

ATAAATTCTAGTATGCCATTCCACAACATACATCCTCTCACCATTGGGGAATGCCCCAAA

TACGTGAAGTCAAACAAGTTGGTCCTTGCGACTGGGCTCAGAAATAGTCCTCTAAGAGAA

AAGAGAAGAAAAAGAGGCCTGTTTGGGGCGATTGCAGGGTTTATAGAGGGAGGATGGCAG

GGAATGGTTGATGGTTGGTATGGGTACCACCATAGCAATGAGCAGGGGAGTGGGTACGCT

GCAGACAAAGAATCCACCCAAAAGGCAATAGATGGAGTTACCAATAAGGTCAACTCAATC

ATTGACAAAATGAACACTCAATTTGAGGCAGTTGGAAGGGAGTTTAATAACTTAGAAAGG

AGGATAGAGAATTTGAACAAGAAAATGGAAGACGGATTCCTAGATGTCTGGACCTATAAT

GCTGAACTTCTAGTTCTCATGGAAAACGAGAGGACTCTAGATTTCCATGATTCAAATGTC

AAAAACCTTTACGACAAAGTCAGACTACAGCTTAGGGATAATGCAAAGGAGCTGGGTAAC

GGCTGTTTCGAATTCTATCACAAATGCGATAATGAATGTATGGAAAGTGTGAGAAATGGG

ACGTATGACTACCCTCAGTATTCAGAAGAAGCAAGATTAAACAGAGAAGAAATAAGCGGA

GTGAAATTAGAATCAATAGGAACTTACCAGATACTGTCAATTTATTCAACAGCGGCGAGT

TCCCTAGCACTGGCAATCATGATGGCTGGTCTATCTTTATGGATGTGCTCCAATGGGTCG

TTACAGTGCAGAATTTGCATTTAA

>A_domestic_duck_Kazakhstan_1-274-20-B_2020_EPI1811611

ATGGAGAACATAGTACTTCTTCTTGCAATAGTTAGCCTTGTTAAAAGTGATCAGATTTGC

ATTGGTTACCATGCAAACAATTCGACAGAGCAAGTTGACACGATAATGGAAAAGAACGTC

ACTGTTACACATGCCCAAGACATACTGGAAAAAACACACAACGGGAAGCTCTGTGATCTA

AATGGGGTGAAGCCTCTGATTTTAAAGGATTGTAGTGTAGCTGGATGGCTCCTCGGAAAC

CCAATGTGCGACGAATTCATCAAAGTGCCGGAATGGTCCTACATAGTGGAGAGGGCTAAT

CCAGCTAATGACCTCTGCTACCCAGGGAGCCTCAATGACTATGAAGAACTGAAACACCTG

TTGAGCAGAATAAATCATTTTGAGAAGATTCTGATCATCCCCAAGAGTTCCTGGCCAAAC

CATGAAACATCACTAGGGGTGAGCGCAGCTTGTCCATACCAGGGAGCGCCCTCCTTTTTC

AGAAATGTGGTGTGGCTTATCAAAAAGAACGATGCATACCCAACAATAAAGATAAGCTAC

AATAATACCAATCGGGAAGATCTCTTGATACTGTGGGGGATTCATCATTCCAACAATGCA

GAAGAGCAGACAAATCTCTATAAAAACCCAACCACCTACATTTCAGTTGGAACATCAACT

TTAAACCAGAGGTTGGTACCAAAAATAGCTACTAGATCCCAAGTAAACGGGCAACGTGGA

AGAATGGACTTCTTCTGGACAATTTTAAAACCGGATGATGCAATCCATTTCGAGAGTAAT

GGAAATTTCATTGCACCAGAATATGCATACAAAATTGTCAAGAAAGGGGACTCAACAATT

ATGAAAAGTGGAGTGGAATATGGCCACTGCAACACCAAATGTCAAACCCCAGTAGGAGCG

ATAAATTCTAGTATGCCATTCCACAACATACATCCTCTCACCATTGGGGAATGCCCCAAA

TACGTGAAGTCAAACAAGTTGGTCCTTGCGACTGGGCTCAGAAATAGTCCTCTAAGAGAA

AAGAGAAGAAAAAGAGGCCTGTTTGGGGCGATAGCAGGGTTTATAGAGGGAGGATGGCAG

GGAATGGTTGATGGTTGGTATGGGTACCACCATAGCAATGAGCAGGGGAGTGGGTACGCT

GCAGACAAAGAATCCACCCAAAAGGCAATCGATGGAGTTACCAATAAGGTCAACTCAATC

ATTGACAAAATGAACACTCAATTTGAGGCAGTTGGAAGGGAGTTTAATAACTTAGAAAGG

AGGATAGAGAATTTGAACAAGAAAATGGAAGACGGATTCCTAGATGTCTGGACCTATAAT

GCTGAACTTCTAGTTCTCATGGAAAACGAGAGGACTCTAGATTTCCATGATTCAAATGTC

AAGAACCTTTACGACAAAGTCAGACTACAGCTTAGGGATAATGCAAAGGAGCTGGGTAAC

GGCTGTTTCGAATTCTACCACAAATGCGATAATGAATGTATGGAAAGTGTGAGAAATGGG

ACGTATGACTACCCTCAGTATTCAGAAGAAGCAAGATTAAAAAGAGAAGAAATAAGCGGA

GTGAAATTAGAATCAATAGGAACTTACCAGATACTGTCAATTTATTCAACAGCGGCGAGT

TCCCTAGCACTGGCAATCATGATGGCTGGTCTATCTTTATGGATGTGCTCCAATGGGTCG

TTACAGTGCAGAATTTGCATTTAA

>A_goose_Omsk_01171_2020_EPI1813201

ATGGAGAACATAGTACTTCTTCTTGCAATAGTTAGCCTTGTTAAAAGTGATCAGATTTGC

ATTGGTTACCATGCAAACAATTCGACAGAGCAAGTTGACACGATAATGGAAAAGAACGTC

ACTGTTACACATGCCCAAGACATACTGGAAAAAACACACAACGGGAAGCTCTGTGATCTA

AATGGGGTGAAGCCTCTGATTTTAAAGGATTGTAGTGTAGCTGGATGGCTCCTCGGAAAC

CCAATGTGCGACGAATTCATCAGAGTGCCGGAATGGTCCTACATAGTGGAGAGGGCTAAT

CCAGCTAATGACCTCTGCTACCCAGGGAGCCTCAATGACTATGAAGAACTGAAACACCTG

TTGAGCAGAATAAATCATTTTGAGAAGATTCTGATCATCCCCAAGAGTTCCTGGCCAAAC

CATGAAACATCACTAGGGGTGAGCTCAGCTTGTCCATACCAGGGAGCGCCCTCCTTTTTC

AGAAATGTGGTGTGGCTTATCAAAAAGAACGATGCATACCCAACAATAAAGATAAGCTAC

AATAATACCAATCGGGAAGATCTCTTGATACTGTGGGGGATTCATCATTCCAACAATGCA

GAAGAGCAGACAAATCTCTATAAAAACCCAACCACCTACATTTCAGTTGGAACATCAACT

TTAAACCAGAGGTTGGTACCAAAAATAGCTACTAGATCCCAAGTAAACGGGCAACGTGGA

AGAATGGACTTCTTCTGGACAATTTTAAAACCGGATGATGCAATCCATTTCGAGAGTAAT

GGAAATTTCATTGCTCCAGAATATGCATACAAAATTGTCAAGAAAGGGGACTCAACAATT

ATGAAAAGTGGAGTGGAATATGGCCACTGCAACACCAAATGTCAAACCCCAGTAGGAGCG

ATAAATTCTAGTATGCCATTCCACAACATACATCCTCTCACCATTGGGGAATGCCCCAAA

TACGTGAAGTCAAACAAGTTGGTCCTTGCGACTGGGCTCAGAAATAGTCCTCTAAGAGAA

AAGAGAAGAAAAAGAGGCCTGTTTGGGGCGATAGCAGGGTTTATAGAGGGAGGATGGCAG

GGAATGGTTGATGGTTGGTATGGGTACCACCATAGCAATGAGCAGGGGAGTGGGTACGCT

GCAGACAAAGAATCCACCCAAAAGGCAATAGATGGAGTTACCAATAAGGTCAACTCAATC

ATTGACAAAATGAACACTCAATTTGAGGCAGTTGGAAGGGAGTTTAATAACTTAGAAAGG

AGGATAGAGAATTTGAACAAGAAAATGGAAGACGGATTCCTAGATGTCTGGACCTATAAT

GCTGAACTTCTAGTTCTCATGGAAAACGAGAGGACTCTAGATTTCCATGATTCAAATGTC

AAGAACCTTTACGACAAAGTCAGACTACAGCTTAGGGATAATGCAAAGGAGCTGGGTAAC

GGCTGTTTCGAATTCTACCACAAATGCGATAATGAATGTATGGAAAGTGTGAGAAATGGG

ACGTATGACTACCCTCAGTATTCAGAAGAAGCAAGATTAAAAAGAGAAGAAATAAGCGGA

GTGAAATTAGAATCAATAGGAACTTACCAGATACTGTCAATTTATTCAACAGCGGCGAGT

TCCCTAGCACTGGCAATCATGATGGCTGGTCTATCTTTATGGATGTGCTCCAATGGGTCG

TTACAGTGCAGAATTTGCATTTAA

>A_chicken_Astrakhan_321-01_2020_EPI1846969

ATGGAGAACATAGTACTTCTTCTTGCAATAGTTAGCCTTGTTAAAAGTGATCAGATTTGC

ATTGGTTATCATGCAAACAATTCGACAGAGCAAGTTGACACGATAATGGAAAAGAACGTC

ACTGTTACACATGCCCAAGACATACTGGAAAAAACACACAACGGGAAGCTCTGTGATCTA

AATGGGGTGAAGCCTCTGATTTTAAAGGATTGTAGTGTAGCTGGATGGCTCCTCGGAAAC

CCAATGTGCGACGAATTCATCAGAGTGCCGGAATGGTCCTACATAGTGGAGAGGGCTAAT

CCAGCTAATGACCTCTGCTACCCAGGGAGCCTCAATGACTATGAAGAACTGAAACACCTG

TTGAGCAGAATAAATCATTTTGAGAAGATTCTGATTATCCCCAAGAGTTCCTGGCCAAAC

CATGAAACATCACTAGGGGTGAGCGCAGCTTGTCCATACCAGGGAGCGCCCTCCTTTTTC

AGAAATGTGGTGTGGCTTATCAAAAAGAACGATGCATACCCAACGATAAAGATAAGCTAC

AATAATACCAATCGGGAAGATCTCTTGATACTGTGGGGGATTCATCATTCCAACAATGCA

GAAGAGCAGACAAATCTCTATAAAAACCCAACCACCTACATTTCAGTTGGAACATCAACT

TTAAACCAGAGGTTGGTACCAAAAATAGCTACTAGATCCCAAGTAAACGGGCAACGTGGA

AGAATGGACTTCTTCTGGACAATTTTAAAACCGGATGATGCAATCCATTTCGAGAGTAAT

GGAAATTTCATTGCTCCAGAATATGCATACAAAATTGTCAAGAAAGGGGACTCAACAATT

ATGAAAAGTGGAGTGGAATATGGCCACTGCAACACCAAATGTCAAACCCCAGTAGGAGCG

ATAAATTCTAGTATGCCATTCCACAACATACATCCTCTCACCATTGGGGAATGCCCCAAA

TACGTGAAGTCAAACAAGTTGGTCCTTGCGACTGGGCTCAGAAATAGTCCTCTAAGAGAA

AAGAGAAGAAAAAGAGGCCTGTTTGGGGCGATAGCAGGGTTTATAGAGGGAGGATGGCAG

GGAATGGTTGATGGTTGGTATGGGTACCACCATAGCAATGAGCAGGGGAGTGGGTACGCT

GCAGACAAAGAATCCACCCAAAAGGCAATAGATGGAGTTACCAATAAGGTCAACTCAATC

ATTGACAAAATGAACACTCAATTTGAGGCAGTTGGAAGGGAGTTTAATAACTTAGAAAGG

AGGATAGAGAATTTGAACAAGAAAATGGAAGACGGATTCCTAGATGTCTGGACCTATAAT

GCTGAACTTCTAGTTCTCATGGAAAACGAGAGGACTCTAGATTTCCATGATTCAAATGTC

AAGAACCTTTACGACAAAGTCAGACTACAGCTTAGGGATAATGCAAAGGAGCTGGGTAAC

GGCTGTTTCGAATTCTACCACAAATGCGATAATGAATGTATGGAAAGTGTGAGAAATGGG

ACGTATGACTACCCTCAGTATTCAGAAGAAGCAAGATTAAAAAGAGAAGAAATAAGCGGA

GTGAAATTAGAATCAATAGGAACTTACCAGATACTGTCAATTTATTCAACAGCGGCGAGT

TCCCTAGCACTGGCAATCATGATGGCTGGTCTATCTTTATGGATGTGCTCCAATGGGTCG

TTACAGTGCAGAATTTGCATTTAA

>A_Astrakhan_3212_2020_EPI1846961

ATGGAGAACATAGTACTTCTTCTTGCAATAGTTAGCCTTGTTAAAAGTGATCAGATTTGC

ATTGGTTATCATGCAAACAATTCGACAGAGCAAGTTGACACGATAATGGAAAAGAACGTC

ACTGTTACACATGCCCAAGACATACTGGAAAAAACACACAACGGGAAGCTCTGTGATCTA

AATGGGGTGAAGCCTCTGATTTTAAAGGATTGTAGTGTAGCTGGATGGCTCCTCGGAAAC

CCAATGTGCGACGAATTCATCAGAGTGCCGGAATGGTCCTACATAGTGGAGAGGGCTAAT

CCAGCTAATGACCTCTGCTACCCAGGGAGCCTCAATGACTATGAAGAACTGAAACACCTG

TTGAGCAGAATAAATCATTTTGAGAAGATTCTGATTATCCCCAAGAGTTCCTGGCCAAAC

CATGAAACATCACTAGGGGTGAGCGCAGCTTGTCCATACCAGGGAGCGCCCTCCTTTTTC

AGAAATGTGGTGTGGCTTATCAAAAAGAACGATGCATACCCAACGATAAAGATAAGCTAC

AATAATACCAATCGGGAAGATCTCTTGATACTGTGGGGGATTCATCATTCCAACAATGCA

GAAGAGCAGACAAATCTCTATAAAAACCCAACCACCTACATTTCAGTTGGAACATCAACT

TTAAACCAGAGGTTGGTACCAAAAATAGCTACTAGATCCCAAGTAAACGGGCAACGTGGA

AGAATGGACTTCTTCTGGACAATTTTAAAACCGGATGATGCAATCCATTTCGAGAGTAAT

GGAAATTTCATTGCTCCAGAATATGCATACAAAATTGTCAAGAAAGGGGACTCAACAATT

ATGAAAAGTGGAGTGGAATATGGCCACTGCAACACCAAATGTCAAACCCCAGTAGGAGCG

ATAAATTCTAGTATGCCATTCCACAACATACATCCTCTCACCATTGGGGAATGCCCCAAA

TACGTGAAGTCAAACAAGTTGGTCCTTGCGACTGGGCTCAGAAATAGTCCTCTAAGAGAA

AAGAGAAGAAAAAGAGGCCTGTTTGGGGCGATAGCAGGGTTTATAGAGGGAGGATGGCAG

GGAATGGTTGATGGTTGGTATGGGTACCACCATAGCAATGAGCAGGGGAGTGGGTACGCT

GCAGACAAAGAATCCACCCAAAAGGCAATAGATGGAGTTACCAATAAGGTCAACTCAATC

ATTGACAAAATGAACACTCAATTTGAGGCAGTTGGAAGGGAGTTTAATAACTTAGAAAGG

AGGATAGAGAATTTGAACAAGAAAATGGAAGACGGATTCCTAGATGTCTGGACCTATAAT

GCTGAACTTCTAGTTCTCATGGAAAACGAGAGGACTCTAGATTTCCATGATTCAAATGTC

AAGAACCTTTACGACAAAGTCAGACTACAGCTTAGGGATAATGCAAAGGAGCTGGGTAAC

GGCTGTTTCGAATTCTACCACAAATGCGATAATGAATGTATGGAAAGTGTGAGAAATGGG

ACGTATGACTACCCTCAGTATTCAGAAGAAGCAAGATTAAAAAGAGAAGAAATAAGCGGA

GTGAAATTAGAATCAATAGGAACTTACCAGATACTGTCAATTTATTCAACAGCGGCGAGT

TCCCTAGCACTGGCAATCATGATGGCTGGTCTATCTTTATGGATGTGCTCCAATGGGTCG

TTACAGTGCAGAATTTGCATTTAA

>A_chicken_Kazakhstan_12-20-B-Talg-45_2020_EPI1882555

ATGGAGAACATAGTACTTCTTCTTGCAATAGTTAGCCTTGTTAAAAGTGATCAGATTTGC

ATTGGTTACCATGCAAACAATTCGACAGAGCAAGTTGACACGATAATGGAAAAGAACGTC

ACTGTTACACATGCCCAAGACATACTGGAAAAAACACACAACGGGAAGCTCTGTGATCTA

AATGGGGTGAAGCCTCTGATTTTAAAGGATTGTAGTGTAGCTGGATGGCTCCTCGGAAAC

CCAATGTGCGACGAATTCATCAGAGTGCCGGAATGGTCCTACATAGTGGAGAGGGCTAAT

CCAGCTAATGACCTCTGCTACCCAGGGAGCCTCAATGACTATGAAGAACTGAAACACCTG

TTGAGCAGAATAAATCATTTTGAGAAGATTCTGATCATCCCCAAGAGTTCCTGGCCAAAC

CATGAAACATCACTAGGGGTGAGCGCAGCTTGTCCATACCAGGGAGCGCCCTCCTTTTTC

AGAAATGTGGTGTGGCTTATCAAAAAGAACGATGCATACCCAACAATAAAGATAAGCTAC

AATAATACCAATCGGGAAGATCTCTTGATACTGTGGGGGATTCATCATTCCAACAATGCA

GAAGAGCAGACAAATCTCTATAAAAACCCAACCACCTACATTTCAGTTGGAACATCAACT

TTAAACCAGAGGTTGGTACCAAAAATAGCTACTAGATCCCAAGTAAACGGGCAACGTGGA

AGAATGGACTTCTTCTGGACAATTTTAAAACCGGATGATGCAATCCATTTCGAGAGTAAT

GGAAATTTCATTGCTCCAGAATATGCATACAAAATTGTCAAGAAAGGGGACTCAACAATT

ATGAAAAGTGGAGTGGAATATGGCCACTGCAACACCAAATGTCAAACCCCAGTAGGAGCG

ATAAATTCTAGTATGCCATTCCACAACATACATCCTCTCACCATTGGGGAATGCCCCAAA

TACGTGAAGTCAAACAAGTTGGTCCTTGCGACTGGGCTCAGAAATAGTCCTCTAAGAGAA

AAGAGAAGAAAAAGAGGCCTGTTTGGGGCGATAGCAGGGTTTATAGAGGGAGGATGGCAG

GGAATGGTTGATGGTTGGTATGGGTACCACCATAGCAATGAGCAGGGGAGTGGGTACGCT

GCAGACAAAGAATCCACCCAAAAGGCAATAGATGGAGTTACCAATAAGGTCAACTCAATC

ATTGACAAAATGAACACTCAATTTGAGGCAGTTGGAAGGGAGTTTAATAACTTAGAAAGG

AGGATAGAGAATTTGAACAAGAAAATGGAAGACGGATTCCTAGATGTCTGGACCTATAAT

GCTGAACTTCTAGTTCTCATGGAAAACGAGAGGACTCTAGATTTCCATGATTCAAATGTC

AAGAACCTTTACGACAAAGTCAGACTACAGCTTAGGGATAATGCAAAGGAGCTGGGTAAC

GGCTGTTTCGAATTCTACCACAAATGCGATAATGAATGTATGGAAAGTGTGAGAAATGGG

ACGTATGACTACCCTCAGTATTCAGAAGAAGCAAGATTAAAAAGAGAAGAAATAAGCGGA

GTGAAATTAGAATCAATAGGAACTTACCAGATACTGTCAATTTATTCAACAGCGGCGAGT

TCCCTAGCACTGGCAATCATGATGGCTGGTCTATCTTTATGGATGTGCTCCAATGGGTCG

TTACAGTGCAGAATTTGCATTTAA

>A_domestic_goose_Kazakhstan_1-242_2-20-B_2020_EPI1811619

ATGGAGAANATAGTACTTCTTCTTGCAATAGTTAGCCTTGTTAAAAGTGATCAGATTTGC

ATTGGTTACCATGCAAACAATTCGACAGAGCAAGTTGACACGATAATGGAAAAGAACGTC

ACTGTTACACATGCCCAAGACATACTGGAAAAAACACACAACGGGAAGCTCTGTGATCTA

AATGGGGTGAAGCCTCTGATTTTAAAGGATTGTAGTGTAGCTGGATGGCTCCTCGGAAAC

CCAATGTGCGACGAATTCATCAGAGTGCCGGAATGGTCCTACATAGTGGAGAGGGCTAAT

CCAGCTAATGACCTCTGTTACCCAGGGAGCCTCAATGACTATGAAGAACTGAAACACCTG

TTGAGCAGAATAAATCATTTTGAGAAGATTCTGATCATCCCCAAGAGTTCCTGGCCAAAC

CATGAAACATCACTAGGGGTGAGCGCAGCTTGTCCATACCAGGGAGCGCCCTCCTTTTTC

AGAAATGTGGTGTGGCTTATCAAAAAGAACGATGCATACCCAACAATAAAGATAAGCTAC

AATAATACCAATCGGGAAGATCTCTTGATACTGTGGGGAATTCATCATTCCAACAATGCA

GAAGAGCAGACAAATCTCTATAAAAACCCAACCACCTACATTTCAGTTGGAACATCAACT

TTAAACCAGAGGTTGGTACCAAAAATAGCTACTAGATCCCAAGTAAACGGGCAACGTGGA

AGAATGGACTTCTTCTGGACAATTTTAAAACCGGATGATGCAATCCATTTCGAGAGTAAT

GGAAATTTCATTGCTCCAGAATATGCATACAAAATTGTCAAGAAAGGGGACTCAACAATT

ATGAAAAGTGGAGTGGAATATGGCCACTGCAACACCAAATGTCAAACCCCAGTAGGAGCG

ATAAATTCTAGTATGCCATTCCACAACATACATCCTCTCACCATTGGGGAATGCCCCAAA

TACGTGAAGTCAAACAAGTTGGTCCTTGCGACTGGGCTCAGAAATAGTCCTCTAAGAGAA

AAGAGAAGAAAAAGAGGCCTGTTTGGGGCGATAGCAGGGTTTATAGAGGGAGGATGGCAG

GGAATGGTTGATGGTTGGTATGGGTACCACCATAGCAATGAGCAGGGGAGTGGGTACGCT

GCAGACAAAGAATCCACCCAAAAGGCAATAGATGGAGTTACCAATAAGGTCAACTCAATC

ATTGACAAAATGAACACTCAATTTGAGGCAGTTGGAAGGGAGTTTAATAACTTAGAAAGG

AGGATAGAGAATTTGAACAAGAAAATGGAAGACGGATTCCTAGATGTCTGGACCTATAAT

GCTGAACTTCTAGTTCTCATGGAAAACGAGAGGACTCTAGATTTCCATGATTCAAATGTC

AAGAACCTTTACGACAAAGTCAGACTACAGCTTAGGGATAATGCAAAGGAGCTGGGTAAC

GGCTGTTTCGAATTCTATCACAAATGCGATAATGAATGTATGGAAAGTGTGAGAAATGGG

ACGTATGACTACCCTCAGTATTCAGAAGAAGCAAGATTAAAAAGAGAAGAAATAAGCGGA

GTGAAATTAGAATCAATAGGAACTTACCAGATACTGTCAATTTATTCAACAGCGGCGAGT

TCCCTAGCACTGGCAATCATGATGGCTGGTCTATCTTTATGGATGTGCTCCAATGGGTCG

TTACAGTGCAGAATTTGCATTTAA

>A_swan_Kazakhstan_1-267-20-B-Talg-52_2020_EPI1882559

ATGGAGAACATAGTACTTCTTCTTGCAATAGTTAGCCTTGTTAAAAGTGATCAGATTTGC

ATTGGTTACCATGCAAACAATTCGACAGAGCAAGTTGACACGATAATGGAAAAGAACGTC

ACTGTTACACATGCCCAAGACATACTGGAAAAAACACACAACGGGAAGCTCTGTGATCTA

AATGGGGTGAAGCCTCTGATTTTAAAGGATTGTAGTGTAGCTGGATGGCTCCTCGGAAAC

CCAATGTGCGACGAATTCATCAGAGTGCCGGAATGGTCCTACATAGTGGAGAGGGCTAAT

CCAGCTAATGACCTCTGTTACCCAGGGAGCCTCAATGACTATGAAGAACTGAAACACCTG

TTGAGCAGAATAAATCATTTTGAGAAGATTCTGATCATCCCCAAGAGTTCCTGGCCAAAC

CATGAAACATCACTAGGGGTGAGCGCAGCTTGTCCATACCAGGGAGCGCCCTCCTTTTTC

AGAAATGTGGTGTGGCTTATCAAAAAGAACGATGCATACCCAACAATAAAGATAAGCTAC

AATAATACCAATCGGGAAGATCTCTTGATACTGTGGGGAATTCATCATTCCAACAATGCA

GAAGAGCAGACAAATCTCTATAAAAACCCAACCACCTACATTTCAGTTGGAACATCAACT

TTAAACCAGAGGTTGGTACCAAAAATAGCTACTAGATCCCAAGTAAACGGGCAACGTGGA

AGAATGGACTTCTTCTGGACAATTTTAAAACCGGATGATGCAATCCATTTCGAGAGTAAT

GGAAATTTCATTGCTCCAGAATATGCATACAAAATTGTCAAGAAAGGGGACTCAACAATT

ATGAAAAGTGGAGTGGAATATGGCCACTGCAACACCAAATGTCAAACCCCAGTAGGAGCG

ATAAATTCTAGTATGCCATTCCACAACATACATCCTCTCACCATTGGGGAATGCCCCAAA

TACGTGAAGTCAAACAAGTTGGTCCTTGCGACTGGGCTCAGAAATAGTCCTCTAAGAGAA

AAGAGAAGAAAAAGAGGCCTGTTTGGGGCGATAGCAGGGTTTATAGAGGGAGGATGGCAG

GGAATGGTTGATGGTTGGTATGGGTACCACCATAGCAATGAGCAGGGGAGTGGGTACGCT

GCAGACAAAGAATCCACCCAAAAGGCAATAGATGGAGTTACCAATAAGGTCAACTCAATC

ATTGACAAAATGAACACTCAATTTGAGGCAGTTGGAAGGGAGTTTAATAACTTAGAAAGG

AGGATAGAGAATTTGAACAAGAAAATGGAAGACGGATTCCTAGATGTCTGGACCTATAAT

GCTGAACTTCTAGTTCTCATGGAAAACGAGAGGACTCTAGATTTCCATGATTCAAATGTC

AAGAACCTTTACGACAAAGTCAGACTACAGCTTAGGGATAATGCAAAGGAGCTGGGTAAC

GGCTGTTTCGAATTCTATCACAAATGCGATAATGAATGTATGGAAAGTGTGAGAAATGGG

ACGTATGACTACCCTCAGTATTCAGAAGAAGCAAGATTAAAAAGAGAAGAAATAAGCGGA

GTGAAATTAGAATCAATAGGAACTTACCAGATACTGTCAATTTATTCAACAGCGGCGAGT

TCCCTAGCACTGGCAATCATGATGGCTGGTCTATCTTTATGGATGTGCTCCAATGGGTCG

TTACAGTGCAGAATTTGCATTTAA

>A_chicken_Omsk_0112_2020_EPI1813345

ATGGAGAACATAGTACTTCTTCTTGCAATAGTTAGCCTTGTTAAAAGTGATCAGATTTGC

ATTGGTTACCATGCAAACAATTCGACAGAGCAAGTTGACACGATAATGGAAAAGAACGTC

ACTGTTACACATGCCCAAGACATACTGGAAAAAACACACAACGGGAAGCTCTGTGATCTA

AATGGGGTGAAGCCTCTGATTTTAAAGGATTGTAGTGTAGCTGGATGGCTCCTCGGAAAC

CCAATGTGCGACGAATTCATCAGAGTGCCGGAATGGTCCTACATAGTGGAGAGGGCTAAT

CCAGCTAATGACCTCTGTTACCCAGGGAGCCTCAATGACTATGAAGAACTGAAACACCTG

TTGAGCAGAATAAATCATTTTGAGAAGATTCTGATCATCCCCAAGAGTTCCTGGCCAAAC

CATGAAACATCACTAGGGGTGAGCGCAGCTTGTCCATACCAGGGAGCGCCCTCCTTTTTC

AGAAATGTGGTGTGGCTTATCAAAAAGAACGATGCATACCCAACAATAAAGATAAGCTAC

AATAATACCAATCGGGAAGATCTCTTGATACTGTGGGGGATTCATCATTCCAACAATGCA

GAAGAGCAGACAAATCTCTATAAAAACCCAACCACCTACATTTCAGTTGGAACATCAACT

TTAAACCAGAGGTTGGTACCAAAAATAGCTACTAGATCCCAAGTAAACGGGCAACGTGGA

AGAATGGACTTCTTCTGGACAATTTTAAAACCGGATGATGCAATCCATTTCGAGAGTAAT

GGAAATTTCATTGCTCCAGAATATGCATACAAAATTGTCAAGAAAGGGGACTCAACAATT

ATGAAAAGTGGAGTGGAATATGGCCACTGCAACACCAAATGTCAAACCCCAGTAGGAGCG

ATAAATTCTAGTATGCCATTCCACAACATACATCCTCTCACCATTGGGGAATGCCCCAAA

TACGTGAAGTCAAACAAGTTGGTCCTTGCGACTGGGCTCAGAAATAGTCCTCTAAGAGAA

AAGAGAAGAAAAAGAGGCCTGTTTGGGGCGATAGCAGGGTTTATAGAGGGAGGATGGCAG

GGAATGGTTGATGGTTGGTATGGGTACCACCATAGCAATGAGCAGGGGAGTGGGTACGCT

GCAGACAAAGAATCCACCCAAAAGGCAATAGATGGAGTTACCAATAAGGTCAACTCAATC

ATTGACAAAATGAACACTCAATTTGAGGCAGTTGGAAGGGAGTTTAATAACTTAGAAAGG

AGGATAGAGAATTTGAACAAGAAAATGGAAGACGGATTCCTAGATGTCTGGACCTATAAT

GCTGAACTTCTAGTTCTCATGGAAAACGAGAGGACTCTAGATTTCCATGATTCAAATGTC

AAGAACCTTTACGACAAAGTCAGACTACAGCTTAGGGATAATGCAAAGGAGCTGGGTAAC

GGCTGTTTCGAATTCTATCACAAATGCGATAATGAATGTATGGAAAGTGTGAGAAATGGG

ACGTATGACTACCCTCAGTATTCAGAAGAAGCAAGATTAAAAAGAGAAGAAATAAGCGGA

GTGAAATTAGAATCAATAGGAACTTACCAGATACTGTCAATTTATTCAACAGCGGCGAGT

TCCCTAGCACTGGCAATCATGATGGCTGGTCTATCTTTATGGATGTGCTCCAATGGGTCG

TTACAGTGCAGAATTTGCATTTAA

>A_chicken_Kostroma_304-06_2020_EPI1848646

ATGGAGAACATAGTACTTCTTCTTGCAATAGTTAGCCTTGTTAAAAGTGATCAGATTTGC

ATTGGTTACCATGCAAACAATTCGACAGAGCAAGTTGACACGATAATGGAAAAGAACGTC

ACTGTTACACATGCCCAAGACATACTGGAAAAAACACACAACGGGAAGCTCTGTGATCTA

AATGGGGTGAAGCCTCTGATTTTAAAGGATTGTAGTGTAGCTGGATGGCTCCTCGGAAAC

CCAATGTGCGACGAATTCATCAGAGTGCCGGAATGGTCCTACATAGTGGAGAGGGCTAAT

CCAGCTAATGACCTCTGTTACCCAGGGAGCCTCAATGACTATGAAGAACTGAAACACCTG

TTGAGCAGAATAAATCATTTTGAGAAGATTCTGATCATCCCCAAGAGTTCCTGGCCAAAC

CATGAAACATCACTAGGGGTGAGCGCAGCTTGTCCATACCAGGGAGCGCCCTCCTTTTTC

AGAAATGTGGTGTGGCTTATCAAAAAGAACGATGCATACCCAACAATAAAGATAAGCTAC

AATAATACCAATCGGGAAGATCTCTTGATACTGTGGGGGATTCATCATTCCAACAATGCA

GAAGAGCAGACAAATCTCTATAAAAACCCAACCACCTACATTTCAGTTGGAACATCAACT

TTAAACCAGAGGTTGGTACCAAAAATAGCTACTAGATCCCAAGTAAACGGGCAACGTGGA

AGAATGGACTTCTTCTGGACAATTTTAAAACCGGATGATGCAATCCATTTCGAGAGTAAT

GGAAATTTCATTGCTCCAGAATATGCATACAAAATTGTCAAGAAAGGGGACTCAACAATT

ATGAAAAGTGGAGTGGAATATGGCCACTGCAACACCAAATGTCAAACCCCAGTAGGAGCG

ATAAATTCTAGTATGCCATTCCACAACATACATCCTCTCACCATTGGGGAATGCCCCAAA

TACGTGAAGTCAAACAAGTTGGTCCTTGCGACTGGGCTCAGAAATAGTCCTCTAAGAGAA

AAGAGAAGAAAAAGAGGCCTGTTTGGGGCGATAGCAGGGTTTATAGAGGGAGGATGGCAG

GGAATGGTTGATGGTTGGTATGGGTACCACCATAGCAATGAGCAGGGGAGTGGGTACGCT

GCAGACAAAGAATCCACCCAAAAGGCAATAGATGGAGTTACCAATAAGGTAAACTCAATC

ATTGACAAAATGAACACTCAATTTGAGGCAGTTGGAAGGGAGTTTAATAACTTAGAAAGG

AGGATAGAGAATTTGAACAAGAAAATGGAAGACGGATTCCTAGATGTCTGGACCTATAAT

GCTGAACTTCTAGTTCTCATGGAAAACGAGAGGACTCTAGATTTCCATGATTCAAATGTC

AAGAACCTTTACGACAAAGTCAGACTACAGCTTAGGGATAATGCAAAGGAGCTGGGTAAC

GGCTGTTTCGAATTCTATCACAAATGCGATAATGAATGTATGGAAAGTGTGAGAAATGGG

ACGTATGACTACCCTCAGTATTCAGAAGAAGCAAGATTAAAAAGAGAAGAAATAAGCGGA

GTGAAATTAGAATCAATAGGAACTTACCAGATACTGTCAATTTATTCAACAGCGGCGAGT

TCCCTAGCACTGGCAATCATGATGGCTGGTCTATCTTTATGGATGTGCTCCAATGGGTCG

TTACAGTGCAGAATTTGCATTTAA

>A_turkey_Rostov-on-Don_332-09_2021_EPI1848758

ATGGAGAACATAGTACTTCTTCTTGCAATAGTTAGCCTTGTTAAAAGTGATCAGATTTGC

ATTGGTTACCATGCAAACAATTCGACAGAGCAAGTTGACACGATAATGGAAAAGAACGTC

ACTGTCACACATGCCCAAGACATACTGGAAAAAACACACAACGGGAAGCTCTGTGATCTA

AATGGGGTGAAGCCTCTGATTTTAAAGGATTGTAGTGTAGCTGGATGGCTCCTCGGAAAC

CCAATGTGCGACGAATTCATCAGAGTGCCGGAATGGTCCTACATAGTGGAGAGGGCTAAT

CCAGCTAATGACCTCTGTTACCCAGGGAGCCTCAATGACTATGAAGAACTGAAACACCTG

TTGAGCAGAATAAATCATTTTGAGAAGATTCTGATCATCCCCAAGAGTTCCTGGCCAAAC

CATGAAACATCACTAGGGGTGAGCGCAGCTTGTCCATACCAGGGAGCGCCCTCCTTTTTC

AGAAATGTGGTGTGGCTTATCAAAAAGAACGATGCATACCCAACAATAAAGATAAGCTAC

AATAATACCAATCGGGAAGATCTCTTGATACTGTGGGGGATTCATCATTCCAACAATGCA

GAAGAGCAGACAAATCTCTATAAAAACCCAACCACCTACATTTCAGTTGGAACATCAACT

TTAAACCAGAGGTTGGTACCAAAAATAGCTACTAGATCCCAAGTAAACGGGCAACGTGGA

AGAATGGACTTTTTCTGGACAATTTTAAAACCGGATGATGCAATCCATTTCGAGAGTAAT

GGAAATTTCATTGCTCCAGAATATGCATACAAAATTGTCAAGAAAGGGGACTCAACAATT

ATGAAAAGTGGAGTGGAATATGGCCACTGCAACACCAAATGTCAAACCCCAGTAGGAGCG

ATAAATTCTAGTATGCCATTCCACAACATACATCCTCTCACCATTGGGGAATGCCCCAAA

TACGTGAAGTCAAACAAGTTGGTCCTTGCGACTGGGCTCAGAAATAGTCCTCTAAGAGAA

AAGAGAAGAAAAAGAGGCCTGTTTGGGGCGATAGCAGGGTTTATAGAGGGAGGATGGCAG

GGAATGGTTGATGGTTGGTACGGGTACCACCATAGCAATGAGCAGGGGAGTGGGTACGCT

GCAGACAAAGAATCCACCCAAAAGGCAATAGATGGAGTTACCAATAAGGTCAACTCAATC

ATTGACAAAATGAACACTCAATTTGAGGCAGTTGGAAGGGAGTTTAATAACTTAGAAAGG

AGGATAGAGAATTTGAACAAGAAAATGGAAGACGGATTCCTAGATGTCTGGACCTATAAT

GCTGAACTTCTAGTTCTCATGGAAAACGAGAGGACTCTAGATTTCCATGATTCAAATGTC

AAGAACCTTTACGACAAAGTCAGACTACAGCTTAGGGATAATGCAAAAGAGCTGGGTAAC

GGCTGTTTCGAATTCTATCACAAATGCGATAATGAATGTATGGAAAGTGTGAGAAATGGG

ACGTATGACTACCCTCAGTATTCAGAAGAAGCAAGATTAAAAAGAGAAGAAATAAGCGGA

GTGAAATTAGAATCAATAGGAACTTACCAGATACTGTCAATTTATTCAACAGCGGCGAGT

TCCCTAGCACTGGCAATCATGATGGCTGGTCTATCTTTATGGATGTGCTCCAATGGGTCG

TTACAGTGCAGAATTTGCATTTAA

>A_whooper_swan_Inner_Mongolia_W1-1_2020_EPI1811644

ATGGAGAACATAGTGCTTCTTCTTGCAATAGTTAGCCTTGTTAAAAGTGATCAGATTTGC

ATTGGTTACCATGCAAACAATTCGACAGAGCAAGTTGACACGATAATGGAAAAGAACGTC

ACTGTTACACATGCCCAAGACATACTGGAAAAAACACACAACGGGAAGCTCTGTGATCTA

AATGGGGTGAAGCCTCTGATTTTAAAGGATTGTAGTGTAGCTGGATGGCTCCTCGGAAAC

CCAATGTGCGACGAATTCATCAGAGTGCCGGAATGGTCCTACATAGTGGAGAGGGCTAAT

CCAGCTAATGACCTCTGTTACCCAGGGAGCCTCAATGACTATGAGGAACTGAAACACCTG

TTGAGCAGAATAAATCATTTTGAGAAGATTCTGATCATCCCCAAGAGTTCATGGCCAAAC

CATGAAACATCACTAGGGGTGAGCGCAGCTTGTCCATACCAGGGAGCGCCCTCCTTTTTC

AGAAATGTGGTGTGGCTTATCAAAAAGAACGATGCATACCCAACAATAAAGATAAGCTAC

AATAATACCAATCGGGAAGATCTCTTGATACTGTGGGGGATTCATCATTCCAACAATGCA

GAAGAGCAGATAAATCTCTATAAAAACCCAACCACCTACATTTCAGTTGGAACATCAACT

TTAAACCAGAGGTTGGTACCAAAAATAGCTACTAGATCCCAAGTAAACGGGCAACGTGGA

AGAATGGACTTCTTCTGGACAATTTTAAAACCGGATGATGCAATCCATTTCGAGAGTAAT

GGAAATTTCATTGCTCCAGAATATGCATACAAAATTGTCAAGAAAGGGGACTCAACAATT

ATGAAAAGTGGAGTGGAATATGGCCACTGCAACACCAAATGTCAAACCCCAGTAGGAGCG

ATAAATTCTAGTATGCCATTCCACAACATACATCCTCTCACCATTGGGGAATGCCCCAAA

TACGTGAAATCAAACAAGTTGGTCCTTGCGACCGGGCTCAGAAATAGTCCTCTAAGAGAA

AAGAGAAGAAAAAGAGGCCTGTTCGGGGCGATAGCAGGGTTTATAGAGGGAGGATGGCAG

GGAATGGTTGATGGTTGGTATGGGTACCACCATAGCAATGAGCAGGGGAGTGGGTACGCT

GCAGACAAAGAATCCACCCAAAAGGCAATAGATGGAGTTACCAATAAGGTCAACTCAATC

ATTGACAAAATGAACACTCAATTTGAGGCAGTTGGAAGGGAGTTTAATAACTTAGAAAGG

AGGATAGAGAATTTGAACAAGAAAATGGAAGACGGATTCCTAGATGTCTGGACCTATAAT

GCTGAACTTCTAGTTCTCATGGAAAACGAGAGGACTCTAGATTTCCATGATTCAAATGTC

AAGAACCTTTACGACAAAGTCAGACTACAGCTTAGGGATAATGCAAAGGAGCTGGGTAAC

GGCTGTTTCGAATTCTATCACAAATGCGATAATGAATGTATGGAAAGTGTGAGAAATGGG

ACGTATGACTACCCTCAGTATTCAGAAGAAGCAAGATTAAAAAGAGAAGAAATAAGCGGA

GTGAAATTAGAATCAATAGGAACTTACCAAATACTGTCAATTTATTCAACAGTGGCGAGT

TCCCTAGCACTGGCAATCATGGTGGCTGGTCTATCTTTATGGATGTGCTCCAATGGGTCG

TTACAGTGCAGAATTTGCATTTAA

>A_mute_swan_Inner_Mongolia_W2-1_2020_EPI1811652

ATGGAGAACATAGTGCTTCTTCTTGCAATAGTTAGCCTTGTTAAAAGTGATCAGATTTGC

ATTGGTTACCATGCAAACAATTCGACAGAGCAAGTTGACACGATAATGGAAAAGAACGTC

ACTGTTACACATGCCCAAGACATACTGGAAAAAACACACAACGGGAAGCTCTGTGATCTA

AATGGGGTGAAGCCTCTGATTTTAAAGGATTGTAGTGTAGCTGGATGGCTCCTCGGAAAC

CCAATGTGCGACGAATTCATCAGAGTGCCGGAATGGTCCTACATAGTGGAGAGGGCTAAT

CCAGCTAATGACCTCTGTTACCCAGGGAGCCTCAATGACTATGAGGAACTGAAACACCTG

TTGAGCAGAATAAATCATTTTGAGAAGATTCTGATCATCCCCAAGAGTTCATGGCCAAAC

CATGAAACATCACTAGGGGTGAGCGCAGCTTGTCCATACCAGGGAGCGCCCTCCTTTTTC

AGAAATGTGGTGTGGCTTATCAAAAAGAACGATGCATACCCAACAATAAAGATAAGCTAC

AATAATACCAATCGGGAAGATCTCTTGATACTGTGGGGGATTCATCATTCCAACAATGCA

GAAGAGCAGATAAATCTCTATAAAAACCCAACCACCTACATTTCAGTTGGAACATCAACT

TTAAACCAGAGGTTGGTACCAAAAATAGCTACTAGATCCCAAGTAAACGGGCAACGTGGA

AGAATGGACTTCTTCTGGACAATTTTAAAACCGGATGATGCAATCCATTTCGAGAGTAAT

GGAAATTTCATTGCTCCAGAATATGCATACAAAATTGTCAAGAAAGGGGACTCAACAATT

ATGAAAAGTGGAGTGGAATATGGCCACTGCAACACCAAATGTCAAACCCCAGTAGGAGCG

ATAAATTCTAGTATGCCACTCCACAACATACATCCTCTCACCATTGGGGAATGCCCCAAA

TACGTGAAATCAAACAAGTTGGTCCTTGCGACCGGGCTCAGAAATAGTCCTCTAAGAGAA

AAGAGAAGAAAAAGAGGCCTGTTCGGGGCGATAGCAGGGTTTATAGAGGGAGGATGGCAG

GGAATGGTTGATGGTTGGTATGGGTACCACCATAGCAATGAGCAGGGGAGTGGGTACGCT

GCAGACAAAGAATCCACCCAAAAGGCAATAGATGGAGTTACCAATAAGGTCAACTCAATC

ATTGACAAAATGAACACTCAATTTGAGGCAGTTGGAAGGGAGTTTAATAACTTAGAAAAG

AGGATAGAGAATTTGAACAAGAAAATGGAAGACGGATTCCTAGATGTCTGGACCTATAAT

GCTGAACTTCTAGTTCTCATGGAAAACGAGAGGACTCTAGATTTCCATGATTCAAATGTC

AAGAACCTTTACGACAAAGTCAGACTACAGCTTAGGGATAATGCAAAGGAGCTGGGTAAC

GGCTGTTTCGAATTCTATCACAAATGCGATAATGAATGTATGGAAAGTGTGAGAAATGGG

ACGTATGACTACCCTCAGTATTCAGAAGAAGCAAGATTAAAAAGAGAAGAAATAAGCGGA

GTGAAATTAGAATCAATAGGAACTTACCAGATACTGTCAATTTATTCAACAGCGGCGAGT

TCCCTAGCACTGGCAATCATGATGGCTGGTCTATCTTTATGGATGTGCTCCAATGGGTCG

TTACAGTGCAGAATTTGCATTTAA

>A_mallard_Korea_WA820_2020_EPI1846597

ATGGAGAACATAGTACTTCTTCTTGCAATAGTTAGCCTTGTTAAAAGTGATCAGATTTGC

ATTGGTTACCATGCAAACAATTCGACAGAGCAAGTTGACACGATAATGGAAAAGAACGTC

ACTGTTACACATGCCCAAGACATACTGGAAAAAACACACAACGGGAAGCTCTGTGATCTA

AATGGGGTGAAGCCTCTGATTTTAAAGGATTGTAGTGTAGCTGGATGGCTCCTCGGAAAC

CCAATGTGCGACGAATTCATCAGAGTGCCGGAATGGTCCTACATAGTGGAGAGGGCTAAT

CCAGCTAATGACCTCTGTTACCCAGGGAGCCTCAATGACTATGAAGAACTGAAACACCTG

TTGAGCAGAATAAATCATTTTGAGAAGATTCTGATCATCCCCAAGAGTTCATGGCCAAAC

CATGAAACATCACTAGGGGTGAGCGCAGCTTGTCCATACCAGGGAGCGCCCTCCTTTTTC

AGAAATGTGGTGTGGCTTGTCAAAAAGAACGATGCATACCCAACAATAAAGATAAGCTAC

AATAATACCAATCGGGAAGATCTCTTGATACTGTGGGGGATTCATCATTCCAACAATGCA

GAAGAGCAGATAAATCTCTATAAAAACCCAACCACCTACATTTCAGTTGGAACATCAACT

TTAAACCAGAGGTTGGTACCAAAAATAGCTACTAGATCCCAAGTAAACGGGCAACGTGGA

AGAATGGACTTCTTCTGGACAATTTTAAAACCGGATGATGCAATCCATTTCGAGAGTAAT

GGAAATTTCATTGCTCCAGAATATGCATACAAAATTGTCAAGAAAGGGGACTCAACAATT

ATGAAAAGTGGAGTGGAATATGGCCACTGCAACACCAAATGTCAAACCCCAGTAGGAGCG

ATAAATTCTAGTATGCCATTCCACAACATACATCCTCTCACCATTGGGGAATGCCCCAAA

TACGTGAAATCAAACAAGTTGGTCCTTGCGACTGGGCTCAGAAATAGTCCTCTAAGAGAA

AAGAGAAGAAAAAGAGGCCTGTTCGGGGCGATAGCAGGGTTTATAGAGGGAGGATGGCAG

GGAATGGTTGATGGTTGGTATGGGTACCACCATAGCAATGAGCAGGGGAGTGGGTACGCT

GCAGACAAAGAATCCACCCAAAAGGCAATAGATGGAGTTACCAATAAGGTCAACTCAATC

ATTGACAAAATGAACACTCAATTTGAGGCAGTTGGAAGGGAGTTTAATAACTTAGAAAGG

AGGATAGAGAATTTGAACAAGAAAATGGAAGACGGATTCCTAGATGTCTGGACCTATAAT

GCTGAACTTCTAGTTCTCATGGAAAACGAGAGGACTCTAGATTTCCATGATTCAAATGTC

AAGAACCTTTACGACAAAGTCAGACTACAGCTTAGGGATAATGCAAAGGAGCTGGGTAAC

GGCTGTTTCGAATTCTATCACAAATGCGATAATGAATGTATGGAAAGTGTGAGAAATGGG

ACGTATGACTACCCTCAGTATTCAGAAGAAGCAAGATTAAAAAGAGAAGAAATAAGCGGA

GTGAAATTAGAATCAATAGGAACTTACCAGATACTGTCAATTTATTCAACAGCGGCGAGT

TCCCTAGCACTGGCAATCATGATGGCTGGTCTATCTTTATGGATGTGCTCCAATGGGTCG

TTACAGTGCAGAATTTGCATTTAA

>A_duck_Korea_H016_2021_EPI1846706

ATGGAGAACATAGTACTTCTTCTTGCAATAGTTAGCCTTGTTAAAAGTGATCAGATTTGC

ATTGGTTACCATGCAAACAATTCGACAGAGCAAGTTGACACGATAATGGAAAAGAACGTC

ACTGTTACACATGCCCAAGACATACTGGAAAAAACACACAACGGGAAGCTCTGTGATCTA

AATGGGGTGAAGCCTCTGATTTTAAAGGATTGTAGTGTAGCTGGATGGCTCCTCGGAAAC

CCAATGTGCGACGAATTCATCAGAGTGCCGGAATGGTCCTACATAGTGGAGAGGGCTAAT

CCAGCTAATGACCTCTGTTACCCAGGGAGCCTCAATGACTATGAAGAACTGAAACACCTG

TTGAGCAGAATAAATCATTTTGAGAAGATTCTGATCATCCCCAAGAGTTCATGGCCAAAC

CATGAAACATCACTAGGGGTGAGCGCAGCTTGTCCATACCAGGGAGCGCCCTCCTTTTTC

AGAAATGTGGTGTGGCTTGTCAAAAAGAACGATGCATACCCAACAATAAAGATAAGCTAC

AATAATACCAATCGGGAAGATCTCTTGATACTGTGGGGGATTCATCATTCCAACAATGCA

GAAGAGCAGATAAATCTCTATAAAAACCCAACCACCTACATTTCAGTTGGAACATCAACT

TTAAACCAGAGGTTGGTACCAAAAATAGCTACTAGATCCCAAGTAAACGGGCAACGTGGA

AGAATGGACTTCTTCTGGACAATTTTAAAACCGGATGATGCAATCCATTTCGAGAGTAAT

GGAAATTTCATTGCTCCAGAATATGCATACAAAATTGTCAAGAAAGGGGACTCAACAATT

ATGAAAAGTGGAGTGGAATATGGCCACTGCAACACCAAATGTCAAACCCCAGTAGGAGCG

ATAAATTCTAGTATGCCATTCCACAACATACATCCTCTCACCATTGGGGAATGCCCCAAA

TACGTGAAATCAAACAAGTTGGTCCTTGCGACTGGGCTCAGAAATAGTCCTCTAAGAGAA

AAGAGAAGAAAAAGAGGCCTGTTCGGGGCGATAGCAGGGTTTATAGAGGGAGGATGGCAG

GGAATGGTTGATGGTTGGTATGGGTACCACCATAGCAATGAGCAGGGGAGTGGGTACGCT

GCAGACAAAGAATCCACCCAAAAGGCAATAGATGGAGTTACCAATAAGGTCAACTCAATC

ATTGACAAAATGAACACTCAATTTGAGGCAGTTGGAAGGGAGTTTAATAACTTAGAAAGG

AGGATAGAGAATTTGAACAAGAAAATGGAAGACGGATTCCTAGATGTCTGGACCTATAAT

GCTGAACTTCTAGTTCTCATGGAAAACGAGAGGACTCTAGATTTCCATGATTCAAATGTC

AAGAACCTTTACGACAAAGTCAGACTACAGCTTAGGGATAATGCAAAGGAGCTGGGTAAC

GGCTGTTTCGAATTCTATCACAAATGCGATAATGAATGTATGGAAAGTGTGAGAAATGGG

ACGTATGACTACCCTCAGTATTCAGAAGAAGCAAGATTAAAAAGAGAAGAAATAAGCGGA

GTGAAATTAGAATCAATAGGAACTTACCAGATACTGTCAATTTATTCAACAGCGGCGAGT

TCCCTAGCACTGGCAATCATGATGGCTGGTCTATCTTTATGGATGTGCTCCAATGGGTCG

TTACAGTGCAGAATTTGCATTTAA

>A_chicken_Korea_H008_2021_EPI1846538

ATGGAGAACATAGTACTTCTTCTTGCAATAGTTAGCCTTGTTAAAAGTGATCAGATCTGC

ATTGGTTACCATGCAAACAATTCGACAGAGCAAGTTGACACGATAATGGAAAAGAACGTC

ACTGTTACACATGCCCAAGACATACTGGAAAAAACACACAACGGGAAGCTCTGTGATCTA

AATGGGGTGAAGCCTCTGATTTTAAAGGATTGTAGTGTAGCTGGATGGCTCCTCGGAAAC

CCAATGTGCGACGAATTCATCAGAGTGCCGGAATGGTCCTACATAGTGGAGAGGGCTAAT

CCAGCTAATGACCTCTGTTACCCAGGGAGCCTCAATGACTATGAAGAACTGAAACACCTG

TTGAGCAGAATAAATCATTTTGAGAAGATTCTGATCATCCCCAAGAGTTCTTGGCCAAAC

CATGAAACATCACTAGGGGTGAGCGCAGCTTGTCCATACCAGGGAGCGCCCTCCTTTTTC

AGAAATGTGGTGTGGCTTATCAAAAAGAACGATGCATACCCAACAATAAAGATAAGCTAC

AATAATACCAATCGGGAAGATCTCTTGATACTGTGGGGGATTCACCATTCCAACAATGCA

GAAGAGCAGATAAATCTCTATAAAAACCCAACCACCTACATTTCAGTTGGAACATCAACT

TTAAACCAGAGGTTGGTACCAAAAATAGCTACTAGATCCCAAGTAAACGGGCAACGTGGA

AGAATGGACTTCTTCTGGACAATTTTAAAACCGGATGATGCAATCCATTTCGAGAGTAAT

GGAAATTTCATTGCTCCAGAATATGCATACAAAATTGTCAAGAAAGGGGACTCAACAATT

ATGAAAAGTGGAGTAGAATATGGCCACTGCAACACCAAATGTCAAACCCCAGTAGGAGCG

ATAAATTCTAGTATGCCATTCCACAACATACATCCTCTCACCATTGGGGAATGCCCCAAA

TACGTGAAATCAAACAAGTTGGTCCTTGCGACTGGGCTCAGAAATAGTCCTCTAAGAGAA

AAGAGAAGAAAAAGAGGCCTGTTTGGGGCGATAGCAGGGTTTATAGAGGGAGGATGGCAG

GGAATGGTTGATGGTTGGTATGGGTACCACCATAGCAATGAGCAGGGGAGTGGGTACGCT

GCAGACAAAGAATCCACCCAAAAGGCAATAGATGGAGTTACCAATAAGGTCAACTCAATC

ATTGACAAAATGAACACTCAATTTGAGGCAGTTGGAAGGGAGTTTAATAACTTAGAAAGG

AGGATAGAGAATTTGAACAAGAAAATGGAAGACGGATTCCTAGATGTCTGGACCTATAAT

GCTGAACTTCTAGTTCTCATGGAAAACGAGAGGACTCTAGATTTCCATGATTCAAATGTC

AAGAACCTTTACGACAAAGTCAGACTACAGCTTAGGGATAATGCAAAGGAGCTGGGTAAC

GGCTGTTTCGAATTCTATCACAAATGCGATAATGAATGTATGGAAAGTGTGAGAAATGGG

ACATATGACTACCCTCAGTATTCAGAAGAAGCAAGATTAAAAAGAGAAGAAATAAGCGGA

GTGAAATTAGAATCAATAGGAACTTACCAGATACTGTCAATTTATTCAACAGCGGCGAGT

TCCCTAGCACTGGCAATCATGATGGCTGGTCTATCTTTATGGATGTGCTCCAATGGGTCG

TTACAGTGCAGAATTTGCATTTAA

>A_crane_Kagoshima_KU-93_2021_EPI1848527

ATGGAGAACATAGTACTTCTTCTTGCAATAGTTAGCCTTGTTAAAAGTGATCAGATTTGC

ATTGGTTACCATGCAAACAATTCGACAGAGCAAGTTGACACGATAATGGAAAAGAACGTC

ACTGTTACACATGCCCAAGACATACTGGAAAAAACACACAACGGGAAGCTCTGTGATCTA

AATGGGGTGAAGCCTCTGATTTTAAAGGATTGTAGTGTAGCGGGATGGCTCCTCGGAAAC

CCAATGTGCGACGAATTCATCAGAGTGCCGGAATGGTCCTACATAGTGGAGAGGGCTAAT

CCAGCTAATGACCTCTGTTACCCAGGGAGCCTCAATGACTATGAAGAACTGAAACACCTG

TTGAGCAGAATAAATCATTTTGAGAAGATTCTGATCATCCCCAAGAGTTCCTGGCCAAAC

CATGAAACATCACTAGGGGTGAGCGCAGCTTGTCCATACCAGGGAGCGCCCTCCTTTTTC

AGAAATGTGGTGTGGCTTATCAAAAAGAACGATGCATACCCAACAATAAAGATAAGCTAC

AATAATACCAATCGGGAAGATCTCTTGATACTGTGGGGGATTCACCATTCCAATAATGCA

GAAGAGCAGATAAATCTCTATAAAAACCCAACCACCTACATTTCAGTTGGAACATCAACT

TTAAACCAGAGGTTGGTACCAAAAATAGCTACTAGATCCCAAGTAAACGGGCAACGTGGA

AGAATGGACTTCTTCTGGACAATTTTAAAACCGGATGATGCAATCCATTTCGAGAGTAAT

GGAAATTTCATTGCTCCAGAATATGCATACAAAATTGTCAAGAAAGGGGACTCAACAATT

ATGAAAAGTGGAGTGGAATATGGCCACTGCAACACCAAATGTCAAACCCCAGTAGGAGCG

ATAAATTCTAGTATGCCATTCCACAACATACATCCTCTCACCATTGGGGAATGCCCCAAA

TACGTGAAATCAAACAAGTTGGTCCTTGCGACTGGGCTCAGAAATAGTCCTCTAAGAGAA

AAGAGAAGAAAAAGAGGCCTGTTTGGGGCGATAGCAGGGTTTATAGAGGGAGGATGGCAG

GGAATGGTTGATGGTTGGTATGGGTACCACCATAGCAATGAGCAGGGNAGTGGGTACGCT

GCAGACAAAGAATCCACCCAAAAGGCAATAGATGGAGTTACCAATAAGGTCAACTCAATC

ATTGACAAAATGAACACTCAATTTGAGGCAGTTGGAAGGGAGTTTAATAACTTAGAAAGG

AGGATAGAGAATTTGAACAAGAAAATGGAAGACGGATTCCTAGATGTCTGGACCTATAAT

GCTGAACTTCTAGTTCTCATGGAAAACGAGAGGACTCTAGATTTCCATGATTCAAATGTC

AAGAACCTTTACGACAAAGTCAGACTACAGCTTAGGGATAATGCAAAGGAGCTGGGTAAC

GGCTGTTTCGAATTCTATCACAAATGCGATAATGAATGTATGGAAAGTGTGAGAAATGGG

ACGTATGACTACCCTCAGTATTCAGAAGAAGCAAGATTAAAAAGAGAAGAAATAAGCGGA

GTGAAATTAGAATCAATAGGAACTTACCAGATACTGTCAATTTATTCAACAGCGGCGAGT

TCCCTAGCACTGGCAATCATGATGGCTGGTCTATCTTTATGGATGTGCTCCAATGGGTCG

TTACAGTGCAGAATTTGCATTTAA

>A_mallard_Kagoshima_KU-D89_2021_EPI1848538

ATGGAGAACATAGTACTTCTTCTTGCAATAGTTGGCCTTGTTAAAAGTGATCAGATTTGC

ATTGGTTACCATGCAAACAATTCGACAGAGCAAGTTGACACGATAATGGAAAAGAACGTC

ACTGTTACACATGCCCAAGACATACTGGAAAAAACACACAACGGGAAGCTCTGTGATCTA

AATGGGGTGAAGCCTCTGATTTTAAAGGATTGTAGTGTAGCTGGATGGCTCCTCGGAAAC

CCAATGTGCGACGAATTCATCAGAGTGCCGGAATGGTCCTACATAGTGGAGAGGGCTAAT

CCAGCTAATGACCTCTGTTACCCAGGGAGCCTCAATGACTATGAAGAACTGAAACACCTG

TTGAGCAGAATAAATCATTTTGAGAAGATTCTGATCATCCCCAAGAGTTCCTGGCCAAAC

CATGAAACATCACTAGGGGTGAGCGCAGCTTGTCCATACCAGGGAGCGCCCTCCTTTTTC

AGAAATGTGGTGTGGCTTATCAAAAAGAACGATGCATACCCAACAATAAAGATAAGCTAC

AATAATACCAATCGGGAAGATCTCTTGATACTGTGGGGGATTCACCATTCCAACAATGCA

GAAGAGCAGATAAATCTCTATAAAAACCCAACCACCTACATTTCAGTTGGAACATCAACT

TTAAACCAGAGGTTGGTACCAAAAATAGCTACTAGATCCCAAGTAAACGGGCAACGTGGA

AGAATGGACTTCTTCTGGACAATTTTAAAACCGGATGATGCAATCCATTTCGAGAGTAAT

GGAAATTTCATTGCTCCAGAATATGCATACAAAATTGTCAAGAAAGGGGACTCAACAATT

ATGAAAAGTGGAGTGGAATATGGCCACTGCAACACCAAATGTCAAACCCCAGTAGGAGCG

ATAAATTCTAGTATGCCATTCCACAACATACATCCTCTCACCATTGGGGAATGCCCCAAA

TACGTGAAATCAAACAAGTTGGTCCTTGCGACTGGGCTCAGAAATAGTCCTCTAAGAGAA

AAGAGAAGAAAAAGAGGCCTGTTTGGGGCGATAGCAGGGTTTATAGAGGGAGGATGGCAG

GGAATGGTTGATGGTTGGTATGGGTACCACCATAGCAATGAGCAGGGGAGTGGGTACGCT

GCAGACAAAGAATCCACCCAAAAGGCAATAGATGGAGTTACCAATAAGGTCAACTCAATC

ATTGACAAAATGAACACTCAATTTGAGGCAGTTGGAAGGGAGTTTAATAACTTAGAAAGG

AGGATAGAGAATTTGAACAAGAAAATGGAAGACGGATTCCTAGATGTCTGGACCTATAAT

GCTGAACTTCTAGTTCTCATGGAAAACGAGAGGACTCTAGATTTCCATGATTCAAATGTC

AAGAACCTTTACGACAAAGTCAGACTACAGCTTAGGGATAATGCAAAGGAGCTGGGTAAC

GGCTGTTTCGAATTCTATCACAAGTGCGATAATGAATGTATGGAAAGTGTGAGAAATGGG

ACGTATGACTACCCTCAGTATTCAGAAGAAGCAAGATTAAAAAGAGAAGAAATAAGCGGA

GTGAAATTAGAATCAATAGGAACTTACCAGATACTGTCAATTTATTCAACAGCGGCGAGT

TCCCTAGCACTGGCAATCATGATGGCTGGTCTATCTTTATGGATGTGCTCCAATGGGTCG

TTACAGTGCAGAATTTGCATTTAA

>A_chicken_Czech_Republic_1566-1_2021_EPI1844083

ATGGAGAACATAGTACTTCTTCTTGCAATAGTTAGCCTTGTTAAAAGTGATCAGATTTGC

ATTGGTTACCATGCAAACAATTCGACAGAGCAAGTTGACACGATAATGGAAAAGAACGTC

ACTGTTACACATGCCCAAGACATACTGGAAAAAACACACAACGGGAAGCTCTGTGATCTA

AATGGGGTGAAGCCTCTGATTTTAAAGGATTGTAGTGTAGCTGGATGGCTCCTCGGAAAC

CCAATGTGCGACGAATTCATCAGAGTGCCGGAATGGTCCTACATAGTGGAGAGGGCTAAT

CCATCTAATGACCTCTGTTACCCAGGGAGCCTCAATGACTATGAAGAACTGAAACACCTG

TTGAGCAGAATAAATCATTTTGAGAAGATTCTGATCATCCCCAAGAGTTCCTGGCCAAAC

CATGAAACATCACTAGGGGTGAGCGCAGCTTGTCCATACCAGGGAGCGCCCTCCTTTTTC

AGAAATGTGGTGTGGCTTATCAAAAAGAACGATGCATACCCAACAATAAAGATAAGCTAC

AATAATACCAATCAGGAAGATCTCTTGATACTGTGGGGGATTCATCATTCCAACAATGCA

GAAGAGCAGACAAATCTCTATAAAAACCCAACCACCTACATTTCAGTTGGAACATCAACT

TTAAACCAGAGGTTGGTACCAAAAATAGCTACTAGATCCCAAGTAAACGGGCAACGTGGA

AGAATGGACTTCTTCTGGACAATTTTAAAACCGGATGATGCAATCCATTTCGAGAGTAAT

GGAAATTTCATTGCTCCAGAATATGCATACAAAATTGTCAAGAAAGGGGACTCAACAATT

ATGAAAAGTGGAGTGGAATATGGCCACTGCAACACTAAATGTCAAACCCCAGTAGGAGCG

ATAAATTCTAGTATGCCATTCCACAACATACATCCTCTCACCATTGGGGAATGCCCCAAA

TACGTGAAGTCAAACAAGTTGGTCCTTGCGACTGGGCTCAGAAATAGTCCTCTAAGAGAA

AAGAGAAGAAAAAGAGGCCTGTTTGGGGCGATAGCAGGGTTTATAGAGGGAGGATGGCAG

GGAATGGTTGATGGTTGGTATGGGTACCACCATAGCAATGAGCAGGGGAGTGGGTACGCT

GCAGACAAAGAATCCACCCAAAAGGCAATAGATGGAGTTACCAATAAGGTCAACTCAATC

ATTGACAAAATGAACACTCAATTTGAGGCAGTTGGAAGGGAGTTTAATAACTTAGAAAGG

AGGATAGAGAATTTGAACAAGAAAATGGAAGACGGATTCCTAGATGTCTGGACCTATAAT

GCTGAACTTCTAGTTCTCATGGAAAACGAAAGGACTCTAGATTTCCATGATTCAAATGTC

AAGAACCTTTACGACAAAGTCAGACTACAGCTTAGGGATAATGCAAAGGAACTGGGTAAC

GGCTGTTTCGAATTCTATCACAAATGCGATAATGAATGTATGGAAAGTGTGAGAAATGGG

ACGTATGACTACCCTCAGTATTCAGGAGAAGCAAGATTAAAAAGAGAAGAAATAAGCGGA

GTGAAACTAGAATCAATAGGAACTTACCAGATACTGTCAATTTATTCAACAGCGGCGAGT

TCCCTAGCACTGGCAATCATGATGGCTGGTCTATCTTTATGGATGTGCTCCAATGGGTCG

TTACAGTGCAGAATTTGCATTTAA

>A_mute_swan_North_Ossetia-Alania_325-03_2020_EPI1848734

ATGGAGAACATAGTACTTCTTCTTGCAATAGTTAGCCTTGTTAAAAGTGATCAGATTTGC

ATTGGTTACCATGCAAACAATTCGACAGAGCAAGTTGACACGATAATGGAAAAAAACGTC

ACTGTTACACATGCCCAAGACATACTGGAAAAAACACACAACGGGAAGCTCTGTGATCTA

AATGGGGTGAAGCCTCTGATTTTAAAGGATTGTAGTGTAGCTGGATGGCTCCTCGGAAAC

CCAATGTGCGACGAATTCATCAGAGTGCCGGAATGGTCCTACATAGTGGAGAGGGCTAAT

CCATCTAATGACCTCTGTTACCCAGGGAGCCTCAATGACTATGAAGAACTGAAACACCTG

TTGAGCAGAATAAATCATTTTGAGAAGATTCTGATCATCCCCAAGAGTTCTTGGCCAAAC

CATGAAACATCACTAGGGGTGAGCGCAGCTTGTCCATACCAGGGAGCGCCCTCCTTTTTC

AGAAATGTGGTGTGGCTTATCAAAAAGAACGATGCATACCCAACAATAAAGATAAGCTAC

AATAATACCAATCGGGAAGATCTCTTGATACTGTGGGGGATTCATCATTCCAACAATGCA

GAAGAGCAGACAAATCTCTATAAAAACCCAACCACCTACATTTCAGTTGGAACATCAACT

TTAAACCAGAGGTTGGTACCAAAAATAGCTACTAGATCCCAAGTAAACGGGCAACGTGGA

AGAATGGACTTCTTCTGGACAATTTTAAAACCGGATGATGCAATCCATTTCGAGAGTAAT

GGAAATTTCATTGCTCCAGAATATGCATACAAAATTGTCAAGAAAGGGGACTCAACAATT

ATGAAAAGTGGAGTGGAATATGGCCACTGCAACACCAAATGTCAAACCCCAGTAGGAGCG

ATAAATTCTAGTATGCCATTCCACAACATACATCCTCTCACCATTGGGGAATGCCCCAAA

TACGTGAAGTCAAACAAGTTGGTCCTTGCGACTGGGCTCAGAAATAGTCCTCTAAGAGAA

AAGAGAAGAAAAAGAGGCCTGTTTGGGGCGATAGCAGGGTTTATAGAGGGAGGATGGCAG

GGAATGGTTGATGGTTGGTATGGGTACCACCATAGCAATGAGCAGGGGAGTGGGTACGCT

GCAGACAAAGAATCCACCCAAAAGGCAATAGATGGAGTTACCAATAAGGTCAACTCAATC

ATTGACAAAATGAACACTCAATTTGAGGCAGTTGGAAGGGAGTTTAATAACTTAGAAAGG

AGGATAGAGAATTTGAACAAGAAAATGGAAGACGGATTCCTAGATGTCTGGACCTATAAT

GCTGAACTTCTAGTTCTCATGGAAAACGAAAGGACTCTAGATTTCCATGATTCAAATGTC

AAGAACCTTTACGACAAAGTCAGACTACAGCTTAGGGATAATGCAAAGGAGCTGGGTAAC

GGCTGTTTCGAATTCTATCACAAATGCGATAATGAATGTATGGAAAGTGTGAGAAATGGG

ACGTATGACTACCCTCAGTATTCAGAAGAAGCAAGATTAAAAAGAGAAGAAATAAGCGGA

GTGAAATTAGAATCAATAGGAACTTACCAGATACTGTCAATTTATTCAACAGCGGCGAGT

TCCCTAGCACTGGCAATCATGATGGCTGGTCTATCTTTATGGATGTGCTCCAATGGGTCG

TTACAGTGCAGAATTTGCATTTAA

>A_mute_swan_Kazakhstan_1-267-20-B_2020_EPI1811584

ATGGAGAACATAGTACTTCTTCTTGCAATAGTTAGCCTTGTTAAAAGTGATCAGATTTGC

ATTGGTTACCATGCAAACAATTCGACAGAGCAAGTTGACACGATAATGGAAAAGAACGTC

ACTGTTACACATGCCCAAGACATACTGGAAAAAACACACAACGGGAAGCTCTGTGATCTA

AATGGGGTGAAGCCTCTAATTTTAAAGGATTGTAGTGTAGCTGGATGGCTCCTCGGAAAT

CCAATGTGCGACGAATTCATCAGAGTGCCGGAATGGTCCTACATAGTGGAGAGGGCTAAT

CCAGCTAATGACCTCTGTTACCCAGGGAGCCTCAATGACTATGAAGAACTGAAACACCTG

TTGAGCAGAATAAATCATTTTGAGAAGATTCTGATCATCCCCAAGAGTTCCTGGCCAAAT

CATGAAACATCACTAGGGGTGAGCGCAGCTTGTCCATACCAGGGAGCGCCCTCCTTTTTC

AGAAATGTGGTGTGGCTTATCAAAAAGAACGATGCATACCCAACAATAAAGATAAGCTAC

AATAATACCAATCGGGAAGATCTCTTGATACTGTGGGGGATTCATCATTCCAACAATGCA

GAAGAGCAGACAAATCTCTATAAAAACCCAACCACCTACATTTCAGTTGGAACATCAACT

TTAAACCAGAGGTTGGTACCAAAAATAGCTACTAGATCCCAAGTAAACGGGCAACGTGGA

AGAATGGACTTCTTCTGGACAATTTTAAAACCGGATGATGCAATCCATTTCGAGAGTAAT

GGAAATTTCATTGCTCCAGAATATGCATACAAAATTGTCAAGAAAGGGGACTCAACAATT

ATGAAAAGTGGAGTGGAATATGGCCACTGCAACACCAAATGTCAAACCCCAGTAGGAGCG

ATAAATTCTAGTATGCCATTCCACAACATACATCCTCTCACCATTGGGGAATGTCCCAAA

TACGTGAAGTCAAACAAGTTGGTCCTTGCGACTGGGCTCAGAAATAGTCCTCTAAGAGAA

AAGAGAAGAAAAAGAGGCCTGTTTGGGGCGATAGCAGGGTTTATAGAGGGAGGATGGCAG

GGAATGGTTGATGGTTGGTATGGGTACCACCATAGCAATGAGCAGGGGAGTGGGTACGCT

GCAGACAAAGAATCCACCCAAAAGGCAATAGATGGAGTTACCAATAAGGTCAACTCAATC

ATTGACAAAATGAACACTCAATTTGAGGCAGTTGGAAGGGAGTTTAATAACTTAGAAAGG

AGGATAGAGAATTTGAACAAGAAAATGGAAGACGGATTCCTAGATGTCTGGACCTATAAT

GCTGAACTTCTAGTTCTCATGGAAAACGAGAGGACTCTAGATTTCCATGATTCGAATGTC

AAGAACCTTTACGACAAAGTCAGACTACAGCTTAGGGATAATGCAAAGGAGCTGGGTAAC

GGCTGTTTCGAATTCTATCACAAATGCGATAATGAATGTATGGAAAGTGTGAGAAATGGG

ACGTATGACTACCCTCAGTATTCAGAAGAAGCAAGATTAAAAAGAGAAGAAATAAGCGGA

GTGAAATTAGAATCAATAGGAACTTACCAGATACTGTCAATTTATTCAACAGCGGCGAGT

TCCCTAGCACTGGCAATCATGATGGCTGGTCTATCTTTATGGATGTGCTCCAATGGGTCG

TTACAGTGCAGAATTTGCATTTAA

>A_goose_Omsk_0002_2020_EPI1813121

ATGGAGAACATAGTACTTCTTCTTGCAATAGTTAGCCTTGTTAAAAGTGATCAGATTTGC

ATTGGTTACCATGCAAACAATTCGACAGAGCAAGTTGACACGATAATGGAAAAGAACGTC

ACTGTTACACATGCCCAAGACATACTGGAAAAAACACACAACGGGAAGCTCTGTGATCTA

AATGGGGTGAAGCCTCTAATTTTAAAGGATTGTAGTGTAGCTGGATGGCTCCTCGGAAAT

CCAATGTGCGACGAATTCATCAGAGTGCCGGAATGGTCCTACATAGTGGAGAGGGCTAAT

CCAGCTAATGACCTCTGTTACCCAGGGAGCCTCAATGACTATGAAGAACTGAAACACCTG

TTGAGCAGAATAAATCATTTTGAGAAGATTCTGATCATCCCCAAGAGTTCCTGGCCAAAT

CATGAAACATCACTAGGGGTGAGCGCAGCTTGTCCATACCAGGGAGCGCCCTCCTTTTTC

AGAAATGTGGTGTGGCTTATCAAAAAGAACGGTGCATACCCAACAATAAAGATAAGCTAC

AATAATACCAATCGGGAAGATCTCTTGATACTGTGGGGGATTCATCATTCCAACAATGCA

GAAGAGCAGACAAATCTCTATAAAAACCCAACCACCTACATTTCAGTTGGAACATCAACT

TTAAACCAGAGGTTGGTACCAAAAATAGCTACTAGATCCCAAGTAAACGGGCAACGTGGA

AGAATGGACTTCTTCTGGACAATTTTAAAACCGGATGATGCAATCCATTTCGAGAGTAAT

GGAAATTTCATTGCTCCAGAATATGCATACAAAATTGTCAAGAAAGGGGACTCAACAATT

ATGAAAAGTGGAGTGGAATATGGCCACTGCAACACCAAATGTCAAACCCCAGTAGGAGCG

ATAAATTCTAGTATGCCATTCCACAACATACATCCTCTCACCATTGGGGAATGCCCCAAA

TACGTGAAGTCAAACAAGTTGGTCCTTGCGACTGGGCTCAGAAATAGTCCTCTAAGAGAA

AAGAGAAGAAAAAGAGGCCTGTTTGGGGCGATAGCAGGGTTTATAGAGGGAGGATGGCAG

GGAATGGTTGATGGTTGGTATGGGTACCACCATAGCAATGAGCAGGGGAGTGGGTACGCT

GCAGACAAAGAATCCACCCAAAAAGCAATAGATGGAGTTACCAATAAGGTCAACTCAATC

ATTGACAAAATGAACACTCAATTTGAGGCAGTTGGAAGGGAGTTTAATAACTTAGAAAGG

AGGATAGAGAATTTGAACAAGAAAATGGAAGACGGATTCCTAGATGTCTGGACCTATAAT

GCTGAACTTCTAGTTCTCATGGAAAACGAGAGGACTCTAGATTTCCATGATTCGAATGTC

AAGAACCTTTACGACAAAGTCAGACTACAGCTTAGGGATAATGCAAAGGAGCTGGGTAAC

GGCTGTTTCGAATTCTATCACAAATGCGATAATGAATGTATGGAAAGTGTGAGAAATGGG

ACGTATGACTACCCTCAGTATTCAGAAGAAGCAAGATTAAAAAGAGAAGAAATAAGCGGA

GTGAAATTAGAATCAATAGGAACTTACCAGATACTGTCAATTTATTCAACAGCGGCGAGT

TCCCTAGCACTGGCAATCATGATGGCTGGTCTATCTTTATGGATGTGCTCCAATGGGTCG

TTACAGTGCAGAATTTGCATTTAA

>A_goose_Omsk_30003_2020_EPI1814289

ATGGAGAACATAGTACTTCTTCTTGCAATAGTTAGTCTTGTTAAAAGTGATCAGATTTGC

ATTGGTTACCATGCAAACAATTCGACAGAGCAAGTTGACACGATAATGGAAAAGAACGTC

ACTGTTACACATGCCCAAGACATACTGGAAAAAACACACAACGGGAAGCTCTGTGATCTA

AATGGGGTGAAGCCTCTGATTTTAAAGGATTGTAGTGTAGCTGGATGGCTCCTCGGAAAT

CCAATGTGCGACGAATTCATCAGAGTGCCGGAATGGTCCTACATAGTGGAGAGGGCTAAT

CCAGCTAATGACCTCTGTTACCCAGGGAGCCTCAATGACTATGAAGAACTGAAACACCTG

TTGAGCAGAATAAATCATTTTGAGAAGATTCTGATCATCCCCAAGAGTTCCTGGCCAAAT

CATGAAACATCACTAGGGGTGAGCGCAGCATGTCCATACCAGGGAGCGCCCTCCTTTTTC

AGAAATGTGGTGTGGCTTATCAAAAAGAACGATGAATACCCAACAATAAAGATAAGCTAC

AATAATACCAATCGGGAAGATCTCTTGATACTGTGGGGGATTCATCATTCCAACAATGCA

GAAGAGCAGACAAATCTCTATAAAAACCCAACCACCTACATTTCAGTTGGAACATCAACT

TTAAACCAGAGGTTGGTACCAAAAATAGCTACTAGATCCCAAGTAAACGGACAACGTGGA

AGAATGGACTTCTTCTGGACAATTTTAAAACCGGATGATGCAATCCATTTCGAGAGTAAT

GGAAATTTCATTGCTCCAGAATATGCATACAAAATTGTCAAGAAAGGGGACTCAACAATT

ATGAAAAGTGGAGTGGAATATGGCCACTGCAACACCAAATGTCAAACCCCAGTAGGAGCG

ATAAATTCTAGTATGCCATTCCACAACATACATCCTCTCACCATTGGGGAATGCCCCAAA

TACGTGAAGTCAAACAAGTTGGTCCTTGCGACTGGGCTCAGAAATAGTCCTCTAAGAGAA

AAGAGAAGAAAAAGAGGCCTGTTTGGGGCGATAGCAGGGTTTATAGAGGGAGGATGGCAG

GGAATGGTTGATGGTTGGTATGGGTACCACCATAGCAATGAGCAGGGGAGTGGGTACGCT

GCAGACAAAGAATCCACCCAAAAGGCAATAGATGGAGTTACCAATAAGGTCAACTCAATC

ATTGACAAAATGAACACTCAATTTGAGGCAGTTGGAAGGGAGTTTAATAACTTAGAAAGG

AGGATAGAGAATTTGAACAAGAAAATGGAAGACGGATTCCTAGATGTCTGGACCTATAAT

GCTGAACTTCTAGTTCTCATGGAAAACGAGAGGACTCTAGATTTCCATGATTCAAATGTC

AAGAACCTTTACGACAAAGTCAGACTACAGCTTAGGGATAATGCAAAGGAGCTGGGTAAC

GGCTGTTTCGAATTCTATCACAAATGCGATAATGAATGTATGGAAAGTGTGAGAAATGGG

ACGTATGACTACCCTCAGTATTCAGAAGAAGCAAGATTAAAAAGAGAAGAAATAAGCGGA

GTGAAATTAGAATCAATAGGAACTTACCAGATACTGTCAATTTATTCAACAGCGGCGAGT

TCCCTAGCACTGGCAATCATGATGGCTGGTCTATCTTTATGGATGTGCTCCAATGGGTCG

TTACAGTGCCGAATTTGCATTTAA

>A_duck_Kazakhstan_12-20-B-Talg-11_2020_EPI1882548

ATGGAGAACATAGTACTTCTTCTTGCAATAGTTAGCCTTGTTAAAAGTGATCAGATTTGC

ATTGGTTACCATGCAAACAATTCGACAGAGCAAGTTGACACGATAATGGAAAAGAACGTC

ACTGTTACACATGCCCAAGACATACTGGAAAAAACACACAACGGGAAGCTCTGTGATCTA

AATGGGGTGAAGCCTCTGATTTTAAAGGATTGTAGTGTAGCTGGATGGCTCCTCGGAAAC

CCAATGTGCGACGAATTCATCAGAGTGCCGGAATGGTCCTACATAGTGGAGCGGGCTAAT

CCAGCTAATGACCTCTGTTACCCAGGGAGCCTCAATGACTATGAAGAACTGAAACACCTG

TTGAGCAGAATAAATCATTTTGAGAAGATTCTGATCATCCCCAAGAGTTCCTGGCCAAAT

CATGAAACATCACTAGGGGTGAGCGCAGCTTGTCCATACCAGGGAGCGCCCTCCTTTTTC

AGAAATGTGGTGTGGCTTATCAAAAAGAACGATGCATACCCAACAATAAAGATAAGCTAC

AATAATACCAATCGGGAAGATCTCTTGATACTGTGGGGGATTCATCATTCCAACAATGCA

GAAGAGCAGACAAATCTCTATAAAAACCCAACCACCTACATTTCAGTTGGAACATCAACT

TTAAACCAGAGGTTGGTACCAAAAATAGCTACTAGATCCCAAGTAAACGGGCAACGTGGA

AGAATGGACTTCTTCTGGACAATTTTAAAACCGGATGATGCAATCCATTTCGAGAGTAAT

GGAAATTTCATTGCTCCAGAATATGCATACAAAATTGTCAAGAAAGGGGACTCAACAATT

ATGAAAAGTGGAGTGGAATATGGCCACTGCAACACCAAATGTCAAACCCCAGTAGGAGCG

ATAAATTCTAGTATGCCATTCCACAACATACATCCTCTCACCATTGGGGAATGCCCCAAA

TACGTGAAGTCAAACAAGTTGGTCCTTGCGACTGGGCTCAGAAATAGTCCTCTAAGAGAA

AAGAGAAGAAAAAGAGGCCTGTTTGGGGCGATAGCAGGGTTTATAGAGGGAGGATGGCAG

GGAATGGTTGATGGTTGGTATGGGTACCATCATAGCAATGAGCAGGGGAGTGGGTACGCT

GCAGACAAAGAATCCACCCAAAAGGCAATAGATGGAGTTACCAATAAGGTCAACTCAATC

ATTGACAAAATGAACACTCAATTTGAGGCAGTTGGAAGGGAGTTTAATAACTTAGAAAGG

AGGATAGAGAATTTGAACAAGAAAATGGAAGACGGATTCCTAGATGTCTGGACCTATAAT

GCTGAACTTCTAGTTCTCATGGAAAACGAGAGGACTCTAGATTTCCATGATTCAAATGTC

AAGAACCTTTACGACAAAGTCAGACTACAGCTTAGGGATAATGCAAAGGAGCTGGGTAAC

GGCTGTTTCGAATTCTATCACAAATGCGATAATGAATGTATGGAAAGTGTGAGAAATGGG

ACGTATGACTACCCTCAGTATTCAGAAGAAGCAAGATTAAAAAGAGAAGAAATAAGCGGA

GTGAAATTAGAATCAATAGGAACTTACCAGATACTGTCAATTTATTCAACAGCGGCGAGT

TCCCTAGCACTGGCAATCATGATGGCTGGTCTATCTTTATGGATGTGCTCCAATGGGTCG

TTACAGTGCAGAATTTGCATTTAA

>A_goose_Kazakhstan_7-20-B-Talg-12_2020_EPI1882551

ATGGAGAACATAGTACTTCTTCTTGCAATAGTTAGCCTTGTTAAAAGTGATCAGATTTGC

ATTGGTTACCATGCAAACAATTCGACAGAGCAAGTTGACACGATAATGGAAAAGAACGTC

ACTGTTACACATGCCCAAGACATACTGGAAAAAACACACAACGGGAAGCTCTGTGATCTA

AATGGGGTGAAGCCTCTGATTTTAAAGGATTGTAGTGTAGCTGGATGGCTCCTCGGAAAC

CCAATGTGCGACGAATTCATCAGAGTGCCGGAATGGTCCTACATAGTGGAGCGGGCTAAT

CCAGCTAATGACCTCTGTTACCCAGGGAGCCTCAATGACTATGAAGAACTGAAACACCTG

TTGAGCAGAATAAATCATTTTGAGAAGATTCTGATCATCCCCAAGAGTTCCTGGCCAAAT

CATGAAACATCACTAGGGGTGAGCGCAGCTTGTCCATACCAGGGAGCGCCCTCCTTTTTC

AGAAATGTGGTGTGGCTTATCAAAAAGAACGATGCATACCCAACAATAAAGATAAGCTAC

AATAATACCAATCGGGAAGATCTCTTGATACTGTGGGGGATTCATCATTCCAACAATGCA

GAAGAGCAGACAAATCTCTATAAAAACCCAACCACCTACATTTCAGTTGGAACATCAACT

TTAAACCAGAGGTTGGTACCAAAAATAGCTACTAGATCCCAAGTAAACGGGCAACGTGGA

AGAATGGACTTCTTCTGGACAATTTTAAAACCGGATGATGCAATCCATTTCGAGAGTAAT

GGAAATTTCATTGCTCCAGAATATGCATACAAAATTGTCAAGAAAGGGGACTCAACAATT

ATGAAAAGTGGAGTGGAATATGGCCACTGCAACACCAAATGTCAAACCCCAGTAGGAGCG

ATAAATTCTAGTATGCCATTCCACAACATACATCCTCTCACCATTGGGGAATGCCCCAAA

TACGTGAAGTCAAACAAGTTGGTCCTTGCGACTGGGCTCAGAAATAGTCCTCTAAGAGAA

AAGAGAAGAAAAAGAGGCCTGTTTGGGGCGATAGCAGGGTTTATAGAGGGAGGATGGCAG

GGAATGGTTGATGGTTGGTATGGGTACCATCATAGCAATGAGCAGGGGAGTGGGTACGCT

GCAGACAAAGAATCCACCCAAAAGGCAATAGATGGAGTTACCAATAAGGTCAACTCAATC

ATTGACAAAATGAACACTCAATTTGAGGCAGTTGGAAGGGAGTTTAATAACTTAGAAAGG

AGGATAGAGAATTTGAACAAGAAAATGGAAGACGGATTCCTAGATGTCTGGACCTATAAT

GCTGAACTTCTAGTTCTCATGGAAAACGAGAGGACTCTAGATTTCCATGATTCAAATGTC

AAGAACCTTTACGACAAAGTCAGACTACAGCTTAGGGATAATGCAAAGGAGCTGGGTAAC

GGCTGTTTCGAATTCTATCACAAATGCGATAATGAATGTATGGAAAGTGTGAGAAATGGG

ACGTATGACTACCCTCAGTATTCAGAAGAAGCAAGATTAAAAAGAGAAGAAATAAGCGGA

GTGAAATTAGAATCAATAGGAACTTACCAGATACTGTCAATTTATTCAACAGCGGCGAGT

TCCCTAGCACTGGCAATCATGATGGCTGGTCTATCTTTATGGATGTGCTCCAATGGGTCG

TTACAGTGCAGAATTTGCATTTAA

>A_crow_Kazakhstan_15-20-B-Talg-4_2020_EPI1882556

ATGGAGAACATAGTACTTCTTCTTGCAATAGTTAGCCTTGTTAAAAGTGATCAGATTTGC

ATTGGTTACCATGCAAACAATTCGACAGAGCAAGTTGACACGATAATGGAAAAGAACGTC

ACTGTTACACATGCCCAAGACATACTGGAAAAAACACACAACGGGAAGCTCTGTGATCTA

AATGGGGTGAAGCCTCTGATTTTAAAGGATTGTAGTGTAGCTGGATGGCTCCTCGGAAAC

CCAATGTGCGACGAATTCATCAGAGTGCCGGAATGGTCCTACATAGTGGAGCGGGCTAAT

CCAGCTAATGACCTCTGTTACCCAGGGAGCCTCAATGACTATGAAGAACTGAAACACCTG

TTGAGCAGAATAAATCATTTTGAGAAGATTCTGATCATCCCCAAGAGTTCCTGGCCAAAT

CATGAAACATCACTAGGGGTGAGCGCAGCTTGTCCATACCAGGGAGCGCCCTCCTTTTTC

AGAAATGTGGTGTGGCTTATCAAAAAGAACGATGCATACCCAACAATAAAGATAAGCTAC

AATAATACCAATCGGGAAGATCTCTTGATACTGTGGGGGATTCATCATTCCAACAATGCA

GAAGAGCAGACAAATCTCTATAAAAACCCAACCACCTACATTTCAGTTGGAACATCAACT

TTAAACCAGAGGTTGGTACCAAAAATAGCTACTAGATCCCAAGTAAACGGGCAACGTGGA

AGAATGGACTTCTTCTGGACAATTTTAAAACCGGATGATGCAATCCATTTCGAGAGTAAT

GGAAATTTCATTGCTCCAGAATATGCATACAAAATTGTCAAGAAAGGGGACTCAACAATT

ATGAAAAGTGGAGTGGAATATGGCCACTGCAACACCAAATGTCAAACCCCAGTAGGAGCG

ATAAATTCTAGTATGCCATTCCACAACATACATCCTCTCACCATTGGGGAATGCCCCAAA

TACGTGAAGTCAAACAAGTTGGTCCTTGCGACTGGGCTCAGAAATAGTCCTCTAAGAGAA

AAGAGAAGAAAAAGAGGCCTGTTTGGGGCGATAGCAGGGTTTATAGAGGGAGGATGGCAG

GGAATGGTTGATGGTTGGTATGGGTACCATCATAGCAATGAGCAGGGGAGTGGGTACGCT

GCAGACAAAGAATCCACCCAAAAGGCAATAGATGGAGTTACCAATAAGGTCAACTCAATC

ATTGACAAAATGAACACTCAATTTGAGGCAGTTGGAAGGGAGTTTAATAACTTAGAAAGG

AGGATAGAGAATTTGAACAAGAAAATGGAAGACGGATTCCTAGATGTCTGGACCTATAAT

GCTGAACTTCTAGTTCTCATGGAAAACGAGAGGACTCTAGATTTCCATGATTCAAATGTC

AAGAACCTTTACGACAAAGTCAGACTACAGCTTAGGGATAATGCAAAGGAGCTGGGTAAC

GGCTGTTTCGAATTCTATCACAAATGCGATAATGAATGTATGGAAAGTGTGAGAAATGGG

ACGTATGACTACCCTCAGTATTCAGAAGAAGCAAGATTAAAAAGAGAAGAAATAAGCGGA

GTGAAATTAGAATCAATAGGAACTTACCAGATACTGTCAATTTATTCAACAGCGGCGAGT

TCCCTAGCACTGGCAATCATGATGGCTGGTCTATCTTTATGGATGTGCTCCAATGGGTCG

TTACAGTGCAGAATTTGCATTTAA

>A_pigeon_Kazakhstan_15-20-B-Talg-5_2020_EPI1882560

ATGGAGAACATAGTACTTCTTCTTGCAATAGTTAGCCTTGTTAAAAGTGATCAGATTTGC

ATTGGTTACCATGCAAACAATTCGACAGAGCAAGTTGACACGATAATGGAAAAGAACGTC

ACTGTTACACATGCCCAAGACATACTGGAAAAAACACACAACGGGAAGCTCTGTGATCTA

AATGGGGTGAAGCCTCTGATTTTAAAGGATTGTAGTGTAGCTGGATGGCTCCTCGGAAAC

CCAATGTGCGACGAATTCATCAGAGTGCCGGAATGGTCCTACATAGTGGAGCGGGCTAAT

CCAGCTAATGACCTCTGTTACCCAGGGAGCCTCAATGACTATGAAGAACTGAAACACCTG

TTGAGCAGAATAAATCATTTTGAGAAGATTCTGATCATCCCCAAGAGTTCCTGGCCAAAT

CATGAAACATCACTAGGGGTGAGCGCAGCTTGTCCATACCAGGGAGCGCCCTCCTTTTTC

AGAAATGTGGTGTGGCTTATCAAAAAGAACGATGCATACCCAACAATAAAGATAAGCTAC

AATAATACCAATCGGGAAGATCTCTTGATACTGTGGGGGATTCATCATTCCAACAATGCA

GAAGAGCAGACAAATCTCTATAAAAACCCAACCACCTACATTTCAGTTGGAACATCAACT

TTAAACCAGAGGTTGGTACCAAAAATAGCTACTAGATCCCAAGTAAACGGGCAACGTGGA

AGAATGGACTTCTTCTGGACAATTTTAAAACCGGATGATGCAATCCATTTCGAGAGTAAT

GGAAATTTCATTGCTCCAGAATATGCATACAAAATTGTCAAGAAAGGGGACTCAACAATT

ATGAAAAGTGGAGTGGAATATGGCCACTGCAACACCAAATGTCAAACCCCAGTAGGAGCG

ATAAATTCTAGTATGCCATTCCACAACATACATCCTCTCACCATTGGGGAATGCCCCAAA

TACGTGAAGTCAAACAAGTTGGTCCTTGCGACTGGGCTCAGAAATAGTCCTCTAAGAGAA

AAGAGAAGAAAAAGAGGCCTGTTTGGGGCGATAGCAGGGTTTATAGAGGGAGGATGGCAG

GGAATGGTTGATGGTTGGTATGGGTACCATCATAGCAATGAGCAGGGGAGTGGGTACGCT

GCAGACAAAGAATCCACCCAAAAGGCAATAGATGGAGTTACCAATAAGGTCAACTCAATC

ATTGACAAAATGAACACTCAATTTGAGGCAGTTGGAAGGGAGTTTAATAACTTAGAAAGG

AGGATAGAGAATTTGAACAAGAAAATGGAAGACGGATTCCTAGATGTCTGGACCTATAAT

GCTGAACTTCTAGTTCTCATGGAAAACGAGAGGACTCTAGATTTCCATGATTCAAATGTC

AAGAACCTTTACGACAAAGTCAGACTACAGCTTAGGGATAATGCAAAGGAGCTGGGTAAC

GGCTGTTTCGAATTCTATCACAAATGCGATAATGAATGTATGGAAAGTGTGAGAAATGGG

ACGTATGACTACCCTCAGTATTCAGAAGAAGCAAGATTAAAAAGAGAAGAAATAAGCGGA

GTGAAATTAGAATCAATAGGAACTTACCAGATACTGTCAATTTATTCAACAGCGGCGAGT

TCCCTAGCACTGGCAATCATGATGGCTGGTCTATCTTTATGGATGTGCTCCAATGGGTCG

TTACAGTGCAGAATTTGCATTTAA

>A_chicken_Kazakhstan_1-20-B-Talg-67_2020_EPI1882563

ATGGAGAACATAGTACTTCTTCTTGCAATAGTTAGCCTTGTTAAAAGTGATCAGATTTGC

ATTGGTTACCATGCAAACAATTCGACAGAGCAAGTTGACACGATAATGGAAAAGAACGTC

ACTGTTACACATGCCCAAGACATACTGGAAAAAACACACAACGGGAAGCTCTGTGATCTA

AATGGGGTGAAGCCTCTGATTTTAAAGGATTGTAGTGTAGCTGGATGGCTCCTCGGAAAC

CCAATGTGCGACGAATTCATCAGAGTGCCGGAATGGTCCTACATAGTGGAGCGGGCTAAT

CCAGCTAATGACCTCTGTTACCCAGGGAGTCTCAATGACTATGAAGAACTGAAACACCTG

TTGAGCAGAATAAATCATTTTGAGAAGATTCTGATCATCCCCAAGAGTTCCTGGCCAAAT

CATGAAACATCACTAGGGGTGAGCGCAGCTTGTCCATACCAGGGAGCGCCCTCCTTTTTC

AGAAATGTGGTGTGGCTTATCAAAAAGAACGATGCATACCCAACAATAAAGATAAGCTAC

AATAATACCAATCGGGAAGATCTCTTGATACTGTGGGGGATTCATCATTCCAACAATGCA

GAAGAGCAGACAAATCTCTATAAAAACCCAACCACCTACATTTCAGTTGGAACATCAACT

TTAAACCAGAGGTTGGTACCAAAAATAGCTACTAGATCCCAAGTAAACGGGCAACGTGGA

AGAATGGACTTCTTCTGGACAATTTTAAAACCGGATGATGCAATCCATTTCGAGAGTAAT

GGAAATTTCATTGCTCCAGAATATGCATACAAAATTGTCAAGAAAGGGGACTCAACAATT

ATGAAAAGTGGAGTGGAATATGGCCACTGCAACACCAAATGTCAAACCCCAGTAGGAGCG

ATAAATTCTAGTATGCCATTCCACAACATACATCCTCTCACCATTGGGGAATGCCCCAAA

TACGTGAAGTCAAACAAGTTGGTCCTTGCGACTGGGCTCAGAAATAGTCCTCTAAGAGAA

AAGAGAAGAAAAAGAGGCCTGTTTGGGGCGATAGCAGGGTTTATAGAGGGAGGATGGCAG

GGAATGGTTGATGGTTGGTATGGGTACCATCATAGCAATGAGCAGGGGAGTGGGTACGCT

GCAGACAAAGAATCCACCCAAAAGGCAATAGATGGAGTTACCAATAAGGTCAACTCAATC

ATTGACAAAATGAACACTCAATTTGAGGCAGTTGGAAGGGAGTTTAATAACTTAGAAAGG

AGGATAGAGAATTTGAACAAGAAAATGGAAGACGGATTCCTAGATGTCTGGACCTATAAT

GCTGAACTTCTAGTTCTCATGGAAAACGAGAGGACTCTAGATTTCCATGATTCAAATGTC

AAGAACCTTTACGACAAAGTCAGACTACAGCTTAGGGATAATGCAAAGGAGCTGGGTAAC

GGCTGTTTCGAATTCTATCACAAATGCGATAATGAATGTATGGAAAGTGTGAGAAATGGG

ACGTATGACTACCCTCAGTATTCAGAAGAAGCAAGATTAAAAAGAGAAGAAATAAGCGGA

GTGAAATTAGAATCAATAGGAACTTACCAGATACTGTCAATTTATTCAACAGCGGCGAGT

TCCCTAGCACTGGCAATCATGATGGCTGGTCTATCTTTATGGATGTGCTCCAATGGGTCG

TTACAGTGCAGAATTTGCATTTAA

>A_goose_Omsk_30001_2020_EPI1814281

ATGGAGAACATAGTACTTCTTCTTGCAATAGTTAGCCTTGTTAAAAGTGATCAGATTTGC

ATTGGTTACCATGCAAACAATTCGACAGAGCAAGTTGACACGATAATGGAAAAGAACGTC

ACTGTTACACATGCCCAAGACATACTGGAAAAAACACACAACGGGAAGCTCTGTGATCTA

AATGGGGTGAAGCCTCTGATTTTAAAGGATTGTAGTGTAGCTGGATGGCTCCTCGGAAAC

CCAATGTGCGACGAATTCATCAGAGTGCCGGAATGGTCCTACATAGTGGAGAGGGCTAAT

CCAGCTAATGACCTCTGTTACCCAGGGAGCCTCAATGACTATGAAGAACTGAAACACCTG

TTGAGCAGAATAAATCATTTTGAGAAGATTCTGATCATCCCCAAGAGTTCTTGGCCAAAT

CATGAAACAGCACTAGGGGTGAGCGCAGCTTGTCCATACCAGGGAGCGCCCTCCTTTTTC

AGAAATGTGGTGTGGCTTATCAAAAAGAACGATGCATACCCAACAATAAAGATAAGCTAC

AATAATACCAATCGGGAAGATCTCTTGATACTGTGGGGGATTCATCATTCCAACAATGCA

GAAGAGCAGACAAATCTCTATAAAAACCCAACCACCTACATTTCAGTTGGAACATCAACT

TTAAACCAGAGGTTGGTACCAAAAATAGCTACTAGATCCCAAGTAAACGGGCAACGTGGA

AGAATGGACTTCTTCTGGACAATTTTAAAACCGGATGATGCAATCCATTTCGAGAGTAAT

GGGAATTTCATTGCTCCAGAATATGCATACAAAATTGTCAAGAAAGGGGACTCAACAATT

ATGAAAAGTGGAGTGGAATATGGCCACTGCAACACCAAATGTCAAACCCCAGTAGGAGCG

ATAAATTCTAGTATGCCATTCCACAACATACATCCTCTCACCATTGGGGAATGCCCCAAA

TACGTGAAGTCAAACAAGTTGGTCCTTGCGACTGGGCTCAGAAATAGTCCTCTAAGAGAA

AAGAGAAGAAAAAGAGGCCTGTTTGGGGCGATAGCAGGGTTTATAGAGGGAGGATGGCAG

GGAATGGTTGATGGTTGGTATGGGTACCACCATAGCAATGAGCAGGGGAGTGGGTACGCT

GCAGACAAAGAATCCACCCAAAAGGCAATAGATGGAGTTACCAATAAGGTCAACTCAATC

ATTGACAAAATGAACACTCAATTTGAGGCAGTTGGAAGGGAGTTTAATAACTTAGAAAGG

AGGATAGAGAATTTGAACAAGAAAATGGAAGACGGATTCCTAGATGTCTGGACCTATAAT

GCTGAACTTCTAGTTCTCATGGAAAACGAGAGGACTCTAGATTTCCATGATTCAAATGTC

AAGAACCTTTACGACAAAGTCAGACTACAGCTTAGGGATAATGCAAAGGAGCTGGGTAAC

GGCTGTTTCGAATTCTATCACAAATGCGATAATGAATGTATGGAAAGTGTGAGAAATGGG

ACGTATGACTACCCTCAGTATTCAGAAGAAGCAAGATTAAAAAGAGAAGAAATAAGCGGA

GTGAAATTAGAATCAATAGGAACTTACCAGATACTGTCAATTTATTCAACAGCGGCGAGT

TCCCTAGCACTGGCAATCATGACGGCTGGTCTATCTTTATGGATGTGCTCCAATGGGTCG

TTACAGTGCAGAATTTGCATTTAA

>A_duck_Omsk_0004_2020_EPI1813337

ATGGAGAACATAGTACTTCTTCTTGCAATAGTTAGCCTTGTTAAAAGTGATCAGATTTGC

ATTGGTTACCATGCAAACAATTCGACAGAGCAAGTTGACACGATAATGGAAAAGAACGTC

ACTGTTACACATGCCCAAGACATACTGGAAAAAACACACAACGGGAAGCTCTGTGATCTA

AATGGGGTGAAGCCTCTGATTTTAAAGGATTGTAGTGTAGCTGGATGGCTCCTCGGAAAC

CCAATGTGCGACGAATTCATCAGAGTGCCGGAATGGTCCTACATAGTGGAGAGGGCTAAT

CCAGCTAATGACCTCTGTTACCCAGGGAGCCTCAATGACTATGAAGAACTGAAACACCTG

TTGAGCAGAATAAATCATTTTGAGAAGATTCTGATCATCCCCAAGAGTTCCTGGCCAAAT

CATGAAACAGCACTAGGGGTGAGCGCAGCTTGTCCATACCAGGGAGCGCCCTCCTTTTTC

AGAAATGTGGTGTGGCTTATCAAAAAGAACGATGCATACCCAACAATAAAGATAAGCTAC

AATAATACCAATCGGGAAGATCTCTTGATACTGTGGGGGATTCATCATTCCAACAATGCA

GAAGAGCAGACAAATCTCTATAAAAACCCAACCACCTACATTTCAGTTGGAACATCAACT

TTAAACCAGAGGTTGGTACCAAAAATAGCTACTAGATCCCAAGTAAACGGGCAACGTGGA

AGAATGGACTTCTTCTGGACAATTTTAAAACCGGATGATGCAATCCATTTCGAGAGTAAT

GGAAATTTCATTGCTCCAGAATATGCATACAAAATTGTCAAGAAAGGGGACTCAACAATT

ATGAAAAGTGGAGTGGAATATGGCCACTGCAACACCAAATGTCAAACCCCAGTAGGAGCG

ATAAATTCTAGTATGCCATTCCACAACATACATCCTCTCACCATTGGGGAATGCCCCAAA

TACGTGAAGTCAAACAAGTTGGTCCTTGCGACTGGGCTCAGAAATAGTCCTCTAAGAGAA

AAGAGAAGAAAAAGAGGTCTGTTTGGGGCGATAGCAGGGTTTATAGAGGGAGGATGGCAG

GGAATGGTTGATGGTTGGTATGGGTACCACCATAGCAATGAGCAGGGGAGTGGGTACGCT

GCAGACAAAGAATCCACCCAAAAGGCAATAGATGGAGTTACCAATAAGGTCAACTCAATC

ATTGACAAAATGAACACTCAATTTGAGGCAGTTGGAAGGGAGTTTAATAACTTAGAAAGG

AGGATAGAGAATTTGAACAAGAAAATGGAAGACGGATTCCTAGATGTCTGGACCTATAAT

GCTGAACTTCTAGTTCTCATGGAAAACGAGAGGACTCTAGATTTCCATGATTCAAATGTC

AAGAACCTTTACGACAAAGTCAGACTACAGCTTAGGGATAATGCAAAGGAGCTGGGTAAC

GGCTGTTTCGAATTCTATCACAAATGCGATAATGAATGTATGGAAAGTGTGAGAAATGGG

ACGTATGACTACCCTCAGTATTCAGAAGAAGCAAGATTAAAAAGAGAAGAAATAAGCGGA

GTGAAATTAGAATCAATAGGAACTTACCAGATACTGTCAATTTATTCAACCGCGGCGAGT

TCCCTAGCACTGGCAATCATGATGGCTGGTCTATCTTTATGGATGTGCTCCAATGGGTCG

TTACAGTGCAGAATTTGCATTTAA

>A_duck_Chelyabinsk_1207-1_2020_EPI1812533

ATGGAGAACATAGTACTTCTTCTTGCAATAGTTAGCCTTGTTAAAAGTGATCAGATTTGC

ATTGGTTACCATGCAAACAATTCGACAGAGCAAGTTGACACGATAATGGAAAAGAACGTC

ACTGTTACACATGCCCAAGACATACTGGAAAAAACACACAACGGGAAGCTCTGTGATCTA

AATGGGGTGAAGCCTCTGATTTTAAAGGATTGTAGTGTAGCTGGATGGCTCCTCGGAAAC

CCAATGTGCGACGAATTCATCAGAGTGCCGGAATGGTCCTACATAGTGGAGAGGGCTAAT

CCAGCTAATGACCTCTGTTACCCAGGGAGCCTCAATGACTATGAAGAACTGAAACACCTG

TTGAGCAGAATAAATCATTTTGAGAAGATTCTGATCATCCCCAAGAGTTCCTGGCCAAAT

CATGAAACAGCACTAGGGGTGAGCGCAGCTTGTCCATACCAGGGAGCGCCCTCCTTTTTC

AGAAATGTGGTGTGGCTTATCAAAAAGAACGATGCATACCCAACAATAAAGATAAGCTAC

AATAATACCAATCGGGAAGATCTCTTGATACTGTGGGGGATTCATCATTCCAACAATGCA

GAAGAGCAGACAAATCTCTATAAAAACCCAACCACCTACATTTCAGTTGGAACATCAACT

TTAAACCAGAGGTTGGTACCAAAAATAGCTACTAGATCCCAAGTAAACGGGCAACGTGGA

AGAATGGACTTCTTCTGGACAATTTTAAAACCGGATGATGCAATCCATTTCGAGAGTAAT

GGAAATTTCATTGCTCCAGAATATGCATACAAAATTGTCAAGAAAGGGGACTCAACAATT

ATGAAAAGTGGAGTGGAATATGGCCACTGCAACACCAAATGTCAAACCCCAGTAGGAGCG

ATAAATTCTAGTATGCCATTCCACAACATACATCCTCTCACCATTGGGGAATGCCCCAAA

TACGTGAAGTCAAACAAGTTGGTCCTTGCGACTGGGCTCAGAAATAGTCCTCTAAGAGAA

AAGAGAAGAAAAAGAGGCCTGTTTGGGGCGATAGCAGGGTTTATAGAGGGAGGATGGCAG

GGAATGGTTGATGGTTGGTATGGGTACCACCATAGCAATGAGCAGGGGAGTGGGTACGCT

GCAGACAAAGAATCCACCCAAAAGGCAATAGATGGAGTTACCAATAAGGTCAACTCAATC

ATTGACAAAATGAACACTCAATTTGAGGCAGTTGGAAGGGAGTTTAATAACTTAGAAAGG

AGGATAGAGAATTTGAACAAGAAAATGGAAGACGGATTCCTAGATGTCTGGACCTATAAT

GCTGAACTTCTAGTTCTCATGGAAAACGAGAGGACTCTAGATTTCCATGATTCAAATGTC

AAGAACATTTACGACAAAGTCAGACTACAGCTTAGGGATAATGCAAAGGAGCTGGGTAAC

GGCTGTTTCGAATTCTATCACAAATGCGATAATGAATGTATGGAAAGTGTGAGAAATGGG

ACGTATGACTACCCTCAGTATTCAGAAGAAGCAAGATTAAAAAGAGAAGAAATAAGCGGA

GTGAAATTAGAATCAATAGGAACTTACCAGATACTGTCAATTTATTCAACAGCGGCGAGT

TCCCTAGCACTGGCAATCATGATGGCTGGTCTATCTTTATGGATGTGCTCCAATGGGTCG

TTACAGTGCAGAATTTGCATTTAA

>A_duck_Omsk_0075_2020_EPI1813281

ATGGAGAACATAGTACTTCTTCTTGCAATAGTTAGCCTTGTTAAAAGTGATCAGATTTGC

ATTGGTTACCATGCAAACAATTCGACAGAGCAAGTTGACACGATAATGGAAAAGAACGTC

ACTGTTACACATGCCCAAGACATACTGGAAAAAACACACAACGGGAAGCTCTGTGATCTA

AATGGGGTGAAGCCTCTGATTTTAAAGGATTGTAGTGTAGCTGGATGGCTCCTCGGAAAC

CCAATGTGCGACGAATTCATCAGAGTGCCGGAATGGTCCTACATAGTGGAGAGGGCTAAT

CCAGCTAATGACCTCTGTTACCCAGGGAGCCTCAATGACTATGAAGAACTGAAACACCTG

TTGAGCAGAATAAATCATTTTGAGAAGATTCTGATCATCCCCAAGAGTTCCTGGCCAAAT

CATGAAACAGCACTAGGGGTGAGCGCAGCTTGTCCATACCAGGGAGCGCCCTCCTTTTTC

AGAAATGTGGTGTGGCTTATCAAAAAGAACGATGCATACCCAACAATAAAGATAAGCTAC

AATAATACCAATCGGGAAGATCTCTTGATACTGTGGGGGATTCATCATTCCAACAATGCA

GAAGAGCAGACAAATCTCTATAAAAACCCAACCACCTACATTTCAGTTGGAACATCAACT

TTAAACCAGAGGTTGGTACCAAAAATAGCTACTAGATCCCAAGTAAACGGGCAACGTGGA

AGAATGGACTTCTTCTGGACAATTTTAAAACCGGATGATGCAATCCATTTCGAGAGTAAT

GGAAATTTCATTGCTCCAGAATATGCATACAAAATTGTCAAGAAAGGGGACTCAACAATT

ATGAAAAGTGGAGTGGAATATGGCCACTGCAACACCAAATGTCAAACCCCAGTAGGAGCG

ATAAATTCTAGTATGCCATTCCACAACATACATCCTCTCACCATTGGGGAATGCCCCAAA

TACGTGAAGTCAAACAAGTTGGTCCTTGCGACTGGGCTCAGAAATAGTCCTCTAAGAGAA

AAGAGAAGAAAAAGAGGCCTGTTTGGGGCGATAGCAGGGTTTATAGAGGGAGGATGGCAG

GGAATGGTTGATGGTTGGTATGGGTACCACCATAGCAATGAGCAGGGGAGTGGGTACGCT

GCAGACAAAGAATCCACCCAAAAGGCAATAGATGGAGTTACCAATAAGGTCAACTCAATC

ATTGACAAAATGAACACTCAATTTGAGGCAGTTGGAAGGGAGTTTAATAACTTAGAAAGG

AGGATAGAGAATTTGAACAAGAAAATGGAGGACGGATTCCTAGATGTCTGGACCTATAAT

GCTGAACTTCTAGTTCTCATGGAAAACGAGAGGACTCTAGATTTCCATGATTCAAATGTC

AAGAACCTTTACGACAAAGTCAGACTACAGCTTAGGGATAATGCAAAGGAGCTGGGTAAC

GGCTGTTTCGAATTCTATCACAAATGCGATAATGAATGTATGGAAAGTGTGAGAAATGGG

ACGTATGACTACCCTCAGTATTCAGAAGAAGCAAGATTAAAAAGAGAAGAAATAAGCGGA

GTGAAATTAGAATCAATAGGAACTTACCAGATACTGTCAATTTATTCAACAGCGGCGAGT

TCCCTAGCACTGGCAATCATGATGGCTGGTCTATCTTTATGGATGTGCTCCAATGGGTCG

TTACAGTGCAGAATTTGCATTTAA

>A_chicken_Chelyabinsk_201_2020_EPI1814337

ATGGAGAACATAGTACTTCTTCTTGCAATAGTTAGCCTTGTTAAAAGTGATCAGATTTGC

ATTGGTTACCATGCAAACAATTCGACAGAGCAAGTTGACACGATAATGGAAAAGAACGTC

ACTGTTACACATGCCCAAGACATACTGGAAAAAACACACAACGGGAAGCTCTGTGATCTA

AATGGGGTGAAGCCTCTGATTTTAAAGGATTGTAGTGTAGCTGGATGGCTCCTCGGAAAC

CCAATGTGCGACGAATTCATCAGAGTGCCGGAATGGTCCTACATAGTGGAGAGGGCTAAT

CCAGCTAATGACCTCTGTTACCCAGGGAGCCTCAATGACTATGAAGAACTGAAACACCTG

TTGAGCAGAATAAATCATTTTGAGAAGATTCTGATCATCCCCAAGAGTTCCTGGCCAAAT

CATGAAACAGCACTAGGGGTGAGCGCAGCTTGTCCATACCAGGGAGCGCCCTCCTTTTTC

AGAAATGTGGTGTGGCTTATCAAAAAGAACGATGCATACCCAACAATAAAGATAAGCTAC

AATAATACCAATCGGGAAGATCTCTTGATACTGTGGGGGATTCATCATTCCAACAATGCA

GAAGAGCAGACAAATCTCTATAAAAACCCAACCACCTACATTTCAGTTGGAACATCAACT

TTAAACCAGAGGTTGGTACCAAAAATAGCTACTAGATCCCAAGTAAACGGGCAACGTGGA

AGAATGGACTTCTTCTGGACAATTTTAAAACCGGATGATGCAATCCATTTCGAGAGTAAT

GGAAATTTCATTGCTCCAGAATATGCATACAAAATTGTCAAGAAAGGGGACTCAACAATT

ATGAAAAGTGGTGTGGAATATGGCCACTGCAACACCAAATGTCAAACCCCAGTAGGAGCG

ATAAATTCTAGTATGCCATTCCACAACATACATCCTCTCACCATTGGGGAATGCCCCAAA

TACGTGAAGTCAAACAAGTTGGTCCTTGCGACTGGGCTCAGAAATAGTCCTCTAAGAGAA

AAGAGAAGAAAAAGAGGCCTGTTTGGGGCGATAGCAGGGTTTATAGAGGGAGGATGGCAG

GGAATGGTTGATGGTTGGTATGGGTACCACCATAGCAATGAGCAGGGGAGTGGGTACGCT

GCAGACAAAGAATCCACCCAAAAGGCAATAGATGGAGTTACCAATAAGGTCAACTCAATC

ATTGACAAAATGAACACTCAATTTGAGGCAGTTGGAAGGGAGTTTAATAACTTAGAAAGG

AGGATAGAGAATTTGAACAAGAAAATGGAAGACGGATTCCTAGATGTCTGGACCTATAAT

GCTGAACTTCTAGTTCTCATGGAAAACGAGAGGACTCTAGATTTCCATGATTCAAATGTC

AAGAACCTTTACGACAAAGTCAGACTACAGCTTAGGGATAATGCAAAGGAGCTGGGTAAC

GGCTGTTTCGAATTCTATCACAAATGCGATAATGAATGTATGGAAAGTGTGAGAAATGGG

ACGTATGACTACCCTCAGTATTCAGAAGAAGCAAGATTAAAAAGAGAAGAAATAAGCGGA

GTGAAATTAGAATCAATAGGAACTTACCAGATACTGTCAATTTATTCAACAGCGGCGAGT

TCCCTAGCACTGGCAATCATGATGGCTGGTCTATCTTTATGGATGTGCTCCAATGGGTCG

TTACAGTGCAGAATTTGCATTTAA

>A_chicken_Kurgan_1005_2020_EPI1814369

ATGGAGAACATAGTACTTCTTCTTGCAATAGTTAGCCTTGTTAAAAGTGATCAGATTTGC

ATTGGTTACCATGCAAACAATTCGACAGAGCAAGTTGACACGATAATGGAAAAGAACGTC

ACTGTTACACATGCCCAAGACATACTGGAAAAAACACACAACGGGAAGCTCTGTGATCTA

AATGGGGTGAAGCCTCTGATTTTAAAGGATTGTAGTGTAGCTGGATGGCTCCTCGGAAAC

CCAATGTGCGACGAATTCATCAGAGTGCCGGAATGGTCCTACATAGTGGAGAGGGCTAAT

CCAGCTAATGACCTCTGTTACCCAGGGAGCCTCAATGACTATGAAGAACTGAAACACCTG

TTGAGCAGAATAAATCATTTTGAGAAGATTCTGATCATCCCCAAGAGTTCCTGGCCAAAT

CATGAAACAGCACTAGGGGTGAGCGCAGCTTGTCCATACCAGGGAGCGCCCTCCTTTTTC

AGAAATGTGGTGTGGCTTATCAAAAAGAACGATGCATACCCAACAATAAAGATAAGCTAC

AATAATACCAATCGGGAAGATCTCTTGATACTGTGGGGGATTCATCATTCCAACAATGCA

GAAGAGCAGACAAATCTCTATAAAAACCCAACCACCTACATTTCAGTTGGAACATCAACT

TTAAACCAGAGGTTGGTACCAAAAATAGCTACTAGATCCCAAGTAAACGGGCAACGTGGA

AGAATGGACTTCTTCTGGACAATTTTAAAACCGGATGATGCAATCCATTTCGAGAGTAAT

GGAAATTTCATTGCTCCAGAATATGCATACAAAATTGTCAAGAAAGGGGACTCAACAATT

ATGAAAAGTGGAGTGGAATATGGCCACTGCAACACCAAATGTCAAACCCCAGTAGGAGCG

ATAAATTCTAGTATGCCATTTCACAACATACATCCTCTCACCATTGGGGAATGCCCCAAA

TACGTGAAGTCAAACAAGTTGGTCCTTGCGACTGGGCTCAGAAATAGTCCTCTAAGAGAA

AAGAGAAGAAAAAGAGGCCTGTTTGGGGCGATAGCAGGGTTTATAGAGGGAGGATGGCAG

GGAATGGTTGATGGTTGGTATGGGTACCACCATAGCAATGAGCAGGGGAGTGGGTACGCT

GCAGACAAAGAATCCACCCAAAAGGCAATAGATGGAGTTACCAATAAGGTCAACTCAATC

ATTGACAAAATGAACACTCAATTTGAGGCAGTTGGAAGGGAGTTTAATAACTTAGAAAGG

AGGATAGAGAATTTGAACAAGAAAATGGAAGACGGATTCCTAGATGTCTGGACCTATAAT

GCTGAACTTCTAGTTCTCATGGAAAACGAGAGGACTCTAGATTTCCATGATTCAAATGTC

AAGAACCTTTACGACAAAGTCAGACTACAGCTTAGGGATAATGCAAAGGAGCTGGGTAAC

GGCTGTTTCGAATTCTATCACAAATGCGATAATGAATGTATGGAAAGTGTGAGAAATGGG

ACGTATGACTACCCTCAGTATTCAGAAGAAGCAAGATTAAAAAGAGAAGAAATAAGCGGA

GTGAAATTAGAATCAATAGGAACTTACCAGATACTGTCAATTTATTCAACAGCGGCGAGT

TCCCTAGCACTGGCAATCATGATGGCTGGTCTATCTTTATGGATGTGCTCCAATGGGTCG

TTACAGTGCAGAATTTGCATTTAA

>A_chicken_Tyumen_302-02_2020_EPI1848614

ATGGAGAACATAGTACTTCTTCTTGCAATAGTTAGCCTTGTTAAAAGTGATCAGATTTGC

ATTGGTTACCATGCAAACAATTCGACAGAGCAAGTTGACACGATAATGGAAAAGAACGTC

ACTGTTACACATGCCCAAGACATACTGGAAAAAACACACAACGGGAAGCTCTGTGATCTA

AATGGGGTGAAGCCTCTGATTTTAAAGGATTGTAGTGTAGCTGGATGGCTCCTCGGAAAC

CCAATGTGCGACGAATTCATCAGAGTGCCGGAATGGTCCTACATAGTGGAGAGGGCTAAT

CCAGCTAATGACCTCTGTTACCCAGGGAGCCTCAATGACTATGAAGAACTGAAACACCTG

TTGAGCAGAATAAATCATTTTGAGAAGATTCTGATCATCCCCAAGAGTTCCTGGCCAAAT

CATGAAACAGCACTAGGGGTGAGCGCAGCTTGTCCATACCAGGGAGCGCCCTCCTTTTTC

AGAAATGTGGTGTGGCTTATCAAAAAGAACGATGCATACCCAACAATAAAGATAAGCTAC

AATAATACCAATCGGGAAGATCTCTTGATACTGTGGGGGATTCATCATTCCAACAATGCA

GAAGAGCAGACAAATCTCTATAAAAACCCAGCCACCTACATTTCAGTTGGAACATCAACT

TTAAACCAGAGGTTGGTACCAAAAATAGCTACTAGATCCCAAGTAAACGGGCAACGTGGA

AGAATGGACTTCTTCTGGACAATTTTAAAACCGGATGATGCAATCCATTTCGAGAGTAAT

GGAAATTTCATTGCTCCAGAATATGCATACAAAATTGTCAAGAAAGGGGACTCAACAATT

ATGAAAAGTGGAGTGGAATATGGCCACTGCAACACCAAATGTCAAACCCCAGTAGGAGCG

ATAAATTCTAGTATGCCATTCCACAACATACATCCTCTCACCATTGGGGAATGCCCCAAA

TACGTGAAGTCAAACAAGTTGGTCCTTGCGACTGGGCTCAGAAATAGTCCTCTAAGAGAA

AAGAGAAGAAAAAGAGGCCTGTTTGGGGCGATAGCAGGGTTTATAGAGGGAGGATGGCAG

GGAATGGTTGATGGTTGGTATGGGTACCACCATAGCAATGAGCAGGGGAGTGGGTACGCT

GCAGACAAAGAATCCACCCAAAAGGCAATAGATGGAGTTACCAATAAGGTCAACTCAATC

ATTGACAAAATGAACACTCAATTTGAGGCAGTTGGAAGGGAGTTTAATAACTTAGAAAGG

AGGATAGAGAATTTGAACAAGAAAATGGAAGACGGATTCCTAGATGTCTGGACCTATAAT

GCTGAACTTCTAGTTCTCATGGAAAACGAGAGGACTCTAGATTTCCATGATTCAAATGTC

AAGAACCTTTACGACAAAGTCAGACTACAGCTTAGGGATAATGCAAAGGAGCTGGGTAAC

GGCTGTTTCGAATTCTATCACAAATGCGATAATGAATGTATGGAAAGTGTGAGAAATGGG

ACGTATGACTACCCTCAGTATTCAGAAGAAGCAAGATTAAAAAGAGAAGAAATAAGCGGA

GTGAAATTAGAATCAATAGGAACTTACCAGATACTGTCAATTTATTCAACAGCGGCGAGT

TCCCTAGCACTGGCAATCATGATGGCTGGTCTATCTTTATGGATGTGCTCCAATGGGTCG

TTACAGTGCAGAATTTGCATTTAA

>A_muscovy_duck_Slovakia_Pah1_21VIR1086-1_2021_EPI1858244

ATGGAGAACATAGTACTTCTTCTTGCAATAGTTAGCCTTGTTAAAAGTGATCAGATTTGC

ATTGGTTACCATGCAAACAATTCGACAGAGCAAGTTGACACGATAATGGAAAAGAACGTC

ACTGTTACACATGCCCAAGACATACTGGAAAAAACACACAACGGGAAGCTCTGTGATCTA

AATGGGGTGAAGCCTCTGATTTTAAAGGATTGTAGTGTAGCTGGATGGCTCCTCGGAAAC

CCAATGTGCGACGAATTCATCAGAGTGCCGGAATGGTCCTACATAGTGGAAAGGGCTAAT

CCAGCTAATGACCTCTGTTACCCAGGGAGCCTCAATGACTATGAAGAACTGAAACACCTG

TTGAGCAGAATAAATCATTTTGAGAAGATTCTGATCATCCCCAAGAGTTCCTGGCCAAAT

CATGAAACAGCACTAGGGGTGAGCGCAGCTTGTCCATACCAGGGAGCGCCCTCCTTTTTC

AGAAATGTGGTGTGGCTTATCAAAAAGAACGATGCATACCCAACAATAAAGATAAGCTAC

AATAATACCAATCGGGAAGATCTCTTGATACTGTGGGGGATTCATCATTCCAACAATGCA

GAAGAGCAGACAAATCTCTATAAAAACCCAACCACCTACATTTCAGTTGGGACATCAACT

TTAAACCAGAGGTTGGTACCAAAAATAGCTACTAGATCCCAAGTAAACGGGCAACGTGGA

AGAATGGACTTCTTCTGGACAATTTTAAAACCGGATGATGCAATCCATTTCGAGAGTAAT

GGAAATTTCATTGCTCCAGAATATGCATACAAAATTGTCAAGAAAGGGGACTCAACAATT

ATGAAAAGTGGAGTGGAATATGGCCACTGCAACACCAAATGTCAAACCCCAGTAGGAGCG

ATAAATTCTAGTATGCCATTCCACAACATACATCCTCTCACCATTGGGGAATGCCCCAAA

TACGTGAAGTCAAACAAGTTGGTCCTTGCGACTGGGCTCAGAAATAGTCCTCTAAGAGAA

AAGAGAAGAAAAAGAGGCCTGTTTGGGGCGATAGCAGGGTTTATAGAGGGAGGATGGCAG

GGAATGGTTGATGGTTGGTATGGGTACCACCATAGCAATGAGCAGGGGAGTGGGTACGCT

GCAGACAAAGAATCCACCCAAAAGGCAATAGATGGAGTTACCAATAAGGTCAACTCAATC

ATTGACAAAATGAACACTCAATTTGAGGCAGTTGGAAGGGAGTTTAATAACTTAGAAAGG

AGGATAGAGAATTTGAACAAGAAAATGGAAGACGGATTCCTAGATGTCTGGACCTATAAT

GCTGAACTTCTAGTTCTCATGGAAAACGAGAGGACTCTAGATTTCCATGATTCAAATGTC

AAGAACCTTTACGACAAAGTCAGACTACAGCTTAGGGATAATGCAAAGGAGCTGGGTAAC

GGCTGTTTCGAATTCTATCACAAATGCGATAATGAATGTATGGAAAGTGTGAGAAATGGG

ACGTATGACTACCCTCAGTATTCAGAAGAAGCAAGATTAAAAAGAGAAGAAATAAGCGGA

GTGAAATTAGAATCAATAGGAACTTACCAGATACTGTCAATTTATTCAACAGCGGCGAGT

TCCCTAGCACTGGCAATCATGATGGCTGGTCTATCTTTATGGATGTGCTCCAATGGGTCG

TTACAGTGCAGAATTTGCATTTAA

>A_duck_Sichuan_NCXJ16_2014_EPI590893_2344a

ATGGAGAAAATAGTGCTTCTTCTTGCAATAGTTAGCCTTGTTAAAGGTGATCAGATTTGC

ATTGGTTACCATGCAAACAACTCGACGGAGCAGGTTGACACGATAATGGAAAAGAACGTC

ACTGTTACACATGCCCAAGACATACTGGAAAAGACACACAATGGAAAGCTCTGCGATTTA

AATGGAGTGAAGCCTCTGATTTTAAAGGATTGTAGTGTAGCTGGATGGCTTCTCGGAAAC

CCAATGTGCGACGAATTCATCAGGGTGCCGGAATGGTCTTACATAGTAGAAAGGGCTAAC

CCAGCCAATGACCTCTGTTACCCAGGGAACCTCAATGATTATGAAGAACTGAAACACCTA

TTGAGCAGAATAAATCATTTTGAGAAAATTCTGATCATCCCCAAGAGTTCTTGGACCAAT

CATGAAACATCATTAGGGGTGAGCGCAGCATGCCCATACCAGGGAACGCCCTCCTTTTTC

AGAAATGTGGTATGGCTTATCAAAAAGAACGATGCATACCCCACAATAAAAATAAGCTAC

AATAATACCAATCAGGAAGATCTGTTGATACTGTGGGGGGTTCACCATTCCAACAATGCA

GCAGAGCAGACGAACCTCTATAAAAACCCAACCACCTATATTTCCGTTGGGACATCAACA

TTAAACCAGAGATTGGTACCAAAAATAGCTACTAGATCCCAAGTGAACGGACAGCGTGGA

AGAATGGATTTCTTCTGGACAATTCTAAAACCGAATGATGCAATCCACTTCGAGAGTAAT

GGAAATTTCATTGCTCCAGAATATGCATACAAAATTGTCAAGAAAGGGGACTCAACAATT

ATGAAAAGTGAAGTGGAATATGGCCACTGCAACACCAAATGTCAAACTCCAATAGGGGCG

ATAAACTCTAGTATGCCATTTCACAATATACACCCTCTCACCATCGGGGAATGCCCCAAA

TACGTGAAATCAAACAAATTAGTCCTTGCGACTGGGCTCAGAAATAGTCCTCTAAGAGAA

AAGAGAAGAAAAAGAGGACTATTTGGAGCTATAGCAGGGTTTATAGAGGGAGGATGGCAG

GGAATGGTAGATGGCTGGTATGGGTACCACCATAGCAATGAGCAGGGGAGTGGGTACGCT

GCAGACAAAGAATCCACCCAAAAGGCTATAGATGGAGTTACCAATAAGGTCAACTCGATT

ATTGACAAAATGAACACTCAATTTGAGGCCGTTGGAAGGGAATTTAATAACTTAGAAAGG

AGAATAGAGAATTTAAACAAGAAAATGGAAGATGGATTCCTAGATGTCTGGACTTATAAT

GCTGAACTTCTAGTTCTCATGGAAAATGAGAGAACTCTAGATTTCCATGACTCAAATGTC

AAGAACCTTTACGATAAAGTCCGACTACAGCTTAGGGACAATGCAAAGGAACTGGGTAAT

GGTTGTTTTGAGTTCTATCACAAATGTGATAATGAATGTATGGAAAGTGTAAGAAATGGG

ACATATGACTACCCGCAATATTCAGAAGAAGCAAGATTAAAAAGAGAAGAAATAAGCGGA

GTGAAATTAGAGTCAATAGGAACTTACCAAATACTGTCAATTTATTCAACAGTGGCGAGT

TCCCTAGCACTGGCAATCATAGTGGCTGGTTTATCTTTATGGATGTGCTCCAATGGGTCG

TTGCAGTGCAGAATTTGCATTTAA

>A_Fujian-Sanyuan_21099_2017_x_PR8_CNIC-21099_EPI1369973_2344b

ATGGAGGACATAGTGCTTCTTCTTGCAATAGTTAGCCTTGTTAAAAGTGATCAGATTTGC

ATTGGTTACCATGCAAACAACTCGACAGAGCAAGTTGACACGATAATGGAAAAGAACGTC

ACTGTTACACATGCCCAAGACATACTGGAAAAAACACACAATGGGAAGCTCTGCGATCTA

AATGGGGTGAAACCCCTGATTTTAAAGGATTGTAGTGTAGCTGGATGGCTCCTCGGAAAC

CCAATGTGCGACGAATTCATCAGAGTGCCGGAATGGTCTTACATAGTGGAGAGGGCTAAC

CCAGCTAATGACCTCTGTTACCCAGGGAGCCTCAATGACTATGAAGAACTGAAACACCTG

TTGAGCAGAATAAATCATTTTGAGAAGATTCTGATCATCCCCAAGAGTTCTTGGCCCAAT

CATGAAACATCATTAGGGGTGAGTGCAGCTTGTCCATACCAGGGGACGCCCTCCTTTTTC

AGAAATGTGGTATGGCTTATCAAAAAGAACGATGCATACCCAACAATAAAGATAAGCTAC

AATAATACCAATCGGGAAGATCTCTTGATACTGTGGGGGATTCATCATTCCAACAATGCA

GAAGAGCAGACAAATCTCTATAAAAACCCAACCACCTATATTTCAGTTGGAACATCAACA

TTAAACCAGAGATTGGTACCAAAAATAGCTACTAGATCCCAAGTAAACGGGCAACGTGGA

AGAATGGACTTCTTCTGGACAATTTTAAAACCGAATGATGCAATCCATTTCGAGAGTAAT

GGAAATTTCATTGCTCCAGAATATGCATACAAAATTGTCAAGAAAGGGGACTCAACAATT

ATGAAAAGTGAAGTGGAATATGGCCACTGCAACACCAAATGTCAAACCCCAGTAGGAGCG

ATAAACTCTAGTATGCCATTCCACAATATACATCCTCTCACCATCGGGGAATGCCCCAAA

TACGTGAAGTCAAACAAGTTGGTCCTTGCGACTGGGCTCAGAAATAGTCCTCTAAGAGAA

ACGAGA---------GGGCTGTTTGGGGCTATAGCAGGTTTTATAGAGGGAGGATGGCAG

GGAATGGTTGATGGTTGGTATGGGTACCACCATAGCAATGAGCAGGGGAGTGGGTACGCT

GCAGACAAAGAATCCACCCAAAAGGCAATAGATGGAGTTACCAATAAGGTCAACTCGATC

ATTGACAAAATGAACACTCAATTTGAGGCAGTTGGAAGGGAGTTTAATAACTTAGAAAGG

AGGATAGAGAATTTGAACAAGAAAATGGAAGACGGATTCCTAGATGTCTGGACCTATAAT

GCTGAACTTCTAGTTCTCATGGAAAACGAGAGGACTCTAGATTTCCATGACTCAAATGTC

AAGAACCTTTACGACAAAGTCAGACTGCAGCTTAGGGATAATGCAAAGGAGCTGGGTAAC

GGTTGTTTCGAGTTCTATCACAAATGTGATAATGAATGTATGGAAAGTGTGAAAAATGGG

ACGTATGACTACCCTCAGTATTCAGAAGAAGCAAGATTAAAAAGAGAAGAAATAAGCGGA

GTGAAATTAGAATCAATAGGAACTTACCAAATACTGTCAATTTATTCAACAGTGGCGAGT

TCCCTAGCACTGGCAATCATGGTGGCTGGTCTATCTTTATGGATGTGCTCCAATGGGTCG

TTACAGTGCAGAATTTGCATTTAA

>A_Perigrine_falcon_Netherlands_18003274-001_2018_EPI1327128_2344b

ATGGAGAACATAGTGCTTCTTCTTGCAATAGTTAGCCTTGTTAAAAGTGATCAGATTTGC

ATTGGTTACCATGCAAACAACTCGACAGAGCAAGTTGACACGATAATGGAAAAGAACGTC

ACTGTTACACATGCCCAAGACATACTGGAAAAAACACACAACGGGAAGCTCTGCGATCTA

AATGGAGTGAAGCCTCTGATTTTAAAGGATTGTAGTGTAGCTGGATGGCTCCTCGGAAAC

CCAATGTGCGACGAATTCATCAGAGTGCCGGAATGGTCTTACATAGTGGAGAGGGATAAT

CCAGCTAATGACCTCTGTTACCCAGGGAGCCTCAATGACTATGAAGAACTGAAACACCTG

TTGAGCAGAATAAATCATTTTGAGAAGATTCTGATCATCCCCAAGAGTTCTTGGCCCAAT

CATGAAACATCATTAGGGGTGAGCGCAGCTTGTCCATACCAGGGAACGCCCTCCTTTTTC

AGAAATGTGGTATGGCTTATCAAAAAGAACGATGCATACCCCACAATAAAGATAAGCTAC

AATAATACCAATCGGGAAGATCTCTTGATACTGTGGGGGATTCATCATTCCAACAATGCA

GAAGAGCAGACAAATCTCTATAAAAACCCAACCACCTATATTTCAGTTGGAACATCAACA

TTAAACCAGAGATTGGTACCAAAAATAGCTACTAGATCCCAAGTAAACGGGCAACGTGGA

AGAATGGACTTTTTCTGGACAATTTTAAAACCGAATGATGCAATCCATTTCGAGAGTAAT

GGAAATTTCATTGCTCCAGAATATGCATACAAAATTGTCAAGAAAGGGGACTCAACAATT

ATGAAAAGTGGAGTGGAATATGGCCACTGCAACACCAAATGTCAAACCCCAGTAGGAGCG

ATAAACTCTAGTATGCCGTTCCACAATATACATCCTCTCACCATTGGGGAATGCCCCAAA

TACGTGAAGTCAAACAAGTTGGTCCTTGCGACTGGGCTTAGAAATAGTCCTCTAAGAGAA

AAGAGAAGAAAAAGAGGGCTGTTTGGGGCGATAGCAGGTTTTATAGAGGGAGGATGGCAG

GGAATGGTTGATGGTTGGTATGGCTACCACCATAGCAATGAGCAGGGGAGTGGGTACGCT

GCAGACAAAGAGTCCACCCAAAAGGCAATAGATGGAGTTACCAATAAGGTCAACTCGATC

ATTGACAAAATGAACACTCAATTTGAGGCAGTTGGAAGGGAGTTTAATAACTTAGAAAGG

AGGATAGAGAATTTGAACAAGAAAATGGAAGACGGATTCCTAGATGTCTGGACCTATAAT

GCTGAACTTCTAGTTCTCATGGAAAACGAGAGGACTCTAGATTTCCATGACTCAAATGTC

AAGAACCTTTACGACAAAGTCAGACTGCAGCTTAGGGATAATGCAAAGGAGCTGGGTAAC

GGTTGTTTCGAATTCTATCACAAATGTGATAATGAATGTATGGAAAGTGTGAGAAATGGG

ACGTATGACTACCCTCAGTACTCAGAAGAAGCAAGATTAAAAAGAGAAGAAATAAGCGGA

GTTAAATTAGAATCAATAGGAACATACCAAATACTGTCAATTTATTCAACAGTGGCGAGT

TCCCTAGCATTGGCAATCATGGTGGCTGGTCTATCTTTATGGATGTGCTCCAATGGGTCG

TTACAGTGCAGAATTTGCATTTAA

>A_chicken_Washington_3490-18_2015_EPI590692_2344c

ATGGAGAAAATAGTGCTTCTTTTTGCAGTGATTAGCCTTGTTAAAAGTGATCAGATTTGC

ATTGGTTACCATGCAAACAACTCAACAAAGCAGGTTGACACGATAATGGAGAAAAACGTC

ACTGTTACACATGCCCAAGACATACTGGAAAAGACACACAACGGGAAGCTCTGCGATCTT

AATGGAGTGAAGCCCCTGATTCTAAAGGATTGTAGCGTAGCTGGGTGGCTCCTTGGAAAT

CCAATGTGCGACGAGTTCATCAGGGTACCGGAATGGTCTTACATCGTGGAGAGGGCTAAC

CCAGCCAACGACCTCTGTTACCCAGGGACCCTCAATGACTATGAGGAACTGAAACACCTA

TTGAGCAGAATAAATCATTTTGAGAAAACTCTGATCATCCCCAGGAGTTCTTGGCCCAAT

CATGAAACATCATTGGGGGTGAGCGCAGCATGTCCATACCAGGGAGCATCCTCATTTTTC

AGAAATGTGGTATGGCTCATCAAAAAGAACGATGCATACCCGACAATAAAGATAAGCTAC

AATAATACCAATCGGGAAGATCTTTTGATACTGTGGGGGATTCATCATTCCAACAATGCA

GCAGAGCAGACAAATCTCTATAAAAACCCAGACACTTATGTTTCCGTTGGGACATCAACA

TTAAACCAGAGATTGGTGCCAAAAATAGCTACTAGATCCCAAGTAAACGGGCAGAGTGGA

AGAATGGATTTCTTCTGGACAATTTTAAAACCGAATGATGCAATCCACTTTGAGAGTAAT

GGAAATTTCATTGCTCCAGAATATGCATACAAAATTGTCAAGAAAGGGGACTCAACAATT

ATGAAAAGTGAAATGGAGTATGGCCACTGCAACACCAAATGTCAAACTCCAATAGGGGCG

ATAAACTCTAGCATGCCATTCCACAATATACACCCTCTCACCATCGGGGAATGCCCCAAA

TACGTGAAGTCAAACAAATTAGTCCTTGCGACTGGGCTCAGAAATAGTCCTCTAAGAGAA

AGAAGAAGAAAAAGAGGACTATTTGGAGCTATAGCAGGGTTTATAGAGGGAGGATGGCAG

GGAATGGTAGACGGTTGGTATGGGTATCATCATAGCAATGAGCAGGGGAGTGGGTACGCT

GCAGACAAAGAATCAACCCAAAAGGCAATAGATGGAGTTACCAATAAGGTCAACTCAATC

ATTGACAAAATGAACACTCAATTTGAGGCCGTTGGAAGGGAATTTAATAACTTAGAAAGG

AGAATAGAGAATTTAAACAAGAAAATGGAAGACGGATTCCTAGATGTCTGGACTTATAAT

GCTGAACTTTTAGTTCTCATGGAAAATGAGAGAACTCTAGATTTCCATGACTCAAATGTC

AAGAACCTTTACGACAAAGTCCGACTACAGCTTAGGGATAATGCAAAGGAGCTGGGTAAT

GGTTGTTTCGAGTTCTATCACAAATGTGATAACGAATGTATGGAGAGCGTAAGAAATGGG

ACGTATGACTACCCTAAGTATTCAGAAGAAGCAATATTAAAGAGAGAAGAAATAAGCGGA

GTGAAATTAGAATCAATAGGAACTTACCAGATACTGTCAATTTATTCAACAGTGGCGAGT

TCCCTAGCACTGGCAATCATAGTGGCTGGTCTATCTTTATGGATGTGCTCTAATGGGTCG

TTACAATGCAGAATTTGCATCTAA

>A_gyrfalcon_Washington_41088-6_2014_EPI569390_2344c

ATGGAGAAAATAGTGCTTCTTCTTGCAGTGATTAGCCTTGTTAAAAGTGATCAGATTTGC

ATTGGTTACCATGCAAACAACTCAACAAAGCAGGTTGACACGATAATGGAGAAAAACGTC

ACTGTTACACATGCCCAAGACATACTGGAAAAGACACACAACGGGAAGCTCTGCGATCTT

AATGGAGTGAAGCCCCTGATTCTAAAGGATTGTAGCGTAGCTGGGTGGCTCCTTGGAAAT

CCAATGTGCGACGAGTTCATCAGGGTACCGGAATGGTCTTACATCGTGGAGAGGGCTAAC

CCAGCCAACGACCTATGTTACCCAGGGACCCTCAATGACTATGAGGAACTGAAACACCTA

TTGAGCAGAATAAATCATTTTGAGAAAACTCTGATCATCCCCAGGAGTTCTTGGCCCAAT

CATGAAACATCATTAGGGGTGAGCGCAGCATGTCCATACCAGGGAGCATCCTCATTTTTC

AGAAATGTGGTATGGCTCATCAAAAAGAACGATGCATACCCGACAATAAAGATAAGCTAC

AATAATACCAATCGGGAAGATCTTTTGATACTGTGGGGGATTCATCATTCCAACAATGCA

GCAGAGCAGACAAATCTCTATAAAAACCCAGACACTTATGTTTCCGTTGGGACATCAACA

TTAAACCAGAGATTGGTGCCAAAAATAGCTACTAGATCCCAAGTAAACGGGCAGAGTGGA

AGAATGGATTTCTTCTGGACAATTTTAAAACCGAATGATGCAATCCACTTTGAGAGTAAT

GGAAATTTCATTGCTCCAGAATATGCATACAAAATTGTCAAGAAAGGGGACTCAACAATT

ATGAAAAGTGAAATGGAGTATGGCCACTGCAACACCAAATGTCAAACTCCAATAGGGGCG

ATAAACTCTAGCATGCCATTCCACAATATACACCCTCTCACCATCGGGGAATGCCCCAAA

TACGTGAAGTCAAACAAATTAGTCCTTGCGACTGGGCTCAGAAATAGTCCTCTAAGAGAA

AGAAGAAGAAAAAGAGGACTATTTGGAGCTATAGCAGGGTTTATAGAGGGAGGATGGCAG

GGAATGGTAGACGGTTGGTATGGGTATCATCATAGCAATGAGCAGGGGAGTGGGTACGCT

GCAGACAAAGAATCCACCCAAAAGGCAATAGATGGAGTTACCAATAAGGTCAACTCAATC

ATTGACAAAATGAACACTCAATTTGAGGCCGTTGGAAGGGAATTTAATAACTTAGAAAGG

AGAATAGAGAATTTAAACAAGAAAATGGAAGACGGATTCCTAGATGTCTGGACTTATAAT

GCTGAACTTTTAGTTCTCATGGAAAATGAGAGAACTCTAGATTTTCATGACTCAAATGTC

AAGAACCTTTACGACAAAGTCCGACTACAGCTTAGGGATAATGCAAAGGAGCTGGGCAAT

GGTTGTTTCGAGTTCTATCACAAATGTGATAACGAATGTATGGAGAGCGTAAGAAATGGG

ACGTATGACTACCCTAAGTATTCAGAAGAAGCAATATTAAAAAGAGAAGAAATAAGCGGA

GTGAAATTAGAATCAATAGGAACTTACCAGATACTGTCAATTTATTCAACAGTGGCGAGT

TCCCTAGCACTGGCAATCATAGTGGCTGGTCTATCTTTATGGATGTGCTCTAATGGGTCG

TTACAATGCAGAATTTGCATCTAA

>A_chicken_Hubei_ZYSJF38_2016_EPI895118_2344d

ATGGAGAAAATAGTGCTTCTTCTTGCAGTGGTTAGCCTTGTCAAAGGTGATCAGATTTGC

ATTGGTTACCATGCAAACAACTCGACTGAGCAGGTTGACACGATAATGGAAAAAAACGTC

ACTGTTACACATGCTCAAGACATACTGGAAAAGACACACAACGGGAAGCTCTGCGATCTG

AATGGAGTGAAACCTCTGATTTTAAAGGATTGTAGTGTAGCTGGATGGCTTCTTGGAAAC

CCAATGTGCGACGAGTTCATCAGAGTGCCGGAATGGTCTTACATAGTGGAAAGGGCTAAC

CCAGCCAATGACCTCTGTTACCCAGGGAACCTCAATGACTATGAAGAACTGAAACACCTA

TTGAGCAGAATAAATCATTTCGAGAAGACTCTGATCATCCCCAAGAGTTCTTGGCCCAAT

CAT---ACATCATCAGGGGTGAGCGCAGCATGTCCATACCTGGGAAAGCCCTCCTTTTTC

AGAAATGTGGTATGGCTTACCAAGAAGAACGATGCATACCCAACAATAAAAATGAGCTAC

AATAACACCAATAGGGAAGATCTTTTGATACTGTGGGGGATTCATCATTCCAATAATGCA

GAAGAGCAGACAAATCTCTATAAAAACCCAACCACTTATGTTTCCGTTGGGACATCAACA

TTAAACCAGAGAGTGGTGCCAAAAATAGCTACTAGATCCCAAGTAAACGGGCAAAGTGGA

AGAATGGATTTCTTCTGGACAATTTTAAAACCGGATGATGCAATCCACTTCGAGAGTAAT

GGAAATTTTATTGCTCCAGAATATGCATACAAAATTGTCAAGAAAGGGGACTCAACAATT

ATGAAAAGTGAAATGGAATATGGCAATTGCAACACCAAATGTCAAACTCCAATAGGGGCG

ATAAACTCTAGTATGCCATTCCACAATATACACCCTCTCACTATCGGGGAGTGCCCCAAA

TACGTGAAATCAAACAAATTAGTCCTTGCGACTGGGCTCAGAAATAGTCCTCTAAGAGAA

AGAAGAAGAAAAAGAGGACTATTTGGGGCCCTAGCAGGGTTTATAGAGGGAGGATGGCAA

GGAATGGTAGATGGTTGGTATGGGTACCACCATAGCAATGAACAAGGGAGTGGGTATGCT

GCAGACAGAGAATCCACCCAAAAGGCAATAGATGGAGTTACCAATAAGGTCAACTCGATA

ATTGACAAAATGAACACTCAATTTGAGGCCGTTGGAAGGGAATTTAATAACTTAGAACGG

AGAATAGAGAATTTAAATAAGAAAATGGAAGACGGATTCCTAGATGTCTGGACTTATAAT

GCTGAACTTTTAGTTCTCATGGAAAATGAGAGAACTCTAGATTTCCATGACTCAAATGTC

AAGAACCTTTATGACAAAGTCCGACTACAGCTTAGGGATAATGCAAAGGAGCTGGGTAAT

GGTTGTTTCGAGTTCTATCACAAATGTGATAATGAATGTATGGAAAGTGTGAGAAATGGG

ACGTATGACTACCCCCAGTATTCAGAAGAAGCAAGATTAAAAAGGGAAGAAATAAGCGGA

GTGAAATTGGAATCAATAGGAACTTACCAAATACTGTCAATTTATTCAACAGTGGCGGGT

TCCCTAGCACTGGCAATCATTGTGGCTGGTCTATCTTTATGGATGTGCTCCAATGGGTCG

TTACAATGCAGAATTTGCATTTAA

>A_Hubei_29578_2016_x_PR8_CNIC-HB29578_EPI1369965_2344d

ATGGAGAAAATAGTGCTTCTTCTTGCAGTGGTTAGCCTTGTCAAAAGTGATCAGATTTGC

ATTGGTTACCATGCAAACAACTCGACTGAGCAGGTTGACACGATAATGGAAAAAAACGTC

ACTGTTACACATGCTCAAGACATACTGGAAAAGACACACAACGGGAAGCTCTGCGATCTG

AATGGAGTGAAACCTCTGATTTTAAAGGATTGTAGTGTAGCTGGATGGCTTCTTGGAAAC

CCAATGTGCGACGAGTTCATCAGAGTGCCGGAATGGTCTTACATAGTGGAAAGGGCTAAC

CCAGCCAATGACCTCTGTTACCCAGGGAACCTCAATGACTATGAAGAACTGAAACACCTA

TTGAGCAGAATAAATCATTTCGAGAAGACTCTGATCATCCCCAAGAGTTCTTGGCCCAAT

CAT---ACATCATCAGGGGTGAGCGCAGCATGTCCATACCTGGGAAAGCCCTCCTTTTTC

AGAAATGTGGTATGGCTTACCAAGAAGAACGATGCATACCCAACAATAAAAATGAGTTAC

AATAACACCAATAGGGAAGATCTTTTGATACTGTGGGGGATTCATCATTCCAATAATGCA

GAAGAGCAGACAAATCTCTATAAAAACCCAACCACTTATGTTTCCGTTGGGACATCAACA

TTAAACCAGAGAGTGGTGCCAAAAATAGCTACTAGATCCCAAGTAAACGGGCAAAGTGGA

AGAATGGATTTCTTCTGGACAATTTTAAAACCGGATGATGCAATCCACTTCGAGAGTAAT

GGAAATTTTATTGCTCCAGAATATGCATACAAAATTGTCAAGAAAGGGGACTCAACAATT

ATGAAAAGTGAAATGGAATATGGCAATTGCAACACCAAATGTCAAACTCCAATAGGGGCG

ATAAACTCTAGTATGCCATTCCACAATATACACCCTCTCACTATCGGGGAGTGCCCCAAA

TACGTGAAATCAAACAAATTAGTCCTTGCGACTGGGCTCAGAAATAGTCCTCTAAGAGAA

---------ACGAGAGGACTATTTGGGGCCATAGCAGGGTTTATAGAGGGAGGATGGCAA

GGGATGGTAGATGGTTGGTATGGGTACCACCATAGCAATGAACAAGGGAGTGGGTATGCT

GCAGACAGAGAATCCACCCAAAAGGCAATAGATGGAGTTACCAATAAGGTCAACTCGATA

ATTGACAAAATGAACACTCAATTTGAGGCCGTTGGAAGGGAATTTAATAACTTAGAACGG

AGAATAGAGAATTTAAATAAGAAAATGGAAGACGGATTCCTAGATGTCTGGACTTATAAT

GCTGAACTTTTAGTTCTCATGGAAAATGAGAGAACTCTAGATTTCCATGACTCAAATGTC

AAGAACCTTTATGACAAAGTCCGACTACAGCTTAGGGATAATGCAAAGGAGCTGGGTAAT

GGTTGTTTCGAGTTCTATCACAAATGTGATAATGAATGTATGGAAAGTGTGAGAAATGGG

ACGTATGACTACCCCCAGTATTCAGAAGAAGCAAGATTAAAAAGGGAAGAAATAAGCGGA

GTGAAATTGGAATCAATAGGAACTTACCAAATACTGTCAATTTATTCAACAGTGGCGGGT

TCCCTAGCACTGGCAATCATTGTGGCTGGTCTATCTTTATGGATGTGCTCCAATGGGTCG

TTACAATGCAGAATTTGCATTTAA

>A_duck_Hyogo_1_2016_EPI866708_2344e

ATGGAGAAAATAGTGCTTCTTCTTGCAGTGGTTAGCCTTGTTAAAAGTGATCAGATTTGC

ATTGGTTACCATGCAAACAACTCGACAGAGCAGGTTGACACGATAATGGAAAAAAACGTC

ACTGTTACACATGCCCAAGACATACTGGAAAAGACACACAACGGGAGGCTCTGCGATCTG

AATGGAGTGAAACCTCTGATTTTAAAGGATTGTAGTGTAGCTGGATGGCTTCTTGGAAAC

CCAATGTGCGACGAATTCATCAGAGTGCCGGAATGGTCTTACATAGTGGAGAGGACTAAC

CCAGCCAATGACCTCTGTTACCCAGGGAACCTCAATGACTATGAAGAACTGAAACACCTA

TTGAGCAGAATAAATCATTTTGAGAAGACTCTGATCATCCCCAAGAGTTCTTGGCCCAAT

CATGAAACATC---AGGGGTGAGCGCAGCATGCCCATACCAGGGAGTGCCCTCCTTTTTC

AGAAATGTGGTATGGCTTACCAAGAAGAACGATGCATACCCAACAATAAAGATGAGCTAC

AATAATACCAATGGGGAAGATCTTTTGATACTGTGGGGGATTCATCATTCCAACAATGCA

GCAGAGCAGACAAATCTCTATAAAAACCCAACCACCTATGTTTCCGTTGGGACATCAACA

TTAAACCAGAGATTGGTGCCAAAAATAGCTACTAGATCCCAAGTAAACGGGCAACAAGGA

AGAATGGATTTCTTCTGGACAATTTTAAAACCGAATGATGCAATCCACTTTGAGAGTAAT

GGAAATTTTATTGCTCCAGAATATGCATACAAAATAGTCAAGAAAGGGGACTCAACAATT

ATGAAAAGTGAAATGGAATATGGCCACTGCAACACCAAATGTCAAACTCCAATAGGGGCG

ATAAACTCTAGTATGCCATTCCACAATATACACCCTCTCACCATCGGGGAGTGCCCCAAA

TACGTGAAATCAAACAAATTAGTCCTTGCGACTGGACTCAGAAATAGTCCTTTAAGAGAA

AGAAGAAGAAAAAGAGGACTATTTGGAGCTATAGCAGGGTTCATAGAGGGAGGATGGCAA

GGAATGGTAGATGGTTGGTATGGGTACCACCATAGCAATGAACAGGGGAGTGGGTACGCT

GCAGACAGAGAATCCACCCAAAAGGCAATAGATGGAGTTACCAATAAGGTCAACTCGATA

ATCGACAAAATGAACACTCAATTTGAGGCCGTTGGAAGGGAGTTTAATAACTTAGAACGG

AGAATAGAGAATTTAAATAAGAAAATGGAAGACGGATTCCTAGATGTCTGGACTTACAAT

GCTGAACTTTTAGTTCTCATGGAAAATGAGAGAACTTTAGATTTTCACGATTCAAATGTA

AAGAACCTTTATGACAAAGTCAGACTACAGCTTAGGGATAATGCAAAGGAGCTAGGTAAT

GGTTGTTTCGAGTTCTATCATAAATGTGATAATGAATGTATGGAAAGTGTAAGAAATGGG

RCGTATGACTATCCCCAGTATTCAGAAGAGGCAAGATTAAAAAGGGAAGAAATAAGCGGA

GTGAAATTGGAATCAATAGGAACTTACCAAATACTGTCAATTTATTCAACAGTGGCGAGT

TCCCTAGCACTGGCAATCATTGTGGCTGGTCTATCTTTATGGATGTGCTCCAATGGGTCG

TTACAATGCAGAATTTGCATTTAA

>A_tundra_swan_Niigata_5112007_2016_EPI1184362_2344e

ATGGAGAAAATAGTACTTCTTCTTGCAGTGGTTAGCCTTGTTAAAAGTGATCAGATTTGC

ATTGGTTACCATGCAAACAACTCGACAGAGCAGGTTGACACGATAATGGAAAAAAACGTC

ACTGTTACACATGCCCAAGACATACTGGAAAAGACACACAACGGGAGGCTCTGCGATCTG

AATGGAGTGAAACCTCTGATTTTAAAGGATTGTAGTGTAGCTGGATGGCTTCTTGGAAAC

CCAATGTGCGACGAATTCATCAGAGTGCCGGAATGGTCTTACATAGTGGAGAGGACTAAC

CCAGCCAATGACCTCTGTTACCCAGGGAACCTCAATGACTATGAAGAACTGAAACACCTA

TTGAGCAGAATAAATCATTTTGAAAAGACTCTGATCATCCCCAAGAGTTCTTGGCCCAAT

CATGAAACATC---AGGGGCGAGCGCAGCATGCCCATACCAGGGAGTGCCCTCCTTTTTC

AGAAATGTGGTATGGCTTACCAAGAAGAACGATGCATACCCAACAATAAAGATGAGCTAC

AATAATACCAATGGGGAAGATCTTTTGATACTGTGGGGGATTCATCATTCCAACAATGCA

GCAGAGCAGACAAATCTCTATAAAAACCCAACCACCTATGTTTCCGTTGGGACATCAACA

TTAAACCAGAGATTGGTGCCAAAAATAGCTACTAGATCCCAAGTAAACGGGCAACAAGGA

AGAATGGATTTCTTCTGGACAATTTTAAAACCGAATGATGCAATCCACTTTGAGAGTAAT

GGAAATTTTATTGCTCCAGAATATGCATACAAAATAGTCAAGAAAGGGGACTCAACAATT

ATGAAAAGTGAAATGGAATATGGCCACTGCAACACCAAATGTCAAACTCCAATAGGGGCG

ATAAACTCTAGTATGCCATTCCACAATATACACCCTCTCACCATCGGGGAGTGCCCCAAA

TACGTGAAATCAAACAAATTAGTCCTTGCGACTGGACTCAGAAATAGTCCTTTAAGAGAA

AGAAGAAGAAAAAGAGGACTATTTGGAGCTATAGCAGGGTTCATAGAGGGAGGATGGCAA

GGAATGGTAGATGGTTGGTATGGGTACCACCATAGCAATGAACAGGGGAGTGGGTACGCT

GCAGACAGAGAATCCACCCAAAAGGCAATAGATGGAGTTACCAATAAGGTCAACTCGATA

ATCGACAAAATGAACACTCAATTTGAGGCCGTTGGAAGGGAGTTTAATAACTTAGAACGG

AGAATAGAGAATTTAAATAAGAAAATGGAAGACGGATTCCTAGATGTCTGGACTTACAAT

GCTGAACTTTTAGTTCTCATGGAAAATGAGAGAACTTTAGATTTTCACGATTCAAATGTA

AAAAACCTTTATGACAAAGTCCGACTACAGCTTAGGGATAATGCAAAGGAGCTAGGTAAT

GGTTGTTTCGAGTTCTATCATAAATGTGATAATGAATGTATGGAAAGTGTAAGAAATGGG

ACGTATGACTATCCCCAGTATTCAGAAGAAGCAAGATTAAAAAGGGAAGAAATAAGCGGA

GTGAAATTGGAATCAATAGGAACTTACCAAATACTGTCAATTTATTCAACAGTGGCGAGT

TCCCTAGCACTGGCAATCATTGTGGCTGGTCTATCTTTATGGATGTGCTCCAATGGGTCG

TTACAATGCAGAATTTGCATTTAA

>A_chicken_Vietnam_NCVD-15A55_2015_EPI895046_2344f

ATGGAGAAAATAGTGCTTCTTCTTGCAGTGGTTAGCCTTGTTAAAAGTGATCAGATTTGC

ATTGGTTACCATGCAAACAACTCGACAGAGCAGGTTGACACGATAATGGAAAAAAACGTC

ACTGTTACACATGCCCAAGACATACTGGAAAAGACACACAACGGGAGGCTCTGCGATCTG

AATGGAGTGAAACCTCTGATTTTAAAGGATTGTAGTGTAGCTGGATGGCTTCTTGGAAAC

CCAATGTGCGACGAGTTCATCAGAGTGCCGGAATGGTCCTACATAGTGGAAAGGGCTAAC

CCAGCCAATGACCTCTGTTACCCAGGGAATCTCAATGACTATGAAGAACTGAAACACTTA

TTGAGCAGAATAAATCATTTTGAGAAGACTCTGATCATCCCCAAGAGTTCTTGGCCCAAT

CATGAAACATCATTAGGGGTGAGCGCAGCTTGTCCATACCAGGGAATGCCCTCCTTTTTC

AGAAATGTGGTATGGCTTACCAAGAAGAACGATGCATACCCAACAATAAAGATGAGCTAC

AATAATACCAATAGGGAAGATCTTTTGATACTGTGGGGGATTCATCATCCCAACAATGAA

GCAGAGCAAACAAGTATCTATAAAAATCCAACCACCTATGTTTCCGTTGGGACATCAACA

TTAAACCAGAGATTGGTGCCAAAAATAGCTACTAGATCCCAAGTAAACGGGCAACGTGGA

AGAATGGATTTCTTCTGGACAATTTTAAAACCGAATGATGCAATCCACTTCGAGAGTAAT

GGAAATTTTATTGCTCCAGAATATGCATACAAAATTGTCAAGAAAGGGGACTCAACAATT

ATGAAAAGTGAAATGGAATACGGCTACTGCAACACCAAATGTCAAACTCCAATAGGGGCG

ATAAACTCTAGTATGCCATTCCACAATATACACCCTCTCACTATCGGGGAGTGCCCCAAA

TACGTGAAATCAAACAAATTAGTCCTTGCGACTGGGCTCAGAAATAGTCCTCTAAGAGAA

AGAAGAAGAAAAAGAGGACTATTTGGAGCTATAGCAGGTTTTATAGAGGGAGGATGGCAA

GGAATGGTAGATGGTTGGTATGGGTATCACCATAGCAATGAACAGGGGAGTGGGTACGCT

GCAGACAGAGAATCCACCCAAAAGGCAATAGATGGAGTTACCAATAAGGTCAACTCGATC

ATTGACAAAATGAACACTCAATTTGAGGCCGTTGGAAGGGAATTCAATAACTTAGAACGG

AGAATAGAGAATTTAAATAAGAAAATGGAAGACGGATTCCTAGATGTCTGGACTTATAAT

GCTGAACTTTTAGTTCTCATGGAAAATGAGAGAACTCTAGATTTCCATGACTCAAATGTC

AAGAACCTTTATGACAAAGTCCGACTACAGCTTAGGGATAATGCAAAGGAGCTGGGTAAT

GGTTGTTTCGAGTTCTATCACAAATGTGATAATGAATGTATGGAAAGTGTAAGAAATGGG

ACGTACGACTACCCCCAATATTCAGAAGAAGCAAGATTAAAAAGGGAAGAAATAAGCGGA

GTGAAACTGGAATCAATAGGAACTTACCAAATACTGTCAATTTATTCAACAGTGGCGAGT

TCCCTAACACTGGCAATCATCGTGGCTGGTCTATCTTTATGGATGTGCTCCAATGGGTCG

TTACAATGCAGAATTTGCATTTAA

>A_chicken_Vietnam_NCVD-15A59_2015_EPI895070_2344f

ATGGAGAAAATAGTGCTTCTTCTTGCATTGGTTAGCCTTGTTAAAAGTGATCAGATTTGC

ATTGGTTACCATGCAAACAACTCGACAGAGCAGGTTGACACAATAATGGAAAAAAACGTC

ACTGTTACACATGCCCAAGACATACTGGAAAAGACACACAACGGGAGGCTCTGCGATCTG

AATGGAGTGAAACCTCTGATCTTAAAGGATTGTAGTGTAGCTGGATGGCTTCTTGGAAAC

CCAATGTGCGACGAGTTCATCAGAGTGCCGGAATGGTCTTACATAGTGGAGAGGGCTAAC

CCAGCCAATGACCTCTGTTACCCAGGGAATCTCAATGACTATGAAGAACTGAAACACTTA

TTGAGCAGAATAAATCATTTTGAGAAGACTCTGATCATCCCCAAGAGTTCTTGGCCCAAT

CATGAAACATCATTGGGGGTGAGCGCAGCATGTCCATACCAGGGAATGCCCTCCTTTTTC

AGAAATGTGGTATGGCTTACCAAGAAGAACGATGCATACCCAACAATAAAGGTGAGCTAC

AATAATACCAATAGGGAAGATCTTTTGATACTGTGGGGGATTCATCATTCCAACAATGCA

GCAGAGCAGACAAATCTCTATAAAAACCCAACCACCTATGTTTCCGTTGGGACATCAACA

TTAAACCAGAGATTGGTGCCCAAAATAGCTACTAGATCCCAAGTAAACGGGCAACGTGGA

AGAATGGATTTCTTCTGGACAATTTTAAAACCGAATGATGCAATCCACTTCGAGAGTAAT

GGAAATTTTATTGCTCCAGAATATGCATACAAAATTGTCAAGAAAGGGGACTCAACAATT

ATGAAAAGTGAAATGGAATACGGCCACTGCAACACCAAATGTCAAACTCCAATAGGGGCG

ATAAACTCTAGTATGCCATTCCACAATATACACCCTCTCACTATCGGGGAGTGCCCCAAA

TACGTGAAATCAAACAAATTAGTCCTTGCGACTGGGCTCAGAAATAGTCCTCTAAGAGAA

AGAAGAAGAAAAAGAGGGCTATTTGGAGCTATTGCAGGTTTTATAGAGGGAGGCTGGCAG

GGAATGGTAGATGGTTGGTATGGGTACCACCATAGCAATGAACAGGGGAGTGGGTACGCT

GCAGACAGAGAATCCACCCAAAAGGCAATAGATGGAGTTACCAATAAGGTCAACTCGATC

ATTGACAAAATGAACACTCAATTTGAGGCCGTTGGAAGGGAATTTAATAACTTAGAACGG

AGAATAGAGAATTTAAATAAGAAAATGGAAGACGGATTCCTAGATGTCTGGACTTATAAT

GCTGAACTTTTAGTTCTCATGGAAAATGAGAGAACTCTAGATTTCCATGACTCAAATGTC

AAGAACCTTTATGACAAAGTCCGACTACAGCTTAGGGATAATGCAAAGGAGCTGGGTAAT

GGTTGTTTCGAGTTCTATCACAAATGTGATAATGAATGTATGGAAAGTGTAAGAAATGGG

ACGTATGACTACCCCCAATATTCAGAAGAAGCAAGATTAAAAAGGGAAGAAATAAGCGGA

GTGAAACTGGAATCAATAGGAACTTACCAAATACTGTCAATTTATTCAACAGTGGCAAGT

TCCCTAACACTGGCAATCATTGTGGCTGGTCTATCTTTATGGATGTGCTCCAATGGGTCG

TTACAATGCAGAATTTGCATTTAA

>A_duck_Wuhan_JXYFB22_2015_EPI682916_2344g

ATGGAGAAAATAGTGCTTCTTCTTGCAGTGGTTAGCCTTGTTAAAAGTGATCAGATTTGC

ATTGGTTACCATGCAAATAACTCGACAGAGCAGGTTGACACGATAATGGAAAAAAACGTC

ACTGTTACACATGCCCAAGACATACTGGAAAAGACACACAACGGGAGGCTCTGCGATCTG

AATGGAGTGAAACCTCTGATTTTAAAGGATTGTAGTGTAGCTGGATGGCTCCTTGGAAAC

CCAATGTGCGACGAGTTCATCAGAGTGCCGGAATGGTCTTATATAGTGGAGAGGGCTAAC

CCATCCAATGACCTCTGTTACCCAGGGAACCTCAATGACTATGAAGAACTGAAACATCTA

TTGAGCAGAATAAATCATTTTGAGAAGACTCTGATCATCCCCAAGAGTTCTTGGCCCAAT

CATGAAACATCATTAGGGGTGAGCGCAGCATGTCCATACCAGGGAATGCCCTCCTTTTTC

AGAAATGTGGTATGGCTCATCAAGAAGAACGATGCATACCCAACAATAAAGATGAGCTAC

AATAATACCAATAGTGAAGATCTTTTGATACTGTGGGGGATTCATCATTCCAACAACGCA

GCAGAGCAGACAAATCTCTATAAAAACCCAACCACCTATGTTTCCGTTGGGACATCAACA

TTAAACCAGAGATTGGTGCCCAAAATAACTACTAGATCCCAAGTAAACGGGCAACGTGGA

AGAATGGATTTCTTCTGGACAATTTTAAAACCGAATGATGCAATCCACTTCGAGAGTAAT

GGAAATTTTATTGCTCCAGAATATGCATACAAAATTGTCAAGAAAGGGGACTCAACAATC

ATGAAAAGTGAAATGGAATATGGCCATTGCAACACCAAATGTCAAACTCCAATAGGGGCG

ATAAACTCTAGTATGCCATTCCACAACATACACCCTCTCACCATCGGGGAATGCCCCAAA

TACGTGAAATCAAACAAATTAGTCCTTGCGACTGGGCTCAGAAATAGTCCTCTAAGGGAG

AGGAGAAGAAAAAGAGGACTATTTGGAGCTATAGCAGGGTTTATAGAGGGAGGATGGCAA

GGAATGGTAGATGGTTGGTATGGGTACCACCATAGCAATGAACAGGGAAGTGGGTACGCT

GCAGACAAAGAATCCACCCAAAAGGCAATAGATGGAGTTACCAATAAGGTCAACTCGATC

ATTGACAAGATGAACACTCAATTTGAGGCCGTTGGAAGGGAATTTAATAACTTAGAACGG

AGAATAGAGAATTTAAATAAGAAAATGGAAGACGGATTCCTAGATGTCTGGACTTATAAT

GCGGAACTTCTAGTTCTCATGGAAAATGAGAGAACTCTAGATTTCCATGACTCAAATGTC

AAGAACCTTTACGACAAAGTCCGACTACAACTTAGGGATAATGCAAAGGAGCTGGGTAAT

GGTTGTTTCGAGTTCTATCACAAATGTGATAATGAATGTATGGAAAGTGTAAGAAATGGG

ACGTATGACTACCCTCAGTATTCAGAAGAAGCAAGATTAAAAAGAGAAGAAATAAGCGGA

GTGAAATTGGAATCAATAGGAACTTACCAGATACTGTCAATTTATTCAACAGTGGCGAGT

TCCCTAGCACTGGCAATCATTGTGGCTGGTCTATCTTTATGGATGTGCTCCAATGGGTCG

TTACAATGCAGAATTTGCATTTAA

>A_goose_Hunan_116_2014_EPI958632_2344g

ATGGAGAAAATAGTGCTTCTTCTTGCAGTGGTTAGCCTTGTTAAAAGTGATCAGATTTGC

ATTGGTTACCATGCAAATAACTCGACAGAGCAGGTTGACACGATAATGGAAAAAAACGTC

ACTGTTACACATGCCCAAGACATACTGGAAAAGACACACAACGGGAGGCTCTGCGATCTG

AATGGAGTGAAACCTCTGATTTTAAAGGATTGTAGTGTAGCTGGATGGCTCCTTGGAAAC

CCAATGTGCGACGAGTTCATCAGAGTGCCGGAATGGTCTTACATAGTGGAGAGGGCTAAC

CCATCCAATGACCTCTGTTACCCAGGGAACCTCAATGACTATGAAGAACTGAAACATCTA

TTGAGCAGAATAAATCATTTTGAGAAGACTCTGATCATCCCCAAGAGTTCTTGGCCCAAT

CATGAAACATCATTAGGGGTGAGCGCAGCATGTCCATACCAGGGAATGCCCTCCTTTTTC

AGAAATGTGGTATGGCTCATCAAGAAGAACGATGCATACCCAACAATAAAGATGAGCTAC

AATAATACCAATAGTGAAGATCTTTTGATACTGTGGGGGATTCATCATTCCAACAACGCA

GCAGAGCAGACAAATCTCTATAAAAACCCAACCACCTATGTTTCCGTTGGGACATCAACA

TTAAACCAGAGATTGGTGCCCAAAATAGCTACTAGATCCCAAGTAAACGGGCAACGTGGA

AGAATGGATTTCTTCTGGACAATTTTAAAACCGAATGATGCAATCCACTTCGAGAGTAAT

GGAAATTTTATTGCTCCAGAATATGCATACAAAATTGTCAAGAAAGGGGACTCAACAATC

ATGAAAAGTGAAATGGAATATGGCCATTGCAACACCAAATGTCAAACTCCAATAGGGGCG

ATAAACTCTAGTATGCCATTCCACAACATACACCCTCTCACCATCGGGGAATGCCCCAAA

TACGTGAAATCAAACAAATTAGTCCTTGCGACTGGGCTCAGAAATAGTCCTCTAAGGGAG

AGGAGAAGAAAAAGAGGACTATTTGGAGCTATAGCAGGGTTTATAGAGGGAGGATGGCAA

GGAATGGTAGATGGTTGGTATGGGTACCACCATAGCAATGAACAGGGGAGTGGGTACGCT

GCAGACAAAGAATCCACCCAAAAGGCAATAGATGGAGTTACCAATAAGGTCAACTCGATC

ATTGACAAGATGAACACTCAATTTGAGGCCGTTGGAAAGGAATTTAATAACTTAGAACGG

AGAATAGAGAATTTAAATAAGAAAATGGAAGACGGATTCCTAGATGTCTGGACTTATAAT

GCGGAACTTCTAGTTCTCATGGAAAATGAGAGAACTCTAGATTTCCATGACTCAAATGTC

AAGAACCTTTACGACAAAGTTCGACTACAGCTTAGGGATAATGCAAAGGAGCTGGGTAAT

GGTTGTTTCGAGTTCTATCACAAATGTGATAATGAATGTATGGAAAGTGTAAGAAATGGG

ACGTATGACTACCCTCAATATTCAGAAGAAGCAAGATTAAAAAGAGAAGAAATAAGCGGA

GTGAAATTGGAATCAATAGGAACTTACCAGATACTGTCAATTTATTCAACAGTGGCGAGT

TCCCTAGCACTGGCAATCATTGTGGCTGGTCTATCTTTATGGATGTGCTCCAATGGGTCG

TTACAATGCAGAATTTGCATTTAA

>A_Guangdong_18SF020_2018_EPI1352813_2344h

ATGGAGAAAATAGTACTTCTTCTTTCAGTGGTTGGCCTTGTTAAAAGTGATCAGATTTGC

ATTGGTTACCATGCAAACAACTCGACAGAGCAGGTTGACACAATAATGGAAAAAAACGTC

ACTGTCACGCATGCCCAAGACATACTGGAAAAGACACACAACGGGAAGCTCTGCGATCTG

AATGGAGTGAAACCTCTGGTTTTAAAGAATTGTAGTGTAGCTGGATGGCTTCTTGGAAAC

CCAATGTGCGACGAGTTCATCAGCGTGCCGGAATGGTCTTATATAGTGGAGAGGGCTAAC

CCAGCCAATGACCTCTGTTACCCAGGGAACCTCAATGACTATGAAGAACTGAAACACCTA

TTGAGCAGAATAAATCATTTTGAGAAGACTCAGATCATCCCCAAGAGGTCTTGGTCCAAT

CATACATCATC---AGGAGTGAGCGCAGCATGTCCATACCAAGGGGTGGCCTCCTTTTTT

AGAAATGTGGTATGGCTTACCAAGAAGAATGATGCATACCCGACAATAAAGATGAGCTAC

AATAATACCAACAAAGAAGATCTTTTGATACTGTGGGGAATCCATCATTCCAACAGTGCA

GAGGAGCAGACAGATCTCTACAAGAACCCAACCACCTATGTTTCCGTTGGGACATCAACA

CTAAACCAGAGGTTGGTGCCAAAAATAGCTACTAGATCCCAAGTAAATGGGCAACGTGGA

AGAATGGATTTCTTCTGGACAATTTTAAGACCGAATGATGCAATCCACTTCGAGAGTAAT

GGGAATTTTATCGCTCCAGAATATGCATACAAAATTATCAAGACAGGAGACTCAACAATT

ATGAAAAGTGAAATAGAATATGGCAACTGCAACACCAAGTGTCAAACTCCAATAGGGGCG

ATAAACTCTAGTATGCCATTCCACAATATACATCCTCTCACTATCGGGGAGTGCCCCAAA

TATGTGAAATCAAACAAATTAGTCCTTGCGACTGGGCTCAGAAATAGTCCCCTAAGAGAA

AGAAGAAGAAAAAGAGGACTGTTTGGAGCTATAGCAGGGTTTATAGAGGGAGGATGGCAA

GGAATGGTAGATGGTTGGTATGGGTACCACCATAGTAATGAACAGGGGAGTGGGTATGCT

GCAGACAGAGAATCCACCCAAAAGGCAATAGATGGAGTCACCAACAAGGTCAACTCGATA

ATTGACAAAATGAACACTCAATTTGAGGCCGTTGGAAGAGAATTTAATAGCTTAGAACGG

AGAATAGAGAATTTAAATAAGAAAATGGAAGACGGATTCCTAGATGTCTGGACTTATAAC

GCTGAACTTTTAGTTCTCATGGAAAATGAGAGAACTCTAGATTTCCATGACTCAAATGTC

AAGAACCTTTATGACAAAGTCCGACTACAGCTTAGGGATAATGCAAAGGAGCTGGGTAAT

GGTTGTTTCGAGTTCTATCACAAATGTGATAATGAATGTATGGAAAGTGTAAGAAATGGA

ACGTATGACTACCCCCAGTACTCAGAAGAAGCAAGATTAAAAAGGGAAGAAATAAGCGGA

GTGAAATTGGAATCAATAGGAACTTACCAAATACTGTCAATTTATTCAACAGTGGCGAGT

TCCCTAGTACTGGCAATCATTATGGCTGGTCTATCTTTATGGATGTGCTCCAATGGGTCG

TTACAATGCAGAATTTGCATTTAA

>A_duck_Bangladesh_43127_2020_EPI1902988_2344h

ATGGAGAAAACAATACTTCTTTTTTCAGTGGTTAGCCTTGTTAAAAGTGATCAGATTTGC

ATTGGTTACCATGCAAACAACTCGACAGAGCAGGTTGACACAATAATGGAAAAAAACGTC

ACTGTTACGCATGCCCAAGACATACTGGAAAAGACACACAACGGGAAGCTCTGCGATCTG

AATGGAGTAAAACCACTGATTTTAAAGGATTGTAGTGTAGCTGGATGGCTTCTTGGAAAC

CCAATGTGCGACGAGTTCATCAGTGTGCCGGAATGGTCTTATATAGTGGAGAGGGCTAAC

CCAGCCAATGACCTCTGTTACCCGGGGAACCTCAATGACTATGAAGAGCTGAAACACCTA

TTGAGCAGAATAAATCATTTTGAGAAGACTCAGATCATCCCCAAGAGGTCTTGGTCCAAT

CATACATCATC---AGGGGTGAGCGCAGCATGTCCATACCAAGGAGTGGCCTCCTTTTTT

AGAAATGTGGTATGGCTTACCAAGAAGAATGATGCATACCCGACAATAAAGATGAGCTAC

AATAATACCAATAAAGAAGATCTTTTGATACTGTGGGGAATCCATCATTCCAACAGTGCA

GAGGAGCAGATAAGTCTCTACAAGAACCCAACCACCTATGTTTCCGTTGGGACATCAACA

TTAAACCAGAGGTTGGTGCCAAAAATAGCTACTAGATCCCAAGTAAATGGGCAACGTGGA

AGAATGGATTTCTTCTGGACAATTTTAAGACCGAGTGATGCAATCCACTTCGAGAGTAAT

GGAAATTTTATTGCTCCAGAATATGCATACAAAATTATCAAGACAGGAGACTCAACAATT

ATGAAAAGTGAATTAGAATATGGCAACTGCAACACCAAGTGTCAAACTCCAATAGGGGCG

ATAAACTCTAGTATGCCATTCCACAATATACATCCTCTCACCATCGGGGAGTGCCCCAAA

TATGTGAAATCAAACAAATTAGTCCTTGCGACTGGGCTCAGAAATAGTCCTCTAAGAGAA

AGAAGAAGAAAAAGAGGACTGTTTGGAGCCATAGCAGGGTTTATAGAGGGAGGATGGCAA

GGAATGGTAGATGGTTGGTATGGGTACCACCATAGTAATGAGCAGGGGAGTGGATACGCT

GCAGACAGAGAATCCACCCAAAAGGCAATAGATGGAGTCACCAACAAGGTCAATTCGATA

ATTGACAAAATGAACACTCAATTTGAGGCCGTTGGAAGAGAATTTAACAGCTTAGAACGG

AGAATAGAGAATTTAAACAAGAAAATGGAAGACGGATTCCTAGATGTCTGGACTTATAAT

GCTGAACTTTTGGTTCTCATGGAAAATGAGAGAACTCTAGATTTCCATGACTCAAATGTC

AAGAACCTTTATGACAAAGTCCGACTACAGCTTAGGGATAATGCAAAGGAGCTGGGTAAT

GGTTGTTTCGAGTTCTATCACAAATGTGATAATGAATGTATGGAAAGTGTAAGAAATGGA

ACGTATGACTACCCCCAGTACTCAGAAGAAGCAAGATTAAAAAGGGAGGAAATAAGCGGA

GTGAAATTGGAATCAATAGGAACTTACCAAATACTGTCAATTTATTCAACAGTGGCGAGT

TCCCTAGTACTGGCAATCATTGTGGCTGGTCTATCTTTATGGATGTGCTCCAATGGGTCG

TTACAATGCAGAATTTGCATTTAA
