# Supplementary material for: Highly pathogenic avian influenza virus of the A/H5N8 subtype, clade 2.3.4.4b, caused outbreaks in Kazakhstan in 2020
Source: PeerJ. 2022 Mar 2;10:e13038. doi: 10.7717/peerj.13038 (PMC8898005; doi:10.7717/peerj.13038)
Supplement: Figure S2 [file peerj-10-13038-s003.docx]

**Fig.S2** Alignment of the nucleotide sequences for the neuraminidase (NA) segment used in this study

>A_turkey_Egypt_AR550_2018_EPI1420338

ATGAATCCAAGTCAGAAAATAGCGACCATTGGCTCCATTTCATTGGGGTTGGTTATATTC

AATGTTCTACTGCATGCCGTGAGCATCATATTAATGGTGTTAGCCCTGGGGAAAAGTGAA

AACAATGGAATCTGCAAGGGAACTATAGTAAGGGAATATAATGAAACAGTTAGGGTAGAG

AAAGTGACTCAATGGTACAATACTAGTGTAGTCGAATATGTACCGCATTGGAACGAGGGC

GCTTATATAAACAACACCGAACTAATATGTGATGTCAAGGGCTTTGCACCTTTTTCCAAG

GACAACGGGATAAGAGTTGGCTCCAGAGGACATATTTTTGTCATAAGGGAGCCTTTCGTC

TCTTGTTCACCTGTAGAGTGCAGAACTTTCTTCCTCACTCAGGGAGCTCTACTCAATGAC

AAACACTCAAATGGAACAGTGAAGGATAGAAGCCCATTCAGAACTCTCATGAGTGTCGAA

GTGGGTCAATCACCCAATGTGTATCAAGCAAGGTTTGAAGCTGTAGCATGGTCAGCAACA

GCCTGTCATGATGGCAAGAAATGGATGACGATTGGTGTGACAGGGCCAGATTCTAAAGCA

GTAGCAGTAGTCCATTACGGAGGGGTGCCTACTGATGTTGTTAACTCCTGGGCAGGAGAT

ATATTAAGGACTCAGGAGTCATCTTGTACTTGCATTCAAGGTAATTGTTATTGGGTAATG

ACTGACGGTCCAGCCAATAGACAGGCGCAGTACAGAATATACAAAGCAAATCAAGGCAAA

ATAATTGACCAAACAGATGTCAGCTTTAGTGGAGGACATATTGAGGAATGCTCTTGTTAT

CCAAATGATGGTAAAGTGGAATGCGTGTGTAGAGACAACTGGACGGGAACTAACAGGCCT

GTGCTAGTTATTTCTCCTGATCTCTCTTACAGGGTTGGTTATTTATGTGCGGGATTGCCC

AGTGACACTCCAAGAGGGGAAGATGCTCAATTTGTCGGTTCATGCACTAGTCCCATGGGA

AATCAGGGATATGGCGTAAAAGGTTTCGGGTTTCGACAGGGAACTGATGTGTGGGTGGGG

CGGACAATTAGTCGAACCTCCAGGTCAGGGTTTGAAATAATAAGGATAAAGAATGGTTGG

ACGCAGACAAGCAAAGAACAGATTAGAAGGCAGGTGGTTGTTGATAATTTGAATTGGTCG

GGATACAGTGGGTCTTTCACTTTACCAGTAGAATTGTCTGGGAGGGAATGTTTAGTCCCC

TGTTTTTGGGTCGAAATGATCAGAGGCAGGCCAGAAGAAAGAACAATCTGGACCTCTAGT

AGCTCCATTGTAATGTGTGGAGTTGATCATGAAATTGCCGATTGGTCATGGCACGATGGA

GCTATCCTTCCCTTTGACATCGATAAGATGTAA

>A_chicken_Egypt_AR553_2018_EPI1638803

ATGAATCCAAATCAGAAAATAGCGACCATTGGCTCCATTTCATTGGGGTTGGTTGTATTA

AATGTTCTACTGCATGCCGTGAGCATCATATTAATGGTGTTAGCCCTGGGGAAAAGTGAA

AACAATGGAATCTGCAAGGGAACTATAGTAAGGGAATATAATGAAACAGTTAGGATAGAG

AAAGTGACTCAATGGTACAATACTAGTGTAGTCGAATATGTACCGCATTGGAACGAGGGC

ACTTATATAAACAACACCGAACCAATATGTGATGTCAAGGGCTTTGCACCTTTTTCCAAG

GACAACGGGATAAGAGTTGGCTCCAGAGGACATATTTTTGTCATAAGGGAGCCTTTCGTC

TCTTGTTCACCTGTAGAGTGCAGAACTTTCTTCCTCACTCAGGGAGCTCTACTCAATGAC

AAACACTCAAATGGAACAGTGAAGGATAGAAGCCCATTCAGAACTCTCATGAGTGTCGAA

GTGGGTCAATCACCCAATGTGTATCAAGCAAGGTTTGAAGCTGTAGCATGGTCAGCAACA

GCCTGTCATGATGGCAAGAAATGGATGACGATTGGTGTGACAGGGCCAGATTCTAAAGCA

GTAGCAGTAGTCCATTACGGAGGGTTGGTTACTGATGTTGTTAACTCCTGGGCAGGAGAT

ATATTAAGGACTCAGGAGTCATCTTGTACTTGCATTCAAGGTAATTGTTATTGGGTAATG

ACTGACGGTCCAGCCAATAGACAGGCGCAGTACAGAATATACAAAGCAAATCAAGGCAAA

ATAATTGACCAAACAGATGTCAGCTTTAGTGGAGGACATATTGAGGAATGCTCTTGTTAT

CCAAATGATGGTAAAGTGGAATGCGTGTGTAGAGACAACTGGACGGGAACTAACAGGCCT

GTGCTAGTTATTTCACCTGATCTCTCTTACAGGGTTGGGTATTTATGTGCGGGATTGCCC

AGTGACACTCCAAGAGGGGAAGATGCTCAATTTGTCGGTTCATGCACTAGTCCCATGGGA

AATCAGGGATATGGCGTAAAAGGTTTCGGGTTTCGACAGGGAACTGATGTGTGGGTGGGG

CGGACAATTAGTCGAACCTCCAGGTCAGGGTTTGAAATAATAAGGATAAAGAATGGTTGG

ACGCAGACAAGCAAAGAACAGATTAGAAGGCAGGTGGTTGTTGATAATTTGAATTGGTCG

GGATACAGTGGGTCTTTCACTTTACCAGTAGAATTGTCTGGGAGGGAATGTTTAGTCCCC

TGTTTTTGGGTCGAAATGATCAGAGGCAGGCCAGAAGAAAGAACAATCTGGACCTCTAGT

AGCTCCATTGTAATGTGTGGAGTTGATCATGAAATTGCCGATTGGTCATGGCACGATGGA

GCTATCCTTCCCTTTGACATCGATAAGACGTAA

>A_chicken_Egypt_AI20286_2019_EPI1638786

ATGAATCCAAATCAGAAAATAGCGACCATTGGCTCCATTTCATTGGGGTTGGTTGTATTC

AATGTTCTACTGCATGCCGTGAGCATCATATTAATGATGTTAGCCCTGGGGAAAAGTGAA

AACAATGGAATCTGCAAGGGAACTATAGTAAGGGAATATAATGAAACAGTTAGGATAGAG

AAAGTGACTCAATGGTACAATACTAGTGTAGTCGAATATGCACCGCATTGGAACGAGGGC

GCTTATATAAACAACACCGAACCAATATGTGATGTCAAGGGCTTTGCACCTTTTTCCAAG

GACAACGGGATAAGAGTTGGCTCCAGAGGACATATTTTTGTCATAAGGGAGCCTTTCGTC

TCATGTTCACCTGTAGAGTGCAGGACTTTCTTCCTCACTCAGGGAGCTCTACTCAATGAC

AAACACTCAAATGGAACAGTGAAGGATAGAAGCCCATTCAGAACTCTCATGAGTGTCGAA

GTGGGTCAATCACCCAATGTGTATCAAGCAAGGTTTGAAGCTGTAGCATGGTCAGCAACA

GCCTGTCATGATGGCAAGAAATGGATGACGATTGGTGTGACAGGGCCAGATTCTAAAGCA

GTAGCAGTAGTCCATTACGGAGGGGTGCCTACTGATGTTGTTAACTCCTGGGCAGGAGAT

ATATTAAGGACTCAGGAGTCATCTTGTACTTGCATTCAAGGTAATTGTTATTGGGTAATG

ACTGACGGTCCAGCCAATAGACAGGCGCAGTATAGAATATACAAAGCAAATCAAGGCAAA

ATAATTGACCAAACAAATGTCATCTTTAGTGGAGGACACATTGAGGAATGCTCTTGTTAT

CCAAATGATGGTAAAGTGGAATGCGTGTGTAGAGACAACTGGACAGGAACTAACAGGCCT

GTGCTAGTTATTTCGCCTGATCTCTCTTACAGGGTTGGGTATTTATGTGCGGGATTGCCC

AGTGATACTCCAAGAGGGGAAGATGCTCAATTTGTCGGTTCATGCACTAGTCCCATGGGA

AATCAGGGATATGGCGTAAAAGGTTTCGGGTTTCGACAGGGAACTGATGTGTGGGTGGGA

CGGACAATTAGTCGAACCTCCAGGTCAGGGTTTGAAATAATAAGGATAAAGAATGGTTGG

ACGCAGACAAGCAAAGAACAGATTAGAAGGCAGGTGGTTGTTGATAATTTGAACTGGTCG

GGATACAGTGGGTCTTTCACTTTACCAGTAAAATTGTCTGGGAGGGAATGTTTAGTTCCC

TGTTTTTGGGTTGAAATGATCAGAGGCAGGCCAGAAGAAGGAACAATCTGGACCTCTAGT

AGCTCCATTGTAATGTGTGGAGTTGATCATGAAATTGCCGATTGGTCATGGCACGATGGA

GCTATTCTTCCCTTTGACATCGATAAGACGTAA

>A_turkey_Egypt_AI20285_2019_EPI1638799

ATGAATCCAAATCAGAAAATAGCGACCATTGGCTCCATTTCATTAGGGTTGGTTGTATTC

AATGTTCTACTGCATGCCGTGAGCATCATATTAATGGTGTTAGCCCTGGGGAAAAGTGAA

AACAATGGAATCTGCAAGGGAACTATAGTAAGGGAATATAATGAAACAGTTAGGATAGAG

AAAGTGACTCAATGGTACAATACTAGTGTAGTCGAATATGTACCGCATTGGAACGAGGGC

GCTTATATAAACAACACCGAACCAATATGTGATGTCAAGGGCTTTGCACCTTTTTCCAAG

GACAACGGGATAAGAGTTGGCTCCAGGGGACATATTTTTGTCATAAGGGAGCCTTTCGTC

TCTTGTTCACCTGTAGAGTGCAGAACTTTCTTCCTCACTCAGGGAGCTCTACTCAATGAC

AAACACTCAAATGGAACAGTGAAGGATAGAAGCCCATTCAGAACTCTCATGAGTGTCGAA

GTGGGTCAATCACCCAATGTGTATCAAGCAAGGTTTGAAGCTGTAGCATGGTCAGCAACA

GCCTGTCATGATGGCAAGAAATGGATGACGATTGGTGTAACAGGGCCAGATTCTAAAGCA

GTAGCAGTAGTCCATTACGGAGGGGTGCCTACTGATGTTGTTAACTCCTGGGCGGGAGAT

ATATTAAGAACTCAGGAGTCATCTTGTACTTGCATTCAAGGTAATTGTTATTGGGTAATG

ACCGACGGTCCAGCCAATAGACAGGCGCAGTATAGAATATACAAAGCAAATCAAGGCAAA

ATAATTGACCAAACAAACGTCAGTTTTAGTGGAGGGCATATTGAGGAATGCTCTTGTTAT

CCAAATGATGGTAAGGTGGAATGCGTGTGTAGAGACAACTGGACGGGAACTAACAGGCCT

GTGCTAGTTATTTCGCCTGATCTCTCTTACAGGGTTGGGTATTTATGTGCGGGATTGCCC

AGTGACACTCCAAGAGGGGAAGATGCTCAATTTGTCGGTTCATGCACTAGTCCCATGGGA

AATCAGGGATATGGGGTAAAAGGTTTCGGGTTTCGACAGGGAACTGATGTGTGGGTGGGG

CGGACAATTAGTCGAACCTCCAGGTCAGGGTTTGAAATAATAAGGATAAAGAATGGTTGG

ACGCAGACAAGCAAAGAACAGATTAGAAGGCAGGTGGTTGTTGATAATTTGAATTGGTCG

GGATACAGTGGGTCTTTCACTTTACCAGTAGAATTGTCTGGGAGGGAATGTTTAGTCCCC

TGTTTTTGGGTCGAAATGATCAGAGGCAGGCCGGAAGAAAGGACAATCTGGACCTCTAGT

AGCTCCATTGTAATGTGTGGAGTTGATCATGAAATTGCCGATTGGTCATGGCACGATGGA

GCTATTCTTCCCTTTGACATCGATAAGACGTAA

>A_duck_Jiangsu_K1203_2010_EPI442019

ATGAATCCAAATCAGAAAATAATAGCCATCGGCTCCATTTCATTAGGGTTGGTTGTATTC

AATGTTCTACTGCATGCCGTGAGCATCATATTAACAGTGTTAGCCCTGGGGAAAAGTGAA

AACAATGGAATCTGCAATGGAACTGTAGTGAGGGAATATAATGAAACAGTTAGGATAGAG

AAAGTGACTCAATGGTACAATACTAGTGTAGTCGAATATGTACCGCATTGGAATGAGGGC

ACTTATATAAATAACACCGAACCAATATGTGATGTCAAGGGCTTTGCACCTTTTTCCAAG

GACAATGGGATAAGAGTTGGCTCCAGGGGACATATTTTTGTCATAAGAGAGCCTTTCGTC

TCTTGTTCACCTGTAGAGTGCAGGACTTTCTTCCTCACTCAGGGATCTCTACTCAATGAC

AAACACTCAAATGGAACAGTGAAGGATAGAAGCCCATTCAGAACTCTCATGAGTGTCGAA

GTGGGTCAATCACCCAATGTATATCAAGCCAGGTTTGAAGCTGTGGCATGGTCAGCAACA

GCCTGTCATGATGGAAAGAAGTGGATGACGATTGGTGTAACAGGGCCAGATTCTAAAGCA

GTAGCAGTAGTTCATTACGGAGGGGTGCCTACTGATATTGTTAACTCCTGGGCAGGAGAT

ATATTAAGGACTCAGGAGTCATCTTGTACTTGCATTCAAGGTAATTGTTATTGGGTAATG

ACTGACGGTCCTGCCAATAGACAGGCGCAGTATAGAATATACAAAGCAAATCAAGGCAAA

ATAATTGGCCAAACAGATGTTAGCTTTAGTGGAGGACATATTGAAGAATGTTCTTGTTAT

CCAAATGATGGTAAAGTGGAATGCGTGTGTAGAGACAACTGGACGGGAACTAACAGGCCT

GTGCTGGTTATTTCGCCTGATCTCTCTTACAGGGTTGGGTATTTATGTGCAGGGCTGCCC

AGTGACACTCCAAGAGGGGAAGATGCTCAATTTGTCGGTTCATGCACTAGTCCCATGGGA

AATCAGGGATATGGCGTAAAAGGTTTCGGATTTCGACAGGGAACTGATGTGTGGGTGGGG

CGGACAATTAGTCGAACCTCCAGGTCAGGATTTGAAATAATAAGGATAAAGAATGGTTGG

ACGCAAACAAGCAAAGAACAGATTAGAAGACAGGTGGTTGTTGATAATTTGAATTGGTCG

GGATACAGTGGGTCTTTCACTTTACCAGTAGAATTGTCTGGGAGGGAATGTTTGGTTCCC

TGTTTTTGGGTCGAAATGATCAGAGGCAGGCCAGAAGAGAGAACAATCTGGACCTCTAGT

AGCTCCATTGTAATGTGTGGAGTTGATTATGAAATTGCCGATTGGTCATGGCACGATGGA

GCTATTCTTCCCTT-------------------

>A_broiler_duck_Korea_Buan2_2014_EPI509706

ATGAATCCAAATCAGAAAATAGTAACCATTGGCTCCATTTCATTAGGGTTGGTTGTATTC

AATGTTCTACTGCATGCCGTGAGCATCATATTAACAGTGTTAGCCCTGGGGAAGAGTGAA

AACAATGGAATCTGCAATGGAACTGTAGTGAGGGAATACAATGAAACAGTTAGAATAGAG

AAAGTGACTCAATGGTACAATACTAGCGTAGTCGAATATGTACCGCATTGGAATGAGGGC

ACTTATATAAATAACACCGAACCAATATGTGATGTCAAGGGCTTTGCACCTTTTTCCAAG

GACAACGGGATAAGAGTTGGCTCCAGGGGACATATTTTTGTCATAAGAGAGCCTTTCGTC

TCTTGTTCACCTGTAGAGTGCAGGACTTTCTTCCTCACTCAGGGATCTCTACTCAATGAC

AAACACTCAAATGGAACAGTGAAGGATAGAAGCCCATTCAGAACTCTCATGAGTGTCGAA

GTGGGCCAATCACCCAATGTATATCAAGCCAGGTTTGAAGCTGTGGCATGGTCAGCAACA

GCCTGTCATGATGGTAAGAAGTGGATGGCGATTGGTGTAACAGGGCCAGATTCTAAAGCA

GTAGCAGTAGTTCATTACGGAGGGGTGCCTACTGACGTTGTTAACTCCTGGGCAGGAGAT

ATATTAAGAACTCAGGAGTCATCTTGTACTTGCATTCAAGGTAATTGTTATTGGGTAATG

ACTGACGGTCCTGCCAATAGACAGGCGCAGTATAGAATATACAAAGCAAATCAAGGCAAA

ATAATTGGCCGAACAGATGTTAGCTTTAGTGGAGGACATATTGAGGAATGTTCTTGTTAT

CCAAATGATGGTAAAGTGGAATGCGTGTGTAGAGACAACTGGACGGGAACTAACAGGCCT

GTGCTAATTATTTCGCCTGATCTCTCTTACAGGGTTGGGTATTTATGTGCAGGGTTGCCC

AGTGACACTCCAAGAGGGGAAGATACTCAATTTGTCGGTTCATGCACTAGTCCCATGGGA

AATCAGGGATATGGCGTAAAAGGGTTCGGGTTTCGACAGGGAACTGATGTGTGGGTGGGG

CGGACAATTAGTCGAACCTCCAGATCAGGATTTGAAATAATAAGGATAAAGAATGGTTGG

ACGCAAACAAGCAAAGAACAGATTAGAAGACAGGTGGTTGTTGATAACTCGAATTGGTCG

GGATACAGTGGGTCTTTCACTTTACCAGTAGAATTGTCTGGGAGGGAATGTTTGGTTCCC

TGTTTTTGGGTCGAAATGATCAGAGGTAGGCCAGAAGAGAGAACAATCTGGACCTCTAGT

AGCTCCATTGTAATGTGTGGAGTTGATTATGAAATTGCCGATTGGTCATGGCACGATGGA

GCTATTCTTCCCTTTGACATCGATAAGATGTAA

>A_goose_Taiwan_TNO15_2015_EPI690746

ATGAATCCAAATCAGAAAATAGTAACCATTGGCTCCATTTCATTAGGGTTGGTTGTATTC

AATGTTCTACTGCATGCCGTGAGCATCATATTAACAGTGTTAGCCCTGGGGAAGAGTGAA

AACAATGGAATCTGCAATGGAACTGTAGTGAGGGAACACAATGAAACAGTTAGAATAGAG

AAAGTGACTCAATGGTACAATACTAGCGTAGTCGAATATGTACCGCATTGGAATGAGGGA

ACTTATATAAACAACACCGAACCAATATGTGATGTCAAGGGCTTTGCACCTTTTTCCAAG

GACAACGGGGTGAGAGTTGGCTCCAGGGGGCATATTTTTGTCATAAGAGAGCCTTTCGTC

TCTTGTTCACCAGTAGGGTGCAGGACTTTCTTCCTCACTCAGGGATCTCTACTCAATGAC

AAACACTCAAATGGAACAGTGAAGGATAGAAGCCCATTCAGAACTCTCATGAGTGTCGAA

GTGGGCCAATCACCCAATGTATATCAAGCCAGGTTTGAAGCTGTGGCATGGTCAGCAACA

GCCTGTCATGATGGTAAGAAGTGGATGGCAATTGGTGTAACAGGGCCAGATTCTAAAGCA

GTAGCAGTAGTTCATTACGGAGGGGTGCCTACTGACGTTGTTAACTCCTGGGCAGGAGAT

ATATTAAGAACTCAGGAGTCATCTTGTACTTGCATTCAAGGTAATTGTTATTGGGTAATG

ACTGACGGTCCTGCCAATAGACAGGCGCAGTATAGAATATACAAAGCAAACCAAGGCAAA

ATAATTGGCCGAAAAGATGTTAGCTTTAGTGGAGGACATATTGAGGAATGTTCTTGTTAT

CCAAATGATGGTAAAGTGGAATGCGTGTGTAGAGACAACTGGACGGGAACTAACAGACCT

GTGCTAATTATTTCGCCTGATCTCTCTTACAGAGTTGGGTATTTATGTGCAGGGTTGCCC

AGTGACACTCCAAGAGGGGAGGATACTCAATTTGTCGGTTCATGCACTAGTCCCATGGGA

AATCAGGGGTATGGCGTAAAAGGGTTCGGGTTTCGACAGGGAACTGATGTGTGGGTGGGG

CGGACAATTAGTCGAACCTCCAGATCAGGATTTGAAATAATAAGGATAAAGAATGGTTGG

ACGCAAACAAGCAAAGAACAGATTAGAAGACAGGTGGTTGTTGATAACTCGAATTGGTCG

GGATACAGTGGGTCTTTCACTTTACCAGCAGAATTGACTGGGAGGGAATGTTTGGTTCCC

TGTTTTTGGGTCGAAATGATCAGAGGTAGGCCAGAAGAGAGAACAATCTGGACCTCTAGT

AGCTCCATTGTAATGTGTGGAGTTGATTATGAAATTGCCGACTGGTCATGGCACGATGGA

GCTATTCTTCCCTTTGACATCGATAAGATGTAA

>A_breeder_duck_Korea_Gochang1_2014_EPI509700

ATGAATCCAAATCAAAAAATAATGACCATTGGCTCCATTTCATTAGGGTTGGCTGTATTC

AATGTTCTACTGCATGCCGTGAGCATCACACTAACGGTGTTAGCCCTGGGGAAAAGTGAA

AACAATGGAATCTGCAAGGGAACTGTAGTGAGGGAATATAATGAAACAGTTAGGATAGAG

AAAGTGACTCAATGGTACAATACTAGTGTAGTCGAATATGTACCGCATTGGAATGAGGGC

ACTTATATAAATAACACCGAACCAATATGTGATGTCAAGGGCTTTGCACCTTTTTCCAAG

GACAACGGGATAAGAGTTGGCTCCAGGGGACATATTTTTGTCATAAGAGAGCCTTTCGTC

TCTTGTTCACCTGTAGAGTGCAGGACTTTCTTCCTCACTCAGGGAGCTCTACTCAATGAC

AAACACTCAAATGGAACAGTGAAGGATAGAAGCCCATTCAGAACTCTCATGAGTGTCGAA

GTGGGTCAATCACCCAATGTATATCAAGCCAGGTTTGAAGCTGTAGCATGGTCAGCAACA

GCCTGTCATGATGGCAAGAAGTGGATGACGATTGGTGTAACAGGGCCAGATTCTAAAGCA

GTAGCAGTAGTTCATTACGGAGGGGTGCCTACTGATGTTGTTAACTCCTGGGCAGGAGAT

ATATTAAGGACTCAGGAGTCATCTTGTACTTGCATTCAAGGTAATTGTTATTGGGTAATG

ACTGACGGTCCTGCCAATAGACAGGCGCAGTATAGAATATACAAAGCAAATCAAGGCAAA

ATAATTGGCCAAACAGATGTTAGCTTTAGTGGAGGACATATTGAGGAGTGTTCTTGTTAT

CCAAATGATGGTAAAGTGGAATGCGTGTGTAGAGACAACTGGACGGGAACTAACAGGCCT

GTGCTAGTTATTTCGCCTGATCTCTCTTACAGGGTTGGGTATTTATGTGCAGGGTTGCCC

AGTGACACTCCAAGAGGGGAAGATGCTCAATTTGTCGGTTCATGCACTAGTCCCATGGGA

AATCAGGGATATGGCGTAAAAGGTTTCGGGTTTCGACAGGGAACTGATGTGTGGGTGGGG

CGGACAATTAGTCGAACCTCCAGGTCAGGATTTGAAATAATAAGGATAAAGAATGGTTGG

ACGCAGACAAGCAAAGAACAGATTAGAAGACAGGTGGTTGTTGATAATTTGAATTGGTCG

GGATACAGTGGGTCTTTCACTTTACCAGTAGAATTGTCTGGGAGGGAATGTTTGGTTCCC

TGTTTTTGGGTCGAAATGATCAGAGGCAGGCCAGAAGAGAGAACAATCTGGACCTCTAGT

AGCTCCATTGTAATGTGTGGAGTTGATTATGAAATTGCCGATTGGTCATGGCACGATGGA

GCTATTCTTCCCTTTGACATCGATAAGATGTAA

>A_whooper_swan_Inner_Mongolia_W1-1_2020_EPI1811646

ATGAATCCAAATCAGAAAATAGCGACCATTGGCTCCATTTCATTGGGACTAGTTGTATTC

AATGTTCTACTGCATGCCTTGAGCATCATATTAATGGTGTTAGCCCTGGGGAAAAGTGAA

AACAATGGAATCTGCAAGGGAACTATAGTAAGGGAATATAATGAAACAGTTAGGATAGAG

AAAGTGACCCAGTGGTACAACACTAGTGTAGTCGAACATGTACCGCATTGGAACGAGGGC

GCTTATATAAACAACACCGAACCAATATGTGATGTCAAGGGCTTTGCACCTTTTTCCAAG

GACAACGGAATAAGAATTGGCTCCAGAGGACATATTTTTGTCATAAGGGAGCCTTTCGTC

TCTTGTTCACCTGTAGAGTGCAGAACTTTCTTCCTCACTCAGGGAGCTCTACTCAATGAC

AAACACTCAAATGGAACAGTGAAGGATAGGAGCCCATTCAGAACTCTCATGAGTGTCGAA

GTGGGTCAATCACCCAATGTGTATCAAGCAAGGTTTGAAGCTGTAGCATGGTCAGCAACA

GCCTGTCATGATGGTAAGAAATGGATGACGATTGGTGTGACAGGGCCAGATTCGAAAGCA

ATAGCAGTAGTCCATTACGGAGGAGTGCCCACTGATATTGTTAACTCCTGGGCAGGAGAC

ATATTACGGACTCAGGAGTCATCTTGTACTTGCATTCAAGGTAATTGTTATTGGGTAATG

ACTGACGGTCCATCCAATAGACAGGCGCAGTATAGAATATACAAAGCAAATCAAGGCAAA

ATAATTGACCAAGCAGATGTCAGCTTTAGTGGAGGGCATATTGAGGAATGCTCTTGTTAT

CCAAATGATGGTAAAGTGGAATGCGTGTGTAGAGACAACTGGATGGGAACTAACAGGCCT

GTGCTAGTTATCTCGCCTGACCTCTCTTACAGGGTTGGGTATTTATGTGCGGGATTGCCC

AGTGACACTCCAAGAGGGGAAGATGCCCAATTTGTCGGTTCGTGCACTAGTCCCATGGGA

AATCAGGGGTATGGCGTAAAAGGTTTCGGGTTTCGACAGGGAACTGATGTGTGGATGGGG

CGGACAATTAGTCGAACCTCCAGGTCAGGGTTTGAAATAATAAGGATAAAGAATGGTTGG

ACGCAGACAAGCAAAGAACAGATTAGAAGGCAGGTGGTTGTTGATAATTTGAATTGGTCG

GGATACAGTGGGTCTTTCACTTTACCAGTAGAATTGTCTGGGAGGGAATGTTTAGTCCCC

TGTTTTTGGGTCGAAATGATCAGAGGCAGGCCAGAAGAAAGAACAATCTGGACCTCTAGT

AGCTCCATTGTAATGTGTGGAGTTGATCATGAAATTGCCGATTGGTCATGGCACGATGGA

GCTATTCTTCCCTTTGACATCGATAAGATGTAA

>A_mute_swan_Inner_Mongolia_W2-1_2020_EPI1811654

ATGAATCCAAATCAGAAAATAGCGACCATTGGCTCCATCTCATTGGGACTAGTTGTATTC

AATGTTCTACTGCATGCCTTGAGCATCATATTAATGGTGTTAGCCCTGGGGAAAAGTGAA

AACAATGGAATCTGCAAGGGAACTATAGTAAGGGAATATAATGAAACAGTTAGGATAGAG

AAAGTGACCCAGTGGTACAACACTAGTGTAGTCGAACATGTACCGCATTGGAACGAGGGC

GCTTATATAAACAACACCGAACCAATATGTGATGTCAAGGGCTTTGCACCTTTTTCCAAG

GACAACGGAATAAGAATTGGCTCCAGAGGACATATTTTTGTCATAAGGGAGCCTTTCGTC

TCTTGTTCACCTGTAGAGTGCAGAACTTTCTTCCTCACTCAGGGAGCTCTACTCAATGAC

AAACACTCAAATGGAACAGTGAAGGATAGGAGCCCATTCAGAACTCTCATGAGTGTCGAA

GTGGGTCAATCACCCAATGTGTATCAAGCAAGGTTTGAAGCTGTAGCATGGTCAGCAACA

GCCTGTCATGATGGTAAGAAATGGATGACGATTGGTGTGACAGGGCCAGATTCGAAAGCA

ATAGCAGTAGTCCATTACGGAGGAGTGCCCACTGATATTGTTAACTCCTGGGCAGGAGAC

ATATTACGGACTCAGGAGTCATCTTGTACTTGCATTCAAGGTAATTGTTATTGGGTAATG

ACTGACGGTCCATCCAATAGACAGGCGCAGTATAGAATATACAAAGCAAATCAAGGCAAA

ATAATTGACCAAGCAGATGTCAGCTTTAGTGGAGGGCATATTGAGGAATGCTCTTGTTAT

CCAAATGATGGTAAAGTGGAATGCGTGTGTAGAGACAACTGGATGGGAACTAACAGGCCT

GTGCTAGTTATCTCGCCTGACCTCTCTTACAGGGTTGGGTATTTATGTGCGGGATTGCCC

AGTGACACTCCAAGAGGGGAAGATGCCCAATTTGTCGGTTCGTGCACTAGTCCCATGGGA

AATCAGGGGTATGGCGTAAAAGGTTTCGGGTTTCGACAGGGAACTGATGTGTGGATGGGG

CGGACAATTAGTCGAACCTCCAGGTCAGGGTTTGAAATAATAAGGATAAAGAATGGTTGG

ACGCAGACAAGCAAAGAACAGATTAGAAGGCAGGTGGTTGTTGATAATTTGAATTGGTCG

GGATACAGTGGGTCTTTCACTTTACCAGTAGAATTGTCTGGGAGGGAATGTTTAGTCCCC

TGTTTTTGGGTCGAAATGATCAGAGGCAGGCCAGAAGAAAGAACAATCTGGACCTCTAGT

AGCTCCATTGTAATGTGTGGAGTTGATCATGAAATTGCCGATTGGTCATGGCACGATGGA

GCTATTCTTCCCTTTGACATCGATAAGATGTAA

>A_duck_Northern_China_ZGL_2020_EPI1844092

ATGAATCCAAATCAGAAAATAGCGACCATTGGCTCCATCTCATTGGGACTAGTTGTATTC

AATGTTCTACTGCATGCCTTGAGCATCATATTAATGGTGTTAGCCCTGGGGAAAAGTGAA

AACAATGGAATCTGCAAGGGAACTGTAGTAAGGGAATATAATGAAACAGTTAGGATAGAG

AAAGTAACCCAGTGGTACAACACTAGTGTAGTCGAACATGTACCGCATTGGAACGAGGGC

GCTTATATAAACAACACCGAACCAATATGTGATGTCAAGGGCTTTGCACCTTTTTCCAAG

GACAACGGAATAAGAATTGGCTCCAGAGGACATATTTTTGTCATAAGGGAGCCTTTCGTC

TCTTGTTCACCTGTAGAGTGCAGAACTTTCTTCCTCACTCAGGGAGCTCTACTCAATGAC

AAACACTCAAATGGAACAGTGAAGGATAGGAGCCCATTCAGAACTCTCATGAGTGTCGAA

GTGGGTCAATCACCCAATGTGTATCAAGCAAGGTTTGAAGCTGTAGCATGGTCAGCAACA

GCCTGTCATGATGGTAAGAAATGGATGACGATTGGTGTGACAGGGCCAGATTCGAAAGCA

ATAGCAGTAGTCCATTACGGAGGAGTGCCCACTGATATTGTTAACTCCTGGGCAGGAGAC

ATATTACGGACTCAGGAGTCATCTTGTACTTGCATTCAAGGTAATTGTTATTGGGTAATG

ACTGACGGTCCATCCAATAGACAGGCGCAGTATAGAATATACAAAGCAAATCAAGGCAAA

ATAATTGACCAAGCAGATGTCAGCTTTAGTGGAGGGCATATTGAGGAATGCTCTTGTTAT

CCAAATGATGGTAAAGTGGAATGCGTGTGTAGAGACAACTGGATGGGAACTAACAGGCCT

GTGCTAGTTATCTCGCCTGACCTCTCTTACAGGGTTGGGTATTTATGTGCGGGATTGCCC

AGTGACACTCCAAGAGGGGAAGATGCCCAATTTGTCGGTTCGTGCACTAGTCCCATGGGA

AATCAGGGGTATGGCGTAAAAGGTTTCGGGTTTCGACAGGGAACTGATGTGTGGATGGGG

CGGACAATTAGTCGAACCTCCAGGTCAGGGTTTGAAATAATAAAGATAAAGAATGGTTGG

ACGCAGACAAGCAAAGAACAGATTAGAAGGCAGGTGGTTGTTGATAATTTGAATTGGTCG

GGATACAGTGGGTCTTTCACTTTACCAGTAGAATTGTCTGGGAGGGAATGTTTAGTCCCC

TGTTTTTGGGTCGAAATGATCAGAGGCAGGCCAGAAGAAAGAACAATCTGGACCTCTAGT

AGCTCCATTGTAATGTGTGGAGTTGATCATGAAATTGCCGATTGGTCATGGCACGATGGA

GCTATTCTTCCCTTTGACATCGATGGGATGTAA

>A_duck_Southwestern_China_B1904_2020_EPI1844103

ATGAATCCAAATCAGAAAATAGCGACCATTGGCTCCATCTCATTGGGACTAGTTGTATTC

AATGTTCTACTGCATGCCTTGAGCATCATATTAATGGTGTTAGCCCTGGGGAAAAGTGAA

AACAATGGAATCTGCAAGGGAACTATAGTAAGGGAATATAATGAAACAGTTAGGATAGAG

AAAGTGACCCAGTGGTACAACACTAGTGTAGTCGAACATGTACCGCATTGGAACGAGGGC

GCTTATATAAACAACACCGAACCAATATGTGATGTCAAGGGCTTTGCACCTTTTTCCAAG

GACAACGGAATAAGAATTGGCTCCAGAGGACATATTTTTGTCATAAGGGAGCCTTTCGTC

TCTTGTTCACCCATAGAGTGCAGAACTTTCTTCCTCACTCAGGGAGCTCTACTCAATGAC

AAACACTCAAATGGAACAGTGAAGGATAGGAGCCCATTCAGAACTCTCATGAGTGTCGAA

GTGGGTCAATCACCCAATGTGTATCAAGCAAGGTTTGAAGCTGTAGCATGGTCAGCAACA

GCCTGTCATGATGGTAAGAAATGGATGACGATTGGTGTGACAGGGCCAGATTCGAAAGCA

ATAGCAGTAGTCCATTACGGAGGAGTGCCCACTGATATTGTTAACTCCTGGGCAGGAGAC

ATATTACGGACTCAGGAGTCATCTTGTACTTGCATTCAAGGTAATTGTTATTGGGTAATG

ACTGACGGTCCATCCAATAGACAGGCGCAGTATAGAATATACAAAGCAAATCAAGGCAAA

ATAATTGACCAAGCAGATGTCAGCTTTAGTGGAGGGCATATTGAGGAATGCTCTTGTTAT

CCAAATGATGGTAAAGTGGAATGCGTGTGTAGAGACAACTGGATGGGAACTAACAGGCCT

GTGCTAGTTATCTCGCCTGACCTCTCTTACAGGGTTGGGTATTTATGTGCGGGATTGCCC

AGTGACACTCCAAGAGGGGAAGATGCCCAATTTGTCGGTTCGTGCACTAGTCCCATGGGA

AATCAGGGGTATGGCGTAAAAGGTTTCGGGTTTCGACAGGGAACTGATGTGTGGATGGGG

CGGACAATTAGTCGAACCTCCAGGTCAGGGTTTGAAATAATAAGGATAAAGAATGGTTGG

ACGCAGACAAGCAAAGAACAGATTAGAAGGCAGGTGGTTGTTGATAATTTGAATTGGTCG

GGATACAGTGGGTCTTTCACTTTACCAGTAGAATTGTCTGGGAGGGAATGTTTAGTCCCC

TGTTTTTGGGTCGAAATGATCAGAGGCAGGCCAGAAGAAAGAACAATCTGGACCTCTAGT

AGCTCCATTGTAATGTGTGGAGTTGATCATGAAATTGCCGATTGGTCATGGCACGATGGA

GCTATTCTTCCCTTTGACATCGATGGGATGTAA

>A_duck_Korea_H411_2020_EPI1845936

ATGAATCCAAATCAGAAAATAGCGACCATTGGCTCCATCTCATTGGGACTAGTTGTATTC

AATGTTCTACTGCATGCCTTAAGCATCATATTAATGGTGTTAGCCCTGGGGAAAAGTGAA

AACAATGGAATCTGCAAGGGAACTATAGTAAGGGAATATAATGAAACAGTTAGGATAGAG

AAAGTGACCCAGTGGTACAACACTAGTGTAGTCGAACATGTACCGCATTGGAACGAGGGC

GCTTATATAAACAACACCGAACCAATATGTGATGTCAAGGGCTTTGCACCTTTTTCCAAG

GACAACGGAATAAGAATTGGCTCCAGAGGACATATTTTTGTCATAAGGGAGCCTTTCGTC

TCTTGTTCACCTGTAGAGTGCAGAACTTTCTTCCTCACTCAGGGAGCTCTACTCAATGAC

AAACACTCAAATGGAACAGTGAAGGATAGGAGCCCATTCAGAACTCTCATGAGTGTCGAA

GTGGGTCAATCACCCAATGTGTATCAAGCAAGGTTTGAAGCTGTAGCATGGTCAGCAACA

GCCTGTCATGATGGTAAGAAATGGATGACGATTGGTGTGACAGGGCCAGATTCGAAAGCA

ATAGCAGTAGTCCATTACGGAGGAGTGCCCACTGATATTGTTAACTCCTGGGCAGGAGAC

ATATTACGGACTCAGGAGTCATCTTGTACTTGCATTCAAGGTAATTGTTATTGGGTAATG

ACTGACGGTCCATCCAATAGACAGGCGCAGTATAGAATATACAAAGCAAATCAAGGCAAA

ATAATTGACCAAGCAGATGTCAGCTTTAGTGGAGGGCATATTGAGGAATGCTCTTGTTAT

CCAAATGATGGTAAAGTGGAATGCGTGTGTAGAGACAACTGGATGGGAACTAACAGGCCT

GTGCTAGTTATCTCGCCTGACCTCTCTTACAGGGTTGGGTATTTATGTGCGGGATTGCCC

AGTGACACTCCAAGAGGGGAAGATGCCCAATTTGTCGGTTCGTGCACTAGTCCCATGGGA

AATCAGGGGTATGGCGTAAAAGGTTTCGGGTTTCGACAGGGAACTGATGTGTGGATGGGG

CGGACAATTAGTCGAACCTCCAGGTCAGGGTTTGAAATAATAAGGATAAAGAATGGTTGG

ACGCAGACAAGCAAAGAACAGATTAGAAAGCAGGTGGTTGTTGATAATTTGAATTGGTCG

GGATACAGTGGGTCTTTCACTTTACCAGTAGAATTGTCTGGGAGGGAATGTTTAGTCCCC

TGTTTTTGGGTCGAAATGATCAGAGGCAGGCCAGAAGAAAGAACAATCTGGACCTCTAGT

AGCTCCATTGTAATGTGTGGAGTTGATCATGAAATTGCCGATTGGTCATGGCACGATGGA

GCTATTCTTCCCTTTGACATCGATGGGATGTAA

>A_duck_Korea_H431_2020_EPI1845960

ATGAATCCAAATCAGAAAATAGCGACCATTGGCTCCATCTCATTGGGACTAGTTGTATTC

AATGTTCTACTGCATGCCTTGAGCATCATATTAATGGTGTTAGCCCTGGGGAAAAGTGAA

AACAATGGAATCTGCAAGGGAACTATAGTAAGGGAATATAATGAAACAGTTAGGATAGAG

AAAGTGACCCAGTGGTACAACACTAGTGTAGTCGAACATGTACCGCATTGGAACGAGGGC

GCTTATATAAACAACACCGAACCAATATGTGATGTCAAGGGCTTTGCACCTTTTTCCAAG

GACAACGGAATAAGAATTGGCTCCAGAGGACATATTTTTGTCATAAGGGAGCCTTTCGTC

TCTTGTTCACCTGTAGAGTGCAGAACTTTCTTCCTCACTCAGGGAGCTCTACTCAATGAC

AAACACTCAAATGGAACAGTGAAGGATAGGAGCCCATTCAGAACTCTCATGAGTGTCGAA

GTGGGTCAATCACCCAATGTGTATCAAGCAAGGTTTGAAGCTGTAGCATGGTCAGCAACA

GCCTGTCATGATGGTAAGAAATGGATGACGATTGGTGTGACAGGGCCAGATTCGAAAGCA

ATAGCAGTAGTCCATTACGGAGGAGTGCCCACTGATATTGTTAACTCCTGGGCAGGAGAC

ATATTACGGACTCAGGAGTCATCTTGTACTTGCATTCAAGGTAATTGTTATTGGGTAATG

ACTGACGGTCCATCCAATAGACAGGCGCAGTATAGAATATACAAAGCAAATCAAGGCAAA

ATAATTGGCCAAGCAGATGTCAGCTTTAGTGGAGGGCATATTGAGGAATGCTCTTGTTAT

CCAAATGATGGTAAAGTGGAATGCGTGTGTAGAGACAACTGGATGGGAACTAACAGGCCT

GTGCTAGTTATCTCGCCTGACCTCTCTTACAGGGTTGGGTATTTATGTGCGGGATTGCCC

AGTGACACTCCAAGAGGGGAAGATGCCCAATTTGTCGGTTCGTGCACTAGTCCCATGGGA

AATCAGGGGTATGGCGTAAAAGGTTTCGGGTTTCGACAGGGAACTGATGTGTGGATGGGG

CGGACAATTAGTCGAACCTCCAGGTCAGGGTTTGAAATAATAAGGATAAAGAATGGTTGG

ACGCAGACAAGCAAAGAACAGATTAGAAGGCAGGTGGTTGTTGATAATTTGAATTGGTCG

GGATACAGTGGGTCTTTCACTTTACCAGTAGAATTGTCTGGGAGGGAATGTTTAGTCCCC

TGTTTTTGGGTCGAAATGATCAGAGGCAGGCCAGAAGAAAGAACAATCTGGACCTCTAGT

AGCTCCATTGTAATGTGTGGAGTTGATCATGAAATTGCCGATTGGTCATGGCACGATGGA

GCTATTCTTCCCTTTGACATCGATGGGATGTAA

>A_duck_Korea_H538_2020_EPI1846152

ATGAATCCAAATCAGAAAATAGCGACCATTGGCTCCATCTCATTGGGACTAGTTGTATTC

AATGTTCTACTGCATGCCTTGAGCATCATATTAATGGTGTTAGCCCTGGGGAAAAGTGAA

AACAATGGAATCTGCAAGGGAACTATAGTAAGGGAATATAATGAAACAGTTAGGATAGAG

AAAGTGACCCAGTGGTACAACACTAGTGTAGTCGAACATGTACCGCATTGGAACGAAGGC

GCTTATATAAACAACACCGAACCAATATGTGATGTCAAGGGCTTTGCACCTTTTTCCAAG

GACAACGGAATAAGAATTGGCTCCAGAGGACATATTTTTGTCATAAGGGAGCCTTTCGTC

TCTTGTTCACCTGTAGAGTGCAGAACTTTCTTCCTCACTCAGGGAGCTCTACTCAATGAC

AAACACTCAAATGGAACAGTGAAGGATAGGAGCCCATTCAGAACTCTCATGAGTGTCGAA

GTGGGTCAATCACCCAATGTGTATCAAGCAAGGTTTGAAGCTGTAGCATGGTCAGCAACA

GCCTGTCATGATGGTAAGAAATGGATGACGATTGGTGTGACAGGGCCAGATTCGAAAGCA

ATAGCAGTAGTCCATTACGGAGGAGTGCCCACTGATATTGTTAACTCCTGGGCAGGAGAC

ATATTACGGACTCAGGAGTCATCTTGTACTTGCATTCAAGGTAATTGTTATTGGGTAATG

ACTGACGGTCCATCCAATAGACAGGCGCAGTATAGAATATACAAAGCAAATCAAGGCAAA

ATAATTGGCCAAGCAGATGTCAGCTTTAGTGGAGGGCATATTGAGGAATGCTCTTGTTAT

CCAAATGATGGTAAAGTGGAATGCGTGTGTAGAGACAACTGGATGGGAACTAACAGGCCT

GTGCTAGTTATCTCGCCTGACCTCTCTTACAGGGTTGGGTATTTATGTGCGGGATTGCCC

AGTGACACTCCAAGAGGGGAAGATGCCCAATTTGTCGGTTCGTGCACTAGTCCCATGGGA

AATCAGGGGTATGGCGTAAAAGGTTTCGGGTTTCGACAGGGAACTGATGTGTGGATGGGG

CGGACAATTAGTCGAACCTCCAGGTCAGGGTTTGAAATGATAAGGATAAAGAATGGTTGG

ACGCAGACAAGCAAAGAACAGATTAGAAGGCAGGTGGTTGTTGATAATTTGAATTGGTCG

GGATACAGTGGGTCTTTCACTTTACCAGTAGAATTGTCTGGGAGGGAATGTTTAGTCCCC

TGTTTTTGGGTCGAAATGATCAGAGGCAGGCCAGAAGAAAGAACAATCTGGACCTCTAGT

AGCTCCATTGTAATGTGTGGAGTTGATCATGAAATTGCCGATTGGTCATGGCACGATGGA

GCTATTCTTCCCTTTGACATCGATGGGATGTAA

>A_duck_Korea_H471_2020_EPI1846032

ATGAATCCAAATCAGAAAATAGCGACCATTGGCTCCATCTCATTGGGACTAGTTGTATTC

AATGTTCTACTGCATGCCTTGAGCATCATATTAATGGTGTTAGCCCTGGGGAAAAGTGAA

AACAATGGAATCTGCAAGGGAACTATAGTAAGGGAATATAATGAAACAGTTAGGATAGAG

AAAGTGACCCAGTGGTACAACACTAGTGTAGTCGAACATGTACCGCATTGGAACGAGGGC

GCTTATATAAACAACACCGAACCAATATGTGATGTCAAGGGCTTTGCACCTTTTTCCAAG

GACAACGGAATAAGAATTGGCTCCAGAGGACATATTTTTGTCATAAGGGAGCCTTTCGTC

TCTTGTTCACCTGTAGAGTGCAGAACTTTCTTCCTCACTCAGGGAGCTCTACTCAATGAC

AAACACTCAAATGGAACAGTGAAGGATAGGAGCCCATTCAGAACTCTCATGAGTGTCGAA

GTGGGTCAATCACCCAATGTGTATCAAGCAAGGTTTGAAGCTGTAGCATGGTCAGCAACA

GCCTGTCATGATGGTAAGAAATGGATGACGATTGGTGTGACAGGGCCAGATTCGAAAGCA

ATAGCAGTAGTCCATTACGGAGGAGTGCCCACTGATATTGTTAACTCCTGGGCAGGAGAC

ATATTACGGACTCAGGAGTCATCTTGTACTTGCATTCAAGGTAATTGTTATTGGGTAATG

ACTGACGGTCCATCCAATAGACAGGCGCAGTATAGAATATACAAAGCAAATCAAGGCAAA

ATAATTGGCCAAGCAGATGTCAGCTTTAGTGGAGGGCATATTGAGGAATGCTCTTGTTAT

CCAAATGATGGTAAAGTGGAATGCGTGTGTAGAGACAACTGGATGGGAACTAACAGGCCT

GTGCTAGTTATCTCGCCTGACCTCTCTTACAGGGTTGGGTATTTATGTGCGGGATTGCCC

AGTGACACTCCAAGAGGGGAAGATGCCCAATTTGTCGGTTCGTGCACTAGTCCCATGGGA

AATCAGGGGTATGGCGTAAAAGGTTTCGGGTTTCGACAGGGAACTGATGTGTGGATGGGG

CGGACAATTAGTCGAACCTCCAGGTCAGGGTTTGAAATAATAAGGATAAAGAATGGTTGG

ACGCAGACAAGCAAAGAACAGATTAGAAGGCAGGTGGTTGTTGATAATTTGAATTGGTCG

GGATACAGTGGGTCTTTCACTTTACCAGTAGAATTGTCTGGGAGGGAATGTTTAGTCCCC

TGTTTTTGGGTCGAAATGATCAGAGGCAGGCCAGAAGAAAGAACAATCTGGACCTCTAGT

AGCTCCATTGTAATGTGTGGAGTTGATCATGAAATTGCCGATTGGTCATGGCACGATGGA

GCTATTCTTCCCTTTGACATCGATGGGATGTAA

>A_chicken_Korea_H510_2020_EPI1846056

ATGAATCCAAATCAGAAAATAGCGACCATTGGTTCCATCTCATTGGGACTAGTTGTATTC

AATGTTCTACTGCATGCCTTGAGCATCATATTAATGGTGTTAGCCCTGGGGAAAAGTGAA

AACAATGGAATCTGCAAGGGAACTATAGTAAGGGAATATAATGAAACAGTTAGGATAGAG

AAAGTGACCCAGTGGTACAACACTAGTGTAGTCGAACATGTACCGCATTGGAACGAGGGC

GCTTATATAAACAACACCGAACCAATATGTGATGTCAAGGGCTTTGCACCTTTTTCCAAG

GACAACGGAATAAGAATTGGCTCCAGAGGACATATTTTTGTCATAAGGGAGCCTTTCGTC

TCTTGTTCACCTGTAGAGTGCAGAACTTTCTTCCTCACTCAGGGAGCTCTACTCAATGAC

AAACACTCAAATGGAACAGTGAAGGATAGGAGCCCATTCAGAACTCTCATGAGTGTCGAA

GTGGGTCAATCACCCAATGTGTATCAAGCAAGGTTTGAAGCTGTAGCATGGTCAGCAACA

GCCTGTCATGATGGTAAGAAATGGATGACGATTGGTGTGACAGGGCCAGATTCGAAAGCA

ATAGCAGTAGTCCATTACGGAGGAGTGCCCACTGATATTGTTAACTCCTGGGCAGGAGAC

ATATTACGGACTCAGGAGTCATCTTGTACTTGCATTCAAGGTAATTGTTATTGGGTAATG

ACTGACGGTCCATCCAATAGACAGGCGCAGTATAGAATATACAAAGCAAATCAAGGCAAA

ATAATTGACCAAGCAGATGTCAGCTTTAGTGGAGGGCATATTGAGGAATGCTCTTGTTAT

CCAAATGATGGTAAAGTGGAATGCGTGTGTAGAGACAACTGGATGGGAACTAACAGGCCT

GTGCTAGTTATCTCGCCTGACCTCTCTTACAGGGTTGGGTATTTATGTGCGGGATTGCCC

AGTGACACTCCAAGAGGGGAAGATGCCCAATTTGTCGGTTCGTGCACTAGTCCCATGGGA

AATCAGGGGTATGGCGTAAAAGGTTTCGGGTTTCGACAGGGAACTGATGTGTGGATGGGG

CGGACAATTAGTCGAACCTCCAGGTCAGGGTTTGAAATAATAAGGATAAAGAATGGTTGG

ACGCAGACAAGCAAAGAACAGATTAGAAGGCAGGTGGTTGTTGATAATTTGAATTGGTCG

GGATACAGTGGGTCTTTCACTTTACCAGTAAAATTGTCTGGGAGGGAATGTTTAGTCCCC

TGTTTTTGGGTCGAAATGATCAGAGGCAGGCCAGAAGAAAGAACAATCTGGACCTCTAGT

AGCTCCATTGTAATGTGTGGAGTTGATCATGAAATTGCCGATTGGTCATGGCACGATGGA

GCTATTCTTCCCTTTGACATCGATGGGATGTAA

>A_mallard_Korea_WA820_2020_EPI1846601

ATGAATCCAAATCAGAAAATAGCGACCATTGGCTCCATCTCATTGGGACTAGTTGTATTC

AATGTTCTACTGCATGCCTTGAGCATCATATTAATGGTGTTAGCCCTGGGGAAAAGTGAA

AACAATGGAATCTGCAAGGGAACTATAGTAAGGGAATATAATGAAACAGTTAGGATAGAG

AAAGTGACCCAGTGGTACAACACTAGTGTAGTCGAACATGTACCGCATTGGAACGAGGGC

GCTTATATAAACAACACCGAACCAATATGTGATGTCAAGGGCTTTGCACCTTTTTCCAAG

GACAACGGAATAAGAATTGGCTCCAGAGGACATATTTTTGTCATAAGGGAGCCTTTCGTC

TCTTGTTCACCTGTAGAGTGCAGAACTTTCTTCCTCACTCAGGGAGCTCTACTCAATGAC

AAACACTCAAATGGAACAGTGAAGGATAGGAGCCCATTCAGAACTCTCATGAGTGTCGAA

GTGGGTCAATCACCCAATGTGTATCAAGCAAGGTTTGAAGCTGTAGCATGGTCAGCAACA

GCTTGTCATGATGGTAAGAAATGGATGACGATTGGTGTGACAGGGCCAGATTCGAAAGCA

ATAGCAGTAGTCCATTACGGAGGAGTGCCCACTGACATTGTTAACTCCTGGGCAGGAGAC

ATATTACGGACTCAGGAGTCATCTTGTACTTGCATTCAAGGTAATTGTTATTGGGTAATG

ACTGACGGTCCATCCAATAGACAGGCGCAGTATAGAATATACAAAGCAAATCAAGGCAAA

ATAATTGACCAAGCAGATGTCAGCTTTAGTGGAGGGCATATTGAGGAATGCTCTTGTTAT

CCAAATGATGGTAAAGTGGAATGCGTGTGTAGAGACAACTGGATGGGAACTAACAGGCCT

GTGCTAGTTATCTCGCCTGACCTCTCTTACAGGGTTGGGTATTTATGTGCGGGATTGCCC

AGTGACACTCCAAGAGGGGAAGATGCCCAATTTGTCGGTTCGTGCACTAGTCCCATGGGA

AATCAGGGGTATGGCGTAAAAGGTTTCGGGTTTCGACAGGGAACTGATGTGTGGATGGGG

CGGACAATTAGTCGAACCTCCAGGTCAGGGTTTGAAATAATAAGGATAAAGAATGGTTGG

ACGCAGACAAGCAAAGAACAGATTAGAAGGCAGGTGGTTGTTGATAATTTGAATTGGTCG

GGATACAGTGGGTCTTTCACTTTACCAGTAGAATTGTCTGGGAGGGAATGTTTAGTCCCC

TGTTTTTGGGTCGAAATGATCAGAGGCAGGCCAGAAGAAAGAACAATCTGGACCTCTAGT

AGCTCCATTGTAATGTGTGGAGTTGATCATGAAATTGCCGATTGGTCATGGCACGATGGA

GCTATTCTTCCCTTTGACATCGATGGGATGTAA

>A_domestic_goose_Kazakhstan_1-248_2-20-B_2020_EPI1811603

ATGAATCCAAATCAGAAAATAGCGGCCATTGGCTCCATCTCATTGGGACTAGTTGTATTC

AATGTTCTACTGCATGCCTTGAGCATCATATTAATGGTGTTAGCCCTGGGGAAAAGTGAA

AACAATGGAATCTGCAAGGGAACTATAGTAAGGGAATATAATGAAACAGTTAGGATAGAG

AAAGTGACCCAGTGGTACAACACTAGTGTAGTCGAATATGTACCGCATTGGAACGAGGGC

GCTTATATAAACAACACCGAACCAATATGTGATGTCAAGGGCTTTGCACCTTTTTCCAAG

GACAACGGAATAAGAATTGGCTCCAGAGGACATATTTTTGTCATAAGGGAGCCTTTCGTC

TCTTGTTCACCTGTTGAGTGCAGAACTTTCTTCCTCACTCAGGGAGCTCTACTCAATGAC

AAACACTCAAATGGAACAGTGAAGGATAGGAGCCCATTCAGAACTCTCATGAGTGTCGAA

GTGGGTCAATCACCCAATGTGTATCAAGCAAGGTTTGAAGCTTTAGCATGGTCAGCAACA

GCCTGTCATGATGGTAAGAAATGGATGACGATTGGTGTGACAGGGCCAGATTCGAAAGCA

ATAGCAGTAGTCCATTACGGAGGGGTGCCTACTGATATTGTTAACTCCTGGGCAGGAGAC

ATATTACGGACTCAGGAGTCATCTTGTACTTGCATTCAAGGTAATTGTTATTGGGTAATG

ACTGACGGTCCATCCAATAGACAGGCGCAGTATAGAATATACAAAGCAAATCAAGGCAAA

ATAATTGACCAAGCAGATGTCAGCTTTAGTGGAGGGCATATTGAGGAATGCTCTTGTTAT

CCAAATGATGGTAAAGTGGAATGCGTGTGTAGAGACAACTGGATGGGAACTAACAGGCCT

GTGCTAGTTATCTCGCCTGACCTCTCTTACAGGGTTGGGTATTTATGTGCGGGATTGCCC

AGTGACACTCCAAGAGGGGAAGATGCCCAATTTGTCGGTTCGTGCACTAGTCCCATGGGA

AATCAGGGGTATGGCGTAAAAGGTTTCGGGTTTCGACAGGGAACTGATGTGTGGATGGGG

CGGACAATTAGTCGAACCTCCAGGTCAGGGTTTGAAATAATAAGGATAAAGAATGGTTGG

ACGCAAACAAGCAAAGAACAGATTAGAAGGCAGGTGGTTGTTGATAATTTGAATTGGTCG

GGATACAGTGGGTCTTTCACTTTACCAGTAGAATTGTCTGGGAGGGAATGTTTAGTCCCC

TGTTTTTGGGTCGAAATGATCAGAGGCAGGCCAGAAGAAAGAACAATCTGGACCTCTAGT

AGCTCCATTGTAATGTGTGGAGTTGATCATGAAATTGCCGATTGGTCATGGCACGATGGA

GCTATTCTTCCCTTTGACATCGATGGGATGTAA

>A_mute_swan_Kazakhstan_1-267-20-B_2020_EPI1811586

ATGAATCCAAATCAGAAAATAGCGACCATTGGCTCCATCTCATTGGGACTAGTTGTATTC

AATGTTCTACTGCATGCCTTGAGCATCATATTAATGGTGTTAGCCCTGGGGAAAAGTGAA

AACAATGGAATCTGCAAGGGAACTATAGTAAGGGAATATAATGAAACAGTTAGAATAGAG

AAAGTGACCCAGTGGTACAACACTAGTATAGTTGAATATGTACCGCATTGGAACGAGGGC

GCTTATATAAACAACACCGAACCAATATGTGATGTCAAGGGCTTTGCACCTTTTTCCAAG

GACAACGGAATAAGAATTGGCTCCAGAGGACATATTTTTGTCATAAGGGAGCCTTTCGTC

TCTTGTTCACCTGTAGAGTGCAGAACTTTCTTCCTCACTCAGGGAGCTCTACTCAATGAC

AAACACTCAAATGGAACAGTGAAGGATAGGAGCCCATTCAGAACTCTCATGAGTGTCGAA

GTGGGTCAATCACCCAATGTGTATCAAGCAAGGTTTGAAGCTGTAGCATGGTCAGCAACA

GCCTGTCATGATGGTAAGAAATGGATGACGATTGGTGTGACAGGGCCAGATTCGAAAGCA

ATAGCAGTAGTCCATTACGGAGGAGTGCCTACTGATATTGTTAACTCCTGGGCAGGAGAC

ATATTACGGACTCAGGAGTCATCTTGTACTTGCATTCAAGGTAATTGTTATTGGGTAATG

ACTGACGGTCCATCCAATAGACAGGCGCAGTATAGAATTTACAAAGCAAATCAAGGCAAA

ATAATTGACCAAGCAGATGTCAGCTTTAGTGGAGGGCATATTGAGGAATGCTCTTGTTAT

CCAAATGATGGTAAAGTGGAATGCGTGTGTAGAGACAACTGGATGGGAACTAACAGGCCT

GTGCTAGTTATCTCGCCTGACCTCTCTTACAGGGTTGGGTATTTATGTGCGGGGTTGCCC

AGTGACACTCCAAGAGGGGAAGATGCCCAATTTGTCGGTTCGTGCACTAGTCCCATGGGA

AATCAGGGGTATGGCGTAAAAGGTTTCGGGTTTCGACAGGGAACTGATGTGTGGATGGGG

CGGACAATTAGTCGAACCTCCAGGTCAGGGTTTGAAATAATAAGGATAAAGAATGGTTGG

ACGCAGACAAGCAAAGAACAGATTAGAAGGCAGGTGGTTGTTGATAATTTGAATTGGTCG

GGATACAGTGGGTCTTTCACTTTACCAGTAGAATTGTCTGGGAGGGAATGTTTAGTCCCC

TGTTTTTGGGTCGAAATGATCAGAGGCAGGCCAGAAGAAAGAACAATCTGGACCTCTAGT

AGCTCCATTGTAATGTGTGGAGTTGATCATGAAATTGCCGATTGGTCATGGCACGATGGA

GCTATTCTTCCCTTTAACATCGATGGGATGTAA

>A_goose_Omsk_01161_2020_EPI1813360

ATGAATCCAAATCAGAAAATAGCGACCATTGGCTCCATCTCATTGGGACTAGTTGTATTC

AATGTTCTACTGCATGCCTTGAGCATCATATTAATGGTGTTAGCCCTGGGGAAAAGTGAA

AACAATGGAATCTGCAAGGGAACTATAGTAAGGGAATATAATGAAACAGTTAGGATAGAG

AAAGTGACCCAGTGGTACAACACTAGTGTAGTTGAATATGTACCGCATTGGAACGAGGGC

GCTTATATAAACAACACCGAACCAATATGTGATGTCAAGGGCTTTGCACCTTTTTCCAAG

GACAACGGAATAAGAATTGGCTCCAGAGGACATATTTTTGTCATAAGGGAGCCTTTCGTC

TCTTGTTCACCTGTAGAGTGCAGAACTTTCTTCCTCACTCAGGGAGCTCTACTCAATGAC

AAACACTCAAATGGAACAGTGAAGGATAGGAGCCCATTCAGAACTCTCATGAGTGTCGAA

GTGGGTCAATCACCCAATGTGTATCAAGCAAGGTTTGAAGCTGTAGCATGGTCAGCAACA

GCCTGTCATGATGGTAAGAAATGGATGACGATTGGTGTGACAGGGCCAGATTCGAAAGCA

ACAGCAGTAGTCCATTACGGAGGAGTGCCTACTGATATTGTTAACTCCTGGGCAGGAGAC

ATATTACGGACTCAGGAGTCATCTTGTACTTGCATTCAAGGTAATTGTTATTGGGTAATG

ACTGACGGTCCATCCAATAGACAGGCGCAGTATAGAATATACAAAGCAAATCAAGGCAAA

ATAATTGACCAAGCAGATGTCAGCTTTAGTGGAGGGCATATTGAGGAATGCTCTTGTTAT

CCAAATGATGGTAAAGTGGAATGCGTGTGTAGAGACAACTGGATGGGAACTAACAGGCCT

GTGCTAGTTATCTCGCCTGACCTCTCTTACAGGGTTGGGTATTTATGTGCGGGATTGCCC

AGTGACACTCCAAGAGGGGAAGATGCCCAATTTGTCGGTTCGTGCACTAGTCCCATGGGA

AATCAGGGGTATGGCGTAAAAGGTTTCGGGTTTCGACAGGGAACTGATGTGTGGATGGGG

CGGACAATTAGTCGAACCTCCAGGTCAGGGTTTGAAATAATAAGGATAAAGAATGGTTGG

ACGCAGAAAAGCAAAGAACAGATTAGAAGGCAGGTGGTTGTTGATAATTTGAATTGGTCG

GGATACAGTGGGTCTTTCACTTTACCAGTAGAATTGTCTGGGAGGGACTGTTTAGTCCCC

TGTTTTTGGGTCGAAATGATCAGAGGCAGGCCAGAAGAAAGAACAATCTGGACCTCTAGT

AGCTCCATTGTAATGTGTGGAGTTGATCATGAAATTGCCGATTGGTCATGGCACGATGGA

GCTATTCTTCCCTTTGACATCGATGGGATGTAA

>A_goose_Omsk_0002_2020_EPI1813120

ATGAATCCAAATCAGAAAATAGCGACCATTGGCTCCATCTCATTGGGACTAGTTGTATTC

AATGTTCTACTGCATGCCTTGAGCATCATATTAATGGTGTTAGCCCTGGGGAAAAGTGAA

AACAATGGAATCTGCAAGGGAACTATAGTAAGGGAATATAATGAAACAGTTAGGATAGAG

AAAGTGACCCAGTGGTACAACACTAGTGTAGTTGAATATGTACCGCATTGGAACGAGGGC

GCTTATATAAACAACACCGAACCAATATGTGATGTCAAGGGCTTTGCACCTTTTTCCAAG

GACAACGGAATAAGAATTGGCTCCAGAGGACATATTTTTGTCATAAGGGAGCCTTTCGTC

TCTTGTTCACCTGTAGAGTGCAGAACTTTCTTCCTCACTCAGGGAGCTCTACTCAATGAC

AAACACTCAAATGGAACAGTGAAGGATAGGAGCCCATTCAGAACTCTCATGAGTGTCGAA

GTGGGTCAATCACCCAATGTGTATCAAGCAAGGTTTGAAGCTGTAGCATGGTCAGCAACA

GCCTGTCATGATGGTAAGAAATGGATGACGATTGGTGTGACAGGGCCAGATTCGAAAGCA

ATAGCAGTAGTCCATTACGGAGGAGTGCCTACTGATATTGTTAACTCCTGGGCAGGAGAC

ATATTACGGACTCAGGAGTCATCTTGTACTTGCATTCAAGGTAATTGTTATTGGGTAATG

ACTGACGGTCCATCCAATAGACAGGCGCAGTATAGAATATACAAAGCAAATCAAGGCAAA

ATAATTGACCAAGCAGATGTCAGCTTTAGTGGAGGGCATATTGAGGAATGCTCTTGTTAT

CCAAATGATGGTAAAGTGGAATGCGTGTGTAGAGACAACTGGATGGGAACTAACAGGCCT

GTGCTAGTTATCTCGCCTGACCTCTCTTACAGGGTTGGGTATTTATGTGCGGGATTGCCC

AGTGACACTCCAAGAGGGGAAGATGCCCAATTTGTCGGTTCGTGCACTAGTCCCATGGGA

AATCAGGGGTATGGCGTAAAAGGTTTCGGGTTTCGACAGGGAACTGATGTGTGGATGGGG

CGGACAATTAGTCGAACCTCCAGGTCAGGGTTTGAAATAATAAGGATAAAGAATGGTTGG

ACGCAGACAAGCAAAGAACAGATTAGAAGGCAGGTGGTTGTTGATAATTTGAATTGGTCG

GGATACAGTGGGTCTTTCACTTTACCAGTAGAATTGTCTGGGAGGGAATGTTTAGTCCCC

TGTTTTTGGGTCGAAATGATCAGAGGCAGGCCAGAAGAAAGAACAATCTGGACCTCTAGT

AGCTCCATTGTAATGTGTGGAGTTGATCATGAAATTGCCGATTGGTCATGGCACGATGGA

GCTATTCTTCCCTTTGACATCGATGGGATGTAA

>A_chicken_Omsk_30007_2020_EPI1814312

ATGAATCCAAATCAGAAAATAGCGACCATTGGCTCCATCTCATTGGGACTAGTTGTATTC

AATGTTCTACTGCATGCCTTGAGCATCATATTAATGGTGTTAGCCCTGGGGAAAAGTGAA

AACAATGGAATCTGCAAGGGAACTATAGTAAGGGAATATAATGAAACAGTTAGGATAGAG

AAAGTGACCCAGTGGTACAACACTAGTGTAGTTGAATATGTACCGCATTGGAACGAGGGC

GCTTATATAAACAACACCGAACCAATATGTGATGTCAAGGGCTTTGCACCTTTTTCCAAG

GACAACGGAATAAGAATTGGCTCCAGAGGACATATTTTTGTCATAAGGGAGCCTTTCGTC

TCTTGTTCACCTGTAGAGTGCAGAACTTTCTTCCTCACTCAGGGAGCTCTACTCAATGAC

AAACACTCAAATGGAACAGTGAAGGATAGGAGCCCATTCAGAACTCTCATGAGTGTCGAA

GTGGGTCAATCACCCAATGTGTATCAAGCAAGGTTTGAAGCTGTAGCATGGTCAGCAACA

GCCTGCCATGATGGTAAGAAATGGATGACGATTGGTGTGACAGGGCCAGATTCAAAAGCA

ATAGCAGTAGTCCATTACGGAGGAGTGCCTACTGATATTGTTAACTCCTGGGCAGGAGAC

ATATTACGGACTCAGGAGTCATCTTGTACTTGCATTCAAGGTAATTGTTATTGGGTAATG

ACTGACGGTCCATCCAATAGACAGGCGCAGTATAGAATATACAAAGCAAATCAAGGCAAA

ATAATTGACCAAGCAGATGTCAGCTTTAGTGGAGGGCATATTGAGGAATGCTCTTGTTAT

CCAAATGATGGTAAAGTGGAATGCGTGTGTAGAGACAACTGGATGGGAACTAACAGGCCT

GTGCTAGTTATCTCGCCTGACCTCTCTTACAGGGTTGGGTATTTATGTGCGGGATTGCCC

AGTGACACTCCAAGAGGGGAAGATGCCCAATTTGTCGGTTCGTGCACTAGTCCCATGGGA

AATCAGGGGTATGGCGTAAAAGGTTTCGGGTTTCGACAGGGAACTGATGTGTGGATGGGG

CGGACAATTAGTCGAACCTCCAGGTCAGGGTTTGAAATAATAAGGATAAAGAATGGTTGG

ACGCAGACAAGCAAAGAACAAATTAGAAGGCAGGTGGTTGTTGATAATTTGAATTGGTCG

GGATACAGTGGGTCTTTCACTTTACCAGTAGAATTGTCTGGGAGGGAATGTTTAGTCCCC

TGTTTTTGGGTCGAAATGATCAGAGGCAGGCCAGAAGAAAGAACAATCTGGACCTCTAGT

AGCTCCATTGTAATGTGTGGAGTTGATCATGAAATTGCCGATTGGTCATGGCACGATGGA

GCTATTATTCCCTTTGACATCGATGGGATGTAA

>A_goose_Omsk_30009_2020_EPI1814320

ATGAATCCAAATCAGAGAATAGCGACCATTGGCTCCATCTCATTGGGACTAGTTGTATTC

AATGTTCTACTGCATGCCTTGAGCATCATATTAATGGTGTTAGCCCTGGGGAAAAGTGAA

AACAATGGAATCTGCAAGGGGACTATAGTAAGGGAATATAATGAAACAGTTAGGATAGAG

AAAGTGACCCAGTGGTACAACACTAGTGTAGTTGAATATGTACCGCATTGGAACGAGGGC

GCTTATATAAACAACACCGAACCAATATGTGATGTCAAGGGCTTTGCACCTTTTTCCAAG

GACAACGGAATAAGAATTGGCTCCAGAGGACATATTTTTGTCATAAGGGAGCCTTTCGTC

TCTTGTTCACCTGTCGAGTGCAGAACTTTCTTCCTCACTCAGGGAGCTCTACTCAATGAC

AAACACTCAAATGGAACAGTGAAGGATAGGAGCCCATTCAGAACTCTCATGAGTGTCGAA

GTGGGTCAATCACCCAATGTGTATCAAGCAAGGTTTGAAGCTGTAGCATGGTCAGCAACA

GCCTGTCATGATGGTAAGAAATGGATGACGATTGGTGTGACAGGGCCAGATTCGAAAGCA

ATAGCAGTAGTCCATTACGGAGGAGTGCCTACTGATATTGTTAACTCCTGGGCAGGAGAC

ATATTACGGACTCAGGAGTCATCTTGTACTTGCATTCAAGGTAATTGTTATTGGGTAATG

ACTGACGGTCCATCCAATAGACAGGCGCAGTATAGAATATACAAAGCAAATCAAGGCAAA

ATAATTGACCAAGCAGATGTCAGCTTTAGTGGAGGGCATATTGAGGAATGCTCTTGTTAT

CCAAATGATGGTAAAGTGGAATGCGTGTGTAGAGACAACTGGATGGGAACTAACAGGCCT

GTGCTAGTTATCTCGCCTGACCTCTCTTACAGGGTTGGGTATTTATGTGCGGGATTGCCC

AGTGACACTCCAAGAGGGGAAGATGCCCAATTTGTCGGTTCGTGCACTAGTCCCATGGGA

AATCAGGGGTATGGCGTAAAAGGTTTCGGGTTTCGACAGGGAACTGATGTGTGGATGGGG

CGGACAATTAGTCGAACCTCCAGGTCAGGGTTTGAAATAATAAGGATAAAGAATGGTTGG

ACGCAGACAAGCAAAGAACAGATTAGAAGGCAGGTGGTTGTTGATAATTTGAATTGGTCG

GGATACAGTGGGTCTTTCACTTTACCAGTAGAATTGTCTGGGAGGGAATGTTTAGTCCCC

TGTTTTTGGGTCGAAATGATCAGAGGCAGGCCAGAAGAAAGAACAATCTGGACCTCTAGT

AGCTCCATTGTAATGTGTGGAGTTGATCATGAAATTGCCGATTGGTCATGGCACGATGGA

GCTATTCTTCCCTTTGACATCGATGGGATGTAA

>A_domestic_duck_Kazakhstan_1-274-20-B_2020_EPI1811613

ATGAATCCAAATCAGAAAATAGCGACCATTGGCTCCATCTCATTGGGACTAGTTGTATTC

AATGTTCTACTGCATGCCTTGAGCATCATATTAATGGTGTTAGCCCTGGGGAAAAGTGAA

AACAATGGAATCTGCAAGGGAACTATAGTAAGGGAATATAATGAAACAGTTAGGATAGAG

AAAGTGACCCAGTGGTACAACACTAGTGTAGTCGAATATGTACCGCATTGGAACGAGGGC

GCTTATATAAACAACACCGAACCAATATGTGATGTCAAGGGCTTTGCACCTTTTTCCAAG

GACAACGGAATAAGAATTGGCTCCAGAGGACATATTTTTGTCATAAGGGAGCCTTTCGTC

TCTTGTTCACCTGTAGAGTGCAGAACTTTCTTCCTCACTCAGGGAGCTCTACTCAATGAC

AAACACTCAAATGGAACAGTGAAGGATAGGAGCCCATTCAGAACTCTCATGAGTGTCGAA

GTGGGTCAATCACCCAATGTGTATCAAGCAAGGTTTGAAGCTGTAGCATGGTCAGCAACA

GCCTGTCATGATGGTAAGAAATGGATGACGATTGGTGTGACAGGACCAGATTCGAAAGCA

ATAGCAGTAGTCCATTACGGAGGAGTGCCTACTGATATTGTTAACTCCTGGGCAGGAGAC

ATATTACGGACTCAGGAGTCATCTTGTACTTGCATTCAAGGTAATTGTTATTGGGTAATG

ACTGACGGTCCATCCAATAGACAGGCGCAGTATAGAATATACAAAGCAAATCAAGGCAAA

ATAATTGACCAAGCAGATGTCAGCTTTAGTGGAGGGCATATTGAGGAATGCTCTTGTTAT

CCAAATGATGGTAAAGTGGAATGCGTGTGTAGAGACAACTGGATGGGAACTAACAGGCCT

GTGCTAGTTATCTCGCCTGACCTCTCTTACAGGGTTGGGTATTTATGTGCGGGATTGCCC

AGTGACACTCCAAGAGGGGAAGATGCCCAATTTGTCGGTTCGTGCACTAGTCCCATGGGA

AATCAGGGGTATGGCGTAAAAGGTTTCGGGTTTCGACAGGGAACTGATGTGTGGATGGGG

CGGACAATTAGTCGAACTTCCAGGTCAGGGTTTGAAATAATAAGGATAAAGAATGGTTGG

ACGCAGACAAGCAAAGAACAGATTAGAAGGCAAGTGGTTGTTGGTAATTTGAATTGGTCG

GGATACAGTGGGTCTTTCACTTTACCAGTAGAATTGTCTGGGAGGGAATGTTTAGTCCCC

TGTTTTTGGGTCGAAATGATCAGAGGCAGGCCAGAAGAAAGAACAATCTGGACTTCTAGT

AGCTCCATTGTAATGTGTGGAGTTGATCATGAAATTGCCGATTGGTCATGGCACGATGGA

GCTATTCTTCCCTTTGACATCGATGGGATGTAA

>A_goose_Omsk_01171_2020_EPI1813200

ATGAATCCAAATCAGAAAATAGCGACCATTGGCTCCATCTCATTGGGACTAGTTGTATTC

AATGTTCTACTGCATGCCTTGAGCATCATATTAATGGTGTTAGCCCTGGGGAAAAGTGAA

AACAATGGAATCTGCAAGGGAACTATAGTAAGGGAATATAATGAAACAGTTAGGATAGAG

AAAGTGACCCAGTGGTACAACACTAGTGTAGTCGAATATGTACCGCATTGGAACGAGGGC

GCTTATATAAACAACACCGAACCAATATGTGATGTCAAGGGCTTTGCACCTTTTTCCAAG

GACAACGGAATAAGAATTGGCTCCAGAGGACATATTTTTGTCATAAGGGAGCCTTTCGTC

TCTTGTTCACCTGTAGAGTGCAGAACTTTCTTCCTCACTCAGGGAGCTCTACTCAATGAC

AAACACTCAAATGGAACAGTGAAGGATAGGAGCCCATTCAGAACTCTCATGAGTGTCGAA

GTGGGTCAATCACCCAATGTGTATCAAGCAAGGTTTGAAGCTGTAGCATGGTCAGCAACA

GCCTGTCATGATGGTAAGAAATGGATGACGATTGGTGTGACAGGACCAGATTCGAAAGCA

ATAGCAGTAGTCCATTACGGAGGAGTGCCTACTGATATTGTTAACTCCTGGGCAGGAGAC

ATATTACGGACTCAGGAGTCATCTTGTACTTGCATTCAAGGTAATTGTTATTGGGTAATG

ACTGACGGTCCATCCAATAGACAGGCGCAGTATAGAATATACAAAGCAAATCAAGGCAAA

ATAATTGACCAAGCAGATGTCAGCTTTAGTGGAGGGCATATTGAGGAATGCTCTTGTTAT

CCAAATGATGGTAAAGTGGAATGCGTGTGTAGAGACAACTGGATGGGAACTAACAGGCCT

GTGCTAGTTATCTCGCCTGACCTCTCTTACAGGGTTGGGTATTTATGTGCGGGATTGCCC

AGTGACACTCCAAGAGGGGAAGATGCCCAATTTGTCGGTTCGTGCACTAGTCCCATGGGA

AATCAGGGGTATGGCGTAAAAGGTTTCGGGTTTCGACAGGGAACTGATGTGTGGATGGGG

CGGACAATTAGTCGAACTTCCAGGTCAGGGTTTGAAATAATAAGGATAAAGAATGGTTGG

ACGCAGACAAGCAAAGAACAGGTTAGAAGGCAAGTGGTTGTTGATAATTTGAATTGGTCG

GGATACAGTGGGTCTTTCACTTTACCAGTAGAATTGTCTGGGAGGGAATGTTTAGTCCCC

TGTTTTTGGGTCGAAATGATCAGAGGCAGGCCAGAAGAAAGAACAATCTGGACCTCTAGT

AGCTCCATTGTAATGTGTGGAGTTGATCATGAAATTGCCGATTGGTCATGGCACGATGGA

GCTATTCTTCCCTTTGACATCGATGGGATGTAA

>A_chicken_Omsk_0119_2020_EPI1813384

ATGAATCCAAATCAGAAAATAGCGACCATTGGCTCCATCTCATTGGGACTAGTTGTATTC

AATGTTCTACTGCATGCCTTGAGCATCATATTAATGGTGTTAGCCCTGGGGAAAAGTGAA

AACAATGGAATCTGCAAGGGAAATATAGTAAGGGAATATAATGAAACAGTTAGGATAGAG

AAAGTGACCCAGTGGTACAACACTAGTGTAGTCGAATATGTACCGCATTGGAACGAGGGC

GCTTATATAAACAACACCGAACCAATATGTGATGTCAAGGGCTTTGCACCTTTTTCCAAG

GACAACGGAATAAGAATTGGCTCCAGAGGACATATTTTTGTCATAAGGGAGCCTTTCGTC

TCTTGTTCACCTGTAGAGTGCAGAACTTTCTTCCTCACTCAGGGAGCTCTACTCAATGAC

AAACACTCAAATGGAACAGTGAAGGATAGGAGCCCATTCAGAACTCTCATGAGTGTCGAA

GTGGGTCAATCACCCAATGTGTATCAAGCAAGGTTTGAAGCTGTAGCATGGTCAGCAACA

GCCTGTCATGATGGTAAGAAATGGATGACGATTGGTGTGACAGGACCAGATTCGAAAGCA

ATAGCAGTAGTCCATTACGGAGGAGTGCCTACTGATATTGTTAACTCCTGGGCAGGAGAC

ATATTACGGACTCAGGAGTCATCTTGTACTTGCATTCAAGGTAATTGTTATTGGGTAATG

ACTGACGGTCCATCCAATAGACAGGCGCAGTATAGAATATACAAAGCAAATCAAGGCAAA

ATAATTGACCAAGCAGATGTCAGCTTTAGTGGAGGGCATATTGAGGAATGCTCTTGTTAT

CCAAATGATGGTAAAGTGGAATGCGTGTGTAGAGACAACTGGATGGGAACTAACAGGCCT

GTGCTAGTTATCTCGCCTGACCTCTCTTACAGGGTTGGGTATTTATGTGCGGGATTGCCC

AGTGACACTCCAAGAGGGGAAGATGCCCAATTTGTCGGTTCGTGCACTAGTCCCATGGGA

AATCAGGGGTATGGCGTGAAAGGTTTCGGGTTTCGACAGGGAACTGATGTGTGGATGGGG

CGGACAATTAGTCGAACTTCCAGGTCAGGGTTTGAAATAATAAGGATAAAGAATGGTTGG

ACGCAGACAAGCAAAGAACAGATTAGAAGGCAAGTGGTTGTTGATAATTTGAATTGGTCG

GGATACAGTGGGTCTTTCACTTTACCAGTAGAATTGTCTGGGAGGGAATGTTTAGTCCCC

TGTTTTTGGGTCGAAATGATCAGAGGCAGGCCAGAAGAAAGAACAATCTGGACCTCTAGT

AGCTCCATTGTAATGTGTGGAGTTGATCATGAAATTGCCGATTGGTCATGGCACGATGGA

GCTATTCTTCCCTTTGACATCGATGGGATGTAA

>A_turkey_Poland_464_2020_EPI1841312

ATGAATCCAAATCAGAAAATAGCGACCATTGGCTCCATCTCATTGGGACTAGTTGTATTC

AATGTTCTACTGCATGCCTTGAGCATCATATTAATGGTGTTAGCCCTGGGGAAAAGTGAA

AACAATGGAATCTGCAAGGGAACTATAGTAAGGGAATATAATGAAACAGTTAGGATAGAG

AAAGTGACCCAGTGGCACAACACTAGTGTAGTCGAATATGTACCGCATTGGAACGAGGGC

GCTTATATAAACAACACCGAACCAATATGTGATGTCAAGGGCTTTGCACCTTTTTCCAAG

GACAACGGAATAAGAATTGGATCCAGAGGACATATTTTTGTCATAAGGGAGCCTTTCGTC

TCTTGTTCACCTGTAGAGTGCAGAACTTTCTTCCTCACTCAGGGAGCTCTACTCAATGAC

AAACACTCAAATGGAACAGTGAAGGATAGGAGCCCATTCAGAACTCTCATGAGTGTCGAA

GTGGGTCAATCACCCAATGTGTATCAAGCAAGGTTTGAAGCTGTAGCATGGTCAGCAACA

GCCTGTCATGATGGTAAGAAATGGATGACGATTGGTGTGACAGGACCAGATTCGAAAGCA

ATAGCAGTAGTCCATTACGGAGGAGTGCCTACTGATATTGTCAACTCCTGGGCAGGAGAC

ATATTACGGACTCAGGAGTCATCTTGTACTTGCATTCAAGGTAATTGTTATTGGGTAATG

ACTGACGGTCCATCCAATAGACAGGCGCAGTATAGAATATACAAAGCAAATCAAGGCAAA

ATAATTGACCAAGCAGATGTCAGCTTTAGTGGAGGGCATATTGAGGAATGCTCTTGTTAT

CCAAATGATGGTAAAGTGGAATGCGTGTGTAGAGACAACTGGATGGGAACTAACAGGCCT

GTGCTAGTTATCTCGCCTGACCTCTCTTACAGGGTTGGGTATTTATGTGCGGGATTGCCC

AGTGACACTCCAAGAGGGGAAGATGCCCAATTTGTCGGTTCGTGCACTAGTCCCATGGGA

AATCAGGGGTATGGCGTAAAAGGTTTCGGGTTTCGACAGGGAACTGATGTGTGGATGGGG

CGGACAATTAGTCGAACTTCCAGGTCAGGGTTTGAAATAATAAGGATAAAGAATGGTTGG

ACGCAGACAAGCAAAGAACAGATTAGAAGGCAAGTGGTTGTTGATAATTTGAATTGGTCG

GGATACAGTGGGTCTTTCACTTTACCAGTAGAATTGTCTGGGAGGGAATGTTTAGTCCCC

TGTTTTTGGGTCGAAATGATCAGAGGCAGGCCAGAAGAAAGAACAATCTGGACCTCTAGT

AGCTCCATTGTAATGTGTGGAGTTGATCATGAAATTGCCGATTGGTCATGGCACGATGGA

GCTATTCTTCCCTTTGACATCGATGGGATGTAA

>A_chicken_Astrakhan_321-01_2020_EPI1846971

ATGAATCCAAATCAGAAAATAGCGACCATTGGCTCCATCTCATTGGGACTAGTTGTATTC

AATGTTCTACTGCATGCCTTGAGCATCATATTAATGGTGTTAGCCCTGGGGAAAAGTGAA

AACAATGGAATCTGCAAGGGAACTATAATAAGGGAATATAATGAAACAGTTAGGATAGAG

AAAGTGACCCAGTGGTACAACACTAGTGTAGTCGAATATGTACCGCATTGGAACGAGGGC

GCTTATATAAACAACACCGAACCAATATGTGATGTCAAGGGCTTTGCACCTTTTTCCAAG

GACAACGGAATAAGAATTGGCTCCAGAGGACATATTTTTGTCATAAGGGAGCCTTTCGTC

TCTTGTTCACCTGTAGAGTGCAGAACTTTCTTCCTCACTCAGGGAGCTCTACTCAATGAC

AAACACTCAAATGGAACAGTGAAGGATAGGAGCCCATTCAGAACTCTCATGAGTGTCGAA

GTGGGTCAATCACCCAATGTGTATCAAGCAAGGTTTGAAGCTGTAGCATGGTCAGCAACA

GCCTGTCATGATGGTAAGAAATGGATGACGATTGGTGTGACAGGACCAGATTCGAAAGCA

ATAGCAGTAGTTCATTACGGAGGAGTGCCTACTGATATTGTTAACTCCTGGGCAGGAGAC

ATATTACGGACTCAGGAGTCATCTTGTACTTGCATTCAAGGTAATTGTTATTGGGTAATG

ACTGACGGTCCATCCAATAGACAGGCGCAGTATAGAATATACAAAGCAAATCAAGGCAAA

ATAATTGACCAAGCAGATGTCAGCTTTAGTGGAGGGCATATTGAGGAATGCTCTTGTTAT

CCAAATGATGGTAAAGTGGAATGCGTGTGTAGAGACAACTGGATAGGAACTAACAGGCCT

GTGCTAGTTATCTCGCCTGACCTCTCTTACAGGGTTGGGTATTTATGTGCGGGATTGCCC

AGTGACACTCCAAGAGGGGAAGATGCCCAATTTGTCGGTTCGTGCACTAGTCCCATGGGA

AATCAGGGGTATGGCGTAAAAGGTTTCGGGTTTCGACAGGGAACTGATGTGTGGATGGGG

CGGACAATTAGTCGAACTTCCAGGTCAGGGTTTGAAATAATAAGGATAAAGAATGGTTGG

ACGCAGACAAGCAAAGAACAGATTAGAAGGCAAGTGGTTGTTGATAATTTGAATTGGTCG

GGATACAGTGGGTCTTTCACTTTACCAGTAGAATTGTCTGGGAGGGAATGTTTAGTCCCC

TGTTTTTGGGTCGAAATGATCAGAGGCAGGCCAGAAGAAAGAACAATCTGGACCTCTAGT

AGCTCCATTGTAATGTGTGGAGTTGATCATGAAATTGCCGATTGGTCATGGCACGATGGA

GCTATTCTTCCCTTTGACATCGATGGGATGTAA

>A_Astrakhan_3212_2020_EPI1846963

ATGAATCCAAATCAGAAAATAGCGACCATTGGCTCCATCTCATTGGGACTAGTTGTATTC

AATGTTCTACTGCATGCCTTGAACATCATATTAATGGTGTTAGCCCTGGGGAAAAGTGAA

AACAATGGAATCTGCAAGGGAACTATAATAAGGGAATATAATGAAACAGTTAGGATAGAG

AAAGTGACCCAGTGGTACAACACTAGTGTAGTCGAATATGTACCGCATTGGAACGAGGGC

GCTTATATAAACAACACCGAACCAATATGTGATGTCAAGGGCTTTGCACCTTTTTCCAAG

GACAACGGAATAAGAATTGGCTCCAGAGGACATATTTTTGTCATAAGGGAGCCTTTCGTC

TCTTGTTCACCTGTAGAGTGCAGAACTTTCTTCCTCACTCAGGGAGCTCTACTCAATGAC

AAACACTCAAATGGAACAGTGAAGGATAGGAGCCCATTCAGAACTCTCATGAGTGTCGAA

GTGGGTCAATCACCCAATGTGTATCAAGCAAGGTTTGAAGCTGTAGCATGGTCAGCAACA

GCCTGTCATGATGGTAAGAAATGGATGACGATTGGTGTGACAGGACCAGATTCGAAAGCA

ATAGCAGTAGTTCATTACGGAGGAGTGCCTACTGATATTGTTAACTCCTGGGCAGGAGAC

ATATTACGGACTCAGGAGTCATCTTGTACTTGCATTCAAGGTAATTGTTATTGGGTAATG

ACTGACGGTCCATCCAATAGACAGGCGCAGTATAGAATATACAAAGCAAATCAAGGCAAA

ATAATTGACCAAGCAGATGTCAGCTTTAGTGGAGGGCATATTGAGGAATGCTCTTGTTAT

CCAAATGATGGTAAAGTGGAATGCGTGTGTAGAGACAACTGGATAGGAACTAACAGGCCT

GTGCTAGTTATCTCGCCTGACCTCTCTTACAGGGTTGGGTATTTATGTGCGGGATTGCCC

AGTGACACTCCAAGAGGGGAAGATGCCCAATTTGTCGGTTCGTGCACTAGTCCCATGGGA

AATCAGGGGTATGGCGTAAAAGGTTTCGGGTTTCGACAGGGAACTGATGTGTGGATGGGG

CGGACAATTAGTCGAACTTCCAGGTCAGGGTTTGAAATAATAAGGATAAAGAATGGTTGG

ACGCAGACAAGCAAAGAACAGATTAGAAGGCAAGTGGTTGTTGATAATTTGAATTGGTCG

GGATACAGTGGGTCTTTCACTTTACCAGTAGAATTGTCTGGGAGGGAATGTTTAGTCCCC

TGTTTTTGGGTCGAAATGATCAGAGGCAGGCCAGAAGAAAGAACAATCTGGACCTCTAGT

AGCTCCATTGTAATGTGTGGAGTTGATCATGAAATTGCCGATTGGTCATGGCACGATGGA

GCTATTCTTCCCTTTGACATCGATGGGATGTAA

>A_chicken_Omsk_0118_2020_EPI1813376

ATGAATCCAAATCAGAAAATAGCGACCATTGGCTCCATCTCATTGGGACTAGTTGTATTC

AATGTTCTACTGCATGCCTTGAGCATCATATTAATGGTGTTAGCCCTGGGGAAAAGTGAA

AACAATGGAATCTGCAAGGGAACTATAGTAAGGGAATATAATGAAACAGTTAGGATAGAG

AAAGTGACCCAGTGGTACAACACTAGTGTAGTCGAATATGTACCGCATTGGAACGAGGGC

GCTTATATAAACAACACCGAACCAATATGTGATGTCAAGGGCTTTGCACCTTTTTCCAAG

GACAACGGAATAAGAATTGGCTCCAGAGGACATATTTTTGTCATAAGGGAGCCTTTCGTC

TCTTGTTCACCTGTAGAGTGCAGAACTTTCTTCCTCACTCAGGGAGCTCTACTCAATGAC

AAACACTCAAATGGAACAGTGAAGGATAGGAGCCCATTCAGAACTCTCATGAGTGTCGAA

GTGGGTCAATCACCCAATGTGTATCAAGCAAGGTTTGAAGCTGTAGCATGGTCAGCAACA

GCCTGTCATGATGGTAAGAAATGGATGACGATTGGTGTGACAGGACCAGATTCGAAAGCA

ATAGCAGTAGTCCATTACGGAGGAGTGCCTACTGATGTTGTTAACTCCTGGGCAGGAGAC

ATATTACGGACTCAGGAGTCATCTTGTACTTGCATTCAAGGTAATTGTTATTGGGTAATG

ACTGACGGTCCATCCAATAGACAGGCGCAGTATAGAATATACAAAGCAAATCAAGGCAAA

ATAATTGACCAAGCAGATGTCAGCTTTAGTGGAGGGCATATTGAGGAATGCTCTTGTTAT

CCAAATGATGGTAAAGTGGAATGCGTGTGTAGAGACAACTGGATGGGAACTAACAGGCCT

GTGCTAGTTATCTCGCCTGACCTCTCTTACAGGGTTGGGTATTTATGTGCGGGATTGCCC

AGTGACACTCCAAGAGGGGAAGATGCCCAATTTGTCGGTTCGTGCACTAGTCCCATGGGA

AATCAGGGGTATGGCGTAAAAGGTTTCGGGTTTCGACAGGGAACTGATGTGTGGATGGGG

CGGACAATTAGTCGAACTTCCAGGTCAGGGTTTGAAATAATAAGGATAAAGAATGGTTGG

ACGCAGACAAGCAAAGAACAGATTAGAAGGCAAGTGGTTGTTGATAATTTGAATTGGTCG

GGATACAGTGGGTCTTTCACTTTACCAGTAGAATTGTCTGGGAGGGAATGTTTAGTCCCC

TGTTTTTGGGTCGAAATGATCAGAGGCAGGCCAGAAGAAAGAACAATCTGGACCTCTAGT

AGCTCCATTGTAATGTGTGGAGTTGATCATGAAATTGCCGATTGGTCATGGCACGATGGA

GCTATTCTTCCCTTTGACATCGATGGGATGTAA

>A_mute_swan_Slovenia_1639-20_21VIR959-1_2020_EPI1858299

ATGAATCCAAATCAGAAAATAGCGACCATTGGCTCCATCTCATTGGGACTAGTTGTATTC

AATGTTCTACTGCATGCCTTGAGCATCATATTAATGGTGTTAGCCCTGGGGAAAAGTGAA

AACAATGGAATCTGCAAGGGAACTATAGTAAGGGAATATAATGAAACAGTTAGGATAGAG

AAAGTGACCCAGTGGTACAACACTAGTGTAGTCGAATATGTACCGCATTGGAACGAGGGC

GCTTATATAAACAACACCGAACCAATATGTGATGTCAAGGGCTTTGCACCTTTTTCCAAG

GACAACGGAATAAGAATTGGCTCCAGAGGACATATTTTTGTCATAAGGGAGCCTTTCGTC

TCTTGTTCACCTGTAGAGTGCAGAACTTTCTTCCTCACTCAGGGAGCTCTACTCAATGAC

AAACACTCAAATGGAACAGTGAAGGATAGGAGCCCATTCAGAACTCTCATGAGTGTCGAA

GTGGGTCAATCACCCAATGTGTATCAAGCAAGGTTTGAAGCTGTAGCATGGTCAGCAACA

GCCTGTCATGATGGTAAGAAATGGATGACGATTGGTGTGACAGGACCAGATTCGAAAGCA

ATAGCAGTAGTCCATTACGGAGGAGTGCCTACTGATATTGTTAACTCCTGGGCAGGAGAC

ATATTACGGACTCAGGAGTCATCTTGTACTTGCATTCAAGGTAATTGTTATTGGGTAATG

ACTGACGGTCCATCCAATAGACAGGCGCAGTATAGAATATACAAAGCAAATCAAGGCAAA

ATAATTGACCAAGCAGATGTCAGCTTTGGTGGAGGGCATATTGAGGAATGCTCTTGTTAT

CCAAATGATGGTAAAGTGGAATGCGTGTGTAGAGACAACTGGATGGGAACTAACAGGCCT

GTGCTAGTTATCTCGCCTGACCTCTCTTACAGGGTTGGGTATTTATGTGCGGGATTGCCC

AGTGACACTCCAAGAGGGGAAGATGCCCAATTTGTCGGTTCGTGCACTAGTCCCATGGGA

AATCAGGGGTATGGCGTAAAAGGTTTCGGGTTTCGACAGGGAACTGATGTGTGGATGGGG

CGGACAATTAGTCGAACTTCCAGGTCAGGGTTTGAAATAATAAGGATAAAGAATGGTTGG

ACGCAGACAAGCAAAGAACAGATTAGAAGGCAAGTGGTTGTTGATAATTTGAATTGGTCG

GGATACAGTGGGTCTTTCACTTTACCAGTAGAATTGTCTGGGAGGGAATGTTTAGTCCCC

TGTTTTTGGGTCGAAATGATCAGAGGCAGGCCAGAAGAAAGAACAATCTGGACCTCTAGT

AGCTCCATTGTAATGTGTGGAGTTGATCATGAGATTGCCGATTGGTCATGGCACGATGGA

GCTATTCTTCCCTTTGACATCGATGGGATGTAA

>A_chicken_Kazakhstan_12-20-B-Talg-45_2020_EPI1882554

ATGAATCCAAATCAGAAAATAGCGACCATTGGCTCCATCTCATTGGGACTAGTTGTATTC

AATGTTCTACTGCATGCCTTGAGCATCATATTAATGGTGTTAGCCCTGGGGAAAAGTGAA

AACAATGGAATCTGCAAGGGAACTATAGTAAGGGAATATAATGAAACAGTTAGGATAGAG

AAAGTGACCCAGTGGTACAACACTAGTGTAGTTGAATATGTACCGCATTGGAACGAGGGC

GCTTATATAAACAACACCGAATCAATATGTGATGTCAAGGGCTTTGCACCTTTTTCCAAG

GACAACGGAATAAGAATTGGCTCCAGAGGACATATTTTTGTCATAAGGGAGCCTTTCGTC

TCTTGTTCACCTGTAGAGTGCAGAACTTTCTTCCTCACTCAGGGAGCTCTACTCAATGAC

AAACACTCAAATGGAACAGTGAAGGATAGGAGCCCATTCAGAACTCTCATGAGTGTCGAA

GTGGGTCAATCACCCAATGTGTATCAAGCAAGGTTTGAAGCTGTAGCATGGTCAGCAACA

GCCTGTCATGATGGTAAGAAATGGATGACGATTGGTGTGACAGGACCAGATTCGAAAGCA

ATAGCAGTAGTCCATTACGGAGGAGTGCCTACTGATATTGTTAACTCCTGGGCAGGAGAC

ATATTACGGACTCAGGAGTCATCTTGTACTTGCATTCAAGGTAATTGTTATTGGGTAATG

ACTGACGGTCCATCCAATAGACAGGCGCAGTATAGAATATACAAAGCAAATCAAGGCAAA

ATAATTGACCAAGCAGATGTCAGCTTTAGTGGAGGGCATATTGAGGAATGCTCTTGTTAT

CCAAATGATGGTAAAGTGGAATGCGTGTGTAGAGACAACTGGATGGGAACTAACAGGCCT

GTGCTAGTTATCTCGCCTGACCTCTCTTACAGGGTTGGGTATTTATGTGCGGGATTGCCC

AGTGACACTCCAAGAGGGGAAGATGCCCAATTTGTCGGTTCGTGCACTAGTCCCATGGGA

AATCAGGGGTATGGCGTAAAAGGTTTCGGGTTTCGACAGGGAACTGATGTGTGGATGGGG

CGGACAATTAGTCGAACTTCCAGGTCAGGGTTTGAAATAATAAGGATAAAGAATGGTTGG

ACGCAGACAAGCAAAGAACAGATTAGAAGGCAAGTGGTTGTTGATAATTTGAATTGGTCA

GGATACAGTGGGTCTTTCACTTTACCAGTAGAATTGTCTGGGAGGGAATGTTTAGTCCCC

TGTTTTTGGGTCGAAATGATCAGAGGCAGGCCAGAAGAAAGAACAATCTGGACCTCTAGT

AGCTCCATTGTAATGTGTGGAGTTGATCATGAAATTGCCGATTGGTCATGGCACGATGGA

GCTATTCTTCCCTTTGACATCGATGGGATGTAA

>A_domestic_goose_Kazakhstan_1-242_2-20-B_2020_EPI1811621

ATGAATCCAAATCAGAAAATAGCGACCATTGGCTCCATCTCATTGGGACTAGTTGTATTC

AATGTTCTACTGCATGCCTTGAGCATCATATTAATGGTGTTAGCCCTGGGGAAAAGTGAA

AACAATGGAATCTGCAAGGGAACTATAGTAAGGGAATATAATGAAACAGTTAGGATAGAG

AAAGTGACCCAGTGGTACAACACTAGTGTAATCGAATATGTACCGCATTGGAACGAGGGC

GCTTATATAAACAACACCGAACCAATATGTGATGTCAAGGGCTTTGCACCTTTTTCCAAG

GACAACGGAATAAGAATTGGCTCCAGAGGACATATTTTTGTCATAAGGGAGCCTTTCGTC

TCTTGTTCACCTGTAGAGTGCAGAACTTTCTTCCTCACTCAGGGAGCTCTACTCAATGAC

AAACACTCAAATGGAACAGTGAAGGATAGGAGCCCATTCAGAACTCTCATGAGTGTCGAA

GTGGGTCAATCACCCAATGTGTATCAAGCAAGGTTTGAAGCTGTAGCATGGTCAGCAACA

GCCTGTCATGATGGTAAGAAATGGATGACGATTGGTGTGACAGGGCCAGATTCCAAAGCA

ATAGCAGTAGTCCATTACGGAGGAGTGCCTACTGATATTGTTAACTCCTGGGCAGGAGAC

ATATTACGGACTCAGGAGTCATCTTGTACTTGCATTCAAGGTAATTGTTATTGGGTAATG

ACTGACGGTCCATCCAATAGACAGGCGCAGTATAGAATATACAAAGCAAATCAAGGCAAA

ATAATTGACCAAGCAGATGTCAGCTTTAGTGGAGGGCATATTGAGGAATGCTCTTGTTAT

CCAAATGATGGTAAAGTGGAATGCGTGTGTAGAGACAACTGGATGGGAACTAACAGGCCT

GTGCTAGTTATCTCGCCTGACCTCTCTTACAGGGTTGGGTATTTATGTGCGGGATTGCCC

AGTGACACTCCAAGAGGGGAAGATGCCCGATTTGTCGGTTCGTGCACTAGTCCCATGGGA

AATCAGGGGTATGGCGTAAAAGGTTTCGGGTTTCGACAGGGAACTGATGTGTGGATGGGG

CGGACAATTAGTCGAACTTCCAGGTCAGGGTTTGAAATAATAAGGATAAAGAATGGTTGG

ACGCAGACAAGCAAAGAACAGATTAGAAGGCAAGTGGTTGTTGATAATTTGAATTGGTCG

GGATACAGTGGGTCTTTCACTTTACCAGTAGAATTGTCTGGGAGGGAATGTTTAGTCCCC

TGCTTTTGGGTCGAAATGATCAGAGGCAGGCCAGAAGAAAGAACAATCTGGACCTCTAGT

AGCTCCATTGTAATGTGTGGAGTTGATCATGAAATTGCCGATTGGTCATGGCACGATGGA

GCTATTCTTCCCTTTGACATCGATGAGATGTAA

>A_swan_Kazakhstan_1-267-20-B-Talg-52_2020_EPI1882558

ATGAATCCAAATCAGAAAATAGCGACCATTGGCTCCATCTCATTGGGACTAGTTGTATTC

AATGTTCTACTGCATGCCTTGAGCATCATATTAATGGTGTTAGCCCTGGGGAAAAGTGAA

AACAATGGAATCTGCAAGGGAACTATAGTAAGGGAATATAATGAAACAGTTAGGATAGAG

AAAGTGACCCAGTGGTACAACACTAGTGTAATCGAATATGTACCGCATTGGAACGAGGGC

GCTTATATAAACAACACCGAACCAATATGTGATGTCAAGGGCTTTGCACCTTTTTCCAAG

GACAACGGAATAAGAATTGGCTCCAGAGGACATATTTTTGTCATAAGGGAGCCTTTCGTC

TCTTGTTCACCTGTAGAGTGCAGAACTTTCTTCCTCACTCAGGGAGCTCTACTCAATGAC

AAACACTCAAATGGAACAGTGAAGGATAGGAGCCCATTCAGAACTCTCATGAGTGTCGAA

GTGGGTCAATCACCCAATGTGTATCAAGCAAGGTTTGAAGCTGTAGCATGGTCAGCAACA

GCCTGTCATGATGGTAAGAAATGGATGACGATTGGTGTGACAGGGCCAGATTCGAAAGCA

ATAGCAGTAGTCCATTACGGAGGAGTGCCTACTGATATTGTTAACTCCTGGGCAGGAGAC

ATATTACGGACTCAGGAGTCATCTTGTACTTGCATTCAAGGTAATTGTTATTGGGTAATG

ACTGACGGTCCATCCAATAGACAGGCGCAGTATAGAATATACAAAGCAAATCAAGGCAAA

ATAATTGACCAAGCAGATGTCAGCTTTAGTGGAGGGCATATTGAGGAATGCTCTTGTTAT

CCAAATGATGGTAAAGTGGAATGCGTGTGTAGAGACAACTGGATGGGAACTAACAGGCCT

GTGCTAGTTATCTCGCCTGACCTCTCTTACAGGGTTGGGTATTTATGTGCGGGATTGCCC

AGTGACACTCCAAGAGGGGAAGATGCCCGATTTGTCGGTTCGTGCACTAGTCCCATGGGA

AATCAGGGGTATGGCGTAAAAGGTTTCGGGTTTCGACAGGGAACTGATGTGTGGATGGGG

CGGACAATTAGTCGAACTTCCAGGTCAGGGTTTGAAATAATAAGGATAAAGAATGGTTGG

ACGCAGACAAGCAAAGAACAGATTAGAAGGCAAGTGGTTGTTGATAATTTGAATTGGTCG

GGATACAGTGGGTCTTTCACTTTACCAGTAGAATTGTCTGGGAGGGAATGTTTAGTCCCC

TGCTTTTGGGTCGAAATGATCAGAGGCAGGCCAGAAGAAAGAACAATCTGGACCTCTAGT

AGCTCCATTGTAATGTGTGGAGTTGATCATGAAATTGCCGATTGGTCATGGCACGATGGA

GCTATTCTTCCCTTTGACATCGATGAGATGTAA

>A_goose_Omsk_0111_2020_EPI1813144

ATGAATCCAAATCAGAAAATAGCGACCATTGGCTCCATCTCATTGGGACTAGTTGTATTC

AATGTTCTACTGCATGCCTTGAGCATCATATTAATGGTGTTAGCCCTGGGGAAAAGTGAA

AACAATGGAATCTGCAAGGGAACTATAGTAAGGGAATATAATGAAACAGTTAGGATAGAG

AAAGTGACCCAGTGGTACAACACTAGTGTAGTCGAATATGTACCGCATTGTAACGAGGGC

GCTTATATAAACAACACCGAACCAATATGTGATGTCAAGGGCTTTGCACCTTTTTCCAAG

GACAACGGAATAAGAATTGGCTCCAGAGGACATATTTTTGTCATAAGGGAGCCTTTCGTC

TCTTGTTCACCTGTAGAGTGCAGAACTTTCTTCCTCACTCAGGGAGCTCTACTCAATGAC

AAACACTCAAATGGAACAGTGAAGGATAGGAGCCCATTCAGAACTCTCATGAGTGTCGAA

GTGGGTCAATCACCCAATGTGTATCAAGCAAGGTTTGAAGCTGTAGCATGGTCAGCAACA

GCCTGTCATGATGGTAAGAAATGGATGACGATTGGTGTGACAGGGCCAGATTCGAAAGCA

ATAGCAGTAGTCCATTACGGAGGAGTGCCTACTGATATTGTTAACTCCTGGGCAGGAGAC

ATATTACGGACTCAGGAGTCATCTTGTACTTGCATTCAAGGTAATTGTTATTGGGTAATG

ACTGACGGTCCATCCAATAGACAGGCGCAGTATAGAATATACAAAGCAAATCAAGGCAAA

ATAATTGACCAAGCAGATGTCAGCTTTAGTGGAGGGCATATTGAGGAATGCTCTTGTTAT

CCAAATGATGGTAAAGTGGAATGCGTGTGTAGAGACAACTGGATGGGAACTAACAGGCCT

GTGCTAGTTATCTCGCCTGACCTCTCTTACAGGGTTGGGTATTTATGTGCGGGATTACCC

AGTGACACTCCAAGAGGGGAAGATGCCCAATTTGTCGGTTCGTGCACTAGTCCTATGGGA

AATCAGGGGTATGGCGTAAAAGGTTTCGGGTTTCGACAGGGAACTGATGTGTGGATGGGG

CGGACAATTAGTCGAACTTCCAGGTCAGGGTTTGAAATAATAAGGATAAAGAATGGTTGG

ACGCAGACAAGCAAAGAACAGATTAGAAGGCAAGTGGTTGTTGATAATTTGAATTGGTCG

GGATACAGTGGGTCTTTCACTTTACCAGTAGAATTGTCTGGGAGGGAATGTTTAGTCCCC

TGTTTTTGGGTCGAAATGATCAGAGGCAGGCCAGAAGAAAGAACAATCTGGACCTCTAGT

AGCTCCATTGTAATGTGTGGAGTTGATCATGAAATTGCCGATTGGTCATGGCACGATGGA

GCTATTCTTCCCTTTGACATCGATGGGATGTAA

>A_chicken_Tyumen_302-01_2020_EPI1848605

ATGAATCCAAATCAGAAAATAGCGACCATTGGCTCCATCTCATTGGGACTAGTTGTATTC

AATGTTCTACTGCATGCCTTGAGCATCATATTAATGGTGTTAGCCCTGGGGAAAAGTGAA

AACAATGGAATCTGCAAGGGAACTATAGTAAGGGAATATAATGAAACAGTTAGGATAGAG

AAAGTGACCCAGTGGTACAACACTAGTGTAGTCGAATATGTACCGCATTGGAACGAGGGC

GCTTATATAAACAACACCGAATCAATATGTGATGTCAAGGGCTTTGCACCTTTTTCCAAG

GACAACGGAATAAGAATTGGCTCCAGAGGACATATTTTTGTCATAAGGGAGCCTTTCGTC

TCTTGTTCACCTGTAGAGTGCAGAACTTTCTTCCTCACTCAGGGAGCTCTACTCAATGAC

AAACACTCAAATGGAACAGTGAAGGATAGGAGCCCATTCAGAACTCTCATGAGTGTCGAA

GTGGGTCAATCACCCAATGTGTATCAAGCAAGGTTTGAAGCTGTAGCATGGTCAGCAACA

GCCTGTCATGATGGTAAGAAATGGATGACGATTGGTGTGACAGGGCCAGATTCGAAAGCA

ATAGCAGTAGTCCATTACGGAGGAGTGCCTACTGATATTGTTAACTCCTGGGCAGGAGAC

ATATTACGGACTCAGGAGTCATCTTGTACTTGCATTCAAGGTAATTGTTATTGGGTAATG

ACTGACGGTCCATCCAATAGACAGGCGCAGTATAGAATATACAAAGCAAATCAAGGCAAA

ATAATTGACCAAGCAGATGTCAGCTTTAGTGGAGGGCATATTGAGGAATGCTCTTGTTAT

CCAAATGATGGTAAAGTGGAATGCGTGTGTAGAGACAACTGGATGGGAACTAACAGGCCT

GTGCTAGTTATCTCGCCTGACCTCTCTTACAGGGTTGGGTATTTATGTGCGGGATTGCCC

AGTGACACTCCAAGAGGGGAAGATGCCCAATTTGTCGGTTCGTGCACTAGTCCCATGGGA

AATCAGGGGTATGGCGTAAAAGGTTTCGGGTTTCGACAGGGAACTGATGTGTGGATGGGG

CGGACAATTAGTCGAACCTCCAGGTCAGGGTTTGAAATAATAAGGATAAAGAATGGTTGG

ACGCAGACAAGCAAAGAACAGATTAGAAGGCAGGTGGTTGTTGATAATTTGAATTGGTCG

GGATATAGTGGGTCTTTCACTTTACCAGTAGAATTGTCTGGGAGGGAATGTTTAGTCCCC

TGTTTTTGGGTCGAAATGATCAGAGGCAGGCCAGAAGAAAGAACAATCTGGACCTCTAGT

AGCTCCATTGTAATGTGTGGAGTTGATCATGAAATTGCCGATTGGTCATGGCACGATGGA

GCTATTCTTCCCTTTGACATCGATGGGATGTAA

>A_chicken_Kazakhstan_Kn-3_2020_EPI1839260

ATGAATCCAAATCAGAAAATAGCGACCATTGGCTCCATCTCATTGGGACTAGTTGTATTC

AATGTTCTACTGCATGCCTTGAGCATCATATTAATGGTGTTAGCCCTGGGGAAAAGTGAA

AACAATGGAATCTGCAAGGGAACTATAGTAAGGGAATATAATGAAACAGTTAGGATAGAG

AAAGTGACCCAGTGGTACAACACTAGTGTAGTCGAATATGTACCGCATTGGAACGAGGGC

GCTTATATAAACAACACCGAACCAATATGTGATGTCAAGGGCTTTGCACCTTTTTCCAAG

GACAACGGAATAAGAATTGGCTCCAGAGGACATATCTTTGTCATAAGGGAGCCTTTCGTC

TCTTGTTCACCTGTAGAGTGCAGAACTTTCTTCCTCACTCAGGGAGCTCTACTCAATGAC

AAACACTCAAATGGAACAGTGAAGGATAGGAGCCCATTCAGAACTCTCATGAGTGTCGAA

GTGGGTCAATCACCCAATGTGTATCAAGCAAGGTTTGAAGCTGTAGCATGGTCAGCAACA

GCCTGTCATGATGGTAAGAAATGGATGACGATTGGTGTGACAGGGCCAGATTCGAAAGCA

ATAGCAGTAGTCCATTACGGAGGAGTGCCTACTGATATTGTTAACTCCTGGGCAGGAGAC

ATATTACGGACTCAGGAGTCATCTTGTACTTGCATTCAAGGTAATTGTTATTGGGTAATG

ACTGACGGTCCATCCAATAGACAGGCGCAGTATAGAATATACAAAGCAAATCAAGGCAAA

ATAATTGACCAAGCAGATGTCAGCTTTAGTGGAGGGCATATTGAGGAATGCTCTTGTTAT

CCAAATGATGGTAAAGTGGAATGCGTGTGTAGAGACAACTGGATGGGAACTAACAGGCCT

GTGCTAGTTATCTCGCCTGACCTCTCTTACAGGGTTGGGTATTTATGTGCGGGATTGCCC

AGTGACACTCCAAGAGGGGAAGATGCCCAATTTGTCGGTTCGTGCACTAGTCCCATGGGA

AATCAGGGGTATGGCGTAAAAGGTTTCGGGTTTCGACAGGGAACTGATGTGTGGATGGGG

CGGACAATTAGTCGAACCTCCAGGTCAGGGTTTGAAATAATAAGGATAAAAAATGGTTGG

ACGCAGACAAGCAAAGAACAGATTAGAAGGCAGGTGGTTGTTGATAATTTGAATTGGTCG

GGATACAGTGGGTCTTTCACTTTACCAGTAGAATTGTCTGGGAGGGAATGTTTAGTCCCC

TGTTTTTGGGTCGAAATGATCAGAGGCAGGCCAGAAGAAAGAACAATCTGGACCTCTAGT

AGCTCCATTGTAATGTGTGGAGTTGAGCATGAAATTGCCGATTGGTCATGGCACGATGGA

GCTATTCTTCCTTTTGACATCGATGGGATGTAA

>A_chicken_Kazakhstan_Kn-6_2020_EPI1839268

ATGAATCCAAATCAGAAAATAGCGACCATTGGCTCCATCTCATTGGGACTAGTTGTATTC

AATGTTCTACTGCATGCCTTGAGCATCATATTAATGGTGTTAGCCCTGGGGAAAAGTGAA

AACAATGGAATCTGCAAGGGAACTATAGTAAGGGAATATAATGAAACAGTTAGGATAGAG

AAAGTGACCCAGTGGTACAACACTAGTGTAGTCGAATATGTACCGCATTGGAACGAGGGC

GCTTATATAAACAACACCGAACCAATATGTGATGTCAAGGGCTTTGCACCTTTTTCCAAG

GACAACGGAATAAGAATTGGCTCCAGAGGACATATCTTTGTCATAAGGGAGCCTTTCGTC

TCTTGTTCACCTGTAGAGTGCAGAACTTTCTTCCTCACTCAGGGAGCTCTACTCAATGAC

AAACACTCAAATGGAACAGTGAAGGATAGGAGCCCATTCAGAACTCTCATGAGTGTCGAA

GTGGGTCAATCACCCAATGTGTATCAAGCAAGGTTTGAAGCTGTAGCATGGTCAGCAACA

GCCTGTCATGATGGTAAGAAATGGATGACGATTGGTGTGACAGGGCCAGATTCGAAAGCA

ATAGCAGTAGTCCATTACGGAGGAGTGCCTACTGATATTGTTAACTCCTGGGCAGGAGAC

ATATTACGGACTCAGGAGTCATCTTGTACTTGCATTCAAGGTAATTGTTATTGGGTAATG

ACTGACGGTCCATCCAATAGACAGGCGCAGTATAGAATATACAAAGCAAATCAAGGCAAA

ATAATTGACCAAGCAGATGTCAGCTTTAGTGGAGGGCATATTGAGGAATGCTCTTGTTAT

CCAAATGATGGTAAAGTGGAATGCGTGTGTAGAGACAACTGGATGGGAACTAACAGGCCT

GTGCTAGTTATCTCGCCTGACCTCTCTTACAGAGTTGGGTATTTATGTGCGGGATTGCCC

AGTGACACTCCAAGAGGGGAAGATGCCCAATTTGTCGGTTCGTGCACTAGTCCCATGGGA

AATCAGGGGTATGGCGTAAAAGGTTTCGGGTTTCGACAGGGAACTGATGTGTGGATGGGG

CGGACAATTAGTCGAACCTCCAGGTCAGGGTTTGAAATAATAAGGATAAAAAATGGTTGG

ACGCAGACAAGCAAAGAACAGATTAGAAGGCAGGTGGTTGTTGATAATTTGAATTGGTCG

GGATACAGTGGGTCTTTCACTTTACCAGTAGAATTGTCTGGGAGGGAATGTTTAGTCCCC

TGTTTTTGGGTCGAAATGATCAGAGGCAGGCCAGAAGAAAGAACAATCTGGACCTCTAGT

AGCTCCATTGTAATGTGTGGAGTTGAGCATGAAATTGCCGATTGGTCATGGCACGATGGA

GCTATTCTTCCTTTTGACATCGATGGGATGTAA

>A_chicken_Omsk_0073_2020_EPI1813408

ATGAATCCAAATCAGAAAATAGCGACCATTGGCTCCATCTCATTGGGACTAGTTGTATTC

AATGTTCTACTGCATGCCTTGAGCATCATATTAATGGTGTTAGCCCTGGGGAAAAGTGAA

AACAATGGAATCTGCAAGGGAACTATAGTAAGGGAATATAATGAAACAGTTAGGATAGAG

AAAGTGACCCAGTGGTACAACACTAGTGTAGTCGAATATGTACCGCATTGGAACGAGGGC

GCTTATATAAACAACACCGAACCAATATGTGATGTCAAGGGCTTTGCACCTTTTTCCAAG

GACAACGGAATAAGAATTGGCTCCAGAGGACATATTTTTGTCATAAGGGAGCCTTTCGTC

TCTTGTTCACCTGTAGAGTGCAGAACTTTCTTCCTCACTCAGGGAGCTCTACTCAATGAC

AAACACTCAAATGGAACAGTGAAGGATAGGAGCCCATTCAGAACTCTCATGAGTGTCGAA

GTGGGTCAATCACCCAATGTGTATCAAGCAAGGTTTGAAGCTGTAGCATGGTCAGCAACA

GCCTGTCATGATGGTAAGAAATGGATGACGATTGGTGTGACAGGGCCAGATTCGAAAGCA

ATAGCAGTAGTCCATTACGGAGGAGTGCCTACTGATATTGTTAACTCCTGGGCAGGAGAC

ATATTACGGACTCAGGAGTCATCTTGTACTTGCATTCAAGGTAATTGTTATTGGGTAATG

ACTGACGGTCCATCCAATAGACAGGCGCAGTATAGAATATACAAAGCAAATCAAGGCAAA

ATAATTGACCAAGCAGATGTCAGCTTTAGTGGAGGGCATATTGAGGAATGCTCTTGTTAT

CCAAATGATGGTAAAGTGGAATGCGTGTGTAGAGACAACTGGATGGGAACTAACAGGCCT

GTGCTAGTTATCTCGCCTGACCTCTCTTACAGGGTTGGGTATTTATGTGCGGGATTGCCC

AGTGACACTCCAAGAGGGGAAGATGCCCAATTTGTCGGTTCGTGCACTAGTCCCATGGGA

AATCAGGGGTATGGCGTAAAAGGTTTCGGGTTTCGACAGGGAACTGATGTGTGGATGGGG

CGGACAATTAGTCGAACCTCCAGGTCAGGGTTTGAAATAATAAGGATAAAGAATGGTTGG

ACGCAGACAAGCAAAGAACAGATTAGAAGGCAGGTGGTTGTTGATAATTTGAATTGGTCG

GGATACAGTGGGTCTTTCACTTTACCAGTAGAATTGTCTGGGAGGGAATGTTTAGTCCCC

TGTTTTTGGGTCGAAATGATCAGAGGCAGGCCAGAAGAAAGAACAATCTGGACCTCTAGT

AGCTCCATTGTAATGTGTGGAGTTGATCATGAAGTTGCCGATTGGTCATGGCACGATGGA

GCTATTCTTCCCTTTGACATCGATGGGATGTAA

>A_duck_Kazakhstan_12-20-B-Talg-11_2020_EPI1882549

ATGAATCCAAATCAGAAAATAGCGACCATTGGCTCCATCTCATTGGGACTAGTTGTATTC

AATGTTCTACTGCATGCCTTGAGCATCATATTAATGGTGTTAGCCCTGGGGAAAAGTGAA

AACAATGGAATCTGCAAGGGAACTATAGTAAGGGAATATAATGAAACAGTTAGGATAGAG

AAAGTGACCCAGTGGTACAACACTAGTGTAGTCGAATATGTACCGCATTGGAACGAGGGC

GCTTATATAAACAACACCGAACCAATATGTGATGTCAAGGGCTTTGCACCTTTTTCCAAG

GACAACGGAATAAGAATTGGCTCCAGAGGACATATTTTTGTCATAAGGGAGCCTTTCGTC

TCTTGTTCACCTGTAGAGTGCAGAACTTTCTTCCTCACTCAGGGAGCTCTACTCAATGAC

AGACACTCAAATGGAACAGTGAAGGATAGGAGCCCATTCAGAACTCTCATGAGTGTCGAA

GTGGGTCAATCACCCAATGTGTATCAAGCAAGGTTTGAAGCTGTAGCATGGTCAGCAACA

GCCTGTCATGATGGTAAGAAATGGATGACGATTGGTGTGACAGGGCCAGATTCGAAAGCA

ATAGCAGTAGTCCATTACGGAGGAGTGCCTACTGATATTGTTAACTCCTGGGCAGGAGAC

ATATTACGGACTCAGGAGTCATCTTGTACTTGCATTCAAGGTAATTGTTATTGGGTAATG

ACTGACGGTCCATCCAATAGACAGGCACAGTATAGAATATACAAAGCAAATCAAGGCAAA

ATAATTGACCAAGCAGATGTCAGCTTTAGTGGAGGGCATATTGAGGAATGCTCTTGTTAT

CCAAATGATGGTAAAGTGGAATGCGTGTGTAGAGACAACTGGATGGGAACTAACAGGCCT

GTGCTAGTTATCTCGCCTGACCTCTCTTACAGGGTTGGGTATTTATGTGCGGGATTGCCC

AGTGACACTCCAAGAGGGGAAGATGCCCAATTTGTCGGTTCGTGCACTAGTCCCATGGGA

AATCAGGGGTATGGCGTAAAAGGTTTCGGGTTTCGACAGGGAACTGATGTGTGGATGGGG

CGGACAATTAGTCGAACCTCCAGGTCAGGGTTTGAAATAATAAGGATAAAGAATGGGTGG

ACGCAGACAAGCAAAGAACAGATTAGAAGGCAGGTGGTTGTTGATAATTTGAATTGGTCG

GGATACAGTGGGTCTTTCACTTTACCAGTAGAATTGTCTGGGAGGGAATGTTTAGTCCCC

TGTTTTTGGGTCGAAATGATCAGAGGCAGGCCAGAAGAAAGAACAATCTGGACCTCTAGT

AGCTCCATTGTAATGTGTGGAGTTGATCATGAAATTGCCGATTGGTCATGGCACGATGGA

GCTATTCTTCCCTTTGACATCGATGGGATGTAA

>A_goose_Kazakhstan_7-20-B-Talg-12_2020_EPI1882550

ATGAATCCAAATCAGAAAATAGCGACCATTGGCTCCATCTCATTGGGACTAGTTGTATTC

AATGTTCTACTGCATGCCTTGAGCATCATATTAATGGTGTTAGCCCTGGGGAAAAGTGAA

AACAATGGAATCTGCAAGGGAACTATAGTAAGGGAATATAATGAAACAGTTAGGATAGAG

AAAGTGACCCAGTGGTACAACACTAGTGTAGTCGAATATGTACCGCATTGGAACGAGGGC

GCTTATATAAACAACACCGAACCAATATGTGATGTCAAGGGCTTTGCACCTTTTTCCAAG

GACAACGGAATAAGAATTGGCTCCAGAGGACATATTTTTGTCATAAGGGAGCCTTTCGTC

TCTTGTTCACCTGTAGAGTGCAGAACTTTCTTCCTCACTCAGGGAGCTCTACTCAATGAC

AGACACTCAAATGGAACAGTGAAGGATAGGAGCCCATTCAGAACTCTCATGAGTGTCGAA

GTGGGTCAATCACCCAATGTGTATCAAGCAAGGTTTGAAGCTGTAGCATGGTCAGCAACA

GCCTGTCATGATGGTAAGAAATGGATGACGATTGGTGTGACAGGGCCAGATTCGAAAGCA

ATAGCAGTAGTCCATTACGGAGGAGTGCCTACTGATATTGTTAACTCCTGGGCAGGAGAC

ATATTACGGACTCAGGAGTCATCTTGTACTTGCATTCAAGGTAATTGTTATTGGGTAATG

ACTGACGGTCCATCCAATAGACAGGCACAGTATAGAATATACAAAGCAAATCAAGGCAAA

ATAATTGACCAAGCAGATGTCAGCTTTAGTGGAGGGCATATTGAGGAATGCTCTTGTTAT

CCAAATGATGGTAAAGTGGAATGCGTGTGTAGAGACAACTGGATGGGAACTAACAGGCCT

GTGCTAGTTATCTCGCCTGACCTCTCTTACAGGGTTGGGTATTTATGTGCGGGATTGCCC

AGTGACACTCCAAGAGGGGAAGATGCCCAATTTGTCGGTTCGTGCACTAGTCCCATGGGA

AATCAGGGGTATGGCGTAAAAGGTTTCGGGTTTCGACAGGGAACTGATGTGTGGATGGGG

CGGACAATTAGTCGAACCTCCAGGTCAGGGTTTGAAATAATAAGGATAAAGAATGGGTGG

ACGCAGACAAGCAAAGAACAGATTAGAAGGCAGGTGGTTGTTGATAATTTGAATTGGTCG

GGATACAGTGGGTCTTTCACTTTACCAGTAGAATTGTCTGGGAGGGAATGTTTAGTCCCC

TGTTTTTGGGTCGAAATGATCAGAGGCAGGCCAGAAGAAAGAACAATCTGGACCTCTAGT

AGCTCCATTGTAATGTGTGGAGTTGATCATGAAATTGCCGATTGGTCATGGCACGATGGA

GCTATTCTTCCCTTTGACATCGATGGGATGTAA

>A_crow_Kazakhstan_15-20-B-Talg-4_2020_EPI1882557

ATGAATCCAAATCAGAAAATAGCGACCATTGGCTCCATCTCATTGGGACTAGTTGTATTC

AATGTTCTACTGCATGCCTTGAGCATCATATTAATGGTGTTAGCCCTGGGGAAAAGTGAA

AACAATGGAATCTGCAAGGGAACTATAGTAAGGGAATATAATGAAACAGTTAGGATAGAG

AAAGTGACCCAGTGGTACAACACTAGTGTAGTCGAATATGTACCGCATTGGAACGAGGGC

GCTTATATAAACAACACCGAACCAATATGTGATGTCAAGGGCTTTGCACCTTTTTCCAAG

GACAACGGAATAAGAATTGGCTCCAGAGGACATATTTTTGTCATAAGGGAGCCTTTCGTC

TCTTGTTCACCTGTAGAGTGCAGAACTTTCTTCCTCACTCAGGGAGCTCTACTCAATGAC

AGACACTCAAATGGAACAGTGAAGGATAGGAGCCCATTCAGAACTCTCATGAGTGTCGAA

GTGGGTCAATCACCCAATGTGTATCAAGCAAGGTTTGAAGCTGTAGCATGGTCAGCAACA

GCCTGTCATGATGGTAAGAAATGGATGACGATTGGTGTGACAGGGCCAGATTCGAAAGCA

ATAGCAGTAGTCCATTACGGAGGAGTGCCTACTGATATTGTTAACTCCTGGGCAGGAGAC

ATATTACGGACTCAGGAGTCATCTTGTACTTGCATTCAAGGTAATTGTTATTGGGTAATG

ACTGACGGTCCATCCAATAGACAGGCACAGTATAGAATATACAAAGCAAATCAAGGCAAA

ATAATTGACCAAGCAGATGTCAGCTTTAGTGGAGGGCATATTGAGGAATGCTCTTGTTAT

CCAAATGATGGTAAAGTGGAATGCGTGTGTAGAGACAACTGGATGGGAACTAACAGGCCT

GTGCTAGTTATCTCGCCTGACCTCTCTTACAGGGTTGGGTATTTATGTGCGGGATTGCCC

AGTGACACTCCAAGAGGGGAAGATGCCCAATTTGTCGGTTCGTGCACTAGTCCCATGGGA

AATCAGGGGTATGGCGTAAAAGGTTTCGGGTTTCGACAGGGAACTGATGTGTGGATGGGG

CGGACAATTAGTCGAACCTCCAGGTCAGGGTTTGAAATAATAAGGATAAAGAATGGGTGG

ACGCAGACAAGCAAAGAACAGATTAGAAGGCAGGTGGTTGTTGATAATTTGAATTGGTCG

GGATACAGTGGGTCTTTCACTTTACCAGTAGAATTGTCTGGGAGGGAATGTTTAGTCCCC

TGTTTTTGGGTCGAAATGATCAGAGGCAGGCCAGAAGAAAGAACAATCTGGACCTCTAGT

AGCTCCATTGTAATGTGTGGAGTTGATCATGAAATTGCCGATTGGTCATGGCACGATGGA

GCTATTCTTCCCTTTGACATCGATGGGATGTAA

>A_pigeon_Kazakhstan_15-20-B-Talg-5_2020_EPI1882561

ATGAATCCAAATCAGAAAATAGCGACCATTGGCTCCATCTCATTGGGACTAGTTGTATTC

AATGTTCTACTGCATGCCTTGAGCATCATATTAATGGTGTTAGCCCTGGGGAAAAGTGAA

AACAATGGAATCTGCAAGGGAACTATAGTAAGGGAATATAATGAAACAGTTAGGATAGAG

AAAGTGACCCAGTGGTACAACACTAGTGTAGTCGAATATGTACCGCATTGGAACGAGGGC

GCTTATATAAACAACACCGAACCAATATGTGATGTCAAGGGCTTTGCACCTTTTTCCAAG

GACAACGGAATAAGAATTGGCTCCAGAGGACATATTTTTGTCATAAGGGAGCCTTTCGTC

TCTTGTTCACCTGTAGAGTGCAGAACTTTCTTCCTCACTCAGGGAGCTCTACTCAATGAC

AGACACTCAAATGGAACAGTGAAGGATAGGAGCCCATTCAGAACTCTCATGAGTGTCGAA

GTGGGTCAATCACCCAATGTGTATCAAGCAAGGTTTGAAGCTGTAGCATGGTCAGCAACA

GCCTGTCATGATGGTAAGAAATGGATGACGATTGGTGTGACAGGGCCAGATTCGAAAGCA

ATAGCAGTAGTCCATTACGGAGGAGTGCCTACTGATATTGTTAACTCCTGGGCAGGAGAC

ATATTACGGACTCAGGAGTCATCTTGTACTTGCATTCAAGGTAATTGTTATTGGGTAATG

ACTGACGGTCCATCCAATAGACAGGCACAGTATAGAATATACAAAGCAAATCAAGGCAAA

ATAATTGACCAAGCAGATGTCAGCTTTAGTGGAGGGCATATTGAGGAATGCTCTTGTTAT

CCAAATGATGGTAAAGTGGAATGCGTGTGTAGAGACAACTGGATGGGAACTAACAGGCCT

GTGCTAGTTATCTCGCCTGACCTCTCTTACAGGGTTGGGTATTTATGTGCGGGATTGCCC

AGTGACACTCCAAGAGGGGAAGATGCCCAATTTGTCGGTTCGTGCACTAGTCCCATGGGA

AATCAGGGGTATGGCGTAAAAGGTTTCGGGTTTCGACAGGGAACTGATGTGTGGATGGGG

CGGACAATTAGTCGAACCTCCAGGTCAGGGTTTGAAATAATAAGGATAAAGAATGGGTGG

ACGCAGACAAGCAAAGAACAGATTAGAAGGCAGGTGGTTGTTGATAATTTGAATTGGTCG

GGATACAGTGGGTCTTTCACTTTACCAGTAGAATTGTCTGGGAGGGAATGTTTAGTCCCC

TGTTTTTGGGTCGAAATGATCAGAGGCAGGCCAGAAGAAAGAACAATCTGGACCTCTAGT

AGCTCCATTGTAATGTGTGGAGTTGATCATGAAATTGCCGATTGGTCATGGCACGATGGA

GCTATTCTTCCCTTTGACATCGATGGGATGTAA

>A_chicken_Kazakhstan_1-20-B-Talg-67_2020_EPI1882562

ATGAATCCAAATCAAAAAATAGCGACCATTGGCTCCATCTCATTGGGACTAGTTGTATTC

AATGTTCTACTGCATGCCTTGAGCATCATATTAATGGTGTTAGCCCTGGGGAAAAGTGAA

AACAATGGAATCTGCAAGGGAACTATAGTAAGGGAATATAATGAAACAGTTAGGATAGAG

AAAGTGACCCAGTGGTACAACACTAGTGTAGTCGAATATGTACCGCATTGGAACGAGGGC

GCTTATATAAACAACACCGAACCAATATGTGATGTCAAGGGCTTTGCACCTTTTTCCAAG

GACAACGGAATAAGAATTGGCTCCAGAGGACATATTTTTGTCATAAGGGAGCCTTTCGTC

TCTTGTTCACCTGTAGAGTGCAGAACTTTCTTCCTCACTCAGGGAGCTCTACTCAATGAC

AAACACTCAAATGGAACAGTGAAGGATAGGAGCCCATTCAGAACTCTCATGAGTGTCGAA

GTGGGTCAATCACCCAATGTGTATCAAGCAAGGTTTGAAGCTGTAGCATGGTCAGCAACA

GCCTGTCATGATGGTAAGAAATGGATGACGATTGGTGTGACAGGGCCAGATTCGAAAGCA

ATAGCAGTAGTCCATTACGGAGGAGTGCCTACTGATATTGTTAACTCCTGGGCAGGAGAC

ATATTACGGACTCAGGAGTCATCTTGTACTTGCATTCAAGGTAATTGTTATTGGGTAATG

ACTGACGGTCCATCCAATAGACAGGCGCAGTATAGAATATACAAAGCAAATCAAGGCAAA

ATAATTGACCAAGCAGATGTCAGCTTTAGTGGAGGGCATATTGAGGAATGCTCTTGTTAT

CCAAATGATGGTAAAGTGGAATGCGTGTGTAGAGACAACTGGATGGGAACTAACAGGCCT

GTGCTAGTTATCTCGCCTGACCTCTCTTACAGGGTTGGGTATTTATGTGCGGGATTGCCC

AGTGACACTCCAAGAGGGGAAGATGCCCAATTTGTCGGTTCGTGCACTAGTCCCATGGGA

AATCAGGGGTATGGCGTAAAAGGTTTCGGGTTTCGACAGGGAACTGATGTGTGGATGGGG

CGGACAATTAGTCGAACCTCCAGGTCAGGGTTTGAAATAATAAGGATAAAGAATGGGTGG

ACGCAGACAAGCAAAGAACAGATTAGAAGGCAGGTGGTTGTTGATAATTTGAATTGGTCG

GGATACAGTGGGTCTTTCACTTTACCAGTAGAATTGTCTGGGAGGGAATGTTTAGTCCCC

TGTTTTTGGGTCGAAATGATCAGAGGCAGGCCAGAAGAAAGAACAATCTGGACTTCTAGT

AGCTCCATTGTAATGTGTGGAGTTGATCATGAAATTGCCGATTGGTCATGGCACGATGGA

GCTATTCTTCCCTTTGACATCGATGGGATGTAA

>A_duck_Omsk_0004_2020_EPI1813336

ATGAATCCAAATCAGAAAATAGCGACCATTGGCTCCATCTCATTGGGACTAGTTGTATTC

AATGTTCTACTGCATGCCTTGAGCATCATATTAATGGTGTTAGCCCTGGGGAAAAGCGAA

AACAATGGAATCTGCAAGGGAACTATAGTAAGGGAATATAATGAAACAGTTAGGATAGAG

AAAGTGACCCAGTGGTACAACACTAGTGTAGTCGAATATGTACCGCATTGGAACGAGGGC

GCTTATATAAACAACACCGAACCAATATGTGATGTCAAGGGCTTTGCACCTTTTTCCAAG

GACAACGGAATAAGAATTGGCTCCAGAGGACATATTTTTGTCATAAGGGAGCCTTTCGTC

TCTTGTTCACCTGTAGAGTGCAGAACTTTCTTCCTCACTCAGGGAGCTCTACTCAATGAC

AAACACTCAAATGGAACAGTGAAGGATAGGAGCCCATTCAGAACTCTCATGAGTGTCGAA

GTGGGTCAATCACCCAATGTGTATCAAGCAAGGTTTGAAGCTGTAGCATGGTCAGCAACA

GCCTGTCATGATGGTAAGAAATGGATGACGATTGGTGTGACAGGGCCAGATTCGAAAGCA

ATAGCAGTAGTCCATTACGGAGGAGTGCCTACTGATATTGTTAACTCCTGGGCAGGAGAC

ATATTACGGACTCAGGAGTCATCTTGTACTTGCATTCAAGGTAATTGTTATTGGGTAATG

ACTGACGGTCCATCCAATAGACAGGCGCAGTATAGAATATACAAAGCAAATCAAGGCAAA

ATAATTGACCAAGCAGATGTCAGCTTTAGTGGAGGGCATATTGAGGAATGCTCTTGTTAT

CCAAATGATGGTAAAGTGGAATGCGTGTGTAGAGACAACTGGATGGGAACTAACAGGCCT

GTGCTAGTTATCTCGCCTGACCTCTCTTACAGGGTTGGGTATTTATGTGCGGGATTGCCC

AGTGACACTCCAAGAGGGGAAGATGCCCAATTTGTCGGTTCGTGCACTAGTCCCATGGGA

AATCAGGGGTATGGCGTAAAAGGTTTCGGGTTTCGACAGGGAACTGATGTGTGGATGGGG

CGGACAATTAGTCGAACCTCCAGGTCAGGGTTTGAAATAATAAGGATAAAGAATGGTTGG

ACGCAGACAAGCAAAGAACAGATTAGAAGGCAGGTGATTGTTGATAATTTGAATTGGTCG

GGATACAGTGGGTCTTTCACCTTACCAGTAGAATTGTCTGGGAGGGAATGTTTAGTCCCC

TGTTTTTGGGTCGAAATGATCAGAGGCAGGCCAGAAGAAAGAACAATCTGGACCTCTAGT

AGCTCCATTGTAATGTGTGGAGTTGATCATGAAATTGCCGATTGGTCATGGCACGATGGA

GCTATTCTTCCCTTTGACATCGATGGGATGTAA

>A_chicken_Poland_474_2020_EPI1842054

ATGAATCCAAATCAGAAAATAGCGACCATTGGCTCCATCTCATTGGGACTAGTTGTATTC

AATGTTCTACTGCATGCCTTGAGCATAATATTAATGGTGTTAGCCCTGGGGAAAAGTGAA

AACAATGGAATCTGCAAGGGAACTATAGTAAGGGAATATAATGAAACAGTTAGGATAGAG

AAAGTGACCCAGTGGTACAACACTAGTGTAGTCGAATATGTACCGCATTGGAACGAGGGC

GCTTATATAAACAACACCGAACCAATATGTGATGTCAAGGGCTTTGCACCTTTTTCCAAG

GACAACGGAATAAGAATTGGCTCCAGAGGACATATTTTTGTCATAAGGGAGCCTTTCGTC

TCTTGTTCACCTGTAGAGTGCAGAACTTTCTTCCTCACTCAGGGAGCTCTACTCAATGAC

AAACACTCAAATGGAACAGTGAAGGATAGGAGCCCATTCAGAACTCTCATGAGTGTCGAA

GTGGGTCAATCACCCAATGTGTATCAAGCAAGGTTTGAAGCTGTAGCATGGTCAGCAACA

GCCTGTCATGATGGTAAGAAATGGATGACAATTGGTGTGACAGGGCCAGATTCGAAAGCA

ATAGCAGTAGTCCATTACGGAGGAGTGCCTACTGATATTGTTAACTCCTGGGCAGGAGAC

ATATTACGGACTCAGGAGTCATCTTGTACTTGCATTCGAGGTAATTGTTATTGGGTAATG

ACTGACGGTCCATCCAATAGACAGGCGCAGTATAGAATATACAAAGCAAATCAAGGCAAA

ATAATTGACCAAGCAGATGTCAGCTTTAGTGGAGGGCATATTGAGGAATGCTCTTGTTAT

CCAAATGATGGTAAAGTGGAATGCGTGTGTAGAGACAACTGGATGGGAACTAACAGGCCT

GTGCTAGTTATCTCGCCTGACCTCTCTTACAGGGTTGGGTATTTATGTGCGGGATTGCCC

AGTGACACTCCAAGAGGGGAAGATGCCCAATTTGTCGGTTCGTGCACTAGYCCCATGGGA

AATCAGGGGTATGGCGTAAAAGGTTTCGGGTTTCGACAGGGAACTGATGTGTGGATGGGG

CGGACAATTAGTCGAACCTCCAGGTCAGGGTTTGAAATAATAAGGATAAAGAATGGTTGG

ACGCAGACAAGCAAAGAACAGATTAGAAGGCAGGTGGTTGTTGATAATTTGAATTGGTCG

GGATACAGTGGGTCTTTCACTTTACCAGTAGAATTGTCTGGGAGGGAATGTTTAGTCCCC

TGTTTTTGGGTCGAAATGATCAGAGGCAGGCCAGAAGAAAGAACAATCTGGACCTCTAGT

AGCTCCATTGTAATGTGTGGAGTTGATCATGAAATTGCCGATTGGTCATGGCACGATGGA

GCTATTCTTCCCTTTGACATCGATGGGATGTAA

>A_swan_Poland_MB141_2020_EPI1850213

ATGAATCCAAATCAGAAAATAGCGACCATTGGCTCCATCTCATTGGGACTAGTTGTATTC

AATGTTCTACTGCATGCCTTGAGCATAATATTAATGGTGTTAGCCCTGGGGAAAAGTGAA

AACAATGGAATCTGCAAGGGAACTATAGTAAGGGAATATAATGAAACAGTTAGGATAGAG

AAAGTGACCCAGTGGTACAACACTAGTGTAGTCGAATATGTACCGCATTGGAACGAGGGC

GCTTATATAAACAACACCGAACCAATATGTGATGTCAAGGGCTTTGCACCTTTTTCCAAG

GACAACGGAATAAGAATTGGCTCCAGAGGACATATTTTTGTCATAAGGGAGCCTTTCGTC

TCTTGTTCACCTGTAGAGTGCAGAACTTTCTTCCTCACTCAGGGAGCTCTACTCAATGAC

AAACACTCAAATGGAACAGTGAAGGATAGGAGCCCATTCAGAACTCTCATGAGTGTCGAA

GTGGGTCAATCACCCAATGTGTATCAAGCAAGGTTTGAAGCTGTAGCATGGTCAGCAACA

GCCTGTCATGATGGTAAGAAATGGATGACGATTGGTGTGACAGGGCCAGATTCGAAAGCA

ATAGCAGTAGTCCATTACGGAGGAGTGCCTACTGATATTGTTAACTCCTGGGCAGGAGAC

ATATTACGGACTCAGGAGTCATCTTGTACTTGCATTCGAGGTAATTGTTATTGGGTAATG

ACTGACGGTCCATCCAATAGACAGGCGCAGTATAGAATATACAAAGCAAATCAAGGCAAA

ATAATTGACCAAGCAGATGTCAGCTTTAGTGGAGGGCATATTGAGGAATGCTCTTGTTAT

CCAAATGATGGTAAAGTGGAATGCGTGTGTAGAGACAACTGGATGGGAACTAACAGGCCT

GTGCTAGTTATCTCGCCTGACCTCTCTTACAGGGTTGGGTATTTATGTGCGGGATTGCCC

AGTGACACTCCAAGAGGGGAAGATGCCCAATTTGTCGGTTCGTGCACTAGTCCCATGGGA

AATCAGGGGTATGGCGTAAAAGGTTTCGGGTTTCGACAGGGAACTGATGTGTGGATGGGG

CGGACAATTAGTCGAACCTCCAGGTCAGGGTTTGAAATAATAAGGATAAAGAATGGTTGG

ACGCAGACAAGCAAAGAACAGATTAGAAGGCAGGTGGTTGTTGATAATTTGAATTGGTCG

GGATACAGTGGGTCTTTCACTTTACCAGTAGAATTGTCTGGGAGGGAATGTTTAGTCCCC

TGTTTTTGGGTCGAAATGATCAGAGGCAAGCCAGAAGAAAGAACAATCTGGACCTCTAGT

AGCTCCATTGTAATGTGTGGAGTTGATCATGAAATTGCCGATTGGTCATGGCACGATGGA

GCTATTCTTCCCTTTGACATCGATGGGATGTAA

>A_goose_Kazakhstan_4-190-20-B-H5N8-1_2020_EPI1882526

ATGAATCCAAATCAGAAAATAGCGACCATTGGCTCCATCTCATTGGGACTAGTTGTATTC

AATGTTCTACTGCATGCCTTGAGCATAATATTAATGGTGTTAGCCCTGGGGAAAAGTGAA

AACAATGGAATCTGCAAGGGAACTATAGTAAGGGAATATAATGAAACAGTTAGGATAGAG

AAAGTGACCCAGTGGTACAACACTAGTGTAGTCGAATATGTACCGCATTGGAACGAGGGC

GCTTATATAAGCAACACCGAACCAATATGTGATGTCAAGGGCTTTGCACCTTTTTCCAAG

GACAACGGAATAAGAATTGGCTCCAGAGGACATATTTTTGTCATAAGGGAGCCTTTCGTC

TCTTGTTCACCTGTAGAATGCAGAACTTTCTTCCTCACTCAGGGAGCTCTACTCAATGAC

AAACACTCAAATGGAACAGTGAAGGATAGGAGCCCATTCAGAACTCTCATGAGTGTCGAA

GTGGGTCAATCACCCAATGTGTATCAAGCAAGGTTTGAAGCTGTAGCATGGTCAGCAACA

GCCTGTCATGATGGTAAGAAATGGATGACGATTGGTGTGACAGGGCCAGATTCGAAAGCA

ATAGCAGTAGTCCATTACGGAGGAGTGCCTACTGATATTGTTAACTCCTGGGCAGGAGAC

ATATTACGGACTCAGGAGTCATCTTGTACTTGCATTCAAGGTAATTGTTATTGGGTAATG

ACTGACGGTCCATCCAATAGACAGGCGCAGTATAGAATATACAAAGCAAATCAAGGCAAA

ATAATTGACCAAGCAGATGTCAGCTTTAGTGGAGGGCATATTGAGGAATGCTCTTGTTAT

CCAAATGATGGTAAAGTGGAATGCGTGTGTAGAGACAACTGGATGGGAACTAACAGGCCT

GTGCTAGTTATCTCGCCTGACCTCTCTTACAGGGTTGGGTATTTATGTGCGGGATTGCCC

AGTGACACTCCAAGAGGGGAAGATGCCCAATTTGTCGGTTCGTGCACTAGTCCCATGGGA

AATCAGGGGTATGGCGTAAAAGGTTTCGGGTTTCGACAGGGAACTGATGTGTGGATGGGG

CGGACAATTAGTCGAACCTCCAGGTCAGGGTTTGAAATAATAAGGATAAAGAATGGTTGG

ACGCAGACAAGCAAAGAACAGATTAGAAGGCAGGTGGTTGTTGATAATTTGAATTGGTCG

GGATACAGTGGGTCTTTCACTTTACCAGTAGAATTGTCTGGGAGGGAATGTTTAGTCCCC

TGTTTTTGGGTCGAAATGATCAGAGGCAGGCCAGAAGAAAGAACAATCTGGACCTCTAGT

AGCTCCATTGTAATGTGTGGAGTTGATCATGAAATTGCCGATTGGTCATGGCACGATGGA

GCTATTCTTCCCTTTGACATCGATGGGATGTAA

>A_chicken_Kazakhstan_220-B-2-H5N8-4_2020_EPI1882546

ATGAATCCAAATCAGAAAATAGCGACCATTGGCTCCATCTCATTGGGACTAGTTGTATTC

AATGTTCTACTGCATGCCTTGAGCATAATATTAATGGTGTTAGCCCTGGGGAAAAGTGAA

AACAATGGAATCTGCAAGGGAACTATAGTAAGGGAATATAATGAAACAGTTAGGATAGAG

AAAGTGACCCAGTGGTACAACACTAGTGTAGTCGAATATGTACCGCATTGGAACGAGGGC

GCTTATATAAGCAACACCGAACCAATATGTGATGTCAAGGGCTTTGCACCTTTTTCCAAG

GACAACGGAATAAGAATTGGCTCCAGAGGACATATTTTTGTCATAAGGGAGCCTTTCGTC

TCTTGTTCACCTGTAGAATGCAGAACTTTCTTCCTCACTCAGGGAGCTCTACTCAATGAC

AAACACTCAAATGGAACAGTGAAGGATAGGAGCCCATTCAGAACTCTCATGAGTGTCGAA

GTGGGTCAATCACCCAATGTGTATCAAGCAAGGTTTGAAGCTGTAGCATGGTCAGCAACA

GCCTGTCATGATGGTAAGAAATGGATGACGATTGGTGTGACAGGGCCAGATTCGAAAGCA

ATAGCAGTAGTCCATTACGGAGGAGTGCCTACTGATATTGTTAACTCCTGGGCAGGAGAC

ATATTACGGACTCAGGAGTCATCTTGTACTTGCATTCAAGGTAATTGTTATTGGGTAATG

ACTGACGGTCCATCCAATAGACAGGCGCAGTATAGAATATACAAAGCAAATCAAGGCAAA

ATAATTGACCAAGCAGATGTCAGCTTTAGTGGAGGGCATATTGAGGAATGCTCTTGTTAT

CCAAATGATGGTAAAGTGGAATGCGTGTGTAGAGACAACTGGATGGGAACTAACAGGCCT

GTGCTAGTTATCTCGCCTGACCTCTCTTACAGGGTTGGGTATTTATGTGCGGGATTGCCC

AGTGACACTCCAAGAGGGGAAGATGCCCAATTTGTCGGTTCGTGCACTAGTCCCATGGGA

AATCAGGGGTATGGCGTAAAAGGTTTCGGGTTTCGACAGGGAACTGATGTGTGGATGGGG

CGGACAATTAGTCGAACCTCCAGGTCAGGGTTTGAAATAATAAGGATAAAGAATGGTTGG

ACGCAGACAAGCAAAGAACAGATTAGAAGGCAGGTGGTTGTTGATAATTTGAATTGGTCG

GGATACAGTGGGTCTTTCACTTTACCAGTAGAATTGTCTGGGAGGGAATGTTTAGTCCCC

TGTTTTTGGGTCGAAATGATCAGAGGCAGGCCAGAAGAAAGAACAATCTGGACCTCTAGT

AGCTCCATTGTAATGTGTGGAGTTGATCATGAAATTGCCGATTGGTCATGGCACGATGGA

GCTATTCTTCCCTTTGACATCGATGGGATGTAA

>A_chicken_Rostov-on-Don_308-02_2020_EPI1848669

ATGAATCCAAATCAGAAAATAGCGACCATTGGCTCCATCTCATTGGGACTAGTTGTATTC

AATGTTCTACTGCATGCCTTGAGCATCATATTAATGGTGTTAGCCCTGGGGAAAAGTGAA

AACAATGGAATCTGCAAGGGAACTATAGTAAGGGAATATAATGAAACAGTTAGGATAGAG

AAAGTGACCCAGTGGTACAACACTAGTGTAGTCGAATATGTACCGCATTGGAACGAGGGC

GCTTATATAAACAACACCGAACCAATATGTGATGTCAAGGGCTTTGCACCTTTTTCCAAG

GACAACGGAATAAGAATTGGCTCCAGAGGACATATTTTTGTCATAAGGGAGCCTTTTGTC

TCTTGTTCACCTGTAGAGTGCAGAACTTTCTTCCTCACTCAGGGAGCTCTACTCAATGAC

AAACACTCAAATGGAACAGTGAAGGATAGGAGCCCATTCAGAACTCTCATGAGTGTCGAA

GTGGGTCAATCACCCAATGTGTATCAAGCAAGGTTTGAAGCTGTAGCATGGTCAGCAACA

GCCTGTCATGATGGTAAGAAATGGATGACGATTGGTGTGACAGGGCCAGATTCGAAAGCA

ATAGCAGTAGTCCATTACGGAGGAGTGCCTACTGATATTGTTAACTCCTGGGCAGGAGAC

ATATTACGGACTCAGGAGTCATCTTGTACTTGCATTCAAGGTAATTGTTATTGGGTAATG

ACTGACGGTCCATCCAATAGACAGGCGCAGTATAGAATATACAAAGCAAATCAAGGCAAA

ATAATTGACCAAGCAGATGTCAGCTTTAGTGGAGGGCATATTGAGGAATGCTCTTGTTAT

CCAAATGATGGTAAAGTGGAATGCGTGTGTAGAGACAACTGGATGGGAACTAACAGGCCT

GTGCTAGTTATCTCGCCTGACCTCTCTTACAGGGTTGGGTATCTATGTGCGGGATTGCCC

AGTGACACTCCAAGAGGGGAAGATGCCCAATTTGTCGGTTCGTGCACTAGTCCCATGGGA

AATCAGGGGTATGGCGTAAAAGGTTTCGGGTTTCGACAGGGAACTGATGTGTGGATGGGG

CGGACAATTAGTCGAACCTCCAGGTCAGGGTTTGAAATAATAAGGATAAAGAATGGTTGG

ACGCAGACAAGCAAAGAACAGATTAGAAGGCAGGTGGTTGTTGATAATTTGAATTGGTCG

GGATACAGTGGGTCTTTCACTTTACCAGTAGAATTGTCTGGGAGGGAATGTTTAGTCCCC

TGTTTTTGGGTCGAAATGATCAGAGGCAGGCCAGAAGAAAGAACAATCTGGACCTCTAGT

AGCTCCATTGTAATGTGTGGAGTTGATCATGAAATTGCCGATTGGTCATGGCACGATGGA

GCTATTCTTCCCTTTGACATCGATGGGATGTAA

>A_swan_Kazakhstan_9-20-B-Talg-39_2020_EPI1882553

ATGAATCCAAATCAGAAAATAGCGACCATTGGCTCCATCTCATTGGGACTAGTTGTATTC

AATGTTCTACTGCATGCCTTGAGCATCATATTAATGGTGTTAGCCCTGGGGAAAAGTGAA

AACAATGGAATCTGCAAGGGAACTATAGTAAGGGAATATAATGAAACAGTTAGGATAGAG

AAAGTGACCCAGTGGTACAACACTAGTGTAGTCAAATATGTACCGCATTGGAACGAGGGC

GCTTATATAAACAACACCGAACCAATATGTGATGTCAAGGGCTTTGCACCTTTTTCCAAG

GACAACGGAATAAGGATTGGCTCCAGAGGACATATTTTTGTCATAAGGGAGCCTTTCGTC

TCTTGTTCACCTGTAGAGTGCAGAACTTTCTTCCTCACTCAGGGAGCTCTACTCAATGAC

AAACACTCAAATGGAACAGTGAAGGATAGGAGCCCATTCAGAACTCTCATGAGTGTCGAA

GTGGGTCAATCACCCAATGTGTATCAAGCAAGGTTTGAAGCTGTAGCATGGTCAGCAACA

GCCTGTCATGATGGTAAGAAATGGATGACGATTGGTGTGACAGGGCCAGATTCGAAAGCA

ATAGCAGTAGTCCATTACGGAGGAGTGCCTACTGATATTGTTAACTCCTGGGCAGGAGAC

ATATTACGGACTCAGGAGTCATCTTGTACTTGCATTCAAGGTAATTGTTATTGGGTAATG

ACTGACGGTCCATCCAATAGACAGGCGCAGTATAGAATATACAAAGCAAATCAAGGCAAA

ATAATTGACCAAGCAGATGTCAGCTTTAGTGGAGGGCATATTGAGGAATGCTCTTGTTAT

CCAAATGATGGTAAAGTGGAATGCGTGTGTAGAGACAACTGGATGGGAACTAACAGGCCT

GTGCTAGTTATCTCGCCTGACCTCTCTTACAGGGTTGGGTATTTATGTGCGGGATTGCCC

AGTGACACTCCAAGAGGGGAAGATGCCCAATTTGTCGGTTCGTGCACTAGTCCCATGGGA

AATCAGGGGTATGGCGTAAAAGGTTTCGGGTTTCGACAGGGAACTGATGTGTGGATGGGG

CGGACAATTAGTCGAACCTCCAGGTCAGGGTTTGAAATAATAAGGATAAAGAATGGTTGG

ACGCAGACAAGCAAAGAACAGATTAGAAGGCAGGTGGTTGTTGATAATTTGAATTGGTCG

GGATACAGTGGGTCTTTCACTTTACCAGTAGAATTGTCTGGGAGGGAATGTTTAGTCCCC

TGTTTTTGGGTCGAAATGATCAGAGGCAGGCCAGAAGAAAGAACAATCTGGACCTCTAGT

AGCTCCATTGTAATGTGTGGAGTTGATCATGAAATTGCCGATTGGTCATGGCACGATGGA

GCTATTCTTCCCTTTGACATCGATGGGATGTAA
