# Supplementary material for: Highly pathogenic avian influenza virus of the A/H5N8 subtype, clade 2.3.4.4b, caused outbreaks in Kazakhstan in 2020
Source: PeerJ. 2022 Mar 2;10:e13038. doi: 10.7717/peerj.13038 (PMC8898005; doi:10.7717/peerj.13038)
Supplement: Figure S3 [file peerj-10-13038-s004.docx]

**Fig.S3** Alignment of the nucleotide sequences for the PB1 segment used in this study

>A_goose_Kazakhstan_4-190-20-B-H5N8-1_2020_EPI1927649

AGCAAAAGCAGGCAAACTATTTGAATGGATGTCAATCCGACTTTACTTTTCTTAAAAGTG

CCAGCGCAAAATGCTATAAGTACTACATTCCCTTACACTGGAGATCCCCCATACAGCCAT

GGAACAGGAACAGGGTATACCATGGACACAGTAAACAGAACACATCAATACTCAGAAAAG

GGAAAGTGGACAACAAACACAGAAACCGGAGCACCCCAACTCAACCCAATTGATGGACCA

TTACCAGAGGACAATGAGCCAAGCGGATATGCACAAACTGATTGCGTGTTGGAAGCAATG

GCTTTCCTTGAAGAATCCCACCCAGGGATATTTGAAAACTCTTGTCTTGAAGCGATGGAA

ATCGTTCAGCAAACAAGAGTAGACAAACTAACCCAAGGTCGCCAGACTTATGACTGGACA

CTGAACAGAAACCAACCAGCTGCAACCTCTTTGGCCAACACTATAGAGGTGTTCAGATCG

AATGGTCTGACAGCCAATGAATCAGGGAGACTGATAGATTTTCTCAGGGATGTGATGGAA

TCAATGGATAAAGAAGAGATGGAAGTAACAACACATTTCCAGAGAAAAAGAAGAGTGAGG

GACAACATGACTAAAAAGATGGTCACACAAAGAACAATAGGGAAGAAGAAGCAGAGGCTG

AACAAGAGGAGTTACTTAATAAGAGCACTGACATTGAATACAATGACCAAAGATGCGGAA

AGAGGCAAGTTGAAGAGACGGGCAATTGCAACACCCGGGATGCAGATTAGAGGATTCGTG

TACTTCGTTGAAACACTAGCGAGGAGCATCTGTGAGAAACTAGAGCAATCTGGGCTCCCT

GTTGGAGGGAATGAGAAGAAGGCTAAATTGGCAAATGTTGTGAGAAAAATGATGACTAAC

TCACAAGATACAGAGCTCTCCTTTACAATTACTGGAGACAACACCAAATGGAATGAGAAT

CAAAACCCTCGGGTGTTTTTGGCAATGATAACATATATCACAAGAAACCAACCTGAATGG

TTTAGAAATGTCTTAAGCATTGCCCCTATAATGTTCTCAAACAAAATGGCGAGATTAGGG

AAAGGATACATGTTTGAAAGTAAGAGCATGAAGCTAAGAACACAAATACCAGCAGAGATG

CTTACAAATATTGATCTGAAGTATTTCAACGAACCAACGAGAAAGAAAATCGAGAAAATA

AGACCTCTGCTGATTGATGGCACGGCCTCATTGAGTCCTGGGATGATGATGGGCATGTTC

AATATGCTGAGCACAGTATTAGGGGTCTCAATCCTGAATCTCGGGCAAAAGAGGTACACC

AAAACCACATACTGGTGGGATGGACTTCAATCCTCTGATGATTTCGCTCTCATAGTGAAT

GCACCGAATCATGAGGGGATACAAGCAGGAGTGGATAGATTCTATAGGACCTGCAAACTG

GTTGGGATCAACATGAGCAAAAAGAAGTCTTACATAAACCGAACAGGAACATTTGAGTTC

ACAAGTTTTTTCTACCGCTATGGATTTGTAGCCAACTTCAGTATGGAATTACCCAGCTTT

GGAGTGTCTGGAATCAATGAATCAGCTGACATGAGCATTGGAGTTACAGTGATAAAAAAC

AATATGATAAACAATGATCTTGGACCAGCAACAGCTCAAATGGCTCTTCAGTTATTCATC

AAAGACTATAGGTACACATACCGATGCCACAGGGGTGATACACAAATTCAAACGAGGAGA

TCATTCGAGCTGAAGAAGCTGTGGGAGCAGACCCGTTCAAAGGCAGGGCTGTTGATATCA

GACGGGGGGCCAAACCTATACAACATTCGGAATCTCCACATCCCAGAGGTCTGCTTGAAG

TGGGAGCTGATGGATGAAGACTACCAAGGCAGGCTGTGCAATCCTCTGAATCCATTTGTC

AGTCATAAAGAGATTGAGTCCGTAAACAATGCTGTAGTAATGCCCGCCCATGGCCCGGCC

AAGAGCATGGAATATGATGCTGTTGCGACCACACACTCGTGGATTCCTAAGAGGAACCGT

TCCATTCTCAATACCAGCCAAAGGGGAATTCTTGAGGATGAGCAGATGTACCAAAAGTGC

TGTAGTCTATTCGAGAAATTCTTCCCCAGCAGTTCATACAGGAGGCCAGTTGGAATTTCC

AGCATGGTGGAGGCCATGGTGTCTAGGGCCCGAATTGATGCACGCATCGACTTCGAATCT

GGAAGGATTAAGAAAGAAGAGTTTGCTGAGATCATGAAGATCTGTTCCACCATTGAAGAG

CTCAGACGGCAAAAATAGTGAATTTAGCTTGTCCTTCATGAAAAAATGCCTTGTTTCTAC

T

>A_chicken_Kazakhstan_220-B-2-H5N8-4_2020_EPI1927655

AGCAAAAGCAGGCAAACTATTTGAATGGATGTCAATCCGACTTTACTTTTCTTAAAAGTG

CCAGCGCAAAATGCTATAAGTACTACATTCCCTTACACTGGAGATCCCCCATACAGCCAT

GGAACAGGAACAGGGTATACCATGGACACAGTAAACAGAACACATCAATACTCAGAAAAG

GGAAAGTGGACAACAAACACAGAAACCGGAGCACCCCAACTCAACCCAATTGATGGACCA

TTACCAGAGGACAATGAGCCAAGCGGATATGCACAAACTGATTGCGTGTTGGAAGCAATG

GCTTTCCTTGAAGAATCCCACCCAGGGATATTTGAAAACTCTTGTCTTGAAGCGATGGAA

ATCGTTCAGCAAACAAGAGTAGACAAACTAACCCAAGGTCGCCAGACTTATGACTGGACA

CTGAACAGAAACCAACCAGCTGCAACCTCTTTGGCCAACACTATAGAGGTGTTCAGATCG

AATGGTCTGACAGCCAATGAATCAGGGAGACTGATAGATTTTCTCAGGGATGTGATGGAA

TCAATGGATAAAGAAGAGATGGAAGTAACAACACATTTCCAGAGAAAAAGAAGAGTGAGG

GACAACATGACTAAAAAGATGGTCACACAAAGAACAATAGGGAAGAAGAAGCAGAGGCTG

AACAAGAGGAGTTACTTAATAAGAGCACTGACATTGAATACAATGACCAAAGATGCGGAA

AGAGGCAAGTTGAAGAGACGGGCAATTGCAACACCCGGGATGCAGATTAGAGGATTCGTG

TACTTCGTTGAAACACTAGCGAGGAGCATCTGTGAGAAACTAGAGCAATCTGGGCTCCCT

GTTGGAGGGAATGAGAAGAAGGCTAAATTGGCAAATGTTGTGAGAAAAATGATGACTAAC

TCACAAGATACAGAGCTCTCCTTTACAATTACTGGAGACAACACCAAATGGAATGAGAAT

CAAAACCCTCGGGTGTTTTTGGCAATGATAACATATATCACAAGAAACCAACCTGAATGG

TTTAGAAATGTCTTAAGCATTGCCCCTATAATGTTCTCAAACAAAATGGCGAGATTAGGG

AAAGGATACATGTTTGAAAGTAAGAGCATGAAGCTAAGAACACAAATACCAGCAGAGATG

CTTACAAATATTGATCTGAAGTATTTCAACGAACCAACGAGAAAGAAAATCGAGAAAATA

AGACCTCTGCTGATTGATGGCACGGCCTCATTGAGTCCTGGGATGATGATGGGCATGTTC

AATATGCTGAGCACAGTATTAGGGGTCTCAATCCTGAATCTCGGGCAAAAGAGGTACACC

AAAACCACATACTGGTGGGATGGACTTCAATCCTCTGATGATTTCGCTCTCATAGTGAAT

GCACCGAATCATGAGGGGATACAAGCAGGAGTGGATAGATTCTATAGGACCTGCAAACTG

GTTGGGATCAACATGAGCAAAAAGAAGTCTTACATAAACCGAACAGGAACATTTGAGTTC

ACAAGTTTTTTCTACCGCTATGGATTTGTAGCCAACTTCAGTATGGAATTACCCAGCTTT

GGAGTGTCTGGAATCAATGAATCAGCTGACATGAGCATTGGAGTTACAGTGATAAAAAAC

AATATGATAAACAATGATCTTGGACCAGCAACAGCTCAAATGGCTCTTCAGTTATTCATC

AAAGACTATAGGTACACATACCGATGCCACAGGGGTGATACACAAATTCAAACGAGGAGA

TCATTCGAGCTGAAGAAGCTGTGGGAGCAGACCCGTTCAAAGGCAGGGCTGTTGATATCA

GACGGGGGGCCAAACCTATACAACATTCGGAATCTCCACATCCCAGAGGTCTGCTTGAAG

TGGGAGCTGATGGATGAAGACTACCAAGGCAGGCTGTGCAATCCTCTGAATCCATTTGTC

AGTCATAAAGAGATTGAGTCCGTAAACAATGCTGTAGTAATGCCCGCCCATGGCCCGGCC

AAGAGCATGGAATATGATGCTGTTGCGACCACACACTCGTGGATTCCTAAGAGGAACCGT

TCCATTCTCAATACCAGCCAAAGGGGAATTCTTGAGGATGAGCAGATGTACCAAAAGTGC

TGTAGTCTATTCGAGAAATTCTTCCCCAGCAGTTCATACAGGAGGCCAGTTGGAATTTCC

AGCATGGTGGAGGCCATGGTGTCTAGGGCCCGAATTGATGCACGCATCGACTTCGAATCT

GGAAGGATTAAGAAAGAAGAGTTTGCTGAGATCATGAAGATCTGTTCCACCATTGAAGAG

CTCAGACGGCAAAAATAGTGAATTTAGCTTGTCCTTCATGAAAAAATGCCTTGTTTCTAC

T

>A_duck_Kazakhstan_12-20-B-Talg-11_2020_EPI1927661

AGCAAAAGCAGGCAAACTATTTGAATGGATGTCAATCCGACTTTACTTTTCTTAAAAGTG

CCAGCGCAAAATGCTATAAGTACTACATTCCCTTACACTGGAGATCCTCCATACAGCCAT

GGAACAGGAACAGGGTATACCATGGACACAGTAAACAGAACACATCAATACTCAGAAAAG

GGAAAGTGGACAACAAACACAGAAACCGGAGCACCCCAACTCAACCCAATTGATGGACCA

TTACCAGAGGACAATGAGCCAAGCGGATATGCACAAACTGATTGCGTGTTGGAAGCAATG

GCTTTCCTTGAAGAATCCCACCCAGGGATATTTGAAAACTCTTGTCTTGAAGCGATGGAA

ATCGTTCAGCAAACAAGAGTGGACAAACTAACCCAAGGTCGCCAGACTTATGACTGGACA

CTGAACAGAAACCAACCAGCTGCAACCTCTTTGGCCAACACTATAGAGGTGTTCAGATCG

AATGGTCTGACAGCCAATGAATCAGGGAGACTGATAGATTTTCTCAGGGATGTGATGGAA

TCAATGGATAAAGAAGAGATGGAAGTAACAACACATTTCCAGAGAAAAAGAAGAGTGAGG

GACAACATGACTAAGAAGATGGTCACACAAAGAACAATAGGGAAGAAGAAGCAGAGGCTG

AACAAGAGGAGTTACTTGATAAGAGCACTGACATTGAATACAATGACCAAAGATGCAGAA

AGAGGCAAATTGAAGAGACGGGCAATTGCAACACCCGGGATGCAGATTAGAGGATTCGTG

TACTTCGTTGAAACACTAGCGAGGAGCATCTGTGAGAAACTAGAGCAATCTGGGCTCCCT

GTTGGAGGGAATGAGAAGAAGGCTAAATTGGCAAATGTTGTGAGAAAAATGATGACTAAC

TCACAAGATACAGAGCTCTCCTTTACAATTACTGGAGACAACACCAAATGGAATGAGAAT

CAAAACCCTCGGATGTTTTTGGCAATGATAACATATATCACAAGAAACCAACCTGAATGG

TTTAGAAATGTCTTAAGCATTGCCCCTATAATGTTCTCAAACAAAATGGCGAGATTAGGG

AAAGGATACATGTTTGAAAGTAAGAGCATGAAGCTAAGAACACAAATACCAGCAGAGATG

CTTACAAATATTGATCTGAAGTATTTCAACGAACCAACGAGAAAGAAAATCGAGAAAATA

AGACCTCTGCTGATTGATGGCACGGCCTCATTGAGTCCTGGGATGATGATGGGCATGTTC

AATATGCTGAGCACAGTATTGGGGGTCTCAATCCTGAATCTCGGGCAAAAGAGGTACACC

AAAACCACATACTGGTGGGATGGACTTCAATCCTCTGATGATTTCGCTCTCATAGTGAAT

GCACCGAATCATGAGGGGATACAAGCAGGAGTGGATAGATTCTATAGGACCTGCAAACTG

GTTGGGATCAACATGAGCAAAAAGAAGTCTTACATAAACCGAACAGGAACATTTGAGTTC

ACAAGTTTTTTCTACCGCTATGGATTTGTAGCTAACTTCAGTATGGAATTACCCAGCTTT

GGAGTGTCTGGAATCAATGAATCAGCTGACATGAGCATTGGAGTTACAGTGATAAAAAAC

AATATGATAAACAATGATCTTGGACCAGCAACAGCTCAAATGGCTCTTCAGTTATTCATC

AAAGACTATAGGTACACATACCGATGCCACAGGGGTGATACACAAATTCAAACGAGGAGA

TCATTCGAGCTGAAGAAGCTGTGGGAGCAGACCCGTTCAAAGGCAGGGCTGTTGATATCA

GACGGGGGGCCAAACCTATACAACATTCGGAACCTCCACATCCCAGAGGTCTGCTTGAAG

TGGGAGCTGATGGATGAAGACTACCAAGGCAGGCTGTGCAATCCTCTGAATCCATTTGTC

AGTCATAAAGAGATTGAGTCCGTAAACAATGCTGTAGTAATGCCCGCCCATGGCCCGGCC

AAGAGCATGGAATATGATGCTGTTGCGACCACACATTCGTGGATTCCTAAGAGGAACCGC

TCCATTCTCAATACCAGCCAAAGGGGAATTCTTGAGGATGAGCAGATGTACCAAAAGTGC

TGTAGTCTATTCGAGAAATTCTTCCCCAGCAGTTCATACAGGAGGCCAGTTGGAATCTCC

AGCATGGTGGAGGCCATGGTGTCTAGGGCCCGAATTGATGCACGCATCGACTTCGAATCT

GGAAGGATTAAGAAAGAAGAGTTTGCTGAGATCATGAAGATCTGTTCCACCATTGAAGAG

CTCAGACGGCAAAAATAGTGAATTTAGCTTGTCCTTCATGAAAAAATGCCTTGTTTCTAC

T

>A_goose_Kazakhstan_7-20-B-Talg-12_2020_EPI1927667

AGCAAAAGCAGGCAAACTATTTGAATGGATGTCAATCCGACTTTACTTTTCTTAAAAGTG

CCAGCGCAAAATGCTATAAGTACTACATTCCCTTACACTGGAGATCCTCCATACAGCCAT

GGAACAGGAACAGGGTATACCATGGACACAGTAAACAGAACACATCAATACTCAGAAAAG

GGAAAGTGGACAACAAACACAGAAACCGGAGCACCCCAACTCAACCCAATTGATGGACCA

TTACCAGAGGACAATGAGCCAAGCGGATATGCACAAACTGATTGCGTGTTGGAAGCAATG

GCTTTCCTTGAAGAATCCCACCCAGGGATATTTGAAAACTCTTGTCTTGAAGCGATGGAA

ATCGTTCAGCAAACAAGAGTGGACAAACTAACCCAAGGTCGCCAGACTTATGACTGGACA

CTGAACAGAAACCAACCAGCTGCAACCTCTTTGGCCAACACTATAGAGGTGTTCAGATCG

AATGGTCTGACAGCCAATGAATCAGGGAGACTGATAGATTTTCTCAGGGATGTGATGGAA

TCAATGGATAAAGAAGAGATGGAAGTAACAACACATTTCCAGAGAAAAAGAAGAGTGAGG

GACAACATGACTAAGAAGATGGTCACACAAAGAACAATAGGGAAGAAGAAGCAGAGGCTG

AACAAGAGGAGTTACTTGATAAGAGCACTGACATTGAATACAATGACCAAAGATGCAGAA

AGAGGCAAATTGAAGAGACGGGCAATTGCAACACCCGGGATGCAGATTAGAGGATTCGTG

TACTTCGTTGAAACACTAGCGAGGAGCATCTGTGAGAAACTAGAGCAATCTGGGCTCCCT

GTTGGAGGGAATGAGAAGAAGGCTAAATTGGCAAATGTTGTGAGAAAAATGATGACTAAC

TCACAAGATACAGAGCTCTCCTTTACAATTACTGGAGACAACACCAAATGGAATGAGAAT

CAAAACCCTCGGATGTTTTTGGCAATGATAACATATATCACAAGAAACCAACCTGAATGG

TTTAGAAATGTCTTAAGCATTGCCCCTATAATGTTCTCAAACAAAATGGCGAGATTAGGG

AAAGGATACATGTTTGAAAGTAAGAGCATGAAGCTAAGAACACAAATACCAGCAGAGATG

CTTACAAATATTGATCTGAGGTATTTCAACGAACCAACGAGAAAGAAAATCGAGAAAATA

AGACCTCTGCTGATTGATGGCACGGCCTCATTGAGTCCTGGGATGATGATGGGCATGTTC

AATATGCTGAGCACAGTATTGGGGGTCTCAATCCTGAATCTCGGGCAAAAGAGGTACACC

AAAACCACATACTGGTGGGATGGACTTCAATCCTCTGATGATTTCGCTCTCATAGTGAAT

GCACCGAATCATGAGGGGATACAAGCAGGAGTGGATAGATTCTATAGGACCTGCAAACTG

GTTGGGATCAACATGAGCAAAAAGAAGTCTTACATAAACCGAACAGGAACATTTGAGTTC

ACAAGTTTTTTCTACCGCTATGGATTTGTAGCTAACTTCAGTATGGAATTACCCAGCTTT

GGAGTGTCTGGAATCAATGAATCAGCTGACATGAGCATTGGAGTTACAGTGATAAAAAAC

AATATGATAAACAATGATCTTGGACCAGCAACAGCTCAAATGGCTCTTCAGTTATTCATC

AAAGACTATAGGTACACATACCGATGCCACAGGGGTGATACACAAATTCAAACGAGGAGA

TCATTCGAGCTGAAGAAGCTGTGGGAGCAGACCCGTTCAAAGGCAGGGCTGTTGATATCA

GACGGGGGGCCAAACCTATACAACATTCGGAACCTCCACATCCCAGAGGTCTGCTTGAAG

TGGGAGCTGATGGATGAAGACTACCAAGGCAGGCTGTGCAATCCTCTGAATCCATTTGTC

AGTCATAAAGAGATTGAGTCCGTAAACAATGCTGTAGTAATGCCCGCCCATGGCCCGGCC

AAGAGCATGGAATATGATGCTGTTGCGACCACACATTCGTGGATTCCTAAGAGGAACCGC

TCCATTCTCAATACCAGCCAAAGGGGAATTCTTGAGGATGAGCAGATGTACCAAAAGTGC

TGTAGTCTATTCGAGAAATTCTTCCCCAGCAGTTCATACAGGAGGCCAGTTGGAATTTCC

AGCATGGTGGAGGCCATGGTGTCTAGGGCCCGAATTGATGCACGCATCGACTTCGAATCT

GGAAGGATTAAGAAAGAAGAGTTTGCTGAGATCATGAAGATCTGTTCCACCATTGAAGAG

CTCAGACGGCAAAAATAGTGAATTTAGCTTGTCCTTCATGAAAAAATGCCTTGTTTCTAC

T

>A_swan_Kazakhstan_9-20-B-Talg-39_2020_EPI1927695

AGCAAAAGCAGGCAAACTATTTGAATGGATGTCAATCCGACTTTACTTTTCTTAAAAGTG

CCAGCGCAAAATGCTATAAGTACTACATTCCCTTACACTGGAGATCCCCCATACAGCCAT

GGAACAGGAACAGGGTATACCATGGACACAGTAAACAGAACACATCAATACTCAGAAAAG

GGAAAGTGGACAACAAACACAGAAACCGGAGCACCCCAACTCAACCCAATTGATGGACCA

TTACCAGAGGACAATGAGCCAAGCGGATATGCACAAACTGATTGCGTGTTGGAAGCAATG

GCTTTCCTTGAAGAATCCCACCCAGGGATATTTGAAAACTCTTGTCTTGAAGCGATGGAA

ATCGTTCAGCAAACAAGAGTGGACAAACTAACCCAAGGTCGCCAGACTTATGACTGGACA

CTGAACAGAAACCAACCAGCTGCAACCTCTTTGGCCAACACTATAGAGGTGTTCAGATCT

AATGGTCTGACAGCCAATGAATCAGGGAGACTGATAGATTTTCTCAGGGATGTGATGGAA

TCAATGGATAAAGAAGAGATGGAAGTAACAACACATTTCCAGAGAAAAAGAAGAGTGAGG

GACAACATGACTAAGAAGATGGTCACACAAAGAACAATAGGGAAGAAGAAGCAGAGGCTG

AACAAGAGGAGTTACTTAATAAGAGCACTGACATTGAATACAATGACCAAAGATGCAGAA

AGAGGCAAGTTGAAGAGACGGGCAATTGCAACACCCGGGATGCAGATTAGAGGATTCGTG

TACTTCGTTGAAACACTAGCGAGGAGCATCTGTGAGAAACTAGAGCAATCTGGGCTCCCT

GTTGGAGGGAATGAGAAGAAGGCTAAATTGGCAAATGTTGTGAGAAAAATGATGACTAAC

TCACAAGATACAGAGCTCTCCTTTACAATTACTGGAGACAACACCAAATGGAATGAGAAT

CAAAACCCTCGGATGTTTTTGGCAATGATAACATATATCACAAGAAACCAACCTGAATGG

TTTAGAAATGTCTTAAGCATTGCCCCTATAATGTTCTCAAACAAAATGGCGAGATTAGGG

AAAGGATACATGTTTGAAAGTAAGAGCATGAAGCTAAGAACACAAATACCAGCAGAGATG

CTTACAAATATTGATCTGAAGTATTTCAACGAACCAACGAGAAAGAAAATCGAGAAAATA

AGACCTCTGCTGATTGATGGCACGGCCTCATTGAGTCCTGGGATGATGATGGGCATGTTC

AATATGCTGAGCACAGTATTAGGGGTCTCAATCCTGAATCTCGGGCAAAAGAGGTACACC

AAAACCACATACTGGTGGGATGGACTTCAATCCTCTGATGATTTCGCTCTCATAGTGAAT

GCACCGAATCATGAGGGGATACAAGCAGGAGTGGATAGATTCTATAGGACCTGCAAACTG

GTTGGGATCAACATGAGCAAAAAGAAGTCTTACATAAACCGAACAGGAACATTTGAGTTC

ACAAGTTTTTTCTACCGCTATGGATTTGTAGCCAACTTCAGTATGGAATTACCCAGCTTT

GGAGTGTCTGGAATCAATGAATCAGCTGACATGAGCATTGGAGTTACAGTGATAAAAAAC

AATATGATAAACAATGATCTTGGACCAGCAACAGCTCAAATGGCTCTTCAGTTATTCATC

AAAGACTATAGGTACACATACCGATGCCACAGGGGTGATACACAAATTCAAACGAGGAGA

TCATTCGAGCTGAAGAAGCTGTGGGAGCAGACCCGTTCAAAGGCAGGGCTGTTGATATCA

GACGGGGGGCCAAACCTATACAACATTCGGAATCTCCACATCCCAGAGGTCTGCTTGAAG

TGGGAGCTGATGGATGAAGACTACCAAGGCAGGCTGTGCAATCCTCTGAATCCATTTGTC

AGTCATAAAGAGATTGAGTCCGTAAACAATGCTGTAGTAATGCCCGCCCATGGCCCGGCC

AAGAGCATGGAATATGATGCTGTTGCGACCACACACTCGTGGATTCCTAAGAGGAACCGT

TCCATTCTCAATACCAGCCAAAGGGGAATTCTTGAGGATGAGCAGATGTACCAAAAGTGC

TGTAGTCTATTCGAGAAATTCTTCCCCAGCAGTTCATACAGGAGGCCAGTTGGAATTTCC

AGCATGGTGGAGGCCATGGTGTCTAGGGCCCGAATTGATGCACGCATCGACTTCGAATCT

GGAAGGATTAAGAAAGAAGAGTTTGCTGAGATCATGAAGATCTGTTCCACCATTGAAGAG

CTCAGACGGCAAAAATAGTGAATTTAGCTTGTCCTTCATGAAAAAATGCCTTGTTTCTAC

T

>A_chicken_Kazakhstan_12-20-B-Talg-45_2020_EPI1927701

AGCAAAAGCAGGCAAACTATTTGAATGGATGTCAATCCGACTTTACTTTTCTTAAAAGTG

CCAGCGCAAAATGCTATAAGTACTACATTCCCTTACACTGGAGATCCTCCATACAGCCAT

GGAACAGGAACAGGGTATACCATGGACACAGTAAACAGAACACATCAATACTCAGAAAAG

GGAAAGTGGACAACAAACACAGAAACCGGAGCACCCCAACTCAACCCAATTGATGGACCA

TTACCAGAGGACAATGAGCCAAGCGGATATGCACAAACTGATTGCGTGTTGGAAGCAATG

GCTTTCCTTGAAGAATCCCACCCAGGGATATTTGAAAACTCTTGTCTTGAAGCGATGGAA

ATCGTTCAGCAAACAAGAGTGGACAAACTAACCCAAGGTCGCCAGACTTATGACTGGACA

CTGAACAGAAACCAACCAGCTGCAACCTCTTTGGCCAACACTATAGAGGTGTTCAGATCG

AATGGTCTGACAGCCAATGAATCAGGGAGACTGATAGATTTTCTCAGGGATGTGATGGAA

TCAATGGATAAAGAAGAGATGGAAGTAACAACACATTTCCAGAGAAAAAGAAGAGTGAGG

GACAACATGACTAAGAAGATGGTCACACAAAGAACAATAGGGAAGAAGAAGCAGAGGCTG

AACAAGAGGAGTTACTTGATAAGAGCACTGACATTGAATACAATGACCAAAGATGCAGAA

AGAGGCAAGTTGAAGAGACGGGCAATTGCAACACCCGGGATGCAGATTAGAGGATTCGTG

TACTTCGTTGAAACACTAGCGAGGAGCATCTGTGAGAAACTAGAGCAATCTGGGCTCCCT

GTTGGAGGGAATGAGAAGAAGGCTAAATTGGCAAATGTTGTGAGAAAAATGATGACTAAC

TCACAAGATACAGAGCTCTCCTTTACAATTACTGGAGACAACACCAAATGGAATGAGAAT

CAAAACCCTCGGATGTTTTTGGCAATGATAACATATATCACAAGAAACCAACCTGAATGG

TTTAGAAATGTCTTAAGCATTGCCCCTATAATGTTCTCAAACAAAATGGCGAGATTAGGG

AAAGGATACATGTTTGAAAGTAAGAGCATGAAGCTAAGAACACAAATACCAGCAGAGATG

CTTACAAATATTGATCTGAAGTATTTCAACGAACCAACGAGAAAGAAAATCGAGAAAATA

AGACCTCTGCTGATTGATGGCACGGCCTCATTGAGTCCTGGGATGATGATGGGCATGTTC

AATATGCTGAGCACAGTATTGGGGGTCTCAATCCTGAATCTCGGGCAAAAGAGGTACACC

AAAACCACATACTGGTGGGATGGACTTCAATCCTCTGATGATTTCGCTCTCATAGTGAAT

GCACCAAATCATGAGGGGATACAAGCAGGAGTGGATAGATTCTATAGGACCTGCAAACTG

GTTGGGATCAACATGAGCAAAAAGAAGTCTTACATAAACCGAACAGGAACATTTGAGTTC

ACAAGTTTTTTCTACCGCTATGGATTTGTAGCTAACTTCAGTATGGAATTACCCAGCTTT

GGAGTGTCTGGAATCAATGAATCAGCTGACATGAGCATTGGAGTTACAGTGATAAAAAAC

AATATGATAAACAATGATCTTGGACCAGCAACAGCTCAAATGGCTCTTCAGTTATTCATC

AAAGACTATAGGTACACATACCGATGCCACAGGGGTGATACACAAATTCAAACGAGGAGA

TCATTCGAGCTGAAGAAGCTGTGGGAGCAGACCCGTTCAAAGGCAGGGCTGTTGATATCA

GACGGGGGGCCAAACCTATACAACATTCGGAACCTCCACATCCCAGAGGTCTGCTTGAAG

TGGGAGCTGATGGATGAAGACTACCAAGGCAGGCTGTGCAATCCTCTGAATCCATTTGTC

AGTCATAAAGAGATTGAGTCCGTAAACAATGCTGTAGTAATGCCCGCCCATGGCCCGGCC

AAGAGCATGGAATATGATGCTGTTGCGACCACACATTCGTGGATTCCTAAGAGGAACCGC

TCCATTCTCAATACCAGCCAAAGGGGAATTCTTGAGGATGAGCAGATGTACCAAAAGTGC

TGTAGTCTATTCGAGAAATTCTTCCCCAGCAGTTCATACAGGAGGCCAGTTGGAATTTCC

AGCATGGTGGAGGCCATGGTGTCTAGGGCCCGAATTGATGCACGCATCGACTTCGAATCT

GGAAGGATTAAGAAAGAAGAGTTTGCTGAGATCATGAAGATCTGTTCCACCATTGAAGAG

CTCAGACGGCAAAAATAGTGAATTTAGCTTGTCCTTCATGAAAAAATGCCTTGTTTCTAC

T

>A_crow_Kazakhstan_15-20-B-Talg-4_2020_EPI1927707

AGCAAAAGCAGGCAAACTATTTGAATGGATGTCAATCCGACTTTACTTTTCTTAAAAGTG

CCAGCGCAAAATGCTATAAGTACTACATTCCCTTACACTGGAGATCCTCCATACAGCCAT

GGAACAGGAACAGGGTATACCATGGACACAGTAAACAGAACACATCAATACTCAGAAAAG

GGAAAGTGGACAACAAACACAGAAACCGGAGCGCCCCAACTCAACCCAATTGATGGACCA

TTACCAGAGGACAATGAGCCAAGCGGATATGCACAAACTGATTGCGTGTTGGAAGCAATG

GCTTTCCTTGAAGAATCCCACCCAGGGATATTTGAAAACTCTTGTCTTGAAGCGATGGAA

ATCGTTCAGCAAACAAGAGTGGACAAACTAACCCAAGGTCGCCAGACTTATGACTGGACA

CTGAACAGAAACCAACCAGCTGCAACCTCTTTGGCCAACACTATAGAGGTGTTCAGATCG

AATGGTCTGACAGCCAATGAATCAGGGAGACTGATAGATTTTCTCAGGGATGTGATGGAA

TCAATGGATAAAGAAGAGATGGAAGTAACAACACATTTCCAGAGAAAAAGAAGAGTGAGG

GACAACATGACTAAGAAGATGGTCACACAAAGAACAATAGGGAAGAAGAAGCAGAGGCTG

AACAAGAGGAGTTACTTGATAAGAGCACTGACATTGAATACAATGACCAAAGATGCAGAA

AGAGGCAAATTGAAGAGACGGGCAATTGCAACACCCGGGATGCAGATTAGAGGATTCGTG

TACTTCGTTGAAACACTAGCGAGGAGCATCTGTGAGAAACTAGAGCAATCTGGGCTCCCT

GTTGGAGGGAATGAGAAGAAGGCTAAATTGGCAAATGTTGTGAGAAAAATGATGACTAAC

TCACAAGATACAGAGCTCTCCTTTACAATTACTGGAGACAACACCAAATGGAATGAGAAT

CAAAACCCTCGGATGTTTTTGGCAATGATAACATATATCACAAGAAACCAACCTGAATGG

TTTAGAAATGTCTTAAGCATTGCCCCTATAATGTTCTCAAACAAAATGGCGAGATTAGGG

AAAGGATACATGTTTGAAAGTAAGAGCATGAAGCTAAGAACACAAATACCAGCAGAGATG

CTTACAAATATTGATCTGAAGTATTTCAACGAACCAACGAGAAAGAAAATCGAGAAAATA

AGACCTCTGCTGATTGATGGCACGGCCTCATTGAGTCCTGGGATGATGATGGGCATGTTC

AATATGCTGAGCACAGTATTGGGGGTCTCAATCCTGAATCTCGGGCAAAAGAGGTACACC

AAAACCACATACTGGTGGGATGGACTTCAATCCTCTGATGATTTCGCTCTCATAGTGAAT

GCACCGAATCATGAGGGGATACAAGCAGGAGTGGATAGATTCTATAGGACCTGCAAACTG

GTTGGGATCAACATGAGCAAAAAGAAGTCTTACATAAACCGAACAGGAACATTTGAGTTC

ACAAGTTTTTTCTACCGCTATGGATTTGTAGCTAACTTCAGTATGGAATTACCCAGCTTT

GGAGTGTCTGGAATCAATGAATCAGCTGACATGAGCATTGGAGTTACAGTGATAAAAAAC

AATATGATAAACAATGATCTTGGACCAGCAACAGCTCAAATGGCTCTTCAGTTATTCATC

AAAGACTATAGGTACACATACCGATGCCACAGGGGTGATACACAAATTCAAACGAGGAGA

TCATTCGAGCTGAAGAAGCTGTGGGAGCAGACCCGTTCAAAGGCAGGGCTGTTGATATCA

GACGGGGGGCCAAACCTATACAACATTCGGAACCTCCACATCCCAGAGGTCTGCTTGAAG

TGGGAGCTGATGGATGAAGACTACCAAGGCAGGCTGTGCAATCCTCTGAATCCATTTGTC

AGTCATAAAGAGATTGAGTCCGTAAACAATGCTGTAGTAATGCCCGCCCATGGCCCGGCC

AAGAGCATGGAATATGATGCTGTTGCGACCACACATTCGTGGATTCCTAAGAGGAACCGC

TCCATTCTCAATACCAGCCAAAGGGGAATTCTTGAGGATGAGCAGATGTACCAAAAGTGC

TGTAGTCTATTCGAGAAATTCTTCCCCAGCAGTTCATACAGGAGGCCAGTTGGAATTTCC

AGCATGGTGGAGGCCATGGTGTCTAGGGCCCGAATTGATGCACGCATCGACTTCGAATCT

GGAAGGATTAAGAAAGAAGAGTTTGCTGAGATCATGAAGATCTGTTCCACCATTGAAGAG

CTCAGACGGCAAAAATAGTGAATTTAGCTTGTCCTTCATGAAAAAATGCCTTGTTTCTAC

T

>A_swan_Kazakhstan_1-267-20-B-Talg-52_2020_EPI1927713

AGCAAAAGCAGGCAAACTATTTGAATGGATGTCAATCCGACTTTACTTTTCTTAAAAGTG

CCAGCGCAAAATGCTATAAGTACTACATTCCCTTACACTGGAGATCCTCCATACAGCCAT

GGAACAGGAACAGGGTATACCATGGACACAGTAAACAGAACACATCAATACTCAGAAAAG

GGAAAGTGGACAACAAACACAGAAACAGGAGCACCCCAACTCAACCCAATTGATGGACCA

TTACCAGAGGACAATGAGCCAAGCGGATATGCACAAACTGATTGCGTGTTGGAAGCAATG

GCTTTCCTTGAAGAATCCCACCCAGGGATATTTGAAAACTCTTGTCTTGAAGCGATGGAA

ATCGTTCAGCAAACAAGAGTGGACAAACTAACCCAAGGTCGCCAGACTTATGACTGGACA

CTGAATAGAAACCAACCAGCTGCAACCTCTTTGGCCAACACTATAGAGGTGTTCAGATCG

AATGGTCTGACAGCCAATGAATCAGGGAGACTGATAGATTTTCTCAGGGATGTGATGGAA

TCAATGGATAAAGAAGAGATGGAAGTAACAACACATTTCCAGAGAAAAAGAAGAGTGAGG

GACAACATGACTAAGAAGATGGTCACACAAAGAACAATAGGGAAGAAGAAGCAGAGGCTG

AACAAGAGGAGTTACTTAATAAGAGCACTGACATTGAATACAATGACCAAAGATGCAGAA

AGAGGCAAGTTGAAGAGACGGGCAATTGCAACACCCGGGATGCAGATTAGAGGATTCGTG

TACTTCGTTGAAACACTAGCGAGGAGCATCTGTGAGAAACTAGAGCAATCTGGGCTCCCT

GTTGGAGGGAATGAGAAGAAGGCTAAATTGGCAAATGTTGTGAGAAAAATGATGACTAAC

TCACAAGATACAGAGCTCTCCTTTACAATTACTGGAGACAACACCAAATGGAATGAGAAT

CAAAACCCTCGGATGTTTTTGGCAATGATAACATATATCACAAGAAACCAACCTGAATGG

TTTAGAAATGTCTTAAGCATTGCCCCTATAATGTTCTCAAACAAAATGGCGAGATTAGGG

AAAGGATACATGTTTGAAAGTAAGAGCATGAAGCTAAGAACACAAATACCAGCAGAGATG

CTTACAAATATTGATCTGAAGTATTTCAACGAACCAACGAGAAAGAAAATCGAGAAAATA

AGACCTCTGCTGATTGATGGCACGGCCTCATTGAGTCCTGGGATGATGATGGGCATGTTC

AATATGCTGAGCACAGTATTGGGGGTCTCAATCCTGAATCTCGGGCAAAAGAGGTACACC

AAAACCACATACTGGTGGGATGGACTTCAATCCTCTGATGATTTCGCTCTCATAGTGAAT

GCACCGAATCATGAGGGGATACAAGCAGGAGTGGATAGATTCTATAGGACCTGCAAACTG

GTTGGGATCAACATGAGCAAAAAGAAGTCTTACATAAACCGAACAGGAACATTTGAGTTC

ACAAGTTTTTTCTACCGCTATGGATTTGTAGCTAACTTCAGTATGGAATTACCCAGCTTT

GGAGTGTCTGGAATCAATGAATCAGCTGACATGAGCATTGGAGTTACAGTGATAAAAAAC

AATATGATAAACAATGATCTTGGACCAGCAACAGCTCAAATGGCTCTTCAGTTATTCATC

AAAGACTATAGGTACACATACCGATGCCACAGGGGTGATACACAAATTCAAACGAGGAGA

TCATTCGAGCTGAAGAAGCTGTGGGAGCAGACCCGTTCAAAGGCAGGGCTGTTGATATCA

GACGGGGGGCCAAACCTATACAACATTCGGAACCTCCACATCCCAGAGGTCTGCTTGAAG

TGGGAGCTGATGGATGAAGACTACCAAGGCAGGCTGTGCAATCCTCTGAATCCATTTGTC

AGTCATAAAGAGATTGAGTCCGTAAACAATGCTGTAGTAATGCCCGCCCATGGCCCGGCC

AAGAGCATGGAATATGATGCTGTTGCGACCACACATTCGTGGATTCCTAAGAGGAACCGT

TCCATTCTCAATACCAGCCAAAGGGGAATTCTTGAGGATGAGCAGATGTACCAAAAGTGC

TGTAGTCTATTCGAGAAATTCTTCCCCAGCAGTTCATACAGGAGGCCAGTTGGAATTTCC

AGCATGGTGGAGGCCATGGTATCTAGGGCCCGAATTGATGCACGCATCGACTTCGAATCT

GGAAGGATTAAGAAAGAAGAGTTTGCTGAGATCATGAAGATCTGTTCCACCATTGAAGAG

CTCAGACGGCAAAAATAGTGAATTTAGCTTGTCCTTCATGAAAAAATGCCTTGTTTCTAC

T

>A_pigeon_Kazakhstan_15-20-B-Talg-5_2020_EPI1927719

AGCAAAAGCAGGCAAACTATTTGAATGGATGTCAATCCGACTTTACTTTTCTTAAAAGTG

CCAGCGCAAAATGCTATAAGTACTACATTCCCTTACACTGGAGATCCTCCATACAGCCAT

GGAACAGGAACAGGGTATACCATGGACACAGTAAACAGAACACATCAATACTCAGAAAAG

GGAAAGTGGACAACAAACACAGAAACCGGAGCACCCCAACTCAACCCAATTGATGGACCA

TTACCAGAGGACAATGAGCCAAGCGGATATGCACAAACTGATTGCGTGTTGGAAGCAATG

GCTTTCCTTGAAGAATCCCACCCAGGGATATTTGAAAACTCTTGTCTTGAAGCGATGGAA

ATCGTTCAGCAAACAAGAGTGGACAAACTAACCCAAGGTCGCCAGACTTATGACTGGACA

CTGAACAGAAACCAACCAGCTGCAACCTCTTTGGCCAACACTATAGAGGTGTTCAGATCG

AATGGTCTGACAGCCAATGAATCAGGGAGACTGATAGATTTTCTCAGGGATGTGATGGAA

TCAATGGATAAAGAAGAGATGGAAGTAACAACACATTTCCAGAGAAAAAGAAGAGTGAGG

GACAACATGACTAAGAAGATGGTCACACAAAGAACAATAGGGAAGAAGAAGCAGAGGCTG

AACAAGAGGAGTTACTTGATAAGAGCACTGACATTGAATACAATGACCAAAGATGCAGAA

AGAGGCAAATTGAAGAGACGGGCAATTGCAACACCCGGGATGCAGATTAGAGGATTCGTG

TACTTCGTTGAAACACTAGCGAGGAGCATCTGTGAGAAACTAGAGCAATCTGGGCTCCCT

GTTGGAGGGAATGAGAAGAAGGCTAAATTGGCAAATGTTGTGAGAAAAATGATGACTAAC

TCACAAGATACAGAGCTCTCCTTTACAATTACTGGAGACAACACCAAATGGAATGAGAAT

CAAAACCCTCGGATGTTTTTGGCAATGATAACATATATCACAAGAAACCAACCTGAATGG

TTTAGAAATGTCTTAAGCATTGCCCCTATAATGTTCTCAAACAAAATGGCGAGATTAGGG

AAAGGATACATGTTTGAAAGTAAGAGCATGAAGCTAAGAACACAAATACCAGCAGAGATG

CTTACAAATATTGATCTGAAGTATTTCAACGAACCAACGAGAAAGAAAATCGAGAAAATA

AGACCTCTGCTGATTGATGGCACGGCCTCATTGAGTCCTGGGATGATGATGGGCATGTTC

AATATGCTGAGCACAGTATTGGGGGTCTCAATCCTGAATCTCGGGCAAAAGAGGTACACC

AAAACCACATACTGGTGGGATGGACTTCAATCCTCTGATGATTTCGCTCTCATAGTGAAT

GCACCGAATCATGAGGGGATACAAGCAGGAGTGGATAGATTCTATAGGACCTGCAAACTG

GTTGGGATCAACATGAGCAAAAAGAAGTCTTACATAAACCGAACAGGAACATTTGAGTTC

ACAAGTTTTTTCTACCGCTATGGATTTGTAGCTAACTTCAGTATGGAATTACCCAGCTTT

GGAGTGTCTGGAATCAATGAATCAGCTGACATGAGCATTGGAGTTACAGTGATAAAAAAC

AATATGATAAACAATGATCTTGGACCAGCAACAGCTCAAATGGCTCTTCAGTTATTCATC

AAAGACTATAGGTACACATACCGATGCCACAGGGGTGATACACAAATTCAAACGAGGAGA

TCATTCGAGCTGAAGAAGCTGTGGGAGCAGACCCGTTCAAAGGCAGGGCTGTTGATATCA

GACGGGGGGCCAAACCTATACAACATTCGGAACCTCCACATCCCAGAGGTCTGCTTGAAG

TGGGAGCTGATGGATGAAGACTACCAAGGCAGGCTGTGCAATCCTCTGAGTCCATTTGTC

AGTCATAAAGAGATTGAGTCCGTAAACAATGCTGTAGTAATGCCCGCCCATGGCCCGGCC

AAGAGCATGGAATATGATGCTGTTGCGACCACACATTCGTGGATTCCTAAGAGGAACCGC

TCCATTCTCAATACCAGCCAAAGGGGAATTCTTGAGGATGAGCAGATGTACCAAAAGTGC

TGTAGTCTATTCGAGAAATTCTTCCCCAGCAGTTCATACAGGAGGCCAGTTGGAATTTCC

AGCATGGTGGAGGCCATGGTGTCTAGGGCCCGAATTGATGCACGCATCGACTTCGAATCT

GGAAGGATTAAGAAAGAAGAGTTTGCTGAGATCATGAAGATCTGTTCCACCATTGAAGAG

CTCAGACGGCAAAAATAGTGAATTTAGCTTGTCCTTCATGAAAAAATGCCTTGTTTCTAC

T

>A_chicken_Kazakhstan_1-20-B-Talg-67_2020_EPI1927725

AGCAAAAGCAGGCAAACTATTTGAATGGATGTCAATCCGACTTTACTTTTCCTAAAAGTG

CCAGCGCAAAATGCTATAAGTACTACATTCCCTTACACTGGAGATCCTCCATACAGCCAT

GGAACAGGAACAGGGTATACCATGGACACAGTAAACAGAACACATCAATACTCAGAAAAG

GGAAAGTGGACAACAAACACAGAAACCGGAGCACCCCAACTCAACCCAATTGATGGACCA

TTACCAGAGGACAATGAGCCAAGCGGATATGCACAAACTGATTGCGTGTTGGAAGCAATG

GCTTTCCTTGAAGAATCCCACCCAGGGATATTTGAAAACTCTTGTCTCGAAGCGATGGAA

ATCGTTCAGCAAACAAGAGTGGACAAACTAACCCAAGGTCGCCAGACTTATGACTGGACA

CTGAACAGAAACCAACCAGCTGCAACCTCTTTGGCCAACACTATAGAGGTGTTCAGATCG

AATGGTCTGACAGCCAATGAATCAGGGAGACTGATAGATTTTCTCAGGGATGTGATGGAA

TCAATGGATAAAGAAGAGATGGAAGTAACAACACATTTCCAGAGAAAAAGAAGAGTGAGG

GACAACATGACTAAGAAGATGGTCACACAAAGAACAATAGGGAAGAAGAAGCAGAGGCTG

AACAAGAGGAGTTACTTGATAAGAGCACTGACATTGAATACAATGACCAAAGATGCAGAA

AGAGGCAAATTGAAGAGACGGGCAATCGCAACACCCGGGATGCAGATTAGAGGATTCGTG

TACTTCGTTGAAACACTAGCGAGGAGCATCTGTGAGAAACTAGAGCAATCTGGGCTCCCT

GTTGGAGGGAATGAGAAGAAGGCTAAATTGGCAAATGTTGTGAGAAAAATGATGACTAAC

TCACAAGATACAGAGCTCTCCTTTACAATTACTGGAGACAACACCAAATGGAATGAGAAT

CAAAACCCTCGGATGTTTTTGGCAATGATAACATATATCACAAGAAACCAACCTGAATGG

TTTAGAAATGTCTTAAGCATTGCCCCTATAATGTTCTCAAACAAAATGGCGAGATTAGGG

AAAGGATACATGTTTGAAAGTAAGAGCATGAAGCTAAGAACACAAATACCAGCAGAGATG

CTTACAAATATTGATCTGAAGTATTTCAACGAACCAACGAGAAAGAAAATCGAGAAAATA

AGACCTCTGCTGATTGATGGCACGGCCTCATTGAGTCCTGGGATGATGATGGGCATGTTC

AATATGCTGAGCACAGTATTGGGGGTCTCAATCCTGAATCTCGGGCAAAAGAGGTACACC

AAAACCACATACTGGTGGGATGGACTTCAATCCTCTGATGATTTCGCTCTCATAGTGAAT

GCACCGAATCATGAGGGGATACAAGCAGGAGTGGATAGATTCTATAGGACCTGCAAACTG

GTTGGGATCAACATGAGCAAAAAGAAGTCTTACATAAACCGAACAGGAACATTTGAGTTC

ACAAGTTTTTTCTACCGCTATGGATTTGTAGCTAACTTCAGTATGGAATTACCCAGCTTT

GGAGTGTCTGGAATCAATGAATCAGCTGACATGAGCATTGGAGTTACAGTGATAAAAAAC

AATATGATAAACAATGATCTTGGACCAGCAACAGCTCAAATGGCTCTTCAGTTATTCATC

AAAGACTATAGGTACACATACCGATGCCACAGGGGTGATACACAAATTCAAACGAGGAGA

TCATTCGAGCTGAAGAAGCTGTGGGAGCAGACCCGTTCAAAGGCAGGGCTGTTGATATCA

GATGGGGGGCCAAACCTATACAACATTCGGAACCTCCACATCCCAGAGGTCTGCTTGAAG

TGGGAGCTGATGGATGAAGACTACCAAGGCAGGCTGTGCAATCCTCTGAATCCATTTGTC

AGTCATAAAGAGATTGAGTCCGTAAACAATGCTGTAGTAATGCCCGCCCATGGCCCGGCC

AAGAGCATGGAATATGATGCTGTTGCGACCACACATTCGTGGATTCCTAAGAGGAACCGC

TCCATTCTCAATACCAGCCAAAGGGGAATTCTTGAGGATGAGCAGATGTACCAAAAGTGC

TGTAGTCTATTCGAGAAATTCTTCCCCAGCAGTTCATACAGGAGGCCAGTTGGAATTTCC

AGCATGGTGGAGGCCATGGTGTCTAGGGCCCGAATTGATGCACGCATCGACTTCGAATCT

GGAAGGATTAAGAAAGAAGAGTTTGCTGAGATCATGAAGATCTGTTCCACCATTGAAGAG

CTCAGACGGCAAAAATAGTGAATTTAGCTTGTCCTTCATGAAAAAATGCCTTGTTTCTAC

T

>A_duck_Lao_961_2010_EPI335157

------------------------ATGGATGTCAATCCGACTTTACTTTTCTTGAAAGTA

CCAGTGCAAAATGCTATAAGTACCACATTCCCTTATACTGGAGACCCTCCATACAGCCAT

GGGACAGGGACAGGGTACACCATGGACACAGTCAACAGAACACACCAATATTCAGAAAAG

GGGAAGTGGACAACAAACACAGAGACTGGAGCACCCCAACTCAACCCGATTGATGGACCA

CTACCTGAGGATAATGAGCCCAGTGGGTATGCACAGACAGATTGTGTATTGGAAGCAATG

GCTTTCCTTGAAGAATCCCACCCAGGGATCTTTGAAAACTCGTGTCTTGAAACGATGGAA

ATCGTTCAACAAACAAGAGTGGATAAACTGACCCAAGGCCGCCAGACTTATGACTGGACA

CTGAATAGAAACCAACCGGCTGCAACTGCTTTGGCCAACACTATAGAAATCTTCAGATCG

AACGGTCTGACAGCAAATGAATCGGGACGGCTAATAGATTTCCTCAAAGACGTGATGGAA

TCAATGGATAAGGAAGAAATGGAGATAACAACACATTTCCAGAGAAAGAGAAGAATACGG

GACAACATGACCAAGAAAATGGTGACACAAAGAACAATAGGGAAGAAAAAACAAAGGCTA

AACAAAAGGAGCTACCTGATACGCGCACTGACACTGAACACAATGACAAAGGATGCAGAA

AGAGGCAAACTGAAGAGGCGCGCAATTGCAACACCCGGAATGCAAATCAGAGGATTCGTG

TACTTTGTTGAAACACTAGCGAGGAGTATCTGTGAGAAACTTGAGCAATCTGGACTCCCA

GTCGGAGGGAACGAGAAGAAAGCTAAATTGGCAAACGTCGTGAGGAAGATGATGACTAAC

TCACAGGATACTGAACTCTCCTTTACAATTACTGGAGACAATACAAAATGGAATGAGAAT

CAGAACCCTAGGATGTTTCTGGCAATGATAACGTACATCACAAGGAACCAGCCAGAATGG

TTTAGAAATGTCTTAAGCATTGCCCCTATAATGTTCTCAAACAAGATGGCGAGGTTAGGG

AAAGGATACATGTTCGAAAGTAAGAGCATGAAGTTACGAACACAAATACCAGCAGAAATG

CTTGCAAACATTGATCTTAAATACTTCAATGAATTAACGAAAAAGAAAATTGAGAAGATA

AGACCTCTATTAATAGATGGTACAGCCTCATTGAGCCCTGGAATGATGATGGGCATGTTC

AACATGTTGAGTACAGTCCTAGGAGTCTCAATCCTGAATCTTGGACAGAAAAGGTACACC

AAAACCACATATTGGTGGGACGGACTCCAGTCCTCTGATGATTTCGCTCTCATCGTCAAT

GCACCGAATCATGAGGGAATACAAGCAGGGGTGGATAGGTTTTATAGGACTTGTAAACTA

GTTGGAATCAATATGAGCAAGAAGAAGTCTTACATAAATCGGACAGGGACATTTGAATTC

ACGAGCTTTTTCTATCGCTATGGATTTGTAGCCAATTTCAGTATGGAACTGCCCAGTTTT

GGAGTGTCTGGAATTAATGAATCGGCCGACATGAGCATTGGTGTTACAGTGATAAAGAAC

AATATGATAAACAACGACCTTGGGCCAGCAACAGCTCAGATGGCTCTTCAGCTATTTATC

AAGGACTACAGATACACATACCGATGCCACAGAGGGGATACGCAAATCCAAACGAGAAGA

TCATTCGAGTTGAAGAAGCTATGGGAGCAAACCCGTTCAAAAGCCGGACTGTTGGTTTCA

GATGGAGGACCAAATCTATACAATATCCGAAATCTCCATATTCCTGAGGTCTGCTTGAAA

TGGGAATTGATGGATGAAGACTACCAGGGCAGACTGTGCAATCCTCTGAATCCATTCGTC

AGCCATAAGGAAATTGAATCTGTCAACAATGCTATAGTAATGCCAGCTCATGGTCCGGCC

AAAAGTATGGAATATGATGCCGTTGCAACTACACATTCATGGGTCCCTAAAAGGAATCGT

TCCATTCTCAATACAAGTCAAAGGGGAATTCTTGAGGATGAACAGATGTACCAAAAGTGC

TGCAACCTATTCGAGAAATTCTTCCCCAGCAGCTCATATCGGAGGCCAGTTGGAATTTCC

AGCATGGTGGAGGCCATGGTGTCTAGGGCCCGAATTGACGCACGAATTGATTTCGAGTCT

GGAAGGATTAAGAAAGAAGAGTTTGCTGAGATCATGAAGATCTGTTCCACCATTGAAGAG

CTCAGACGGCAAAAATAG------------------------------------------

-

>A_breeder_duck_Korea_Gochang1_2014_EPI509697

--------------AACCATTTGAATGGATGTCAACCCGACTTTACTTTTCTTGAAAGTG

CCAGCGCAAAATGCCATAAGTACCACATTCCCTTATACTGGAGATCCTCCATACAGCCAT

GGAACGGGGACAGGATACACCATGGACACAGTAAACAGAACACATCAATACTCAGAAAAG

GGAAAGTGGACAACAAACACTGAAACTGGAGCACCCCAACTTAATCCAATTGATGGGCCA

TTGCCTGAGGATAACGAGCCAAGCGGATATGCACAAACGGATTGTGTATTGGAAGCAATG

GCTTTCCTTGAAGAGTCCCACCCAGGGATCTTTGAAAACTCATGTCTTGAAACGATGGAA

ATTGTTCAGCAAACAAGAGTGGACAAACTGACCCAAGGTCGCCAGACCTATGATTGGACA

TTGAATAGAAACCAGCCGGCAGCAACTGCTTTAGCCAACACTATAGAAGTCTTCAGATCA

AACGGTCTAACAGCCAATGAATCAGGGAGATTGATAGATTTCCTCAAGGATGTGGTGGAG

TCAATGGATAAAGAAGAAATGGAAATAACAACACATTTCCAAAGAAAGAGAAGAGTAAGA

GACAATATGACCAAGAAAATGGTCACACAAAGAACAATAGGGAAGAAAAAACAAAGGCTG

AACAAGAAGAGCTATTTGATAAGAGCACTGACACTGAACACAATGACAAAGGATGCAGAA

AGAGGCAAATTGAAAAGGCGGGCAATTGCAACACCGGGGATGCAGATCAGAGGTTTCGTG

TATTTTGTCGAAACACTAGCAAGGAGCATCTGTGAAAAACTTGAGCAATCTGGGCTCCCT

GTTGGAGGGAATGAGAAGAAGGCTAAATTGGCGAATGTAGTGAGAAAAATGATGACTAAC

TCACAAGACACAGAGCTCTCCTTTACAATTACTGGAGACAATACCAAATGGAATGAAAAT

CAGAACCCTCGGATGTTTTTGGCGATGATAACATACATCACAAGAAACCAACCTGAATGG

TTTAGAAATATATTGAGTATCGCCCCTATAATGTTCTCAAACAAGATGGCAAGATTAGGG

AAAGGATACATGTTCGAGAGTAAGAGCATGAAACTACGAACACAAATACCAGCAGAAATG

CTCGCAGACATTGATCTGAAATACTTCAATGAATCAACAAGGAAGAAAATTGAGAAAATA

AGACCTCTCCTAATAGATGGGACTGCCTCATTGAGTCCTGGAATGATGATGGGCATGTTC

AACATGCTGAGCACAGTATTAGGAGTCTCAATCCTAAATCTTGGGCAAAAGAGGTACACC

AAAACCACATACTGGTGGGACGGACTCCAATCTTCTGATGATTTCGCTCTCATAGTAAAT

GCACCAAACCATGAGGGGATACAGGCCGGAGTAGACAGGTTCTATAGAACCTGTAAGCTG

GTTGGGATCAATATGAGTAAAAAGAAGTCTTACATAAATCGGACAGGAACATTTGAGTTC

ACAAGCTTTTTCTACCGTTATGGATTTGTAGCCAACTTCAGTATGGAGCTGCCAAGCTTT

GGAGTTTCTGGGATTAATGAATCGGCTGACATGAGCATTGGAGTTACAGTGATAAAGAAC

AATATGATAAACAATGATCTTGGACCAGCAACAGCTCAAATGGCTCTTCAGTTATTCATC

AAGGACTACAGATACACATATCGATGCCACAGGGGCGATACACAAATTCAAACGAGGAGA

TCATTCGAGCTAAAGAAACTGTGGGAGCAGACCCGTTCAAAAGCAGGACTGTTGGTTTCA

GATGGAGGACCAAACCTATACAATATTCGGAATCTCCACATTCCCGAGGTCTGCTTGAAG

TGGGAACTGATGGATGAAGATTACCAGGGTAGACTGTGTAATCCTCTGAATCCCTTTGTC

AGTCATAAGGAAATTGAGTCTGTGAACAATGCTGTAGTAATGCCAGCCCATGGTCCAGCC

AAAAGCATGGAATATGATGCTGTTGCGACTACACACTCATGGACCCCTAAAAGGAACCGT

TCCATTCTGAATACTAGCAAAAGAGGAATCCTTGAGGATGAACAGATGTACCAGAAGTGC

TGCAATCTATTTGAAAAATTCTTCCCTAGTAGTTCATACAGGAGGCCAGTTGGAATCTCC

AGCATGGTGGAGGCCATGGTGTCTAGGTCCCGAATTGATGCACGGATTGACTTCGAATCT

GGAAGGATTAAGAAGGAAGATTTTGCTGAGATCATGAAGATCTGTTCCACCATTGAAGAG

CTCAGACGGCAGAAATAGTGAATTTAGCTTGTCCTTCATGAAA-----------------

-

>A_broiler_duck_Korea_Buan2_2014_EPI509703

--------------AACTATTTGAATGGATGTCAACCCGACTTTACTCTTCTTGAAAGTG

CCAGCGCAAAATGCTATAAGTACCACATTCCCTTATACTGGAGATCCTCCATACAGCCAT

GGAACAGGAACAGGATACACCATGGACACAGTCAACAGAACGCATCAATACTCAGAAAAG

GGAAAGTGGACAACAAACACCGAGACTGGAGCACCCCAACTCAACCCAATTGATGGACCA

TTGCCTGAGGATAACGAGCCAAGCGGATATGCACAAACGGATTGTGTGTTGGAAGCAATG

GCTTTCCTTGAAGAGTCCCACCCAGGGATCTTTGAAAACTCATGTCTTGAAACAATGGAA

ATTGTTCAACAAACAAGAGTGGATAAACTGACCCAAGGTCGTCAGACCTATGACTGGACA

TTGAATAGAAACCAGCCGGCTGCAACTGCTTTAGCCAACACTATAGAAGTCTTCAGATCG

AACGGTCTAACAGCCAATGAGTCAGGGAGACTGATAGATTTCCTCAAAGATGTGATGGAG

TCAATGGACAAAGAAGAAATGGAAATAACAACACATTTCCAAAGAAAGAGAAGAGTAAGA

GACAATATGACCAAGAAAATGGTCACACAAAGAACAATAGGGAAGAAAAAACAGAGACTG

AACAAGAAGAACTACTTGGTAAGGGCACTGACACTGAACACAATGACAAAAGATGCAGAA

AGAGGCAAGTTGAAGAGGCGGGCAATTGCAACACCCGGGATGCAAATCAGAGGGTTCGTG

TACTTTGTCGAAACATTAGCGAGGAGCATCTGCGAGAAACTTGAGCAATCTGGGCTCCCT

GTTGGAGGAAATGAAAAAAAGGCTAAATTGGCAAATGTCGTGAGAAAGATGATGACTAAC

TCACAAGACACAGAGCTATCCTTTACAATTACTGGAGACAATACCAAGTGGAACGAGAAT

CAGAATCCTCGGATTTTTTTGGCAATGATAACATATATCACAAGAAATCAACCTGAGTGG

TTTAGAAATGTGTTAAGTATTGCCCCTATAATGTTCTCAAACAAAATGGCAAGATTAGGG

AAAGGATACATGTTCGAAAGTAAGAGCATGAAGCTACGGACACAAATACCAGCAGAAATG

CTTGCAACCATTGACCTGAAATATTTCAACGAATCGACAAGAAAGAAAATTGAGAAAATA

AGGCCTCTCCTAATAGAAGGGACAGCCTCGTTGAGTCCTGGAATGATGATGGGCATGTTC

AACATGCTGAGTACAGTCTTGGGAGTATCAATTCTAAATCTTGGCCAAAAGAGGTACACC

AAAACCACATACTGGTGGGACGGACTTCAATCCTCTGATGATTTCGCTCTCATAGTAAAT

GCACCGAATCATGAGGGAATACAGGCAGGAGTGGACAGGTTCTATAGGACTTGTAAATTG

GTTGGGATCAATATGAGTAAAAAGAAATCCTATATAAATCGGACAGGAACATTTGAATTC

ACAAGCTTTTTCTACCGTTATGGGTTTGTAGCCAACTTCAGCATGGAGCTGCCCAGCTTT

GGAGTTTCTGGGATTAATGAATCGGCTGACATGAGCATTGGAGTTACAGTAATAAAGAAT

AACATGATAAACAACGATCTTGGACCAGCAACAGCTCAAATGGCTCTTCAGCTATTTATC

AAGGACTACAGATATACATATCGATGCCACAGGGGTGATACACAAATACAAACGAGGAGA

TCATTCGAGCTAAAGAAGCTGTGGGAGCAGACCCGTTCAAAGGCAGGACTGTTGGTTTCA

GATGGAGGCCCAAATTTATACAATATACGGAATCTCCACATCCCAGAGGTCTGCTTGAAG

TGGGAACTGATGGATGAAGATTACCAGGGTAGACTTTGTAATCCCCTGAACCCCTTTGTC

AGTCATAAGGAAATTGAGTCCGTAAACAATGCTGTAGTGATGCCAGCCCATGGTCCGGCC

AAAAGCATGGAATATGATGCTGTTGCGACCACACACTCATGGGTCCCTAAGAGGAACCGT

TCCATTCTGAATACCAGTCAAAGAGGAATCCTTGAGGATGAACAGATGTATCAGAAGTGC

TGCAATCTATTTGAAAAATTCTTCCCTAGTAGCTCATACAGGAGGCCAGTTGGAATCTCC

AGTATGGTGGAGGCCATGGTGTCTAGGGCCCGAATTGATGCACGGATTGACTTCGAGTCT

GGTAGGATTAAGAAGGAAGAGTTTGCTGAGATCATGAAGATCTGTTCCACCATTGAAGAG

ATCAGACGGCAAAAACAGTGAATTTAGCTTGTCCTTCATGAAA-----------------

-

>A_goose_Taiwan_TNO15_2015_EPI690742

------------------------ATGGATGTCAATCCGACTTTACTTTTCTTAAAAGTG

CCAGCGCAAAATGCTATAAGCACTACATTCCCTTACACTGGAGATCCTCCATACAGCCAT

GGAACAGGAACAGGATATACCATGGACACAGTCAACAGAACACATCAATACTCAGAGAAG

GGAAAGTGGACAACAAACACAGAGACCGGAGCACCCCAACTCAACCCAATTGATGGACCA

TTACCGGAGGACAATGAGCCAAGCGGATATGCACAAACAGACTGCGTGTTGGAAGCAATG

GCTTTCCTTGAAGAATCCCACCCAGGGATCTTTGAAAACTCTTGTCTTGAAACGATGGAA

GTCGTTCAGCAAACAAGAGTGGACAAACTAACCCAAGGTCGCCAGACTTATGACTGGACA

CTGAATAGAAACCAACCGGCTGCAACTGCTTTGGCCAACACTATAGAAGTCTTCAGATCG

AATGGTCTGGCAGCCAATGAATCGGGGAGACTAATAGATTTCCTCAAGGATGTGATGGAG

TCAATGGATAAAGAAGAAATGGAAATAACAACACATTTCCAGAGAAAGAGAAGGGTAAGA

GACAACATGACCAAGAAGATGGTCACACAAAGAACAATAGGGAAGAAGAAGCAAAGGCTA

AACAAGAGGAGCTATTTGATAAGAGCACTGACACTGAACACAATGACAAAAGATGCAGAA

CGAGGCAAATTGAAGAGGCGGGCAATTGCAACACCCGGGATGCAGATTAGAGGATTCGTG

TATTTTGTCGAAACACTAGCGAGGAGCATCTGTGAGAAACTCGAGCAATCTGGGCTCCCT

GTTGGAGGGAATGAGAAGAAGGCTAAATTGGCAAATGTCGTGAGAAAAATGATGACTAAC

TCACAAGATACAGAGCTTTCCTTCACAATCACAGGAGACAACACCAAATGGAATGAGAAT

CAAAATCCTCGGATGTTTTTGGCAATGATAACATACATCACAAGGAATCAACCTGAATGG

TTTAGGAATGTCTTGAGCATTGCCCCTATAATGTTCTCAAACAAAATGGCAAGATTAGGG

AAAGGATACATGTTTGAAAGTAAGAGCATGAAGCTACGGACACAAATACCGGCAGAAATG

CTTGCAAACATTGACTTGAAATACTTCAACGAATCCACGAGAAAGAAAATCGAGAAAATA

AGACCTCTGCTGATCGATGGCACAGCCTCATTGAGTCCTGGAATGATGATGGGCATGTTC

AACATGCTGAGCACAGTACTAGGGGTCTCAATCCTGAATCTTGGACAAAAGAGGTACACC

AAAACCACATACTGGTGGGATGGACTTCAATCCTCTGATGATTTCGCTCTCATAGTGAAT

GCGCCGAATCATGAGGGGATACAAGCAGGAGTGGATAGGTTCTATAGGACTTGCAAACTG

GTTGGGATCAACATGAGCAAAAAGAAGTCTTACATAAACCGGACAGGGACATTTGAGTTC

ACAAGCTTTTTCTATCGCTACGGATTTGTAGCCAACTTCAGTATGGAGCTACCCAGCTTT

GGAGTATCTGGGATCAATGAATCGGCTGACATGAGCATTGGAGTCACAGTGATAAAAAAC

AATATGATAAACAATGATCTTGGACCAGCAACAGCTCAGATGGCTCTCCAACTATTCATC

AAAGACTATAGGTACACATACCGATGCCACAGGGGTGATACACAAATTCAAACGAGGAGA

TCATTCGAGCTGAAGAAGCTGTGGGAGCAGACCCGTTCAAAGGCAGGGCTGTTGGTATCA

GATGGAGGGCCAAATCTATACAACATTCGGAATCTCCACATCCCAGAGGTCTGCTTGAAG

TGGGAACTGATGGATGAAGATTACCAGGGCAGGCTGTGTAATCCTCAGAACCCGTTTGTT

AGTCATAAGGAAATTGAGTCCGTAAACAATGCTGTGGTAATGCCAGCCCATGGTCCGGCC

AAGAGCATGGAATATGATGCTGTTGCGACTACACATTCATGGATTCCTAAGAGGAACCGT

TCCATTCTCAATACCAGCCAAAGGGGAATCCTTGAGGATGAGCAGATGTACCAGAAGTGC

TGCAGTCTATTCGAGAAATTCTTCCCCAGTAGTTCATACAGGAGGCCAGTTGGAATTTCC

AGCATGGTGGAGGCCATGGTGTCTAGGGCCCGAATTGATGCACGCATTGATTTCGAATCT

GGAAGGATCAAGAAAGAAGAGTTTGCTGAGATCATGAAGATCTGTTCCACCATTGAAGAG

CTCAGACGGCAAAAATAGTAG---------------------------------------

-

>A_wigeon_Sakha_1_2014_EPI1201484

AGCGAAAGCAGGCAAACTATTTGAATGGATGTCAACCCGACTTTACTCTTCTTGAAAGTG

CCAGCGCAAAATGCTATAAGTACCACATTCCCTTATACTGGAGATCCTCCATACAGCCAT

GGAACAGGAACAGGATACACCATGGACACAGTCAACAGAACGCATCAATACTCAGAAAAG

GGAAAGTGGACAAAAAACACCGAGACTGGAGCACCCCAACTCAACCCAATTGATGGACCA

TTACCTGAGGATAACGAGCCAAGCGGATATGCACAAACGGATTGTGTGTTGGAAGCAATG

GCTTTCCTTGAAGAGTCCCACCCAGGGATCTTTGAAAACTCATGTCTTGAAACAATGGAA

ATTGTTCAACAAACAAGAGTGGACAAACTGACCCAAGGTCGTCAGACCTATGACTGGACA

TTGAATAGAAACCAGCCGGCTGCAACTGCTTTAGCCAACACTATAGAAGTCTTCAGATCG

AACGGTCTAACAGCCAATGAGTCAGGGAGACTGATAGATTTCCTCAAAGATGTGATGGAG

TCAATGGACAAAGAAGAAATGGAAATAACAACACATTTCCAAAGAAAGAGAAGAGTAAGA

GACAATATGACCAAGAAAATGGTCACACAAAGAACAATAGGGAAGAAAAAACAGAGACTG

AACAAGAAGAACTACTTGGTAAGGGCACTGACACTGAACACAATGACAAAAGATGCAGAA

AGAGGCAAGTTGAAGAGGCGGGCAATTGCAACACCCGGGATGCAAATCAGAGGGTTCGTG

TACTTTGTCGAAACATTAGCGAGGAGCATCTGCGAGAAACTTGAGCAATCTGGGCTCCCT

GTTGGAGGAAATGAAAAAAAGGCTAAGTTGGCAAATGTCGTGAGAAAGATGATGACTAAC

TCACAAGACACAGAGCTATCCTTTACAATTACTGGAGACAATACCAAGTGGAACGAGAAT

CAGAATCCTCGGATTTTTTTGGCAATGATAACATATATCACAAGAAATCAACCTGAGTGG

TTTAGAAATGTGTTAAGTATTGCCCCTATAATGTTCTCAAACAAAATGGCAAGATTAGGG

AAAGGATACATGTTCGAAAGTAAGAGCATGAAGCTACGGACACAAATACCAGCAGAAATG

CTTGCAACCATTGACCTGAAATATTTCAACGAATCGACAAGAAAGAAAATTGAGAAAATA

AGGCCTCTCCTAATAGAAGGGACAGCCTCGTTGAGTCCTGGAATGATGATGGGCATGTTC

AACATGCTGAGTACAGTCTTGGGAGTATCAATTCTAAATCTTGGCCAAAAGAGGTACACC

AAAACCACATACTGGTGGGACGGACTCCAATCCTCTGATGATTTCGCTCTCATAGTAAAT

GCACCGAATCATGAGGGAATACAGGCAGGAGTGGACAGGTTCTATAGGACTTGTAAATTG

GTTGGGATCAATATGAGTAAAAAGAAATCCTATATAAATCGGACAGGAACATTTGAATTC

ACAAGCTTTTTCTACCGTTATGGGTTTGTAGCCAACTTCAGCATGGAGCTGCCCAGCTTT

GGAGTTTCTGGGATTAATGAATCGGCTGACATGAGCATTGGAGTTACAGTAATAAAGAAT

AACATGATAAACAACGATCTTGGACCAGCAACAGCTCAAATGGCTCTTCAGCTATTTATC

AAGGACTACAGATATACATATCGATGCCACAGGGGTGATACACAAATACAAACAAGGAGA

TCATTCGAGCTAAAGAAGCTGTGGGAGCAGACCCGTTCAAAGGCAGGACTGTTGGTTTCA

GATGGAGGCCCAAACTTATACAATATACGGAATCTCCACATCCCAGAGGTCTGCTTGAAG

TGGGAACTGATGGATGAAGATTACCAGGGTAGACTTTGTAATCCCCTGAACCCCTTTGTC

AGTCATAAGGAAATTGAATCCGTAAACAATGCTGTAGTGATGCCAGCCCATGGTCCGGCC

AAAAGCATGGAATATGATGCTGTTGCGACCACACACTCATGGGTCCCTAAGAGGAACCGT

TCCATTCTGAATACCAGTCAAAGAGGAATCCTTGAGGATGAACAGATGTATCAGAAGTGC

TGCAATCTATTTGAAAAATTCTTCCCTAGTAGCTCATACAGGAGGCCAGTTGGAATCTCC

AGTATGGTGGAGGCCATGGTGTCTAGGGCCCGAATTGATGCACGGATTGACTTCGAGTCT

GGTAGGATTAAGAAGGAAGAGTTTGCTGAGATCATGAAGATCTGTTCCACCATTGAAGAG

ATCAGACGGCAAAAACAGTGAATTTAGCTTGTCCTTCATGAAAAAATGCCTTGTTTCTAC

T

>A_duck_Nigeria_SK28T_19VIR8424-2_2019_EPI1777112

AGCGAAAGCAGGCAAACCATTTGAATGGATGTCAATCCGACTTTACTTTTCTTAAAAGTG

CCAGCGCAAAATGCTATAAGTGCTACATTCCCTTACACTGGAGACCCTCCATACAGCCAT

GGAACAGGAACAGGATATACCATGGACACAGTAAACAGAACACATCAATACTCAGAAAAG

GGAAAGTGGACAGCAAACACAGAAACCGGGGCACCCCAACTCAACCCAATTGATGGACCA

TTACCAGAGGACAATGAGCCAAGCGGGTATGCACAAACTGATTGCGTGTTGGAAGCAATG

GCTTTCCTTGAGGAATCCCATCCAGGGATATTTGAAAACTCTTGTCTTGAAGCGATGGAA

GTCGTTCAGCAAACAAGAGTGGACAAACTAACCCAAGGTCGCCAGACTTATGACTGGACA

CTGAACAGAAACCAACCAGCTGCAACTGCTCTGGCCAACACTATAGAGGTGTTCAGATCG

AATGGTCTGACAGCCAGTGAATCAGGGAGACTGATAGATTTTCTCAGGGATGTGATGGAA

TCAATGAATAAAGAAGAGATGGAAATAACAACACATTTCCAGAGAAAAAGAAGAGTGAGG

GACAACATCACCAAGAAGATGGTCACACAAAGAACAATAGGAAAGAAGAAGCAGAGGCTG

AACAAGAGGAGTTACTTAATAAGAGCACTGACATTGAACACAATGACCAAAGATGCAGAA

AGAGGCAAATTAAAGAGACGGGCAATTGCAACACCCGGAATGCAGATTAGAGGATTCGTG

TACTTTGTCGAAACACTAGCGAGGAGCATCTGTGAGAAACTCGAGCAATCTGGGCTCCCT

GTTGGAGGGAATGAGAAGAAGGCTAAATTGGCAAATGTCGTGAGAAAAATGATGACTAAC

TCACAAGATACAGAGCTCTCCTTCACAATTACTGGTGACAACACCAAATGGAATGAGAAT

CAAAACCCTCGGATGTTTTTGGCAATGATAACATACATCACAAGAAACCAACCTGAATGG

TTTAGAAATGTCTTAAGCATTGCCCCTATAATGTTCTCAAACAAAATGGCGAAGTTAGGG

AAAGGATACATGTTTGAAAGTAAGAGCATGAAACTAAGGACACAGATACCGGCAGAAATG

CTTACAAACATTGATCTGAAATATTTCAACGAATCAACGAGAAAGAAAATCGAGAAAATA

AGACCTCTGCTGATTGATGGCACGGCCTCATTGAGTCCTGGGATGATGATGGGCATGTTC

AATATGCTGAGCACAGTATTAGGGGTCTCAATCCTGAATCTCGGACAAAAGAGGTACACC

AAAACCACATACTGGTGGGATGGACTTCAATCCTCTGATGATTTCGCTCTCATAGTGAAT

GCACCGAATCATGAGGGGATACAAGCAGGAGTGGATAGGTTCTATAGGACCTGCAAACTG

GTTGGGATCAACATGAGCAAAAAGAAATCTTACATAAACCGAACAGGAACATTTGAGTTC

ACAAGTTTTTTCTACCGCTATGGATTTGTAGCTAACTTCAGTATGGAATTACCCAGCTTT

GGAGTATCTGGAATCAATGAATCGGCTGACATGAGCATTGGAGTTACAGTGATAAAAANC

AATATGATAAACAATGACCTTGGACCAGCAACAGCTCAAATGGCTCTTCAGTTATTCATC

AAAGACTATAGGTACACGTACCGATGCCACAGGGGTGATGCACAAATTCAAACGAGGAGA

TCATTCGAGCTGAAGAAGCTGTGGGAGCAGACTCGTTCAAAGGCAGGGCTGTTGGTATCA

GACGGAGGGCCAAACCTATACAATATTCGGAATCTCCACATCCCAGAGGTCTGCTTGAAG

TGGGAACTGATGGATGAAGACTACCAAGGCAGGCTGTGCAACCCTCTGAACCCATTTGTC

AGTCATAAAGAGATTGAGTCCGTAAACAATGCTGTAGTAATGCCAGCCCATGGCCCGGCC

AAGAGCATGGAATATGATGCTGTTGCGACTACACACTCGTGGATTCCTAAAAGGAACCGT

TCCATTCTCAATACCAGCCAGAGGGGAATTCTTGAGGATGAGCAAATGTACCAAAAGTGC

TGTAGTCTATTCGAGAAATTCTTCCCCAGCAGTTCATACAGGAGGCCAGTTGGAATTTCC

AGCATGGTGGAGGCCATGGTGTCTAGGGCCCGAATTGATGCACGCATCGATTTCGAATCT

GGAAGGATTAAGAAAGAAGAGTTTGCTGAGATCATGAAGATCTGTTCCACCATTGAAGAG

CTCAGACGGCAAAAATAGTGAATTTAGCTTGTCCTTCATGAAAAAATGCCTTGTTTCTAC

T

>A_mute_swan_Kazakhstan_1-267-20-B_2020_EPI1811581

--TGATAGCAGGCAAACTATTTGAATGGATGTCAATCCGACTTTACTTTTCTTAAAAGTG

CCAGCGCAAAATGCTATAAGTACTACATTCCCTTACACTGGAGATCCCCCATACAGCCAT

GGAACAGGAACAGGGTATACCATGGACACAGTAAACAGAACACATCAATACTCAGAAAAG

GGAAAGTGGACAACAAACACAGAAACCGGAGCACCCCAACTCAACCCAATTGATGGACCA

TTACCAGAGGACAATGAGCCAATCGGATATGCACAAACTGATTGCGTGTTGGAAGCAATG

GCTTTCCTTGAAGAATCCCACCCAGGGATATTTGAAAACTCTTGTCTTGAAGCGATGGAA

ATCGTTCAGCAAACAAGAGTGGACAAACTAACCCAAGGTCGCCAGACTTATGACTGGACA

CTGAACAGAAACCAACCAGCTGCAACCTCTTTGGCCAACACTATAGAGGTGTTCAGATCG

AATGGTCTGACAGCCAATGAATCAGGGAGACTGATAGATTTTCTCAGGGATGTGATGGAA

TCAATGGATAAAGAAGAGATGGAAGTAACAACACATTTCCAGAGAAAAAGAAGAGTGAGG

GACAACATGACTAAGAAGATGGTCACACAAAGAACAATAGGGAAGAAGAAGCAGAGGCTG

AACAAGAGGAGTTACTTAATAAGAGCACTGACATTGAATACAATGACCAAAGATGCAGAA

AGAGGCAAGTTGAAGAGACGGGCAATTGCAACACCCGGGATGCAGATTAGAGGATTCGTG

TACTTCGTTGAAACACTAGCGAGGAGCATCTGTGAGAAACTAGAGCAATCTGGGCTCCCT

GTTGGAGGGAATGAGAAGAAGGCTAAATTGGCAAATGTTGTGAGAAAAATGATGACTAAC

TCACAAGATACAGAGCTCTCCTTTACAATTACTGGAGACAACACCAAATGGAATGAGAAT

CAAAACCCTCGGATGTTTTTGGCAATGATAACATATATCACAAGAAACCAACCTGAATGG

TTTAGAAATGTCTTAAGCATTGCCCCTATAATGTTCTCAAACAAAATGGCGAGATTAGGG

AAAGGATACATGTTTGAAAGTAAGAGCATGAAGCTAAGAACACAAATACCAGCAGAGATG

CTTACAAATATTGATCTGAAGTATTTCAACGAACCAACGAGAAAGAAAATCGAGAAAATA

AGACCTCTGCTGATTGATGGCACGGCCTCATTGAGTCCTGGGATGATGATGGGCATGTTC

AATATGCTGAGCACAGTATTAGGGGTCTCAATCCTGAATCTCGGGCAAAAGAGGTACACC

AAAACCACATACTGGTGGGATGGACTTCAATCCTCTGATGATTTCGCTCTCATAGTGAAT

GCACCGAATCATGAGGGGATACAAGCAGGAGTGGATAGATTCTATAGGACCTGCAAACTG

GTTGGGATCAACATGAGCAAAAAGAAGTCTTACATAAACCGAACAGGAACATTTGAGTTC

ACAAGTTTTTTCTACCGCTATGGATTTGTAGCTAACTTCAGTATGGAACTACCCAGCTTT

GGAGTGTCTGGAATCAATGAATCAGCTGACATGAGCATTGGAGTTACAGTGATAAAAAAC

AATATGATAAACAATGATCTTGGACCAGCAACAGCTCAAATGGCTCTTCAGTTATTCATC

AAAGACTATAGGTACACATACCGATGCCACAGGGGTGATACACAAATTCAAACGAGGAGA

TCATTCGAGCTGAAGAAGCTGTGGGAGCAGACCCGTTCAAAGGCAGGGCTGTTGATATCA

GACGGGGGGCCAAACCTATACAACATTCGGAATCTCCACATCCCAGAGGTCTGCTTGAAG

TGGGAGCTGATGGATGAAGACTACCAAGGCAGGCTGTGCAATCCTCTGAATCCATTTGTC

AGTCATAAAGAGATTGAGTCCGTAAACAATGCTGTAGTAATGCCCGCCCATGGCCCGGCC

AAGAGCATGGAATATGATGCTGTTGCGACCACACACTCGTGGATTCCTAAGAGGAACCGT

TCCATTCTCAATACCAGCCAAAGGGGAATTCTTGAGGATGAGCAGATGTACCAAAAGTGC

TGTAGTCTATTCGAGAAATTCTTCCCCAGCAGTTCATACAGGAGGCCAGTTGGAATTTCC

AGCATGGTGGAGGCCATGGTGTCTAGGGCCCGAATTGATGCACGCATCGACTTCGAATCT

GGAAGGATTAAGAAAGAAGAGTTTGCTGAGATCATGAAGATCTGTTCCACCATTGAAGAG

CTCAGACGGCAAAAATAGTGAATTTAGCTTGTCCTTCATGAAAAAATGCCTTGTTTC---

-

>A_domestic_goose_Kazakhstan_1-248_2-20-B_2020_EPI1811599

----------GGCGAACTATTTGAATGGATGTCAATCCGACTTTACTTTTCTTAAAAGTG

CCAGCGCAAAATGCTATAAGTACTACATTCCCTTACACTGGAGATCCCCCATACAGCCAT

GGAACAGGAACAGGGTATACCATGGACACAGTAAACAGAACACATCAATACTCAGAAAAG

GGAAAGTGGACAACAAACACAGAAACCGGAGCACCCCAACTCAACCCAATTGATGGACCA

TTACCAGAGGACAATGAGCCAAGCGGATATGCACAAACTGATTGCGTGTTGGAAGCAATG

GCTTTCCTTGAAGAATCCCACCCAGGGATATTTGAAAACTCTTGTCTTGAAGCGATGGAA

ATCGTTCAGCAAACAAGAGTGGACAAACTAACCCAAGGTCGCCAGACTTATGACTGGACA

CTGAACAGAAACCAACCAGCTGCAACCTCTTTGGCCAACACTATAGAGGTGTTCAGATCG

AATGGTCTGACAGCCAATGAATCAGGGAGACTGATAGATTTTCTCAGGGATGTGATGGAA

TCAATGGATAAAGAAGAGATGGAAGTAACAACACATTTCCAGAGAAAAAGAAGAGTGAGG

GACAACATGACTAAGAAGATGGTCACACAAAGAACAATAGGGAAGAAGAAGCAGAGGCTG

AACAAGAGGAGTTACTTAATAAGAGCACTGACATTGAATACAATGACCAAAGATGCAGAA

AGAGGCAAGTTGAAGAGACGGGCAATTGCAACACCCGGGATGCAGATTAGAGGATTCGTG

TACTTCGTTGAAACACTAGCGAGGAGCATCTGTGAGAAACTAGAGCAATCTGGGCTCCCT

GTTGGAGGGAATGAGAAGAAGGCTAAATTGGCAAATGTTGTGAGAAAAATGATGACTAAC

TCACAAGATACAGAGCTCTCCTTTACAATTACTGGAGACAACACCAAATGGAATGAGAAT

CAAAACCCTCGGATGTTTTTGGCAATGATAACATATATCACAAGAAACCAACCTGAATGG

TTTAGAAATGTCTTAAGCATTGCCCCTATAATGTTCTCAAACAAAATGGCGAGATTAGGG

AAAGGATACATGTTTGAAAGTAAGAGCATGAAGCTAAGAACACAAATACCAGCAGAGATG

CTTACAAATATTGATCTGAAGTATTTCAACGAACCAACGAGAAAGAAAATCGAGAAAATA

AGACCTCTGCTGATTGATGGCACGGCCTCATTGAGTCCTGGGATGATGATGGGCATGTTC

AATATGCTGAGCACAGTATTAGGGGTCTCAATCCTGAATCTCGGGCAAAAGAGGTACACC

AAAACCACATACTGGTGGGATGGACTTCAATCCTCTGATGATTTCGCTCTCATAGTGAAT

GCACCGAATCATGAGGGGATACAAGCAGGAGTGGATAGATTCTATAGGACCTGCAAACTG

GTTGGGATCAACATGAGCAAAAAGAAGTCTTACATAAACCGAACAGGAACATTTGAGTTC

ACAAGTTTTTTCTACCGCTATGGATTTGTAGCCAACTTCAGTATGGAACTACCCAGCTTT

GGAGTGTCTGGAATCAATGAATCAGCTGACATGAGCATTGGAGTTACAGTGATAAAAAAC

AATATGATAAACAATGATCTTGGACCAGCAACAGCTCAAATGGCTCTTCAGTTATTCATC

AAAGACTATAGGTACACATACCGATGCCACAGGGGTGATACACAAATTCAAACGAGGAGA

TCATTCGAGCTGAAGAAGCTGTGGGAGCAGACCCGTTCAAAGGCAGGGCTGTTGATATCA

GACGGGGGGCCAAACCTATACAACATTCGGAATCTCCACATCCCAGAGGTCTGCTTGAAG

TGGGAGCTGATGGATGAAGACTACCAAGGCAGGCTGTGCAATCCTCTGAATCCATTTGTC

AGTCATAAAGAGATTGAGTCCGTAAACAATGCTATAGTAATGCCCGCCCATGGCCCGGCC

AAGAGCATGGAATATGATGCTGTTGCGACCACACACTCGTGGATTCCTAAGAGGAACCGT

TCCATTCTCAATACCAGCCAAAGGGGAATTCTTGAGGATGAGCAGATGTACCAAAAGTGC

TGTAGTCTATTCGAGAAATTCTTCCCCAGCAGTTCATACAGGAGGCCAGTTGGAATTTCC

AGCATGGTGGAGGCCATGGTGTCTAGGGCCCGAATTGATGCACGCATCGACTTCGAATCT

GGAAGGATTAAGAAAGAAGAGTTTGCTGAGATCATGAAGATCTGTTCCACCATTGAAGAG

CTCAGACGGCAAAAGTAGTGTATTTAGCTTGTCCTTCATGA-------------------

-

>A_domestic_duck_Kazakhstan_1-274-20-B_2020_EPI1811609

AGCGAAAGCAGGCAAACTATTTGAATGGATGTCAATCCGACTTTACTTTTCTTAAAAGTG

CCAGCGCAAAATGCTATAAGTACTACATTCCCTTACACTGGAGATCCTCCATACAGCCAT

GGAACAGGAACAGGGTATACCATGGACACAGTAAACAGAACACATCAATACTCAGAAAAG

GGAAAGTGGACAACAAACACAGAAACCGGAGCACCCCAACTCAACCCAATTGATGGACCA

TTACCAGAGGACAATGAGCCAAGCGGATATGCACAAACTGATTGCGTGTTGGAAGCAATG

GCTTTCCTTGAAGAATCCCACCCAGGGATATTTGAAAACTCTTGTCTTGAAGCGATGGAA

ATCGTTCAGCAAACAAGAGTGGACAAACTAACCCAAGGTCGCCAGACTTATGACTGGACA

CTGAACAGAAACCAACCAGCTGCAACCTCTTTGGCCAACACTATAGAGGTGTTCAGATCG

AATGGTCTGACAGCCAATGAATCAGGGAGACTGATAGATTTTCTCAGGGATGTGATGGAA

TCAATGGATAAAGAAGAGATGGAAGTAACAACACATTTCCAGAGAAAAAGAAGAGTGAGG

GACAACATGACTAAGAAGATGGTCACACAAAGAACAATAGGGAAGAAGAAGCAGAGGCTG

AACAAGAGGAGTTACTTAATAAGAGCACTGACATTGAATACAATGACCAAAGATGCAGAA

AGAGGCAAATTGAAGAGACGGGCAATTGCAACACCCGGGATGCAGATTAGAGGATTCGTG

TACTTCGTTGAAACACTAGCGAGGAGCATCTGTGAGAAACTAGAGCAATCTGGGCTCCCT

GTTGGAGGGAATGAGAAGAAGGCTAAATTGGCAAATGTTGTGAGAAAAATGATGACTAAC

TCACAAGATACAGAGCTCTCCTTTACAATTACTGGAGACAACACCAAATGGAATGAGAAT

CAAAACCCTCGGATGTTTTTGGCAATGATAACATATATCACAAGAAACCAACCTGAATGG

TTTAGAAATGTCTTAAGCATTGCCCCTATAATGTTCTCAAACAAAATGGCGAGATTAGGG

AAAGGATACATGTTTGAAAGTAAGAGCATGAAGCTAAGAACACAAATACCAGCAGAGATG

CTTACAAATATTGATCTGAAGTATTTCAACGAACCAACGAGAAAGAAAATCGAGAAAATA

AGACCTCTGCTGATTGATGGCACGGCCTCATTGAGTCCTGGGATGATGATGGGCATGTTC

AATATGCTGAGCACAGTATTGGGGGTCTCAATCCTGAATCTCGGGCAAAAGAGGTACACC

AAAACCACATACTGGTGGGATGGACTTCAATCCTCTGATGATTTCGCTCTCATAGTGAAT

GCACCGAATCATGAGGGGATACAAGCAGGAGTGGATAGATTCTATAGGACCTGCAAACTG

GTTGGAATCAACATGAGCAAAAAGAAGTCTTACATAAACCGAACAGGAACATTTGAGTTC

ACAAGTTTTTTCTACCGCTACGGATTTGTAGCTAACTTCAGTATGGAATTACCCAGCTTT

GGAGTGTCTGGAATCAATGAATCAGCTGACATGAGCATTGGAGTTACAGTGATAAAAAAC

AATATGATAAACAATGATCTTGGACCAGCGACAGCTCAAATGGCTCTTCAGTTATTCATC

AAAGACTATAGGTACACATACCGATGCCACAGGGGTGATACACAAATTCAAACGAGGAGA

TCATTCGAGCTGAAGAAGCTGTGGGAGCAGACCCGTTCAAAGGCAGGGCTGTTGATATCA

GACGGGGGGCCAAACCTATACAACATTCGGAACCTCCACATCCCAGAGGTCTGCTTGAAG

TGGGAGCTGATGGATGAAGACTACCAAGGCAGGCTGTGCAATCCTCTGAATCCATTTGTC

AGTCATAAAGAGATTGAGTCCGTAAACAATGCTGTAGTAATGCCCGCCCATGGCCCGGCC

AAGAGCATGGAATATGATGCTGTTGCGACCACACATTCGTGGATTCCTAAGAGGAACCGT

TCCATTCTCAATACCAGCCAAAGGGGAATTCTTGAGGATGAGCAGATGTACCAAAAGTGC

TGTAGTCTATTCGAGAAATTCTTCCCCAGCAGTTCATACAGGAGGCCAGTTGGAATTTCC

AGCATGGTGGAGGCCATGGTGTCTAGGGCCCGAATTGATGCACGCATCGACTTCGAATCT

GGAAGGATTAAGAAAGAAGAGTTTGCTGAGATCATGAAGATCTGTTCCACCATTGAAGAG

CTCAGACGGCAAAAATAGTGAATTTAGCTTGTCCTTCATGAAAAAAT-------------

-

>A_domestic_goose_Kazakhstan_1-242_2-20-B_2020_EPI1811617

------------------------ATGGATGTCAATYCGACTTTACYTTTCTTAAAAGTG

CCAGCGCAAAATGCTATAAGTACTACATTCCCTTACACTGGAGATCCTCCATACAGCCAT

GGAACAGGAACAGGGTATACCATGGACACAGTAAACAGAACACATCAATACTCAGAAAAG

GGAAAGTGGACAACAAACACAGAAACAGGAGCACCCCAACTCAACCCAATTGATGGACCA

TTACCAGAGGACAATGAGCCAAGCGGATATGCACAAACTGATTGCGTGTTGGAAGCAATG

GCTTTCCTTGAAGAATCCCACCCAGGGATATTTGAAAACTCTTGTCTTGAAGCGATGGAA

ATCGTTCAGCAAACAAGAGTGGACAAANTAACCCAAGGTCGCCAGACTTATGACTGGACA

CTGAATAGAAACCAACCAGCTGCAACCTCTTTGGCCAACACTATAGAGGTGTTCAGATCG

AATGGTCTGACAGCCAATGAATCAGGGAGACTGATAGATTTTCTCAGGGATGTGATGGAA

TCAATGGATAAAGAAGAGATGGAAGTAACAACACATTTCCAGAGAAAAAGAAGAGTGAGG

GACAACATGACTAAGAAGATGGTCACACAAAGAACAATAGGGAAGAAGAAGCAGAGGCTG

AACAAGAGGAGTTACTTAATAAGAGCACTGACATTGAATACAATGACCAAAGATGCAGAA

AGAGGCAAGTTGAAGAGACGGGCAATTGCAACACCCGGGATGCAGATTAGAGGATTCGTG

TACTTCGTTGAAACACTAGCGAGGAGCATCTGTGAGAAACTAGAGCAATCTGGGCTCCCT

GTTGGAGGGAATGAGAAGAAGGCTAAATTGGCAAATGTTGTGAGAAAAATGATGACTAAC

TCACAAGATACAGAGCTCTCCTTTACAATTACTGGAGACAACACCAAATGGAATGAGAAT

CAAAACCCTCGGATGTTTTTGGCAATGATAACATAYATCACAAGAAACCAACCTGAATGG

TTTAGAAATGTCTTAAGCATTGCCCCTATAATGTTCTCAAACAAAATGGCGAGATTAGGG

AAAGGATACATGTTTGAAAGTAAGAGCATGAAGCTAAGAACACAAATACCAGCAGAGATG

CTTACAAATATTGATCTGAAGTATTTCAACGAACCAACGAGAAAGAAAATCGAGAAAATA

AGACCTCTGCTGATTGATGGCACGGCCTCATTGAGTCCTGGGATGATGATGGGCATGTTC

AATATGCTGAGCACAGTATTGGGGGTCTCAATCCTGAATCTCGGGCAAAAGAGGTACACC

AAAACCACATACTGGTGGGATGGACTTCAATCCTCTGATGATTTCGCTCTCATAGTGAAT

GCACCGAATCATGAGGGGATACAAGCAGGAGTGGATAGATTCTATAGGACCTGCAAACTG

GTTGGGATCAACATGAGCAAAAAGAAGTCTTACATAAACCGAACAGGAACATTTGAGTTC

ACAAGTTTTTTCTACCGCTATGGATTTGTAGCTAACTTCAGTATGGAATTACCCAGCTTT

GGAGTGTCTGGAATCAATGAATCAGCTGACATGAGCATTGGAGTTACAGTGATAAAAAAC

AATATGATAAACAATGATCTTGGACCAGCAACAGCTCAAATGGCTCTTCAGTTATTCATC

AAAGACTATAGGTACACATACCGATGCCACAGGGGTGATACACAAATTCAAACGAGGAGA

TCATTCGAGCTGAAGAAGCTGTGGGAGCAGACCCGTTCAAAGGCAGGGCTGTTGATATCA

GACGGGGGGCCAAACCTATACAACATTCGGAACCTCCACATCCCAGAGGTCTGCTTGAAG

TGGGAGCTGATGGATGAAGACTACCAAGGCAGGCTGTGCAATCCTCTGAATCCATTTGTC

AGTCATAAAGAGATTGAGTCCGTAAACAATGCTGTAGTAATGCCCGCCCATGGCCCGGCC

AAGAGCATGGAATATGATGCTGTTGCGACCACACATTCGTGGATTCCTAAGAGGAACCGT

TCCATTCTCAATACCAGCCAAAGGGGAATTCTTGAGGATGAGCAGATGTACCAAAAGTGC

TGTAGTCTATTCGAGAAATTCTTCCCCAGCAGTTCATACAGGAGGCCAGTTGGAATTTCC

AGCATGGTGGAGGCCATGGTATCTAGGGCCCGAATTGATGCACGCATCGACTTCGAATCT

GGAAGGATTAAGAAAGAAGAGTTTGCTGAGATCATGAAGATCTGTTCCACCATTGAAGAG

CTCAGACGGCAAAAATAG------------------------------------------

-

>A_chicken_Iraq_1_2020_EPI1811626

AGCGAAAGCAGGCAAACTATTTGAATGGATGTCAATCCGACTTTACTTTTCTTAAAAGTG

CCAGCGCAAAATGCTATAAGTACTACATTCCCTTACACTGGAGATCCTCCATACAGCCAT

GGAACAGGAACAGGGTATACCATGGACACAGTAAACAGAACACATCAATACTCAGAAAAG

GGAAAGTGGACAACAAACACAGAAACCGGAGCACCCCAACTCAACCCAATTGATGGACCA

TTACCAGAGGACAATGAGCCAAGCGGATATGCACAAACCGATTGCGTGTTGGAAGCAATG

GCTTTCCTTGAAGAATCCCACCCAGGGATATTTGAAAACTCTTGTCTTGAAGCGATGGAA

ATCGTTCAGCAAACAAGAGTGGACAAACTAACCCAAGGTCGCCAGACTTATGACTGGACA

CTGAACAGAAACCAACCAGCTGCAACCTCTTTGGCCAACACTATAGAGGTGTTCAGATCG

AATGGTCTGACAGCCAATGAATCAGGGAGACTGATAGATTTTCTCAGGGATGTGATGGAA

TCAATGGATAAAGAAGAGATGGAAATAACAACACATTTCCAGAGAAAAAGAAGAGTGAGG

GACAACATGACTAAGAAGATGGTCACACAAAGAACAATAGGGAAGAAGAAGCAGAGGCTG

AACAAGAGGAGTTACTTAATAAGAGCACTGACATTGAATACAATGACCAAAGATGCAGAA

AGAGGCAAATTGAAGAGACGGGCAATTGCAACACCCGGGATGCAGATTAGAGGATTCGTG

TACTTCGTTGAAACACTAGCGAGGAGCATCTGTGAGAAACTAGAGCAATCTGGGCTCCCT

GTTGGAGGGAATGAGAAGAAGGCTAAATTGGCAAATGTTGTGAGAAAAATGATGACTAAC

TCACAAGATACAGAGCTCTCCTTTACAATTACTGGAGACAACACCAAATGGAATGAGAAT

CAAAACCCTCGGATGTTTTTGGCAATGATAACATATATCACAAGAAACCAACCTGAATGG

TTTAGAAATGTCTTAAGCATTGCCCCTATAATGTTCTCAAACAAAATGGCGAGATTAGGG

AAAGGATACATGTTTGAAAGTAAGAGGATGAAGCTAAGAACACAAATACCAGCAGAGATG

CTTACAAATATTGATCTGAAGTATTTCAACGAACCAACGAGAAAGAAAATCGAGAAAATA

AGACCTCTGCTGATTGATGGCACGGCCTCATTGAGTCCTGGGATGATGATGGGCATGTTC

AATATGCTGAGCACAGTATTAGGGGTCTCAATCCTGAATCTCGGGCAAAAGAGGTACACC

AAAACCACATACTGGTGGGATGGACTTCAATCCTCTGATGATTTCGCTCTCATAGTGAAT

GCACCGAATCATGAGGGGATACAAGCAGGAGTGGATAGATTCTATAGGACCTGCAAACTG

GTTGGGATCAACATGAGCAAAAAGAAGTCTTACATAAACCGAACAGGAACATTTGAGTTC

ACAAGTTTTTTCTACCGCTATGGATTTGTAGCTAACTTCAGTATGGAATTACCCAGCTTT

GGAGTGTCTGGAATCAATGAATCAGCTGACATGAGCATTGGAGTTACAGTGATAAAAAAC

AATATGATAAACAATGATCTTGGACCAGCAACAGCTCAAATGGCTCTTCAGTTATTCATC

AAAGACTATAGGTACACGTACCGATGCCACAGGGGTGACACACAAATTCAAACGAGGAGA

TCATTCGAGCTGAAGAAGCTGTGGGAGCAGACCCGTTCAAAGGCTGGGCTGTTGATATCA

GACGGGGGGCCAAACCTATACAACATTCGGAATCTCCACATCCCAGAGGTCTGCTTGAAG

TGGGAGCTGATGGATGAAGACTACCAAGGCAGGCTGTGCAATCCTCTGAATCCATTTGTC

AGTCATAAAGAGATTGAGTCCGTAAACAATGCTGTTGTAATGCCCGCCCATGGCCCGGCC

AAGAGCATGGAATATGATGCTGTTGCGACCACACACTCGTGGATTCCTAAGAGGAACCGT

TCCATTCTCAATACCAGCCAAAGGGGAATTCTTGAGGATGAGCAGATGTACCAAAAGTGC

TGTAGTCTATTCGAGAAATTCTTCCCCAGCAGTTCATACAGGAGGCCAGTTGGAATTTCC

AGCATGGTGGAGGCCATGGTGTCTAGGGCCCGAATTGATGCACGCATCGACTTCGAATCT

GGAAGGATTAAGAAAGAAGAGTTTGCTGAGATCATGAAGATCTGTTCCACCATTGAAGAG

CTCAGACGGCAAAAATAGTGAATTTAGCTTGTCCTTCATGAAAAAATGCMTTGTTT----

-

>A_whooper_swan_Inner_Mongolia_w1-1_2020_EPI1811642

------------------------ATGGATGTCAATCCGACTTTACTTTTCTTAAAAGTG

CCAGCGCAAAATGCTATAAGTACTACATTCCCTTACACTGGAGATCCTCCATACAGCCAT

GGAACAGGAACAGGGTATACCATGGACACAGTAAACAGAACACATCAATACTCAGAAAAG

GGAAAGTGGACAACAAACACAGAAACCGGAGCACCCCAACTCAACCCAATTGATGGACCA

TTACCAGAGGACAATGAGCCAAGCGGATATGCACAAACTGATTGCGTGTTGGAAGCAATG

GCTTTCCTTGAAGAATCCCACCCAGGGATATTTGAAAACTCTTGTCTTGAAGCGATGGAA

ATCGTTCAGCAAACAAGAGTGGACAAACTAACCCAAGGTCGCCAGACTTATGACTGGACA

CTGAACAGAAACCAACCAGCTGCAACCTCTTTGGCCAACACTATAGAGGTGTTCAGATCG

AATGGTCTGACAGCCAATGAATCAGGGAGACTGATAGATTTTCTCAAGGATGTGATGGAA

TCAATGGATAAAGAAGAGATGGAAGTAACAACACATTTCCAGAGAAAAAGAAGAGTGAGG

GACAACATGACTAAGAAGATGGTCACACAAAGAACAATAGGGAAGAAGAAGCAGAGGCTG

AACAAGAGGAGTTACTTGATAAGAGCACTGACATTGAATACAATGACCAAAGATGCAGAA

AGAGGCAAATTGAAGAGACGGGCAATTGCAACACCCGGGATGCAGATTAGAGGATTCGTG

TACTTCGTTGAAACACTAGCGAGGAGCATCTGTGAGAAACTAGAGCAATCTGGGCTCCCT

GTTGGAGGGAATGAGAAGAAGGCTAAATTGGCAAATGTTGTGAGAAAAATGATGACTAAC

TCACAAGATACAGAGCTCTCCTTTACAATTACTGGAGACAACACCAAATGGAATGAGAAT

CAAAACCCTCGGATGTTTTTGGCAATGATAACATATATCACAAGAAACCAACCTGAATGG

TTTAGAAATGTCTTAAGCATTGCCCCTATAATGTTCTCAAACAAAATGGCGAGATTAGGG

AAAGGATACATGTTTGAAAGTAAGAGCATGAAGCTAAGAACACAAATACCAGCAGAGATG

CTTACAAATATTGATCTGAAGTATTTCAACGAACCAACGAGAAAGAAAATCGAGAAAATA

AGACCTCTGCTGATTGATGGCACGGCCTCATTGAGTCCTGGGATGATGATGGGCATGTTC

AATATGCTGAGCACAGTATTGGGGGTCTCAATCCTGAATCTCGGGCAAAAGAGGTACACC

AAAACCACATACTGGTGGGATGGACTTCAATCCTCTGATGATTTCGCTCTCATAGTGAAT

GCACCGAATCATGAGGGGATACAAGCAGGAGTGGATAGATTCTATAGGACCTGCAAACTG

GTTGGGATCAACATGAGCAAAAAGAAGTCTTACATAAACCGAACAGGAACATTTGAGTTC

ACAAGTTTTTTCTACCGCTATGGATTTGTAGCTAACTTCAGTATGGAATTACCCAGCTTT

GGAGTGTCTGGAATCAATGAATCAGCTGACATGAGCATTGGAGTTACAGTGATAAAAAAC

AATATGATAAACAATGATCTTGGACCAGCAACAGCTCAAATGGCTCTTCAGTTATTCATC

AAAGACTATAGGTACACATACCGATGCCACAGGGGTGATACACAAATTCAAACGAGGAGA

TCATTCGAGCTGAAGAAGCTGTGGGAGCAGACCCGGTCAAAGGCAGGGCTGTTGATATCA

GACGGGGGGCCAAACCTATACAACATTCGGAACCTCCACATCCCAGAGGTCTGCTTGAAG

TGGGAGCTGATGGATGAAGACTACCAAGGCAGGCTGTGCAACCCTCTGAATCCATTTGTC

AGTCATAAAGAGATTGAGTCCGTAAACAATGCTGTAGTAATGCCCGCCCATGGCCCGGCC

AAGAGCATGGAATATGATGCTGTTGCGACCACACATTCGTGGATTCCTAAGAGGAACCGC

TCCATTCTCAATACCAGCCAAAGGGGAATTCTTGAGGATGAGCAGATGTACCAAAAGTGC

TGTAGTCTATTCGAGAAATTCTTCCCCAGCAGTTCATACAGGAGGCCAGTTGGAATTTCC

AGCATGGTGGAGGCCATGGTGTCTAGGGCCCGAATTGATGCACGCATCGACTTCGAATCT

GGCAGGATTAAGAAAGAAGAGTTTGCTGAGATCATGAAGATCTGTTCCACCATTGAAGAG

CTCAGACGGCAAAAATAG------------------------------------------

-

>A_mute_swan_Inner_Mongolia_w2-1_2020_EPI1811650

------------------------ATGGATGTCAATCCGACTTTACTTTTCTTAAAAGTG

CCAGCGCAAAATGCTATAAGTACTACATTCCCTTACACTGGAGATCCTCCATACAGCCAT

GGAACAGGAACAGGGTATACCATGGACACAGTAAACAGAACACATCAATACTCAGAAAAG

GGAAAGTGGACAACAAACACAGAAACCGGAGCACCCCAACTCAACCCAATTGATGGACCA

TTACCAGAGGACAATGAGCCAAGCGGATATGCACAAACTGATTGCGTGTTGGAAGCAATG

GCTTTCCTTGAAGAATCCCACCCAGGGATATTTGAAAACTCTTGTCTTGAAGCGATGGAA

ATCGTTCAGCAAACAAGAGTGGACAAACTAACCCAAGGTCGCCAGACTTATGACTGGACA

CTGAACAGAAACCAACCAGCTGCAACCTCTTTGGCCAACACTATAGAGGTGTTCAGATCG

AATGGTCTGACAGCCAATGAATCAGGGAGACTGATAGATTTTCTCAAGGATGTGATGGAA

TCAATGGATAAAGAAGAGATGGAAGTAACAACACATTTCCAGAGAAAAAGAAGAGTGAGG

GACAACATGACTAAGAAGATGGTCACACAAAGAACAATAGGGAAGAAGAAGCAGAGGCTG

AACAAGAGGAGTTACTTGATAAGAGCACTGACATTGAATACAATGACCAAAGATGCAGAA

AGAGGCAAATTGAAGAGACGGGCAATTGCAACACCCGGGATGCAGATTAGAGGATTCGTG

TACTTCGTTGAAACACTAGCGAGGAGCATCTGTGAGAAACTAGAGCAATCTGGGCTCCCT

GTTGGAGGGAATGAGAAGAAGGCTAAATTGGCAAATGTTGTGAGAAAAATGATGACTAAC

TCACAAGATACAGAGCTCTCCTTTACAATTACTGGAGACAACACCAAATGGAATGAGAAT

CAAAACCCTCGGATGTTTTTGGCAATGATAACATATATCACAAGAAACCAACCTGAATGG

TTTAGAAATGTCTTAAGCATTGCCCCTATAATGTTCTCAAACAAAATGGCGAGATTAGGG

AAAGGATACATGTTTGAAAGTAAGAGCATGAAGCTAAGAACACAAATACCAGCAGAGATG

CTTACAAATATTGATCTGAAGTATTTCAACGAACCAACGAGAAAGAAAATCGAGAAAATA

AGACCTCTGCTGATTGATGGCACGGCCTCATTGAGTCCTGGGATGATGATGGGCATGTTC

AATATGCTGAGCACAGTATTGGGGGTCTCAATCCTGAATCTCGGACAAAAGAGGTACACC

AAAACCACATACTGGTGGGATGGACTTCAATCCTCTGATGATTTCGCTCTCATAGTGAAT

GCACCGAATCATGAGGGGATACAAGCAGGAGTGGATAGATTCTATAGGACCTGCAAACTG

GTTGGGATCAACATGAGCAAAAAGAAGTCTTACATAAACCGAACAGGAACATTTGAGTTC

ACAAGTTTTTTCTACCGCTATGGATTTGTAGCTAACTTCAGTATGGAATTACCCAGCTTT

GGAGTGTCTGGAATCAATGAATCAGCTGACATGAGCATTGGAGTTACAGTGATAAAAAAC

AATATGATAAACAATGATCTTGGACCAGCAACAGCTCAAATGGCTCTTCAGTTATTCATC

AAAGACTATAGGTACACATACCGATGCCACAGGGGTGATACACAAATTCAAACGAGGAGA

TCATTCGAGCTAAAGAAGCTGTGGGAGCAGACCCGGTCAAAGGCAGGGCTGTTGATATCA

GACGGGGGGCCAAACCTATACAACATTCGGAACCTCCACATCCCAGAGGTCTGCTTGAAG

TGGGAGCTGATGGATGAAGACTACCAAGGCAGGCTGTGCAACCCTCTGAATCCATTTGTC

AGTCATAAAGAGATTGAGTCCGTAAACAATGCTGTAGTAATGCCCGCCCATGGCCCGGCC

AAGAGCATGGAATATGATGCTGTTGCGACCACACATTCGTGGATTCCTAAGAGGAACCGC

TCCATTCTCAATACCAGCCAAAGGGGAATTCTTGAGGATGAGCAGATGTACCAAAAGTGC

TGTAGTCTATTCGAGAAATTCTTCCCCAGCAGTTCATACAGGAGGCCAGTTGGAATTTCC

AGCATGGTGGAGGCCATGGTGTCTAGGGCCCGAATTGATGCACGCATCGACTTCGAATCT

GGCAGGATTAAGAAAGAAGAGTTTGCTGAGATCATGAAGATCTGTTCCACCATTGAAGAG

CTCAGACGGCAAAAATAG------------------------------------------

-

>A_goose_Russian_Federation_Kurgan_1345-25_2020_EPI1811686

------------CAAACTATTTGAATGGATGTCAATCCGACTTTACTTTTCTTAAAAGTG

CCAGCGCAAAATGCTATAAGTACTACATTCCCTTACACTGGAGATCCTCCATACAGCCAT

GGAACAGGAACAGGGTATACCATGGACACAGTAAACAGAACACATCAATACTCAGAAAAG

GGAAAGTGGACAACAAACACAGAAACCGGAGCACCCCAACTCAACCCAATTGATGGACCA

TTACCAGAGGACAATGAGCCAAGCGGATATGCACAAACTGATTGCGTGTTGGAAGCAATG

GCTTTCCTTGAAGAATCCCACCCAGGGATATTTGAAAACTCTTGTCTTGAAGCGATGGAA

ATCGTTCAGCAAACAAGAGTGGACAAACTAACCCAGGGTCGCCAGACTTATGACTGGACA

CTGAACAGAAACCAACCAGCTGCAACCTCTTTGGCCAACACTATAGAGGTGTTCAGATCG

AATGGTCTGACAGCCAATGAATCAGGGAGACTGATAGATTTTCTCAGGGATGTGATGGAA

TCAATGGATAAAGAAGAGATGGAAGTAACAACACATTTCCAGAGAAAAAGAAGAGTGAGG

GACAACATGACTAAGAAGATGGTCACACAAAGAACAATAGGGAAGAAGAAGCAGAGGCTG

AACAAGAGGAGTTACTTAATAAGAGCACTGACATTGAATACAATGACCAAAGATGCAGAA

AGAGGCAAGTTGAAGAGACGGGCAATTGCAACACCCGGGATGCAGATTAGAGGATTCGTG

TACTTCGTTGAAACACTAGCGAGGAGCATCTGTGAGAAACTAGAGCAATCTGGGCTCCCT

GTTGGAGGGAATGAGAAGAAGGCTAAATTGGCAAATGTTGTGAGAAAAATGATGACTAAC

TCACAAGATACAGAGCTCTCCTTTACAATTACTGGAGACAACACCAAATGGAATGAGAAT

CAAAACCCTCGGATGTTTTTGGCAATGATAACATATATCACAAGAAACCAACCTGAATGG

TTTAGAAATGTCTTAAGCATTGCCCCTATAATGTTCTCAAACAAAATGGCGAGATTAGGG

AAAGGATACATGTTTGAAAGTAAGAGCATGAAGCTAAGAACACAAATACCAGCAGAGATG

CTTACAAATATCGATCTGAAGTATTTCAACGAACCAACGAGAAAGAAAATCGAGAAAATA

AGACCTCTGCTGATTGATGGCACGGCCTCATTGAGTCCTGGGATGATGATGGGCATGTTC

AATATGCTGAGCACAGTATTAGGGGTCTCAATCCTGAATCTCGGGCAAAAAAGGTACACC

AAAACCACATACTGGTGGGATGGACTTCAATCCTCTGATGATTTCGCTCTCATAGTGAAT

GCACCGAATCATGAGGGGATACAAGCAGGAGTGGATAGATTCTATAGGACCTGCAAACTG

GTTGGGATCAACATGAGCAAAAAGAAGTCTTACATAAACCGAACAGGAACATTTGAGTTC

ACAAGTTTTTTCTACCGCTATGGATTTGTAGCTAACTTCAGTATGGAATTACCCAGCTTT

GGAGTGTCTGGAATCAATGAATCAGCTGACATGAGCATTGGAGTTACAGTGATAAAAAAC

AATATGATAAACAATGATCTTGGACCAGCAACAGCTCAAATGGCTCTTCAGTTATTCATC

AAAGACTATAGGTACACATACCGATGCCACAGGGGTGATACACAAATTCAAACGAGGAGA

TCATTCGAGCTGAAGAAGCTGTGGGAGCAGACCCGTTCAAAGGCAGGGCTGTTGATATCA

GACGGGGGGCCAAACCTATACAACATTCGGAATCTCCACATCCCAGAGGTCTGCTTGAAG

TGGGAGCTGATGGATGAAGACTACCAAGGCAGGCTGTGCAATCCTCTGAATCCATTTGTC

AGTCATAAAGAGATTGAGTCCGTAAACAATGCTGTAGTAATGCCCGCCCATGGCCCGGCC

AAGAGCATGGAATATGATGCTGTTGCGACCACACACTCGTGGATTCCTAAGAGGAACCGT

TCCATTCTCAATACCAGCCAAAGGGGAATTCTTGAGGATGAGCAGATGTACCAAAAGTGC

TGTAGTCTATTCGAGAAATTCTTCCCCAGCAGTTCATACAGGAGGCCAGTTGGAATTTCC

AGCATGGTGGAGGCCATGGTGTCTAGGGCCCGAATTGATGCACGCATCGACTTCGAATCT

GGAAGGATTAAGAAAGAAGAGTTTGCTGAGATCATGAAGATCTGTTCCACCATTGAAGAG

CTCAGACGGCAAAAATAGTGAATTTAGCGTGTCCTTCATGAAAAAATG------------

-

>A_duck_Chelyabinsk_1207-1_2020_EPI1812531

------------CAAACTATTTGAATGGATGTCAATCCGACTTTACTTTTCTTAAAAGTG

CCAGCGCAAAATGCTATAAGTACTACATTCCCTTACACTGGAGATCCTCCATACAGCCAT

GGAACAGGAACAGGTTATACCATGGACACAGTAAACAGAACACATCAATACTCAGAAAAG

GGAAAGTGGACAACAAACACAGAAACCGGAGCACCCCAACTCAACCCAATTGATGGACCA

TTACCAGAGGACAATGAGCCAAGCGGATATGCACAAACTGATTGCGTGTTGGAAGCAATG

GCTTTCCTTGAAGAATCCCACCCAGGGATATTTGAAAACTCTTGTCTTGAAGCGATGGAA

ATCGTTCAGCAAACAAGAGTGGACAAACTAACCCAAGGTCGCCAGACTTATGACTGGACA

CTGAACAGAAACCAACCAGCTGCAACCTCTTTGGCCAACACTATAGAGGTGTTCAGATCG

AATGGTCTGACAGCCAATGAATCAGGGAGACTGATAGATTTTCTCAGGGATGTGATGGAA

TCAATGGATAAAGAAGAGATGGAAGTAACAACACATTTCCAGAGAAAAAGAAGAGTGAGG

GACAACATGACTAGGAAGATGGTCACACAAAGAACAATAGGGAAGAAGAAGCAGAGGCTG

AACAAGAGGAGTTACTTAATAAGAGCACTGACATTGAATACAATGACCAAAGATGCAGAA

AGAGGCAAGTTGAAGAGACGGGCAATTGCAACACCCGGGATGCAGATTAGAGGATTCGTG

TACTTCGTTGAAACACTAGCGAGGAGCATCTGTGAGAAACTAGAGCAATCTGGGCTCCCT

GTTGGAGGGAATGAGAAGAAGGCTAAATTGGCAAATGTTGTGAGAAAAATGATGACTAAC

TCACAAGATACAGAGCTCTCCTTCACAATTACTGGAGACAACACCAAATGGAATGAGAAT

CAAAACCCTCGGATGTTTTTGGCAATGATAACATATGTCACAAGAAACCAACCTGAATGG

TTTAGAAATGTCTTAAGCATTGCCCCTATAATGTTCTCAAACAAAATGGCGAGATTAGGG

AAAGGATACATGTTTGAAAGTAAGAGCATGAAGCTAAGAACACAAATACCAGCAGAGATG

CTTACAAATATTGATCTGAAGTATTTCAACGAACCAACGAGAAAGAAAATCGAGAAAATA

AGACCTCTGCTGATTGATGGCACGGCCTCATTGAGTCCTGGGATGATGATGGGCATGTTC

AATATGCTGAGCACAGTATTAGGGGTCTCAATCCTGAATCTCGGGCAAAAAAGGTACACC

AAAACCACATACTGGTGGGATGGACTCCAATCCTCTGATGATTTCGCTCTCATAGTGAAT

GCACCGAATCATGAGGGGATACAAGCAGGAGTGGATAGATTCTATAGGACCTGCAAACTG

GTTGGGATCAACATGAGCAAAAAGAAGTCTTACATAAACCGAACAGGAACATTTGAGTTC

ACAAGTTTTTTCTACCGCTATGGATTTGTAGCTAACTTCAGTATGGAATTACCCAGCTTT

GGAGTGTCTGGAATCAATGAATCAGCTGACATGAGCATTGGAGTTACAGTGATAAAAAAC

AATATGATAAACAATGATCTTGGACCAGCAACAGCTCAAATGGCTCTTCAGTTATTCATC

AAAGACTATAGGTACACATACCGATGCCACAGGGGTGATACACAAATTCAAACGAGGAGA

TCATTCGAGCTGAAGAAGCTGTGGGAGCAGACCCGTTCAAAGGCAGGGCTGTTGATATCA

GACGGGGGGCCAAACCTATACAACATTCGGAATCTCCACATCCCAGAGGTCTGCTTGAAG

TGGGAGCTGATGGATGAAGACTACCAAGGCAGGCTGTGCAATCCTCTGAATCCATTTGTC

AGTCATAAAGAGATTGAGTCCGTAAACAATGCTGTAGTAATGCCCGCCCATGGCCCGGCC

AAGAGCATGGAATATGATGCTGTTGCGACCACACACTCGTGGATTCCTAAGAGGAACCGT

TCCATTCTCAATACCAGCCAAAGGGGAATTCTTGAGGATGAGCAGATGTACCAAAAGTGC

TGTAGTCTATTCGAGAAATTCTTCCCCAGCAGTTCATACAGGAGGCCAGTTGGAATTTCC

AGCATGGTGGAGGCCATGGTGTCTAGGGCCCGAATTGATGCACGCATCGACTTCGAATCT

GGAAGGATTAAGAAAGAAGAGTTTGCTGAGATCATGAAGATCTGTTCCACCATTGAAGAG

CTCAGACGGCAAAAATAGTGAATTTAGCTTGTCCTTCATGAAAAAATG------------

-

>A_goose_Omsk_0002_2020_EPI1813119

AGCAAAAGCAGGCAAACTATTTGAATGGATGTCAATCCGACTTTACTTTTCTTAAAAGTG

CCAGCGCAAAATGCTATAAGTACTACATTCCCTTACACTGGAGATCCCCCATACAGCCAT

GGAACAGGAACAGGGTATACCATGGACACAGTAAACAGAACACATCAATACTCAGAAAAG

GGAAAGTGGACAACAAACACAGAAACCGGAGCACCCCAACTCAACCCAATTGATGGACCA

TTACCAGAGGACAATGAGCCAAGCGGATATGCACAAACTGATTGCGTGTTGGAAGCAATG

GCTTTCCTTGAAGAATCCCACCCAGGGATATTTGAAAACTCTTGTCTTGAAGCGATGGAA

ATCGTTCAGCAAACAAGAGTGGACAAACTAACCCAAGGTCGCCAGACTTATGACTGGACA

CTGAACAGAAACCAACCAGCTGCAACCTCTTTGGCCAACACTATAGAGGTGTTCAGATCG

AATGGTCTGACAGCCAATGAATCAGGGAGACTGATAGATTTTCTCAGGGATGTGATGGAA

TCAATGGATAAAGAAGAGATGGAAGTAACAACACATTTCCAGAGAAAAAGAAGAGTGAGG

GACAACATGACTAAGAAGATGGTCACACAAAGAACAATAGGGAAGAAGAAGCAGAGGCTG

AACAAGAGGAGTTACTTAATAAGAGCACTGACATTGAATACAATGACCAAAGATGCAGAA

AGAGGCAAGTTGAAGAGACGGGCAATTGCAACACCCGGGATGCAGATTAGAGGATTCGTG

TACTTCGTTGAAACACTAGCGAGAAGCATCTGTGAGAAACTAGAGCAATCTGGGCTCCCT

GTTGGAGGGAATGAGAAGAAGGCTAAATTGGCAAATGTTGTGAGAAAAATGATGACTAAC

TCACAAGATACAGAGCTCTCCTTTACAATTACTGGAGACAACACCAAATGGAATGAGAAT

CAAAACCCTCGGATGTTTTTGGCAATGATAACATATATCACAAGAAACCAACCTGAATGG

TTTAGAAATGTCTTAAGCATTGCCCCTATAATGTTCTCAAACAAAATGGCGAGATTAGGG

AAAGGATACATGTTTGAAAGTAAGAGCATGAAGCTAAGAACACAAATACCAGCAGAGATG

CTTACAAATATTGATCTGAAGTATTTCAACGAACCAACGAGAAAGAAAATCGAGAAAATA

AGACCTCTGCTGATTGATGGCACGGCCTCATTGAGTCCTGGGATGATGATGGGCATGTTC

AATATGCTGAGCACAGTATTAGGGGTCTCAATCCTGAATCTCGGGCAAAAGAGGTACACC

AAAACCACATACTGGTGGGATGGACTCCAATCCTCTGATGATTTCGCTCTCATAGTGAAT

GCACCGAATCATGAGGGGATACAAGCAGGAGTGGATAGATTCTATAGGACCTGCAAACTG

GTTGGGATCAACATGAGCAAAAAGAAGTCTTACATAAACCGAACAGGAACATTTGAGTTC

ACAAGTTTTTTCTACCGCTATGGATTTGTAGCTAACTTCAGTATGGAACTACCCAGCTTT

GGAGTGTCTGGAATCAATGAATCAGCTGACATGAGCATTGGAGTTACAGTGATAAAAAAC

AATATGATAAACAATGATCTTGGACCAGCAACAGCTCAAATGGCTCTTCAGTTATTCATC

AAAGACTATAGGTACACATACCGATGCCACAGGGGTGATACACAAATTCAAACGAGGAGA

TCATTCGAGCTGAAGAAGCTGTGGGAGCAGACCCGTTCAAAGGCAGGGCTGTTGATATCA

GACGGGGGGCCAAACCTATACAACATTCGGAATCTCCACATCCCAGAGGTCTGCTTGAAG

TGGGAGCTGATGGATGAAGACTACCAAGGCAGGCTGTGCAATCCTCTGAATCCATTTGTC

AGTCATAAAGAGATTGAGTCCGTAAACAATGCTGTAGTAATGCCCGCCCATGGCCCGGCC

AAGAGCATGGAATATGATGCTGTTGCGACCACACACTCGTGGATTCCTAAGAGGAACCGT

TCCATTCTCAATACCAGCCAAAGGGGAATTCTTGAGGATGAGCAGATGTACCAAAAGTGC

TGTAGTCTATTCGAGAAATTCTTCCCCAGCAGTTCATACAGGAGGCCAGTTGGAATTTCC

AGCATGGTGGAGGCCATGGTGTCTAGGGCCCGAATTGATGCACGCATCGACTTCGAATCT

GGAAGGATTAAGAAAGAAGAGTTTGCTGAGATCATGAAGATCTGTTCCACCATTGAAGAG

CTCAGACGGCAAAAATAGTGAATTTAGCTTGTCCTTCATGAAAAAATGCCTTGTTTCTAC

T

>A_goose_Omsk_01171_2020_EPI1813199

AGCAAAAGCAGGCAAACTATTTGAATGGATGTCAATCCGACTTTACTTTTCTTAAAAGTG

CCAGCGCAAAATGCTATAAGTACTACATTCCCTTACACTGGAGATCCTCCATACAGCCAT

GGAACAGGAACAGGGTATACCATGGACACAGTAAACAGAACACATCAATACTCAGAAAAG

GGAAAGTGGACAACAAACACAGAAACCGGAGCACCCCAACTCAACCCAATTGATGGACCA

TTACCAGAGGACAATGAGCCAAGCGGATATGCACAAACTGATTGCGTGTTGGAAGCAATG

GCTTTCCTTGAAGAATCCCACCCAGGGATATTTGAAAACTCTTGTCTTGAAGCGATGGAA

ATCGTTCAGCAAACAAGAGTGGACAAACTAACCCAAGGTCGCCAGACTTATGACTGGACA

CTGAACAGAAACCAACCAGCTGCAACCTCTTTGGCCAACACTATAGAGGTGTTCAGATCG

AATGGTCTGACAGCCAATGAATCAGGGAGACTGATAGATTTTCTCAGGGATGTGATGGAA

TCAATGGATAAAGAAGAGATGGAAGTAACAACACATTTCCAGAGAAAAAGAAGAGTGAGG

GACAACATGACTAAGAAGATGGTCACACAAAGAACAATAGGGAAGAAGAAGCAGAGGCTG

AACAAGAGGAGTTACTTAATAAGAGCACTGACATTGAATACAATGACCAAAGATGCAGAA

AGAGGCAAGTTGAAGAGACGGGCAATTGCAACACCCGGGATGCAGATTAGAGGATTCGTG

TACTTCGTTGAAACACTAGCGAGGAGCATCTGTGAGAAACTAGAGCAATCTGGGCTCCCT

GTTGGAGGGAATGAGAAGAAGGCTAAATTGGCAAATGTTGTGAGAAAAATGATGACTAAC

TCACAAGATACAGAGCTCTCCTTTACAATTACTGGAGACAACACCAAATGGAATGAGAAT

CAAAACCCTCGGATGTTTTTGGCAATGATAACATATATCACAAGAAACCAACCTGAATGG

TTTAGAAATGTCTTAAGCATTGCCCCTATAATGTTCTCAAACAAAATGGCGAGATTAGGG

AAAGGATACATGTTTGAAAGTAAGAGCATGAAGCTAAGAACACAAATACCAGCAGAGATG

CTCACAAATATTGATCTGAAGTATTTCAACGAACCAACGAGAAAGAAAATCGAGAAAATA

AGACCTCTGCTGATTAATGGCACGGCCTCATTGAGTCCTGGGATGATGATGGGCATGTTC

AATATGCTGAGCACAGTATTGGGGGTCTCAATCCTGAATCTCGGGCAAAAGAGGTACACC

AAAACCACATACTGGTGGGATGGACTTCAATCCTCTGATGATTTCGCTCTCATAGTGAAT

GCACCGAATCATGAGGGGATACAAGCAGGAGTGGATAGATTCTATAGGACCTGCAAACTG

GTTGGGATCAACATGAGCAAAAAGAAGTCTTACATAAACCGAACAGGAACATTTGAGTTC

ACAAGTTTTTTCTACCGCTATGGATTTGTAGCTAACTTCAGTATGGAATTACCCAGCTTT

GGAGTGTCTGGAATCAATGAATCAGCTGACATGAGCATTGGAGTTACAGTGATAAAAAAC

AATATGATAAACAATGATCTTGGACCAGCGACAGCTCAAATGGCTCTTCAGTTATTCATC

AAAGACTATAGGTACACATACCGATGCCACAGGGGTGATACACAAATTCAAACGAGGAGA

TCATTCGAGCTGAAGAAGCTGTGGGAGCAGACCCGTTCAAAGGCAGGGCTGTTGATATCA

GACGGGGGGCCAAACCTATACAACATTCGGAACCTCCACATCCCAGAGGTCTGCTTGAAG

TGGGAGCTGATGGATGAAGACTACCAAGGCAGGCTGTGCAATCCTCTGAATCCATTTGTC

AGTCATAAAGAGATTGAGTCCGTAAACAATGCTGTAGTAATGCCCGCCCATGGCCCGGCC

AAGAGCATGGAATATGATGCTGTTGCGACCACACATTCGTGGATTCCTAAGAGGAACCGT

TCCATTCTCAATACCAGCCAAAGGGGAATTCTTGAGGATGAGCAGATGTACCAAAAGTGC

TGTAGTCTATTCGAGAAATTCTTCCCCAGCAGTTCATACAGGAGGCCAGTTGGAATTTCC

AGCATGGTGGAGGCCATGGTGTCTAGGGCCCGAATTGATGCACGCATCGACTTCGAATCT

GGAAGGATTAAGAAAGAAGAGTTTGCTGAGATCATGAAGATCTGTTCCACCATTGAAGAG

CTCAGACGGCAAAAATAGTGAATTTAGCTTGTCCTTCATGAAAAAATGCCTTGTTTCTAC

T

>A_duck_Omsk_0075_2020_EPI1813279

AGCAAAAGCAGGCAAACTATTTGAATGGATGTCAATCCGACTTTACTTTTCTTAAAAGTG

CCAGCGCAAAATGCTATAAGTACTACATTCCCTTACACTGGAGATCCTCCATACAGCCAT

GGAACAGGAACAGGGTATACCATGGACACAGTAAACAGAACACATCAATACTCAGAAAAG

GGAAAGTGGACAACAAACACAGAAACCGGAGCACCCCAACTCAACCCAATTGATGGACCA

TTACCAGAGGACAATGAGCCAAGCGGATATGCACAAACTGATTGCGTGTTGGAAGCGATG

GCTTTCCTTGAAGAATCCCACCCAGGGATATTTGAAAACTCTTGTCTTGAAGCGATGGAA

ATCGTTCAGCAAACAAGAGTGGACAAACTAACCCAAGGTCGCCAGACTTATGACTGGACA

CTGAACAGAAACCAACCAGCTGCAACCTCTTTGGCCAACACTATAGAGGTGTTCAGATCG

AATGGTCTGACAGCCAATGAATCAGGGAGACTGATAGATTTTCTCAGGGATGTGATGGAA

TCAATGGATAAAGAAGAGATGGAAGTAACAACACATTTCCAGAGAAAAAGAAGAGTGAGG

GACAACATAACTAGGAAGATGGTCACACAAAGAACAATAGGGAAGAAGAAGCAGAGGCTG

AACAAGAGGAGTTACTTAATAAGAGCACTGACATTGAATACAATGACCAAAGATGCAGAA

AGAGGCAAGTTGAAGAGACGGGCAATTGCAACACCCGGGATGCAGATTAGAGGATTCGTG

TACTTCGTTGAAACACTAGCGAGGAGCATCTGTGAGAAATTAGAGCAATCTGGGCTCCCT

GTTGGAGGGAATGAGAAGAAGGCTAAATTGGCAAATGTTGTGAGAAAAATGATGACTAAC

TCACAAGATACAGAGCTCTCCTTTACAATTACTGGAGACAACACCAAATGGAATGAGAAT

CAAAACCCTCGGATGTTTTTGGCAATGATAACATATATCACAAGAAACCAACCTGAATGG

TTTAGAAATGTCTTAAGCATTGCCCCTATAATGTTCTCAAACAAAATGGCGAGATTAGGG

AAAGGATACATGTTTGAAAGTAAGAGCATGAAGCTAAGAACACAAATACCAGCAGAGATG

CTTACAAATATTGATCTGAAGTATTTCAACGAACCAACGAGAAAGAAAATCGAGAAAATA

AGACCTCTGCTGATTGATGGCACGGCCTCATTGAGTCCTGGGATGATGATGGGCATGTTC

AATATGCTGAGCACAGTATTAGGGGTCTCAATCCTGAATCTCGGGCAAAAAAGGTACACC

AAAACCACATACTGGTGGGATGGACTTCAATCCTCTGATGATTTCGCTCTCATAGTGAAT

GCACCGAATCATGAGGGGATACAAGCAGGAGTGGATAGATTCTATAGGACCTGCAAACTG

GTTGGGATCAACATGAGCAAAAAGAAGTCTTACATAAACCGAACAGGAACATTTGAGTTC

ACAAGTTTTTTCTACCGCTATGGATTTGTAGCTAACTTCAGTATGGAATTACCCAGCTTT

GGAGTGTCTGGAATCAATGAATCAGCTGACATGAGCATTGGAGTTACAGTGATAAAAAAC

AATATGATAAACAATGATCTTGGACCAGCAACAGCTCAAATGGCTCTTCAGTTATTCATC

AAAGACTATAGGTACACATACCGATGCCACAGGGGTGATACACAAATTCAAACGAGGAGA

TCATTCGAGCTGAAGAAGCTGTGGGAGCAGACCCGTTCAAAGGCAGGGCTGTTGATATCA

GACGGGGGGCCAAACCTATACAACATTCGGAATCTCCACATCCCAGAGGTCTGCTTGAAG

TGGGAGCTGATGGATGAAGACTACCAAGGCAGGCTGTGCAATCCTCTGAATCCATTTGTC

AGTCATAAAGAGATTGAGTCCGTAAACAATGCTGTAGTAATGCCCGCCCATGGCCCGGCC

AAGAACATGGAATATGATGCTGTTGCGACCACACACTCGTGGATTCCTAAGAGGAACCGT

TCCATTCTCAATACCAGCCAAAGGGGAATTCTTGAGGATGAGCAGATGTACCAAAAGTGC

TGTAGTCTATTCGAGAAATTCTTCCCCAGCAGTTCATACAGGAGGCCAGTTGGAATTTCC

AGCATGGTGGAGGCCATGGTGTCTAGGGCCCGAATTGATGCACGCATCGACTTCGAATCT

GGAAGGATTAAGAAAGAAGAGTTTGCTGAGATCATGAAGATCTGTTCCACCATTGAAGAG

CTCAGACGGCAAAAATAGTGAATTTAGCTTGTCCTTCATGAAAAAATGCCTTGTTTCTAC

T

>A_duck_Omsk_0004_2020_EPI1813335

AGCAAAAGCAGGCAAACTATTTGAATGGATGTCAATCCGACTTTACTTTTCTTAAAAGTG

CCAGCGCAAAATGCTATAAGTACTACATTCCCTTACACTGGAGATCCTCCATACAGCCAT

GGAACAGGAACAGGGTATACCATGGACACAGTAAACAGAACACATCAATACTCAGAAAAG

GGAAAGTGGACAACAAACACAGAAACCGGAGCACCCCAACTCAACCCAATTGATGGACCA

TTACCAGAGGACAATGAGCCAAGCGGATATGCACAAACTGATTGCGTGTTGGAAGCAATG

GCTTTCCTTGAAGAATCCCACCCAGGGATATTTGAAAACTCTTGTCTTGAAGCGATGGAA

ATCGTTCAGCAAACAAGAGTGGACAAACTAACCCAAGGTCGCCAGACTTATGACTGGACA

CTGAACAGAAACCAACCAGCTGCAACCTCTTTGGCCAACACTATAGAGGTGTTCAGATCG

AATGGTCTGACAGCCAATGAATCAGGGAGACTGATAGATTTTCTCAGGGATGTGATGGAA

TCAATGGATAAAGAAGAGATGGAAGTAACAACACATTTCCAGAGAAAAAGAAGAGTGAGG

GACAACATGACTAGGAAGATGGTCACACAAAGAACAATAGGGAAGAAGAAGCAGAGGCTG

AACAAGAGGAGTTACTTAATAAGAGCACTGACATTGAATACAATGACCAAAGATGCAGAA

AGAGGCAAGTTGAAGAGACGGGCAATTGCAACACCCGGGATGCAGATTAGAGGATTCGTG

TACTTCGTTGAAACACTAGCGAGGAGCATCTGTGAGAAACTAGAGCAATCTGGGCTCCCT

ATTGGAGGGAATGAGAAGAAGGCTAAATTGGCAAATGTTGTGAGAAAAATGATGACTAAC

TCACAAGATACAGAGCTCTCCTTTACAATTACTGGAGACAACACCAAATGGAATGAGAAT

CAAAACCCTCGGATGTTTTTGGCAATGATAACATATATCACAAGAAACCAACCTGAATGG

TTTAGAAATGTCTTAAGCATTGCCCCTATAATGTTCTCAAACAAAATGGCGAGATTAGGG

AAAGGATACATGTTTGAAAGTAAGAGCATGAAGCTAAGAACACAAATACCAGCAGAGATG

CTTACAAATATTGATCTGAAGTATTTCAACGAACCAACGAGAAAGAAAATCGAGAAAATA

AGACCTCTGCTGATTGATGGCACGGCCTCATTGAGTCCTGGGATGATGATGGGCATGTTC

AATATGCTGAGCACAGTATTAGGGGTCTCAATCCTGAATCTCGGGCAAAAAAGGTACACC

AAAACCACATACTGGTGGGATGGACTTCAATCCTCTGATGATTTCGCTCTCATAGTGAAT

GCACCGAATCATGAGGGGATACAAGCAGGAGTGGATAGATTCTATAGGACCTGCAAACTG

GTTGGGATCAACATGAGCAAAAAGAAGTCTTACATAAACCGAACAGGAACATTTGAGTTC

ACAAGTTTTTTCTACCGCTATGGATTTGTAGCTAACTTCAGTATGGAATTACCCAGCTTT

GGAGTGTCTGGAATCAATGAATCAGCTGACATGAGCATTGGAGTTACAGTGATAAAAAAC

AATATGATAAACAATGATCTTGGACCAGCAACAGCTCAAATGGCTCTTCAGTTATTCATC

AAAGACTATAGGTACACATACCGATGCCACAGGGGTGATACACAAATTCAAACGAGGAGA

TCATTCGAGCTGAAGAAGCTGTGGGAGCAGACCCGTTCAAAGGCAGGGCTGTTGATATCA

GACGGGGGGCCAAACCTATACAACATTCGGAATCTCCACATCCCAGAGGTCTGCTTGAAG

TGGGAGCTGATGGATGAAGACTACCAAGGCAGGCTGTGCAATCCTCTGAATCCATTTGTC

AGTCATAAAGAGATTGAGTCCGTAAACAATGCTGTAGTAATGCCCGCCCATGGCCCGGCC

AAGAGCATGGAATATGATGCTGTTGCGACCACACACTCGTGGATTCCTAAGAGGAACCGT

TCCATTCTCAATACCAGCCAAAGGGGAATTCTTGAGGATGAGCAGATGTACCAAAAGTGC

TGTAGTCTATTCGAGAAATTCTTCCCCAGCAGTTCATACAGGAGGCCAGTTGGAATTTCC

AGCATGGTGGAGGCCATGGTGTCTAGGGCCCGAATTGATGCACGCATCGACTTCGAATCT

GGAAGGATTAAGAAAGAAGAGTTTGCTGAGATCATGAAGATCTGTTCCACCATTGAAGAG

CTCAGACGGCAAAAATAGTGAATTTAGCTTGTCCTTCATGAAAAAATGCCTTGTTTCTAC

T

>A_chicken_Omsk_0112_2020_EPI1813343

AGCAAAAGCAGGCAAACTATTTGAATGGATGTCAATCCGACTTTACTTTTCTTAAAAGTG

CCAGCGCAAAATGCTATAAGTACTACATTCCCTTACACTGGAGATCCTCCATACAGCCAT

GGAACAGGAACAGGGTATACCATGGACACAGTAAACAGAACACATCAATACTCAGAAAAG

GGAAAGTGGACAACAAACACAGAAACCGGAGCACCCCAACTCAACCCAATTGATGGACCA

TTACCAGAGGACAATGAGCCAAGCGGATATGCACAAACTGATTGCGTGTTGGAAGCAATG

GCTTTCCTTGAAGAATCCCACCCAGGGATATTTGAAAACTCTTGTCTTGAAGCGATGGAA

ATCGTTCAGCAAACAAGAGTGGACAAACTAACCCAAGGTCGCCAGACTTATGACTGGACA

CTGAACAGAAACCAACCAGCTGCAACCTCTTTGGCCAACACTATAGAGGTGTTCAGATCG

AATGGTCTGACAGCCAATGAATCAGGGAGACTGATAGATTTTCTCAGGGATGTGATGGAA

TCAATGGATAAAGAAGAGATGGAAGTAACAACACATTTCCAGAGAAAAAGAAGAGTGAGG

GACAACATGACTAAGAAGATGGTCACACAAAGAACAATAGGGAAGAAGAAGCAGAGGCTG

AACAAGAGGAGTTACTTAATAAGAGCACTGACATTGAATACAATGACCAAAGATGCAGAA

AGAGGCAAGTTGAAGAGACGGGCAATTGCAACACCCGGGATGCAGATTAGAGGATTCGTG

TACTTCGTTGAAACACTAGCGAGGAGCATCTGTGAGAAACTAGAGCAATCTGGGCTCCCT

GTTGGAGGGAATGAGAAGAAGGCTAAATTGGCAAATGTTGTGAGAAAAATGATGACTAAC

TCACAAGATACAGAGCTCTCCTTTACAATTACTGGAGACAACACCAAATGGAATGAGAAT

CAAAACCCTCGGATGTTTTTGGCAATGATAACATATATCACAAGAAACCAACCTGAATGG

TTTAGAAATGTCTTAAGCATTGCCCCTATAATGTTCTCAAACAAAATGGCGAGATTAGGG

AAAGGATACATGTTTGAAAGTAAGAGCATGAAGCTAAGAACACAAATACCAGCAGAGATG

CTTACAAATATTGATCTGAAGTATTTCAACGAACCAACGAGAAAGAAAATCGAGAAAATA

AGACCTCTGCTGATTGATGGCACGGCCTCATTGAGTCCTGGGATGATGATGGGCATGTTC

AATATGCTGAGCACAGTATTGGGGGTCTCAATCCTGAATCTCGGGCAAAAGAGGTACACC

AAAACCACATACTGGTGGGATGGACTTCAATCCTCTGATGATTTCGCTCTCATAGTGAAT

GCACCGAATCATGAGGGGATACAAGCAGGAGTGGATAGATTCTATAGGACCTGCAAACTG

GTTGGGATCAACATGAGCAAAAAGAAGTCTTACATAAACCGAACAGGAACATTTGAGTTC

ACAAGTTTTTTCTACCGCTATGGATTTGTAGCTAACTTCAGTATGGAATTACCCAGCTTT

GGAGTGTCTGGAATCAATGAATCAGCTGACATGAGCATTGGAGTTACAGTGATAAAAAAC

AATATGATAAACAATGATCTTGGACCAGCAACAGCTCAAATGGCTCTTCAGTTATTCATC

AAAGACTATAGGTACACATACCGATGCCACAGGGGTGATACACAAATTCAAACGAGGAGA

TCATTCGAGCTGAAGAAGCTGTGGGAGCAGACCCGTTCAAAGGCAGGGCTGTTGATATCA

GACGGGGGGCCAAACCTATACAACATTCGGAACCTCCACATCCCAGAGGTCTGCTTGAAG

TGGGAGCTGATGGATGAAGACTACCAAGGCAGGCTGTGCAATCCTCTGAATCCATTTGTC

AGTCATAAAGAGATTGAGTCCGTAAACAATGCTGTAGTAATGCCCGCCCATGGCCCGGCC

AAGAGCATGGAATATGATGCTGTTGCGACCACACATTCGTGGATCCCTAAGAGGAACCGT

TCCATTCTCAATACCAGCCAAAGGGGAATTCTTGAGGATGAGCAGATGTACCAAAAGTGC

TGTAGTCTATTCGAGAAATTCTTCCCCAGCAGTTCATACAGGAGGCCAGTTGGAATTTCC

AGCATGGTGGAGGCCATGGTGTCTAGGGCCCGAATTGATGCACGCATCGACTTCGAATCT

GGAAGGATTAAGAAAGAAGAGTTTGCTGAGATCATGAAGATCTGTTCCACCATTGAAGAG

CTCAGACGGCAAAAATAGTGAATTTAGCTTGTCCTTCATGAAAAAATGCCTTGTTTCTAC

T

>A_duck_Saratov_29804_2020_EPI1814263

AGCAAAAGCAGGCAAACTATTTGAATGGATGTCAATCCGACTTTACTTTTCTTAAAAGTG

CCAGCGCAAAATGCTATAAGTACTACATTCCCTTACACTGGAGATCCCCCATACAGCCAT

GGAACAGGAACAGGGTATACCATGGACACAGTAAACAGAACACATCAATACTCAGAAAAG

GGAAAGTGGACAACAAACACAGAAACCGGAGCACCCCAACTCAACCCAATTGATGGACCA

TTACCAGAGGACAATGAGCCAAGCGGATATGCACAAACTGATTGCGTGTTGGAAGCAATG

GCTTTCCTTGAAGAATCCCACCCAGGGATATTTGAAAACTCTTGTCTTGAAGCGATGGAA

ATCGTTCAGCAAACAAGAGTGGACAAACTAACCCAAGGTCGCCAGACTTATGACTGGACA

CTGAACAGAAACCAACCAGCTGCAACCTCTTTGGCCAACACTATAGAGGTGTTCAGATCG

AATGGTCTGACAGCCAATGAATCAGGGAGACTGATAGATTTTCTCAGGGATGTGATGGAA

TCAATGGATAAAGAAGAGATGGAAGTAACAACACATTTCCAGAGAAAAAGAAGAGTGAGG

GACAACATGACTAAGAAGATGGTCACACAAAGAACAATAGGGAAGAAGAAGCAGAGGCTG

AACAAGAGGAGTTACTTAATAAGAGCACTGACATTGAATACAATGACCAAAGATGCAGAA

AGAGGCAAGTTGAAGAGACGGGCAATTGCAACACCCGGGATGCAGATTAGAGGATTCGTG

TACTTCGTTGAAACACTAGCGAGGAGCATCTGTGAGAAACTAGAGCAATCTGGGCTCCCT

GTTGGAGGGAATGAGAAGAAGGCTAAATTGGCAAATGTTGTGAGAAAAATGATGACTAAC

TCACAAGATACAGAGCTCTCCTTTACAATTACTGGAGACAACACCAAATGGAATGAGAAT

CAAAACCCTCGGATGTTTTTGGCAATGATAACATATATCACAAGAAACCAACCTGAATGG

TTTAGAAATGTCTTAAGCATTGCCCCTATAATGTTCTCAAACAAAATGGCGAGATTAGGG

AAAGGATACATGTTTGAAAGTAAGAGCATGAAGCTAAGAACACAAATACCAGCAGAGATG

CTTACAAATATTGATCTGAAGTATTTCAACGAACCAACGAGAAAGAAAATCGAGAAAATA

AGACCTCTGCTGATTGATGGCACGGCCTCATTGAGTCCTGGGATGATGATGGGCATGTTC

AATATGCTGAGCACAGTATTAGGGGTCTCAATCCTGAATCTCGGGCAAAAGAGGTACACC

AAAACCACATACTGGTGGGATGGACTTCAATCCTCTGATGATTTCGCTCTCATAGTGAAT

GCACCGAATCATGAGGGGATACAAGCAGGAGTGGATAGATTCTATAGGACCTGCAAACTG

GTTGGGATCAACATGAGCAAAAAGAAGTCTTACATAAACCGAACAGGAACATTTGAGTTC

ACAAGTTTTTTCTACCGCTATGGATTTGTAGCCAACTTCAGTATGGAATTACCCAGCTTT

GGAGTGTCTGGAATCAATGAATCAGCTGACATGAGCATTGGAGTTACAGTGATAAAAAAC

AATATGATAAACAATGATCTTGGACCAGCAACAGCTCAAATGGCTCTTCAGTTATTCATC

AAAGACTATAGGTACACATACCGATGCCACAGGGGTGATACACAAATTCAAACGAGGAGA

TCATTCGAGCTGAAGAAGCTGTGGGAGCAGACCCGTTCAAAGGCAGGGCTGTTGATATCA

GACGGGGGGCCAAACCTATACAACATTCGGAATCTCCACATCCCAGAGGTCTGCTTGAAG

TGGGAGCTGATGGATGAAGACTACCAAGGCAGGCTGTGCAATCCTCTGAATCCATTTGTC

AGTCATAAAGAGATTGAGTCCGTAAACAATGCTGTAGTAATGCCCGCCCATGGCCCGGCC

AAGAGCATGGAATATGATGCTGTTGCGACCACACACTCGTGGATTCCTAAGAGGAACCGT

TCCGTTCTCAATACCAGCCAAAGGGGAATTCTTGAGGATGAGCAGATGTACCAAAAGTGC

TGTAGTCTATTCGAGAAATTCTTCCCCAGCAGTTCATACAGGAGGCCAGTTGGAATTTCC

AGCATGGTGGAGGCCATGGTGTCTAGGGCCCGAATTGATGCACGCATCGACTTCGAATCT

GGAAGGATTAAGAAAGAAGAGTTTGCTGAGATCATGAAGATCTGTTCCACCATTGAAGAG

CTCAGACGGCAAAAATAGTGAATTTAGCTTGTCCTTCATGAAAAAATGCCTTGTTTCTAC

T

>A_goose_Omsk_30001_2020_EPI1814279

AGCAAAAGCAGGCAAACTATTTGAATGGATGTCAATCCGACTTTACTTTTCTTAAAAGTG

CCAGCGCAAAATGCTATAAGTACTACATTCCCTTACACTGGAGATCCTCCATACAGCCAT

GGAACAGGAACAGGGTATACCATGGACACAGTAAACAGAACACATCAATACTCAGAAAAG

GGAAAGTGGACAACAAACACAGAAACCGGAGCACCCCAACTCAACCCAATTGATGGACCA

TTACCAGAGGACAATGAGCCAAGCGGATATGCACAAACTGATTGCGTGTTGGAAGCAATG

GCTTCCCTTGAAGAATCCCACCCAGGGATATTTGAAAACTCTTGTCTTGAAGCGATGGAA

ATCGTTCAGCAAACAAGAGTGGACAAACTAACCCAAGGTCGCCAGACTTATGACTGGACA

CTGAACAGAAACCAACCAGCTGCAACCTCTTTGGCCAACACTATAGAGGTGTTCAGATCG

AATGGTCTGACAGCCAATGAATCAGGGAGACTGATAGATTTTCTCAGGGATGTGATGGAA

TCAATGGATAAAGAAGAGATGGAAGTAACAACACATTTCCAGAGAAAAAGAAGAGTGAGG

GACAACATGACTAGGAAGATGGTCACACAAAGAACAATAGGGAAGAAGAAGCAGAGGCTG

AACAAGAGGAGTTACTTAATAAGAGCACTGACATTGAATACAATGACCAAAGATGCAGAA

AGAGGCAAGTTGAAGAGACGGGCAATTGCAACACCCGGGATGCAGATTAGAGGATTCGTG

TACTTCGTTGAAACACTAGCGAGGAGCATCTGTGAGAAACTAGAGCAATCTGGGCTCCCT

GTTGGAGGGAATGAGAAGAAGGCTAAATTGGCAAATGTTGTGAGAAAAATGATGACTAAC

TCACAAGATACAGAGCTCTCCTTTACAATTACTGGAGACAACACCAAATGGAATGAGAAT

CAAAACCCTCGGATGTTTTTGGCAATGATAACATATATCACAAGAAACCAACCTGAATGG

TTTAGAAATGTCTTAAGCATTGCCCCTATAATGTTCTCAAACAAAATGGCGAGATTAGGG

AAAGGATACATGTTTGAAAGTAAGAGCATGAAGCTAAGAACACAAATACCAGCAGAGATG

CTTACAAATATTGATCTGAAGTATTTCAACGAACCAACGAGAAAGAAAATCGAGAAAATA

AGACCTCTGCTGATTGATGGCACGGCCTCATTGAGTCCTGGGATGATGATGGGCATGTTC

AATATGCTGAGCACAGTATTAGGGGTCTCAATCCTGAATCTCGGGCAAAAAAGGTACACC

AAAACCACATACTGGTGGGATGGACTTCAATCCTCTGATGATTTCGCTCTCATAGTGAAT

GCACCGAATCATGAGGGGATACAAGCAGGAGTGGATAGATTCTATAGGACCTGCAAACTG

GTTGGGATCAACATGAGCAAAAAGAAGTCTTACATAAACCGAACAGGAACATTTGAGTTC

ACAAGTTTTTTCTACCGCTATGGATTTGTAGCTAACTTCAGTATGGAATTACCCAGCTTT

GGGGTGTCTGGAATCAATGAATCAGCTGACATGAGCATTGGAGTTACAGTGATAAAAAAC

AATATGATAAACAATGATCTTGGACCAGCAACAGCTCAAATGGCTCTTCAGTTATTCATC

AAAGACTATAGGTACACATACCGATGCCACAGGGGTGATACACAAATTCAAACGAGGAGA

TCATTCGAGCTGAAGAAGCTGTGGGAGCAGACCCGTTCAAAGGCAGGGCTGTTGATATCA

GACGGGGGGCCAAACCTATACAACATTCGGAATCTCCACATCCCAGAGGTCTGCTTGAAG

TGGGAGCTGATGGATGAAGACTACCAAGGCAGGCTGTGCAATCCTCTGAATCCATTTGTC

AGTCATAAAGAGATTGAGTCCGTAAACAATGCTGTAGTAATGCCCGCCCATGGCCCGGCC

AAGAGCATGGAATATGATGCTGTTGCGACCACACACTCGTGGATTCCTAAGAGGAACCGT

TCCATTCTCAATACCAGCCAAAGGGGAATTCTTGAGGATGAGCAGATGTACCAAAAGTGC

TGTAGTCTATTCGAGAAATTCTTCCCCAGCAGTTCATACAGGAGGCCAGTTGGAATTTCC

AGCATGGTGGAGGCCATGGTGTCTAGGGCCCGAATTGATGCACGCATCGACTTCGAATCT

GGAAGGATTAAGAAAGAAGAGTTTGCTGAGATCATGAAGATCTGTTCCACCATTGAAGAG

CTCAGACGGCAAAAATAGTGAATTTAGCTTGTCCTTCATGAAAAAATGCCTTGTTTCTAC

T

>A_goose_Omsk_30003_2020_EPI1814287

AGCAAAAGCAGGCAAACTATTTGAATGGATGTCAATCCGACTTTACTTTTCTTAAAAGTG

CCAGCGCAAAATGCTATAAGTACTACATTCCCTTACACTGGAGATCCCCCATACAGCCAT

GGAACAGGAACAGGGTATACCATGGACACAGTAAACAGAACACATCAATACTCAGAAAAG

GGAAAGTGGACAACAAACACAGAAACCGGAGCACCCCAACTCAACCCAATTGATGGACCA

TTACCAGAGGACAATGAGCCAAGCGGATATGCACAAACTGATTGCGTGTTGGAAGCAATG

GCTTTCCTTGAAGAATCCCACCCAGGGATATTTGAAAACTCTTGTCTTGAAGCGATGGAA

ATCGTTCAGCAAACAAGAGTGGACAAACTAACCCAAGGTCGCCAGACTTATGACTGGACA

CTGAACAGAAACCAACCAGCTGCAACCTCTTTGGCCAACACTATAGAGGTGTTCAGATCG

AATGGTCTGACAGCCAATGAATCAGGGAGACTGATAGATTTTCTCAGGGATGTGATGGAA

TCAATGGATAAAGAAGAGATGGAAGTAACAACACATTTCCAGAGAAAAAGAAGAGTGAGG

GACAACATGACTAAGAAGATGGTCACACAAAGAACAATAGGGAAGAAGAAGCAGAGGCTG

AACAAGAGGAGTTACTTAATAAGAGCACTGACATTGAATACAATGACCAAAGATGCAGAA

AGAGGCAAGTTGAAGAGACGGGCAATTGCAACACCCGGGATGCAGATTAGAGGGTTCGTG

TACTTCGTTGAAACACTAGCGAGGAGCATCTGTGAGAAACTAGAGCAATCTGGGCTCCCT

GTTGGAGGGAATGAGAAGAAGGCTAAATTGGCAAATGTTGTGAGAAAAATGATGACTAAC

TCACAAGATACAGAGCTCTCCTTTACAATTACTGGAGACAACACCAAATGGAATGAGAAT

CAAAACCCTCGGATGTTTTTGGCAATGATAACATATATCACAAGAAACCAACCTGAATGG

TTTAGAAATGTCTTAAGCATTGCCCCTATAATGTTCTCAAACAAAATGGCGAGATTAGGG

AAAGGATACATGTTTGAAAGTAAGAGCATGAAGCTAAGAACACAAATACCAGCAGAGATG

CTTATAAATATTGATCTGAAGTATTTCAACGAACCAACGAGAAAGAAAATCGAGAAAATA

AGACCTCTGCTGATTGATGGCACGGCCTCATTGAGTCCTGGGATGATGATGGGCATGTTC

AATATGCTGAGCACAGTATTAGGGGTCTCAATCCTGAATCTCGGGCAAAAGAGGTACACC

AAAACCACATACTGGTGGGATGGACTTCAATCCTCTGATGATTTCGCTCTCATAGTGAAT

GCACCGAATCATGAGGGGATACAAGCAGGAGTGGATAGATTCTATAGGACCTGCAAACTG

GTTGGGATCAACATGAGCAAAAAGAAGTCTTACATAAACCGAACAGGAACATTTGAGTTC

ACAAGTTTTTTCTACCGCTATGGATTTGTAGCTAACTTCAGTATGGAATTACCCAGCTTT

GGAGTGTCTGGAATCAATGAATCAGCTGACATGAGCATTGGAGTTACAGTGATAAAAAAC

AATATGATAAACAATGATCTTGGACCAGCAACAGCTCAAATGGCTCTTCAGTTATTCATC

AAAGACTATAGGTACACATACCGATGCCACAGGGGTGATACACAAATTCAAACGAGGAGA

TCATTCGAGCTGAAGAAGCTGTGGGAGCAGACCCGTTCAAAGGCAGGGCTGTTGATATCA

GACGGGGGGCCAAACCTATACAACATTCGGAATCTCCACATCCCAGAGGTCTGCTTGAAG

TGGGAGCTGATGGATGAAGACTACCAAGGCAGGCTGTGCAATCCTCTGAATCCATTTGTC

AGTCATAAAGAGATTGAGTCCGTAAACAATGCTGTAGTAATGCCCGCCCATGGCCCGGCC

AAGAGCATGGAATATGATGCTGTTGCGACCACACACTCGTGGATTCCTAAGAGGAACCGT

TCCATTCTCAATACCAGCCAAAGGGGAATTCTTGAGGATGAGCAGATGTACCAAAAGTGC

TGTAGTCTATTCGAGAAATTCTTCCCCAGCAGTTCATACAGGAGGCCAGTTGGAATTTCC

AGCATGGTGGAGGCCATGGTGTCTAGGGCCCGAATTGATGCACGCATCGACTTCGAATCT

GGAAGGATTAAGAAAGAAGAGTTTGCTGAGATCATGAAGATCTGTTCCACCATTGAAGAG

CTCAGACGGCAAAAATAGTGAATTTAGCTTGTCCTTCATGAAAAAATGCCTTGTTTCTAC

T

>A_swan_Tumen_1479-2_2020_EPI1814682

------------CAAACTATTTGAATGGATGTCAATCCGACTTTACTTTTCTTAAAAGTG

CCAGCGCAAAATGCTATAAGTACTACATTCCCTTACACTGGAGATCCCCCATACAGCCAT

GGAACAGGAACAGGGTATACCATGGACACAGTAAACAGAACACATCAATACTCAGAAAAG

GGAAAGTGGACAACAAACACAGAAACCGGAGCACCCCAACTCAACCCAATTGATGGACCA

TTACCAGAGGACAATGAGCCAAGCGGATATGCACAAACTGATTGCGTGTTGGAAGCAATG

GCTTTCCTTGAAGAATCCCACCCAGGGATATTTGAGAACTCTTGTCTTGAAGCGATGGAA

ATCGTTCAGCAAACAAGAGTGGACAAACTAACCCAAGGTCGCCAGACTTATGACTGGACA

CTGAACAGAAACCAACCAGCTGCAACCTCTTTGGCCAACACTATAGAGGTGTTCAGATCG

AATGGTCTGACAGCCAATGAATCAGGGAGACTGATAGATTTTCTCAGGGATGTGATGGAA

TCAATGGATAAAGAAGAGATGGAAGTAACAACACATTTCCAGAGAAAAAGAAGAGTGAGG

GACAACATGACTAAGAAGATGGTCACACAAAGAACAATAGGGAAGAAGAAGCAGAGGCTG

AACAAGAGGAGTTACTTAATAAGGGCACTGACATTGAATACAATGACCAAAGATGCAGAA

AGAGGCAAGTTGAAGAGACGGGCAATTGCAACACCCGGGATGCAGATTAGAGGATTCGTG

TACTTCGTTGAAACACTAGCGAGGAGCATCTGTGAGAAACTAGAGCAATCTGGGCTCCCT

GTTGGAGGGAATGAGAAGAAGGCTAAATTGGCAAATGTTGTGAGAAAAATGATGACTAAC

TCACAAGATACAGAGCTCTCCTTTACAATTACTGGAGACAACACCAAATGGAATGAGAAT

CAAAACCCTCGGATGTTTTTGGCAATGATAACATATATCACAAGACACCAACCTGAATGG

TTTAGAAATGTCTTAAGCATTGCCCCTATAATGTTCTCAAACAAAATGGCGAGATTAGGG

AAAGGATACATGTTTGAAAGTAAGAGCATGAAGCTAAGAACACAAATACCAGCAGAGATG

CTTACAAATATTGATCTGAAGTATTTCAACGAACCAACGAGAAAGAAAATCGAGAAAATA

AGACCTCTACTAATTGATGGCACGGCCTCATTGAGTCCTGGGATGATGATGGGCATGTTC

AATATGCTGAGCACAGTATTAGGGGTCTCAATCCTGAATCTCGGGCAAAAGAGGTACACC

AAAACCACATACTGGTGGGATGGACTTCAATCCTCTGATGATTTCGCTCTCATAGTGAAT

GCACCGAATCATGAGGGGATACAAGCAGGAGTGGATAGATTCTATAGGACCTGCAAACTG

GTTGGGATCAACATGAGCAAAAAGAAGTCTTACATAAACCGAACAGGAACATTTGAGTTC

ACAAGTTTTTTCTACCGCTATGGATTTGTAGCCAACTTCAGTATGGAATTACCCAGCTTT

GGAGTGTCTGGAATCAATGAATCAGCTGACATGAGCATTGGAGTTACAGTGATAAAAAAC

AATATGATAAACAATGATCTTGGACCAGCAACAGCTCAAATGGCTCTTCAGTTATTCATC

AAAGACTATAGGTACACATACCGATGCCACAGGGGTGATACACAAATTCAAACGAGGAGA

TCATTCGAGCTGAAGAAGCTGTGGGAGCAGACCCGTTCAAAGGCAGGGCTGTTGATATCA

GACGGGGGGCCAAACCTATACAACATTCGGAATCTCCACATCCCAGAGGTCTGCTTGAAG

TGGGAGCTGATGGATGAAGACTACCAAGGCAGGCTGTGCAATCCTCTGAATCCATTTGTC

AGTCATAAAGAGATTGAGTCCGTAAACAATGCTGTAGTAATGCCCGCCCATGGCCCGGCC

AAGAGCATGGAATATGATGCTGTTGCGACCACACACTCGTGGATTCCTAAGAGGAACCGT

TCCATTCTCAATACCAGCCAAAGGGGAATTCTTGAGGATGAGCAGATGTACCAAAAGTGC

TGTAGTCTATTCGAGAAATTCTTCCCCAGCAGTTCATACAGGAGGCCAGTTGGAATTTCC

AGCATGGTGGAGGCCATGGTGTCTAGGGCCCGAATTGATGCACGCATCGACTTCGAATCT

GGAAGGATTAAGAAAGAAGAGTTTGCTGAGATCATGAAGATCTGTTCCACCATTGAAGAG

CTCAGACGGCAAAAATAGTGAATTTAGCTTGTTCTTCATGAAAAAATG------------

-

>A_Whooper_swan_Mongolia_24_2020_EPI1831870

AGCGAAAGCAGGCAAACCATTTGAATGGATGTCAACCCGACTTTACTTTTCTTGAAAGTG

CCAGTGCAAAATGCTATAAGTACCACATTCCCTTATACTGGAGACCCTCCATACAGCCAT

GGAACAGGAACAGGGTACACCATGGACACAGTAAACAGAACACACCAATACTCAGAAAGG

GGGGAGTGGACAACAAACACAGAGACCGGGGCACCCCAACTCAACCCGATTGATGGACCA

TTGCCTGAGGATAACGAGCCCAGTGGGTATGCGCAAACAGATTGTGTGTTAGAAGCAATG

GCTTTCCTTGAAGAATCCCACCCAGGAATCTTTGAAAATTCATGCCTTGAAACGATGGAA

ATTGTCCAACAAACGAGAGTGGACAGACTGACCCAAGGTCGCCAGACTTATGACTGGACA

TTGAATAGAAACCAGCCGGCTGCAACTGCTCTGGCCAACACTATAGAAGTCTTCAGATCA

AACGGTCTGACAGCGAATGAGTCCGGACGCCTAATAGATTTCCTCAAGGATGTGATAGAA

TCAATGGATAAAGAAGTAATGGAAATAACAACACATTTCCAGAGAAAGAGAAGAGTAAGG

GACAACATGACCAAGAAAATGGTAACGCAAAGAACAATCGGGAAGAAGAAGCAAAGGCTG

AACAAGAGGAACTACCTGATAAGAGCGCTGACACTGAACACAATGACCAAGGATGCGGAA

AGAGGCAAATTGAAGAGGCGTGCAATTGCAACACCCGGAATGCAAATCAGAGGATTTGTA

TATTTCGTAGAAACACTAGCAAGGAGTATCTGTGAGAAACTTGAGCAATCTGGGCTCCCA

GTCGGAGGGAATGAGAAGAAAGCTAAACTGGCAAACGTCGTGAGGAAGATGATGACCAAC

TCACAGGATACTGAACTCTCCTTTACAATTACTGGAGACAATACAAAATGGAATGAGAAT

CAAAATCCTAGGATGTTTCTGGCAATGATAACGTACATCACAAGGAACCAGCCAGAATGG

TTCCGAAATGTTCTAAGCATTGCCCCTATAATGTTCTCAAACAAAATGGCGAGGCTCGGG

AAAGGATACATGTTCGAAAGTAAGAGCATGAAGTTGCGAACACAAATACCAGCAGAAATG

CTTGCAAACATTGACCTTAAATACTTCAATGAATCAACAAAAAAGAAAATTGAGAGAATA

AGACCCCTATTAATAGATGGTACAGCCTCGTTGAGCCCTGGGATGATGATGGGCATGTTC

AACATGCTGAGTACAGTCCTAGGGGTTTCAATCCTAAATCTTGGACAGAAAAGATACACC

AAAACCACATATTGGTGGGACGGGCTTCAATCCTCTGATGACTTCGCTCTCATTATAAAT

GCCCCGAATCATGAAGGGATACAAGCAGGAGTGGATAGGTTTTATAGAACTTGTAAACTA

GTTGGGATCAATATGAGCAAGAAGAAGTCTTACATAAATCGGACCGGGACATTTGAATTT

ACGAGCTTTTTCTATCGCTATGGGTTCGTAGCCAATTTCAGTATGGAGCTGCCCAGTTTT

GGAGTATCTGGAATTAATGAATCAGCCGACATGAGCATTGGTGTTACAGTGATAAAGAAC

AATATGATAAACAATGACCTTGGGCCAGCAACAGCTCAGATGGCTCTTCAGCTATTCATC

AAAGATTACAGATACACATACCGATGCCACAGAGGAGATACTCAAATCCAAACGAGGAGA

TCATTCGAGCTGAAGAAGCTATGGGAACAAACCCGTTCAAAGGCAGGACTATTGGTTTCA

GATGGAGGACCAAACCTGTACAATATCCGAAATCTCCATATTCCTGAGGTCTGCTTGAAA

TGGGAATTGATGGATGAAGACTACCAGGGTAGACTGTGCAATCCTCTGAATCCATTCGTC

AACCATAGGGAAATTGAATCTGTCAACAATGCTATAGTAATGCCAGCTCATGGTCCGGCC

AAGGGTATAGAATATGATGCCGTAGCAACCACACACTCATGGATTCCTAAAAGGAATCGT

TCCATTCTTAATACAAGTCAGAGGGGGATTCTTGAGGATGAACAGATGTATCAAAAGTGC

TGCAGTCTATTCGAGAAATTCTTCCCTAGTAGTTCGTATCGGAGACCAGTTGGAATTTCC

AGTATGGTGGAGGCCATGGTTTCTCGGGCCAGAATTGACGCACGAATTGATTTCGAGTCT

GGAAGGATTAAGAAGGAAGAGTTTGCTGAGATCATGAAGATCTGTTCCACCATTGAAGAG

CTCAGACGGCAAAAATAGTGAATTTGGCTTGTCCTTCGTGAAAAA---------------

-

>A_Whooper_swan_Mongolia_25_2020_EPI1831878

AGCGAAAGCAGGCAAACCATTTGAATGGATGTCAACCCGACTTTACTTTTCTTGAAAGTG

CCAGTGCAAAATGCTATAAGTACCACATTCCCTTATACTGGAGACCCTCCATACAGCCAT

GGAACAGGAACAGGGTACACCATGGACACAGTAAACAGAACACACCAATACTCAGAAAGG

GGGGAGTGGACAACAAACACAGAGACCGGGGCACCCCAACTCAACCCGATTGATGGACCA

TTGCCTGAGGATAACGAGCCCAGTGGGTATGCGCAAACAGATTGTGTGTTAGAAGCAATG

GCTTTCCTTGAAGAATCCCACCCAGGAATCTTTGAAAATTCATGCCTTGAAACGATGGAA

ATTGTCCAACAAACGAGAGTGGACAGACTGACCCAAGGTCGCCAGACTTATGACTGGACA

TTGAATAGAAACCAGCCGGCTGCAACTGCTCTGGCCAACACTATAGAAGTCTTCAGATCA

AACGGTCTGACAGCGAATGAGTCCGGACGCCTAATAGATTTCCTCAAGGATGTGATAGAA

TCAATGGATAAAGAAGTAATGGAAATAACAACACATTTCCAGAGAAAGAGAAGAGTAAGG

GACAACATGACCAAGAAAATGGTAACGCAAAGAACAATCGGGAAGAAGAAGCAAAGGCTG

AACAAGAGGAACTACCTGATAAGAGCGCTGACACTGAACACAATGACAAAGGATGCGGAA

AGAGGCAAATTGAAGAGGCGTGCAATTGCAACACCCGGAATGCAAATCAGAGGATTTGTA

TATTTCGTAGAAACACTAGCAAGGAGTATCTGTGAGAAACTTGAGCAATCTGGGCTCCCA

GTCGGAGGGAATGAGAAGAAAGCTAAACTGGCAAACGTCGTGAGGAAGATGATGACCAAC

TCACAGGATACTGAACTCTCCTTTACAATTACTGGAGACAATACAAAATGGAATGAGAAT

CAAAATCCTAGGATGTTTCTGGCAATGATAACGTACATCACAAGGAACCAGCCAGAATGG

TTCCGAAATGTTCTAAGCATTGCCCCTATAATGTTCTCAAACAAAATGGCGAGGCTCGGG

AAAGGATACATGTTCGAAAGTAAGAGCATGAAGTTGCGAACACAAATACCAGCAGAAATG

CTTGCAAACATTGACCTTAAATACTTCAATGAATCAACAAAAAAGAAAATTGAGAGAATA

AGACCCCTATTAATAGATGGTACAGCCTCATTGAGCCCTGGGATGATGATGGGCATGTTC

AACATGCTGAGTACAGTCCTAGGGGTTTCAATCCTAAATCTTGGACAGAAAAGATACACC

AAAACCACATATTGGTGGGACGGGCTTCAATCCTCTGATGACTTCGCTCTCATTATAAAT

GCCCCGAATCATGAAGGGATACAAGCAGGAGTGGATAGGTTTTATAGAACTTGTAAACTA

GTTGGGATCAATATGAGCAAGAAGAAGTCTTACATAAATCGGACCGGGACATTTGAATTT

ACGAGCTTTTTCTATCGCTATGGGTTCGTAGCCAATTTCAGTATGGAGCTGCCCAGTTTT

GGAGTATCTGGAATTAATGAATCAGCCGACATGAGCATTGGTGTTACAGTGATAAAGAAC

AATATGATAAACAATGACCTTGGGCCAGCAACAGCTCAGATGGCTCTTCAGCTATTCATC

AAAGATTACAGATACACATACCGATGCCACAGAGGAGATACTCAAATCCAAACGAGGAGA

TCATTCGAGCTGAAGAAGCTATGGGAACAAACCCGTTCAAAGGCAGGACTATTGGTTTCA

GATGGAGGACCAAACCTGTACAATATCCGAAATCTCCATATTCCTGAGGTCTGCTTGAAA

TGGGAATTGATGGATGAAGACTACCAGGGTAGACTGTGCAATCCTCTGAATCCATTCGTC

AACCATAGGGAAATTGAATCTGTCAACAATGCTATAGTAATGCCAGCTCATGGTCCGGCC

AAGGGTATAGAATATGATGCCGTAGCAACCACACACTCATGGATTCCTAAAAGGAATCGT

TCCATTCTTAATACAAGTCAGAGGGGGATTCTTGAGGATGAACAGATGTATCAAAAGTGC

TGCAGTCTATTCGAGAAATTCTTCCCTAGTAGTTCGTATCGGAGACCAGTTGGAATTTCC

AGTATGGTGGAGGCCATGGTTTCTCGGGCCAGAATTGACGCACGAATTGATTTCGAGTCT

GGAAGGATTAAGAAGGAAGAGTTTGCTGAGATCATGAAGATCTGTTCCACCATTGAAGAG

CTCAGACGGCAAAAATAGTGAATTTGGCTTGTCCTTCGTGAAAAA---------------

-

>A_chicken_Kazakhstan_Kn-3_2020_EPI1839259

------------------------ATGGATGTCAATCCGACTTTACTTTTCTTAAAAGTG

CCAGCGCAAAATGCTATAAGTACTACATTCCCTTACACTGGAGATCCCCCATACAGCCAT

GGAACAGGAACAGGGTATACCATGGACACAGTAAACAGAACACATCAATACTCAGAAAAG

GGAAAGTGGACAACAAACACAGAAACCGGAGCACCCCAACTCAACCCAATTGATGGACCA

TTACCAGAGGACAATGAGCCAAGCGGATATGCACAAACTGATTGCGTGTTGGAAGCAATG

GCTTTCCTTGAAGAATCCCACCCAGGGATATTTGAAAACTCTTGTCTTGAAGCGATGGAA

ATCGTTCAGCAAACAAGAGTGGACAAACTAACCCAAGGTCGCCAGACTTATGACTGGACA

CTGAACAGAAACCAACCAGCTGCAACCTCTTTGGCCAACACTATAGAGGTGTTCAGATCG

AATGGTCTGACAGCCAATGAATCAGGGAGACTGATAGATTTTCTCAGGGATGTGATGGAA

TCAATGGATAAAGAAGAGATGGAAGTAACAACACATTTCCAGAGAAAAAGAAGAGTGAGG

GACAACATGACTAAGAAGATGGTCACACAAAGAACAATAGGGAAGAAGAAGCAGAGGCTG

AACAAGAGGAGTTACTTAATAAGAGCACTGACATTGAATACAATGACCAAAGATGCAGAA

AGAGGCAAGTTGAAGAGACGGGCAATTGCAACACCCGGGATGCAGATTAGAGGATTCGTG

TACTTCGTTGAAACACTAGCGAGGAGCATCTGTGAGAAACTAGAGCAATCTGGGCTCCCT

GTTGGAGGGAATGAGAAGAAGGCTAAATTGGCAAATGTTGTGAGAAAAATGATGACTAAC

TCACAAGATACAGAGCTCTCCTTTACAATTACTGGAGACAACACCAAATGGAATGAGAAT

CAAAACCCTCGGATGTTTTTGGCAATGATAACATATATCACAAGAAACCAACCTGAATGG

TTTAGAAATGTCTTAAGCATTGCCCCTATAATGTTCTCAAACAAAATGGCGAGATTAGGG

AAAGGATACATGTTTGAAAGTAAGAGCATGAAGCTAAGAACACAAATACCAGCAGAGATG

CTTACAAATATTGATCTGAAGTATTTCAACGAACCAACGAGAAAGAAAATCGAGAAAATA

AGACCTCTGCTGATTGATGGCACGGCCTCATTGAGTCCTGGGATGATGATGGGCATGTTC

AATATGCTGAGCACAGTATTAGGGGTCTCAATCCTGAATCTCGGGCAAAAGAGGTACACC

AAAACCACATACTGGTGGGATGGACTTCAATCCTCTGATGATTTCGCTCTCATAGTGAAT

GCACCGAATCATGAGGGGATACAAGCAGGAGTGGATAGATTCTATAGGACCTGCAAACTG

GTTGGGATCAACATGAGCAAAAAGAAGTCTTACATAAACCGAACAGGAACATTTGAGTTC

ACAAGTTTTTTCTACCGCTATGGATTTGTAGCCAACTTCAGTATGGAATTACCCAGCTTT

GGAGTGTCTGGAATCAATGAATCAGCTGACATGAGCATTGGAGTTACAGTGATAAAAAAC

AATATGATAAACAATGATCTTGGACCAGCAACAGCTCAAATGGCTCTTCAGTTATTCATC

AAAGACTATAGGTACACATACCGATGCCACAGGGGTGATACACAAATTCAAACGAGGAGA

TCATTCGAGCTGAAGAAGCTGTGGGAGCAGACCCGTTCAAAGGCAGGGCTGTTGATATCA

GACGGGGGGCCAAATCTATACAACATTCGGAATCTCCACATCCCAGAGGTCTGCTTGAAG

TGGGAGCTGATGGATGAAGACTACCAAGGCAGGCTGTGCAATCCTCTGAATCCATTTGTC

AGTCATAAAGAGATTGAGTCCGTAAACAATGCTGTAGTAATGCCCGCCCATGGCCCGGCC

AAGAGCATGGAATATGATGCTGTTGCGACCACACACTCGTGGATTCCTAAGAGGAACCGT

TCCATTCTCAATACCAGCCAAAGGGGAATTCTTGAGGATGAGCAGATGTACCAAAAGTGC

TGTAGTCTATTCGAGAAATTCTTCCCCAGCAGTTCATACAGGAGGCCAGTTGGAATTTCC

AGCATGGTGGAGGCCATGGTGTCTAGGGCCCGAATTGATGCACGCATCGACTTCGAATCT

GGAAGGATTAAGAAAGAAGAGTTTGCTGAGATCATGAAGATCTGTTCCACCATTGAAGAG

CTCAGACGGCAAAAATAG------------------------------------------

-

>A_chicken_Kazakhstan_Kn-6_2020_EPI1839267

------------------------ATGGATGTCAATCCGACTTTACTTTTCTTAAAAGTG

CCAGCGCAAAATGCTATAAGTACTACATTCCCTTACACTGGAGATCCCCCATACAGCCAT

GGAACAGGAACAGGGTATACCATGGACACAGTAAACAGAACACATCAATACTCAGAAAAG

GGAAAGTGGACAACAAACACAGAAACCGGAGCACCCCAACTCAACCCAATTGATGGACCA

TTACCAGAGGACAATGAGCCAAGCGGATATGCACAAACTGATTGCGTGTTGGAAGCAATG

GCTTTCCTTGAAGAATCCCACCCAGGGATATTTGAAAACTCTTGTCTTGAAGCGATGGAA

ATCGTTCAGCAAACAAGAGTGGACAAACTAACCCAAGGTCGCCAGACTTATGACTGGACA

CTGAACAGAAACCAACCAGCTGCAACCTCTTTGGCCAACACTATAGAGGTGTTCAGATCG

AATGGTCTGACAGCCAATGAATCAGGGAGACTGATAGATTTTCTCAGGGATGTGATGGAA

TCAATGGATAAAGAAGAGATGGAAGTAACAACACATTTCCAGAGAAAAAGAAGAGTGAGG

GACAACATGACTAAGAAGATGGTCACACAAAGAACAATAGGGAAGAAGAAGCAGAGGCTG

AACAAGAGGAGTTACTTAATAAGAGCACTGACATTGAATACAATGACCAAAGATGCAGAA

AGAGGCAAGTTGAAGAGACGGGCAATTGCAACACCCGGGATGCAGATTAGAGGATTCGTG

TACTTCGTTGAAACACTAGCGAGGAGCATCTGTGAGAAACTAGAGCAATCTGGGCTCCCT

GTTGGAGGGAATGAGAAGAAGGCTAAATTGGCAAATGTTGTGAGAAAAATGATGACTAAC

TCACAAGATACAGAGCTCTCCTTTACAATTACTGGAGACAACACCAAATGGAATGAGAAT

CAAAACCCTCGGATGTTTTTGGCAATGATAACATATATCACAAGAAACCAACCTGAATGG

TTTAGAAATGTCTTAAGCATTGCCCCTATAATGTTCTCAAACAAAATGGCGAGATTAGGG

AAAGGATACATGTTTGAAAGTAAGAGCATGAAGCTAAGAACACAAATACCAGCAGAGATG

CTTACAAATATTGATCTGAAGTATTTCAACGAACCAACGAGAAAGAAAATCGAGAAAATA

AGACCTCTGCTGATTGATGGCACGGCCTCATTGAGTCCTGGGATGATGATGGGCATGTTC

AATATGCTGAGCACAGTATTAGGGGTCTCAATCCTGAATCTCGGGCAAAAGAGGTACACC

AAAACCACATACTGGTGGGATGGACTTCAATCCTCTGATGATTTCGCTCTCATAGTGAAT

GCACCGAATCATGAGGGGATACAAGCAGGAGTGGATAGATTCTATAGGACCTGCAAACTG

GTTGGGATCAACATGAGCAAAAAGAAGTCTTACATAAACCGAACAGGAACATTTGAGTTC

ACAAGTTTTTTCTACCGCTATGGATTTGTAGCCAACTTCAGTATGGAATTACCCAGCTTT

GGAGTGTCTGGAATCAATGAATCAGCTGACATGAGCATTGGAGTTACAGTGATAAAAAAC

AATATGATAAACAATGATCTTGGACCAGCAACAGCTCAAATGGCTCTTCAGTTATTCATC

AAAGACTATAGGTACACATACCGATGCCACAGGGGTGATACACAAATTCAAACGAGGAGA

TCATTCGAGCTGAAGAAGCTGTGGGAGCAGACCCGTTCAAAGGCAGGGCTGTTGATATCA

GACGGGGGGCCAAATCTATACAACATTCGGAATCTCCACATCCCAGAGGTCTGCTTGAAG

TGGGAGCTGATGGATGAAGACTACCAAGGCAGGCTGTGCAATCCTCTGAATCCATTTGTC

AGTCATAAAGAGATTGAGTCCGTAAACAATGCTGTAGTAATGCCCGCCCATGGCCCGGCC

AAGAGCATGGAATATGATGCTGTTGCGACCACACACTCGTGGATTCCTAAGAGGAACCGT

TCCATTCTCAATACCAGCCAAAGGGGAATTCTTGAGGATGAGCAGATGTACCAAAAGTGC

TGTAGTCTATTCGAGAAATTCTTCCCCAGCAGTTCATACAGGAGGCCAGTTGGAATTTCC

AGCATGGTGGAGGCCATGGTGTCTAGGGCCCGAATTGATGCACGCATCGACTTCGAATCT

GGAAGGATTAAGAAAGAAGAGTTTGCTGAGATCATGAAGATCTGTTCCACCATTGAAGAG

CTCAGACGGCAAAAATAG------------------------------------------

-

>A_Muscovy_duck_China_FJFZ21_H5N6_2020_EPI1841918

------------------------ATGGATGTCAACCCGACTTTACTTTTCTTGAAAGTG

CCAGTGCAAAATGCTATAAGTACCACATTCCCTTATACTGGAGACCCTCCATACAGCCAT

GGAACAGGAACAGGGTACACCATGGACACAGTGAACAGAACACACCAATACTCAGAAAAG

GGGGAGTGGACAACAAACACAGAGACCGGGGCACCCCAACTCAACCCGATTGATGGACCA

TTGCCTGAGGATAACGAGCCCAGTGGGTATGCGCAAACAGATTGTGTGTTAGAAGCAATG

GCTTTCCTTGAAGAGTCCCACCCAGGAATCTTTGAAAATTCGTGCCTTGAAACGATGGAA

ATTGTCCAACAAACGAGAGTGGACAGACTGACCCAAGGTCGCCAGACTTATGACTGGACA

TTGAACAGAAACCAACCGGCTGCAACTGCTCTGGCCAACACTATAGAAGTCTTCAGATCA

AACGGTCTGACAGCGAATGAGTCCGGACGCCTAATAGATTTCCTCAAGGATGTGATGGAA

TCAATGGATAAAGAAGTAATGGAAATAACGACACATTTCCAGAGAAAGAGAAGAGTAAGG

GACAACATGACCAAGAAAATGGTAACGCAAAGAACAATCGGGAAGAAGAAGCAAAGGCTG

AACAAGAGGAGCTACCTGATAAGAGCGCTGACACTGAACACAATGACCAAGGATGCGGAA

AGAGGCAAATTGAAGAGGCGTGCAATTGCAACACCCGGAATGCAAATCAGAGGATTTGTA

TACTTCGTAGAAACACTAGCAAGGAGTATCTGTGAGAAACTAGAGCAATCTGGGCTCCCA

GTCGGAGGGAATGAGAAGAAAGCTAAACTGGCAAACGTCGTGAGGAAGATGATGACCAAC

TCACAGGATACTGAACTCTCCTTTACAATTACTGGAGACAATACAAAATGGAATGAGAAT

CAAAATCCTAGGATGTTTCTGGCAATGATAACGTACATCACAAGGAACCAGCCAGAATGG

TTCCGAAATGTTCTAAGCATTGCCCCTATAATGTTCTCAAACAAAATGGCGAGGCTAGGG

AAAGGATACATGTTCGAAAGTAAGAGCATGAAGTTGCGAACACAAATACCAGCAGAAATG

CTTGCAAACATTGACCTTAAATACTTCAATGAATCAACAAAAAAGAAAATTGAGAAAATA

AGACCTCTATTAATAGATGGTACAGCCTCATTGAGCCCTGGGATGATGATGGGCATGTTC

AACATGCTGAGTACAGTCCTAGGGGTTTCAATCCTAAATCTCGGGCAGAAAAGATACACC

AAAACCACATATTGGTGGGACGGGCTTCAATCCTCTGATGACTTCGCTCTCATTATAAAT

GCCCCGAATCATGAAGGAATACAAGCAGGGGTGGATAGGTTTTATAGAACTTGTAAACTA

GTTGGGATCAATATGAGCAAGAAGAAGTCTTACATAAATCGGACCGGGACATTTGAATTT

ACGAGCTTTTTCTATCGCTATGGATTCGTAGCCAATTTCAGTATGGAGCTGCCCAGTTTT

GGAGTGTCTGGAATTAATGAATCGGCCGACATGAGCATTGGTGTTACAGTGATAAAGAAC

AATATGATAAACAACGACCTTGGGCCAGCAACAGCTCAGATGGCTCTTCAGCTATTCATC

AAAGACTACAGATACACATACCGATGCCACAGAGGAGATACTCAAATCCAAACGAGGAGA

TCATTCGAGCTGAAGAAGCTATGGGAACAAACCCGTTCAAAGGCAGGACTATTGGTTTCA

GATGGAGGACCAAACCTGTACAATATCCGAAATCTCCATATTCCTGAGGTCTGCTTGAAA

TGGGAATTGATGGATGAAGACTACCAGGGTAGACTGTGCAATCCTCTGAATCCATTCGTC

AGCCATAGGGAAATTGAATCTGTCAACAATGCTATAGTAATGCCAGCTCATGGTCCGGCC

AAGGGTATAGAATATGATGCCGTAGCAACCACACATTCATGGATACCTAAAAGGAATCGT

TCCATTCTTAATACAAGTCAGAGGGGGATTCTTGAGGATGAACAGATGTACCAAAAGTGC

TGCAGTTTATTCGAGAAATTCTTCCCCAGTAGTTCGTATCGGAGACCAGTTGGAATTTCC

AGTATGGTGGAGGCCATGGTTTCTCGGGCCAGAATTGACGCACGAATTGATTTCGAGTCT

GGAAGGATTAAAAAGGAAGAGTTTGCTGAGATCATGAAGATCTGTTCCACCATTGAAGAG

CTCAGACGGCAAAGATAG------------------------------------------

-

>A_mute_swan_Czech_Republic_1410-2_2021_EPI1843604

------------------------ATGGATGTCAATCCGACTTTACTTTTCTTAAAAGTG

CCAGCGCAAAATGCTATAAGTACTACATTCCCTTACACTGGAGATCCCCCATACAGCCAT

GGAACAGGAACAGGGTATACCATGGACACAGTAAACAGAACACATCAATACTCAGAAAAG

GGAAAGTGGACAACAAACACAGAAACCGGAGCACCCCAACTCAACCCAATTGATGGACCA

TTACCAGAGGACAATGAGCCAAGCGGATATGCACAAACTGATTGCGTGTTGGAAGCAATG

GCTTTCCTTGAAGAATCCCACCCAGGGATATTTGAAAACTCTTGTCTTGAAGCAATGGAA

ATCGTTCAGCAAACAAGAGTGGACAAACTAACCCAAGGTCGCCAGACTTATGACTGGACA

CTGAACAGAAACCAACCAGCTGCAACCTCTTTGGCCAACACTATAGAGGTGTTCAGATCG

AATGGTCTGACAGCCAATGAATCAGGGAGACTGATAGATTTTCTCAGGGATGTGATGGAA

TCAATGGATAAAGAAGAGATGGAAATAACAACACATTTCCAGAGAAAAAGAAGAGTGAGG

GACAACATGACTAAGAAGATGGTCACACAAAGAACAATAGGGAAGAAGAAGCAGAGGCTG

AACAAGAGGAGTTACTTAATAAGAGCACTGACATTGAATACAATGACCAAAGATGCAGAA

AGAGGCAAGTTGAAGAGACGGGCAATTGCAACACCCGGGATGCAGATTAGAGGATTCGTG

TACTTCGTTGAAACACTAGCGAGGAGCATCTGTGAGAAACTAGAGCAATCTGGGCTCCCT

GTTGGAGGGAATGAGAAAAAGGCTAAATTGGCAAATGTTGTGAGAAAAATGATGACTAAC

TCACAAGATACAGAGCTCTCCTTTACAATTACTGGAGACAACACCAAATGGAATGAGAAT

CAAAACCCTCGGATGTTTTTGGCAATGATAACATATATCACAAGAAACCAACCTGAATGG

TTTAGAAATGTCTTAAGCATTGCCCCTATAATGTTCTCAAACAAAATGGCGAGATTAGGG

AAAGGATACATGTTTGAAAGTAAGAGTATGAAGCTAAGAACACAAATACCAGCAGAGATG

CTTACAAATATTGATCTGAAGTATTTCAACGAACCAACGAGAAAGAAAATCGAGAAAATA

AGACCTCTGCTGATTGATGGCACGGCCTCATTGAGTCCTGGGATGATGATGGGCATGTTC

AATATGCTGAGCACAGTATTAGGGGTCTCAATCCTGAATCTCGGGCAAAAGAGGTACACC

AAAACCACATACTGGTGGGATGGACTTCAATCCTCTGATGATTTCGCTCTCATAGTGAAT

GCACCGAATCATGAGGGGATACAAGCAGGAGTGGATAGATTCTATAGGACCTGCAAACTG

GTTGGGATCAACATGAGCAAAAAGAAGTCTTACATAAACCGAACAGGAACATTTGAGTTC

ACAAGTTTTTTCTACCGCTATGGATTTGTAGCCAACTTCAGTATGGAATTACCCAGCTTT

GGAGTGTCTGGAATCAATGAATCAGCTGACATGAGCATTGGAGTTACAGTGATAAAAAAC

AATATGATAAACAATGATCTTGGACCAGCAACAGCTCAAATGGCTCTTCAGTTATTCATC

AAAGACTATAGGTACACATACCGATGCCACAGGGGTGATACACAAATTCAAACGAGGAGA

TCATTCGAGCTGAAGAAGCTGTGGGAGCAGACCCGTTCAAAGGCAGGGCTGTTGATATCA

GACGGGGGGCCAAACCTATACAACATTCGGAATCTCCACATCCCAGAGGTCTGCTTGAAG

TGGGAGCTGATGGATGAAGACTACCAAGGCAGGCTGTGCAATCCTCTGAATCCATTTGTC

AGTCATAAAGAGATTGAGTCCGTAAACAATGCTGTAGTAATGCCCGCCCATGGCCCGGCC

AAGAGCATGGAATATGATGCTGTTGCGACCACACACTCGTGGATTCCTAAGAGGAACCGT

TCCATCCTCAATACCAGCCAAAGGGGAATTCTTGAGGATGAGCAGATGTACCAAAAGTGC

TGTAGTCTATTCGAGAAATTCTTCCCCAGCAGTTCATACAGGAGGCCAGTTGGAATTTCC

AGCATGGTGGAGGCCATGGTGTCTAGGGCCCGAATTGATGCACGCATCGACTTCGAATCT

GGAAGGATTAAGAAAGAAGAGTTTGCTGAGATCATGAAGATCTGTTCCACCATTGAAGAG

CTCAGACGGCAAAAATAGTGAATTTAGCTTGTCCTTCATGAAA-----------------

-

>A_chicken_Czech_Republic_1566-1_2021_EPI1844081

------------------------ATGGATGTCAATCCGACTTTACTTTTCTTAAAAGTG

CCAGCGCAAAATGCTATAAGTACTACATTCCCTTACACTGGAGATCCTCCATACAGCCAT

GGAACAGGAACAGGGTATACCATGGACACAGTAAACAGAACACATCAATACTCAGAAAAG

GGAAAGTGGACAACAAACACAGAAACCGGAGCACCCCAACTCAACCCAATTGATGGACCA

TTACCAGAGGACAATGAGCCAAGCGGATATGCACAAACTGATTGCGTGTTGGAAGCAATG

GCTTTCCTTGAAGAATCCCACCCAGGGATATTTGAAAACTCTTGTCTTGAAGCGATGGAA

ATCGTTCAGCAAACAAGAGTGGACAAACTAACCCAAGGTCGCCAGACTTATGACTGGACA

CTGAACAGAAACCAACCAGCTGCAACCTCTTTGGCCAACACTATAGAGGTGTTCAGATCG

AATGGTCTGACAGCCAATGAATCAGGGAGACTGATAGATTTTCTCAGGGATGTGATGGAA

TCAATGGATAAAGAAGAGATGGAAGTAACAACACATTTCCAGAGAAAAAGAAGAGTGAGG

GACAACATGACTAAGAAGATGGTCACACAAAGAACAATAGGGAAGAAGAAGCAGAGGCTG

AACAAGAGGAGTTACTTAATAAGAGCACTGACATTGAATACAATGACCAAAGATGCAGAA

AGAGGCAAGTTGAAGAGACGGGCAATTGCAACACCCGGGATGCAGATTAGAGGATTCGTG

TACTTCGTTGAAACACTAGCGAGGAGCATCTGTGAGAAACTAGAGCAATCTGGGCTCCCT

GTTGGAGGGAATGAGAAGAAGGCTAAATTGGCAAATGTTGTGAGAAAAATGATGACTAAC

TCACAAGATACAGAGCTCTCCTTTACAATTACTGGAGACAACACCAAATGGAATGAGAAT

CAAAACCCTCGGATGTTTTTGGCAATGATAACATATATCACAAGAAACCAACCTGAATGG

TTTAGAAATGTCTTAAGCATTGCCCCTATAATGTTCTCAAACAAAATGGCGAGATTAGGG

AAAGGATACATGTTTGAAAGTAAGAGCATGAAGCTAAGAACACAAATACCAGCAGAGATG

CTTACAAATATTGATCTGAAGTATTTCAACGAACCAACGAGAAAGAAAATCGAGAAAATA

AGACCTCTGCTGATTGATGGCACGGCCTCATTGAGTCCTGGGATGATGATGGGCATGTTC

AATATGCTGAGCACAGTATTGGGGGTCTCAATCCTGAATCTCGGGCAAAAGAGGTACACC

AAAACCACATACTGGTGGGATGGACTTCAATCCTCTGATGATTTCGCTCTCATAGTGAAT

GCACCGAATCATGAGGGGATACAAGCAGGAGTGGATAGATTCTATAGGACCTGCAAACTG

GTTGGGATCAACATGAGCAAAAAGAAGTCTTACATAAACCGAACAGGAACATTTGAGTTC

ACAAGTTTTTTCTACCGCTATGGATTTGTAGCTAACTTCAGTATGGAATTACCCAGCTTT

GGAGTGTCTGGAATCAATGAATCAGCTGACATGAGCATTGGAGTTACAGTGATAAAAAAC

AATATGATAAACAATGATCTTGGACCAGCGACAGCTCAAATGGCTCTTCAGTTATTCATC

AAAGACTATAGGTACACATACCGATGCCACAGGGGTGATACACAAATTCAAACGAGGAGA

TCATTCGAGCTGAAGAAGCTGTGGGATCAGACCCGTTCAAAGGCAGGGCTGTTGATATCA

GACGGGGGGCCAAACCTATACAACATTCGGAACCTCCACATCCCAGAGGTCTGCTTGAAG

TGGGAGCTGATGGATGAAGACTACCAAGGCAGGCTGTGCAATCCTCTGAATCCATTTGTC

AGTCATAAAGAGATTGAGTCCGTAAACAATGCTGTAGTAATGCCCGCCCATGGCCCGGCC

AAGAGCATGGAATATGATGCTGTTGCGACCACACATTCGTGGATTCCTAAGAGGAACCGG

TCCATTCTCAATACCAGCCAAAGGGGAATTCTTGAGGATGAGCAGATGTACCAAAAGTGC

TGTAGTCTATTCGAGAAATTCTTCCCCAGCAGTTCATACAGGAGGCCAGTTGGAATTTCC

AGCATGGTGGAGGCCATGGTGTCTAGGGCCCGAATTGATGCACGCATCGACTTCGAATCT

GGAAGGATTAAGAAAGAAGAGTTTGCTGAGATCATGAAGATCTGTTCCACCATTGAAGAG

CTCAGACGGCAAAAATAGTGAATTTAGCTTGTCCTTCATGAAA-----------------

-

>A_chicken_Korea_H008_2021_EPI1846536

------------------------ATGGATGTCAATCCGACTTTACTTTTCTTAAAAGTG

CCAGCGCAAAATGCTATAAGTACTACATTCCCTTACACTGGAGATCCTCCATACAGCCAT

GGAACAGGAACAGGGTATACCATGGACACAGTAAACAGAACACATCAATACTCAGAAAAG

GGAAAGTGGACAACAAACACAGAAACCGGAGCACCCCAACTCAACCCAATTGATGGACCA

TTACCAGAGGACAATGAGCCAAGCGGATATGCACAAACTGATTGCGTGTTGGAAGCAATG

GCTTTCCTTGAAGAATCCCACCCAGGGATATTTGAAAACTCTTGTCTTGAAGCGATGGAA

ATCGTTCAGCAAACAAGAGTGGACAAACTAACCCAAGGTCGCCAGACTTATGACTGGACA

CTGAACAGAAACCAACCAGCTGCAACCTCTTTGGCCAACACTATAGAGGTGTTCAGATCT

AATGGTCTGACAGCCAATGAATCAGGGAGACTGATAGATTTTCTCAGGGATGTGATGGAA

TCAATGAATAAAGAAGAGATGGAAGTAACAACACATTTCCAGAGAAAAAGAAGAGTGAGG

GACAACATGACTAAGAAGATGGTCACACAAAGAACAATAGGGAAGAAGAAGCAGAGGCTG

AACAAGAGGAGTTACTTGATAAGAGCACTGACATTGAATACAATGACCAAAGATGCAGAA

AGAGGCAAGTTGAAGAGACGGGCAATTGCAACACCCGGGATGCAGATTAGAGGATTCGTG

TACTTCGTTGAAACACTAGCGAGGAGCATCTGTGAGAAACTAGAGCAATCTGGGCTCCCT

GTTGGAGGGAATGAGAAGAAGGCTAAATTGGCAAATGTTGTGAGAAAAATGATGACTAAC

TCACAAGATACAGAGCTCTCCTTTACAATTACTGGAGACAACACCAAATGGAATGAGAAT

CAAAACCCTCGGATGTTTTTGGCAATGATAACATATATCACAAGGAACCAACCTGAATGG

TTTAGAAATGTCTTAAGCATTGCCCCTATAATGTTCTCAAACAAAATGGCGAGATTAGGG

AAAGGATACATGTTTGAAAGTAAGAGCATGAAGCTAAGAACACAAATACCAGCAGAGATG

CTTACAAATATTGATCTGAAGTATTTCAACGAATCAACGAGAAAGAAAATCGAGAAAATA

AGACCTCTGCTGATTGATGGCACGGCCTCATTGAGTCCTGGGATGATGATGGGCATGTTC

AATATGCTGAGCACAGTATTGGGGGTCTCAATCCTGAATCTCGGGCAAAAGAGGTACACC

AAAACCACATACTGGTGGGATGGACTTCAATCCTCTGATGATTTCGCTCTCATAGTGAAT

GCACCGAATCATGAGGGGATACAAGCAGGAGTGGATAGATTCTATAGGACCTGCAAACTG

GTTGGGATCAACATGAGCAAAAAGAAGTCTTACATAAACCGAACAGGAACATTTGAGTTC

ACAAGTTTTTTCTACCGCTATGGATTTGTAGCTAACTTCAGTATGGAATTACCCAGCTTT

GGAGTGTCTGGAATCAATGAATCAGCTGACATGAGCATTGGAGTTACAGTGATAAAAAAC

AATATGATAAACAATGATCTTGGACCAGCAACAGCTCAAATGGCTCTTCAATTATTCATC

AAAGACTATAGGTACACATACCGATGCCACAGGGGTGATACACAAATTCAAACGAGGAGA

TCATTCGAGCTGAAGAAGCTGTGGGAGCAGACCCGTTCAAAGGCAGGGCTGTTGATATCA

GACGGGGGGCCAAACCTATACAACATTCGGAACCTCCACATCCCAGAGGTCTGCTTGAAG

TGGGAGCTGATGGATGAAGACTACCAAGGCAGGCTGTGCAATCCTCTGAATCCATTTGTC

AGTCATAAAGAGATTGAGTCCGTAAACAATGCTGTAGTAATGCCCGCCCATGGCCCGGCC

AAGAGCATGGAATATGATGCTGTTGCGACCACACATTCGTGGATTCCTAAGAGGAACCGC

TCCATTCTCAATACCAGCCAAAGGGGAATTCTTGAGGATGAGCAGATGTACCAAAAGTGC

TGTAGTCTATTCGAGAAATTCTTCCCCAGCAGTTCATACAGGAGGCCAGTTGGAATTTCC

AGCATGGTGGAGGCCATGGTGTCTAGGGCCCGAATTGATGCACGCATCGACTTCGAATCT

GGAAGGATTAAGAAAGAAGAGTTTGCTGAGATCATGAAGATCTGTTCCACCATTGAAGAG

CTCAGACGGCAAAAATAG------------------------------------------

-

>A_mallard_Korea_WA820_2020_EPI1846600

------------------------ATGGATGTCAATCCGACTTTACTTTTCTTAAAAGTG

CCAGCGCAAAATGCTATAAGTACTACATTCCCTTACACTGGAGATCCTCCATACAGCCAT

GGAACAGGAACAGGGTATACCATGGACACAGTAAACAGAACACATCAATACTCAGAAAAG

GGAAAGTGGACAACAAACACAGAAACCGGAGCACCCCAACTCAACCCAATTGATGGACCA

TTACCAGAGGACAATGAGCCAAGCGGATATGCACAAACTGATTGCGTGTTGGAAGCAATG

GCTTTCCTTGAAGAATCCCACCCAGGGATATTTGAAAACTCTTGTCTTGAAGCGATGGAA

ATCGTTCAGCAAACAAGAGTGGACAAACTAACCCAAGGTCGCCAGACTTATGACTGGACA

CTGAACAGAAATCAACCAGCTGCAACCTCTTTGGCCAACACTATAGAGGTGTTCAGATCG

AATGGTCTGACAGCCAATGAATCAGGGAGACTGATAGATTTTCTCAGGGATGTGATGGAA

TCAATGGATAAAGAAGAGATGGAAGTAACAACACATTTCCAGAGAAAAAGAAGAGTGAGG

GACAACATGACTAAGAAGATGGTCACACAAAGAACAATAGGGAAGAAGAAGCAGAGGCTG

AACAAGAGGAGTTACTTGATAAGAGCACTGACATTGAATACAATGACCAAAGATGCAGAA

AGAGGCAAGTTGAAGAGACGGGCAATTGCAACACCCGGGATGCAGATTAGAGGATTCGTG

TACTTCGTTGAAACACTAGCGAGGAGCATCTGTGAGAAACTAGAGCAATCTGGGCTCCCT

GTTGGAGGGAATGAGAAGAAGGCTAAATTGGCAAATGTTGTGAGAAAAATGATGACTAAC

TCACAAGATACAGAGCTCTCCTTTACAATTACTGGAGACAACACCAAATGGAATGAGAAT

CAAAACCCTCGGATGTTTTTGGCAATGATAACATATATCACAAGAAACCAACCTGAATGG

TTTAGAAATGTCTTAAGCATTGCTCCTATAATGTTCTCAAACAAAATGGCGAGATTAGGG

AAAGGATACATGTTTGAAAGTAAGAGCATGAAGCTAAGAACACAAATACCAGCAGAGATG

CTTACAAATATTGATCTGAAGTATTTCAACGAACCAACGAGAAAGAAAATCGAGAAAATA

AGACCTCTGCTGATTGATGGCACGGCCTCATTGAGTCCTGGGATGATGATGGGCATGTTC

AATATGCTGAGCACAGTATTGGGGGTCTCAATCCTGAATCTCGGGCAAAAGAGGTACACC

AAAACCACATACTGGTGGGATGGACTTCAATCCTCTGATGATTTCGCTCTCATAGTGAAT

GCACCGAATCATGAGGGGATACAAGCAGGAGTGGATAGATTCTATAGGACCTGCAAACTA

GTTGGGATCAACATGAGCAAAAAGAAGTCTTACATAAACCGAACAGGAACATTTGAGTTC

ACAAGTTTTTTCTACCGCTATGGATTTGTAGCTAACTTCAGTATGGAATTACCCAGCTTT

GGAGTGTCTGGAATCAATGAATCAGCTGACATGAGCATTGGAGTTACAGTGATAAAAAAC

AATATGATAAACAATGATCTTGGACCAGCAACAGCTCAAATGGCTCTTCAGTTATTCATC

AAAGACTATAGGTACACATACCGATGCCACAGGGGTGATACACAAATTCAAACGAGGAGA

TCATTCGAGCTGAAGAAGCTGTGGGAGCAGACCCGTTCAAAGGCAGGGCTGTTGATATCA

GACGGGGGGCCAAACCTATACAACATTCGGAACCTCCACATCCCAGAGGTCTGCTTGAAG

TGGGAGCTGATGGATGAAGACTACCAAGGCAGGCTGTGCAATCCTCTGAATCCATTTGTC

AGTCATAAAGAGATTGAGTCCGTAAACAATGCTGTAGTAATGCCCGCCCATGGCCCGGCC

AAGAGCATGGAATATGATGCTGTTGCGACCACACATTCGTGGATTCCTAAGAGGAACCGC

TCCATTCTCAATACCAGCCAAAGGGGAATTCTTGAGGATGAGCAGATGTACCAAAAGTGC

TGTAGTCTATTCGAGAAATTCTTCCCCAGCAGTTCATACAGGAGGCCAGTTGGAATTTCC

AGCATGGTGGAGGCCATGGTGTCTAGGGCCCGAATTGATGCACGCATCGACTTCGAATCT

GGCAGGATTAAGAAAGAAGAGTTTGCTGAGATCATGAAGATCTGTTCCACCATTGAAGAG

CTCAGACGGCAAAAATAG------------------------------------------

-

>A_duck_Korea_H016_2021_EPI1846704

------------------------ATGGATGTCAATCCGACTTTACTTTTCTTAAAAGTG

CCAGCGCAAAATGCTATAAGTACTACATTCCCTTACACTGGAGATCCTCCATACAGCCAT

GGAACAGGAACAGGGTATACCATGGACACAGTAAACAGAACACATCAATACTCAGAAAAG

GGAAAGTGGACAACAAACACAGAAACCGGAGCACCCCAACTCAACCCAATTGATGGACCA

TTACCAGAGGACAATGAGCCAAGCGGATATGCACAAACTGATTGCGTGTTGGAAGCAATG

GCTTTCCTTGAAGAATCCCACCCAGGGATATTTGAAAACTCTTGTCTTGAAGCGATGGAA

ATCGTTCAGCAAACAAGAGTGGACAAACTAACCCAAGGTCGCCAGACTTATGACTGGACA

CTGAACAGAAACCAACCAGCTGCAACCTCTTTGGCCAACACTATAGAGGTGTTCAGATCG

AATGGTCTGACAGCCAATGAATCAGGGAGACTGATAGATTTTCTCAGGGATGTGATGGAA

TCAATGGATAAAGAAGAGATGGAAGTAACAACACATTTCCAGAGAAAAAGAAGAGTGAGG

GACAACATGACTAAGAAGATGGTCACACAAAGAACAATAGGGAAGAAGAAGCAGAGGCTG

AACAAGAGGAGTTACTTGATAAGAGCACTGACATTGAATACAATGACCAAAGATGCAGAA

AGAGGCAAGTTGAAGAGACGGGCAATTGCAACACCCGGGATGCAGATTAGAGGATTCGTG

TACTTCGTTGAAACACTAGCGAGGAGCATCTGTGAGAAACTAGAGCAATCTGGGCTCCCT

GTTGGAGGGAATGAGAAGAAGGCTAAATTGGCAAATGTTGTGAGAAAAATGATGACTAAC

TCACAAGATACAGAGCTCTCCTTTACAATTACTGGAGACAACACCAAATGGAATGAGAAT

CAAAACCCTCGGATGTTTTTGGCAATGATAACATATATCACAAGAAACCAACCTGAATGG

TTTAGAAATGTCTTAAGCATTGCTCCTATAATGTTCTCAAACAAAATGGCGAGATTAGGG

AAAGGATACATGTTTGAAAGTAAGAGCATGAAGCTAAGAACACAAATACCAGCAGAGATG

CTTACAAATATTGATCTGAAGTATTTCAACGAACCAACGAGAAAGAAAATCGAGAAAATA

AGACCTCTGCTGATTGATGGCACGGCCTCATTGAGTCCTGGGATGATGATGGGCATGTTC

AATATGCTGAGCACAGTATTGGGGGTCTCAATCCTGAATCTCGGGCAAAAGAGGTACACC

AAAACCACATACTGGTGGGATGGACTTCAATCCTCTGATGATTTCGCTCTCATAGTGAAT

GCACCGAATCATGAGGGGATACAAGCAGGAGTGGATAGATTCTATAGGACCTGCAAACTA

GTTGGGATCAACATGAGCAAAAAGAAGTCTTACATAAACCGAACAGGAACATTTGAGTTC

ACAAGTTTTTTCTACCGCTATGGATTTGTAGCTAACTTCAGTATGGAATTACCCAGCTTT

GGAGTGTCTGGAATCAATGAATCAGCTGACATGAGCATTGGAGTTACAGTGATAAAAAAC

AATATGATAAACAATGATCTTGGACCAGCAACAGCTCAAATGGCTCTTCAGTTATTCATC

AAAGACTATAGGTACACATACCGATGCCACAGGGGTGATACACAAATTCAAACGAGGAGA

TCATTCGAGCTGAAGAAGCTGTGGGAGCAGACCCGTTCAAAGGCAGGGCTGTTGATATCA

GACGGGGGGCCAAACCTATACAACATTCGGAACCTCCACATCCCAGAGGTCTGCTTGAAG

TGGGAGCTGATGGATGAAGACTACCAAGGCAGGCTGTGCAATCCTCTGAATCCATTTGTC

AGTCATAAAGAGATTGAGTCCGTAAACAATGCTGTAGTAATGCCCGCCCATGGCCCGGCC

AAGAGCATGGAATATGATGCTGTTGCGACCACACATTCGTGGATTCCTAAGAGGAACCGC

TCCATTCTCAATACCAGCCAAAGGGGAATTCTTGAGGATGAGCAGATGTACCAAAAGTGC

TGTAGTCTATTCGAGAAATTCTTCCCCAGCAGTTCGTACAGGAGGCCAGTTGGAATTTCC

AGCATGGTGGAGGCCATGGTGTCTAGGGCCCGAATTGATGCACGCATCGACTTCGAATCT

GGCAGGATTAAGAAAGAAGAGTTTGCTGAGATCATGAAGATCTGTTCCACCATTGAAGAG

CTCAGACGGCAAAAATAG------------------------------------------

-

>A_chicken_Astrakhan_321-01_2020_EPI1846967

AGCAAAAGCAGGCAAACTATTTGAATGGATGTCAATCCGACTTTACTTTTCTTAAAAGTG

CCAGCGCAAAATGCTATAAGTACTACATTCCCTTACACTGGAGATCCTCCATACAGCCAT

GGAACAGGAACAGGGTATACCATGGACACAGTAAACAGAACACATCAATACTCAGAAAAG

GGAAAGTGGACAACAAACACAGAAACCGGAGCACCCCAACTCAACCCAATTGATGGACCA

CTACCAGAGGACAATGAGCCAAGCGGATATGCACAAACTGATTGCGTGTTGGAAGCAATG

GCTTTCCTTGAAGAATCCCACCCAGGGATATTTGAAAACTCTTGTCTTGAAGCGATGGAA

ATCGTTCAGCAAACAAGAGTGGACAAACTAACCCAAGGTCGCCAGACTTATGACTGGACA

CTGAACAGAAACCAACCAGCTGCAACCTCTTTGGCCAACACTATAGAGGTGTTCAGATCG

AATGGTCTGACAGCCAATGAATCAGGGAGACTGATAGATTTTCTCAGGGATGTGATGGAA

TCAATGGATAAAGAAGAGATGGAAGTAACAACACATTTCCAGAGAAAAAGAAGAGTGAGG

GACAACATGACTAAGAAGATGGTCACACAAAGAACAATAGGGAAGAAGAAGCAGAGGCTG

AACAAGAGGAATTACTTAATAAGAGCACTGACATTGAATACAATGACCAAAGATGCAGAA

AGAGGCAAGTTGAAGAGACGGGCAATTGCAACACCCGGGATGCAGATTAGAGGATTCGTG

TACTTCGTTGAAACACTAGCGAGGAGCATCTGTGAGAAGCTAGAGCAATCTGGGCTCCCT

GTTGGAGGGAATGAGAAGAAGGCTAAATTGGCAAATGTTGTGAGAAAAATGATGACTAAC

TCACAAGATACAGAGCTCTCCTTTACAATTACTGGAGACAACACCAAATGGAATGAGAAT

CAAAACCCTCGGATGTTTTTGGCAATGATAACATATATCACAAGAAACCAACCTGAATGG

TTTAGAAATGTCTTAAGCATTGCCCCTATAATGTTCTCAAACAAAATGGCGAGATTAGGG

AAAGGATACATGTTTGAAAGTAAGAGCATGAAGCTAAGAACACAAATACCAGCAGAGATG

CTTACAAATATTGATCTGAAGTATTTCAACGAACCAACGAGAAAGAAAATCGAGAAAATA

AGACCTCTGCTGATTGATGGCACGGCCTCATTGAGTCCTGGGATGATGATGGGCATGTTC

AATATGCTGAGCACAGTATTGGGGGTCTCAATCCTGAATCTCGGGCAAAAGAGGTACACC

AAAACCACATACTGGTGGGATGGACTTCAATCCTCTGATGATTTCGCTCTCATAGTGAAT

GCACCGAATCATGAGGGGATACAAGCAGGAGTGGATAGATTCTATAGGACCTGCAAACTG

GTTGGGATCAACATGAGCAAAAAGAAGTCTTACATAAACCGAACAGGAACATTTGAGTTC

ACAAGTTTTTTCTACCGCTATGGATTTGTAGCTAACTTCAGTATGGAATTACCCAGCTTT

GGAGTGTCTGGAATCAATGAATCAGCTGACATGAGCATTGGAGTTACAGTGATAAAAAAC

AATATGATAAACAATGATCTTGGACCAGCGACAGCTCAAATGGCTCTTCAGTTATTCATC

AAAGACTATAGGTACACATACCGATGCCACAGGGGTGATACACAAATTCAAACGAGGAGA

TCATTCGAGCTGAAGAAGCTGTGGGAGCAGACCCGTTCAAAGGCAGGGCTGTTGATATCA

GACGGGGGGCCAAACCTATACAACATTCGGAACCTCCACATCCCAGAGGTCTGCTTGAAG

TGGGATCTGATGGATGAAGACTACCAAGGCAGGCTGTGCAATCCTCTGAATCCATTTGTC

AGTCATAAAGAGATTGAGTCCGTAAACAATGCTGTAGTAATGCCCGCCCATGGCCCGGCC

AAGAGCATGGAATATGATGCTGTTGCGACCACACATTCGTGGATTCCTAAGAGGAACCGT

TCCATTCTCAATACCAGCCAAAGGGGAATTCTTGAGGATGAGCAGATGTACCAAAAGTGC

TGTAGTCTATTCGAGAAATTCTTCCCCAGCAGTTCATACAGGAGGCCAGTTGGAATTTCC

AGCATGGTGGAGGCCATGGTGTCTAGGGCCCGAATTGATGCACGCATCGACTTCGAATCT

GGAAGGATCAAGAAAGAAGAGTTTGCTGAGATCATGAAGATCTGTTCCACCATTGAAGAG

CTCAGACGGCAAAAATAGTGAATTTAGCTTGTCCTTCATGAAAAAATGCCTTGTTTCTAC

T

>A_crane_Kagoshima_KU-93_2021_EPI1848525

------------------------ATGGATGTCAATCCGACTTTACTTTTCTTAAAAGTG

CCAGCGCAAAATGCTATAAGTACTACATTCCCTTACACTGGAGATCCTCCATACAGCCAT

GGAACAGGAACAGGGTATACCATGGACACAGTAAACAGAACACATCAATACTCAGAAAAG

GGAAAGTGGACAACAAACACAGAAACCGGAGCACCCCAACTCAACCCAATTGATGGACCA

TTACCAGAGGACAATGAGCCAAGCGGATATGCACAAACTGATTGCGTGTTGGAAGCAATG

GCTTTCCTTGAAGAATCCCACCCAGGGATATTTGAAAACTCTTGTCTTGAAGCGATGGAA

ATCGTTCAGCAAACAAGAGTGGACAAACTAACCCAAGGTCGCCAGACTTATGACTGGACA

CTGAACAGAAACCAACCAGCTGCAACCTCTTTGGCCAACACTATAGAGGTGTTCAGATCG

AATGGTCTGACAGCCAATGAATCNRGGAGACTGATAGATTTTCTCAGGGATGTGATGGAA

TCAATGAATAAAGAAGAGATGGAAGTAACAACACATTTCCAGAGAAAAAGAAGAGTGAGG

GACAACATGACTAAGAAGATGGTCACACAAAGAACAATAGGGAAGAAGAAGCAGAGGCTG

AACAAGAGGAGTTACTTGATAAGAGCACTGACATTGAATACAATGACCAAAGATGCAGAA

AGAGGCAAGTTGAAAAGACGGGCAATTGCAACACCCGGGATGCAGATTAGAGGATTCGTG

TACTTCGTTGAAACACTAGCGAGGAGCATCTGTGAGAAACTAGAGCAATCTGGGCTCCCT

GTTGGAGGGAATGAGAAGAAGGCTAAATTGGCAAATGTTGTGAGAAAAATGATGACTAAC

TCACAAGATACAGAGCTCTCCTTTACAATTACTGGAGACAACACCAAATGGAATGAGAAT

CAAAACCCTCGGATGTTTTTGGCAATGATAACATATATCACAAGGAACCAACCTGAATGG

TTTAGAAATGTCTTAAGCATTGCCCCTATAATGTTCTCAAACAAAATGGCGAGATTAGGG

AAAGGATACATGTTTGAAAGTAAGAGCATGAAGCTAAGAACACAAATACCAGCAGAGATG

CTTACAAATATTGATCTGAAGTATTTCAACGAATCAACGAGAAAGAAAATCGAGAAAATA

AGACCTCTGCTGATTGATGGCACGGCCTCATTGAGTCCTGGGATGATGATGGGCATGTTC

AATATGCTGAGCACAGTATTGGGGGTCTCAATCCTGAATCTCGGGCAAAAGAGGTACACC

AAAACCACATACTGGTGGGATGGACTTCAATCCTCTGATGATTTCGCTCTCATAGTGAAT

GCACCGAATCATGAGGGGATACAAGCAGGAGTGGATAGATTCTATAGGACCTGCAAACTG

GTTGGGATCAACATGAGCAAAAAGAAGTCTTACATAAACCGAACAGGAACATTTGAGTTC

ACAAGTTTTTTCTACCGCTATGGATTTGTAGCTAACTTCAGTATGGAATTACCCAGCTTT

GGAGTGTCTGGAATCAATGAATCAGCTGACATGAGCATTGGAGTTACAGTGATAAAAAAC

AATATGATAAACAATGATCTTGGACCAGCAACAGCTCAAATGGCTCTTCAATTATTCATC

AAAGACTATAGGTACACATACCGATGCCACAGGGGTGATACACAAATTCAAACGAGGAGA

TCATTCGAGCTGAAGAAGCTGTGGGAGCAGACCCGTTCAAAGGCAGGGCTGTTGATATCA

GACGGNGGGCCAAACCTATACAACATTCGGAACCTCCACATCCCAGAGGTCTGCTTGAAG

TGGGAGCTGATGGATGAAGACTACCAAGGCAGGCTGTGCAATCCTCTGAATCCATTTGTC

AGTCATAAAGAGATTGAGTCCGTAAACAATGCTGTAGTAATGCCCGCCCATGGCCCGGCC

AAGAGCATGGAATATGATGCTGTTGCGACCACACATTCGTGGATTCCTAAGAGGAACCGC

TCCATTCTCAATACCAGCCAAAGGGGAATTCTTGAGGATGAGCAGATGTACCAAAAGTGC

TGTAGTCTATTCGAGAAATTCTTCCCCAGCAGTTCATACAGGAGGCCAGTTGGAATTTCC

AGCATGGTGGAGGCCATGGTGTCTAGGGCCCGAATTGATGCACGCATCGACTTCGAATCT

GGAAGGATTAAGAAAGAAGAGTTTGCTGAGATCATGAAGATCTGTTCCACCATTGAAGAG

CTCAGACGGCAAAAATAG------------------------------------------

-

>A_mallard_Kagoshima_KU-d89_2021_EPI1848536

------------------------ATGGATGTCAATCCGACTTTACTTTTCTTAAAAGTG

CCAGCGCAAAATGCTATAAGTACTACATTCCCTTACACTGGAGATCCTCCATACAGCCAT

GGAACAGGAACAGGGTATACCATGGACACAGTAAACAGAACACATCAATACTCAGAAAAG

GGAAAGTGGACAACAAACACAGAAACCGGAGCACCCCAACTCAACCCAATTGATGGACCA

TTACCAGAGGACAATGAGCCAAGCGGATATGCACAAACTGATTGCGTGTTGGAAGCAATG

GCTTTCCTTGAAGAATCCCACCCAGGGATATTTGAAAACTCTTGTCTTGAAGCGATGGAA

ATCGTTCAGCAAACAAGAGTGGACAAACTAACCCAAGGTCGCCAGACTTATGACTGGACA

CTGAACAGAAACCAACCAGCTGCAACCTCTTTGGCCAACACTATAGAGGTGTTCAGATCG

AATGGTCTGACAGCCAATGAATCAGGGAGACTGATAGATTTTCTCAGGGATGTGATGGAA

TCAATGAATAAAGAAGAGATGGAAGTAACAACACATTTCCAGAGAAAAAGAAGAGTGAGG

GACAACATGACTAAGAAGATGGTCACACAAAGAACAATAGGGAAGAAGAAGCAGAGGCTG

AACAAGAGGTGTTACTTGATAAGAGCACTGACATTGAATACAATGACCAAAGATGCAGAA

AGAGGCAAGTTGAAAAGACGGGCAATTGCAACACCCGGGATGCAGATTAGGGGATTCGTG

TACTTCGTTGAAACACTAGCGAGGAGCATCTGTGAGAAACTAGAGCAATCTGGGCTCCCT

GTTGGAGGGAATGAGAAGAAGGCTAAATTGGCAAATGTTGTGAGAAAAATGATGACTAAC

TCACAAGATACAGAGCTCTCCTTTACAATTACTGGAGACAACACCAAATGGAATGAGAAT

CAAAACCCTCGGATGTTTTTGGCAATGATAACATATATCACAAGGAACCAACCTGAATGG

TTTAGAAATGTCTTAAGCATTGCCCCTATAATGTTCTCAAACAAAATGGCGAGATTAGGG

AAAGGATACATGTTTGAAAGTAAGAGCATGAAGCTAAGAACACAAATACCAGCAGAGATG

CTTACAAATATTGATCTGAAGTATTTCAACGAATCAACGAGAAAGAAAATCGAGAAAATA

AGACCTCTGCTGATTGATGGCACGGCCTCATTGAGTCCTGGGATGATGATGGGCATGTTC

AATATGCTGAGCACAGTATTGGGGGTCTCAATCCTGAATCTCGGGCAAAAGAGGTACACC

AAAACCACATACTGGTGGGATGGACTTCAATCCTCTGATGATTTCGCTCTCATAGTAAAT

GCACCGAATCATGAGGGGATACAAGCAGGAGTGGATAGATTCTATAGGACCTGCAAACTG

GTTGGGATCAACATGAGCAAAAAGAAGTCTTACATAAACCGAACAGGAACATTTGAGTTC

ACAAGTTTTTTCTACCGCTATGGATTTGTAGCTAACTTCAGTATGGAATTACCCAGCTTT

GGAGTGTCTGGAATCAATGAATCAGCTGACATGAGCATTGGAGTTACAGTGATAAAAAAC

AATATGATAAACAATGATCTTGGACCAGCAACAGCTCAAATGGCTCTTCAATTATTCATC

AAAGACTATAGGTACACATACCGATGCCACAGGGGTGATACACAAATTCAAACGAGGAGA

TCATTCGAGCTGAAGAAGCTGTGGGAGCAGACCCGTTCAAAGGCAGGGCTGTTGATATCA

GACGGGGGGCCAAACCTATACAACATTCGGAACCTCCACATCCCAGAGGTCTGCTTGAAG

TGGGAGCTGATGGATGAAGACTACCAAGGCAGGCTGTGCAATCCTCTGAATCCATTTGTC

AGTCATAAAGAGATTGAGTCCGTAAACAATGCTGTAGTAATGCCCGCCCATGGCCCGGCC

AAGAGCATGGAATATGATGCTGTTGCGACCACACATTCGTGGATTCCTAAGAGGAACCGC

TCCATTCTCAATACCAGCCAAAGGGGAATTCTTGAGGATGAGCAGATGTACCAAAAGTGC

TGTAGTCTATTCGAGAAATTCTTCCCCAGCAGTTCATACAGGAGGCCAGTTGGAATTTCC

AGCATGGTGGAGGCCATGGTGTCTAGGGCCCGAATTGATGCACGCATCGACTTCGAATCT

GGAAGGATTAAGAAAGAAGAGTTTGCTGAGATCATGAAGATCTGTTCCACCATTGAAGAG

CTCAGACGGCAAAAATAG------------------------------------------

-

>A_chicken_Kostroma_304-06_2020_EPI1848644

AGCAAAAGCAGGCAAACTATTTGAATGGATGTCAATCCGACTTTACTTTTCTTAAAAGTG

CCAGCGCAAAATGCTATAAGTACTACATTCCCTTACACTGGAGATCCTCCATACAGCCAT

GGAACAGGAACAGGGTATACCATGGACACAGTAAACAGAACACATCAATACTCAGAAAAG

GGAAAGTGGACAACAAACACAGAAACCGGAGCACCCCAACTCAACCCAATTGATGGACCA

TTACCAGAGGACAATGAGCCAAGCGGATATGCACAAACTGATTGCGTGTTGGAAGCAATG

GCTTTCCTTGAAGAATCCCACCCAGGGATATTTGAAAACTCTTGTCTTGAAGCGATGGAA

ATCGTTCAGCAAACAAGAGTGGACAAACTAACCCAAGGTCGCCAGACTTATGACTGGACA

CTGAACAGAAACCAACCAGCTGCAACCTCTTTGGCCAACACTATAGAGGTGTTCAGATCG

AATGGTCTGACAGCCAATGAATCAGGAAGACTGATAGATTTTCTCAGGGATGTGATGGAA

TCAATGGATAAAGAAGAGATGGAAGTAACAACACATTTCCAGAGAAAAAGAAGAGTGAGG

GACAACATGACTAAGAAGATGGTCACACAAAGAACAATAGGGAAGAAGAAGCAGAGGCTG

AACAAGAGGAGTTACTTAATAAGAGCACTGACATTGAATACAATGACCAAAGATGCAGAA

AGAGGCAAGTTGAAGAGACGGGCAATTGCAACACCCGGGATGCAGATTAGAGGATTCGTG

TACTTCGTTGAAACACTAGCAAGGAGCATCTGTGAGAAACTAGAGCAATCTGGGCTCCCT

GTTGGAGGGAATGAGAAGAAGGCTAAATTGGCAAATGTTGTGAGAAAAATGATGACTAAC

TCACAAGATACAGAGCTCTCCTTTACAATTACTGGAGACAACACCAAATGGAATGAGAAT

CAAAACCCTCGGATGTTTTTGGCAATGGTAACATATATCACAAGAAACCAACCTGAATGG

TTTAGAAATGTCTTAAGCATTGCCCCTATAATGTTCTCAAACAAAATGGCGAGATTAGGG

AAAGGATACATGTTTGAAAGTAAGAGCATGAAGCTAAGAACACAAATACCAGCAGAGATG

CTCACAAATATTGATCTGAAGTATTTCAACGAACCAACGAGAAAGAAAATCGAGAAAATA

AGACCCCTGCTGATTGATGGCACGGCCTCATTGAGTCCTGGGATGATGATGGGCATGTTC

AATATGCTGAGCACAGTATTGGGGGTCTCAATCCTGAATCTCGGGCAAAAGAGGTACACC

AAAACCACATACTGGTGGGATGGACTTCAATCCTCTGATGATTTCGCTCTCATAGTGAAT

GCACCGAATCATGAGGGGATACAAGCAGGAGTGGATAGATTCTATAGGACCTGCAAACTG

GTTGGGATCAACATGAGCAAAAAGAAGTCTTACATAAACCGAACAGGAACATTTGAGTTC

ACAAGTTTTTTCTACCGCTATGGATTTGTAGCTAACTTCAGTATGGAATTACCCAGCTTT

GGAGTGTCTGGAATCAATGAATCAGCTGACATGAGCATTGGAGTTACAGTGATAAAAAAC

AATATGATAAACAATGATCTTGGACCAGCAACAGCTCAAATGGCTATTCAGTTATTCATC

AAAGACTATAGGTACACATACCGATGCCACAGGGGTGATACACAAATTCAAACGAGGAGA

TCATTCGAGCTGAAGAAGCTGTGGGAGCAGACCCGTTCAAAGGCAGGGCTGTTGATATCA

GACGGGGGGCCAAACCTATACAACATTCGGAACCTCCACATCCCAGAGGTCTGCTTGAAG

TGGGAGCTGATGGATGAAGACTACCAAGGCAGGCTGTGCAATCCTCTGAATCCATTTGTC

AGTCATAAAGAGATTGAGTCCGTAAACAATGCTGTAGTAATGCCCGCCCATGGCCCGGCC

AAGAGCATGGAATATGATGCTGTTGCGACCACACATTCGTGGATTCCAAAGAGGAACCGC

TCCATTCTCAATACCAGCCAAAGGGGAATTCTTGAGGATGAGCAGATGTACCAAAAGTGC

TGTAGTCTATTCGAGAAATTCTTCCCCAGCAGTTCATACAGGAGGCCAGTTGGAATTTCC

AGCATGGTGGAGGCCATGGTGTCTAGGGCCCGAATTGATGCACGCATCGACTTCGAATCT

GGAAGGATTAAGAAAGAAGAGTTTGCTGAGATCATGAAGATCTGTTCCACCATTGAAGAG

CTCAGACGGCAAAAATAGTGAATTTAGCTTGTCCTTCATGAAAAAATGCCTTGTTTCTAC

T

>A_chicken_Rostov-on-Don_308-02_2020_EPI1848668

AGCAAAAGCAGGCAAACTATTTGAATGGATGTCAATCCGACTTTACTTTTCTTAAAAGTG

CCAGCGCAAAATGCTATAAGTACTACATTCCCTTACACTGGAGATCCTCCATACAGCCAT

GGAACAGGAACAGGGTATACCATGGACACAGTAAACAGAACACATCAATACTCAGAAAAG

GGAAAGTGGACAACAAACACAGAAACCGGAGCACCCCAACTCAACCCAATTGATGGACCA

TTACCAGAGGACAATGAGCCAAGCGGATATGCACAAACTGATTGCGTGTTGGAAGCAATG

GCTTTCCTTGAAGAATCCCACCCAGGGATATTTGAAAACTCTTGTCTTGAAGCGATGGAA

ATCGTTCAGCAAACAAGAGTGGACAAACTAACCCAAGGTCGCCAGACTTATGACTGGACA

CTGAACAGAAACCAACCAGCTGCAACCTCTTTGGCCAACACTATAGAGGTGTTCAGATCG

AATGGTCTGACAGCCAATGAATCAGGGAGACTGATAGATTTTCTCAGGGATGTGATGGAA

TCAATGGATAAAGAAGAGATGGAAGTAACAACACATTTCCAGAGAAAAAGAAGAGTGAGG

GACAACATGACTAAGAAGATGGTCACACAAAGAACAATAGGGAAGAAGAAGCAGAGGCTG

AATAAGAGGAGTTACTTAATAAGAGCACTGACATTGAATACAATGACCAAAGATGCAGAA

AGAGGCAAGTTGAAGAGACGGGCAATTGCAACACCCGGGATGCAGATTAGAGGATTCGTG

TACTTCGTTGAAACACTAGCGAGGAGCATCTGTGAGAAACTAGAGCAATCTGGGCTCCCT

GTTGGAGGGAATGAGAAGAAGGCTAAATTGGCAAATGTTGTGAGAAAAATGATGACTAAC

TCACAAGATACAGAGCTCTCCTTCACAGTTACTGGAGACAACACCAAATGGAATGAGAAT

CAAAACCCTCGAATGTTTTTGGCAATGATAACATATATCACAAGAAACCAACCTGAATGG

TTTAGAAATGTCTTAAGCATTGCCCCTATAATGTTCTCAAACAAAATGGCGAGATTAGGG

AAAGGATACATGTTTGAAAGTAAGAGCATGAAGCTAAGAACACAAATACCAGCAGAGATG

CTTACAAATATCGATCTGAAGTATTTCAACGAACCAACGAGAAAGAAAATCGAGAAAATA

AGACCTCTGCTGATTGATGGCACGGCCTCATTGAGTCCTGGGATGATGATGGGCATGTTC

AATATGCTGAGCACAGTATTAGGGGTCTCAATCCTGAATCTCGGGCAAAAAAGGTACACC

AAAACCACATACTGGTGGGATGGACTTCAATCCTCTGATGATTTCGCTCTCATAGTGAAT

GCACCGAATCATGAGGGGATACAAGCAGGAGTGGATAGATTCTATAGGACCTGCAAACTG

GTTGGGATCAACATGAGCAAAAAGAAGTCTTACATAAACCGAACAGGAACATTTGAGTTC

ACAAGTTTTTTCTACCGCTATGGATTTGTAGCTAACTTCAGTATGGAATTACCCAGCTTT

GGAGTGTCTGGAATCAATGAATCAGCTGACATGAGCATTGGAGTTACAGTGATAAAAAAC

AATATGATAAACAATGATCTTGGACCAGCAACAGCTCAAATGGCTCTTCAGTTATTCATC

AAAGACTATAGGTACACATACCGATGCCACAGGGGTGATACACAAATTCAAACGAGGAGA

TCATTCGAGCTGAAGAAGCTGTGGGAGCAGACCCGTTCAAAGGCAGGGCTGTTGATATCA

GACGGGGGGCCAAACCTATACAACATTCGGAATCTCCACATCCCAGAGGTCTGCTTGAAA

TGGGAGCTGATGGATGAAGACTACCAAGGCAGGCTGTGCAATCCTCTGAATCCATTTGTC

AGTCATAAAGAGATTGAGTCCGTAAACAATGCTGTAGTAATGCCCGCCCATGGCCCGGCC

AAGAGCATGGAATATGATGCTGTTGCGACCACACACTCGTGGATTCCTAAGAGGAACCGT

TCCATTCTCAATACCAGCCAAAGGGGAATTCTTGAGGATGAGCAGATGTACCAAAAGTGC

TGTAGTCTATTCGAGAAATTCTTCCCCAGCAGTTCATACAGGAGGCCAGTTGGAATTTCC

AGCATGGTGGAGGCCATGGTGTCTAGGGCCCGAATTGATGCACGCATCGACTTCGAATCT

GGAAGGATTAAGAAAGAAGAGTTTGCTGAGATCATGAAGATCTGTTCCACCATTGAAGAG

CTCAGACGGCAAAAATAGTGAATTTAGCTTGTCCTTCATGAAAAAATGCCTTGTTTCTAC

T

>A_turkey_Stavropol_320-02_2020_EPI1848700

AGCAAAAGCAGGCAAACTATTTGAATGGATGTCAATCCGACTTTACTTTTCTTAAAAGTG

CCAGCGCAAAATGCTATAAGTACTACATTCCCTTACACTGGAGATCCTCCATACAGCCAT

GGAACAGGAACAGGGTATACCATGGACACAGTAAACAGAACACATCAATACTCAGAAAAG

GGAAAGTGGACAACAAACACAGAAACCGGAGCACCCCAACTCAACCCAATTGATGGACCA

TTACCAGAGGACAATGAGCCAAGCGGATATGCACAAACTGATTGCGTGTTGGAAGCAATG

GCTTTCCTTGAAGAATCCCACCCAGGGATATTTGAAAACTCTTGTCTTGAAGCGATGGAA

ATCGTTCAGCAAACAAGAGTGGACAAACTAACCCAGGGTCGCCAGACTTATGACTGGACA

CTGAACAGAAACCAACCAGCTGCAACCTCTTTGGCCAACACTATAGAGGTGTTCAGATCG

AATGGTCTGACAGCCAATGAATCAGGGAGACTGATAGATTTTCTCAGGGATGTGATGGAG

TCAATGGATAAAGAAGAGATGGAAGTAACAACACATTTCCAGAGAAAAAGAAGAGTGAGG

GACAACATGACTAGGAAGATGGTCACACAAAGAACAATAGGGAAGAAGAAGCAGAGGCTG

AACAAGAGGAGTTACTTAATAAGAGCACTGACATTGAATACAATGACCAAAGATGCAGAA

AGAGGCAAGTTGAAGAGACGGGCAATTGCAACACCCGGGATGCAGATTAGAGGATTCGTG

TACTTCGTTGAAACACTAGCGAGGAGCATCTGTGAGAAACTAGAGCAATCTGGGCTCCCT

GTTGGAGGGAATGAGAAGAAGGCTAAATTGGCAAATGTTGTGAGAAAAATGATGACTAAC

TCACAAGATACAGAGCTCTCCTTTACAATTACTGGAGACAACACCAAATGGAATGAGAAT

CAAAACCCTCGGATGTTTTTGGCAATGATAACATATATCACAAGAAACCAACCTGAATGG

TTTAGAAATGTCTTAAGCATTGCCCCTATAATGTTCTCAAACAAAATGGCGAGATTAGGG

AAAGGATACATGTTTGAAAGTAAGAGCATGAAGCTAAGAACACAAATACCGGCAGAGCTG

CTTACAAATATCGATCTGAAGTATTTCAACGAACCAACGAGAAAGAAAATCGAGAAAATA

AGACCTCTGCTGATTGATGGCACGGCCTCATTGAGTCCTGGGATGATGATGGGCATGTTC

AATATGCTGAGCACAGTATTAGGGGTCTCAATCCTGAATCTCGGGCAAAAAAGGTACACC

AAAACCACATACTGGTGGGATGGACTTCAATCCTCTGATGATTTCGCTCTCATAGTGAAT

GCACCGAATCATGAGGGGATACAAGCAGGAGTGGATAGATTCTATAGGACCTGCAAACTG

GTTGGGATCAACATGAGCAAAAAGAAGTCTTACATAAACCGAACAGGAACATTTGAGTTC

ACAAGTTTTTTCTACCGCTATGGATTTGTAGCTAACTTCAGTATGGAATTACCCAGCTTT

GGAGTGTCTGGAATCAATGAATCAGCTGACATGAGCATTGGAGTTACAGTGATAAAAAAC

AATATGATAAACAATGATCTTGGACCAGCAACAGCTCAAATGGCTCTTCAGTTATTCATC

AAAGACTATAGGTACACATACCGATGCCACAGGGGTGATACACAAATTCAAACGAGGAGA

TCATTCGAGCTGAAGAAGCTGTGGGAGCAGACCCGTTCAAAGGCAGGGCTGTTGATATCA

GACGGGGGGCCAAACCTATACAACATTCGGAATCTCCACATCCCAGAGGTCTGCTTGAAG

TGGGAGCTGATGGATGAAGACTACCAAGGCAGGCTGTGCAATCCTCTGAATCCATTTGTC

AGTCATAAAGAGATTGAGTCCGTAAACAATGCTGTAGTAATGCCCGCCCATGGCCCGGCC

AAGAGCATGGAATATGATGCTGTTGCGACCACACACTCGTGGATTCCTAAGAGGAACCGT

TCCATTCTCAATACCAGCCAAAGGGGAATTCTTGAGGATGAGCAGATGTACCAAAAGTGC

TGTAGTCTATTCGAGAAATTCTTCCCCAGCAGTTCATACAGGAGGCCAGTTGGAATTTCC

AGCATGGTGGAGGCCATGGTGTCTAGGGCCCGAATTGATGCACGCATCGACTTCGAATCT

GGAAGGATTAAGAAAGAAGAGTTTGCTGAGATCATGAAGATCTGTTCCACCATTGAAGAG

CTCAGACGGCAAAAATAGTGAATTTAGCGTGTCCTTCATGAAAAAATGCCTTGTTTCTAC

T

>A_mute_swan_North_Ossetia-Alania_325-03_2020_EPI1848732

AGCAAAAGCAGGCAAACTATTTGAATGGATGTCAATCCGACTTTACTTTTCTTAAAAGTG

CCAGCGCAAAATGCTATAAGTACCACATTCCCTTACACTGGAGATCCTCCATACAGCCAT

GGAACAGGAACAGGGTATACCATGGACACAGTAAACAGAACACATCAATACTCAGAAAAG

GGAAAGTGGACAACAAACACAGAAACCGGAGCACCCCAACTCAACCCAATTGATGGACCA

TTACCAGAGGACAATGAGCCAAGCGGATATGCACAAACTGATTGCGTGTTGGAAGCAATG

GCTTTCCTTGAAGAATCCCACCCAGGGATATTTGAAAACTCTTGTCTTGAAGCGATGGAA

ATCGTTCAGCAAACAAGAGTGGACAAATTAACCCAAGGTCGCCAGACTTATGACTGGACA

CTGAACAGAAACCAACCAGCTGCAACCTCTTTGGCCAACACTATAGAGGTGTTCAGATCG

AATGGTCTGACAGCCAATGAATCAGGGAGACTGATAGATTTTCTCAGGGATGTGATGGAA

TCAATGGATAAAGAAGAGATGGAAGTAACAACACATTTCCAGAGAAAAAGAAGAGTGAGG

GACAACATGACTAAGAAGATGGTCACACAAAGAACAATAGGGAAGAAGAAGCAGAGGCTG

AACAAGAGGAGTTACTTAATAAGAGCACTGACATTGAATACAATGACCAAAGATGCAGAA

AGGGGCAAGTTGAAGAGACGGGCAATTGCAACACCCGGGATGCAGATTAGAGGATTCGTG

TACTTCGTTGAAACACTAGCGAGGAGCATCTGTGAGAAACTAGAGCAATCTGGGCTCCCT

GTTGGAGGGAATGAGAAGAAGGCTAAATTGGCAAATGTTGTGAGAAAAATGATGACTAAC

TCACAAGATACAGAGCTCTCCTTTACAATTACTGGAGACAACACCAAATGGAATGAGAAT

CAAAACCCTCGGATGTTTTTGGCAATGATAACATATATCACAAGAAACCAACCTGAATGG

TTTAGAAATGTCTTAAGCATTGCCCCTATAATGTTCTCAAACAAAATGGCGAGATTAGGG

AAAGGATACATGTTTGAGAGTAAGAGCATGAAGCTAAGAACACAAATACCAGCAGAGATG

CTTACAAATATTGATCTGAAGTATTTCAACGAACCAACGAGAAAGAAAATCGAGAAAATA

AGACCTCTGCTGATTGATGGCACGGCCTCATTGAGTCCTGGGATGATGATGGGCATGTTC

AATATGCTGAGCACAGTATTGGGGGTCTCAATCCTGAATCTCGGGCAAAAGAGGTACACC

AAAACCACATACTGGTGGGATGGACTTCAATCCTCTGATGATTTCGCTCTCATAGTGAAT

GCACCGAATCATGAGGGGATACAAGCAGGAGTGGATAGATTCTATAGGACCTGCAAACTG

GTTGGGATCAACATGAGCAAAAAGAAGTCTTACATAAACCGAACAGGAACATTTGAATTC

ACAAGTTTTTTCTACCGCTATGGATTTGTAGCTAACTTCAGTATGGAATTACCCAGCTTT

GGAGTGTCTGGAATCAATGAATCAGCTGACATGAGCATTGGAGTTACAGTGATAAAAAAC

AATATGATAAACAATGATCTTGGACCAGCGACAGCTCAAATGGCTCTTCAGTTATTCATC

AAAGACTATAGGTACACATACCGATGCCACAGGGGTGATACACAAATTCAAACGAGGAGA

TCATTCGAGCTGAAGAAGCTGTGGGAGCAGACCCGTTCAAAGGCAGGGCTGTTGATATCA

GACGGGGGGCCAAACCTATACAACATTCGGAACCTCCACATCCCAGAGGTCTGCTTGAAG

TGGGAGCTGATGGATGAAGACTACCAAGGCAGGCTGTGCAATCCTCTGAATCCATTTGTC

AGTCATAAAGAGATTGAGTCCGTAAACAATGCTGTAGTAATGCCCGCCCATGGCCCGGCC

AAGAGCATGGAATATGATGCTGTTGCGACCACACATTCGTGGATTCCTAAGAGGAACCGT

TCCATTCTCAATACCAGCCAAAGGGGAATTCTTGAGGATGAGCAGATGTACCAAAAGTGC

TGTAGTCTATTCGAGAAATTCTTCCCCAGCAGTTCATACAGGAGGCCAGTTGGAATTTCC

AGCATGGTGGAGGCCATGGTGTCTAGGGCCCGAATTGATGCACGCATCGACTTCGAATCT

GGAAGGATTAAGAAAGAAGAGTTTGCTGAGATCATGAAGATCTGTTCCACCATTGAAGAG

CTCAGACGGCAAAAATAGTGAATTTAGCTTGTCCTTCATGAAAAAATGCCTTGTTTCTAC

T

>A_turkey_Rostov-on-Don_332-09_2021_EPI1848756

AGCAAAAGCAGGCAAACTATTTGAATGGATGTCAATCCGACTTTACTTTTCTTAAAAGTG

CCAGCGCAAAATGCTATAAGTACTACATTCCCTTACACTGGAGATCCTCCATACAGCCAT

GGGACAGGAACAGGGTATACCATGGACACAGTAAACAGAACACATCAATACTCAGAAAAG

GGAAAGTGGACAACAAACACAGAAACCGGAGCACCCCAACTCAACCCAATTGATGGACCA

TTACCAGAGGACAATGAGCCAAGCGGATATGCACAAACTGATTGCGTGTTGGAAGCAATG

GCTTTCCTTGAAGAATCCCACCCAGGGATATTTGAAAACTCTTGTCTTGAAGCGATGGAA

ATCGTTCAGCAAACAAGAGTGGACAAACTAACCCAAGGTCGCCAGACTTATGACTGGACA

CTGAACAGAAACCAACCAGCTGCAACCTCTTTGGCCAACACTATAGAGGTGTTCAGATCG

AATGGTCTGACAGCCAATGAATCAGGGAGACTGATAGATTTTCTCAGGGATGTGATGGAA

TCAATGGATAAAGAAGAGATGGAAGTAACAACACATTTCCAGAGAAAAAGAAGAGTGAGG

GACAACATGACTAAGAAGATGGTCACACAAAGAACAATAGGGAAGAAGAAGCAGAGGATG

AACAAGAGGAGTTACTTGATAAGAGCACTGACATTGAATACAATGACCAAAGATGCAGAA

AGAGGCAAGTTGAAGAGACGGGCAATTGCAACACCCGGGATGCAGATTAGAGGATTCGTG

TACTTCGTTGAAACACTAGCGAGGAGCATCTGTGAGAAACTAGAGCAATCTGGGCTCCCT

GTTGGAGGGAATGAGAAGAAGGCTAAATTGGCAAATGTTGTGAGAAAAATGATGACTAAC

TCACAAGATACAGAGCTCTCCTTTACAATTACTGGAGACAACACCAAATGGAATGAGAAT

CAAAACCCTCGGATGTTTTTGGCAATGATAACATATATCACAAGAAACCAACCTGAATGG

TTTAGAAATGTCTTAAGCATTGCCCCTATAATGTTCTCAAACAAAATGGCGAGATTAGGG

AAAGGATACATGTTTGAAAGTAAGAGCATGAAGCTAAGAACACAAATACCAGCAGAGATG

CTTACAAATATTGATCTGAAGTATTTCAACGAACCAACGAGAAAGAAAATCGAGAAAATA

AGACCTCTGCTGATTGATGGCACGGCCTCATTGAGTCCTGGGATGATGATGGGCATGTTC

AATATGCTGAGCACAGTATTGGGGGTCTCAATCCTGAATCTCGGGCAAAAGAGGTACACC

AAAACCACATACTGGTGGGATGGACTTCAATCCTCTGATGATTTCGCTCTCATAGTGAAT

GCACCGAATCATGAGGGGATACAAGCAGGAGTGGATAGATTCTATAGGACCTGCAAACTG

GTTGGGATCAACATGAGCAAAAAGAAGTCTTACATAAACCGAACAGGAACATTTGAGTTC

ACAAGTTTTTTCTACCGCTATGGATTTGTAGCTAACTTCAGTATGGAATTACCCAGCTTT

GGAGTGTCTGGAATCAATGAATCAGCTGACATGAGCATTGGAGTTACAGTGATAAAAAAC

AATATGATAAACAATGATCTTGGACCAGCAACAGCTCAAATGGCTCTTCAGTTATTCATC

AAAGACTATAGGTACACATACCGATGCCACAGGGGTGATACACAAATTCAAACGAGGAGA

TCATTCGAGCTGAAGAAGCTGTGGGAGCAGACCCGTTCAAAGGCAGGGCTGTTGATATCA

GACGGGGGGCCAAACCTATACAACATTCGGAACCTCCACATCCCAGAGGTCTGCTTGAAG

TGGGAGCTGATGGATGAAGACTACCAAGGCAGGCTGTGCAATCCTCTGAATCCATTTGTC

AGTCATAAAGAGATTGAGTCCGTAAACAATGCTGTAGTAATGCCCGCCCATGGCCCGGCC

AAGAGCATGGAATATGATGCTGTTGCGACCACACATTCGTGGATTCCTAAGAGGAACCGC

TCCATTCTCAATACCAACCAAAGGGGAATTCTTGAGGATGAGCAGATGTACCAAAAGTGC

TGTAGTCTATTCGAGAAATTCTTCCCCAGCAGTTCATACAGGAGGCCAGTTGGAATTTCC

AGCATGGTGGAGGCCATGGTGTCTAGGGCCCGAATTGATGCACGCATCGACTTCGAATCT

GGAAGGATTAAGAAGGAAGAGTTTGCTGAGATCATGAAGATCTGTTCCACCATTGGAGAG

CTCAGACGGCAAAAATAGTGAATTTAGCTTGTCCTTCATGAAAAAATGCCTTGTTTCTAC

T

>A_chicken_Krasnodar_334-03_2021_EPI1848804

AGCAAAAGCAGGCAAACTATTTGAATGGATGTCAATCCGACTTTACTTTTCTTAAAAGTG

CCAGCGCAAAATGCTATAAGTACTACATTCCCTTACACTGGAGATCCTCCATACAGCCAT

GGAACAGGAACAGGGTATACCATGGACACAGTAAACAGAACACATCAATACTCAGAAAAG

GGAAAGTGGACAACAAACACAGAAACCGGAGCACCCCAACTCAACCCAATTGATGGACCA

TTACCAGAGGACAATGAGCCAAGCGGATATGCACAAACTGATTGCGTGTTGGAAGCAATG

GCTTTCCTTGAAGAATCCCACCCAGGGATATTTGAAAACTCTTGTCTTGAAGCGATGGAA

ATCGTTCAGCAAACAAGAGTGGACAAACTAACCCAAGGTCGCCAGACTTATGACTGGACA

CTGAACAGAAACCAACCAGCTGCAACCTCTTTGGCCAACACTATAGAGGTGTTCAGATCG

AATGGTCTGACAGCCAATGAATCAGGGAGACTGATAGATTTTCTCAGGGATGTGATGGAA

TCAATGGATAAAGAAGAGATAGAAGTAACAACACATTTCCAGAGAAAAAGAAGAGTGAGG

GACAACATGACTAAGAAGATGGTCACACAAAGAACAATAGGGAAGAAGAAGCAGAGGCTG

AACAAGAGGAGTTACTTAATAAGAGCACTGACATTGAATACAATGACCAAAGATGCAGAA

AGAGGCAAGTTGAAGAGACGGGCAATTGCAACACCCGGGATGCAGATTAGAGGATTCGTG

TACTTTGTTGAAACACTAGCGAGGAGCATCTGTGAGAAACTAGAGCAATCTGGGCTCCCT

GTTGGAGGGAATGAGAAGAAGGCTAAATTGGCAAATGTTGTGAGAAAAATGATGACTAAC

TCACAAGATACAGAGCTCTCCTTTACAATTACTGGAGACAACACCAAATGGAATGAGAAT

CAAAACCCTCGGATGTTTTTGGCAATGATAACATATATCACAAGAAATCAACCTGAATGG

TTTAGAAATGTCTTAAGCATTGCCCCTATAATGTTCTCAAACAAAATGGCGAGATTAGGA

AAAGGATACATGTTTGAAAGTAAGAGCATGAAGCTAAGAACACAAATACCAGCAGAGATG

CTTACAAATATCGATCTGAAGTATTTCAACGAACCAACGAGAAAGAAAATCGAGAAAATA

AGACCTCTGCTGATTGATGGCACGGCCTCATTGAGTCCTGGGATGATGATGGGCATGTTC

AATATGCTGAGCACAGTATTAGGGGTCTCAATCCTGAATCTCGGGCAAAAAAGGTACACC

AAAACCACATACTGGTGGGATGGACTTCAATCCTCTGATGATTTTGCTCTCATAGTGAAT

GCACCGAATCATGAGGGGATACAAGCAGGAGTGAATAGATTCTATAGGACCTGCAAACTG

GTTGGGATCAACATGAGCAAAAAGAAGTCTTACATAAACCGAACAGGAACATTTGAGTTC

ACAAGTTTTTTCTACCGCTATGGATTTGTAGCTAACTTCAGTATGGAATTACCCAGCTTT

GGAGTGTCTGGAATCAATGAATCAGCTGACATGAGCATTGGAGTTACAGTGATAAAAAAC

AATATGATAAACAATGATCTTGGACCAGCAACAGCTCAAATGGCTCTTCAGTTATTCATC

AAAGATTATAGGTACACATACCGATGCCACAGGGGTGATACACAAATTCAAACGAGGAGA

TCATTCGAGCTGAAGAAGCTGTGGGAGCAGACCCGTTCAAAAGCAGGGCTGTTGATATCA

GACGGGGGGCCAAACCTATACAACATTCGGAATCTCCACATCCCAGAGGTCTGCTTGAAG

TGGGAGCTGATGGATGAAGACTACCAAGGCAGGCTGTGCAATCCTCTGAATCCATTTGTC

AGTCATAAAGAGATTGAGTCCGTAAACAATGCTGTAGTAATGCCCGCCCATGGCCCGGCC

AAGAGCATGGAATATGATGCTGTTGCGACCACACACTCGTGGATTCCTAAGAGGAACCGT

TCCATTCTCAATACCAGCCAGAGGGGAATTCTTGAGGATGAGCAGATGTACCAAAAGTGC

TGTAGTCTATTCGAGAAATTCTTCCCCAGCAGTTCATACAGGAGGCCAGTTGGAATTTCC

AGCATGGTGGAGGCCATGGTGTCTAGGGCCCGAATTGATGCACGCATCGACTTCGAATCT

GGAAGGATTAAGAAAGAAGAGTTTGCTGAGATCATGAAGATCTGTTCCACCATTGAAGAG

CTCAGACGGCAAAAATAGTGAATTTAGCTTGTCCTTCATGAAAAAATGCCTTGTTTCTAC

T

>A_pheasant_Wales_000252_2021_EPI1848884

AGCGAAAGCAGGCAAACTATTTGAATGGATGTCAATCCGACTTTACTTTTCTTAAAAGTG

CCAGCGCAAAATGCTATAAGTACTACATTCCCTTACACTGGAGATCCTCCATACAGCCAT

GGAACAGGAACAGGGTATACCATGGACACAGTAAACAGAACACATCAATACTCAGAAAAG

GGAAAGTGGACAACAAACACAGAAACCGGAGCACCCCAACTCAACCCAATTGATGGACCA

TTACCAGAGGACAATGAGCCAAGCGGATATGCACAAACTGATTGCGTGTTGGAAGCAATG

GCTTTCCTTGAAGAATCCCACCCAGGGATATTTGAAAACTCTTGTCTAGAAGCGATGGAA

ATCGTTCAGCAAACAAGAGTGGACAAACTAACCCAAGGTCGCCAGACTTATGACTGGACA

CTGAACAGAAACCAACCAGCTGCAACCTCTTTGGCCAACACTATAGAGGTGTTCAGATCG

AATGGTCTGACAGCCAATGAATCAGGGAGACTGATAGATTTTCTCAGAGATGTGATGGAA

TCAATGGATAAAGAAGAGATGGAAGTAACAACACATTTCCAGAGAAAAAGAAGAGTGAGG

GACAACATGACTAAGAAGATGGTCACACAAAGAACAATAGGGAAGAAGAAGCAGAGGCTG

AACAAGAGGAGTTACTTAATAAGAGCACTGACATTGAATACAATGACCAAAGATGCAGAA

AGAGGCAAGTTGAAGAGACGGGCAATTGCAACACCCGGGATGCAGATTAGAGGATTCGTG

TACTTCGTTGAAACACTAGCGAGGAGCATCTGTGAGAAACTAGAGCAATCTGGGCTCCCT

GTTGGAGGGAATGAGAAGAAGGCTAAATTGGCAAATGTTGTGAGAAAAATGATGACTAAC

TCACAAGATACAGAGCTCTCCTTTACAATTACTGGAGACAACACCAAATGGAATGAGAAT

CAAAACCCTCGGATGTTTTTGGCAATGATAACATATATCACAAGAAACCAACCTGAATGG

TTTAGAAATGTCTTAAGCATTGCCCCTATAATGTTCTCAAACAAAATGGCGAGATTAGGG

AAAGGATACATGTTTGAAAGTAAGAGCATGAAGCTAAGAACACAAATACCAGCAGAGATG

CTTACAAATATCGATCTGAAGTATTTCAACGAACCAACGAGAAAGAAAATCGAGAAAATA

AGACCTCTGCTGATTGATGGCACGGCCTCATTGAGTCCTGGGATGATGATGGGCATGTTC

AATATGCTGAGCACAGTATTAGGGGTCTCAATCCTGAATCTCGGGCAAAAAAGGTACACC

AAAACCACATACTGGTGGGATGGACTTCAATCCTCTGATGATTTCGCTCTCATAGTGAAT

GCACCGAATCATGAGGGGATACAAGCAGGAGTGGATAGATTCTATAGGACCTGCAAACTG

GTTGGGATCAACATGAGCAAGAAGAAGTCTTACATAAACCGAACAGGAACATTTGAGTTC

ACAAGTTTTTTCTACCGCTATGGATTTGTAGCTAACTTCAGTATGGAATTACCCAGCTTT

GGAGTGTCTGGAATCAATGAATCAGCTGACATGAGCATTGGAGTTACAGTGATAAAAAAC

AACATGATAAACAATGATCTTGGACCAGCAACAGCTCAAATGGCTCTTCAGTTATTCATC

AAAGACTATAGATACACATACCGATGCCACAGGGGTGATACACAAATTCAAACGAGGAGA

TCATTCGAGCTGAAGAAGCTGTGGGAGCAGACCCGTTCAAAGGCAGGGCTGTTGATATCA

GACGGGGGGCCAAACCTATACAACATTCGGAATCTCCACATCCCAGAGGTCTGCTTGAAG

TGGGAGCTGATGGATGAAGACTACCAAGGCAGGCTGTGCAATCCTCTGAATCCATTTGTC

AGTCATAAAGAGATTGAGTCCGTAAACAATGCTGTAGTAATGCCCGCTCATGGCCCGGCC

AAGAGCATGGAATATGATGCTGTTGCGACCACACACTCGTGGATTCCTAAGAGGAACCGT

TCCATTCTCAATACCAGCCAAAGGGGAATTCTTGAGGATGAGCAGATGTACCAAAAGTGC

TGTAGTCTATTCGAGAAATTCTTCCCCAGCAGTTCATACAGGAGGCCAGTTGGAATTTCC

AGCATGGTGGAGGCCATGGTGTCTAGGGCCCGAATTGATGCACGCATCGACTTCGAATCT

GGAAGGATTAAGAAAGAAGAGTTTGCTGAGATCATGAAGATCTGTTCCACCATTGAAGAG

CTCAGACGGCAAAAATAGTGAATTTAGCTTGTCCTTCATGAAAAAATGCCTTGTTTCTAC

T

>A_mute_swan_Czech_Republic_1656-1_2021_EPI1850126

------------------------ATGGATGTCAATCCGACTTTACTTTTCTTAAAAGTG

CCAGCGCAAAATGCTATAAGTACTACATTCCCTTACACTGGAGATCCCCCATACAGCCAT

GGAACAGGAACAGGGTATACCATGGACACAGTAAACAGAACACATCAATACTCAGAAAAG

GGAAAGTGGACAACAAACACAGAAACCGGAGCACCCCAACTCAACCCAATTGATGGACCA

TTACCAGAGGACAATGAGCCAAGCGGATATGCACAAACTGATTGCGTGTTGGAAGCAATG

GCTTTCCTTGAAGAATCCCACCCAGGGATATTTGAAAACTCTTGTCTTGAAGCGATGGAA

ATCGTTCAGCAAACAAGAGTGGACAAACTAACCCAAGGTCGCCAGACTTATGACTGGACA

CTGAACAGAAACCAACCAGCTGCAACCTCTTTGGCCAACACTATAGAGGTGTTCAGATCG

AATGGTCTGACAGCCAATGAATCAGGGAGACTGATAGATTTTCTCAGGGATGTGATGGAA

TCAATGGATAAAGAAGAGATGGAAGTAACAACACATTTCCAGAGAAAAAGAAGAGTGAGG

GACAACATGACTAAGAAGATGGTCACACAAAGAACAATAGGGAAGAAGAAGCAGAGGCTG

AACAAGAGGAGTTACTTAATAAGAGCACTGACATTGAATACAATGACCAAAGATGCAGAA

AGAGGCAAGTTGAAGAGACGGGCAATTGCAACACCCGGGATGCAGATTAGAGGATTCGTG

TACTTCGTTGAAACACTAGCGAGGAGCATCTGTGAGAAACTAGAGCAATCTGGGCTCCCT

GTTGGAGGGAATGAGAAGAAGGCTAAATTGGCAAATGTTGTGAGAAAAATGATGACTAAC

TCACAAGATACAGAGCTCTCCTTTACAATTACTGGAGACAACACCAAATGGAATGAGAAT

CAAAACCCTCGGATGTTTTTGGCAATGATAACATATATCACAAGAAACCAACCTGAATGG

TTTAGAAATGTCTTAAGCATTGCCCCTATAATGTTCTCAAACAAAATGGCGAGATTAGGG

AAAGGATACATGTTTGAAAGTAAGAGCATGAAGCTAAGAACACAAATACCAGCAGAGATG

CTTACAAATATTGATCTGAAGTATTTCAACGAACCAACGAGAAAGAAAATCGAGAAAATA

AGACCTCTGCTGATTGATGGCACGGCCTCATTGAGTCCTGGGATGATGATGGGCATGTTC

AATATGCTGAGCACAGTATTAGGGGTCTCAATCCTGAATCTCGGGCAAAAGAGGTACACC

AAAACCACATACTGGTGGGATGGACTTCAATCCTCTGATGATTTCGCTCTCATAGTGAAT

GCACCGAATCATGAGGGGATACAAGCAGGAGTGGATAGATTCTATAGGACCTGCAAACTG

GTTGGGATCAACATGAGCAAAAAGAAGTCTTACATAAACCGAACAGGAACATTTGAGTTC

ACAAGTTTTTTCTACCGCTATGGATTTGTAGCCAACTTCAGTATGGAATTACCCAGCTTT

GGAGTGTCTGGAATCAATGAATCAGCTGACATGAGCATTGGAGTTACAGTGATAAAAAAC

AATATGATAAACAATGATCTTGGACCAGCAACAGCTCAAATGGCTCTTCAGTTATTCATC

AAAGACTATAGGTACACATACCGATGCCACAGGGGTGATACACAAATTCAAACGAGGAGA

TCATTCGAGCTGAAGAAGCTGTGGGAGCAGACCCGTTCAAAGGCAGGGCTGTTGATATCA

GACGGGGGGCCAAATCTATACAACATTCGGAATCTCCACATCCCAGAGGTCTGCTTGAAG

TGGGAGCTGATGGATGAAGACTACCAAGGCAGGCTGTGCAATCCTCTGAATCCATTTGTC

AGTCATAAAGAGATTGAGTCCGTAAACAATGCTGTAGTAATGCCCGCCCATGGCCCGGCC

AAGAGCATGGAATATGATGCTGTTGCGACCACACACTCGTGGATTCCTAAGAGGAACCGT

TCCATTCTCAATACCAGCCAAAGGGGAATTCTTGAGGATGAGCAGATGTACCAAAAGTGC

TGTAGTCTATTCGAGAAATTCTTCCCCAGCAGTTCATACAGGAGGCCAGTTGGAATTTCC

AGCATGGTGGAGGCCATGGTGTCTAGGGCCCGAATTGATGCACGCATCGACTTCGAATCT

GGAAGGATTAAGAAAGAAGAGTTTGCTGAGATCATGAAGATCTGTTCCACCATTGAAGAG

CTCAGACGGCAAAAATAGTGAATTTAGCTTGTCCTTCATGAAA-----------------

-

>A_mute_swan_Croatia_14_2021_EPI1850960

------------CAAACTATTTGAATGGATGTCAATCCGACTTTACTTTTCTTAAAAGTG

CCAGCGCAAAATGCTATAAGTACTACATTCCCTTACACTGGAGATCCTCCATACAGCCAT

GGAACAGGAACAGGGTATACCATGGACACAGTAAACAGAACACATCAATACTCAGAAAAG

GGAAAATGGACAACAAACACAGAAACCGGAGCACCCCAACTCAACCCAATTGATGGACCA

TTACCAGAGGACAATGAGCCAAGCGGATATGCACAAACTGATTGCGTGTTGGAAGCAATG

GCTTTCCTTGAAGAATCCCACCCAGGGATATTTGAAAACTCTTGTCTTGAAGCGATGGAA

ATCGTTCAGCAAACAAGAGTGGACAAACTAACCCAAGGTCGCCAGACTTATGACTGGACA

CTGAACAGAAACCAACCAGCTGCAACCTCTTTGGCCAACACTATAGAGGTGTTCAGATCG

AATGGTCTGACAGCCAATGAATCAGGGAGACTGATAGATTTTCTCAGGGATGTGATGGAA

TCAATGGATAAAGAAGAGATGGAAGTAACAACACATTTCCAGAGAAAAAGAAGAGTGAGG

GACAACATGACTAAGAAGATGGTCACACAAAGAACAATAGGGAAGAAGAAGCAGAGGCTG

AACAAGAGGAGTTACTTAATAAGAGCACTGACATTGAATACAATGACCAAAGATGCAGAA

AGAGGCAAGTTGAAGAGACGGGCAATTGCAACACCCGGGATGCAGATTAGAGGATTCGTG

TACTTCGTTGAAACACTAGCGAGGAGCATCTGTGAGAAACTAGAGCAATCTGGGCTCCCT

GTTGGAGGGAATGAGAAGAAGGCTAAATTGGCAAATGTTGTGAGAAAAATGATGACTAAC

TCACAAGATACAGAGCTCTCCTTTACAATTACTGGGGACAACACCAAATGGAATGAGAAT

CAAAACCCTCGGATGTTTTTGGCAATGATAACATATATCACAAGAAACCAACCTGAATGG

TTTAGAAATGTCTTAAGCATTGCCCCTATAATGTTCTCAAACAAAATGGCGAGATTAGGG

AAAGGATACATGTTTGAAAGTAAGAGCATGAAGCTAAGAACACAAATACCAGCAGAGATG

CTTACAAATATCGATCTGAAGTATTTCAACGAACCAACGAGAAAGAAAATCGAGAAAATA

AGACCTCTGCTGATTGATGGCACGGCCTCATTGAGTCCTGGGATGATGATGGGCATGTTC

AATATGCTGAGCACAGTATTAGGGGTCTCAATCCTGAATCTCGGGCAAAAAAGGTACACC

AAAACCACATACTGGTGGGATGGACTTCAATCCTCTGATGATTTCGCTCTCATAGTGAAT

GCACCGAATCATGAGGGGATACAAGCAGGAGTGGATAGATTCTATAGGACCTGCAAACTG

GTTGGGATCAACATGAGCAAAAAGAAGTCTTACATAAACCGAACAGGAACATTTGAGTTC

ACAAGTTTTTTCTATCGCTATGGATTTGTAGCTAACTTCAGTATGGAATTACCCAGCTTT

GGAGTGTCTGGAATCAATGAATCAGCTGACATGAGCATTGGAGTTACAGTGATAAAAAAC

AATATGATAAACAATGATCTTGGACCAGCAACAGCTCAAATGGCTCTTCAGTTATTCATC

AAAGATTATAGGTACACATACCGATGCCACAGGGGTGATACACAAATTCAAACGAGGAGA

TCATTCGAGCTGAAGAAGCTGTGGGAGCAGACCCGTTCAAAGGCAGGGCTGTTGATATCA

GACGGGGGGCCAAACCTATACAACATTCGGAATCTCCACATCCCAGAGGTCTGCTTGAAG

TGGGAGCTGATGGATGAAGACTACCAAGGCAGGCTGTGCAATCCTCTGAATCCATTTGTC

AGTCATAAAGAGATTGAGTCCGTAAACAATGCTGTAGTGATGCCCGCCCATGGCCCGGCC

AAGAGCATGGAATATGATGCTGTTGCGACCACACACTCGTGGATTCCTAAGAGGAACCGT

TCCATTCTCAATACCAGCCAAAGGGGAATTCTTGAGGATGAGCAGATGTACCAAAAGTGC

TGTAGTCTATTCGAGAAATTCTTCCCCAGCAGTTCATACAGGAGGCCAGTTGGAATTTCC

AGCATGGTGGAGGCCATGGTGTCTAGGGCCCGAATTGATGCACGCATCGACTTCGAATCT

GGAAGGATTAAGAAAGAAGAGTTTGCTGAGATCATGAAGATCTGTTCCACCATTGAAGAG

CTCAGACGGCAAAAATAGTGAATTTAGCTTGTCCTTCATG--------------------

-

>A_chicken_Vietnam_Raho4-Cd-20-421_2020_EPI1853936

------------CAAACCATTTGAATGGATGTCAATCCGACTTTACTTTTCTTGAAAGTT

CCAGCTCAAAATGCTATAAGTACTACATTCCCTTATACTGGAGACCCTCCATACAGCCAT

GGAACGGGAACAGGATATACCATGGATACAGTCAACAGAACACATCAATACTCAGAAAAG

GGGGAATGGACAACAAACACAGAGACTGGAGCACCTCAACTCAATCCAATTGATGGACCA

TTACCCGAGGACAATGAGCCAAGTGGATATGCACAAACAGATTGTGTATTGGAAGCAATG

GCTTTCCTTGAAGAATCCCACCCGGGGATCTTTGAAAACTCGTGTCTTGAAACGATGGAA

ATTGTCCAGCAAACAAGGGTGGATAAACTGACCCAAGGTCGCCAGACCTATGACTGGACA

TTGAATAGAAACCAGCCGGCTGCAACTGCATTGGCCAACACTATAGAGGTCTTCAGATCT

AACGGTCTAACAGCCAATGAATCAGGAAGACTAATAGATTTTCTTAAGGACGTGATGGAA

TCAATGGATAAGGAAGGAATGGAAATAACAACACATTTCCAGAGAAAGAGAAGAGTGAGG

GACAATATGACCAAGAAAATGATCACACAGAGAACAATAGGGAAGAAAAAACAAAGGTTG

AGCAAAAGGAGCTACCTAATAAGAGCATTAACACTGAACACAATGACAAAAGATGCAGAA

AGAGGAAAACTGAAGAGACGGGCAATTGCTACACCCGGAATGCAAATCAGGGGATTTGTA

TACTTTGTCGAAACACTAGCAAGGAGTATCTGTGAGAAACTTGAGCAATCTGGACTCCCA

GTCGGAGGGAATGAGAAGAAAGCTAAATTGGCTAATGTTGTGAGGAAAATGATGACTAGC

TCACAAGACACAGAACTCTCCTTTACAATTACTGGAGACAACACCAAATGGAATGAGAAT

CAAAATCCTCGAATGTTTCTGGCAATGATAACATACATCACAAGGAACCAACCAGAATGG

TTTAGAAATGTCTTAAGCATTGCCCCTATAATGTTCTCAAACAAAATGGCGAGATTAGGG

AAAGGGTACATGTTCGAAAGTAAAAGCATGAAGCTACGGACGCAAATACCAGCAGAAATG

CTTGCAAACATTGATCTGAAATACTTCAATGAATCAACGAGAAAGAAAATTGAAAGAATA

AGACCTCTACTAATAGATGGCACAGCCTCATTGAGTCCTGGAATGATGATGGGCATGTTC

AACATGCTGAGTACTGTCTTAGGAGTCTCAATCCTGAATCTTGGACAGAAGAGATACACC

AAAACAACATACTGGTGGGATGGACTTCAATCCTCTGATGATTTCGCTCTCATAGTGAAT

GCACCGAATCATGAGGGAATACAAGCAGGGGTGGATAGATTCTACAGAACTTGCAAACTA

GTTGGGATCAACATGAGCAAGAAGAAATCTTACATAAATCGAACAGGAACATTTGAGTTT

ACAAGCTTTTTCTACCGTTACGGATTTGTGGCCAATTTCAGTATGGAACTGCCCAGCTTT

GGAGTGTCTGGAATCAATGAATCAGCTGACATGAGCATTGGTGTCACAGTGATAAAAAAC

AATATGATAAACAATGATCTTGGACCAGCAACAGCTCAGATGGCTCTTCAGCTATTCATC

AAGGATTACAGATACACTTACCGGTGCCACAGGGGTGATACGCAAATCCAAACGAGGAGA

TCATTTGAGTTGAAGAAACTGTGGGAACAGACCCGTTCAAAGGCAGGGCTGCTGGTTTCA

GATGGAGGACCAAACCTATACAACATCCGAAACCTCCACATTCCAGAGGTCTGCTTGAAA

TGGGAGTTGATGGATGACGATTATCAGGGCAGACTGTGTAATCCTTTGAATCCATTTGTC

AGTCATAAGGAGATTGAATCTGTCAACAACGCTGTAGTAATGCCAGCCCATGGTCCAGCT

AAGAACATGGAATATGATGCTGTTGCAACCACCCACTCATGGATCCCTAAAAGGAACCGC

TCCATTCTTAACACAAGCCAAAGAGGAATCCTTGAGGATGAGCAGATGTACCAAAAGTGT

TGCAATCTATTTGAGAAGTTCTTCCCTAGTAGTTCATATCGGAGACCAGTTGGAATCTCC

AGCATGGTGGAGGCCATGGTGTCTAGGGCCCGAATTGATGCACGAATTGACTTCGAGTCT

GGAAGGATTAAGAAAGAGGAGTTTGCTGAGATCATGAAGATCTGTTCCACCATTGAAGAG

CTCAGACGGCAAAAATAGTGAATTTAGCTTGTCCTTCATGAAAAAATG------------

-

>A_chicken_Czech_Republic_3531-1_2021_EPI1854241

------------------------ATGGATGTCAATCCGACTTTACTTTTCTTAAAAGTG

CCAGCGCAAAATGCTATAAGTACTACATTCCCTTACACTGGAGATCCTCCATACAGCCAT

GGAACAGGAACAGGGTATACCATGGACACAGTAAACAGAACACATCAATACTCAGAAAAG

GGAAAGTGGACAACAAACACAGAAACCGGAGCACCCCAACTCAACCCAATTGATGGACCA

TTGCCAGAGGACAATGAGCCAAGCGGATATGCACAAACTGATTGCGTGTTGGAAGCAATG

GCTTTCCTTGAAGAATCCCACCCAGGGATATTTGAAAACTCTTGTCTTGAAGCGATGGAA

ATCGTTCAGCAAACAAGAGTGGACAAACTAACCCAAGGTCGTCAGACTTATGACTGGACA

CTGAACAGAAACCAACCAGCTGCAACCTCTTTGGCCAACACTATAGAGGTGTTCAGATCG

AATGGTCTGACAGCCAATGAATCAGGGAGACTGATAGATTTTCTCAGGGATGTGATGGAA

TCAATGGATAAAGAAGAGATGGAAGTAACAACACATTTCCAGAGAAAAAGAAGAGTGAGG

GACAACATGACTAAGAAGATGGTCACACAAAGAACAATAGGGAAGAAGAAGCAGAGGCTG

AACAAGAGGAGTTACTTAATAAGAGCACTGACATTGAATACAATGACCAAAGATGCAGAA

AGAGGCAAGTTGAAGAGACGGGCAATTGCAACACCCGGGATGCAGATTAGAGGATTCGTG

TACTTCGTTGAAACACTAGCGAGGAGCATCTGTGAGAAACTAGAGCAATCTGGGCTCCCT

GTTGGAGGGAATGAGAAGAAGGCTAAATTGGCAAATGTTGTGAGAAAAATGATGACTAAC

TCACAAGATACAGAGCTCTCCTTTACAATTACTGGAGACAACACCAAATGGAATGAGAAT

CAAAACCCTCGGATGTTTTTGGCAATGATAACATATATCACAAGAAACCAACCTGAATGG

TTTAGAAATGTCTTAAGCATTGCCCCTATAATGTTCTCAAACAAAATGGCGAGATTAGGG

AAAGGATACATGTTTGAAAGTAAGAGCATGAAGCTAAGAACACAAATACCAGCAGAGATG

CTTACAAATATCGATCTGAAGTATTTCAACGAACCAACGAGAAAGAAAATCGAGAAAATA

AGACCTCTGCTGATTGATGGCACGGCCTCATTGAGTCCTGGAATGATGATGGGCATGTTC

AATATGCTGAGCACAGTATTAGGGGTCTCAATCCTGAATCTCGGGCAAAAAAGGTACACC

AAAACCACATACTGGTGGGATGGACTTCAATCCTCTGATGATTTCGCTCTCATAGTGAAT

GCACCGAATCATGAGGGGATACAAGCAGGAGTGGATAGATTCTATAGGACCTGCAAACTG

GTTGGGATCAACATGAGCAAAAAGAAGTCTTACATAAACCGAACAGGAACATTTGAGTTC

ACAAGTTTYTTCTACCGCTATGGATTTGTAGCTAACTTCAGTATGGAATTACCCAGCTTT

GGAGTGTCTGGAATCAATGAATCAGCTGACATGAGCATTGGAGTTACAGTGATAAAAAAC

AATATGATAAACAATGATCTTGGACCAGCAACAGCTCAAATGGCTCTTCAGTTATTCATC

AAAGACTATAGGTACACATACCGATGCCACAGGGGTGATACACAAATTCAAACGAGGAGA

TCATTCGAGCTGAAGAAGCTGTGGGAGCAGACCCGTTCAAAGGCAGGGCTGTTGATATCA

GACGGGGGGCCAAACCTATACAACATTCGGAATCTCCACATCCCAGAGGTCTGCTTGAAG

TGGGAGCTGATGGATGAAGACTACCAAGGCAGGCTGTGCAATCCTCTGAATCCATTTGTC

AGTCATAAAGAGATTGAGTCCGTAAACAATGCTGTAGTAATGCCCGCCCATGGCCCGGCC

AAGAGCATGGAATATGATGCTGTTGCGACCACACACTCGTGGATTCCTAAGAGGAACCGT

TCCATTCTCAATACCAGCCAAAGGGGAATTCTTGAGGATGAGCAGATGTACCAAAAGTGC

TGTAGCCTATTCGAGAAATTCTTCCCCAGCAGTTCATACAGGAGGCCAGTTGGAATTTCC

AGCATGGTGGAGGCCATGGTGTCTAGGGCCCGAATTGATGCACGCATCGACTTCGAATCT

GGAAGGATTAAGAAAGAAGAGTTTGCTGAGATCATGAAGATCTGTTCCACCATTGAAGAG

CTCAGACGGCAAAAATAGTGAATTTAGCTTGTCCTTCATGAAA-----------------

-

>A_wigeon_Latvia_23903_2021_EPI1855973

--------------AACTATTTGAATGGATGTCAATCCGACTTTACTTTTCTTAAAAGTG

CCAGCGCAAAATGCTATAAGTACTACATTCCCTTACACTGGAGATCCCCCATACAGCCAT

GGAACAGGAACAGGGTATACCATGGACACAGTAAACAGAACACATCAATACTCAGAAAAG

GGAAAGTGGACAACAAACACAGAAACCGGAGCACCCCAACTCAACCCAATTGATGGACCA

TTACCAGAGGACAATGAGCCAAGCGGATATGCACAAACTGATTGCGTGTTGGAAGCAATG

GCTTTCCTTGAAGAATCCCACCCAGGGATATTTGAAAACTCTTGTCTTGAAGCGATGGAA

ATCGTTCAGCAAACAAGAGTGGACAAACTAACCCAAGGTCGCCAGACTTATGACTGGACA

CTGAACAGAAACCAACCAGCTGCAACCTCTTTGGCCAACACTATAGAGGTGTTCAGATCG

AATGGTCTGACAGCCAATGAATCAGGGAGACTGATAGATTTTCTCAGGGATGTGATGGAA

TCAATGGATAAAGAAGAGATGGAAGTAACAACACATTTCCAGAGAAAAAGAAAAGTGAGG

GACAACATGACTAAGAAGATGGTCACACAAAGAACAATAGGGAAGAAGAAGCAGAGGCTG

AACAAGAGGAGTTACTTAATAAGAGCACTGACATTGAATACAATGACCAAAGATGCAGAA

AGAGGCAAGTTGAAGAGACGGGCAATTGCAACACCCGGGATGCAGATTAGAGGATTCGTG

TACTTCGTTGAAACACTAGCGAGGAGCATCTGTGAGAAACTAGAGCAATCTGGGCTCCCT

GTTGGAGGGAATGAGAAGAAGGCTAAATTGGCAAATGTTGTGAGAAAAATGATGACTAAC

TCACAAGATACAGAGCTCTCCTTTACAATTACTGGAGACAACACCAAATGGAATGAGAAT

CAAAACCCTCGGATGTTTTTGGCAATGATAACATATATCACAAGAAACCAACCTGAATGG

TTTAGAAATGTCTTAAGCATTGCCCCTATAATGTTCTCAAACAAAATGGCGAGATTAGGG

AAAGGATACATGTTTGAAAGTAAGAGCATGAAGCTAAGAACACAAATACCAGCAGAGATG

CTTACAAATATTGATCTGAAGTATTTCAACGAACCAACGAGAAAGAAAATCGAGAAAATA

AGACCTCTGCTGATTGATGGCACGGCCTCATTGAGTCCTGGGATGATGATGGGCATGTTC

AATATGCTGAGCACAGTATTAGGGGTCTCAATCCTGAATCTCGGGCAAAAGAGGTACACC

AAAACCACATACTGGTGGGATGGACTTCAATCCTCTGATGATTTCGCTCTCATAGTGAAT

GCACCGAATCATGAGGGGATACAAGCAGGAGTGGATAGATTCTATAGGACCTGCAAACTG

GTTGGGATCAACATGAGCAAAAAGAAGTCTTACATAAACCGAACAGGAACATTTGAGTTC

ACAAGTTTTTTCTACCGCTATGGATTTGTAGCCAACTTCAGTATGGAATTACCCAGCTTT

GGAGTGTCTGGAATCAATGAATCAGCTGACATGAGCATTGGAGTTACAGTGATAAAAAAC

AATATGATAAACAATGATCTTGGACCAGCAACAGCTCAAATGGCTCTTCAGTTATTCATC

AAAGACTATAGGTACACATACCGATGCCACAGGGGTGATACACAAATTCAAACGAGGAGA

TCATTCGAGCTGAAGAAGCTGTGGGAGCAGACCCGTTCAAAGGCAGGGCTGTTGATATCA

GACGGGGGGCCAAATCTATACAACATTCGGAATCTCCACATCCCAGAGGTCTGCTTGAAG

TGGGAGCTGATGGATGAAGACTACCAAGGCAGGCTGTGCAATCCTCTGAATCCATTTGTC

AGTCATAAAGAGATTGAGTCCGTAAACAATGCTGTAGTAATGCCCGCCCATGGCCCGGCC

AAGAGCATGGAATATGATGCTGTTGCGACCACACACTCGTGGATTCCTAAGAGGAACCGT

TCCATTCTCAATACCAGCCAAAGGGGAATTCTTGAGGATGAGCAGATGTATCAAAAGTGC

TGTAGTCTATTCGAGAAATTCTTCCCCAGCAGTTCATACAGGAGGCCAGTTGGAATTTCC

AGCATGGTGGAGGCCATGGTGTCTAGGGCCCGAATTGATGCACGCATCGACTTCGAATCT

GGAAGGATTAAGAAAGAAGAGTTTGCTGAGATCATGAAGATCTGTTCCACCATTGAAGAG

CTCAGACGGCAAAAATAGTGAATTTAGCTTGTCCTTCATGAAA-----------------

-

>A_chicken_Czech_Republic_4980_2021_EPI1858498

------------------------ATGGATGTCAATCCGACTTTACTTTTCTTAAAAGTG

CCAGCGCAAAATGCTATAAGTACTACATTCCCTTACACTGGAGATCCTCCATACAGCCAT

GGAACAGGAACAGGGTATACCATGGACACAGTAAACAGAACACATCAATACTCAGAAAAG

GGAAAGTGGACAACAAACACAGAAACCGGAGCACCCCAACTCAACCCAATTGATGGACCA

TTACCAGAGGACAATGAGCCAAGCGGATATGCACAAACTGATTGCGTGTTGGAAGCAATG

GCTTTCCTTGAAGAATCCCACCCAGGGATATTTGAAAACTCTTGTCTTGAAGCGATGGAA

ATCGTTCAGCAAACAAGAGTGGACAAACTAACCCAAGGTCGCCAGACTTATGACTGGACA

CTGAACAGAAACCAACCAGCTGCAACCTCTTTGGCCAACACTATAGAGGTGTTCAGATCG

AATGGTCTGACAGCCAATGAATCAGGGAGACTGATAGATTTTCTCAGGGATGTGATGGAA

TCAATGGATAAAGAAGAGATGGAAGTAACAACACATTTCCAGAGAAAAAGAAGAGTGAGG

GACAACATGACTAAGAAGATGGTCACACAAAGAACAATAGGGAAGAAGAAGCAGAGGCTG

AACAAGAGGAGTTACTTAATAAGAGCACTGACATTGAATACAATGACCAAAGATGCAGAA

AGAGGCAAGTTGAAGAGACGGGCAATTGCAACACCCGGGATGCAGATTAGAGGATTCGTG

TACTTCGTTGAAACACTAGCGAGGAGCATCTGTGAGAAACTAGAGCAATCTGGGCTCCCT

GTTGGAGGGAATGAGAAGAAGGCTAAATTGGCAAATGTTGTGAGAAAAATGATGACTAAC

TCACAAGATACAGAGCTCTCCTTTACAATTACTGGAGACAACACCAAATGGAATGAGAAT

CAAAACCCTCGGATGTTTTTGGCAATGATAACATATATCACAAGAAACCAACCTGAATGG

TTTAGAAATGTCTTAAGCATTGCCCCTATAATGTTCTCAAACAAAATGGCGAGATTAGGG

AAAGGATACATGTTTGAAAGTAAGAGCATGAAGCTAAGAACACAAATACCAGCAGAGATG

CTTACAAATATCGATCTGAAGTATTTCAACGAACCAACGAGAAAGAAAATCGAGAAAATA

AGACCTCTGCTGATTGATGGCACGGCCTCATTGAGTCCTGGGATGATGATGGGCATGTTC

AATATGCTGAGCACAGTATTAGGGGTCTCAATCCTGAATCTCGGGCAAAAAAGGTACACC

AAAACCACATACTGGTGGGATGGACTTCAATCCTCTGATGATTTCGCTCTCATAGTGAAT

GCACCGAATCATGAGGGGATACAAGCAGGAGTGGATAGATTCTATAGGACCTGCAAACTG

GTTGGGATCAACATGAGCAAAAGGAAGTCTTACATAAACCGAACAGGAACATTTGAGTTC

ACAAGTTTTTTCTACCGCTATGGATTTGTAGCTAACTTCAGTATGGAATTACCCAGCTTT

GGAGTGTCTGGAATCAATGAATCAGCTGACATGAGCATTGGAGTTACAGTGATAAAAAAC

AATATGATAAACAATGATCTTGGACCAGCAACAGCTCAAATGGCTCTTCAGTTATTCATC

AAAGACTACAGGTACACATACCGATGCCACAGGGGTGATACACAAATTCAAACGAGGAGA

TCATTCGAGCTGAAGAAGCTGTGGGAGCAGACCCGCTCAAAGGCAGGGCTGTTGATATCA

GACGGGGGGCCAAACCTATACAACATTCGGAATCTCCACATCCCAGAGGTCTGCTTGAAG

TGGGAGTTGATGGATGAAGACTACCAAGGCAGGCTGTGCAATCCTCTGAATCCATTTGTC

AGTCATAAAGAGATTGAGTCCGTAAACAATGCTGTAGTAATGCCCGCCCATGGCCCGGCC

AAGAGCATGGAATATGATGCTGTTGCGACCACACACTCGTGGATTCCTAAGAGGAACCGT

TCCATTCTCAATACCAGCCAAAGGGGAATTCTTGAAGATGAGCAGATGTACCAAAAGTGC

TGTAGTCTATTCGAGAAATTCTTCCCCAGCAGTTCATACAGGAGGCCAGTTGGAATTTCC

AGCATGGTGGAGGCCATGGTGTCTAGGGCCCGAATTGATGCACGCATCGACTTCGAATCT

GGAAGGATTAAGAAAGAAGAGTTTGCTGAGATCATGAAGATCTGTTCCACCATTGAAGAG

CTCAGACGGCAAAAATAGTGAATTTAGCTTGTCCTTCATGAAA-----------------

-

>A_swan_Lithuania_1258PG1_21VIR2606-2_2021_EPI1858572

AGCRAAAGCAGGCAAACTATTTGAATGGATGTCAATCCGACTTTACTTTTCTTAAAAGTG

CCAGCGCAAAATGCTATAAGTACTACATTCCCTTACACTGGAGATCCTCCATACAGCCAT

GGAACAGGAACAGGGTATACCATGGACACAGTAAACAGAACACATCAATACTCAGAAAAG

GGAAAGTGGACAACAAACACAGAAACCGGAGCACCCCAACTCAACCCAATTGATGGACCA

TTACCAGAGGACAATGAGCCAAGCGGATATGCACAAACTGATTGCGTGTTGGAAGCAATG

GCTTTCCTTGAAGAATCCCACCCAGGGATATTTGAAAACTCTTGTCTTGAAGCGATGGAA

ATCGTTCAGCAAACAAGAGTGGACAAACTAACCCAAGGTCGCCAGACTTATGACTGGACA

CTGAACAGAAACCAACCAGCTGCAACCTCTTTGGCCAACACTATAGAGGTGTTCAGATCG

AATGGTCTGACAGCCAACGAATCAGGGAGACTGATAGATTTTCTCAGGGATGTGATGGAA

TCAATGGATAAAGAAGAGATGGAAGTAACAACACATTTCCAGAGAAAAAGAAGAGTGAGG

GACAACATGACTAAGAAGATGGTCACACAAAGAACAATAGGGAAGAAGAAGCAGAGGCTG

AACAAGAGGAGTTACTTAATAAGAGCACTGACATTGAATACAATGACCAAAGATGCAGAA

AGAGGCAAGTTGAAGAGACGGGCAATTGCAACACCCGGGATGCAGATTAGAGGATTCGTG

TACTTCGTTGAAACACTAGCGAGGAGCATCTGTGAGAAACTAGAGCAATCTGGGCTCCCT

GTTGGAGGGAATGAGAAGAAGGCTAAATTGGCAAATGTTGTGAGAAAAATGATGACTAAC

TCACAAGATACAGAGCTCTCCTTTACAATTACTGGAGACAACACCAAATGGAATGAGAAT

CAAAACCCTCGGATGTTTTTGGCAATGATAACATATATCACAAGAAACCAACCTGAATGG

TTTAGAAATGTCTTAAGCATTGCCCCTATAATGTTCTCAAACAAAATGGCGAGATTAGGG

AAAGGATACATGTTTGAAAGTAAGAGCATGAAGCTAAGAACACAAATACCAGCAGAGATG

CTTACAAATATCGATCTGAAGTATTTCAACGAACCAACGAGAAAGAAAATCGAGAAAATA

AGACCTCTGCTGATTGATGGCACGGCCTCATTGAGTCCTGGGATGATGATGGGCATGTTC

AATATGCTGAGCACAGTATTAGGGGTCTCAATCCTGAATCTCGGGCAAAAAAGGTACACC

AAAACCACATACTGGTGGGATGGACTTCAATCCTCTGATGATTTCGCTCTCATAGTGAAT

GCACCGAATCATGAGGGGATACAAGCAGGAGTGGATAGATTCTATAGGACCTGCAAACTG

GTTGGGATCAACATGAGCAAAAAGAAGTCTTACATAAACCGAACAGGAACATTTGAGTTC

ACAAGTTTTTTCTACCGCTATGGATTTGTAGCTAACTTCAGTATGGAATTACCCAGCTTT

GGAGTGTCTGGAATCAATGAATCAGCTGACATGAGCATTGGAGTTACAGTGATAAAAAAC

AATATGATAAACAATGATCTTGGACCAGCAACAGCTCAAATGGCTCTTCAGTTATTCATC

AAAGACTATAGGTACACATACCGATGCCACAGGGGTGATACACAAATTCAAACGAGGAGA

TCATTCGAGCTGAAGAAGCTGTGGGAGCAGACCCGTTCAAAGGCAGGGCTGTTGATATCA

GACGGGGGGCCAAACCTATACAACATTCGGAATCTCCACATCCCAGAGGTCTGCTTGAAG

TGGGAGTTGATGGATGAAGACTACCAAGGCAGGCTGTGCAATCCTCTGAATCCATTTGTC

AGTCATAAAGAGATTGAGTCCGTAAACAATGCTGTAGTAATGCCCGCCCATGGCCCGGCC

AAGAGCATGGAATATGATGCTGTTGCGACCACACACTCGTGGATTCCTAAGAGGAACCGT

TCCATTCTCAATACCAGCCAAAGGGGAATTCTTGAGGATGAGCAGATGTACCAAAAGTGC

TGTAGTCTATTCGAGAAATTCTTCCCCAGCAGTTCATACAGGAGGCCAGTTGGAATTTCC

AGCATGGTGGAGGCCATGGTGTCTAGGGCCCGAATTGATGCACGCATCGACTTCGAATCT

GGAAGGATTAAGAAAGAAGAGTTTGCTGAGATCATGAAGATCTGTTCCACCATTGAAGAG

CTCAGACGGCAAAAATAGTGAATTTAGCTTGTCCTTCATGAAAAAATGCCTTGTTTCTAC

T

>A_swan_Lithuania_1298PG1_21VIR2606-3_2021_EPI1858580

AGCRAAAGCAGGCAAACTATTTGAATGGATGTCAATCCGACTTTACTTTTCTTAAAAGTG

CCAGCGCAAAATGCTATAAGTACTACATTCCCTTACACTGGAGATCCCCCATACAGCCAT

GGAACAGGAACAGGGTATACCATGGACACAGTAAACAGAACACATCAATACTCAGAAAAG

GGAAAGTGGACAACAAACACAGAAACCGGAGCACCCCAACTCAACCCAATTGATGGACCA

TTACCAGAGGACAATGAGCCAAGCGGATATGCACAAACTGATTGCGTGTTGGAAGCAATG

GCTTTTCTTGAAGAATCCCACCCAGGGATATTTGAAAACTCTTGTCTTGAAGCGATGGAA

ATCGTTCAGCAAACAAGAGTGGACAAACTAACCCAAGGTCGCCAGACTTATGACTGGACA

CTGAACAGAAACCAACCAGCTGCAACCTCTTTGGCCAACACTATAGAGGTGTTCAGATCG

AATGGTCTGACAGCCAATGAATCAGGGAGACTGATAGATTTTCTCAGGGATGTGATGGAA

TCAATGGATAAAGAAGAGATGGAAGTAACAACACATTTCCAGAGAAAAAGAAGAGTGAGG

GACAACATGACTAAGAAGATGGTCACACAAAGAACAATAGGGAAGAAGAAGCAGAGGCTG

AACAAGAGGAGTTACTTAATAAGAGCACTGACATTGAATACAATGACCAAAGATGCAGAA

AGAGGCAAGTTGAAGAGACGGGCAATTGCAACACCCGGGATGCAGATTAGAGGATTCGTG

TACTTCGTTGAAACACTAGCGAGGAGCATCTGTGAGAAACTAGAGCAATCTGGGCTCCCT

GTTGGAGGGAATGAGAAGAAGGCTAAATTGGCAAATGTTGTGAGAAAAATGATGACTAAC

TCACAAGATACAGAGCTCTCCTTTACAATTACTGGAGACAACACCAAATGGAATGAGAAT

CAAAACCCTCGGATGTTTTTGGCAATGATAACATATATCACAAGAAACCAACCTGAATGG

TTTAGAAATGTCTTAAGCATTGCCCCTATAATGTTCTCAAACAAAATGGCAAGATTAGGG

AAAGGATACATGTTTGAAAGTAAGAGCATGAAGCTAAGAACACAAATACCAGCAGAGATG

CTTACAAATATTGATCTGAAGTATTTCAACGAACCAACGAGAAAGAAAATCGAGAAAATA

AGACCTCTGCTGATTGATGGCACGGCCTCATTGAGTCCTGGGATGATGATGGGCATGTTC

AATATGCTGAGCACAGTATTAGGGGTCTCAATCCTGAATCTCGGGCAAAAGAGGTACACC

AAAACCACATACTGGTGGGATGGACTTCAATCCTCTGATGATTTCGCTCTCATAGTGAAT

GCACCGAATCATGAGGGGATACAAGCAGGAGTGGATAGATTCTATAGGACCTGCAAACTG

GTTGGGATCAACATGAGCAAAAAGAAGTCTTACATAAACCGAACAGGAACATTTGAGTTC

ACAAGTTTTTTCTACCGCTATGGATTTGTAGCCAACTTCAGTATGGAATTACCCAGCTTT

GGAGTGTCTGGAATCAATGAATCAGCTGACATGAGCATTGGAGTTACAGTGATAAAAAAC

AATATGATAAACAATGATCTTGGACCAGCAACAGCTCAAATGGCTCTTCAGTTATTCATC

AAAGACTATAGGTACACATACCGATGCCACAGGGGTGATACACAAATTCAAACGAGGAGA

TCATTCGAGCTGAAGAAGCTGTGGGAGCAGACCCGTTCAAAGGCAGGGCTGTTGATATCA

GACGGGGGGCCAAATCTATACAACATTCGGAATCTCCACATCCCAGAGGTCTGCTTGAAA

TGGGAGCTGATGGATGAAGACTACCAAGGCAGGCTGTGCAATCCTCTGAATCCATTTGTC

AGTCATAAAGAGATTGAGTCCGTAAACAATGCTGTAGTAATGCCCGCCCATGGCCCGGCC

AAGAGCATGGAATATGATGCTGTTGCGACCACACACTCGTGGATTCCTAAGAGGAACCGT

TCCATTCTCAATACCAGCCAAAGGGGAATTCTTGAGGATGAGCAGATGTACCAAAAGTGC

TGTAGTCTATTCGAGAAATTCTTCCCCAGCAGTTCATACAGGAGGCCAGTTGGAATTTCC

AGCATGGTGGAGGCCATGGTGTCTAGGGCCCGAATTGATGCACGCATCGACTTCGAATCT

GGAAGGATTAAGAAAGAAGAGTTTGCTGAGATCATGAAGATCTGTTCCACCATTGAAGAG

CTCAGACGGCAAAAATAGTGAATTTAGCTTGTCCTTCATGAAAAAATGCCTTGTTTCTAC

N

>A_chicken_Bulgaria_50-1_21VIR1454-9_2021_EPI1858620

AGCRAAAGCAGGCAAACTATTTGAATGGATGTCAATCCGACTTTACTTTTCTTAAAAGTG

CCAGCGCAAAATGCTATAAGTACTACATTCCCTTACACTGGAGATCCTCCATACAGCCAT

GGAACAGGAACAGGGTATACCATGGACACAGTAAACAGAACACATCAATACTCAGAAAAG

GGAAAGTGGACAACAAACACAGAAACCGGAGCACCCCAACTCAACCCAATTGATGGACCA

TTACCAGAGGACAATGAGCCAAGCGGATATGCACAAACTGATTGCGTGTTGGAAGCAATG

GCTTTCCTTGAAGAATCCCACCCAGGGATATTTGAAAACTCTTGTCTTGAAGCGATGGAA

ATCGTTCAGCAAACAAGAGTGGACAAACTAACCCAAGGYCGCCAGACTTATGACTGGACA

CTGAACAGAAACCAACCAGCTGCAACCTCTTTGGCCAACACTATAGAGGTGTTCAGATCG

AATGGTCTGACAGCCAATGAATCAGGGAGACTGATAGATTTTCTCAGGGATGTGATGGAA

TCAATGGATAAAGAAGAGATGGAAGTAACAACACATTTCCAGAGGAAAAGAAGAGTGAGG

GACAACATGACTAAGAAGATGGTCACACAAAGAACAATAGGGAAGAAGAAGCAGAGGCTG

AACAAGAGGAGTTACTTAATAAGAGCACTGACATTGAATACAATGACCAAAGATGCAGAA

AGAGGCAAGTTGAAGAGACGGGCAATTGCAACACCCGGGATGCAGATTAGAGGATTCGTG

TACTTCGCTGAAACACTAGCGAGGAGCATCTGTGAGAAACTAGAGCAATCTGGGCTCCCT

GTTGGAGGGAATGAGAAGAAGGCTAAATTGGCAAATGTTGTGAGAAAAATGATGACTAAC

TCACAAGATACAGAGCTCTCCTTTACAATTACTGGAGACAACACCAAATGGAATGAGAAT

CAAAACCCTCGGATGTTTTTGGCAATGATAACATATATCACAAGAAACCAACCTGAATGG

TTTAGAAATGTCTTAAGCATTGCCCCTATAATGTTCTCAAACAAAATGGCGAGATTAGGG

AAAGGATACATGTTTGAAAGTAAGAGCATGAAGCTGAGAACACAAATACCAGCAGAGATG

CTTACAAATATCGATCTGAAGTATTTCAACGAACCAACGAGAAAGAAAATCGAGAAAATA

AGACCTCTGCTGATTGATGGCACGGCCTCATTGAGTCCTGGGATGATGATGGGCATGTTC

AATATGCTGAGCACAGTATTAGGGGTCTCAATCCTGAATCTCGGGCAAAAAAGGTACACC

AAAACCACATACTGGTGGGATGGACTTCAATCCTCTGATGATTTCGCTCTCATAGTGAAT

GCACCGAATCATGAGGGGATACAAGCAGGAGTGGATAGATTCTATAGGACCTGCAAACTG

GTTGGGATCAACATGAGCAAAAAGAAGTCTTACATAAACCGAACAGGAACATTTGAGTTC

ACAAGTTTTTTCTACCGCTATGGATTTGTAGCTAACTTCAGTATGGAATTACCCAGCTTT

GGAGTGTCTGGAATCAATGAATCAGCTGACATGAGCATTGGAGTTACAGTAATAAAAAAC

AATATGATAAACAATGATCTTGGACCAGCAACAGCTCAAATGGCTCTTCAGTTATTCATC

AAAGATTATAGGTACACATACCGATGCCACAGGGGTGATACACAAATTCAAACGAGGAGA

TCATTCGAGCTGAAGAGGCTGTGGGAGCAGACCCGTTCAAAGGCAGGGCTGTTGATATCA

GACGGGGGGCCAAACCTATACAACATTCGGAATCTCCACATCCCAGAGGTCTGCTTGAAG

TGGGAGCTAATGGATGAAGACTACCAAGGCAGGCTGTGCAATCCTCTGAATCCATTTGTC

AGTCATAAAGAGATTGAGTCCGTAAACAATGCTGTAGTAATGCCCGCCCATGGCCCGGCC

AAGAGCATGGAATATGATGCTGTTGCGACCACACACTCGTGGATTCCTAAGAGGAACCGT

TCCATTCTCAATACCAGCCAAAGGGGAATTCTTGAGGATGAGCAGATGTACCAAAAGTGC

TGTAGTCTATTCGAGAAATTCTTCCCCAGCAGTTCATACAGGAGGCCAGTTGGAATTTCC

AGCATGGTGGAGGCCATGGTGTCTAGGGCCCGAATTGATGCACGCATCGACTTCGAATCT

GGAAGGATTAAGAAAGAAGAGTTTGCTGAGATCATGAAGATCTGTTCCACCATTGAAGAG

CTCAGACGGCAAAAATAGTGAATTTAGCTTGTCCTTCATGAAAAAATGCCTTGTTTCTAC

T

>A_mute_swan_Poland_MB189_2021_EPI1859653

------------CAAACTATTTGAATGGATGTCAATCCGACTTTACTTTTCTTAAAAGTG

CCAGCGCAAAATGCTATAAGTACTACATTCCCTTACACTGGAGATCCCCCATACAGCCAT

GGAACAGGAACAGGGTATACCATGGATACAGTAAACAGAACACATCAATACTCAGAAAAG

GGAAAGTGGACAACAAACACAGAAACCGGAGCACCCCAACTCAACCCAATTGATGGACCA

TTACCAGAGGACAATGAGCCAAGCGGATATGCACAAACTGATTGCGTGTTGGAAGCAATG

GCTTTCCTTGAAGAATCCCACCCAGGGATATTTGAAAACTCTTGTCTTGAAGCGATGGAA

ATCGTTCAGCAAACAAGAGTGGACAAACTAACCCAAGGTCGCCAGACTTATGACTGGACA

CTGAACAGAAACCAACCAGCTGCAACCTCTTTGGCCAACACTATAGAGGTGTTCAGATCG

AATGGTCTGACAGCCAATGAATCAGGGAGACTGATAGATTTTCTCAGGGATGTGATGGAA

TCAATGGATAAAGAAGAGATGGAAGTAACAACACATTTCCAGAGAAAAAGAAGAGTGAGG

GACAACATGACTAAGAAGATGGTCACACAAAGAACAATAGGGAAGAAGAAGCAGAGGCTG

AACAAGAGGAGTTACTTAATAAGAGCACTGACATTGAATACAATGACCAAAGATGCAGAA

AGAGGCAAGTTGAAGAGACGGGCAATTGCAACACCCGGGATGCAGATTAGAGGATTCGTG

TACTTCGTTGAAACACTAGCGAGGAGCATCTGTGAGAAACTAGAGCAATCTGGGCTCCCT

GTTGGAGGGAATGAGAAAAAGGCTAAATTGGCAAATGTTGTGAGAAAAATGATGACTAAC

TCACAAGATACAGAGCTCTCCTTTACAATTACTGGAGACAACACCAAATGGAATGAGAAT

CAAAACCCTCGGATGTTTTTGGCAATGATAACATATATCACAAGAAACCAACCTGAATGG

TTTAGAAATGTCTTAAGCATTGCCCCTATAATGTTCTCAAACAAAATGGCGAGATTAGGG

AAAGGATACATGTTTGAAAGTAAGAGCATGAAGCTAAGAACACAAATACCAGCAGAGATG

CTTACAAATATTGATCTGAAGTATTTCAACGAACCAACGAGAAAGAAAATCGAGAAAATA

AGACCTCTGCTGATTGATGGCACGGCCTCATTGAGTCCTGGGATGATGATGGGCATGTTC

AATATGCTGAGCACAGTATTAGGGGTCTCAATCCTGAATCTCGGGCAAAAGAGGTACACC

AAAACCACATACTGGTGGGATGGACTTCAATCCTCTGATGATTTCGCTCTCATAGTGAAT

GCACCGAATCATGAGGGGATACAAGCAGGAGTGGATAGATTCTATAGGACCTGCAAACTG

GTTGGGATCAACATGAGCAAAAAGAAGTCTTACATAAACCGAACAGGAACATTTGAGTTC

ACAAGTTTTTTCTACCGCTATGGATTTGTAGCCAACTTCAGTATGGAATTACCCAGCTTT

GGAGTGTCTGGAATCAATGAATCAGCCGACATGAGCATTGGAGTTACAGTGATAAAAAAC

AATATGATAAACAATGATCTTGGACCAGCAACAGCTCAAATGGCCCTTCAGTTATTCATC

AAGGACTATAGGTACACATACCGATGCCACAGGGGTGATACACAAATTCAAACGAGGAGA

TCATTCGAGCTGAAGAAGCTGTGGGAGCAGACCCGTTCAAAGGCAGGGCTGTTGATATCA

GACGGGGGGCCAAACCTATACAACATTCGGAATCTCCACATCCCAGAGGTCTGCTTGAAG

TGGGAGCTGATGGATGAAGACTACCAAGGCAGGCTGTGCAATCCTCTGAATCCATTTGTC

AGTCATAAAGAGATTGAGTCCGTAAACAATGCTGTAGTAATGCCCGCCCATGGCCCGGCC

AAGAGCATGGAATATGATGCTGTTGCGACCACACACTCGTGGATTCCTAAGAGGAACCGT

TCCATCCTCAATACCAGCCAAAGGGGAATTCTTGAGGATGAGCAGATGTACCAAAAGTGC

TGTAGTCTATTCGAGAAATTCTTCCCCAGCAGTTCATACAGGAGGCCAGTTGGAATTTCC

AGCATGGTGGAGGCCATGGTGTCTAGGGCCAGAATTGATGCACGCATCGACTTCGAATCT

GGAAGGATTAAGAAAGAAGAGTTTGCTGAGATCATGAAGATCTGTTCCACCATTGAAGAG

CTCAGACGGCAAAAATAGTGAATTTAGCTTGTCCTTCATGAAAAAATGC-----------

-

>A_mute_swan_Poland_MB272_2021_EPI1859669

------------CAAACTATTTGAATGGATGTCAATCCGACTTTACTTTTCTTAAAAGTG

CCAGCGCAAAATGCTATAAGTACTACATTCCCTTACACTGGAGATCCTCCATACAGCCAT

GGAACAGGAACAGGGTATACCATGGACACAGTAAACAGAACACATCAATACTCAGAAAAG

GGAAAGTGGACAACAAACACAGAAACCGGAGCACCCCAACTCAACCCAATTGATGGACCA

TTACCAGAGGACAATGAGCCAAGCGGATATGCACAAACTGATTGCGTGTTGGAAGCAATG

GCTTTCCTTGAAGAATCCCACCCAGGGATATTTGAAAACTCTTGTCTTGAAGCGATGGAA

ATCGTTCAGCAAACAAGAGTGGACAAACTAACCCAAGGTCGCCAGACTTATGACTGGACA

CTGAACAGAAACCAACCAGCTGCAACCTCTTTGGCCAACACTATAGAGGTGTTCAGATCG

AATGGTCTGACAGCCAATGAATCAGGGAGACTGATAGATTTTCTCAGGGATGTGATGGAA

TCAATGGATAAAGAAGAGATGGAAGTAACAACACATTTCCAGAGAAAAAGAAGAGTGAGA

GACAACATGACTAAGAAGATGGTCACACAAAGAACAATAGGGAAGAAGAAGCAGAGGCTG

ATCAAGAGGAGTTACTTAATAAGAGCACTGACATTGAATACAATGACCAAAGATGCAGAA

AGAGGCAAGTTGAAGAGACGGGCAATTGCAACACCCGGGATGCAGATTAGAGGATTCGTG

TACTTCGTTGAAACACTAGCGAGGAGCATCTGTGAGAAACTAGAGCAATCTGGGCTCCCT

GTTGGAGGGAATGAGAAGAAGGCTAAATTGGCAAATGTTGTGAGAAAAATGATGACTAAC

TCACAAGATACAGAGCTCTCCTTTACAATTACTGGAGACAACACCAAATGGAATGAGAAT

CAAAACCCTCGGATGTTTTTGGCAATGATAACATATATCACAAGAAACCAACCTGAATGG

TTTAGAAATGTCTTAAGCATTGCCCCTATAATGTTCTCAAACAAAATGGCGAGATTAGGG

AAAGGATACATGTTTGAAAGTAAGAGCATGAAGCTAAGAACACAAATACCAGCAGAGATG

CTTACAAATATCGATCTGAAGTATTTCAACGAACCAACGAGAAAGAAAATCGAGAAAATA

AGACCTCTGCTGATTGATGGCACGGCCTCATTGAGTCCTGGAATGATGATGGGCATGTTC

AATATGCTGAGCACAGTATTAGGGGTCTCAATCCTGAATCTCGGGCAAAAAAGGTACACC

AAAACCACATACTGGTGGGATGGACTTCAATCCTCTGATGATTTCGCTCTCATAGTGAAT

GCACCGAATCATGAGGGGATACAAGCAGGAGTGGATAGATTCTATAGGACCTGCAAACTG

GTTGGGATCAACATGAGCAAAAGGAAGTCTTACATAAACCGAACAGGAACATTTGAGTTC

ACAAGTTTTTTCTACCGCTATGGATTTGTAGCTAACTTCAGTATGGAATTACCCAGCTTT

GGAGTGTCTGGAATCAATGAATCAGCTGACATGAGCATTGGAGTTACAGTGATAAAAAAC

AATATGATAAACAATGATCTTGGACCAGCAACAGCTCAAATGGCTCTTCAGTTATTCATC

AAAGACTATAGGTACACATACCGATGCCACAGGGGTGATACACAAATTCAAACGAGGAGA

TCATTCGAGCTGAAGAAGCTGTGGGAGCAGACCCGTTCAAAGGCAGGGCTGTTGATATCA

GACGGGGGGCCAAACCTATACAACATTCGGAATCTCCACATCCCAGAGGTCTGCTTGAAG

TGGGAGCTGATGGATGAAGACTACCAAGGCAGGCTGTGCAATCCTCTGAATCCATTTGTC

AGTCATAAAGAGATTGAGTCCGTAAACAATGCTGTAGTAATGCCCGCCCATGGCCCGGCC

AAGAGCATGGAATATGATGCTGTTGCGACCACACACTCGTGGATTCCTAAGAGGAACCGT

TCCATTCTCAATACCAGCCAAAGGGGAATTCTTGAGGATGAGCAGATGTACCAAAAGTGC

TGTAATCTATTCGAGAAATTCTTCCCCAGCAGTTCATACAGGAGGCCAGTTGGAATTTCC

AGCATGGTGGAGGCCATGGTGTCTAGGGCCCGAATTGATGCACGCATCGACTTCGAATCT

GGAAGGATTAAGAAAGAAGAGTTTGCTGAGATCATGAAGATCTGTTCCACCATTGAAGAG

CTCAGACGGCAAAAATAGTGAATTTAGCTTGTCCTTCATGAAAAAATGC-----------

-

>A_anser_anser_Spain_297-1_21VIR1230-5_2021_EPI1860069

--------------AACTATTTGAATGGATGTCAATCCGACTTTACTTTTCTTAAAAGTG

CCAGCGCAAAATGCTATAAGTACTACATTCCCTTACACTGGAGATCCTCCATACAGCCAT

GGAACAGGAACAGGGTATACCATGGACACAGTAAACAGAACACATCAATACTCAGAAAAG

GGAAAGTGGACAACAAACACAGAAACCGGAGCACCCCAACTCAACCCAATTGATGGACCA

TTACCAGAGGACAATGAGCCAAGCGGATATGCACAAACTGATTGCGTGTTGGAAGCAATG

GCTTTCCTTGAAGAATCCCACCCAGGGATATTTGAAAACTCTTGTCTTGAAGCGATGGAA

ATCGTTCAGCAAACAAGAGTGGACAAACTAACCCAAGGTCGCCAGACTTATGACTGGACA

CTGAACAGAAACCAACCAGCTGCAACTTCTTTGGCCAACACTATAGAGGTGTTCAGATCG

AATGGTCTGACAGCCAATGAATCAGGGAGACTGATAGATTTTCTCAGAGATGTGATGGAA

TCAATGGATAAAGAAGAGATGGAAGTAACAACACATTTCCAGAGAAAAAGAAGAGTGAGG

GACAACATGACTAAGAAGATGGTCACACAAAGAACAATAGGGAAGAAGAAGCAGAGGCTG

AACAAGAGGAGTTACTTAATAAGAGCACTGACATTGAATACAATGACCAAAGATGCAGAA

AGAGGCAAGTTGAAGAGACGGGCAATTGCAACACCCGGGATGCAGATTAGAGGATTCGTG

TACTTCGTTGAAACACTAGCGAGGAGCATCTGTGAGAAACTAGAGCAATCTGGGCTCCCT

GTTGGAGGGAATGAGAAGAAGGCTAAATTGGCAAATGTTGTGAGAAAAATGATGACTAAC

TCACAAGATACAGAGCTCTCCTTTACAATTACTGGAGACAACACCAAATGGAATGAGAAT

CAAAACCCTCGGATGTTTTTGGCAATGATAACATATATCACAAGAAACCAACCTGAATGG

TTTAGAAATGTCTTAAGCATTGCCCCTATAATGTTCTCAAACAAAATGGCGAGATTAGGG

AAAGGATACATGTTTGAAAGTAAGAGCATGAAGCTAAGAACACAAATACCAGCAGAGATG

CTTACAAATATCGATCTGAAGTATTTCAACGAACCAACGAGAAAGAAAATCGAGAAAATA

AGACCTCTGCTGATTGATGGCACGGCCTCATTGAGTCCTGGGATGATGATGGGCATGTTC

AATATGCTGAGCACAGTATTAGGGGTCTCAATCCTGAATCTCGGGCAAAAAAGGTACACC

AAAACCACATACTGGTGGGATGGACTTCAATCCTCTGATGATTTCGCTCTCATAGTGAAT

GCACCGAATCATGAGGGGATACAAGCAGGAGTGGATAGATTCTATAGGACCTGCAAACTG

GTTGGGATCAACATGAGCAAAAAGAAGTCTTACATAAACCGAACAGGAACATTTGAGTTC

ACAAGTTTTTTCTACCGCTATGGATTTGTAGCTAACTTCAGTATGGAATTACCCAGCTTT

GGAGTGTCTGGAATCAATGAATCAGCTGACATGAGCATTGGAGTTACAGTGATAAAAAAC

AATATGATAAACAATGATCTTGGACCAGCAACAGCTCAAATGGCTCTTCAGTTATTCATC

AAAGACTATAGGTACACATACCGATGCCACAGGGGTGATACACAAATTCAAACGAGGAGA

TCATTCGAGCTGAAGAAGCTGTGGGAGCAGACCCGTTCAAAGGCAGGGCTGTTGATATCA

GACGGGGGGCCAAACCTATACAACATTCGGAATCTCCACATCCCAGAGGTCTGCTTGAAG

TGGGAGCTGATGGATGAAGACTACCAAGGCAGGCTGTGCAATCCTCTGAATCCATTTGTC

AGTCATAAAGAGATTGAGTCCGTAAACAATGCTGTAGTAATGCCCGCCCATGGCCCGGCC

AAGAGCATGGAATATGATGCTGTTGCGACCACACACTCGTGGATTCCTAAGAGGAACCGT

TCCATTCTCAATACCAGCCAAAGGGGAATTCTTGAGGATGAGCAGATGTACCAAAAGTGC

TGTAGTCTATTCGAGAAATTCTTCCCCAGCAGTTCATACAGGAGGCCAGTTGGAATTTCC

AGCATGGTGGAGGCCATGGTGTCTAGGGCCCGAATTGATGCACGCATCGACTTCGAATCT

GGAAGGATTAAGAAAGAAGAGTTTGCTGAGATCATGAAGATCTGTTCCACCATTGAAGAG

CTCAGACGGCAAAAATAGTGAATTTAGCTTGTCCTTCATGAAA-----------------

-

>A_ciconia_ciconia_Spain_102-1_21VIR1230-2_2021_EPI1860077

--------------NNCTATTTGAATGGATGTCAATCCGACTTTACTTTTCTTAAAAGTG

CCAGCGCAAAATGCTATAAGTACTACATTCCCTTACACTGGAGATCCTCCATACAGCCAT

GGAACAGGAACAGGGTATACCATGGACACAGTAAACAGAACACATCAATACTCAGGAAAG

GGAAAGTGGACAACAAACACAGAAACCGGAGCACCCCAACTCAACCCAATTGATGGACCA

TTACCAGAGGACAATGAGCCAAGCGGATATGCACAAACTGATTGCGTGTTGGAAGCAATG

GCTTTCCTTGAAGAATCCCACCCAGGGATATTTGAAAACTCTTGTCTTGAAGCGATGGAA

ATCGTTCAGCAAACAAGAGTGGACAAACTAACCCAAGGTCGCCAGACTTATGACTGGACA

CTGAACAGAAACCAACCAGCTGCAACCTCTTTGGCCAACACTATAGAGGTGTTCAGATCA

AATGGTCTGACAGCCAATGAATCAGGGAGACTGATAGATTTTCTCAGGGATGTGATGGAA

TCAATGGATAAAGAAGAGATGGAAGTAACAACACATTTCCAGAGAAAAAGAAGAGTGAGG

GACAACATGACTAAGAAGATGGTCACACAAAGAACAATAGGGAAGAAGAAGCAGAGGCTG

AACAAGAGGAGTTACTTAATAAGAGCACTGACATTGAATACAATGACCAAAGATGCAGAA

AGAGGCAAGTTGAAGAGACGGGCAATTGCAACACCCGGGATGCAGATTAGAGGATTCGTG

TACTTCGTTGAAACACTAGCGAGGAGCATCTGTGAGAAACTAGAGCAATCTGGGCTCCCT

GTTGGAGGGAATGAGAAGAAGGCTAAACTGGCAAATGTTGTGAGAAAAATGATGACTAAC

TCACAAGATACAGAGCTCTCCTTTACAATTACTGGAGACAACACCAAATGGAATGAGAAT

CAAAACCCTCGGATGTTTTTGGCAATGATAACATATATCACAAGAAACCAACCTGAATGG

TTTAGAAATGTCTTAAGCATTGCCCCTATAATGTTCTCAAACAAAATGGCGAGATTAGGG

AAAGGATACATGTTTGAAAGTAAGAGCATGAAGCTAAGAACACAAATACCAGCAGAGATG

CTTACAAATATCGATCTGAAGTATTTCAACGAACCAACGAGAAAGAAAATCGAGAAAATA

AGACCTCTGCTGATTGATGGCACGGCCTCATTGAGTCCTGGGATGATGATGGGCATGTTC

AATATGCTGAGCACAGTATTAGGGGTCTCAATCCTGAATCTCGGGCAAAAAAGGTACACC

AAAACCACATACTGGTGGGATGGACTTCAATCCTCTGATGATTTCGCTCTCATCGTGAAT

GCACCGAATCATGAGGGGATACAAGCAGGAGTGGATAGATTCTATAGGACCTGCAAACTG

GTTGGGATCAACATGAGCAAAAAGAAGTCTTACATAAACCGAACAGGAACATTTGAGTTC

ACAAGTTTTTTCTACCGCTATGGATTTGTAGCTAACTTCAGTATGGAATTACCCAGCTTT

GGAGTGTCTGGAATCAATGAATCAGCTGACATGAGCATTGGAGTTACAGTGATAAAAAAC

AATATGATAAACAATGATCTTGGACCAGCAACAGCTCAAATGGCTCTTCAGTTATTCATC

AAAGATTATAGGTACACATACCGATGCCACAGGGGTGATACACAAATTCAAACGAGGAGA

TCATTCGAGCTGAAGAAGCTGTGGGAGCAGACCCGTTCAAAGGCAGGGCTGTTGATATCA

GACGGGGGGCCAAACCTATACAACATTCGGAATCTCCACATCCCAGAGGTCTGCTTGAAG

TGGGAGCTGATGGATGAAGACTACCAAGGCAGGCTGTGCAATCCTCTGAATCCATTTGTC

AGTCATAAAGAGATTGAGTCCGTAAACAATGCTGTAGTAATGCCCGCCCATGGCCCGGCC

AAGAGCATGGAATATGACGCTGTTGCGACCACACACTCATGGATTCCTAAGAGGAACCGT

TCCATTCTCAATACCAGCCAAAGGGGAATTCTTGAGGATGAGCAGATGTACCAAAAGTGC

TGTAGTCTATTCGAGAAATTCTTCCCCAGCAGTTCATACAGGAGGCCAGTTGGAATTTCC

AGCATGGTGGAGGCCATGGTGTCTAGGGCCCGAATTGATGCACGCATCGACTTCGAATCT

GGAAGGATTAAGAAAGAAGAGTTTGCTGAGATCATGAAGATCTGTTCCACCATTGAAGAG

CTCAGACGGCAAAAATAGTGAATTTAGCTTGTCCTTCATGAAA-----------------

-

>A_duck_Jiangsu_k1203_2010_EPI442015

------------------------ATGGATGTCAATCCGACTTTTCTTTTCTTGAAAGTG

CCAGCGCAAAATGCCATAAGTACCACATTCCCTTATACTGGAGATCCTCCATACAGCCAT

GGAACAGGAACAGGATACACCATGGACACAGTCAACAGAACACATCAATACTCAGAAAAG

GGAAAGTGGACAACAAACACTGAAACTGGAGCACCCCAACTCAATCCAATTGATGGACCA

TTACCTGAGGATAACGAGCCAAGCGGATATGCACAAACGGATTGTGTATTGGAAGCAATG

GCTTTCCTTGAAGAGTCCCACCCAGGGATCTTTGAAAACTCATGTCTTGAAACGATGGAA

ATTGTTCAGCAAACAAGAGTGGACAAACTGACCCAAGGTCGCCAGACCTATGATTGGACA

TTGAATAGAAACCAGCCGGCAGCAACTGCTTTAGCCAACACTATAGAAGTCTTCAGATCA

AACGGTCTAACAGCCAATGAATCAGGGAGATTGATAGATTTCCTCAAGGATGTGGTGGAG

TCAATGGATAAAGAAGAAATGGAAATAACAACACATTTCCAAAGAAAGAGAAGAGTAAGA

GACAATATGACCAAGAAAATGGTCACACAAAGAACAATAGGGAAGAAAAAACAAAGGCTG

AACAAGAAGAGCTATTTGATAAGAGCACTGACACTGAACACAATGACAAAGGATGCAGAA

AGAGGCAAATTGAAAAGGCGGGCAATTGCAACACCCGGGATGCAGATCAGAGGTTTCGTG

TACTTTGTCGAAACATTAGCGAGGAGCATCTGTGAAAAACTTGAGCAATCTGGGCTCCCT

GTTGGAGGGAATGAGAAGAAGGCTAAATTGGCAAATGTCGTGAGAAAAATGATGACTAAC

TCACAAGACACAGAGCTCTCCTTTACAATTACTGGAGACAATACCAAATGGAATGAAAAT

CAGAACCCTCGGATGTTTTTGGCAATGATAACATACATCACAAGAAACCAACCTGAATGG

TTTAGAAATGTATTAAGTATCGCTCCTATAATGTTCTCAAACAAGATGGCAAGATTAGGG

AAAGGATACATGTTCGAGAGTAAGAGCATGAAACTACGAACACAAATACCAGCAGAAATG

CTCGCAGACATTGACCTGAAATACTTCAATGAATCAACAAGGAAGAAAATTGAGAAAATA

AGACCTCTCCTAATAGATGGGACTGCCTCATTGAGTCCTGGAATGATGATGGGCATGTTC

AACATGCTGAGCACAGTCTTAGGAGTCTCAATCCTAAATCTTGGGCAAAAGAGGTACACC

AAAACCACATACTGGTGGGACGGACTCCAATCTTCTGATGATTTCGCTCTCATAGTAAAT

GCACCAAACCATGAGGGGATACAGGCAGGAGTAGACAGGTTCTATAGAACCTGTAAACTG

GTTGGGATCAATATGAGTAAAAAGAAGTCTTACATAAATCGGACAGGAACATTTGAGTTC

ACAAGCTTTTTCTACCGTTATGGATTTGTAGCCAACTTCAGTATGGAGCTGCCAAGCTTT

GGAGTTTCTGGGATTAATGAATCGGCTGACATGAGCATTGGAGTTACAGTGATAAAGAAC

AATATGATAAACAATGATCTTGGACCAGCAACAGCTCAAATGGCTCTTCAGTTATTCATC

AAGGACTACAGATACACATATCGATGCCACAGGGGCGATACACAAATTCAAACGAGGAGA

TCATTCGAGCTAAAGAAACTGTGGGAGCAGACCCGTTCAAGAGCAGGACTGTTGGTTTCA

GATGGAGGACCAAACCTATACAATATTCGGAATCTCCACATTCCCGAGGTCTGCTTGAAG

TGGGAACTGATGGATGAAGATTACCAGGGTAGACTGTGTAATCCTCTGAATCCCTTTGTC

AGTCATAAGGAAATTGAGTCTGTAAACAATGCTGTAGTAATGCCAGCCCATGGTCCAGCC

AAAAGCATGGAATATGATGCTGTTGCGACTACACACTCATGGATCCCTAAGAGGAACCGT

TCCATTCTGAATACTAGCCAAAGAGGAATCCTTGAGGATGAACAGATGTACCAGAAGTGC

TGCAATCTATTTGAAAAATTCTTCCCTAGTAGTTCATACAGGAGGCCAGTTGGAATCTCC

AGCATGGTGGAGGCCATGGTGTCTAGGGCCCGAATTGATGCACGGATTGACTTCGAGTCT

GGAAGGATTAAGAAGGAAGATTTTGCTGAGATCATGAAGATCTGTTCCACCATTGAAGAG

CTCAGACGGCAGAAATAGTGA---------------------------------------

-

>A_Turkey_Egypt_AR550_2018_EPI1420342

------------CAAACCATTTGAATGGATGTCAATCCGACTTTACTTTTCTTAAAAGTG

CCAGCGCAAAATGCTATAAGTACTACATTCCCTTACACTGGAGATCCTCCATACAGCCAT

GGAACAGGAACAGGATATACCATGGACACAGTAAACAGAACACATCAATACTCAGAAAAG

GGAAAGTGGACAACAAACACAGAAACCGGAGCACCCCAACTCAACCCAATTGATGGACCA

TTACCAGAGAACAATGAGCCAAGCGGATATGCACAAACTGATTGCGTGTTGGAAGCAATG

GCTTTCCTTGAAGAATCCCACCCAGGGATATTTGAAAACTCTTGTCTTGAAGCGATGGAA

ATCGTTCAGCAAACAAGAGTGGACAAACTAACCCAAGGTCGCCAGACTTATGACTGGACA

CTGAACAGAAACCAACCAGCTGCAACTGCTTTGGCCAACACTATAGAGGTGTTCAGATCG

AATGGTCTGACAGCCAATGAATCAGGGAGACTGATAGATTTTCTCAGGGATGTGATGGAA

TCAATGGATAAAGAAGAGATGGAAATAACAACACATTTCCAGAGAAAAAGAAGAGTGAGG

GACAACATGACCAAGAAGATGGTCACACAAAGAACAATAGGAAAGAAGAAGCAGRGACTG

AACAAGAGGAGTTACTTAATAAGAGCACTGACATTGAACACAATGACCAAAGATGCAGAA

AGAGGCAAATTGAAGAGACGGGCAATTGCAACACCCGGGATGCAGATTAGAGGATTCGTG

TACTTCGTCGAAACACTAGCGAGGAGCATCTGTGAGAAACTCGAGCAATCTGGGCTCCCT

GTTGGAGGGAATGAGAAGAAGGCTAAATTGGCAAATGTCGTGAGAAAAATGATGACTAAC

TCACAAGATACAGAGCTCTCCTTTACAATTACTGGAGACAACACCAAATGGAATGAGAAT

CAAAACCCTCGGATGTTTTTGGCAATGATAACATACATCACAAGAAACCAACCTGAATGG

TTTAGAAATGTCTTAAGCATTGCCCCTATAATGTTCTCAAACAAAATGGCGAGATTAGGG

AAAGGATACATGTTTGAAAGTAAGAGCATGAAGCTAAGGACACAAATACCGGCAGAAATG

CTTGCAAACATTGACTTAAAATACTTCAATGAATCAACAAGAAAGAAAATCGAGAAAATA

AGACCTCTGCTAATTGATGGCACAGCCTCATTGAGTCCTGGAATGATGATGGGCATGTTC

AATATGCTGAGCACAGTATTGGGGGTCTCAATCCTGAATCTTGGACAAAAGAGGTACACT

AAAACCACATACTGGTGGGATGGGCTCCAATCCTCTGATGATTTCGCTCTCATAGTGAAT

GCACCGAATCATGAGGGGATACAAGCAGGGGTGGATAGGTTCTATAGGACCTGCAAACTG

GTTGGGATCAACATGAGCAAAAAGAAGTCTTACATAAACCGGACAGGAACWTTTGAGTTC

ACAAGCTTTTTCTAYCGCTATGGATTTGTGGCCAACTTCAGTATGGAGTTACCCAGYTTT

GGAGTGTCTGGAATCAATGAATCAGCTGACATGAGCATTGGAGTTACAGTGATAAARAAC

AATATGATAAACAATGAYCTTGGACCAGCAACAGCTCAAATGGCTCTTCAGYTATTCATC

AAGGACTATAGGTACACGTACCGATGCCACAGGGGTGATACACAAATTCAAACGAGGAGA

TCATTCGAGCTGAAGAAGCTGTGGGAGCAGACCCGTTCAAAAGCAGGGCTGTTGGTATCA

GATGGRGGACCAAATCTATACAACATYCGGAATCTCCACATCCCAGAGGTCTGCTTGAAA

TGGGAACTGATGGATGAAGAKTACCAGGGCAGGCTGTGTAATCCWCTGAATCCATTTGTC

AGTCATAAGGAAATTGAGTCCGTRAACAATGCTGTGGTAATGCCAGCCCATGGCCCWGCC

AAGAGCATGGAATATGATGCTGTTGCGACTACACACTCGTGGATTCCTAAGAGGAACCGT

TCCATTCTCAATACCAGCCAAAGGGGAATTCTTGAGGATGAGCAAATGTACCAAAGGTGC

TGTAGTCTATTCGAGAAATTCTTCCCCAGCAGTTCATACAGGAGGCCAGTTGGGATTTCC

AGCATGGTGGAGGCCATGGTGTCTAGGGCCCGAATTGATGCACGCATCGATTTCGAATCT

GGAAGGACCAAGAAAGAAGAGTTTGCTGAGATCATGAAGATCTGTTCCACCATTGAAGAG

CTCAGACGGCAAAAACAGTGAATTTAGCTTGTCCTTCATGAAAAAATG------------

-

>A_Chicken_Egypt_AI20286_2019_EPI1638783

------------------------ATGGATGTCAATCCGACTTTACTTTTCTTAAAAGTG

CCAGCGCAAAATGCTATAAGTACTACATTTCCTTACACTGGAGATCCTCCATACAGCCAT

GGAACAGGAACAGGATACACCATGGACACAGTAAACAGAACACATCAATACTCAGAAAAG

GGAAAGTGGACAACAAACACAGAAACCGGAGCACCCCAACTCAACCCAATTGATGGACCA

TTACCAGAGGACAATGAGCCAAGCGGGTATGCACAAACTGATTGCGTGTTGGAAGCAATG

GCTTTCCTTGAAGAATCCCACCCAGGGATATTTGAAAACTCTTGTCTTGAAGCGATGGAA

ATCGTTCAGCAAACAAGAGTGGACAAACTAACCCAGGGTCGCCAGACTTATGACTGGACA

CTGAACAGAAACCAACCAGCGGCAACTGCTTTGGCCAACACTATAGAGGTGTTCAGATCG

AATGGTCTGACAGCCAATGAATCAGGGAGACTGATAGATTTTCTCAGGGATGTGATGGAA

TCAATGGATAAAGAAGTGATGGAAATAACAACACATTTCCAAAGAAAAAGAAGAGTGAGG

GACAATATGACCAAGAAGATGGTCACACAAAGAACAATAGGAAAGAAGAAGCAGAGGCTG

AACAAGAGGAGTTACTTAATAAGAGCACTGACATTGAACACAATGACCAAAGATGCAGAA

AGAGGCAAATTGAAGAGACGGGCAATTGCAACACCCGGGATGCAGATTAGAGGATTCGTG

TACTTTGTCGAAACACTAGCGAGGAGCATCTGTGAGAAACTCGAGCAATCTGGGCTCCCT

GTTGGAGGGAATGAGAAGAAGGCTAAATTGGCAAATGTCGTGAGAAAAATGATGACTAAC

TCACAAGATACAGAGCTCTCCTTTACAATTACTGGAGACAACACCAAATGGAATGAGAAT

CAAAACCCTCGGATGTTTTTGGCAATGATAACATACATTACAAGAAACCAACCTGAATGG

TTTAGAAATGTCTTAAGCATTGCCCCTATAATGTTCTCAAACAAAATGGCGAGATTAGGG

AAAGGATACATGTTTGAAAGTAAGAGCATGAAGCTAAGGACACAAATACCAGCAGAGATG

CTTACAAACATTGATCTGAAGTATTTCAACGAAACAACGAGAAAGAAAATCGAGAAAATA

AGACCTCTGCTGATTGATGGCACGGCCTCATTGAGTCCTGGGATGATGATGGGCATGTTC

AATATGCTGAGCACAGTATTAGGGGTCTCAATCTTGAATCTCGGGCAAAAGAGGTACACC

AAAACCACATACTGGTGGGATGGACTTCAATCCTCTGATGATTTCGCTCTCATAGTGAAT

GCACCGAATCATGAGGGGATACAAGCAGGAGTGGATAGGTTCTATAGGACCTGCAAACTG

GTTGGGATCAACATGAGCAAAAAGAAGTCTTACATAAACCGGACAGGAACATTTGAGTTC

ACAAGTTTTTTCTACCGCTATGGATTTGTAGCTAACTTCAGTATGGAATTACCCAGCTTT

GGAGTGTCTGGAATCAATGAATCAGCTGACATGAGCATTGGAGTTACAGTGATAAAAAAC

AATATGATAAACAATGATCTTGGACCAGCAACAGCTCAAATGGCTCTTCAGTTATTCATC

AAAGACTATAGGTACACGTACCGATGCCACAGGGGTGATACACAAATTCAAACGAGGAGA

TCATTCGAGCTGAAGAAGCTGTGGGAGCAGACCCGTTCAAAGGCAGGGCTGTTGATATCA

GACGGAGGACCAAACCTATACAACATTCGGAATCTCCACATCCCAGAGGTCTGCTTGAAG

TGGGAACTGATGGATGAAGACTACCAAGGCAGGCTGTGCAATCCTCTGAATCCATTTGTC

AGTCATAAAGAGATTGAGTCCGTAAACAATGCTGTAGTGATGCCAGCCCATGGCCCGGCC

AAGAGCATGGAATATGATGCTGTTGCGACTACACACTCGTGGATTCCTAAGAGGAACCGT

TCCATTCTCAATACCAGCCAAAGGGGAATTCTTGAGGATGAGCAAATGTACCAAAAGTGC

TGTAGTCTATTCGAGAAATTCTTCCCCAGCAGTTCATACAGGAGGCCAGTTGGAATTTCC

AGCATGGTGGAGGCCATGGTGTCTAGGGCCCGAATTGATGCACGCATCGATTTCGAATCT

GGAAGGATTAAGAAAGAAGAGTTTGCTGAGATCATGAAGATCTGTTCCACCATTGAAGAG

CTCAGACGGCAAAAATAG------------------------------------------

-

>A_Chicken_Egypt_AR553_2018_EPI1638791

------------------------ATGGATGTCAATCCGACTTTACTTTTCTTAAAAGTG

CCAGCGCAAAATGCTATAAGTACTACATTCCCTTACACTGGAGATCCTCCATACAGCCAT

GGAACAGGAACAGGATATACCATGGACACAGTAAACAGAACACATCAATACTCAGAAAAG

GGAAAGTGGACAACAAACACAGAAACCGGAGCACCCCAACTCAACCCAATTGATGGACCA

TTACCAGAGAACAATGAGCCAAGCGGATATGCACAAACTGATTGCGTGTTGGAAGCAATG

GCTTTCCTTGAAGAATCCCACCCAGGGATATTTGAAAACTCTTGTCTTGAAGCGATGGAA

ATCGTTCAGCAAACAAGAGTGGACAAACTAACCCAAGGTCGCCAGACTTATGACTGGACA

CTGAACAGAAACCAACCAGCTGCAACTGCTTTGGCCAACACTATAGAGGTGTTCAGATCG

AATGGTCTGACAGCCAATGAATCAGGGAGACTGATAGATTTTCTCAGGGATGTGATGGAA

TCAATGGATAAAGAAGAGATGGAAATAACAACACATTTCCAGAGAAAAAGAAGAGTGAGG

GACAACATGACCAAGAAGATGGTCACACAAAGAACAATAGGAAAGAAGAAGCAGAGACTG

AACAAGAGGAGTTACTTAATAAGAGCACTGACATTGAACACAATGACCAAAGATGCAGAA

AGAGGCAAATTGAAGAGACGGGCAATTGCAACACCCGGGATGCAGATTAGAGGATTCGTG

TACTTCGTCGAAACACTAGCGAGGAGCATCTGTGAGAAACTCGAGCAATCTGGGCTCCCT

GTTGGAGGGAATGAGAAGAAGGCTAAATTGGCAAATGTCGTGAGAAAAATGATGACTAAC

TCACAAGATACAGAGCTCTCCTTTACAATTACTGGAGACAACACCAAATGGAATGAGAAT

CAAAACCCTCGGATGTTTTTGGCAATGATAACATACATCACAAGAAACCAACCTGAATGG

TTTAGAAATGTCTTAAGCATTGCCCCTATAATGTTCTCAAACAAAATGGCGAGATTAGGG

AAAGGATACATGTTTGAAAGTAAGAGCATGAAGCTAAGGACACAAATACCAGCAGAGATG

CTTACAAACATTGATCTGAAATATTTCAACGAATCAACGAGAAAGAAAATCGAGAAAATA

AGACCTCTGCTGATTGATGGCACGGCCTCATTGAGTCCTGGGATGATGATGGGCATGTTC

AATATGCTGAGCACAGTATTAGGGGTCTCAATCCTGAATCTCGGGCAAAAGAGGTACACC

AAAACCACATACTGGTGGGATGGACTTCAATCCTCTGATGATTTCGCTCTCATAGTGAAT

GCACCGAATCATGAGGGGATACAAGCAGGAGTGGATAGGTTCTATAGGACCTGCAAACTG

GTTGGGATCAACATGAGCAAAAAGAAGTCTTACATAAACCGGACAGGAACATTTGAGTTC

ACAAGTTTTTTCTACCGCTATGGATTTGTAGCTAACTTCAGTATGGAATTACCCAGCTTT

GGAGTGTCTGGAATCAATGAATCAGCTGACATGAGCATTGGAGTTACAGTGATAAAAAAC

AATATGATAAACAATGATCTTGGACCAGCAACAGCTCAAATGGCTCTTCAGCTATTCATC

AAAGACTATAGGTACACGTACCGATGCCACAGGGGTGATACACAAATTCAAACGAGGAGA

TCATTCGAGCTGAAGAAGCTGTGGGAGCAGACCCGTTCAAAGGCAGGGCTGTTGATATCA

GACGGAGGACCAAACCTATACAACATTCGGAATCTCCACATCCCAGAGGTCTGCTTGAAG

TGGGAACTGATGGATGAAGACTACCAAGGCAGGCTGTGCAATCCTCTGAATCCATTTGTC

AGTCATAAAGAGATTGAGTCCGTAAACAATGCTGTAGTGATGCCAGCCCATGGCCCGGCC

AAGAGCATGGAATATGATGCTGTTGCGACTACACACTCGTGGATTCCTAAGAGGAACCGT

TCCATTCTCAATACCAGCCAAAGGGGAATTCTTGAGGATGAGCAAATGTACCAAAAGTGC

TGTAGTCTATTCGAGAAATTCTTCCCCAGCAGTTCATACAGGAGGCCAGTTGGAATTTCC

AGCATGGTGGAGGCCATGGTGTCTAGGGCCCGAATTGATGCACGCATCGATTTCGAATCT

GGAAGGATTAAGAAAGAAGAGTTTGCTGAGATCATGAAGATCTGTTCCACCATTGAAGAG

CTCAGACGGCAAAAATAG------------------------------------------

-

>A_Turkey_Egypt_AI20285_2019_EPI1638796

------------------------------------------TTACTTTTCTTAAAAGTG

CCAGCGCAAAATGCTATAAGTACTACATTCCCTTACACTGGAGATCCTCCATACAGCCAT

GGAACAGGAACAGGATATACCATGGACACAGTAAACAGAACACATCAATACTCAGAAAAG

GGAAAGTGGACAAAAAACACAGAAACCGGAGCACCCCAACTCAACCCAATTGATGGACCA

TTACCAGAGGACAATGAGCCAAGCGGATATGCACAAACTGATTGCGTGTTGGAAGCAATG

GCTTTCCTTGAAGAATCCCACCCAGGGATATTTGAAAACTCTTGTCTTGAAGCGATGGAA

ACCGTTCAGCAAACAAGAGTGGACAAACTAACCCAAGGTCGCCAGACTTATGACTGGACA

CTGAACAGAAACCAACCAGCTGCAACCGCTTTGGCCAACACTATAGAGGTGTTCAGATCG

AATGGTCTGACAGCCAATGAATCAGGGAGACTGATAGATTTTCTCAGGGATGTGATGGAA

TCAATGGATAAAGAGGAGATGGAAATAACAACACATTTCCAGAGAAAAAGAAGAGTGAGG

GACAATATGACCAAGAAGATGGTCACACAAAGAACAATAGGAAAGAAGAAGCAGAGGCTG

AACAAGAGGAGTTACTTAATAAGAGCACTGACATTGAACACAATGACCAAAGATGCAGAA

AGAGGAAAATTGAAGAGACGGGCAATTGCAACACCCGGGATGCAGATTAGAGGATTCGTG

TATTTCGTCGAAACACTAGCGAGGAGCATCTGTGAGAAACTCGAGCAATCTGGGCTCCCT

GTTGGAGGGAATGAGAAGAAGGCTAAATTGGCAAATGTCGTGAGAAAAATGATGACTAAC

TCACAAGATACAGAGCTCTCCTTTACAATTACTGGAGACAACACCAAATGGAATGAGAAT

CAAAACCCTCGGATGTTTTTGGCAATGATAACATACATTACAAGAAACCAACCTGAATGG

TTTAGAAATGTCTTAAGCATTGCCCCTATAATGTTCTCAAACAAAATGGCGAGATTAGGG

AAAGGATACATGTTTGAAAGTAAGAGCATGAAGCTAAGGACACAAATACCAGCAGAGATG

CTTACAAACATTGACCTGAAGTATTTCAACGAACCAACGAGAAAGAAAATCGAGAAAATA

AGACCTCTGCTGATTGATGGCACGGCCTCATTGAGTCCTGGGATGATGATGGGCATGTTC

AATATGCTGAGCACAGTATTAGGGGTCGCAATCCTGAATCTCGGGCAAAAGAGGTACACA

AAAACCACATACTGGTGGGATGGACTTCAATCCTCTGATGACTTCGCTCTCATAGTGAAT

GCACCGAATCATGAGGGGATACAAGCAGGAGTGGATAGGTTCTATAGGACCTGCAAACTG

GTTGGGATCAACATGAGCAAAAAGAAATCTTACATAAACCGGACAGGAACATTTGAGTTC

ACAAGTTTTTTCTACCGCTATGGATTTGTAGCTAATTTCAGTATGGAATTACCAAGCTTT

GGAGTGTCTGGAATCAATGAATCTGCTGACATGAGCATTGGAGTTACTGTGATAAAAAAC

AATATGATAAACAATGATCTTGGACCAGCAACAGCTCAAATGGCTCTTCAGTTATTCATC

AAAGACTATAGGTACACGTACCGATGCCACAGGGGTGACACACAAATTCAAACGAGGAGA

TCATTCGAGCTGAAGAAGCTGTGGGAGCAGACCCGTTCAAAGGCAGGGCTGTTGATATCA

GACGGAGGACCAAACCTATACAACATTCGGAATCTCCACATCCCAGAGGTCTGCTTGAAG

TGGGAACTGATGGATGAAGACTACCAAGGCAGACTGTGCAATCCTCTGAATCCATTTGTC

AGTCATAAAGAGATTGAGTCCGTAAACAATGCTGTAGTAATGCCAGCCCATGGCCCGGCC

AAGAGCATGGAATATGATGCTGTTGCGACTACACACTCGTGGATTCCTAAGAGGAACCGT

TCCATTCTCAATACCAGCCAAAGGGGAATTCTTGAGGATGAGCAAATGTACCAAAAGTGC

TGTAGTCTATTCGAGAAATTCTTCCCCAGCAGTTCATACAGGAGGCCAGTTGGAATTTCC

AGCATGGTGGAGGCCATGGTGTCTAGGGCCCGAATTGATGCACGCATCGATTTCGAATCT

GGAAGGATTAAGAAAGAAGAGTTTGCTGAGATCATGAAGATCTGTTCCACCATTGAAGAG

CTCAGACGGCAAAAACAGTGAATTT-----------------------------------

-

>A_goose_Omsk_0111_2020_EPI1813143

AGCAAAAGCAGGCAAACTATTTGAATGGATGTCAATCCGACTTTACTTTTCTTAAAAGTG

CCAGCGCAAAATGCTATAAGTACTACATTCCCTTACACTGGAGATCCTCCATACAGCCAT

GGAACAGGAACAGGGTATACCATGGACACAGTAAACAGAACACATCAATACTCAGAAAAG

GGAAAGTGGACAACAAACACAGAAACCGGAGCACCCCAACTCAACCCAATTGATGGACCA

TTACCAGAGGACAATGAGCCAAGCGGATATGCACAAACTGATTGCGTGTTGGAAGCAATG

GCTTTCCTTGAAGAATCCCACCCAGGGATATTTGAAAACTCTTGTCTTGAAGCGATGGAA

ATCGTTCAGCAAACAAGAGTGGACAAACTAACCCAAGGTCGCCAGACTTATGACTGGACA

CTGAACAGAAACCAACCAGCTGCAACCTCTTTGGCCAACACTATAGAGGTGTTCAGATCG

AATGGTCTGACAGCCAATGAATCAGGGAGACTGATAGATTTTCTCAGGGATGTGATGGAA

TCAATGGATAAAGAAGAGATGGAAGTAACAACACATTTCCAGAGAAAAAGAAGAGTGAGG

GACAACATGACTAAGAAGATGGTCACACAAAGAACAATAGGGAAGAAGAAGCAGAGGCTG

AACAAGAGGAGTTACTTAATAAGAGCACTGACATTGAATACAATGACCAAAGATGCAGAA

AGAGGCAAGTTGAAGAGACGGGCAATTGCAACACCCGGGATGCAGATTAGAGGATTCGTG

TACTTCGTTGAAACACTAGCGAGGAGCATCTGTGAGAAACTAGAGCAATCTGGGCTCCCT

GTTGGAGGGAATGAGAAGAAGGCTAAATTGGCAAATGTTGTGAGAAAAATGATGACTAAC

TCACAAGATACAGAGCTCTCCTTTACAATTACTGGAGACAACACCAAATGGAATGAGAAT

CAAAACCCTCGGATGTTTTTGGCAATGATAACATATATCACAAGAAACCAACCTGAATGG

TTTAGAAATGTCTTAAGCATTGCCCCTATAATGTTCTCAAACAAAATGGCGAGATTAGGG

AAAGGATACATGTTTGAAAGTAAGAGCATGAAGCTAAGAACACAAATACCAGCAGAGATG

CTTACAAATATTGATCTGAAGTATTTCAACGAACCAACGAGAAAGAAAATCGAGAAAATA

AGACCTCTGCTGATTGATGGCACGGCCTCATTGAGTCCTGGGATGATGATGGGCATGTTC

AATATGCTGAGCACAGTATTGGGGGTCTCAATCCTGAATCTCGGGCAAAAGAGGTACACC

AAAACCACATACTGGTGGGATGGACTTCAATCCTCTGATGATTTCGCTCTCATAGTGAAT

GCACCGAATCATGAGGGGATACAAGCAGGAGTGGATAGATTCTATAGGACCTGCAAACTG

GTTGGGATCAACATGAGCAAAAAGAAGTCTTACATAAACCGAACAGGAACATTTGAGTTC

ACAAGTTTTTTCTACCGCTATGGATTTGTAGCTAACTTCAGTATGGAATTACCCAGCTTT

GGAGTGTCTGGAATCAATGAATCAGCTGACATGAGCATTGGAGTTACAGTGATAAAAAAC

AATATGATAAACAATGATCTTGGACCAGCAACAGCTCAAATGGCTCTTCAGTTATTCATC

AAAGACTATAGGTACACATACCGATGCCACAGGGGTGATACACAAATTCAAACGAGGAGA

TCATTCGAGCTGAAGAAGCTGTGGGAGCAGACCCGTTCAAAGGCAGGGCTGTTGATATCA

GACGGGGGGCCAAACCTATACAACATTCGGAACCTCCACATCCCAGAGGTCTGCTTGAAG

TGGGAGCTGATGGATGAAGACTACCAAGGCAGGCTGTGCAATCCTCTGAATCCATTTGTC

AGTCATAAAGAGATTGAGTCCGTAAACAATGCTGTAGTAATGCCCGCCCATGGCCCGGCC

AAGAGCATGGAATATGATGCTGTTGCGACCACACATTCGTGGATCCCTAAGAGGAACCGT

TCCATTCTCAATACCAGCCAAAGGGGAATTCTTGAGGATGAGCAGATGTACCAAAAGTGC

TGTAGTCTATTCGAGAAATTCTTCCCCAGCAGTTCATACAGGAGGCCAGTTGGAATTTCC

AGCATGGTGGAGGCCATGGTGTCTAGGGCCCGAATTGATGCACGCATCGACTTCGAATCT

GGAAGGATTAAGAAAGAAGAGTTTGCTGAGATCATGAAGATCTGTTCCACCATTGAAGAG

CTCAGACGGCAAAAATAGTGAATTTAGCTTGTCCTTCATGAAAAAATGCCTTGTTTCTAC

T

>A_goose_Omsk_01161_2020_EPI1813359

AGCAAAAGCAGGCAAACTATTTGAATGGATGTCAATCCGACTTTACTTTTCTTAAAAGTG

CCAGCGCAAAATGCTATAAGTACTACATTCCCTTACACTGGAGATCCCCCATACAGCCAT

GGAACAGGAACAGGGTATACCATGGACACAGTAAACAGAACACATCAATACTCAGAAAAG

GGAAAGTGGACAACAAACACAGAAACCGGAGCACCCCAACTCAACCCAATTGATGGACCA

TTACCAGAGGACAATGAGCCAAGCGGATATGCACAAACTGATTGCGTGTTGGAAGCAATG

GCTTTCCTTGAAGAATCCCACCCAGGGATATTTGAAAACTCTTGTCTTGAAGCGATGGAA

ATCGTTCAGCAAACAAGAGTGGACAAACTAACCCAAGGTCGCCAGACTTATGACTGGACA

CTGAACAGAAACCAACCAGCTGCAACCTCTTTGGCCAACACTATAGAGGTGTTCAGATCG

AATGGTCTGACAGCCAATGAATCAGGGAGACTGATAGATTTTCTCAGGGATGTGATGGAA

TCAATGGATAAAGAAGAGATGGAAGTAACAACACATTTCCAGAGAAAAAGAAGAGTGAGG

GACAACATGACTAAGAAGATGGTCACACAAAGAACAATAGGGAAGAAGAAGCAGAGGCTG

AACAAGAGGAGTTACTTAATAAGAGCACTGACATTGAATACAATGACCAAAGATGCAGAA

AGAGGCAAGTTGAAGAGACGGGCAATTGCAACACCCGGGATGCAGATTAGAGGGTTCGTG

TACTTCGTTGAAACACTAGCGAGGAGCATCTGTGAGAAACTAGAGCAATCTGGGCTCCCT

GTTGGAGGGAATGAGAAGAAGGCTAAATTGGCAAATGTTGTGAGAAAAATGATGACTAAC

TCACAAGATACAGAGCTCTCCTTTACAATTACTGGAGACAACACCAAATGGAATGAGAAT

CAAAACCCTCGGATGTTTTTGGCAATGATAACATATATCACAAGAAACCAACCTGAATGG

TTTAGAAATGTCTTAAGCATTGCCCCTATAATGTTCTCAAACAAAATGGCGAGATTAGGG

AAAGGATACATGTTTGAAAGTAAGAGCATGAAGCTAAGAACACAAATACCAGCAGAGATG

CTTACAAATATTGATCTGAAGTATTTCAACGAACCAACGAGAAAGAAAATCGAGAAAATA

AGACCTCTGCTGATTGATGGCACGGCCTCATTGAGTCCTGGGATGATGATGGGCATGTTC

AATATGCTGAGCACAGTATTAGGGGTCTCAATCCTGAATCTCGGGCAAAAGAGGTACACC

AAAACCACATACTGGTGGGATGGACTTCAATCCTCTGATGATTTCGCTCTCATAGTGAAT

GCACCGAATCATGAGGGGATACAAGCAGGAGTGGATAGATTCTATAGGACCTGCAAACTG

GTTGGGATCAACATGAGCAAAAAGAAGTCTTACATAAACCGAACAGGAACATTTGAGTTC

ACAAGTTTTTTCTACCGCTATGGATTTGTAGCTAACTTCAGTATGGAATTACCCAGCTTT

GGAGTGTCTGGAATCAATGAATCAGCTGACATGAGCATTGGAGTTACAGTGATAAAAAAC

AATATGATAAACAATGATCTTGGACCAGCAACAGCTCAAATGGCTCTTCAGTTATTCATC

AAAGACTATAGGTACACATACCGATGCCACAGGGGTGATACACAAATTCAAACGAGGAGA

TCATTCGAGCTGAAGAAGCTGTGGGAGCAGACCCGTTCAAAGGCAGGGCTGTTGATATCA

GACGGGGGGCCAAACCTATACAACATTCGGAATCTCCACATCCCAGAGGTCTGCTTGAAG

TGGGAGCTGATGGATGAAGACTACCAAGGCAGGCTGTGCAATCCTCTGAATCCATTTGTC

AGTCATAAAGAGATTGAGTCCGTAAACAATGCTGTAGTAATGCCCGCCCATGGCCCGGCC

AAGAGCATGGAATATGATGCTGTTGCGACCACACACTCGTGGATTCCTAAGAGGAACCGT

TCCATTCTCAATACCAGCCAAAGGGGAATTCTTGAGGATGAGCAGATGTACCAAAAGTGC

TGTAGTCTATTCGAGAAATTCTTCCCCAGCAGTTCATACAGGAGGCCAGTTGGAATTTCC

AGCATGGTGGAGGCCATGGTGTCTAGGGCCCGAATTGATGCACGCATCGACTTCGAATCT

GGAAGGATTAAGAAAGAAGAGTTTGCTGAGATCATGAAGATCTGTTCCACCATTGAAGAG

CTCAGACGGCAAAAATAGTGAATTTAGCTTGTCCTTCATGAAAAAATGCCTTGTTTCTAC

T

>A_chicken_Omsk_0118_2020_EPI1813375

AGCAAAAGCAGGCAAACTATTTGAATGGATGTCAATCCGACTTTACTTTTCTTAAAAGTG

CCAGCGCAAAATGCTATAAGTACTACATTCCCTTACACTGGAGATCCTCCATACAGCCAT

GGAACAGGAACAGGGTATACCATGGACACAGTAAACAGAACACATCAATACTCAGAAAAG

GGAAAGTGGACAACAAACACAGAAACCGGAGCACCCCAACTCAACCCAATTGATGGACCA

CTACCAGAGGACAATGAGCCAAGCGGATATGCACAAACTGATTGCGTGTTGGAAGCAATG

GCTTTCCTTGAAGAATCCCACCCAGGGATATTTGAAAACTCTTGTCTTGAAGCGATGGAA

ATCGTTCAGCAAACAAGAGTGGACAAACTAACCCAAGGTCGCCAGACTTATGACTGGACA

CTGAACAGAAACCAACCAGCTGCAACCTCTTTGGCCAACACTATAGAGGTGTTCAGATCG

AATGGTCTGACAGCCAATGAATCAGGGAGACTGATAGATTTTCTCAGGGATGTGATGGAA

TCAATGGATAAAGAAGAGATGGAAGTAACAACACATTTCCAGAGAAAAAGAAGAGTGAGG

GACAACATGACTAAGAAGATGGTCACACAAAGAACAATAGGGAAGAAGAAGCAGAGGCTG

AACAAGAGGAGTTACTTAATAAGAGCACTGACATTGAATACAATGACCAAAGATGCAGAA

AGAGGCAAGTTGAAGAGACGGGCAATTGCAACACCCGGGATGCAGATTAGAGGATTCGTG

TACTTCGTTGAAACACTAGCGAGGAGCATCTGTGAGAAACTAGAGCAATCTGGGCTCCCT

GTTGGAGGGAATGAGAAGAAGGCTAAATTGGCAAATGTTGTGAGAAAAATGATGACTAAC

TCACAAGATACAGAGCTCTCCTTTACAATTACTGGAGACAACACCAAATGGAATGAGAAT

CAAAACCCTCGGATGTTTTTGGCAATGATAACATATATCACAAGAAACCAACCTGAATGG

TTTAGAAATGTCTTAAGCATTGCCCCTATAATGTTCTCAAACAAAATGGCGAGATTAGGG

AAAGGATACATGTTTGAAAGTAAGAGCATGAAGCTAAGAACACAAATACCAGCAGAGATG

CTTACAAATATTGATCTGAAGTATTTCAACGAACCAACGAGAAAGAAAATCGAGAAAATA

AGACCTCTGCTGATTGATGGCACGGCCTCATTGAGTCCTGGGATGATGATGGGCATGTTC

AATATGCTGAGCACAGTATTGGGGGTCTCAATCCTGAATCTCGGGCAAAAGAGGTACACC

AAAACCACATACTGGTGGGATGGACTTCAATCCTCTGATGATTTCGCTCTCATAGTGAAT

GCACCGAATCATGAGGGGATACAAGCAGGAGTGGATAGATTCTATAGGACCTGCAAACTG

GTTGGGATCAACATGAGCAAAAAGAAGTCTTACATAAACCGAACAGGAACATTTGAGTTC

ACAAGTTTTTTCTACCGCTATGGATTTGTAGCTAACTTCAGTATGGAATTACCCAGCTTT

GGAGTGTCTGGAATCAATGAATCAGCTGACATGAGCATTGGAGTTACAGTGATAAAAAAC

AATATGATAAACAATGATCTTGGACCAGCGACAGCTCAAATGGCTCTTCAGTTATTCATC

AAAGACTATAGGTACACATACCGATGCCACAGGGGTGATACACAAATTCAAACGAGGAGA

TCATTCGAGCTGAAGAAGCTGTGGGAGCAGACCCGTTCAAAGGCAGGGCTGTTGATATCA

GACGGGGGGCCAAACCTATACAACATTCGGAACCTCCACATCCCAGAGGTCTGCTTGAAG

TGGGAGCTGATGGATGAAGACTACCAAGGCAGGCTGTGCAATCCTCTGAATCCATTTGTC

AGTCATAAAGAGATTGAGTCCGTAAACAATGCTGTAGTAATGCCCGCCCATGGCCCGGCC

AAGAGCATGGAATATGATGCTGTTGCGACCACACATTCGTGGATTCCTAAGAGGAACCGT

TCCATTCTCAATACCAGCCAAAGGGGAATTCTTGAGGATGAGCAGATGTACCAAAAGTGC

TGTAGTCTATTCGAGAAATTCTTCCCCAGCAGTTCATACAGGAGGCCAGTTGGAATTTCC

AGCATGGTGGAGGCCATGGTGTCTAGGGCCCGAATTGATGCACGCATCGACTTCGAATCT

GGAAGGATTAAGAAAGAAGAGTTTGCTGAGATCATGAAGATCTGTTCCACCATTGAAGAG

CTCAGACGGCAAAAATAGTGAATTTAGCTTGTCCTTCATGAAAAAATGCCTTGTTTCTAC

T

>A_chicken_Omsk_0119_2020_EPI1813383

AGCAAAAGCAGGCAAACTATTTGAATGGATGTCAATCCGACTTTGCTTTTCTTAAAAGTG

CCAGCGCAAAATGCTATAAGTACTACATTCCCTTACACTGGAGATCCTCCATACAGCCAT

GGAACAGGAACAGGGTATACCATGGACACAGTAAACAGAACACATCAATACTCAGAAAAG

GGAAAGTGGACAACAAACACAGAAACCGGAGCACCCCAACTCAACCCAATTGATGGACCA

TTACCAGAGGACAATGAGCCAAGCGGATATGCACAAACTGATTGCGTGTTGGAAGCAATG

GCTTTCCTTGAAGAATCCCACCCAGGGATATTTGAAAACTCTTGTCTTGAAGCGATGGAA

ATCGTTCAGCAAACAAGAGTGGACAAACTAACCCAAGGTCGCCAGACTTATGACTGGACA

CTGAACAGAAACCAACCAGCTGCAACCTCTTTGGCCAACACTATAGAGGTGTTCAGATCG

AATGGTCTGACAGCCAATGAATCAGGGAGACTGATAGATTTTCTCAGGGATGTGATGGAA

TCAATGGATAAAGAAGAGATGGAAGTAACAACACATTTCCAGAGAAAAAGAAGAGTGAGG

GACAACATGACTAAGAAGATGGTCACACAAAGAACAATAGGGAAGAAGAAGCAGAGGCTG

AACAAGAGGAGTTACTTAATAAGAGCACTGACATTGAATACAATGACCAAAGATGCAGAA

AGAGGCAAGTTGAAGAGACGGGCAATTGCAACACCCGGGATGCAGATTAGAGGATTCGTG

TACTTCGTTGAAACACTAGCGAGGAGCATCTGTGAGAAACTAGAGCAATCTGGGCTCCCT

GTTGGAGGGAATGAGAAGAAGGCTAAATTGGCAAATGTTGTGAGAAAAATGATGACTAAC

TCACAAGATACAGAGCTCTCCTTTACAATTACTGGAGACAACACCAAATGGAATGAGAAT

CAAAACCCTCGGATGTTTTTGGCAATGATAACATATATCACAAGAAACCAACCTGAATGG

TTTAGAAATGTCTTAAGCATTGCCCCTATAATGTTCTCAAACAAAATGGCGAGATTAGGG

AAAGGATACATGTTTGAAAGTAAGAGCATGAAGCTAAGAACACAAATACCAGCAGAGATG

CTTACAAATATTGATCTGAAGTATTTCAACGAACCAACGAGAAAGAAAATCGAGAAAATA

AGACCTCTGCTGATTGATGGCACGGCCTCATTGAGTCCTGGGATGATGATGGGCATGTTC

AATATGCTGAGCACAGTATTGGGGGTCTCAATCCTGAATCTCGGGCAAAAGAGGTACACC

AAAACCACATACTGGTGGGATGGACTTCAATCCTCTGATGATTTCGCTCTCATAGTGAAT

GCACCGAATCATGAGGGGATACAAGCAGGAGTGGATAGATTCTATAGGACCTGCAAACTG

GTTGGGATCAACATGAGCAAAAAGAAGTCTTACATAAACCGAACAGGAACATTTGAGTTC

ACAAGTTTTTTCTACCGCTATGGATTTGTAGCTAACTTCAGTATGGAATTACCCAGCTTT

GGAGTGTCTGGAATCAATGAATCAGCTGACATGAGCATTGGAGTTACAGTGATAAAAAAC

AATATGATAAACAATGATCTTGGACCAGCGACAGCTCAAATGGCTCTTCAGTTATTCATC

AAAGACTATAGGTACACATACCGATGCCACAGGGGTGATACACAAATTCAAACGAGGAGA

TCATTCGAGCTGAAGAAGCTGTGGGAGCAGACCCGTTCAAAGGCAGGGCTGTTGATATCA

GACGGGGGGCCAAACCTATACAACATTCGGAACCTCCACATCCCAGAGGTCTGCTTGAAG

TGGGAGCTGATGGATGAAGACTACCAAGGCAGGCTGTGCAATCCTCTGAATCCATTTGTC

AGTCATAAAGAGATTGAGTCCGTAAACAATGCTGTAGTAATGCCCGCCCATGGCCCGGCC

AAGAGCATGGAATATGATGCTGTTGCGACCACACATTCGTGGATTCCTAAGAGGAACCGT

TCCATTCTCAATACCAGCCAAAGGGGAATTCTTGAGGATGAGCAGATGTACCAAAAGTGC

TGTAGTCTATTCGAGAAATTCTTCCCCAGCAGTTCATACAGGAGGCCAGTTGGAATTTCC

AGCATGGTGGAGGCCATGGTGTCTAGGGCCCGAATTGATGCACGCATCGACTTCGAATCT

GGAAGGATTAAGAAAGAAGAGTTTGCTGAGATCATGAAGATCTGTTCCACCATTGAAGAG

CTCAGACGGCAAAAATAGTGAATTTAGCTTGTCCTTCATGAAAAAATGCCTTGTTTCTAC

T

>A_chicken_Omsk_0073_2020_EPI1813407

AGCAAAAGCAGGCAAACTATTTGAATGGATGTCAATCCGACTTTACTTTTCTTAAAAGTG

CCAGCGCAAAATGCTATAAGTACTACATTCCCTTACACTGGAGATCCTCCATACAGCCAT

GGAACAGGAACAGGGTATACCATGGACACAGTAAACAGAACACATCAATACTCAGAAAAG

GGAAAGTGGACAACAAACACAGAAACCGGAGCACCCCAACTCAACCCAATTGATGGACCA

TTACCAGAGGACAATGAGCCAAGCGGATATGCACAAACTGATTGCGTGTTGGAAGCAATG

GCTTTCCTTGAAGAATCCCACCCAGGGATATTTGAAAACTCTTGTCTTGAAACGATGGAA

ATCGTTCAGCAAACAAGAGTGGACAAACTAACCCAAGGTCGCCAGACTTATGACTGGACA

CTGAACAGAAACCAACCAGCTGCAACCTCTTTGGCCAACACTATAGAGGTGTTCAGATCG

AATGGTCTGACAGCCAATGAATCAGGGAGACTGATAGATTTTCTCAGGGATGTGATGGAA

TCAATGGATAAAGAAGAGATGGAAGTAACAACACATTTCCAGAGAAAGAGAAGAGTGAGG

GACAACATGACTAGGAAGATGGTCACACAAAGAACAATAGGGAAGAAGAAGCAGAGGCTG

AACAAGAGGAGTTACTTAATAAGAGCACTGACATTGAATACAATGACCAAAGATGCAGAA

AGAGGCAAGTTGAAGAGACGGGCAATTGCAACGCCCGGGATGCAGATTAGAGGATTCGTG

TACTTCGTTGAAACACTAGCGAGGAGCATCTGTGAGAAACTAGAGCAATCTGGGCTCCCT

GTTGGAGGGAATGAGAAGAAGGCTAAATTGGCAAATGTTGTGAGAAAAATGATGACTAAC

TCACAAGATACAGAGCTCTCCTTTACAATTACTGGAGACAACACCAAATGGAATGAGAAT

CAAAACCCTCGGATGTTTTTGGCAATGATAACATATATCACAAGAAACCAACCTGAATGG

TTTAGAAATGTCTTAAGCATTGCCCCTATAATGTTCTCAAACAAAATGGCGAGATTAGGG

AAAGGATACATGTTTGAAAGTAAGAGCATGAAGCTAAGAACACAAATACCAGCAGAGATG

CTTACAAATATTGATCTGAAGTATTTCAACGAACCAACGAGAAAGAAAATCGAGAAAATA

AGACCTCTGCTGATTGATGGCACGGCCTCATTGAGTCCTGGGATGATGATGGGCATGTTC

AATATGCTGAGCACAGTATTAGGGGTCTCAATCCTGAATCTCGGGCAAAAAAGGTACACC

AAAACCACATACTGGTGGGATGGACTTCAATCCTCTGATGATTTCGCTCTCATAGTGAAT

GCACCGAATCATGAGGGGATACAAGCAGGAGTGGATAGATTCTATAGGACCTGCAAACTG

GTTGGGATCAACATGAGCAAAAAGAAGTCTTACATAAACCGAACAGGAACATTTGAGTTC

ACAAGTTTTTTCTACCGCTATGGATTTGTAGCTAACTTCAGTATGGAATTACCCAGCTTT

GGGGTGTCTGGAATCAATGAATCAGCTGACATGAGCATTGGAGTTACAGTGATAAAAAAC

AATATGATAAACAATGATCTTGGACCAGCAACAGCTCAAATGGCTCTTCAGTTATTCATC

AAAGACTATAGGTACACATACCGATGCCACAGGGGTGATACACAAATTCAAACGAGGAGA

TCATTCGAGCTGAAGAAGCTGTGGGAGCAGACCCGTTCAAAGGCAGGGCTGTTGATATCA

GATGGGGGGCCAAACCTATACAACATTCGGAATCTCCACATCCCAGAGGTCTGCTTGAAG

TGGGAGCTGATGGATGAAGACTACCAAGGCAGGCTGTGCAATCCTCTGAATCCATTTGTC

AGTCATAAAGAGATTGAGTCCGTAAACAATGCTGTAGTAATGCCCGCCCATGGCCCGGCC

AAGAGCATGGAATATGATGCTGTTGCGACCACACACTCGTGGATTCCTAAGAGGAACCGT

TCCATTCTCAATACCAGCCAAAGGGGAATTCTTGAGGATGAGCAGATGTACCAAAAGTGC

TGTAGTCTATTCGAGAAATTCTTCCCCAGCAGTTCATACAGGAGGCCAGTTGGAATTTCC

AGCATGGTGGAGGCCATGGTGTCTAGGGCCCGAATTGATGCACGCATCGACTTCGAATCT

GGAAGGATTAAGAAAGAAGAGTTTGCTGAGATCATGAAGATCTGTTCCACCATTGAAGAG

CTCAGACGGCAAAAATAGTGAATTTAGCTTGTCCTTCATGAAAAAATGCCTTGTTTCTAC

T

>A_chicken_Omsk_30007_2020_EPI1814311

AGCAAAAGCAGGCAAACTATTTGAATGGATGTCAACCCGACTTTACTTTTCTTAAAAGTG

CCAGCGCAAAATGCTATAAGTACTACATTCCCTTACACTGGAGATCCCCCATACAGCCAT

GGAACAGGAACAGGGTATACCATGGACACAGTAAACAGAACACATCAATACTCAGAAAAG

GGAAAGTGGACAACAAACACAGAAACCGGAGCACCCCAACTCAACCCAATTGATGGACCA

TTACCAGAGGACAATGAGCCAAGCGGATATGCACAAACTGATTGCGTGTTGGAAGCAATG

GCTTTCCTTGAAGAATCCCACCCAGGGATATTTGAAAACTCTTGTCTTGAAGCGATGGAA

ATCGTCCAGCAAACAAGAGTGGACAAACTAACCCAAGGTCGCCAGACTTATGACTGGACA

CTGAACAGAAACCAACCAGCTGCAACCTCTTTGGCCAACACTATAGAGGTGTTCAGATCG

AATGGTCTGACAGCCAATGAATCAGGGAGACTGATAGATTTTCTCAGGGATGTGATGGAA

TCAATGGATAAAGAAGAGATGGAAGTAACAACACATTTCCAGAGAAAAAGAAGAGTGAGG

GACAACATGACTAAGAAGATGGTCACACAAAGAACAATAGGGAAGAAGAAGCAGAGGCTG

AACAAGAGGAGTTACTTAATAAGAGCACTGACATTGAATACAATGACCAAAGATGCAGAA

AGAGGCAAGTTGAAAAGACGGGCAATTGCAACACCCGGGATGCAGATTAGAGGATTCGTG

TACTTCGTTGAAACACTAGCGAGGAGCATCTGTGAGAAACTAGAGCAATCTGGGCTCCCT

GTTGGAGGGAATGAGAAGAAGGCTAAATTGGCAAATGTTGTGAGAAAAATGATGACTAAT

TCACAAGATACAGAGCTCTCCTTTACAATTACTGGAGACAACACCAAATGGAATGAGAAT

CAAAACCCTCGGATGTTTTTGGCAATGATAACATATATCACAAGAAACCAACCTGAATGG

TTTAGAAATGTCTTAAGCATTGCCCCTATAATGTTCTCAAACAAAATGGCGAGATTAGGG

AAAGGATACATGTTTGAAAGTAAGAGCATGAAGCTAAGAACACAAATACCAGCAGAGATG

CTTACAAATATTGATCTGAAGTATTTCAACGAACCAACGAGAAAGAAAATCGAGAAAATA

AGACCTCTGCTGATTGATGGCACGGCCTCATTGAGTCCTGGGATGATGATGGGCATGTTC

AATATGCTGAGCACAGTATTAGGGGTCTCAATCCTGAATCTCGGGCAAAAGAGGTACACC

AAAACCACATACTGGTGGGATGGACTTCAATCCTCTGATGATTTCGCTCTCATAGTGAAT

GCACCGAATCATGAGGGGATACAAGCAGGAGTGGATAGATTCTATAGGACCTGCAAACTG

GTTGGGATCAACATGAGCAAAAAGAAGTCTTACATAAACCGAACAGGAACATTTGAGTTC

ACAAGTTTTTTCTACCGCTATGGATTTGTAGCTAACTTCAGTATGGAACTACCCAGCTTT

GGAGTGTCTGGAATCAATGAATCAGCTGACATGAGCATTGGAGTTACAGTGATAAAAAAC

AATATGATAAACAATGATCTTGGACCAGCAACAGCTCAAATGGCTCTTCAGTTATTCATC

AAAGACTATAGGTACACATACCGATGCCACAGGGGTGATACACAAATTCAAACGAGGAGA

TCATTCGAGCTGAAGAAGCTGTGGGAGCAGACCCGTTCAAAGGCAGGGCTGTTGATATCA

GACGGGGGGCCAAACCTATACAACATTCGGAATCTCCACATCCCAGAGGTCTGCTTGAAG

TGGGAGCTGATGGATGAAGACTACCAAGGCAGGCTGTGCAATCCTCTGAATCCATTTGTC

AGTCATAAAGAGATTGAGTCCGTAAACAATGCTGTAGTAATGCCCGCCCATGGCCCGGCC

AAGAGCATGGAATATGATGCTGTTGCGACCACACACTCGTGGATTCCTAAGAGGAACCGT

TCCATTCTCAATACCAGCCAAAGGGGAATTCTTGAGGATGAGCAGATGTACCAAAAGTGC

TGTAGTCTATTCGAGAAATTCTTCCCCAGCAGTTCATACAGGAGGCCAGTTGGAATTTCC

AGCATGGTGGAGGCCATGGTGTCTAGGGCCCGAATTGATGCACGCATCGACTTCGAATCT

GGAAGGATTAAGAAAGAAGAGTTTGCTGAGATCATGAAGATCTGTTCCACCATTGAAGAG

CTCAGACGGCAAAAATAGTGAATTTAGCTTGTCCTTCATGAAAAAATGCCTTGTTTCTAC

T

>A_goose_Omsk_30009_2020_EPI1814319

AGCAAAAGCAGGCAAACTATTTGAATGGATGTCAATCCGACTTTACTTTTCTTAAAAGTG

CCAGCGCAAAATGCTATAAGTACTACATTCCCTTACACTGGAGATCCCCCATACAGCCAT

GGAACAGGAACAGGGTATACCATGGACACAGTAAACAGAACACATCAATACTCAGAAAAG

GGAAAGTGGACAACAAACACAGAAACCGGAGCACCCCAACTCAACCCAATTGATGGACCA

TTACCAGAGGACAATGAGCCAAGCGGATATGCACAAACTGATTGCGTGTTGGAAGCAATG

GCTTTCCTTGAAGAATCCCACCCAGGGATATTTGAAAACTCTTGTCTTGAAGCGATGGAA

ATCGTTCAGCAAACAAGAGTGGACAAACTAACCCAAGGTCGCCAGACTTATGACTGGACA

CTGAACAGAAACCAACCAGCTGCAACCTCTTTGGCCAACACTATAGAGGTGTTCAGATCG

AATGGTCTGACAGCCAATGAATCAGGGAGACTGATAGATTTTCTCAGGGATGTGATGGAA

TCAATGGATAAAGAAGAGATGGAAGTAACAACACATTTCCAGAGAAAAAGAAGAGTGAGG

GACAACATGACTAAGAAGATGGTCACACAAAGAACAATAGGGAAGAAGAAGCAGAGGCTG

AACAAGAGGAGTTACTTAATAAGAGCACTGACATTGAATACAATGACCAAAGATGCAGAA

AGAGGCAAGTTGAAGAGACGGGCAATTGCAACACCCGGGATGCAGATTAGAGGATTCGTG

TACTTCGTTGAAACACTAGCGAGGAGCATCTGTGAGAAACTAGAGCAATCTGGGCTCCCT

GTTGGAGGGAATGAGAAGAAGGCTAAATTGGCAAATGTTGTGAGAAAAATGATGACTAAC

TCACAAGATACAGAGCTCTCCTTTACAATTACTGGAGACAACACCAAATGGAATGAGAAT

CAAAACCCTCGGATGTTTTTGGCAATGATAACATATATCACAAGAAACCAACCTGAATGG

TTTAGAAATGTCTTAAGCATTGCCCCTATAATGTTCTCAAACAAAATGGCGAGATTAGGG

AAAGGATACATGTTTGAAAGTAAGAGCATGAAGCTAAGAACACAAATACCAGCAGAGATG

CTTACAAATATTGATCTGAAGTATTTCAACGAACCAACGAGAAAGAAAATCGAGAAAATA

AGACCTCTGCTGATTGATGGCACGGCCTCATTGAGTCCTGGGATGATGATGGGCATGTTC

AATATGCTGAGCACAGTATTAGGGGTCTCAATCCTGAATCTCGGGCAAAAGAGGTACACC

AAAACCACATACTGGTGGGATGGACTTCAATCCTCTGATGATTTCGCTCTCATTGTGAAT

GCACCGAATCATGAGGGGATACAAGCAGGAGTGGATAGATTCTATAGGACCTGCAAACTG

GTTGGGATCAACATGAGCAAAAAGAAGTCTTACATAAACCGAACAGGAACATTTGAGTTC

ACAAGTTTTTTCTACCGCTATGGATTTGTAGCTAACTTCAGTATGGAACTACCCAGCTTT

GGAGTGTCTGGAATCAATGAATCAGCTGACATGAGCATTGGAGTTACAGTGATAAAAAAC

AATATGATAAACAATGATCTTGGACCAGCAACAGCTCAAATGGCTCTTCAGTTATTCATC

AAAGACTATAGGTACACATACCGATGCCACAGGGGTGATACACAAATTCAAACGAGGAGA

TCATTCGAGCTGAAGAAGCTGTGGGAGCAGACCCGTTCAAAGGCAGGGCTGTTGATATCA

GACGGGGGGCCAAACCTATATAACATTCGGAATCTCCACATCCCAGAGGTCTGCTTGAAG

TGGGAGCTGATGGATGAAGACTACCAAGGCAGGCTGTGCAATCCTCTGAATCCATTTGTC

AGTCATAAAGAGATTGAGTCCGTAAACAATGCTGTAGTAATGCCCGCCCATGGCCCGGCC

AAGAGCATGGAATATGATGCAGTTGCGACCACACACTCGTGGATTCCTAAGAGGAACCGT

TCCATTCTCAATACCAGCCAAAGGGGAATTCTTGAGGATGAGCAGATGTACCAAAAGTGC

TGTAGTCTATTCGAGAAATTCTTCCCCAGCAGTTCATACAGGAGGCCAGTTGGAATTTCC

AGCATGGTGGAGGCCATGGTGTCTAGGGCCCGAATTGATGCACGCATCGACTTCGAATCT

GGAAGGATTAAGAAAGAAGAGTTTGCTGAGATCATGAAGATCTGTTCCACCATTGAAGAG

CTCAGACGGCAAAAATAGTGAATTTAGCTTGTCCTTCATGAAAAAATGCCTTGTTTCTAC

T

>A_chicken_Chelyabinsk_201_2020_EPI1814335

AGCAAAAGCAGGCAAACTATTTGAATGGATGTCAATCCGACTTTACTTTTCTTAAAAGTG

CCAGCGCAAAATGCTATAAGTACTACATTCCCTTACACTGGAGATCCTCCATACAGCCAT

GGAACAGGAACAGGTTATACCATGGACACAGTAAACAGAACACATCAATACTCAGAAAAG

GGAAAGTGGACAACAAACACAGAAACCGGAGCACCCCAACTCAACCCAATTGATGGACCA

TTACCAGAGGACAATGAGCCAAGCGGATATGCACAAACTGATTGCGTGTTGGAAGCAATG

GCTTTCCTTGAAGAATCCCACCCAGGGATATTTGAAAACTCTTGTCTTGAAGCGATGGAA

ATCGTTCAGCAAACAAGAGTGGACAAACTAACCCAAGGTCGCCAGACTTATGACTGGACA

CTGAACAGAAACCAACCAGCTGCAACCTCTTTGGCCAACACTATAGAGGTGTTCAGATCG

AATGGTCTGACAGCCAATGAATCAGGGAGACTGATAGATTTTCTCAGGGATGTGATGGAA

TCAATGGATAAAGAAGAGATGGAAGTAACAACACATTTCCAGAGAAAAAGAAGAGTGAGG

GACAACATGACTAGGAAGATGGTCACACAAAGAACAATAGGGAAGAAGAAGCAGAGGCTG

AACAAGAGGAGTTACTTAATAAGAGCACTGACATTGAATACAATGACCAAAGATGCAGAA

AGAGGCAAGTTGAAGAGACGGGCAATTGCAACACCCGGGATGCAGATTAGAGGATTCGTG

TACTTCGTTGAAACACTAGCGAGGAGCATCTGTGAGAAACTAGAGCAATCTGGGCTCCCT

GTTGGAGGGAATGAGAAGAAGGCTAAATTGGCAAATGTTGTGAGAAAAATGATGACTAAC

TCACAAGATACAGAGCTCTCCTTTACAATTACTGGAGACAACACCAAATGGAATGAGAAT

CAAAACCCTCGGATGTTTTTGGCAATGATAACATATGTCACAAGAAAACAACCTGAATGG

TTTAGAAATGTCTTAAGCATTGCCCCTATAATGTTCTCAAACAAAATGGCGAGATTAGGG

AAAGGATACATGTTTGAAAGTAAGAGCATGAAGCTAAGAACACAAATACCAGCAGAGATG

CTTACAAATATTGATCTGAAGTATTTCAACGAACCAACGAGAAAGAAAATCGAGAAAATA

AGACCTCTGCTGATTGATGGCACGGCCTCATTGAGTCCTGGGATGATGATGGGCATGTTC

AATATGCTGAGCACAGTATTAGGGGTCTCAATCCTGAATCTCGGGCAAAAAAGGTACACC

AAAACCACATACTGGTGGGATGGACTTCAATCCTCTGATGATTTCGCTCTCATAGTGAAT

GCACCGAATCATGAGGGGATACAAGCAGGAGTGGATAGATTCTATAGGACCTGCAAACTG

GTTGGGATCAACATGAGCAAAAAGAAGTCTTACATAAACCGAACAGGAACATTTGAGTTC

ACAAGTTTTTTCTACCGCTATGGATTTGTAGCTAACTTCAGTATGGAATTACCCAGCTTT

GGAGTGTCTGGAATCAATGAATCAGCTGACATGAGCATTGGAGTTACAGTGATAAAAAAC

AATATGATAAACAATGATCTTGGACCAGCAACAGCTCAAATGGCTCTTCAGTTATTCATC

AAAGACTATAGGTACACATACCGATGCCACAGGGGTGATACACAAATTCAAACGAGGAGA

TCATTCGAGCTGAAGAAGCTGTGGGAGCAGACCCGTTCAAAGGCAGGGCTGTTGATATCA

GACGGGGGGCCAAACCTATACAACATTCGGAATCTCCACATCCCAGAGGTCTGCTTGAAG

TGGGAGCTGATGGATGAAGACTACCAAGGCAGGCTGTGCAATCCTCTGAATCCATTTGTC

AGTCATAAAGAGATTGAGTCCGTAAACAATGCTGTAGTAATGCACGCCCATGGCCCGGCC

AAGAGCATGGAATATGATGCTGTTGCGACCACACACTCGTGGATTCCTAAGAGGAACCGT

TCCATTCTCAATACCAGCCAAAGGGGAATTCTTGAGGATGAGCAGATGTACCAAAAGTGC

TGTAGTCTATTCGAGAAATTCTTCCCCAGCAGTTCATACAGGAGGCCAGTTGGAATTTCC

AGCATGGTGGAGGCCATGGTGTCTAGGGCCCGAATTGATGCACGCATCGACTTCGAATCT

GGAAGGATTAAGAAAGAAGAGTTTGCTGAGATCATGAAGATCTGTTCCACCATTGAAGAG

CTCAGACGGCAAAAATAGTGAATTTAGCTTGTCCTTCATGAAAAAATGCCTTGTTTCTAC

T

>A_chicken_Kurgan_1005_2020_EPI1814367

AGCAAAAGCAGGCAAACTATTTGAATGGATGTCAATCCGACTTTACTTTTCTTAAAAGTG

CCAGCGCAAAATGCTATAAGTACTACATTCCCTTACACTGGAGATCCTCCATACAGCCAT

GGAACAGGAACAGGTTATACCATGGACACAGTAAACAGAACACATCAATACTCAGAAAAG

GGAAAGTGGACAACAAACACAGAAACCGGAGCACCCCAACTCAACCCAATTGATGGACCA

TTACCAGAGGACAATGAGCCAAGCGGATATGCACAAACTGATTGCGTGTTGGAAGCAATG

GCTTTCCTTGAAGAATCCCACCCAGGGATATTTGAAAACTCTTGTCTTGAAGCGATGGAA

ATCGTTCAGCAAACAAGAGTGGACAAACTAACCCAAGGTCGCCAGACTTATGACTGGACA

CTGAACAGGAACCAACCAGCTGCAACCTCTTTGGCCAACACTATAGAGGTGTTCAGATCG

AATGGTCTGACAGCCAATGAATCAGGGAGACTGATAGATTTTCTCAGGGATGTGATGGAA

TCAATGGATAAAGAAGAGATGGAAGTAACAACACATTTCCAGAGAAAAAGAAGAGTGAGG

GACAACATGACTAGGAAGATGGTCACACAAAGAACAATAGGGAAGAAGAAGCAGAGGCTG

AACAAGAGGAGTTACCTAATAAGAGCACTGACATTGAATACAATGACCAAAGATGCAGAA

AGAGGCAAGTTGAAGAGACGGGCAATTGCAACACCCGGGATGCAGATTAGAGGATTCGTG

TACTTCGTTGAAACACTAGCGAGGAGCATCTGTGAGAAACTAGAGCAATCTGGGCTCCCT

GTTGGAGGGAATGAGAAGAAGGCTAAATTGGCAAATGTTGTGAGAAAAATGATGACTAAC

TCACAAGATACAGAGCTCTCCTTTACAATTACTGGAGACAACACCAAATGGAATGAGAAT

CAAAACCCTCGGATGTTTTTGGCAATGATAACATATGTCACAAGAAACCAACCTGAATGG

TTTAGAAATGTCTTAAGCATTGCCCCTATAATGTTCTCAAACAAAATGGCGAGATTAGGG

AAAGGATACATGTTTGAAAGTAAGAGCATGAAGCTAAGAACACAAATACCAGCAGAGATG

CTTACAAATATTGATCTGAAGTATTTCAACGAACCAACGAGAAAGAAAATCGAGAAAATA

AGACCTCTGCTGATTGATGGCACGGCCTCATTGAGTCCTGGGATGATGATGGGCATGTTC

AATATGCTGAGCACAGTATTAGGGGTCTCAATCCTGAATCTCGGGCAAAAAAGGTACACC

AAAACCACATACTGGTGGGATGGACTTCAATCCTCTGATGATTTCGCTCTCATAGTGAAT

GCACCGAATCATGAGGGGATACAAGCAGGAGTGGATAGATTCTATAGGACCTGCAAACTG

GTTGGGATCAACATGAGCAAAAAGAAGTCTTACATAAACCGAACAGGAACATTTGAGTTC

ACAAGTTTTTTCTACCGCTATGGATTTGTAGCTAACTTCAGTATGGAATTACCCAGCTTT

GGAGTGTCTGGAATCAATGAATCAGCTGACATGAGCATTGGAGTTACAGTGATAAAAAAC

AATATGATAAACAATGATCTTGGACCAGCAACAGCTCAAATGGCTCTTCAGTTATTCATC

AAAGACTATAGGTACACATACCGATGCCACAGGGGTGATACACAAATTCAAACGAGGAGA

TCATTCGAGCTGAAGAAGCTGTGGGAGCAGACCCGTTCAAAGGCAGGGCTGTTGATATCA

GACGGGGGGCCAAACCTATACAACATTCGGAATCTCCACATCCCAGAGGTCTGCTTGAAG

TGGGAGCTGATGGATGAAGACTACCAAGGCAGGCTGTGCAATCCTCTGAATCCATTTGTC

AGTCATAAAGAGATTGAGTCCGTAAACAATGCTGTAGTAATGCCCGCCCATGGCCCGGCC

AAGAGCATGGAATATGATGCTGTTGCGACCACACACTCGTGGATTCCTAAGAGGAACCGT

TCCATTCTCAATACCAGCCAAAGGGGAATTCTTGAGGATGAGCAGATGTATCAAAAGTGC

TGTAGTCTATTCGAGAAATTCTTCCCCAGCAGTTCATACAGGAGGCCAGTTGGAATTTCC

AGCATGGTGGAGGCCATGGTGTCTAGGGCCCGAATTGATGCACGCATCGACTTCGAATCT

GGAAGGATTAAGAAAGAAGAGTTTGCTGAGATCATGAAGATCTGTTCCACCATTGAAGAG

CTCAGACGGCAAAAATAGTGAATTTAGCTTGTCCTTCATGAAAAAATGCCTTGTTTCTAC

T

>A_turkey_Poland_464_2020_EPI1841308

------------CAAACTATTTGAATGGATGTCAATCCGACTTTACTTTTCTTAAAAGTG

CCAGCGCAAAATGCTATAAGTACTACATTCCCTTACACTGGAGATCCTCCATACAGCCAT

GGAACAGGAACAGGGTATACCATGGACACAGTAAACAGAACACATCAATACTCAGAAAAG

GGAAAGTGGACAACAAACACAGAAACCGGAGCACCCCAACTCAACCCAATTGATGGACCA

TTACCAGAGGACAATGAGCCAAGCGGATATGCACAAACTGATTGCGTGTTGGAAGCAATG

GCTTTCCTTGAAGAATCCCACCCAGGGATATTTGAAAACTCTTGTCTTGAAGCAATGGAA

ATCGTTCAGCAAACAAGAGTGGACAAACTAACCCAAGGTCGCCAGACTTATGACTGGACA

CTGAACAGAAACCAACCAGCTGCAACCTCTTTGGCCAACACTATAGAGGTGTTCAGATCG

AATGGTCTGACAGCCAATGAATCAGGGAGACTGATAGATTTTCTCAGGGATGTGATGGAA

TCAATGGATAAAGAAGAGATGGAAGTAACAACACATTTCCAGAGAAAAAGAAGAGTGAGG

GACAACATGACTAAGAAGATGGTCACACAAAGAACAATAGGGAAGAAGAAGCAGAGGCTG

AACAAGAGGAGTTACTTAATAAGAGCACTGACATTGAATACAATGACCAAAGATGCAGAA

AGAGGCAAGTTGAAGAGACGGGCAATTGCAACACCCGGGATGCAGATTAGAGGATTCGTG

TACTTCGTTGAAACACTAGCGAGGAGCATCTGTGAGAAACTAGAGCAATCTGGGCTCCCT

GTTGGAGGGAATGAGAAGAAGGCTAAATTGGCAAATGTTGTGAGAAAAATGATGACTAAC

TCACAAGATACAGAGCTCTCCTTTACAATTACTGGAGACAACACCAAATGGAATGAGAAT

CAAAACCCTCGGATGTTTTTGGCAATGATAACATATATCACAAGAAACCAACCTGAATGG

TTTAGAAATGTCTTAAGCATTGCCCCTATAATGTTCTCAAACAAAATGGCGAGATTAGGG

AAAGGATACATGTTTGAAAGTAAGAGCATGAAGCTAAGAACACAAATACCAGCAGAGATG

CTTACAAATATTGATCTGAAGTATTTCAACGAACCAACGAGAAAGAAAATCGAGAAAATA

AGACCTCTGCTGATTGATGGCACGGCCTCATTGAGTCCTGGGATGATGATGGGCATGTTC

AATATGCTGAGCACAGTATTGGGGGTCTCAATCCTGAATCTCGGGCAAAAGAGGTACACC

AAAACCACATACTGGTGGGATGGACTTCAATCCTCTGATGATTTCGCTCTCATAGTGAAT

GCACCGAATCATGAGGGGATACAAGCAGGAGTGGATAGATTCTATAGGACCTGCAAACTG

GTTGGGATCAACATGAGCAAAAAGAAGTCTTACATAAACCGAACAGGAACATTTGAGTTC

ACAAGTTTTTTCTACCGCTATGGATTTGTAGCTAACTTCAGTATGGAATTACCCAGCTTT

GGAGTGTCTGGAATCAATGAATCAGCTGACATGAGCATTGGAGTTACAGTGATAAAAAAC

AATATGATAAACAATGATCTTGGACCAGCGACAGCTCAAATGGCTCTTCAGTTATTCATC

AAAGACTATAGGTACACATACCGATGCCACAGGGGTGATACACAAATTCAAACGAGGAGA

TCATTYGAGCTGAAGAAGCTGTGGGAGCAGACCCGTTCAAAGGCAGGGCTGTTGATATCA

GACGGGGGGCCAAACCTATACAACATTCGGAACCTCCACATCCCAGAGGTCTGCTTGAAG

TGGGGGCTGATGGATGAAGACTACCAAGGCAGGCTGTGCAATCCTCTGAATCCATTTGTC

AGTCATAAAGAGATTGAGTCCGTAAACAATGCTGTAGTAATGCCCGCCCATGGCCCGGCC

AAGAGCATGGAATATGATGCTGTTGCGACCACACATTCGTGGATTCCTAAGAGGAACCGG

TCCATTCTCAATACCAGCCAAAGGGGAATTCTTGAGGATGAGCAGATGTACCAAAAGTGC

TGTAGTCTATTCGAGAAATTCTTCCCCAGCAGTTCATACAGGAGGCCAGTTGGAATTTCC

AGCATGGTGGAGGCCATGGTGTCTAGGGCCCGAATTGATGCACGCATCGACTTCGAATCT

GGAAGGATTAAGAAAGAAGAGTTTGCTGAGATCATGAAGATCTGTTCCACCATTGAAGAG

CTCAGACGGCAAAAATAGTGAATTTAGCTTGTCCTTCATGAAAAAATGC-----------

-

>A_duck_Northern_China_ZGL_2020_EPI1844089

------------------------ATGGATGTCAATCCGACTTTACTTTTCTTAAAAGTG

CCAGCGCAAAATGCTATAAGTACTACATTCCCTTACACTGGAGATCCTCCATACAGCCAT

GGAACAGGAACAGGGTATACCATGGACACAGTAAACAGAACACATCAATACTCAGAAAAG

GGAAAGTGGACAACAAACACAGAAACCGGAGCACCCCAACTCAACCCAATTGATGGACCA

TTACCAGAGGACAATGAGCCAAGCGGATATGCACAAACTGATTGCGTGTTGGAAGCAATG

GCTTTCCTTGAAGAATCCCACCCAGGGATATTTGAAAACTCTTGTCTTGAAGCGATGGAA

ATCGTTCAGCAAACAAGAGTGGACAAACTAACCCAAGGTCGCCAGACTTATGACTGGACA

CTGAACAGAAACCAACCAGCTGCAACCTCTTTGGCCAACACTATAGAGGTGTTCAGATCG

AATGGTCTGACAGCCAATGAATCAGGGAGACTGATAGATTTTCTCAGGGATGTGATGGAA

TCAATGGATAAAGAAGAGATGGAAGTGACAACACATTTCCAGAGAAAAAGAAGAGTGAGG

GACAACATGACTAAGAAGATGGTCACACAAAGAACAATAGGGAAGAAGAAGCAGAGGCTG

AACAAGAGGAGTTACTTGATAAGAGCACTGACATTGAATACAATGACCAAAGATGCAGAA

AGAGGCAAGTTGAAGAGACGGGCAATTGCAACACCCGGGATGCAGATTAGAGGATTCGTG

TACTTCGTTGAAACACTAGCGAGAAGCATCTGTGAGAAACTAGAGCAATCTGGACTCCCT

GTTGGAGGGAATGAGAAGAAGGCTAAATTGGCAAATGTTGTGAGAAAAATGATGACTAAC

TCACAAGATACAGAGCTCTCCTTTACAATTACTGGAGACAACACCAAATGGAATGAGAAT

CAAAACCCTCGGATGTTTTTGGCAATGATAACATATATCACAAGAAACCAACCTGAATGG

TTTAGAAATGTCTTAAGCATTGCCCCTATAATGTTCTCAAACAAAATGGCGAGATTAGGG

AAAGGATACATGTTTGAAAGTAAGAGCATGAAGCTAAGAACACAAATACCAGCAGAGATG

CTTACAAATATTGATCTGAAGTATTTCAACGAACCAACGAGAAAGAAAATCGAGAAAATA

AGACCTCTGCTGATTGATGGCACGGCCTCATTGAGTCCTGGGATGATGATGGGCATGTTC

AATATGCTGAGCACAGTATTGGGGGTCTCAATCCTGAATCTCGGGCAAAAGAGGTACACC

AAAACCACATACTGGTGGGATGGACTTCAATCCTCTGATGATTTCGCTCTCATAGTGAAT

GCACCGAATCATGAGGGGATACAAGCAGGAGTGGATAGATTCTATAGGACCTGCAAACTG

GTTGGGATCAACATGAGCAAAAAGAAGTCTTACATAAACCGAACAGGAACATTTGAGTTC

ACAAGTTTTTTCTACCGCTATGGATTTGTAGCTAACTTCAGTATGGAATTACCCAGCTTT

GGAGTGTCTGGAATCAATGAATCAGCTGACATGAGCATTGGAGTTACAGTGATAAAAAAC

AATATGATAAACAATGATCTTGGACCAGCAACAGCTCAAATGGCTCTTCAGTTATTCATC

AAAGACTATAGGTACACATACCGATGCCACAGGGGTGATACACAAATTCAAACGAGGAGA

TCATTCGAGCTGAAGAAGCTGTGGGAGCAGACCCGTTCAAAGGCAGGGCTGTTGATATCA

GACGGGGGGCCAAACCTATACAACATTCGGAACCTCCACATCCCAGAGGTCTGCTTGAAG

TGGGAGCTGATGGATGAAGACTACCAAGGCAGGCTGTGCAATCCTCTGAATCCATTTGTC

AGTCATAAAGAGATTGAGTCCGTAAACAATGCTGTAGTAATGCCCGCCCATGGCCCGGCC

AAGAGCATGGAATATGATGCTGTTGCGACCACACATTCGTGGATTCCTAAGAGGAACCGC

TCCATTCTCAATACCAGCCAAAGGGGAATTCTTGAGGATGAGCAGATGTACCAAAAGTGC

TGTAGTCTATTCGAGAAATTCTTCCCCAGCAGTTCATACAGGAGGCCAGTTGGAATTTCC

AGCATGGTGGAGGCCATGGTGTCTAGGGCCCGAATTGATGCACGCATCGACTTCGAATCT

GGAAGGATTAAGAAAGAAGAGTTTGCTGAGATCATGAAGATCTGTTCCACCATTGAAGAG

CTCAGACGGCAAAAATAG------------------------------------------

-

>A_duck_Southwestern_China_B1904_2020_EPI1844097

------------------------ATGGATGTCAATCCGACTTTACTTTTCTTAAAAGTG

CCAGCGCAAAATGCTATAAGTACTACATTCCCTTACACTGGAGATCCTCCATACAGCCAT

GGAACAGGAACAGGGTATACCATGGACACAGTAAACAGAACACATCAATACTCAGAAAAG

GGAAAGTGGACAACAAACACAGAAACCGGAGCACCTCAACTCAACCCAATTGATGGACCA

TTACCAGAGGACAATGAGCCAAGCGGATATGCACAAACTGATTGCGTGTTGGAAGCAATG

GCTTTCCTTGAAGAATCCCACCCAGGGATATTTGAAAACTCTTGTCTCGAAGCGATGGAA

ATCGTTCAGCAAACAAGAGTGGACAAACTAACCCAAGGTCGCCAGACTTATGACTGGACA

CTGAACAGAAACCAACCAGCTGCAACCTCTTTGGCCAACACTATAGAGGTGTTCAGATCG

AATGGTCTGACAGCCAATGAATCAGGGAGACTGATAGATTTTCTCAAGGATGTGATGGAA

TCAATGGATAAAGAAGAGATGGAAGTAACAACACATTTCCAGAGAAAAAGAAGAGTGAGG

GACAACATGACTAAGAAGATGGTCACACAAAGAACAATAGGGAAGAAGAAGCAGAGGCTG

AACAAGAGGAGTTACTTGATAAGAGCACTGACATTGAATACAATGACCAAAGATGCAGAA

AGAGGCAAATTGAAGAGACGGGCAATTGCAACACCCGGGATGCAGATTAGAGGATTCGTG

TACTTCGTTGAAACATTAGCGAGGAGCATCTGTGAGAAACTAGAGCAATCTGGGCTCCCT

GTTGGAGGGAATGAGAAAAAGGCTAAATTGGCAAATGTTGTGAGAAAAATGATGACTAAC

TCACAAGATACAGAGCTCTCCTTTACAATTACTGGAGACAACACCAAATGGAATGAGAAT

CAAAACCCTCGGATGTTTTTGGCAATGATAACATATATCACAAGAAACCAACCTGAATGG

TTTAGAAATGTCTTAAGCATTGCCCCTATAATGTTCTCAAACAAAATGGCGAGATTAGGG

AAAGGATACATGTTTGAAAGTAAGAGCATGAAGCTAAGAACACAAATACCAGCAGAGATG

CTTACAAATATTGATCTGAAGTATTTCAACGAACCAACGAGAAAGAAAATCGAGAAAATA

AGACCTCTGCTGATTGATGGCACGGCCTCATTGAGTCCTGGGATGATGATGGGCATGTTC

AATATGCTGAGCACAGTATTGGGGGTCTCAATCCTGAATCTCGGGCAAAAGAGGTACACC

AAAACCACATACTGGTGGGATGGACTTCAATCCTCTGATGATTTCGCTCTCATAGTGAAT

GCACCGAATCATGAGGGGATACAAGCAGGAGTGGATAGATTCTATAGGACCTGCAAACTG

GTTGGGATCAACATGAGCAAAAAGAAGTCTTACATAAACCGAACAGGAACATTTGAGTTC

ACAAGTTTTTTCTACCGCTATGGATTTGTAGCTAACTTCAGTATGGAATTACCCAGCTTT

GGAGTGTCTGGAATCAATGAATCAGCTGACATGAGCATTGGAGTTACAGTGATAAAAAAC

AATATGATAAACAATGATCTTGGACCAGCGACAGCTCAAATGGCTCTTCAGTTATTCATC

AAAGACTATAGGTACACATACCGGTGCCACAGGGGTGATACACAAATTCAAACGAGGAGA

TCATTCGAGCTGAAGAAGCTGTGGGAGCAGACCCGTTCAAAGGCAGGGCTGTTGATATCA

GACGGGGGGCCAAACCTATACAACATTCGGAACCTCCACATCCCAGAGGTCTGCTTGAAG

TGGGAGCTGATGGATGAAGACTACCAAGGCAGGCTGTGCAATCCTCTGAATCCATTTGTC

AGTCATAAAGAGATTGAGTCCGTAAACAATGCTGTAGTAATGCCCGCCCATGGCCCGGCC

AAGAGCATGGAATATGATGCTGTTGCGACCACACATTCGTGGATCCCTAAGAGGAACCGC

TCCATTCTCAATACCAGCCAAAGGGGAATTCTTGAGGATGAGCAGATGTACCAAAAGTGC

TGTAGTCTATTCGAGAAATTCTTCCCCAGCAGTTCATACAGGAGGCCAGTTGGAATTTCC

AGCATGGTGGAGGCCATGGTGTCTAGGGCCCGAATTGATGCACGCATCGACTTCGAATCT

GGCAGGATTAAGAAAGAAGAGTTTGCTGAGATCATGAAGATCTGTTCCACCATTGAAGAG

CTCAGACGGCAAAAATAG------------------------------------------

-

>A_duck_Korea_H411_2020_EPI1845935

------------------------ATGGATGTCAATCCGACTTTACTTTTCTTAAAAGTG

CCAGCGCAAAATGCTATAAGTACTACATTCCCTTACACTGGAGATCCTCCATACAGCCAT

GGAACAGGAACAGGGTATACCATGGACACAGTAAACAGAACACATCAATACTCAGAAAAG

GGAAAGTGGACAACAAACACAGAAACCGGAGCACCCCAACTCAACCCAATTGATGGACCA

TTACCAGAGGACAATGAGCCAAGCGGATATGCACAAACTGATTGCGTGTTGGAAGCAATG

GCTTTCCTTGAAGAATCCCACCCAGGGATATTTGAAAACTCTTGTCTTGAAGCGATGGAA

ATCGTTCAGCAAACAAGAGTGGACAAACTAACCCAAGGTCGCCAGACTTATGACTGGACA

CTGAACAGAAACCAACCAGCTGCAACCTCTTTGGCCAACACTATAGAGGTGTTCAGATCG

AATGGTCTGACAGCCAATGAATCAGGGAGACTGATAGATTTTCTCAGGGATGTGATGGAA

TCAATGAATAAAGAAGAGATGGAAGTAACAACACATTTCCAGAGAAAAAGAAGAGTGAGG

GACAACATGACTAAGAAGATGGTCACACAAAGAACAATAGGGAAGAAGAAGCAGAGGCTG

AACAAGAGGAGTTACTTGATAAGAGCACTGACATTGAATACAATGACCAAAGATGCAGAA

AGAGGCAAGTTGAAAAGACGGGCAATTGCAACACCCGGGATGCAGATTAGAGGATTCGTG

TACTTCGTTGAAACACTAGCGAGGAGCATCTGTGAGAAACTAGAGCAATCTGGGCTCCCT

GTTGGAGGGAATGAGAAGAAGGCTAAATTGGCAAATGTTGTGAGAAAAATGATGACTAAC

TCACAAGATACAGAGCTCTCCTTTACAATTACTGGAGACAACACCAAATGGAATGAGAAT

CAAAACCCTCGGATGTTTTTGGCAATGATAACATATATCACAAGGAACCAACCTGAATGG

TTTAGAAATGTCTTAAGCATTGCCCCTATAATGTTCTCAAACAAAATGGCGAGATTAGGG

AAAGGATACATGTTTGAAAGTAAGAGCATGAAGCTAAGAACACAAATACCAGCAGAGATG

CTTACAAATATTGATCTGAAGTATTTCAACGAATCAACGAGAAAGAAAATCGAGAAAATA

AGACCTCTGCTGATTGATGGCACGGCCTCATTGAGTCCTGGGATGATGATGGGCATGTTC

AATATGCTGAGCACAGTATTGGGGGTCTCAATCCTGAATCTCGGGCAAAAGAGGTACACC

AAAACCACATACTGGTGGGATGGACTTCAATCCTCTGATGATTTCGCTCTCATAGTGAAT

GCACCGAATCATGAGGGGATACAAGCAGGAGTGGATAGATTCTATAGGACCTGCAAACTG

GTTGGGATCAACATGAGCAAAAAGAAGTCTTACATAAACCGAACAGGAACATTTGAGTTC

ACAAGTTTTTTCTACCGCTATGGATTTGTAGCTAACTTCAGTATGGAATTACCCAGCTTT

GGAGTGTCTGGAATCAATGAATCAGCTGACATGAGCATTGGAGTTACAGTGATAAAAAAC

AATATGATAAACAATGATCTTGGACCAGCAACAGCTCAAATGGCTCTTCAATTATTCATC

AAAGACTATAGGTACACATACCGATGCCACAGGGGTGATACACAAATTCAAACGAGGAGA

TCATTCGAGCTGAAGAAGCTGTGGGAGCAGACCCGTTCAAAGGCAGGGCTGTTGATATCA

GACGGGGGGCCAAACCTATACAACATTCGGAACCTCCACATCCCAGAGGTCTGCTTGAAG

TGGGAGCTGATGGATGAAGACTACCAAGGCAGGCTGTGCAATCCTCTGAATCCATTTGTC

AGTCATAAAGAGATTGAGTCCGTAAACAATGCTGTAGTAATGCCCGCCCATGGCCCGGCC

AAGAGCATGGAATATGATGCTGTTGCGACCACACATTCGTGGATTCCTAAGAGGAACCGC

TCCATTCTCAATACCAGCCAAAGGGGAATTCTTGAGGATGAGCAGATGTACCAAAAGTGC

TGTAGTCTATTCGAGAAATTCTTCCCCAGCAGTTCATACAGGAGGCCAGTTGGAATTTCC

AGCATGGTGGAGGCCATGGTGTCTAGGGCCCGAATTGATGCACGCATCGACTTCGAATCT

GGAAGGATTAAGAAAGAAGAGTTTGCTGAGATCATGAAGATCTGTTCCACCATTGAAGAG

CTCAGACGGCAAAAATAG------------------------------------------

-

>A_duck_Korea_H431_2020_EPI1845959

------------------------ATGGATGTCAATCCGACTTTACTTTTCTTAAAAGTG

CCAGCGCAAAATGCTATAAGTACTACATTCCCTTACACTGGAGATCCTCCATACAGCCAT

GGAACAGGAACAGGGTATACCATGGACACAGTAAACAGAACACATCAATACTCAGAAAAG

GGAAAGTGGACAACAAACACAGAAACCGGAGCACCCCAACTCAACCCAATTGATGGACCA

TTACCAGAGGACAATGAGCCAAGCGGATATGCACAAACTGATTGCGTGTTGGAAGCAATG

GCTTTCCTTGAAGAATCCCACCCAGGGATATTTGAAAACTCTTGTCTTGAAGCGATGGAA

ATCGTTCAGCAAACAAGAGTGGACAAACTAACCCAAGGTCGCCAGACTTATGACTGGACA

CTGAACAGAAACCAACCAGCTGCAACCTCTTTGGCCAACACTATAGAGGTGTTCAGATCG

AATGGTCTGACAGCCAATGAATCAGGGAGACTGATAGATTTTCTCAGGGATGTGATGGAA

TCAATGAATAAAGAAGAGATGGAAGTAACAACACATTTCCAGAGAAAAAGAAGAGTGAGG

GACAACATGACTAAGAAGATGGTCACACAAAGAACAATAGGGAAGAAGAAGCAGAGGCTG

AACAAGAGGAGTTACTTGATAAGAGCACTGACATTGAATACAATGACCAAAGATGCAGAA

AGAGGCAAGTTGAAAAGACGGGCAATTGCAACACCCGGGATGCAGATTAGAGGATTCGTG

TACTTCGTTGAAACACTAGCGAGGAGCATCTGTGAGAAACTAGAGCAATCTGGGCTCCCT

GTTGGAGGGAATGAGAAGAAGGCTAAATTGGCAAATGTTGTGAGAAAAATGATGACTAAC

TCACAAGATACAGAGCTCTCCTTTACAATTACTGGAGACAACACCAAATGGAATGAGAAT

CAAAACCCTCGGATGTTTTTGGCAATGATAACATATATCACAAGGAACCAACCTGAATGG

TTTAGAAATGTCTTAAGCATTGCCCCTATAATGTTCTCAAACAAAATGGCGAGATTAGGG

AAAGGATACATGTTTGAAAGTAAGAGCATGAAGCTAAGAACACAAATACCAGCAGAGATG

CTTACAAATATTGATCTGAAGTATTTCAACGAATCAACGAGAAAGAAAATCGAGAAAATA

AGACCTCTGCTGATTGATGGCACGGCCTCATTGAGTCCTGGGATGATGATGGGCATGTTC

AATATGCTGAGCACAGTATTGGGGGTCTCAATCCTGAATCTCGGGCAAAAGAGGTACACC

AAAACCACATACTGGTGGGATGGACTTCAATCCTCTGATGATTTCGCTCTCATAGTGAAT

GCACCGAATCATGAGGGGATACAAGCAGGAGTGGATAGATTCTATAGGACCTGCAAACTG

GTTGGGATCAACATGAGCAAAAAGAAGTCTTACATAAACCGAACAGGAACATTTGAGTTC

ACAAGTTTTTTCTACCGCTATGGATTTGTAGCTAACTTCAGTATGGAATTACCCAGCTTT

GGAGTGTCTGGAATCAATGAATCAGCTGACATGAGCATTGGAGTTACAGTGATAAAAAAC

AATATGATAAACAATGATCTTGGACCAGCAACAGCTCAAATGGCTCTTCAATTATTCATC

AAAGACTATAGGTACACATACCGATGCCACAGGGGTGATACACAAATTCAAACGAGGAGA

TCATTCGAGCTGAAGAAGCTGTGGGAGCAGACCCGTTCAAAGGCAGGGCTGTTGATATCA

GACGGGGGGCCAAACCTATACAACATTCGGAACCTCCACATCCCAGAGGTCTGCTTGAAG

TGGGAGCTGATGGATGAAGACTACCAAGGCAGGCTGTGCAATCCTCTGAATCCATTTGTC

AGTCATAAAGAGATTGAGTCCGTAAACAGTGCTGTAGTAATGCCCGCCCATGGCCCGGCC

AAGAGCATGGAATATGATGCTGTTGCGACCACACATTCGTGGATTCCTAAGAGGAACCGC

TCCATTCTCAATACCAGCCAAAGGGGAATTCTTGAGGATGAGCAGATGTACCAAAAGTGC

TGTAGTCTATTCGAGAAATTCTTCCCCAGCAGTTCATACAGGAGGCCAGTTGGAATTTCC

AGCATGGTGGAGGCCATGGTGTCTAGGGCCCGAATTGATGCACGCATCGACTTCGAATCT

GGAAGGATTAAGAAAGAAGAGTTTGCTGAGATCATGAAGATCTGTTCCACCATTGAAGAG

CTCAGACGGCAAAAATAG------------------------------------------

-

>A_duck_Korea_H471_2020_EPI1846031

------------------------ATGGATGTCAATCCGACTTTACTTTTCTTAAAAGTG

CCAGCGCAAAATGCTATAAGTACTACATTCCCTTACACTGGAGATCCTCCATACAGCCAT

GGAACAGGAACAGGGTATACCATGGACACAGTAAACAGAACACATCAATACTCAGAAAAG

GGAAAGTGGACAACAAACACAGAAACCGGAGCACCCCAACTCAACCCAATTGATGGACCA

TTACCAGAGGACAATGAGCCAAGCGGATATGCACAAACTGATTGCGTGTTGGAAGCAATG

GCTTTCCTTGAAGAATCCCACCCAGGGATATTTGAAAACTCTTGTCTTGAAGCGATGGAA

ATCGTTCAGCAAACAAGAGTGGACAAACTAACCCAAGGTCGCCAGACTTATGACTGGACA

CTGAACAGAAACCAACCAGCTGCAACCTCTTTGGCCAACACTATAGAGGTGTTCAGATCG

AATGGTCTGACAGCCAATGAATCAGGGAGACTGATAGATTTTCTCAGGGATGTGATGGAA

TCAATGAATAAAGAAGAGATGGAAGTAACAACACATTTCCAGAGAAAAAGAAGAGTGAGG

GACAACATGACTAAGAAGATGGTCACACAAAGAACAATAGGGAAGAAGAAGCAGAGGCTG

AACAAGAGGAGTTACTTGATAAGAGCACTGACATTGAATACAATGACCAAAGATGCAGAA

AGAGGCAAGTTGAAAAGACGGGCAATTGCAACACCCGGGATGCAGATTAGAGGATTCGTG

TACTTCGTTGAAACACTAGCGAGGAGCATCTGTGAGAAACTAGAGCAATCTGGGCTCCCT

GTTGGAGGGAATGAGAAGAAGGCTAAATTGGCAAATGTTGTGAGAAAAATGATGACTAAC

TCACAAGATACAGAGCTCTCCTTTACAATTACTGGAGACAACACCAAATGGAATGAGAAT

CAAAACCCTCGGATGTTTTTGGCAATGATAACATATATCACAAGGAACCAACCTGAATGG

TTTAGAAATGTCTTAAGCATTGCCCCTATAATGTTCTCAAACAAAATGGCGAGATTAGGG

AAAGGATACATGTTTGAAAGTAAGAGCATGAAGCTAAGAACACAAATACCAGCAGAGATG

CTTACAAATATTGATCTGAAGTATTTCAACGAATCAACGAGAAAGAAAATCGAGAAATTA

AGACCTCTGCTGATTGATGGCACGGCCTCATTGAGTCCTGGGATGATGATGGGCATGTTC

AATATGCTGAGCACAGTATTGGGGGTCTCAATCCTGAATCTCGGGCAAAAGAGGTACACC

AAAACCACATACTGGTGGGATGGACTTCAATCCTCTGATGATTTCGCTCTCATAGTGAAT

GCACCGAATCATGAGGGGATACAAGCAGGAGTGGATAGATTCTATAGGACCTGCAAACTG

GTTGGGATCAACATGAGCAAAAAGAAGTCTTACATAAACCGAACAGGAACATTTGAGTTC

ACAAGTTTTTTCTACCGCTATGGATTTGTAGCTAACTTCAGTATGGAATTACCCAGCTTT

GGAGTGTCTGGAATCAATGAATCAGCTGACATGAGCATTGGAGTTACAGTGATAAAAAAC

AATATGATAAACAATGATCTTGGACCAGCAACAGCTCAAATGGCTCTTCAATTATTCATC

AAAGACTATAGGTACACATACCGATGCCACAGGGGTGATACACAAATTCAAACGAGGAGA

TCATTCGAGCTGAAGAAGCTGTGGGAGCAGACCCGTTCAAAGGCAGGGCTGTTGATATCA

GACGGGGGGCCAAACCTATACAACATTCGGAACCTCCACATCCCAGAGGTCTGCTTGAAG

TGGGAGCTGATGGATGAAGACTACCAAGGCAGGCTGTGCAATCCTCTGAATCCATTTGTC

AGTCATAAAGAGATTGAGTCCGTAAACAGTGCTGTAGTAATGCCCGCCCATGGCCCGGCC

AAGAGCATGGAATATGATGCTGTTGCGACCACACATTCGTGGATTCCTAAGAGGAACCGC

TCCATTCTCAATACCAGCCAAAGGGGAATTCTTGAGGATGAGCAGATGTACCAAAAGTGC

TGTAGTCTATTCGAGAAATTCTTCCCCAGCAGTTCATACAGGAGGCCAGTTGGAATTTCC

AGCATGGTGGAGGCCATGGTGTCTAGGGCCCGAATTGATGCACGCATCGACTTCGAATCT

GGAAGGATTAAGAAAGAAGAGTTTGCTGAGATCATGAAGATCTGTTCCACCATTGAAGAG

CTCAGACGGCAAAAATAG------------------------------------------

-

>A_chicken_Korea_H510_2020_EPI1846055

------------------------ATGGATGTCAATCCGACTTTACTTTTCTTAAAAGTG

CCAGCGCAAAATGCTATAAGTACTACATTCCCTTACACTGGAGATCCTCCATACAGCCAT

GGAACAGGAACAGGGTATACCATGGACACAGTAAACAGAACACATCAATACTCAGAAAAG

GGAAAGTGGACAACAAACACAGAAACCGGAGCACCCCAACTCAACCCAATTGATGGACCA

TTACCAGAGGACAATGAGCCAAGCGGATATGCACAAACTGATTGCGTGTTGGAAGCAATG

GCTTTCCTTGAAGAATCCCACCCAGGGATATTTGAAAACTCTTGTCTTGAAGCGATGGAA

ATCGTTCAGCAAACAAGAGTGGACAAACTAACCCAAGGTCGCCAGACTTATGACTGGACA

CTGAACAGAAACCAACCAGCTGCAACCTCTTTGGCCAACACTATAGAGGTGTTCAGATCG

AATGGTCTGACAGCCAATGAATCAGGGAGACTGATAGATTTTCTCAGGGATGTGATGGAA

TCAATGGATAAAGAAGAGATGGAAGTAACAACACATTTCCAGAGAAAAAGAAGAGTGAGG

GACAACATGACTAAGAAGATGGTCACACAAAGAACAATAGGGAAGAAGAAGCAGAGGCTG

AACAAGAGGAGTTACTTGATAAGAGCACTGACATTGAATACAATGACCAAAGATGCAGAA

AGAGGCAAGTTGAAGAGACGGGCAATTGCAACACCCGGGATGCAGATTAGAGGATTCGTG

TACTTCGTTGAAACACTAGCGAGGAGCATCTGTGAGAAACTAGAGCAATCTGGGCTCCCT

GTTGGAGGGAATGAGAAGAAGGCTAAATTGGCAAATGTTGTGAGAAAAATGATGACTAAC

TCACAAGATACAGAGCTCTCCTTTACAATTACTGGAGACAACACCAAATGGAATGAGAAT

CAAAACCCTCGGATGTTTTTGGCAATGATAACATATATCACAAGGAACCAACCTGAATGG

TTTAGAAATGTCTTAAGCATTGCCCCTATAATGTTCTCAAACAAAATGGCGAGATTAGGG

AAAGGATACATGTTTGAAAGTAAGAGCATGAAGCTAAGAACACAAATACCAGCAGAGATG

CTTACAAATATTGATCTGAAGTATTTCAACGAATCAACGAGAAAGAAAATCGAGAAAATA

AGACCTCTGCTGATTGATGGCACGGCCTCATTGAGTCCTGGGATGATGATGGGCATGTTC

AATATGCTGAGCACAGTATTGGGGGTCTCAATCCTGAATCTCGGGCAAAAGAGGTACACC

AAAACCACATACTGGTGGGATGGACTTCAATCCTCTGATGATTTCGCTCTCATAGTGAAT

GCACCGAATCATGAGGGGATACAAGCAGGAGTGGATAGATTCTATAGGACCTGCAAACTG

GTTGGGATCAACATGAGCAAAAAGAAGTCTTACATAAACCGAACAGGAACATTTGAGTTC

ACAAGTTTTTTCTACCGCTATGGATTTGTAGCTAACTTCAGTATGGAATTACCCAGCTTT

GGAGTGTCTGGAATCAATGAATCAGCTGACATGAGCATTGGAGTTACAGTGATAAAAAAC

AATATGATAAACAATGATCTTGGACCAGCAACAGCTCAAATGGCCCTTCAATTATTCATC

AAAGACTATAGGTACACATACCGATGCCACAGGGGTGATACACAAATTCAAACGAGGAGA

TCATTCGAGCTGAAGAAGCTGTGGGAGCAGACCCGTTCAAAGGCAGGGCTGTTGATATCA

GACGGGGGGCCAAACCTATACAACATTCGGAACCTCCACATCCCGGAGGTCTGCTTGAAG

TGGGAGCTGATGGATGAAGACTACCAAGGCAGGCTGTGCAATCCTATGAATCCATTTGTC

AGTCATAAAGAGATTGAGTCCGTAAACAATGCTGTAGTAATGCCCGCCCATGGCCCGGCC

AAGAGCATGGAATATGATGCTGTTGCGACCACACATTCGTGGATTCCTAAGAGGAACCGC

TCCATTCTCAATACCAGCCAAAGGGGAATTCTTGAGGATGAGCAGATGTACCAAAAGTGC

TGTAGTCTATTCGAGAAATTCTTCCCCAGCAGTTCATACAGGAGGCCAGTTGGAATTTCC

AGCATGGTGGAGGCCATGGTGTCTAGGGCCCGAATTGATGCACGCATCGACTTCGAATCT

GGAAGGATTAAGAAAGAAGAGTTTGCTGAGATCATGAAGATCTGTTCCACCATTGAAGAG

CTCAGACGGCAAAAATAG------------------------------------------

-

>A_duck_Korea_H538_2020_EPI1846151

------------------------ATGGATGTCAATCCGACTTTACTTTTCTTAAAAGTG

CCAGCGCAAAATGCTATAAGTACCACATTCCCTTACACTGGAGATCCTCCATACAGCCAT

GGAACAGGAACAGGGTATACCATGGACACAGTAAACAGAACACATCAATACTCAGAAAAG

GGAAAGTGGACAACAAACACAGAAACCGGAGCACCCCAACTCAACCCAATTGATGGACCA

TTACCAGAGGACAATGAGCCAAGCGGATATGCACAAACTGATTGCGTGTTGGAAGCAATG

GCTTTCCTTGAAGAATCCCACCCAGGGATATTTGAAAACTCTTGTCTTGAAGCGATGGAA

ATCGTTCAGCAAACAAGAGTGGACAAACTAACCCAAGGTCGCCAGACTTATGACTGGACA

CTGAACAGAAACCAACCAGCTGCAACCTCTTTGGCCAACACTATAGAGGTGTTCAGATCG

AATGGTCTGACAGCCAATGAATCAGGGAGACTGATAGATTTTCTCAGGGATGTGATGGAA

TCAATGAATAAAGAAGAGATGGAAGTAACAACACATTTCCAGAGAAAAAGAAGAGTGAGG

GACAACATGACTAAGAAGATGGTCACACAAAGAACAATAGGGAAGAAGAAGCAGAGGCTG

AACAAGAGGAGTTACTTGATAAGAGCACTGACATTGAATACAATGACCAAAGATGCAGAA

AGAGGCAAGTTGAAAAGACGGGCAATTGCAACACCCGGGATGCAGATTAGAGGATTCGTG

TACTTCGTTGAAACACTAGCGAGGAGCATCTGTGAGAAACTAGAGCAATCTGGGCTCCCT

GTTGGAGGGAATGAGAAGAAGGCTAAATTGGCAAATGTTGTGAGAAAAATGATGACTAAC

TCACAAGATACAGAGCTCTCCTTTACAATTACTGGAGACAACACCAAATGGAATGAGAAT

CAAAACCCTCGGATGTTTTTGGCAATGATAACATATATCACAAGGAACCAACCTGAATGG

TTTAGAAATGTCTTAAGCATTGCCCCTATAATGTTCTCAAACAAAATGGCGAGATTAGGG

AAAGGATACATGTTTGAAAGTAAGAGCATGAAGCTAAGAACACAAATACCAGCAGAGATG

CTTACAAATATTGATCTGAAGTATTTCAACGAATCAACGAGAAAGAAAATCGAGAAAATA

AGACCTCTGCTGATTGATGGCACGGCCTCATTGAGTCCTGGGATGATGATGGGCATGTTC

AATATGCTGAGCACAGTATTGGGGGTCTCAATCCTGAATCTCGGGCAAAAGAGGTACACC

AAAACCACATACTGGTGGGATGGACTTCAATCCTCTGATGATTTCGCTCTCATAGTGAAT

GCACCGAATCATGAGGGGATACAAGCAGGAGTGGATAGATTCTATAGGACCTGCAAACTG

GTTGGGATCAACATGAGCAAAAAGAAGTCTTACATAAACCGAACAGGAACATTTGAGTTC

ACAAGTTTTTTCTACCGCTATGGATTTGTAGCTAACTTCAGTATGGAATTACCCAGCTTT

GGAGTGTCTGGAATCAATGAATCAGCTGACATGAGCATTGGAGTTACAGTGATAAAAAAC

AATATGATAAACAATGATCTTGGACCAGCAACAGCTCAAATGGCTCTTCAATTATTCATC

AAAGACTATAGGTACACATACCGATGCCACAGGGGTGATACACAAATTCAAACGAGGAGA

TCATTCGAGCTGAAGAAGCTGTGGGAGCAGACCCGTTCAAGGGCAGGGCTGTTGATATCA

GACGGGGGGCCAAACCTATACAACATTCGGAACCTCCACATCCCAGAGGTCTGCTTGAAG

TGGGAGCTGATGGATGAAGACTACCAAGGCAGGCTGTGCAACCCTCTGAATCCATTTGTC

AGTCATAAAGAGATTGAGTCCGTAAACAATGCTGTAGTAATGCCCGCCCATGGCCCGGCC

AAGAGCATGGAATATGATGCTGTTGCGACCACACATTCGTGGATTCCTAAGAGGAACCGC

TCCATTCTCAATACCAGCCAAAGGGGAATTCTTGAGGATGAGCAGATGTACCAAAAGTGC

TGTAGTCTATTCGAGAAATTCTTCCCCAGCAGTTCATACAGGAGGCCAGTTGGAATTTCC

AGCATGGTGGAGGCCATGGTGTCTAGGGCCCGAATTGATGCACGCATCGACTTCGAATCT

GGAAGGATTAAGAAAGAAGAGTTTGCTGAGATCATGAAGATCTGTTCCACCATTGAAGAG

CTCAGACGGCAAAAATAG------------------------------------------

-

>A_chicken_Tyumen_302-01_2020_EPI1848604

AGCAAAAGCAGGCAAACTATTTGAATGGATGTCAATCCGACTTTACTTTTCTTAAAAGTG

CCAGCGCAAAATGCTATAAGTACTACATTCCCCTACACTGGAGATCCTCCATACAGCCAT

GGAACAGGAACAGGGTATACCATGGACACAGTAAACAGAACACATCAATACTCAGAAAAG

GGAAAGTGGACAACAAACACAGAAACCGGAGCACCCCAACTCAACCCAATTGATGGACCA

TTACCAGAGGACAATGAGCCAAGCGGATATGCACAAACTGATTGCGTGTTGGAAGCAATG

GCTTTCCTTGAAGAATCCCACCCAGGGATATTTGAAAACTCTTGTCTTGAAGCGATGGAA

ATCGTTCAGCAAACAAGAGTGGACAAACTAACCCAAGGTCGCCAGACTTATGACTGGACA

CTGAACAGAAACCAACCAGCTGCAACCTCTTTGGCCAACACTATAGAGGTGTTCAGATCG

AATGGTCTGACAGCCAATGAATCAGGGAGACTGATAGATTTTCTCAGGGATGTGATGGAA

TCAATGGATAAAGAAGAGATGGAAGTAACAACACATTTCCAGAGAAAAAGAAGAGTGAGG

GACAACATGACTAGGAAGATGGTCACACAAAGAACAATAGGGAAGAAGAAGCAGAGGCTG

AACAAGAGGAGTTACTTAATAAGAGCACTGACATTGAATACAATGACCAAAGATGCAGAA

AGAGGCAAGTTGAAGAGACGGGCAATTGCAACACCCGGGATGCAGATTAGAGGATTCGTG

TACTTCGTTGAAACACTAGCGAGGAGCATCTGTGAGAAATTAGAGCAATCTGGGCTCCCT

GTTGGAGGGAATGAGAAGAAGGCTAAATTGGCAAATGTTGTGAGAAAAATGATGACTAAC

TCACAAGATACAGAGCTCTCCTTTACAATTACTGGAGACAACACCAAATGGAATGAGAAT

CAAAACCCTCGGATGTTTTTGGCAATGATAACATATATCACAAGAAACCAACCTGAATGG

TTTAGAAATGTCTTAAGCATTGCCCCTATAATGTTCTCAAACAAAATGGCGAGATTAGGG

AAAGGATACATGTTTGAAAGTAAGAGCATGAAGCTAAGAACACAAATACCAGCAGAGATG

CTTACAAATGTTGATCTGAAGTATTTCAACGAACCAACGAGAAAGAAAATCGAGAAAATA

AGACCTCTGCTGATTGATGGCACGGCCTCATTGAGTCCTGGGATGATGATGGGCATGTTC

AATATGCTGAGCACAGTATTAGGGGTCTCAATCCTGAATCTCGGGCAAAAAAGGTACACC

AAAACCACATACTGGTGGGATGGACTTCAATCCTCTGATGATTTCGCTCTCATAGTGAAT

GCACCGAATCATGAGGGGATACAAGCAGGAGTGGATAGATTCTATAGGACCTGCAAACTG

GTTGGGATCAACATGAGCAAAAAGAAGTCTTACATAAACCGAACAGGAACATTTGAGTTC

ACAAGTTTTTTCTACCGCTATGGATTTGTAGCTAACTTCAGTATGGAATTACCCAGCTTT

GGAGTGTCTGGAATCAATGAATCAGCTGACATGAGCATTGGAGTTACAGTGATAAAAAAC

AATATGATAAACAATGATCTTGGACCAGCAACAGCTCAAATGGCTCTTCAGTTATTCATC

AAAGACTATAGGTACACATACCGATGCCACAGGGGTGATACACAAATTCAAACGAGGAGA

TCATTCGAGCTGAAGAAGCTGTGGGAGCAGACCCGTTCAAAGGCAGGGCTGTTGATATCA

GACGGGGGGCCAAACCTATACAACATTCGGAATCTCCACATCCCAGAGGTCTGCTTGAAG

TGGGAGCTGATGGATGAAGACTACCAAGGCAGGCTGTGCAATCCTCTGAATCCATTTGTC

AGTCATAAAGAGATTGAGTCCGTAAACAATGCTGTAGTAATGCCCGCCCATGGCCCGGCC

AAGAGCATGGAATATGATGCTGTTGCGACCACACACTCGTGGATTCCTAAGAGGAACCGT

TCCATTCTCAATACCAGCCAAAGGGGAATTCTTGAGGATGAGCAGATGTACCAAAAGTGC

TGTAGTCTATTCGAGAAATTCTTCCCCAGCAGTTCATACAGGAGGCCAGTTGGAATTTCC

AGCATGGTGGAGGCCATGGTGTCTAGGGCCCGAATTGATGCACGCATCGACTTCGAATCT

GGAAGGATTAAGAAAGAAGAGTTTGCTGAGATCATGAAGATCTGTTCCACCATTGAAGAG

TTCAGACGGCAAAAATAGTGAATTTAGCTTGTCCTTCATGAAAAAATGCCTTGTTTCTAC

T

>A_chicken_Tyumen_302-02_2020_EPI1848612

AGCAAAAGCAGGCAAACTATTTGAATGGATGTCAATCCGACTTTACTTTTCTTAAAAGTG

CCAGCGCAAAATGCTATAAGTACTACATTCCCCTACACTGGAGATCCTCCATACAGCCAT

GGAACAGGAACAGGGTATACCATGGACACAGTAAACAGAACACATCAATACTCAGAAAAG

GGAAAGTGGACAACAAACACAGAAACCGGAGCACCCCAACTCAACCCAATTGATGGACCA

TTACCAGAGGACAATGAGCCAAGCGGATATGCACAAACTGATTGCGTGTTGGAAGCAATG

GCTTTCCTTGAAGAATCCCACCCAGGGATATTTGAAAACTCTTGTCTTGAAGCGATGGAA

ATCGTTCAGCAAACAAGAGTGGACAAACTAACCCAAGGTCGCCAGACTTATGACTGGACA

CTGAACAGAAACCAACCAGCTGCAACCTCTTTGGCCAACACTATAGAGGTGTTCAGATCG

AATGGTCTGACAGCCAATGAATCAGGGAGACTGATAGATTTTCTCAGGGATGTGATGGAA

TCAATGGATAAAGAAGAGATGGAAGTAACAACACATTTCCAGAGAAAAAGAAGAGTGAGG

GACAACATGACTAGGAAGATGGTCACACAAAGAACAATAGGGAAGAAGAAGCAGAGGCTG

AACAAGAGGAGTTACTTAATAAGAGCACTGACATTGAATACAATGACCAAAGATGCAGAA

AGAGGCAAGTTGAAGAGACGGGCAATTGCAACACCCGGGATGCAGATTAGAGGATTCGTG

TACTTCGTTGAAACACTAGCGAGGAGCATCTGTGAGAAATTAGAGCAATCTGGGCTCCCT

GTTGGAGGGAATGAGAAGAAGGCTAAATTGGCAAATGTTGTGAGAAAAATGATGACTAAC

TCACAAGATACAGAGCTCTCCTTTACAATTACTGGAGACAACACCAAATGGAATGAGAAT

CAAAACCCTCGGATGTTTTTGGCAATGATAACATATATCACAAGAAACCAACCTGAATGG

TTTAGAAATGTCTTAAGCATTGCCCCTATAATGTTCTCAAACAAAATGGCGAGATTAGGG

AAAGGATACATGTTTGAAAGTAAGAGCATGAAGCTAAGAACACAAATACCAGCAGAGATG

CTTACAAATGTTGATCTGAAGTATTTCAACGAACCAACGAGAAAGAAAATCGAGAAAATA

AGACCTCTGCTGATTGATGGCACGGCCTCATTGAGTCCTGGGATGATGATGGGCATGTTC

AATATGCTGAGCACAGTATTAGGGGTCTCAATCCTGAATCTCGGGCAAAAAAGGTACACC

AAAACCACATACTGGTGGGATGGACTTCAATCCTCTGATGATTTCGCTCTCATAGTGAAT

GCACCGAATCATGAGGGGATACAAGCAGGAGTGGATAGATTCTATAGGACCTGCAAACTG

GTTGGGATCAACATGAGCAAAAAGAAGTCTTACATAAACCGAACAGGAACATTTGAGTTC

ACAAGTTTTTTCTACCGCTATGGATTTGTAGCTAACTTCAGTATGGAATTACCCAGCTTT

GGAGTGTCTGGAATCAATGAATCAGCTGACATGAGCATTGGAGTTACAGTGATAAAAAAC

AATATGATAAACAATGATCTTGGACCAGCAACAGCTCAAATGGCTCTTCAGTTATTCATC

AAAGACTATAGGTACACATACCGATGCCACAGGGGTGATACACAAATTCAAACGAGGAGA

TCATTCGAGCTGAAGAAGCTGTGGGAGCAGACCCGTTCAAAGGCAGGGCTGTTGATATCA

GACGGGGGGCCAAACCTATACAACATTCGGAATCTCCACATCCCAGAGGTCTGCTTGAAG

TGGGAGCTGATGGATGAAGACTACCAAGGCAGGCTGTGCAATCCTCTGAATCCATTTGTC

AGTCATAAAGAGATTGAGTCCGTAAACAATGCTGTAGTAATGCCCGCCCATGGCCCGGCC

AAGAGCATGGAATATGATGCTGTTGCGACCACACACTCGTGGATTCCTAAGAGGAACCGT

TCCATTCTCAATACCAGCCAAAGGGGAATTCTTGAGGATGAGCAGATGTACCAAAAGTGC

TGTAGTCTATTCGAGAAATTCTTCCCCAGCAGTTCATACAGGAGGCCAGTTGGAATTTCC

AGCATGGTGGAGGCCATGGTGTCTAGGGCCCGAATTGATGCACGCATCGACTTCGAATCT

GGAAGGATTAAGAAAGAAGAGTTTGCTGAGATCATGAAGATCTGTTCCACCATTGAAGAG

TTCAGACGGCAAAAATAGTGAATTTAGCTTGTCCTTCATGAAAAAATGCCTTGTTTCTAC

T

>A_chicken_Poland_474_2020_EPI1850191

------------CAAACTATTTGAATGGATGTCAATCCGACTTTACTTTTCTTAAAAGTG

CCAGCGCAAAATGCTATAAGTACTACATTCCCTTACACTGGAGATCCCCCATACAGCCAT

GGAACAGGAACAGGGTATACCATGGACACAGTAAACAGAACACATCAATACTCAGAAAAG

GGAAAGTGGACAACAAACACAGAAACCGGAGCACYCCAACTCAACCCAATTGATGGACCA

TTACCAGAGGACAATGAGCCAAGCGGATATGCACAAACTGATTGCGTGTTGGAAGCAATG

GCTTTCCTTGAAGAATCCCACCCAGGGATATTTGAAAACTCTTGTCTTGAAGCGATGGAA

ATCGTTCAGCAAACAAGAGTGGACAAACTAACCCAAGGTCGCCAGACTTATGACTGGACA

CTGAATAGAAACCAACCAGCTGCAACCTCTTTGGCCAACACTATAGAGGTGTTCAGATCG

AATGGTCTGACAGCCAATGAATCAGGGAGACTGATAGATTTTCTCAGGGATGTGATGGAA

TCAATGGATAAAGAAGAGATGGAAGTAACAACACATTTCCAGAGAAAAAGAAGAGTGAGG

GACAACATGACTAAGAAGATGGTCACACAAAGAACAATAGGGAAGAAGAAGCAGAGGCTG

AACAAGAGGAGTTACTTAATAAGAGCACTGACATTGAACACAATGACCAAAGATGCAGAA

AGAGGCAAGTTGAAGAGACGGGCAATTGCAACACCCGGGATGCAGATTAGAGGATTCGTG

TACTTCGTTGAAACACTAGCGAGGAGCATCTGTGAAAAACTAGAGCAATCTGGGCTCCCT

GTTGGAGGGAATGAGAAGAAGGCTAAATTGGCAAATGTTGTGAGAAAAATGATGACTAAC

TCACAAGATACAGAGCTCTCCTTTACAATTACTGGAGACAACACCAAATGGAATGAGAAT

CAAAACCCTCGGATGTTTTTGGCAATGATAACATATATCACAAGAAACCAACCTGAATGG

TTTAGAAATGTCTTAAGCATTGCCCCTATAATGTTCTCAAACAAAATGGCGAGATTAGGG

AAAGGATACATGTTTGAAAGTAAGAGCATGAAGCTAAGAACACAAATACCAGCAGAGATG

CTTACAAATATTGATCTGAAGTATTTCAACGAACCAACGAGAAAGAAAATCGAGAAAATA

AGACCTCTGCTGATTGATGGCACGGCCTCATTGAGTCCTGGGATGATGATGGGCATGTTC

AATATGCTGAGCACAGTATTAGGGGTCTCAATCCTGAATCTCGGGCAAAAGAGGTACACC

AAAACCACATACTGGTGGGATGGACTTCAATCCTCTGATGATTTCGCTCTCATAGTGAAT

GCACCGAATCATGAGGGGATACAAGCAGGAGTGGATAGATTCTATAGGACCTGCAAACTG

GTTGGGATCAACATGAGCAAAAAGAAGTCTTACATAAACCGAACAGGAACATTTGAGTTC

ACAAGTTTTTTCTACCGCTATGGATTTGTAGCCAACTTCAGTATGGAATTACCCAGCTTT

GGAGTGTCTGGAATCAATGAATCAGCTGACATGAGCATTGGAGTTACAGTGATAAAAAAC

AATATGATAAACAATGATCTTGGACCAGCAACAGCTCAAATGGCTCTTCAGTTATTCATC

AAAGACTATAGGTACACATACAGATGCCACAGGGGTGATACACAAATTCAAACGAGGAGA

TCATTCGAGCTGAAGAAGCTGTGGGAGCAGACCCGTTCAAAGGCAGGGCTGTTGATATCA

GACGGGGGGCCAAATCTATACAACATTCGGAATCTCCACATCCCAGAGGTCTGCTTGAAG

TGGGAGCTGATGGATGAAGACTACCAAGGCAGGCTGTGCAATCCTCTGAATCCATTTGTC

AGTCATAAAGAGATTGAGTCCGTAAACAATGCTGTAGTAATGCCCGCCCATGGCCCGGCC

AAGAGCATGGAATATGATGCTGTTGCGACCACACACTCGTGGATTCCTAAGAGGAACCGT

TCCATTCTCAATACCAGCCAAAGGGGAATTCTTGAGGATGAGCAGATGTACCAAAAGTGC

TGTAGTCTATTCGAGAAATTCTTCCCCAGCAGTTCATACAGGAGGCCAGTTGGAATTTCC

AGCATGGTGGAGGCCATGGTGTCTAGGGCCCGAATTGATGCACGCATCGACTTCGAATCT

GGAAGGATTAAGAAAGAAGAGTTTGCTGAGATCATGAAGATCTGTTCCACCATTGAAGAG

CTCAGACGGCAAAAATAGTGAATTTAGCTTGTCCTTCATGAAAAAATGC-----------

-

>A_swan_Poland_MB141_2020_EPI1850216

------------CAAACTATTTGAATGGATGTCAATCCGACTTTACTTTTCTTAAAAGTG

CCAGCGCAAAATGCTATAAGTACTACATTCCCTTACACTGGAGATCCCCCATACAGCCAT

GGAACAGGAACAGGGTATACCATGGACACAGTAAACAGAACACATCAATACTCAGAAAAG

GGAAAGTGGACAACAAACACAGAAACCGGAGCACCCCAACTCAACCCAATTGATGGACCA

TTACCAGAGGACAATGAGCCAAGCGGATATGCACAAACTGATTGCGTGTTGGAAGCAATG

GCTTTCCTTGAAGAATCCCACCCAGGGATATTTGAAAACTCTTGTCTTGAAGCGATGGAA

ATCGTTCAGCAAACAAGAGTGGACAAACTAACCCAAGGTCGCCAGACTTATGACTGGACA

CTGAACAGAAACCAACCAGCTGCAACCTCTTTGGCCAACACTATAGAGGTGTTCAGATCG

AATGGTCTAACAGCCAATGAATCAGGGAGACTGATAGATTTTCTCAGGGATGTGATGGAA

TCAATGGATAAAGAAGAGATGGAAGTAACAACACATTTCCAGAGAAAAAGAAGAGTGAGG

GACAACATGACTAAGAAGATGGTCACACAAAGAACAATAGGGAAGAAGAAGCAGAGGATG

AACAAGAGGAGTTACTTAATAAGAGCACTGACATTGAATACAATGACCAAAGATGCAGAA

AGAGGCAAGTTGAAGAGACGGGCAATTGCAACACCCGGGATGCAGATTAGAGGATTCGTG

TACTTCGTTGAAACACTAGCGAGGAGCATCTGTGAGAAACTAGAGCAATCTGGGCTCCCT

GTTGGAGGGAATGAGAAGAAGGCTAAATTGGCAAATGTTGTGAGAAAAATGATGACTAAC

TCACAAGATACAGAGCTCTCCTTTACAATTACTGGAGACAACACCAAATGGAATGAGAAT

CAAAACCCTCGGATGTTTTTGGCAATGATAACATATATCACAAGAAACCAACCTGAATGG

TTTAGAAATGTCTTAAGCATTGCCCCTATAATGTTCTCAAACAAAATGGCGAGATTAGGG

AAAGGATACATGTTTGAAAGTAAGAGCATGAAGCTAAGAACACAAATACCAGCAGAGATG

CTTACAAATATTGATCTGAAGTATTTCAACGAACCAACGAGAAAGAAAATCGAGAAAATA

AGACCTCTGCTGATTGATGGCACGGCCTCATTGAGTCCTGGGATGATGATGGGCATGTTC

AATATGCTGAGCACAGTATTAGGGGTCTCAATCCTGAATCTCGGGCAAAAGAGGTACACC

AAAACCACATACTGGTGGGATGGACTTCAATCCTCTGATGATTTCGCTCTCATAGTGAAT

GCACCGAATCATGAGGGGATACAAGCAGGAGTGGATAGATTCTATAGGACCTGCAAACTG

GTTGGGATCAACATGAGCAAAAAGAAGTCTTACATAAACCGAACAGGAACATTTGAGTTC

ACAAGTTTTTTCTACCGCTATGGATTTGTAGCCAACTTCAGTATGGAATTACCCAGCTTT

GGAGTGTCTGGAATCAATGAATCAGCTGACATGAGCATTGGAGTTACAGTGATAAAAAAC

AATATGATAAACAATGATCTTGGACCAGCAACAGCTCAAATGGCTCTTCAGTTATTCATC

AAAGACTATAGGTACACATACCGATGCCACAGGGGTGATACACAAATTCAAACGAGGAGA

TCATTCGAGCTGAAGAAGCTGTGGGAGCAGACCCGTTCAAAGGCAGGGCTGTTGATATCA

GACGGGGGGCCAAATCTATACAACATTCGGAATCTCCACATCCCAGAGGTCTGCTTGAAG

TGGGAGCTGATGGATGAAGACTACCAAGGCAGGCTGTGCAATCCTCTGAATCCATTTGTC

AGTCATAAAGAAATTGAGTCCGTAAACAATGCTGTAGTAATGCCCGCCCATGGCCCGGCC

AAGAGCATGGAATATGATGCTGTTGCGACCACACACTCGTGGATTCCTAAGAGGAACCGT

TCCATTCTCAATACCAGCCAAAGGGGAATTCTTGAGGACGAGCAGATGTACCAAAAGTGC

TGTAGTCTATTCGAGAAATTCTTCCCCAGCAGTTCATACAGGAGGCCAGTTGGAATTTCC

AGCATGGTGGAGGCCATGGTGTCTAGGGCCCGAATTGATGCACGCATCGACTTCGAATCT

GGAAGGATTAAGAAAGAAGAGTTTGCTGAGATCATGAAGATCTGTTCCACCATTGAAGAG

CTCAGACGGCAAAAATAGTGAATTTAGCTTGTCCTTCATGAAAAAAT-------------

-

>A_muscovy_duck_Slovakia_Pah1_21VIR1086-1_2021_EPI1858242

------------------------ATGGATGTCAATCCGACTTTACTTTTCTTAAAAGTG

CCAGCGCAAAATGCTATAAGTACTACATTCCCTTACACTGGAGATCCTCCATACAGCCAT

GGGACAGGAACAGGGTATACCATGGACACAGTAAACAGAACACATCAATACTCAGAAAAG

GGAAAGTGGACAACAAACACAGAAACCGGAGCACCCCAACTCAACCCAATTGATGGACCA

TTACCAGAGGACAATGAGCCAAGCGGATATGCACAAACTGATTGCGTGTTGGAAGCAATG

GCTTTCCTTGAAGAATCCCACCCAGGGATATTTGAAAACTCTTGTCTTGAAGCGATGGAA

ATCGTTCAGCAAACAAGAGTGGACAAACTAACCCAAGGTCGCCAGACTTATGACTGGACA

CTGAACAGAAACCAACCAGCTGCAACCTCTTTGGCCAACACTATAGAGGTGTTCAGATCG

AATGGTCTGACAGCCAATGAATCAGGGAGACTGATAGATTTTCTCAGGGATGTGATGGAA

TCAATGGATAAAGAAGAGGTGGAAGTAACAACACATTTCCAGAGAAAAAGAAGAGTGAGG

GACAACATGACTAGGAAGATGGTCACACAAAGAACAATAGGGAAAAAGAAGCAGAGGCTG

AACAAGAGGAGTTACTTAATAAGAGCACTGACATTGAATACAATGACCAAAGATGCAGAA

AGAGGCAAGTTGAAGAGACGGGCAATTGCAACACCCGGGATGCAGATTAGAGGATTCGTG

TACTTCGTTGAAACACTAGCGAGGAGCATCTGTGAGAAACTAGAGCAATCTGGGCTCCCT

GTTGGAGGGAATGAGAAGAAGGCTAAATTGGCAAATGTTGTGAGAAAAATGATGACTAAC

TCACAAGATACAGAGCTCTCCTTTACAATTACTGGAGACAACACCAAATGGAATGAGAAT

CAAAACCCTCGGATGTTTTTGGCAATGATAACATATATCACAAGAAACCAACCTGAATGG

TTTAGAAATGTCTTAAGCATTGCCCCTATAATGTTCTCAAACAAAATGGCGAGATTAGGG

AAAGGATACATGTTTGAAAGTAAGAGCATGAAGCTAAGAACACAAATACCAGCAGAGATG

CTTACAAATATTGATCTGAAGTATTTCAACGAACCAACGAGAAAGAAAATCGAGAAAATA

AGACCTCTGCTGATTGATGGCACGGCCTCATTGAGTCCTGGGATGATGATGGGCATGTTC

AATATGCTGAGCACAGTATTAGGGGTCTCAATCCTGAATCTCGGGCAAAAAAGGTACACC

AAAACCACATACTGGTGGGATGGACTTCAATCCTCTGATGATTTCGCTCTCATAGTGAAT

GCACCGAATCATGAGGGGATACAAGCAGGAGTGGATAGATTCTATAGGACCTGCAAACTG

GTTGGGATCAACATGAGCAAAAAGAAGTCTTACATAAACCGAACAGGAACATTTGAGTTC

ACAAGTTTTTTCTACCGCTATGGATTTGTAGCTAACTTCAGTATGGAATTACCCAGCTTT

GGAGTGTCTGGAATCAATGAATCAGCTGACATGAGCATTGGAGTTACAGTGATAAAAAAC

AATATGATAAACAATGATCTTGGACCAGCAACAGCTCAAATGGCTCTTCAGTTATTCATC

AAAGACTATAGGTACACATACCGATGCCACAGGGGTGATACACAAATTCAAACGAGGAGA

TCATTCGAGCTGAAGAAGCTGTGGGAGCAGACCCGTTCAAAGGCAGGGCTGTTGATATCA

GACGGGGGGCCAAACCTATACAACATTCGGAATCTCCACATCCCAGAGGTCTGCTTGAAG

TGGGAGCTGATGGATGAAGACTACCAAGGCAGGCTGTGCAATCCTCTGAATCCATTTGTC

AGTCATAAAGAGATTGAGTCCGTAAACAATGCTGTAGTAATGCCCGCCCATGGCCCGGCC

AAGAGCATGGAATATGATGCTGTTGCGACCACACACTCGTGGATCCCTAAGAGGAATCGT

TCCATTCTCAATACCAGCCAAAGGGGAATTCTTGAGGATGAGCAGATGTACCAAAAGTGC

TGTAGTCTATTCGAGAAATTCTTCCCCAGCAGTTCATACAGGAGGCCAGTTGGAATTTCC

AGCATGGTGGAGGCCATGGTGTCTAGGGCCCGAATTGATGCACGCATCGACTTCGAATCT

GGAAGGATTAAGAAAGAAGAGTTTGCTGAGATCATGAAGATCTGTTCCACCATTGAAGAG

CTCAGACGGCAAAAATAG------------------------------------------

-

>A_mute_swan_Slovenia_1639-20_21VIR959-1_2020_EPI1858298

------------------------ATGGATGTCAATCCGACTTTACTTTTCTTAAAAGTG

CCAGCGCAAAATGCTATAAGTACTACATTCCCTTACACTGGAGATCCTCCATACAGCCAT

GGAACAGGAACAGGGTATACCATGGACACAGTAAACAGAACACATCAATACTCAGAAAAG

GGAAAGTGGACAACAAACACAGAAACCGGAGCACCCCAACTCAACCCAATTGATGGACCA

TTACCAGAGGACAATGAGCCAAGCGGATATGCACAAACTGATTGCGTGTTGGAAGCAATG

GCTTTCCTTGAAGAATCCCACCCAGGGATATTTGAAAACTCTTGTCTTGAAGCGATGGAA

ATCGTTCAGCAAACAAGAGTGGACAAACTAACCCAAGGTCGCCAGACTTATGACTGGACA

CTGAACAGAAACCAACCAGCTGCAACCTCTTTGGCCAACACTATAGAGGTGTTCAGATCG

AATGGTCTGACAGCCAATGAATCAGGGAGACTGATAGATTTTCTCAGGGATGTGATGGAA

TCAATGGATAAAGAAGAGATGGAAGTAACAACACATTTCCAGAGAAAAAGAAGAGTGAGG

GACAACATGACTAAGAAGATGGTCACACAAAGAACAATAGGGAAGAAGAAGCAGAGGCTG

AACAAGAGGAGTTACTTAATAAGAGCACTGACATTGAATACAATGACCAAAGATGCAGAA

AGGGGCAAGTTGAAGAGACGGGCAATTGCAACACCCGGAATGCAGATTAGAGGATTCGTG

TACTTCGTTGAAACACTAGCGAGGAGCATCTGTGAGAAACTAGAGCAATCTGGGCTCCCT

GTTGGAGGGAATGAGAAGAAGGCTAAATTGGCAAATGTTGTGAGAAAAATGATGACTAAC

TCACAAGATACAGAGCTCTCCTTTACAATTACTGGAGACAACACCAAATGGAATGAAAAT

CAAAACCCTCGGATGTTTTTGGCAATGATAACATATATCACAAGAAACCAACCTGAATGG

TTTAGAAATGTCTTAAGCATTGCCCCTATAATGTTCTCAAACAAAATGGCGAGATTAGGG

AAAGGATACATGTTTGAAAGTAAGAGCATGAAGCTAAGAACACAAATACCAGCAGAGATG

CTTACAAATATTGATCTGAAGTATTTCAACGAAACAACGAGAAAGAAAATCGAGAAAATA

AGACCTCTGCTGATTGATGGCACGGCCTCATTGAGTCCTGGGATGATGATGGGCATGTTC

AATATGCTGAGCACAGTATTGGGGGTCTCAATCCTGAATCTCGGGCAAAAGAGGTACACC

AAAACCACATACTGGTGGGATGGACTTCAATCCTCTGATGATTTCGCTCTCATAGTGAAT

GCACCGAATCATGAGGGGATACAAGCAGGAGTGGATAGATTCTATAGGACCTGCAAACTG

GTTGGGATCAACATGAGCAAAAAGAAGTCTTACATAAACCGAACAGGAACATTTGAGTTC

ACAAGTTTTTTCTACCGCTATGGATTTGTAGCTAACTTCAGTATGGAATTACCCAGCTTT

GGAGTGTCTGGAATCAATGAATCAGCTGACATGAGCATTGGAGTTACAGTGATAAAAAAC

AACATGATAAACAATGATCTTGGACCAGCGACAGCTCAAATGGCTCTTCAGTTATTCATC

AAAGACTATAGGTACACATACCGATGCCACAGGGGTGATACACAAATTCAAACGAGGAGA

TCATTCGAGCTGAAGAAGCTGTGGGAGCAGACCCGTTCAAAGGCAGGGCTGTTGATATCA

GACGGGGGGCCAAACCTATACAACATTCGGAACCTCCACATCCCAGAGGTCTGCTTGAAG

TGGGAGCTGATGGATGAAGACTACCAAGGCAGGCTGTGCAATCCTCTGAATCCATTTGTC

AGTCATAAAGAGATTGAGTCCGTAAACAATGCTGTAGTAATGCCCGCCCATGGCCCGGCC

AAGAGCATGGAATATGATGCTGTTGCGACCACACATTCGTGGATTCCTAAGAGGAACCGT

TCCATTCTCAATACCAGCCAAAGGGGAATTCTTGAGGATGAGCAGATGTACCAAAAGTGC

TGTAGTCTATTCGAGAAATTCTTCCCCAGCAGTTCATACAGGAGGCCAGTTGGAATTTCC

AGCATGGTGGAGGCCATGGTGTCTAGGGCCCGAATTGATGCACGCATCGACTTCGAATCT

GGAAGGATTAAGAAAGAAGAGTTTGCTGAGATCATGAAGATCTGTTCCACCATTGAAGAG

CTCAGACGGCAAAAATAG------------------------------------------

-

>A_Sichuan_26221_2014_EPI533586

------------------------ATGGATGTCAACCCGACTTTACTTTTCTTGAAAGTG

CCAGTGCAAAATGCTATAAGTACCACATTCCCTTATACTGGAGACCCTCCATACAGCCAT

GGAACAGGGACAGGGTACACCATGGACACAGTCAACAGAACACACCAATATTCAGAAAAG

GGGGAGTGGACAACAAACACAGAGACCGGAGCACCCCAACTTAACCCGATTGATGGACCA

TTACCTGAGGATAACGAGCCCAGTGGGTATGCACAAACAGATTGTGTATTAGAGGCAATG

GCTTTCCTTGAAGAATCCCACCCAGGAATCTTTGAAAATTCGTGCCTTGAAACGATGGAA

ATTGTCCAACAAACAAGAGTGGACAAACTGACCCAAGGCCGCCAGACTTATGACTGGACA

TTGAATAGAAACCAACCGGCTGCAACTGCTTTGGCCAACACTATAGAAATTTTCAGATCA

AACGGTCTGACAGCAAATGAATCGGGACGCCTAATAGATTTCCTCAAGGATGTAATGGAA

TCAATGGATAAGGAAGAAATGGAGATAACAACACATTTCCAGAGAAAGAGAAGAGTGAGG

GACAACATGACCAAGAAAATGGTAACGCAAAGAACAATCGGGAAGAAAAAACAAAGGCTG

AACAAAAGGAGTTACCTGATAAGAGCGCTGACACTGAACACAATGACCAAGGATGCAGAA

AGAGGCAAATTGAAGAGGCGAGCGATTGCAACACCCGGAATGCAAATCAGAGGATTCGTG

TACTTCGTTGAAACACTAGCGAGGAGTATTTGTGAGAAACTTGAGCAATCTGGCCTCCCA

GTCGGAGGGAATGAGAAGAAAGCTAAACTGGCAAACGTCGTGAGGAAGATGATGACCAAC

TCACAGGATACTGAACTCTCCTTTACAATTACTGGGGACAATACAAAATGGAATGAGAAT

CAAAATCCTAGGATGTTTCTGGCAATGATAACGTACATCACAAGGAACCAGCCAGAATGG

TTTCGAAATGTTCTAAGCATTGCCCCTATAATGTTTTCAAACAAAATGGCGAGGCTAGGG

AAAGGGTACATGTTCGAAAGTAAGAGCATGAAGTTACGAACACAAATACCAGCAGAAATG

CTTGCAAACATTGACCTTAAATACTTCAATGAATCAACGAAGAAGAAAATTGAGAAGATA

AGACCTCTATTAATAGATGGTACAGCCTCATTGAGCCCTGGAATGATGATGGGCATGTTC

AACATGCTGAGTACAGTCCTAGGAGTTTCAATTCTAAATCTTGGACAGAAAAAATACACC

AAAACCACATATTGGTGGGACGGGCTCCAATCCTCTGATGATTTCGCTCTCATTATAAAT

GCCCCGAATCATGAAGGAATACAAGCAGGGGTGGATAGGTTCTATAGAACTTGTAAACTA

GTTGGGATTAATATGAGCAAGAAGAAGTCTTACATAAATCGGACAGGGACATTTGAATTC

ACTAGCTTTTTCTACCGTTATGGATTCGTAGCCAATTTCAGTATGGAGCTGCCCAGTTTT

GGAGTGTCTGGAATTAATGAGTCGGCCGACATGAGCATTGGTGTTACAGTGATAAAGAAC

AATATGATAAACAACGACCTTGGGCCAGCAACAGCTCAGATGGCTCTTCAGCTATTCATC

AAGGACTACAGGTACACATACCGATGCCACAGGGGAGATACGCAAATCCAAACGAGGAGA

TCATTCGAGCTGAAGAAGCTATGGGAACAAACCCGTTCAAAGGCAGGACTCTTGGTCTCA

GATGGAGGACCAAATCTATACAATATCCGAAATCTCCATATTCCTGAGGTCTGCTTGAAA

TGGGAATTGATGGATGAAGACTACAAGGGTAGACTGTGCAATCCTCTGAATCCATTCGTC

AGCCATAAGGAAATTGAATCTGTCAACAATGCTATAGTAATGCCAACTCATGGCCCGGCC

AAGAGTATGGAATATGATGCCGTAGCAACCACACATTCATGGATTCCTAAAAGGAATCGT

TCCATTCTCAACACAAGTCAAAGGGGAATTCTTGAGGATGAACAGATGTACCAAAAGTGC

TGCAATCTATTCGAGAAATTCTTTCCCAGTAGTTCATATCGGAGACCAGTTGGAATTTCC

AGTATGGTGGAGGCCATGGTTTCTCGGGCTCGAATTGACGCACGAATTGATTTCGAGTCT

GGAAGGATTAAGAAAGAAGAGTTTGCTGAGATCATGAAGATCTGTTCCACCATTGAAGAG

CTCAGACGGCAAAAATAG------------------------------------------

-

>A_duck_Sichuan_NCXJ16_2014_EPI590840

------------------------ATGGATGTCAACCCGACTTTACTTTTCTTGAAAGTG

CCAGTGCAAAATGCTATAAGTACCACATTCCCTTATACTGGAGACCCTCCATACAGCCAT

GGAACAGGGACAGGGTACACCATGGACACAGTCAACAGAACACACCAATATTCAGAAAAG

GGGGAGTGGACAACAAACACAGAGACCGGAGCACCCCAACTTAACCCGATTGATGGACCA

TTACCTGAGGATAACGAGCCCAGTGGGTATGCACAAACAGATTGTGTATTAGAGGCAATG

GCTTTCCTTGAAGAATCCCACCCAGGAATCTTTGAAAATTCGTGCCTTGAAACGATGGAA

ATTGTCCAACAAACAAGAGTGGACAAACTGACCCAAGGCCGCCAGACTTATGACTGGACA

TTGAATAGAAACCAACCGGCTGCAACTGCTTTGGCCAACACTATAGAAATTTTCAGATCA

AACGGTCTGACAGCAAATGAATCGGGACGCCTAATAGATTTCCTCAAGGATGTGATGGAA

TCAATGGATAAGGAAGAAATGGAGATAACAACACATTTCCAGAGAAAGAGAAGAGTGAGG

GACAACATGACCAAGAAAATGGTAACGCAAAGAACAATCGGGAAGAAAAAACAAAGGCTG

AACAAAAGGAGTTACCTGATAAGAGCGCTGACACTGAACACAATGACCAAGGATGCAGAA

AGAGGCAAATTGAAGAGGCGAGCGATTGCAACACCCGGAATGCAAATCAGAGGATTCGTG

TACTTCGTTGAAACACTAGCGAGGAGTATTTGTGAGAAACTTGAGCAATCTGGCCTACCA

GTCGGAGGGAATGAGAAGAAAGCTAAACTGGCAAACGTCGTGAGGAAGATGATGACCAAC

TCACAGGATACTGAACTCTCCTTTACAATTACTGGGGACAATACAAAATGGAATGAGAAT

CAAAATCCTAGGATGTTTCTGGCAATGATAACGTACATCACAAGGAACCAGCCAGAATGG

TTTCGAAATGTTCTAAGCATTGCCCCTATAATGTTTTCAAACAAAATGGCGAGGCTAGGG

AAAGGGTACATGTTCGAAAGTAAGAGCATGAAGTTACGAACACAAATACCAGCAGAAATG

CTTGCAAACATTGACCTTAAATACTTCAATGAATCAACGAAGAAGAAAATTGAGAAGATA

AGACCTCTATTAATAGATGGTACAGCCTCATTGAGCCCTGGAATGATGATGGGCATGTTC

AACATGCTGAGTACAGTCCTAGGAGTTTCAATTCTAAATCTTGGACAGAAAAAATACACC

AAAACCACATATTGGTGGGACGGGCTCCAATCCTCTGATGATTTCGCTCTCATTATAAAT

GCCCCGAATCATGAAGGAATACAAGCAGGGGTGGATAGGTTCTATAGAACTTGTAAACTA

GTTGGGATTAATATGAGCAAGAAGAAGTCTTACATAAATCGGACAGGGACATTTGAATTC

ACTAGCTTTTTCTACCGTTATGGATTCGTAGCCAATTTCAGTATGGAGCTGCCCAGTTTT

GGAGTGTCTGGAATTAATGAGTCGGCCGACATGAGCATTGGTGTTACAGTGATAAAGAAC

AATATGATAAACAACGACCTTGGGCCAGCAACAGCTCAGATGGCTCTTCAGCTATTCATC

AAGGACTACAGGTACACATACCGATGCCACAGGGGAGATACGCAAATCCAAACGAGGAGA

TCATTCGAGCTGAAGAAGCTATGGGAACAAACCCGTTCAAAGGCAGGACTCTTGGTCTCA

GATGGAGGACCAAATCTATACAATATCCGAAATCTCCATATTCCTGAGGTCTGCTTGAAA

TGGGAATTGATGGATGAAGACTACAAGGGTAGACTGTGCAATCCTCTGAATCCATTCGTC

AGCCATAAGGAAATTGAATCTGTCAACAATGCTATAGTAATGCCAACTCATGGCCCGGCC

AAGAGTATGGAATATGATGCCGTAGCAACCACACATTCATGGATTCCTAAAAGGAATCGT

TCCATTCTCAACACAAGTCAAAGGGGAATTCTTGAGGATGAACAGATGTACCAAAAGTGC

TGCAATCTATTCGAGAAATTCTTTCCCAGTAGTTCATATCGGAGACCAGTTGGAATTTCC

AGTATGGTGGAGGCCATGGTTTCTCGGGCTCGAATTGACGCACGAATTGATTTCGAGTCT

GGAAGGATTAAGAAAGAAGAGTTTGCTGAGATCATGAAGATCTGTTCCACCATTGAAGAG

CTCAGACGGCAAAAATAG------------------------------------------

-

>A_Fujian-Sanyuan_21099_2017_x_PR8_CNI_1369971

------------------------ATGGATGTCAATCCGACCTTACTTTTCTTAAAAGTG

CCAGCACAAAATGCTATAAGCACAACTTTCCCTTATACTGGAGACCCTCCTTACAGCCAT

GGGACAGGAACAGGATACACCATGGATACTGTCAACAGGACACATCAGTACTCAGAAAAG

GGAAGATGGACAACAAACACCGAAACTGGAGCACCGCAACTCAACCCGATTGATGGGCCA

CTGCCAGAAGACAATGAACCAAGTGGTTATGCCCAAACAGATTGTGTATTGGAGGCGATG

GCTTTCCTTGAGGAATCCCATCCTGGTATTTTTGAAAACTCGTGTATTGAAACGATGGAG

GTTGTTCAGCAAACACGAGTAGACAAGCTGACACAAGGCCGACAGACCTATGACTGGACT

CTAAATAGAAACCAACCTGCTGCAACAGCATTGGCCAACACAATAGAAGTGTTCAGATCA

AATGGCCTCACGGCCAATGAGTCTGGAAGGCTCATAGACTTCCTTAAGGATGTAATGGAG

TCAATGAACAAAGAAGAAATGGGGATCACAACTCATTTTCAGAGAAAGAGACGGGTGAGA

GACAATATGACTAAGAAAATGATAACACAGAGAACAATGGGTAAAAAGAAGCAGAGATTG

AACAAAAGGAGTTATCTAATTAGAGCATTGACCCTGAACACAATGACCAAAGATGCTGAG

AGAGGGAAGCTAAAACGGAGAGCAATTGCAACCCCAGGGATGCAAATAAGGGGGTTTGTA

TACTTTGTTGAGACACTGGCAAGGAGTATATGTGAGAAACTTGAACAATCAGGGTTGCCA

GTTGGAGGCAATGAGAAGAAAGCAAAGTTGGCAAATGTTGTAAGGAAGATGATGACCAAT

TCTCAGGACACCGAACTTTCTTTCACCATCACTGGAGATAACACCAAATGGAACGAAAAT

CAGAATCCTCGGATGTTTTTGGCCATGATCACATATATGACCAGAAATCAGCCCGAATGG

TTCAGAAATGTTCTAAGTATTGCTCCAATAATGTTCTCAAACAAAATGGCGAGACTGGGA

AAAGGGTATATGTTTGAGAGCAAGAGTATGAAACTTAGAACTCAAATACCTGCAGAAATG

CTAGCAAGCATCGATTTGAAATATTTCAATGATTCAACAAGAAAGAAGATTGAAAAAATC

CGACCGCTCTTAATAGAGGGGACTGCATCATTGAGCCCTGGAATGATGATGGGCATGTTC

AATATGTTAAGCACTGTATTAGGCGTCTCCATCCTGAATCTTGGACAAAAGAGATACACC

AAGACTACTTACTGGTGGGATGGTCTTCAATCCTCTGACGATTTTGCTCTGATTGTGAAT

GCACCCAATCATGAAGGGATTCAAGCCGGAGTCGACAGGTTTTATCGAACCTGTAAGCTA

CTTGGAATCAATATGAGCAAGAAAAAGTCTTACATAAACAGAACAGGTACATTTGAATTC

ACAAGTTTTTTCTATCGTTATGGGTTTGTTGCCAATTTCAGCATGGAGCTTCCCAGTTTT

GGGGTGTCTGGGATCAACGAGTCAGCGGACATGAGTATTGGAGTTACTGTCATCAAAAAC

AATATGATAAACAATGATCTTGGTCCAGCAACAGCTCAAATGGCCCTTCAGTTGTTCATC

AAAGATTACAGGTACACGTACCGATGCCATATAGGTGACACACAAATACAAACCCGAAGA

TCATTTGAAATAAAGAAACTGTGGGAGCAAACCCGTTCCAAAGCTGGACTGCTGGTCTCC

GACGGAGGCCCAAATTTATACAACATTAGAAATCTCCACATTCCTGAAGTCTGCCTAAAA

TGGGAATTGATGGATGAGGATTACCAGGGGCGTTTATGCAACCCACTGAACCCATTTGTC

AGCCATAAAGAAATTGAATCAATGAACAATGCAGTGATGATGCCAGCACATGGTCCAGCC

AAAAACATGGAGTATGATGCTGTTGCAACAACACACTCCTGGATCCCCAAAAGAAATCGA

TCCATCTTGAATACAAGTCAAAGAGGAGTACTTGAGGATGAACAAATGTACCAAAGGTGC

TGCAATTTATTTGAAAAATTCTTCCCCAGCAGTTCATACAGAAGACCAGTCGGGATATCC

AGTATGGTGGAGGCTATGGTTTCCAGAGCCCGAATTGATGCACGGATTGATTTCGAATCT

GGAAGGATAAAGAAAGAAGAGTTCACTGAGATCATGAAGATCTGTTCCACCATTGAAGAG

CTCAGACGGCAAAAATAG------------------------------------------

-

>A_Perigrine_falcon_Netherlands_1800327_1327126

------------CAAACCATTTGAATGGATGTCAATCCGACTTTACTTTTCTTAAAAGTG

CCAGCGCAAAATGCTATAAGTACTACATTCCCTTACACTGGAGACCCTCCATACAGCCAT

GGAACAGGAACAGGATATACCATGGACACAGTAAACAGAACACATCAATACTCAGAAAAG

GGAAAGTGGACAACAAACACAGAAACCGGAGCACCCCAACTCAACCCAATTGATGGACCA

TTACCAGAGGACAATGAGCCAAGCGGGTATGCACAAACTGATTGCGTGTTGGAAGCAATG

GCTTTCCTTGAAGAATCCCACCCAGGGATATTTGAAAACTCTTGTCTTGAAGCGATGGAA

GTCGTTCAGCAAACAAGAGTGGACAAACTAACCCAAGGTCGCCAGACTTATGACTGGACA

CTGAACAGAAACCAACCAGCTGCAACTGCTCTGGCCAACACTATAGAGGTGTTCAGATCG

AATGGTCTGACAGCCAGTGAATCAGGGAGACTGATAGATTTTCTCAGGGATGTGATGGAA

TCAATGGATAAAGAAGAGATGGAAATAACAACACATTTCCAGAGAAAAAGAAGAGTGAGG

GACAACATTACCAAGAAGATGGTCACACAAAGAACAATAGGAAAGAAGAAGCAGAGGCTG

AACAAGAGGAGTTACTTAATAAGAGCACTGACCTTGAACACAATGACCAAAGATGCAGAA

AGAGGCAAATTAAAGAGACGGGCAATTGCAACACCCGGAATGCAGATTAGAGGATTCGTG

TACTTTGTCGAAACACTAGCGAGGAGCATCTGTGAGAAACTCGAGCAATCTGGGCTCCCT

GTTGGAGGGAATGAGAAGAAGGCTAAATTGGCAAATGTCGTGAGAAAAATGATGACTAAC

TCACAAGATACAGAGCTCTCCTTTACAATTACTGGAGACAACACCAAATGGAATGAGAAT

CAAAACCCTCGGATGTTTTTGGCAATGATAACATACATCACAAGAAACCAACCTGAATGG

TTTAGAAATGTCTTAAGCATTGCCCCTATAATGTTCTCAAACAAAATGGCGAGGTTAGGG

AAAGGATACATGTTTGAAAGTAAGAGCATGAAGCTAAGGACACAAATACCAGCAGAAATG

CTTACAAACATTGATCTGAAATATTTCAACGAATCAACAAGAAAGAAAATCGAGAAAATA

AGACCTCTGCTGATTGATGGCACGGCCTCATTGAGTCCTGGGATGATGATGGGCATGTTC

AATATGCTGAGCACAGTATTAGGGGTCTCAATCCTGAATCTCGGGCAAAAGAGGTACACC

AAAACCACATACTGGTGGGATGGACTTCAATCCTCTGATGATTTCGCTCTCATAGTGAAT

GCACCGAATCATGAGGGGATACAAGCAGGAGTGGATAGGTTCTATAGGACCTGCAAACTG

GTTGGGATCAACATGAGCAAAAAGAAATCTTACATAAACCGAACAGGAACATTTGAGTTC

ACAAGTTTTTTCTACCGCTATGGATTTGTAGCTAACTTCAGTATGGAATTACCCAGCTTT

GGAGTGTCTGGAATCAATGAATCAGCTGACATGAGCATTGGAGTTACAGTGATAAAAAAC

AATATGATAAACAATGACCTTGGACCAGCAACAGCTCAAATGGCTCTTCAGTTATTCATC

AAAGACTATAGGTACACGTACCGATGCCACAGGGGTGATGCACAAATTCAAACGAGGAGA

TCATTCGAGCTGAAGAAGCTGTGGGAGCAGACTCGTTCAAAGGCAGGGCTGTTGGTATCA

GACGGAGGGCCAAACCTATACAATATTCGGAATCTCCACATCCCAGAGGTCTGCTTGAAG

TGGGAACTGATGGATGAAGACTACCAAGGCAGGCTGTGCAACCCTCTGAACCCATTTGTC

AGTCATAAAGAGATTGAGTCCGTAAACAATGCTGTAGTAATGCCAGCCCATGGCCCGGCC

AAGAGCATGGAATATGATGCTGTTGCGACTACACACTCGTGGATTCCTAAGAGGAACCGT

TCCATTCTCAATACCAGCCAGAGGGGAATTCTTGAGGATGAGCAAATGTACCAAAAGTGC

TGTAGTCTATTCGAGAAATTCTTCCCCAGCAGTTCATACAGGAGGCCAGTTGGAATTTCC

AGCATGGTGGAGGCCATGGTGTCTAGGGCCCGAATTGATGCACGCATCGATTTCGAATCT

GGAAGGATTAAGAAAGAAGAGTTTGCTGAGATCATGAAGATCTGTTCCACCATTGAAGAG

CTCAGACGGCAAAAATAGTGAATTTAGCTTGTCCTTCATGAAAAAATG------------

-

>A_chicken_Washington_3490-18_2015_EPI590691

------------------------ATGGATGTCAATCCGACTTTACTTTTCTTGAAAGTT

CCAGCGCAAAATGCCATAAGCACCACATTCCCGTATACCGGAGATCCTCCATACAGCCAT

GGAACAGGAACAGGATACACCATGGACACGGTTAACAGAACACATCAATATTCAGAAAAA

GGAAAATGGACAACAAACTCAGAGACTGGAGCACCTCAACTCAATCCAATTGATGGACCA

TTGCCTGAGGACAATGAGCCAAGTGGATATGCACAAACAGACTGTGTCCTTGAAGCAATG

GCTTTCCTTGAAGAGTCCCACCCAGGAATCTTTGAAAACTCGTGTCTTGAAACGATGGAA

GTCGTTCAACAAACAAGGGTGGACAAATTGACCCAAGGCCGTCAGACCTATGATTGGACA

TTAAACAGGAATCAGCCGGCTGCAACTGCATTAGCTAATACTATAGAGGTCTTCAGATCG

AACGGTCTTACGGCTAATGAATCAGGAAGGCTAATAGATTTTCTCAAGGATGTGATGGAA

TCAATGGATAAAGAGGAAATGGAAATAACAACGCATTTCCAAAGGAAAAGAAGAGTGAGA

GACAACATGACCAAGAAAATGGTCACACAAAGGACAATAGGAAAGAAGAAACAGAGGCTA

AACAAAAGGAGCTATCTAATAAGAGCATTGACACTGAACACAATGACAAAAGACGCCGAA

AGAGGCAAATTAAAGAGAAGGGCAATTGCGACACCCGGAATGCAAATCAGAGGGTTTGTG

TACTTTGTTGAGACATTAGCAAGGAGCATTTGTGAGAAGCTCGAACAATCTGGACTCCCA

GTTGGAGGCAATGAAAAGAAGGCTAAACTGGCAAATGTTGTGAGAAAAATGATGACTAAT

TCACAAGACACTGAGCTCTCTTTCACAATCACTGGAGACAACACTAAATGGAATGAAAAT

CAGAACCCTAGGATGTTTCTGGCAATGATAACATATATAACAAGGGACCAACCTGAATGG

TTCAGGAATGTCTTGAGCATTGCACCTATAATGTTCTCAAATAAAATGGCAAGACTAGGG

AAAGGATACATGTTCGAAAGTAAGAGCATGAAGCTTCGAACACAAATACCGGCAGAAATG

CTAGCAAGCATTGATCTGAAGTACTTCAATGAGTCAACAAGAAAGAAAATAGAGAAGATA

AGACCTCTTCTAATAGATGGTACAGCCTCATTAAGCCCTGGAATGATGATGGGCATGTTC

AACATGCTGAGTACAGTTTTGGGAGTTTCGATTCTAAATCTAGGGCAAAAGAGGTACACC

AAAACAACATACTGGTGGGACGGACTCCAATCCTCTGATGACTTTGCTCTCATAGTGAAT

GCTCCGAATCATGAGGGAATACAAGCAGGAGTAGACAGATTCTATAGAACCTGCAAGCTG

GTCGGAATCAACATGAGCAAAAAGAAGTCCTACATAAACAGGACAGGAACATTTGAATTC

ACAAGTTTTTTCTACCGCTATGGATTTGTAGCCAACTTCAGCATGGAGTTGCCCAGCTTT

GGAGTGTCTGGGATCAATGAATCTGCAGACATGAGCATTGGAGTAACAGTGATAAAGAAC

AACATGATCAACAATGATCTTGGACCAGCAACCGCCCAAATGGCTCTTCAGCTATTCATC

AAGGATTACAGATACACATATCGATGCCACAGAGGAGACACACAAATTCAGACAAGGAGG

TCATTCGAGCTGAAGAAGTTGTGGGAACAAACCCGCTCAAAAGCAGGACTGCTGGTCTCA

GATGGAGGACCAAATCTATATAATATCCGAAATCTCCACATTCCGGAAGTCTGCTTGAAA

TGGGAGCTAATGGACGAAGACTATCAGGGAAGGCTTTGTAACCCCCTGAATCCATTTGTC

AGCCACAAAGAGATAGAGTCTGTGAACAATGCTGTGGTGATGCCAGCTCATGGCCCAGCC

AAGAGCATGGAATATGATGCTGTTGCAACCACTCACTCCTGGATCCCTAAGAGGAACCGC

TCCATTCTCAATACAAGCCAAAGGGGAATCCTTGAAGACGAACAGATGTATCAAAAGTGC

TGCAATCTATTCGAGAAATTCTTCCCTAGCAGTTCATACAGGAGGCCGGTTGGAATTTCC

AGCATGGTGGAGGCCATGGTTTCTAGGGCCCGAATTGATGCGCGAATTGACTTCGAATCT

GGACGGATTAAGAAGGAGGAGTTTGCTGAGATCATGAAGATCTGTTCCACCATTGAAGAG

CTCAGACGGCAGAAATAG------------------------------------------

-

>A_gyrfalcon_Washington_41088-6_2014_EPI569388

------------------------ATGGATGTCAACCCGACTTTACTCTTCTTGAAAGTG

CCAGCGCAAAATGCTATAAGTACCACATTCCCTTATACTGGAGATCCTCCATACAGCCAT

GGAACAGGAACAGGATACACCATGGACACAGTCAACAGAACGCATCAATACTCAGAAAAG

GGAAAGTGGACAACAAACACCGAGACTGGAGCACCCCAACTCAACCCAATTGATGGACCA

TTACCTGAGGATAACGAGCCAAGCGGATATGCACAAACGGATTGTGTGTTGGAAGCAATG

GCTTTCCTTGAAGAGTCCCACCCAGGGATCTTTGAAAACTCATGTCTTGAAACAATGGAA

ATTGTTCAACAAACAAGAGTGGATAAACTGACCCAAGGTCGTCAGACCTATGACTGGACA

TTGAATAGAAACCAGCCGGCTGCAACTGCTTTAGCCAACACTATAGAAGTCTTCAGATCG

AACGGTCTAACAGCCAATGAATCAGGGAGACTGATAGATTTCCTCAAAGATGTGATGGAG

TCAATGGACAAAGAAGAAATGGAAATAACAACACATTTCCAAAGAAAGAGAAGAGTAAGA

GACAATATGACCAAGAAAATGGTCACACAAAGAACAATAGGGAAGAAAAAACAGAGACTG

AATAAGAAGAACTACTTGATAAGGGCACTGACACTGAACACAATGACAAAAGATGCAGAA

AGAGGCAAGTTGAAGAGGCGGGCAATTGCAACACCCGGGATGCAAATCAGAGGGTTCGTG

TACTTTGTCGAAACATTAGCGAGGAGCATCTGCGAGAAACTTGAGCAATCTGGGCTCCCT

GTTGGAGGAAATGAAAAAAAGGCTAAATTGGCAAATGTCGTGAGAAAGATGATGACTAAC

TCACAAGACACAGAGCTATCCTTTACAATTACTGGAGACAATACCAAGTGGAACGAGAAT

CAGAATCCTCGGATTTTTTTGGCAATGATAACATATATCACAAGAAATCAACCTGAGTGG

TTTAGAAATGTGTTAAGTATTGCCCCTATAATGTTCTCCAACAAAATGGCAAGATTAGGG

AAAGGATACATGTTCGAAAGTAAGAGCATGAAGCTACGGACACAAATACCTGCAGAAATG

CTTGCAACTATTGACCTGAAATATTTCAACGAATCGACAAGAAAGAAAATTGAGAAAATA

AGGCCTCTCCTAATAGAAGGGACAGCCTCGTTGAGTCCTGGAATGATGATGGGCATGTTC

AACATGCTGAGTACAGTCTTGGGAGTATCAATTCTAAATCTTGGCCAAAAGAGGTACACC

AAAACCACATACTGGTGGGACGGACTCCAATCCTCTGATGATTTCGCTCTCATAGTAAAT

GCACCGAATCATGAGGGGATACAGGCAGGAGTGGACAGGTTCTATAGGACTTGTAAATTG

GTTGGGATCAATATGAGTAAAAAGAAATCCTATATAAATCGGACAGGAACATTTGAATTC

ACAAGCTTTTTCTACCGTTATGGGTTTGTAGCCAACTTCAGTATGGAGCTGCCCAGCTTT

GGAGTTTCTGGGATTAATGAATCGGCTGACATGAGCATTGGAGTTACAGTAATAAAGAAT

AACATGATAAACAACGATCTTGGACCAGCAACAGCTCAAATGGCTCTTCAGCTATTTATC

AAGGACTACAGATATACATATCGATGCCACAGGGGTGATACACAAATACAAACGAGGAGA

TCATTCGAGCTAAAGAAGCTGTGGGAGCAGACCCGTTCAAAGGCAGGACTGTTGGTTTCA

GATGGAGGCCCAAACTTATACAATATACGGAATCTCCACATCCCAGAGGTCTGCTTGAAG

TGGGAACTGATGGATGAAGATTACCAGGGTAGACTTTGTAATTCCCTGAACCCCTTTGTC

AGTCATAAGGAAATTGAGTCCGTAAACAATGCTGTAGTGATGCCAGCTCATGGTCCGGCC

AAAAGCATGGAATATGATGCTGTTGCGACCACACACTCATGGGTCCCTAAAAGGAACCGT

TCCATTCTGAATACCAGTCAAAGAGGAATCCTTGAGGATGAACAGATGTATCAGAAGTGC

TGCAATCTATTTGAAAAATTCTTCCCTAGTAGCTCATACAGGAGACCAGTTGGAATCTCC

AGTATGGTGGAGGCCATGGTGTCTAGGGCCCGAATTGATGCGCGGATTGACTTCGAGTCT

GGTAGGATTAAGAAGGAAGAGTTTGCTGAGATCATGAAGATCTGTTCCACCATTGAAGAG

ATCAGACGGCAAAAACAGTGAATTTAGCTTGTCC--------------------------

-

>A_chicken_Hubei_ZYSJF38_2016_EPI895204

------------------------ATGGATGTCAATCCGACTTTACTTTTCTTGAAAGTG

CCAGTGCAAAATGCTATAAGTACCACTTTCCCTTATACTGGAGACCCTCCATACAGCCAT

GGAACAGGAACAGGATACACCATGGACACAGTCAACAGAACACATAAATACTCAGAAAAA

GGAAAGTGGACAACGAACACAGAGACTGGAGCACCCCAACTCAATCCAATTGATGGACCA

TTACCTGAGGACAACGAGCCAAGTGGGTATGCACAAACGGATTGTGTGTTGGAAGCAATG

GCTTTCCTTGAAGAATCTCATCCAGGGATCTTTGAGAACTCGTGTCTCGAAACGATGGAA

ATTGTCCAGCAAACAAGAGTGGATAAACTGACCCAAGGCCGCCAGACTTATGACTGGACG

TTGAATAGAAATCAGCCGGCTGCTACCGCATTGGCCAACACTATAGAGGTATTCAGATCG

AATGGCCTGACAGCCAATGAATCAGGAAGGTTGATCGATTTCCTCAAGGACGTGATGGAT

TCAATGGATAAGGAAGAAATGGAGATTACAACACATTTCCAGAGGAAGAGGAGAGTAAGG

GACAACATGACCAAGAAAATGGTCACACAGAGAACAATAGGGAAGAAAAAACAAAGACTG

AACAAAAGGAGCTACCTAATAAGAGCACTAACATTGAACACAATGACGAAGGATGCTGAA

AGAGGCAAGCTGAAAAGGAGGGCAATCGCAACACCCGGGATGCAAATCAGAGGATTCGTG

TATTTTGTAGAAGCACTAGCGAGGAGCATCTGTGAGAAACTTGAGCAATCTGGCCTCCCT

GTCGGAGGGAATGAGAAGAAAGCTAAATTGGCAAATGTTGTGAGGAAGATGATGACTAAT

TCACAGGATACAGAGCTCTCCTTCACAATTACTGGGGACAACACCAAATGGAATGAGAAT

CAAAACCCCCGGATGTTTCTGGCAATGATAACATACATCACAAGAAACCAGCCAGAATGG

TTTAGAAATGTCTTAAGCATTGCTCCTATAATGTTCTCAAACAAGATGGCGAGATTGGGA

AAAGGGTACATGTTCGAAAGTAAGAGTATGAGGTTACGGACACAAGTACCAGCGGAAATG

CTCGCAAATATTGACCTGAAATACTTCAACAAATCAACAAGAGAGAAAATCGAGAAAATA

AGACCTCTACTGATAGATGGCACAGCCTCATTGAGTCCTGGAATGATGATGGGCATGTTC

AACATGTTGAGTACAGTCTTAGGAGTTTCAATTCTGAATCTCGGGCAGAAGAAGTACACC

AAAACCACATATTGGTGGGACGGACTCCAATCCTCAGATGACTTTGCCCTCATAGTGAAT

GCACCGAATCATGAGGGAATACAGGCAGGAGTAGATAGGTTCTATAGAACCTGCAAATTA

GTTGGAATAAACATGAGCAAGAAGAAATCTTACATAAATCGGACAGGAACATTCGAATTC

ACAAGCTTTTTCTACCGCTATGGGTTCGTGGCTAACTTCAGTATGGAGTTGCCCAGTTTT

GGAGTGTCCGGGATTAATGAGTCAGCTGACATGAGCGTTGGTGTTACAGTAATAAAGAAC

AATATGATAAACAACGATCTTGGACCAGCAACAGCCCAAATGGCCCTTCAGCTATTTATC

AAAGATTACAGATACACATACCGATGTCACAGGGGCGATACGCAAATTCAAACGAGGAGA

GCATTCGAGCTGAAGAAGCTGTGGGAGCAGACCCGTTCGAAGGCAGGACTGCTGGTTTCA

GATGGAGGGCCAAACCTGTACAATATCCGGAACCTCCACATTCCAGAAGTCTGCTTGAAA

TGGGAATTGATGGATGAAGACTACCAAGGCAGGTTGTGTAATCCCATGAACCCGTTTGTC

AGTCATAAGGAAATTGATTCAGTCAACAATGCTGTGGTGATGCCAGCTCATGGCCCAGCC

AAAAGCATGGAGTATGATGCCGTTGCAACCACACATTCATGGATTCCTAAGAGGAATCGC

TCCATTCTCAACACCAGCCAAAGGGGGATTCTTGAGGATGAACAGATGTACCAGAAGTGC

TGCAACCTATTCGAAAAGTTCTTCCCCAGCAGTTCATACAGAAGGCCAGTTGGAATTTCC

AGCATGGTGGAGGCCATGGTGTCTAGGGCCCGAATTGATGCACGAATTGACTTCGAATCT

GGAAGGATTAAGAAAGAGGAGTTTGCTGAGATCATGAAGATCTGTTCCACCATTGAAGAG

CTCAGACGGCAAAAATGGTGAATTTA----------------------------------

-

>A_Hubei_29578_2016_x_PR8_CNIC-HB2957_1369963

------------------------ATGGATGTCAATCCGACCTTACTTTTCTTAAAAGTG

CCAGCACAAAATGCTATAAGCACAACTTTCCCTTATACTGGAGACCCTCCTTACAGCCAT

GGGACAGGAACAGGATACACCATGGATACTGTCAACAGGACACATCAGTACTCAGAAAAG

GGAAGATGGACAACAAACACCGAAACTGGAGCACCGCAACTCAACCCGATTGATGGGCCA

CTGCCAGAAGACAATGAACCAAGTGGTTATGCCCAAACAGATTGTGTATTGGAGGCGATG

GCTTTCCTTGAGGAATCCCATCCTGGTATTTTTGAAAACTCGTGTATTGAAACGATGGAG

GTTGTTCAGCAAACACGAGTAGACAAGCTGACACAAGGCCGACAGACCTATGACTGGACT

CTAAATAGAAACCAACCTGCTGCAACAGCATTGGCCAACACAATAGAAGTGTTCAGATCA

AATGGCCTCACGGCCAATGAGTCTGGAAGGCTCATAGACTTCCTTAAGGATGTAATGGAG

TCAATGAACAAAGAAGAAATGGGGATCACAACTCATTTTCAGAGAAAGAGACGGGTGAGA

GACAATATGACTAAGAAAATGATAACACAGAGAACAATGGGTAAAAAGAAGCAGAGATTG

AACAAAAGGAGTTATCTAATTAGAGCATTGACCCTGAACACAATGACCAAAGATGCTGAG

AGAGGGAAGCTAAAACGGAGAGCAATTGCAACCCCAGGGATGCAAATAAGGGGGTTTGTA

TACTTTGTTGAGACACTGGCAAGGAGTATATGTGAGAAACTTGAACAATCAGGGTTGCCA

GTTGGAGGCAATGAGAAGAAAGCAAAGTTGGCAAATGTTGTAAGGAAGATGATGACCAAT

TCTCAGGACACCGAACTTTCTTTCACCATCACTGGAGATAACACCAAATGGAACGAAAAT

CAGAATCCTCGGATGTTTTTGGCCATGATCACATATATGACCAGAAATCAGCCCGAATGG

TTCAGAAATGTTCTAAGTATTGCTCCAATAATGTTCTCAAACAAAATGGCGAGACTGGGA

AAAGGGTATATGTTTGAGAGCAAGAGTATGAAACTTAGAACTCAAATACCTGCAGAAATG

CTAGCAAGCATCGATTTGAAATATTTCAATGATTCAACAAGAAAGAAGATTGAAAAAATC

CGACCGCTCTTAATAGAGGGGACTGCATCATTGAGCCCTGGAATGATGATGGGCATGTTC

AATATGTTAAGCACTGTATTAGGCGTCTCCATCCTGAATCTTGGACAAAAGAGATACACC

AAGACTACTTACTGGTGGGATGGTCTTCAATCCTCTGACGATTTTGCTCTGATTGTGAAT

GCACCCAATCATGAAGGGATTCAAGCCGGAGTCGACAGGTTTTATCGAACCTGTAAGCTA

CTTGGAATCAATATGAGCAAGAAAAAGTCTTACATAAACAGAACAGGTACATTTGAATTC

ACAAGTTTTTTCTATCGTTATGGGTTTGTTGCCAATTTCAGCATGGAGCTTCCCAGTTTT

GGGGTGTCTGGGATCAACGAGTCAGCGGACATGAGTATTGGAGTTACTGTCATCAAAAAC

AATATGATAAACAATGATCTTGGTCCAGCAACAGCTCAAATGGCCCTTCAGTTGTTCATC

AAAGATTACAGGTACACGTACCGATGCCATATAGGTGACACACAAATACAAACCCGAAGA

TCATTTGAAATAAAGAAACTGTGGGAGCAAACCCGTTCCAAAGCTGGACTGCTGGTCTCC

GACGGAGGCCCAAATTTATACAACATTAGAAATCTCCACATTCCTGAAGTCTGCCTAAAA

TGGGAATTGATGGATGAGGATTACCAGGGGCGTTTATGCAACCCACTGAACCCATTTGTC

AGCCATAAAGAAATTGAATCAATGAACAATGCAGTGATGATGCCAGCACATGGTCCAGCC

AAAAACATGGAGTATGATGCTGTTGCAACAACACACTCCTGGATCCCCAAAAGAAATCGA

TCCATCTTGAATACAAGTCAAAGAGGAGTACTTGAGGATGAACAAATGTACCAAAGGTGC

TGCAATTTATTTGAAAAATTCTTCCCCAGCAGTTCATACAGAAGACCAGTCGGGATATCC

AGTATGGTGGAGGCTATGGTTTCCAGAGCCCGAATTGATGCACGGATTGATTTCGAATCT

GGAAGGATAAAGAAAGAAGAGTTCACTGAGATCATGAAGATCTGTTCCACCATTGAAGAG

CTCAGACGGCAAAAATAG------------------------------------------

-

>A_duck_Hyogo_1_2016_EPI866706

------------------------ATGGATGTCAATCCGACTTTACTTTTCTTGAAAATC

CCAGCGCAAAATGCTATAAGTACTACATTCCCTTATACTGGAGACCCTCCATACAGCCAT

GGAACGGGAACAGGATACACAATGGATACAGTCAACAGAACACATCAATATTCAGAAAAG

GGAAAATGGACAACAAACACAGAAACTGGAGCACCTCAACTCAACCCTATTGACGGACCA

CTACCTGAGGATAATGAACCGAGTGGATATGCACAAACAGATTGTGTATTGGAAGCAATG

GCCTTCCTTGAGGAATCTCATCCAGGAATATTTGAAAACTCATGTATTGAAACGATGGAA

ATTGTTCAGCAAACAAGAGTGGATAAACTGACCCAAGGTCGCCAGACCTATGACTGGACA

TTGAATAGAAACCAGCCGGCTGCAACTGCCTTGGCCAACACTATAGAGGTCTTCAGATCG

AACGGTCTAACGACCAATGAATCAGGAAGATTAATAGATTTCCTCAAGGACGTGATGGAA

TCAATGGATAAAGAAGGAATGGAAATAACGACACATTTCCAAAGGAAGAGAAGAGTGAGA

GACAACATGACCAAGAAAATGATCACACAGAGAACAATAGGAAAGAAAAAGCAAAGATTG

AACAAAAGAAGCTATCTAATAAGAGCATTGACATTGAACACAATGACGAAAGATGCAGAA

AGAGGCAAACTAAAGAGAAGGGCAATTGCAACACCCGGAATGCAAATTAGAGGGTTTGTG

TACTTTGTTGAAACACTAGCAAGAAGTATCTGTGAGAAACTTGAGCAATCTGGACTTCCT

GTTGGAGGGAATGAGAAGAAAGCTAAATTGGCGAATGTCGTGAGGAAAATGATGACTAAT

TCACAAGATACAGAAGTTTCCTTTACAATTACTGGAGACAACACCAAATGGAATGAGAAT

CAAAATCCTCGGATGTTTCTGGCAATGATAACATACATCACAAGGAACCAACCTGAATGG

TTCAGAAATGTTTTAAGCATTGCCCCTATAATGTTCTCAAACAAGATGGCGAGATTAGGG

AAAGGATACATGTTCGAAAGTAAAAGCATGAAACTACGGACACAGATACCGGCAGAAATG

CTTGCAAACATTGATCTGAAGTATTTCAATGAATCAACGAAAAAGAAAATCGAAAAAATA

AGACCTCTACTAATAGATGGCACAGCCTCTCTGAGCCCTGGAATGATGATGGGCATGTTC

AACATGCTGAGTACAGTATTAGGAGTCTCAATTCTGAATCTTGGACAAAAGAGGTACACC

AAAACCACGTACTGGTGGGATGGGCTCCAATCCTCTGACGATTTCGCTCTCATAGTGAAT

GCACCGAACCATGAGGGGATACAAGCAGGAGTAGATAGATTCTATAGGACTTGCAAATTA

GTTGGGATCAATATGAGCAAGAAAAAATCCTACATAAATCGGACAGGGACATTTGAGTTC

ACAAGCTTTTTCTATCGTTATGGATTTGTAGCCAATTTCAGTATGGAACTGCCCAGCTTT

GGAGTATCTGGGATTAATGAATCAGCTGACATGAGCATTGGTGTTACGGTGATAAAGAAC

AATATGATAAACAATGATCTTGGACCAGCAACAGCTCAGATGGCTCTTCAACTATTTATT

AAGGATTACAGATACACATATCGGTGCCACAGAGGTGATACACAAATTCAAACGAGGAGA

TCATTTGAACTGAAGAAGCTGTGGGAGCAAACCCGTTCTAAGGCAGGACTGCTGGTTTCA

GATGGAGGACCAAATCTATACAACATCCGGAATCTCCATATTCCGGAGGTATGTTTGAAA

TGGGAATTAATGGATGAAGATTACCAGGGCAGGCTGTGCAATCCTTTGAACCCATTTGTC

AGTCATAAGGAAATTGAATCCGTCAACAACGCTGTAGTGATGCCAGCTCATGGCCCAGCC

AAGAGCATGGAATATGATGCCGTTGCAACCACACACTCATGGATCCCTAAGAGGAACCGC

TCCATTCTTAACACCAGCCAAAGGGGAATTCTTGAGGATGAGCAGATGTATCAGAAGTGC

TGCAACCTATTCGAGAAATTCTTCCCTAGTAGCTCATACCGGAGACCAGTTGGAATCTCT

AGTATGGTGGAGGCCATGGTATCTAGGGCCCGAATTGATGCACGAATTGACTTCGAATCT

GGAAGGATTAAGAAGGAGGAGTTTGCTGAGATCATGAAGACCTGTTCCACCATTGAAGAG

CTCAGACGGCAAAAATAG------------------------------------------

-

>A_tundra_swan_Niigata_5112007_2016_EPI1184360

--------------AACCATTTGAATGGATGTCAATCCGACTTTACTTTTCTTGAAAATT

CCAGCGCAAAATGCTATAAGTACTACATTCCCTTATACTGGAGACCCTCCATACAGCCAT

GGAACGGGAACAGGATACACAATGGATACAGTCAACAGAACACATCAATATTCAGAAAAG

GGAAAATGGACAACAAACACAGAAACTGGAGCACCTCAACTCAACCCTATTGACGGACCA

TTACCTGAGGATAATGAACCGAGTGGATATGCACAAACAGATTGTGTATTGGAAGCAATG

GCCTTCCTTGAAGAATCTCATCCAGGAATATTTGAAAACTCATGTATTGAAACGATGGAA

ATTGTTCAGCAAACAAGAGTGGATAAACTGACCCAAGGTCGCCAGACCTATGACTGGACA

TTGAATAGAAACCAGCCGGCTGCAACTGCCTTGGCCAACACTATAGAGGTCTTCAGATCG

AACGGTCTAACGACCAATGAATCAGGAAGATTAATAGATTTCCTCAAGGACGTGATGGAA

TCAATGGATAAAGAAGGAATGGAAATAACGACACATTTCCAAAGGAAGAGAAGAGTGAGA

GACAACATGACCAAGAAAATGATCACACAGAGAACAATGGGAAAGAAAAAGCAAAGATTG

AACAAAAGAAGCTATCTAATAAGAGCATTGACATTGAACACAATGACGAAAGATGCAGAA

AGAGGCAAACTAAAGAGAAGGGCAATTGCAACACCCGGAATGCAAATTAGAGGGTTTGTG

TACTTTGTTGAAACGCTAGCAAGAAGTATCTGTGAGAAACTTGAGCAATCTGGACTTCCT

GTTGGAGGGAATGAGAAGAAAGCTAAATTGGCGAATGTCGTGAGGAAAATGATGACTAAT

TCACAAGATACAGAAGTTTCCTTTACAATTACTGGAGACAACACCAAATGGAATGAGAAT

CAAAATCCTCGGATGTTTCTGGCAATGATAACATACATCACAAGGAACCAACCTGAATGG

TTCAGAAATGTTTTAAGCATTGCCCCTATAATGTTCTCAAACAAGATGGCGAGATTAGGG

AAAGGATACATGTTCGAAAGTAAAAGCATGAAACTACGGACACAGATACCGGCAGAAATG

CTTGCAAACATTGATCTGAAGTATTTCAATGAATCAACGAAAAAGAAAATCGAAAAAATA

AGACCTCTACTAATAGATGGCACAGCCTCTCTGAGCCCTGGAATGATGATGGGCATGTTC

AACATGCTGAGTACAGTATTAGGAGTCTCAATTCTGAATCTTGGACAAAAGAGGTACACC

AAAACCACGTACTGGTGGGATGGGCTCCAATCCTCTGACGATTTCGCTCTCATAGTGAAT

GCGCCGAACCATGAGGGGATACAAGCAGGAGTAGATAGATTCTATAGGACTTGCAAATTA

GTTGGGATCAATATGAGCAAGAAAAAATCCTACATAAATCGGACAGGGACATTTGAGTTC

ACAAGTTTTTTCTACCGTTATGGATTTGTATCCAATTTCAGTATGGAACTGCCCAGCTTT

GGAGTATCTGGGATTAATGAATCAGCTGACATGAGCATTGGTGTTACAGTGATAAAGAAC

AATATGATAAACAATGATCTTGGACCAGCAACAGCTCAGATGGCTCTTCAACTATTTATT

AAGGATTACAGATACACATATCGGTGCCACAGAGGTGATACACAAATTCAAACGAGGAGA

TCATTTGAACTGGAGAAGCTGTGGGAGCAAACCCGTTCTAAGGCAGGACTGCTGGTTTCA

GATGGAGGACCAAATCTATACAACATCCGGAATCTCCATATTCCGGAGGTATGTTTGAAA

TGGGAATTAATGGATGAAGATTACCAGGGCAGGCTGTGCAATCCTTTGAACCCATTTGTC

AGTCATAAGGAAATTGAATCCGTCAACAACGCTGTAGTGATGCCAGCTCATGGCCCAGCC

AAGAGCATGGAATATGATGCCGTTGCAACCACACACTCATGGATCCCTAAGAGGAACCGC

TCCATTCTTAACACCAGCCAAAGGGGAATTCTTGAGGATGAGCAGATGTATCAGAAGTGC

TGCAACCTATTCGAGAAATTCTTCCCTAGTAGCTCATACCGGAGACCAGTTGGAATCTCT

AGTATGGTGGAGGCCATGGTATCTAGGGCCCGAATTGATGCACGAATTGACTTCGAATCT

GGAAGGATTAAGAAGGAGGAGTTTGCTGAGATCATGAAGACCTGTTCCACCATTGAAGAG

CTCAGACGGCAAAAATAGTGA---------------------------------------

-

>A_chicken_Vietnam_NCVD-15A59_2015_EPI895061

------------CAAACCATTTGAATGGATGTCAATCCGACTTTACTTTTCTTGAAAGTG

CCAGTGCAAAATGCTATAAGTACCACATTCCCTTATACTGGAGACCCTCCATACAGCCAT

GGAACAGGAACAGGGTACACCATGGACACAGTCAACAGAACACACCAATATTCAGAAAAG

GGGGAGTGGACAACAAACACAGAGACCGGGGCACCCCAACTCAACCCGATTGACGGACCA

TTACCTGAGGATAACGAGCCCAGTGGGTATGCGCAAACAGATTGTGTGTTAGAAGCGATG

GCTTTCCTTGAAGAATCCCACCCAGGAATCTTTGAAAATTCATGCATTGAAACGATGGAA

ATTGTCCAACAAACAAGAGTGGACAAACTGACCCAAGGTCGCCAGACTTATGACTGGACA

TTGAATAGAAACCAACCGGCTGCAACTGCCTTGGCCAACACTATAGAAATCTTCAGATCA

AACAGTCTGACAGCAAATGAATCGGGACGCCTAATAGATTTCCTCAAGGATGTGATGGAA

TCAATGGATAGGGAAGAAATGGAGATAACAACACATTTCCAGAGAAAGAGAAGAGTAAGG

GACAACATGACCAAGAAGATGGTAACACAAAGAACAATCGGGAAGAAAAAACAAAGGCTG

AACAAAAGGAGCTACCTGATAAGAGCGCTGACACTAAACACAATGACCAAAGATGCAGAA

AGAGGCAAATTGAAGAGGCGTGCAATTGCAACACCCGGAATGCAAATCAGAGGATTCGTG

TACTTCGTTGAAACACTAGCGAGGAGTATCTGTGAGAAACTTGAGCAATCTGGACTCCCA

GTCGGAGGGAATGAGAAGAAAGCTAAACTGGCAAATGTCGTGAGGAAGATGATGACCAAC

TCACAGGATACAGAACTCTCCTTTACAATTACCGGGGACAATACAAAATGGAATGAGAAT

CAAAATCCTAGGATGTTTCTGGCAATGATAACATACATCACAAGGAACCAGCCAGAATGG

TTTCGAAATGTTCTAAGCGTTGCCCCTATAATGTTTTCAAACAAAATGGCGAGGCTAGGG

AAAGGATACATGTTCGAAAGTAAGAGCATGAAGTTACGAACACAAATACCAGCAGAAATG

CTTGCAAACATTGACCTTAAATACTTCAATGAATCAACAAAAAAGAAAATTGAGAAGATA

AGACCTCTATTAATAGATGGTACAGCCTCATTGAGCCCTGGAATGATGATGGGCATGTTC

AACATGCTGAGTACAGTCCTAGGAGTTTCAATCCTAAATCTTGGACAGAAAAGATACACC

AAAACCACATATTGGTGGGACGGGCTCCAATCCTCTGATGATTTCGCTCTCATTGTAAAT

GCCCCGAATCATGAAGGAATACAAGCAGGGGTGGATAGGTTTTATAGAACTTGTAAACTA

GTTGGGATCAATATGAGCAAGAAGAAGTCTTACATAAATCGGACAGGGACATTTGAATTC

ACGAGCTTTTTCTACCGCTATGGATTCGTAGCCAATTTCAGTATGGAGCTGCCCAGTTTT

GGAGTGTCTGGAATTAATGAGTCGGCCGACATGAGCATTGGTGTTACAGTGATAAAGAAC

AATATGATAAACAACGACCTTGGGCCAGCAACAGCTCAGATGGCTCTTCAGCTATTCATC

AAGGACTACAGATACACATACCGATGCCACAGGGGAGATACGCAAATCCAGACAAGGAGA

TCATTCGAGCTAAAGAAGCTATGGGAACAAACCCGTTCAAAGGCAGGACTATTGGTTTCA

GATGGAGGACCAAATCTATACAATATCCGAAATCTCCATATTCCTGAGGTCTGCTTGAAA

TGGGAATTGATGGATGAAGACTACCAGGGTAGACTGTGCAATCCTCTGAATCCATTCGTC

AGCCATAGGGAAATTGAATCTGTCAACAATGCTACAGTGATGCCAGCTCATGGCCCGGCC

AAGAGTATGGAATATGATGCTGTAGCAACCACACATTCATGGATTCCTAAAAGGAATCGT

TCCATTCTCAACACGAGTCAAAGGGGAATTCTTGAGGATGAACAGATGTACCAGAAGTGC

TGCAATCTATTCGAGAAATTCTTCCCCAGTAGTTCGTATCGGAGACCAGTTGGAATTTCC

AGTATGGTGGAGGCCATGGTTTCTCGGGCTAGAATTGACGCACGAATTGATTTCGAGTCT

GGAAGGATTAAGAAAGAAGAGTTTGCTGAGATCATGAAGATCTGTTCCACCATTGAAGAG

CTCAGACGGCAAAGATAGTGAATTTAGCTTGTCCTTCGTGAAAAAATG------------

-

>A_duck_Wuhan_JXYFB22_2015_EPI683145

AGCGAAAGCAGGCAAACCATTTGAATGGATGTCAACCCGACTTTACTTTTCTTGAAAGTG

CCAGTGCAAAATGCTATAAGTACCACATTCCCTTATACTGGGGACCCTCCATACAGCCAT

GGAACAGGGACAGGGTACACCATGGACACAGTCAACAGAACACACCAATATTCAGAAAAG

GGGGAGTGGACAACAAACACAGAGACCGGGGCACCCCAACTCAACCCGATTGATGGACCA

TTACCTGAGGATAACGAGCCCAGTGGATATGCGCAAACAGATTGTGTGTTAGAAGCAATG

GCTTTCCTTGAAGAATCCCACCCAGGAATCTTTGAAAATTCGTGCCTTGAAACGATGGAA

ATTGTCCAACAAACAAGAGTGGACAAACTGACCCAAGGTCGCCAGACTTATGACTGGACA

TTGAATAGAAACCAACCGGCTGCAACTGCTTTGGCCAACACTATAGAAATCTTCAGATCA

AACGGTCTGACAGCAAATGAGTCGGGACGCCTAATAGATTTCCTCAAGGATGTGATGGAA

TCAATGGATAAGAAAGAAATGGAGATAACAACACATTTCCAGAGAAAGAGAAGAGTAAGG

GACAACATGACCAAGAAGATGGTAACGCAAAGAACAATCGGGAAGAAAAAACAAAGGCTG

AACAAGAGGAGCTACCTGATAAGAGCGCTGACACTGAACACAATGACCAAGGATGCAGAA

AGAGGCAAATTGAAGAGGCGTGCAATTGCAACACCCGGAATGCAAATAAGAGGATTCGTG

TACTTCGTTGAAACACTAGCGAGGAGTATCTGTGAGAAACTTGAGCAATCTGGACTCCCA

GTCGGAGGAAATGAGAAGAAAGCTAAACTGGCAAATGTCGTGAGGAAGATGATGACCAAC

TCACAGGATACTGAACTCTCCTTTACAATTACTGGGGACAATACAAAATGGAATGAGAAT

CAAAATCCTAGGATGTTTCTGGCAATGATAACGTACATCACAAGGAACCAGCCAGAATGG

TTTCGAAATGTTCTAAGCATTGCCCCTATAATGTTTTCAAACAAAATGGCGAGGCTAGGG

AAAGGATACATGTTCGAAAGTAAGAGCATGAAGTTACGAACACAAATATCAGCAGAAATG

CTTGCAAACATTGACCTTAAATACTTCAATGAATCAACAAAAAAGAAAATTGAGAAGATA

AGACCTCTATTGATAGATGGTACAGCCTCATTGAGCCCTGGAATGATGATGGGCATGTTC

AACATGCTGAGTACAGTCCTAGGGGTTTCAATCCTAAATCTTGGACAGAAAAGGTACACC

AAAACCACATATTGGTGGGACGGGCTCCAATCCTCTGATGATTTCGCTCTCATTATAAAT

GCCCCGAATCATGAAGGAATACAAGCAGGGGTGGATAGGTTTTATAGAACTTGTAAACTA

GTTGGGATCAATATGAGCAAGAAAAAGTCTTACATAAATCGGACAGGGACATTTGAATTC

ACGAGCTTTTTCTACCGCTATGGATTCGTAGCCAATTTCAGTATGGAGCTGCCCAGTTTT

GGAGTGTCTGGAATTAATGAGTCGGCCGACATGAGCATTGGTGTTACAGTGATAAAGAAC

AATATGATAAATAACGACCTTGGGCCAGCAACAGCTCAAATGGCTCTTCAGCTATTCATC

AAGGACTACAGATACACATACCGATGCCACAGGGGAGATACGCAAATCCAAACAAGGAGA

TCATTCGAGCTGAAGAAGCTATGGGAACAAACCCGTTCAAAGGCAGGACTATTGGTTTCA

GATGGAGGACCAAACCTATACAATATCCGAAATCTCCATATTCCCGAGGTCTGCTTGAAA

TGGGAATTAATGGATGAAGACTATCAGGGTAGACTGTGCAATCCTCTGAATCCATTCGTC

AGCCATAGGGAAATTGAATCTGTCAACAATGCTATAGTAATGCCAGCTCATGGCCCGGCC

AAGGGTATGGAATATGATGCCGTAGCAACCACACATTCATGGATTCCTAAAAGGAATCGT

TCCATTCTCAACACAAGTCAAAGGGGAATTCTTGAGGATGAACAGATGTACCAAAAGTGC

TGCAATCTATTCGAGAAGTTCTTTCCCAGTAGTTCGTATCGGAGACCAGTTGGAATTTCC

AGTATGGTGGAGGCCATGGTTTCTCGGGCTAGAATTGACGCACGAATTGATTTCGAGTCT

GGAAGGATTAAGAAAGAAGAGTTTGCTGAGATCATGAAGATCTGTTCCACCATTGAAGAG

CTCAGACGGCAAAAATAGTGAATTTGGCTTGACCTTCGTGACAAAATGCCTTGTTTCTAC

T

>A_goose_Hunan_116_2014_EPI958630

------------------------ATGGATGTCAATCCGACTTTACTTTTCTTGAAAGTG

CCAGTGCAAAATGCTATAAGTACCACATTCCCTTATACTGGAGACCCTCCATACAGCCAT

GGAACAGGAACAGGATACACCATGGACACAGTCAACAGAACACATCAATATTCAGAAAAG

GGAAAGTGGACAACGAACACAGAAACTGGAGCACCCCAACTCAATCCGATTGATGGACCA

TTACCTGAGGATAACGAGCCCAGTGGGTATGCGCAAACAGATTGTGTGTTAGAAGCAATG

GCTTTCCTTGAAGAATCCCACCCAGGAATCTTTGAAAATTCGTGCCTTGAAACGATGGAA

ATTGTCCAACAAACAAGAGTGGACAAACTGACCCAAGGTCGCCAGACTTATGACTGGACA

TTGAATAGAAACCAACCGGCTGCAACTGCTTTGGCCAACACTATAGAAATTTTCAGATCA

AACGGTCTGACAGCAAATGAATCGGGACGCCTAATAGATTTCCTCAAGGATGTGATGGAA

TCAATGGATAAGGAAGAAATGGAGATAACAACACATTTCCAGAGAAAGAGAAGAGTAAGG

GACAACATGACCAAGAAGATGGTAACACAAAGAACAATCGGGAAGAAAAAACAAAGGCTG

AACAAAAGGAGCTACCTGATAAGAGCGCTGACACTGAACACAATGACCAAGGATGCCGAA

AGAGGCAAATTGAAGAGGCGTGCAATTGCAACACCCGGAATGCAAATCAGAGGATTCGTG

TACTTCGTTGAAACACTAGCGAGGAGTATCTGTGAGAAACTTGAGCAATCTGGACTCCCA

GTCGGAGGGAATGAGAAGAAAGCTAAACTGGCAAACGTCGTGAGGAAGATGATGACCAAC

TCACAGGATACTGAACTCTCCTTTACAATTACTGGGGACAATACAAAATGGAATGAGAAT

CAAAATCCTAGGATGTTTCTGGCAATGATAACGTACATCACAAGGAATCAGCCAGAATGG

TTTCGAAATGTTCTAAGCATTGCCCCTATAATGTTTTCAAACAAAATGGCGAGGCTAGGG

AAAGGATACATGTTCGAAAGTAAGAGCATGAAGTTACGAACACAAATACCAGCAGAAATG

CTTGCAAACATTGACCTTAAATACTTCAATGAATCAACAAAAAAGAAAATTGAGAAGATA

AGACCTCTATTAATAGATGGTACAGCCTCATTGAGCCCTGGAATGATGATGGGCATGTTC

AACATGCTGAGTACAGTCCTAGGAGTTTCAATCCTAAATCTTGGACAAAAAAGATACACC

AAAACCACATATTGGTGGGACGGGCTCCAATCCTCTGATGATTTCGCTCTCATTGTAAAT

GCCCCGAATCATGAAGGAATACAAGCAGGGGTGGATAGGTTTTATAGAACTTGTAAACTA

GTTGGGATCAATATGAGCAAGAAGAAGTCTTACATAAATCGGACAGGGACATTTGAATTC

ACAAGCTTTTTCTACCGCTATGGATTCGTAGCCAATTTCAGTATGGAGCTGCCCAGTTTT

GGAGTGTCTGGAATTAATGAGTCGGCCGACATGAGCATTGGTGTTACAGTGATAAAGAAC

AATATGATAAACAACGACCTTGGGCCAGCAACAGCTCAGATGGCTCTTCAGCTATTCATC

AAGGACTACAGATACACATACCGATGCCACAGGGGAGATACGCAAATCCAAACTAGGAGA

TCATTCGAGCTGAAGAAGCTATGGGAACAAACCCGTTCAAAGGCAGGACTATTAGTTTCA

GATGGAGGACCAAATCTATACAATATCCGAAATCTCCATATTCCTGAAGTCTGCTTGAAA

TGGGAATTGATGGATGAAGACTACCAGGGTAGACTGTGCAATCCTCTGAATCCATTTGTC

AGTCATAGGGAAATTGAATCTGTCAACAATGCTATAGTAATGCCAGCTCATGGCCCGGCC

AAGAGTATGGAATATGATGCCGTAGCAACCACACATTCATGGATTCCTAAAAGGAATCGT

TCCATTCTCAACACAAGTCAAAGGGGAATTCTTGAGGATGAACAGATGTACCAAAAGTGC

TGCAATCTATTCGAGAAATTCTTTCCCAGTAGTTCGTATCGGAGACCAGTTGGAATTTCC

AGTATGGTGGAGGCCATGGTTTCTAGGGCTAGAATTGACGCACGAATTGATTTCGAGTCT

GGAAGGATTAAGAAAGAAGAGTTTGCTGAGATCATGAAGATCTGTTCCACCATTGAAGAA

CTCAGACGGCAAAAATAG------------------------------------------

-

>A_Guangdong_18SF020_2018_EPI1352811

------------------------ATGGATGTCAACCCGACTTTACTTTTCTTGAAAGTG

CCAGTGCAAAATGCTATAAGTACCACATTCCCTTATACTGGAGACCCTCCATACAGCCAT

GGAACAGGAACAGGGTACACCATGGACACAGTAAACAGAACACACCAATACTCAGAAAAA

GGGGAGTGGACAACAAACACAGAGACCGGGGCACCTCAACTCAACCCGATTGATGGACCA

TTGCCTGAGGATAACGAGCCCAGTGGGTATGCGCAAACAGATTGTGTATTAGAAGCAATG

GCTTTCCTTGAAGAATCCCACCCAGGAATCTTTGAAAATTCGTGCCTTGAAACGATGGAA

ATTGTCCAACAAACGAGAGTGGACAAACTGACCCAAGGTCGCCAGACTTATGACTGGACA

TTGAATAGAAACCAACCGGCTGCAACTGCTTTGGCCAACACTATAGAAGTCTTCAGATCA

AACGGTCTGACAGCGAATGAGTCCGGACGCCTAATAGATTTCCTCAAGGATGTGATGGAA

TCAATGGATAAGGAAGCAATGGAAATAACAACACATTTCCAGAGAAAGAGAAGAGTAAGG

GACAACATGACCAAGAAAATGGTGACGCAAAGAACAATTGGGAAGAAGAAGCAAAGGCTG

AACAAGAGGAGCTACCTGATAAGAGCGCTGACACTGAACACAATGACCAAGGATGCGGAA

AGAGGCAAATTGAAGAGGCGTGCAATTGCAACACCCGGAATGCAAATCAGAGGATTTGTA

TACTTCGTTGAAACACTAGCAAGGAGTATCTGTGAGAAACTTGAGCAATCTGGGCTCCCA

GTCGGAGGGAATGAGAAGAAAGCTAAGCTGGCAAACGTCGTGAGGAAGATGATGACCAAC

TCACAGGATACTGAACTCTCCTTTACAATTACTGGAGACAATACAAAATGGAATGAGAAT

CAAAATCCTAGGATGTTTCTGGCAATGATAACGTACATCACAAGGAACCAGCCAGAATGG

TTTCGAAATGTTCTAAGCATTGCCCCTATAATGTTCTCAAACAAAATGGCGAGGCTAGGG

AAAGGATACATGTTCGAAAGTAAGAGCATGAAGTTGCGAACACAAATACCAGCAGAGATG

CTTGCAAACATTGACCTTAAATACTTTAATGAATCGACAAAAAAGAAAATTGAGAAAATA

AGGCCTCTATTAATAGATGGTACAGCCTCATTGAGCCCTGGGATGATGATGGGCATGTTC

AACATGCTGAGTACAGTCCTAGGGGTTTCAATCCTAAATCTTGGACAGAAAAGATACACC

AAAACCACATATTGGTGGGACGGGCTTCAATCCTCTGATGATTTCGCTCTCATTATAAAT

GCCCCGAATCATGAAGGAATACAAGCAGGGGTGGATAGGTTTTATAGAACTTGTAAACTA

GTTGGGATCAATATGAGCAAAAAGAAGTCTTACATAAATCGGACTGGGACATTTGAATTT

ACGAGCTTTTTCTATCGTTATGGATTCGTAGCCAATTTCAGTATGGAACTGCCCAGTTTT

GGAGTGTCTGGAATTAATGAATCGGCCGACATGAGCATTGGTGTTACAGTGATAAAGAAC

AATATGATAAACAACGACCTTGGGCCAGCAACAGCTCAAATGGCTCTTCAGCTATTCATC

AAAGACTACAGATACACATACCGGTGCCACAGAGGAGATACTCAAATCCAAACTAGGAGA

TCATTCGAGCTGAAGAAGCTATGGGAACAAACCCGTTCAAAGGCAGGACTATTGGTTTCA

GATGGAGGACCAAACCTATACAATATCCGAAATCTCCATATTCCTGAGGTCTGCTTGAAA

TGGGAATTGATGGATGAAGACTACCAGGGTAGACTGTGCAATCCTCTGAACCCATTCGTC

AGCCATAGGGAAATTGAATCTGTCAACAATGCTATAGTAATGCCAGCTCATGGCCCGGCC

AAGGGCATGGAATATGATGCCGTAGCAACCACACATTCATGGATTCCTAAAAGGAATCGT

TCCATTCTTAACACGAGTCAGAGAGGGATTCTTGAGGATGAACAGATGTACCAAAAGTGC

TGCAATCTATTCGAGAAATTCTTCCCCAGCAGTTCGTATCGGAGACCGGTTGGAATTTCC

AGTATGGTGGAGGCCATGGTTTCTCGGGCCAGAATTGACGCACGAATTGATTTCGAGTCT

GGAAGGATTAAGAAGGAAGAGTTTGCTGAGATCATGAAGATCTGTTCCACCATTGAAGAG

CTCAGACGGCAAAAATAG------------------------------------------

-

>A_duck_Bangladesh_43127_2020_EPI1903531

------------------------ATGGATGTCAACCCGGCTTTACTTTTCTTGAAAGTG

CCAGTGCAAAATGCTATAAGTACCACATTCCCTTATACTGGAGACCCTCCATACAGCCAT

GGAACAGGAACAGGGTACACCATGGACACAGTAAACAGAACACACCAATACTCAGAAAGG

GGGGAGTGGACAACAAACACAGAGACCGGGGCACCCCAACTCAACCCGATTGATGGACCA

TTGCCTGAGGATAACGAGCCCAGTGGGTATGCGCAAACGGATTGTGTGTTAGAAGCAATG

GCTTTCCTTGAAGAATCCCACCCAGGAATCTTTGAAAATTCATGCCTTGAAACGATGGAA

ATTGTCCAACAAACGAGAGTGGACAGACTGACCCAAGGTCGCCAGACTTATGACTGGACA

TTGAATAGAAACCAGCCGGCTGCAACTGCTCTGGCCAACACTATAGAAGTCTTCAGATCA

AACGGTCTGACAGCGAATGAGTCCGGACGCCTAATAGATTTCCTCAAGGATGTGATAGAA

TCAATGGATAAAGAAGTAATGGAAATAACAACACATTTCCAGAGAAAGAGAAGAGTAAGG

GACAACATGACCAAGAAAATGGTAACGCAAAGAACAATCGGGAAGAAGAAGCAAAGGCTG

AACAAGAGGAACTACCTGATAAGAGCACTGACACTGAACACAATGACCAAGGATGCGGAA

AGAGGCAAATTGAAGAGGCGTGCAATTGCAACACCCGGAATGCAAATCAGAGGATTTGTA

TATTTCGTAGAAACACTAGCAAGGAGTATCTGTGAGAAACTTGAGCAATCTGGGCTCCCA

GTCGGAGGGAATGAGAAGAAAGCTAAACTGGCAAACGTCGTGAGGAAGATGATGACCAAC

TCACAGGATACTGAACTCTCCTTTACAATTACTGGAGACAATACAAAATGGAATGAGAAT

CAAAATCCTAGGATGTTTCTGGCAATGATAACGTACATCACAAGGAACCAGCCAGAATGG

TTCCGAAATGTTCTAAGCATTGCCCCTATAATGTTCTCAAACAAAATGGCGAGGCTCGGG

AAAGGATACATGTTCGAAAGTAAGAGCATGAAGTTGCGAACACAAATACCAGCAGAAATG

CTTGCAAACATTGACCTTAAATACTTCAATGAATCAACAAAAAAGAAAATTGAGAGAATA

AGACCCCTATTAATAGATGGTACAGCCTCATTGAGCCCTGGGATGATGATGGGCATGTTC

AACATGCTAAGTACAGTCCTAGGGGTTTCAATCCTAAATCTCGGACAGAAAAGATACACC

AAAACCACATATTGGTGGGACGGGCTTCAATCCTCTGATGACTTCGCTCTCATTATAAAT

GCCCCGAATCGTGAAGGGATACAAGCAGGAGTGGATAGGTTTTATAGAACTTGTAAACTA

GTTGGGATCAATATGAGCAAGAAGAAGTCTTACATAAATCGGACCGGGACATTTGAATTT

ACGAGCTTTTTCTATCGCTATGGATTCGTAGCCAATTTCAGTATGGAGCTGCCCAGTTTT

GGAGTATCTGGAATTAATGAATCAGCCGACATGAGCATTGGTGTTACAGTGATAAAGAAC

AATATGATAAACAATGACCTTGGGCCAGCAACAGCTCAGATGGCTCTTCAGCTATTCATC

AAAGATTACAGATACACATACCGATGCCACAGAGGAGATACTCAAATCCAAACGAGGAGA

TCATTCGAGCTGAAGAAGCTATGGGAACAAACCCGTTCAAAGGCAGGACTATTGGTTTCA

GATGGAGGACCAAACCTGTACAATATCCGAAATCTCCATATTCCTGAGGTCTGCTTGAAA

TGGGAATTGATGGATGAAGACTACCAGGGTAGACTGTGCAATCCTCTGAATCCATTCGTC

AACCATAGGGAAATTGAATCTGTCAACAATGCTGTAGTAATGCCAGCTCATGGTCCGGCC

AAGGGTATAGAATATGATGCCGTAGCAACCACACACTCATGGATTCCTAAAAGGAATCGT

TCCATTCTTAATACAAGTCAGAGGGGGATTCTTGAGGATGAACAGATGTATCAAAAGTGC

TGCAGTCTATTCGAGAAATTCTTCCCTAGTAGTTCGTATCGGAGACCAGTTGGAATTTCC

AGTATGGTGGAGGCCATGGTTTCTCGGGCCAGAATTGACGCACGAATTGATTTCGAGTCT

GGAAGGATTAAGAAGGAAGAGTTTGCTGAGATCATGAAGATCTGTTCCACCATTGAAGAG

CTCAGACGGCAAAAATAGTGAATTTGGCTTGTC---------------------------

-
