# Supplementary material for: Highly pathogenic avian influenza virus of the A/H5N8 subtype, clade 2.3.4.4b, caused outbreaks in Kazakhstan in 2020
Source: PeerJ. 2022 Mar 2;10:e13038. doi: 10.7717/peerj.13038 (PMC8898005; doi:10.7717/peerj.13038)
Supplement: Figure S4 [file peerj-10-13038-s005.docx]

**Fig.S4** Alignment of the nucleotide sequences for the PB2 segment used in this study

>A_goose_Kazakhstan_4-190-20-B-H5N8-1_2020_EPI1927648

AGCAAAAGCAGGTCAAATATATTCAATATGGAGAGAATAAAAGAACTAAGAGATTTGATG

TCGCAGTCTCGCACTCGCGAGATACTAACAAAAACCACTGTGGACCATATGGCCATAATA

AAGAAATACACATCAGGGAGACAGGAGAAGAACCCTGCCCTCAGGATGAAATGGATGATG

GCAATGAAATATCCTATTACAGCTGACAAAAGAATAATGGAGATGATCCCTGAAAGGAAT

GAGCAAGGTCAGACTCTCTGGAGCAAAACAAATGATGCTGGATCAGACAGAGTGATGGTC

TCACCTCTGGCTGTGACATGGTGGAATAGAAATGGGCCAACAACAAGTACAGTACACTAC

CCAAAAGTCTACAAAACCTACTTTGAAAAGGTAGAAAGGTTGAAACATGGAACCTTTGGT

CCTGTTCACTTTCGAAATCAGGTTAAGATACGCCGCAGGGTTGACATAAACCCGGGCCAT

GCAGATCTCAGTGCCAAAGAAGCACAGGATGTCATCATGGAGGTTGTTTTCCCAAATGAA

GTTGGAGCCAGGATCTTGACATCAGAATCACAATTAACAATAACAATGGAAAAGAAGGAG

GAACTTCAGGATTGCAAGATTGCTCCTTTGATGGTGGCATACATGTTGGAAAGAGAACTG

GTTCGCAAGACCAGATTTTTACCAGTAGCTGGCGGAACAAGCAGCGTATACATCGAGGTA

TTGCATTTGACTCAAGGGACCTGCTGGGAACAAATGTACACACCAGGAGGGGAGGTGAGA

AATGATGATGTTGATCAGAGTTTGATCATTGCTGCTAGAAATATAGTTAGGAGGGCAACA

GTATCAGCAGACCCATTGGCTTCGCTCTTGGAAATGTGCCACAGTACACAAATTGGTGGA

GTGAGGATGGTGGACATTCTTAGGCAGAACCCAACAGAGGAGCAAGCTGTGGATATATGC

AAAGCAGCAATGGGTTTAAGAATCAGTTCATCCTTCAGCTTTGGAGGTTTCACTTTCAAA

AGGACAAGTGGGTCGTCTGTCAAAAGAGAAGAAGAAATACTCACTGGCAACCTCCAAACA

CTGAAAGTAAGAATACATGAAGGATATGAGGAATTCACAATGGTTGGGCGAAGAGCTACA

GCCATTTTGAGGAAAGCAACCAGGAGACTGATCCAATTAATAGTGAGTGGACGAGACGAG

CAGTCAATCGCTGAAGCAATCATAGTGGCAATGGTTTTCTCACAGGAGGATTGCATGATA

AAAGCAGTACGAGGTGATTTGAATTTTGTCAACAGAGCGAATCAGCGGCTAAATCCTATG

CATCAACTTCTGAGGCATTTCCAAAAGGATGCAAAAGTGCTGTTTCAAAACTGGGGGATT

GAACCAATTGACAATGTAATGGGGATGATCGGGATACTGCCTGACATGACCCCCAGCACA

GAGGTGTCACTGAGAGGAGTGAGAGTCAGCAAAATGGGAGTGGATGAATATTCCAGTACT

GAGAGAGTGGTCGTGAGCATTGATCGCTTCTTGAGAGTCCGAGATCAGAGGGGAAATGTG

CTTCTGTCTCCTGAGGAAGTTAGTGAAACACATGGAACAGAGAAACTGACGATAACGTAT

TCATCGTCTATGATGTGGGAAATCAATGGTCCGGAATCCGTGCTAGTCAACACATATCAA

TGGATCATTAGAAATTGGGAAACTGTGAAGATTCAGTGGTCCCAGGACCCTACGATGTTG

TACAATAAGATGGAATTTGAGCCCTTCCAATCCTTGGTGCCCAAGGCTGCTAGAGGCCAG

TATAGTGGGTTTGTGAGGACATTATTCCAACAGATGCGTGATGTGTTGGGGACATTTGAC

ACTGTCCAAATAATAAAGCTCCTACCATTTGCAGCAGCCCCACCGGAACAGAGTAGGATG

CAATTTTCCTCTCTGACTGTGAACGTAAGAGGTTCAGGAATGAGAATACTTGTGAGGGGC

AACTCCCCTGTGTTCAACTATAATAAGGCCACCAAGAGACTCACAGTTCTTGGAAAGGAT

GCAGGCGCCTTGACAGAATATCCAGATGAGGGAACAGCAGGAGTGGAGTCTGCAGTATTA

AGAGGATTTCTAATTCTGGGCAAAGAGGACAAAAGATATGGACCAGCATTGAGCATCAAC

GAATTGAGCAATCTTGCGAAAGGGGAAAAGGCTAATGTGTTGATAGGACAAGGAGACGTG

GTGTTGGTAATGAAACGGAAACGGGACTCTAGCATACTTACTGACAGCCAGACAGCGACC

AAAAGAATTCGGATGGCCATCAATTAGTGTCGAATTGTTTAAAAACGACCTTGTTTCTAC

T---------------------

>A_chicken_Kazakhstan_220-B-2-H5N8-4_2020_EPI1927654

AGCAAAAGCAGGTCAAATATATTCAATATGGAGAGAATAAAAGAACTAAGAGATTTGATG

TCGCAGTCTCGCACTCGCGAGATACTAACAAAAACCACTGTGGACCATATGGCCATAATA

AAGAAATACACATCAGGGAGACAGGAGAAGAACCCTGCCCTCAGGATGAAATGGATGATG

GCAATGAAATATCCTATTACAGCTGACAAAAGAATAATGGAGATGATCCCTGAAAGGAAT

GAGCAAGGTCAGACTCTCTGGAGCAAAACAAATGATGCTGGATCAGACAGAGTGATGGTC

TCACCTCTGGCTGTGACATGGTGGAATAGAAATGGGCCAACAACAAGTACAGTACACTAC

CCAAAAGTCTACAAAACCTACTTTGAAAAGGTAGAAAGGTTGAAACATGGAACCTTTGGT

CCTGTTCACTTTCGAAATCAGGTTAAGATACGCCGCAGGGTTGACATAAACCCGGGCCAT

GCAGATCTCAGTGCCAAAGAAGCACAGGATGTCATCATGGAGGTTGTTTTCCCAAATGAA

GTTGGAGCCAGGATCTTGACATCAGAATCACAATTAACAATAACAATGGAAAAGAAGGAG

GAACTTCAGGATTGCAAGATTGCTCCTTTGATGGTGGCATACATGTTGGAAAGAGAACTG

GTTCGCAAGACCAGATTTTTACCAGTAGCTGGCGGAACAAGCAGCGTATACATCGAGGTA

TTGCATTTGACTCAAGGGACCTGCTGGGAACAAATGTACACACCAGGAGGGGAGGTGAGA

AATGATGATGTTGATCAGAGTTTGATCATTGCTGCTAGAAATATAGTTAGGAGGGCAACA

GTATCAGCAGACCCATTGGCTTCGCTCTTGGAAATGTGCCACAGTACACAAATTGGTGGA

GTGAGGATGGTGGACATTCTTAGGCAGAACCCAACAGAGGAGCAAGCTGTGGATATATGC

AAAGCAGCAATGGGTTTAAGAATCAGTTCATCCTTCAGCTTTGGAGGTTTCACTTTCAAA

AGGACAAGTGGGTCGTCTGTCAAAAGAGAAGAAGAAATACTCACTGGCAACCTCCAAACA

CTGAAAGTAAGAATACATGAAGGATATGAGGAATTCACAATGGTTGGGCGAAGAGCTACA

GCCATTTTGAGGAAAGCAACCAGGAGACTGATCCAATTAATAGTGAGTGGACGAGACGAG

CAGTCAATCGCTGAAGCAATCATAGTGGCAATGGTTTTCTCACAGGAGGATTGCATGATA

AAAGCAGTACGAGGTGATTTGAATTTTGTCAACAGAGCGAATCAGCGGCTAAATCCTATG

CATCAACTTCTGAGGCATTTCCAAAAGGATGCAAAAGTGCTGTTTCAAAACTGGGGGATT

GAACCAATTGACAATGTAATGGGGATGATCGGGATACTGCCTGACATGACCCCCAGCACA

GAGGTGTCACTGAGAGGAGTGAGAGTCAGCAAAATGGGAGTGGATGAATATTCCAGTACT

GAGAGAGTGGTCGTGAGCATTGATCGCTTCTTGAGAGTCCGAGATCAGAGGGGAAATGTG

CTTCTGTCTCCTGAGGAAGTTAGTGAAACACATGGAACAGAGAAACTGACGATAACGTAT

TCATCGTCTATGATGTGGGAAATCAATGGTCCGGAATCCGTGCTAGTCAACACATATCAA

TGGATCATTAGAAATTGGGAAACTGTGAAGATTCAGTGGTCCCAGGACCCTACGATGTTG

TACAATAAGATGGAATTTGAGCCCTTCCAATCCTTGGTGCCCAAGGCTGCTAGAGGCCAG

TATAGTGGGTTTGTGAGGACATTATTCCAACAGATGCGTGATGTGTTGGGGACATTTGAC

ACTGTCCAAATAATAAAGCTCCTACCATTTGCAGCAGCCCCACCGGAACAGAGTAGGATG

CAATTTTCCTCTCTGACTGTGAACGTAAGAGGTTCAGGAATGAGAATACTTGTGAGGGGC

AACTCCCCTGTGTTCAACTATAATAAGGCCACCAAGAGACTCACAGTTCTTGGAAAGGAT

GCAGGCGCCTTGACAGAATATCCAGATGAGGGAACAGCAGGAGTGGAGTCTGCAGTATTA

AGAGGATTTCTAATTCTGGGCAAAGAGGACAAAAGATATGGACCAGCATTGAGCATCAAC

GAATTGAGCAATCTTGCGAAAGGGGAAAAGGCTAATGTGTTGATAGGACAAGGAGACGTG

GTGTTGGTAATGAAACGGAAACGGGACTCTAGCATACTTACTGACAGCCAGACAGCGACC

AAAAGAATTCGGATGGCCATCAATTAGTGTCGAATTGTTTAAAAACGACCTTGTTTCTAC

T---------------------

>A_duck_Kazakhstan_12-20-B-Talg-11_2020_EPI1927660

AGCAAAAGCAGGTCAAATATATTCAATATGGAGAGAATAAAAGAACTAAGAGATTTGATG

TCGCAGTCTCGCACTCGCGAGATACTAACAAAAACCACTGTGGACCATATGGCCATAATA

AAGAAATACACATCAGGGAGACAGGAGAAGAACCCTGCCCTCAGGATGAAATGGATGATG

GCAATGAAATATCCTATTACAGCTGACAAAAGAATAATGGAGATGATCCCTGAAAGGAAT

GAGCAAGGTCAGACTCTCTGGAGCAAAACAAATGATGCTGGATCAGACAGAGTGATGGTC

TCACCTCTGGCTGTGACATGGTGGAATAGAAATGGGCCAACAACAAGTACAGTACACTAC

CCAAAAGTCTACAAAACCTACTTTGAAAAGGTAGAAAGGTTGAAACATGGAACCTTTGGT

CCTGTTCACTTTCGAAATCAGGTTAAGATACGTCGCAGGGTTGACATAAACCCGGGCCAT

GCAGATCTCAGTGCCAAAGAAGCACAGGATGTCATCATGGAGGTTGTTTTCCCAAATGAA

GTTGGAGCCAGGATCTTGACATCAGAATCACAATTAACAATAACAAGGGAAAAGAAGGAG

GAACTTCAGGATTGCAAGATTGCTCCTTTGATGGTGGCATACATGTTGGAAAGAGAACTG

GTTCGCAAGACCAGATTTTTACCAGTAGCTGGCGGAACAAGCAGCGTATACATCGAGGTA

TTGCATTTGACTCAAGGGACCTGCTGGGAACAAATGTACACACCAGGAGGGGAGGTGAGA

AATGATGATGTTGATCAGAGTTTGATCATTGCTGCTAGAAATATAGTTAGGAGGGCAACA

GTATCAGCAGACCCATTGGCTTCGCTCTTGGAAATGTGCCACAGTACACAAATTGGTGGA

GTGAGGATGGTGGACATTCTTAGGCAGAACCCAACAGAGGAGCAAGCTGTGGATATATGC

AAAGCAGCAATGGGTTTAAGAATCAGTTCATCCTTCAGCTTTGGAGGTTTCACTTTCAAA

AGGACAAGTGGGTCGTCTGTCAAAAGAGAAGAAGAAATACTCACTGGCAACCTCCAAACA

CTGAAAGTAAGAATACATGAAGGATATGAGGAATTCACAATGGTTGGGCGAAGAGCTACA

GCCATTTTGAGGAAAGCAACCAGGAGACTGATCCAATTAATAGTGAGTGGACGAGACGAG

CAGTCAATCGCTGAAGCAATCATAGTGGCGATGGTTTTCTCACAGGAGGATTGCATGATA

AAAGCAGTACGAGGTGATTTGAATTTTGTCAACAGAGCGAATCAGCGGCTAAATCCTATG

CATCAACTTCTGAGGCATTTCCAAAAGGATGCAAAAGTGCTGTTTCAAAACTGGGGGATT

GAACCAATTGACAATGTAATGGGGATGATCGGGATACTGCCTGACATGACCCCCAGCACA

GAGATGTCACTGAGAGGAGTGAGAGTCAGCAAAATGGGAGTGGATGAATATTCCAGTACT

GAGAGAGTGGTCGTGAGCATTGATCGCTTCTTGAGAGTCCGAGATCAGAGGGGAAATGTG

CTTCTGTCTCCTGAGGAAGTTAGTGAAACACATGGAACAGAGAAACTGACGATAACGTAT

TCATCGTCTATGATGTGGGAAATCAATGGTCCGGAATCCGTGCTAGTCAACACATATCAA

TGGATCATTAGAAATTGGGAAACTGTGAAGATTCAGTGGTCCCAGGACCCTACGATGTTG

TACAATAAGATGGAATTTGAGCCCTTCCAATCCTTGGTGCCCAAGGCTGCTAGAGGCCAG

TATAGTGGGTTTGTGAGGACATTATTCCAACAGATGCGTGATGTGTTGGGGACATTTGAC

ACTGTCCAAATAATAAAGCTCCTACCATTTGCAGCAGCCCCACCGGAACAGAGTAGGATG

CAATTTTCCTCTCTGACTGTGAACGTAAGAGGTTCAGGAATGAGAATACTTGTGAGGGGC

AACTCCCCTGTGTTCAACTATAATAAGGCCACCAAAAGACTCACAGTTCTTGGAAAGGAT

GCAGGCGCCTTGACAGAATATCCAGATGAGGGAACAGCAGGAGTGGAGTCTGCAGTATTA

AGAGGATTTCTAATTCTGGGCAAAGAGGACAAAAGATATGGACCAGCATTGAGCATCAAC

GAATTGAGCAATCTTGCGAAAGGGGAAAAGGCTAATGTGTTGATAGGACAAGGAGACGTG

GTGTTGGTAATGAAACGGAAACGGGACTCTAGCATACTTACTGACAGCCAGACAGCGACC

AAAAGAATTCGGATGGCCATCAATTAGTGTCGAATTGTTTAAAAACGACCTTGTTTCTAC

T---------------------

>A_goose_Kazakhstan_7-20-B-Talg-12_2020_EPI1927666

AGCAAAAGCAGGTCAAATATATTCAATATGGAGAGAATAAAAGAACTAAGAGATTTGATG

TCGCAGTCTCGCACTCGCGAGATACTAACAAAAACCACTGTGGACCATATGGCCATAATA

AAGAAATACACATCAGGGAGACAGGAGAAGAACCCTGCCCTCAGGATGAAATGGATGATG

GCAATGAAATATCCTATTACAGCTGACAAAAGAATAATGGAGATGATCCCTGAAAGGAAT

GAGCAAGGTCAGACTCTCTGGAGCAAAACAAATGATGCTGGATCAGACAGAGTGATGGTC

TCACCTCTGGCTGTGACATGGTGGAATAGAAATGGGCCAACAACAAGTACAGTACACTAC

CCAAAAGTCTACAAAACCTACTTTGAAAAGGTAGAAAGGTTGAAACATGGAACCTTTGGT

CCTGTTCACTTTCGAAATCAGGTTAAGATACGTCGCAGGGTTGACATAAACCCGGGCCAT

GCAGATCTCAGTGCCAAAGAAGCACAGGATGTCATCATGGAGGTTGTTTTCCCAAATGAA

GTTGGAGCCAGGATCTTGACATCAGAATCACAATTAACAATAACAAGGGAAAAGAAGGAG

GAACTTCAGGATTGCAAGATTGCTCCTTTGATGGTGGCATACATGTTGGAAAGAGAACTG

GTTCGCAAGACCAGATTTTTACCAGTAGCTGGCGGAACAAGCAGCGTATACATCGAGGTA

TTGCATTTGACTCAAGGGACCTGCTGGGAACAAATGTACACACCAGGAGGGGAGGTGAGA

AATGATGATGTTGATCAGAGTTTGATCATTGCTGCTAGAAATATAGTTAGGAGGGCAACA

GTATCAGCAGACCCATTGGCTTCGCTCTTGGAAATGTGCCACAGTACACAAATTGGTGGA

GTGAGGATGGTGGACATTCTTAGGCAGAACCCAACAGAGGAGCAAGCTGTGGATATATGC

AAAGCAGCAATGGGTTTAAGAATCAGTTCATCCTTCAGCTTTGGAGGTTTCACTTTCAAA

AGGACAAGTGGGTCGTCTGTCAAAAGAGAAGAAGAAATACTCACTGGCAACCTCCAAACA

CTGAAAGTAAGAATACATGAAGGATATGAGGAATTCACAATGGTTGGGCGAAGAGCTACA

GCCATTTTGAGGAAAGCAACCAGGAGACTGATCCAATTAATAGTGAGTGGACGAGACGAG

CAGTCAATCGCTGAAGCAATCATAGTGGCGATGGTTTTCTCACAGGAGGATTGCATGATA

AAAGCAGTACGAGGTGATTTGAATTTTGTCAACAGAGCGAATCAGCGGCTAAATCCTATG

CATCAACTTCTGAGGCATTTCCAAAAGGATGCAAAAGTGCTGTTTCAAAACTGGGGGATT

GAACCAATTGACAATGTAATGGGGATGATCGGGATACTGCCTGACATGACCCCCAGCACA

GAGATGTCACTGAGAGGAGTGAGAGTCAGCAAAATGGGAGTGGATGAATATTCCAGTACT

GAGAGAGTGGTCGTGAGCATTGATCGCTTCTTGAGAGTCCGAGATCAGAGGGGAAATGTG

CTTCTGTCTCCTGAGGAAGTTAGTGAAACACATGGAACAGAGAAACTGACGATAACGTAT

TCATCGTCTATGATGTGGGAAATCAATGGTCCGGAATCCGTGCTAGTCAACACATATCAA

TGGATCATTAGAAATTGGGAAACTGTGAAGATTCAGTGGTCCCAGGACCCTACGATGTTG

TACAATAAGATGGAATTTGAGCCCTTCCAATCCTTGGTGCCCAAGGCTGCTAGAGGCCAG

TATAGTGGGTTTGTGAGGACATTATTCCAACAGATGCGTGATGTGTTGGGGACATTTGAC

ACTGTCCAAATAATAAAGCTCCTACCATTTGCAGCAGCCCCACCGGAACAGAGTAGGATG

CAATTTTCCTCTCTGACTGTGAACGTAAGAGGTTCAGGAATGAGAATACTTGTGAGGGGC

AACTCCCCTGTGTTCAACTATAATAAGGCCACCAAAAGACTCACAGTTCTTGGAAAGGAT

GCAGGCGCCTTGACAGAATATCCAGATGAGGGAACAGCAGGAGTGGAGTCTGCAGTATTA

AGAGGATTTCTAATTCTGGGCAAAGAGGACAAAAGATATGGACCAGCATTGAGCATCAAC

GAATTGAGCAATCTTGCGAAAGGGGAAAAGGCTAATGTGTTGATAGGACAAGGAGACGTG

GTGTTGGTAATGAAACGGAAACGGGACTCTAGCATACTTACTGACAGCCAGACAGCGACC

AAAAGAATTCGGATGGCCATCAATTAGTGTCGAATTGTTTAAAAACGACCTTGTTTCTAC

T---------------------

>A_swan_Kazakhstan_9-20-B-Talg-39_2020_EPI1927694

AGCAAAAGCAGGTCAAATATATTCAATATGGAGAGAATAAAAGAACTAAGAGATTTGATG

TCGCAGTCTCGCACTCGCGAGATACTAACAAAAACCACTGTGGACCATATGGCCATAATA

AAGAAATACACATCAGGGAGACAGGAGAAGAACCCTGCCCTCAGGATGAAATGGATGATG

GCAATGAAATATCCTATTACAGCTGACAAAAGAATAATGGAGATGATCCCTGAAAGGAAT

GAGCAAGGTCAGACTCTCTGGAGCAAAACAAATGATGCTGGATCAGACAGAGTGATGGTC

TCACCTCTGGCTGTGACATGGTGGAATAGAAATGGGCCAACAACAAGTACAGTACACTAC

CCAAAAGTCTACAAAACCTACTTTGAAAAGGTAGAAAGGTTGAAACATGGAACCTTTGGT

CCTGTTCACTTTCGAAATCAGGTTAAGATACGCCGCAGGGTTGACATAAACCCGGGCCAT

GCAGATCTCAGTGCCAAAGAAGCACAGGATGTCATCATGGAGGTTGTTTTCCCAAATGAA

GTTGGAGCCAGGATCTTGACATCAGAATCACAATTAACAATAACAAGGGAAAAGAAGGAG

GAACTTCAGGATTGCAAGATTGCTCCTTTGATGGTGGCATACATGTTGGAAAGAGAACTG

GTTCGCAAGACCAGATTTTTACCAGTAGCTGGCGGAACAAGCAGCGTATACATCGAGGTA

TTGCATTTGACTCAAGGGACCTGCTGGGAACAAATGTACACACCAGGAGGGGAGGTGAGA

AATGATGATGTTGATCAGAGTTTGATCATTGCTGCTAGAAATATAGTTAGGAGGGCAACA

GTATCAGCAGACCCATTGGCTTCGCTCTTGGAAATGTGCCACAGTACACAAATTGGTGGA

GTGAGGATGGTGGACATTCTTAGGCAGAACCCAACAGAGGAGCAAGCTGTGGATATATGC

AAAGCAGCAATGGGTTTAAGAATCAGTTCATCCTTCAGCTTTGGAGGTTTCACTTTCAAA

AGGACAAGTGGGTCGTCTGTCAAAAGAGAAGAAGAAATACTCACTGGCAACCTCCAAACA

CTGAAAGTAAGAATACATGAAGGATATGAGGAATTCACAATGGTTGGGCGAAGAGCTACA

GCCATTTTGAGGAAAGCAACCAGGAGACTGATCCAATTAATAGTGAGTGGACGAGACGAG

CAGTCAATCGCTGAAGCAATCATAGTGGCAATGGTTTTCTCACAGGAGGATTGCATGATA

AAAGCAGTACGAGGTGATTTGAATTTTGTCAACAGAGCGAATCAGCGGCTAAATCCTATG

CATCAACTTCTGAGGCATTTCCAAAAGGATGCAAAAGTGCTGTTTCAAAACTGGGGGATT

GAACCAATTGACAATGTAATGGGGATGATCGGGATACTGCCTGACATGACCCCCAGCACA

GAGATGTCACTGAGAGGGGTGAGAGTCAGCAAAATGGGAGTGGATGAATATTCCAGTACT

GAGAGAGTGGTCGTGAGCATTGATCGCTTCTTGAGAGTCCGAGATCAGAGGGGAAATGTG

CTTCTGTCTCCTGAGGAAGTTAGTGAAACACATGGAACAGAGAAACTGACGATAACGTAT

TCATCGTCTATGATGTGGGAAATCAATGGTCCGGAATCCGTGCTAGTCAACACATATCAA

TGGATCATTAGAAATTGGGAAACTGTGAAGATTCAGTGGTCCCAGGACCCTACGATGTTG

TACAATAAGATGGAATTTGAGCCCTTTCAATCCTTGGTGCCCAAGGCTGCTAGAGGCCAG

TATAGTGGGTTTGTGAGGACATTATTCCAACAGATGCGTGATGTGTTGGGGACATTTGAC

ACTGTCCAAATAATAAAGCTCCTACCATTTGCAGCAGCCCCACCGGAACAGAGTAGGATG

CAATTTTCCTCTCTGACTGTGAACGTAAGAGGTTCAGGAATGAGAATACTTGTGAGGGGC

AACTCCCCTGTGTTCAACTATAATAAGGCCACCAAGAGACTCACAGTTCTTGGAAAGGAT

GCAGGCGCCTTGACAGAATATCCAGATGAGGGAACAGCAGGAGTGGAGTCTGCAGTATTA

AGAGGATTTCTAATTCTGGGCAAAGAGGACAAAAGATATGGACCAGCATTGAGCATCAAC

GAATTGAGCAATCTTGCGAAAGGGGAAAAGGCTAATGTGTTGATAGGACAAGGAGACGTG

GTGTTGGTAATGAAACGGAAACGGGACTCTAGCATACTTACTGACAGCCAGACAGCGACC

AAAAGAATTCGGATGGCCATCAATTAGTGTCGAATTGTTTAAAAACGACCTTGTTTCTAC

T---------------------

>A_chicken_Kazakhstan_12-20-B-Talg-45_2020_EPI1927700

AGCAAAAGCAGGTCAAATATATTCAATATGGAGAGAATAAAAGAACTAAGAGATTTGATG

TCGCAGTCTCGCACTCGCGAGATACTAACAAAAACCACTGTGGACCATATGGCCATAATA

AAGAAATACACATCAGGAAGACAGGAGAAGAACCCTGCCCTCAGGATGAAATGGATGATG

GCAATGAAATATCCTATTACAGCTGACAAAAGAATAATGGAGATGATCCCTGAAAGGAAT

GAGCAAGGTCAGACTCTCTGGAGCAAAACAAATGATGCTGGATCAGACAGAGTGATGGTC

TCACCTCTGGCTGTGACATGGTGGAATAGGAATGGGCCAACAACAAGTACAGTACACTAC

CCAAAAGTCTACAAAACCTACTTTGAAAAGGTAGAAAGGTTGAAACATGGAACCTTTGGT

CCTGTTCACTTTCGAAATCAGGTTAAGATACGCCGCAGGGTTGACATAAACCCGGGCCAT

GCAGATCTCAGTGCCAAAGAAGCACAGGATGTCATCATGGAGGTTGTTTTCCCAAATGAA

GTTGGAGCCAGGATCTTGACATCAGAATCACAATTAACAATAACAAGGGAAAAGAAGGAG

GAACTTCAGGATTGCAAGATTGCTCCTTTGATGGTGGCATACATGTTGGAAAGAGAACTG

GTTCGCAAGACCAGATTTTTACCAGTAGCTGGCGGAACAAGCAGCGTATACATCGAGGTA

TTGCATTTGACTCAAGGGACCTGCTGGGAACAAATGTACACACCAGGAGGGGAGGTGAGA

AATGATGATGTTGATCAGAGTTTGATCATTGCTGCTAGAAATATAGTTAGGAGGGCAACA

GTATCAGCAGACCCATTGGCTTCGCTCTTGGAAATGTGCCACAGTACACAAATTGGTGGA

GTAAGGATGGTGGACATTCTTAGGCAGAACCCAACAGAGGAGCAAGCTGTGGATATATGC

AAAGCAGCAATGGGTTTAAGAATCAGTTCATCCTTCAGCTTTGGAGGTTTCACTTTCAAA

AGGACAAGTGGGTCGTCTGTCAAAAGAGAAGAAGAAATACTCACTGGCAACCTCCAAACA

CTGAAAGTTAGAATACATGAAGGATATGAGGAATTCACAATGGTTGGGCGAAGAGCTACA

GCCATTTTGAGGAAAGCAACCAGGAGACTGATCCAATTAATAGTGAGTGGAAGAGACGAG

CAGTCAATCGCTGAAGCAATCATAGTGGCAATGGTTTTCTCACAGGAGGATTGCATGATA

AAAGCAGTACGAGGTGATTTGAATTTTGTCAACAGAGCGAATCAGCGGCTAAATCCTATG

CATCAACTTCTGAGGCATTTCCAAAAGGATGCAAAAGTGCTGTTTCAAAACTGGGGGATT

GAACCAATTGACAATGTAATGGGGATGATCGGGATACTGCCAGACATGACCCCCAGCACA

GAGATGTCACTGAGAGGAGTGAGAGTCAGCAAAATGGGAGTGGATGAATATTCCAGTACT

GAGAGAGTGGTCGTGAGCATTGATCGCTTCTTGAGAGTCCGAGATCAGAGGGGAAATGTG

CTTCTGTCTCCTGAGGAAGTTAGTGAAACACATGGAACAGAGAAACTGACGATAACGTAT

TCATCGTCTATGATGTGGGAAATCAATGGTCCGGAATCCGTGCTAGTCAACACATATCAA

TGGATCATTAGAAGTTGGGAAACTGTGAAGATTCAATGGTCCCAGGACCCTACGATGTTG

TACAATAAGATGGAATTTGAGCCCTTCCAATCCTTGGTGCCCAAGGCTGCTAGAGGCCAG

TATAGTGGGTTTGTGAGGACATTATTCCAACAGATGCGTGATGTGTTGGGGACATTTGAC

ACTGTCCAAATAATAAAGCTCCTACCATTTGCAGCAGCCCCACCGGAACAGAGTAGGATG

CAATTTTCCTCTCTGACTGTGAACGTAAGAGGTTCAGGAATGAGAATACTTGTGAGGGGC

AACTCCCCTGTGTTCAACTATAATAAGGCCACCAAGAGACTCACAGTTCTTGGAAAGGAT

GCAGGCGCCTTGACAGAATATCCAGATGAGGGAACAGCAGGAGTGGAGTCTGCAGTATTA

AGAGGATTTCTAATTCTGGGCAAAGAGGACAAAAGATATGGACCAGCATTGAGCATCAAC

GAATTGAGCAATCTTGCGAAAGGGGAAAAGGCTAATGTGTTGATAGGACAAGGAGACGTG

GTGTTGGTAATGAAACGGAAACGGGACTCTAGCATACTTACTGACAGCCAGACAGCGACC

AAAAGAATTCGGATGGCCATCAATTAGTGTCGAATTGTTTAAAAACGACCTTGTTTCTAC

T---------------------

>A_crow_Kazakhstan_15-20-B-Talg-4_2020_EPI1927706

AGCRAAAGCAGGTCAAATATATTCAATATGGAGAGAATAAAAGAACTAAGAGATTTGATG

TCGCAGTCTCGCACTCGCGAGATACTAACAAAAACCACTGTGGACCATATGGCCATAATA

AAGAAATACACATCAGGGAGACAGGAGAAGAACCCTGCCCTCAGGATGAAATGGATGATG

GCAATGAAATATCCTATTACAGCTGACAAAAGAATAATGGAGATGATCCCTGAAAGGAAT

GAGCAAGGTCAGACTCTCTGGAGCAAAACAAATGATGCTGGATCAGACAGAGTGATGGTC

TCACCTCTGGCTGTGACATGGTGGAATAGAAATGGGCCAACAACAAGTACAGTACACTAC

CCAAAAGTCTACAAAACCTACTTTGAAAAGGTAGAAAGGTTGAAACATGGAACCTTTGGT

CCTGTTCACTTTCGAAATCAGGTTAAGATACGTCGCAGGGTTGACATAAACCCGGGCCAT

GCAGATCTCAGTGCCAAAGAAGCACAGGATGTCATCATGGAGGTTGTTTTCCCAAATGAA

GTTGGAGCCAGGATCTTGACATCAGAATCACAATTAACAATAACAAGGGAAAAGAAGGAG

GAACTTCAGGATTGCAAGATTGCTCCTTTGATGGTGGCATACATGTTGGAAAGAGAACTG

GTTCGCAAGACCAGATTTTTACCAGTAGCTGGCGGAACAAGCAGCGTATACATCGAGGTA

TTGCATTTGACTCAAGGGACCTGCTGGGAACAAATGTACACACCAGGAGGGGAGGTGAGA

AATGATGATGTTGATCAGAGTTTGATCATTGCTGCTAGAAATATAGTTAGGAGGGCAACA

GTATCAGCAGACCCATTGGCTTCGCTCTTGGAAATGTGCCACAGTACACAAATTGGTGGA

GTGAGGATGGTGGACAYTCTTAGGCAGAACCCAACAGAGGAGCAAGCTGTGGATATATGC

AAAGCAGCAATGGGTTTAAGAATCAGTTCATCCTTCAGCTTTGGAGGTTTCACTTTCAAA

AGGACAAGTGGGTCGTCTGTCAAAAGAGAAGAAGAAATACTCACTGGCAACCTCCAAACA

CTGAAAGTAAGAATACATGAAGGATATGAGGAATTCACAATGGTTGGGCGAAGAGCTACA

GCCATTTTGAGGAAAGCAACCAGGAGACTGATCCAATTAATAGTGAGTGGACGAGACGAG

CAGTCAATCGCTGAAGCAATCATAGTGGCGATGGTTTTCTCACAGGAGGATTGCATGATA

AAAGCAGTACGAGGTGATTTGAATTTTGTCAACAGAGCGAATCAGCGGCTAAATCCTATG

CATCAACTTCTGAGGCATTTCCAAAAGGATGCAAAAGTGCTGTTTCAAAACTGGGGGATT

GAACCAATTGACAATGTAATGGGGATGATCGGGATACTGCCTGACATGACCCCCAGCACA

GAGATGTCACTGAGAGGAGTGAGAGTCAGCAAAATGGGAGTGGATGAATATTCCAGTACT

GAGAGAGTGGTCGTGAGCATTGATCGCTTCTTGAGAGTCCGAGATCAGAGGGGAAATGTG

CTTCTGTCTCCTGAGGAAGTTAGTGAAACACATGGAACAGAGAAACTGACGATAACGTAT

TCATCGTCTATGATGTGGGAAATCAATGGTCCGGAATCCGTGCTAGTCAACACATATCAA

TGGATCATTAGAAATTGGGAAACTGTGAAGATTCAGTGGTCCCAGGACCCTACGATGTTG

TACAATAAGATGGAATTTGAGCCCTTCCAATCCTTGGTGCCCAAGGCTGCTAGAGGCCAG

TATAGTGGGTTTGTGAGGACATTATTCCAACAGATGCGTGATGTGTTGGGGACATTTGAC

ACTGTCCAAATAATAAAGCTCCTACCATTTGCAGCAGCCCCACCGGAACAGAGTAGGATG

CAATTTTCCTCTCTGACTGTGAACGTAAGAGGTTCAGGAATGAGAATACTTGTGAGGGGC

AACTCCCCTGTGTTCAACTATAATAAGGCCACCAAGAGACTCACAGTTCTTGGAAAGGAT

GCAGGCGCCTTGACAGAATATCCAGATGAGGGAACAGCAGGAGTGGAGTCTGCAGTATTA

AGAGGATTTCTAATTCTGGGCAAAGAGGACAAAAGATATGGACCAGCATTGAGCATCAAC

GAATTGAGCAATCTTGCGAAAGGGGAAAAGGCTAATGTGTTGATAGGACAAGGAGACGTG

GTGTTGGTAATGAAACGGAAACGGGACTCTAGCATACTTACTGACAGCCAGACAGCGACC

AAAAGAATTCGGATGGCCATCAATTAGTGTCGAATTGTTTAAAAACGACCTTGTTTCTAC

T---------------------

>A_swan_Kazakhstan_1-267-20-B-Talg-52_2020_EPI1927712

AGCAAAAGCAGGTCAAATATATTCAATATGGAGAGAATAAAAGAACTAAGAGATTTGATG

TCGCAGTCTCGCACTCGCGAGATACTAACAAAAACCACTGTGGACCATATGGCCATAATA

AAGAAATACACATCAGGAAGACAGGAGAAGAACCCTGCCCTCAGGATGAAATGGATGATG

GCAATGAAATATCCTATTACAGCTGACAAAAGAATAATGGAGATGATCCCTGAAAGGAAT

GAGCAAGGTCAGACTCTCTGGAGCAAAACAAATGATGCTGGATCAGACAGAGTGATGGTC

TCACCTCTGGCTGTGACATGGTGGAATAGAAATGGGCCAACAACAAGTACAGTACACTAC

CCAAAAGTCTACAAAACCTACTTTGAAAAGGTAGAAAGGTTGAAACATGGAACCTTTGGT

CCTGTTCACTTTCGAAATCAGGTTAAGATACGCCGCAGGGTTGACATAAACCCGGGCCAT

GCAGATCTCAGTGCCAAAGAAGCACAGGATGTCATCATGGAGGTTGTTTTCCCAAATGAA

GTTGGAGCCAGGATCTTGACATCAGAATCACAATTAACAATAACAAGGGAAAAGAAGGAG

GAACTTCAGGATTGCAAGATTGCTCCTTTGATGGTGGCATACATGTTGGAAAGAGAACTG

GTTCGCAAGACCAGATTTTTACCAGTAGCTGGCGGAACAAGCAGCGTATACATCGAGGTA

TTGCATTTGACTCAAGGGACCTGCTGGGAACAAATGTACACACCAGGAGGGGAGGTGAGA

AATGATGATGTTGATCAGAGTTTGATCATTGCTGCTAGAAATATAGTTAGGAGGGCAACA

GTATCAGCAGACCCATTGGCTTCGCTCTTGGAGATGTGCCACAGTACACAAATTGGTGGA

GTAAGGATGGTGGACATTCTTAGGCAGAACCCAACAGAGGAGCAAGCTGTGGATATATGC

AAAGCAGCAATGGGTTTAAGAATCAGTTCATCCTTCAGCTTTGGAGGTTTCACTTTCAAA

AGGACAAGTGGGTCGTCTGTCAAAAGAGAAGAAGAAATACTCACTGGCAACCTCCAAACA

CTGAAAGTAAGAATACATGAAGGATATGAGGAATTCACAATGGTTGGGCGAAGAGCTACA

GCCATTTTGAGGAAAGCAACCAGGAGACTGATCCAATTAATAGTGAGTGGAAGAGACGAG

CAGTCAATCGCTGAAGCAATCATAGTGGCAATGGTTTTCTCACAGGAGGATTGCATGATA

AAAGCAGTACGAGGTGATTTGAATTTTGTCAACAGAGCGAATCAGCGGCTAAATCCTATG

CACCAACTTCTGAGGCATTTCCAAAAGGATGCAAAAGTGCTGTTTCAAAACTGGGGGATT

GAACCAATTGACAATGTAATGGGGATGATCGGGATACTGCCTGACATGACCCCCAGCACA

GAGATGTCACTGAGAGGAGTGAGAGTCAGCAAAATGGGAGTGGATGAATATTCCAGTACT

GAGAGAGTGGTCGTGAGCATTGATCGCTTCTTGAGAGTCCGAGACCAGAGGGGAAATGTG

CTTCTGTCTCCTGAGGAAGTTAGTGAAACACATGGAACAGAGAAACTGACGATAACGTAT

TCATCGTCTATGATGTGGGAAATCAATGGTCCGGAATCCGTGCTAGTCAACACATATCAA

TGGATCATTAGAAGTTGGGAAACTGTGAAGATTCAATGGTCCCAGGACCCTACGATGTTG

TACAATAAGATGGAATTTGAGCCCTTCCAATCCTTGGTGCCCAAGGCTGCTAGAGGCCAG

TATAGTGGGTTTGTGAGGACATTATTCCAACAGATGCGTGATGTGTTGGGGACATTTGAC

ACTGTCCAAATAATAAAGCTCCTACCATTTGCAGCAGCCCCACCGGAACAGAGTAGGATG

CAGTTTTCCTCTCTGACTGTGAACGTAAGAGGTTCAGGAATGAGAATACTTGTGAGGGGC

AACTCCCCTGTGTTCAACTATAATAAGGCCACCAAGAGACTCACAGTTCTTGGAAAGGAT

GCAGGCGCCTTGACAGAATATCCAGATGAGGGAACAGCAGGAGTGGAGTCTGCAGTATTA

AGAGGATTTCTAATTCTGGGCAAAGAGGACAAAAGATATGGACCAGCATTGAGCATCAAC

GAATTGAGCAATCTTGCGAAAGGGGAAAAGGCTAATGTGTTGATAGGACAAGGAGACGTG

GTGTTGGTAATGAAACGGAAACGGGACTCTAGCATACTTACTGACAGCCAGACAGCGACC

AAAAGAATTCGGATGGCCATCAATTAGTGTCGAATTGTTTAAAAACGACCTTGTTTCTAC

T---------------------

>A_pigeon_Kazakhstan_15-20-B-Talg-5_2020_EPI1927718

AGCRAAAGCAGGTCAAATATATTCAATATGGAGAGAATAAAAGAACTAAGAGATTTGATG

TCGCAGTCTCGCACTCGCGAGATACTAACAAAAACCACTGTGGACCATATGGCCATAATA

AAGAAATACACATCAGGGAGACAGGAGAAGAACCCTGCCCTCAGGATGAAATGGATGATG

GCAATGAAATATCCTATTACAGCTGACAAAAGAATAATGGAGATGATCCCTGAAAGGAAT

GAGCAAGGTCAGACTCTCTGGAGCAAAACAAATGATGCTGGATCAGACAGAGTGATGGTC

TCACCTCTGGCTGTGACATGGTGGAATAGAAATGGGCCAACAACAAGTACAGTACACTAC

CCAAAAGTCTACAAAACCTACTTTGAAAAGGTAGAAAGGTTGAAACATGGAACCTTTGGT

CCTGTTCACTTTCGAAATCAGGTTAAGATACGTCGCAGGGTTGACATAAACCCGGGCCAT

GCAGATCTCAGTGCCAAAGAAGCACAGGATGTCATCATGGAGGTTGTTTTCCCAAATGAA

GTTGGAGCCAGGATCTTGACATCAGAATCACAATTAACAATAACAAGGGAAAAGAAGGAG

GAACTTCAGGATTGCAAGATTGCTCCTTTGATGGTGGCATACATGTTGGAAAGAGAACTG

GTTCGCAAGACCAGATTTTTACCAGTAGCTGGCGGAACAAGCAGCGTATACATCGAGGTA

TTGCATTTGACTCAAGGGACCTGCTGGGAACAAATGTACACACCAGGAGGGGAGGTGAGA

AATGATGATGTTGATCAGAGTTTGATCATTGCTGCTAGAAATATAGTTAGGAGGGCAACA

GTATCAGCAGACCCATTGGCTTCGCTCTTGGAAATGTGCCACAGTACACAAATTGGTGGA

GTGAGGATGGTGGACATTCTTAGGCAGAACCCAACAGAGGAGCAAGCTGTGGATATATGC

AAAGCAGCAATGGGTTTAAGAATCAGTTCATCCTTCAGCTTTGGAGGTTTCACTTTCAAA

AGGACAAGTGGGTCGTCTGTCAAAAGAGAAGAAGAAATACTCACTGGCAACCTCCAAACA

CTGAAAGTAAGAATACATGAAGGATATGAGGAATTCACAATGGTTGGGCGAAGAGCTACA

GCCATTTTGAGGAAAGCAACCAGGAGACTGATCCAATTAATAGTGAGTGGACGAGACGAG

CAGTCAATCGCTGAAGCAATCATAGTGGCGATGGTTTTCTCACAGGAGGATTGCATGATA

AAAGCAGTACGAGGTGATTTGAATTTTGTCAACAGAGCGAATCAGCGGCTAAATCCTATG

CATCAACTTCTGAGGCATTTCCAAAAGGATGCAAAAGTGCTGTTTCAAAACTGGGGGATT

GAACCAATTGACAATGTAATGGGGATGATCGGGATACTGCCTGACATGACCCCCAGCACA

GAGATGTCACTGAGAGGAGTGAGAGTCAGCAAAATGGGAGTGGATGAATATTCCAGTACT

GAGAGAGTGGTCGTGAGCATTGATCGCTTCTTGAGAGTCCGAGATCAGAGGGGAAATGTG

CTTCTGTCTCCTGAGGAAGTTAGTGAAACACATGGAACAGAGAAACTGACGATAACGTAT

TCATCGTCTATGATGTGGGAAATCAATGGTCCGGAATCCGTGCTAGTCAACACATATCAA

TGGATCATTAGAAATTGGGAAACTGTGAAGATTCAGTGGTCCCAGGACCCTACGATGTTG

TACAATAAGATGGAATTTGAGCCCTTCCAATCCTTGGTGCCCAAGGCTGCTAGAGGCCAG

TATAGTGGGTTTGTGAGGACATTATTCCAACAGATGCGTGATGTGTTGGGGACATTTGAC

ACTGTCCAAATAATAAAGCTCCTACCATTTGCAGCAGCCCCACCGGAACAGAGTAGGATG

CAATTTTCCTCTCTGACTGTGAACGTAAGAGGTTCAGGAATGAGAATACTTGTGAGGGGC

AACTCCCCTGTGTTCAACTATAATAAGGCCACCAAAAGACTCACAATTCTTGGAAAGGAT

GCAGGCGCCTTGACAGAATATCCAGATGAGGGAACAGCAGGAGTGGAGTCTGCAGTATTA

AGAGGATTTCTAATTCTGGGCAAAGAGGACAAAAGATATGGACCAGCATTGAGCATCAAC

GAATTGAGCAATCTTGCGAAAGGGGAAAAGGCTAATGTGTTGATAGGACAAGGAGACGTG

GTGTTGGTAATGAAACGGAAACGGGACTCTAGCATACTTACTGACAGCCAGACAGCGACC

AAAAGAATTCGGATGGCCATCAATTAGTGTCGAATTGTTTAAAAACGACCTTGTTTCTAC

T---------------------

>A_chicken_Kazakhstan_1-20-B-Talg-67_2020_EPI1927724

AGCRAAAGCAGGTCAAATATATTCAATATGGAGAGAATAAAAGAACTAAGAGATTTGATG

TCGCAGTCTCGCACTCGCGAGATACTAACAAAAACCACTGTGGACCATATGGCCATAATA

AAGAAATACACATCAGGGAGACAGGAGAAGAACCCTGCCCTCAGGATGAAATGGATGATG

GCAATGAAATATCCTATTACAGCTGACAAAAGAATAATGGAGATGATCCCTGAAAGGAAT

GAGCAAGGTCAGACTCTCTGGAGCAAAACAAATGATGCTGGATCAGACAGAGTGATGGTC

TCACCTCTGGCTGTGACATGGTGGAATAGAAATGGGCCAACAACAAGTACAGTACACTAC

CCAAAAGTCTACAAAACCTACTTTGAAAAGGTAGAAAGGTTGAAACATGGAACCTTTGGT

CCTGTTCACTTTCGAAATCAGGTTAAGATACGTCGCAGGGTTGACATAAACCCGGGCCAT

GCAGATCTCAGTGCCAAAGAAGCACAGGATGTCATCATGGAGGTTGTTTTCCCAAATGAA

GTTGGAGCCAGRATCTTGACATCAGAATCACAATTAACAATAACAAGGGAAAAGAAGGAG

GAACTTCAGGATTGCAAGATTGCTCCTTTGATGGTGGCATACATGTTGGAAAGAGAACTG

GTTCGCAAGACCAGATTTTTACCAGTAGCTGGCGGAACAAGCAGCGTATACATCGAGGTA

TTGCATTTGACTCAAGGGACCTGCTGGGAACAAATGTACACACCAGGAGGGGAGGTGAGA

AATGATGATGTTGATCAGAGTTTGATCATTGCTGCTAGAAATATAGTTAGGAGGGCAACA

GTATCAGCAGACCCATTGGCTTCGCTCTTGGAAATGTGCCACAGTACACAAATTGGTGGA

GTGAGGATGGTGGACATTCTTAGGCAGAACCCAACAGAGGAGCAAGCTGTGGATATATGC

AAAGCAGCAATGGGTTTAAGAATCAGTTCATCCTTCAGCTTTGGAGGTTTCACTTTCAAA

AGGACAAGTGGGTCGTCTGTCAAAAGAGAAGAAGAAATACTCACTGGCAACCTCCAAACA

CTGAAAGTAAGAATACATGAAGGATATGAGGAATTCACAATGGTTGGGCGAAGAGCTACA

GCCATTTTGAGGAAAGCAACCAGGAGACTGATCCAATTAATAGTGAGTGGACGAGACGAG

CAGTCAATCGCTGAAGCAATCATAGTGGCGATGGTTTTCTCACAGGAGGATTGCATGATA

AAAGCAGTACGAGGTGATTTGAATTTTGTCAACAGAGCGAATCAGCGGCTAAATCCTATG

CATCAACTTCTGAGGCATTTCCAAAAGGATGCAAAAGTGCTGTTTCAAAACTGGGGGATT

GAACCAATTGACAATGTAATGGGGATGATCGGGATACTGCCTGACATGACCCCCAGCACA

GAGATGTCACTGAGAGGAGTGAGAGTCAGCAAAATGGGAGTGGATGAATATTCCAGTACT

GAGAGAGTGGTCGTGAGCATTGATCGCTTCTTGAGAGTCCGAGATCAGAGGGGAAATGTG

CTTCTGTCTCCTGAGGAAGTTAGTGAAACACATGGAACAGAGAAACTGACGATAACGTAT

TCATCGTCTATGATGTGGGAAATCAATGGTCCGGAATCCGTGCTAGTCAACACATATCAA

TGGATCATTAGAAATTGGGAAACTGTGAAGATTCAGTGGTCCCAGGACCCTACGATGTTG

TACAATAAGATGGAATTTGAGCCCTTCCAATCCTTGGTGCCCAAGGCTGCTAGAGGCCAG

TATAGTGGGTTTGTGAGGACATTATTCCAACAGATGCGTGATGTGTTGGGGACATTTGAC

ACTGTCCAAATAATAAAGCTCCTACCATTTGCAGCAGCCCCACCGGAACAGAGTAGGATG

CAATTTTCCTCTCTGACTGTGAACGTAAGAGGTTCAGGAATGAGAATACTTGTGAGGGGC

AACTCCCCTGTGTTCAACTATAATAAGGCCACCAAGAGACTCACAATTCTTGGAAAGGAT

GCAGGCGCCTTGACAGAATATCCAGATGAGGGAACAGCAGGAGTGGAGTCTGCAGTATTA

AGAGGATTTCTAATTCTGGGCAAAGAGGACAAAAGATATGGACCAGCATTGAGCATCAAC

GAATTGAGCAATCTTGCGAAAGGGGAAAAGGCTAATGTGTTGATAGGACAAGGAGACGTG

GTGTTGGTAATGAAACGGAAACGGGACTCTAGCATACTTACTGACAGCCAGACAGCGACC

AAAAGAATTCGGATGGCCATCAATTAGTGTCGAATTGTTTAAAAACGACCTTGTTTCTAC

T---------------------

>A_duck_Lao_961_2010_EPI335158

AGCAAAAGCAGGTCAAATATATTCAATATGGAGAGAATAAAAGAACTAAGAGATCTAATG

TCACAGTCTCGCACTCGCGAGATACTCACCAAAACCACTGTGGACCACATGGCCATAATC

AAGAAATACACATCAGGAAGACAAGAGAAGAATCCTGCTCTCAGAATGAAATGGATGATG

GCAATGAGGTATCCGATTACAGCGGACAAGAGAATAATAGATATGATTCCTGAAAGGAAT

GAACAAGGGCAGACGCTCTGGAGCAAGACAAATGATGCTGGGTCGGACAGGGTGATGGTG

TCCCCTCTAGCTGTAACTTGGTGGAATAGGAATGGGCCGACGACAAGTACAGTTCATTAT

CCAAAAGTTTACAAAACATACTTCGAGAAGGTTGAAAGGTTAAAACATGGAACCTTCGGT

CCCGTTCATTTCCGAAACCAAGTTAAAATACGCCGCCGAGTTGATACAAACCCTGGCCAT

GCAGATCTTAGTGCTAAAGAAGCACAAGATGTCATCATGGAAGTTGTTTTCCCAAATGAG

GTGGGAGCTAGAATATTGACTTCAGAGTCACAATTGACAATAACAAAAGAGAAGAAAGAA

GAGCTCCAAGATTGTAAGATTGCTCCCTTAATGGTTGCATACATGTTGGAGAGGGAACTG

GTCCGAAAAACCAGATTCCTACCGGTAGCAGGCGGGACAAGCAGTGTGTACATTGAGGTA

CTGCATTTGACTCAAGGAACCTGCTGGGAACAGATGTACACTCCAGGCGGAGAAGTAAGA

AATGATGATGTTGACCAGAGTTTGATCATTGCTGCCAGAAACATTGTTAGGAGAGCAACG

GTATCAGCGGATCCATTGGCATCACTGCTGGAGATGTGTCACAGCACACAAATTGGTGGG

ACAAGGATGGTGGATATCCTTAGGCAAAATCCAACTGAGGAACAAGCTGTGGATATATGC

AAAGCAGCAATGGGTCTGAGGATCAGCTCATCTTTTAGTTTTGGAGGTTTCACTTTCAAA

AGAACAAGTGGGTCATCCGTCACGAAGGAAGAGGAAGTGCTTACGGGCAACCTCCAAACA

TTGAAAATAAGAGTACATGAGGGGTATGAAGAATTCACAATGGTTGGGCAGAGGGCAACA

GCTATCCTGAGGAAAGCAACTAGGAGGCTGATTCAGTTGATAGTAAGCGGAAGAAACGAA

CAATCAATCGCTGAGGCAATCATTGTAGCGATGGTGTTCTCACAGGAGGATTGCATGATA

AAGGCAGTCAGAGGCGATCTAAATTTCGTAAACAGAGCAAACCAAAGATTAAATCCCATG

CATCAACTCCTGAGACATTTTCAAAAGGACGCAAAGGTGCTATTTCAGAATTGGGGAATT

GAACCCATTGATAATGTCATGGGGATGATCGGGATATTACCTGACATGACTCCCAGCACA

GAACTGTCACTGAGAGGAGTGAGAGTTAGTAAAATGGGAGTGGATGAATATTCCAGCACT

GAAAGAGTAGTTGTAAGCATTGACCGCTTTTTAAGGGTTCGAGATCAGCGGGGAAATGTA

CTCTTATCTCCCGAAGAGGTCAGNNNNNNNNNNNNNNNNGAAAAGTTGACAATAACATAT

TCGTCATCAATGATGTGGGAAATCAACGGTCCTGAATCCGTGCTTGTCAACACCTACCAA

TGGATCATCAGAAATTGGGAAACCGTGAAGATTCAATGGTCTCAGGATCCCACGATGCTG

TACAATAAGATGGAGTTCGAACCGTTCCAATCCTTGGTACCTAAAGCTGCCAGAGGTCAA

TACAGTGGATTTGTGAGAACATTATTTCAACAAATGCGTGACGTACTAGGGACATTTGAT

ACTGTCCAGATAATAAAGCTGCTACCATTTGCAGCAGCACCACCTGAGCAGAGCAGAATG

CAGTTTTCTTCTCTAACTGTGAATGTGAGAGGCTCAGGAATGAGAATACTCGTAAGGGGC

AATTCCCCTGTGTTCAACTACAATAAGACAACCAAAAGGCTTACCGTNNNNNNNNNNNNN

GCAGGTGCATTAGCAGAGGATCCAGACGAGGGGACAGCCGGAGTGGAATCTGCAGTACTG

AGGGGATTCCTAATTCTAGGCAAGGAGGACAAAAGATATGGACCAGCATTGAGCATCAAT

GAACTGAGCAATCTTGCAAAAGGGGAGAAAGCTAATGTGCTGATAGGACAAGGAGACGTG

GTGTTGGTAATGAAACGGAAACGGGACTCGAGCATACTTACTGACAGCCAGACAGCGACC

AAAAGAATTCGGATGGCCATCAATTAGTGTCGAATTATTTAAAAACGACCTTGTTTCTAC

T---------------------

>A_duck_Lao_567_2010_EPI335180

AGCAAAAGCAGGTCAAATATATTCAATATGGAGAGAATAAAAGAATTAAGAGATCTAATG

TCACAGTCCCGCACTCGCGAGATACTAACAAAAACCACTGTGGACCATATGGCCATAATC

AAGAAATACACATCAGGAAGACAAGAGAAGAATCCTGCTCTCAGAATGAAATGGATGATG

GCAATGAAATATCCGATCACAGCGGACAAGAGAATAATAGAGATGATTCCTGAGAGGAAT

GAACAGGGGCAGACACTCTGGAGCAAGACAAATGATGCTGGATCGGACAGGGTGATGGTG

TCTCCCCTAGCTGTAACTTGGTGGAATAGGAATGGGCCTACGACAAGTACAGTTCATTAT

CCAAAAGTTTACAAAACATACTTCGAGAAGGTTGAAAGATTAAGACATGGAACCTTCGGC

CCCGTTCATTTCAGAAACCAAGTTAAAATACGCCGCCGAGTTGATACAAACCCTGGCCAT

TCAGATCTCAGTGCTAAAGAAGCACAAGATGTCATCATGGAGGTCGTTTTCCCAAATGAA

GTGGGAGCTAGAATATTGACTTCAGAATCGCAATTGACAATAACAAAAGAGAAGAAAGAA

GAGCTCCAAGATTGTAAGATTGCTCCCTTAATGGTTGCATACATGTTGGAAAGGGAGCTG

GTCCGCAAAACCAGATTCTTACCGGTAGCAGGCGGAACAAGCAGTGTGTACATTGAGGTA

CTGCATTTGACTCAAGGAACCTGCTGGGAACAGATGTACACTCCAGGCGGAGAAGTAAGA

AATGACGATGTTGACCAGAGTTTGATCATTGCTGCCAGAAACATTGTTAGGAGAGCTACA

GTATCAGCAGATCCACTGGCATCACTGCTGGAGATGTGCCACAGCACACAAATTGGTGGG

ATAAGGATGGTGGACATACTTAGGCAAAATCCAACTGAGGAACAAGCTGTGGATATATGC

AAAGCAGCAATGGGTCTGAGGATCAGTTCATCCTTTAGCTTTGGAGGTTTCACTTTCAAA

AGAACAAGTGGATCATCCGTCATGAAGGAAGAGGAAGTGCTTACGGGCAATCTCCAAACA

TTGAAAATAAGAGTACATGAGGGGTATGAGGAATTCACAATGGTTGGGCAGAGGGCAACA

GCTATCCTGAGGAAAGCAACTAGAAGGCTGATTCAGCTGATAGTAAGTGGAAGAAACGAA

CAATCAATCGCTGAGGCAATCATTGTAGCAATGGTATTCTCACAGGAGGATTGCATGATA

AAGGCAGTCCGAGGCGATCTAAATTTCGTAAACAGAGCCAACCAAAGATTAAACCCCATG

CATCAACTCCTGAGGCATTTTCAAAAGGACGCAAAAGTGTTATTTCAGAATTGGGGAATT

GAACCCATTGATAATGTCATGGGGATGGTTGGAATATTACCTGACATGACTCCCAGCACA

GAAATGTCACTGAGAGGAATGAGAGTTAGTAAAATGGGAGTGGATGAATATTCCAGCACT

GAAAGAGTAGTTGTAAGCATTGACCGCTTCTTAAGGGTTCGAGATCAGCGGGGGAATGTA

CTCTTATCTCCCGAAGAGGTCAGCGAAACCCAGGGAACAGAGAAGTTGACAATAACATAT

TCGTCATCAATGATGTGGGAAATCAACGGCCCTGAGTCAGTGCTTGTCAACACTTACCAA

TGGATCATCAGAAACTGGGAGACCTTGAAGATTCAATGGTCTCAGGACCCCACTATGCTG

TACAATAAGATGGAGTTTGAACNGTTCCAATCCTTGGTACCTAAAGCTGCCAGAGGTCAA

TACAGTGGATTTGTGAGAACATTATTTCAACAAATGCGTGACGTACTGGGGACATTTGAT

ACTGTCCAGATAATAAAGCTGCTACCATTTGCAGCAGCACCACCGGAGCAGAGCAGAATG

CAGTTTTCTTCTCTAACTGTGAATGTGAGGGGCTCAGGAATGAGAATACTTGTAAGGGGC

AATTCCCCTGTGTTCAACCATAATAAGGCAACCAAAAGGCTTACCGTTCTTGGAAAGGAC

GCAGGTGCATTAACAGAGGATCCAGACGAGGGAACAGCAGGAGTGGAATCTGCAGTACTG

AGAGGATTCCTAATTCTAGGCAAGGAGGACAAACGGTATGGACCAGCATTGAGCATCAAT

GAACTGAGCAATCTTGCAAAAGGGGAGAAAGCTAATGTGCTGATAGGACAAGGAGACGTG

GTGTTGGTAATGAAACGGAAACGGGACTCTAGCATACTTACTGACAGCCAGACAGCGACC

AAAAGAATTCGGATGGCCATCAATTAGTGTCAAACTGTTTAAAAACGACCTTGTTTCTAC

T---------------------

>A_breeder_duck_Korea_Gochang1_2014_EPI509696

--------------AAATATATTCAATATGGAGAGAATAAAAGAACTAAGAGATTTGATG

TCGCAGTCTCGCACTCGCGAGATACTGACAAAAACCACTGTGGACCATATGGCCATAATC

AAGAAGTATACGTCAGGAAGACAGGAGAAGAATCCTGCTCTTAGGATGAAATGGATGATG

GCAATGAAATACCCGATTACAGCAGACAAGAGGATAATGGAGATGATCCCTGAGAGAAAT

GAGCAAGGTCAGACTCTTTGGAGCAAAACGAATGATGCTGGATCAGACAGAGTGATGGTA

TCACCTCTTGCTGTGACGTGGTGGAACAGAAATGGACCAATGACAAGTACAGTCCATTAT

CCAAAGGTTTACAAAACCTACTTTGAGAAGGTCGAAAGATTAAAGCATGGAACCTTCGGT

CCCGTTCACTTTCGAAATCAGGTCAAAATACGTCGCAGGGTTGACATAAACCCGGGTCAC

GCAGATCTTAGTGCTAAAGAAGCACAAGACGTCATAATGGAGGTCGTTTTCCCAAACGAA

GTAGGAGCCAGGATATTGACATCAGAGTCACAGTTAACAATAACAAAAGAAAAGAAGGAA

GAGCTCCAGGACTGTAAGATCGCTCCTTTGATGGTGGCATACATGTTGGAAAGGGAACTA

GTTCGCAAAACCAGATTCCTACCAGTAGCTGGCGGGACAAGCAGCGTGTATATCGAGGTG

TTGCACTTGACCCAAGGGACCTGCTGGGAACAAATGTACACACCGGGAGGGGAGGTGAGA

AATGATGATGTTGATCAGAGTTTAATTATTGCTGCTAGAAATATTGTCAGGAGAGCAACA

GTATCAGCAGACCCGTTGGCTTCGCTCTTGGAAATGTGCCATAGTACAAAAATTGGCGGA

ATAAGGATGGTAGACATCCTTAGACAAAATCCAACAGAAGAGCAAGCTGTGGATATATGC

AAAGCAGCAATGGGTCTAAGGATCAGTTCATCCTTCAGCTTTGGAGGTTTCACTTTCAAA

AGGACAAGTGGGTCATCTGTCAAAAGAGAAGAAGAAGTGCTCACAGGCAATCTCCAGACA

TTGAAAATAAGAGTGCACGAAGGATATGAGGAATTCACAATGGTCGGGCGAAGAGCAACA

GCCATTCTAAGGAAAGCAACCAGAAGGCTGATCCAATTGATAGTGAGTGGAAGAGACGAG

CAGTCAATTGCCGAAGCAATCATAGTGGCAATGGTGTTCTCACAAGAGGATTGCATGATA

AAAGCAGTGCGAGGTGATTTGAATTTTGTCAACAGAGCGAACCAGCGGCTAAATCCCATG

CATCAACTTCTGAGGCATTTCCAGAAGGATGCAAAGGTGCTGTTTCAAAACTGGGGAGTT

GAACCCATTGACAATGTCATGGGAATGATCGGGATATTACCTGACATGACCCCCAACACA

GAGATGTCACTAAGGGGAGTGAGAGTCAGTAAAATGGGAGTGGATGAATATTCCAGTACT

GAGAGAGTGGTCGTGAGCATTGATCGTTTCTTGAGGGTCCGAGACCAGAGGGGAAATGTG

CTCTTGTCTCCTGAAGAGGTTAGTGAAACACAGGGAACAGAAAAGCTGACGATAACATAT

TCATCGTCCATGATGTGGGAAATCAATGGCCCGGAATCAGTGTTAGTCAACACATATCAA

TGGATCATTAGAAACTGGGAAACTGTGAAGATCCAGTGGTCTCAAGACCCTACAATGCTA

TACAATAAGATGGAATTTGAGCCCTTCCAATCCTTGGTGCCTAAGGCTGCCAGAGGCCAG

TACAGCGGCTTTGTGAGGACGCTATTCCAGCAGATGCGTGATGTGCTGGGGACATTTGAC

ACTGTCCAGATAATAAAGCTGCTACCATTTGCAGCAGCCCCACCAGAACAGAGTAGGATG

CAGTTCTCTTCTCTAACTGTGAACGTAAGGGGTTCAGGAATGAGAATACTTGTGAGAGGC

AATTCCCCTGTGTTCAATTATAACAAGGCAACCAAGAGGCTCACAGTCCTTGGAAAGGAT

GCAGGTGCATTGACAGAAGATCCAGATGAGGGAACAGCAGGAGTGGAATCTGCGGTATTA

AGAGGGTTTCTAATTCTGGGCAAAGAAGACAAAAGATATGGACCAGCATTGAGCATCAAC

GAATTGAGCAATCTTGCGAAAGGGGAGAAGGCTAATGTGTTGATAGGGCAAGGAGACGTG

GTGTTGGTAATGAAACGGAAACGGGACTCTAGCATACTTACTGACAGCCAGACAGCGACC

AAAAGAATTCGGATGGCCATCAATTAGTGTCGCATTGTTTAA------------------

----------------------

>A_broiler_duck_Korea_Buan2_2014_EPI509702

--------------AAATATATTCAGTATGGAGAGAATAAAAGAACTAAGAGATCTAATG

TCTCAATCCCGCACTCGCGAGATACTAACAAAAACCACTGTGGACCATATGGCCATAATC

AAGAAATACACATCAGGAAGACAAGAGAAGAACCCTGCTCTCAGAATGAAATGGATGATG

GCAATGAAATATCCAATCACAGCAGACAAGAGAATAATGGAAATGATTCCTGAAAGAAAT

GAACAAGGCCAGACGCTTTGGAGCAAGACAAATGATGCTGGATCAGACAGAGTGATGGTG

TCTCCCCTAGCTGTAACTTGGTGGAATAGAAATGGACCGACAGCAAGTACAGTCCATTAT

CCAAAGGTCTACAAAACATACTTTGAGAAGGTTGAAAGGTTAAAGCATGGAACCTTCGGT

CCCGTTCACTTCCGAAACCAAATTAAAATACGCCGCCGAGTTGACATAAACCCAGGCCAC

GCAGATCTCAGTGCCAAAGAAGCACAAGATGTCATCATGGAGGTCGTTTTCCCAAATGAA

GTGGGAGCTAGAATATTGACATCAGAGTCACAATTGACAATAACGAAAGAGAAAAAAGAA

GAACTCCAGGATTGCAAGATTGCTCCTTTAATGGTGGCATACATGTTGGAAAGAGAACTG

GTCCGCAAAACCAGATTCCTACCAGTAGCAGGTGGGACAAGCAGTGTGTACATTGAGGTA

CTGCACTTGACCCAAGGGACCTGCTGGGAACAGATGTACACTCCAGGCGGAGAAGTGAGA

AATGACGATGTTGACCAGAGTTTGATCATCGCGGCCAGAAACATTGTTAGGAGAGCAACG

GTATCAGCGGATCCACTGGCATCATTATTGGAGATGTGCCACAGCACACAAATTGGTGGG

ACAAGGATGGTGGATATCCTTAGGCAAAATCCAACTGAGGAACAAGCTGTGGATATATGC

AAAGCAGCAATGGGTTTGAGGATTAGTTCATCCTTTAGCTTTGGAGGATTCACCTTCAAA

AGAACAAGTGGTTCATCCGTTAGAAAGGAAGAGGAAGTGCTTACAGGCAACCTCCAAACA

TTGAAAATAAGAGTACATGAGGGGTATGAGGAGTTCACAATGGTTGGGCGAAGAGCAACA

GCCATTCTAAGGAAAGCAACTAGAAGGCTGATTCAGTTGATAGTAAGTGGAAGAGACGAA

CAATCAATCGCTGAAGCAATCATCGTAGCCATGGTGTTCTCACAGGAGGATTGCATGATA

AAGGCAGTCCGAGGCGATCTAAATTTTGTGAACAGAGCAAACCAAAGATTGAACCCCATG

CATCAACTCCTGAGACACTTCCAAAAAGATGCAAAAGTGCTGTTTCAAAATTGGGGGATT

GAACCTATTGATAATGTCATGGGGATGATTGGAATATTACCTGACATGACTCCAAGCACA

GAGATGTCACTAAGAGGAGTAAGAGTTAGTAAAATGGGAGTAGATGAATATTCCAGCACT

GAGAGAGTGGTTGTAAGCATTGACCGTTTCTTGCGGGTTCGAGATCAGCAGGGGAACGTA

CTCCTATCTCCCGAAGAGGTCAGCGAAACACTGGGAACAGAAAAATTAACAATAACATAT

TCATCATCAATGATGTGGGAAATCAATGGTCCTGAGTCAGTGCTGGTCAACACCTATCAA

TGGATCATCAGAAATTGGGAGATTGTGAAGATTCAATGGTCTCAAGACCCCACGATGCTG

TACAATAAGGTGGAGTTTGAACCGTTCCAATCCTTGGTACCTAAAGCTGCCAGAGGCCAA

TACAGTGGATTTGTGAGAACACTGTTCCAACAAATGCGTGACGTATTGGGGACCTTTGAT

ACTATTCAGATAATAAAGCTGTTACCGTTTGCAGCAGCCCCACCGGAGCATAGCAGAATG

CAATTTTCTTCCCTGACCGTGAATGTAAGAGGCTCGGGAATGAGAATACTCGTAAGGGGT

AACTCCCCTGTGTTCAACTACAATAAGGCAACCAAAAGGCTTGCCGTCCTTGGAAAGGAC

GCAGGTGCATTAACAGAGGATCCAGATGAGGGGACAACAGGAGTGGAATCTGCAGTGCTG

AGGGGGTTCCTAATTCTGGGCAAGGAGGACAGAAGATATGGACCAGCACTAAGCATCAAT

GAACTGAGCAATCTTGCGAAAGGGGAGAAAGCCAATGTGCTGATAGGGCAAGGAGACGTG

GTGCTGGTAATGAAACGGAAACGGGACTCTAGCATACTTACTGACAGCCAGACAGCGACC

AAAAGAATTCGGATGGTCATCAATTAGTATCGAGTTGTTTAA------------------

----------------------

>A_goose_Taiwan_TNO15_2015_EPI690741

---------------------------ATGGAGAGAATAAAAGAACTAAGAGATCTAATG

TCTCAATCCCGCACTCGCGAGATACTAACAAAAACCACTGTGGACCATATGGCCATAATC

AAGAAATACACATCAGGAAGACAAGAGAAGAATCCTGCTCTCAGAATGAAATGGATGATG

GCAATGAAATATCCAATCACAGCAGACAAGAGAATAATGGAAATGATTCCTGAAAGAAAT

GAACAAGGCCAGACGCTTTGGAGCAAGACAAATGATGCTGGATCAGACAGAGTGATGGTG

TCTCCCCTAGCTGTAACTTGGTGGAATAGAAATGGACCAACAGCAAGTACAGTCCATTAT

CCAAAGGTCTACAAAACATACTTTGAGAAGGTTGAAAGGTTAAAGCATGGAACCTTCGGT

CCCGTTCACTTCCGAAACCAAATTAAGATACGCCGCCGAGTTGACATAAACCCAGGCCAT

GCAGATCTCAGTGCCAAAGAAGCACAAGATGTCATCATGGAGGTCGTTTTCCCAAATGAA

GTGGGAGCTAGAATATTGACATCAGAGTCACAATTGACAATAACGAAAGAGAAAAAAGAA

GAACTCCAGGATTGCAAGATTGCTCCTTTAATGGTGGCATACATGTTGGAAAGAGAACTG

GTCCGCAAAACCAGATTCCTACCAGTAGCAGGTGGGACAAGCAGTGTGTACATTGAGGTA

CTGCACTTGACTCAAGGGACCTGCTGGGAACAGATGTACACTCCAGGCGGAAAAGTGAGG

AATGACGATGTTGACCAGAGTTTGATCATCGCGGCCAGAAACATTGTCAGGAGAGCAACG

GTATCAGCGGATCCACTGGCATCATTATTGGAGATGTGCCACAGCACACAAATTGGTGGG

ACAAGGATGGTGGATATTCTTAGGCAAAATCCAACTGAGGAACAAGCTGTGGATATATGC

AAAGCAGCAATGGGTTTGAGGATTAGTTCATCCTTTAGCTTTGGAGGATTCACCTTCAAA

AGAACAAGTGGTTCATCCGTTAGAAAGGAAGAGGAAGTGCTTACAGGCAACCTCCAAACA

TTGAAAATAAGAGTACATGAGGGGTATGAGGAGTTCACAATGGTTGGGCGAAGAGCAACA

GCCATTCTAAGGAAAGCAACTAGAAGGCTGATTCAGTTGATAGTAAGTGGAAGAGACGAA

CAATCAATCGCTGAAGCAATCATCGTAGCCATGGTGTTCTCACAGGAGGATTGCATGATA

AAGGCAGTCCGAGGCGATCTAAATTTTGTGAACAGAGCAAACCAAAGATTGAACCCCATG

CATCAACTCCTGAGACACTTCCAAAAAGATGCAAAAGTGCTGTTTCAAAATTGGGGGATT

GAACCTATTGATAATGTCATGGGGATGATTGGGATATTACCTGACATGACTCCAAGCACA

GAGATGTCACTAAGAGGAGTAAGAGTTAGTAAAATGGGAGTAGATGAATATTCCAGCACT

GAGAGAGTGGTTGTAAGCATTGACCGTTTCTTGCGGGTTCGAGATCAGCAGGGGAACGTA

CTCCTATCTCCCGAAGAGGTCAGCGAAACACTGGGAACAGAAAAATTAACAATAACATAT

TCATCATCAATGATGTGGGAAATCAATGGTCCTGAGTCAGTGCTGGTCAACACCTATCAA

TGGATCATCAGAAATTGGGAAATTGTGAAGATTCAATGGTCTCAAGACCCCACGATGCTG

TACAATAAGGTGGAGTTTGAACCGTTCCAATCCTTGGTACCTAAAGCTGCCAGAGGCCAA

TACAGTGGATTTGTGAGAACACTGTTCCAACAAATGCGTGACGTATTGGGGACATTTGAT

ACTATTCAGATAATAAAGCTGTTACCGTTTGCAGCAGCCCCACCGGAGCATAGCAGAATG

CAATTTTCTTCCCTGACCGTGAATGTAAGAGGCTCGGGAATGAGAATACTCGTAAGGGGT

AACTCCCCTGTGTTCAACTACAATAAGGCAACCAAAAGGCTTGCAGTCCTTGGAAAGGAC

GCAGGTGCATTAACAGAGGATCCAGATGAGGGGACAACAGGAGTGGAATCCGCAGTACTG

AGGGGGTTCCTAATTCTGGGCAAGGAGGACAGAAGATATGGACCAGCACTAAGCATCAAT

GAACTGAGCAATCTTGCGAAAGGGGAGAAAGCCAATGTGCTGATAGGGCAAGGAGACGTG

GTGCTGGTAATGAAACGGAAACGGGACTCTAGCATACTTACTGACAGCCAGACAGCGACC

AAAAGAATTCGGATGGTCATCAATTAG---------------------------------

----------------------

>A_wigeon_Sakha_1_2014_EPI1201974

---------------------------ATGGAGAGAATAAAAGAACTAAGAGATCTAATG

TCTCAATCCCGCACTCGCGAGATACTAACAAAAACCACTGTGGACCATATGGCCATAATC

AAGAAATACACATCAGGAAGACAAGAGAAGAACCCTGCTCTCAGAATGAAATGGATGATG

GCAATGAAATATCCAATCACAGCAGACAAGAGAATACTGGAAATGATTCCTGAAAGAAAT

GAACAAGGCCAGACGCTTTGGAGCAAGACAAATGATGCTGGATCAGACAGAGTGATGGTG

TCTCCCCTAGCTGTAACTTGGTGGAATAGAAATGGACCGACAGCAAGTACAGTCCATTAT

CCAAAGGTCTACAAAACATACTTTGAGAAGGTTGAAAGGTTAAAGCATGGAACCTTCGGT

CCCGTTCACTTCCGAAACCAAATTAAAATACGCCGCCGAGTTGACATAAACCCAGGCCAC

GCAGATCTCAGTGCCAAAGAAGCACAAGATGTCATCATGGAGGTCGTTTTCCCAAATGAA

GTGGGAGCTAGAATATTGACATCAGAGTCACAATTGACAATAACGAAAGAGAAAAAAGAA

GAACTCCAGGATTGCAAGATTGCTCCTTTAATGGTGGCATACATGTTGGAAAGAGAACTG

GTCCGCAAAACCAGATTCCTACCAGTAGCAGGTGGGACAAGCAGTGTGTACATTGAGGTA

CTGCACTTGACCCAAGGGACCTGCTGGGAACAGATGTACACTCCAGGCGGAGAAGTGAGA

AATGACGATGTTGACCAGAGTTTGATCATCGCGGCCAGAAACATTGTTAGGAGAGCAACG

GTATCAGCGGATCCACTGGCATCATTATTGGAGATGTGCCACAGCACACAAATTGGTGGG

ACAAGGATGGTGGATATCCTTAGGCAAAATCCAACTGAGGAACAAGCTGTGGATATATGC

AAAGCAGCAATGGGTTTAAGGATTAGTTCATCCTTTAGCTTTGGAGGATTCACCTTCAAA

AGAACAAGTGGTTCATCCATTAGAAAGGAAGAGGAAGTGCTTACAGGCAACCTCCAAACA

TTGAAAATAAGAGTACATGAGGGGTATGAGGAGTTCACAATGGTTGGGCGAAGAGCAACA

GCCATTCTAAGGAAAGCAACTAGAAGGCTGATTCAGTTGATAGTAAGTGGAAGAGACGAA

CAATCAATCGCTGAAGCAATCATCGTAGCCATGGTGTTCTCACAGGAGGATTGCATGATA

AAGGCAGTCCGAGGCGATCTAAATTTTGTGAACAGAGCAAACCAAAGATTGAACCCCATG

CATCAACTCCTGAGACACTTCCAAAAAGATGCAAAAGTGCTGTTTCAAAATTGGGGGATT

GAACCCATTGATAATGTCATGGGGATGATTGGAATATTGCCTGACATGACTCCAAGCACA

GAGATGTCACTAAGAGGAGTAAGAGTTAGTAAAATGGGAGTAGATGAATATTCCAGCACT

GAGAGAGTGGTTGTAAGCATTGACCGTTTCTTGCGGGTTCGAGATCAGCAGGGGAACGTA

CTCCTATCTCCCGAAGAAGTCAGCGAAACACTGGGAACAGAAAAATTAACAATAACATAT

TCATCATCAATGATGTGGGAAATCAATGGTCCTGAGTCAGTGCTGGTCAACACCTATCAA

TGGATCATCAGAAATTGGGAGATTGTGAAGATTCAATGGTCTCAAGACCCCACGATGCTG

TACAATAAGGTGGAGTTTGAACCGTTCCAATCCTTGGTACCTAAAGCTGCCAGAGGCCAA

TACAGTGGATTTGTGAGAACACTGTTCCAACAAATGCGTGACGTATTGGGGACATTTGAT

ACTATTCAGATAATAAAGCTGTTACCGTTTGCAGCAGCCCCACCGGAGCATAGCAGAATG

CAATTTTCTTCCCTGACCGTGAATGTAAGAGGCTCGGGAATGAGAATACTCGTAAGGGGT

AACTCCCCTGTGTTCAACTACAATAAGGCAACCAAAAGGCTTGCCGTCCTTGGAAAGGAC

GCAGGTGCATTAACAGAGGATCCAGATGAGGGGACAACAGGAGTGGAATCTGCAGTGCTG

AGGGGGTTCCTAATTCTGGGCAGGGAGGACAGAAGATATGGACCAGCACTAAGCATCAAT

GAACTGAGCAATCTTGCGAAAGGGGAGAAAGCCAATGTGCTGATAGGGCAAGGAGACGTG

GTGCTGGTAATGAAACGGAAACGGGACTCTAGCATACTTACTGACAGCCAGACAGCGACC

AAAAGAATTCGGATGGTCATCAATTAGTATCGAGTTGTTTAAAAACGACCTTGTTTCTAC

TGGTCATTAGCCTGTTTTCCTG

>A_duck_Nigeria_SK28T_19VIR8424-2_2019_EPI1777106

AGCAAAAGCAGGTCAAATATATTCAATATGGAGAGAATAAAAGAATTAAGAGATTTGATG

TCGCAGTCTCGCACTCGCGAGATACTAACAAAANCCACTGTGGACCATATGGCCATAATC

AAGAAATATACGTCAGGAAGACAGGAGAAGAATCCTGCTCTTAGGATGAAATGGATGATG

GCAATGAAATATCCGATCACAGCAGACAAAAGGATAATGGAGATGATCCCTGAAAGAAAT

GAGCAAGGTCAGACTCTCTGGAGCAAGACGAATGATGCTGGATCGGATAGAGTTATGGTG

TCACCTCTGGCTGTGACGTGGTGGAATAGAAATGGGCCAACGACGAGTACAATCCATTAT

CCAAAGGTCTATAAAACCTATTTTGAAAAGGTCGAAAGGTTAAAACATGGAACCTTCGGT

CCCGTCCACTTTCGAAATCAGGTTAAAATACGCCGCAGGGTTGACATAAACCCAGGCCAT

GCAGATCTCAGTGCCAAAGAAGCACAGGATGTCATCATGGAGGTCGTTTTCCCAAATGAA

GTAGGAGCTAGGATATTGACATCAGAGTCACAGTTAACAATAACAAAGGAAAAGAAGGAG

GAGCTTCAGGACTGTAAGATTGCTCCTTTGATGGTGGCATACATGTTGGAAAGAGAATTG

GTTCGAAAAACCAGATTTCTACCAGTAGCTGGTGGGACAAGCAGTGTGTACATTGAAGTG

TTACACTTGACTCAGGGGACCTGCTGGGAACAAATGTATACGCCGGGAGGGGATGTGATA

AATGATGATGTTGATCAGAGTTTAATTATTGCTGCTCGAAATATTGTTAGAAGGGCAGTA

GTATCAGCAGACCCATTGGCTTCGCTCTTGGAGATGTGCCATAGCACGCAGATTGGCGGG

ATAAGGATGGTAGACATCCTTAGACAAAACCCAACAGAAGAGCAAGCTGTGGACATATGC

AAAGCTGCAATGGGTCTAAGGATCAGTTCATCCTTCAGCTTTGGAGGTTTCACTTTCAAG

AGGACGAGTGGATCATCTATCAAAANAGAAGAAGAAGTGCTCACAGGTAACCTCCAAACA

TTGAAAATAAGAGTGCATGAAGGATATGAAGAATTCACAATGGTTGGGCGAAGAGCAACA

GCTATTCTAAGGAAAGCAACCAGAAGGCTGATCCAACTGATAGTGAGTGGGAAAGACGAT

CAGTCAATTGCCGAGGCGATCATAGTGGCAATGGTGTTCTCACAAGAAGACTGTGTGATA

AAAGCAGTACGAGGTGATCTGAATTTTGTCAACAGAGCAAACCAGCGGTTAAATCCTATG

CATCAGCTATTGAGGCATTTCCAGAAGGATGCAAAGGTGTTGTTTCAAAACTGGGGAATT

GAGCCCATCGACAATGTCATGGGGATGATCGGAATATTACCTGACATGACCCCTAGCACC

AAGATGTCACTAAGAGGAGTGAGAGTCAGTAAAATGGGAGTGGATGAATACTCCAGTACT

GAGCGGGTAGTCGTAAGCATAGATCGTTTCTTGAGGGTCCGAGACCAGAGGGGAAACGTG

CTCTTATCTCCAGAAGAAGTTAGTGAAACACAGGGAACAGAAAAACTGACAATAACATAT

TCGTCATCCATGATGTGGGAAATCAACGGCCCGGAATCAGTGTTAGTGAACACATATCAA

TGGATCATTAGAAATTGGGAAACTGTGAAAATTCAGTGGTCCCAAGACCCCACAATGCTA

TACAATAAGATGGAGTTTGAGCCCTTTCAATCCTTGGTGCCTAAGGCTGCCAGAGGTCAG

TACAGTGGATTTGTGAGAACGCTATTCCAACAGATGCGTGATGTACTGGGGACATTTGAC

ACCGTCCAAATAATAAAGCTGCTGCCATTTGCAGCAGCCCCACCAGAACAGAGTAGAATG

CAGTTCTCTTCTCTAACCGTGAACGTGAGGGGTTCAGGAATGAGAATACTTGTGAGAGGC

AACTCCCCTGTGTTCAACTATAACAAGGCAACCAAAAGGCTTACAGTCCTAGGAAAGGAT

GCAGGTGCATTGACAGAAGATTCAGATGAGGGAACAGCAGGGGTGGAATCTGCGGTATTA

AGAGGATTTCTAATCCTAGGTAAAGAAGACAAAAGATATGGACCGGCATTGAGCATCAAC

GAGTTGAGCAATCTTGCGAAAGGGGAGAAGGCTAATGTGTTGATAGGGCAAGGAGACGTG

GTGTTGGTAATGAAACGGAAACGGGACTCTAGCATACTTACTGACAGCCAGACAGCGACC

AAAAGAATTCGGATGGCCATCAATTAGTGTCGAATTGTTTAAAAACGACCTTGTTTCTAC

T---------------------

>A_mute_swan_Kazakhstan_1-267-20-B_2020_EPI1811580

--------------AAATATATTCAATATGGAGAGAATAAAAGAACTAAGAGATTTGATG

TCGCAGTCTCGCACTCGCGAGATACTAACAAAAACCACTGTGGACCATATGGCCATAATA

AAGAAATACACATCAGGGAGACAGGAGAAGAACCCTGCCCTCAGGATGAAATGGATGATG

GCAATGAAATATCCTATTACAGCTGACAAAAGAATAATGGAGATGATCCCTGAAAGGAAT

GAGCAAGGTCAGACTCTCTGGAGCAAAACAAATGATGCTGGATCAGACAGAGTGATGGTC

TCACCTCTGGCTGTGACATGGTGGAATAGAAATGGGCCAACAACAAGTACAGTACACTAC

CCAAAAGTCTACAAAACCTACTTTGAAAAGGTAGAAAGGTTGAAACATGGAACCTTTGGT

CCTGTTCACTTTCGAAATCAGGTTAAGATACGCCGCAGGGTTGACATAAACCCGGGCCAT

GCAGATCTCAGTGCCAAAGAAGCACAGGATGTCATCATGGAGGTTGTTTTCCCAAATGAA

GTTGGAGCCAGGATCTTGACATCAGAGTCACAATTAACAATAACAAGGGAAAAGAAGGAG

GAACTTCAGGATTGCAAGATTGCTCCTTTGATGGTGGCATACATGTTGGAAAGAGAACTG

GTTCGCAAGACCAGATTTTTACCAGTAGCTGGCGGAACAAGCAGCGTATACATCGAGGTA

TTGCATTTGACTCAAGGGACCTGCTGGGAACAAATGTACACACCAGGAGGGGAGGTGAGA

AATGATGATGTTGATCAGAGTTTGATCATTGCTGCTAGAAATATAGTTAGGAGGGCAACA

GTATCAGCAGACCCATTGGCTTCGCTCTTGGAAATGTGCCACAGTACACAAATTGGTGGA

GTGAGGATGGTGGACATTCTTAGGCAGAACCCAACAGAGGAGCAAGCTGTGGATATATGC

AAAGCAGCAATGGGTTTAAGAATCAGTTCATCCTTCAGCTTTGGAGGTTTCACTTTCAAA

AGGACAAGTGGGTCGTCTGTCAAAAGAGAAGAAGAAATACTCACTGGCAACCTCCAAACA

CTGAAAGTAAGAATACATGAAGGATATGAGGAATTCACAATGGTTGGGCGAAGAGCTACA

GCCATTTTGAGGAAAGCAACCAGGAGACTGATCCAATTAATAGTGAGTGGACGAGACGAG

CAGTCAATCGCTGAAGCAATCATAGTGGCAATGGTTTTCTCACAGGAGGATTGCATGATA

AAAGCAGTACGAGGTGATTTGAATTTTGTCAACAGAGCGAATCAGCGGCTAAATCCTATG

CATCAACTTCTGAGGCATTTCCAAAAGGATGCAAAAGTGCTGTTTCAAAACTGGGGGATT

GAACCAATTGACAATGTAATGGGGATGATCGGGATACTGCCTGACATGACCCCCAGCACA

GAGATGTCACTGAGAGGAGTGAGAGTCAGCAAAATGGGAGTGGATGAATACTCCAGTACT

GAGAGAGTGGTCGTGAGCATTGATCGCTTCTTGAGAGTCCGAGATCAGAGGGGAAATGTG

CTTCTGTCTCCTGAGGAAGTTAATGAAACACATGGAACAGAGAAACTGACGATAACGTAT

TCATCGTCTATGATGTGGGAAATCAATGGTCCGGAATCCGTGCTAGTCAACACATATCAA

TGGATCATTAGAAATTGGGAAACTGTGAAGATTCAGTGGTCCCAGGACCCTACGATGTTG

TACAATAAGATGGAATTTGAGCCCTTCCAATCCTTGGTGCCCAAGGCTGCTAGAGGCCAG

TATAGTGGGTTTGTGAGGACATTATTCCAACAGATGCGTGATGTGTTGGGGACATTTGAC

ACTGTCCAAATAATAAAGCTCCTACCATTTGCAGCAGCCCCACCGGAACAGAGTAGGATG

CAATTTTCCTCTCTGACTGTGAACGTAAGAGGTTCAGGAATGAGAATACTTGTGAGGGGC

AACTCCCCTGTGTTCAACTATAATAAGGCCACCAAGAGACTCACAGTTCTTGGAAAGGAT

GCAGGCGCCTTGACAGAATATCCAGATGAGGGAACAGCAGGAGTGGAGTCTGCAGTATTA

AGAGGATTTCTAATTCTGGGCAAAGAGGACAAAAGATATGGACCAGCATTGAGCATCAAC

GAATTGAGCAATCTTGCGAAAGGGGAAAAGGCTAATGTGTTGATAGGACAAGGAGACGTG

GTGTTGGTAATGAAACGGAAACGGGACTCTAGCATACTTACTGACAGCCAGACAGCGACC

AAAAGAATTCGGATGGCCATCAATTAGTGTCGAATTGTTTAA------------------

----------------------

>A_domestic_goose_Kazakhstan_1-248_2-20-B_2020_EPI1811598

--------------AAATATATTCAATATGGAGAGAATAAAAGAACTAAGAGATTTG---

------------------------CTAACAAAAACCACTGTGGACCATATGGCCATAATA

AAGAAATACACATCAGGGAGACAGGAGAAGAACCCTGCCCTCAGGATGAAATGGATGATG

GCAATGAAATATCCTATTACAGCTGACAAAAGAATAATGGAGATGATCCCTGAAAGGAAT

GAGCAAGGTCAGACTCTCTGGAGCAAAACAAATGATGCTGGATCAGACAGAGTGATGGTC

TCACCTCTGGCTGTGACATGGTGGAATAGAAATGGGCCAACAACAAGTACAGTACACTAC

CCAAAAGTCTACAAAACCTACTTTGAAAAGGTAGAAAGGTTGAAACATGGAACCTTTGGT

CCTGTTCACTTTCGAAATCAGGTTAAGATACGCCGCAGGGTTGACATAAACCCGGGCCAT

GCAGATCTCAGTGCCAAAGAAGCACAGGATGTCATCATGGAGGTTGTTTTCCCAAATGAA

GTTGGAGCCAGGATCTTGACATCAGAATCACAATTAACAATAACAAGGGAAAAGAAGGAG

GAACTTCAGGATTGCAAGATTGCTCCTTTGATGGTGGCATACATGTTGGAAAGAGAACTG

GTTCGCAAGACCAGATTTTTACCAGTAGCTGGCGGAACAAGCAGCGTATACATCGAGGTA

TTGCATTTGACTCAAGGGACCTGCTGGGAACAAATGTACACACCAGGAGGGGAGGTGAGA

AATGATGATGTTGATCAGAGTTTGATCATTGCTGCTAGAAATATAGTTAGGAGGGCAACA

GTATCAGCAGACCCATTGGCTTCGCTCTTGGAAATGTGCCACAGTACACAAATTGGTGGA

GTGAGGATGGTGGACATTCTTARGCAGAACCCAACAGAGGAGCAAGCTGTGGATATATGC

AAAGCAGCAATGGGTTTAAGAATCAGTTCATCCTTCAGCTTTGGAGGTTTCACTTTCAAA

AGGACAAGTGGGTCGTCTGTCAAAAGAGAAGAAGAAATACTCACTGGCAACCTCCAAACA

CTGAAAGTAAGAATACATGAAGGATATGAGGAATTCACAATGGTTGGGCGAAGAGCTACA

GCCATTTTGAGGAAAGCAACCAGGAGACTGATCCAATTAATAGTGAGTGGACGAGACGAG

CAGTCAATCGCTGAAGCAATCATAGTGGCAATGGTTTTCTCACAGGAGGATTGCATGATA

AAAGCAGTACGAGGTGATTTGAATTTTGTCAACAGAGCGAATCAGCGGCTAAATCCTATG

CATCAACTTCTGAGGCATTTCCAAAAGGATGCAAAAGTGCTGTTTCAAAACTGGGGGATT

GAACCAATTGACAATGTAATGGGGATGATCGGGATACTGCCTGACATGACCCCCAGCACA

GAGATGTCACTGAGAGGAGTGAGAGTCAGCAAAATGGGAGTGGATGAATATTCCAGTACT

GAGAGAGTGGTCGTGAGCATTGATCGCTTCTTGAGAGTCCGAGATCAGAGGGGAAATGTG

CTTCTGTCTCCTGAGGAAGTTAGTGAAACACATGGAACAGAGAAACTGACGATAACGTAT

TCATCGTCTATGATGTGGGAAATCAATGGTCCGGAATCCGTGCTAGTCAACACATATCAA

TGGATCATTAGAAATTGGGAAACTGTGAAGATTCAGTGGTCCCAGGACCCTACGATGTTG

TACAATAAGATGGAATTTGAGCCCTTCCAATCCTTGGTGCCCAAGGCTGCTAGAGGCCAG

TATAGTGGGTTTGTGAGGACATTATTCCAACAGATGCGTGATGTGTTGGGGACATTTGAC

ACTGTCCAAATAATAAAGCTCCTACCATTTGCAGCAGCCCCACCGGAACAGAGTAGGATG

CAATTTTCCTCTCTGACTGTGAACGTAAGAGGTTCAGGAATGAGAATACTTGTGAGGGGC

AACTCCCCTGTTTTCAACTATAATAAGGCCACCAAGAGACTCACAGTTCTTGGAAAGGAT

GCAGGCGCCTTGACAGAATATCCAGATGAGGGAACAGCAGGAGTGGAGTCTGCAGTATTA

AGAGGATTTCTAATTCTGGGCAAAGAGGACAAAAGATATGGACCAGCATTGAGCATCAAC

GAATTGAGCAATCTTGCGAAAGGGGAAAAGGCTAATGTGTTGATAGGACAAGGAGACGTG

GTGCTGGTAATGAAACGGAAACGGGACTCTAGCATACTTACTGACAGCCAGACAGCGACC

AAAAGAATTCGGATGGCCATCAATTAGTGTCGAATTGTTTAA------------------

----------------------

>A_domestic_duck_Kazakhstan_1-274-20-B_2020_EPI1811608

------AGCAGGTCAAATATATTCAATATGGAGAGAATAAAAGAACTAAGAGATTTGATG

TCGCAGTCTCGCACTCGCGAGATACTAACAAAAACCACTGTGGACCATATGGCCATAATA

AAGAAATACACATCAGGAAGACAGGAGAAGAACCCTGCCCTCAGGATGAAATGGATGATG

GCAATGAAATATCCTATTACAGCTGACAAAAGAATAATGGAGATGATCCCTGAAAGGAAT

GAGCAAGGTCAGACTCTCTGGAGCAAAACAAATGATGCTGGATCAGACAGAGTGATGGTC

TCACCTCTGGCTGTGACATGGTGGAATAGAAATGGGCCAACAACAAGTACAGTACACTAC

CCAAAAGTCTACAAAACCTACTTTGAAAAGGTAGAAAGGTTGAAACATGGAACCTTTGGT

CCTGTTCACTTTCGAAATCAGGTTAAGATACGCCGCAGGGTTGACATAAACCCGGGCCAT

GCAGATCTCAGTGCCAAAGAAGCACAGGATGTCATCATGGAGGTTGTTTTCCCAAATGAA

GTTGGAGCCAGGATCTTGACATCAGAATCACAATTAACAATAACAAGGGAAAAGAAGGAG

GAACTTCAGGATTGCAAGATTGCTCCTTTGATGGTGGCATACATGTTGGAAAGAGAACTG

GTTCGCAAGACCAGATTTTTACCAGTAGCTGGCGGAACAAGCAGCGTATACATCGAGGTA

TTGCATTTGACTCAAGGGACCTGCTGGGAACAAATGTACACACCAGGAGGGGAGGTGAGA

AATGATGATGTTGATCAGAGTTTGATCATTGCTGCTAGAAATATAGTTAGGAGGGCAACA

GTATCAGCAGACCCATTGGCTTCGCTCTTGGAAATGTGCCACAGTACACAAATTGGTGGA

GTAAGGATGGTGGACATTCTTAGGCAGAACCCAACAGAGGAGCAAGCTGTGGATATATGC

AAAGCAGCAATGGGTTTAAGAATCAGTTCATCCTTCAGCTTTGGAGGTTTCACTTTCAAA

AGGACAAGTGGGTCGTCTGTCAAAAGAGAAGAAGAAATACTCACTGGCAACCTCCAAACA

CTGAAAGTAAGAATACATGAAGGATATGAGGAATTCACAATGGTTGGGCGAAGAGCTACA

GCCATTTTGAGGAAAGCAACCAGGAGACTGATCCAATTAATAGTGAGTGGAAGAGACGAG

CAGTCAATCGCTGAAGCAATCATAGTGGCAATGGTTTTCTCACAGGAGGATTGCATGATA

AAAGCAGTACGAGGTGATTTGAATTTTGTCAACAGAGCGAATCAGCGGCTAAATCCTATG

CATCAACTTCTGAGGCATTTCCAAAAGGATGCAAAAGTGCTGTTTCAAAACTGGGGGATT

GAACCAATTGACAATGTAATGGGGATGATCGGGATACTGCCCGACATGACCCCCAGCACA

GAGATGTCACTGAGAGGAGTGAGAGTCAGCAAAATGGGAGTGGATGAATATTCCAGTACT

GAGAGAGTGGTCGTGAGCATTGATCGCTTCTTGAGAGTCCGAGATCAAAGGGGAAATGTG

CTTCTGTCTCCTGAGGAAGTTAGTGAAACACATGGAACAGAGAAACTGACGATAACGTAT

TCATCGTCTATGATGTGGGAAATCAATGGTCCGGAATCCGTGCTAGTCAACACATATCAA

TGGATCATTAGAAGTTGGGAAACTGTGAAGATTCAATGGTCCCAGGACCCTACGATGTTG

TACAATAAGATGGAATTTGAGCCCTTCCAATCCTTGGTGCCCAAGGCTGCTAGAGGCCAG

TATAGTGGGTTTGTGAGGACATTATTCCAACAGATGCGTGATGTGTTGGGGACATTTGAC

ACTGTCCAAATAATAAAGCTCCTACCATTTGCAGCAGCCCCACCGGAACAGAGTAGGATG

CAATTTTCCTCTCTGACTGTGAACGTAAGAGGTTCAGGAATGAGAATACTTGTGAGGGGC

AACTCCCCTGTGTTCAACTATAATAAGGCCACCAAGAGACTCACAGTTCTTGGAAAGGAT

GCAGGCGCCTTGACAGAATATCCAGATGAGGGAACAGCAGGAGTGGAGTCTGCAGTATTA

AGAGGATTTCTAATTCTGGGCAAAGAGGACAAAAGATATGGACCAGCATTGAGCATCAAC

GAATTGAGCAATCTTGCGAAAGGGGAAAAGGCTAATGTGTTGATAGGACAAGGAGACGTG

GTGTTGGTAATGAAACGGAAACGGGACTCTAGCATACTTACTGACAGCCAGACAGCGACC

AAAAGAATTCGRATGGCCATCAATTAGTGTCGAATTGTTTAAAAACGACC----------

----------------------

>A_domestic_goose_Kazakhstan_1-242_2-20-B_2020_EPI1811616

--------------AAATATATTCAATATGGAGAGAATAAAAGAACTAAGAGATTTGATG

TCGCAGTCTCGCACTCGCGAGATACTAACAAAAACCACTGTGGACCATATGGCCATAATA

AAGAAATACACATCAGGAAGACAGGAGAAGAACCCTGCCCTCAGGATGAAATGGATGATG

GCAATGAAATATCCTATTACAGCTGACAAAAGAATAATGGAGATGATCCCTGAAAGGAAT

GAGCAAGGTCAGACTCTCTGGAGCAAAACAAATGATGCTGGATCAGACAGAGTGATGGTC

TCACCTCTGGCTGTGACATGGTGGAATAGAAATGGGCCAACAACAAGTACAGTACACTAC

CCAAAAGTCTACAAAACCTACTTTGAAAAGGTAGAAAGGTTGAAACATGGAACCTTTGGT

CCTGTTCACTTTCGAAATCAGGTTAAGATACGCCGCAGGGTTGACATAAACCCGGGCCAT

GCAGATCTCAGTGCCAAAGAAGCACAGGATGTCATCATGGAGGTTGTTTTCCCAAATGAA

GTTGGAGCCAGGATCTTGACATCAGAATCACAATTAACAATAACAARGGAAAAGAAGGAG

GAACTTCAGGATTGCAAGATTGCTCCTTTGATGGTGGCATACATGTTGGAAAGAGAACTG

GTTCGCAAGACCAGATTTTTACCAGTAGCTGGCGGAACAAGCAGCGTATACATCGAGGTA

TTGCATTTGACTCAAGGGACCTGCTGGGAACAAATGTACACACCAGGAGGGGAGGTGAGA

AATGATGATGTTGATCAGAGTTTGATCATTGCTGCTAGAAATATAGTTAGGAGGGCAACA

GTATCAGCAGACCCATTGGCTTCGCTCTTGGAGATGTGCCACAGTACACAAATTGGTGGA

GTAAGGATGGTGGACATTCTTAGGCAGAACCCAACAGAGGAGCAAGCTGTGGATATATGC

AAAGCAGCAATGGGTTTAAGAATCAGTTCATCCTTCAGCTTTGGAGGTTTCACTTTCAAA

AGGACAAGTGGGTCGTCTGTCAAAAGAGAAGAAGAAATACTCACTGGCAACCTCCAAACA

CTGAAAGTAAGAATACATGAAGGATATGAGGAATTCACAATGGTTGGGCGAAGAGCTACA

GCCATTTTGAGGAAAGCAACCAGGAGACTGATCCAATTAATAGTGAGTGGAAGAGACGAG

CAGTCAATCGCTGAAGCAATCATAGTGGCAATGGTTTTCTCACAGGAGGATTGCATGATA

AAAGCAGTACGAGGTGATTTGAATTTTGTCAACAGAGCGAATCAGCGGCTAAATCCTATG

CACCAACTTCTGAGGCATTTCCAAAAGGATGCAAAAGTGCTGTTTCAAAACTGGGGGATT

GAACCAATTGACAATGTAATGGGGATGATCGGGATACTGCCTGACATGACCCCCAGCACA

GAGATGTCACTGAGAGGAGTGAGAGTCAGCAAAATGGGAGTGGATGAATATTCCAGTACT

GAGAGAGTGGTCGTGAGCATTGATCGCTTCTTGAGAGTCCGAGACCAGAGGGGAAATGTG

CTTCTGTCTCCTGAGGAAGTTAGTGAAACACATGGAACAGAGAAACTGACGATAACGTAT

TCATCGTCTATGATGTGGGAAATCAATGGTCCGGAATCCGTGCTAGTCAACACATATCAA

TGGATCATTAGAAGTTGGGAAACTGTGAAGATTCAATGGTCCCAGGACCCTACGATGTTG

TACAATAAGATGGAATTTGAGCCCTTCCAATCCTTGGTGCCCAAGGCTGCTAGAGGCCAG

TATAGTGGGTTTGTGAGGACATTATTCCAACAGATGCGTGATGTGTTGGGGACATTTGAC

ACTGTCCAAATAATAAAGCTCCTACCATTTGCAGCAGCCCCACCGGAACAGAGTAGGATG

CAGTTTTCCTCTCTGACTGTGAACGTAAGAGGTTCAGGAATGAGAATACTTGTGAGGGGC

AACTCCCCTGTGTTCAACTATAATAAGGCCACCAAGAGACTCACAGTTCTTGGAAAGGAT

GCAGGCGCCTTGACAGAATATCCAGATGAGGGAACAGCAGGAGTGGAGTCTGCAGTATTA

AGAGGATTTCTAATTCTGGGCAAAGAGGACAAAAGATATGGACCAGCATTGAGCATCAAC

GAATTGAGCAATCTTGCGAAAGGGGAAAAGGCTAATGTGTTGATAGGACAAGGAGACGTG

GTGTTGGTAATGAAACGGAAACGGGACTCTAGCATACTTACTGACAGCCAGACAGCGACC

AAAAGAATTCGGATGGCCATCAATTAGTGTCG----------------------------

----------------------

>A_chicken_Iraq_1_2020_EPI1811625

--------------AAATATATTCAATATGGAGAGAATAAAAGAACTAAGAGATTTGATG

TCGCAGTCTCGCACTCGCGAGATACTAACAAAAACCACTGTGGACCATATGGCCATAATA

AAGAAATACACATCAGGAAGACAGGAGAAGAACCCTGCTCTCAGGATGAAATGGATGATG

GCAATGAAATATCCTATTACAGCTGACAAAAGAATAATGGAGATGATCCCTGAAAGGAAT

GAGCAAGGTCAGACTCTCTGGAGCAAAACAAATGATGCTGGATCAGACAGAGTGATGGTC

TCACCTCTGGCTGTGACATGGTGGAATAGAAATGGACCAACAACAAATACAGTACACTAC

CCAAAAGTCTACAAAACCTACTTTGAAAAGGTAGAAAGGTTGAAACATGGAACCTTTGGT

CCTGTTCACTTTCGAAATCAGGTTAAGATACGCCGCAGGGTTGACATAAACCCGGGCCAT

GCAGATCTCAGTGCCAAAGAAGCACAGGATGTCATCATGGAGGTTGTTTTCCCAAATGAA

GTTGGAGCCAGGATCTTGACGTCAGAATCACAATTAACAATAACAAGGGAAAAGAAAGAG

GAACTTCAGGATTGCAAGATTGCTCCTTTGATGGTGGCATACATGTTGGAAAGAGAACTG

GTTCGCAAGACCAGATTTTTACCAGTAGCTGGCGGAACAAGCAGCGTATACATCGAGGTA

TTGCATTTGACTCAAGGGACCTGCTGGGAACAAATGTACACACCAGGAGGGGAGGTGAGA

AATGATGATGTTGATCAGAGTTTGATCATTGCTGCTAGAAATATCGTTAGGAGGGCAACA

GTATCAGCAGACCCATTGGCTTCGCTCTTGGAAATGTGCCACAGTACACAAATTGGTGGA

GTAAGGATGGTGGACATTCTTAGGCAGAACCCAACAGAGGAGCAAGCTGTGGATATATGC

AAAGCAGCAATGGGTTTAAGAATCAGTTCATCCTTCAGCTTTGGAGGTTTCACTTTCAAA

AGGACAAGTGGGTCGTCTGTCAAAAGAGAAGAAGAAATACTCACTGGCAACCTCCAAACA

CTGAAAGTAAGAATACATGAAGGATATGAGGAATTCACAATGGTTGGGCGAAGAGCTACA

GCCATTTTGAGGAAAGCAACCAGGAGACTGATCCAATTAATAGTGAGTGGAAGAGACGAG

CAGTCAATCGCTGAAGCAATCATAGTGGCAATGGTTTTCTCACAGGAGGATTGCATGATA

AAAGCAGTACGAGGTGATTTGAATTTTGTCAACAGAGCGAATCAGCGGCTAAATCCTATG

CATCAACTTCTAAGGCATTTCCAAAAGGATGCAAAAGTGCTGTTTCAAAACTGGGGGATT

GAACCAATTGACAATGTAATGGGGATGATCGGGATACTGCCTGACATGACCCCCAGCACA

GAGATGTCACTGAGAGGAGTGAGAGTCAGCAAAATGGGAGTGGATGAATATTCCAGTACT

GAGAGAGTGGTCGTGAGCATTGATCGCTTCTTGAGAGTCCGAGATCAGAGGGGAAATGTG

CTTCTGTCTCCTGAGGAAGTTAGTGAAACACATGGAACAGAAAAACTGACGATAACGTAT

TCATCGTCTATGATGTGGGAAATCAATGGTCCGGAATCCGTGCTAGTCAACACATATCAA

TGGATCATTAGAAATTGGGAAACTGTGAAGATTCAGTGGTCCCAGGACCCTACGATGTTG

TACAATAAGATGGAATTTGAGCCCTTCCAATCCTTGGTGCCCAAGGCTGCTAGAGGCCAG

TATAGTGGATTTGTGAGGACATTATTCCAACAGATGCGTGATGTGTTGGGGACATTTGAC

ACTGTCCAAATAATAAAGCTCCTACCATTTGCAGCAGCTCCACCGGAACAGAGTAGGATG

CAATTTTCCTCTCTGACTGTGAACGTAAGGGGTTCAGGAATGAGAATACTTGTGAGGGGC

AACTCCCCCGTGTTCAACTATAATAAGGCCACCAAGAGACTCACAGTTCTTGGAAAGGAT

GCAGGCGCCTTGACAGAATATCCAGATGAGGGAACAGCAGGAGTGGAGTCTGCAGTATTA

AGAGGATTTCTAATTCTGGGCAAAGAGGACAAAAGATATGGACCAGCATTGAGCATCAAC

GAATTGGGCAATCTTGCGAAAGGGGAAAAGGCTAATGTGTTGATAGGACAAGGAGACGTG

GTGTTGGTAATGAAACGGAAACGGGACTCTAGCATACTTACTGACAGCCAGACAGCGACC

AAAAGAATTCGGATGGCCATCAATTAGTGTCGAATTGTTTAA------------------

----------------------

>A_whooper_swan_Inner_Mongolia_w1-1_2020_EPI1811641

---------------------------ATGGAGAGAATAAAAGAACTAAGAGATTTGATG

TCGCAGTCTCGCACTCGCGAGATACTAACAAAAACCACTGTGGACCATATGGCCATAATA

AAGAAATACACATCAGGAAGACAGGAGAAGAACCCTGCCCTCAGGATGAAATGGATGATG

GCAATGAAATATCCTATTACAGCTGACAAAAGAATAATGGAGATGATCCCTGAAAGGAAT

GAGCAAGGTCAGACTCTCTGGAGCAAAACAAATGATGCTGGATCAGACAGAGTGATGGTC

TCACCTCTGGCTGTGACATGGTGGAATAGGAATGGGCCAACAACAAGTACAGTACACTAC

CCAAAAGTCTACAAAACCTACTTTGAAAAGGTAGAAAGGTTGAAACATGGAACCTTTGGT

CCTGTTCACTTTCGAAATCAGGTTAAGATACGCCGCAGGGTTGACATAAACCCGGGCCAT

GCAGATCTCAGTGCCAAAGAAGCACAGGATGTCATCATGGAGGTTGTTTTCCCAAATGAA

GTTGGAGCCAGGATCTTGACATCAGAATCACAATTAACAATAACAAGGGAAAAGAAGGAG

GAACTTCAGGATTGCAAGATTGCTCCTTTGATGGTGGCATACATGTTGGAAAGAGAACTG

GTTCGCAAGACCAGATTTTTACCAGTAGCTGGCGGAACAAGCAGCGTATACATCGAGGTA

TTGCATTTGACTCAAGGGACCTGCTGGGAACAAATGTACACACCAGGAGGGGAGGTGAGA

AATGATGATGTTGATCAGAGTTTGATCATTGCTGCTAGAAATATAGTTAGGAGGGCAACA

GTATCAGCAGACCCATTGGCTTCGCTCTTGGAAATGTGCCACAGTACACAAATTGGTGGA

GTAAGGATGGTGGACATTCTTAGGCAGAACCCAACAGAGGAGCAAGCTGTGGATATATGC

AAAGCAGCAATGGGTTTAAGAATCAGTTCATCCTTCAGCTTTGGAGGTTTCACTTTCAAA

AGGACAAGTGGGTCGTCTGTCAAAAGAGAAGAAGAAATACTCACTGGCAACCTCCAAACA

CTGAAAGTTAGAATACATGAAGGATATGAGGAATTCACAATGGTTGGGCGAAGAGCTACA

GCCATTTTGAGGAAAGCAACCAGGAGACTGATCCAATTAATAGTGAGTGGAAGAGACGAG

CAGTCAATCGCTGAAGCAATCATAGTGGCAATGGTTTTCTCACAGGAGGATTGCATGATA

AAAGCAGTACGAGGTGATCTGAATTTTGTCAACAGAGCGAATCAGCGGCTAAATCCTATG

CATCAACTTCTGAGGCATTTCCAAAAGGATGCAAAAGTGCTGTTTCAAAACTGGGGGATT

GAACCAATTGACAATGTAATGGGGATGATCGGGATACTGCCAGACATGACCCCCAGCACA

GAGATGTCACTGAGAGGAGTGAGAGTCAGCAAAATGGGAGTGGATGAATATTCCAGTACT

GAGAGAGTGGTCGTGAGCATTGATCGCTTCTTGAGAGTCCGAGATCAGAGGGGAAATGTG

CTTCTGTCTCCTGAGGAAGTTAGTGAAACACATGGAACAGAGAAACTGACGATAACGTAT

TCATCGTCTATGATGTGGGAAATCAATGGTCCGGAATCCGTGCTAGTCAACACATATCAA

TGGATCATTAGAAGTTGGGAAACTGTGAAGATTCAATGGTCCCAGGACCCTACGATGTTG

TACAATAAGATGGAATTTGAGCCCTTCCAATCCTTGGTGCCCAAGGCTGCTAGAGGCCAG

TATAGTGGGTTTGTGAGGACATTATTCCAACAGATGCGTGATGTGTTGGGGACATTTGAC

ACTGTCCAAATAATAAAGCTCCTACCATTTGCAGCAGCCCCACCGGAACAGAGTAGGATG

CAATTTTCCTCTCTGACTGTGAACGTAAGAGGTTCAGGAATGAGAATACTTGTGAGGGGC

AACTCCCCTGTGTTCAACTATAATAAAGCCACCAAGAGACTCACAGTTCTTGGAAAGGAT

GCAGGCGCCTTGACAGAATATCCAGATGAGGGAACAGCAGGAGTGGAGTCTGCAGTATTA

AGAGGATTTCTAATTCTGGGCAAAGAGGACAAAAGATATGGACCAGCATTGAGCATCAAC

GAATTGAGCAATCTTGCGAAAGGGGAAAAGGCTAATGTGTTGATAGGACAAGGAGACGTG

GTGTTGGTAATGAAACGGAAACGGGACTCTAGCATACTTACTGACAGCCAGACAGCGACC

AAAAGAATTCGGATGGCCATCAATTAG---------------------------------

----------------------

>A_mute_swan_Inner_Mongolia_w2-1_2020_EPI1811649

---------------------------ATGGAGAGAATAAAAGAACTAAGAGATTTGATG

TCGCAGTCTCGCACTCGCGAGATACTAACAAAAACCACTGTGGACCATATGGCCATAATA

AAGAAATACACATCAGGAAGACAGGAGAAGAACCCTGCCCTCAGGATGAAATGGATGATG

GCAATGAAATATCCTATTACAGCTGACAAAAGAATAATGGAGATGATCCCTGAAAGGAAT

GAGCAAGGTCAGACTCTCTGGAGCAAAACAAATGATGCTGGATCAGACAGAGTGATGGTC

TCACCTCTGGCTGTGACATGGTGGAATAGGAATGGGCCAACAACAAGTACAGTACACTAC

CCAAAAGTCTACAAAACCTACTTTGAAAAGGTAGAAAGGTTGAAACATGGAACCTTTGGT

CCTGTTCACTTTCGAAATCAGGTTAAGATACGCCGCAGGGTTGACATAAACCCGGGCCAT

GCAGATCTCAGTGCCAAAGAAGCACAGGATGTCATCATGGAGGTTGTTTTCCCAAATGAA

GTTGGAGCCAGGATCTTGACATCAGAATCACAATTAACAATAACAAGGGAAAAGAAGGAG

GAACTTCAGGATTGCAAGATTGCTCCTTTGATGGTGGCATACATGTTGGAAAGAGAACTG

GTTCGCAAGACCAGATTTTTACCAGTAGCTGGCGGAACAAGCAGCGTATACATCGAGGTA

TTGCATTTGACTCAAGGGACCTGCTGGGAACAAATGTACACACCAGGAGGGGAGGTGAGA

AATGATGATGTTGATCAGAGTTTGATCATTGCTGCTAGAAATATAGTTAGGAGGGCAACA

GTATCAGCAGACCCATTGGCTTCGCTCTTGGAAATGTGCCACAGTACACAAATTGGTGGA

GTAAGGATGGTGGACATTCTTAGGCAGAACCCAACAGAGGAGCAAGCTGTGGATATATGC

AAAGCAGCAATGGGTTTAAGAATCAGTTCATCCTTCAGCTTTGGAGGTTTCACTTTCAAA

AGGACAAGTGGGTCGTCTGTCAAAAGAGAAGAAGAAATACTCACTGGCAACCTCCAAACA

CTGAAAGTTAGAATACATGAAGGATATGAGGAATTCACAATGGTTGGGCGAAGAGCTACA

GCCATTTTGAGGAAAGCAACCAGGAGACTGATCCAATTAATAGTGAGTGGAAGAGACGAG

CAGTCAATCGCTGAAGCAATCATAGTGGCAATGGTTTTCTCACAGGAGGATTGCATGATA

AAAGCAGTACGAGGTGATCTGAATTTTGTCAACAGAGCGAATCAGCGGCTAAATCCTATG

CATCAACTTCTGAGGCATTTCCAAAAGGATGCAAAAGTGCTGTTTCAAAACTGGGGGATT

GAACCAATTGACAATGTAATGGGGATGATCGGGATACTGCCAGACATGACCCCCAGCACA

GAGATGTCACTGAGAGGAGTGAGAGTCAGCAAAATGGGAGTGGATGAATATTCCAGTACT

GAGAGAGTGGTCGTGAGCATTGATCGCTTCTTGAGAGTCCGAGATCAGAGGGGAAATGTG

CTTCTGTCTCCTGAGGAAGTTAGTGAAACACATGGAACAGAGAAACTGACGATAACGTAT

TCATCGTCTATGATGTGGGAAATCAATGGTCCGGAATCCGTGCTAGTCAACACATATCAA

TGGATCATTAGAAGTTGGGAAACTGTGAAGATTCAATGGTCCCAGGACCCTACGATGTTG

TACAATAAGATGGAATTTGAGCCCTTCCAATCCTTGGTGCCCAAGGCTGCTAGAGGCCAG

TATAGTGGGTTTGTGAGGACATTATTCCAACAGATGCGTGATGTGTTGGGGACATTTGAC

ACTGTCCAAATAATAAAGCTCCTACCATTTGCAGCAGCCCCACCGGAACAGAGTAGGATG

CAATTTTCCTCTCTGACTGTGAACGTAAGAGGTTCAGGAATGAGAATACTTGTGAGGGGC

AACTCCCCTGTGTTCAACTATAATAAAGCCACCAAGAGACTCACAGTTCTTGGAAAGGAT

GCAGGCGCCTTGACAGAATATCCAGATGAGGGAACAGCAGGAGTGGAGTCTGCAGTATTA

AGAGGATTTCTAATTCTGGGCAAAGAGGACAAAAGATATGGACCAGCATTGAGCATCAAC

GAATTGAGCAATCTTGCGAAAGGGGAAAAGGCTAATGTGTTGATAGGACAAGGAGACGTG

GTGTTGGTAATGAAACGGAAACGGGACTCTAGCATACTTACTGACAGCCAGACAGCGACC

AAAAGAATTCGGATGGCCATCAATTAG---------------------------------

----------------------

>A_goose_Russian_Federation_Kurgan_1345-25_2020_EPI1811685

------------TCAAATATATTCAATATGGAGAGAATAAAAGAACTAAGAGATTTGATG

TCGCAGTCTCGCACTCGCGAGATACTAACAAAAACCACTGTGGACCATATGGCCATAATA

AAGAAATACACATCAGGAAGACAGGAGAAGAACCCTGCCCTCAGGATGAAATGGATGATG

GCAATGAAATATCCTATTACAGCTGACAAAAGAATAATGGAGATGATCCCTGAAAGGAAT

GAGCAAGGTCAGACTCTCTGGAGCAAAACAAATGATGCTGGATCAGACAGAGTGATGGTC

TCACCTCTGGCTGTGACATGGTGGAATAGAAATGGGCCAACAACAAGTACAGTACACTAC

CCAAAAGTCTACAAAACCTACTTTGAAAAGGTAGAAAGGTTGAAACATGGAACCTTTGGT

CCTGTTCACTTTCGAAATCAGGTTAAGATACGCCGCAGGGTTGACATAAACCCGGGCCAT

GCAGATCTCAGTGCCAAAGAAGCACAGGATGTCATCATGGAGGTTGTTTTCCCAAATGAA

GTTGGAGCCAGGATCTTGACATCAGAATCACAATTAACAATAACAAGGGAAAAGAAGGAG

GAACTTCAGGATTGCAAGATTGCTCCTTTGATGGTGGCATACATGTTGGAAAGAGAACTG

GTTCGCAAGACCAGATTTTTACCAGTAGCTGGCGGAACAAGCAGCGTATACATCGAGGTA

TTGCATTTGACTCAAGGGACCTGCTGGGAACAAATGTACACACCAGGAGGGGAGGTGAGA

AATGATGATGTTGATCAGAGTTTGATCATTGCTGCTAGAAATATAGTTAGGAGGGCAACA

GTATCAGCAGACCCATTGGCTTCGCTTTTGGAAATGTGCCACAGTACACAAATTGGTGGA

GTAAGGATGGTGGACATTCTTAGGCAGAACCCAACAGAGGAGCAAGCTGTGGATATATGC

AAAGCAGCAATGGGTTTAAGAATCAGTTCATCCTTCAGCTTTGGAGGTTTCACTTTCAAA

AGGACAAGTGGGTCGTCTGTCAAAAGAGAAGAAGAAATACTCACTGGCAACCTCCAAACA

CTGAAAGTAAGAATACATGAAGGATATGAGGAATTCACAATGGTTGGGCGAAGAGCTACA

GCCATTTTGAGGAAAGCAACCAGGAGACTGATCCAATTAATAGTGAGTGGAAGAGACGAG

CAGTCAATCGCTGAAGCAATCATAGTGGCAATGGTTTTCTCACAGGAGGATTGCATGATA

AAAGCAGTACGAGGTGATTTGAATTTTGTCAACAGAGCGAATCAGCGGCTAAATCCTATG

CATCAACTTCTGAGGCATTTCCAAAAGGATGCAAAAGTGCTGTTTCAAAACTGGGGGATT

GAACCAATTGACAATGTAATGGGGATGATCGGGATACTGCCTGACATGACCCCCAGCACA

GAGATGTCACTGAGAGGAGTGAGAGTCAGCAAAATGGGAGTGGATGAATATTCCAGTACT

GAGAGAGTGGTCGTGAGCATTGATCGCTTCTTGAGAGTCCGAGATCAGAGGGGAAATGTG

CTTCTGTCTCCTGAGGAAGTTAGTGAAACACATGGAACAGAGAAACTGACGATAACGTAT

TCATCGTCTATGATGTGGGAAATCAATGGTCCGGAATCCGTGCTAGTCAACACATATCAA

TGGATCATTAGAAATTGGGAAACTGTGAAGATTCAGTGGTCCCAGGACCCTACGATGTTG

TACAATAAGATGGAATTTGAGCCCTTCCAATCCTTGGTGCCCAAGGCTGCTAGAGGCCAG

TATAGTGGGTTTGTGAGGACATTATTCCAACAGATGCGTGATGTGTTGGGGACATTTGAC

ACTGTCCAAATAATAAAGCTCCTACCATTTGCAGCAGCCCCACCGGAACAGAGTAGGATG

CAATTTTCCTCTCTGACTGTGAACGTAAGAGGTTCAGGAATGAGAATACTTGTGAGGGGC

AACTCCCCTGTGTTCAACTATAATAAGGCCACCAAGAGACTCACAGTTCTTGGAAAGGAT

GCAGGCGCCTTGACAGAATATCCAGATGAGGGAACAGCAGGAGTGGAGTCTGCAGTATTA

AGAGGATTTCTAATTCTGGGCAAAGAGGACAAAAGATATGGACCAGCATTGAGCATCAAC

GAATTGAGCAATCTTGCGAAAGGGGAAAAGGCTAATGTGTTGATAGGACAAGGAGACGTG

GTGTTGGTAATGAAACGGAAACGGGACTCTAGCATACTTACTGACAGCCAGACAGCGACC

AAAAGAATTCGGATGGCCATCAATTAGTGTCGAATTGTTTAAAAACGA------------

----------------------

>A_duck_Chelyabinsk_1207-1_2020_EPI1812530

------------TCAAATATATTCAATATGGAGAGAATAAAAGAACTAAGAGATTTGATG

TCGCAGTCTCGCACTCGCGAGATACTAACAAAAACCACTGTGGACCATATGGCCATAATA

AAGAAATACACATCAGGAAGACAGGAGAAGAACCCTGCCCTCAGGATGAAATGGATGATG

GCAATGAAATATCCTATTACAGCTGACAAAAGAATAATGGAGATGATCCCTGAAAGGAAT

GAGCAAGGTCAGACTCTCTGGAGCAAAACAAATGATGCTGGATCAGACAGAGTGATGGTC

TCACCTCTGGCTGTGACATGGTGGAATAGAAATGGGCCAACAACAAGTACAGTACACTAC

CCAAAAGTCTACAAAACCTACTTTGAAAAGGTAGAAAGGTTGAAACATGGAACCTTTGGT

CCTGTTCACTTTCGAAATCAGGTTAAGATACGCCGCAGGGTTGACATAAACCCGGGCCAT

GCAGATCTCAGTGCCAAAGAAGCACAGGATGTCATCATGGAGGTTGTTTTCCCAAATGAA

GTTGGAGCCAGGATCTTGACATCAGAATCACAATTAACAATAACAAGGGAAAAGAAGGAG

GAACTTCAGGATTGCAAGATTGCTCCTTTGATGGTGGCATACATGTTGGAAAGAGAACTG

GTTCGCAAGACCAGATTTTTACCAGTAGCTGGCGGAACAAGCAGCGTATACATCGAGGTA

TTGCATTTGACTCAAGGGACCTGCTGGGAACAAATGTACACACCAGGAGGGGAGGTGAGA

AATGATGATGTTGATCAGAGTTTGATCATTGCTGCTAGAAATATAGTTAGGAGGGCAACA

GTATCAGCAGACCCATTGGCTTCGCTCTTGGAAATGTGCCACAGTACACAAATTGGTGGA

GTAAGGATGGTGGACATTCTTAGGCAGAACCCAACAGAGGAGCAAGCTGTGGATATATGC

AAAGCAGCAATGGGTTTAAGAATCAGTTCATCCTTCAGCTTTGGAGGTTTCACTTTCAAA

AGGACAAGTGGGTCGTCTGTCAAAAGAGAAGAAGAAATACTCACTGGCAACCTCCAAACA

CTGAAAGTAAGAATACATGAAGGATATGAGGAATTCACAATGGTTGGGCGAAGAGCTACA

GCCATTTTGAGGAAAGCAACCAGGAGACTGATCCAATTAATAGTGAGTGGAAGAGACGAA

CAGTCAATCGCTGAAGCAATCATAGTGGCAATGGTTTTCTCACAGGAGGATTGCATGATA

AAAGCAGTACGAGGTGATTTGAATTTTGTCAACAGAGCGAATCAGCGGCTAAATCCTATG

CATCAACTTCTGAGGCATTTCCAAAAGGATGCAAAAGTGCTGTTTCAAAACTGGGGGATT

GAACCAATTGACAATGTAATGGGGATGATCGGGATACTGCCTGACATGACCCCCAGCACA

GAGATGTCACTGAGAGGAGTGAGAGTCAGCAAAATGGGAGTGGATGAATATTCCAGTACT

GAGAGAGTGGTCGTGAGCATTGATCGCTTCTTGAGAGTCCGAGATCAGAGGGGAAATGTG

CTTCTGTCTCCTGAGGAAGTTAGTGAAACACATGGAACAGAGAAACTGACGATAACGTAT

TCATCGTCTATGATGTGGGAAATCAATGGTCCGGAATCCGTGCTAGTCAACACATATCAA

TGGATCATTAGAAATTGGGAAACTGTGAAGATTCAGTGGTCCCAGGACCCTACGATGTTG

TACAATAAGATGGAATTTGAACCCTTCCAATCCTTGGTGCCCAAGGCTGCTAGAGGCCAG

TATAGTGGGTTTGTGAGGACATTATTCCAACAGATGCGTGATGTGTTGGGGACATTTGAC

ACTGTCCAAATAATAAAGCTCCTACCATTTGCAGCAGCCCCACCGGAACAGAGTAGGATG

CAATTTTCCTCTCTGACTGTGAACGTAAGAGGTTCAGGAATGAGAATACTTGTGAGGGGC

AACTCCCCTGTGTTCAACTATAATAAGGCCACCAAGAGACTCACAGTTCTTGGAAAGGAT

GCAGGCGCCTTGACAGAATATCCAGACGAGGGAACAGCAGGAGTGGAGTCTGCAGTATTA

AGAGGATTTCTAATTCTGGGCAAAGAGGACAAAAGATATGGACCAGCATTGAGCATCAAC

GAATTGAGCAATCTTGCGAAAGGGGAAAAGGCTAATGTGTTGATAGGACAAGGAGACGTG

GTGTTGGTAATGAAACGGAAACGGGACTCTAGCATACTTACTGACAGCCAGACAGCGACC

AAAAGAATTCGGATGGCCATCAATTAGTGTCGAATTGTTTAAAAACGA------------

----------------------

>A_goose_Omsk_0002_2020_EPI1813118

AGCAAAAGCAGGTCAAATATATTCAATATGGAGAGAATAAAAGAACTAAGAGATTTGATG

TCGCAGTCTCGCACTCGCGAGATACTAACAAAAACCACTGTGGACCATATGGCCATAATA

AAGAAATACACATCAGGGAGACAGGAGAAGAACCCTGCCCTCAGGATGAAATGGATGATG

GCAATGAAATATCCTATTACAGCTGACAAAAGAATAATGGAGATGATCCCTGAAAGGAAT

GAGCAAGGTCAGACTCTCTGGAGCAAAACAAATGATGCTGGATCAGACAGAGTGATGGTC

TCACCTCTGGCTGTGACATGGTGGAATAGAAATGGGCCAACAACAAGTACAGTACACTAC

CCAAAAGTCTACAAAACCTACTTTGAAAAGGTAGAAAGGTTGAAACATGGAACCTTTGGT

CCTGTTCACTTTCGAAATCAGGTTAAGATACGCCGCAGGGTTGACATAAACCCGGGCCAT

GCAGATCTCAGTGCCAAAGAAGCACAGGATGTCATCATGGAGGTTGTTTTCCCAAATGAA

GTTGGAGCCAGGATCTTGACATCAGAATCACAATTAACAATAACAAGGGAAAAGAAGGAG

GAACTTCAGGATTGCAAGATTGCTCCTTTGATGGTGGCATACATGTTGGAAAGAGAACTG

GTTCGCAAGACCAGATTTTTACCAGTAGCTGGCGGAACAAGCAGCGTATACATCGAGGTA

TTGCATTTGACTCAAGGGACCTGCTGGGAACAAATGTACACACCAGGAGGGGAGGTGAGA

AATGATGATGTTGATCAGAGTTTGATCATTGCTGCTAGAAATATAGTTAGGAGGGCAACA

GTATCAGCAGACCCATTGGCTTCGCTCTTGGAAATGTGCCACAGTACACAAATTGGTGGA

GTGAGGATGGTGGACATTCTTAGGCAGAACCCAACAGAGGAGCAAGCTGTGGATATATGC

AAAGCAGCAATGGGTTTAAGAATCAGTTCATCCTTCAGCTTTGGAGGTTTCACTTTCAAA

AGGACAAGTGGGTCGTCTGTCAAAAGAGAAGAAGAAATACTCACTGGCAACCTCCAAACA

CTGAAAGTAAGAATACATGAAGGATATGAGGAATTCACAATGGTTGGGCGAAGAGCTACA

GCCATTTTGAGGAAAGCAACCAGGAGACTGATCCAATTAATAGTGAGTGGACGAGACGAG

CAGTCAATCGCTGAAGCAATCATAGTGGCAATGGTTTTCTCACAGGAGGATTGCATGATA

AAAGCAGTACGAGGTGATTTGAATTTTGTCAACAGAGCGAATCAGCGGCTAAATCCTATG

CATCAACTTCTGAGGCATTTCCAAAAGGATGCAAAAGTGCTGTTTCAAAACTGGGGGATT

GAACCAATTGACAATGTAATGGGGATGATCGGGATACTGCCTGACATGACCCCCAGCACA

GAGATGTCACTGAGAGGAGTGAGAGTCAGCAAAATGGGAGTGGATGAATATTCCAGTACT

GAGAGAGTGGTCGTGAGCATTGATCGCTTCTTGAGAGTCCGAGATCAGAGGGGAAATGTG

CTTCTGTCTCCTGAGGAAGTTAATGAAACACATGGAACAGAGAAACTGACGATAACGTAT

TCATCGTCTATGATGTGGGAAATCAATGGTCCGGAATCCGTGCTAGTCAACACATATCAA

TGGATCATTAGAAATTGGGAAACTGTGAAGATTCAGTGGTCCCAGGACCCTACGATGTTG

TACAATAAGATGGAATTTGAGCCCTTCCAATCCTTAGTGCCCAAGGCTGCTAGAGGCCAG

TATAGTGGGTTTGTGAGGACATTATTCCAACAGATGCGTGATGTGTTGGGGACATTTGAC

ACTGTCCAAATAATAAAGCTCCTACCATTTGCAGCAGCCCCACCGGAACAGAGTAGGATG

CAATTTTCCTCTCTGACTGTGAACGTAAGAGGTTCAGGAATGAGAATACTTGTGAGGGGC

AACTCCCCTGTGTTCAACTATAATAAGGCCACCAAGAGACTCACAGTTCTTGGAAAGGAT

GCAGGCGCCTTGACAGAATATCCAGATGAGGGAACAGCAGGAGTGGAGTCTGCAGTATTA

AGAGGATTTCTAATTCTGGGCAAAGAGGACAAAAGATATGGACCAGCATTGAGCATCAAC

GAATTGAGCAATCTTGCGAAAGGGGAAAAGGCTAATGTGTTGATAGGACAAGGAGACGTG

GTGTTGGTAATGAAACGGAAACGGGACTCTAGCATACTTACTGACAGCCAGACAGCGACC

AAAAGAATTCGGATGGCCATCAATTAGTGTCGAATTGTTTAAAAACGACCTTGTTTCTAC

T---------------------

>A_goose_Omsk_01171_2020_EPI1813198

AGCAAAAGCAGGTCAAATATATTCAATATGGAGAGAATAAAAGAACTAAGAGATTTGATG

TCGCAGTCTCGCACTCGCGAGATACTAACAAAAACCACTGTGGACCATATGGCCATAATA

AAGAAATACACATCAGGAAGACAGGAGAAGAACCCTGCCCTCAGGATGAAATGGATGATG

GCAATGAAATATCCTATTACAGCTGACAAAAGAATAATGGAGATGATCCCTGAAAGGAAT

GAGCAAGGTCAGACTCTCTGGAGCAAAACAAATGATGCTGGATCAGACAGAGTGATGGTC

TCACCTCTGGCTGTGACATGGTGGAATAGAAATGGGCCAACAACAAGTACAGTACACTAC

CCAAAAGTCTACAAAACCTACTTTGAAAAGGTAGAAAGGTTGAAACATGGAACCTTTGGT

CCTGTTCACTTTCGAAATCAGGTTAAGATACGCCGCAGGGTTGACATAAACCCGGGCCAT

GCAGATCTCAGTGCCAAAGAAGCACAGGATGTCATCATGGAGGTTGTTTTCCCAAATGAA

GTTGGAGCCAGGATCTTGACATCAGAATCACAATTAACAATAACAAGGGAAAAGAAGGAG

GAACTTCAGGATTGCAAGATTGCTCCTTTGATGGTGGCATACATGTTGGAAAGAGAACTG

GTTCGCAAGACCAGATTTTTACCAGTAGCTGGCGGAACAAGCAGCGTATACATCGAGGTA

TTGCATTTGACTCAAGGGACCTGCTGGGAACAAATGTACACACCAGGAGGGGAGGTGAGA

AATGATGATGTTGATCAGAGTTTGATCATTGCTGCTAGAAATATAGTTAGGAGGGCAACA

GTATCAGCAGACCCATTGGCTTCGCTCTTGGAAATGTGCCACAGTACACAAATTGGTGGA

GTAAGGATGGTGGACATTCTTAGGCAGAACCCAACAGAGGAGCAAGCTGTGGATATATGC

AAAGCAGCAATGGGTTTAAGAATCAGTTCATCCTTCAGCTTTGGAGGTTTCACTTTCAAA

AGGACAAGTGGGTCGTCTGTCAAAAGAGAAGAAGAAATACTCACTGGCAACCTCCAAACA

CTGAAAGTAAGAATACATGAAGGATATGAGGAATTCACAATGGTTGGGCGAAGGGCTACA

GCCATTTTGAGGAAAGCAACCAGGAGACTGATCCAATTAATAGTGAGTGGAAGAGACGAG

CAGTCAATCGCTGAAGCAATCATAGTGGCAATGGTTTTCTCACAGGAGGATTGCATGATA

AAAGCAGTACGAGGTGATTTGAATTTTGTCAACAGAGCGAATCAGCGGCTAAATCCTATG

CATCAACTTCTGAGGCATTTCCAAAAGGATGCAAAAGTGCTGTTTCAAAACTGGGGGATT

GAACCAATTGACAATGTAATGGGGATGATCGGGATACTGCCCGACATGACCCCCAGCACA

GAGATGTCACTGAGAGGAGTGAGAGTCAGCAAAATGGGAGTGGATGAATATTCCAGTACT

GAGAGAGTGGTCGTGAGCATTGATCGCTTCTTGAGAGTCCGAGATCAGAGGGGAAATGTG

CTTCTGTCTCCTGAGGAAGTTAGTGAAACACATGGAACAGAGAAACTGACGATAACGTAT

TCATCGTCTATGATGTGGGAAATCAATGGTCCGGAATCCGTGCTAGTCAACACATATCAA

TGGATCATTAGAAGTTGGGAAACTGTGAAGATTCAATGGTCCCAGGACCCTACGATGTTG

TACAATAAGATGGAATTTGAGCCCTTCCAATCCTTGGTGCCCAAGGCTGCTAGAGGCCAG

TATAGTGGGTTTGTGAGGACATTATTCCAACAGATGCGTGATGTGTTGGGGACATTTGAC

ACTGTCCAAATAATAAAGCTCCTACCATTTGCAGCAGCCCCACCGGAACAGAGTAGGATG

CAATTTTCCTCTCTGACTGTGAACGTAAGAGGTTCAGGAATGAGAATACTTGTGAGGGGC

AACTCCCCTGTGTTCAACTATAATAAGGCCACCAAGAGACTCACAGTTCTTGGAAAGGAT

GCAGGCGCCTTGACAGAATATCCAGATGAGGGAACAGCAGGAGTGGAGTCTGCAGTATTA

AGAGGATTTCTAATTCTGGGCAAAGAGGACAAAAGATATGGACCAGCATTGAGCATCAAC

GAATTGAGCAATCTTGCGAAAGGGGAAAAGGCTAATGTGTTGATAGGACAAGGAGACGTG

GTGTTGGTAATGAAACGGAAACGGGACTCTAGCATACTTACTGACAGCCAGACAGCGACC

AAAAGAATTCGGATGGCCATCAATTAGTGTCGAATTGTTTAAAAACGACCTTGTTTCTAC

T---------------------

>A_duck_Omsk_0075_2020_EPI1813278

AGCAAAAGCAGGTCAAATATATTCAATATGGAGAGAATAAAAGAACTAAGAGATTTGATG

TCGCAGTCTCGCACTCGCGAGATACTAACAAAAACCACTGTGGACCATATGGCCATAATA

AAGAAATACACATCAGGAAGACAGGAGAAGAACCCTGCCCTCAGGATGAAATGGATGATG

GCAATGAAATATCCTATTACAGCTGACAAAAGAATAATGGAGATGATCCCTGAAAGGAAT

GAGCAAGGTCAGACTCTCTGGAGCAAAACAAATGATGCTGGATCAGACAGAGTGATGGTC

TCACCTCTGGCTGTGACATGGTGGAATAGAAATGGGCCAACAACAAGTACAGTACACTAC

CCAAAAGTCTACAAAACCTACTTTGAAAAGGTAGAAAGGTTGAAACATGGAACCTTTGGT

CCTGTTCACTTTCGAAATCAGGTTAAGATACGCCGCAGGGTTGACATAAACCCGGGCCAT

GCAGATCTCAGTGCCAAAGAAGCACAGGATGTCATCATGGAGGTTGTTTTCCCAAATGAA

GTTGGAGCCAGGATCTTGACATCAGAATCACAATTAACAATAACAAGGGAAAAGAAGGAG

GAACTTCAGGATTGCAAGATTGCTCCTTTGATGGTGGCATACATGTTGGAAAGAGAACTG

GTTCGCAAGACCAGATTTTTACCAGTAGCTGGCGGAACAAGCAGCGTATACATCGAGGTA

TTGCATTTGACTCAAGGGACCTGCTGGGAACAAATGTACACACCAGGAGGGGAGGTGAGA

AATGATGATGTTGATCAGAGTTTGGTCATTGCTGCTAGAAATATAGTTAGGAGGGCAACA

GTATCAGCAGACCCATTGGCTTCGCTCTTGGAAATGTGCCACAGTACACAAATTGGTGGA

GTAAGGATGGTGGACATTCTTAGGCAGAACCCAACAGAGGAGCAAGCTGTGGATATATGC

AAAGCAGCAATGGGTTTAAGAATCAGTTCATCCTTCAGCTTTGGAGGTTTCACTTTCAAA

AGGACAAGTGGGTCGTCTGTCAAAAGAGAAGAAGAAATACTCACTGGCAACCTCCAAACA

CTGAAAGTAAGGATACATGAAGGATATGAGGAATTCACAATGGTTGGGCGAAGAGCTACA

GCCATTTTGAGGAAAGCAACCAGGAGACTGATCCAATTAATAGTGAGTGGAAGAGACGAG

CAGTCAATCGCTGAAGCAATCATAGTGGCAATGGTTTTCTCACAGGAGGATTGCATGATA

AAAGCAGTACGAGGTGATTTGAATTTTGTCAACAGAGCGAATCAGCGGCTAAATCCTATG

CATCAACTTCTGAGGCATTTCCAAAAGGATGCAAAAGTGCTGTTTCAAAACTGGGGGATT

GAACCAATTGACAATGTAATGGGGATGATCGGGATACTGCCTGACATGACCCCCAGCACA

GAGATGTCACTGAGAGGAGTGAGAGTCAGCAAAATGGGAGTGGATGAATATTCCAGTACT

GAGAGAGTGGTCGTGAGCATTGATCGCTTCTTGAGAGTCCGAGATCAGAGGGGAAATGTG

CTTCTGTCTCCTGAGGAAGTTAGTGAAACACATGGAACAGAGAAACTGACGATAACGTAT

TCATCGTCTATGATGTGGGAAATCAATGGTCCGGAATCCGTGCTAGTCAACACATATCAA

TGGATCATTAGAAATTGGGAAACTGTGAAGATTCAGTGGTCCCAGGACCCTACGATGTTG

TACAATAAGATGGAATTTGAGCCCTTCCAATCCTTGGTGCCCAAGGCTGCTAGAGGCCAG

TATAGTGGGTTTGTGAGGACATTATTCCAACAGATGCGTGATGTGTTGGGGACATTTGAC

ACTGTCCAAATAATAAAGCTCCTACCATTTGCAGCAGCCCCACCGGAACAGAGTAGGATG

CAATTTTCCTCTCTGACTGTGAACGTAAGAGGTTCAGGAATGAGAATACTTGTGAGGGGC

AACTCCCCTGTGTTCAACTATAATAAGGCCACCAAGAGACTCACAGTTCTTGGAAAGGAT

GCAGGCGCCTTGACAGAATATCCAGATGAGGGAACAGCAGGAGTGGAGTCTGCAGTATTA

AGAGGATTTCTAATTCTGGGCAAAGAGGACAAAAGATATGGACCAGCATTGAGCATCAAC

GAATTGAGCAATCTTGCGAAAGGGGAAAAGGCTAATGTGTTGATAGGACAAGGAGACGTG

GTGTTGGTAATGAAACGGAAACGGGACTCTAGCATACTTACTGACAGCCAGACAGCGACC

AAAAGAATTCGGATGGCCATCAATTAGTGTCGAATTGTTTAAAAACGACCTTGTTTCTAC

T---------------------

>A_duck_Omsk_0004_2020_EPI1813334

AGCAAAAGCAGGTCAAATATATTCAATATGGAGAGAATAAAAGAACTAAGAGATTTGATG

TCGCAGTCTCGCACTCGCGAGATACTAACAAAAACCACTGTGGACCATATGGCCATAATA

AAGAAATACACATCAGGAAGACAGGAGAAGAACCCTGCCCTCAGGATGAAATGGATGATG

GCAATGAAATATCCTATTACAGCTGACAAAAGAATAATGGAGATGATCCCTGAAAGGAAT

GAGCAAGGTCAGACTCTCTGGAGCAAAACAAATGATGCTGGATCAGACAGAGTGATGGTC

TCACCTCTGGCTGTGACATGGTGGAATAGAAATGGGCCAACAACAAGTACAGTACACTAC

CCAAAAGTCTACAAAACCTACTTTGAAAAGGTAGAAAGGTTGAAACATGGAACCTTTGGT

CCTGTTCACTTTCGAAATCAGGTTAAGATACGCCGCAGGGTTGACATAAACCCAGGCCAT

GCAGATCTCAGTGCCAAAGAAGCACAGGATGTCATCATGGAGGTTGTTTTCCCAAATGAA

GTTGGAGCCAGGATCTTGACATCAGAATCACAATTAACAATAACAAGGGAAAAGAAGGAG

GAACTTCAGGATTGCAAGATTGCTCCTTTGATGGTGGCATACATGTTGGAAAGAGAACTG

GTTCGCAAGACCAGATTTTTACCAGTAGCTGGCGGAACAAGCAGCGTATACATCGAGGTA

TTGCATTTGACTCAAGGGACCTGCTGGGAACAAATGTACACACCAGGAGGGGAGGTGAGA

AATGATGATGTTGATCAGAGTTTGATCATTGCTGCTAGAAATATAGTTAGGAGGGCAACA

GTGTCAGCAGACCCATTGGCTTCGCTCTTGGAAATGTGCCACAGTACACAAATTGGTGGA

GTAAGGATGGTGGACATTCTTAGGCAGAACCCAACAGAGGAGCAAGCTGTGGATATATGC

AAAGCAGCAATGGGTTTAAGAATCAGTTCATCCTTCAGCTTTGGAGGTTTCACTTTCAAA

AGGACAAGTGGGTCGTCTGTCAAAAGAGAAGAAGAAATACTCACTGGCAACCTCCAAACA

CTGAAAGTAAGAATACATGAAGGATATGAGGAATTCACAATGGTTGGGCGAAGAGCTACA

GCCATTTTGAGGAAAGCAACCAGGAGACTGATCCAATTAATAGTGAGTGGAAGAGACGAG

CAGTCAATCGCTGAAGCAATCATAGTGGCAATGGTTTTCTCACAGGAGGATTGCATGATA

AAAGCAGTACGAGGTGATTTGAATTTTGTCAACAGAGCGAATCAGCGGCTAAATCCTATG

CATCAACTTCTGAGGCATTTCCAAAAGGATGCAAAAGTGCTGTTTCAAAACTGGGGGATT

GAACCAATTGACAATGTAATGGGGATGATCGGGATACTGCCTGACATGACCCCTAGCACA

GAGATGTCACTGAGAGGAGTGAGAGTCAGCAAAATGGGAGTGGATGAATATTCCAGTACT

GAGAGAGTGGTCGTGAGCATTGATCGCTTCTTGAGAGTCCGAGATCAGAGGGGAAATGTG

CTTCTGTCTCCTGAGGAAGTTAGTGAAACACATGGAACAGAGAAACTGACGATAACGTAT

TCATCGTCTATGATGTGGGAAATCAATGGTCCGGAATCCGTGCTAGTCAACACATATCAA

TGGATCATTAGAAATTGGGAAACTGTGAAGATTCAGTGGTCCCAGGACCCTACGATGTTG

TACAATAAGATGGAATTTGAGCCCTTCCAATCCTTGGTGCCCAAGGCTGCTAGAGGCCAG

TATAGTGGGTTTGTGAGGACATTATTCCAACAGATGCGTGATGTGTTGGGGACATTTGAC

ACTGTCCAAATAATAAAGCTCCTACCATTTGCAGCAGCCCCACCGGAACAGAGTAGGATG

CAATTTTCCTCTCTGACTGTGAACGTAAGAGGTTCAGGAATGAGAATACTTGTGAGGGGC

AACTCCCCTGTGTTCAACTATAATAAGGCCACCAAGAGACTCACAGTTCTTGGAAAGGAT

GCAGGCGCCTTGACAGAATATCCAGATGAGGGAACAGCAGGAGTGGAGTCTGCAGTATTA

AGAGGATTTCTAATTCTGGGCAAAGAGGACAAAAGATATGGACCAGCATTGAGCATCAAC

GAATTGAGCAATCTTGCGAAAGGGGAAAAGGCTAATGTGTTGATAGGACAAGGAGACGTG

GTGTTGGTAATGAAACGGAAACGGGACTCTAGCATACTTACTGACAGCCAGACAGCGACC

AAAAGAATTCGGATGGCCATCAATTAGTGTCGAATTGTTTAAAAACGACCTTGTTTCTAC

T---------------------

>A_chicken_Omsk_0112_2020_EPI1813342

AGCAAAAGCAGGTCAAATATATTCAATATGGAGAGAATAAAAGAACTAAGAGATTTGATG

TCGCAGTCTCGCACTCGCGAGATACTAACAAAAACCACTGTGGACCATATGGCCATAATA

AAGAAATACACATCAGGAAGACAGGAGAAGAACCCTGCCCTCAGGATGAAATGGATGATG

GCAATGAAATATCCTATTACAGCTGACAAAAGAATAATGGAGATGATCCCTGAAAGGAAT

GAGCAAGGTCAGACTCTCTGGAGCAAAACAAATGATGCTGGATCAGACAGAGTGATGGTC

TCACCTCTGGCTGTGACATGGTGGAATAGAAATGGGCCAACAACAAGTACAGTACACTAC

CCAAAAGTCTACAAAACCTACTTTGAAAAGGTAGAAAGGTTGAAACATGGAACCTTTGGT

CCTGTTCACTTTCGAAATCAGGTTAAGATACGCCGCAGGGTTGACATAAACCCGGGCCAT

GCAGATCTCAGTGCCAAAGAAGCACAGGATGTCATCATGGAGGTTGTTTTCCCAAATGAA

GTTGGAGCCAGGATCTTGACATCAGAATCACAATTAACAATAACAAGGGAAAAGAAGGAG

GAACTTCAGGATTGCAAGATTGCTCCTTTGATGGTGGCATACATGTTGGAAAGAGAACTG

GTTCGCAAGACCAGATTTTTACCAGTAGCTGGCGGAACAAGCAGCGTATACATCGAGGTA

TTGCATTTGACTCAAGGGACCTGCTGGGAACAAATGTACACACCAGGAGGGGAGGTGAGA

AATGATGATGTTGATCAGAGTTTGATCATTGCTGCTAGAAATATAGTTAGGAGGGCAACA

GTATCAGCAGACCCATTGGCTTCGCTCTTGGAAATGTGCCACAGTACACAAATTGGTGGA

GTAAGGATGGTGGACATTCTTAGGCAGAACCCAACAGAGGAGCAAGCTGTGGATATATGC

AAAGCAGCAATGGGTTTAAGAATCAGTTCATCCTTCAGCTTTGGAGGTTTCACTTTCAAA

AGGACAAGTGGGTCGTCTGTCAAAAGAGAAGAAGAAATACTCACTGGCAACCTCCAAACA

CTGAAAGTAAGAATACATGAAGGATATGAGGAATTCACAATGGTTGGGCGAAGAGCTACA

GCCATTTTGAGGAAAGCAACCAGGAGACTGATCCAATTAATAGTGAGTGGAAGAGACGAG

CAGTCAATCGCTGAAGCAATCATAGTGGCAATGGTTTTCTCACAGGAGGATTGCATGATA

AAAGCAGTACGAGGTGATTTGAATTTTGTCAACAGAGCGAATCAGCGGCTAAATCCTATG

CATCAACTTCTGAGGCATTTCCAAAAGGATGCAAAAGTGCTGTTTCAAAACTGGGGGATT

GAACCAATTGACAATGTAATGGGGATGATCGGGATACTGCCTGACATGACCCCCAGCACA

GAGATGTCACTGAGAGGAGTGAGAGTCAGCAAAATGGGAGTGGATGAATATTCCAGTACT

GAGAGAGTGGTCGTGAGCATTGATCGCTTCTTGAGAGTCCGAGATCAGAGGGGAAATGTG

CTTCTGTCTCCTGAGGAAGTTAGTGAAACACATGGAACAGAGAAACTGACGATAACGTAT

TCATCGTCTATGATGTGGGAAATCAATGGTCCGGAATCCGTGCTAGTCAACACATATCAA

TGGATCATTAGAAGTTGGGAAACTGTGAAGATTCAATGGTCCCAGGACCCTACGATGTTG

TACAATAAGATGGAATTTGAGCCCTTCCAATCCTTGGTGCCCAAGGCTGCTAGAGGCCAG

TATAGTGGGTTTGTGAGGACATTATTCCAACAGATGCGTGATGTGTTGGGGACATTTGAC

ACTGTCCAAATAATAAAGCTCCTACCATTTGCAGCAGCCCCACCGGAACAGAGTAGGATG

CAATTTTCCTCTCTGACTGTGAACGTAAGAGGTTCAGGAATGAGAATACTTGTGAGGGGC

AACTCCCCTGTGTTCAACTATAATAAGGCCACCAAGAGACTCACAGTTCTTGGAAAGGAT

GCAGGCGCCTTGACAGAATATCCAGATGAGGGAACAGCAGGAGTGGAGTCTGCAGTATTA

AGAGGATTTCTAATTCTGGGCAAAGAGGACAAAAGATATGGACCAGCATTGAGCATCAAC

GAATTGAGCAATCTTGCGAAAGGGGAAAAGGCTAATGTGTTGATAGGACAAGGAGACGTG

GTGTTGGTAATGAAACGGAAACGGGACTCTAGCATACTTACTGACAGCCAGACAGCGACC

AAAAGAATTCGGATGGCCATCAATTAGTGTCGAATTGTTTAAAAACGACCTTGTTTCTAC

T---------------------

>A_duck_Saratov_29804_2020_EPI1814262

AGCAAAAGCAGGTCAAATATATTCAATATGGAGAGAATAAAAGAACTAAGAGATTTGATG

TCGCAGTCTCGCACTCGCGAGATACTAACAAAAACCACTGTGGACCATATGGCCATAATA

AAGAAATACACATCAGGGAGACAGGAGAAGAACCCTGCCCTCAGGATGAAATGGATGATG

GCAATGAAATATCCTATTACAGCTGACAAAAGAATAATGGAGATGATCCCTGAAAGGAAT

GAGCAAGGTCAGACTCTCTGGAGCAAAACAAATGATGCTGGATCAGACAGAGTGATGGTC

TCACCTCTGGCTGTGACATGGTGGAATAGAAATGGGCCAACAACAAGTACAGTACACTAC

CCAAAAGTCTACAAAACCTACTTTGAAAAGGTAGAAAGGTTGAAACATGGAACCTTTGGT

CCTGTTCATTTTCGAAATCAGGTTAAGATACGCCGCAGGGTTGACATAAACCCGGGCCAT

GCAGATCTCAGTGCCAAAGAAGCACAGGATGTCATCATGGAGGTTGTTTTCCCAAATGAA

GTTGGAGCCAGGATCTTGACATCAGAATCACAATTAACAATAACAAGGGAAAAGAAGGAG

GAACTTCAGAATTGCAAGATTGCTCCTTTGATGGTGGCATACATGTTGGAAAGAGAACTG

GTTCGCAAGACCAGATTTTTACCAGTAGCTGGCGGAACAAGTAGCGTATACATCGAGGTA

TTGCATTTGACTCAAGGGACCTGCTGGGAACAAATGTACACACCAGGAGGGGAGGTGAGA

AATGATGATGTTGATCAGAGTTTGATCATTGCTGCTAGAAATATAGTTAGGAGGGCAACA

GTATCAGCAGACCCATTGGCTTCGCTCTTGGAAATGTGCCACAGTACACAAATTGGTGGA

GTGAGGATGGTGGACATTCTTAGGCAGAACCCAACAGAGGAGCAAGCTGTGGATATATGC

AAAGCAGCAATGGGTTTAAGAATCAGTTCATCCTTCAGCTTTGGAGGTTTCACTTTCAAA

AGGACAAGTGGGTCGTCTGTCAAAAGAGAAGAAGAAATACTCACTGGCAACCTCCAAACA

CTGAAAGTAAGAATACATGAAGGATATGAGGAATTCACAATGGTTGGGCGAAGAGCTACA

GCCATTTTGAGGAAAGCAACCAGGAGACTGATCCAATTAATAGTGAGTGGACGAGACGAG

CAGTCAATCGCTGAAGCAATCATAGTGGCAATGGTTTTCTCACAGGAGGATTGCATGATA

AAAGCAGTACGAGGTGATTTGAATTTTGTCAACAGAGCGAACCAGCGGCTAAATCCTATG

CATCAACTTCTGAGGCATTTCCAAAAGGATGCAAAAGTGCTGTTTCAAAACTGGGGGATT

GAACCAATTGACAATGTAATGGGGATGATCGGGATACTGCCTGACATGACCCCCAGCACA

GAGATGTCACTGAGAGGAGTGAGAGTCAGCAAAATGGGAGTGGATGAATATTCCAGTACT

GAGAGAGTGGTCGTGAGCATTGATCGCTTCTTGAGAGTCCGAGATCAGAGGGGAAATGTG

CTTCTGTCTCCTGAGGAAGTTAGTGAAACACATGGAACAGAGAAACTGACGATAACGTAT

TCATCGTCTATGATGTGGGAAATCAATGGTCCGGAATCCGTGCTAGTCAACACATATCAA

TGGATCATTAGAAATTGGGAAACTGTGAAGATTCAGTGGTCCCAGGACCCTACGATGTTG

TACAATAAGATGGAATTTGAGCCCTTCCAATCCTTGGTGCCCAAGGCTGCTAGAGGCCAG

TATAGTGGGTTTGTGAGGACATTATTCCAACAGATGCGTGATGTGCTGGGGACATTTGAC

ACTGTCCAAATAATAAAGCTCCTACCATTTGCAGCAGCCCCACCGGAACAGAGTAGGATG

CAATTTTCCTCTCTGACTGTGAACGTAAGAGGTTCAGGAATGAGAATACTTGTGAGGGGC

AACTCCCCTGTGTTCAACTATAATAAGGCCACCAAGAGACTCACAGTTCTTGGAAAGGAT

GCAGGCGCCTTGACAGAATATCCAGATGAGGGAACAGCAGGAGTGGAGTCTGCAGTATTA

AGAGGATTTCTAATTCTGGGCAAAGAGGACAAAAGATATGGACCAGCATTGAGCATCAAC

GAATTGAGCAATCTTGCGAAAGGGGAAAAAGCTAATGTGTTGATAGGACAAGGAGACGTG

GTGTTGGTAATGAAACGGAAACGGGACTCTAGCATACTTACTGACAGCCAGACAGCGACC

AAAAGAATTCGGATGGCCATCAATTAGTGTCGAATTGTTTAAAAACGACCTTGTTTCTAC

T---------------------

>A_goose_Omsk_30001_2020_EPI1814278

AGCAAAAGCAGGTCAAATATATTCAATATGGAGAGAATAAAAGAACTAAGAGATTTGATG

TCGCAGTCTCGCACTCGCGAGATACTAACAAAAACCACTGTGGACCATATGGCCATAATA

AAGAAATACACATCAGGAAGACAGGAGAAGAACCCTGCCCTCAGGATGAAATGGATGATG

GCAATGAAATATCCTATTACAGCTGACAAAAGAGTAATGGAGATGATCCCTGAAAGGAAT

GAGCAAGGTCAGACTCTCTGGAGCAAAACAAATGATGCTGGATCAGACAGAGTGATGGTC

TCACCTCTAGCTGTGACATGGTGGAATAGAAATGGGCCAACAACAAGTACAGTACACTAC

CCAAAAGTCTACAAAACCTACTTTGAAAAGGTAGAAAGGTTGAAACATGGAACCTTTGGT

CCTGTTCACTTTCGAAATCAGGTTAAGATACGCCGCAGGGTTGACATAAACCCGGGCCAT

GCAGATCTCAGTGCCAAAGAAGCACAGGATGTCATCATGGAGGTTGTTTTCCCAAATGAA

GTTGGAGCCAGGATCTTGACATCAGAATCACAATTAACAATAACAAGGGAAAAGAAGGAG

GAACTTCAGGATTGCAAGATTGCTCCTTTGATGGTGGCATACATGTTGGAAAGAGAACTG

GTTCGCAAGACCAGATTTTTACCAGTAGCTGGCGGAACAAGCAGCGTATACATCGAGGTA

TTGCATTTGACTCAAGGGACCTGCTGGGAACAAATGTACACACCAGGAGGGGAGGTGAGA

AATGATGATGTTGATCAGAGTTTGATCATTGCTGCTAGAAATATAGTTAGGAGGGCAACA

GTATCAGCAGACCCATTGGCTTCGCTCTTGGAAATGTGCCACAGTACACAAATTGGTGGA

GTAAGGATGGTGGACATTCTTAGGCAGAACCCAACAGAGGAGCAAGCTGTGGATATATGC

AAAGCAGCAATGGGTTTAAGAATCAGTTCATCCTTCAGCTTTGGAGGTTTCACTTTCAAA

AGGACAAGTGGGTCGTCTGTCAAAAGAGAAGAAGAAATACTCACTGGCAACCTCCAAACA

CTGAAAGTAAGAATACATGAAGGATATGAGGAATTCACAATGGTTGGGCGAAGAGCTACA

GCCATTTTGAGGAAAGCAACCAGGAGACTGATCCAATTAATAGTGAGTGGAAGAGACGAG

CAGTCAATCGCTGAAGCAATCATAGTGGCAATGGTTTTCTCACAGGAGGATTGCATGATA

AAAGCAGTACGAGGTGATTTGAATTTTGTCAACAGAGCGAATCAGCGGCTAAATCCTATG

CATCAACTTCTGAGGCATTTCCAAAAGGATGCAAAAGTGCTGTTTCAAAACTGGGGGATT

GAACCAATTGACAATGTAATGGGGATGATCGGGATACTGCCTGACATGACCCCCAGCACA

GAGATGTCACTGAGAGGAGTGAGAGTCAGCAAAATGGGAGTGGATGAATATTCCAGTACT

GAGAGAGTGGTCGTGAGCATTGATCGCTTCTTGAGAGTCCGAGATCAGAGGGGAAATGTG

CTTCTGTCTCCTGAGGAAGTTAGTGAAACACATGGAACAGAGAAACTGACGATAACGTAT

TCATCGTCTATGATGTGGGAAATCAATGGTCCGGAATCCGTGCTAGTCAACACATATCAA

TGGATCATTAGAAATTGGGAAACTGTGAAGATTCAGTGGTCCCAGGACCCTACGATGTTG

TACAATAAGATGGAATTTGAGCCCTTCCAATCCTTGGTGCCCAAGGCTGCTAGAGGCCAG

TATAGTGGGTTTGTGAGGACATTATTCCAACAGATGCGTGATGTGTTGGGGACATTTGAC

ACTGTCCAAATAATAAAGCTCCTACCATTTGCAGCAGCCCCACCTGAACAGAGTAGGATG

CAATTTTCCTCTCTGACTGTGAACGTAAGAGGTTCAGGAATGAGAATACTTGTGAGGGGC

AACTCCCCTGTGTTCAACTATAATAAGGCCACCAAGAGACTCACAGTTCTTGGAAAGGAT

GCAGGCGCCTTGACAGAATATCCAGATGAGGGAACAGCAGGAGTGGAGTCTGCAGTATTA

AGAGGATTTCTAATTCTGGGCAAAGAGGACAAAAGATATGGACCAGCATTGAGCATCAAC

GAATTGAGCAATCTTGCGAAAGGGGAAAAGGCTAATGTGTTGATAGGACAAGGAGACGTG

GTGTTGGTAATGAAACGGAAACGGGACTCTAGCATACTTACTGACAGCCAGACAGCGACC

AAAAGAATTCGGATGGCCATCAATTAGTGTCGAATTGTTTAAAAACGACCTTGTTTCTAC

T---------------------

>A_goose_Omsk_30003_2020_EPI1814286

AGCAAAAGCAGGTCAAATATATTCAATATGGAGAGAATAAAAGAACTAAGAGATTTGATG

TCGCAGTCTCGCACTCGCGAGATACTAACAAAAACCACTGTGGACCATATGGCCATAATA

AAGAAATACACATCAGGGAGACAGGAGAAGAACCCTGCCCTCAGGATGAAATGGATGATG

GCAATGAAATATCCTATTACAGCTGACAAAAGAATAATGGAGATGATCCCTGAAAGGAAT

GAGCAAGGTCAGACTCTCTGGAGCAAAACAAATGATGCTGGATCAGACAGAGTGATGGTC

TCACCTCTGGCTGTGACATGGTGGAATAGAAATGGGCCAACAACAAGTACAGTACACTAC

CCAAAAGTCTACAAAACCTACTTTGAAAAGGTAGAAAGGTTGAAACATGGAACCTTTGGT

CCTGTTCACTTTCGAAATCAGGTTAAGATACGCCGCAGGGTTGACATAAACCCGGGCCAT

GCAGATCTCAGTGCCAAAGAAGCACAGGATGTCATCATGGAGGTTGTTTTCCCAAATGAA

GTTGGAGCCAGGATCTTGACATCAGAATCACAATTAACAATAACAAGGGAAAAGAAGGAG

GAACTTCAGGATTGCAAGATTGCTCCTTTGATGGTGGCATACATGTTGGAAAGAGAACTG

GTTCGCAAGACCAGATTTTTACCAGTAGCTGGCGGAACAAGCAGCGTATACATCGAGGTA

TTGCATTTGACTCAAGGGACCTGCTGGGAACAAATGTACACACCAGGAGGGGAGGTGAGA

AATGATGATGTTGATCAGAGTTTGATCATTGCTGCTAGAAATATAGTTAGGAGGGCAACA

GTATCAGCAGACCCATTGGCTTCGCTCTTGGAAATGTGCCACAGTACACAAATTGGTGGA

GTGAGGATGGTGGACATTCTTAGGCAGAACCCAACAGAGGAGCAAGCTGTGGATATATGC

AAAGCAGCAATGGGTTTAAGAATCAGTTCATCCTTCAGCTTTGGAGGTTTCACTTTCAAA

AGGACAAGTGGGTCGTCTGTCAAAAGAGAAGAAGAAATACTCACTGGCAACCTCCAAACA

CTGAAAGTAAGAATACATGAAGGATATGAGGAATTCACAATGGTTGGGCGAAGAGCTACA

GCCATTTTGAGGAAAGCAACCAGGAGACTGATCCAATTAATAGTGAGTGGACGAGACGAG

CAGTCAATCGCTGAAGCAATCATAGTGGCAATGGTTTTCTCACAGGAGGATTGCATGATA

AAAGCAGTACGAGGTGATTTGAATTTTGTCAACAGAGCGAATCAGCGGCTAAATCCTATG

CATCAACTTCTGAGGCATTTCCAAAAGGATGCAAAAGTGCTGTTTCAAAACTGGGGGATT

GAACCAATTGACAATGTAATGGGGATGATCGGGATACTGCCTGACATGACCCCCAGCACA

GAGATGTCACTGAGAGGAGTGAGAGTCAGCAAAATGGGAGTGGATGAATATTCCAGTACT

GAGAGAGTGGTCGTGAGCATTGATCGCTTCTTGAGAGTCCGAGATCAGAGGGGAAATGTG

CTTCTGTCTCCTGAGGAAGTTAATGAAACACATGGAACAGAGAAACTGACGATAACGTAT

TCATCGTCTATGATGTGGGAAATCAATGGTCCGGAATCCGTGCTAGTCAACACATATCAA

TGGATCATTAGAAATTGGGAAACTGTGAAGATTCAGTGGTCCCAGGACCCTACGATGTTG

TACAATAAGATGGAATTTGAGCCCTTCCAATCCTTGGTGCCCAAGGCTGCTAGAGGCCAG

TATAGTGGGTTTGTGAGGACATTATTCCAACAGATGCGTGATGTGTTGGGGACATTTGAC

ACTGTCCAAATAATAAAGCTCCTACCATTTGCAGCAGCCCCACCGGAACAGAGTAGGATG

CAATTTTCCTCTCTGACTGTGAACGTAAGAGGTTCAGGAATGAGAATACTTGTGAGGGGC

AACTCCCCTGTGTTCAACTATAATAAGGCCACCAAGAGACTCACAGTTCTTGGAAAGGAT

GCAGGCGCCTTGACAGAATATCCAGATGAGGGAACAGCAGGAGTGGAGTCTGCAGTATTA

AGAGGATTTCTAATTCTGGGCAAAGAGGACAAAAGATATGGACCAGCATTGAGCATCAAC

GAATTGAGCAATCTTGCGAAAGGGGAAAAGGCTAATGTGTTGATAGGACAAGGAGACGTG

GTGTTGGTAATGAAACGGAAACGGGACTCTAGCATACTTACTGACAGCCAGACAGCGACC

AAAAGAATTCGGATGGCCATCAATTAGTGTCGAATTGTTTAAAAACGACCTTGTTTCTAC

T---------------------

>A_swan_Tumen_1479-2_2020_EPI1814681

------------TCAAATATATTCAATATGGAGAGAATAAAAGAACTAAGAGATTTGATG

TCGCAGTCTCGCACTCGCGAGATACTAACAAAAACCACTGTGGACCATATGGCCATAATA

AAGAAATACACATCAGGGAGACAGGAGAAGAACCCTGCCCTCAGGATGAAATGGATGATG

GCAATGAAATATCCTATTACAGCTGACAAAAGAATAATGGAGATGATCCCTGAAAGGAAT

GAGCAAGGTCAGACTCTCTGGAGCAAAACAAATGATGCTGGATCAGACAGAGTGATGGTC

TCACCTCTGGCTGTGACATGGTGGAATAGAAATGGGCCAACAACAAGTACAGTACACTAC

CCAAAAGTCTACAAAACCTACTTTGAAAAGGTAGAAAGGTTGAAACATGGAACCTTTGGT

CCTGTTCACTTTCGAAATCAGGTTAAGATACGCCGCAGGGTTGACATAAACCCGGGCCAT

GCAGATCTCAGTGCCAAAGAAGCACAGGATGTCATCATGGAGGTTGTTTTCCCAAATGAA

GTTGGAGCCAGGATCTTGACATCAGAATCACAATTAACAATAACAAGGGAAAAGAAGGAG

GAACTTCAGGATTGCAAGATTGCTCCTTTGATGGTGGCATACATGTTGGAAAGAGAACTG

GTTCGCAAGACCAGATTTTTACCAGTAGCTGGCGGAACAAGCAGCGTATACATCGAGGTA

TTGCATTTGACTCAAGGGACCTGCTGGGAACAAATGTACACACCAGGAGGGGAGGTGAGA

AATGATGATGTTGATCAGAGTTTGATCATTGCTGCTAGAAATATAGTTAGGAGGGCAACA

GTATCAGCAGACCCATTGGCTTCGCTCTTGGAAATGTGCCACAGTACACAAATTGGTGGA

GTGAGGATGGTGGACATTCTTAGGCAGAACCCAACAGAGGAGCAAGCTGTGGATATATGC

AAAGCAGCAATGGGTTTAAGAATCAGTTCATCCTTCAGCTTTGGAGGTTTCACTTTCAAA

AGGACAAGTGGGTCGTCTGTCAAAAGAGAAGAAGAAATACTCACTGGCAACCTCCAAACA

CTGAAAGTAAGAATACATGAAGGATATGAGGAATTCACAATGGTTGGGCGAAGAGCTACA

GCCATTTTGAGGAAAGCAACCAGGAGACTGATCCAATTAATAGTGAGTGGACGAGACGAG

CAGTCAATCGCTGAAGCAATCATAGTGGCAATGGTTTTCTCACAGGAGGATTGCATGATA

AAAGCAGTACGAGGTGATTTGAATTTTGTCAACAGAGCGAATCAGCGGCTAAATCCTATG

CATCAACTTCTGAGGCATTTCCAAAAGGATGCAAAAGTGCTGTTTCAAAACTGGGGGATT

GAACCAATTGACAATGTAATGGGGATGATCGGGATACTGCCTGACATGACCCCCAGCACA

GAGATGTCACTGAGAGGAGTGAGAATCAGCAAAATGGGAGTGGATGAATATTCCAGTACT

GAGAGAGTGGTCGTGAGCATTGATCGCTTCTTGAGAGTCCGAGATCAGAGGGGAAATGTG

CTTCTGTCTCCTGAGGAAGTTAGTGAAACACATGGAACAGAGAAACTGACGATAACGTAC

TCATCGTCTATGATGTGGGAAATCAATGGTCCGGAATCCGTGCTAGTCAACACATATCAA

TGGATCATTAGAAATTGGGAAACTGTGAAGATTCAGTGGTCCCAGGACCCTACGATGTTG

TACAATAAGATGGAATTTGAGCCCTTCCAATCCTTGGTGCCCAAGGCTGCTAGAGGCCAG

TATAGTGGGTTTGTGAGGACATTATTCCAACAGATGCGTGATGTGTTGGGGACATTTGAC

ACTGTCCAAATAATAAAGCTCCTACCATTTGCAGCAGCCCCACCGGAACAGAGTAGGATG

CAATTTTCCTCCCTGACTGTGAACGTAAGAGGTTCAGGAATGAGAATACTTGTGAGGGGC

AACTCCCCTGTGTTCAACTATAATAAGGCCACCAAGAGACTCACAGTTCTTGGAAAGGAT

GCAGGCGCCTTGACAGAATATCCAGATGAGGGAACAGCAGGAGTGGAGTCTGCAGTATTA

AGAGGATTTCTAATTCTGGGCAAAGAGGACAAAAGATATGGACCAGCATTGAGCATCAAC

GAATTGAGCAATCTTGCGAAAGGGGAAAAGGCTAATGTGTTGATAGGACAAGGAGACGTG

GTGTTGGTAATGAAACGGAAACGGGACTCTAGCATACTTACTGACAGCCAGACAGCGACC

AAAAGAATTCGGATGGCCATCAATTAGTGTCGAATTGTTTAAAAACGA------------

----------------------

>A_Whooper_swan_Mongolia_24_2020_EPI1831871

AGCGAAAGCAGGTCAAATATATTCAGTATGAACAGAATAAAAGAACTAAGAGATCTAATG

TCACAGTCTCGCACCCGCGAGATACTGACAAAGACCACTGTGGACCATATGGCCATAATC

AAAAAATACACATCAGGAAGACAGGAAAAGAATCCCGCTCTCAGGATGAAATGGATGATG

GCAATGAAATACCCGATCACAGCTGACAAAAAGATAATGGAGATGATTCCTGAACGAAAT

GAACAAGGTCAAACTCTTTGGAGCAAGACAAATGATGCCGGGTCAGACAGAGTAATGGTA

TCACCTCTGGCTGTGACTTGGTGGAACAGGAATGGACCAACAACAAGCACAGTCCACTAT

CCCAAGGTGTACAAAACCTACTTTGAGAAGGTTGAAAGATTAAAACACGGAACCTTTGGG

CCCGTTCATTTCCGAAGTCAAGTCAAAATACGCCGCAGGGTTGACATAAATCCAGGCCAT

GCGGATCTCAGTGCGAAAGAAGCACAAGATGTTATAATGGAGGTTGTTTTCCCAAATGAA

GTTGGAGCGAGGATATTGACTTCAGAGTCACAAATGACAATAACAAAGGAAAAGAAAGAA

GAACTCCAGGATTGCAAGATTGCTCCATTGATGGTGGCATATATGTTGGAAAGAGAACTG

GTTCGCAAGACAAGATTCCTACCAGTGGCTGGCGGGACAAGCAGCGTGTATATAGAAGTG

TTACATCTGACTCAAGGAACTTGCTGGGAACAAATGTATACACCAGGAGGAGAAGTGAGA

AATGATGACATTGACCAGAGTCTAATCGTTGCTGCTAGAAACATTGTGAGAAGAGCAACA

GTGTCGGCAGACCCATTAGCATCACTCTTGGAGATGTGCCACAGTACACAAATTGGCGGG

ATAAGAATGGTGGACATCCTTAGGCAAAATCCAACAGAAGAGCAAGCTGTAGACATATGC

AAGGCAGCAATGGGTCTGAGAATTAGCTCATCCTTCAGTTTTGGAGGCTTCACTTTCAAA

AGAACAAGTGGTTCTTCTATTAAAAGAGAGGAAGAAGTGCTTACAGGCAACCTCCAAACA

TTGAAAATAAGAGTGCATGAAGGATATGAAGAATTCACAATGGTTGGGCGAAGAGCAACA

GCAATTCTGAGGAAAGCAACCAGGAGGCTGATTCAATTGATAGTAAGTGGGAGAGATGAA

CAATCAATTGCTGAAGCAATCATTGTAGCAATGGTATTCTCACAAGAGGACTGCATGATA

AAGGCAGTCCGGGGTGATTTGAACTTTGTGAACAGAGCAAACCAACGGCTTAACCCCATG

CACCAACTCCTGAGGCACTTCCAAAAGGACGCAAAGGTACTATTCCAGAACTGGGGACTT

GAGACCATTGACAATGTAATGGGAATGGTTGGAATATTGCCTGATATGACTCCCAGTACG

GAAATGTCATTAAGGGGAGTGAGAGTCAGTAAAATGGGAGTAGACGAATATTCTAACACT

GAAAGAGTGGTCGTGAGCATTGATCGTTTCCTGAGAGTACGGGATCAACAAGGGAACATA

CTCTTATCCCCTGAAGAAGTTAGTGAAACACAGGGAACGGAAAAGTTAACAATAACATAC

TCTTCATCTATGATGTGGGAGATTAACGGCCCAGAATCAGTGCTGGTCAACACATACCAA

TGGATCATTAGGAATTGGGAGAATGTGAAGATCCAATGGTCCCAAGACCCTACTACGTTA

TACAATAAGATGGAATTCGAGCCCTTTCAATCTTTGGTACCTAAAGCTGTTAGAGGTCAA

TACAGTGGGTTCGTGAGGACACTATTCCAGCAAATGCGTGATGTGTTGGGAACATTTGAC

ACTGTTCAAATAATAAAGCTTCTACCATTTGCAGCAGCACCACCAGAGCAAAGCAGAATG

CAATTTTCTTCTCTGACGGTGAATGTACGAGGATCTGGGATGAGAATACTTGTAAGAGGC

AACTCCCCTGTGTTTAACTATAACAAATCAACTAAGAGGCTCACAGTTCTCGGGAAAGAT

GCGGGTGCACTGACAGAAGATCCGAATGAGGGAACAGCAGGAGTAGAGTCTGCAGTACTG

AGAGGATTTCTAATTCTAGGCAGAGAAGACAAGAGATATGGACCAGCATTAAGCATTAAC

GAGTTGAGCAATCTTGCTAAAGGGGAGAAGGCTAATGTGTTGATAGGGCAAGGAGATGCG

GTGTTGGTAATGAAACGGAAACGGGACTCTAGCATACTTACTGACAGCCAGACAGCGACC

AAAAGAATTCGTATGGCCATCAATTAGTGTTGAATTGTTTAAAAACGACCTTGTTTCTAC

T---------------------

>A_Whooper_swan_Mongolia_25_2020_EPI1831879

AGCGAAAGCAGGTCAAATATATTCAGTATGAACAGAATAAAAGAACTAAGAGATCTAATG

TCACAGTCTCGCACCCGCGAGATACTGACAAAGACCACTGTGGACCATATGGCCATAATC

AAAAAATACACATCAGGAAGACAGGAAAAGAATCCCGCTCTCAGGATGAAATGGATGATG

GCAATGAAATACCCGATCACAGCTGACAAAAAGATAATGGAGATGATTCCTGAACGAAAT

GAACAAGGTCAAACTCTTTGGAGCAAGACAAATGATGCCGGGTCAGACAGAGTAATGGTA

TCACCTCTGGCTGTGACTTGGTGGAACAGGAATGGACCAACAACAAGCACAGTCCACTAT

CCCAAGGTGTACAAAACCTACTTTGAGAAGGTTGAAAGATTAAAACACGGAACCTTTGGG

CCCGTTCATTTCCGAAGTCAAGTCAAAATACGCCGCAGGGTTGACATAAATCCAGGCCAT

GCGGATCTCAGTGCGAAAGAAGCACAAGATGTTATAATGGAGGTTGTTTTCCCAAATGAA

GTTGGAGCGAGGATATTGACTTCAGAGTCACAAATGACAATAACAAAGGAAAAGAAAGAA

GAACTCCAGGATTGCAAGATTGCTCCATTGATGGTGGCATATATGTTGGAAAGAGAACTG

GTTCGCAAGACAAGATTCCTACCAGTGGCTGGCGGGACAAGCAGCGTGTATATAGAAGTG

TTACATCTGACTCAAGGAACTTGCTGGGAACAAATGTATACACCAGGAGGAGAAGTGAGA

AATGATGACATTGACCAGAGTCTAATCGTTGCTGCTAGAAACATTGTGAGAAGAGCAACA

GTGTCGGCAGACCCATTAGCATCACTCTTGGAGATGTGCCACAGTACACAAATTGGCGGG

ATAAGAATGGTGGACATCCTTAGGCAAAATCCAACAGAAGAGCAAGCTGTAGACATATGC

AAGGCAGCAATGGGTCTGAGAATTAGCTCATCCTTCAGTTTTGGAGGTTTCACTTTCAAA

AGAACAAGTGGTTCTTCTATTAAAAGAGAGGAAGAAGTGCTTACAGGCAACCTCCAAACA

TTGAAAATAAGAGTGCATGAAGGATATGAAGAATTCACAATGGTTGGGCGAAGAGCAACA

GCAATTCTGAGGAAAGCAACCAGGAGGCTGATTCAATTGATAGTAAGTGGGAGAGATGAA

CAATCAATTGCTGAAGCAATCATTGTAGCAATGGTATTCTCACAAGAGGACTGCATGATA

AAGGCAGTCCGGGGTGATTTGAACTTTGTGAACAGAGCAAACCAACGGCTTAACCCCATG

CACCAACTCCTGAGGCACTTCCAAAAGGACGCAAAGGTACTATTCCAGAACTGGGGACTT

GAGACCATTGACAATGTAATGGGAATGGTTGGAATATTGCCTGATATGACTCCCAGTACG

GAAATGTCATTAAGGGGAGTGAGAGTCAGTAAAATGGGAGTAGACGAATATTCTAACACT

GAAAGAGTGGTCGTGAGCATTGATCGTTTCCTGAGAGTACGGGATCAACAAGGGAACGTA

CTCTTATCCCCTGAAGAAGTTAGTGAAACACAGGGAACGGAAAAGTTAACAATAACATAC

TCTTCATCTATGATGTGGGAGATTAACGGCCCAGAATCAGTGCTGGTCAACACATACCAA

TGGATCATTAGGAATTGGGAGAATGTGAAGATCCAATGGTCCCAAGACCCTACTACGTTA

TACAATAAGATGGAATTCGAGCCCTTTCAATCTTTGGTACCTAAAGCTGTTAGAGGTCAA

TACAGTGGGTTCGTGAGGACACTATTCCAGCAAATGCGTGATGTGTTGGGAACATTTGAC

ACTGTTCAAATAATAAAGCTTCTACCATTTGCAGCAGCACCACCAGAGCAAAGCAGAATG

CAATTTTCTTCTCTGACGGTGAATGTACGAGGATCTGGGATGAGAATACTTGTAAGAGGC

AACTCCCCTGTGTTTAACTATAACAAATCAACTAAGAGGCTCACAGTTCTCGGGAAAGAT

GCGGGTGCACTGACAGAAGATCCGAATGAGGGAACAGCAGGAGTAGAGTCTGCAGTACTG

AGAGGATTTCTAATTCTAGGCAGAGAAGACAAGAGATATGGACCAGCATTAAGCATTAAC

GAGTTGAGCAATCTTGCTAAAGGGGAGAAGGCTAATGTGTTGATAGGGCAAGGAGATGCG

GTGTTGGTAATGAAACGGAAACGGGACTCTAGCATACTTACTGACAGCCAGACAGCGACC

AAAAGAATTCGTATGGCCATCAATTAGTGTTGAATTGTTTAAAAACGACCTTGTTTCTAC

T---------------------

>A_chicken_Kazakhstan_Kn-3_2020_EPI1839258

---------------------------ATGGAGAGAATAAAAGAACTAAGAGATTTGATG

TCGCAGTCTCGCACTCGCGAGATACTAACAAAAACCACTGTGGACCATATGGCCATAATA

AAGAAATACACATCAGGGAGACAGGAGAAGAACCCTGCCCTCAGGATGAAATGGATGATG

GCAATGAAATATCCTATTACAGCTGACAAAAGAATAATGGAGATGATCCCTGAAAGGAAT

GAGCAAGGTCAGACTCTCTGGAGCAAAACAAATGATGCTGGATCAGACAGAGTGATGGTC

TCACCTCTGGCTGTGACATGGTGGAATAGAAATGGGCCGACAACAAGTACAGTACACTAC

CCAAAAGTCTACAAAACCTACTTTGAAAAGGTTGAAAGGTTGAAACATGGAACCTTTGGT

CCTGTTCACTTTCGAAATCAGGTTAAGATACGCCGCAGGGTTGACATAAACCCGGGCCAT

GCAGATCTCAGTGCCAAAGAAGCACAGGATGTCATCATGGAGGTTGTTTTCCCAAATGAA

GTTGGAGCCAGGATCTTGACATCAGAATCGCAATTAACAATAACAAGGGAAAAGAAGGAG

GAACTTCAGGATTGCAAGATTGCTCCTTTGATGGTGGCATACATGTTGGAAAGAGAACTG

GTTCGCAAGACCAGATTTTTACCAGTAGCTGGCGGAACAAGCAGCGTATACATCGAGGTA

TTGCATTTGACTCAAGGGACCTGCTGGGAACAAATGTACACACCAGGAGGGGAGGTGAGA

AATGATGATGTTGATCAGAGTTTGATCATTGCTGCTAGAAATATAGTTAGGAGGGCAACA

GTATCAGCAGACCCATTGGCTTCGCTCTTGGAAATGTGCCACAGTACACAAATTGGTGGA

GTGAGGATGGTGGACATTCTTAGGCAGAACCCAACAGAGGAGCAAGCTGTGGATATATGC

AAAGCAGCAATGGGTTTAAGAATCAGTTCATCCTTCAGCTTTGGAGGTTTCACTTTCAAA

AGGACAAGTGGGTCGTCTGTCAAAAGAGAAGAAGAAATACTCACTGGCAACCTCCAAACA

CTGAAAGTAAGAATACATGAAGGATATGAGGAATTCACAATGGTTGGGCGAAGAGCTACA

GCCATTTTGAGGAAAGCAACCAGGAGACTGATCCAATTAATAGTGAGTGGACGAGACGAG

CAGTCAATCGCTGAAGCAATCATAGTGGCAATGGTTTTCTCACAGGAGGATTGCATGATA

AAAGCAGTACGAGGTGATTTGAATTTTGTCAACAGAGCGAATCAGCGGCTAAATCCTATG

CATCAACTTCTGAGGCATTTCCAAAAGGATGCAAAAGTGCTGTTTCAAAACTGGGGGATT

GAACCAATTGACAATGTAATGGGGATGATCGGGATACTGCCTGACATGACCCCCAGCACA

GAGATGTCACTGAGAGGAGTGAGAGTCAGCAAAATGGGAGTGGATGAATATTCCAGTACT

GAGAGAGTGGTCGTGAGCATTGATCGCTTCTTGAGAGTCCGAGATCAGAGGGGAAATGTG

CTTCTATCTCCTGAGGAAGTTAGTGAAACACATGGAACAGAGAAACTGACGATAACGTAT

TCATCGTCTATGATGTGGGAAATCAATGGTCCGGAATCCGTGCTAGTCAACACATATCAA

TGGATCATTAGAAATTGGGAAACTGTGAAGATTCAGTGGTCCCAGGACCCTACGATGTTG

TACAATAAGATGGAATTTGAGCCCTTTCAATCCTTGGTGCCCAAGGCTGCTAGAGGCCAG

TATAGTGGGTTTGTGAGGACATTATTCCAACAGATGCGTGATGTGTTGGGGACATTTGAC

ACTGTCCAAATAATAAAGCTCCTACCATTTGCAGCAGCCCCACCGGAACAGAGTAGGATG

CAATTTTCCTCTCTGACTGTGAACGTAAGAGGTTCAGGAATGAGAATACTTGTGAGGGGC

AACTCCCCTGTGTTCAACTATAATAAGGCCACCAAGAGACTCACAGTTCTTGGAAAGGAT

GCAGGCGCCTTGACAGAATATCCAGATGAGGGAACAGCAGGAGTGGAGTCTGCAGTATTA

AGAGGATTTCTAATTCTGGGCAAAGAGGACAAAAGATATGGACCAGCATTGAGCATCAAC

GAATTGAGCAATCTTGCGAAAGGGGAAAAGGCTAATGTGTTGATAGGACAAGGAGACGTG

GTGTTGGTAATGAAACGGAAACGGGACTCTAGCATACTTACTGACAGCCAGACAGCGACC

AAAAGAATTCGGATGGCCATCAAT------------------------------------

----------------------

>A_chicken_Kazakhstan_Kn-6_2020_EPI1839266

---------------------------ATGGAAAGAATAAAAGAACTAAGAGATTTGATG

TCGCAGTCTCGCACTCGCGAGATACTAACAAAAACCACTGTGGACCATATGGCCATAATA

AAGAAATACACATCAGGGAGACAGGAGAAGAACCCTGCCCTCAGGATGAAATGGATGATG

GCAATGAAATATCCTATTACAGCTGACAAAAGAATAATGGAGATGATCCCTGAAAGGAAT

GAGCAAGGTCAGACTCTCTGGAGCAAAACAAATGATGCTGGATCAGACAGAGTGATGGTC

TCACCTCTGGCTGTGACATGGTGGAATAGAAATGGGCCGACAACAAGTACAGTACACTAC

CCAAAAGTCTACAAAACCTACTTTGAAAAGGTTGAAAGGTTGAAACATGGAACCTTTGGT

CCTGTTCACTTTCGAAATCAGGTTAAGATACGCCGCAGGGTTGACATAAACCCGGGCCAT

GCAGATCTCAGTGCCAAAGAAGCACAGGATGTCATCATGGAGGTTGTTTTCCCAAATGAA

GTTGGAGCCAGGATCTTGACATCAGAATCGCAATTAACAATAACAAGGGAAAAGAAGGAG

GAACTTCAGGATTGCAAGATTGCTCCTTTGATGGTGGCATACATGTTGGAAAGAGAACTG

GTTCGCAAGACCAGATTTTTACCAGTAGCTGGCGGAACAAGCAGCGTATACATCGAGGTA

TTGCATTTGACTCAAGGGACCTGCTGGGAACAAATGTACACACCAGGAGGGGAGGTGAGA

AATGATGATGTTGATCAGAGTTTGATCATTGCTGCTAGAAATATAGTTAGGAGGGCAACA

GTATCAGCAGACCCATTGGCTTCGCTCTTGGAAATGTGCCACAGTACACAAATTGGTGGA

GTGAGGATGGTGGACATTCTTAGGCAGAACCCAACAGAGGAGCAAGCTGTGGATATATGC

AAAGCAGCAATGGGTTTAAGAATCAGTTCATCCTTCAGCTTTGGAGGTTTCACTTTCAAA

AGGACAAGTGGGTCGTCTGTCAAAAGAGAAGAAGAAATACTCACTGGCAACCTCCAAACA

CTGAAAGTAAGAATACATGAAGGATATGAGGAATTCACAATGGTTGGGCGAAGAGCTACA

GCCATTTTGAGGAAAGCAACCAGGAGACTGATCCAATTAATAGTGAGTGGACGAGACGAG

CAGTCAATCGCTGAAGCAATCATAGTGGCAATGGTTTTCTCACAGGAGGATTGCATGATA

AAAGCAGTACGAGGTGATTTGAATTTTGTCAACAGAGCGAATCAGCGGCTAAATCCTATG

CATCAACTTCTGAGGCATTTCCAAAAGGATGCAAAAGTGCTGTTTCAAAACTGGGGGATT

GAACCAATTGACAATGTAATGGGGATGATCGGGATACTGCCTGACATGACCCCCAGCACA

GAGATGTCACTGAGAGGAGTGAGAGTCAGCAAAATGGGAGTGGATGAATATTCCAGTACT

GAGAGAGTGGTCGTGAGCATTGATCGCTTCTTGAGAGTCCGAGATCAGAGGGGAAATGTG

CTTCTGTCTCCTGAGGAAGTTAGTGAAACACATGGAACAGAGAAACTGACGATAACGTAT

TCATCGTCTATGATGTGGGAAATCAATGGTCCGGAATCCGTGCTAGTCAACACATATCAA

TGGATCATTAGAAATTGGGAAACTGTGAAGATTCAGTGGTCCCAGGACCCTACGATGTTG

TACAATAAGATGGAATTTGAGCCCTTTCAATCCTTGGTGCCCAAGGCTGCTAGAGGCCAG

TATAGTGGGTTTGTGAGGACATTATTCCAACAGATGCGTGATGTGTTGGGGACATTTGAC

ACTGTCCAAATAATAAAGCTCCTACCATTTGCAGCAGCCCCACCGGAACAGAGTAGGATG

CAATTTTCCTCTCTGACTGTGAACGTAAGAGGTTCAGGAATGAGAATACTTGTGAGGGGC

AACTCCCCTGTGTTCAACTATAATAAGGCCACCAAGAGACTCACAGTTCTTGGAAAGGAT

GCAGGCGCCTTGACAGAATATCCAGATGAGGGAACAGCAGGAGTGGAGTCTGCAGTATTA

AGAGGATTTCTAATTCTGGGCAAAGAGGACAAAAGATATGGACCAGCATTGAGCATCAAC

GAATTGAGCAATCTTGCGAAAGGGGAAAAGGCTAATGTGTTGATAGGACAAGGAGACGTG

GTGTTGGTAATGAAACGGAAACGGGACTCTAGCATACTTACTGACAGCCAGACAGCGACC

AAAAGAATTCGGATGGCCATCAAT------------------------------------

----------------------

>A_Muscovy_duck_China_FJFZ21_H5N6_2020_EPI1841919

---------------------------ATGAACAGAATAAAAGAACTAAGAGATCTAATG

TCACAGTCTCGCACCCGCGAGATACTGACAAAGACCACTGTGGACCATATGGCCATAATC

AAAAAATACACATCAGGAAGACAGGAAAAGAATCCCGCTCTCAGGATGAAATGGATGATG

GCAATGAAATACCCGATCACAGCCGACAAAAAGATAATGGAGATGATTCCTGAACGAAAT

GAACAAGGTCAAACTCTTTGGAGCAAGACAAATGATGCCGGGTCAAACAGAGTAATGGTA

TCACCTCTGGCTGTGACTTGGTGGAACAGGAATGGGCCAACAACAACCACAGTCCATTAT

CCCAAGGTGTACAAAACCTACTTTGAAAAGGTTGAAAAATTAAAACACGAAACCTTTGGG

CCCGTTCATTTCAGGAGTCAAGTCAAAATACCCCCCAGGGTTGACATAAATCCAGGCCAT

GCGAATCTCATTGCAAAAAAACCACAAAATGTTATAATGGAGGTTGTTTTCCCAAACAAA

TTTGGACCAAGGATATTGACTTCAAATTCACAATTGACAATAACAAAGGAAAAAAAAAAA

AAACTCCAGGATTGTAAAATTGCTCCATTGATGGTGGCATATATGTTGAAAAGAGAACTG

GTTCGCAGAACAAAATTCCTACCATTGGCTGGCGGGACAACCACCGTGTATATAAAATTG

TTACATTTGACTCAAGGAACTTGCTGGGAGCAAATGTACACACCAGGAGGAGAAGTGAAA

AATGATGACATTGACCAGAGTTTAATCATTGCTGCTAGAAACATTGTGAGAAGAGCAACA

GTGTCAGCAGACCCATTAGCATCACTCTTGGAGATGTGCCACAGTACACAAATTGGCGGG

ATAAGAATGGTGGACGTTCTTAAGCAAAATCCAACAGAAGAGCAAGCTGTAGACATATGC

AAGGCAGCAATGGGTCTGAGAATTAGTTCATCCTTCAGTTTTGGAGGTTTCACCTTCAAA

AGAACAAGTGGTTCTTCTATTAAAAGAGAGGAAGAAGTGCTTACAGGCAACCTCCAAACA

TTGAAAATAAGAGTGCATGAAGGATATGAAGAATTCACAATGGTTGGGCGAAGAGCAACA

GCAATTCTGAGGAAAGCAACCAGGAGGCTGATTCAATTGATAGTAAGTGGGAGAGACGAA

CAATCAATTGCTGAAGCAATCATTGTAGCAATGGTATTCTCACAAGAGGACTGCATGATA

AAGGCAGTCCGAGGTGATTTGAACTTTGTGAACAGAGCGAACCAACGGCTGAACCCCATG

CATCAACTCCTGAGGCACTTCCAAAAGGACGCAAAGGTACTATTCCAGAACTGGGGGCTT

GAGCCCATCGACAATGTAATGGGGATGGTTGGAATATTGCCTGATATGACTCCCAGTACG

GAAATGTCATTAAGGGGAGTGAGAGTCAGCAAAATGGGAGTAGACGAATATTCTAACACT

GAAAGAGTGGTCGTGAGCATTGATCGTTTCCTGAGAGTACGGGATCAACAAGGGAACGTA

CTCTTATCCCCTGAAGAAGTTAGTGAAACACAGGGAACGGAAAAGTTAACAATAACATAT

TCTTCATCTATGATGTGGGAGATTAACGGCCCAGAATCAGTGCTGGTCAACACATACCAA

TGGATCATTAGGAATTGGGAGAATGTGAAGATCCAATGGTCCCAAGACCCTACTATGTTA

TACAATAAGATGGAGTTTGAGCCCTTTCAATCTTTGGTACCTAAAGCTTTTAGAGGTCAA

TACAGTGGGTTCGTGAGGACACTATTCCAGCAAATGCGTGATGTGTTGGGAACATTTGAC

ACTGTTCAAATAATAAAGCTGCTACCATTTGCAGCAGCACCACCAGAGCAAAGCAGAATG

CAATTTTCTTCCCTGACGGTGAATATACGAGGATCTGGAATGAGAATACTCGTAAGAGGC

AACTCCCCTGTGTTTAACTATAACAAATCAACTAAGAGGCTCACAGTCCTCGGGAAAGAT

GCGGGTGCACTGACAGAAGATCCGAATGAGGGAACAGCAGGAGTAGAATCTGCAGTACTG

AGAGGATTTCTAATTCTAGGCAGGGAAGACAAGAGATACGGACCAGCATTAAGCATTAAC

GAGTTGAGCAATCTTGCTAAAGGGGAGAAGGCTAATGTGTTGATAGGGCAAGGAGACGCG

GTGTTGGTAATGAAACGGAAACGAGACTCTAGCATACTTACTGACAGCCAGACAGCGACC

AAAAGAATTCGTTTGGCCATCAATTAG---------------------------------

----------------------

>A_mute_swan_Czech_Republic_1410-2_2021_EPI1843603

------------------------AATATGGAGAGAATAAAAGAACTAAGAGATTTGATG

TCACAGTCTCGCACTCGCGAGATACTAACAAAAACCACTGTGGACCATATGGCCATAATA

AAGAAATACACATCAGGGAGACAGGAGAAGAACCCTGCCCTCAGGATGAAATGGATGATG

GCAATGAAATATCCTATTACAGCTGACAAAAGAATAATGGAGATGATCCCTGAAAGGAAT

GAGCAAGGTCAGACTCTCTGGAGCAGAACAAATGATGCTGGATCAGACAGAGTGATGGTC

TCACCTCTGGCTGTGACATGGTGGAATAGAAATGGGCCAACAACAAGTACAGTACACTAC

CCAAAAGTCTACAAAACCTACTTTGAAAAGGTAGAAAGGTTGAAACATGGAACCTTTGGT

CCTGTTCACTTTCGAAATCAGGTTAAGATACGCCGCAGGGTTGACATAAACCCGGGCCAT

GCAGATCTCAGTGCCAAAGAAGCACAGGATGTCATCATGGAGGTTGTTTTCCCAAATGAA

GTTGGAGCCAGGATCTTGACATCAGAATCACAGTTAACAATAACAATGGAAAAGAAGGAG

GAACTTCAGGATTGCAAGATTGCTCCTTTGATGGTGGCATACATGTTGGAAAGAGAACTG

GTTCGCAAGACCAGATTTTTACCAGTAGCTGGCGGAACAAGCAGCGTATACATCGAGGTA

TTGCATTTGACTCAAGGGACCTGCTGGGAACAAATGTACACACCAGGAGGGGAGGTGAGA

AATGATGATGTTGATCAGAGTTTGATCATTGCTGCTAGAAATATAGTTAGGAGGGCAACA

GTATCAGCAGACCCATTGGCTTCGCTCTTGGAAATGTGCCACAGTACACAAATTGGTGGA

GTGAGGATGGTGGACATTCTTAGGCAGAACCCAACAGAGGAGCAAGCTGTGGATATATGC

AAAGCAGCAATGGGTTTAAGAATCAGTTCATCCTTCAGCTTTGGAGGTTTCACTTTCAAA

AGGACAAGTGGGTCGTCTGTCAAAAGAGAAGAAGAAATACTCACTGGCAACCTCCAAACA

CTGAAAGTAAGAATACATGAAGGATATGAGGAATTCACAATGGTTGGGCGAAGAGCTACA

GCCATTTTGAGGAAAGCAACCAGGAGACTGATCCAATTAATAGTGAGTGGACGAGACGAA

CAGTCAATCGCTGAAGCAATCATAGTGGCAATGGTTTTCTCACAGGAGGATTGCATGATA

AAAGCAGTACGAGGTGATTTGAATTTTGTCAACAGAGCGAATCAGCGGCTAAATCCTATG

CATCAACTTCTGAGGCATTTCCAAAAAGATGCAAAAGTGCTGTTTCAAAACTGGGGGATT

GAACCAATTGACAATGTAATGGGGATGATCGGGATACTGCCTGACATGACCCCCAGCACA

GAGATGTCACTGAGAGGAGTGAGAGTCAGCAAAATGGGAGTGGATGAATATTCCAGTACT

GAGAGAGTGGTCGTGAGCATTGATCGCTTCTTGAGAGTCCGAGATCAGAGGGGAAATGTG

CTTCTGTCTCCTGAGGAAGTTAGTGAAACACATGGAACAGAGAAACTGACGATAACGTAT

TCATCGTCTATGATGTGGGAAATCAATGGTCCGGAATCCGTGCTAGTCAACACATATCAA

TGGATCATTAGAAATTGGGAAACTGTGAAGATTCAGTGGTCCCAGGACCCTACGATGTTG

TACAATAAGATGGAATTTGAGCCCTTCCAATCCTTGGTGCCCAAGGCTGCTAGAGGCCAG

TATAGTGGGTTTGTGAGGACATTATTCCAACAGATGCGTGATGTGTTGGGGACATTTGAC

ACTGTCCAAATAATAAAGCTCCTACCATTTGCAGCAGCCCCACCGGAACAGAGTAGGATG

CAATTTTCCTCTCTGACTGTGAACGTAAGAGGTTCAGGAATGAGAATACTTGTGAGGGGC

AACTCCCCTGTGTTCAACTATAATAAGACCACCAAGAGACTCACAGTTCTTGGAAAGGAT

GCAGGCGCCTTGACAGAATATCCAGATGAGGGAACAGCAGGAGTGGAGTCTGCAGTATTA

AGAGGATTTCTAATTCTGGGCAAAGAGGACAAAAGATATGGACCAGCATTGAGCATCAAC

GAATTGAGCAATCTTGCGAAAGGGGAAAAGGCTAATGTGTTGATAGGACAAGGAGACGTG

GTGTTGGTAATGAAACGGAAACGGGACTCTAGCATACTTACTGACAGCCAGACAGCGACC

AAAAGAATTCGGATGGCCATCAATTAGTGTCGAATTGTTTAA------------------

----------------------

>A_chicken_Czech_Republic_1566-1_2021_EPI1844080

------------------------AATATGGAGAGAATAAAAGAACTAAGAGATTTGATG

TCGCAGTCTCGCACTCGCGAGATACTAACAAAAACCACTGTGGACCATATGGCCATAATA

AAGAAATACACATCAGGAAGACAGGAGAAGAACCCTGCCCTCAGGATGAAATGGATGATG

GCAATGAAATATCCTATTACAGCTGACAAAAGAATAATGGAGATGATCCCTGAAAGGAAT

GAGCAAGGTCAGACTCTCTGGAGCAAAACAAATGATGCTGGATCAGACAGAGTGATGGTC

TCACCCCTGGCTGTGACATGGTGGAATAGAAATGGGCCAACAACAAGTACAGTACACTAC

CCAAAAGTCTACAAAACCTACTTTGAAAAGGTAGAAAGGTTGAAACATGGAACCTTTGGT

CCTGTTCACTTTCGAAATCAGGTTAAGATACGCCGCAGGGTTGACATAAACCCGGGCCAT

GCAGATCTCAGTGCCAAAGAAGCACAGGATGTCATCATGGAGGTTGTTTTCCCAAATGAA

GTTGGAGCCAGGATCTTGACATCAGAATCACAATTAACAATAACAAGAGAAAAGAAGGAG

GAACTTCAGGATTGCAAGATTGCTCCTTTGATGGTGGCATACATGTTGGAAAGAGAACTG

GTTCGCAAGACCAGATTTTTACCAGTAGCTGGCGGAACAAGCAGCGTATACATCGAGGTA

TTGCATTTGACTCAAGGGACCTGCTGGGAACAAATGTACACACCAGGAGGGGAGGTGAGA

AATGATGATGTTGATCAGAGTTTGATCATTGCTGCTAGAAATATAGTTAGGAGGGCAACA

GTATCAGCAGACCCTTTGGCTTCGCTCTTGGAAATGTGCCACAGTACACAAATTGGTGGA

ATAAGGATGGTGGACATTCTTAGGCAGAACCCAACAGAGGAGCAAGCTGTGGATATATGC

AAAGCAGCAATGGGTTTAAGAATCAGTTCATCCTTCAGCTTTGGAGGTTTCACTTTCAAA

AGGACAAGTGGGTCGTCTGTCAAAAGAGAAGAAGAAATACTCACTGGCAACCTCCAAACA

CTGAAAGTAAGAATACATGAAGGATATGAGGAATTCACAATGGTTGGGCGAAGAGCTACA

GCCATTTTGAGGAAAGCAACCAGGAGACTGATCCAATTAATAGTGAGTGGAAGAGACGAG

CAGTCAATCGCTGAAGCAATCATAGTGGCAATGGTTTTCTCACAGGAGGATTGCATGATA

AAAGCAGTACGAGGTGATTTGAATTTTGTCAACAGAGCGAATCAGCGGCTAAATCCTATG

CATCAACTTCTGAGGCATTTCCAAAAGGATGCAAAAGTGCTGTTTCAAAACTGGGGGATT

GAACCAATTGACAATGTAATGGGGATGATCGGGATACTGCCTGACATGACCCCCAGCACA

GAGATGTCACTGAGAGGAGTGAGAGTCAGCAAAATGGGAGTGGATGAATATTCCAGTACT

GAGAGAGTGGTCGTGAGCATTGATCGCTTCTTGAGAGTCCGAGATCAGAGGGGAAATGTG

CTTTTGTCTCCTGAGGAAGTTAGTGAAACACATGGAACAGAGAAACTGACGATAACGTAT

TCATCGTCTATGATGTGGGAAATCAATGGTCCGGAATCCGTGCTAGTCAACACATATCAA

TGGATCATTAGAAGTTGGGAAACTGTGAAGATTCAATGGTCCCAGGACCCTACGATGTTG

TACAATAAGATGGAATTTGAGCCCTTCCAATCCTTGGTGCCCAAGGCTGCTAGAGGCCAG

TATAGTGGGTTTGTGAGGACATTATTCCAACAGATGCGTGATGTGTTGGGGACATTTGAC

ACTGTCCAAATAATAAAGCTCCTACCATTTGCAGCAGCCCCACCGGAACAGAGTAGGATG

CAATTTTCCTCTCTGACTGTGAACGTAAGAGGTTCAGGAATGAGAATACTTGTGAGGGGC

AACTCCCCTGTGTTCAACTATAATAAGGCCACCAAGAGACTCACAGTTCTTGGAAAGGAT

GCAGGCGCCTTGACAGAATATCCAGATGAGGGAACAGCAGGAGTTGAGTCTGCAGTATTA

AGAGGATTTCTAATTCTGGGCAAAGAGGACAAAAGATATGGACCAGCATTGAGCATCAAC

GAATTGAGCAATCTTGCGAAAGGGGAAAAGGCTAATGTGTTGATAGGACAAGGAGACGTG

GTGTTGGTAATGAAACGGAAACGGGACTCTAGCATACTTACTGACAGCCAGACAGCGACC

AAAAGAATTCGGATGGCCATCAATTAGTGTCGAATTGTTTAA------------------

----------------------

>A_chicken_Korea_H008_2021_EPI1846535

---------------------------ATGGAGAGAATAAAAGAACTAAGAGATTTGATG

TCGCAGTCTCGCACTCGCGAGATACTAACAAAAACCACTGTGGACCATATGGCCATAATA

AAGAAATACACATCAGGAAGACAGGAGAAGAACCCTGCCCTCAGGATGAAATGGATGATG

GCAATGAAATATCCTATTACAGCTGACAAAAGAATAATGGAGATGATCCCTGAAAGGAAT

GAGCAAGGTCAGACTCTCTGGAGCAAAACAAATGATGCTGGATCAGACAGAGTGATGGTC

TCACCTCTGGCTGTGACATGGTGGAATAGGAATGGGCCAACAACAAGTACAGTACACTAC

CCAAAAGTCTACAAAACCTACTTTGAAAAGGTAGAAAGGTTGAAACATGGAACCTTTGGT

CCTGTTCACTTTCGAAATCAGGTTAAGATACGCCGCAGGGTTGACATAAACCCGGGCCAT

GCAGATCTCAGTGCCAAAGAAGCACAGGATGTCATCATGGAGGTTGTTTTCCCAAATGAA

GTTGGAGCCAGGATCTTGACATCAGAATCACAATTAACAATAACAAGGGAAAAGAAGGAG

GAACTTCAGGATTGCAAGATTGCTCCTTTGATGGTGGCATACATGTTGGAAAGAGAACTG

GTTCGCAAGACCAGATTTTTACCAGTAGCTGGCGGAACAAGCAGCGTATACATCGAGGTA

TTGCATTTGACTCAAGGGACCTGCTGGGAACAAATGTACACACCAGGAGGGGAGGTGAGA

AATGATGATGTTGATCAGAGTTTGATCATTGCTGCTAGAAATATAGTTAGGAGGGCAACA

GTATCAGCAGACCCATTGGCTTCGCTCTTGGAAATGTGCCACAGTACACAAATTGGTGGA

GTAAGGATGGTGGACATTCTCAGGCAGAACCCAACAGAGGAGCAAGCTGTGGATATATGC

AAAGCAGCAATGGGTTTAAGAATCAGTTCATCCTTCAGCTTTGGAGGTTTCACTTTCAAA

AGGACAAGTGGGTCGTCTGTCAAAAGAGAAGAAGAAATACTCACTGGCAACCTCCAAACG

CTGAAAGTTAGAATACATGAAGGATATGAGGAATTCACAATGGTTGGGCGAAGAGCTACA

GCCATTTTGAGGAAAGCAACCAGGAGACTGATCCAATTAATAGTGAGTGGAAGAGACGAG

CAGTCAATCGCTGAAGCAATCATAGTGGCAATGGTTTTCTCACAGGAGGATTGCATGATA

AAAGCAGTACGAGGTGATTTGAATTTTGTCAACAGAGCGAATCAGCGGCTAAATCCTATG

CATCAACTTCTGAGGCATTTCCAAAAGGATGCAAAAGTGCTGTTTCAAAACTGGGGGATT

GAATCAATTGACAATGTAATGGGGATGATCGGGATACTGCCAGACATGACCCCCAGCACA

GAGATGTCACTGAGAGGAGTGAGAGTCAGCAAAATGGGAGTGGATGAATATTCCAGTACT

GAGAGAGTGGTCGTGAGCATTGATCGCTTCTTGAGAGTCCGAGATCAGAGGGGAAATGTG

CTTCTGTCTCCTGAGGAAGTTAGTGAAACACATGGAACAGAGAAACTGACGATAACGTAT

TCATCGTCTATGATGTGGGAAATCAATGGTCCGGAATCCGTGCTAGTCAACACATATCAA

TGGATCATTAGAAGTTGGGAAACTGTGAAGATTCAATGGTCCCAGGACCCTACGATGTTG

TACAATAAGATGGAATTTGAGCCCTTCCAATCCTTGGTGCCCAAGGCTGCTAGAGGCCAG

TATAGTGGGTTTGTGAGGACATTATTCCAACAGATGCGTGATGTGTTGGGGACATTTGAC

ACTGTCCAAATAATAAAGCTCCTACCATTTGCAGCAGCCCCACCGGAACAGAGTAGGATG

CAATTTTCCTCTCTGACTGTGAACGTAAGAGGTTCAGGAATGAGAATACTTGTGAGGGGC

AACTCCCCTGTGTTCAACTATAATAAGGCCACCAAGAGACTCACAGTTCTTGGAAAGGAT

GCAGGCGCCTTGACAGAATATCCGGATGAGGGAACAGCAGGAGTGGAGTCTGCAGTATTA

AGAGGATTTCTAATTCTGGGCAAAGAGGACAAAAGATATGGACCAGCATTGAGCATCAAC

GAATTGAGCAATCTTGCGAAAGGGGAAAAGGCTAATGTGTTGATAGGACAAGGAGACGTG

GTGTTGGTAATGAAACGGAAACGGGACTCTAGCATACTTACTGACAGCCAGACAGCGACC

AAAAGAATTCGGATGGCCATCAATTAG---------------------------------

----------------------

>A_mallard_Korea_WA820_2020_EPI1846599

---------------------------ATGGAGAGAATAAAAGAACTAAGAGATTTGATG

TCGCAGTCTCGCACTCGCGAGATACTAACAAAAACCACTGTGGACCATATGGCCATAATA

AAGAAATACACATCAGGAAGACAGGAGAAGAACCCTGCCCTCAGGATGAAATGGATGATG

GCAATGAAATATCCTATTACAGCTGATAAAAGAATAATGGAGATGATCCCTGAAAGGAAT

GAGCAAGGTCAGACTCTCTGGAGCAAAACAAATGATGCTGGATCAGACAGAGTGATGGTC

TCACCTCTGGCTGTGACATGGTGGAATAGGAATGGGCCAACAACAAGTACAGTACACTAC

CCAAAAGTCTACAAAACCTACTTTGAAAAGGTAGAAAGGTTGAAACATGGAACCTTTGGT

CCTGTTCACTTTCGAAATCAGGTTAAGATACGCCGCAGGGTTGACATAAACCCGGGCCAT

GCAGATCTCAGTGCCAAAGAAGCACAGGATGTCATCATGGAGGTTGTTTTCCCAAATGAA

GTTGGAGCCAGGATCTTGACATCAGAATCACAATTAACAATAACAAGGGAAAAGAAGGAG

GAACTTCAGGATTGCAAGATTGCTCCTTTGATGGTGGCATACATGTTGGAAAGAGAACTG

GTTCGCAAGACCAGATTTTTACCAGTAGCTGGCGGAACAAGCAGCGTATACATCGAGGTA

TTGCATTTGACTCAAGGGACCTGCTGGGAACAAATGTACACACCAGGAGGGGAGGTGAGA

AATGATGATGTTGATCAGAGTTTGATCATTGCTGCTAGAAATATAGTTAGGAGGGCAACA

GTATCAGCAGACCCATTGGCTTCGCTCTTGGAAATGTGCCACAGTACACAAATTGGTGGA

GTAAGGATGGTGGACATTCTTAGGCAGAACCCAACAGAGGAGCAAGCTGTGGATATATGC

AAAGCAGCAATGGGTTTAAGAATCAGTTCATCCTTCAGCTTTGGAGGTTTCACTTTCAAA

AGGACAAGTGGGTCGTCTGTCAAAAGAGAAGAAGAAATACTCACTGGCAACCTCCAAACA

CTGAAAGTTAGAATACATGAAGGATATGAGGAATTCACAATGGTTGGGCGAAGAGCTACA

GCCATTTTGAGGAAAGCAACCAGGAGACTGATCCAATTAATAGTGAGTGGAAGAGACGAG

CAGTCAATCGCTGAAGCAATCATAGTGGCAATGGTTTTCTCACAGGAGGATTGCATGATA

AAAGCAGTACGAGGTGATTTGAATTTTGTCAACAGAGCGAATCAGCGGCTAAATCCTATG

CATCAACTTCTGAGGCATTTCCAAAAGGATGCAAAAGTGCTGTTTCAAAACTGGGGGATT

GAACCAATTGACAATGTAATGGGGATGATCGGGATACTGCCAGACATGACCCCCAGCACA

GAGATGTCACTGAGAGGAGTGAGAGTCAGCAAAATGGGAGTGGATGAATATTCCAGTACT

GAGAGAGTGGTCGTGAGCATTGATCGCTTCTTGAGAGTCCGAGATCAGAGGGGAAATGTG

CTTCTGTCTCCTGAGGAAGTTAGTGAAACACATGGAACAGAGAAACTGACGATAACGTAT

TCATCGTCTATGATGTGGGAAATCAATGGTCCGGAATCCGTGTTAGTCAACACATATCAA

TGGATCATTAGAAGTTGGGAAACTGTGAAGATTCAGTGGTCCCAGGACCCTACGATGTTG

TACAATAAGATGGAATTTGAGCCCTTCCAATCCTTGGTGCCCAAGGCTGCTAGAGGCCAG

TATAGTGGGTTTGTGAGGACATTATTCCAACAGATGCGTGATGTGTTGGGGACATTTGAC

ACTGTCCAAATAATAAAGCTCCTACCATTTGCAGCAGCCCCACCGGAACAGAGTAGGATG

CAATTTTCCTCTCTGACTGTGAACGTAAGAGGTTCAGGAATGAGAATACTTGTGAGGGGC

AACTCCCCTGTGTTCAACTATAATAAGGCCACCAAGAGACTCACAGTTCTTGGAAAGGAT

GCAGGCGCCTTGACAGAATATCCAGATGAGGGAACAGCAGGAGTGGAGTCTGCAGTATTA

AGAGGATTTCTAATTCTGGGCAAAGAGGACAAAAGATATGGACCAGCATTGAGCATCAAC

GAATTGAGCAATCTTGCGAAAGGGGAAAAGGCTAATGTGTTGATAGGGCAAGGAGACGTG

GTGTTGGTAATGAAACGGAAACGGGACTCTAGCATACTTACTGACAGCCAGACAGCGACC

AAAAGAATTCGGATGGCCATCAATTAG---------------------------------

----------------------

>A_chicken_Astrakhan_321-01_2020_EPI1846966

AGCAAAAGCAGGTCAAATATATTCAATATGGAGAGAATAAAAGAACTAAGAGATTTGATG

TCGCAGTCTCGCACTCGCGAGATACTAACAAAAACCACTGTGGACCATATGGCCATAATA

AAGAAATACACATCAGGAAGACAGGAGAAGAACCCTGCCCTCAGGATGAAATGGATGATG

GCAATGAAATATCCTATTACAGCTGACAAAAGAATAATGGAGATGATCCCTGAAAGGAAT

GAGCAAGGTCAGACTCTCTGGAGCAAAACAAATGATGCTGGTTCAGACAGAGTGATGGTC

TCACCTCTGGCTGTGACATGGTGGAATAGAAATGGGCCAACAACAAGTACAGTACACTAC

CCAAAAGTCTACAAAACCTACTTTGAAAAGGTAGAAAGGTTGAAACATGGAACCTTTGGT

CCTGTTCACTTTCGAAATCAGGTTAAGATACGCCGCAGGGTTGACATAAACCCGGGCCAT

GCAGATCTCAGTGCCAAAGAAGCACAGGATGTCATCATGGAGGTTGTTTTCCCAAATGAA

GTTGGAGCCAGGATCTTGACATCAGAATCACAATTAACAATAACAAGGGAAAAGAAGGAG

GAACTTCAGGATTGCAAGATTGCTCCTTTGATGGTGGCATACATGTTGGAAAGAGAACTG

GTTCGCAAGACCAGATTTTTACCAGTAGCTGGCGGAACAAGCAGCGTATACATCGAGGTA

TTGCATTTGACTCAAGGGACCTGCTGGGAACAAATGTACACACCAGGAGGGGAGGTGAGA

AATGATGATGTTGATCAGAGTTTGATCATTGCTGCTAGAAATATAGTTAGGAGGGCAACA

GTATCAGCAGACCCATTGGCTTCGCTCTTGGAAATGTGCCACAGTACACAAATTGGTGGA

GTAAGGATGGTGGACATTCTTAGGCAGAACCCAACAGAGGAGCAAGCTGTGGATATATGC

AAAGCAGCAATGGGTTTAAGAATCAGTTCATCCTTCAGTTTTGGAGGTTTCACTTTCAAA

AGGACAAGTGGGTCGTCTGTCAAAAGAGAAGAAGAAATACTCACTGGCAACCTCCAAACA

CTGAAAGTAAGAATACATGAAGGATATGAGGAATTCACAATGGTTGGGCGAAGGGCTACA

GCCATTTTGAGGAAAGCAACCAGGAGACTGATCCAATTAATAGTGAGTGGAAGAGACGAG

CAGTCAATCGCTGAAGCAATCATAGTGGCAATGGTTTTCTCACAGGAGGATTGCATGATA

AAAGCAGTACGAGGTGATTTGAATTTTGTCAACAGAGCGAATCAGCGGCTAAATCCTATG

CATCAACTTCTGAGGCATTTCCAAAAGGATGCAAAAGTGCTGTTTCAAAACTGGGGGATT

GAACCAATTGACAATGTAATGGGGATGATCGGGATACTGCCCGACATGACCCCCAGCACA

GAGATGTCACTGAGAGGAGTGAGAGTCAGCAAAATGGGAGTGGATGAATATTCCAGTACT

GAGAGAGTGGTCGTGAGCATTGATCGCTTCTTGAGAGTCCGAGATCAGAGGGGAAATGTG

CTTCTGTCTCCTGAGGAAGTTAGTGAAACACATGGAACAGAGAAACTGACGATAACGTAT

TCATCGTCTATGATGTGGGAAATCAATGGTCCGGAATCCGTGCTAGTCAACACATATCAA

TGGATCATTAGAAGTTGGGAAACTGTGAAGATTCAATGGTCCCAGGACCCTACGATGTTG

TACAATAAGATGGAATTTGAGCCCTTCCAATCCTTGGTGCCCAAGGCTGCTAGAGGCCAG

TATAGTGGGTTTGTGAGGACATTATTCCAACAGATGCGTGATGTGTTGGGGACATTTGAC

ACTGTCCAAATAATAAAGCTCCTACCATTTGCAGCAGCCCCACCGGAACAGAGTAGGATG

CAATTTTCCTCTCTGACTGTGAACGTAAGAGGTTCAGGAATGAGAATACTTGTGAGGGGC

AACTCCCCTGTGTTCAACTATAATAAGGCCACCAAGAGACTCACAGTTCTTGGAAAGGAT

GCAGGCGCCTTGACAGGATATCCAGATGAGGGAACAGCAGGAGTGGAGTCTGCAGTATTA

AGAGGATTTCTAATTCTGGGCAAAGAGGACAAAAGATATGGACCAGCATTGAGCATCAAC

GAATTGAGCAATCTTGCGAAAGGGGAAAAGGCTAATGTGTTGATAGGACAAGGAGACGTG

GTGTTGGTAATGAAACGGAAACGGGACTCTAGCATACTTACTGACAGCCAGACAGCGACC

AAAAGAATTCGGATGGCCATCAATTAGTGTCGAATTATTTAAAAACGACCTTGTTTCTAC

T---------------------

>A_crane_Kagoshima_KU-93_2021_EPI1848524

---------------------------ATGGAGAGAATAAAAGAACTAAGAGATTTGATG

TCGCAGTCTCGCACTCGCGAGATACTAACAAAAACCACTGTGGACCATATGGCCATAATA

AAGAAATACACATCAGGAAGACAGGAGAAGAACCCTGCCCTCAGGATGAAATGGATGATG

GCAATGAAATATCCTATTACAGCTGACAAAAGAATAATGGAGATGATCCCTGAAAGGAAT

GAGCAAGGTCAGACTCTCTGGAGCAAAACAAATGATGCTGGATCAGACAGAGTGATGGTC

TCACCTCTGGCTGTGACATGGTGGAATAGAAATGGGCCAACAACAAGTACAGTACACTAC

CCAAAAGTCTACAAAACCTACTTTGAAAAGGTAGAAAGGTTGAAACATGGAACCTTTGGT

CCTGTTCACTTTCGAAATCAGGTTAAGATACGCCGCAGGGTTGACATAAACCCGGGCCAT

GCAGATCTCAGTGCCAAAGAAGCACAGGATGTCATCATGGAGGTTGTTTTCCCAAATGAA

GTTGGAGCCAGGATCTTGACATCAGAATCACAATTAACAATAACAAGGGAAAAGAAGGAG

GAACTTCAGGATTGCAAGATTGCTCCTTTGATGGTGGCATACATGTTGGAAAGAGAACTG

GTTCGCAAGACCAGATTTTTACCAGTAGCTGGCGGAACAAGCAGCGTATACATCGAGGTA

TTGCATTTGACTCAAGGGACCTGCTGGGAACAAATGTACACACCAGGAGGGGAGGTGAGA

AATGATGATGTTGATCAGAGTTTGATCATTGCTGCTAGAAATATAGTTAGGAGGGCAACA

GTATCAGCAGACCCATTGGCTTCGCTCTTGGAAATGTGCCACAGTACACAAATTGGTGGA

GTAAGGATGGTGGACATTCTTAGGCAGAACCCAACAGAGGAGCACGCTGTGCATACATGC

AAAGCACCAATGGGTCTAAGAATCAGTTCACCCTCCACCTTTGGAGGTTTCACTTTCAAA

AGGACAAGTGGGTCGTCTGTCAAAAGAGAAGAAGAAATACTCACTGGCAACCTCCAAACG

CTGAAAGTTAGAATACATGAAGGATATGAGGAATTCACAATGGTTGGGCGAAGAGCTACA

GCCATTTTGAGGAAAGCAACCAGGAGACTGATCCAATTAATAGTGAGTGGAAGAGACGAG

CAGTCAATCGCTGAAGCAATCATAGTGGCAATGGTTTTCTCACAGGAGGATTGCATGATA

AAAGCAGTACGAGGTGATTTGAATTTTGTCAACAGAGCGAATCAGCGGCTAAATCCTATG

CATCAACTTCTGAGGCATTTCCAAAAGGATGCAAAAGTGCTGTTTCAAAACTGGGGNATT

GAATCAATTGACAATGTAATGGGGATGATCGGGATACTGCCAGACATGACCCCCAGCACA

GAGATGTCACTGAGAGGAGTGAGAGTCAGCAAAATGGGAGTGGATGAATATTCCAGTACT

GAGAGAGTGGTCGTGAGCATTGACCGCTTCTTGAGAGTCCGAGATCARARRGGAAATGTG

CTTCTGTCTCCTGAGGAAGTTAGTGAAACACAAGGAACAGAGAAACTGACGATAACGTAT

TCATCGTCTATGATGTGGGAAATCAATGGTCCGGAATCCGTGCTAGTCAACACATATCAA

TGGATCATTAGAAGTTGGGAAACTGTGAAGATTCAATGGTCCCAGGACCCTACGATGTTG

TACAATAAGATGGAATTTGAGCCCTTCCAATCCTTGGTGCCCAAGGCTGCTAGAGGCCAG

TATAGTGGGTTTGTGAGGACATTATTCCAACAGATGCGTGATGTGTTGGGGACATTTGAC

ACTGTCCAAATAATAAAGCTTCTACCATTTGCAGCAGCCCCACCGGAACAGAGTAGGATG

CAATTTTCCTCTCTGACTGTGAACATAAGAGGTTCAGGAATGAGAATACTTGTGAGGGGC

AACTCCCCTGTGTTCAACTATAATAAGGCCACCAAGAGACTCACAGTTCTTGGAAAGGAT

GCAGGCGCCTTGACAGAATATCCAGATGAGGGAACAGCAGGAGTGGAGTCTGCAGTATTA

AGAGGATTTCTAATTCTGGGCAAAGAGGACAAAAGATATGGACCAGCATTGAGCATCAAC

GAATTGAGCAATCTTGCGAAAGGGGAAAAGGCTAATGTGTTGATAGGACAAGGAGACGTG

GTGTTGGTAATGAAACGGAAACGGGACTCTAGCATACTTACTGACAGCCAGACAGCGACC

AAAAGAATTCGGATGGCCATCAATTAG---------------------------------

----------------------

>A_mallard_Kagoshima_KU-d89_2021_EPI1848535

---------------------------ATGGAGAGAATAAAAGAACTAAGAGATTTGATG

TCGCAGTCTCGCACTCGCGAGATACTAACAAAAACCACTGTGGACCATATGGCCATAATA

AAGAAATACACATCAGGAAGACAGGAGAAGAACCCTGCCCTCAGGATGAAATGGATGATG

GCAATGAAATATCCTATTACAGCTGACAAAAGAATAATGGAGATGATCCCTGAAAGGAAT

GAGCAAGGTCAGACTCTCTGGAGCAAAACAAATGATGCTGGATCAGACAGAGTGATGGTC

TCACCTCTGGCTGTGACATGGTGGAATAGGAATGGGCCAACAACAAGTACAGTACACTAC

CCAAAAGTCTACAAAACCTACTTTGAAAAGGTAGAAAGGTTGAAACATGGAACCTTTGGT

CCTGTTCACTTTCGAAATCAGGTTAAGATACGCCGCAGGGTTGACATAAACCCGGGCCAT

GCAGATCTCAGTGCCAAAGAAGCACAGGATGTCATCATGGAGGTTGTTTTCCCAAATGAA

GTTGGAGCCAGGATCTTGACATCAGAATCACAATTAACAATAACAAGGGAAAAGAAGGAG

GAACTTCAGGATTGCAAGATTGCTCCTTTGATGGTGGCATACATGTTGGAAAGAGAACTG

GTTCGCAAGACCAGATTTTTACCAGTAGCTGGCGGAACAAGCAGCGTATACATCGAGGTA

TTGCATTTGACTCAAGGGACCTGCTGGGAACAAATGTACACACCAGGAGGGGAGGTGAGA

AATGATGATGTTGATCAGAGTTTGATCATTGCTGCTAGAAATATAGTTAGGAGGGCAACA

GTATCAGCAGACCCATTGGCTTCGCTCTTGGAAATGTGCCACAGTACACAAATTGGTGGA

GTAAGGATGGTGGACATTCTTAGGCAGAACCCAACAGAGGAGCAAGCTGTGGATATATGC

AAAGCAGCAATGGGTTTAAGAATCAGTTCATCCTTCAGCTTTGGAGGTTTCACTTTCAAA

AGGACAAGTGGGTCGTCTGTCAAAAGAGAAGAAGAAATACTCACTGGCAACCTYCAAACG

CTGAAAGTTAGAATACATGAAGGATATGAGGAATTCACAATGGTTGGGCGAAGAGCTACA

GCCATTTTGAGGAAAGCAACCAGGAGACTGATCCAATTAATAGTGAGTGGAAGAGACGAG

CAGTCAATCGCTGAAGCAATCATAGTGGCAATGGTTTTCTCACAGGAGGATTGCATGATA

AAAGCAGTACGAGGTGATTTGAATTTTGTCAACAGAGCGAATCAGCGGCTAAATCCTATG

CATCAACTTCTGAGGCATTTCCAAAAGGATGCAAAAGTGCTGTTTCAAAACTGGGGGATT

GAATCAATTGACAATGTAATGGGGATGATCGGGATACTGCCAGACATGACCCCCAGCACA

GAGATGTCACTGAGAGGAGTGAGAGTCAGCAAAATGGGAGTGGATGAATATTCCAGTACT

GAGAGAGTGGTCGTGAGCATTGATCGCTTCTTGAGAGTCCGAGATCAGAGGGGAAATGTG

CTTCTGTCTCCTGAGGAAGTTAGTGAAACACATGGAACAGAGAAACTGACGATAACGTAT

TCATCGTCTATGATGTGGGAAATCAATGGTCCGGAATCCGTGCTAGTCAACACATATCAA

TGGATCATTAGAAGTTGGGAAACTGTGAAGATTCAATGGTCCCAGGACCCTACGATGTTG

TACAATAAGATGGAATTTGAGCCCTTCCAATCCTTGGTGCCCAAGGCTGCTAGAGGCCAG

TATAGTGGGTTTGTGAGGACATTATTCCAACAGATGCGTGATGTGTTGGGGACATTTGAC

ACTGTCCAAATAATAAAGCTCCTACCATTTGCAGCAGCCCCACCGGAACAGAGTAGGATG

CAATTTTCCTCTCTGACTGTGAACGTAAGAGGTTCAGGAATGAGAATACTTGTGAGGGGC

AACTCCCCTGTGTTCAACTATAATAAGGCCACCAAGAGACTCACAGTTCTTGGAAAGGAT

GCAGGCGCCTTGACAGAATATCCAGATGAGGGAACAGCAGGAGTGGAGTCTGCAGTATTA

AGAGGATTTCTAATTCTRGGCAAAGAGGACAAAAGATATGGACCAGCATTGAGCATCAAC

GAATTGAGCAATCTTGCGAAAGGGGAAAAGGCTAATGTGTTGATAGGACAAGGAGACGTG

GTGTTGGTAATGAAACGGAAACGGGACTCTAGCATACTTACTGACAGCCAGACAGCGACC

AAAAGAATTCGGATGGCCATCAATTAG---------------------------------

----------------------

>A_chicken_Kostroma_304-06_2020_EPI1848643

AGCAAAAGCAGGTCAAATATATTCAATATGGAGAGAATAAAAGAACTAAGAGATTTGATG

TCGCAGTCTCGCACTCGCGAGATACTAACAAAAACCACTGTGGACCATATGGCCATAATA

AAGAAATACACATCAGGAAGACAGGAGAAGAACCCTGCCCTCAGGATGAAATGGATGATG

GCAATGAAATATCCTATTACAGCTGACAAAAGAATAATGGAGATGATCCCTGAAAGGAAT

GAGCAAGGTCAGACTCTCTGGAGCAAAACAAATGATGCTGGATCAGACAGAGTGATGGTC

TCACCCCTGGCTGTGACATGGTGGAATAGAAATGGGCCAACAACAAGTACAGTACACTAC

CCAAAAGTCTACAAAACCTACTTTGAAAAGGTAGAAAGGTTGAAACATGGAACCTTTGGT

CCTGTTCACTTTCGAAATCAGGTTAAGATACGCCGCAGGGTTGACATAAACCCGGGCCAT

GCAGATCTCAGTGCCAAAGAAGCACAGGATGTCATCATGGAGGTTGTTTTCCCAAATGAA

GTTGGAGCCAGGATCTTGACATCAGAATCACAATTAACAATAACAAGGGAAAAGAAGGAG

GAACTTCAGGATTGCAAGATTGCTCCTTTGATGGTGGCATACATGTTGGAAAGAGAACTG

GTTCGCAAGACCAGATTTTTACCAGTAGCTGGCGGAACAAGCAGCGTATACATCGAGGTA

TTGCATTTGACTCAAGGGACCTGCTGGGAACAAATGTACACACCAGGAGGGGAGGTGAGA

AATGATGATGTTGATCAGAGTTTGATCATTGCTGCTAGAAATATAGTTAGGAGGGCAACA

GTATCAGCAGACCCATTGGCTTCGCTCTTGGAAATGTGCCACAGTACACAAATTGGTGGA

GTAAGGATGGTGGACATTCTTAGGCAGAACCCAACAGAGGAGCAAGCTGTGGATATATGC

AAAGCAGCAATGGGTTTAAGAATCAGTTCATCCTTCAGCTTTGGAGGTTTCACTTTCAAA

AGGACAAGTGGGTCGTCTGTCAAAAGAGAAGAAGAAATACTCACTGGCAACCTCCAAACA

CTGAAAGTAAGAATACATGAAGGATATGAGGAATTCACAATGGTTGGGCGAAGAGCTACA

GCCATTTTGAGGAAAGCAACCAGGAGACTGATCCAATTAATAGTGAGTGGAAGAGACGAG

CAGTCAATCGCTGAAGCAATCATAGTGGCAATGGTTTTCTCACAGGAGGATTGCATGATA

AAAGCAGTACGAGGTGATTTGAATTTTGTCAACAGAGCGAATCAGCGGCTAAATCCTATG

CATCAACTTCTGAGGCATTTCCAAAAGGATGCAAAAGTGCTGTTTCAAAACTGGGGGATT

GAACCAATTGACAATGTAATGGGGATGATCGGGATACTGCCTGACATGACCCCCAGCACA

GAGATGTCACTGAGAGGAGTGAGAGTCAGCAGAATGGGAGTGGATGAATATTCCAGTACT

GAGAGAGTGGTCGTGAGCATTGATCGCTTCTTGAGAGTCCGAGATCAGAGGGGAAATGTG

CTTCTGTCTCCTGAGGAAGTTAGTGAAACACATGGAACAGAGAAACTGACGATAACGTAT

TCATCGTCTATGATGTGGGAAATCAATGGTCCGGAATCCGTGCTAGTCAACACATATCAA

TGGATCATTAGAAGTTGGGAAACTGTGAAGATTCAATGGTCCCAGGACCCTACGATGTTG

TACAATAAGATGGAATTTGAGCCCTTCCAATCCTTGGTGCCCAAGGCTGCTAGAGGCCAG

TATAGTGGGTTTGTGAGGACATTATTCCAACAGATGCGTGATGTGTTGGGGACATTTGAC

ACTGTCCAAATAATAAAGCTCCTACCATTTGCAGCAGCCCCACCGGAACAGAGTAGGATG

CAATTTTCCTCTCTGACTGTGAACGTAAGAGGTTCAGGAATGAGAATACTTGTGAGGGGC

AACTCCCCTGTGTTCAACTATAATAAGGCCACCAAGAGACTCACAGTTCTTGGAAAGGAT

GCAGGCGCCTTGACAGAATATCCAGATGAGGGAACAGCAGGAGTGGAGTCTGCAGTATTA

AGAGGATTTCTAATTCTGGGCAAAGAGGACAAAAGATATGGACCAGCATTGAGCATCAAC

GAATTGAGCAATCTTGCGAAAGGGGAAAAGGCTAATGTGTTGATAGGACAAGGAGACGTG

GTGTTGGTAATGAAACGGAAACGGGACTCTAGCATACTTACTGACAGCCAGACAGCGACC

AAAAGAATTCGGATGGCCATCAATTAGTGTCGAATTGTTTAAAAACGACCTTGTTTCTAC

T---------------------

>A_chicken_Rostov-on-Don_308-02_2020_EPI1848667

AGCAAAAGCAGGTCAAATATATTCAATATGGAGAGAATAAAAGAACTAAGAGATTTGATG

TCGCAGTCTCGCACTCGCGAGATACTAACAAAAACCACTGTGGACCATATGGCCATAATA

AAGAAATACACATCAGGAAGACAGGAGAAGAACCCTGCCCTCAGGATGAAATGGATGATG

GCAATGAAATATCCTATTACAGCTGACAAAAGAATAATGGAGATGATCCCTGAAAGGAAT

GAGCAAGGTCAGACTCTCTGGAGCAAAACAAATGATGCTGGATCAGACAGAGTGATGGTC

TCACCTCTGGCTGTGACATGGTGGAATAGAAATGGGCCAACAACAAGTACAGCACACTAC

CCAAAAGTCTACAAAACCTACTTTGAAAAGGTAGAAAGGTTGAAACATGGAACCTTTGGT

CCTGTTCATTTTCGAAATCAGGTTAAGATACGCCGCAGGGTTGACATAAACCCGGGCCAT

GCAGATCTCAGTGCCAAAGAAGCACAGGATGTCATCATGGAGGTTGTTTTCCCAAATGAA

GTTGGAGCCAGGATCTTGACATCAGAATCACAATTAACAATAACAAGGGAAAAGAAGGAG

GAACTTCAGGATTGCAAGATTGCTCCTTTGATGGTGGCATACATGTTGGAAAGAGAACTG

GTTCGCAAGACCAGATTTTTACCAGTAGCTGGCGGAACAAGCAGCGTATACATCGAGGTA

TTGCATTTGACTCAAGGGACCTGCTGGGAACAAATGTACACACCAGGAGGGGAGGTGAGA

AATGATGATGTTGATCAGAGTTTGATCATTGCTGCTAGAAATATAGTTAGGAGGGCAACA

GTATCAGCAGACCCATTGGCTTCGCTTTTGGAAATGTGCCACAGTACACAAATTGGTGGA

GTAAGGATGGTGGACATTCTTAGGCAGAACCCAACAGAGGAGCAAGCTGTGGATATATGC

AAAGCAGCAATGGGTTTAAGAATCAGTTCATCCTTCAGCTTTGGAGGTTTCACTTTCAAA

AGGACAAGTGGGTCGTCTGTCAAAAGAGAAGAAGAAATACTCACTGGCAACCTCCAAACA

CTGAAAGTAAGAATACATGAAGGATATGAGGAATTCACAATGGTTGGGCGAAGAGCTACA

GCCATTTTGAGGAAAGCAACCAGGAGACTGATCCAATTAATAGTGAGTGGAAGAGACGAG

CAGTCAATCGCTGAAGCAATCATAGTGGCAATGGTTTTCTCACAGGAGGATTGCATGATA

AAAGCAGTACGAGGTGATTTGAATTTTGTCAACAGAGCGAATCAGCGGCTAAATCCTATG

CATCAACTTCTGAGGCATTTCCAAAAGGATGCAAAAGTGCTGTTTCAAAACTGGGGGATT

GAACCAATTGACAATGTAATGGGGATGATCGGGATACTGCCTGACATGACCCCCAGCACA

GAGATGTCACTGAGAGGAGTGAGAGTCAGCAAAATGGGAGTGGATGAATATTCCAGTACT

GAGAGAGTGGTCGTGAGCATTGATCGCTTCTTGAGAGTCCGAGATCAGAAGGGAAATGTG

CTTCTGTCTCCTGAGGAAGTTAGTGAAACACATGGAACAGAGAAACTGACGATAACGTAT

TCATCGTCTATGATGTGGGAAATCAATGGTCCGGAATCCGTGCTAGTCAACACATATCAA

TGGATCATTAGAAATTGGGAAACTGTGAAGATTCAGTGGTCCCAGGACCCTACGATGTTG

TACAATAAGATGGAATTTGAGCCCTTCCAATCCTTGGTGCCCAAGGCTGCTAGAGGCCAG

TATAGTGGGTTTGTGAGGACGTTATTCCAACAGATGCGTGATGTGTTGGGGACATTTGAC

ACTGTCCAAATAATAAAGCTCCTACCATTTGCAGCAGCCCCACCGGAACAGAGTAGGATG

CAATTTTCCTCTCTGACTGTGAACGTAAGAGGTTCAGGAATGAGAATACTTGTGAGGGGC

AACTCCCCTGTGTTCAACTATAATAAGGCCACCAAGAGACTCACAGTTCTTGGAAAGGAT

GCAGGCGCCTTGACAGAATATCCAGATGAGGGAACAGCAGGAGTGGAGTCTGCAGTATTA

AGAGGATTTCTAATTCTGGGCAAAGAGGACAAAAGATATGGACCAGCATTGAGCATCAAC

GAATTGAGCAATCTTGCGAAAGGGGAAAAGGCTAATGTGTTGATAGGACAAGGAGACGTG

GTGTTGGTAATGAAACGGAAACGGGACTCTAGCATACTTACTGACAGCCAGACAGCGACC

AAAAGAATTCGGATGGCCATCAATTAGTGTCGAATTGTTTAAAAACGACCTTGTTTCTAC

T---------------------

>A_turkey_Stavropol_320-02_2020_EPI1848699

AGCAAAAGCAGGTCAAATATATTCAATATGGAGAGAATAAAAGAACTAAGAGATTTGATG

TCGCAGTCTCGCACTCGCGAGATACTAACAAAAACCACTGTGGACCATATGGCCATAATA

AAGAAATACACATCAGGAAGACAGGAGAAGAACCCTGCCCTCAGGATGAAATGGATGATG

GCAATGAAATATCCTATTACAGCTGACAAAAGAATAATGGAGATGATCCCTGAAAGGAAT

GAGCAAGGTCAGACTCTCTGGAGCAAAACAAATGATGCTGGATCAGACAGAGTGATGGTC

TCACCTCTGGCTGTGACATGGTGGAATAGAAATGGGCCAACAACAAGTACAGTACACTAC

CCAAAAGTCTACAAAACCTACTTTGAAAAGGTAGAAAGGTTGAAACATGGAACCTTTGGT

CCTGTTCACTTTCGAAATCAGGTTAAGATACGCCGCAGGGTTGACATAAACCCGGGCCAT

GCAGATCTCAGTGCCAAAGAAGCACAGGATGTCATCATGGAGGTTGTTTTCCCAAATGAA

GTTGGAGCCAGGATCTTGACATCAGAATCACAATTAACAATAACAAGGGAAAAGAAGGAG

GAACTCCAGGATTGCAAGATTGCTCCTTTGATGGTGGCATACATGTTGGAAAGAGAACTG

GTTCGCAAGACCAGATTTTTACCAGTAGCTGGCGGAACAAGCAGCGTATACATCGAGGTA

TTGCATTTGACTCAAGGGACCTGCTGGGAACAAATGTACACACCAGGAGGGGAGGTGAGA

AATGATGATGTTGATCAGAGTTTGATCATTGCTGCTAGAAATATAGTTAGGAGGGCAACA

GTATCAGCAGACCCATTGGCTTCGCTTTTGGAAATGTGCCACAGTACACAAATTGGTGGA

GTAAGGATGGTGGACATTCTTAGGCAGAACCCAACAGAGGAGCAAGCTGTGGATATATGC

AAAGCAGCAATGGGTTTAAGAATCAGTTCATCCTTCAGCTTTGGAGGTTTCACTTTCAAA

AGGACAAGTGGGTCGTCTGTCAAAAGAGAAGAAGAAATACTCACTGGCAACCTCCAAACA

CTGAAAGTAAGAATACATGAAGGATATGAGGAATTCACAATGGTTGGGCGAAGAGCTACA

GCCATTTTGAGGAAAGCAACCAGGAGACTGATCCAATTAATAGTGAGTGGAAGAGACGAG

CAGTCAATCGCTGAAGCAATCATAGTGGCAATGGTTTTCTCACAGGAGGATTGCATGATA

AAAGCAGTACGAGGTGATTTGAATTTTGTCAACAGAGCGAATCAGCGGCTAAATCCTATG

CATCAACTTCTGAGGCATTTCCAAAAGGATGCAAAAGTGCTGTTTCAAAACTGGGGGATT

GAACCAATTGACAATGTAATGGGGATGATCGGGATACTGCCTGACATGACCCCCAGCACA

GAGATGTCACTGAGAGGAGTGAGAGTCAGCAAAATGGGAGTGGATGAATATTCCAGTACT

GAGAGAGTGGTCGTGAGCATTGATCGCTTCTTGAGAGTCCGAGATCAGAGGGGAAATGTG

CTTCTGTCTCCTGAGGAAGTTAGTGAAACACATGGAACAGAGAAACTGACGATAACGTAT

TCATCGTCTATGATGTGGGAAATCAATGGTCCGGAATCCGTGCTAGTCAACACATATCAA

TGGATCATTAGAAATTGGGAAACTGTGAAGATTCAGTGGTCCCAGGACCCTACGATGTTG

TACAATAAGATGGAATTTGAGCCCTTCCAATCCTTGGTGCCCAAGGCTGCTAGAGGCCAG

TATAGTGGGTTTGTGAGGACATTATTCCAACAGATGCGTGATGTGTTGGGGACATTTGAC

ACTGTCCAAATAATAAAGCTCCTACCATTTGCAGCAGCCCCACCGGAACAGAGTAGGATG

CAATTTTCCTCTCTGACTGTGAACGTAAGAGGTTCAGGAATGAGAATACTTGTGAGGGGC

AACTCCCCTGTGTTCAACTATAATAAGGCCACCAAAAGACTCACAGTTCTTGGAAAGGAT

GCAGGCGCCTTGACAGAATATCCAGATGAGGGAACAGCAGGAGTGGAGTCTGCAGTATTA

AGAGGATTTCTAATCCTGGGCAAAGAGGACAAAAGATATGGACCAGCATTGAGCATCAAC

GAATTGAGCAATCTTGCGAAAGGGGAAAAGGCTAATGTGTTGATAGGACAAGGAGACGTG

GTGTTGGTAATGAAACGGAAACGGGACTCTAGCATACTTACTGACAGCCAGACAGCGACC

AAAAGAATTCGGATGGCCATCAATTAGTGTCGAATTGTTTAAAAACGACCTTGTTTCTAC

T---------------------

>A_mute_swan_North_Ossetia-Alania_325-03_2020_EPI1848731

AGCAAAAGCAGGTCAAATATATTCAATATGGAGAGAATAAAAGAACTAAGAGATTTGATG

TCGCAGTCTCGCACTCGCGAGATACTAACAAAAACCACTGTGGACCATATGGCCATAATA

AAGAAATACACATCAGGAAGACAGGAGAAGAACCCTGCCCTCAGGATGAAATGGATGATG

GCAATGAAATATCCTATTACAGCTGACAAAAGAATAATGGAGATGATCCCTGAAAGGAAT

GAGCAAGGTCAGACTCTCTGGAGCAAAACAAATGATGCTGGATCAGACAGAGTGATGGTC

TCACCTCTGGCTGTGACATGGTGGAATAGAAATGGGCCAACAACAAGTACAGTACACTAC

CCAAAAGTCTACAAAACCTACTTTGAAAAGGTAGAAAGGTTGAAACATGGAACCTTTGGT

CCTGTTCACTTTCGAAATCAGGTTAAGATACGCCGCAGGGTTGACATAAACCCGGGCCAT

GCAGATCTCAGTGCCAAAGAAGCACAGGATGTCATCATGGAGGTTGTTTTCCCAAATGAA

GTTGGAGCCAGGATCTTGACATCAGAATCACAATTAACAATAACAAGGGAAAAGAAGGAG

GAACTTCAGGATTGCAAGATTGCTCCTTTGATGGTGGCATACATGTTGGAAAGAGAACTG

GTTCGCAAGACCAGATTTTTACCAGTAGCTGGCGGAACAAGCAGCGTATACATCGAGGTA

TTGCATTTGACTCAAGGGACCTGCTGGGAACAAATGTACACACCAGGAGGGGAGGTGAGA

AATGATGATGTTGATCAGAGTTTGATCATTGCTGCTAGAAATATAGTTAGGAGGGCAACA

GTATCAGCAGACCCTTTGGCTTCGCTCTTGGAAATGTGCCATAGTACACAAATTGGTGGG

GTAAGGATGGTGGACATTCTTAGGCAGAACCCAACAGAGGAGCAAGCTGTGGATATATGC

AAAGCAGCAATGGGTTTAAGAATCAGTTCATCCTTCAGCTTTGGAGGTTTCACTTTCAAA

AGAACAAGTGGGTCGTCTGTCAAAAGAGAAGAAGAAATACTCACTGGCAACCTCCAAACA

CTGAAAGTAAGAATACATGAAGGATATGAGGAATTCACAATGGTTGGGCGAAGAGCTACA

GCCATTTTGAGGAAAGCAACCAGGAGACTGATCCAATTAATAGTGAGTGGAAGAGACGAA

CAGTCAATCGCTGAAGCAATCATAGTGGCAATGGTTTTCTCACAGGAGGATTGCATGATA

AAAGCAGTACGAGGTGATTTGAATTTTGTCAACAGAGCGAATCAGCGGCTAAATCCTATG

CATCAACTTCTGAGGCATTTCCAAAAGGATGCAAAAGTGCTGTTTCAAAACTGGGGGATT

GAACCAATTGACAATGTAATGGGGATGATCGGGATACTGCCTGACATGACCCCCAGCACA

GAGATGTCACTGAGAGGAGTGAGAGTCAGCAAAATGGGAGTGGATGAATATTCCAGTACT

GAGAGAGTGGTCGTGAGCATTGATCGCTTCTTGAGAGTCCGAGATCAGAGGGGAAATGTG

CTTTTGTCTCCTGAGGAAGTTAGTGAAACACATGGAACAGAGAAACTGACGATAACGTAT

TCATCGTCTATGATGTGGGAAATCAATGGTCCGGAATCCGTGCTAGTCAACACATATCAA

TGGATCATTAGAAGTTGGGAAACTGTAAAGATTCAATGGTCCCAGGACCCTACGATGTTG

TACAATAAGATGGAATTTGAGCCCTTCCAATCCTTGGTGCCCAAGGCTGCTAGAGGCCAG

TATAGTGGGTTTGTGAGGACATTATTCCAACAGATGCGTGATGTGTTGGGGACATTTGAC

ACTGTCCAAATAATAAAGCTCCTACCATTTGCAGCAGCCCCACCGGAACAGAGTAGGATG

CAATTTTCCTCTCTGACTGTGAACGTAAGAGGTTCAGGAATGAGAATACTTGTGAGGGGC

AACTCCCCTGTGTTCAACTATAATAAGGCCACCAAGAGACTCACAGTTCTTGGAAAGGAT

GCAGGCGCCTTGACAGAATATCCAGATGAGGGAACAGCAGGAGTGGAGTCTGCAGTATTA

AGAGGATTTCTAATTCTGGGCAAAGAGGACAAAAGATATGGACCAGCATTGAGCATCAAC

GAATTGAGCAATATTGCGAAAGGGGAAAAGGCTAATGTGTTGATAGGACAAGGAGACGTG

GTGTTGGTAATGAAACGGAAACGGGACTCTAGCATACTTACTGACAGCCAGACAGCGACC

AAAAGAATTCGGATGGCCATCAATTAGTGTCGAATTGTTTAAAAACGACCTTGTTTCTAC

T---------------------

>A_turkey_Rostov-on-Don_332-09_2021_EPI1848755

AGCAAAAGCAGGTCAAATATATTCAATATGGAGAGAATAAAAGAACTAAGAGATTTGATG

TCGCAGTCTCGCACTCGCGAGATACTAACAAAAACCACTGTGGACCATATGGCCATAATA

AAGAAATACACATCAGGAAGACAGGAGAAGAACCCTGCCCTCAGGATGAAATGGATGATG

GCAATGAAATATCCTATTACAGCTGACAAAAGAATAATGGAGATGATCCCTGAAAGGAAT

GAGCAAGGTCAGACTCTCTGGAGCAAAACAAATGATGCTGGATCAGACAGAGTGATGGTC

TCACCTCTGGCTGTGACATGGTGGAATAGGAATGGGCCAACAACAAGTACAGTACACTAC

CCAAAAGTCTACAAAACCTACTTTGAAAAGGTAGAAAGGTTGAAACATGGAACCTTTGGT

CCTGTTCACTTTCGAAATCAGGTTAAGATACGCCGCAGGGTTGACATAAACCCGGGCCAT

GCAGATCTCAGTGCCAAAGAAGCACAGGATGTCATCATGGAGGTTGTTTTCCCAAATGAA

GTTGGAGCCAGGATCTTGACATCAGAATCACAATTAACAATAACAAGGGAAAAGAAGGAG

GAACTTCAGGATTGCAAGATTGCTCCTTTGATGGTGGCATACATGTTGGAAAGAGAACTG

GTTCGCAAGACCAGATTTTTACCAGTAGCTGGCGGAACAAGCAGCGTATACATCGAGGTA

TTGCATTTGACTCAAGGGACCTGCTGGGAACAAATGTACACACCAGGAGGGGAGGTGAGA

AATGATGATGTTGATCAGAGTTTGATCATTGCTGCTAGAAATATAGTTAGGAGGGCAACA

GTATCAGCAGACCCATTGGCTTCGCTCTTGGAAATGTGCCACAGTACACAAATTGGTGGA

GTAAGGATGGTGGACATCCTTAGGCATAACCCAACAGAGGAGCAAGCTGTGGATATATGC

AAAGCAGCAATGGGTTTAAGAATCAGTTCATCCTTCAGCTTTGGAGGTTTCACTTTCAAA

AGGACAAGTGGGTCGTCTGTCAAAAGAGAAGAAGAAATACTCACTGGCAACCTCCAAACA

CTGAAAGTAAGAATACATGAAGGATATGAGGAATTCACAATGGTTGGGCGAAGAGCTACA

GCCATTTTGAGGAAAGCAACCAGGAGACTGATCCAATTAATAGTGAGTGGAAGAGACGAG

CAGTCAATCGCTGAAGCAATCATAGTGGCAATGGTTTTCTCACAGGAGGATTGCATGATA

AAAGCAGTACGAGGTGATTTGAATTTTGTCAACAGAGCGAATCAGCGGCTAAATCCTATG

CATCAACTTCTGAGGCATTTCCAAAAGGATGCAAAAGTGCTGTTTCAAAACTGGGGGATT

GAACCAATTGACAATGTAATGGGGATGATCGGGATACTGCCAGACATGACCCCCAGCACA

GAGATGTCACTGAGAGGAGTGAGAGTCAGCAAAATGGGAGTGGATGAATATTCCAGTACT

GAGAGAGTGGTCGTGAGCATTGATCGCTTCTTGAGAGTCCGAGATCAGAGGGGAAATGTG

CTTCTGTCTCCTGAGGAAGTTAGTGAAACACATGGAACAGAGAAACTGACGATAACGTAT

TCATCGTCTATGATGTGGGAAATCAATGGTCCGGAATCCGTGCTAGTCAACACATATCAA

TGGATCATTAGAAGTTGGGAAACTGTGAAGATTCAATGGTCCCAGGACCCTACGATGTTG

TACAATAAGATGGAATTTGAGCCCTTCCAATCCTTGGTGCCCAAGGCTGCTAGAGGCCAG

TATAGTGGGTTTGTGAGGACATTGTTCCAACAGATGCGTGATGTGTTGGGGACATTTGAC

ACTGTCCAAATAATAAAGCTCCTACCATTTGCAGCAGCCCCACCGGAACAGAGTAGGATG

CAATTTTCCTCTCTAACTGTGAACGTAAGAGGTTCAGGAATGAGAATACTTGTGAGGGGC

AACTCCCCTGTGTTCAACTATAATAAGGCCACCAAGAGACTCACAGTTCTTGGAAAGGAT

GCAGGCGCCTTGACAGAATATCCAGATGAGGGAACAGCAGGAGTGGAGTCTGCAGTATTA

AGAGGATTTCTAATTCTGGGCAAAGAGGACAAAAGATATGGACCAGCATTGAGCATCAAC

GAATTGAGCAATCTTGCGAAAGGGGAAAAGGCTAATGTGTTGATAGGACAAGGAGACGTG

GTGTTGGTAATGAAACGGAAACGGGACTCTAGCATACTTACTGACAGCCAGACAGCGACC

AAAAGAATTCGGATGGCCATCAATTAGTGTCGAATTGTTTAAAAACGACCTTGTTTCTAC

T---------------------

>A_chicken_Krasnodar_334-03_2021_EPI1848803

AGCAAAAGCAGGTCAAATATATTCAATATGGAGAGAATAAAAGAACTAAGAGATTTGATG

TCGCAGTCTCGCACTCGCGAGATACTAACAAAAACCACTGTGGACCATATGGCCATAATA

AAGAAATACACATCAGGAAGACAGGAGAAGAACCCTGCCCTCAGGATGAAATGGATGATG

GCAATGAAATATCCTATTACAGCTGACAAAAGAATAATGGAGATGATCCCTGAAAGGAAT

GAGCAAGGTCAGACTCTCTGGAGCAAAACAAATGATGCTGGATCAGACAGAGTGATGGTC

TCACCTCTGGCTGTGACATGGTGGAATAGAAATGGGCCAACAACAAGTACAGTACACTAC

CCAAAAGTCTACAAAACCTACTTTGAAAAGGTAGAAAGGTTGAAACATGGAACCTTTGGT

CCTGTTCACTTTCGAAATCAGGTTAAGATACGCCGCAGGGTTGACATAAACCCGGGCCAT

GCAGATCTCAGTGCCAAAGAAGCACAGGATGTCATCATGGAGGTTGTTTTCCCAAATGAA

GTTGGAGCCAGGATCTTGACATCAGAATCACAATTAACAATAACAAGGGAAAAGAAGGAG

GAACTTCAGGATTGCAAGATTGCTCCTTTGATGGTGGCATACATGTTGGAAAGAGAACTG

GTTCGCAAGACCAGATTTTTACCAGTAGCTGGCGGAACAAGCAGCGTATACATCGAGGTA

TTGCATTTGACTCAAGGGACCTGCTGGGAACAAATGTACACACCAGGAGGGGAGGTGAGA

AATGATGATGTTGATCAGAGTTTGATCATTGCTGCTAGAAATATAGTTAGGAGGGCAACA

GTATCAGCAGACCCATTGGCTTCGCTTTTGGAAATGTGCCACAGTACACAAATTGGTGGA

GTAAGGATGGTGGACATTCTTAGGCAGAACCCAACAGAGGAGCAAGCTGTGGATATATGC

AAAGCAGCAATGGGTTTAAGAATCAGTTCATCCTTCAGCTTTGGAGGTTTCACTTTCAAA

AGGACAAGTGGGTCGTCTGTCAAAAGAGAAGAAGAAATACTCACTGGCAACCTCCAAACA

CTGAAAGTAAGAATACATGAAGGATATGAGGAATTCACAATGGTTGGGCGAAGAGCTACA

GCCATTTTGAGGAAAGCAACCAGGAGACTGATCCAATTAATAGTGAGTGGAAGAGACGAG

CAGTCAATCGCTGAAGCAATCATAGTGGCAATGGTTTTCTCACAGGAGGATTGCATGATA

AAAGCAGTACGAGGTGATTTGAATTTTGTCAACAGAGCGAATCAGCGGCTAAATCCTATG

CATCAACTTCTGAGGCATTTCCAAAAGGATGCAAAAGTGCTGTTTCAAAACTGGGGGATT

GAACCAATTGACAATGTAATGGGGATGATCGGGATACTGCCTGACATGACCCCCAGCACA

GAGATGTCACTGAGAGGAGTGAGAGTCAGCAAAATGGGAGTGGATGAATATTCCAGTACT

GAGAGAGTGGTCGTGAGCATTGATCGCTTCTTGAGAGTCCGAGATCAGAGGGGAAATGTG

CTTCTGTCTCCTGAGGAAGTTAGTGAAACACATGGAACAGAGAAACTGACGATAACGTAT

TCATCGTCTATGATGTGGGAAATCAATGGTCCGGAATCCGTGCTAGTCAACACATATCAA

TGGATCATTAGAAATTGGGAAACTGTGAAGATTCAGTGGTCCCAGGACCCTACGATGTTA

TACAATAAGATGGAATTTGAGCCCTTCCAATCCTTGGTGCCCAAGGCTGCTAGAGGTCAG

TATAGTGGGTTTGTAAGGACATTATTCCAACAGATGCGTGATGTGTTGGGGACATTTGAC

ACTGTCCAAATAATAAAGCTCCTACCATTTGCAGCAGCCCCACCGGAACAGAGTAGGATG

CAATTTTCCTCTCTGACTGTGAACGTAAGAGGTTCAGGAATGAGAATACTTGTGAGGGGC

AACTCCCCTGTGTTCAACTATAATAAGGCCACCAAGAGACTCACAGTTCTTGGAAAGGAT

GCAGGCGCCTTGACAGAATATCCAGATGAGGGAACAGCAGGAGTGGAGTCTGCAGTATTA

AGAGGATTTCTAATTCTGGGCAAAGAGGACAAAAGATATGGACCAGCATTGAGCATCAAC

GAATTGAGCAATCTTGCGAAAGGGGAAAAGGCTAATGTGTTGATAGGACAAGGAGACGTG

GTGTTGGTAATGAAACGGAAACGGGACTCTAGCATACTTACTGACAGCCAGACAGCGACC

AAAAGAATTCGGATGGCCATCAATTAGTGTCGAATTGTTTAAAAACGACCTTGTTTCTAC

T---------------------

>A_pheasant_Wales_000252_2021_EPI1848883

--------------AAATATATTCAATATGGAGAGAATAAAAGAACTAAGAGATTTGATG

TCGCAGTCTCGCACTCGCGAGATACTAACAAAAACCACTGTGGACCATATGGCCATAATA

AAGAAATACACATCAGGAAGACAGGAGAAGAACCCTGCCCTCAGGATGAAATGGATGATG

GCAATGAAATATCCTATTACAGCTGACAAAAGAATAATGGAGATAATCCCTGAAAGGAAT

GAGCAAGGTCAGACTCTCTGGAGCAAAACAAATGATGCTGGATCAGACAGAGTGATGGTC

TCACCTCTGGCTGTGACATGGTGGAATAGAAATGGGCCAACAACAAGTACAGTACACTAC

CCAAAAGTCTACAAAACCTACTTTGAAAAGGTAGAAAGGTTGAAACATGGAACCTTTGGT

CCTGTTCACTTTCGAAATCAGGTTAAGATACGCCGCAGGGTTGACATAAACCCGGGCCAT

GCAGATCTCAGTGCCAAAGAAGCACAGGATGTCATCATGGAGGTTGTTTTCCCAAATGAA

GTTGGAGCCAGGATCTTGACATCAGAATCACAATTAACAATAACAAGGGAAAAGAAGGAG

GAACTTCAGGATTGCAAGATTGCTCCTTTGATGGTGGCATACATGTTGGAAAGAGAACTG

GTTCGCAAGACCAGATTCTTACCAGTAGCTGGAGGAACAAGCAGCGTATACATCGAGGTA

TTGCATTTGACTCAAGGGACCTGCTGGGAACAAATGTACACACCAGGAGGGGAGGTGAGA

AATGATGATGTTGATCAGAGTTTGATCATTGCTGCTAGAAATATAGTTAGGAGGGCAACA

GTATCAGCAGACCCATTGGCTTCGCTTTTGGAAATGTGCCACAGTACACAAATTGGTGGA

GTAAGGATGGTGGACATTCTTAGGCAGAACCCAACAGAGGAGCAAGCTGTGGATATATGC

AAAGCAGCAATGGGTTTAAGAATCAGTTCATCCTTCAGCTTTGGAGGTTTCACTTTCAAA

AGGACAAGTGGGTCGTCTGTCAAAAGAGAAGAAGAAATACTCACTGGCAACCTCCAAACA

CTGAAAGTAAGAATACATGAAGGATATGAGGAATTCACAATGGTTGGGCGAAGAGCTACA

GCCATTTTGAGGAAAGCAACCAGGAGACTGATCCAATTAATAGTGAGTGGAAGAGACGAG

CAGTCAATCGCTGAAGCAATCATAGTGGCAATGGTTTTCTCACAGGAGGATTGCATGATA

AAAGCAGTACGAGGTGATTTGAATTTTGTCAACAGAGCGAATCAGCGGCTAAATCCTATG

CATCAACTTCTGAGGCATTTCCAAAAGGATGCAAAAGTGCTGTTTCAAAACTGGGGGATT

GAACCAATTGACAATGTAATGGGGATGATCGGGATACTGCCTGACATGACCCCCAGCACA

GAGATGTCACTGAGAGGAGTGAGAGTCAGCAAAATGGGAGTGGATGAATATTCCAGTACT

GAGAGAGTGGTCGTGAGCATTGATCGCTTCTTGAGAGTCCGAGATCAGAGGGGAAATGTG

CTTCTGTCCCCTGAGGAAGTTAGTGAAACACATGGAACAGAGAAACTGACGATAACGTAT

TCATCGTCTATGATGTGGGAAATCAATGGTCCGGAATCCGTGCTAGTCAACACATATCAA

TGGATCATTAGAAATTGGGAAACTGTGAAGATTCAGTGGTCCCAGGACCCTACGATGTTG

TACAATAAGATGGAATTTGAGCCCTTCCAATCCTTGGTGCCCAAGGCTGCTAGGGGCCAG

TATAGTGGGTTTGTGAGGACATTATTCCAACAGATGCGTGATGTGTTGGGGACATTTGAC

ACTGTCCAAATAATAAAGCTCCTACCATTTGCAGCAGCCCCACCGGAACAGAGTAGGATG

CAATTTTCCTCTCTGACTGTGAACGTAAGAGGTTCAGGAATGAGAATACTTGTGAGGGGC

AACTCCCCTGTGTTCAACTATAATAAGGCCACCAAGAGACTCACAGTTCTTGGAAAGGAT

GCAGGCGCCTTGACAGAATATCCAGATGAGGGAACAGCAGGAGTGGAGTCTGCAGTATTA

AGAGGATTTCTAATTCTGGGCAAAGAGGACAAAAGATATGGACCAGCATTGAGCATCAAC

GAATTGAGCAATCTTGCGAAAGGGGAAAAGGCTAATGTGTTGATAGGACAAGGAGACGTG

GTGTTGGTAATGAAACGGAAACGGGACTCTAGCATACTTACTGACAGCCAGACAGCGACC

AAAAGAATTCGGATGGCCATCAATTAGTGTCGAATTGTTTAA------------------

----------------------

>A_mute_swan_Czech_Republic_1656-1_2021_EPI1850125

------------------------AATATGGAGAGAATAAAAGAACTAAGAGATTTGATG

TCACAGTCTCGCACTCGCGAGATACTAACAAAAACCACTGTGGACCATATGGCCATAATA

AAGAAATACACATCAGGGAGACAGGAGAAGAACCCTGCCCTCAGGATGAAATGGATGATG

GCAATGAAATATCCTATTACAGCTGACAAAAGAATAATGGAGATGATCCCTGAAAGGAAT

GAGCAAGGTCAGACTCTCTGGAGCAAAACAAATGATGCTGGATCAGACAGAGTGATGGTC

TCACCTCTGGCTGTGACATGGTGGAATAGAAACGGGCCAACAACAAGTACAGTACACTAC

CCAAAAGTCTACAAAACCTACTTTGAAAAGGTGGAAAGGTTGAAACATGGAACCTTTGGT

CCTGTTCACTTTCGAAATCAGGTTAAGATACGCCGCAGGGTTGACATAAACCCGGGCCAT

GCAGATCTCAGTGCCAAAGAAGCACAGGATGTCATCATGGAGGTTGTTTTCCCAAATGAA

GTTGGAGCCAGGATCTTGACATCAGAATCACAATTAACAATAACAAGGGAAAAGAAGGAG

GAACTTCAGGATTGCAAGATTGCTCCTTTGATGGTGGCATACATGTTGGAAAGAGAACTG

GTTCGCAAGACCAGATTTTTACCAGTAGCTGGCGGAACAAGCAGCGTGTACATCGAGGTA

TTGCATTTGACTCAAGGGACCTGCTGGGAACAAATGTACACACCAGGAGGGGAGGTGAGA

AATGATGATGTTGATCAGAGTTTGATCATTGCTGCTAGAAATATAGTTAGGAGGGCAACA

GTATCAGCAGACCCATTGGCTTCGCTCTTGGAAATGTGCCACAGTACACAAATTGGTGGA

GTGAGGATGGTGGACATTCTTAGGCAGAACCCAACAGAGGAGCAAGCTGTGGATATATGC

AAAGCAGCAATGGGTTTAAGAATCAGTTCATCCTTCAGCTTTGGAGGTTTCACTTTCAAA

AGGACAAGTGGGTCGTCTGTCAAAAGAGAAGAAGAAATACTCACTGGCAACCTCCAAACA

CTGAAAGTAAGAATACATGAAGGATATGAGGAATTCACAATGGTTGGGCGAAGAGCTACA

GCCATTTTGAGGAAAGCAACCAGGAGACTGATCCAATTAATAGTGAGTGGACGAGACGAG

CAGTCAATCGCTGAAGCAATCATAGTGGCAATGGTTTTCTCACAGGAGGATTGCATGATA

AAAGCAGTACGAGGTGATTTGAATTTTGTCAACAGAGCGAATCAGCGGCTAAATCCTATG

CATCAACTTCTGAGGCATTTCCAAAAGGATGCAAAAGTGCTGTTTCAAAACTGGGGGATT

GAACCAATTGACAATGTAATGGGGATGATCGGGATACTGCCTGACATGACCCCCAGCACA

GAGATGTCACTGAGAGGAGTGAGAGTCAGCAAAATGGGAGTGGATGAATATTCCAGTACT

GAGAGAGTGGTCGTGAGCATTGATCGCTTCTTGAGAGTCCGAGATCAGAGGGGAAATGTG

CTTCTGTCTCCTGAGGAAGTTAGTGAAACACATGGAACAGAGAAACTGACGATAACGTAT

TCATCGTCTATGATGTGGGAAATCAATGGTCCGGAATCCGTGCTAGTCAACACATATCAA

TGGATCATTAGAAATTGGGAAACTGTGAAGATTCAGTGGTCCCAGGACCCTACGATGTTG

TACAATAAGATGGAATTTGAGCCCTTTCAATCCTTGGTGCCCAAGGCTGCTAGAGGCCAG

TATAGTGGGTTTGTGAGGACATTATTCCAACAGATGCGTGATGTGTTGGGGACATTTGAC

ACTGTCCAAATAATAAAGCTCCTACCATTTGCAGCAGCCCCACCGGAACAGAGTAGGATG

CAATTTTCCTCTCTGACTGTGAACGTAAGAGGTTCCGGAATGAGAATACTTGTGAGGGGC

AACTCCCCTGTGTTCAACTATAATAAGGCCACCAAGAGACTCACAGTTCTTGGAAAGGAT

GCAGGCGCCTTGACAGAATATCCAGATGAGGGAACAGCAGGAGTGGAGTCTGCAGTATTA

AGAGGATTTCTAATTCTGGGCAGAGAGGACAAAAGATATGGACCAGCATTGAGCATCAAC

GAATTGAGCAATCTTGCGAAAGGGGAAAAGGCTAATGTGTTGATAGGACAAGGAGACGTG

GTGTTGGTAATGAAACGGAAACGGGACTCTAGCATACTTACTGACAGCCAGACAGCGACC

AAAAGAATTCGGATGGCCATCAATTAGTGTCGAATTGTTTAA------------------

----------------------

>A_duck_Korea_H016_2021_EPI1850686

---------------------------ATGGAGAGAATAAAAGAACTAAGAGATTTGATG

TCGCAGTCTCGCACTCGCGAGATACTAACAAAAACCACTGTGGACCATATGGCCATAATA

AAGAAATACACATCAGGAAGACAGGAGAAGAACCCTGCCCTCAGGATGAAATGGATGATG

GCAATGAAATATCCTATTACAGCTGATAAAAGAATAATGGAGATGATCCCTGAAAGGAAT

GAGCAAGGTCAGACTCTCTGGAGCAAAACAAATGATGCTGGATCAGACAGAGTGATGGTC

TCACCTCTGGCTGTGACATGGTGGAATAGGAATGGGCCAACAACAAGTACAGTACACTAC

CCAAAAGTCTACAAAACCTACTTTGAAAAGGTAGAAAGGTTGAAACATGGAACCTTTGGT

CCTGTTCACTTTCGAAATCAGGTTAAGATACGCCGCAGGGTTGACATAAACCCGGGCCAT

GCAGATCTCAGTGCCAAAGAAGCACAGGATGTCATCATGGAGGTTGTTTTCCCAAATGAA

GTTGGAGCCAGGATCTTGACATCAGAATCACAATTAACAATAACAAGGGAAAAGAAGGAG

GAACTTCAGGATTGCAAGATTGCTCCTTTGATGGTGGCATACATGTTGGAAAGAGAACTG

GTTCGCAAGACCAGATTTTTACCAGTAGCTGGCGGAACAAGCAGCGTATACATCGAGGTA

TTGCATTTGACTCAAGGGACCTGCTGGGAACAAATGTACACACCAGGAGGGGAGGTGAGA

AATGATGATGTTGATCAGAGTTTGATCATTGCTGCTAGAAATATAGTTAGGAGGGCAACA

GTATCAGCAGACCCATTGGCTTCGCTCTTGGAAATGTGCCACAGTACACAAATTGGTGGA

GTAAGGATGGTGGACATTCTTAGGCAGAACCCAACAGAGGAGCAAGCTGTGGATATATGC

AAAGCAGCAATGGGTTTAAGAATCAGTTCATCCTTCAGCTTTGGAGGTTTCACTTTCAAA

AGGACAAGTGGGTCGTCTGTCAAAAGAGAAGAAGAAATACTCACTGGCAACCTCCAAACA

CTGAAAGTTAGAATACATGAAGGATATGAGGAATTCACAATGGTTGGGCGAAGAGCTACA

GCCATTTTGAGGAAAGCAACCAGGAGACTGATCCAATTAATAGTGAGTGGAAGAGACGAG

CAGTCAATCGCTGAAGCAATCATAGTGGCAATGGTTTTCTCACAGGAGGATTGCATGATA

AAAGCAGTACGAGGTGATTTGAATTTTGTCAACAGAGCGAATCAGCGGCTAAATCCTATG

CATCAACTTCTGAGGCATTTCCAAAAGGATGCAAAAGTGCTGTTTCAAAACTGGGGGATT

GAACCAATTGACAATGTAATGGGGATGATCGGGATACTGCCAGACATGACCCCCAGCACA

GAGATGTCACTGAGAGGAGTGAGAGTCAGCAAAATGGGAGTGGATGAATATTCCAGTACT

GAGAGAGTGGTCGTGAGCATTGATCGCTTCTTGAGAGTCCGAGATCAGAGGGGAAATGTG

CTTCTGTCTCCTGAGGAAGTTAGTGAAACACATGGAACAGAGAAACTGACGATAACGTAT

TCATCGTCTATGATGTGGGAAATCAATGGTCCGGAATCCGTGTTAGTCAACACATATCAA

TGGATCATTAGAAGTTGGGAAACTGTGAAGATTCAATGGTCCCAGGACCCTACGATGTTG

TACAATAAGATGGAATTTGAGCCCTTCCAATCCTTGGTGCCCAAGGCTGCTAGAGGCCAG

TATAGTGGGTTTGTGAGGACATTATTCCAACAGATGCGTGATGTGTTGGGGACATTTGAC

ACTGTCCAAATAATAAAGCTCCTACCATTTGCAGCAGCCCCACCGGAACAGAGTAGGATG

CAATTTTCCTCTCTGACTGTGAACGTAAGAGGTTCAGGAATGAGAATACTTGTGAGGGGC

AACTCCCCTGTGTTCAACTATAATAAGGCCACCAAGAGACTCACAGTTCTTGGAAAGGAT

GCAGGCGCCTTGACAGAATATCCAGATGAGGGAACAGCAGGAGTGGAGACTGCAGTATTA

AGAGGATTTCTAATTCTGGGCAAAGAGGACAAAAGATATGGACCAGCATTGAGCATCAAC

GAATTGAGCAATCTTGCGAAAGGGGAAAAGGCTAATGTGTTGATAGGGCAAGGAGACGTG

GTGTTGGTAATGAAACGGAAACGGGACTCTAGCATACTTACTGACAGCCAGACAGCGACC

AAAAGAATTCGGATGGCCATCAATTAG---------------------------------

----------------------

>A_mute_swan_Croatia_14_2021_EPI1850959

------------TCAAATATATTCAATATGGAGAGAATAAAAGAACTAAGAGATTTGATG

TCGCAGTCTCGCACTCGCGAGATACTAACAAAAACCACTGTGGACCATATGGCCATAATA

AAGAAATACACATCAGGAAGACAGGAGAAGAACCCTGCCCTCAGGATGAAATGGATGATG

GCAATGAAATATCCTATTACAGCTGACAAAAGAATAATGGAGATGATCCCTGAAAGGAAT

GAGCAAGGTCAGACTCTCTGGAGCAAAACAAATGATGCTGGATCAGACAGAGTGATGGTC

TCACCTCTGGCTGTGACATGGTGGAATAGAAATGGGCCAACAACAAGTACAGTACACTAC

CCAAAAGTCTACAAAACCTACTTTGAAAAGGTAGAAAGGTTGAAACATGGAACCTTTGGT

CCTGTTCACTTTCGAAATCAGGTTAAGATACGCCGCAGGGTTGACATAAACCCGGGCCAT

GCAGATCTCAGTGCCAAAGAAGCACAGGATGTCATCATGGAGGTTGTTTTCCCAAATGAA

GTTGGAGCCAGGATCTTGACATCAGAATCACAATTAACAATAACAAGGGAAAAGAAGGAG

GAACTTCAGGATTGCAAGATTGCTCCTTTGATGGTGGCATACATGTTGGAAAGAGAACTG

GTTCGCAAGACCAGATTTTTACCAGTAGCTGGCGGAACAAGCAGCGTATACATCGAGGTA

TTGCATTTGACTCAAGGGACCTGCTGGGAACAAATGTACACACCAGGAGGGGAGGTGAGA

AATGATGATGTTGATCAGAGTTTGATCATTGCTGCTAGAAATATAGTTAGGAGGGCAACA

GTATCAGCAGACCCATTGGCTTCGCTTTTGGAAATGTGCCACAGTACACAAATTGGTGGA

GTAAGGATGGTGGACATTCTTAGGCAGAACCCAACAGAGGAGCAAGCTGTGGATATATGC

AAAGCAGCAATGGGTTTAAGAATCAGTTCATCCTTCAGCTTTGGAGGTTTCACTTTCAAA

AGGACAAGTGGGTCGTCTGTCAAAAGAGAAGAAGAAATACTCACTGGCAACCTCCAAACA

CTGAAAGTAAGAATACATGAAGGATATGAGGAATTCACAATGGTTGGGCGAAGAGCTACA

GCCATTTTGAGGAAAGCAACCAGGAGACTGATCCAATTAATAGTGAGTGGAAGAGACGAG

CAGTCAATCGCTGAAGCAATCATAGTGGCAATGGTTTTCTCACAGGAGGATTGCATGATA

AAAGCAGTACGAGGTGATTTGAATTTTGTCAACAGAGCGAATCAGCGGCTAAATCCTATG

CATCAACTTCTGAGGCATTTCCAAAAGGATGCAAAAGTGCTGTTTCAAAACTGGGGGATT

GAACCAATTGACAATGTAATGGGGATGATCGGGATACTGCCTGACATGACCCCCAGCACA

GAGATGTCACTGAGAGGAGTGAGAGTCAGCAAAATGGGAGTGGATGAATATTCCAGTACT

GAGAGAGTAGTCGTGAGCATTGATCGCTTCTTGAGAGTCCGAGATCAGAGGGGAAATGTG

CTTTTGTCTCCTGAGGAAGTTAGTGAAACACATGGAACAGAGAAACTGACGATAACGTAT

TCATCGTCTATGATGTGGGAAATCAATGGTCCGGAATCCGTGCTAGTCAACACATATCAA

TGGATCATTAGAAATTGGGAAACTGTGAAGATTCAGTGGTCCCAGGACCCTACGATGTTA

TACAATAAGATGGAATTTGAGCCCTTCCAATCCTTGGTGCCCAAGGCTGCTAGAGGCCAG

TATAGTGGGTTTGTGAGGACATTATTCCAACAGATGCGTGATGTGTTGGGGACATTTGAC

ACTGTCCAAATAATAAAGCTCCTACCATTTGCAGCAGCCCCACCGGAACAGAGTAGGATG

CAATTTTCCTCTCTGACTGTGAACGTAAGAGGTTCAGGAATGAGAATACTTGTGAGGGGC

AACTCCCCTGTGTTCAACTATAATAAGGCCACCAAGAGACTCACAGTTCTTGGAAAGGAT

GCAGGCGCCTTGACAGAATATCCAGATGAGGGAACAGCAGGAGTGGAGTCTGCAGTATTA

AGAGGATTTCTAATTCTGGGCAAAGAGGACAAAAGATATGGACCAGCATTGAGCATCAAC

GAATTGAGCAATCTTGCGAAAGGGGAAAAGGCTAATGTGTTGATAGGACAAGGAGACGTG

GTGTTGGTAATGAAACGGAAACGGGACTCTAGCATACTTACTGACAGCCAGACAGCGACC

AAAAGAATTCGGATGGCCATCAATTAGTGTCGAA--------------------------

----------------------

>A_chicken_Vietnam_Raho4-Cd-20-421_2020_EPI1853935

------------TCAAATATATTCAATATGGAAAGAATAAAAGAACTAAGAGATTTGATG

TCACAGTCTCGCACCCGCGAGATACTGACAAAAACCACTGTGGACCATATGGCCATAATA

AAGAAATACACATCGGGAAGACAGGAGAAGAACCCTGCCCTCAGGATGAAATGGATGATG

GCAATGAAATATCCGATTACAGCAGACAGAAGAATAATGGAGATGATCCCTGAAAAAAAT

GAGCAAGGTCAAATCCTTTGGAGCAAAACAAATGATGCTGGATCAGACAGGGTGATGGTG

TCACCTTTGGCAGTGACATGGTGGAACAGAAATGGGCCAGCTACAAGCACAGTCCACTAC

CCAAAGGTCTACAAAACCTATTTTGAAAAGGTTGAAAGGTTGAAACATGGAACCTTCGGT

CCTGTTCACTTTCGAAATCAAGTCAAAATACGCCGCAGGGTTGACATAAACCCAGGTCAT

GCAGACCTCAGTGCCAAAGAAGCACAAGATGTCATCATGGAGGTCGTTTTCCCAAATGAA

GTTGGAGCCAAAATATTGACGTCAGAATCACAGTTAACAATAACAAGGGAAAAGAAGGAA

GAGCTCCAGGACTGTAAAATTGCTCCTTTGATGGTGGCATACATGTTGGAGAGAGAACTG

GTTCGCAAAACAAGATTCCTACCAGTAGCTGGCGGAACGAGCAGTGTGTATATCGAGGTG

TTGCATTTAACTCAAGGGACCTGCTGGGAACAAATGTATACACCAGGAGGAGAGGTTAGA

AATGATGATGTCGACCAGAGTTTGATCATTGCTGCTAGAAATATTGTTAGGAGAGCAATA

GTGTCAGCAGACCCATTGGCTTCACTTTTGGAGATGTGCCATAGTACGCAAATTGGCGGG

ATAAGGATGATGGACATTCTTAGGCAGAACCCAACAGAAGAGCAGGCTGTGGATATATGC

AAAGCAGCAGTGGGTTTAAGAATTAGTTCATCCTTCAGCTTTGGGGGTTTCACTTTTAAG

AGGACAAGTGGATCATCCGTCAAAAAAGAAGAAGAAGTGCTCACAGGCAACCTCCAAACA

CTGAAAATAAGAGTGCATGAAGGATATGAAGAATTCACAATGGTTGGGCGAAGAGCTACA

GCCATTCTAAGGAAAGCAACCAGAAGGCTGATCCAATTGATAGTGAGTGGGAGAGACGAG

CATTCAATCGCCGAAGCAATCATCGTGGCAATGGTTTTCTCGCAGGAGGATTGCATGATA

AAAGCAGTGCGAGGCGATTTGAATTTTGTCAACAGAGCGAATCAGCGGCTAAATCCTATG

CATCAACTTCTGAGACATTTTCAAAAGGATGCAAAAGTGCTGTTTCAAAACTGGGGGATA

GAGCCAATTGACAATGTTATGGGGATAATCGGAATATTGCCTGACATGACCCCCAGCACA

GAGATGTCACTAAGAGGAGTGAGAGTCAGTAAAATGGGGGTGGATGAGTATTCCAGTACT

GAAAAGGTGGTCGTGAGCATTGATCGTTTCTTGAGAGTTCGGGACCAGAGGGGAAACGTG

CTCTTGTCCCCTGAGGAAGTCAGTGAGACGCAGGGAACAGAGAAACTGACGATAACATAT

TCGTCGTCCATGATGTGGGAAATCAATGGTCCGGAGTCAGTGCTAGTCAACACATATCAA

TGGATCATTAGGAACTGGGAAACTGTAAAGATTCAATGGTCCCAAGACCCTACAATATTG

TACAATAAGATGGAGTTTGAGCCCTTCCAATCCTTGGTGCCCAAGGCTGCCAGGGGCCAG

TATAGTGGGTTTGTGAGAGCATTATTCCAGCAGATGCGTGATGTGCTGGGGACATTTGAC

ACTGTCCAAATAATAAAGCTCCTGCCATTCGCAGCAGCCCCACCGGAACAGAGTAGGATG

CAGTTCTCCTCTCTGACTGTGAATGTAAGAGGATCGGGAATGAGAATACTTGTGAGAGGC

AACTCCCCTGTGTTCAACTATAACAAGGCAACCAAGAGACTCACAATTCTTGGAAAGGAT

GCAGGTGCCTTGACAAAAGATCCAGATGAAGGAACGGCAGGAGTGGAATCTGCGGTATTA

AGAGGATTTCTGATTCTGGGCAAAGAAGACAAAAGATATGGACCAGCATTGAGCATCAAT

GAATTGAGCAATCTCGCGAAAGGAGAAAAAGCTAATGTTCTGATAGGGCAAGGAGACGTG

GTGTTGGTAATGAAACGGAAACGGGACTCTAGCATACTTACTGACAGCCAGACAGCGACC

AAAAGAATTCGGATGGCCATCAATTAGTGTCGAATTATTTAAAAACGA------------

----------------------

>A_chicken_Czech_Republic_3531-1_2021_EPI1854240

------------------------AATATGGAGAGAATAAAAGAACTAAGAGATTTGATG

TCGCAGTCTCGCACTCGCGAGATACTAACAAAAACCACTGTGGACCATATGGCCATAATA

AAGAAATACACATCAGGAAGACAGGAGAAGAACCCTGCCCTCAGGATGAAATGGATGATG

GCAATGAAATATCCTATTACAGCTGACAAAAGAATAATGGAGATGATCCCTGAAAGGAAT

GAGCAAGGTCAGACTCTCTGGAGCAAAACAAATGATGCTGGATCAGACAGAGTGATGGTC

TCACCTCTGGCTGTGACATGGTGGAATAGAAATGGGCCAACAACAAGTACAGTACACTAC

CCAAAAGTCTACAAAACCTACTTTGAAAAGGTAGAAAGGTTGAAACATGGAACCTTTGGT

CCTGTTCACTTTCGAAATCAGGTTAAGATACGCCGCAGGGTTGACATAAACCCGGGCCAT

GCAGATCTCAGTGCCAAAGAAGCACAGGATGTCATCATGGAGGTTGTTTTCCCAAATGAA

GTTGGAGCCAGGATCTTGACATCAGAATCACAATTAACAATAACAAGGGAAAAGAAGGAG

GAACTTCAGGATTGCAAGATTGCTCCTTTGATGGTGGCATACATGTTGGAAAGAGAACTG

GTTCGCAAGACCAGATTTTTACCAGTAGCTGGCGGAACAAGCAGCGTATACATCGAGGTA

TTGCATTTGACTCAAGGGACCTGCTGGGAACAAATGTACACACCAGGAGGGGAGGTGAGA

AATGATGATGTTGATCAGAGTTTGATCATTGCTGCTAGAAATGTAGTTAGGAGGGCAACA

GTATCAGCAGACCCATTGGCTTCGCTTTTGGAAATGTGCCACAGTACACAAATTGGTGGA

GTAAGGATGGTGGACATTCTTAGGCAGAACCCAACAGAGGAGCAAGCTGTGGATATATGC

AAAGCAGCAATGGGTTTAAGAATCAGTTCATCCTTCAGCTTTGGAGGTTTCACTTTCAAA

AGGACAAGTGGGTCGTCTGTCAAAAGAGAAGAAGAAATACTCACTGGCAACCTCCAAACA

CTGAAAGTAAGAATACATGAAGGATATGAGGAATTCACAATGGTTGGGCGAAGAGCTACA

GCCATTTTGAGGAAAGCAACCAGGAGACTGATCCAATTAATAGTGAGTGGAAGAGACGAG

CAGTCAATCGCTGAAGCAATCATAGTGGCAATGGTTTTCTCACAGGAGGATTGCATGATA

AAAGCAGTACGAGGTGATTTGAATTTTGTCAACAGAGCGAATCAGCGGCTAAATCCTATG

CATCAACTTCTGAGGCATTTCCAAAAGGATGCAAAAGTGCTGTTTCAAAACTGGGGGATT

GAACCAATTGACAATGTAATGGGGATGATCGGGATACTGCCTGACATGACCCCCAGCACA

GAGATGTCACTGAGAGGAGTGAGAGTCAGCAAAATGGGAGTGGATGAATATTCCAGTACT

GAGAGAGTGGTCGTGAGCATTGATCGCTTCTTGAGAGTCCGAGATCAGAGGGGAAATGTG

CTTCTGTCTCCTGAGGAAGTTAGTGAAACACATGGAACAGAGAAACTGACGATAACGTAT

TCATCGTCTATGATGTGGGAAATCAATGGCCCGGAATCCGTGCTAGTCAACACATATCAA

TGGATCATTAGAAATTGGGAAACTGTGAAGATTCAGTGGTCCCAGGACCCTACGATGTTG

TACAATAAGATGGAATTTGAGCCCTTCCAATCCTTGGTGCCCAAGGCTGCTAGAGGCCAG

TATAGTGGGTTTGTGAGGACATTATTCCAACAGATGCGTGATGTGTTGGGGACATTTGAC

ACTGTCCAAATAATAAAGCTCCTACCATTTGCAGCAGCCCCACCGGAACAGAGTAGGATG

CAATTTTCCTCTCTGACTGTGAACGTAAGAGGTTCAGGAATGAGAATACTTGTGAGGGGC

AATTCCCCTGTGTTCAACTATAATAAGGCCACCAAGAGACTAACAGTTCTTGGAAAGGAT

GCAGGCGCCTTGACAGAATATCCAGATGAGGGAACAGCAGGAGTGGAGTCTGCAGTATTA

AGAGGATTTCTAATTCTGGGCAAAGAGGACAAAAGATATGGACCAGCATTGAGCATCAAC

GAATTGAGCAATCTTGCGAAAGGGGAAAAGGCTAATGTGTTRATAGGACAAGGAGACGTG

GTGTTGGTAATGAAACGGAAACGGGACTCTAGCATACTTACTGACAGCCAGACAGCGACC

AAAAGAATTCGGATGGCCATCAATTAGTGTCGAATTGTTTAA------------------

----------------------

>A_wigeon_Latvia_23903_2021_EPI1855972

--------------AAATATATTCAATATGGAGAGAATAAAAGAACTAAGAGATTTGATG

TCACAGTCTCGCACTCGCGAGATACTAACAAAAACCACTGTGGACCATATGGCCATAATA

AAGAAATACACATCAGGGAGACAGGAGAAGAACCCTGCCCTCAGGATGAAATGGATGATG

GCAATGAAATATCCTATTACAGCTGACAAAAGAATAATGGAGATGATCCCTGAAAGGAAT

GAGCAAGGTCAGACTCTCTGGAGCAAAACAAATGATGCTGGATCAGACAGAGTGATGGTC

TCACCTCTGGCTGTGACATGGTGGAATAGAAACGGGCCAACAACAAGTACAGTACACTAC

CCAAAAGTCTACAAAACCTACTTTGAAAAGGTGGAAAGGTTGAAACATGGAACCTTTGGT

CCTGTTCACTTTCGAAATCAGGTTAAGATACGCCGCAGGGTTGACATAAACCCGGGCCAT

GCAGATCTCAGTGCCAAAGAAGCACAGGATGTCATCATGGAGGTTGTTTTCCCAAATGAA

GTTGGAGCCAGGATCTTGACATCAGAATCACAATTAACAATAACAAGGGAAAAGAAGGAG

GAACTTCAGGATTGCAAGATTGCTCCTTTGATGGTGGCATACATGTTGGAAAGAGAACTG

GTTCGCAAGACCAGATTTTTACCAGTAGCTGGCGGAACAAGCAGCGTGTACATCGAGGTA

TTGCATTTGACTCAAGGGACCTGCTGGGAACAAATGTACACACCAGGAGGGGAGGTGAGA

AATGATGATGTTGATCAGAGTTTGATCATTGCTGCTAGAAATATAGTTAGGAGGGCAACA

GTATCAGCAGACCCATTGGCTTCGCTCTTGGAAATGTGCCACAGTACACAAATTGGTGGA

GTGAGGATGGTGGACATTCTTAGGCAGAACCCAACAGAGGAGCAAGCTGTGGATATATGC

AAAGCAGCAATGGGTTTAAGAATCAGTTCATCCTTCAGCTTTGGAGGTTTCACTTTCAAA

AGGACAAGTGGGTCGTCTGTCAAAAGAGAAGAAGAAATACTCACTGGCAACCTCCAAACA

CTGAAAGTAAGAATACATGAAGGATATGAGGAATTCACAATGGTTGGGCGAAGAGCTACA

GCCATTTTGAGGAAAGCAACCAGGAGACTGATCCAATTAATAGTGAGTGGACGAGACGAG

CAGTCAATCGCTGAAGCAATCATAGTGGCAATGGTTTTCTCACAGGAGGATTGCATGATA

AAAGCAGTACGAGGTGATTTGAATTTTGTCAACAGAGCGAATCAGCGGCTAAATCCTATG

CATCAACTTCTGAGGCATTTCCAAAAGGATGCAAAAGTGCTGTTTCAAAACTGGGGGATT

GAACCAATTGACAATGTAATGGGGATGATCGGGATACTGCCTGACATGACCCCCAGCACA

GAGATGTCACTGAGAGGAGTGAGAGTCAGCAAAATGGGAGTGGATGAATATTCCAGTACT

GAGAGAGTGGTCGTGAGCATTGATCGCTTCTTGAGAGTCCGAGATCAGAGGGGAAATGTG

CTTCTGTCTCCTGAGGAAGTTAGTGAAACACATGGAACAGAGAAACTGACGATAACGTAT

TCATCGTCTATGATGTGGGAAATCAATGGTCCGGAATCCGTGCTAGTCAACACATATCAA

TGGATCATTAGAAATTGGGAAACTGTGAAGATTCAGTGGTCCCAGGACCCTACGATGTTG

TACAATAAGATGGAATTTGAGCCCTTTCAATCCTTGGTGCCCAAGGCTGCTAGAGGCCAG

TATAGTGGGTTTGTGAGGACATTATTCCAACAGATGCGTGATGTGTTGGGGACATTTGAC

ACTGTCCAAATAATAAAGCTCCTACCATTTGCAGCAGCCCCACCGGAACAGAGTAGGATG

CAATTTTCCTCTCTGACTGTGAACGTAAGAGGTTCAGGAATGAGAATACTTGTGAGGGGC

AACTCCCCTGTGTTCAACTATAATAAGGCCACCAAGAGACTCACAGTTCTTGGAAAGGAT

GCAGGCGCCTTGACAGAATATCCAGATGAGGGAACAGCAGGAGTGGAGTCTGCAGTATTA

AGAGGATTTCTAATTCTGGGCAAAGAGGACAAAAGATATGGACCAGCATTGAGCATCAAC

GAATTGAGCAATCTTGCGAAAGGGGAAAAGGCTAATGTGTTGATAGGACAAGGAGACGTG

GTATTGGTAATGAAACGGAAACGGGACTCTAGCATACTTACTGACAGCCAGACAGCGACC

AAAAGAATTCGGATGGCCATCAATTAGTGTCGAATTGTTTAA------------------

----------------------

>A_chicken_Czech_Republic_4980_2021_EPI1858497

------------------------AATATGGAGAGAATAAAAGAACTAAGAGATTTGATG

TCGCAGTCTCGCACTCGCGAGATACTAACAAAAACCACTGTGGACCATATGGCCATAATA

AAGAAATACACATCAGGAAGACAGGAGAAGAACCCTGCCCTCAGGATGAAATGGATGATG

GCAATGAAATATCCTATTACAGCTGACAAAAGAATAATGGAGATGATCCCTGAAAGGAAT

GAGCAAGGTCAGACTCTCTGGAGCAAAACAAATGATGCTGGATCAGACAGAGTGATGGTC

TCACCTCTGGCTGTGACATGGTGGAATAGAAATGGGCCAACAACAAGTACAGTACACTAC

CCAAAAGTCTACAAAACCTACTTTGAAAAGGTAGAAAGGTTGAAACATGGAACCTTTGGT

CCTGTTCACTTTCGAAATCAGGTTAAGATACGCCGCAGGGTTGACATAAACCCGGGCCAT

GCAGATCTCAGTGCCAAAGAAGCACAGGATGTCATCATGGAGGTTGTTTTCCCAAATGAA

GTTGGAGCCAGGATCTTGACATCAGAATCACAATTAACAATAACAAGGGAAAAGAAGGAG

GAACTTCAGGATTGCAATATTGCTCCTTTGATGGTGGCATACATGTTGGAAAGAGAACTG

GTTCGCAAGACCAGATTTTTACCAGTAGCTGGCGGAACAAGCAGCGTATACATCGAGGTA

TTGCATTTGACTCAAGGGACCTGCTGGGAACAAATGTACACACCAGGAGGGGAGGTGAGA

AATGATGATGTTGATCAGAGTTTGATCATTGCTGCTAGAAATATAGTTAGGAGGGCAACA

GTATCAGCAGACCCATTGGCTTCGCTTTTGGAAATGTGCCACAGTACACAAATTGGTGGA

GTAAGGATGGTGGACATTCTTAGGCAGAACCCAACAGAGGAGCAAGCTGTGGATATATGC

AAAGCAGCAATGGGTTTAAGAATCAGTTCATCCTTCAGCTTTGGAGGTTTCACTTTCAAA

AGGACAAGTGGGTCGTCTGTCAAAAGAGAAGAAGAAATACTCACTGGCAACCTCCAAACA

CTGAAAGTAAGAATACATGAAGGATATGAGGAATTCACAATGGTTGGGCGAAGAGCTACA

GCCATTTTGAGGAAAGCAACCAGGAGACTGATCCAATTAATAGTGAGTGGAAGAGACGAG

CAGTCAATCGCTGAAGCAATCATAGTGGCAATGGTTTTCTCACAGGAGGATTGCATGATA

AAAGCAGTACGAGGTGATTTGAATTTTGTCAACAGAGCGAATCAGCGGCTAAATCCTATG

CATCAACTTCTGAGGCATTTCCAAAAGGATGCAAAAGTGCTGTTTCAAAACTGGGGGATT

GAACCAATTGACAATGTAATGGGGATGATCGGGATAATGCCTGACATGACCCCCAGCACA

GAGATGTCACTGAGAGGAGTGAGAGTCAGCAAAATGGGAGTGGATGAATATTCCAGTACT

GAGAGAGTGGTCGTGAGCATTGATCGCTTCTTGAGAGTCCGAGATCAGAGGGGAAATGTG

CTTCTGTCTCCTGAGGAAGTTAGTGAAACACATGGAACAGAGAAACTGACGATAACGTAT

TCATCGTCTATGATGTGGGAAATCAATGGTCCGGAATCCGTGCTAGTCAACACATATCAA

TGGATCATTAGAAATTGGGAAACTGTGAAGATTCAGTGGTCCCAGGACCCTACGATGTTA

TACAATAAGATGGAATTTGAGCCCTTCCAATCCTTGGTGCCCAAGGCTGCTAGAGGCCAG

TATAGTGGGTTTGTGAGGACATTATTCCAACAGATGCGTGATGTGTTGGGAACATTTGAC

ACTGTCCAAATAATAAAGCTCCTACCATTTGCAGCAGCCCCACCGGAACAGAGTAGGATG

CAATTTTCCTCTCTGACTGTGAACGTAAGAGGTTCAGGAATGAGAATACTTGTGAGGGGC

AACTCCCCTGTGTTCAACTATAATAAGGCCACCAAGAGACTCACAGTTCTTGGAAAGGAT

GCAGGCGCCTTGACAGAATATCCAGATGAGGGAACAGCAGGAGTGGAGTCTGCAGTATTA

AGAGGATTTCTAATTCTGGGCAAAGAGGACAAAAGATATGGACCAGCATTGAGCATCAAC

GAATTGAGCAATCTTGTGAAAGGGGAAAAGGCTAATGTGTTGATAGGACAAGGAGACGTG

GTGTTGGTAATGAAACGGAAACGGGACTCTAGCATACTTACTGACAGCCAGACAGCGACC

AAAAGAATTCGGATGGCCATCAATTAGTGTCGAATTGTTTAA------------------

----------------------

>A_swan_Lithuania_1258PG1_21VIR2606-2_2021_EPI1858571

AGCRAAAGCAGGTCAAATATATTCAATATGGAGAGAATAAAAGAACTAAGAGATTTGATG

TCGCAGTCTCGCACTCGCGAGATACTAACAAAAACCACTGTGGACCATATGGCCATAATA

AAGAAATACACATCAGGAAGACAGGAGAAGAACCCTGCCCTCAGGATGAAATGGATGATG

GCAATGAAATATCCTATTACAGCTGACAAAAGAATAATGGAGATGATCCCTGAAAGGAAT

GAGCAAGGTCAGACTCTCTGGAGCAAAACAAATGATGCTGGATCAGACAGAGTGATGGTC

TCACCTCTGGCTGTGACATGGTGGAATAGAAATGGGCCAACAACAAGTACAGTACACTAC

CCAAAAGTCTACAAAACCTACTTTGAAAAGGTAGAAAGGTTGAAACATGGAACCTTTGGT

CCTGTTCACTTTCGAAATCAGGTTAAGATACGCCGCAGGGTTGACATAAACCCGGGCCAT

GCAGATCTCAGTGCCAAAGAAGCACAGGATGTCATCATGGAGGTTGTTTTCCCAAATGAA

GTTGGAGCCAGGATCTTGACATCAGAATCACAATTAACAATAACAAGGGAAAAGAAGGAG

GAACTTCAGGATTGCAAGATTGCTCCTTTGATGGTGGCATACATGTTGGAAAGAGAACTG

GTTCGCAAGACCAGATTTTTACCAGTAGCTGGCGGAACAAGCAGCGTATACATCGAGGTA

TTGCATTTGACTCAAGGGACCTGCTGGGAACAAATGTACACACCAGGAGGGGAGGTGAGA

AATGATGATGTTGATCAGAGTTTGATCATTGCTGCTAGAAATATAGTTAGGAGGGCAACA

GTATCAGCAGACCCATTGGCTTCGCTTTTGGAAATGTGCCACAGTACACAAATTGGTGGA

GTAAGAATGGTGGACATTCTTAGGCAGAACCCAACAGAGGAGCAAGCTGTGGATATATGC

AAAGCAGCAATGGGTTTAAGAATCAGTTCATCCTTCAGCTTTGGAGGTTTCACTTTCAAA

AGGACAAGTGGGTCGTCTGTCAAAAGAGAAGAAGAAATACTCACTGGCAACCTCCAAACA

CTGAAAGTAAGAATACATGAAGGATATGAGGAATTCACAATGGTTGGGCGAAGAGCTACA

GCCATTTTGAGGAAAGCAACCAGGAGACTGATCCAATTAATAGTGAGTGGAAGAGACGAG

CAGTCAATCGCTGAAGCAATCATAGTGGCAATGGTTTTCTCACAGGAGGATTGCATGATA

AAAGCAGTACGAGGTGATTTGAATTTTGTCAACAGAGCGAATCAGCGGCTAAATCCTATG

CATCAACTTCTGAGGCATTTCCAAAAGGATGCAAAAGTGCTGTTTCAAAACTGGGGGATT

GAACCAATTGACAATGTAATGGGGATGATCGGGATACTGCCTGACATGACCCCCAGCACA

GAGATGTCACTGAGAGGAGTGAGAGTCAGCAAAATGGGAGTGGATGAATATTCCAGTACT

GAGAGAGTGGTCGTGAGCATTGATCGCTTCTTGAGAGTCCGAGATCAGAGGGGAAATGTG

CTTCTGTCTCCTGAGGAAGTTAGTGAAACACATGGAACAGAGAAACTGACGATAACGTAT

TCATCGTCTATGATGTGGGAAATCAATGGTCCGGAATCCGTGCTAGTCAACACATATCAA

TGGATCATTAGAAATTGGGAAACTGTGAAGATTCAGTGGTCCCAGGACCCTACGATGTTA

TACAATAAGATGGAATTTGAGCCCTTCCAATCCTTGGTGCCCAAGGCTGCTAGAGGCCAG

TATAGTGGGTTTGTGAGGACATTATTCCAACAGATGCGTGATGTGTTGGGAACATTTGAC

ACTGTCCAAATAATAAAGCTCCTACCATTTGCAGCAGCCCCACCGGAACAGAGTAGGATG

CAATTTTCCTCTCTGACTGTGAACGTAAGAGGTTCAGGAATGAGAATACTTGTGAGGGGC

AACTCCCCTGTGTTCAACTATAATAAGGCCACCAAGAGACTCACAGTTCTTGGAAAGGAT

GCAGGCGCCTTGACAGAATATCCAGATGAGGGAACAGCAGGAGTGGAGTCTGCAGTATTA

AGAGGATTTCTAATTCTGGGCAAAGAGGACAAAAGATATGGACCAGCATTGAGCATCAAC

GAATTGAGCAATCTTGCGAAAGGGGAAAAGGCTAATGTGTTGATAGGACAAGGAGACGTG

GTGTTGGTAATGAAACGGAAACGGGACTCTAGCATACTTACTGACAGCCAGACAGCGACC

AAAAGAATTCGGATGGCCATCAATTAGTGTCGAATTGTTTAAAAACGACCTTGTTTCTAC

T---------------------

>A_swan_Lithuania_1298PG1_21VIR2606-3_2021_EPI1858579

AGCRAAAGCAGGTCAAATATATTCAATATGGAGAGAATAAAAGAACTAAGAGATTTGATG

TCACAGTCTCGCACTCGCGAGATACTAACAAAAACCACTGTGGACCATATGGCCATAATA

AAGAAATACACATCAGGGAGACAGGAGAAGAACCCTGCCCTCAGGATGAAATGGATGATG

GCAATGAAATATCCTATTACAGCTGACAAAAGAATAATGGAGATGATCCCTGAAAGGAAT

GAGCAAGGTCAGACTCTCTGGAGCAAAACAAATGATGCTGGATCAGACAGAGTGATGGTC

TCACCTCTGGCTGTGACATGGTGGAATAGAAACGGGCCAACAACAAGTACAGTACACTAC

CCAAAAGTCTACAAAACCTACTTTGAAAAGGTGGAAAGGTTGAAACATGGAACCTTTGGT

CCTGTTCACTTTCGAAATCAGGTTAAGATACGCCGCAGGGTTGACATAAACCCGGGCCAT

GCAGATCTCAGTGCCAAAGAAGCACAGGATGTCATCATGGAGGTTGTTTTCCCAAATGAA

GTTGGAGCCAGGATCTTGACATCAGAATCACAATTAACAATAACAAGGGAAAAGAAGGAG

GAACTTCAGGATTGCAAGATTGCTCCTTTGATGGTGGCATACATGTTGGAAAGAGAACTG

GTTCGCAAGACCAGATTTTTACCAGTAGCTGGCGGAACAAGCAGCGTGTACATCGAGGTA

TTGCATTTGACTCAAGGGACCTGCTGGGAACAAATGTACACACCAGGAGGGGAGGTGAGA

AATGATGATGTTGATCAGAGTTTGATCGTTGCTGCTAGAAATATAGTTAGGAGGGCAACA

GTATCAGCAGACCCATTGGCTTCGCTCTTGGAAATGTGCCACAGTACACAAATTGGTGGA

GTGAGGATGGTGGACATTCTTAGGCAGAACCCAACAGAGGAGCAAGCTGTGGATATATGC

AAAGCAGCAATGGGTTTAAGAATCAGTTCATCCTTCAGCTTTGGAGGTTTCACTTTCAAA

AGGACAAGTGGGTCGTCTGTCAAAAGAGAAGAAGAAATACTCACTGGCAACCTCCAAACA

CTGAAAGTAAGAATACATGAAGGATATGAGGAATTCACAATGGTTGGGCGAAGAGCTACA

GCCATTTTGAGGAAAGCAACCAGGAGACTGATCCAATTAATAGTGAGCGGACGAGACGAG

CAGTCAATCGCTGAAGCAATCATAGTGGCAATGGTTTTCTCACAGGAGGATTGCATGATA

AAAGCAGTACGAGGTGATTTGAATTTTGTCAACAGAGCGAATCAGCGGCTAAATCCTATG

CATCAACTTCTGAGGCATTTCCAAAAGGATGCAAAAGTGCTGTTTCAAAACTGGGGGATT

GAACCAATTGACAATGTAATGGGGATGATCGGGATACTGCCTGACATGACCCCCAGCACA

GAGATGTCACTGAGAGGAGTGAGAGTCAGCAAAATGGGAGTGGATGAATATTCCAGTACT

GAGAGAGTGGTCGTGAGCATTGATCGCTTCTTGAGAGTCCGAGATCAGAGGGGAAATGTG

CTTCTGTCTCCTGAGGAAGTTAGTGAAACACATGGAACAGAGAAACTGACGATAACGTAT

TCATCGTCTATGATGTGGGAAATCAATGGTCCGGAATCCGTGCTAGTCAACACATATCAA

TGGATCATTAGAAATTGGGAAACTGTGAAGATTCAGTGGTCCCAGGACCCTACGATGTTG

TACAATAAGATGGAATTTGAGCCCTTTCAATCCTTGGTGCCCAAGGCTGCTAGAGGCCAG

TATAGTGGGTTTGTGAGGACATTATTCCAACAGATGCGTGATGTGTTGGGGACATTTGAC

ACTGTCCAAATAATAAAGCTCCTACCATTTGCAGCAGCCCCACCGGAACAGAGTAGGATG

CAATTTTCCTCTCTGACTGTGAACGTAAGAGGTTCAGGAATGAGAATACTTGTGAGGGGC

AACTCCCCTGTGTTCAACTATAATAAGGCCACCAAGAGACTCACAGTTCTTGGAAAGGAT

GCAGGCGCCTTGACAGAATATCCAGATGAGGGAACAGCAGGAGTGGAGTCTGCAGTATTA

AGAGGATTTCTAATTCTGGGCAAAGAGGACAAAAGATATGGACCAGCATTGAGCATCAAC

GAATTGAGCAATCTTGCGAAAGGGGAAAAGGCTAATGTGTTGATAGGACAAGGAGACGTG

GTGTTGGTAATGAAACGGAAACGGGACTCTAGCATACTTACTGACAGCCAGACAGCGACC

AAAAGAATTCGGATGGCCATCAATTAGTGTCGAATTGTTTAAAAACGACCTTGTTTCTAC

T---------------------

>A_chicken_Bulgaria_50-1_21VIR1454-9_2021_EPI1858619

AGCRAAAGCAGGTCAAATATATTCAATATGGAGAGAATAAAAGAACTAAGAGATTTGATG

TCGCAGTCTCGCACTCGCGAGATACTAACAAAAACCACTGTGGACCATATGGCCATAATA

AAGAAATACACATCAGGAAGACAGGAGAAGAACCCTGCCCTCAGGATGAAATGGATGATG

GCAATGAAATATCCTATTACAGCTGACAAAAGAATAATAGAGATGATCCCTGAAAGGAAT

GAGCAAGGTCAGACTCTCTGGAGCAAAACAAATGATGCTGGATCAGACAGAGTGATGGTC

TCACCTCTGGCTGTGACATGGTGGAATAGAAATGGGCCAACAACAAGTACAGTACACTAC

CCAAAAGTCTACAAAACCTACTTTGAAAAGGTAGAAAGGTTGAAACATGGGACCTTTGGT

CCTGTTCACTTTCGAAATCAGGTTAAGATACGCCGCAGGGTTGACATAAACCCGGGCCAT

GCAGATCTCAGTGCCAAAGAAGCACAGGATGTCATCATGGAGGTTGTTTTCCCAAATGAA

GTTGGAGCCAGGATCTTGACATCAGAATCACAATTAACAATAACAAGGGAAAAGAAGGAG

GAACTTCAGGATTGCAAGATTGCTCCTTTGATGGTGGCATACATGTTGGAAAGAGAACTG

GTTCGCAAGACCAGATTTTTACCAGTAGCTGGCGGAACAAGCAGCGTATACATCGAGGTA

TTGCATTTGACTCAAGGGACCTGCTGGGAACAAATGTACACACCAGGAGGGGAGGTGAGA

AATGATGATGTTGATCAGAGTTTGATCATTGCTGCTAGAAATATAGTTAGGAGGGCAACA

GTATCAGCAGACCCATTGGCTTCGCTTTTGGAAATGTGCCACAGTACACAAATTGGTGGA

GTAAGGATGGTGGACATTCTTAGGCAGAACCCAACAGAGGAGCAAGCTGTGGATATATGC

AAAGCAGCAATGGGTTTAAGAATCAGTTCATCCTTCAGCTTTGGAGGTTTCACTTTCAAA

AGGACAAGTGGGTCGTCTGTCAAAAGAGAAGAAGAAATACTCACTGGCAACCTCCAAACA

CTGAAAGTAAGAATACATGAAGGATATGAGGAATTCACAATGGTTGGGCGAAGAGCTACA

GCCATTTTGAGGAAAGCAACCAAGAGACTGATCCAATTAATAGTGAGTGGAAGAGACGAG

CAGTCAATCGCTGAAGCAATCATAGTGGCAATGGTTTTCTCACAGGAGGATTGCATGATA

AAAGCAGTACGAGGTGATTTGAATTTTGTCAACAGAGCGAATCAGCGGCTAAATCCTATG

CATCAACTTCTGAGGCATTTCCAAAAGGATGCAAAAGTGCTGTTTCAAAACTGGGGGATT

GAACCAATTGACAATGTAATGGGGATGATCGGGATACTGCCTGACATGACCCCCAGCACA

GAGATGTCACTGAGAGGAGTGAGAGTCAGCAAAATGGGAGTGGATGAATATTCCAGTACT

GAGAGAGTGGTCGTGAGCATTGATCGCTTCTTGAGAGTCCGAGATCAGAGGGGAAATGTG

CTTCTGTCTCCTGAGGAAGTTAGTGAAACACATGGAACAGAGAAACTGACGATAACGTAT

TCATCGTCTATGATGTGGGAAATCAATGGTCCGGAATCCGTGCTAGTCAACACATATCAG

TGGATCATTAGAAATTGGGAAACTGTGAAGATTCAGTGGTCCCAGGACCCTACGATGTTA

TACAATAAGATGGAATTTGAGCCCTTCCAATCCTTGGTGCCCAAGGCTGCTAGAGGCCAG

TATAGTGGGTTTGTGAGGACATTATTCCAACAGATGCGTGATGTGTTGGGGACATTTGAC

ACTGTCCAAATAATAAAGCTCCTACCATTTGCAGCAGCCCCACCGGAACAGAGTAGGATG

CAATTTTCCTCTCTGACTGTGAACGTAAGAGGTTCAGGAATGAGAATACTTGTGAGGGGC

AACTCCCCTGTGTTCAACTATAATAAGGCCACCAAGAGACTCACAGTTCTTGGAAAGGAT

GCAGGCGCCTTGACAGAATATCCAGATGAGGGAACAGCAGGAGTGGAGTCTGCAGTATTA

AGAGGATTTCTAATTCTGGGCAAAGAGGACAAAAGATATGGACCAGCATTGAGCATCAAC

GAATTGAGCAATCTTTCGAAAGGGGAAAAGGCTAATGTGTTGATAGGACAAGGAGACGTG

GTGTTGGTAATGAAACGGAAACGGGACTCTAGCATACTTACTGACAGCCAGACAGCGACC

AAAAGAATTCGGATGGCCATCAATTAGTGTCGAATTGTTTAAAAACGACCTTGTTTCTA-

----------------------

>A_mute_swan_Poland_MB189_2021_EPI1859652

------------TCAAATATATTCAATATGGAGAGAATAAAAGAACTAAGAGATTTGATG

TCGCAGTCTCGCACTCGCGAGATACTAACAAAAACCACTGTGGACCATATGGCCATAATA

AAGAAATACACATCAGGGAGACAGGAGAAGAACCCTGCCCTCAGGATGAAATGGATGATG

GCAATGAAATATCCTATTACAGCTGACAAAAGAATAATGGAGATGATCCCTGAGAGGAAT

GAGCAAGGTCAGACTCTCTGGAGCAAAACAAATGATGCTGGATCAGACAGAGTGATGGTC

TCACCTCTGGCTGTGACATGGTGGAATAGAAATGGGCCAACAACAAGTACAGTACACTAC

CCAAAAGTCTACAAAACCTACTTTGAAAAGGTAGAAAGGTTGAAACATGGAACCTTTGGT

CCTGTTCACTTTCGAAATCAGGTTAAGATACGCCGCAGGGTTGACATAAACCCGGGCCAT

GCAGATCTCAGTGTCAAAGAAGCACAGGATGTCATCATGGAGGTTGTTTTCCCAAATGAA

GTTGGAGCCAGGATCTTGACATCAGAATCACAATTAACAATAACAATGGAAAAGAAGGAG

GAACTTCAGGATTGCAAGATTGCTCCTTTGATGGTGGCATACATGTTGGAAAGAGAACTG

GTTCGCAAGACCAGATTTTTACCAGTAGCTGGCGGGACAAGCAGCGTATACATCGAGGTA

TTGCATTTGACTCAAGGGACCTGCTGGGAACAAATGTACACACCAGGAGGGGAGGTGAGA

AATGATGATGTTGATCAGAGTTTGATCATTGCTGCTAGAAATATAGTTAGGAGGGCAACA

GTATCAGCAGACCCACTGGCTTCGCTCTTGGAAATGTGCCACAGTACACAAATTGGTGGA

GTGAGGATGGTGGACATTCTTAGGCAGAACCCAACAGAGGAGCAAGCTGTGGATATATGC

AAAGCAGCAATGGGTTTAAGAATCAGTTCATCCTTCAGCTTTGGAGGTTTCACTTTCAAA

AGGACAAGTGGGTCGTCTGTCAAAAGAGAAGAAGAAATACTCACTGGCAACCTCCAAACA

CTGAAAGTAAGAATACATGAAGGATATGAGGAATTCACAATGGTTGGGCGAAGAGCTACA

GCCATTTTGAGGAAAGCAACCAGGAGACTGATCCAATTAATAGTGAGTGGACGAGACGAA

CAGTCAATCGCTGAAGCAATCATAGTGGCAATGGTTTTCTCACAGGAGGATTGCATGATA

AAAGCAGTACGAGGTGATTTGAATTTTGTCAACAGAGCGAATCAGCGGCTAAATCCTATG

CATCAACTTCTGAGGCATTTCCAAAAGGATGCAAAAGTGCTGTTTCAAAACTGGGGGATT

GAACCAATTGACAATGTAATGGGGATGATCGGGATACTGCCTGACATGACCCCCAGCACA

GAGATGTCACTGAGAGGAGTGAGAGTCAGCAAAATGGGAGTGGATGAATATTCCAGTACT

GAGAGAGTGGTCGTGAGCATTGATCGCTTCTTGAGAGTCCGAGATCAGAGGGGAAATGTG

CTTCTGTCTCCTGAGGAAGTTAGTGAAACACATGGAACAGAGAAACTGACGATAACGTAT

TCATCGTCTATGATGTGGGAAATCAATGGTCCGGAATCCGTGCTAGTCAACACATATCAA

TGGATCATTAGAAATTGGGAAACTGTGAAGATTCAGTGGTCCCAGGACCCTACGATGTTG

TACAATAAGATGGAATTTGAGCCCTTCCAATCCTTGGTGCCCAAGGCTGCTAGAGGCCAG

TATAGTGGGTTTGTGAGGACATTATTCCAACAGATGCGTGATGTGTTGGGGACATTTGAC

ACTGTCCAAATAATAAAGCTCCTACCATTTGCAGCAGCCCCACCGGAACTGAGTAGGATG

CAATTTTCCTCTCTGACTGTGAACGTAAGAGGTTCAGGAATGAGAATACTTGTGAGGGGC

AATTCCCCTGTGTTCAACTATAATAAGACCACCAAGAGACTCACAGTTCTTGGAAAGGAT

GCAGGCGCCTTGACAGAATATCCAGATGAGGGAACAGCAGGAGTGGAGTCTGCAGTATTA

AGAGGATTTCTAATTCTGGGCAAAGAGGACAAAAGATATGGACCAGCATTGAGCATCAAC

GAATTGAGCAATCTTGCGAAAGGGGAAAAGGCTAATGTGTTGATAGGACAAGGAGACGTG

GTGTTGGTAATGAAACGGAAACGGGACTCTAGCATACTTACTGACAGCCAGACAGCGACC

AAAAGAATTCGGATGGCCATCAATTAGTGTCGAATTGTTTAAAAACGA------------

----------------------

>A_mute_swan_Poland_MB272_2021_EPI1859668

------------TCAAATATATTCAATATGGAGAGAATAAAAGAACTAAGAGATTTGATG

TCGCAGTCTCGCACTCGCGAGATACTAACAAAAACCACTGTGGACCATATGGCCATAATA

AAGAAATACACATCAGGAAGACAGGAGAAGAACCCTGCCCTCAGGATGAAATGGATGATG

GCAATGAAATATCCTATTACAGCTGACAAAAGAATAATGGAGATGATCCCTGAAAGGAAT

GAGCAAGGTCAGACTCTCTGGAGCAAAACAAATGATGCTGGATCAGACAGAGTGATGGTC

TCACCTCTGGCTGTGACATGGTGGAATAGAAATGGGCCAACAACAAGTACAGTACACTAC

CCAAAAGTCTACAAAACCTACTTTGAAAAGGTAGAAAGGTTGAAACATGGAACCTTTGGT

CCTGTTCACTTTCGAAATCAGGTTAAGATACGCCGCAGGGTTGACATAAACCCGGGCCAT

GCAGATCTCAGTGCCAAAGAAGCACAGGATGTCATCATGGAGGTTGTTTTCCCAAATGAA

GTTGGAGCCAGGATCTTGACATCAGAATCACAATTAACAATAACAAGGGAAAAGAAGGAG

GAACTTCAGGATTGCAAGATTGCTCCTTTGATGGTGGCATACATGTTGGAAAGAGAACTG

GTTCGCAAGACCAGATTTTTACCAGTAGCTGGCGGAACAAGCAGCGTATACATCGAGGTA

TTGCATTTGACTCAAGGGACCTGCTGGGAACAAATGTACACACCAGGAGGGGAGGTGAGA

AATGATGATGTTGATCAGAGTTTGATCATTGCTGCTAGAAATATAGTTAGGAGGGCAACA

GTATCAGCAGACCCATTGGCTTCGCTTTTGGAAATGTGCCACAGTACACAAATTGGTGGA

GTAAGGATGGTGGACATTCTTAGGCAGAACCCAACAGAGGAGCAAGCTGTGGATATATGC

AAAGCAGCAATGGGTTTAAGAATCAGTTCATCCTTCAGCTTTGGAGGTTTCACTTTCAAA

AGGACAAGTGGGTCGTCTGTCAAAAGAGAAGAAGAAATACTCACTGGCAACCTCCAAACA

CTGAAAGTAAGAATACATGAAGGATATGAGGAATTCACAATGGTTGGGCGAAGAGCTACA

GCCATTTTGAGGAAAGCAACCAGGAGACTGATCCAATTAATAGTGAGTGGAAGAGACGAG

CAGTCAATCGCTGAAGCAATCATAGTGGCAATGGTTTTCTCACAGGAGGATTGCATGATA

AAAGCAGTACGAGGTGATTTGAATTTTGTCAACAGAGCGAATCAGCGGCTAAATCCTATG

CATCAACTTCTGAGACATTTCCAAAAGGATGCAAAAGTGCTGTTTCAAAACTGGGGGATT

GAACCAATTGACAATGTAATGGGGATGATCGGGATACTGCCTGACATGACCCCCAGCACA

GAGATGTCACTGAGAGGAGTGAGAGTCAGCAAAATGGGAGTGGATGAATATTCCAGTACT

GAGAGAGTGGTCGTGAGCATTGATCGCTTCTTGAGAGTCCGAGATCAGAGGGGAAATGTG

CTTCTGTCTCCTGAGGAAGTTAGTGAAACACATGGAACAGAGAAACTGACGATAACGTAT

TCATCGTCTATGATGTGGGAAATCAATGGACCGGAATCCGTGCTAGTCAACACATATCAA

TGGATCATTAGAAATTGGGAAACTGTGAAGATTCAGTGGTCCCAGGACCCTACGATGTTA

TACAATAAGATGGAATTTGAGCCCTTCCAATCCTTGGTGCCCAAGGCTGCTAGAGGCCAG

TATAGTGGGTTTGTGAGGACATTATTCCAACAGATGCGTGATGTGTTGGGAACATTTGAC

ACTGTCCAAATAATAAAGCTCCTACCATTTGCAGCAGCCCCACCGGAACAGAGTAGGATG

CAATTTTCCTCTCTGACTGTGAACGTAAGAGGTTCAGGAATGAGAATACTTGTGAGGGGC

AACTCCCCTGTGTTCAACTATAATAAGGCCACCAAGAGACTCACAGTTCTTGGAAAGGAT

GCAGGCGCCTTGACAGAATATCCAGATGAGGGAACAGCAGGAGTGGAGTCTGCAGTATTA

AGAGGATTTCTAATTCTGGGCAAAGAGGACAAAAGATATGGACCAGCATTGAGCATCAAC

GAATTGAGCAATCTTGCGAAAGGGGAAAAGGCTAATGTGTTGATAGGACAAGGAGACGTG

GTGTTGGTAATGAAACGGAAACGGGACTCTAGCATACTTACTGACAGCCAGACAGCGACC

AAAAGAATTCGGATGGCCATCAATTAGTGTCGAATTGTTTAAAAACGA------------

----------------------

>A_anser_anser_Spain_297-1_21VIR1230-5_2021_EPI1860068

---------------AATATATTCAATATGGAGAGAATAAAAGAACTAAGAGATTTGATG

TCGCAGTCTCGCACTCGCGAGATACTAACAAAAACCACTGTGGACCATATGGCCATAATA

AAGAAATACACATCAGGAAGACAGGAGAAGAACCCTGCCCTCAGGATGAAATGGATGATG

GCAATGAAATATCCTATTACAGCTGACAAAAGAATAATGGAGATAATCCCTGAAAGGAAT

GAGCAAGGTCAGACTCTCTGGAGCAAAACAAATGATGCTGGATCAGACAGAGTGATGGTC

TCACCTCTGGCTGTGACATGGTGGAATAGAAATGGGCCAACAACAAGTACAGTACACTAC

CCAAAAGTCTACAAAACCTTCTTTGAAAAGGTAGAAAGGTTGAAACATGGAACCTTTGGT

CCTGTTCACTTTCGAAATCAGGTTAAGATACGCCGCAGGGTTGACATAAACCCGGGCCAT

GCAGATCTCAGTGCCAAAGAAGCACAGGATGTCATCATGGAGGTTGTTTTCCCAAATGAA

GTTGGAGCCAGGATCTTGACATCAGAATCACAATTAACAATAACAAGGGAAAAGAAGGAG

GAACTTCAGGATTGCAAGATTGCTCCTTTGATGGTGGCATACATGTTGGAAAGAGAACTG

GTTCGCAAGACCAGATTTTTACCAGTAGCTGGAGGAACAAGCAGCGTATACATCGAGGTA

TTGCATTTGACTCAAGGGACCTGCTGGGAACAAATGTACACACCAGGAGGGGAGGTGAGA

AATGATGATGTTGACCAGAGTTTGATCATTGCTGCTAGAAATATAGTTAGGAGGGCAACA

GTATCAGCAGACCCATTGGCTTCGCTTTTGGAAATGTGCCACAGTACACAAATTGGTGGA

GTAAGGATGGTGGACATTCTTAGGCAGAACCCAACAGAGGAGCAAGCTGTGGATATATGC

AAAGCAGCAATGGGTTTAAGAATCAGTTCATCCTTCAGCTTTGGAGGTTTCACTTTCAAA

AGGACAAGTGGGTCGTCTGTCAAAAGAGAAGAAGAAATACTCACTGGCAACCTCCAAACA

CTGAAAGTAAGAATACATGAAGGATATGAGGAATTCACAATGGTTGGGCGAAGAGCTACA

GCCATTTTGAGGAAAGCAACCAGGAGACTGATCCAATTAATAGTGAGTGGAAGAGACGAG

CAGTCAATCGCTGAAGCAATCATAGTGGCAATGGTTTTCTCACAGGAGGATTGCATGATA

AAAGCAGTACGAGGTGATTTGAATTTTGTCAACAGAGCGAATCAGCGGCTAAATCCTATG

CATCAACTTCTGAGGCATTTCCAAAAGGATGCAAAAGTGCTGTTTCAAAACTGGGGGATT

GAACCAATTGACAATGTAATGGGGATGATCGGGATACTGCCTGACATGACCCCCAGCACA

GAGATGTCACTGAGAGGAGTGAGAGTCAGCAAAATGGGAGTGGATGAATATTCCAGTACT

GAGAGAGTGGTCGTGAGCATTGATCGCTTCTTGAGAGTCCGAGATCAGAGGGGAAATGTG

CTTCTGTCCCCTGAGGAAGTTAGTGAAACACATGGAACAGAGAAACTGACGATAACGTAT

TCATCGTCTATGATGTGGGAAATCAATGGTCCGGAATCCGTGCTAGTCAACACATATCAA

TGGATCATTAGAAATTGGGAAACTGTGAAGATTCAGTGGTCCCAGGACCCTACAATGTTG

TACAATAAGATGGAATTTGAGCCCTTCCAATCCTTGGTGCCCAAGGCTGCTAGAGGCCAG

TATAGTGGGTTTGTGAGGACATTATTCCAACAGATGCGTGATGTGTTGGGGACATTTGAC

ACTGTCCAAATAATAAAGCTCCTACCATTTGCAGCAGCCCCACCGGAACAGAGTAGGATG

CAATTTTCCTCTCTGACTGTGAACGTAAGAGGTTCAGGAATGAGAATACTTGTGAGGGGC

AACTCCCCTGTGTTCAACTATAATAAGGCCACCAAGAGACTCACAGTTCTTGGAAAGGAT

GCAGGCGCCTTGACAGAATATCCAGATGAGGGAACAGCAGGAGTGGAGTCTGCAGTATTA

AGAGGATTTCTAATTCTGGGCAAAGAGGACAAAAGATATGGACCAGCATTGAGCATCAAC

GAATTGAGCAATCTTGCGAAAGGGGAAAAGGCTAATGTGTTGATAGGACAAGGAGACGTG

GTGTTGGTAATGAAACGGAAACGGGACTCTAGCATACTTACTGACAGCCAGACAGCGACC

AAAAGAATTCGGATGGCCATCAATTAGTGTCGAATTGTTTAAAAACGA------------

----------------------

>A_ciconia_ciconia_Spain_102-1_21VIR1230-2_2021_EPI1860076

--------------AAATATATTCAATATGGAGAGAATAAAAGAACTAAGAGATTTGATG

TCGCAGTCTCGCACTCGCGAGATACTAACAAAAACCACTGTGGACCATATGGCCATAATA

AAGAAATACACATCAGGAAGACAGGAGAAGAACCCTGCCCTCAGGATGAAATGGATGATG

GCAATGAAATATCCTATTACAGCTGACAAAAGAATAATGGAGATGATCCCTGAAAGGAAT

GAGCAAGGTCAGACTCTCTGGAGCAAAACAAATGATGCTGGATCAGACAGAGTGATGGTC

TCACCTCTGGCTGTGACATGGTGGAATAGAAATGGGCCAACAACAAGTACAGTACACTAC

CCAAAAGTCTACAAAACCTACTTTGAAAAGGTAGAAAGGTTGAAACATGGAACCTTTGGG

CCTGTTCACTTTCGAAATCAGGTTAAGATACGCCGCAGGGTTGACATAAACCCGGGCCAT

GCAGATCTCAGTGCCAAAGAAGCACAGGATGTCATCATGGAGGTTGTTTTCCCAAATGAA

GTTGGAGCCAGGATCTTGACATCAGAATCACAATTAACAATAACAAGGGAAAAGAAGGAG

GAACTTCAGGATTGCAAGATTGCTCCTTTGATGGTGGCATACATGTTGGAAAGAGAACTG

GTTCGCAAGACCAGATTTTTACCAGTAGCTGGCGGAACAAGCAGCGTATACATCGAGGTA

TTGCATTTGACTCAAGGGACCTGCTGGGAACAAATGTACACACCAGGAGGGGAGGTGAGA

AATGATGATGTTGATCAGAGTTTGATCATTGCTGCTAGAAATATAGTTAGGAGGGCAACA

GTATCAGCAGACCCATTGGCTTCGCTTTTGGAAATGTGCCACAGTACACAAATTGGTGGA

GTAAGGATGGTGGACATTCTTAGGCAGAACCCAACAGAGGAGCAAGCTGTGGATATATGC

AAAGCAGCAATGGGTTTAAGAATCAGTTCATCCTTCAGCTTTGGAGGTTTCACTTTCAAA

AGGACAAGTGGGTCGTCTGTCAAAAGAGAAGAAGAAATACTCACTGGCAACCTCCAAACA

CTGAAAGTAAGAATACATGAAGGATATGAGGAATTCACAATGGTTGGGCGAAGAGCTACA

GCCATTTTGAGGAAAGCAACCAGGAGACTGATCCAATTAATAGTGAGTGGAAGAGACGAG

CAGTCAATCGCTGAAGCAATCATAGTGGCAATGGTTTTCTCACAGGAGGATTGCATGATA

AAAGCAGTACGAGGTGATTTGAATTTTGTCAACAGAGCGAATCAGCGGCTAAATCCTATG

CATCAACTTCTGAGGCATTTCCAAAAGGATGCAAAAGTGCTGTTTCAAAACTGGGGGATT

GAACCAATTGACAATGTAATGGGGATGATCGGGATACTGCCTGACATGACCCCCAGCACA

GAGATGTCACTGAGAGGAGTGAGAGTCAGCAAAATGGGAGTGGATGAATATTCCAGTACT

GAGAGAGTGGTCGTGAGCATTGATCGCTTCTTGAGAGTCCGAGATCAGAGGGGAAATGTG

CTTCTGTCTCCTGAGGAAGTTAGTGAAACACATGGAACAGAGAAACTGACGATAACATAT

TCATCGTCTATGATGTGGGAAATCAATGGTCCGGAATCCGTGCTAGTCAACACATATCAA

TGGATCATTAGAAATTGGGAAACTGTGAAGATTCAGTGGTCCCAGGACCCTACGATGTTA

TACAATAAGATGGAATTTGAGCCCTTCCAATCCTTGGTGCCCAAGGCTGCTAGAGGCCAG

TATAGTGGGTTTGTGAGGACATTATTCCAACAGATGCGTGATGTGTTGGGGACATTTGAC

ACTGTCCAAATAATAAAGCTCCTACCATTTGCAGCAGCCCCACCGGAACAGAGTAGGATG

CAATTTTCCTCTCTGACTGTGAACGTAAGAGGTTCAGGAATGAGAATACTTGTGAGGGGC

AACTCCCCTGTGTTCAACTATAATAAGGCCACCAAGAGACTCACAGTTCTTGGAAAGGAT

GCAGGCGCCTTGACAGAATATCCAGATGAGGGAACAGCAGGAGTGGAGTCTGCAGTATTA

AGAGGATTTCTAATTCTGGGCAAAGAGGACAAAAGATATGGACCAGCATTGAGCATCAAC

GAATTGAGCAATCTTGCGAAAGGGGAAAAGGCTAATGTGTTGATAGGACAAGGAGACGTG

GTGTTGGTAATGAAACGGAAACGGGACTCTAGCATACTTACTGACAGCCAGACAGCGACC

AAAAGAATTCGGATGGCCATCAATTAGTGTCGAATTGTTTAA------------------

----------------------

>A_duck_Jiangsu_k1203_2010_EPI442014

AGCGAAAGCAGGTCAAATATATTCAATATGGAGAGAATAAAAGAACTAAGAGATCTAATG

TCTCAATCCCGCACTCGCGAGATACTAACAAAAACCACTGTGGACCATATGGCCATAATC

AAGAAATACACATCAGGAAGACAAGAGAAGAACCCTGCTCTCAGAATGAAATGGATGATG

GCAATGAAATATCCAATCACAGCAGACAAGAGAATAATGGAAATGATTCCTGAAAAAAAT

GAACAAGGCCAGACGCTTTGGAGCAAGACAAATGATGCTGGATCAGACAGAGTGATGGTG

TCTCCCCTAGCTGTAACTTGGTGGAATAGAAATGGACCGACAGCAAGTACAGTCCATTAT

CCAAAGGTTTACAAAACATACTTTGAGAAGGTTGAAAGGTTAAAGCATGGAACCTTCGGT

CCCGTTCACTTCCGAAACCAAATTAAAATACGCCGCCGAGTTGACATAAATCCAGGCCAC

GCAGATCTCAGTGCCAAAGAAGCACAAGATGTCATCATGGAGGTCGTTTTCCCAAATGAA

GTGGGAGCTAGAATATTGACATCAGAGTCACAATTGACAATAACGAAAGAGAAAAAAGAA

GAGCTCCAGGATTGTAAGATTGCTCCTTTAATGGTGGCATACATGTTGGAAAGAGAACTG

GTCCGCAAAACCAGATTCCTACCGGTAGCAGGTGGGACAAGCAGTGTGTACATTGAGGTA

CTGCACTTGACCCAAGGGACCTGCTGGGAACAGATGTACACTCCAGGCGGAGAAGTGAGA

AATGACGATGTTGACCAGAGTTTGATTATCGCTGCCAGAAACATTGTTAGGAGAGCAACG

GTATCAGCGGATCCACTGGCATCATTATTGGAGATGTGCCACAGCACACAAATTGGTGGG

ACAAGGATGGTGGATATCCTTAGGCAAAATCCAACTGAGGAACAAGCTGTGGATATATGC

AAAGCAGCAATGGGTTTGAGGATTAGTTCATCCTTTAGCTTTGGAGGATTCACCTTCAAA

AGAACAAGTGGGTCATCCGTTAGAAAGGAAGAGGAAGTGCTTACGGGCAACCTCCAAACA

TTGAAAATAAGAGTACATGAGGGGTATGAGGAGTTCACAATGGTTGGGCGAAGAGCAACA

GCCATCCTAAGGAAAGCAACTAGAAGGCTGATTCAGTTGATAGTAAGTGGAAGAGACGAA

CAATCAATCGCTGAAGCAATCATCGTAGCCATGGTGTTCTCACAGGAGGATTGCATGATA

AAGGCAGTCCGAGGCGATCTGAATTTCGTGAACAGAGCAAACCAAAGATTGAACCCCATG

CATCAACTCCTGAGACACTTCCAAAAAGATGCAAAAGTGCTGTTTCAAAATTGGGGAATT

GAACCTATTGATAATGTCATGGGGATGATTGGAATATTACCTGACATGACTCCAAGCACA

GAGATGTCACTAAGAGGAGTAAGAGTTAGTAAAATGGGAGTAGATGAATATTCCAGCACT

GAGAGAGTGGTTGTAAGCATTGACCGTTTCTTGAGGGTTCGAGATCAGCAGGGGAACGTA

CTCTTATCTCCCGAAGAGGTCAGCGAAACACTGGGAACAGAAAAATTGACAATAACATAT

TCATCATCAATGATGTGGGAAATCAATGGTCCTGAGTCAGTACTGGTTAACACCTATCAA

TGGATCATCAGAAATTGGGAGATTGTGAAGATTCAATGGTCTCAAGACCCCACGATGCTG

TACAATAAGGTGGAGTTTGAACCGTTCCAATCCTTGGTACCTAAAGCTGCCAGAGGCCAA

TACAGTGGATTTGTGAGAACACTGTTCCAACAAATGCGTGACGTATTGGGGACATTTGAT

ACTGTCCAGATAATAAAGCTGTTACCGTTTGCAGCAGCCCCACCGGAGCAGAGCAGAATG

CAATTTTCTTCCCTGACTGTGAATGTGAGAGGCTCGGGAATGAGAATACTCGTAAGGGGC

AACTCCCCTGTGTTCAACTACAATAAGGCAACCAAAAGGCTTGCCGTCCTTGGAAAGGAC

GCAGGTGCATTAACAGAGGATCCAGATGAGGGGACAACAGGAGTGGAATCTGCAGTGCTG

AGGGGGTTCCTAATTCTGGGCAAGGAGGACAGAAGATATGGACCAGCACTAAGCATCAAT

GAACTGAGCAATCTTGCGAAAGGGGAGAAAGCCAATGTGCTGATAGGGCAAGGAGACGTG

GTGCTGGTAATGAAACGGAAACGGGACTCTAGCATACTTACTGACAGCCAGACAGCGACC

AAAAGAATTCGGG-----------------------------------------------

----------------------

>A_Turkey_Egypt_AR550_2018_EPI1420343

---------------------------ATGGAGAGAATAAAAGAACTAAGAGATTTGATG

TCGCAGTCTCGCACTCGCGAGATACTGACAAAAACCACTGTGGACCATATGGCCATAATA

AAGAAATACACATCAGGAAGACAGGAGAAGAACCCTGCCCTCAGGATGAAATGGATGATG

GCAATGAAATATCCTATTACAGCTGACAAAAGAATAATGGAGATGATCCCTGAAAGGAAT

GAGCAAGGTCAGACTCTCTGGAGCAAAACAAATGATGCTGGATCAGACAGAGTGATGGTA

TCACCTCTGGCTGTGACATGGTGGAATAGAAATGGGCCAACAACAAGTACAGTGCACTAC

CCAAAAGTCTACAAAACCTACTTTGAAAAGGTAGAAAGGTTGAAACATGGAACCTTTGGT

CCTGTTCACTTTCGAAATCAGGTTAAGGTACGCCGCAGGGTTGACATAAACCCGGGCCAT

GCGGATCTCAGTGCCAAAGAAGCACAGGATGTCATCATGGAGGTCGTTTTCCCAAATGAA

GTTGGAGCCAGGATCTTGACATCAGAATCACAATTAACAATAACAAAGGAAAAGAAGGAG

GAACTTCAGGATTGCAAGATTGCTCCTTTGATGGTGGCATACATGTTGGAGAGAGAACTG

GTTCGCAAGACCAGATTTTTACCAGTAGCTGGCGGAACAAGCAGCGTATATATCGAGGTA

TTGCATTTGACTCAAGGGACCTGCTGGGAACAAATGTACACACCAGGAGGGGAGGTGAGA

AATGATGATGTTGATCAGAGTTTGATCATTGCTGCTAGAAATATCGTTAGGAGGGCAACA

GTATCAGCAGACCCATTGGCTTCGCTCTTGGAAATGTGCCACAGTACACAAATTGGTGGA

GTAAGGATGGTGGACATTCTTAGGCAGAACCCAACAGAAGAGCAAGCTGTGGATATATGC

AAAGCAGCAATGGGTTTAAGAATCAGTTCATCCTTCAGCTTTGGAGGTTTCACTTTCAAA

AGGACAAGTGGGTCGTCTGTCAAAAGAGAAGAAGAAATACTCACTGGCAACCTCCAAACA

CTGAAAGTAAGAGTACATGAAGGATATGAGGAATTCACAATGGTTGGGCGAAGAGCTACA

GCCATTTTGAGGAAAGCAACCAGGAGACTGATCCAATTAATAGTGAGTGGAAGAGACGAG

CAGTCAATCGCTGAAGCAATCATAGTGGCAATGGTTTTCTCACAGGAGGATTGCATGATA

AAAGCAGTACGAGGTGATCTGAATTTTGTCAACAGAGCGAATCAGCGGCTAAATCCTATG

CATCAACTTCTGAGGCATTTCCAAAAGGATGCAAAAGTGCTGTTTCAAAACTGGGGGATT

GAACCAATTGACAATGTAATGGGGATGATCGGGATACTTCCTGACATGACCCCCAGCACA

GAGATGTCACTGAGAGGAGTGAGAGTCAGCAAAATGGGAGTGGATGAATATTCCAGTACT

GAGAGAGTGGTCGTGAGCATTGATCGCTTCTTGAGAGTCCGAGATCAGAGNGGAAATGTG

CTTCTGTCTCCTGAGGAAGTTAGTGAAACACAGGGAACAGAGAAACTGACGATAACGTAT

TCATCGTCCATGATGTGGGAAATCAATGGTCCGGAATCCGTGCTAGTCAACACATATCRA

TGGATCATTAGAAATTGGGAAACTGTGAAGATTCAGTGGTCCCAGGACCCTACGATGTTG

TACAATAAGATGGAATTTGAGCCCTTCCAATCCTTGGTGCCCAAGGCTGCCAGAGGCCAG

TATAGTGGATTTGTGAGGACATTATTCCAACAGATGCGTGATGTGCTGGGGACATTTGAC

ACTGTCCAAATGATAAAGCTCCTACCANTTGCAGCAGCTCCACCGGAACAGAGTAGGATG

CAATTTTCCTCTCTGACTGTGAACGTAAGAGGTTCAGGAATGAGAATACTTGTGAGAGGC

AACTCCCCTGTGTTCAACTATAACAAGGCCACCAAGAGACTCACAGTTCTTGGAAAGGAT

GCAGGCGCCTTGACAGAATATCCAGATGAGGGAACAGCAGGAGTGGAGTCTGCAGTATTA

AGAGGATTTCTAATTCTGGGCAAAGAGGACAAAAGATATGGACCAGCATTGAGCATCAAT

GAATTGAGCAATCTTGCGAAAGGGGAAAAGGCTAATGTGTTGATAGGGCAAGGAGACGTG

GTGTTGGTAATGAAACGGAAACGGGACTCTAGCATACTTACTGACAGCCAGACAGCGACC

AAAAGAATTCGGATGGCCATCAATTAG---------------------------------

----------------------

>A_Chicken_Egypt_AR553_2018_EPI1638998

---------------------------ATGGAGAGAATAAAAGAACTAAGAGATTTGATG

TCGCAGTCTCGCACTCGCGAGATACTGACAAAAACCACTGTGGACCATATGGCCATAATA

AAGAAATACACATCAGGAAGACAGGAGAAGAACCCTGCCCTCAGGATGAAATGGATGATG

GCAATGAAATATCCTATTACAGCTGACAAAAGAATAATGGAGATGATCCCTGAAAGGAAT

GAGCAAGGTCAGACTCTCTGGAGCAAAACAAATGATGCTGGATCAGACAGAGTGATGGTA

TCACCTCTGGCTGTGACATGGTGGAATAGAAATGGGCCAACAACAAGTACAGTGCACTAC

CCAAAAGTCTACAAAACCTACTTTGAAAAGGTAGAAAGGTTGAAACATGGAACCTTTGGT

CCTGTTCACTTTCGAAATCAGGTTAAGGTACGCCGCAGGGTTGACATAAACCCGGGCCAT

GCGGATCTCAGTGCCAAAGAAGCACAGGATGTCATCATGGAGGTCGTTTTCCCAAATGAA

GTTGGAGCCAGGATCTTGACATCAGAATCACAATTAACAATAACAAAGGAAAAGAAGGAG

GAACTTCAGGATTGCAAGATTGCTCCTTTGATGGTGGCATACATGTTGGAGAGAGAACTG

GTTCGCAAGACCAGATTTTTACCAGTAGCTGGCGGAACAAGCAGCGTATATATCGAGGTA

TTGCATTTGACTCAAGGGACCTGCTGGGAACAAATGTACACACCAGGAGGGGAGGTGAGA

AATGATGATGTTGATCAGAGTTTGATCATTGCTGCTAGAAATATCGTTAGGAGGGCAACA

GTATCAGCAGACCCATTGGCTTCGCTCTTGGAAATGTGCCACAGTACACAAATTGGTGGA

GTAAGGATGGTGGACATTCTTAGGCAGAACCCAACAGAAGAGCAAGCTGTGGATATATGC

AAAGCAGCAATGGGTTTAAGAATCAGTTCATCCTTCAGCTTTGGAGGTTTCACTTTCAAA

AGGACAAGTGGGTCGTCTGTCAAAAGAGAAGAAGAAATACTCACTGGCAACCTCCAAACA

CTGAAAGTAAGAGTACATGAAGGATATGAGGAATTCACAATGGTTGGGCGAAGAGCTACA

GCCATTTTGAGGAAAGCAACCAGGAGACTGATCCAATTAATAGTGAGTGGAAGAGACGAG

CAGTCAATCGCTGAAGCAATCATAGTGGCAATGGTTTTCTCACAGGAGGATTGCATGATA

AAAGCAGTACGAGGTGATCTGAATTTTGTCAACAGAGCGAATCAGCGGCTAAATCCTATG

CATCAACTTCTGAGGCATTTCCAAAAGGATGCAAAAGTGCTGTTTCAAAACTGGGGGATT

GAACCAATTGACAATGTAATGGGGATGATCGGGATACTTCCTGACATGACCCCCAGCACA

GAGATGTCACTGAGAGGAGTGAGAGTCAGCAAAATGGGAGTGGATGAATATTCCAGTACT

GAGAGAGTGGTCGTGAGCATTGATCGCTTCTTGAGAGTCCGAGATCAGAGGGGAAATGTG

CTTCTGTCTCCTGAGGAAGTTAGTGAAACACAGGGAACAGAGAAACTGACGATAACGTAT

TCATCGTCCATGATGTGGGAAATCAATGGTCCGGAATCCGTGCTAGTCAACACATATCAA

TGGATCATTAGAAATTGGGAAACTGTGAAGATTCAGTGGTCCCAGGACCCTACGATGTTG

TACAATAAGATGGAATTTGAGCCCTTCCAATCCTTGGTGCCCAAGGCTGCCAGAGGCCAG

TATAGTGGATTTGTGAGGACATTATTCCAACAGATGCGTGATGTGCTGGGGACATTTGAC

ACTGTCCAAATAATAAAGCTCCTACCATTTGCAGCAGCTCCACCGGAACAGAGTAGGATG

CAATTTTCCTCTCTGACTGTGAACGTAAGAGGTTCAGGAATGAGAATACTTGTGAGAGGC

AACTCCCCTGTGTTCAACTATAACAAGGCCACCAAGAGACTCACAGTTCTTGGAAAGGAT

GCAGGCGCCTTGACAGAATATCCAGATGAGGGAACAGCAGGAGTGGAGTCTGCAGTATTA

AGAGGATTTCTAATTCTGGGCAAAGAGGACAAAAGATATGGACCAGCATTGAGCATCAAT

GAATTGAGCAATCTTGCGAAAGGGGAAAAGGCTAATGTGTTGATAGGGCAAGGAGACGTG

GTGTTGGTAATGAAACGGAAACGGGACTCTAGCATACTTACTGACAGCCAGACAGCGACC

AAAAGAATTCGGATGGCCATCAATTAG---------------------------------

----------------------

>A_Turkey_Egypt_AI20285_2019_EPI1638999

---------------------------ATGGAGAGAATAAAAGAACTAAGAGATTTGATG

TCGCAGTCTCGCACTCGCGAGATACTGACAAAAACCACTGTGGACCATATGGCCATAATA

AAGAAATACACATCAGGAAGACAGGAGAAGAACCCTGCCCTCAGGATGAAATGGATGATG

GCAATGAAATATCCCATTACAGCTGACAAAAGAATAATGGAGATAATCCCTGAAAGGAAT

GAGCAAGGTCAGACTCTCTGGAGCAAAACAAATGACGCTGGATCAGACAAAGTGATGGTA

TCACCTCTGGCTGTAACATGGTGGAATAGAAATGGGCCAACAACAAGTACAGTACACTAC

CCAAAAGTCTACAAAACCTACTTTGAAAAGGTAGAAAGGTTGAAACATGGAACATTTGGT

CCTGTTCACTTTCGAAATCAGGTTAAAATACGCCGCAGGGTTGACATAAACCCGGGCCAT

GCGGATCTCAGTGCCAAAGAAGCACAGGATGTCATCATGGAGGTCGTTTTCCCAAATGAA

GTTGGAGCCAGGATCTTGACATCAGAATCACAATTAACAATAACAAAGGAAAAGAAGGAG

GAACTTCAGGATTGCAAGATTGCTCCTTTGATGGTGGCTTACATGTTGGAGAGAGAACTG

GTTCGCAAGACCAGATTTTTACCAGTAGCTGGCGGAACAAGCAGCGTATATATCGAGGTA

TTGCATTTGACTCAGGGGACCTGCTGGGAACAAATGTACACACCAGGAGGGGAGGTGAGA

AATGATGATGTTGATCAGAGTTTGATCATTGCTGCTAGAAATATCGTTAGGAGGGCAACA

GTATCAGCAGACCCATTGGCTTCGCTCTTGGAAATGTGCCACAGTACACAAATTGGTGGA

GTAAGGATGGTTGATATTCTTAGGCAGAACCCAACAGAAGAGCAAGCTGTGGATATATGC

AAAGCAGCAATGGGTTTAAGAATCAGTTCATCCTTCAGCTTTGGAGGTTTCACTTTCAAA

AGGACAAGTGGGTCGTCTGTCAAAAGAGAAGAAGAAATACTCACTGGCAACCTCCAAACA

CTGAAAGTAAGAGTACATGAAGGATATGAGGAATTTACAATGGTTGGGCGAAGAGCTACA

GCCATTTTGAGAAAAGCAACCAGAAGACTGATCCAATTAATAGTGAGTGGAAGAGACGAG

CAGTCAATCGCTGAAGCAATCATAGTGGCAATGGTTTTCTCACAGGAAGATTGCATGATA

AAAGCAGTACGAGGTGATCTGAATTTTGTCAACAGAGCGAATCAGCGGCTAAATCCTATG

CATCAACTTCTGAGGCATTTCCAAAAGGATGCAAAAGTGCTGTTTCAAAACTGGGGGATT

GAACCAATTGACAATGTAATGGGGATGATCGGGATACTGCCTGACATGACCCCCAGCACA

GAGATGTCACTGAGAGGAGTGAGAGTCAGCAAAATGGGAGTGGATGAATATTCCAGTACC

GAGAGAGTGGTCGTGAGCATTGATCGTTTCTTGAGAGTCCGAGATCAGAGGGGAAATGTG

CTTCTGTCTCCTGAGGAAGTTAGTGAAACACAGGGAACAGAGAGACTGACGATAACGTAT

TCATCGTCCATGATGTGGGAAATCAATGGTCCGGAATCCGTGCTAGTCAACACATATCAA

TGGATCATTAGAAATTGGGAAACTGTGAAGATTCAGTGGTCCCAGGACCCTACAATGTTG

TACAATAAGATGGAGTTTGAGCCTTTCCAATCCTTGGTGCCCAAGGCTGCCAGAGGCCAG

TATAGTGGATTTGTGAGGACATTATTCCAACAGATGCGTGATGTGCTGGGGACATTTGAC

ACTGTCCAAATAATAAAGCTCCTACCATTTGCAGCAGCTCCACCGGAACAGAGTAGGATG

CAATTTTCCTCTCTGACTGTGAACGTAAGAGGTTCAGGAATGAGAATACTTGTGAGAGGC

AACTCCCCTGTGTTCAACTATAACAAGGCCACCAAGAGACTCACAGTTCTTGGAAAGGAT

GCAGGCGCCTTGACAGAATATCCAGATGAGGGAACAGCAGGAGTGGAGTCTGCAGTATTA

AGAGGATTTCTAATTCTGGGCAAAGAGGACAAAAGATATGGACCAGCATTGAGCATCAAT

GAATTGAGCAATCTTGCGAAAGGGGAAAAGGCTAATGTGTTGATAGGGCAAGGAGACGTG

GTGTTGGTAATGAAACGGAAACGGGACTCTAGCATACTTACTGACAGCCAGACAGCGACC

AAAAGAATTCGGATGGCCATCAATTAG---------------------------------

----------------------

>A_Chicken_Egypt_AI20286_2019_EPI1639000

---------------------------ATGGAGAGAATAAAAGAACTAAGAGATTTGATG

TCGCAGTCTCGCACTCGCGAGATACTGACAAAAACCACTGTGGACCATATGGCTATAATA

AAGAAATACACATCAGGAAGACAGGAGAAGAACCCTGCCCTCAGGATGAAATGGATGATG

GCAATGAAATATCCCATTACAGCTGACAAAAGAATAATGGAGATAATCCCTGAAAGGAAT

GAGCAAGGTCAGACTCTCTGGAGCAAAACAAATGACGCTGGATCAGACAAAGTGATGGTA

TCACCTCTGGCTGTAACATGGTGGAATAGAAATGGGCCAACAACAAGTACAGTACACTAC

CCAAAAGTCTACAAAACCTACTTTGAAAAGGTAGAAAGGTTGAAACATGGAACATTTGGT

CCTGTTCACTTTCGAAATCAGGTTAAAATACGCCGCAGGGTTGACATAAACCCGGGCCAT

GCGGATCTCAGTGCCAAAGAAGCACAGGATGTCATCATGGAGGTCGTTTTCCCAAATGAA

GTTGGAGCCAGGATCTTGACATCAGAATCACAATTAACAATAACAAAGGAAAAGAAGGAG

GAACTTCAGGATTGCAAGATTGCTCCTTTGATGGTGGCTTACATGTTGGAGAGAGAACTG

GTTCGCAAGACCAGATTTTTACCAGTAGCTGGCGGAACAAGCAGCGTATATATCGAGGTA

TTGCATTTGACTCAGGGGACCTGCTGGGAACAAATGTACACACCAGGAGGGGAGGTGAGA

AATGATGATGTTGATCAGAGTTTGATCATTGCTGCTAGAAATATCGTTAGGAGGGCAACA

GTATCAGCAGACCCATTGGCTTCGCTCTTGGAAATGTGCCACAGTACACAAATTGGTGGA

GTAAGGATGGTTGACATTCTTAGGCAGAACCCAACAGAAGAGCAAGCTGTGGATATATGC

AAAGCAGCAATGGGTTTAAGAATCAGTTCATCCTTCAGCTTTGGAGGTTTCACTTTCAAA

AGGACAAGTGGGTCGTCTGTCAAAAGAGAAGAAGAAATACTCACTGGCAACCTCCAAACA

CTGAAAGTAAGAGTATATGAAGGATATGAGGAATTCACAATGGTTGGGCGAAGAGCTACA

GCCATTTTGAGGAAAGCAACCAGGAGACTGATCCAATTAATAGTGAGTGGAAGAGACGAG

CAGTCAATCGCTGAAGCAATCATAGTGGCAATGGTTTTCTCACAGGAGGATTGCATGATA

AAAGCAGTACGAGGTGATCTGAATTTTGTCAACAGAGCGAATCAGCGGCTAAATCCTATG

CATCAACTTCTGAGGCATTTCCAAAAGGATGCAAAAGTGCTGTTTCAAAACTGGGGGATT

GAACCAATTGACAATGTAATGGGGATGATCGGGATACTGCCTGACATGACCCCCAGCACA

AAAATGTCACTGAGAGGAGTGAGAGTCAGCAAAATGGGAGTGGATGAATATTCCAGTACT

GAGAGAGTGGTCGTGAGCATTGATCGATTCTTGAGAGTCCGAGATCAGAGGGGAAATGTG

CTTCTGTCTCCTGAGGAAGTTAGTGAAACACAGGGAACAGAGAAACTGACGATAACGTAT

TCATCGTCCATGATGTGGGAAATCAACGGTCCGGAATCCGTGCTAGTCAACACATATCAA

TGGATCATTAGAAGTTGGGAAACTGTGAAGATTCAGTGGTCCCAGGACCCCACGATGTTG

TACAATAAGATGGAATTTGAGCCCTTCCAATCCTTGGTGCCCAAAGCTGCCAGAGGCCAG

TATAGTGGATTTGTGAGGACATTATTCCAACAGATGCGTGATGTGCTGGGGACATTTGAC

ACTGTCCAAATAATAAAGCTCCTACCATTTGCAGCAGCTCCACCGGAACAGAGTAGGATG

CAATTTTCCTCTCTGACTGTGAACGTAAGAGGTTCAGGAATGAGAATACTTGTGAGAGGC

AACTCCCCTGTGTTCAACTATAACAAGGCCACCAAGAGACTCACAGTTCTTGGAAAGGAT

GCAGGCGCCTTGACAGAATATCCAGATGAGGGAACAGCAGGAGTGGAGTCTGCAGTATTA

AGAGGATTTCTAATTCTGGGCAAAGAGGACAAAAGATATGGACCAGCATTGAGCATCAAT

GAATTGAGCAATCTTGCGAAAGGGGAAAAGGCTAATGTGTTGATAGGGCAAGGAGACGTG

GTGTTGGTAATGAAACGGAAACGGGACTCTAGCATACTTACTGACAGCCAGACAGCGACC

AAAAGAATTCGGATGGCCATCAATTAG---------------------------------

----------------------

>A_goose_Omsk_0111_2020_EPI1813142

AGCAAAAGCAGGTCAAATATATTCAATATGGAGAGAATAAAAGAACTAAGAGATTTGATG

TCGCAGTCTCGCACTCGCGAGATACTAACAAAAACCACTGTGGACCATATGGCCATAATA

AAGAAATACACATCAGGAAGACAGGAGAAGAACCCTGCCCTCAGGATGAAATGGATGATG

GCAATGAAATATCCTATTACAGCTGACAAAAGAATAATGGAGATGATCCCTGAAAGGAAT

GAGCAAGGTCAGACTCTCTGGAGCAAAACAAATGATGCTGGATCAGACAGAGTGATGGTC

TCACCTCTGGCTGTGACATGGTGGAATAGAAATGGGCCAACAACAAGTACAGTACACTAC

CCAAAAGTCTACAAAACCTACTTTGAAAAGGTAGAAAGGTTGAAACATGGAACCTTTGGT

CCTGTTCACTTTCGAAATCAGGTTAAGATACGCCGCAGGGTTGACATAAACCCGGGCCAT

GCAGATCTCAGTGCCAAAGAAGCACAGGATGTCATCATGGAGGTTGTTTTCCCAAATGAA

GTTGGAGCCAGGATCTTGACATCAGAATCACAATTAACAATAACAAGGGAAAAGAAGGAG

GAACTTCAGGATTGCAAGATTGCTCCTTTGATGGTGGCATACATGTTGGAAAGAGAACTG

GTTCGCAAGACCAGATTTTTACCAGTAGCTGGCGGAACAAGCAGCGTATACATCGAGGTA

TTGCATTTGACTCAAGGGACCTGCTGGGAACAAATGTACACACCAGGAGGGGAGGTGAGA

AATGATGATGTTGATCAGAGTTTGATCATTGCTGCTAGAAATATAGTTAGGAGGGCAACA

GTATCAGCAGACCCATTGGCTTCGCTCTTGGAAATGTGCCACAGTACACAAATTGGTGGA

GTAAGGATGGTGGACATTCTTAGGCAGAACCCAACAGAGGAGCAAGCTGTGGATATATGC

AAAGCAGCAATGGGTTTAAGAATCAGTTCATCCTTCAGCTTTGGAGGTTTCACTTTCAAA

AGGACAAGTGGGTCGTCTGTCAAAAGAGAAGAAGAAATACTCACTGGCAACCTCCAAACA

CTGAAAGTAAGAATACATGAAGGATATGAGGAATTCACAATGGTTGGGCGAAGAGCTACA

GCCATTTTGAGGAAAGCAACCAGGAGACTGATCCAATTAATAGTGAGTGGAAGAGACGAG

CAGTCAATCGCTGAAGCAATCATAGTGGCAATGGTTTTCTCACAGGAGGATTGCATGATA

AAAGCAGTACGAGGTGATTTGAATTTTGTCAACAGAGCGAATCAGCGGCTAAATCCTATG

CATCAACTTCTGAGGCATTTCCAAAAGGATGCAAAAGTGCTGTTTCAAAACTGGGGGATT

GAACCAATTGACAATGTAATGGGGATGATCGGGATACTGCCTGACATGACCCCCAGCACA

GAGATGTCACTGAGAGGAGTGAGAGTCAGCAAAATGGGAGTGGATGAATATTCCAGTACT

GAGAGAGTGGTCGTGAGCATTGATCGCTTCTTGAGAGTCCGAGATCAGAGGGGAAATGTG

CTTCTGTCTCCTGAGGAAGTTAGTGAAACACATGGAACAGAGAAACTGACGATAACGTAT

TCATCGTCTATGATGTGGGAAATCAATGGTCCGGAATCCGTGCTAGTCAACACATATCAA

TGGATCATTAGAAGTTGGGAAACTGTGAAGATTCAATGGTCCCAGGACCCTACGATGTTG

TACAATAAGATGGAATTTGAGCCCTTCCAATCCTTGGTGCCCAAGGCTGCTAGAGGCCAG

TATAGTGGGTTTGTGAGGACATTATTCCAACAGATGCGTGATGTGTTGGGGACATTTGAC

ACTGTCCAAATAATAAAGCTCCTACCATTTGCAGCAGCCCCACCGGAACAGAGTAGGATG

CAATTTTCCTCTCTGACTGTGAACGTAAGAGGTTCAGGAATGAGAATACTTGTGAGGGGC

AACTCCCCTGTGTTCAACTATAATAAGGCCACCAAGAGACTCACAGTTCTTGGAAAGGAT

GCAGGCGCCTTGACAGAATATCCAGATGAGGGAACAGCAGGAGTGGAGTCTGCAGTATTA

AGAGGATTTCTAATTCTGGGCAAAGAGGACAAAAGATATGGACCAGCATTGAGCATCAAC

GAATTGAGCAATCTTGCGAAAGGGGAAAAGGCTAATGTGTTGATAGGACAAGGAGACGTG

GTGTTGGTAATGAAACGGAAACGGGACTCTAGCATACTTACTGACAGCCAGACAGCGACC

AAAAGAATTCGGATGGCCATCAATTAGTGTCGAATTGTTTAAAAACGACCTTGTTTCTAC

T---------------------

>A_goose_Omsk_01161_2020_EPI1813358

AGCAAAAGCAGGTCAAATATATTCAATATGGAGAGAATAAAAGAACTAAGAGATTTGATG

TCGCAGTCTCGCACTCGCGAGATACTAACAAAAACCACTGTGGACCATATGGCCATAATA

AAGAAATACACATCAGGGAGACAGGAGAAGAACCCTGCCCTCAGGATGAAATGGATGATG

GCAATGAAATATCCTATTACAGCTGACAAAAGAATAATGGAGATGATCCCTGAAAGGAAT

GAGCAAGGTCAGACTCTCTGGAGCAAAACAAATGATGCTGGATCAGACAGAGTGATGGTC

TCACCTCTGGCTGTGACATGGTGGAATAGAAATGGGCCAACAACAAGTACAGTACACTAC

CCAAAAGTCTACAAAACCTACTTTGAAAAGGTAGAAAGGTTGAAACATGGAACCTTTGGT

CCTGTTCACTTTCGAAATCAGGTTAAGATACGCCGCAGGGTTGACATAAACCCGGGCCAT

GCAGATCTCAGTGCCAAAGAAGCACAGGATGTCATCATGGAGGTTGTTTTCCCAAATGAA

GTAGGAGCCAGGATCTTGACATCAGAATCACAATTAACAATAACAAGGGAAAAGAAGGAG

GAACTTCAGGATTGCAAGATTGCTCCTTTGATGGTGGCATACATGTTGGAAAGAGAACTG

GTTCGCAAGACCAGATTTTTACCAGTAGCTGGCGGAACAAGCAGCGTATACATCGAGGTA

TTGCATTTGACTCAAGGGACCTGCTGGGAACAAATGTACACACCAGGAGGGGAGGTGAGA

AATGATGATGTTGATCAGAGTTTGATCATTGCTGCTAGAAATATAGTTAGGAGGGCAACA

GTATCAGCAGACCCATTGGCTTCGCTCTTGGAAATGTGCCACAGTACACAAATTGGTGGA

GTGAGGATGGTGGACATTCTTAGGCAGAACCCAACAGAGGAGCAAGCTGTGGATATATGC

AAAGCAGCAATGGGTTTAAGAATCAGTTCATCCTTCAGCTTTGGAGGTTTCACTTTCAAA

AGGACAAGTGGGTCGTCTGTCAAAAGAGAAGAAGAAATACTCACTGGCAACCTCCAAACA

CTGAAAGTAAGAATACATGAAGGATATGAGGAATTCACAATGGTTGGGCGAAGAGCTACA

GCCATTTTGAGGAAAGCAACCAGGAGACTGATCCAATTAATAGTGAGTGGACGAGACGAG

CAGTCAATCGCTGAAGCAATCATAGTGGCAATGGTTTTCTCACAGGAGGATTGCATGATA

AAAGCAGTACGAGGTGATTTGAATTTTGTCAACAGAGCGAATCAGCGGCTAAATCCTATG

CATCAACTTCTGAGGCATTTCCAAAAGGATGCAAAAGTGCTGTTTCAAAACTGGGGGATT

GAACCAATTGACAATGTAATGGGGATGATCGGGATACTGCCTGACATGACCCCCAGCACA

GAGATGTCACTGAGAGGAGTGAGAGTCAGCAAAATGGGAGTGGATGAATATTCCAGTACT

GAGAGAGTGGTCGTGAGCATTGATCGCTTCTTGAGAGTCCGAGATCAGAGGGGAAATGTG

CTTCTGTCTCCTGAGGAAGTTAATGAAACACATGGAACAGAGAAACTGACGATAACGTAT

TCATCGTCTATGATGTGGGAAATCAATGGTCCGGAATCCGTGCTAGTCAACACATATCAA

TGGATCATTAGAAATTGGGAAACTGTGAAGATTCAGTGGTCCCAGGACCCTACGATGTTG

TACAATAAGATGGAATTTGAGCCCTTCCAATCCTTGGTGCCCAAGGCTGCTAGAGGCCAG

TATAGTGGGTTTGTGAGGACATTATTCCAACAGATGCGTGATGTGTTGGGGACATTTGAC

ACTGTCCAAATAATAAAGCTCCTACCATTTGCAGCAGCCCCACCGGAACAGAGTAGGATG

CAATTTTCCTCTCTGACTGTGAACGTAAGAGGTTCAGGAATGAGAATACTTGTGAGGGGC

AACTCCCCTGTGTTCAACTATAATAAGGCCACCAAGAGACTCACAGTTCTTGGAAAGGAT

GCAGGCGCCTTGACAGAATATCCAGATGAGGGAACAGCAGGAGTGGAGTCTGCAGTATTA

AGAGGATTTCTAATTCTGGGCAAAGAGGACAAAAGATATGGACCAGCATTGAGCATCAAC

GAATTGAGCAATCTTGCGAAAGGGGAAAAGGCTAATGTGTTGATAGGACAAGGAGACGTG

GTGTTGGTAATGAAACGGAAACGGGACTCTAGCATACTTACTGACAGCCAGACAGCGACC

AAAAGAATTCGGATGGCCATCAATTAGTGTCGAATTGTTTAAAAACGACCTTGTTTCTAC

T---------------------

>A_chicken_Omsk_0118_2020_EPI1813374

AGCAAAAGCAGGTCAAATATATTCAATATGGAGAGAATAAAAGAACTAAGAGATTTGATG

TCGCAGTCTCGCACTCGCGAGATACTAACAAAAACCACTGTGGACCATATGGCCATAATA

AAGAAATACACATCAGGAAGACAGGAGAAGAACCCTGCCCTCAGGATGAAATGGATGATG

GCAATGAAATATCCTATTACAGCTGACAAAAGAATAATGGAGATGATCCCTGAAAGGAAT

GAGCAAGGTCAGACTCTCTGGAGCAAAACAAATGATGCTGGATCAGACAGAGTGATGGTC

TCACCTCTGGCTGTGACATGGTGGAATAGAAATGGGCCAACAACAAGTACAGTACACTAC

CCAAAAGTCTACAAAACCTACTTTGAAAAGGTAGAAAGGTTGAAACATGGAACCTTTGGT

CCTGTTCACTTTCGAAATCAGGTTAAGATACGCCGCAGGGTTGACATAAACCCGGGCCAT

GCAGATCTCAGTGCCAAAGAAGCACAGGATGTCATCATGGAGGTTGTTTTCCCAAATGAA

GTTGGAGCCAGGATCTTGACATCAGAATCACAATTAACAATAACAAGGGAAAAGAAGGAG

GAACTTCAGGATTGCAAGATTGCTCCTTTGATGGTGGCATACATGTTGGAAAGAGAACTG

GTTCGCAAGACCAGATTTTTACCAGTAGCTGGCGGAACAAGCAGCGTATACATCGAGGTA

TTGCATTTGACTCAAGGGACCTGCTGGGAACAAATGTACACACCAGGAGGGGAGGTGAGA

AATGATGATGTTGATCAGAGTTTGATCATTGCTGCTAGAAATATAGTTAGGAGGGCAACA

GTATCAGCAGACCCATTGGCTTCGCTCTTGGAAATGTGCCACAGTACACAAATTGGTGGA

GTAAGGATGGTGGACATTCTTAGGCAGAACCCAACAGAGGAGCAAGCTGTGGATATATGC

AAAGCAGCAATGGGTTTAAGAATCAGTTCATCCTTCAGCTTTGGAGGTTTCACTTTCAAA

AGGACAAGTGGGTCGTCTGTCAAAAGAGAAGAAGAAATACTCACTGGCAACCTCCAAACA

CTGAAAGTAAGAATACATGAAGGATATGAGGAATTCACAATGGTTGGGCGAAGGGCTACA

GCCATTTTGAGGAAAGCAACCAGGAGACTGATCCAATTAATAGTGAGTGGAAGAGACGAG

CAGTCAATCGCTGAAGCAATCATAGTGGCAATGGTTTTCTCACAGGAGGATTGCATGATA

AAAGCAGTACGAGGTGATTTGAATTTTGTCAACAGAGCGAATCAGCGGCTAAATCCTATG

CATCAACTTCTGAGGCATTTCCAAAAGGATGCAAAAGTGCTGTTTCAAAACTGGGGGATT

GAACCAATTGACAATGTAATGGGGATGATCGGGATACTGCCCGACATGACCCCCAGCACA

GAGATGTCACTGAGAGGAGTGAGAGTCAGCAAAATGGGAGTGGATGAATATTCCAGTACT

GAGAGAGTGGTCGTGAGCATTGATCGCTTCTTGAGAGTCCGAGATCAGAGGGGAAATGTG

CTTCTGTCTCCTGAGGAAGTTAGTGAAACACATGGAACAGAGAAACTGACGATAACGTAT

TCATCGTCTATGATGTGGGAAATCAATGGTCCGGAATCCGTGCTAGTCAACACATATCAA

TGGATCATTAGAAGTTGGGAAACTGTGAAGATTCAATGGTCCCAGGACCCTACGATGTTG

TACAATAAGATGGAATTTGAGCCCTTCCAATCCTTGGTGCCCAAGGCTGCTAGAGGCCAG

TATAGTGGGTTTGTGAGGACATTATTCCAACAGATGCGTGATGTGTTGGGGACATTTGAC

ACTGTCCAAATAATAAAGCTCCTACCATTTGCAGCAGCCCCACCGGAACAGAGTAGGATG

CAATTTTCCTCTCTGACTGTGAACGTAAGAGGTTCAGGAATGAGAATACTTGTGAGGGGC

AACTCCCCTGTGTTCAACTATAATAAGGCCACCAAGAGACTCACAGTTCTTGGAAAGGAT

GCAGGCGCCTTGACAGAATATCCAGATGAGGGAACAGCAGGAGTGGAGTCTGCAGTATTA

AGAGGATTTCTAATTCTGGGCAAAGAGGACAAAAGATATGGACCAGCATTGAGCATCAAC

GAATTGAGCAATCTTGCGAAAGGGGAAAAGGCTAATGTGTTGATAGGACAAGGAGACGTG

GTGTTGGTAATGAAACGGAAACGGGACTCTAGCATACTTACTGACAGCCAGACAGCGACC

AAAAGAATTCGGATGGCCATCAATTAGTGTCGAATTGTTTAAAAACGACCTTGTTTCTAC

T---------------------

>A_chicken_Omsk_0119_2020_EPI1813382

AGCAAAAGCAGGTCAAATATATTCAATATGGAGAGAATAAAAGAACTAAGAGATTTGATG

TCGCAGTCTCGCACTCGCGAGATACTAACAAAAACCACTGTGGACCATATGGCCATAATA

AAGAAATACACATCAGGAAGACAGGAGAAGAACCCTGCCCTCAGGATGAAATGGATGATG

GCAATGAAATATCCTATTACAGCTGACAAAAGAATAATGGAGATGATCCCTGAAAGGAAT

GAGCAAGGTCAGACTCTCTGGAGCAAAACAAATGATGCTGGATCAGACAGAGTGATGGTC

TCACCTCTGGCTGTGACATGGTGGAATAGAAATGGGCCAACAACAAGTACAGTACACTAC

CCAAAAGTCTACAAAACCTACTTTGAAAAGGTAGAAAGGTTGAAACATGGAACCTTTGGT

CCTGTTCACTTTCGAAATCAGGTTAAGATACGCCGCAGGGTTGACATAAACCCGGGCCAT

GCAGATCTCAGTGCCAAAGAAGCACAGGATGTCATCATGGAGGTTGTTTTCCCAAATGAA

GTTGGAGCCAGGATCTTGACATCAGAATCACAATTAACAATAACAAGGGAAAAGAAGGAG

GAACTTCAGGATTGCAAGATTGCTCCTTTGATGGTGGCATACATGTTGGAAAGAGAACTG

GTTCGCAAGACCAGATTTTTACCAGTAGCTGGCGGAACAAGCAGCGTATACATCGAGGTA

TTGCATTTGACTCAAGGGACCTGCTGGGAACAAATGTACACACCAGGAGGGGAGGTGAGA

AATGATGATGTTGATCAGAGTTTGATCATTGCTGCTAGAAATATAGTTAGGAGGGCAACA

GTATCAGCAGACCCATTGGCTTCGCTCTTGGAAATGTGCCACAGTACACAAATTGGTGGA

GTAAGGATGGTGGACATTCTTAGGCAGAACCCAACAGAGGAGCAAGCTGTGGATATATGC

AAAGCAGCAATGGGTTTAAGAATCAGTTCATCCTTCAGCTTTGGAGGTTTCACTTTCAAA

AGGACAAGTGGGTCGTCTGTCAAAAGAGAAGAAGAAATACTCACTGGCAACCTCCAAACA

CTGAAAGTAAGAATACATGAAGGATATGAGGAATTCACAATGGTTGGGCGAAGAGCTACA

GCCATTTTGAGGAAAGCAACCAGGAGACTGATCCAATTAATAGTGAGTGGAAGAGACGAG

CAGTCAATCGCTGAAGCAATCATAGTGGCAATGGTTTTCTCACAGGAGGATTGCATGATA

AAAGCAGTACGAGGTGATTTGAATTTTGTCAACAGAGCGAATCAGCGGCTAAATCCTATG

CATCAACTTCTGAGGCATTTCCAAAAGGATGCAAAAGTGCTGTTTCAAAACTGGGGGATT

GAACCAATTGACAATGTAATGGGGATGATCGGGATACTGCCCGACATGACCCCCAGCACA

GAGATGTCACTGAGAGGAGTGAGAGTCAGCAAAATGGGAGTGGATGAATATTCCAGTACT

GAGAGAGTGGTCGTGAGCATTGATCGCTTCTTGAGAGTCCGAGATCAGAGGGGAAATGTG

CTTCTGTCTCCTGAGGAAGTTAGTGAAACACATGGAACAGAGAAACTGACGATAACGTAT

TCATCGTCTATGATGTGGGAAATCAATGGTCCGGAATCCGTGCTAGTCAACACATATCAA

TGGATCATTAGAAGTTGGGAAACTGTGAAGATTCAATGGTCCCAGGACCCTACGATGTTG

TACAATAAGATGGAATTTGAGCCCTTCCAATCCTTGGTGCCCAAGGCTGCTAGAGGCCAG

TATAGTGGGTTTGTGAGGACATTATTCCAACAGATGCGTGATGTGTTGGGGACATTTGAC

ACTGTCCAAATAATAAAGCTCCTACCATTTGCAGCAGCCCCACCGGAACAGAGTAGGATG

CAATTTTCCTCTCTGACTGTGAACGTAAGAGGTTCAGGAATGAGAATACTTGTGAGGGGC

AACTCCCCTGTGTTCAACTATAATAAGGCCACCAAGAGACTCACAGTTCTTGGAAAGGAT

GCAGGCGCCTTGACAGAATATCCAGATGAGGGAACAGCAGGAGTGGAGTCTGCAGTATTA

AGAGGATTTCTAATTCTGGGCAAAGAGGACAAAAGATATGGACCAGCATTGAGCATCAAC

GAATTGAGCAATCTTGCGAAAGGGGAAAAGGCTAATGTGTTGATAGGACAAGGAGACGTG

GTGTTGGTAATGAAACGGAAACGGGACTCTAGCATACTTACTGACAGCCAGACAGCGACC

AAAAGAATTCGGATGGCCATCAATTAGTGTCGAATTGTTTAAAAACGACCTTGTTTCTAC

T---------------------

>A_chicken_Omsk_0073_2020_EPI1813406

AGCAAAAGCAGGTCAAATATATTCAATATGGAGAGAATAAAAGAACTAAGAGATTTGATG

TCGCAGTCTCGCACTCGCGAGATACTAACAAAAACCACTGTGGACCATATGGCCATAATA

AAGAAATACACATCAGGAAGACAGGAGAAGAACCCTGCCCTCAGGATGAAATGGATGATG

GCAATGAAATATCCTATTACAGCTGACAAAAGAATAATGGAGATGATCCCTGAAAGGAAT

GAGCAAGGTCAGACTCTCTGGAGCAAAACAAATGATGCTGGATCAGACAGAGTGATGGTC

TCACCTCTGGCTGTGACATGGTGGAATAGAAATGGGCCAACAACAAGTACAGTACACTAC

CCAAAAGTCTACAAAACCTACTTTGAAAAGGTAGAAAGGTTGAAACATGGAACCTTTGGT

CCTGTTCACTTTCGAAATCAGGTTAAGATACGCCGCAGGGTTGACATAAACCCGGGCCAT

GCAGATCTCAGTGCCAAAGAAGCACAGGATGTCATCATGGAGGTTGTTTTCCCAAATGAA

GTTGGAGCCAGGATCTTGACATCAGAATCACAATTAACAATAACAAGGGAAAAGAAGGAG

GAACTTCAGGATTGCAAGATTGCTCCTTTGATGGTGGCATACATGTTGGAAAGAGAACTG

GTTCGCAAGACCAGATTTTTACCAGTAGCTGGCGGAACAAGCAGCGTATACATCGAGGTA

TTGCATTTGACTCAAGGGACCTGCTGGGAACAAATGTACACACCAGGAGGGGAGGTGAGA

AATGATGATGTTGATCAGAGTTTGATCATTGCTGCTAGAAATATAGTTAGGAGGGCAACA

GTATCAGCAGACCCATTGGCTTCGCTCTTGGAAATGTGCCACAGTACACAAATTGGTGGA

GTAAGGATGGTGGACATTCTTAGGCAGAACCCAACAGAGGAGCAAGCTGTGGATATATGC

AAAGCAGCAATGGGTTTAAGAATCAGTTCATCCTTCAGCTTTGGAGGTTTCACTTTCAAA

AGGACAAGTGGGTCGTCTGTCAAAAGAGAAGAAGAAATACTCACTGGCAACCTCCAAACA

CTGAAAGTAAGAATACATGAAGGATATGAGGAATTCACAATGGTTGGGCGAAGAGCTACA

GCCATTTTGAGGAAAGCAACCAGGAGACTGATCCAATTAATAGTGAGTGGAAGAGACGAG

CAGTCAATCGCTGAAGCAATCATAGTGGCAATGGTTTTCTCACAGGAGGATTGCATGATA

AAAGCAGTACGAGGTGATTTGAATTTTGTCAACAGAGCGAATCAGCGGCTAAATCCTATG

CATCAACTTCTGAGGCATTTCCAAAAGGATGCAAAAGTGCTGTTTCAAAACTGGGGGATT

GAACCAATTGACAATGTAATGGGGATGATCGGGATACTGCCTGACATGACCCCCAGCACA

GAGATGTCACTGAGAGGAGTGAGAGTCAGCAAAATGGGAGTGGATGAATATTCCAGTACT

GAGAGAGTGGTCGTGAGCATTGATCGCTTCTTGAGAGTCCGAGATCAGAGGGGAAATGTG

CTTCTGTCTCCTGAGGAAGTTAGTGAAACACATGGAACAGAGAAACTGACGATAACGTAT

TCATCGTCTATGATGTGGGAAATCAATGGTCCGGAATCCGTGCTAGTCAACACATATCAA

TGGATCATTAGAAATTGGGAAACTGTGAAGATTCAGTGGTCCCAGGACCCTACGATGTTG

TACAATAAGATGGAATTTGAGCCCTTCCAATCCTTGGTGCCCAAGGCTGCTAGAGGCCAG

TATAGTGGGTTTGTGAGGACATTATTCCAACAGATGCGTGATGTGTTGGGGACATTTGAC

ACTGTCCAAATAATAAAGCTCCTACCATTTGCAGCAGCCCCACCTGAACAGAGTAGGATG

CAATTTTCCTCTCTGACTGTGAACGTAAGAGGTTCAGGAATGAGAATACTTGTGAGGGGC

AACTCCCCTGTGTTCAACTATAATAAGGCCACCAAGAGACTCACAGTTCTTGGAAAGGAT

GCAGGCGCCTTGACAGAATATCCAGATGAGGGAACAGCAGGAGTGGAGTCTGCAGTATTA

AGAGGATTTCTAATTCTGGGCAAAGAGGACAAAAGATATGGACCAGCATTGAGCATCAAC

GAATTGAGCAATCTTGCGAAAGGGGAAAAGGCTAATGTGTTGATAGGACAAGGAGACGTG

GTGTTGGTAATGAAACGGAAACGGGACTCTAGCATACTTACTGACAGCCAGACAGCGACC

AAAAGAATTCGGATGGCCATCAATTAGTGTCGAATTGTTTAAAAACGACCTTGTTTCTAC

T---------------------

>A_chicken_Omsk_30007_2020_EPI1814310

AGCAAAAGCAGGTCAAATATATTCAATATGGAGAGAATAAAAGAACTAAGAGATTTGATG

TCGCAGTCTCGCACTCGCGAGATACTAACAAAAACCACTGTGGACCATATGGCCATAATA

AAGAAATACACATCAGGGAGACAGGAGAAGAACCCTGCCCTCAGGATGAAATGGATGATG

GCAATGAAATATCCTATTACAGCTGACAAAAGAATAATGGAGATGATCCCTGAAAGGAAT

GAGCAAGGTCAGACTCTCTGGAGCAAAACAAATGATGCTGGATCAGACAGAGTGATGGTC

TCACCTCTGGCTGTGACATGGTGGAATAGAAATGGGCCAACAACAAGTACAGTACACTAC

CCAAAAGTCTACAAAACCTACTTTGAAAAGGTAGAAAGGTTGAAACATGGAACCTTTGGT

CCTGTTCACTTTCGAAATCAGGTTAAGATACGCCGCAGGGTTGACATAAACCCGGGCCAT

GCAGATCTCAGTGCCAAAGAAGCACAGGATGTCATCATGGAGGTTGTTTTCCCAAATGAA

GTTGGAGCCAGGATCTTGACATCAGAATCACAATTAACAATAACAAGGGAAAAGAAGGAG

GAACTTCAGGATTGCAAGATTGCTCCTTTGATGGTGGCATACATGTTGGAAAGAGAACTG

GTTCGCAAGACCAGATTTTTACCAGTAGCTGGCGGAACAAGCAGCGTATACATCGAGGTA

TTGCATTTGACTCAAGGGACCTGCTGGGAACAAATGTACACACCAGGAGGGGAGGTGAGA

AATGATGATGTTGATCAGAGTTTGATCATTGCTGCTAGAAATATAGTTAGGAGGGCAACA

GTATCAGCAGACCCATTGGCTTCGCTCTTGGAAATGTGCCACAGTACACAAATTGGTGGA

GTGAGGATGGTGGACATTCTTAGGCAGAACCCAACAGAGGAGCAAGCTGTGGATATATGC

AAAGCAGCAATGGGTTTAAGAATCAGTTCATCCTTCAGCTTTGGAGGTTTCACTTTCAAA

AGGACAAGTGGGTCGTCTGTCAAAAGAGAAGAAGAAATACTCACTGGCAACCTCCAAACA

CTGAAAGTAAGAATACATGAAGGATATGAGGAATTCACAATGGTTGGGCGAAGAGCTACA

GCCATTTTGAGGAAAGCAACCAGGAGACTGATCCAATTAATAGTGAGTGGACGAGACGAG

CAGTCAATCGCTGAAGCAATCATAGTGGCAATGGTTTTCTCACAGGAGGATTGCATGATA

AAAGCAGTACGAGGTGATTTGAATTTTGTCAACAGAGCGAATCAGCGGCTAAATCCTATG

CATCAACTTCTGAGGCATTTCCAAAAGGATGCAAAAGTGCTGTTTCAAAACTGGGGGATT

GAACCAATTGACAATGTAATGGGGATGATCGGGATACTGCCTGACATGACCCCCAGCACA

GAGATGTCACTGAGAGGAGTGAGAGTCAGCAAAATGGGAGTGGATGAATATTCCAGTACT

GAGAGAGTGGTCGTGAGCATTGATCGCTTCTTGAGAGTCCGAGATCAGAGGGGAAATGTG

CTTCTGTCTCCTGAGGAAGTTAATGAAACACATGGAACAGAGAAACTGACGATAACGTAT

TCATCGTCTATGATGTGGGAAATCAATGGTCCGGAATCCGTGCTAGTCAACACATATCAA

TGGATCATTAGAAATTGGGAAACTGTGAAGATTCAGTGGTCCCAGGACCCTACGATGTTG

TACAATAAGATGGAATTTGAGCCCTTCCAATCCTTGGTGCCCAAGGCTGCTAGAGGCCAG

TATAGTGGGTTTGTGAGGACATTATTCCAACAGATGCGTGATGTGTTGGGGACATTTGAC

ACTGTCCAAATAATAAAGCTCCTACCATTTGCAGCAGCCCCACCGGAACAGAGTAGGATG

CAATTTTCCTCCCTGACTGTGAACGTAAGAGGTTCAGGAATGAGAATACTTGTGAGGGGC

AACTCCCCTGTGTTCAACTATAATAAGGCCACCAAGAGACTCACAGTTCTTGGAAAGGAT

GCAGGCGCCTTGACAGAATATCCAGATGAGGGAACAGCAGGAGTGGAGTCTGCAGTATTA

AGAGGATTTCTAATTCTGGGCAAAGAGGACAAAAGATATGGACCAGCATTGAGCATCAAC

GAATTGAGCAATCTTGCGAAAGGGGAAAAGGCTAATGTGTTGATAGGACAAGGAGACGTG

GTGTTGGTAATGAAACGGAAACGGGACTCTAGCATACTTACTGACAGCCAGACAGCGACC

AAAAGAATTCGGATGGCCATCAATTAGTGTCGAATTGTTTAAAAACGACCTTGTTTCTAC

T---------------------

>A_goose_Omsk_30009_2020_EPI1814318

AGCAAAAGCAGGTCAAATATATTCAATATGGAGAGAATAAAAGAACTAAGAGATTTGATG

TCGCAGTCTCGCACTCGCGAGATACTAACAAAAACCACTGTGGACCATATGGCCATAATA

AAGAAATACACATCAGGGAGACAGGAGAAGAACCCTGCCCTCAGGATGAAATGGATGATG

GCAATGAAATATCCTATTACAGCTGACAAAAGAATAATGGAGATGATCCCTGAAAGGAAT

GAGCAAGGTCAGACTCTCTGGAGCAAAACAAATGATGCTGGGTCAGACAGAGTGATGGTC

TCACCTCTGGCTGTGACATGGTGGAATAGAAATGGGCCAACAACAAGTACAGTACACTAC

CCAAAAGTCTACAAAACCTACTTTGAAAAGGTAGAAAGGTTGAAACATGGAACCTTTGGT

CCTGTTCACTTTCGAAATCAGGTTAAGATACGCCGCAGGGTTGACATAAACCCGGGCCAT

GCAGATCTCAGTGCCAAAGAAGCACAGGATGTCATCATGGAGGTTGTTTTCCCAAATGAA

GTTGGAGCCAGGATCTTGACATCAGAATCACAATTAACAATAACAAGGGAAAAGAAGGAG

GAACTTCAGGATTGCAAGATTGCTCCTTTGATGGTGGCATACATGTTGGAAAGAGAACTG

GTTCGCAAGACCAGATTTTTACCAGTAGCTGGCGGAACAAGCAGCGTATACATCGAGGTA

TTGCATTTGACTCAAGGGACCTGCTGGGAACAAATGTACACACCAGGAGGGGAGGTGAGA

AATGATGATGTTGATCAGAGCTTGATCATTGCTGCTAGAAATATAGTTAGGAGGGCAACA

GTATCAGCAGACCCATTGGCTTCGCTCTTGGAAATGTGCCACAGTACACAAATTGGTGGA

GTGAGGATGGTGGACATTCTTAGGCAGAACCCAACAGAGGAGCAAGCTGTGGATATATGC

AAAGCAGCAATGGGTTTAAGAATCAGTTCATCCTTCAGCTTTGGAGGTTTCACTTTCAAA

AGGACAAGTGGGTCGTCTGTCAAAAGAGAAGAAGAAATACTCACTGGCAACCTCCAAACA

CTGAAAGTAAGAATACATGAAGGATATGAGGAATTCACAATGGTTGGGCGAAGAGCTACA

GCCATTTTGAGGAAAGCAACCAGGAGACTGATCCAATTAATAGTGAGTGGACGAGACGAG

CAGTCAATCGCTGAAGCAATCATAGTGGCAATGGTTTTCTCACAGGAGGATTGCATGATA

AAAGCAGTACGAGGTGATTTGAATTTTGTCAACAGAGCGAATCAGCGGCTAAATCCTATG

CATCAACTTCTGAGGCATTTCCAAAAGGATGCAAAAGTGCTGTTTCAAAACTGGGGGATT

GAACCAATTGACAATGTAATGGGGATGATCGGGATACTGCCTGACATGACCCCCAGCACA

GAGATGTCACTGAGAGGAGTGAGAGTCAGCAAAATGGGAGTGGATGAATATTCCAGTACT

GAGAGAGTGGTCGTGAGCATTGATCGCTTCTTGAGAGTCCGAGATCAGAGGGGAAATGTG

CTTCTGTCTCCTGAGGAAGTTAATGAAACACATGGAACAGAGAAACTGACGATAACGTAT

TCATCGTCTATGATGTGGGAAATCAATGGTCCGGAATCCGTGCTAGTCAACACATATCAA

TGGATCATTAGAAATTGGGAAACTGTGAAGATTCAGTGGTCCCAGGACCCTACGATGTTG

TACAATAAGATGGAATTTGAGCCCTTCCAATCCTTGGTGCCCAAGGCTGCTAGAGGCCAG

TATAGTGGGTTTGTGAGGACATTATTCCAACAGATGCGTGATGTGTTGGGGACATTTGAC

ACTGTCCAAATAATAAAGCTCCTACCATTTGCAGCAGCCCCACCGGAACAGAGTAGGATG

CAATTTTCCTCTCTGACTGTGAACGTAAGAGGTTCAGGAATGAGAATACTTGTGAGGGGC

AACTCCCCTGTGTTCAACTATAATAAGGCCACCAAGAGACTCACAGTTCTTGGAAAGGAT

GCAGGCGCCTTGACAGAATATCCAGATGAGGGAACAGCAGGAGTGGAGTCTGCAGTATTA

AGAGGATTTCTAATTCTGGGCAAAGAGGACAAAAGATATGGACCAGCATTGAGCATCAAC

GAATTGAGCAATCTTGCGAAAGGGGAAAAGGCTAATGTGTTGATAGGACAAGGAGACGTG

GTGTTGGTAATGAAACGGAAACGGGACTCTAGCATACTTACTGACAGCCAGACAGCGACC

AAAAGAATTCGGATGGCCATCAATTAGTGTTGAATTGTTTAAAAACGACCTTGTTTCTAC

T---------------------

>A_chicken_Chelyabinsk_201_2020_EPI1814334

AGCAAAAGCAGGTCAAATATATTCAATATGGAGAGAATAAAAGAACTAAGAGATTTGATG

TCGCAGTCTCGCACTCGCGAGATACTAACAAAAACCACTGTGGACCATATGGCCATAATA

AAGAAATACACATCAGGAAGACAGGAGAAGAACCCTGCCCTCAGGATGAAATGGATGATG

GCAATGAAATATCCTATTACAGCTGACAAAAGAATAATGGAGATGATCCCTGAAAGGAAT

GAGCAAGGTCAGACTCTCTGGAGCAAAACAAATGATGCTGGATCAGACAGAGTGATGGTC

TCACCTCTGGCTGTGACATGGTGGAATAGAAATGGGCCAACAACAAGTACAGTACACTAC

CCAAAAGTCTACAAAACCTACTTTGAAAAGGTAGAAAGGTTGAAACATGGAACCTTTGGT

CCTGTTCACTTTCGAAATCAGGTTAAGATACGCCGCAGGGTTGACATAAACCCGGGCCAT

GCAGATCTCAGTGCCAAAGAAGCACAGGATGTCATCATGGAGGTTGTTTTCCCAAATGAA

GTTGGAGCCAGGATCTTGACATCAGAATCACAATTAACAATAACAAGGGAAAAGAAGGAG

GAACTTCAGGATTGCAAGATTGCTCCTTTGATGGTGGCATACATGTTGGAAAGAGAACTG

GTTCGCAAGACCAGATTTTTACCAGTAGCTGGCGGAACAAGCAGCGTATACATCGAGGTA

TTGCATTTGACTCAAGGGACCTGCTGGGAACAAATGTACACACCAGGAGGGGAGGTGAGA

AATGATGATGTTGATCAGAGTTTGATCATTGCTGCTAGAAATATAGTTAGGAGGGCAACA

GTATCAGCAGACCCATTGGCTTCGCTCTTGGAAATGTGCCACAGTACACAAATTGGTGGA

GTAAGGATGGTGGACATTCTTAGGCAGAACCCAACAGAGGAGCAAGCTGTGGATATATGC

AAAGCAGCAATGGGTTTAAGAATCAGTTCATCCTTCAGCTTTGGAGGTTTCACTTTCAAA

AGGACAAGTGGGTCGTCTGTCAAAAGAGAAGAAGAAATACTCACTGGCAACCTCCAAACA

CTGAAAGTAAGAATACATGAAGGATATGAGGAATTCACAATGGTTGGGCGAAGAGCTACA

GCCATTTTGAGGAAAGCAACCAGGAGACTGATCCAATTAATAGTGAGTGGAAGAGACGAA

CAGTCAATCGCTGAAGCAATCATAGTGGCAATGGTTTTCTCACAGGAGGATTGCATGATA

AAAGCAGTACGAGGTGATTTGAATTTTGTCAACAGAGCGAATCAGCGGCTAAATCCTATG

CATCAACTTCTGAGGCATTTCCAAAAGGATGCAAAAGTGCTGTTTCAAAACTGGGGGATT

GAACCAATTGACAATGTAATGGGGATGATCGGGATACTGCCTGACATGACCCCCAGCACA

GAGATGTCACTGAGAGGAGTGAGAGTCAGCAAAATGGGAGTGGATGAATATTCCAGTACT

GAGAGAGTGGTCGTGAGCATTGATCGCTTCTTGAGAGTCCGAGATCAGAGGGGAAATGTG

CTTCTGTCTCCTGAGGAAGTTAGTGAAACACATGGAACAGAGAAACTGACGATAACGTAT

TCATCGTCTATGATGTGGGAAATCAATGGTCCGGAATCCGTGCTAGTCAACACATATCAA

TGGATCATTAGAAATTGGGAAACTGTGAAGATTCAGTGGTCCCAGGACCCTACGATGTTG

TACAATAAGATGGAATTTGAGCCCTTCCAATCCTTGGTGCCCAAGGCTGCTAGAGGCCAG

TATAGTGGGTTTGTGAGGACATTATTCCAACAGATGCGTGATGTGTTGGGGACATTTGAC

ACTGTCCAAATAATAAAGCTCCTACCATTTGCAGCAGCCCCACCGGAACAGAGTAGGATG

CAATTTTCCTCTCTGACTGTGAACGTAAGAGGTTCAGGAATGAGAATACTTGTGAGGGGC

AACTCCCCTGTGTTCAACTATAATAAGGCCACCAAGAGACTCACAGTTCTTGGAAAGGAT

GCAGGCGCCTTGACAGAATATCCAGACGAGGGAACAGCAGGAGTGGAGTCTGCAGTATTA

AGAGGATTTCTAATTCTGGGCAAAGAGGACAAAAGATATGGACCAGCATTGAGCATCAAC

GAATTGAGCAATCTTGCGAAAGGGGAAAAGGCTAATGTGTTGATAGGACAAGGAGACGTG

GTGTTGGTAATGAAACGGAAACGGGACTCTAGCATACTTACTGACAGCCAGACAGCGACC

AAAAGAATTCGGATGGCCATCAATTAGTGTCGAATTGTTTAAAAACGACCTTGTTTCTAC

T---------------------

>A_chicken_Kurgan_1005_2020_EPI1814366

AGCAAAAGCAGGTCAAATATATTCAATATGGAGAGAATAAAAGAACTAAGAGATTTGATG

TCGCAGTCTCGCACTCGCGAGATACTAACAAAAACCACTGTGGACCATATGGCCATAATA

AAGAAATACACATCAGGAAGACAGGAGAAGAACCCTGCCCTCAGGATGAAATGGATGATG

GCAATGAAATATCCTATTACAGCTGACAAAAGAATAATGGAGATGATCCCTGAAAGGAAT

GAGCAAGGTCAGACTCTCTGGAGCAAAACAAATGATGCTGGATCAGACAGAGTGATGGTC

TCACCTCTGGCTGTGACATGGTGGAATAGAAATGGGCCAACAACAAGTACAGTACACTAC

CCAAAAGTCTACAAAACCTACTTTGAAAAGGTAGAAAGGTTGAAACATGGAACCTTTGGT

CCTGTTCACTTTCGAAATCAGGTTAAGATACGCCGCAGGGTTGACATAAACCCGGGCCAT

GCAGATCTCAGTGCCAAAGAAGCACAGGATGTCATCATGGAGGTTGTTTTCCCAAATGAA

GTTGGAGCCAGGATCTTGACATCAGAATCACAATTAACAATAACAAGGGAAAAGAAGGAG

GAACTTCAGGATTGCAAGATTGCTCCTTTGATGGTGGCATACATGTTGGAAAGAGAACTG

GTTCGCAAGACCAGATTTTTACCAGTAGCTGGCGGAACAAGCAGCGTATACATCGAGGTA

TTGCATTTGACTCAAGGGACCTGCTGGGAACAAATGTACACACCAGGAGGGGAGGTGAGA

AATGATGATGTTGATCAGAGTTTGATCATTGCTGCTAGAAATATAGTTAGGAGGGCAACA

GTATCAGCAGACCCATTGGCTTCGCTCTTGGAAATGTGCCACAGTACACAAATTGGTGGA

GTAAGGATGGTGGACATTCTTAGGCAGAACCCAACAGAGGAGCAAGCTGTGGATATATGC

AAAGCAGCAATGGGTTTAAGAATCAGTTCATCCTTCAGCTTTGGAGGTTTCACTTTCAAA

AGGACAAGTGGGTCGTCTGTCAAAAGAGAAGAAGAAATACTCACTGGCAACCTCCAAACA

CTGAAAGTAAGAATACATGAAGGATATGAGGAATTCACAATGGTTGGGCGAAGAGCTACA

GCCATTTTGAGGAAAGCAACCAGGAGACTGATCCAATTAATAGTGAGTGGAAGAGACGAA

CAGTCAATCGCTGAAGCAATCATAGTGGCAATGGTTTTCTCACAGGAGGATTGCATGATA

AAAGCAGTACGAGGTGATTTGAATTTTGTCAACAGAGCGAATCAGCGGCTAAATCCTATG

CATCAACTTCTGAGGCATTTCCAAAAGGATGCAAAAGTGCTGTTTCAAAACTGGGGGATT

GAACCAATTGACAATGTAATGGGGATGATCGGGATACTGCCTGACATGACCCCCAGCACA

GAGATGTCACTGAGAGGAGTGAGAGTCAGCAAAATGGGAGTGGATGAATATTCCAGTACT

GAGAGAGTGGTCGTGAGCATTGATCGCTTCTTGAGAGTCCGAGATCAGAGGGGAAATGTG

CTTCTATCTCCTGAGGAAGTTAGTGAAACACATGGAACAGAGAAACTGACGATAACGTAT

TCATCGTCTATGATGTGGGAAATCAATGGTCCGGAATCCGTGCTAGTCAACACATATCAA

TGGATCATTAGAAATTGGGAAACTGTGAAGATTCAGTGGTCCCAGGACCCTACGATGTTG

TACAATAAGATGGAATTTGAGCCCTTCCAATCCTTGGTGCCCAAGGCTGCTAGAGGCCAG

TATAGTGGGTTTGTGAGGACATTATTCCAACAGATGCGTGATGTGTTGGGGACATTTGAC

ACTGTCCAAATAATAAAGCTCCTACCATTTGCAGCAGCCCCACCGGAACAGAGTAGGATG

CAATTTTCCTCTCTGACTGTGAACGTAAGAGGTTCAGGAATGAGGATACTTGTGAGGGGC

AACTCCCCTGTGTTCAACTATAATAAGGCCACCAAGAGACTCACAGTTCTTGGAAAGGAT

GCAGGCGCCTTGACAGAATATCCAGACGAGGGAACAGCAGGAGTGGAGTCTGCAGTATTA

AGAGGATTTCTAATTCTGGGCAAAGAGGACAAAAGATATGGACCAGCATTGAGCATCAAC

GAATTGAGCAATCTTGCGAAAGGGGAAAAGGCTAATGTGTTGATAGGACAAGGAGACGTG

GTGTTGGTAATGAAACGGAAACGGGACTCTAGCATACTTACTGACAGCCAGACAGCGACC

AAAAGAATTCGGATGGCCATCAATTAGTGTCGAATTGTTTAAAAACGACCTTGTTTCTAC

T---------------------

>A_turkey_Poland_464_2020_EPI1841307

------------TCAAATATATTCAATATGGAGAGAATAAAAGAACTAAGAGATTTGATG

TCGCAGTCTCGCACTCGCGAGATACTAACAAAAACCACTGTGGACCATATGGCCATAATA

AAGAAATACACATCAGGAAGRCAGGAGAAGAACCCTGCCCTCAGGATGAAATGGATGATG

GCAATGAAATATCCTATTACAGCTGACAAAAGAATAATGGAGATGATCCCTGAAAGGAAT

GAGCAAGGTCAGACTCTCTGGAGCAAAACAAATGATGCTGGATCAGACAGAGTGATGGTC

TCACCCCTGGCTGTGACATGGTGGAATAGAAATGGGCCAACAACAAGTACAGTACACTAC

CCAAAAGTCTACAAAACCTACTTTGAAAAGGTAGAAAGGTTGAAACATGGAACCTTTGGT

CCTGTTCACTTTCGAAATCAGGTTAAGATACGCCGCAGGGTTGACATAAACCCGGGCCAT

GCAGATCTCAGTGCCAAAGAAGCACAGGATGTCATCATGGAGGTTGTTTTCCCAAATGAA

GTTGGAGCCAGGATCTTGACATCAGAATCACAATTAACAATAACAAGAGAAAAGAAGGAG

GAACTTCAGGATTGCAAGATTGCTCCTTTGATGGTGGCATACATGTTGGAAAGAGAACTG

GTTCGCAAGACCAGATTTTTACCAGTAGCTGGCGGAACAAGCAGCGTATACATCGAGGTA

TTGCATTTGACTCAAGGGACCTGCTGGGAACAAATGTACACACCAGGAGGGGAGGTGAGA

AATGATGATGTTGATCAGAGTTTGATCATTGCTGCTAGAAATATAGTTAGGAGGGCAACA

GTATCAGCAGACCCTTTGGCTTCGCTCTTGGAAATGTGCCACAGTACACAAATTGGTGGA

GTAAGGATGGTGGACATTCTTAGGCAGAACCCAACAGAGGAGCAAGCTGTGGATATATGC

AAAGCAGCAATGGGTTTAAGAATCAGTTCATCCTTCAGCTTTGGAGGTTTCACTTTCAAA

AGGACAAGTGGGTCGTCTGTCAAAAGAGAAGAAGAAATACTCACTGGCAACCTCCAAACA

CTGAAAGTAAGAATACATGAAGGATATGAGGAATTCACAATGGTTGGGCGAAGAGCTACA

GCCATTTTGAGGAAAGCAACCAGGAGACTGATCCAATTAATAGTGAGTGGAAGAGACGAG

CAGTCAATCGCTGAAGCAATCATAGTGGCAATGGTTTTCTCACAGGAGGATTGCATGATA

AAAGCAGTACGAGGTGATTTGAATTTTGTCAACAGAGCGAATCAGCGGCTAAATCCTATG

CATCAACTTCTGAGGCATTTCCAAAAGGATGCAAAAGTGCTGTTTCAAAACTGGGGGATT

GAACCAATTGACAATGTAATGGGGATGATCGGGATACTGCCTGACATGACCCCCAGCACA

GAGATGTCACTGAGAGGAGTGAGAGTCAGCAAAATGGGAGTGGATGAATATTCCAGTACT

GAGAGAGTGGTCGTGAGCATTGATCGCTTCTTGAGAGTCCGAGATCAGAGGGGAAATGTG

CTTTTGTCTCCTGAGGAAGTTAGTGAAACACATGGAACAGAGAAACTGACGATAACGTAT

TCATCGTCTATGATGTGGGAAATCAATGGTCCGGAATCCGTGCTAGTCAACACATATCAA

TGGATCATTAGAAGTTGGGAAACTGTGAAGATTCAATGGTCCCAGGACCCTACGATGCTG

TACAATAAGATGGAATTTGAGCCCTTCCAATCCTTGGTGCCCAAGGCTGCTAGAGGCCAG

TATAGTGGGTTTGTGAGGACATTATTCCAACAGATGCGTGATGTGTTGGGGACATTTGAC

ACTGTCCAAATAATAAAGCTCCTACCATTTGCAGCAGCCCCACCGGAACAGAGTAGGATG

CAATTTTCCTCTCTGACTGTGAACGTAAGAGGTTCAGGAATGAGAATACTTGTGAGGGGC

AACTCCCCTGTGTTCAACTATAATAAGGCCACCAAGAGACTCACAGTTCTTGGAAAGGAT

GCAGGCGCCTTGACAGAATATCCAGATGAGGGAACAGCAGGAGTTGAGTCTGCAGTATTA

AGAGGATTTCTAATTCTGGGCAAAGAGGACAAAAGATATGGACCAGCATTGAGCATCAAC

GAATTGAGCAATCTTGCGAAAGGGGAAAAGGCTAATGTGTTGATAGGACAAGGAGACGTG

GTGTTGGTAATGAAACGGAAACGGGACTCTAGCATACTTACTGACAGCCAGACAGCGACC

AAAAGAATTCGGATGGCCATCAATTAGTGTCGAATTGTTTAAAAACGA------------

----------------------

>A_duck_Northern_China_ZGL_2020_EPI1844088

---------------------------ATGGAGAGAATAAAAGAACTAAGAGATTTGATG

TCGCAGTCTCGCACTCGCGAGATACTAACAAAAACCACTGTGGACCATATGGCCATAATA

AAGAAATACACATCAGGAAGACAGGAGAAGAACCCTGCCCTCAGGATGAAATGGATGATG

GCAATGAAATATCCTATTACAGCTGACAAAAGAATAATGGAGATGATCCCTGAAAGGAAT

GAGCAAGGTCAGACTCTCTGGAGCAAAACAAATGATGCTGGATCGGACAGAGTGATGGTC

TCACCTCTGGCTGTGACATGGTGGAATAGGAATGGGCCAACAACAAGTACAGTACACTAC

CCAAAAGTCTACAAAACCTACTTTGAAAAGGTAGAAAGGTTGAAACATGGAACCTTTGGT

CCTGTTCACTTTCGAAATCAGGTTAAGATACGCCGCAGGGTTGACATAAACCCGGGCCAT

GCAGATCTCAGTGCCAAAGAAGCACAGGATGTCATCATGGAGGTTGTTTTCCCAAATGAA

GTTGGAGCCAGGATCTTGACATCAGAATCACAATTAACAATAACAAGGGAAAAGAAGGAG

GAACTTCAGGATTGCAAGATTGCTCCTTTGATGGTGGCATACATGTTGGAAAGAGAACTG

GTTCGCAAGACCAGATTTTTACCAGTAGCTGGCGGAACAAGCAGCGTATACATCGAGGTA

TTGCATTTGACTCAAGGGACCTGCTGGGAACAAATGTACACACCAGGAGGGGAGGTGAGA

AATGATGATGTTGATCAGAGTTTGATCATTGCTGCTAGAAATATAGTTAGGAGGGCAACA

GTATCAGCAGACCCATTGGCTTCGCTCTTGGAAATGTGCCACAGTACACAAATTGGTGGA

GTAAGGATGGTGGACATTCTTAGGCAGAACCCAACAGAGGAGCAAGCTGTGGATATATGC

AAAGCAGCAATGGGTTTAAGAATCAGTTCATCCTTCAGCTTTGGAGGTTTCACTTTCAAA

AGGACAAGTGGGTCGTCTGTCAAAAGAGAAGAAGAAATACTCACTGGCAACCTCCAAACA

CTGAAAGTAAGAATACATGAAGGATATGAGGAATTCACAATGGTTGGGCGAAGAGCTACA

GCCATTTTGAGGAAAGCAACCAGGAGACTGATCCAATTAATAGTGAGTGGAAGAGACGAG

CAGTCAATCGCTGAAGCAATCATAGTGGCAATGGTTTTCTCACAGGAGGATTGCATGATA

AAAGCAGTACGAGGTGATTTGAATTTTGTCAACAGAGCGAATCAGCGGCTAAATCCTATG

CATCAACTTCTGAGGCATTTCCAAAAGGATGCAAAAGTGCTGTTTCAAAACTGGGGGATT

GAACCAATTGACAATGTAATGGGGATGATCGGGATACTGCCAGACATGACCCCCAGCACA

GAGATGTCACTGAGAGGAGTGAGAGTCAGCAAAATGGGAGTGGATGAATATTCCAGTACT

GAGAGAGTGGTCGTGAGCATTGATCGCTTCTTGAGAGTCCGAGATCAGAGGGGAAATGTG

CTTCTGTCTCCTGAGGAAGTTAGTGAAACACATGGAACAGAGAAACTGACGATAACGTAT

TCATCGTCTATGATGTGGGAAATCAATGGTCCGGAATCCGTGCTAGTCAACACATATCAA

TGGATCATTAGAAGTTGGGAAACTGTGAAGATYCAATGGTCCCAGGACCCTACGATGTTG

TACAATAAGATGGAATTTGAGCCCTTCCAATCCTTGGTGCCCAAGGCTGCTAGAGGCCAG

TATAGTGGGTTTGTGAGGACATTATTCCAACAGATGCGTGATGTGTTGGGGACATTTGAC

ACTGTCCAAATAATAAAGCTCCTACCATTTGCAGCAGCCCCACCGGAACAGAGTAGGATG

CAATTTTCCTCTCTGACTGTGAACGTAAGAGGTTCAGGAATGAGAATACTTGTGAGAGGC

AACTCCCCTGTGTTCAACTATAATAAGGCCACCAAGAGACTCACAGTTCTTGGAAAGGAT

GCAGGCGCCTTGACAGAATATCCAGATGAGGGAACAGCAGGAGTGGAGTCTGCAGTATTA

AGAGGATTTCTAATTCTGGGCAAAGAGGACAAAAGATATGGACCAGCATTGAGCATCAAC

GAATTGAGCAATCTTGCGAAAGGGGAAAAGGCTAATGTGTTGATAGGACAAGGAGACGTG

GTGTTGGTAATGAAACGGAAACGGGACTCTAGCATACTTACTGACAGCCAGACAGCGACC

AAAAGAATTCGGATGGCCATCAATTAG---------------------------------

----------------------

>A_duck_Southwestern_China_B1904_2020_EPI1844096

---------------------------ATGGAGAGAATAAAAGAACTAAGAGATTTGATG

TCGCAGTCTCGCACTCGCGAGATACTAACAAAAACCACTGTGGACCATATGGCCATAATA

AAGAAATACACATCAGGAAGACAGGAGAAGAACCCTGCCCTCAGGATGAAATGGATGATG

GCAATGAAATATCCTATTACAGCTGACAAAAGAATAATGGAGATGATCCCTGAAAGGAAT

GAGCAAGGTCAGACTCTCTGGAGCAAAACAAATGATGCTGGATCAGACAGAGTGATGGTC

TCACCTCTGGCTGTGACATGGTGGAATAGGAATGGGCCAACAACAAGTACAGTACACTAC

CCAAAAGTCTACAAAACCTACTTTGAAAAGGTAGAAAGGTTGAAACATGGAACCTTTGGT

CCTGTTCACTTTCGAAATCAGGTTAAGATACGCCGCAGGGTTGACATAAACCCGGGCCAT

GCAGATCTCAGTGCCAAAGAAGCACAGGATGTCATCATGGAGGTTGTTTTCCCAAATGAA

GTTGGAGCCAGGATCTTGACATCAGAATCACAATTAACAATAACAAGGGAAAAGAAGGAG

GAACTTCAGGATTGCAAGATTGCTCCTTTGATGGTGGCATACATGTTGGAAAGAGAACTG

GTTCGCAAGACCAGATTTTTACCAGTAGCTGGCGGAACAAGCAGCGTATACATCGAGGTA

TTGCATTTGACTCAAGGGACCTGCTGGGAACAAATGTACACACCAGGAGGGGAGGTGAGA

AATGATGATGTTGATCAGAGTTTGATCATTGCTGCTAGAAATATAGTTAGGAGGGCAACA

GTATCAGCAGACCCATTGGCTTCGCTCTTGGAAATGTGCCACAGTACACAAATTGGTGGA

GTAAGGATGGTGGACATTCTTAGGCAGAACCCAACAGAGGAGCAAGCTGTGGATATATGC

AAAGCAGCAATGGGTTTAAGAATCAGTTCATCCTTCAGCTTTGGAGGTTTCACTTTCAAA

AGGACAAGTGGGTCGTCTGTCAAAAGAGAAGAAGAAATACTCACTGGCAACCTCCAAACA

CTGAAAGTTAGAATACATGAAGGATATGAGGAATTCACAATGGTTGGGCGAAGAGCTACA

GCCATTTTGAGGAAAGCAACCAGGAGACTGATCCAATTAATAGTGAGTGGAAGAGACGAG

CAGTCAATCGCTGAAGCAATCATAGTGGCAATGGTTTTCTCACAGGAGGATTGCATGATA

AAAGCAGTACGAGGTGATTTGAATTTTGTCAACAGAGCGAATCAGCGGCTAAATCCTATG

CATCAACTTCTGAGGCATTTCCAAAAGGATGCAAAAGTGCTGTTTCAAAACTGGGGGATT

GAACCAATTGACAATGTAATGGGGATGATCGGGATACTGCCAGACATGACCCCCAGCACA

GAGATGTCACTGAGAGGAGTGAGAGTCAGCAAAATGGGAGTGGATGAATATTCCAGTACT

GAGAGAGTGGTCGTGAGCATTGATCGCTTCTTGAGAGTCCGAGATCAGAGGGGAAATGTG

CTTCTGTCTCCTGAGGAAGTTAGTGAAACACATGGAACAGAGAAACTGACGATAACGTAT

TCATCGTCTATGATGTGGGAAATCAATGGTCCGGAATCCGTGCTAGTCAACACATATCAA

TGGATCATTAGAAGTTGGGAAACTGTGAAGATTCAATGGTCCCAGGACCCTACGATGTTG

TACAATAAGATGGAATTTGAGCCCTTCCAATCCTTGGTGCCCAAGGCTGCTAGAGGCCAG

TATAGTGGGTTTGTGAGGACATTATTCCAACAGATGCGTGATGTGTTGGGGACATTTGAC

ACTGTCCAAATAATAAAGCTCCTACCATTTGCAGCAGCCCCACCGGAACAGAGTAGGATG

CAATTTTCCTCTCTGACTGTGAACGTAAGAGGTTCAGGAATGAGAATACTTGTGAGGGGC

AACTCCCCTGTGTTCAACTATAATAAGGCCACCAAGAGACTCACAGTTCTTGGAAAAGAT

GCAGGCGCCTTGACAGAATATCCAGATGAGGGAACAGCAGGAGTGGAGTCTGCAGTATTA

AGAGGATTTCTAATTCTGGGCAAAGAGGACAAAAGATATGGACCAGCATTGAGCATCAAC

GAATTGAGCAATCTTGCGAAAGGGGAAAAGGCTAATGTGTTGATAGGACAAGGAGACGTG

GTGTTGGTAATGAAACGGAAACGGGACTCTAGCATACTTACTGACAGCCAGACAGCGACC

AAAAGAATTCGGATGGCCATCAATTAG---------------------------------

----------------------

>A_duck_Korea_H411_2020_EPI1845934

---------------------------ATGGAGAGAATAAAAGAACTAAGAGATTTGATG

TCGCAGTCTCGCACTCGCGAGATACTAACAAAAACCACTGTGGACCATATGGCCATAATA

AAGAAATACACATCAGGAAGACAGGAGAAGAACCCTGCCCTCAGGATGAAATGGATGATG

GCAATGAAATATCCTATTACAGCTGACAAAAGAATAATGGAGATGATCCCTGAAAGGAAT

GAGCAAGGTCAGACTCTCTGGAGCAAAACAAATGATGCTGGATCAGACAGAGTGATGGTC

TCACCTCTGGCTGTGACATGGTGGAATAGGAATGGGCCAACAACAAGTACAGTACACTAC

CCAAAAGTCTACAAAACCTACTTTGAAAAGGTAGAAAGGTTGAAACATGGAACCTTTGGT

CCTGTTCACTTTCGAAATCAGGTTAAGATACGCCGCAGGGTTGACATAAACCCGGGCCAT

GCAGATCTCAGTGCCAAAGAAGCACAGGATGTCATCATGGAGGTTGTTTTCCCAAATGAA

GTTGGAGCCAGGATCTTGACATCAGAATCACAATTAACAATAACAAGGGAAAAGAAGGAG

GAACTTCAGGATTGCAAGATTGCTCCTTTGATGGTGGCATACATGTTGGAAAGAGAACTG

GTTCGCAAGACCAGATTTTTACCAGTAGCTGGCGGAACAAGCAGCGTATACATCGAGGTA

TTGCATTTGACTCAAGGGACCTGCTGGGAACAAATGTACACACCAGGAGGGGAGGTGAGA

AATGATGATGTTGATCAGAGTTTGATCATTGCTGCTAGAAATATAGTTAGGAGGGCAACA

GTATCAGCAGACCCATTGGCTTCGCTCTTGGAAATGTGCCACAGTACACAAATTGGTGGA

GTAAGGATGGTGGACATTCTTAGGCAGAACCCAACAGAGGAGCAAGCTGTGGATATATGC

AAAGCAGCAATGGGTTTAAGAATCAGTTCATCCTTCAGCTTTGGAGGTTTCACTTTCAAA

AGGACAAGTGGGTCGTCTGTCAAAAGAGAAGAAGAAATACTCACTGGCAACCTCCAAACG

CTGAAAGTTAGAATACATGAAGGATATGAGGAATTCACAATGGTTGGGCGAAGAGCTACA

GCCATTTTGAGGAAAGCAACCAGGAGACTGATCCAATTAATAGTGAGTGGAAGAGACGAG

CAGTCAATCGCTGAAGCAATCATAGTGGCAATGGTTTTCTCACAGGAGGATTGCATGATA

AAAGCAGTACGAGGTGATTTGAATTTTGTCAACAGAGCGAATCAGCGGCTAAATCCTATG

CATCAACTTCTGAGGCATTTCCAAAAGGATGCAAAAGTGCTGTTTCAAAACTGGGGGATT

GAATCAATTGACAATGTAATGGGGATGATCGGGATACTGCCCGACATGACCCCCAGCACA

GAGATGTCACTGAGAGGAGTGAGAGTCAGCAAAATGGGAGTGGATGAATATTCCAGTACT

GAGAGAGTGGTCGTGAGCATTGATCGCTTCTTGAGAGTCCGAGATCAGAGGGGAAATGTG

CTTCTGTCTCCTGAGGAAGTTAGTGAAACACATGGAACAGAGAAACTGACGATAACGTAT

TCATCGTCTATGATGTGGGAAATCAATGGTCCGGAATCCGTGCTAGTCAACACATATCAA

TGGATCATTAGAAGTTGGGAAACTGTGAAGATTCAATGGTCCCAGGACCCTACGATGTTG

TACAATAAGATGGAATTTGAGCCCTTCCAATCCTTGGTGCCCAAGGCTGCTAGAGGCCAG

TATAGTGGGTTTGTGAGGACATTATTCCAACAGATGCGTGATGTGTTGGGGACATTTGAC

ACTGTCCAAATAATAAAGCTCCTACCATTTGCAGCAGCCCCACCGGAACAGAGTAGGATG

CAATTTTCCTCTCTGACTGTGAACGTAAGAGGTTCAGGAATGAGAATACTTGTGAGGGGC

AACTCCCCTGTGTTCAACTATAATAAGGCCACCAAGAGACTCACAGTTCTTGGAAAGGAT

GCAGGCGCCTTGACAGAATATCCAGATGAGGGAACAGCAGGAGTGGAGTCTGCAGTATTA

AGAGGATTTCTAATTCTGGGCAAAGAGGACAAAAGATATGGACCAGCATTGAGCATCAAC

GAATTGAGCAATCTTGCGAAAGGGGAAAAGGCTAATGTGTTGATAGGACAAGGAGACGTG

GTGTTGGTAATGAAACGGAAACGGGACTCTAGCATACTTACTGACAGCCAGACAGCGACC

AAAAGAATTCGGATGGCCATCAATTAG---------------------------------

----------------------

>A_duck_Korea_H431_2020_EPI1845958

---------------------------ATGGAGAGAATAAAAGAACTAAGAGATTTGATG

TTGCAGTCTCGCACTCGCGAGATACTAACAAAAACCACTGTGGACCATATGGCCATAATA

AAGAAATACACATCAGGAAGACAGGAGAAGAACCCTGCCCTCAGGATGAAATGGATGATG

GCAATGAAATATCCTATTACAGCTGACAAAAGAATAATGGAGATGATCCCTGAAAGGAAT

GAGCAAGGTCAGACTCTCTGGAGCAAAACAAATGATGCTGGATCAGACAGAGTGATGGTC

TCACCTCTGGCTGTGACATGGTGGAATAGGAATGGGCCAACAACAAGTACAGTACACTAC

CCAAAAGTCTACAAAACCTACTTTGAAAAGGTAGAAAGGTTGAAACATGGAACCTTTGGT

CCTGTTCACTTTCGAAATCAGGTTAAGATACGCCGCAGGGTTGACATAAACCCGGGCCAT

GCAGATCTCAGTGCCAAAGAAGCACAGGATGTCATCATGGAGGTTGTTTTCCCAAATGAA

GTTGGAGCCAGGATCTTGACATCAGAATCACAATTAACAATAACAAGGGAAAAGAAGGAG

GAACTTCAGGATTGCAAGATTGCTCCTTTGATGGTGGCATACATGTTGGAAAGAGAACTG

GTTCGCAAGACCAGATTTTTACCAGTAGCTGGCGGAACAAGCAGCGTATACATCGAGGTA

TTGCATTTGACTCAAGGGACCTGCTGGGAACAAATGTACACACCAGGAGGGGAGGTGAGA

AATGATGATGTTGATCAGAGTTTGATCATTGCTGCTAGAAATATAGTTAGGAGGGCAACA

GTATCAGCAGACCCATTGGCTTCGCTCTTGGAAATGTGCCACAGTACACAAATTGGTGGA

GTAAGGATGGTGGACATTCTTAGGCAGAACCCAACAGAGGAGCAAGCTGTGGATATATGC

AAAGCAGCAATGGGTTTAAGAATCAGTTCATCCTTCAGCTTTGGAGGTTTCACTTTCAAA

AGGACAAGTGGGTCGTCTGTCAAAAGAGAAGAAGAAATACTCACTGGCAACCTCCAAACG

CTGAAAGTTAGAATACATGAAGGATATGAAGAATTCACAATGGTTGGGCGAAGAGCTACA

GCCATTTTGAGGAAAGCAACCAGGAGACTGATCCAATTAATAGTGAGTGGAAGAGACGAG

CAGTCAATCGCTGAAGCAATCATAGTGGCAATGGTTTTCTCACAGGAGGATTGCATGATA

AAAGCAGTACGAGGTGATTTGAATTTTGTCAACAGAGCGAATCAGCGGCTAAATCCTATG

CATCAACTTCTGAGGCATTTCCAAAAGGATGCAAAAGTGCTGTTTCAAAACTGGGGGATT

GAATCAATTGACAATGTAATGGGGATGATCGGGATACTGCCAGACATGACCCCCAGCACA

GAGATGTCACTGAGAGGAGTGAGAGTCAGCAAAATGGGAGTGGATGAATATTCCAGTACT

GAGAGAGTGGTCGTGAGCATTGATCGCTTCTTGAGAGTCCGAGATCAGAGGGGAAATGTG

CTTCTGTCTCCTGAGGAAGTTAGTGAAACACATGGAACAGAGAAACTGACGATAACGTAT

TCATCGTCTATGATGTGGGAAATCAATGGTCCGGAATCCGTGCTAGTCAACACATATCAA

TGGATCATTAGAAGTTGGGAAACTGTGAAGATTCAATGGTCCCAGGACCCTACGATGTTG

TACAATAAGATGGAATTTGAGCCCTTCCAATCCTTGGTGCCCAAGGCTGCTAGAGGCCAG

TATAGTGGGTTTGTGAGGACATTATTCCAACAGATGCGTGATGTGTTGGGGACATTTGAC

ACTGTCCAAATAATAAAGCTCCTACCATTTGCAGCAGCCCCACCGGAACAGAGTAGGATG

CAATTTTCCTCTCTGACTGTGAACGTAAGAGGTTCAGGAATGAGAATACTTGTGAGGGGC

AACTCCCCTGTGTTCAACTATAATAAGGCCACCAAGAGACTCACAGTTCTTGGAAAGGAT

GCAGGCGCCTTGACAGAATATCCAGATGAGGGAACAGCAGGAGTGGAGTCTGCAGTATTA

AGAGGATTTCTAATTCTGGGCAAAGAGGACAAAAGATATGGACCAGCATTGAGCATCAAC

GAATTGAGCAATCTTGCGAAAGGGGAAAAGGCTAATGTGTTGATAGGACAAGGAGACGTG

GTGTTGGTAATGAAACGGAAACGGGACTCTAGCATACTTACTGACAGCCAGACAGCGACC

AAAAGAATTCGGATGGCCATCAATTAG---------------------------------

----------------------

>A_duck_Korea_H471_2020_EPI1846030

---------------------------ATGGAGAGAATAAAAGAACTAAGAGATTTGATG

TTGCAGTCTCGCACTCGCGAGATACTAACAAAAACCACTGTGGACCATATGGCCATAATA

AAGAAATACACATCAGGAAGACAGGAGAAGAACCCTGCCCTCAGGATGAAATGGATGATG

GCAATGAAATATCCTATTACAGCTGACAAAAGAATAATGGAGATGATCCCTGAAAGGAAT

GAGCAAGGTCAGACTCTCTGGAGCAAAACAAATGATGCTGGATCAGACAGAGTGATGGTC

TCACCTCTGGCTGTGACATGGTGGAATAGGAATGGGCCAACAACAAGTACGGTACACTAC

CCAAAAGTCTACAAAACCTACTTTGAAAAGGTAGAAAGGTTGAAACATGGAACCTTTGGT

CCTGTTCACTTTCGAAATCAGGTTAAGATACGCCGCAGGGTTGACATAAACCCGGGCCAT

GCAGATCTCAGTGCCAAAGAAGCACAGGATGTCATCATGGAGGTTGTTTTCCCAAATGAA

GTTGGAGCCAGGATCTTGACATCAGAATCACAATTAACAATAACAAGGGAAAAGAAGGAG

GAACTTCAGGATTGCAAGATTGCTCCTTTGATGGTGGCATACATGTTGGAAAGAGAACTG

GTTCGCAAGACCAGATTTTTACCAGTAGCTGGCGGAACAAGCAGCGTATACATCGAGGTA

TTGCATTTGACTCAAGGGACCTGCTGGGAACAAATGTACACACCAGGAGGGGAGGTGAGA

AATGATGATGTTGATCAGAGTTTGATCATTGCTGCTAGAAATATAGTTAGGAGGGCAACA

GTATCAGCAGACCCATTGGCTTCGCTCTTGGAAATGTGCCACAGTACACAAATTGGTGGA

GTAAGGATGGTGGACATTCTTAGGCAGAACCCAACAGAGGAGCAAGCTGTGGATATATGC

AAAGCAGCAATGGGTTTAAGAATCAGTTCATCCTTCAGCTTTGGAGGTTTCACTTTCAAA

AGGACAAGTGGGTCGTCTGTCAAAAGAGAAGAAGAAATACTCACTGGCAACCTCCAAACG

CTGAAAGTTAGAATACATGAAGGATATGAAGAATTCACAATGGTTGGGCGAAGAGCTACA

GCCATTTTGAGGAAAGCAACCAGGAGACTGATCCAATTAATAGTGAGTGGAAGAGACGAG

CAGTCAATCGCTGAAGCAATCATAGTGGCAATGGTTTTCTCACAGGAGGATTGCATGATA

AAAGCAGTACGAGGTGATTTGAATTTTGTCAACAGAGCGAATCAGCGGCTAAATCCTATG

CATCAACTTCTGAGGCATTTCCAAAAGGATGCAAAAGTGCTGTTTCAAAACTGGGGGATT

GAATCAATTGACAATGTAATGGGGATGATCGGGATACTGCCAGACATGACCCCCAGCACA

GAGATGTCACTGAGAGGAGTGAGAGTCAGCAAAATGGGAGTGGATGAATATTCCAGTACT

GAGAGAGTGGTCGTGAGCATTGATCGCTTCTTGAGAGTCCGAGATCAGAGGGGAAATGTG

CTTCTGTCTCCTGAGGAAGTTAGTGAAACACATGGAACAGAGAAACTGACGATAACGTAT

TCATCGTCTATGATGTGGGAAATCAATGGTCCGGAATCCGTGCTAGTCAACACATATCAA

TGGATCATTAGAAGTTGGGAAACTGTGAAGATTCAATGGTCCCAGGACCCTACGATGTTG

TACAATAAGATGGAATTTGAGCCCTTCCAATCCTTGGTGCCCAAGGCTGCTAGAGGCCAG

TATAGTGGGTTTGTGAGGACATTATTCCAACAGATGCGTGATGTGTTGGGGACATTTGAC

ACTGTCCAAATAATAAAGCTCCTACCATTTGCAGCAGCCCCACCGGAACAGAGTAGGATG

CAATTTTCCTCTCTGACTGTGAACGTAAGAGGTTCAGGAATGAGAATACTTGTGAGGGGC

AACTCCCCTGTGTTCAACTATAATAAGGCCACCAAGAGACTCACAGTTCTTGGAAAGGAT

GCAGGCGCCTTGACAGAATATCCAGATGAGGGAACAGCAGGAGTGGAGTCTGCAGTATTA

AGAGGATTTCTAATTCTGGGCAAAGAGGACAAAAGATATGGACCAGCATTGAGCATCAAC

GAATTGAGCAATCTTGCGAAAGGGGAAAAGGCTAATGTGTTGATAGGACAAGGAGACGTG

GTGTTGGTAATGAAACGGAAACGGGACTCTAGCATACTTACTGACAGCCAGACAGCGACC

AAAAGAATTCGGATGGCCATCAATTAG---------------------------------

----------------------

>A_chicken_Korea_H510_2020_EPI1846054

---------------------------ATGGAGAGAATAAAAGAACTAAGAGATTTGATG

TCGCAGTCTCGCACTCGCGAGATACTAACAAAAACCACTGTGGACCATATGGCCATAATA

AAGAAATACACATCAGGAAGACAGGAGAAGAACCCTGCCCTCAGGATGAAATGGATGATG

GCAATGAAATATCCTATTACAGCTGACAAAAGAATAATGGAGATGATCCCTGAAAGGAAT

GAGCAAGGTCAGACTCTCTGGAGCAAAACAAATGATGCTGGATCAGACAGAGTGATGGTC

TCACCTCTGGCTGTGACATGGTGGAATAGGAATGGGCCAACAACAAGTACAGTACACTAC

CCAAAAGTCTACAAAACCTACTTTGAAAAGGTAGAAAGGTTGAAACATGGAACCTTTGGT

CCTGTTCACTTTCGAAATCAGGTTAAGATACGCCGCAGGGTTGACATAAACCCGGGCCAT

GCAGATCTCAGTGCCAAAGAAGCACAGGATGTCATCATGGAGGTTGTTTTCCCAAATGAA

GTTGGAGCCAGGATCTTGACATCAGAATCACAATTAACAATAACAAGGGAAAAGAAGGAG

GAACTTCAGGATTGCAAGATTGCTCCTTTGATGGTGGCATACATGTTGGAAAGAGAACTG

GTTCGCAAGACCAGATTTTTACCAGTAGCTGGCGGAACAAGCAGCGTATACATCGAGGTA

TTGCATTTGACTCAAGGGACCTGCTGGGAACAAATGTACACACCAGGAGGGGAGGTGAGA

AATGATGATGTTGATCAGAGTTTGATCATTGCTGCTAGAAATATAGTTAGGAGGGCAACA

GTATCAGCAGACCCATTGGCTTCGCTCTTGGAAATGTGCCACAGTACACAAATTGGTGGA

GTAAGGATGGTGGACATTCTTAGGCAGAACCCAACAGAGGAGCAAGCTGTGGATATATGC

AAAGCAGCAATGGGTTTAAGAATCAGTTCATCCTTCAGCTTTGGAGGTTTCACTTTCAAA

AGGACAAGTGGGTCGTCTGTCAAAAGAGAAGAAGAAATACTCACTGGCAACCTCCAAACG

CTGAAAGTTAGAATACATGAAGGATATGAGGAATTCACAATGGTTGGGCGAAGAGCTACA

GCCATTTTGAGGAAGGCAACCAGGAGACTGATCCAATTAATAGTGAGTGGAAGAGACGAG

CAGTCAATCGCTGAAGCAATCATAGTGGCAATGGTTTTCTCACAGGAGGATTGCATGATA

AAAGCAGTACGAGGTGATTTGAATTTTGTCAACAGAGCGAATCAGCGGCTAAATCCTATG

CATCAACTTCTGAGGCATTTCCAAAAGGATGCAAAAGTGCTGTTTCAAAACTGGGGGATT

GAATCAATTGACAATGTAATGGGGATGATCGGGATACTGCCAGACATGACCCCCAGCACA

GAGATGTCACTGAGAGGAGTGAGAGTCAGCAAAATGGGAGTGGATGAATATTCCAGTACT

GAGAGAGTGGTCGTGAGCATTGATCGCTTCTTGAGAGTCCGAGATCAGAGGGGAAATGTG

CTTCTGTCTCCTGAGGAAGTTAGTGAAACACATGGAACAGAGAAACTGACGATAACGTAT

TCATCGTCTATGATGTGGGAAATCAATGGTCCGGAATCCGTGCTAGTCAACACATATCAA

TGGATCATTAGAAGTTGGGAAACTGTGAAGATTCAATGGTCCCAGGACCCTACGATGTTG

TACAATAAGATGGAATTTGAGCCCTTCCAATCCTTGGTGCCCAAGGCTGCTAGAGGCCAG

TATAGTGGGTTTGTGAGGACATTATTCCAACAGATGCGTGATGTGTTGGGGACATTTGAC

ACTGTCCAAATAATAAAGCTCCTACCATTTGCAGCAGCCCCACCGGAACAGAGTAGGATG

CAATTTTCCTCTCTGACTGTGAACGTAAGAGGTTCAGGAATGAGAATACTTGTGAGGGGC

AACTCCCCTGTGTTCAACTATAATAAGGCCACCAAGAGACTCACAGTTCTTGGAAAGGAT

GCAGGCGCCTTGACAGAATATCCAGATGAGGGAACAGCAGGAGTGGAGTCTGCAGTATTA

AGAGGATTTCTAATTCTGGGCAAAGAGGACAAAAGATATGGACCAGCATTGAGCATCAAC

GAATTGAGCAATCTTGCGAAAGGGGAAAAGGCTAATGTGTTGATAGGACAAGGAGACGTG

GTGTTGGTAATGAAACGGAAACGGGACTCTAGCATACTTACTGACAGCCAGACAGCGACC

AAAAGAATTCGGATGGCCATCAATTAG---------------------------------

----------------------

>A_duck_Korea_H538_2020_EPI1846150

---------------------------ATGGAGAGAATAAAAGAACTAAGAGATTTGATG

TCGCAGTCTCGCACTCGCGAGATACTAACAAAAACCACTGTGGACCATATGGCCATAATA

AAGAAATACACATCAGGAAGACAGGAGAAGAACCCTGCCCTCAGGATGAAATGGATGATG

GCAATGAAATATCCTATTACAGCTGACAAAAGAATAATGGAGATGATCCCTGAAAGGAAT

GAGCAAGGTCAGACTCTCTGGAGCAAAACAAATGATGCTGGATCAGACAGAGTGATGGTC

TCACCTCTGGCTGTGACATGGTGGAATAGGAATGGGCCAACAACAAGTACAGTACACTAC

CCAAAAGTCTACAAAACCTACTTTGAAAAGGTAGAAAGGTTGAAACATGGAACCTTTGGT

CCTGTTCACTTTCGAAATCAGGTTAAGATACGCCGCAGGGTTGACATAAACCCGGGCCAT

GCAGATCTCAGTGCCAAAGAAGCACAGGATGTCATCATGGAGGTTGTTTTCCCAAATGAA

GTTGGAGCCAGGATCTTGACATCAGAATCACAATTAACAATAACAAGGGAAAAGAAGGAG

GAACTTCAGGATTGCAAGATTGCTCCTTTGATGGTGGCATACATGTTGGAAAGAGAACTG

GTTCGCAAGACCAGATTTTTACCAGTAGCTGGCGGAACAAGCAGCGTATACATCGAGGTA

TTGCATTTGACTCAAGGGACCTGCTGGGAACAAATGTACACACCAGGAGGGGAGGTGAGA

AATGATGATGTTGATCAGAGTTTGATCATTGCTGCTAGAAATATAGTTAGGAGGGCAACA

GTATCAGCAGACCCATTGGCTTCGCTCTTGGAAATGTGCCACAGTACACAAATTGGTGGA

GTAAGGATGGTGGACATTCTTAGGCAGAACCCAACAGAGGAGCAAGCTGTGGATATATGC

AAAGCAGCAATGGGTTTAAGAATCAGTTCATCCTTCAGCTTTGGAGGTTTCACTTTCAAA

AGGACAAGTGGGTCGTCTGTCAAAAGAGAAGAAGAAATACTCACTGGCAACCTCCAAACG

CTGAAAGTTAGAATACATGAAGGATATGAGGAATTCACAATGGTTGGGCGAAGAGCTACA

GCCATTTTGAGGAAAGCAACCAGGAGACTGATCCAATTAATAGTGAGTGGAAGAGACGAG

CAGTCAATCGCTGAAGCAATCATAGTGGCAATGGTTTTCTCACAGGAGGATTGCATGATA

AAAGCAGTACGAGGTGATTTGAATTTTGTCAACAGAGCGAATCAGCGGCTAAATCCTATG

CATCAACTTCTGAGGCATTTCCAAAAGGATGCAAAAGTGCTGTTTCAAAACTGGGGGATT

GAATCAATTGACAATGTAATGGGGATGATCGGGATACTGCCAGACATGACCCCCAGCACA

GAGATGTCACTGAGAGGAGTGAGAGTCAGCAAAATGGGAGTGGATGAATATTCCAGTACT

GAGAGAGTGGTCGTGAGCATTGATCGCTTCTTGAGAGTCCGAGATCAGAGGGGAAATGTG

CTTCTGTCTCCTGAGGAAGTTAGTGAAACACATGGAACAGAGAAACTGACGATAACGTAT

TCATCGTCTATGATGTGGGAAATCAATGGTCCGGAATCCGTGCTAGTCAACACATATCAA

TGGATCATTAGAAGTTGGGAAACTGTGAAGATTCAATGGTCCCAGGACCCTACGATGTTG

TACAATAAGATGGAATTTGAGCCCTTCCAATCCTTGGTGCCCAAGGCTGCTAGAGGCCAG

TATAGTGGGTTTGTGAGGACATTATTCCAACAGATGCGTGATGTGTTGGGGACATTTGAC

ACTGTCCAAATAATAAAGCTCCTACCATTTGCAGCAGCCCCACCGGAACAGAGTAGGATG

CAATTTTCCTCTCTGACTGTGAACGTAAGAGGTTCAGGAATGAGAATACTTGTGAGGGGC

AACTCCCCTGTGTTCAACTATAATAAGGCCACCAAGAGACTCACAGTTCTTGGAAAGGAT

GCAGGCGCCTTGACAGAATATCCAGATGAGGGAACAGCAGGAGTGGAGTCTGCAGTATTA

AGAGGATTTCTAATTCTGGGCAAAGAGGACAAAAGATATGGACCAGCATTGAGCATCAAC

GAATTGAGCAATCTTGCGAAAGGGGAAAAGGCTAATGTGTTGATAGGACAAGGAGACGTG

GTGTTGGTAATGAAACGGAAACGGGACTCTAGCATACTTACTGACAGCCAGACAGCGACC

AAAAGAATTCGGATGGCCATCAATTAG---------------------------------

----------------------

>A_chicken_Tyumen_302-01_2020_EPI1848603

AGCAAAAGCAGGTCAAATATATTCAATATGGAGAGAATAAAAGAACTAAGAGATTTGATG

TCGCAGTCTCGCACTCGCGAGATACTAACAAAAACCACTGTGGACCATATGGCCATAATA

AAGAAATACACATCAGGAAGACAGGAGAAGAACCCTGCCCTCAGGATGAAATGGATGATG

GCAATGAAATATCCTATTACAGCTGACAAAAGAATAATGGAGATGATCCCTGAAAGGAAT

GAGCAAGGTCAGACTCTCTGGAGCAAAACAAATGATGCTGGATCAGACAGAGTGATGGTC

TCACCTCTGGCTGTGACATGGTGGAATAGAAATGGGCCAACAACAAGTACAGTACACTAC

CCAAAAGTCTACAAAACCTACTTTGAAAAGGTAGAAAGGTTGAAACATGGAACCTTTGGT

CCTGTTCACTTTCGAAATCAGGTTAAGATACGCCGCAGGGTTGACATAAACCCGGGCCAT

GCAGATCTCAGTGCCAAAGAAGCACAGGATGTCATCATGGAGGTTGTTTTCCCAAATGAA

GTTGGAGCCAGGATCTTGACATCAGAATCACAATTAACAATAACAAGGGAAAAGAAGGAG

GAACTTCAGGATTGCAAGATTGCTCCTTTGATGGTGGCATACATGTTGGAAAGAGAACTG

GTTCGCAAGACCAGATTTTTACCAGTAGCTGGCGGAACAAGCAGCGTATACATCGAGGTA

TTGCATTTGACTCAAGGGACCTGCTGGGAACAAATGTACACACCAGGAGGGGAGGTGAGA

AATGATGATGTTGATCAGAGTTTGATCATTGCTGCTAGAAATATAGTTAGGAGGGCAACA

GTATCAGCAGACCCATTGGCTTCGCTCTTGGAAATGTGCCACAGTACACAAATTGGTGGA

GTAAGGATGGTGGACATTCTTAGGCAGAACCCAACAGAGGAGCAAGCTGTGGATATATGC

AAAGCAGCAATGGGTTTAAGAATCAGTTCATCCTTCAGCTTTGGAGGTTTCACTTTCAAA

AGGACAAGTGGATCGTCTGTCAAAAGAGAAGAAGAAATACTCACTGGCAACCTCCAAACA

CTGAAAGTAAGAATACATGAAGGATATGAGGAATTCACAATGGTTGGGCGAAGAGCTACA

GCCATTTTGAGGAAAGCAACCAGGAGACTGATCCAATTAATAGTGAGTGGAAGAGACGAG

CAGTCAATCGCTGAAGCAATCATAGTGGCAATGGTTTTCTCACAGGAGGATTGCATGATA

AAAGCAGTACGAGGTGATTTGAATTTTGTCAACAGAGCGAATCAGCGGCTAAATCCTATG

CATCAACTTCTGAGGCATTTCCAAAAGGATGCAAAAGTGCTGTTTCAAAACTGGGGGATT

GAACCAATTGACAATGTAATGGGGATGATCGGGATACTGCCTGACATGACCCCCAGCACA

GAGATGTCACTGAGAGGAGTGAGAGTCAGCAAAATGGGAGTGGATGAATATTCCAGTACT

GAGAGAGTGGTCGTGAGCATTGATCGCTTCTTGAGAGTCCGAGATCAGAGGGGAAATGTG

CTTCTGTCTCCTGAGGAAGTTAGTGAAACACATGGAACAGAGAAACTGACGATAACGTAT

TCATCGTCTATGATGTGGGAAATCAATGGTCCGGAATCCGTGCTAGTCAACACATATCAA

TGGATCATTAGAAATTGGGAAACTGTGAAGATTCAGTGGTCCCAGGACCCTACGATGTTG

TACAATAAGATGGAATTTGAGCCCTTCCAATCCTTGGTGCCCAAGGCTGCTAGAGGCCAG

TATAGTGGGTTTGTGAGGACATTATTCCAACAGATGCGTGATGTGTTGGGGACATTTGAC

ACTGTCCAAATAATAAAGCTCCTACCATTTGCAGCAGCCCCGCCGGAACAGAGTAGGATG

CAATTTTCCTCTCTGACTGTGAACGTAAGAGGTTCAGGAATGAGAATACTTGTGAGGGGC

AACTCCCCTGTGTTCAACTATAATAAGGCCACCAAGAGGCTCACAGTTCTTGGAAAGGAT

GCAGGCGCCTTGACAGAATATCCAGATGAGGGAACAGCAGGAGTGGAGTCTGCAGTATTA

AGAGGATTTCTAATTCTGGGCAAAGAGGACAAAAGATATGGACCAGCATTGAGCATCAAC

GAATTGAGCAATCTTGCGAAAGGGGAAAAGGCTAATGTGTTGATAGGACAAGGAGACGTG

GTGTTGGTAATGAAACGGAAACGGGACTCTAGCATACTTACTGACAGCCAGACAGCGACC

AAAAGAATTCGGATGGCCATCAATTAGTGTCGAATTGTTTAAAAACGACCTTGTTTCTAC

T---------------------

>A_chicken_Tyumen_302-02_2020_EPI1848611

AGCAAAAGCAGGTCAAATATATTCAATATGGAGAGAATAAAAGAACTAAGAGATTTGATG

TCGCAGTCTCGCACTCGCGAGATACTAACAAAAACCACTGTGGACCATATGGCCATAATA

AAGAAATACACATCAGGAAGACAGGAGAAGAACCCTGCCCTCAGGATGAAATGGATGATG

GCAATGAAATATCCTATTACAGCTGACAAAAGAATAATGGAGATGATCCCTGAAAGGAAT

GAGCAAGGTCAGACTCTCTGGAGCAAAACAAATGATGCTGGATCAGACAGAGTGATGGTC

TCACCTCTGGCTGTGACATGGTGGAATAGAAATGGGCCAACAACAAGTACAGTACACTAC

CCAAAAGTCTACAAAACCTACTTTGAAAAGGTAGAAAGGTTGAAACATGGAACCTTTGGT

CCTGTTCACTTTCGAAATCAGGTTAAGATACGCCGCAGGGTTGACATAAACCCGGGCCAT

GCAGATCTCAGTGCCAAAGAAGCACAGGATGTCATCATGGAGGTTGTTTTCCCAAATGAA

GTTGGAGCCAGGATCTTGACATCAGAATCACAATTAACAATAACAAGGGAAAAGAAGGAG

GAACTTCAGGATTGCAAGATTGCTCCTTTGATGGTGGCATACATGTTGGAAAGAGAACTG

GTTCGCAAGACCAGATTTTTACCAGTAGCTGGCGGAACAAGCAGCGTATACATCGAGGTA

TTGCATTTGACTCAAGGGACCTGCTGGGAACAAATGTACACACCAGGAGGGGAGGTGAGA

AATGATGATGTTGATCAGAGTTTGATCATTGCTGCTAGAAATATAGTTAGGAGGGCAACA

GTATCAGCAGACCCATTGGCTTCGCTCTTGGAAATGTGCCACAGTACACAAATTGGTGGA

GTAAGGATGGTGGACATTCTTAGGCAGAACCCAACAGAGGAGCAAGCTGTGGATATATGC

AAAGCAGCAATGGGTTTAAGAATCAGTTCATCCTTCAGCTTTGGAGGTTTCACTTTCAAA

AGGACAAGTGGATCGTCTGTCAAAAGAGAAGAAGAAATACTCACTGGCAACCTCCAAACA

CTGAAAGTAAGAATACATGAAGGATATGAGGAATTCACAATGGTTGGGCGAAGAGCTACA

GCCATTTTGAGGAAAGCAACCAGGAGACTGATCCAATTAATAGTGAGTGGAAGAGACGAG

CAGTCAATCGCTGAAGCAATCATAGTGGCAATGGTTTTCTCACAGGAGGATTGCATGATA

AAAGCAGTACGAGGTGATTTGAATTTTGTCAACAGAGCGAATCAGCGGCTAAATCCTATG

CATCAACTTCTGAGGCATTTCCAAAAGGATGCAAAAGTGCTGTTTCAAAACTGGGGGATT

GAACCAATTGACAATGTAATGGGGATGATCGGGATACTGCCTGACATGACCCCCAGCACA

GAGATGTCACTGAGAGGAGTGAGAGTCAGCAAAATGGGAGTGGATGAATATTCCAGTACT

GAGAGAGTGGTCGTGAGCATTGATCGCTTCTTGAGAGTCCGAGATCAGAGGGGGAATGTG

CTTCTGTCTCCTGAGGAAGTTAGTGAAACACATGGAACAGAGAAACTGACGATAACGTAT

TCATCGTCTATGATGTGGGAAATCAATGGTCCGGAATCCGTGCTAGTCAACACATATCAA

TGGATCATTAGAAATTGGGAAACTGTGAAGATTCAGTGGTCCCAGGACCCTACGATGTTG

TACAATAAGATGGAATTTGAGCCCTTCCAATCCTTGGTGCCCAAGGCTGCTAGAGGCCAG

TATAGTGGGTTTGTGAGGACATTATTCCAACAGATGCGTGATGTGTTGGGGACATTTGAC

ACTGTCCAAATAATAAAGCTCCTACCATTTGCAGCAGCCCCGCCGGAACAGAGTAGGATG

CAATTTTCCTCTCTGACTGTGAACGTAAGAGGTTCAGGAATGAGAATACTTGTGAGGGGC

AACTCCCCTGTGTTCAACTATAATAAGGCCACCAAGAGGCTCACAGTTCTTGGAAAGGAT

GCAGGCGCCTTGACAGAATATCCAGATGAGGGAACAGCAGGAGTGGAGTCTGCAGTATTA

AGAGGATTTCTAATTCTGGGCAAAGAGGACAAAAGATATGGACCAGCATTGAGCATCAAC

GAATTGAGCAATCTTGCGAAAGGGGAAAAGGCTAATGTGTTGATAGGACAAGGAGACGTG

GTGTTGGTAATGAAACGGAAACGGGACTCTAGCATACTTACTGACAGCCAGACAGCGACC

AAAAGAATTCGGATGGCCATCAATTAGTGTCGAATTGTTTAAAAACGACCTTGTTTCTAC

T---------------------

>A_chicken_Poland_474_2020_EPI1850190

------------TCAAATATATTCAATATGGAGAGAATAAAAGAACTAAGAGATTTGATG

TCACAGTCTCGCACTCGCGAGATACTAACAAAAACCACTGTGGACCATATGGCCATAATA

AAGAAATACACATCAGGGAGACAGGAGAAGAACCCTGCTCTCAGGATGAAATGGATGATG

GCAATGAAATATCCTATTACAGCTGACAAAAGAATAATGGAGATGATCCCTGAAAGGAAT

GAGCAAGGTCAGACTCTCTGGAGCAAAACAAATGATGCTGGATCAGACAGAGTGATGGTC

TCACCTCTGGCTGTGACATGGTGGAATAGAAACGGGCCAACAACAAGTACAGTACACTAC

CCAAAAGTCTACAAAACCTACTTTGAAAAGGTGGAAAGGTTGAAACATGGAACCTTTGGT

CCTGTTCACTTTCGAAATCAGGTTAAGATACGCCGCAGGGTTGACATAAACCCGGGCCAT

GCAGATCTCAGTGCCAAAGAAGCACAGGATGTCATCATGGAGGTTGTTTTCCCAAATGAA

GTTGGAGCCAGGATCTTGACATCAGAATCACAATTAACAATAACAAGGGAAAAGAAGGAG

GAACTTCAGGATTGCAAGATTGCTCCTTTGATGGTGGCATACATGTTGGAAAGAGAACTG

GTTCGCAAGACCAGATTTTTACCAGTAGCTGGCGGAACAAGCAGCGTGTACATCGAGGTA

TTGCATTTGACTCAAGGGACCTGCTGGGAACAAATGTACACACCAGGAGGGGAGGTGAGA

AATGATGATGTTGATCAGAGTTTGATCATTGCTGCTAGAAATATAGTTAGGAGGGCAACA

GTATCAGCAGACCCATTGGCTTCGCTCTTGGAAATGTGCCACAGTACACAAATTGGTGGA

GTGAGGATGGTGGACATTCTTAGGCAGAACCCAACAGAGGAGCAAGCTGTGGATATATGC

AAAGCAGCAATGGGTTTAAGAATCAGTTCATCCTTCAGCTTTGGAGGTTTCACTTTCAAA

AGGACAAGTGGGTCGTCTGTCAAAAGAGAAGAAGAAATACTCACTGGCAACCTCCAAACA

CTGAAAGTAAGAATACATGAAGGATATGAGGAATTCACAATGGTTGGGCGAAGAGCTACA

GCCATTTTGAGGAAAGCAACCAGGAGACTGATCCAATTAATAGTGAGTGGACGAGACGAG

CAGTCAATCGCTGAAGCAATCATAGTGGCAATGGTTTTCTCACAGGAGGATTGCATGATA

AAAGCAGTACGAGGTGATTTGAATTTTGTCAACAGAGCGAATCAGCGGCTAAATCCTATG

CATCAACTTCTGAGGCATTTCCAAAAGGATGCAAAAGTGCTGTTTCAAAACTGGGGGATT

GAACCAATTGACAATGTAATGGGGATGATCGGGATACTGCCTGACATGACCCCCAGCACA

GAGATGTCACTGAGAGGAGTGAGAGTCAGCAAAATGGGAGTGGATGAATATTCCAGTACT

GAGAGAGTGGTCGTGAGCATTGATCGCTTCTTGAGAGTCCGAGATCAGAGGGGAAATGTG

CTTCTGTCTCCTGAGGAAGTTAGTGAAACACATGGAACAGAGAAACTGACGATAACGTAT

TCATCGTCTATGATGTGGGAAATCAATGGTCCGGAATCCGTGCTAGTCAACACATATCAA

TGGATCATTAGAAATTGGGAAACTGTGAAGATTCAGTGGTCCCAGGACCCTACGATGTTG

TACAATAAGATGGAATTTGAGCCCTTTCAATCCTTGGTGCCCAAGGCTGCTAGAGGCCAG

TATAGTGGGTTTGTGAGGACATTATTCCAACAGATGCGTGATGTGTTGGGGACATTTGAC

ACTGTCCAAATAATAAAGCTCCTACCATTTGCAGCAGCCCCACCGGAACAGAGTAGGATG

CAATTTTCCTCTCTGACTGTGAACGTAAGAGGTTCAGGAATGAGAATACTTGTGAGGGGC

AACTCCCCTGYGTTCAACTATAATAAGGCCACCAAGAGACTCACAGTTCTTGGAAAGGAT

GCAGGCGCCTTGACAGAATATCCAGATGAGGGAACAGCAGGAGTGGAGTCTGCAGTATTA

AGAGGATTTCTAATTCTGGGCAAAGAGGACAAAAGATATGGACCAGCATTGAGCATCAAC

GAGTTGAGCAATCTTGCGAAAGGGGAAAAGGCTAATGTGTTGATAGGACAAGGAGACGTG

GTGTTGGTRATGAAACGGAAACGGGACTCTAGCATACTTACTGACAGCCAGACAGCGACC

AAAAGAATTCGGATGGCCATCAATTAGTGTCGAATTGTTTAAAAACGA------------

----------------------

>A_swan_Poland_MB141_2020_EPI1850211

------------TCAAATATATTCAATATGGAGAGAATAAAAGAACTAAGAGATTTGATG

TCACAGTCTCGCACTCGCGAGATACTAACAAAAACCACTGTGGACCATATGGCCATAATA

AAGAAATACACATCAGGGAGACAGGAGAAGAACCCTGCCCTCAGGATGAAATGGATGATG

GCAATGAAATATCCTATTACAGCTGACAAAAGAATAATGGAGATGATCCCTGAAAGGAAT

GAGCAAGGTCAGACTCTCTGGAGCAAAACAAATGATGCTGGATCAGACAGAGTGATGGTC

TCACCTCTGGCTGTGACATGGTGGAATAGAAACGGGCCAACAACAAGTACAGTACACTAC

CCAAAAGTCTACAAAACCTACTTTGAAAAGGTGGAAAGGTTGAAACATGGAACCTTTGGT

CCTGTTCACTTTCGAAATCAGGTTAAGATACGCCGCAGGGTTGACATAAACCCGGGCCAT

GCAGATCTCAGTGCCAAAGAAGCACAGGATGTCATCATGGAGGTTGTTTTCCCAAATGAA

GTTGGAGCCAGGATCTTGACATCAGAATCACAATTAACAATAACAAGGGAAAAGAAGGAG

GAACTTCAGGATTGCAAGATTGCTCCTTTGATGGTGGCATACATGTTGGAAAGAGAACTG

GTTCGCAAGACCAGATTTTTACCAGTAGCTGGCGGAACAAGCAGCGTGTACATCGAGGTA

TTGCATTTGACTCAAGGGACCTGCTGGGAACAAATGTACACACCAGGAGGGGAGGTGAGA

AATGATGATGTTGATCARAGTTTGATCATTGCTGCTAGAAATATAGTTAGGAGGGCAACA

GTATCAGCAGACCCATTGGCTTCGCTCTTGGAAATGTGCCACAGTACACAAATTGGTGGA

GTGAGGATGGTGGACATTCTTAGGCAGAACCCAACAGAGGAGCAAGCTGTGGATATATGC

AAAGCAGCAATGGGTTTAAGAATCAGTTCATCCTTCAGCTTTGGAGGTTTCACTTTCAAA

AGGACAAGTGGGTCGTCTGTCAAAAGAGAAGAAGAAATACTCACTGGCAACCTCCAAACA

CTGAAAGTAAGAATACATGAAGGATATGAGGAATTCACAATGGTTGGGCGAAGAGCTACA

GCCATTTTGAGGAAAGCAACCAGGAGACTGATCCAATTAATAGTGAGTGGACGAGACGAG

CAGTCAATCGCTGAAGCAATCATAGTGGCAATGGTTTTCTCACAGGAGGATTGCATGATA

AAAGCAGTACGAGGTGATTTGAATTTTGTCAACAGAGCGAATCAGCGGCTAAATCCTATG

CATCAACTTCTGAGGCATTTCCAAAAGGATGCAAAAGTGCTGTTTCAAAACTGGGGGATT

GAACCAATTGACAATGTAATGGGGATGATCGGGATACTGCCTGACATGACCCCCAGCACA

GAGATGTCACTGAGAGGAGTGAGAGTCAGCAAAATGGGAGTGGATGAATATTCCAGTACT

GAGAGAGTGGTCGTGAGCATTGATCGCTTCTTGAGAGTCCGAGATCAGAGGGGAAATGTG

CTTCTGTCTCCTGAGGAAGTTAGTGAAACACATGGAACAGAGAAACTGACGATAACGTAT

TCATCGTCTATGATGTGGGAAATCAATGGTCCGGAATCCGTGCTAGTCAACACATATCAA

TGGATCATTAGAAATTGGGAAACTGTGAAGATTCAGTGGTCCCAGGACCCTACGATGTTG

TACAATAAGATGGAATTTGAGCCCTTTCAATCCTTGGTGCCCAAGGCTGCTAGAGGCCAG

TATAGTGGGTTTGTGAGGACATTATTCCAACAGATGCGTGATGTGTTGGGGACATTTGAC

ACTGTCCAAATAATAAAGCTCCTACCATTTGCAGCAGCCCCACCGGAACAGAGTAGGATG

CAATTTTCCTCTCTGACTGTGAACGTAAGAGGTTCAGGAATGAGAATACTTGTGAGGGGC

AACTCCCCTGTGTTCAACTATAATAAGGCCACCAAGAGACTCACAGTTCTTGGAAAGGAT

GCAGGTGCCTTGACAGAATATCCAGATGAGGGAACAGCAGGAGTGGAGTCTGCAGTATTA

AGAGGATTTCTAATTCTGGGCAAAGAGGACAAAAGATATGGACCAGCATTGAGCATCAAC

GAATTGAGCAATCTTGCGAAAGGGGAAAAGGCTAATGTGTTGATAGGACAAGGAGACGTG

GTGTTGGTAATGAAACGGAAACGGGACTCTAGCATACTTACTGACAGCCAGACAGCGACC

AAAAGAATTCGGATGGCCATCAATTAGTGTCGAATTGTTTAAAAACGA------------

----------------------

>A_muscovy_duck_Slovakia_Pah1_21VIR1086-1_2021_EPI1858241

---------------------------ATGGAGAGAATAAAAGAACTAAGAGATTTGATG

TCGCAGTCTCGCACTCGCGAGATACTAACAAAAACCACTGTGGACCATATGGCCATAATA

AAGAAATACACATCAGGAAGACAGGAGAAGAACCCTGCCCTCAGGATGAAATGGATGATG

GCAATGAAATATCCTATTACAGCTGACAAAAGAATAATGGAGATGATCCCTGAAAGGAAT

GAGCAAGGTCAGACTCTCTGGAGCAAAACAAATGATGCTGGATCAGACAGAGTGATGGTC

TCACCTCTGGCTGTGACATGGTGGAATAGAAATGGGCCAACAACAAGTACAGTACACTAC

CCAAAAGTCTACAAAACCTACTTTGAAAAGGTAGAAAGGTTGAAACATGGAACCTTTGGT

CCTGTTCACTTTCGAAATCAGGTTAAGATACGCCGCAGGGTTGACATAAACCCGGGCCAT

GCAGATCTCAGTGCCAAAGAAGCACAGGATGTCATCATGGAGGTTGTTTTCCCAAATGAA

GTTGGAGCCAGGATCTTGACATCAGAATCACAATTAACACTAACAAGGGAAAAGAAGGAG

GAACTTCAGGATTGCAAGATTGCTCCTTTGATGGTGGCATACATGTTGGAAAGAGAACTG

GTTCGCAAGACCAGATTTTTACCAGTAGCTGGCGGAACAAGCAGCGTATACATCGAGGTA

TTGCATTTGACTCAAGGGACCTGCTGGGAACAAATGTACACACCAGGAGGGGAGGTGAGA

AATGATGATGTTGATCAGAGTTTGATCATTGCTGCTAGAAATATAGTTAGGAGGGCAACA

GTATCAGCAGACCCATTGGCTTCGCTCTTGGAAATGTGCCACAGTACACAAATTGGTGGA

GTAAGGATGGTGGACATTCTTAGGCAGAACCCAACAGAGGAGCAAGCTGTGGATATATGC

AAAGCAGCAATGGGTTTAAGAATCAGTTCATCCTTCAGCTTTGGAGGTTTCACTTTCAAA

AGGACAAGTGGGTCGTCTGTCAAAAGAGAAGAAGAAATACTCACTGGCAACCTCCAAACA

CTGAAAGTAAGAATACATGAAGGATATGAGGAATTCACAATGGTTGGGCGAAGAGCTACA

GCCATTTTGAGGAAAGCAACCAGGAGACTGATCCAATTAATAGTGAGTGGAAGAGACGAG

CAGTCAATCGCTGAAGCAATCATAGTGGCAATGGTTTTCTCACAGGAGGATTGTATGATA

AAAGCAGTACGAGGTGATTTGAATTTTGTCAACAGAGCGAATCAGCGGCTAAATCCTATG

CACCAACTTCTGAGGCATTTCCAAAAGGATGCAAAAGTGCTGTTTCAAAACTGGGGGATT

GAACCAATTGACAATGTAATGGGGATGATCGGGATACTGCCTGACATGACCCCCAGCACA

GAGATGTCACTGAGAGGAGTGAGAGTCAGCAAAATGGGAGTGGATGAATATTCCAGTACT

GAGAGAGTGGTCGTGAGCATTGATCGCTTCTTGAGAGTCCGAGATCAGAGGGGAAATGTG

CTTCTGTCTCCTGAGGAAGTTAGTGAAACACATGGAACAGAGAAACTGACGATAACGTAT

TCATCGTCTATGATGTGGGAAATCAATGGTCCGGAATCCGTGCTAGTCAACACATATCAA

TGGATCATTAGAAATTGGGAAACTGTGAAGATTCAGTGGTCCCAGGACCCTACGATGTTG

TACAATAAGATGGAATTTGAGCCCTTCCAATCCTTGGTGCCCAAGGCTGCTAGAGGCCAG

TATAGTGGGTTTGTGAGGACATTATTCCAACAGATGCGTGATGTGTTGGGGACATTTGAC

ACTGTCCAAATAATAAAGCTCCTACCATTTGCAGCAGCCCCACCGGAACAGAGTAGGATG

CAATTTTCCTCTCTGACTGTGAACGTAAGAGGTTCAGGAATGAGAATACTTGTGAGGGGC

AACTCCCCTGTGTTCAACTATAATAAGGCCACCAAGAGACTCACAGTTCTTGGAAAGGAT

GCAGGCGCCTTGACAGAATATCCAGATGAGGGAACAGCAGGAGTGGAGTCTGCAGTATTA

AGAGGATTTCTAATTCTGGGCAAAGAGGACAAAAGATATGGACCAGCATTGAGCATCAAC

GAATTGAGCAATCTTGCGAAGGGGGAAAAGGCTAATGTGTTGATAGGACAAGGAGACGTG

GTGTTGGTAATGAAACGGAAACGGAACTCTAGCATACTTACTGACAGCCAGACAGCGACC

AAAAGAATTCGGATGGCCATCAATTAG---------------------------------

----------------------

>A_mute_swan_Slovenia_1639-20_21VIR959-1_2020_EPI1858297

---------------------------ATGGAGAGAATAAAAGAACTAAGAGATTTGATG

TCGCAGTCTCGCACTCGCGAGATACTAACAAAAACCACTGTGGACCATATGGCCATAATA

AAGAAATACACATCAGGAAGACAGGAGAAGAACCCTGCCCTCAGGATGAAATGGATGATG

GCAATGAAATATCCTATTACAGCTGACAAAAGAATAATGGAGATGATCCCTGAAAGGAAT

GAGCAAGGTCAGACTCTCTGGAGCAAAACAAATGATGCTGGATCAGACAGAGTGATGGTC

TCACCTCTGGCTGTGACATGGTGGAATAGAAATGGGCCAACAACAAGTACAGTACACTAC

CCAAAAGTCTACAAAACCTACTTTGAAAAGGTAGAAAGGTTGAAACATGGAACCTTTGGT

CCTGTTCACTTTCGAAATCAGGTTAAGATACGCCGCAGGGTTGACATAAACCCGGGCCAT

GCAGATCTCAGTGCCAAAGAAGCACAGGATGTCATCATGGAGGTTGTTTTCCCAAATGAA

GTTGGAGCCAGGATCTTGACATCAGAATCACAATTAACAATAACAAGGGAAAAGAAGGAG

GAACTTCAGGATTGCAAGATTGCTCCTTTGATGGTGGCATACATGTTGGAAAGAGAACTG

GTTCGCAAGACCAGATTTTTACCAGTAGCTGGCGGAACAAGCAGCGTATACATCGAGGTA

TTGCATTTGACTCAAGGGACCTGCTGGGAACAAATGTACACACCAGGAGGGGAGGTGAGA

AATGATGATGTTGATCAGAGTTTGATCATTGCTGCTAGAAATATAGTTAGGAGGGCAACA

GTATCAGCAGACCCTTTGGCTTCGCTCTTGGAAATGTGCCACAGTACACAAATTGGTGGA

GTAAGGATGGTGGACATTCTTAGGCAGAACCCAACAGAGGAGCAAGCTGTGGATATATGC

AAAGCAGCAATGGGTTTAAGAATCAGTTCATCCTTCAGCTTTGGAGGTTTCACTTTCAAA

AGGACAAGTGGGTCGTCTGTCAAAAGAGAAGAAGAAATACTCACTGGCAACCTCCAAACA

CTGAAAGTAAGAATACATGAAGGATATGAGGAATTCACAATGGTTGGGCGAAGAGCTACA

GCCATTTTGAGGAAAGCAACCAGGAGACTGATCCAATTAATAGTGAGTGGAAGAGACGAG

CAGTCAATCGCTGAAGCAATCATAGTGGCAATGGTTTTCTCACAGGAGGATTGCATGATA

AAAGCAGTACGAGGTGATTTGAATTTTGTCAACAGAGCGAATCAGCGGCTAAATCCTATG

CATCAACTTCTGAGGCATTTCCAAAAGGATGCAAAAGTGCTGTTTCAAAACTGGGGGATT

GAACCAATTGACAATGTAATGGGGATGATCGGGATACTGCCTGACATGACCCCCAGCACA

GAGATGTCACTGAGAGGAGTGAGAGTCAGCAAAATGGGAGTGGATGAATATTCCAGTACT

GAGAGAGTGGTCGTGAGCATTGATCGCTTCTTGAGAGTCCGAGATCAGAGGGGAAATGTG

CTTTTGTCTCCTGAGGAAGTTAGTGAAACACATGGAACAGAGAAACTGACGATAACGTAT

TCATCGTCGATGATGTGGGAAATCAATGGTCCGGAATCCGTGCTAGTCAACACATATCAA

TGGATCATTAGAAGTTGGGAAACTGTGAAGATTCAATGGTCCCAGGACCCTACGATGTTG

TACAATAAGATGGAATTTGAGCCCTTCCAATCCTTGGTGCCCAAGGCTGCTAGAGGCCAG

TATAGTGGGTTTGTGAGGACATTATTCCAACAGATGCGTGATGTGTTGGGGACATTTGAC

ACTGTCCAAATAATAAAGCTCCTACCATTTGCAGCAGCCCCACCGGAACAGAGTAGGATG

CAATTTTCCTCTCTGACTGTGAACGTAAGAGGTTCAGGAATGAGAATACTTGTGAGGGGC

AACTCCCCTGTGTTCAACTATAATAAGGCCACCAAGAGACTCACAGTTCTTGGAAAGGAT

GCAGGCGCCTTGACAGAATATCCAGATGAGGGAACAGCAGGAGTGGAGTCTGCAGTATTA

AGAGGATTTCTAATTCTGGGCAAAGAGGACAAAAGATATGGACCAGCATTGAGCATCAAC

GAATTGAGCAATCTTGCGAAAGGGGAAAAGGCTAATGTGTTGATAGGACAAGGAGACGTG

GTGTTGGTAATGAAACGGAAACGGGACTCTAGCATACTTACTGACAGCCAGACAGCGACC

AAAAGAATTCGGATGGCCATCAATTAG---------------------------------

----------------------

>A_duck_Bangladesh_43127_2020_EPI1902994

------------------------AGTATGAACAGAATAAAAGAACTAAGAGATCTAATG

TCACAGTCTCGCACCCGCGAGATACTGACAAAGACCACTGTGGACCATATGGCCATAATC

AAAAAATACACATCAGGAAGACAGGAAAAGAATCCCGCTCTCAGGATGAAATGGATGATG

GCAATGAAATACCCGATCACAGCTGACAAAAAGATAATGGAGATGATTCCTGAACGAAAT

GAACAAGGTCAAACTCTTTGGAGCAAGACAAATGATGCCGGGTCAGACAGAGTAATGGTA

TCACCTCTGGCTGTGACTTGGTGGAACAGGAATGGACCAACAACAAGCACAGTCCACTAT

CCCAAGGTGTACAAAACCTACTTTGAGAAGGTTGAAAGATTAAAACACGGAACCTTTGGG

CCCGTTCATTTCCGAAGTCAAGTCAAAATACGCCGCAGGGTTGACATAAATCCAGGCCAT

GCGGATCTCAGTGCGAAAGAAGCACAAGATGTTATAATGGAGGTTGTTTTCCCAAATGAA

GTTGGAGCGAGGATATTGACTTCAGAGTCACAAATGACAATAACAAAGGAAAAGAAAGAA

GAACTCCAGGATTGCAAGATTGCTCCATTGATGGTGGCATATATGTTGGAAAGAGAACTG

GTTCGCAAGACAAGATTCCTACCAGTGGCTGGCGGGACAAGCAGCGTGTATATAGAAGTG

TTACATCTGACTCAAGGAACTTGCTGGGAACAAATGTATACACCAGGAGGAGAAGTGAGA

AATGATGACATTGACCAGAGTCTAATCATTGCTGCTAGAAACATTGTGAGGAGAGCAACA

GTGTCGGCAGACCCATTAGCATCACTCTTGGAGATGTGCCACAGTACACAAATTGGCGGG

ATAAGAATGGTGGACATCCTTAGGCAAAATCCAACAGAAGAGCAAGCTGTAGACATATGC

AAGGCAGCAATGGGTCTGAGAATTAGCTCATCCTTCAGTTTTGGAGGTTTCACTTTCAAA

AGAACAAGTGGTTCTTCTATTAAAAGAGAGGAAGAAGTGCTTACAGGCAACCTCCAAACA

TTGAAAATAAGAGTGCATGAAGGATATGAAGAATTCACAATGGTTGGGCGAAGAGCAACA

GCAATTCTGAGGAAAGCAACCAGGAGGCTGATTCAATTGATAGTAAGTGGGAGAGATGAA

CAATCAATTGCTGAAGCAATCATTGTAGCAATGGTATTCTCACAAGAGGACTGCATGATA

AAGGCAGTCCGGGGTGATTTGAACTTTGTGAACAGAGCAAACCAACGGCTTAACCCCATG

CACCAACTCCTGAGGCACTTCCAAAAGGACGCAAAGGTACTATTCCAGAACTGGGGACTT

GAGACCATTGACAATGTAATGGGAATGGTTGGAATATTGCCTGATATGACTCCCAGTACG

GAAATGTCATTAAGGGGAGTGAGAGTCAGTAAAATGGGAGTAGACGAATATTCTAACACT

GAAAGAGTGGTCGTGAGCATTGATCGTTTCCTGAGAGTACGGGATCAACAAGGGAACGTA

CTCTTATCCCCTGAAGAAGTTAGTGAAACACAGGGAACGGAAAAGTTAACAATAACATAC

TCTTCATCTATGATGTGGGAGATTAACGGCCCAGAATCAGTGCTGGTCAACACATACCAA

TGGATCATTAGGAATTGGGAGAATGTGAAGATCCAATGGTCCCAAGACCCTACTACGTTA

TACAATAAGATGGAATTCGAGCCCTTTCAATCTTTGGTACCTAAAGCTGTTAGAGGTCAA

TACAGTGGGTTCGTGAGGACACTATTCCAGCAAATGCGTGATGTGTTGGGAACATTTGAC

ACTGTTCAAATAATAAAGCTTCTACCATTTGCAGCAGCACCACCAGAGCAAAGCAGAATG

CAATTTTCTTCTCTGACGGTGAATGTACGAGGATCTGGGATGAGAATACTTGTAAGAGGC

AACTCCCCTGTGTTTAACTATAACAAATCAACTAAGAGGCTCACAGTTCTCGGGAAAGAT

GCGGGTGCACTGACAGAAGATCCGAATGAGGGAACAGCAGGAGTAGAGTCTGCAGTACTG

AGAGGATTTCTAATTCTAGGCAGAGAAGACAAGAGATATGGACCAGCATTAAGCATTAAC

GAGTTGAGCAATCTTGCTAAAGGGGAGAAGGCTAATGTGTTGATAGGGCAAGGAGATGCG

GTGTTGGTAATGAAACGGAAACGGGACTCTAGCATACTTACTGACAGCCAGACAGCGACC

AAAAGAATTCGTATGGCCATCAATTAGTGTTGAATT------------------------

----------------------

>A_Guangdong_18SF020_2018_EPI1352810

-GCGAAAGCAGGTCAAATATATTCAGTATGAACAGAATAAAAGAACTAAGAGATCTAATG

TCACAGTCTCGCACCCGCGAGATACTGACAAAGACCACTGTGGACCATATGGCCATAATC

AAAAAATACACATCAGGAAGACAGGAAAAGAATCCCGCTCTCAGGATGAAATGGATGATG

GCAATGAAATACCCGATCACAGCTGACAAAAAGATAATGGAGATGATTCCTGAACGAAAT

GAACAAGGTCAAACTCTTTGGAGCAAGACAAATGATGCCGGGTCAGACAGAGTAATGGTA

TCACCTCTGGCTGTGACTTGGTGGAACAGGAATGGGCCAACAACAAGCACCGTCCATTAT

CCCAAGGTGTACAAAACCTACTTTGAAAAGGTTGAAAGATTAAAACACGGAACCTTTGGG

CCCGTTCATTTCCGGAGTCAAGTCAAAATACGCCGCAGGGTTGACATAAATCCAGGCCAT

GCGGATCTCAGTGCGAAAGAAGCACAAGATGTTATAATGGAGGTTGTTTTCCCAAACGAG

GTTGGAGCGAGGATATTGACTTCAGAGTCACAAATGACAATAACAAAGGAAAAGAAAGAA

GAACTCCAGGATTGTAAGATTGCGCCATTGATGGTGGCATATATGTTGGAAAGAGAACTG

GTTCGCAAGACAAGATTCCTACCAGTGGCTGGCGGGACAAGCAGCGTGTATATAGAAGTT

TTACATTTGACTCAAGGAACTTGCTGGGAGCAAATGTACACACCAGGAGGAGAAGTGAAA

AATGATGACATTGACCAGAGTCTAATCATTGCTGCTAGAAACATTGTGAGAAGAGCAACA

GTGTCAGCAGACCCATTAGCATCACTCTTGGAGATGTGCCACAGTACACAAATTGGCGGG

ATAAGAATGGTGGACATTCTTAGGCAAAATCCAACAGAAGAGCAAGCTGTAGACATATGC

AAGGCAGCAATGGGTCTGAGAATTAGTTCATCCTTCAGTTTTGGAGGTTTCACTTTCAAA

AGAACAAGTGGTTCTTCTATTAAAAGGGAGGAAGAAGTGCTTACAGGCAACCTCCAAACA

TTGAAAATAAGAGTGCATGAAGGATATGAAGAATTCACAATGGTTGGGCGAAGAGCAACA

GCAATTCTGAGGAAAGCAACCAGGAGGCTGATTCAATTGATAGTAAGTGGGAGAGACGAA

CAATCAATTGCTGAAGCAATCATTGTAGCAATGGTATTCTCACAAGAGGACTGCATGATA

AAGGCAGTCCGAGGTGATTTGAACTTTGTTAACAGAGCGAACCAACGCCTAAACCCCATG

CATCAACTCCTGAGGCACTTCCAAAAGGACGCAAAGGTACTATTCCAGAACTGGGGGCTT

GAGCCCATCGACAATGTAATGGGGATGGTGGGAATATTGCCTGATATGACTCCCAGTACG

GAAATGTCATTAAGGGGAGTGAGAGTCAGTAAAATGGGAGTAGACGAATATTCTAACACT

GAAAGAGTGGTCGTGAACATTGATCGTTTCCTGAGAGTACGGGATCAACAAGGGAACGTA

CTCTTATCCCCTGAAGAAGTTAGTGAAACACAGGGAACGGAAAAGTTAACAATAACATAT

TCTTCATCTATGATGTGGGAGATTAACGGCCCAGAATCAGTGCTGGTCAACACATACCAA

TGGATCATTAGGAATTGGGAGAATGTGAAGATCCAATGGTCCCAAGACCCTACTATGTTA

TACAATAAGATGGAATTCGAGCCCTTTCAGTCTTTGGTACCTAAAGCTGTTAGAGGTCAA

TACAGTGGGTTCGTGAGAACACTATTCCAGCAAATGCGTGATGTGTTGGGAACATTTGAC

ACTGTTCAAATAATAAAGCTGCTACCATTTGCAGCAGCACCACCARWGCAAAGCAGAATG

CAATTTTCTTCTCTGACGGTGAATGTACGAGGATCTGGAATGAGAATACTTGTAAGAGGC

AACTCCCCTGTGTTTAACTATAACAGATCAACTAAGAGACTCACAGTCCTCGGGAAAGAT

GCGGGTGCACTGACAGAAGATCCGAATGAGGGAACAGCAGGAGTAGAGTCTGCAGTACTG

AGAGGATTCCTAATTCTAGGCAGAGAAGACAAGAGATATGGACCAGCATTAAGCATTAAC

GAGTTGAGCAATCTTGCTAAAGGGGAGAAGGCTAATGTGTTGATAGGGCAAGGAGACGCG

GTGTTGGTAATGAAACGGAAACGGGACTCTAGCATACTTACTGACAGCCAGACAGCGACC

AAAAGAATTCGTATGGCCATCAATTAGTGTTGAATTGTTTAAAAACGAC-----------

----------------------

>A_goose_Hunan_116_2014_EPI958629

---------------------------ATGGAACGAATAAAGGAATTAAGAGATCTAATG

TCACAGTCCCGCACCCGCGAGATACTAACAAAAACCACTGTGGACCACATGGCCATAATC

AAGAAATACACATCGGGAAGGCAAGAGAAGAATCCTGCCCTCAGAATGAAATGGATGATG

GCAATGAAGTATCCGATTACAGCGGACAAGAGAATAATAGATATGATTCCTGAAAGGAAT

GAACAAGGGCAGACACTCTGGAGCAAGACAAATGATGCTGGGTCGGACAGGGTGATGGTG

TCCCTCCTAGCTGTAACTTGGTGGAATAGGAATGGGCCAACGACAAATACAGTTCATTAT

CCAAAAGTTTACAAAACATACTTCGAGAAGGTTGAAAGGTTAAAGCATGGAACATTCGGT

CCTGTCCATTTCCGAAACCAAGTTAAAATACGCCGCCGAGTTGATACAAACCCTGGCCAT

GCAGATCTCAATGCCAAAGAAGCACAAGATGTCATCATGGAAGTTGTTTTCCCAAATGAG

GTGGGAGCTAGAATACTGACTTCAGAGTCACAATTGACAATAACGAAAGAGAAGAAAGAA

GAGCTTCAAGATTGTAAGATTGCTCCCTTAATAGTTGCATACATGTTGGAGAGGGAACTG

GTCCGCAAAACCAGGTTCCTACCTGTAGCAGGCGGTACAAGCAGTGTGTACATTGAGGTA

CTGCACTTGACTCAAGGAACCTGCTGGGAACAGATGTACACCCCAGGCGGAGAAGTAAGA

AATGACGATGTTGACCAGAGTTTGATCATTGCTGCTAGAAACATTGTTAGGAGAGCAACG

GTGTCAGCGGATCCATTGGCATCACTGTTGGAGATGTGTCACAGTACACAAATTGGTGGA

ATAAGGATGGTGGACATCCTTAAGCAAAATCCAACTGAGGAACAAGCTGTGGATATATGC

AAAGCAGCAATGGGTCTGAGGATCAGCTCATCCTTTAGTTTTGGAGGTTTCACTTTCAAA

AGAACAAGTGGGTCATCCGTCACGAAAGAAGAGGAAGTGCTTACGGGCAACCTTCAGACA

TTAAAAATAAAAGTACATGAGGGGTATGAAGAATTCACAATGGTCGGGCAGAGAGCAACA

GCTATCCTGAGGAAAGCAACTAGGAGGCTGATTCAATTGATAGTAAGCGGGAGGAACGAA

CAATCAATCGCTGAGGCAATCATTGTAGCGATGGTGTTCTCACAGGAGGATTGCATGATA

AAAGCAGTACGAGGCGATCTAAATTTCGTAAACAGAGCAAACCAAAGATTAAATCCCATG

CATCAACTTCTGAGACATTTTCAAAAGGACGCCAAGGTGCTATTTCAAAATTGGGGAATT

GAACCCATTGATAATGTTATGGGGATGATCGGGATACTACCCGACATGACTCCCAGCACA

GGACTGTCATTGAGAGGAGTGAGAATTAGTAAAATGGGAGTGGATGAATATTCCAGCACT

GAAAGAGTAGTTGTAAGCATTGACCGTTTTTTAAGGGTTCGAGATCAGCGGGGAAATGTA

CTCTTATCTCCCGAAGAGGTCAGCGAAACTCAGGGAACAGAAAAGTTGACAATAACATAT

TCATCATCAATGATGTGGGAAATCAACGGTCCTGAATCAGTGCTCGTCAACACCTATCAA

TGGATCATCAGAAATTGGGAAACTGTGAAGATTCAATGGTCTCAGGATCCCACAATGCTA

TACAATAAGATGGAGTTTGAACCATTCCAATCCTTGGTACCTAAAGCTACCAGAGGTCAA

TACAGTGGATTTGTGAGAACATTATTTCAACAAATGCGTGATGTACTAGGGACATTTGAT

ACTGTCCAGATAATAAAGCTGCTACCATTTGCAGCAGCACCACCTGAGCAAAGCAGAATG

CAGTTTTCTTCTCTAATTGTGAATGTGAGGGGGTCAGGGATGAGAATACTCATAAGGGGC

AATTCCCCTGTGTTCAACTACAATAAGGCGACCAAAAGGCTTACTGTTCTTGGAAAGGAC

GCAGGTGCATTAACTGAGGATCCGGACGAGGGGGTAGCCGGAGTGGAATCTGCAGTATTG

AGGGGATTCCTAATTCTAGGCAAGGAGGACAAAAGATATGGACCAGCATTGAGCATCAAT

GAACTGAGCAATCTTGCAAAAGGGGAGAAAGCTAATGTGCTGATAGGGCAAGGAGACGTA

GTATTGGTAATGAAACGAAAACGGGACTCTAGCATACTTACTGACAGCCAGACAGCGACC

AAAAGAATTCGAATGGCCATCAATTAG---------------------------------

----------------------

>A_duck_Wuhan_JXYFB22_2015_EPI683188

---------------------------ATGGAACGAATAAAGGAATTAAGAGATCTAATG

TCACAGTCCCGCACCCGCGAGATACTAACAAAAACCACTGTGGACCACATGGCCATAATC

AAGAAATACACATCGGGAAGGCAAGAGAAGAATCCTGCCCTCAGAATGAAATGGATGATG

GCAATGAAGTATCCGATTACAGCGGACAAGAGAATAATAGATATGATTCCTGAAAGGAAT

GAACAAGGGCAGACACTCTGGAGCAAGACAAATGATGCTGGGTCGGACAGGGTGATGGTG

TCCCCCCTAGCTGTAACTTGGTGGAATAGGAATGGGCCAACGACAAATACAGTTCATTAT

CCAAAAGTTTACAAAACATACTTCGAGAAAGTTGAAAGGTTGAAGCATGGAACATTCGGT

CCTGTCCATTTCAGAAACCAAGTTAAAATACGCCGCCGAGTTGATACAAACCCTGGCCAT

GCAGATCTCAGTGCCAAAGAAGCACAGGATGTCATCATGGAAGTTGTTTTCCCAAATGAG

GTGGGAGCTAGAATATTGACTTCAGAGTCACAATTGACAATAACGAAAGAGAAGAAAGAA

GAGCTTCAAGATTGTAAGATTGCTCCCTTAATGGTTGCATACATGTTGGAGAGGGAATTG

GTCCGAAAAACCAGATTCCTACCTGTGGCAGGCGGTACAAGCAGTGTGTACATTGAGGTA

CTGCACTTGACTCAAGGAACCTGCTGGGAGCAGATGTACACTCCAGGCGGAGAAGTAAGA

AATGACGATGTTGACCAGAGTTTGATCATTGCTGCTAGAAACATTGTTAGGAGAGCAACG

GTGTCAGCGGATCCATTGGCATCACTGCTGGAGATGTGTCACAGTACACAAATTGGTGGA

ATAAGGATGGTGGACATCCTTAAGCAAAATCCAACTGAGGAACAAGCTGTGGATATATGC

AGAGCAGCCATGGGTCTGAGGATCAGCTCATCCTTTAGTTTTGGAGGTTTCACTTTCAAA

AGAACAAGTGGGTCATCCGTCACGAAAGAAGAGGAAGTGCTTACGGGCAATCTTCAGACA

TTAAAAATAAAAGTACATGAGGGGTATGAAGAATTCACAATGGTCGGGCAGAGAGCAACA

GCTATCCTGAGGAAAGCAACTAGGAGGCTGATTCAGTTGATAGTAAGCGGGAGGAACGAA

CAATCAATCGCTGAGGCAATCATTGTAGCGATGGTGTTCTCACAGGAGGATTGCATGATA

AAGGCAGTACGAGGCGATCTAAATTTCGTAAACAGAGCAAACCAAAGATTAAATCCCATG

CATCAACTCCTGAGACATTTTCAAAAGGACGCCAAGGTGCTATTTCAGAATTGGGGAATT

GAACCCATTGACAATGTCATGGGGATGATCGGGATACTACCCGACATGACTCCCAGCACA

GAACTGTCATTGAGAGGAGTGAGAATTAGTAAAATGGGAGTGGATGAATATTCCAGCACT

GAAAGAGTAGTTGTAAGCATTGACCGCTTTTTAAGGGTTCGAGATCAGCGAGGAAATGTA

CTCTTATCTCCCGAAGAGGTCAGCGAAACTCAGGGAACAGAAAAGTTGACAATAACATAT

TCATCATCAATGATGTGGGAAATCAACGGTCCTGAATCAGTGCTTGTCAACACCTATCAA

TGGATCATCAGAAATTGGGAAACTGTGAAGATTCAATGGTCTCAGGATCCCACAATGCTA

TACAATAAGATGGAGTTTGAACCATTCCAATCCTTGGTACCTAAAGCTACCAGAGGTCAA

TACAGTGGATTTGTTAGAACATTATTTCAACAAATGCGTGATGTACTAGGGACATTTGAT

ACTGCCCAGATAATAAAGCTGCTACCATTTGCAGCAGCACCACCTGAGCAGAGCAGAATG

CAGTTTTCTTCTCTAATTGTGAATGTGAGGGGGTCAGGGATGAGAATACTTATAAGGGGC

AATTCCCCTGTGTTCAACTACAATAAGGCGACTAAAAGGCTTACTGTTCTTGGAAAGGAC

GCAGGTGCATTAACTGAGGGTCCGGACGAGGGGGCAGCCGGAGTGGAATCTGCAGTATTG

AGGGGATTCCTGATTCTGGGAAAGGAGGACAAAAGATACGGACCAGCATTGAGCATCAAT

GAACTGAGCAATCTTGCAAAAGGGGAGAAAGCTAATGTGCTGATAGGGCAAGGAGACGTG

GTGTTGGTAATGAAACGGAAACGGGACTCTAGCATACTTACTGACAGCCAGACAGCGACC

AAAAGAATTCGAATGGCCATCAATTAG---------------------------------

----------------------

>A_chicken_Vietnam_NCVD-15A59_2015_EPI895060

------------TCAAATATATTCAATATGGAGCGAATAAAGGAATTAAGAGATCTAATG

TCACAGTCCCGCACCCGCGAGATACTAACAAAAACCACTGTGGACCACATGGCCATAATC

AAGAAGTACACATCGGGAAGGCAAGAGAAGAATCCTGCCCTCAGAATGAAATGGATGATG

GCAATGAAGTATCCGATTACAGCGGACAAGAGAATAATAGACATGATTCCTGAAAGGAAT

GAACAAGGGCAGACACTCTGGAGCAAGACAAATGATGCTGGGTCGGACAGGGTGATGGTG

TCCCCCCTAGCTGTAACTTGGTGGAATAGGAATGGGCCAACGACAAATACAGTTCATTAC

CCAAAAGTTTACAAAACATACTTCGAGAAGGTTGAAAGGTTAAAGCATGGAACATTCGGT

CCTGTCCATTTCCGAAACCAAGTTAAAATACGCCGCCGAGTTGATACAAACCCTGGCCAT

GCAGATCTCAGTGCCAAAGAAGCACAAGATGTCATCATGGAAGTTGTTTTCCCAAATGAG

GTGGGAGCTAGAATATTGACTTCAGAGTCACAATTGACAATAACGAAAGAGAAGAAAGAA

GAGCTTCAAGATTGTAAGATTGCTCCCTTAATGGTTGCATACATGTTGGAGAGGGAACTG

GTCCGCAAAACCAGATTCCTGCCCGTAGCAGGCGGTACAAGCAGTGTGTATATTGAGGTA

CTGCACTTGACTCAAGGAACCTGCTGGGAGCAGATGTACACTCCAGGCGGAGAAGTAAGA

AATGACGATGTTGACCAGAGTTTGATCATTGCTGCTAGAAACATTGTTAGGAGAGCAACG

GTGTCAGCGGATCCATTGGCATCACTGCTGGAGATGTGTCACAGTACACAAATTGGTGGA

ATAAGGATGGTGGACATCCTTAAGCAAAATCCAACTGAGGAACAAGCTGTGGATATATGC

AGAGCAGCAATGGGTCTGAGGATCAGCTCATCCTTTAGTTTTGGAGGTTTCACTTTCAAA

AGAACAAGTGGGTCATCCGTCACGAAAGAAGAGGAAGTGCTTACGGGCAATCTTCAGACA

TTAAAAATAAAGGTACATGAGGGGTATGAAGAATTCACAATGGTCGGGCAGAGAGCAACA

GCTATCCTGAGGAAAGCAACTAGGAGGCTGATTCAGTTGATAGTAAGCGGGAGGAACGAA

CAATCAATCGCTGAGGCAATCATTGTAGCGATGGTGTTCTCACAGGAGGATTGCATGATA

AAGGCAGTACGAGGCGATCTAAATTTCGTAAACAGAGCAAACCAAAGATTAAATCCCATG

CATCAACTCCTGAGACATTTTCAAAAGGACGCCAAGGTGCTATTTCAGAATTGGGGAATT

GAACCCATTGATAATGTTATGGGGATGATCGGGATACTACCCGACATGACTCCCAGCACA

GAACTGTCATTGAGAGGAGTGAGAATTAGTAAAATGGGAGTGGATGAATATTCCAGCACT

GAAAGAGTAGTTGTAAGCATTGACCGCTTTTTAAGAGTTCGAGATCAACGGGGAAATGTA

CTTTTATCTCCCGAAGAGGTCAGCGAAACTCAGGGAACAGAAAAGTTGACAATAACATAT

TCATCATCAATGATGTGGGAAATCAACGGTCCTGAATCAGTGCTTGTCAACACCTATCAA

TGGATCATCAGAAATTGGGAAACTGTGAAGATTCAATGGTCTCAGGATCCCACAATGCTA

TATAATAAGATGGAGTTTGAACCATTCCAATCTTTGGTACCTAAAGCTACCAGAGGTCAA

TACAGTGGATTTGTGAGAACATTATTTCAACAAATGCGTGATGTACTAGGGACATTTGAT

ACTGCCCAGATAATAAAGCTGCTACCATTTGCAGCAGCACCACCTGAGCAGAGCAGAATG

CAGTTTTCTTCTCTAATTGTGAATGTGAGGGGGTCAGGGATGAGAATACTCATAAGGGGC

AATTCCCCTGTGTTCAACTACAATAAGGCGACCAAAAGGCTCACTGTTCTTGGAAAGGAC

GCAGGCGCATTAACTGAGGATCCGGATGAGGGGGCAGCCGGAGTGGAATCTGCAGTATTG

AGGGGATTCCTAATCCTAGGCAAGGAGGACAAAAGATATGGACCAGCATTGAGCATCAAT

GAACTGAGCAATCTTGCAAAAGGGGAGAAAGCTAATGTGCTGATAGGGCAAGGAGACGTA

GTGTTGGTAATGAAACGAAAACGGGACTCTAGCATACTTACTGACAGCCAGACAGCGACC

AAAAGAATTCGAATGGCCATCAATTAGTGTCGAATTATTTAAAAACGA------------

----------------------

>A_chicken_Vietnam_NCVD-15A55_2015_EPI895044

------------TCAAATATATTCAGTATGAACAGAATAAAAGAACTAAGAGATCTAATG

TCACAGTCTCGCACCCGCGAGATACTGACAAAGACCACTGTGGACCATATGGCCATAATC

AAAAAATACACATCAGGAAGACAGGAAAAGAATCCTGCTCTCAGGATGAAATGGATGATG

GCAATGAAATACCCGATCACAGCTGACAAAAAGATAATGGAAATGATTCCTGAACGAAAT

GAACAAGGTCAAACTCTTTGGAGCAAGACAAATGATGCCGGGTCAGACAGAGTAATGGTA

TCACCTCTGGCTGTGACTTGGTGGAACAGGAATGGGCCAACAACAAGCACAGTCCATTAC

CCCAAGGTATACAAAACCTACTTTGAGAAGGTTGAAAGATTAAAACACGGAACCTTTGGC

CCCGTTCATTTCCGGAATCAAGTCAAAATACGCCGCAGGGTTGACATAAATCCAGGCCAT

GCGGATCTCAGTGCGAAAGAAGCACAAGATGTTATAATGGAGGTTGTTTTCCCAAACGAA

GTTGGAGCTAGGATATTGGCTTCAGAGTCACAAATGACAATAACAAAGGAAAAGAAAGAA

GAACTCCAGGATTGTAAGATTGCCCCATTGATGGTGGCATATATGTTGGAAAGAGAACTG

GTTCGCAAGACAAGATTCCTACCAGTGGCTGGCGGGACAAGCAGCGTGTATATAGAAGTG

TTACATTTGACCCAAGGAACTTGCTGGGAACAAATGTACACACCAGGAGGGGAAGTGAGA

AATGATGACATTGACCAGAGTCTAATCATTGCTGCTAGAAACATTGTGAGGAGAGCAACA

GTGTCGGCAGACCCGTTGGCATCACTCTTGGAGATGTGCCACAGTACACAAATTGGCGGG

ATAAGAATGGTGGACATTCTTAGGCAAAATCCAACAGAAGAGCAAGCTGTAGACATATGC

AAGGCAGCAATGGGCCTGAGAATTAGTTCATCCTTCAGTTTTGGAGGTTTCACTTTCAAA

AGAACAAGTGGTTCTTCTATTAAAAGAGAGGAAGAAGTGCTTACAGGCAACCTCCAAACA

TTGAAAATAAGAGTGCATGAAGGGTATGAAGAGTTCACAATGGTTGGGCGAAGAGCAACA

GCAATTCTGAGGAAAGCAACCAGGAGGCTGATTCAATTGATAGTAAGTGGGAGAGATGAA

CAATCAATTGCTGAAGCAATCATTGTAGCAATGGTATTCTCACAAGAGGACTGCATGATA

AAGGCAGTCCGAGGTGATTTGAATTTTGTGAACAGAGCGAACCAACGGCTGAACCCCATG

CATCAACTCCTGAGGCACTTCCAAAAGAACGCAAAGGTACTATTCCAGAATTGGGGACTT

GAGCACATCGACAATGTAATGGGGATGGTTGGAATATTGCCTGACATGACTCCCAGTACG

GAAATGTCATTAAGAGGAGTGAGAGTCAGTAAAATGGGAGTGGACGAATATTCTAACACT

GAAAGGGTGGTCGTGAGCATTGATCGTTTCCTGAGAGTACGGGATCAGCAAGGGAACGTA

CTCTTATCCCCTGAAGAAGTTAGTGAAACACAGGGAACGGAAAAGTTAACGATAACATAT

TCATCATCTATGATGTGGGAGATTAACGGCCCAGAATCAGTGCTGGTCAACACATACCAA

TGGATCATTAGAAATTGGGAGAGTGTGAAGATTCAATGGTCCCAAGACCCTACTATGTTA

TACAATAAGATGGAATTCGAGCCCTTTCAATCTTTGGTACCTAAAGCTGTTAGAGGTCAA

TACAGTGGGTTCGTAAGGACACTATTCCAGCAAATGCGTGATGTACTGGGAACATTTGAC

ACTGTTCAAATAATAAAGCTGCTACCATTTGCAGCAGCACCACCAGAACAAAGCAGAATG

CAATTTTCTTCTCTGACAGTGAATGTACGGGGATCTGGAATGAGAATACTTGTAAGAGGC

AACTCCCCTGTGTTTAACTATAACAAATCAACTAAGAGGCTCACAGTCCTCGGGAAAGAT

GCAGGTGCACTTACAGAAGATCCGAATGAGGGAACAGCAGGAGTAGAATCTGCAGTACTA

AGAGGATTTCTAATCCTAGGCAAAGAAGACAAGAGATATGGGCCAGCATTGAGCATTAAC

GAGTTGAGCAATCTTGCTAAAGGGGAGAAGGCTAATGTGTTGATAGGGCAAGGAGACGCG

GTGTTGGTGATGAAACGGAAACGGGACTCTAGCATACTTACTGACAGCCAGACAGCGACC

AAAAGAATTCGTATGGCCATCAATTAGTGTTGAATTGTTTAAAAACGA------------

----------------------

>A_tundra_swan_Niigata_5112007_2016_EPI1184359

--------CAGGTCAAATATATTCAGCATGAACAGAATAAAAGAACTAAGAGATCTAATG

TCACAGTCTCGCACCCGCGAGATACTGACAAAGACCACTGTGGACCATATGGCCATAATC

AAAAAATACACATCAGGAAGACAGGAAAAGAATCCTGCTCTCAGGATGAAATGGATGATG

GCAATGAAATACCCGATCACAGCTGACAAAAAGATAATGGAGATGATTCCTGAACGAAAT

GAACAAGGTCAAACTCTTTGGAGCAAGACAAATGATGCCGGTTCAGACAGAGTAATGGTA

TCACCTCTGGCTGTGACTTGGTGGAACAGGAATGGGCCAACAACAAGCACAGTCCATTAT

CCCAAGGTGTACAAAACCTACTTTGAAAAGGTTGAAAGATTAAAACACGGAACTTTTGGC

CCCGTTCATTTCCGGAATCAAGTCAAAATACGCCGCAGGGTTGACATAAATCCAGGCCAT

GCGGATCTCAGTGCGAAAGAAGCACAAGATGTTATAATGGAGGTTGTTTTCCCAAACGAA

GTTGGAGCTAGGATATTGGCTTCAGAGTCACAAATGACAATAACAAAGGAAAAGAAAGAA

GAACTCCAGGATTGTAAGATTGCTCCATTGATGGTGGCATATATGTTGGAAAGAGAACTG

GTTCGCAAAACAAGATTCCTACCAGTGGCTGCCGGGACAAGCAGCGTGTATATAGAAGTG

TTACATTTGACTCAAGGAACTTGCTGGGAACAAATGTACACACCAGGAGGGGAAGTGAGA

AATGATGATATTGACCAGAGTCTAATCATTGCTGCTAGAAACATTGTGAGAAGAGCAACA

GTGTCGGCAGACCCGTTGGCATCACTCTTGGAGATGTGCCACAGTACACAAATTGGCGGG

ATAAGAATGGTGGACATTCTTAGGCAAAATCCAACAGAAGAGCAAGCTGTGGACATATGC

AAGGCAGCAATGGGTCTGAGAATTAGTTCATCCTTCAGTTTTGGAGGTTTCACTTTCAAA

AGAACAAGTGGTTCTTCTATTAAAAGAGAGGAAGAAGTGCTTACAGGCAACCTCCAAACA

TTGAAAATAAGAGTGCATGAAGGGTATGAAGAATTCACAATGGTTGGGCGAAGAGCAACA

GCAATTCTGAGGAAGGCAACCAGGAGGCTGATTCAATTGATAGTAAGTGGGAGAGACGAA

CAATCAATTGCTGAAGCAATCATTGTAGCAATGGTATTCTCACAAGAAGACTGCATGATA

AAGGCAGTCCGAGGTGATTTGAATTTTGTGAACAGAGCGAACCAACGGTTGAACCCCATG

CATCAACTCCTGAGGCATTTCCAAAAGAACGCAAAGGTACTATTCCAGAATTGGGGACTT

GAGCCCATCGACAATGTAATGGGGATGGTTGGGATATTGCCTGACATGACTCCCAGTACG

GAAATGTCATTAAGAGGAGTGAGAGTCAGTAAAATGGGAGTAGACGAATATTCTAATACT

GAAAGAGTGGTCGTGAGCATTGATCGTTTCCTGAGAGTACGGGATCAGCAAGGGAACGTA

CTCTTATCCCCTGAAGAAGTTAGTGAAACACAGGGAACGGAAAAGTTAACGATAACATAT

TCATCATCTATGATGTGGGAGATTAATGGCCCAGAATCAGTGCTGGTCAACACATACCAA

TGGATCATTAGGAATTGGGAGAGTGTGAAGATTCAATGGTCCCAAGACCCTACTATGTTA

TACAATAAGATGGAATTCGAGCCCTTTCAATCTTTGGTACCTAAAGCTGTTAGAGGTCAA

TACAGTGGGTTCGTGAGGACACTATTCCAGCAAATGCGTGATGTGCTGGGAACATTTGAT

ACTGTTCAAATAATAAAACTGCTACCATTTGCAGCAGCACCACCAGAACAAAGCAGAATG

CAATTTTCTTCTCTGACAGTGAATGTGCGGGGATCTGGAATGAGAATACTTGTAAGAGGC

AACTCCCCTGTGTTTAACTATAACAAATCAACTAAGAGGCTCACAGTCCTCGGGAAAGAT

GCAGGTGCACTTACAGAAGATCCGAATGAGGGAACAGCAGGAGTAGAATCTGCAGTACTA

AGAGGATTTCTAATCCTAGGCAAAGAAGACAAGAGATATGGGCCAGCATTGAGCATTAAC

GAGTTGAGCAATCTTGCTAAAGGGGAGAAGGCTAATGTGTTGATAGGGCAAGGAGACGCG

GTGTTGGTGATGAAACGGAAACGGGACTCTAGCATACTTACTGACAGCCAGACAGCGACC

AAAAGAATTCGTATGGCCATCAATTAGTGTTGAATTGTTTAAAAACGACCTTGT------

----------------------

>A_duck_Hyogo_1_2016_EPI866704

---------------------------ATGAACAGAATAAAAGAACTAAGAGATCTAATG

TCACAGTCTCGCACCCGCGAGATACTGACAAAGACCACTGTGGACCATATGGCCATAATC

AAAAAATACACATCAGGAAGGCAGGAAAAGAATCCTGCTCTCAGGATGAAATGGATGATG

GCAATGAAATACCCGATCACAGCTGACAAAAAGATAATGGAGATGATTCCTGAACGAAAT

GAACAAGGTCAAACTCTTTGGAGCAAGACAAATGATGCCGGTTCAGACAGAGTAATGGTA

TCACCTCTGGCTGTGACTTGGTGGAACAGGAATGGGCCAACAACAAGCACAGTCCATTRT

CCCAAGGTGTACAAAACCTACTTTGAAAAGGTTGAAAGATTAAAACACGGAACTTTCGGC

CCCGTTCATTTCCGGAATCAAGTCAAAATACGCCGCAGGGTTGACATAAATCCAGGCCAT

GCGGATCTCAGTGCGAAAGAAGCACAAGATGTTATAATGGAGGTTGTTTTCCCAAACGAA

GTTGGAGCTAGGATATTGGCTTCAGAGTCACAAATGACAATAACAAAGGAAAAGAAAGAA

GAACTCCAGGATTGTAAGATTGCTCCATTGATGGTGGCATATATGTTGGAAAGAGAACTG

GTTCGCAAGACAAGATTCCTACCAGTGGCTGGCGGGACAAGCAGCGTGTATATAGAAGTG

TTACATTTGACTCAAGGGACTTGCTGGGAACAAATGTACACACCAGGAGGGGAAGTGAGA

AATGATGATATTGACCAGAGTCTAATCATTGCTGCTAGAAACATTGTGAGAAGAGCAACA

GTGTCGGCAGACCCGTTGGCATCACTCTTGGAGATGTGCCACAGTACACAAATTGGCGGG

ATAAGAATGGTGGACATTCTTAGGCAAAATCCAACAGAAGAGCAAGCTGTGGACATATGC

AAGGCAGCAATGGGTCTGAGAATTAGTTCATCCTTCAGTTTTGGAGGTTTCACTTTCAAA

AGAACAAGTGGTTCTTCTATTAAGAGAGAGGAAGAAGTGCTTACAGGCAACCTCCAAACA

TTGAAAATAAGAGTGCATGAAGGGTATGAAGAATTCACAATGGTTGGGCGAAGAGCAACA

GCAATTCTGAGGAAAGCAACCAGGAGGCTGATTCAATTGATAGTAAGTGGGAGAGACGAA

CAATCAATTGCTGAAGCAGTCATTGTAGCAATGGTATTCTCACAAGAAGACTGCATGATG

AAGGCAGTCCGAGGTGATTTGAATTTTGTGAACAGAGCGAACCAACGGTTGAACCCCATG

CATCAACTCCTGAGGCATTTCCAAAAGAACGCAAAGGTACTATTCCAGAATTGGGGACTT

GAGCCCATCGACAATGTAATGGGGATGGTTGGAATATTGCCTGACATGACTCCCAGTACG

GAAATGTCATTAAGAGGAGTGAGAGTCAGTAAAATGGGAGTAGACGAATATTCTAATACT

GAAAGAGTGGTCGTGAGCATTGACCGTTTCCTGAGAGTACGGGATCAGCAAGGGAACGTA

CTCTTATCCCCTGAAGAAGTTAGTGAAACACAGGGAACGGAAAAGTTAACGATAACATAT

TCATCATCTATGATGTGGGAGATTAATGGCCCAGAATCAGTGCTGGTCAACACATACCAA

TGGATCATTAGGAATTGGGAGAGTGTGAAGATTCAATGGTCCCAAGACCCTACTATGTTA

TACAATAAGATGGAATTCGAGCCCTTTCAATCTTTGGTACCTAAAGCTGTTAGAGGTCAA

TACAGTGGGTTCGTGAGGACACTATTCCAGCAAATGCGTGATGTGCTGGGAACATTTGAC

ACTGTTCAAATAATAAAACTGCTACCATTTGCAGCAGCACCACCAGAACAAAGCAGAATG

CAATTTTCTTCTCTGACAGTGAATGTGCGGGGATCTGGAATGAGAATACTTGTAAGAGGC

AACTCCCCTGTGTTTAACTATAACAAATCAACTAAGAGGCTCACAGTCCTCGGGAAAGAT

GCAGGTGCACTTACAGAAGATCCGAATGAGGGAACAGCAGGAGTAGAATCTGCAGTGCTA

AGAGGATTTCTAATCCTAGGCAAAGAAGACAAGAGATATGGGCCAGCATTGAGCATTAAC

GAGTTGAGCAATCTTGCTAAAGGGGAGAAGGCTAATGTGTTGATAGGGCAAGGAGACGCG

GTGTTGGTGATGAAACGGAAACGGGACTCTAGCATACTTACTGACAGCCAGACAGCGACC

AAAAGAATTCGTATGGCCATCAATTAG---------------------------------

----------------------

>A_Hubei_29578_2016_x_PR8_CNIC-HB2957_1369962

----AAAGCAGGTCAATTATATTCAATATGGAAAGAATAAAAGAACTACGAAATCTAATG

TCGCAGTCTCGCACCCGCGAGATACTCACAAAAACCACCGTGGACCATATGGCCATAATC

AAGAAGTACACATCAGGAAGACAGGAGAAGAACCCAGCACTTAGGATGAAATGGATGATG

GCAATGAAATATCCAATTACAGCAGACAAGAGGATAACGGAAATGATTCCTGAGAGAAAT

GAGCAAGGACAAACTTTATGGAGTAAAATGAATGATGCCGGATCAGACCGAGTGATGGTA

TCACCTCTGGCTGTGACATGGTGGAATAGGAATGGACCAATAACAAATACAGTTCATTAT

CCAAAAATCTACAAAACTTATTTTGAAAGAGTCGAAAGGCTAAAGCATGGAACCTTTGGC

CCTGTCCATTTTAGAAACCAAGTCAAAATACGTCGGAGAGTTGACATAAATCCTGGTCAT

GCAGATCTCAGTGCCAAGGAGGCACAGGATGTAATCATGGAAGTTGTTTTCCCTAACGAA

GTGGGAGCCAGGATACTAACATCGGAATCGCAACTAACGATAACCAAAGAGAAGAAAGAA

GAACTCCAGGATTGCAAAATTTCTCCTTTGATGGTTGCATACATGTTGGAGAGAGAACTG

GTCCGCAAAACGAGATTCCTCCCAGTGGCTGGTGGAACAAGCAGTGTGTACATTGAAGTG

TTGCATTTGACTCAAGGAACATGCTGGGAACAGATGTATACTCCAGGAGGGGAAGTGAGG

AATGATGATGTTGATCAAAGCTTGATTATTGCTGCTAGGAACATAGTGAGAAGAGCTGCA

GTATCAGCAGATCCACTAGCATCTTTATTGGAGATGTGCCACAGCACACAGATTGGTGGA

ATTAGGATGGTAGACATCCTTAGGCAGAACCCAACAGAAGAGCAAGCCGTGGATATATGC

AAGGCTGCAATGGGACTGAGAATTAGCTCATCCTTCAGTTTTGGTGGATTCACATTTAAG

AGAACAAGCGGATCATCAGTCAAGAGAGAGGAAGAGGTGCTTACGGGCAATCTTCAAACA

TTGAAGATAAGAGTGCATGAGGGATATGAAGAGTTCACAATGGTTGGGAGAAGAGCAACA

GCCATACTCAGAAAAGCAACCAGGAGATTGATTCAGCTGATAGTGAGTGGGAGAGACGAA

CAGTCGATTGCCGAAGCAATAATTGTGGCCATGGTATTTTCACAAGAGGATTGTATGATA

AAAGCAGTCAGAGGTGATCTGAATTTCGTCAATAGGGCGAATCAACGATTGAATCCTATG

CATCAACTTTTAAGACATTTTCAGAAGGATGCGAAAGTGCTTTTTCAAAATTGGGGAGTT

GAACCTATCGACAATGTGATGGGAATGATTGGGATATTGCCCGACATGACTCCAAGCATC

GAGATGTCAATGAGAGGAGTGAGAATCAGCAAAATGGGTGTAGATGAGTACTCCAGCACG

GAGAGGGTAGTGGTGAGCATTGACCGTTTTTTGAGAATCCGGGACCAACGAGGAAATGTA

CTACTGTCTCCCGAGGAGGTCAGTGAAACACAGGGAACAGAGAAACTGACAATAACTTAC

TCATCGTCAATGATGTGGGAGATTAATGGTCCTGAATCAGTGTTGGTCAATACCTATCAA

TGGATCATCAGAAACTGGGAAACTGTTAAAATTCAGTGGTCCCAGAACCCTACAATGCTA

TACAATAAAATGGAATTTGAACCATTTCAGTCTTTAGTACCTAAGGCCATTAGAGGCCAA

TACAGTGGGTTTGTAAGAACTCTGTTCCAACAAATGAGGGATGTGCTTGGGACATTTGAT

ACCGCACAGATAATAAAACTTCTTCCCTTCGCAGCCGCTCCACCAAAGCAAAGTAGAATG

CAGTTCTCCTCATTTACTGTGAATGTGAGGGGATCAGGAATGAGAATACTTGTAAGGGGC

AATTCTCCTGTATTCAACTATAACAAGGCCACGAAGAGACTCACAGTTCTCGGAAAGGAT

GCTGGCACTTTAACTGAAGACCCAGATGAAGGCACAGCTGGAGTGGAGTCCGCTGTTCTG

AGGGGATTCCTCATTCTGGGCAAAGAAGACAAGAGATATGGGCCAGCACTAAGCATCAAT

GAACTGAGCAACCTTGCGAAAGGAGAGAAGGCTAATGTGCTAATTGGGCAAGGAGACGTG

GTGTTGGTAATGAAACGGAAACGGGACTCTAGCATACTTACTGACAGCCAGACAGCGACC

AAAAGAATTCGGATGGCCATCAATTAGTGTCGAATAGTTTAAAAACGACCTTGTTTCTAC

T---------------------

>A_chicken_Hubei_ZYSJF38_2016_EPI895218

---------------------------ATGGAAAGAATAAAAGAACTAAGAGATTTGATG

TCACAGTCTCGCACTCGCGAGATACTGACAAAAACAACAGTGGACCATATGGCCATAATC

AAGAAATATACATCAGGAAGACAGGAGAAGAATCCTGCCCTTAGGATGAAGTGGATGATG

GCGATGAAATATCCAATCACAGCAGACAAAAGGATAATGGAGATGATCCCAGAAAGAAAT

GAGCAAGGTCAGACTCTTTGGAGCAAGACAAATGATGCCGGATCAGACAGGGTGATGGTG

TCACCTCTGGCTGTGACGTGGTGGAATAGAAATGGGCCAACAACAAGTACAGTCCATTAT

CCAAAGGTCTACAAAACCTATTTTGAAAAGGTCGAAAGGCTAAAACATGGAACCTTTGGC

CCCGTTCACTTCCGAAACCAGGTTAAAATACGCCGCAGGGTCGACATAAACCCAGGCCAT

GCAGATCTTAGTGCTAAAGAAGCACAAGATGTCATCATGGAGGTCGTATTCCCAAACGAA

GTTGGAGCCAGGATACTGACATCAGAGTCACAGTTAACGATAACCAAGGAAAAGAAGGAG

GAGCTTCAGGACTGCAAAATTGCTCCTTTAATGGTGGCATACATGTTGGAGAGAGAACTG

GTTCGCAAAACAAGGTTTCTACCAGTGGCTGGAGGGACAAGCAGTGTGTATATCGAAGTA

TTGCATTTGACCCAAGGAACCTGTTGGGAGCAAATGTACACACCAGGAGGGGAAGTGAGA

AATGATGATGTTGATCAGAGTTTAATTATTGCTGCTAGAAATATTGTTAGAAGGGCAACA

GTATCAGCAGACCCATTGGCTTCGCTTTTGGAGATGTGCCATAGTACACAGATTGGAGGG

GTTAGGATGGTTGACATCCTCAGACAAAACCCAACAGAGGAACAGGCTGTGGATATATGC

AAAGCAGCAATGGGTCTAAGGATCAGTTCATCCTTCAGCTTCGGAGGTTTCACTTTTAAG

AGGACAAGTGGGTCATCTGTCAAAAGGGAAGAAGAAGTGCTCACAGGCAACCTCCAAACA

TTGAAAATAAGAGTACATGAAGGATATGAGGAATTCACAATGGTTGGGAGAAGAGCAACA

GCCATTCTAAGGAAAGCAACCAGAAGACTGATTCAACTGATAGTGAGTGGGAAAGACGAG

CAATCAATCGCCGAGGCAATCATAGTGGCAATGGTGTTCTCACAAGAGGATTGTATGATA

AAGGCAGTGAGAGGTGATTTGAACTTTGTCAACAGAGCGAACCAGCGGCTAAATCCCATG

CATCAACTCCTGAGGCATTTCCAAAAGGATGCAAAGGTCCTGTTCCAAAACTGGGGAATT

GAGCCCATTGACAATGTAATGGGGATGATCGGAATATTGCCTGACATGACCCCCAGCACA

GAGATGTCCTTGAGAGGAGTGAGAGTTAGTAAAATGGGAGTAGATGAATATTCCAGTACC

GAGAGAGTGGTCGTGAGTATTGATCGTTTCTTGAGGGTTCGAGACCAGAGAGGAAACATA

CTCCTGTCTCCTGAGGAGGTTAGTGAAACACAGGGAACAGAAAAGTTGACTATAACATAT

TCATCGTCCTTGATGTGGGAAATCAATGGTCCGGAATCAGTGCTAGTTAACACCTATCAA

TGGATCATTAGAAATTGGGAAACTGTAAAGATTCAATGGTCCCAGGACCCTACAATTCTA

TACAATAAAATGGAATTTGAACCCTTTCAATCCCTAGTGCCCAAAGCTGCCAGAGGCCAA

TATAGTGGATTCGTAAGGGTCCTATTTCAGCAGATGCGTGACGTACTGGGGACGTTCGAC

ACCGTCCAAATAATAAAGCTACTACCATTTGCAGCAGCCCCGCCGGAACAGAGTAGGATG

CAGTTCTCTTCTCTAACTGTGAACGTAAGAGGTTCAGGAATGAAAGTGGTTGTGAGAGGC

AATTCTCCTGTGTTCAACTACAACAAGGCAACAAAAAGGCTTACAGTGCTTGGGAAGGAT

GCAGGTGCGTTAATGGAAGACCCAGACGAGGGAACAGCAGGAGTAGAATCTGCGGTATTG

AGAGGATTTCTGATTCTAGGCAAAGAAGACAAAAGGTATGGGCCAGCATTGAGCATCAAC

GAGTTGAGCAACCTTGCGAAAGGGGAAAAGGCTAATGTGTTGATAGGGCAAGGAGACGTG

GTGTTGGTACTGAAACGGAAACGGGACTCTAGCATACTTACTGACAGTCAGACAGCGACC

AAAAGGATTCGGATGGCCATCAATTAATGTCGAAT-GTTAAG------------------

----------------------

>A_gyrfalcon_Washington_41088-6_2014_EPI569380

---------------------------ATGGAGAGAATAAAAGAACTAAGAGATCTAATG

TCTCAATCCCGCACTCGCGAGATACTAACAAAAACCACTGTGGACCATATGGCCATAATC

AAGAAATACACATCAGGAAGACAAGAGAAGAATCCTGCTCTCAGAATGAAATGGATGATG

GCAATGAAATATCCAATCACAGCAGACAAGAGAATAATGGAAATGATTCCTGAAAGAAAT

GAACAAGGCCAGACGCTTTGGAGCAAGACAAATGATGCTGGATCAGACAGAGTGATGGTG

TCTCCCCTAGCTGTAACTTGGTGGAATAGAAATGGACCGACAGCAAGTACAGTCCATTAT

CCAAAGGTCTACAAAACATACTTTGAGAAGGTTGAAAGGTTAAAGCATGGAACCTTCGGT

CCCGTTCACTTCCGAAACCAAATTAAAATACGCCGCCGAGTTGACATAAACCCAGGCCAC

GCAGATCTCAGTGCCAAAGAAGCACAAGATGTCATCATGGAGGTTGTTTTCCCAAATGAA

GTGGGAGCTAGAATATTGACATCAGAGTCACAATTGACAATAACGAAAGAGAAAAAAGAA

GAACTCCAGGATTGCAAGATTGCTCCTTTAATGGTGGCATACATGTTGGAAAGAGAACTG

GTCCGCAAAACCAGATTCCTACCAGTAGCAGGTGGGACAAGCAGTGTGTACATTGAGGTA

CTGCACTTGACTCAAGGGACCTGCTGGGAACAGATGTACACTCCAGGCGGAAAAGTGAGG

AATGACGATGTTGACCAGAGTTTGATCATCGCGGCCAGAAACATTGTTAGGAGAGCAACG

GTATCAGCGGATCCACTGGCATCATTATTGGAGATGTGCCACAGCACACAAATTGGTGGG

ACAAGGATGGTGGATATTCTTAGGCAAAATCCAACTGAGGAACAAGCTGTGGATATATGC

AAAGCAGCAATGGGTTTGAGGATTAGTTCATCCTTTAGCTTTGGAGGATTCACCTTCAAA

AGAACAAGTGGTTCATCCGTTAGAAAGGAAGAGGAAGTGCTTACAGGTAACCTCCAAACA

TTGAAAATAAGAGTACATGAGGGGTATGAGGAGTTCACAATGGTTGGGCGAAGAGCAACA

GCCATTCTAAGGAAAGCAACTAGAAGGCTGATTCAGTTGATAGTAAGTGGAAGAGACGAA

CAATCAATCGCTGAAGCAATCATCGTAGCCATGGTGTTCTCACAGGAGGATTGCATGATA

AAGGCAGTCCGAGGCGATCTAAATTTTGTGAACAGAGCAAACCAAAGATTGAACCCCATG

CATCAACTCCTGAGACACTTCCAAAAAGATGCAAAAGTGCTGTTTCAAAATTGGGGGATT

GAACCTATTGATAATGTCATGGGGATGATTGGAATATTACCTGACATGACTCCAAGCACA

GAGATGTCACTAAGAGGAGTAAGAGTTAGTAAAATGGGAGTAGATGAATATTCCAGCACT

GAGAGAGTGGTTGTAAGCATTGACCGTTTCTTGCGGGTTCGAGATCAGCAGGGGAACGTA

CTCCTATCTCCCGAAGAGGTCAGCGAAACACTGGGAACAGAAAAATTAACAATAACATAT

TCATCATCAATGATGTGGGAAATCAATGGTCCTGAGTCAGTGCTGGTCAACACCTATCAA

TGGATCATCAGAAATTGGGAGATTGTGAAGATTCAATGGTCTCAAGACCCCACGATGCTG

TACAATAAGGTGGAGTTTGAACCGTTCCAATCCTTGGTACCTAAAGCTGCCAGAGGCCAA

TACAGTGGATTTGTGAGAACACTGTTCCAACAAATGCGTGACGTATTGGGGACATTTGAT

ACTATTCAGATAATAAAGCTGTTACCGTTTGCAGCAGCCCCACCGGAGCATAGCAGAATG

CAATTTTCTTCCCTGACCGTGAATGTAAGAGGCTCGGGAATGAGAATACTCGTAAGGGGT

AACTCCCCTGTGTTCAACTACAATAAGGCAACCAAAAGGCTTGCAGTCCTTGGAAAGGAC

GCAGGTGCATTAACAGAGGATCCAGATGAGGGGACAACAGGAGTGGAATCCGCAGTACTG

AGGGGGTTCCTAATTCTGGGCAAGGAGGACAGAAGATATGGACCAGCACTAAGCATCAAT

GAACTGAGCAATCTTGCGAAAGGGGAGAAAGCCAATGTGCTGATAGGGCAAGGAGACGTG

GTGCTGGTAATGAAACGGAAACGGGACTCTAGCATACTTACTGACAGCCAGACAGCGACC

AAAAGAATTCGGATGGTCATCAATTAGTATCGAG--------------------------

----------------------

>A_chicken_Washington_3490-18_2015_EPI590690

---------------------------ATGGAGAGAATAAAAGAACTAAGAGATCTAATG

TCTCAATCCCGCACTCGCGAGATACTAACAAAAACCACTGTGGACCATATGGCCATAATC

AAGAAATACACATCAGGAAGACAAGAGAAGAATCCTGCTCTCAGAATGAAATGGATGATG

GCAATGAGATATCCAATCACAGCAGACAAGAGAATAATGGAAATGATTCCTGAAAGAAAT

GAACAAGGCCAGACGCTTTGGAGCAAGACAAATGATGCTGGATCAGGCAGAGTGATGGTG

TCTCCCCTAGCTGTAACTTGGTGGAATAGAAATGGACCGACAGCAAGTACAGTCCATTAT

CCAAAGGTCTACAAAACATACTTTGAGAAGGTTGAAAGGTTAAAGCATGGAACCTTCGGT

CCCGTTCACTTCCGAAACCAAATTAAAATACGCCGCCGAGTTGACATAAACCCAGGCCAC

GCAGATCTCAGTGCCAAAGAAGCACAAGATGTCATCATGGAGGTTGTTTTCCCAAATGAA

GTGGGAGCTAGAATATTGACATCAGAGTCACAATTGACAATAACGAAAGAGAAAAAAGAA

GAACTCCAGGATTGCAAGATTGCTCCTTTAATGGTGGCATACATGTTGGAAAGAGAACTG

GTCCGAAAAACCAGATTCCTACCAGTAGCAGGTGGGACAAGCAGTGTGTACATTGAGGTA

CTGCACTTGACTCAAGGGACCTGCTGGGAACAGATGTACACTCCAGGCGGAAAAGTGAGG

AATGACGATGTTGACCAGAGTTTGATCATCGCGGCCAGAAACATTGTTAGGAGAGCAACG

GTATCAGCGGATCCACTGGCATCATTATTGGAGATGTGCCACAGCACACAAATTGGTGGG

ACAAGGATGGTGGATATTCTTAGGCAAAATCCAACTGAGGAACAAGCTGTGGATATATGC

AAAGCAGCAATGGGTTTGAGGATTAGTTCATCCTTTAGCTTCGGAGGATTCACCTTCAAA

AGAACAAGTGGTTCATCCGTTAGAAAGGAAGAGGAAGTGCTTACAGGTAACCTCCAAACA

TTGAAAATAAGAGTACATGAGGGGTATGAGGAGTTCACAATGGTTGGGCGAAGAGCAACA

GCCATTCTAAGGAAAGCAACTAGAAGGCTGATTCAGTTGATAGTAAGTGGAAGAGACGAA

CAATCAATCGCTGAAGCAATCATCGTAGCCATGGTGTTCTCACAGGAGGATTGCATGATA

AAGGCAGTCCGAGGCGATCTAAATTTTGTGAACAGAGCAAACCAAAGATTGAACCCCATG

CATCAACTCCTGAGACACTTCCAAAAAGATGCAAAAGTGCTGTTTCAAAATTGGGGGATT

GAACCTATTGATAATGTCATGGGGATGATTGGAATATTACCTGACATGACTCCAAGCACA

GAGATGTCACTAAGAGGAGTAAGAGTTAGTAAAATGGGAGTAGATGAATATTCCAGCACT

GAGAGAGTGGTTGTAAGCATTGACCGTTTCTTGCGGGTTCGAGATCAGCAGGGGAACGTA

CTCCTATCTCCCGAAGAGGTCAGCGAAACACTGGGAACAGAAAAATTAACAATAACATAT

TCATCATCAATGATGTGGGAAATCAATGGTCCTGAGTCAGTGCTGGTCAACACCTATCAA

TGGATCATCAGAAATTGGGAGATTGTGAAGATTCAATGGTCTCAAGACCCCACGATGCTG

TACAATAAGGTGGAGTTTGAACCGTTCCAATCCTTGGTACCTAAAGCTGCCAGAGGCCAA

TACAGTGGATTTGTGAGAACACTGTTCCAACAAATGCGTGACGTATTGGGGACATTTGAT

ACTATTCAGATAATAAAGCTGTTACCGTTTGCAGCAGCCCCACCGGAGCATAGCAGAATG

CAATTTTCTTCCCTGACCGTGAATGTAAGAGGCTCGGGAATGAGAATACTCGTAAGGGGT

AACTCCCCTGTGTTCAACTACAATAAGGCAACCAAAAGGCTTGCAGTCCTTGGAAAGGAC

GCAGGTGCATTAACAGAGGATCCAGATGAGGGGACAACAGGAGTGGAATCCGCAGTACTG

AGGGGGTTCCTAATTCTGGGCAAGGAGGACAGAAGATATGGACCAGCACTAAGCATCAAT

GAACTGAGCAATCTTGCGAAAGGGGAGAAAGCCAATGTGCTGATAGGGCAAGGAGACGTG

GTGCTGGTAATGAAACGGAAACGGGACTCTAGCATACTTACTGACAGCCAGACAGCGACC

AAAAGAATTCGGATGGTCATCAATTAG---------------------------------

----------------------

>A_Perigrine_falcon_Netherlands_1800327_1327125

------------TCAAATATATTCAATATGGAGAGAATAAAAGAATTAAGAGATTTGATG

TCGCAGTCTCGCACTCGCGAGATACTAACAAAAACCACTGTGGACCATATGGCCATAATC

AAGAAATATACGTCAGGAAGACAGGAGAAGAATCCTGCTCTTAGGATGAAATGGATGATG

GCAATGAAATATCCGATTACAGCAGACAAAAGGATAATGGAGATGATCCCTGAAAGAAAT

GAGCAAGGTCAGACTCTTTGGAGCAATACGAATGATGCTGGATCGGATAGAGTTATGGTG

TCACCTCTGGCTGTGACGTGGTGGAATAGAAATGGGCCAACGACGAGTACAGTCCATTAT

CCAAAGGTCTATAAAACCTATTTTGAAAAGGTCGAAAGGTTAAAGCATGGAACCTTCGGT

CCCGTCCACTTTCGAAATCAGGTCAAAATACGCCGCAGGGTTGACATAAACCCAGGCCAT

GCAGATCTCAGTGCCAAAGAAGCACAGGATGTCATCATGGAGGTCGTTTTCCCAAATGAA

GTAGGAGCTAGGATATTGACATCAGAGTCACAGTTAACAATAACAAAGGAAAAGAAGGAG

GAGCTTCAGGACTGTAAGATTGCTCCTTTGATGGTGGCATACATGTTGGAAAGAGAATTG

GTTCGCAAAACCAGATTTCTACCAGTAGCTGGTGGGACAAGCAGCGTGTACATTGAAGTG

TTACACTTGACTCAAGGGACCTGCTGGGAACAAATGTATACGCCGGGAGGGGATGTGAGA

AATGATGATGTTGATCAGAGTTTAATTATTGCTGCTCGAAATATTGTTAGGAGGGCAGTA

GTATCAGCAGACCCATTGGCTTCGCTCTTGGAGATGTGCCATAGCACGCAAATTGGCGGG

ATAAGGATGGTAGACATCCTTAGACAAAACCCAACAGAAGAGCAAGCTGTAGACATATGC

AAAGCTGCAATGGGTCTAAGGATCAGTTCATCCTTCAGCTTTGGAGGTTTCACTTTCAAG

AGGACGAGTGGATCATCTGTCAAAAGAGAAGAAGAAGTGCTCACAGGCAACCTCCAAACA

TTGAAAATAAGAGTGCATGAAGGATATGAAGAATTCACAATGGTTGGGCGAAGAGCAACA

GCTATTCTAAGGAAAGCAACCAGAAGGCTGATCCAACTGATAGTGAGTGGGAAAGACGAG

CAGTCAATTGCCGAGGCGATCATAGTGGCAATGGTGTTCTCACAAGAAGACTGTATGATA

AAAGCAGTACGAGGTGATCTGAATTTTGTCAACAGAGCAAACCAGCGGTTAAATCCTATG

CATCAGCTATTGAGGCATTTCCAGAAGGATGCAAAGGTGTTGTTTCAAAACTGGGGAATT

GAGCCCATCGACAATGTCATGGGGATGATCGGAATACTACCTGACATGACCCCTAGCACC

GAGATGTCACTAAGAGGAGTGAGAGTCAGTAAAATGGGAGTGGATGAATACTCCAGTACT

GAGCGGGTGGTTGTGAGCATTGATCGCTTCTTGAGGGTCCGAGACCAGAGGGGAAACGTG

CTCTTATCTCCAGAAGAAGTTAGTGAAACACAGGGAACAGAAAAACTGACAATAACATAT

TCGTCATCCATGATGTGGGAAATCAACGGCCCGGAATCAGTGTTAGTGAACACATATCAA

TGGATCATTAGAAATTGGGAAACTGTGAAAATTCAGTGGTCCCAAGACCCCACAATGCTA

TACAATAAGATGGAGTTTGAGCCCTTTCAATCCTTGGTGCCTAAGGCTGCCAGAGGTCAG

TACAGTGGATTTGTGAGAACGCTATTCCAACAGATGCGTGATGTACTGGGGACATTTGAC

ACCGTCCAAATAATAAAGCTGCTACCATTTGCAGCAGCCCCACCAGAACAGAGTAGAATG

CAGTTCTCTTCTCTAACCGTGAACGTGAGGGGTTCAGGAATGAGAATACTTGTGAGAGGC

AACTCCCCTGTGTTCAACTATAACAAGGCAACCAAAAGGCTTACAGTCCTAGGAAAGGAT

GCAGGTGCATTGACAGAAGATCCAGATGAGGGAACAGCAGGGGTGGAATCTGCGGTATTA

AGAGGATTTCTAATCCTAGGTAAAGAAGACAAAAGATATGGACCAGCATTGAGCATCAAC

GAATTGAGCAATCTTGCGAAAGGGGAGAAGGCTAATGTGTTGGTAGGGCAAGGAGACGTG

GTGTTGGTAATGAAACGGAAACGGGACTCTAGCATACTTACTGACAGCCAGACAGCGACC

AAAAGAATTCGGATGGCCATCAATTAGTGTCGAATTGTTTAAAAACGA------------

----------------------

>A_Fujian-Sanyuan_21099_2017_x_PR8_CNI_1369970

----AAAGCAGGTCAATTATATTCAATATGGAAAGAATAAAAGAACTACGAAATCTAATG

TCGCAGTCTCGCACCCGCGAGATACTCACAAAAACCACCGTGGACCATATGGCCATAATC

AAGAAGTACACATCAGGAAGACAGGAGAAGAACCCAGCACTTAGGATGAAATGGATGATG

GCAATGAAATATCCAATTACAGCAGACAAGAGGATAACGGAAATGATTCCTGAGAGAAAT

GAGCAAGGACAAACTTTATGGAGTAAAATGAATGATGCCGGATCAGACCGAGTGATGGTA

TCACCTCTGGCTGTGACATGGTGGAATAGGAATGGACCAATAACAAATACAGTTCATTAT

CCAAAAATCTACAAAACTTATTTTGAAAGAGTCGAAAGGCTAAAGCATGGAACCTTTGGC

CCTGTCCATTTTAGAAACCAAGTCAAAATACGTCGGAGAGTTGACATAAATCCTGGTCAT

GCAGATCTCAGTGCCAAGGAGGCACAGGATGTAATCATGGAAGTTGTTTTCCCTAACGAA

GTGGGAGCCAGGATACTAACATCGGAATCGCAACTAACGATAACCAAAGAGAAGAAAGAA

GAACTCCAGGATTGCAAAATTTCTCCTTTGATGGTTGCATACATGTTGGAGAGAGAACTG

GTCCGCAAAACGAGATTCCTCCCAGTGGCTGGTGGAACAAGCAGTGTGTACATTGAAGTG

TTGCATTTGACTCAAGGAACATGCTGGGAACAGATGTATACTCCAGGAGGGGAAGTGAGG

AATGATGATGTTGATCAAAGCTTGATTATTGCTGCTAGGAACATAGTGAGAAGAGCTGCA

GTATCAGCAGATCCACTAGCATCTTTATTGGAGATGTGCCACAGCACACAGATTGGTGGA

ATTAGGATGGTAGACATCCTTAGGCAGAACCCAACAGAAGAGCAAGCCGTGGATATATGC

AAGGCTGCAATGGGACTGAGAATTAGCTCATCCTTCAGTTTTGGTGGATTCACATTTAAG

AGAACAAGCGGATCATCAGTCAAGAGAGAGGAAGAGGTGCTTACGGGCAATCTTCAAACA

TTGAAGATAAGAGTGCATGAGGGATATGAAGAGTTCACAATGGTTGGGAGAAGAGCAACA

GCCATACTCAGAAAAGCAACCAGGAGATTGATTCAGCTGATAGTGAGTGGGAGAGACGAA

CAGTCGATTGCCGAAGCAATAATTGTGGCCATGGTATTTTCACAAGAGGATTGTATGATA

AAAGCAGTCAGAGGTGATCTGAATTTCGTCAATAGGGCGAATCAACGATTGAATCCTATG

CATCAACTTTTAAGACATTTTCAGAAGGATGCGAAAGTGCTTTTTCAAAATTGGGGAGTT

GAACCTATCGACAATGTGATGGGAATGATTGGGATATTGCCCGACATGACTCCAAGCATC

GAGATGTCAATGAGAGGAGTGAGAATCAGCAAAATGGGTGTAGATGAGTACTCCAGCACG

GAGAGGGTAGTGGTGAGCATTGACCGTTTTTTGAGAATCCGGGACCAACGAGGAAATGTA

CTACTGTCTCCCGAGGAGGTCAGTGAAACACAGGGAACAGAGAAACTGACAATAACTTAC

TCATCGTCAATGATGTGGGAGATTAATGGTCCTGAATCAGTGTTGGTCAATACCTATCAA

TGGATCATCAGAAACTGGGAAACTGTTAAAATTCAGTGGTCCCAGAACCCTACAATGCTA

TACAATAAAATGGAATTTGAACCATTTCAGTCTTTAGTACCTAAGGCCATTAGAGGCCAA

TACAGTGGGTTTGTAAGAACTCTGTTCCAACAAATGAGGGATGTGCTTGGGACATTTGAT

ACCGCACAGATAATAAAACTTCTTCCCTTCGCAGCCGCTCCACCAAAGCAAAGTAGAATG

CAGTTCTCCTCATTTACTGTGAATGTGAGGGGATCAGGAATGAGAATACTTGTAAGGGGC

AATTCTCCTGTATTCAACTATAACAAGGCCACGAAGAGACTCACAGTTCTCGGAAAGGAT

GCTGGCACTTTAACTGAAGACCCAGATGAAGGCACAGCTGGAGTGGAGTCCGCTGTTCTG

AGGGGATTCCTCATTCTGGGCAAAGAAGACAAGAGATATGGGCCAGCACTAAGCATCAAT

GAACTGAGCAACCTTGCGAAAGGAGAGAAGGCTAATGTGCTAATTGGGCAAGGAGACGTG

GTGTTGGTAATGAAACGGAAACGGGACTCTAGCATACTTACTGACAGCCAGACAGCGACC

AAAAGAATTCGGATGGCCATCAATTAGTGTCGAATAGTTTAAAAACGACCTTGTTTCTAC

T---------------------

>A_duck_Sichuan_NCXJ16_2014_EPI590850

---------------------------ATGGAACGAATAAAAGAATTAAGAGATCTAATG

TCACAGTCCCGCACCCGCGAGATACTAACAAAAACCACTGTGGACCATATGGCCATAATC

AAGAAATACACATCAGGAAGACAAGAGAAGAATCCTGCCCTCAGGATGAAATGGATGATG

GCAATGAAGTATCCGATTACAGCGGACAAGAGAATAATAGATATGATTCCTGAAAGGAAT

GAACAAGGACAGACACTCTGGAGCAAGACAAATGATGCTGGGTCGGACAGGGTGATGGTG

TCCCCCCTAGCTGTAACTTGGTGGAATAGGAATGGGCCAACGACAAATACAGTTCATTAT

CCAAAAGTTTACAAAACATACTTCGAGAAAGTTGAAAGGTTAAAGCATGGAACATTCGGT

CCTGTCCATTTCCGAAACCAAGTTAAAATACGCCGCCGAGTTGACACAAACCCTGGCCAT

GCAGATCTCAGTGCTAAAGAAGCACAAGATGTCATCATGGAAGTTGTTTTCCCAAATGAG

GTTGGAGCTAGAATATTGACTTCGGAGTCACAATTGACAATAACAAAGGAGAAGAAAGAA

GAGCTTCAAGATTGTAAGATTGCTCCCTTAATGGTCGCATACATGTTGGAGAGGGAACTG

GTCCGCAAAACCAGATTCCTACCTGTAGCAGGCGGTACAAGCAGTGTATACATTGAGGTA

CTGCATTTGACCCAAGGAACCTGCTGGGAGCAGATGTACACTCCAGGCGGAGAAGTAAGA

AATGACGACGTTGATCAGAGTTTGATCATTGCTGCTAGAAACATTGTTAGGAGAGCAACG

GTGTCAGCGGATCCATTGGCATCACTGTTGGAGATGTGTCACAGTACACAAATTGGTGGA

ATAAGGATGGTGGACATCCTTAGGCAAAATCCAACTGAGGAACAAGCTGTGGATATATGC

AGAGCAGCAATGGGTCTGAGGATCAGCTCATCCTTTAGTTTTGGAGGTTTCACTTTCAAA

AGAACAAGTGGGTCATCCGTCATGAAAGAAGAGGAAGTGCTAACAGGCAACCTTCAAACA

TTAAAAATAAAAGTACATGAGGGGTATGAAGAATTCACAATGGTCGGGCAGAGAGCAACA

GCTATCCTGAGGAAGGCAACTAGGAGGCTGATTCAGTTGATAGTAAGCGGGAGGAACGAA

CAATCAATCGCTGAGGCAATCATTGTGGCGATGGTGTTCTCACAGGAGGATTGCATGATA

AAGGCAGTCCGAGGCGATCTAAATTTCGTAAACAGAGCAAACCAAAGATTAAATCCCATG

CATCAACTCCTGAGGCATTTTCAAAAGGACGCAAAGGTGCTATTTCAGAATTGGGGACTT

GAACCCATTGATAATGTCATGGGGATGATCGGGATACTACCTGACATGACTCCCAGCACA

GAACTGTCATTGAGAGGAGTGCGAATTAGTAAAATGGGAGTGGATGAATATTCCAGCACT

GAAAGAGTAGTTGTAAGCATTGACCGCTTTTTAAGGGTTCGAGATCAGCGGGGAAATGTA

CTCTTATCTCCCGAAGAGGTCAGCGAAACTCAGGGAACAGAGAAGTTGACAATAACATAT

TCATCATCAATGATGTGGGAAATCAACGGTCCTGAATCAGTGCTTGTCAACACCTATCAA

TGGATCATCAGAAATTGGGAAACTGTGAAGATTCAATGGTCTCAGGATCCCACGATGCTG

TACAATAAGATGGAGTTCGAACCATTCCAATCCTTGGTACCTAAAGCTACCAGAGGTCAA

TACAGTGGATTTGTGAGAACATTATTTCAACAAATGCGTGATGTACTAGGGACATTTGAT

ACAGCCCAGATAATAAAGCTGCTACCATTTGCAGCAGCACCACCTGAGCAGAGCAGAATG

CAGTTTTCTTCTCTAACTGTGAATGTGAGGGGGTCAGGAATGAGAATACTCATAAGGGGC

AATTCCCCTGTGTTCAACTACAATAAGGCGACCAAAAGGCTTACTGTTCTTGGAAAGGAC

GCAGGTGCATTAACTGAGGATCCGGACGAGGGGGCAGCCGGAGTGGAATCTGCTGTGCTG

AGGGGATTCCTAATTCTAGGCAAGGAGGACAAAAGATATGGACCAGCATTGAGCATCAAT

GAACTGAGCAATCTTGCAAAAGGGGAGAAAGCTAATGTGCTGATAGGGCAAGGAGACGTG

GTGTTGGTAATGAAACGAAAACGGGACTCTAGCATACTTACTGACAGCCAGACAGCGACC

AAAAGAATTCGAATGGCCATCAATTAG---------------------------------

----------------------

>A_Sichuan_26221_2014_EPI533585

---------------------------ATGGAACGAATAAAAGAATTAAGAGATCTAATG

TCACAGTCCCGCACCCGCGAGATACTAACAAAAACCACTGTGGACCATATGGCCATAATC

AAGAAATACACATCAGGAAGACAAGAGAAGAATCCTGCCCTCAGGATGAAATGGATGATG

GCAATGAAGTATCCGATTACAGCGGACAAGAGAATAATAGATATGATTCCTGAAAGGAAT

GAACAAGGACAGACACTCTGGAGCAAGACAAATGATGCTGGGTCGGACAGGGTGATGGTG

TCCCCCCTAGCTGTAACTTGGTGGAATAGGAATGGGCCAACGACAAATACAGTTCATTAT

CCAAAAGTTTACAAAACATACTTCGAGAAAGTTGAAAGGTTAAAGCATGGAACATTCGGT

CCTGTCCATTTCCGAAACCAAGTTAAAATACGCCGCCGAGTTGACACAAACCCTGGCCAT

GCAGATCTCAGTGCTAAAGAAGCACAAGATGTCATCATGGAAGTTGTTTTCCCAAATGAG

GTTGGAGCTAGAATATTGACTTCGGAGTCACAATTGACAATAACAAAGGAGAAGAAAGAA

GAGCTTCAAGATTGTAAGATTGCTCCCTTAATGGTCGCATACATGTTGGAGAGGGAACTG

GTCCGCAAAACCAGATTCCTACCTGTAGCAGGCGGTACAAGCAGTGTATACATTGAGGTA

CTGCATTTGACCCAAGGAACCTGCTGGGAGCAGATGTACACTCCAGGCGGAGAAGTAAGA

AATGACGACGTTGATCAGAGTTTGATCATTGCTGCTAGAAACATTGTTAGGAGAGCAACG

GTGTCAGCGGATCCATTGGCATCACTGTTGGAGATGTGTCACAGTACACAAATTGGTGGA

ATAAGGATGGTGGACATCCTTAGGCAAAATCCAACTGAGGAACAAGCTGTGGATATATGC

AGAGCAGCAATGGGTCTGAGGATCAGCTCATCCTTTAGTTTTGGAGGTTTCACTTTCAAA

AGAACAAGTGGGTCATCCGTCATGAAAGAAGAGGAAGTGCTAACAGGCAACCTTCAAACA

TTAAAAATAAAAGTACATGAGGGGTATGAAGAATTCACAATGGTCGGGCAGAGAGCAACA

GCTATCCTGAGGAAGGCAACTAGGAGGCTGATTCAGTTGATAGTAAGCGGGAGGAACGAA

CAATCAATCGCTGAGGCAATCATTGTGGCGATGGTGTTCTCACAGGAGGATTGCATGATA

AAGGCAGTCCGAGGCGATCTAAATTTCGTAAACAGAGCAAACCAAAGATTAAATCCCATG

CATCAACTCCTGAGGCATTTTCAAAAGGACGCAAAGGTGCTATTTCAGAATTGGGGACTT

GAACCCATTGATAATGTCATGGGGATGATCGGGATACTACCTGACATGACTCCCAGCACA

GAACTGTCATTGAGAGGAGTGCGAATTAGTAAAATGGGAGTGGATGAATATTCCAGCACT

GAAAGAGTAGTTGTAAGCATTGACCGCTTTTTAAGGGTTCGGGATCAGCGGGGAAATGTA

CTCTTATCTCCCGAAGAGGTCAGCGAAACTCAGGGAACAGAGAAGTTGACAATAACATAT

TCATCATCAATGATGTGGGAAATCAACGGTCCTGAATCAGTGCTTGTCAACACCTATCAA

TGGATCATCAGAAATTGGGAAACTGTGAAGATTCAATGGTCTCAGGATCCCACGATGCTG

TACAATAAGATGGAGTTCGAACCATTCCAATCCTTGGTACCTAAAGCTACCAGAGGTCAA

TACAGTGGATTTGTGAGAACATTATTTCAACAAATGCGTGATGTACTAGGGACATTTGAT

ACAGCCCAGATAATAAAGCTGCTACCATTTGCAGCAGCACCACCTGAGCAGAGCAGAATG

CAGTTTTCTTCTCTAACTGTGAATGTGAGGGGGTCAGGAATGAGAATACTCATAAGGGGC

AATTCCCCTGTGTTCAACTACAATAAGGCGACCAAAAGGCTTACTGTTCTTGGAAAGGAC

GCAGGTGCATTAACTGAGGATCCGGACGAGGGGGCAGCCGGAGTGGAATCTGCTGTGCTG

AGGGGATTCCTAATTCTAGGCAAGGAGAACAAAAGATATGGACCAGCATTGAGCATCAAT

GAACTGAGCAATCTTGCAAAAGGGGAGAAAGCTAATGTGCTGATAGGGCAAGGAGACGTG

GTGTTGGTAATGAAACGAAAACGGGACTCTAGCATACTTACTGACAGCCAGACAGCGACC

AAAAGAATTCGAATGGCCATCAATTAG---------------------------------

----------------------
