# Supplementary material for: Highly pathogenic avian influenza virus of the A/H5N8 subtype, clade 2.3.4.4b, caused outbreaks in Kazakhstan in 2020
Source: PeerJ. 2022 Mar 2;10:e13038. doi: 10.7717/peerj.13038 (PMC8898005; doi:10.7717/peerj.13038)
Supplement: Figure S5 [file peerj-10-13038-s006.docx]

**Fig.S5** Alignment of the nucleotide sequences for the PA segment used in this study

>A_goose_Kazakhstan_4-190-20-B-H5N8-1_2020_EPI1927650

AGCAAAAGCAGGTACTGATCCAAAATGGAAGACTTTGTGCGACAATGCTTCAATCCAATG

ATTGTCGAGCTTGCGGAAAAGGCAATGAAAGAATATGGGGAAGATCCTAAGATCGAAACA

AACAAGTTTGCCGCAATATGTACACACTTAGAAGTCTGCTTCATGTATTCAGATTTCCAT

TTCATTGATGAACGAGGCGAATCAATAATCGTAGAATCTGGCGACCCGAATGCATTATTG

AAGCACCGATTTGAGATAATTGAAGGGAGAGACCGAACAATGGCCTGGACAGTGGTGAAT

AGTATATGCAACACTACAGGAGTCGAAAAGCCCAAGTTCCTTCCTGATTTGTATGACTAC

AGAGAAAACCGATTCATTGAAATTGGAGTAACGCGAAGGGAAGTTCACATATACTATCTT

GAAAAAGCCAACAAGATAAAATCAGAGAAAACACACATTCACATATTCTCATTCACTGGG

GAGGAAATGGCCACCAAGGCAGATTACACTCTTGATGAAGAGAGCAGAGCAAGAATAAAA

ACCAGGCTATTCACTATAAGACAAGAGATGGCCAATAGGGGTCTATGGGATTCCTTTCGT

CAGTCCGAGAGAGGCGAAGAGACAATTGAAGAAAGATTTGAAATCACAGGAACCATGCGC

AGGCTTGCTGACCAGAGTCTCCCACCGAACTTCACCAGCCTTGAAAACTTTAGAGCCTAT

GTGGATGGATTTGAACCGAACGGCTGCATTGAGGGCAAGCTTTCTCAAATGTCAAAAGAA

GTGAATGCCAGAATTGAGCCATTTCTGAAGACAACACCACGCCCTCTCAGATTACCTGAT

GGACCTCCCTGTTCCCAGCGGTCGAAGTTCTTGCTAATGGATGCCCTTAAATTGAGCATT

GAAGACCCGAGCCATGAGGGGGAGGGTATACCGCTGTACGATGCAACCAAATGCATGAAG

ACATTTTTTGGCTGGAAGGAGCCCAACATCGTGAAACCACATGAAAAGGGCATAAACCCT

AATTACCTCCTGGCTTGGAAGCAGGTGCTATCAGAACTCCAAGATATTGAAAACGAGGAG

AAAATCCCAAAAACAAAGAACATGAAGAAGACGAGCCAACTGAAGTGGGCACTTGGTGAA

AACATGGCACCAGAGAAAGTGGACTTTGAGGACTGTAAAGATGTTAGCGATCTAAAACAG

TACGACAGTGACGAACCAGAATCTAGATCACTAGCAAGTTGGATCCAGAGTGAATTTAAC

AAGGCTTGCGAACTGACAGATTCGAGTTGGATTGAACTTGATGAAATAGGGGAAGACGTT

GCTCCAATTGAACACATTGCGAGTATGAGAAGGAACTATTTCACAGCGGAAGTATCCCAT

TGCAGGGCTACAGAATACATAATGAAAGGAGTATACATAAACACAGCTTTATTGAATGCA

TCCTGTGCAGCCATGGATGACTTCCAACTGATTCCAATGATAAGCAAATGCAGAACCAAA

GAAGGGAGACGGAAGACAAATCTGTATGGCTTCATTATAAAAGGAAGATCCCATTTGAGG

AATGACACCGATGTGGTAAACTTTGTGAGCATGGAATTCTCTCTCACTGACCCAAGGCTG

GAGCCACACAAATGGGAAAAGTACTGTGTGCTTGAGGTAGGAGACATGCTCCTACGGACT

GCAATAGGCCAAGTGTCAAGGCCCATGTTCCTGTATGTGAGAACCAATGGGACTTCTAAG

ATCAAAATGAAATGGGGTATGGAGATGAGGCGATGCCTTCTTCAATCCCTTCAACAAATT

GAGAGCATGATTGAAGCCGAGTCTTCTGTCAAAGAAAAAGACATGACCAAGGAATTCTTT

GAAAACAGATCAGAAACATGGCCAATTGGAGAGTCACCCAAAGGAGTGGAGGAAGGCTCC

ATTGGGAAGGTGTGCAGAACATTGCTAGCAAAGTCTGTGTTCAACAGCCTATATGCATCT

CCACAACTCGAGGGGTTTTCAGCTGAATCAAGAAAGTTGCTTCTCATTGTTCAGGCACTT

AGGGACAACCTGGAACCTGGAACCTTCGATCTTGGGGGGCTATATGAAGCAATTGAGGAG

TGCCTGATTAACGATCCCTGGGTTTTGCTTAATGCGTCTTGGTTCAACTCCTTCCTCACA

CATGCACTGAAATAGTCGTGACAATGCTACTA---TTTGCTATCCATACTGTCCAAAAAA

GTACCTTGTTTCTACT

>A_chicken_Kazakhstan_220-B-2-H5N8-4_2020_EPI1927656

AGCAAAAGCAGGTACTGATCCAAAATGGAAGACTTTGTGCGACAATGCTTCAATCCAATG

ATTGTCGAGCTTGCGGAAAAGGCAATGAAAGAATATGGGGAAGATCCTAAGATCGAAACA

AACAAGTTTGCCGCAATATGTACACACTTAGAAGTCTGCTTCATGTATTCAGATTTCCAT

TTCATTGATGAACGAGGCGAATCAATAATCGTAGAATCTGGCGACCCGAATGCATTATTG

AAGCACCGATTTGAGATAATTGAAGGGAGAGACCGAACAATGGCCTGGACAGTGGTGAAT

AGTATATGCAACACTACAGGAGTCGAAAAGCCCAAGTTCCTTCCTGATTTGTATGACTAC

AGAGAAAACCGATTCATTGAAATTGGAGTAACGCGAAGGGAAGTTCACATATACTATCTT

GAAAAAGCCAACAAGATAAAATCAGAGAAAACACACATTCACATATTCTCATTCACTGGG

GAGGAAATGGCCACCAAGGCAGATTACACTCTTGATGAAGAGAGCAGAGCAAGAATAAAA

ACCAGGCTATTCACTATAAGACAAGAGATGGCCAATAGGGGTCTATGGGATTCCTTTCGT

CAGTCCGAGAGAGGCGAAGAGACAATTGAAGAAAGATTTGAAATCACAGGAACCATGCGC

AGGCTTGCTGACCAGAGTCTCCCACCGAACTTCACCAGCCTTGAAAACTTTAGAGCCTAT

GTGGATGGATTTGAACCGAACGGCTGCATTGAGGGCAAGCTTTCTCAAATGTCAAAAGAA

GTGAATGCCAGAATTGAGCCATTTCTGAAGACAACACCACGCCCTCTCAGATTACCTGAT

GGACCTCCCTGTTCCCAGCGGTCGAAGTTCTTGCTAATGGATGCCCTTAAATTGAGCATT

GAAGACCCGAGCCATGAGGGGGAGGGTATACCGCTGTACGATGCAACCAAATGCATGAAG

ACATTTTTTGGCTGGAAGGAGCCCAACATCGTGAAACCACATGAAAAGGGCATAAACCCT

AATTACCTCCTGGCTTGGAAGCAGGTGCTATCAGAACTCCAAGATATTGAAAACGAGGAG

AAAATCCCAAAAACAAAGAACATGAAGAAGACGAGCCAACTGAAGTGGGCACTTGGTGAA

AACATGGCACCAGAGAAAGTGGACTTTGAGGACTGTAAAGATGTTAGCGATCTAAAACAG

TACGACAGTGACGAACCAGAATCTAGATCACTAGCAAGTTGGATCCAGAGTGAATTTAAC

AAGGCTTGCGAACTGACAGATTCGAGTTGGATTGAACTTGATGAAATAGGGGAAGACGTT

GCTCCAATTGAACACATTGCGAGTATGAGAAGGAACTATTTCACAGCGGAAGTATCCCAT

TGCAGGGCTACAGAATACATAATGAAAGGAGTATACATAAACACAGCTTTATTGAATGCA

TCCTGTGCAGCCATGGATGACTTCCAACTGATTCCAATGATAAGCAAATGCAGAACCAAA

GAAGGGAGACGGAAGACAAATCTGTATGGCTTCATTATAAAAGGAAGATCCCATTTGAGG

AATGACACCGATGTGGTAAACTTTGTGAGCATGGAATTCTCTCTCACTGACCCAAGGCTG

GAGCCACACAAATGGGAAAAGTACTGTGTGCTTGAGGTAGGAGACATGCTCCTACGGACT

GCAATAGGCCAAGTGTCAAGGCCCATGTTCCTGTATGTGAGAACCAATGGGACTTCTAAG

ATCAAAATGAAATGGGGTATGGAGATGAGGCGATGCCTTCTTCAATCCCTTCAACAAATT

GAGAGCATGATTGAAGCCGAGTCTTCTGTCAAAGAAAAAGACATGACCAAGGAATTCTTT

GAAAACAGATCAGAAACATGGCCAATTGGAGAGTCACCCAAAGGAGTGGAGGAAGGCTCC

ATTGGGAAGGTGTGCAGAACATTGCTAGCAAAGTCTGTGTTCAACAGCCTATATGCATCT

CCACAACTCGAGGGGTTTTCAGCTGAATCAAGAAAGTTGCTTCTCATTGTTCAGGCACTT

AGGGACAACCTGGAACCTGGAACCTTCGATCTTGGGGGGCTATATGAAGCAATTGAGGAG

TGCCTGATTAACGATCCCTGGGTTTTGCTTAATGCGTCTTGGTTCAACTCCTTCCTCACA

CATGCACTGAAATAGTCGTGACAATGCTACTA---TTTGCTATCCATACTGTCCAAAAAA

GTACCTTGTTTCTACT

>A_duck_Kazakhstan_12-20-B-Talg-11_2020_EPI1927662

AGCAAAAGCAGGTACTGATCCAAAATGGAAGACTTTGTGCGACAATGCTTCAATCCAATG

ATTGTCGAGCTTGCGGAAAAGGCAATGAAAGAATATGGGGAAGATCCTAAGATCGAAACA

AACAAGTTTGCCGCAATATGTACACACTTAGAAGTCTGCTTCATGTATTCGGATTTCCAT

TTCATTGATGAACGAGGCGAATCAATAATCGTGGAATCTGGCGACCCGAATGCATTATTG

AAGCACCGATTTGAGATAATTGAAGGGAGAGACCGAACAATGGCCTGGACAGTGGTGAAT

AGTATATGCAACACTACAGGAGTCGAAAAGCCCAAGTTCCTTCCTGATTTGTATGACTAC

AGAGAAAACCGATTCATTGAAATTGGAGTAACGCGAAGGGAAGTTCACATATACTATCTT

GAAAAAGCCAACAAGATAAAATCAGAGAAAACACACATTCACATATTCTCATTCACTGGG

GAGGAAATGGCCACCAAGGCAGATTACACTCTTGATGAAGAGAGCAGAGCAAGAATAAAA

ACCAGGCTATTCACTATAAGACAAGAGATGGCCAATAGGGGTCTATGGGATTCCTTTCGT

CAGTCCGAGAGAGGCGAAGAGACAATTGAAGAAAGATTTGAAATCACAGGAACCATGCGC

AGGCTTGCTGACCAAAGTCTCCCACCGAACTTCGCCAGCCTTGAAAACTTTAGAGCCTAT

GTGGATGGATTTGAACCGAACGGCTGCATTGAGGGCAAGCTTTCTCAAATGTCAAAAGAA

GTGAATGCCAGAATTGAGCCATTTCTGAAGAAAACACCACGCCCTCTCAGATTACCTGAT

GGACCTCCCTGTTCCCAGCGGTCGAAGTTCTTGCTAATGGATGCCCTTAAATTGAGCATT

GAAGACCCGAGCCATGAGGGGGAGGGTATACCGCTGTACGATGCAACCAAATGCATGAAG

ACATTTTTTGGCTGGAAGGAGCCCAACATCGTGAAACCACATGAAAAGGGCATAAACCCT

AATTACCTCCTGGCTTGGAAGCAGGTGCTATCAGAACTCCAAGATATTGAAAACGAGGAG

AAAATCCCAAAAACAAAGAACATGAAGAAGACGAGCCAACTGAAGTGGGCACTTGGTGAA

AACATGGCACCAGAGAAAGTGGACTTTGAGGACTGTAAAGATGTTAGCGATCTAAAACAG

TACGACAGTGGCGAACCAGAATCTAGATCACTAGCAAGTTGGATCCAGAGTGAATTTAAC

AAGGCTTGCGAACTGACAGATTCGAGTTGGATTGAACTTGATGAAATAGGGGAAGACGTT

GCTCCAATTGAACACATTGCGAGTATGAGGAGGAACTATTTCACAGCGGAAGTATCCCAT

TGCAGGGCTACAGAATACATAATGAAAGGAGTATACATAAACACAGCTTTATTGAATGCA

TCCTGTGCAGCCATGGATGACTTCCAACTGATTCCAATGATAAGCAAATGCAGAACCAAA

GAAGGGAGACGGAAGACAAATCTGTATGGCTTCATTATAAAAGGAAGATCCCATTTGAGG

AATGACACCGATGTGGTAAACTTTGTGAGCATGGAATTCTCTCTCACTGACCCAAGGCTG

GAGCCACACAAATGGGAAAAGTACTGTGTGCTTGAGGTAGGAGACATGCTCCTACGGACT

GCAATAGGCCAAGTGTCAAGGCCCATGTTCCTGTATGTGAGAACCAATGGGACTTCTAAG

ATCAAAATGAAATGGGGTATGGAGATGAGGCGATGCCTTCTTCAATCCCTTCAACAAATT

GAGAGCATGATTGAAGCCGAGTCTTCTGTCAAAGAAAAAGACATGACCAAGGAATTCTTT

GAAAACAGATCAGAAACATGGCCAATTGGAGAGTCACCCAAAGGAGTGGAGGAAGGCTCC

ATTGGGAAGGTGTGCAGAACATTGCTAGCAAAGTCTGTGTTCAACAGCCTATATGCATCT

CCACAACTCGAGGGGTTTTCAGCTGAATCAAGAAAGTTGCTTCTCATTGTTCAGGCACTT

AGGGACAACCTGGAACCTGGAACCTTCGATCTTGGGGGGCTATATGAAGCAATTGAGGAG

TGCCTGATTAACGATCCCTGGGTTTTGCTTAATGCGTCTTGGTTCAACTCCTTCCTCACA

CATGCACTGAAATAGTCGTGGCAATGCTACTA---TTTGCTATCCATACTGTCCAAAAAA

GTACCTTGTTTCTACT

>A_goose_Kazakhstan_7-20-B-Talg-12_2020_EPI1927668

AGCRAAAGCAGGTACTGATCCAAAATGGAAGACTTTGTGCGACAATGCTTCAATCCAATG

ATTGTCGAGCTTGCGGAAAAGGCAATGAAAGAATATGGGGAAGATCCTAAGATCGAAACA

AACAAGTTTGCCGCAATATGTACACACTTAGAAGTCTGCTTCATGTATTCGGATTTCCAT

TTCATTGATGAACGAGGCGAATCAATAATCGTGGAATCTGGCGACCCGAATGCATTATTG

AAGCACCGATTTGAGATAATTGAAGGGAGAGACCGAACAATGGCCTGGACAGTGGTGAAT

AGTATATGCAACACTACAGGAGTCGAAAAGCCCAAGTTCCTTCCTGATTTGTATGACTAC

AGAGAAAACCGATTCATTGAAATTGGAGTAACGCGAAGGGAAGTTCACATATACTATCTT

GAAAAAGCCAACAAGATAAAATCAGAGAAAACACACATTCACATATTCTCATTCACTGGG

GAGGAAATGGCCACCAAGGCAGATTACACTCTTGATGAAGAGAGCAGAGCAAGAATAAAA

ACCAGGCTATTCACTATAAGACAAGAGATGGCCAATAGGGGTCTATGGGATTCCTTTCGT

CAGTCCGAGAGAGGCGAAGAGACAATTGAAGAAAGATTTGAAATCACAGGAACCATGCGC

AGGCTTGCTGACCAAAGTCTCCCACCGAACTTCGCCAGCCTTGAAAACTTTAGAGCCTAT

GTGGATGGATTTGAACCGAACGGCTGCATTGAGGGCAAGCTTTCTCAAATGTCAAAAGAA

GTGAATGCCAGAATTGAGCCATTTCTGAAGACAACACCACGCCCTCTCAGATTACCTGAT

GGACCTCCCTGTTCCCAGCGGTCGAAGTTCTTGCTAATGGATGCCCTTAAATTGAGCATT

GAAGACCCGAGCCATGAGGGGGAGGGTATACCGCTGTACGATGCAACCAAATGCATGAAG

ACATTTTTTGGCTGGAAGGAGCCCAACATCGTGAAACCACATGAAAAGGGCATAAACCCT

AATTACCTCCTGGCTTGGAAGCAGGTGCTATCAGAACTCCAAGATATTGAAAACGAGGAG

AAAATCCCAAAAACAAAGAACATGAAGAAGACGAGCCAACTGAAGTGGGCACTTGGTGAA

AACATGGCACCAGAGAAAGTGGACTTTGAGGACTGTAAAGATGTTAGCGATCTAAAACAG

TACGACAGTGGCGAACCAGAATCTAGATCACTAGCAAGTTGGATCCAGAGTGAATTTAAC

AAGGCTTGCGAACTGACAGATTCGAGTTGGATTGAACTTGATGAAATAGGGGAAGACGTT

GCTCCAATTGAACACATTGCGAGTATGAGGAGGAACTATTTCACAGCGGAAGTATCCCAT

TGCAGGGCTACAGAATACATAATGAAAGGAGTATACATAAACACAGCTTTATTGAATGCA

TCCTGTGCAGCCATGGATGACTTCCAACTGATTCCAATGATAAGCAAATGCAGAACCAAA

GAAGGGAGACGGAAGACAAATCTGTATGGCTTCATTATAAAAGGAAGATCCCATTTGAGG

AATGACACCGATGTGGTAAACTTTGTGAGCATGGAATTCTCTCTCACTGACCCAAGGCTG

GAGCCACACAAATGGGAAAAGTACTGTGTGCTTGAGGTAGGAGACATGCTCCTACGGACT

GCAATAGGCCAAGTGTCAAGGCCCATGTTCCTGTATGTGAGAACCAATGGGACTTCTAAG

ATCAAAATGAAATGGGGTATGGAGATGAGGCGATGCCTTCTTCAATCCCTTCAACAAATT

GAGAGCATGATTGAAGCCGAGTCTTCTGTCAAAGAAAAAGACATGACCAAGGAATTCTTT

GAAAACAGATCAGAAACATGGCCAATTGGAGAGTCACCCAAAGGAGTGGAGGAAGGCTCC

ATTGGGAAGGTGTGCAGAACATTGCTAGCAAAGTCTGTGTTCAACAGCCTATATGCATCT

CCACAACTCGAGGGGTTTTCAGCTGAATCAAGAAAGTTGCTTCTCATTGTTCAGGCACTT

AGGGACAACCTGGAACCTGGAACCTTCGATCTTGGGGGGCTATATGAAGCAATTGAGGAG

TGCCTGATTAACGATCCCTGGGTTTTGCTTAATGCGTCTTGGTTCAACTCCTTCCTCACA

CATGCACTGAAATAGTCGTGGCAATGCTACTA---TTTGCTATCCATACTGTCCAAAAAA

GTACCTTGTTTCTACT

>A_swan_Kazakhstan_9-20-B-Talg-39_2020_EPI1927696

AGCAAAAGCAGGTACTGATCCAAAATGGAAGACTTTGTGCGACAATGCTTCAATCCAATG

ATTGTCGAGCTTGCGGAAAAGGCAATGAAAGAATATGGGGAAGATCCTAAGATCGAAACA

AACAAGTTTGCCGCAATATGTACACACTTAGAAGTCTGCTTCATGTATTCGGATTTCCAT

TTCATTGATGAACGAGGCGAATCAATAATCGTAGAATCTGGCGACCCGAATGCATTATTG

AAGCACCGATTTGAGATAATTGAAGGGAGAGACCGAACAATGGCCTGGACAGTGGTGAAT

AGTATATGCAACACTACAGGAGTCGAAAAGCCCAAGTTCCTTCCTGATTTGTATGACTAC

AGAGAAAACCGATTCATTGAAATTGGAGTAACGCGAAGGGAAGTTCACATATACTATCTT

GAAAAAGCCAACAAGATAAAATCAGAGAAAACACACATTCACATATTCTCATTCACTGGG

GAGGAAATGGCCACCAAGGCAGATTACACTCTTGATGAAGAGAGCAGAGCAAGAATAAAA

ACCAGGCTATTCACTATAAGACAAGAGATGGCCAATAGGGGTCTATGGGATTCCTTTCGT

CAGTCCGAGAGAGGCGAAGAGACAATTGAAGAAAGATTTGAAATCACAGGAACCATGCGC

AGGCTTGCTGACCAAAGTCTCCCACCGAACTTCGCCAGCCTTGAAAACTTTAGAGCCTAT

GTGGATGGATTTGAACCGAACGGCTGCATTGAGGGCAAGCTTTCTCAAATGTCAAAAGAA

GTGAATGCCAGAATTGAGCCATTTCTGAAGACAACACCACGCCCTCTCAGATTGCCTGAT

GGACCTCCCTGTTCCCAGCGGTCGAAGTTCTTGCTAATGGATGCCCTTAAATTGAGCATT

GAAGACCCGAGCCATGAGGGGGAGGGTATACCGCTGTACGATGCAACCAAATGCATGAAG

ACATTTTTTGGCTGGAAGGAGCCCAACATCGTGAAACCACATGAAAAGGGCATAAACCCT

AATTACCTCCTGGCTTGGAAGCAGGTGCTATCAGAACTCCAAGATATTGAAAACGAGGAG

AAAATCCCAAAAACAAAGAACATGAAGAAGACGAGCCAACTGAAGTGGGCACTTGGTGAA

AACATGGCACCAGAGAAAGTGGACTTTGAGGACTGTAAAGATGTTAGCGATCTAAAACAG

TACGACAGTGACGAACCAGAATCTAGATCACTAGCAAGTTGGATCCAGAGTGAATTTAAC

AAGGCTTGCGAACTGACAGATTCGAGTTGGATTGAACTTGATGAAATAGGGGAAGACGTT

GCTCCAATTGAACACATTGCGAGTATGAGGAGGAACTATTTCACAGCGGAAGTATCCCAT

TGCAGGGCTACAGAATACATAATGAAAGGAGTATACATAAACACAGCTTTATTGAATGCA

TCCTGTGCAGCCATGGATGACTTCCAACTGATTCCAATGATAAGCAAATGCAGAACCAAA

GAAGGGAGACGGAAGACAAATCTGTATGGCTTCATTATAAAAGGAAGATCCCATTTGAGG

AATGACACCGATGTGGTAAACTTTGTGAGCATGGAATTCTCTCTCACTGACCCAAGGCTG

GAGCCACACAAATGGGAAAAGTACTGTGTGCTTGAGGTAGGAGACATGCTCCTACGGACT

GCAATAGGCCAAGTGTCAAGGCCCATGTTCCTGTATGTGAGAACCAATGGGACTTCTAAG

ATCAAAATGAAATGGGGTATGGAGATGAGGCGATGCCTTCTTCAATCCCTTCAACAAATT

GAGAGCATGATTGAAGCCGAGTCTTCTGTCAAAGAAAAAGACATGACCAAGGAATTCTTT

GAAAACAGATCAGAAACATGGCCAATTGGAGAGTCACCCAAAGGAGTGGAGGAAGGCTCC

ATTGGGAAGGTGTGCAGAACATTGCTAGCAAAGTCTGTGTTCAATAGCCTATATGCATCT

CCACAACTCGAGGGGTTTTCAGCTGAATCAAGAAAGTTGCTTCTCATTGTTCAGGCACTT

AGGGACAACCTGGAACCTGGAACCTTCGATCTTGGGGGGCTATATGAAGCAATTGAGGAG

TGCCTGATTAACGATCCCTGGGTTTTGCTTAATGCGTCTTGGTTCAACTCCTTCCTCACA

CATGCACTGAAATAGTCGTGGCAATGCTACTA---TTTGCTATCCATACTGTCCAAAAAA

GTACCTTGTTTCTACT

>A_chicken_Kazakhstan_12-20-B-Talg-45_2020_EPI1927702

AGCAAAAGCAGGTACTGATCCAAAATGGAAGACTTTGTGCGACAATGCTTCAATCCAATG

ATTGTCGAGCTTGCGGAAAAGGCAATGAAAGAATATGGGGAAGATCCTAAGATCGAAACA

AACAAGTTTGCCGCAATATGTACACACTTAGAAGTCTGCTTCATGTATTCGGATTTCCAT

TTCATTGATGAACGAGGCGAATCAATAATCGTAGAATCTGGCGACCCGAATGCATTATTG

AAGCACCGATTTGAGATAATTGAAGGGAGAGACCGAACAATGGCCTGGACAGTGGTGAAT

AGTATATGCAACACTACAGGAGTCGAAAAGCCCAAGTTCCTTCCTGATTTGTATGACTAC

AGAGAAAACCGATTCATTGAAATTGGAGTAACGCGAAGGGAAGTTCACATATACTATCTT

GAAAAAGCCAACAAGATAAAATCAGAGAAAACACACATTCACATATTCTCATTCACTGGG

GAGGAAATGGCCACCAAGGCAGATTACACTCTTGATGAAGAGAGCAGAGCAAGAATAAAA

ACCAGGCTATTCACTATAAGACAAGAGATGGCCAATAGGGGTCTATGGGATTCCTTTCGT

CAGTCCGAGAGAGGCGAAGAGACAATTGAAGAAAGATTTGAAATCACAGGAACCATGCGC

AGGCTTGCTGACCAAAGTCTCCCACCGAACTTCGCCAGCCTTGAAAACTTTAGAGCCTAT

GTGGATGGATTTGAACCGAACGGCTGCATTGAGGGCAAGCTTTCTCAAATGTCAAAAGAA

GTGAATGCCAGAATTGAGCCATTTCTGAAGACAACACCACGCCCTCTCAGATTACCTGAT

GGACCTCCCTGTTCCCAGCGGTCGAAGTTCTTGCTAATGGATGCCCTTAAATTGAGCATT

GAAGACCCGAGCCATGAGGGGGAGGGTATACCGCTGTACGATGCAACCAAATGCATGAAG

ACATTTTTTGGCTGGAAGGAGCCCAACATCGTGAAACCACATGAAAAGGGCATAAACCCT

AATTACCTCCTGGCTTGGAAGCAGGTGCTATCAGAACTCCAAGATATTGAAAACGAGGAG

AAAATCCCAAAAACAAAGAACATGAAGAAGACGAGCCAACTGAAGTGGGCACTTGGTGAA

AACATGGCACCAGAGAAAGTGGACTTTGAGGACTGTAAAGATGTTAGCGATCTAAGACAG

TACGACAGTGACGAACCAGAATCTAGATCACTAGCAAGTTGGATCCAGAGTGAATTTAAC

AAGGCTTGCGAACTGACAGATTCGAGTTGGATTGAACTTGATGAAATAGGGGAAGACGTT

GCTCCGATTGAACACATTGCGAGTATGAGGAGGAACTATTTCACAGCGGAAGTATCCCAT

TGCAGGGCTACAGAATACATAATGAAAGGAGTATACATAAACACAGCTTTATTGAATGCA

TCCTGTGCAGCCATGGATGACTTCCAACTGATTCCAATGATAAGCAAATGCAGAACCAAA

GAAGGGAGACGGAAGACAAATCTGTATGGCTTCATTATAAAAGGAAGATCCCATTTGAGG

AATGACACCGATGTGGTAAACTTTGTGAGCATGGAATTCTCTCTCACTGACCCAAGGCTG

GAGCCACACAAATGGGAAAAGTACTGTGTGCTTGAGGTAGGAGACATGCTCCTACGGACT

GCAATAGGCCAAGTGTCAAGGCCCATGTTCCTGTATGTGAGAACCAATGGGACTTCTAAG

ATCAAAATGAAATGGGGTATGGAGATGAGGCGATGCCTTCTTCAATCCCTTCAACAAATT

GAGAGCATGATTGAAGCCGAGTCTTCTGTCAAAGAAAAAGACATGACCAAGGAATTCTTT

GAAAACAGATCAGAAACATGGCCAATTGGAGAGTCACCCAAAGGAGTGGAGGAAGGCTCC

ATTGGGAAGGTGTGCAGAACATTGCTAGCAAAGTCTGTGTTCAACAGCCTATATGCATCT

CCACAACTCGAGGGGTTTTCAGCTGAATCAAGAAAGTTGCTTCTCATTGTTCAGGCACTT

AGGGACAACCTGGAACCTGGGACCTTCGATCTTGGGGGGCTATATGAAGCAATTGAGGAG

TGCCTGATTAACGATCCCTGGGTTTTGCTTAATGCGTCTTGGTTCAACTCCTTCCTCACA

CATGCACTGAAATAGTCGTGGCAATGCTACTA---TTTGCTATCCATACTGTCCAAAAAA

GTACCTTGTTTCTACT

>A_crow_Kazakhstan_15-20-B-Talg-4_2020_EPI1927708

AGCRAAAGCAGGTACTGATCCAAAATGGAAGACTTTGTGCGACAATGCTTCAATCCAATG

ATTGTCGAGCTTGCGGAAAAGGCAATGAAAGAATATGGGGAAGATCCTAAGATCGAAACA

AACAAGTTTGCCGCAATATGTACACACTTAGAAGTCTGCTTCATGTATTCGGATTTCCAT

TTCATTGATGAACGAGGCGAATCAATAATCGTGGAATCTGGCGACCCGAATGCATTATTG

AAGCACCGATTTGAGATAATTGAAGGGAGAGACCGAACAATGGCCTGGACAGTGGTGAAT

AGTATATGCAACACTACAGGAGTCGAAAAGCCCAAGTTCCTTCCTGATTTGTATGACTAC

AGAGAAAACCGATTCATTGAAATTGGAGTAACGCGAAGGGAAGTTCACATATACTATCTT

GAAAAAGCCAACAAGATAAAATCAGAGAAAACACACATTCACATATTCTCATTCACTGGG

GAGGAAATGGCCACCAAGGCAGATTACACTCTTGATGAAGAGAGCAGAGCAAGAATAAAA

ACCAGGCTATTCACTATAAGACAAGAGATGGCCAATAGGGGTCTATGGGATTCCTTTCGT

CAGTCCGAGAGAGGCGAAGAGACAATTGAAGAAAGATTTGAAATCACAGGAACCATGCGC

AGGCTTGCTGACCAAAGTCTCCCACCGAACTTCGCCAGCCTTGAAAACTTTAGAGCCTAT

GTGGATGGATTTGAACCGAACGGCTGCATTGAGGGCAAGCTTTCTCAAATGTCAAAAGAA

GTGAATGCCAGAATTGAGCCATTTCTGAAGACAACACCACGCCCTCTCAGATTACCTGAT

GGACCTCCCTGTTCCCAGCGGTCGAAGTTCTTGCTAATGGATGCCCTTAAATTGAGCATT

GAAGACCCGAGCCATGAGGGGGAGGGTATACCGCTGTACGATGCAACCAAATGCATGAAG

ACATTTTTTGGCTGGAAGGAGCCCAACATCGTGAAACCACATGAAAAGGGCATAAACCCT

AATTACCTCCTGGCTTGGAAGCAGGTGCTATCAGAACTCCAAGATATTGAAAACGAGGAG

AAAATCCCAAAAACAAAGAACATGAAGAAGACGAGCCAACTGAAGTGGGCACTTGGTGAA

AACATGGCACCAGAGAAAGTGGACTTTGAGGACTGTAAAGATGTTAGCGATCTAAAACAG

TACGACAGTGGCGAACCAGAATCTAGATCACTAGCAAGTTGGATCCAGAGTGAATTTAAC

AAGGCTTGCGAACTGACAGATTCGAGTTGGATTGAACTTGATGAAATAGGGGAAGACGTT

GCTCCAATTGAACACATTGCGAGTATGAGGAGGAACTATTTCACAGCGGAAGTATCCCAT

TGCAGGGCTACAGAATACATAATGAAAGGAGTATACATAAACACAGCTTTATTGAATGCA

TCCTGTGCAGCCATGGATGACTTCCAACTGATTCCAATGATAAGCAAATGCAGAACCAAA

GAAGGGAGACGGAAGACAAATCTGTATGGCTTCATTATAAAAGGAAGATCCCATTTGAGG

AATGACACCGATGTGGTAAACTTTGTGAGCATGGAATTCTCTCTCACTGACCCAAGGCTG

GAGCCACACAAATGGGAAAAGTACTGTGTGCTTGAGGTAGGAGACATGCTCCTACGGACT

GCAATAGGCCAAGTGTCAAGGCCCATGTTCCTGTATGTGAGAACCAATGGGACTTCTAAG

ATCAAAATGAAATGGGGTATGGAGATGAGGCGATGCCTTCTTCAATCCCTTCAACAAATT

GAGAGCATGATTGAAGCCGAGTCTTCTGTCAAAGAAAAAGACATGACCAAGGAATTCTTT

GAAAACAGATCAGAAACATGGCCAATTGGAGAGTCACCCAAAGGAGTGGAGGAAGGCTCC

ATTGGGAAGGTGTGCAGAACATTGCTAGCAAAGTCTGTGTTCAACAGCCTATATGCATCT

CCACAACTCGAGGGGTTTTCAGCTGAATCAAGAAAGTTGCTTCTCATTGTTCAGGCACTT

AGGGACAACCTGGAACCTGGAACCTTCGATCTTGGGGGGCTATATGAAGCAATTGAGGAG

TGCCTGATTAACGATCCCTGGGTTTTGCTTAATGCGTCTTGGTTCAACTCCTTCCTCACA

CATGCACTGAAATAGTCGTGGCAATGCTACTA---TTTGCTATCCATACTGTCCAAAAAA

GTACCTTGTTTCTACT

>A_swan_Kazakhstan_1-267-20-B-Talg-52_2020_EPI1927714

AGCAAAAGCAGGTACTGATCCAAAATGGAAGACTTTGTGCGACAATGCTTCAATCCAATG

ATTGTCGAGCTTGCGGAAAAGGCAATGAAAGAATATGGGGAAGATCCTAAGATCGAAACA

AACAAGTTTGCCGCAATATGTACACACTTAGAAGTCTGCTTCATGTATTCGGATTTACAT

TTCATTGATGAACGAGGCGAATCAATAATCGTAGAATCTGGCGACCCGAATGCATTATTG

AAGCACCGATTTGAGATAATTGAAGGGAGAGACCGAACAATGGCCTGGACAGTGGTGAAT

AGTATATGCAACACTACAGGGGTCGAAAAGCCCAAGTTCCTTCCTGATTTGTATGACTAC

AGAGAAAACCGATTCATTGAAATTGGAGTAACGCGAAGGGAAGTTCACATATACTATCTT

GAAAAAGCCAACAAGATAAAATCAGAGAAAACACACATTCACATATTCTCATTCACTGGG

GAGGAAATGGCCACCAAGGCAGATTACACTCTTGATGAAGAGAGCAGAGCAAGAATAAAA

ACCAGGCTATTCACTATAAGACAAGAGATGGCCAATAGGGGTCTATGGGATTCCTTTCGT

CAGTCCGAGAGAGGCGAAGAGACAATTGAAGAAAGATTTGAAATCACAGGAACCATGCGC

AGGCTTGCTGACCAAAGTCTCCCACCGAACTTCGCCAGCCTTGAAAACTTTAGAGCCTAT

GTGGATGGATTTGAACCGAACGGCTGCATTGAGGGCAAGCTTTCTCAAATGTCAAAAGAA

GTGAATGCCAGAATTGAGCCATTTCTGAAGACAACACCACGCCCTCTCAGATTACCTGAT

GGACCTCCCTGTTCCCAGCGGTCGAAGTTCTTGCTAATGGATGCCCTTAAATTGAGCATT

GAAGACCCGAGCCATGAGGGGGAGGGTATACCGCTGTACGATGCAACCAAATGCATGAAG

ACATTTTTTGGCTGGAAGGAGCCCAACATCGTGAAACCACATGAAAAGGGCATAAACCCT

AATTACCTCCTGGCTTGGAAGCAGGTGCTATCAGAACTCCAAGATATTGAAAACGAGGAG

AAAATCCCAAAAACAAAGAACATGAAGAAGACGAGCCAACTGAAGTGGGCACTTGGTGAA

AACATGGCACCAGAGAAAGTGGACTTTGAGGACTGTAAAGATGTTAGCGATCTAAGACAG

TACGACAGTGACGAACCAGAATCTAGATCACTAGCAAGTTGGATCCAGAGTGAATTTAAC

AAGGCTTGCGAACTGACAGATTCGAGTTGGATTGAACTTGATGAAATAGGGGAAGACGTT

GCTCCGATTGAACACATTGCGAGTATGAGGAGGAACTATTTCACAGCGGAAGTATCCCAT

TGCAGGGCTACAGAATACATAATGAAAGGAGTATACATAAACACAGCTTTATTGAATGCA

TCCTGTGCAGCCATGGATGACTTCCAACTGATTCCAATGATAAGCAAATGCAGAACCAAA

GAAGGGAGACGGAAGACAAATCTGTATGGCTTCATTATAAAAGGAAGATCCCATTTGAGG

AATGACACCGATGTGGTAAACTTTGTGAGCATGGAATTCTCTCTCACTGACCCAAGGCTG

GAGCCACACAAATGGGAAAAGTACTGTGTGCTTGAGGTAGGAGACATGCTCCTACGGACT

GCAATAGGCCAAGTATCAAGGCCCATGTTCCTGTATGTGAGAACCAATGGGACTTCTAAG

ATCAAAATGAAATGGGGTATGGAGATGAGGCGATGCCTTCTTCAATCCCTTCAACAAATT

GAGAGCATGATTGAAGCCGAGTCTTCTGTCAAAGAAAAAGACATGACCAAGGAATTCTTT

GAAAACAGATCAGAAACATGGCCAATTGGAGAGTCACCCAAAGGAGTGGAGGAAGGCTCC

ATTGGGAAGGTGTGCAGAACATTGCTAGCAAAGTCTGTGTTCAACAGCCTATATGCATCT

CCACAACTCGAGGGGTTTTCAGCTGAATCAAGAAAGTTGCTTCTCATTGTTCAGGCACTT

AGGGACAACCTGGAACCTGGGACCTTCGATCTTGGGGGGCTATATGAAGCAATTGAGGAG

TGCCTGATTAACGATCCCTGGGTTTTGCTTAATGCGTCTTGGTTCAACTCCTTCCTCACA

CATGCACTGAAATAGTCGTGGCAATGCTACTA---TTTGCTATCCATACTGTCCAAAAAA

GTACCTTGTTTCTACT

>A_pigeon_Kazakhstan_15-20-B-Talg-5_2020_EPI1927720

AGCAAAAGCAGGTACTGATCCAAAATGGAAGACTTTGTGCGACAATGCTTCAATCCAATG

ATTGTCGAGCTTGCGGAAAAGGCAATGAAAGAATATGGGGAAGATCCTAAGATCGAAACA

AACAAGTTTGCCGCAATATGTACACACTTAGAAGTCTGCTTCATGTATTCGGATTTCCAT

TTCATTGATGAACGAGGCGAATCAATAATCGTAGAATCTGGCGACCCGAATGCATTATTG

AAGCACCGATTTGAGATAATTGAAGGGAGAGACCGAACAATGGCCTGGACAGTGGTGAAT

AGTATATGCAACACTACAGGAGTCGAAAAGCCCAAGTTCCTTCCTGATTTGTATGACTAC

AGAGAAAACCGATTCATTGAAATTGGAGTAACGCGAAGGGAAGTTCACATATACTATCTT

GAAAAAGCCAACAAGATAAAATCAGAGAAAACACACATTCACATATTCTCATTCACTGGG

GAGGAAATGGCCACCAAGGCAGATTACACTCTTGATGAAGAGAGCAGAGCAAGAATAAAA

ACCAGGCTATTCACTATAAGACAAGAGATGGCCAATAGGGGTCTATGGGATTCCTTTCGT

CAGTCCGAGAGAGGCGAAGAGACAATTGAAGAAAGATTTGAAATCACAGGAACCATGCGC

AGGCTTGCTGACCAAAGTCTCCCACCGAACTTCGCCAGCCTTGAAAACTTTAGAGCCTAT

GTGGATGGATTTGAACCGAACGGCTGCATTGAGGGCAAGCTTTCTCAAATGTCAAAAGAA

GTGAATGCCAGAATTGAGCCATTTCTGAAGACAACACCACGCCCTCTCAGATTACCTGAT

GGACCTCCCTGTTCCCAGCGGTCGAAGTTCTTGCTAATGGATGCCCTTAAATTGAGCATT

GAAGACCCGAGCCATGAGGGGGAGGGTATACCGCTGTACGATGCAACCAAATGCATGAAG

ACATTTTTTGGCTGGAAGGAGCCCAACATCGTGAAACCACATGAAAAGGGCATAAACCCT

AATTACCTCCTGGCTTGGAAGCAGGTGCTATCAGAACTCCAAGATATTGAAAACGAGGAG

AAAATCCCAAAAACAAAGAACATGAAGAAGACGAGCCAACTGAAGTGGGCACTTGGTGAA

AACATGGCACCAGAGAAAGTGGACTTTGAGGACTGTAAAGATGTTAGCGATCTAAGACAG

TACGACAGTGACGAACCAGAATCTAGATCACTAGCAAGTTGGATCCAGAGTGAATTTAAC

AAGGCTTGCGAACTGACAGATTCGAGTTGGATTGAACTTGATGAAATAGGGGAAGACGTT

GCTCCGATTGAACACATTGCGAGTATGAGGAGGAACTATTTCACAGCGGAAGTATCCCAT

TGCAGGGCTACAGAATACATAATGAAAGGAGTATACATAAACACAGCTTTATTGAATGCA

TCCTGTGCAGCCATGGATGACTTCCAACTGATTCCAATGATAAGCAAATGCAGAACCAAA

GAAGGGAGACGGAAGACAAATCTGTATGGCTTCATTATAAAAGGAAGATCCCATTTGAGG

AATGACACCGATGTGGTAAACTTTGTGAGCATGGAATTCTCTCTCACTGACCCAAGGCTG

GAGCCACACAAATGGGAAAAGTACTGTGTGCTTGAGGTAGGAGACATGCTCCTACGGACT

GCAATAGGCCAAGTGTCAAGGCCCATGTTCCTGTATGTGAGAACCAATGGGACTTCTAAG

ATCAAAATGAAATGGGGTATGGAGATGAGGCGATGCCTTCTTCAATCCCTTCAACAAATT

GAGAGCATGATTGAAGCCGAGTCTTCTGTCAAAGAAAAAGACATGACCAAGGAATTCTTT

GAAAACAGATCAGAAACATGGCCAATTGGAGAGTCACCCAAAGGAGTGGAGGAAGGCTCC

ATTGGGAAGGTGTGCAGAACATTGCTAGCAAAGTCTGTGTTCAACAGCCTATATGCATCT

CCACAACTCGAGGGGTTTTCAGCTGAATCAAGAAAGTTGCTTCTCATTGTTCAGGCACTT

AGGGACAACCTGGAACCTGGGACCTTCGATCTTGGGGGGCTATATGAAGCAATTGAGGAG

TGCCTGATTAACGATCCCTGGGTTTTGCTTAATGCGTCTTGGTTCAACTCCTTCCTCACA

CATGCACTGAAATAGTCGTGGCAATGCTACTA---TTTGCTATCCATACTGTCCAAAAAA

GTACCTTGTTTCTACT

>A_chicken_Kazakhstan_1-20-B-Talg-67_2020_EPI1927726

AGCAAAAGCAGGTACTGATCCAAAATGGAAGACTTTGTGCGACAATGCTTCAATCCAATG

ATTGTCGAGCTTGCGGAAAAGGCAATGAAAGAATATGGGGAAGATCCTAAGATCGAAACA

AACAAGTTTGCCGCAATATGTACACACTTAGAAGTCTGCTTCATGTATTCGGATTTCCAT

TTCATTGATGAACGAGGCGAATCAATAATCGTGGAATCTGGCGACCCGAATGCATTATTG

AAGCACCGATTTGAGATAATTGAAGGGAGAGACCGAACAATGGCCTGGACAGTGGTGAAT

AGTATATGCAACACTACAGGAGTCGAAAAGCCCAAGTTCCTTCCTGATTTGTATGACTAC

AGAGAAAACCGATTCATTGAAATTGGAGTAACGCGAAGGGAAGTTCACATATACTATCTT

GAAAAAGCCAACAAGATAAAATCAGAGAAAACACACATTCACATATTCTCATTCACTGGG

GAGGAAATGGCCACCAAGGCAGATTACACTCTTGATGAAGAGAGCAGAGCAAGAATAAAA

ACCAGGCTATTCACTATAAGACAAGAGATGGCCAATAGGGGTCTATGGGATTCCTTTCGT

CAGTCCGAGAGAGGCGAAGAGACAATTGAAGAAAGATTTGAAATCACAGGAACCATGCGC

AGGCTTGCTGACCAAAGTCTCCCACCGAACTTCGCCAGCCTTGAAAACTTTAGAGCCTAT

GTGGATGGATTTGAACCGAACGGCTGCATTGAGGGCAAGCTTTCTCAAATGTCAAAAGAA

GTGAATGCCAGAATTGAGCCATTTCTGAAGAAAACACCACGCCCTCTCAGATTACCTGAT

GGACCTCCCTGTTCCCAGCGGTCGAAGTTCTTGCTAATGGATGCCCTTAAATTGAGCATT

GAAGACCCGAGCCATGAGGGGGAGGGTATACCGCTGTACGATGCAACCAAATGCATGAAG

ACATTTTTTGGCTGGAAGGAGCCCAACATCGTGAAACCACATGAAAAGGGCATAAACCCT

AATTACCTCCTGGCTTGGAAGCAGGTGCTATCAGAACTCCAAGATATTGAAAACGAGGAG

AAAATCCCAAAAACAAAGAACATGAAGAAGACGAGCCAACTGAAGTGGGCACTTGGTGAA

AACATGGCACCAGAGAAAGTGGACTTTGAGGACTGTAAAGATGTTAGCGATCTAAAACAG

TACGACAGTGACGAACCAGAATCTAGATCACTAGCAAGTTGGATCCAGAGTGAATTTAAC

AAGGCTTGCGAACTGACAGATTCGAGTTGGATTGAACTTGATGAAATAGGGGAAGACGTT

GCTCCAATTGAACACATTGCGAGTATGAGGAGGAACTATTTCACAGCGGAAGTATCCCAT

TGCAGGGCTACAGAATACATAATGAAAGGAGTATACATAAACACAGCTTTATTGAATGCA

TCCTGTGCAGCCATGGATGACTTCCAACTGATTCCAATGATAAGCAAATGCAGAACCAAA

GAAGGGAGACGGAAGACAAATCTGTATGGCTTCATTATAAAAGGAAGATCCCATTTGAGG

AATGACACCGATGTGGTAAACTTTGTGAGCATGGAATTCTCTCTCACTGACCCAAGGCTG

GAGCCACACAAATGGGAAAAGTACTGTGTGCTTGAGGTAGGAGACATGCTCCTACGGACT

GCAATAGGCCAAGTGTCAAGGCCCATGTTCCTGTATGTGAGAACCAATGGGACTTCTAAG

ATCAAAATGAAATGGGGTATGGAGATGAGGCGATGCCTTCTTCAATCCCTTCAACAAATT

GAGAGCATGATTGAAGCCGAGTCTTCTGTCAAAGAAAAAGACATGACCAAGGAATTCTTT

GAAAACAGATCAGAAACATGGCCAATTGGAGAGTCACCCAAAGGAGTGGAGGAAGGCTCC

ATTGGGAAGGTGTGCAGAACATTGCTAGCAAAGTCTGTGTTCAACAGCCTATATGCATCT

CCACAACTCGAGGGGTTTTCAGCTGAATCAAGAAAGTTGCTTCTCATTGTTCAGGCACTT

AGGGACAACCTGGAACCTGGAACCTTCGATCTTGGGGGGCTATATGAAGCAATTGAGGAG

TGCCTGATTAACGATCCCTGGGTTTTGCTTAATGCGTCTTGGTTCAACTCCTTCCTCACA

CATGCACTGAAATAGTCGTGGCAATGCTACTA---TTTGCTATCCATACTGTCCAAAAAA

GTACCTTGTTTCTACT

>A_duck_Lao_961_2010_EPI335156

------------------------ATGGAAGACTTTGTGCGACAATGCTTCAACCCAATG

ATCGTCGAGCTTGCGGAAAAGGCAATGAAAGAATATGGGGAAAATCCGAAAATCGAAACG

AACAAATTCGCCGCGATATGCACACACCTAGAAGTCTGCTTCATGTATTCGGATTTCCAC

TTTATTGATGAACGAGGCGAATCAATAATTGTAGAATCTGGCGATCCGAATGCATTATTG

AAACACCGATTTGAGATAATTGAAGGGAGAGACCGAACAATGGCTTGGACAGTGGTAAAT

AGTATCTGCAACACCACAGGAGTCGATAAGCCTAAATTCCTCCCAGATTTGTATGACTAC

AAAGAGAACCGATTTATTGAAATTGGAGTGACACGGAGGGAAGTTCACATATACTACCTA

GAAAAAGCAAGTAAGATAAAATCAGAGAAGACACACATTCACATATTCTCATTCACCGGA

GAGGAAATGGCCACCAAAGCGGACTATACCCTTGATGAGGAGAGCAGAGCAAGAATCAAA

ACCAGACTGTTCACTATAAGGCAAGAAATGGCCAGTAGGGGTCTATGGGATTCCTTTCGT

CAATCCGAACGAGGCGAAGAGACAATTGAAGAAAGATTTGAAATCACAGGAACCATGCGC

AGGCTTGCCGACCAAAGTCTCCCACCGAACTTCTCCAGCCTTGAAAATTTTAGAGCCTAT

GTGGATGGATTCGAACCGAACGGCTGCATTGAGGGCAAGCTTTCCCAAATGTCAAAAGAA

GTGAACGCCAGAATTGAGCCATTTATGAAAACAACACCACGCCCTCTAAGACTACCTGAT

GGGCCTCCCTGCTCTCAGCGGTCGAAGTTCTTACTGATGGATGCCCTTAAATTAAGCANN

NNNNNTCCAAGCCATGAGGGGGAAGGTATACCGCTATATGACGCAATCAAATGCATGAAG

ACATTTTTCGGCTGGAAAGAGCCCAACATCGTAAAACCACATGAAAAGGGCATAAATCCC

AATTACCTCCTGGCTTGGAAGCAGGTGATGGCAGAACTCCAAGATATTGAAAATGAGGAG

AAAATCCCAAAGACAAAGAACATGAAGAAAACAAGCCAACTAAAGTGGGCACTTGGTGAG

AACATGGCACCAGAAAAAGTAGACTTTGAGGACTGCAAAGATGTTAGCGATCTAAAACAG

TATGACAGTGATGAACCAGAGCCTAGATCATTATCAAGCTGGATTCAGGGTGAATTCAAC

AAAGCATGCGAATTGACAGATTCGAGTTGGATTGAACTTGATGAAATAGGAGAAGATGTT

GCTCCAATTGAGCACATCGCGAGTATGAGAAGAAACTATTTCACAGCGGAAGTGTCTCAT

TGCAGGGCTACTGAATACATAATGAAGGGAGTGTATATAAATACAGCCCTGTTGAATGCA

TCCTGTGCAGCCATGGATGACTTTCAACTGATTCCAATGATAAGCAAGTGCAGAACCAAA

GAAGGAAGACGGAAGACAAATCTGTATGGATTCATTATAAAAGGAAGGTCCCATTTGAGG

AATGACACCGATGTGGTAAACTTTGTGAGCATGGAATTCTCTCTCACTGACCCGAGGCTG

GAACCACACAAGTGGGAAAAGTACTGTGTTCTCGAGATAGGAGACATGCTCCTGCGAACT

GCAATAGGCCAGGTGTCAAGACCCATGTTCCTGTATGTAAGAACCAATGGGACTTCCAAG

ATCAAGATGAAATGGGGCATGGAAATGAGGCGATGCCTTCTTCAATCCCTTCAACAAATT

GAGAGCATGATTGAGGCCGAGTCTTCTGTCAAAGAGAAAGACATGACCAAAGAATTCTTT

GAAACCAAATCAGAAACGTGGCCAATTGGGGAATCACCTAAAGGGGTGGAGGAAAGCTCC

ATTGGGAAGGTGTGCAGAACATTACTAGCAAAATCTGTATTTAACAGCCTATATGCATCT

CCACAACTTGAGGGGTTTTCAGCTGAATCAAGAAAATTACTTCTCATTGTTCAGGCGCTT

AGGGACAACCTGGAACCTGGAACCTTCGATCTTGGGGGGCTATATGAAGCAATTGAGGAG

TGCCTGATTAATGATCCTTGGGTTTTGCTTAATGCATCTTGGTTCAACTCCTTCCTCATA

CATGCACTGGAATAGTTGTGGCAATGCTACTA---TTTGCTATCCATACTGTCCAAAAAA

GTACCTTGTTTCTACT

>A_breeder_duck_Korea_Gochang1_2014_EPI509692

---------------TGATCCAAGATGGAAGATTTTGTGCGGCAATGCTTCAATCCAATG

ATCGTCGAGCTTGCAGAAAAGGCAATGAAAGAATATGGAGAAAATCCAAAAATCGAAACG

AACAAATTCGCAGCAATATGCACTCACTTAGAAGTCTGTTTCATGTATTCGGATTTCCAC

TTTATTGATGAACGAGGTGAATCAATGATTGTAGAATCTGGCGATCCGAATGCATTATTG

AAACACCGATTTGAGATAATTGAAGGGAGAGACCGAACAATGGCTTGGACAGTGGTAAAT

AGCATCTGCAACACCACAGGAGTCGATAAGCCTAAATTCCTCCCAGATTTGTATGATTAC

AAAGAAAACCGATTCATTGAAATTGGAGTGACAAGGAGGGAAGTTCACACGTACTACCTA

GAGAAGGCAAATAAGATAAAATCAGAGAAGACACACATTCACATATTCTCATTCACTGGA

GAGGAGATGGCCACCAAAGCTGACTATACCCTTGATGAAGAGAGCAGAGCAAGGATCAAA

ACCAGGCTGTTCACTATCAGGCAAGAAATGGCCAATAGGGGTCTATGGGATTCCTTTCGT

CAATCCGAAAGAGGCGAAGAAACAATTGAAGAAAGATTTGAAATCACAGGAACCATGCGC

AGGCTTGCTGACCAAAGTCTCCCACCGAATTTCTCCAGCCTTGAAAATTTTAGAGCCTAT

GTGGATGGATTCAAACCGAACGGCTGCATTGAGGGCAAGCTTTCTCAAATGTCAAAAGAA

GTGAACGCCAGAATTGAGCCATTTGTGAAGACAACACCACGCCCTCTCAGATTACCTGAT

GGGCCTCCTTGTTCTCAGCGGTCGAAATTCTTACTGATGGATGCCCTTAAATTAAGCATC

GAAGATCCAAGCCATGAGGGAGAAGGTATCCCGCTATATGATGCAATCAAATGTATGAAG

ACATTTTTTGGTTGGAAAGAGCCCAACATCGTAAAACCACATGTAAAAGGCATAAATCCC

AACTACCTCCTGGCTTGGAAGCAGGTGCTGGCAGAACTCCAAGACATTGAAACTGAGGAG

AAAATCCCAAAAACAAAGAACATGAAGAAAACAAGCCAATTGAAGTGGGCACTTGGTGAG

AACATGGCACCTGAAAAGGTGGACTTCGAGGACTGCAGAGATGTTAGCGATCTAAGACAG

TATGACAGTGATGAACCAGAGCCCAGATCATTATCAAGCTGGGTCCAGAGCGAATTCAAC

AAAGCATGCGAGTTGACAGATTCAAGTTGGATTGAACTTGATGAAATAGGGGAAGATGTT

GCTCCAATTGAGCACATTGCGAGTATGAGACGAAACTATTTCACAGCGGAAGTGTCTCAT

TGCAGGGCTACTGAGTATATAATGAAAGGAGTGTATGCAAATACAGCTCTGTTGAATGCA

TCCTGTGCTGCCATGGATGATTTCCAATTGATTCCAATAATAAGCAAGTGCAGAACCAAA

GAAGGAAGACGGAAGACAAATCTATATGGATTCATTATAAAAGGAAGATCCCATTTGAGG

AATGACACTGATGTGGTAAACTTTGTGAGCATGGAGTTTTCTCTTACTGACCCGAGGCTG

GAACCACACAAGTGGGAGAAGTACTGTGTTCTCGAGATAGGAGACATGCTCCTACGAACT

GCAATAGGCCAAGTGTCAAGACCCATGTTTCTATATGTGAGAACCAATGGGACCTCCAAG

ATCAAGATGAAATGGGGCATGGAGATGAGGCGATGCCTTCTTCAATCCCTTCAACAAATT

GAGAGCATGATTGAGGCAGAGTCTTCTGTCAAAGAGAAAGACATGACCAAGGAATTCTTT

GAGAACAAATCAGAAACTTGGCCAATTGGGGAATCACCTAGGGGAGTGGAGGAAAGCTCC

ATTGGGAAGGTGTGCAGAACATTACTAGCAAAATCTGTATTCAACAGCCTATATGCATCT

CCACAACTCGAGGGGTTTTCAGCTGAATCAAGAAAATTACTTCTCATTGTTCAGGCATTT

AGGGACAACCTGGAACCTGGGACCTTCGATCTTGGGGGGCTATATGAAGCAATTGAGGAG

TGCCTGATTAATGATCCCTGGGTTTTGCTTAATGCGTCTTGGTTCAACTCCTTCCTCGCA

CATGCACTGAAATAGTTGTGGCAATGCTACTA---TTTGCTATCCATACTGTCCAAAA--

----------------

>A_broiler_duck_Korea_Buan2_2014_EPI509693

---------------TGATCCAAAATGGAAGACTTTGTGCGACAATGCTTCAATCCAATG

ATCGTCGAGCTTGCGGAAAAGACAATGAAAGAATATGGGGAAAATCCAAAAATCGAAACG

AACAAATTCGCTGCAATATGCACTCACTTAGAGGTCTGTTTCATGTATTCGGATTTCCAC

TTTATTGATGAACGAGGTAAATCAATAATTGTAGAATCTGGCGATCCGAATGCATTATTG

AAACACCGATTTGAGATAATTGAAGGGAGAGACCGAACGATGGCTTGGACAGTGGTAAAT

AGTATCTGCAACACCACAGGGGTCGATAAGCCTAAATTCCTCCCAGATTTGTATGATTAC

AAGGAGAACCGATTCATTGAAATTGGAGTGACAAGGAGGGAAGTTCACACATACTACCTA

GAAAAGGCAAATAAGATAAAATCAGAGAAGACACACATTCACATATTCTCATTCACTGGG

GAGGAGATGGCCACCAAAGCTGACTATACCCTTGATGAAGAGAGCAGAGCAAGGATCAAA

ACCAGGTTGTTCACTATCAGGCAAGAAATGGCCAATAGGGGTCTGTGGGATTCCTTTCGT

CAATCTGAGAGAGGCGAAGAGACAATTGAAGAAAGGTTTGAAATCACAGGAACCATGCGC

AGGCTTGCCGACCAAAGTCTCCCACCGAATTTCTCCAGCCTTGAAAATTTTAGAGCCTAT

GTGGATGGATTCAAACCGAACGGCTGCCTTGAGGGCAAGCTTTCTCAAATGTCAAAAGAA

GTGAACGCCAGAATTGAGCCATTCATGAAGACAACACCACGCCCTCTCAGATTACCTGAT

GGTCCTCCTTGCTCTCAGCGGTCGAAATTCTTACTGATGGATGCCCTTAAATTGAGCATC

GAAGACCCAAGCCATGAAGGAGAAGGTATACCGCTATATGATGCAATCAAATGCATGAAG

ACGTTTTTTGGTTGGAAAGAGCCCAACATTGTAAAACCACATGTAAAAGGCATAAATCCC

AACTATCTCTTGGCTTGGAAGCAGGTGCTGGCAGAACTCCAAGACATTGAAAATGAAGAG

AAAATCCCAAAAACAAAAAACATGAAGAAAACAAGCCAACTAAAGTGGGCACTCGGTGAG

AATATGGCACCTGAAAAAGTGGACTTTGAGGACTGCAGAGATGTTAGCGATCTAAGACAG

TATGACAGTGATGAACCAGAGCCCAGATCATTATCAAGCTGGATCCAGAGCGAATTCAAC

AAAGCATGCGAATTGACAGATTCGAGTTGGATTGAACTTGATGAAATAGGAGAAGATGTT

GCTCCAATTGAGCACATTGCGAGTATGAGAAGAAACTACTTCACAGCGGAAGTGTCTCAT

TGCAGGGCTACTGAATATATAATGAAAGGAGTTTATATAAATACAGCCCTGTTGAATTCA

TCCTGTGCAGCCATGGATGACTTCCAATTGATTCCAATGATAAGCAAGTGCAGAACCAAA

GAAGGAAGACGGAAGACAAATCTATATGGGTTCATTATAAAAGGAAGATCCCATTTGAGG

AATGATACCGATGTGGTAAATTTTGTGAGCATGGAGTTCTCTCTTACTGACCCGAGGCTG

GAACCACACAAGTGGGAAAAGTACTGTGTTCTCGAAATAGGAGACATGCTCCTACGAACT

GCAATAGGCCAAGTATCAAGACCCATGTTTCTTTATGTAAGAACCAATGGGACTTCCAAG

ATCAAGATGAAATGGGGCATGGAGATGAGGCGATGCCTTCTTCAATCCCTCCAACAAATT

GAGAGCATGATTGAGGCAGAGTCTTCTGTCAAAGAGAAAGACATGACCAAGGAATTCTTT

GAAAATAAATCAGAAACGTGGCCAATTGGGGAATCACCTAAGGGGGTGGAGGAAAGCTCT

ATTGGGAAGGTGTGTAGAACATTACTAGCAAAATCTGTATTCAACAGCCTATATGCATCT

CCACAACTTGAGGGGTTTTCAGCTGAGTCGAGAAAGTTACTTCTCATTGTTCAGGCATTT

AGGGACAACCTGGAACCTGGGACCTTCGATCTTGGGGGGCTATATGAAGCAATTGAGGAG

TGCCTGATTAATGATCCCTGGGTTTTGCTTAATGCATCTTGGTTCAACTCCTTCCTTACA

CATGCACTGAAATAGTTGTGGCAATGCTACTA---TTTGCTATCCATACTGTCCAAAA--

----------------

>A_goose_Taiwan_TNO15_2015_EPI690743

------------------------ATGGAAGACCTTGTGCGGCAATGCTTCAATCCAATG

ATCGTCGAGCTTGCGGAAAAGACAATGAAAGAATATGGGGAAAATCCAAAAATCGAAACG

AACAAATTCGCTGCAATATGCACTCACTTAGAGGTCTGTTTCATGTATTCGGATTTCCAC

TTTATTGATGAACGAGGCAAATCAATAATTGTAGAATCTGGCGATCCGAATGCATTATTG

AAACACCGATTTGAGATAATTGAAGGGAGAGACCGAACGATGGCTTGGACAGTGGTAAAT

AGTATCTGCAACACCACAGGAGTCGATAAGCCTAAATTCCTCCCAGATTTGTATGATTAC

AAGGAGAACCGATTCATTGAAATTGGAGTGACAAGGAGGGAAGTTCACACATACTACCTA

GAAAAGGCAAATAAGATAAAATCAGAGAAGACACACATTCACATATTCTCATTCACTGGG

GAGGAGATGGCCACCAAAGCTGACTATACCCTTGATGAAGAGAGCAGAGCAAGGATCAAA

ACCAGGTTGTTCACTATCAGGCAAGAAATGGCCAATAGGGGTCTGTGGGATTCCTTTCGT

CAATCTGAGAGAGGCGAAGAGACAATTGAAGAAAGGTTTGAAATCACAGGAACCATGCGC

AGGCTTGCCGACCAAAGCCTCCCACCGAATTTCTCCAGCCTTGAAAATTTTAGAGCCTAT

GTGGATGGATTCAAACCGAACGGCTGCCTTGAGGGCAAGCTTTCTCAAATGTCAAAAGAA

GTGAACGCCAGAATTGAGCCATTCATGAAGACAACACCACGCCCTCTCAGATTACCTGAT

GGTCCTCCTTGCTCTCAGCGGTCGAAATTCTTACTGATGGATTCCCTTAAATTGAGCATC

GAAGACCCAAGCCATGAGGGAGAAGGTATACCGCTATATGATGCAATCAAATGCATGAAG

ACGTTTTTTGGTTGGAAAGAGCCCAACATTGTAAAACCACATGTAAAAGGCATAAATCCC

AACTATCTCTTGGCTTGGAAGCAGGTGCTGGCAGAACTCCAAGACATTGAGAATGAAGAG

ACAATCCCAAAAACAAAAAACATGAAGAAAACAAGCCAACTAAAGTGGGCACTCGGTGAG

AATATGGCACCTGAAAAAGTGGACTTTGAGGACTGCAGAGATGTTAGCGATCTAAGACAG

TATGACAGTGATGAACCAGAGCCCAGATCATTATCAAGCTGGATCCAGAGCGAATTCAAC

AAAGCATGCGAATTGACAGATTCGAGTTGGATTGAACTTGATGAAATAGGAGAAGATGTT

GCTCCAATTGAGCACATTGCGAGTATGAGAAGAAACTACTTCACAGCGGAAGTGTCTCAT

TGCAGGGCAACTGAATATATAATGAAAGGAGTTTATATAAATACAGCCCTGTTGAATTCA

TCCTGTGCAGCCATGGATGACTTCCAATTGATTCCAATGATAAGCAAGTGCAGAACCAAA

GAAGGAAGACGGAAGACAAATTTATATGGGTTCATTATAAAAGGAAGATCCCATTTGAGG

AATGATACCGATGTGGTAAATTTTGTGAGCATGGAGTTCTCCCTCACTGACCCGAGGCTG

GAACCACACAAGTGGGAAAAGTACTGTGTTCTCGAAATAGGAGACATGCTCCTACGAACT

GCAATAGGCCAAGTATCAAGATCCATGTTTCTTTATGTAAGAACCAATGGGACTTCCAAG

ATCAAGATGAAATGGGGCATGGAGATGAGGCGATGCCTTCTTCAATCCCTCCAACAAATT

GAGAGCATGATTGAGGCAGAGTCTTCTGTCAAAGAGAAAGACATGACCAAGGAATTCTTT

GAAAATAAATCAGAAACGTGGCCAATTGGGGAATCACCTAAGGGGGTGGAGGAAAGCTCT

ATTGGGAAGGTGTGTAGAACATTATTAGCAAAATCTGTATTCAACAGCCTATATGCATCT

CCACAACTTGAGGGGTTTTCAGCTGAGTCGAGAAAGTTACTTCTCATTGTTCAGGCATTT

AGGGACAACCTGGAACCTGGGACCTTCGATCTTGGGGGGCTATATGAAGCAATTGAGGAG

TGCCTGATTAATGATCCCTGGGTTTTGCTTAATGCATCTTGGTTCAACTCCTTCCTTACA

CATGCACTGAAATAG---------------------------------------------

----------------

>A_wigeon_Sakha_1_2014_EPI1201483

AGCGAAAGCAGGTACTGATCCAAAATGGAAGACTTTGTGCGACAATGCTTCAATCCAATG

ATCGTCGAGCTTGCGGAAAAGACAATGAAAGAATATGGGGAAAATCCAAAAATCGAAACG

AACAAATTCGCTGCAATATGCACTCACTTAGAGGTCTGTTTCATGTATTCGGATTTCCAC

TTTATTGATGAACGAGGTAAATCAATAATTGTAGAATCTGGCGATCCGAATGCATTATTG

AAACACCGATTTGAGATAATTGAAGGGAGAGACCGAACGATGGCTTGGACAGTGGTAAAT

AGTATCTGCAACACCACAGGAGTCGATAAGCCTAAATTCCTCCCAGATTTGTATGATTAC

AAGGAGAACCGATTCATTGAAATTGGAGTGACAAGGAGGGAAGTTCACACATACTACCTA

GAAAAGGCAAATAAGATAAAATCAGAGAAGACACACATTCACATATTCTCATTCACTGGG

GAGGAGATGGCCACCAAAGCTGATTATATCCTTGATGAAGAGAGCAGAGCAAGGATCAAA

ACCAGGTTGTTCACTATCAGGCAAGAAATGGCCAATAGGGGTCTGTGGGATTCCTTTCGT

CAATCTGAGAGAGGCGAAGAGACAATTGAAGAAAGGTTTGAAATCACAGGAACCATGCGC

AGGCTTGCTGACCAAAGTCTCCCACCGAATTTCTCCAGCCTTGAAAATTTTAGAGCCTAT

GTGGATGGATTCAAACCGAACGGCTGCCTTGAGGGCAAGCTTTCTCAAATGTCAAAAGAA

GTGAACGCCAGAATTGAGCCATTCATGAAGACAACACCACGCCCTCTCAGATTACCTGAT

GGTCCTCCTTGCTCTCAGCGGTCGAAATTCTTACTGATGGATGCCCTTAAATTGAGCATC

GAAGACCCAAGCCATGAGGGAGAAGGTATACCGCTATATGATGCAATCAAATGCATGAAG

ACGTTTTTTGGTTGGAAAGAGCCCAACATTGTAAAACCACATGTAAAAGGCATAAATCCC

AACTATCTCTTGGCTTGGAAGCAGGTGCTGGTAGAACTCCAAGACATTGAAAATGAAGAG

AAAATCCCAAAAACAAAAACCATGAAGAAAACAAGCCAACTAAAGTGGGCACTCGGTGAG

AATATGGCACCTGAAAAAGTGGACTTTGAGGACTGCAAAGATGTTAGCGATCTAAGACAG

TATGACAGTGATGAACCAGAGCCCAGATCATTATCAAGCTGGATCCAGAGCGAATTCAAC

AAAGCATGCGAATTGACAGATTCGAGTTGGATTGAACTTGATGAAATAGGAGAAGATGTT

GCTCCAATTGAGCACATTGCGAGTATGAGAAGAAACTACTTCACAGCGGAAGTGTCTCAT

TGCAGGGCTACTGAATATATAATGAAAGGAGTTTATATAAATACAGCCCTGTTGAATTCA

TCCTGTGCAGCCATGGATGACTTCCAATTGATTCCAATGATAAGCAAGTGCAGAACCAAA

GAAGGAAGACGGAAGACAAATCTATATGGGTTCATTATAAAAGGAAGATCCCATTTGAGG

AATGATACCGATGTGGTAAATTTTGTGAGCATGGAGTTCTCTCTTACTGACCCGAGGCTG

GAACCACACAAGTGGGAAAAGTACTGTGTTCTCGAAATAGGAGACATGCTCCTACGAACT

GCAATAGGCCAAGTATCAAGACCCATGTTTCTTTATGTAAGGACCAATGGGACTTCCAAG

ATCAAGATGAAATGGGGCATGGAGATGAGGCGATGCCTTCTTCAATCCCTCCAACAAATT

GAGAGCATGATTGAGGCAGAGTCTTCTGTCAAAGAGAAGGACATGACCAAGGAATTCTTT

GAAAATAAATCAGAAACGTGGCCAATTGGGGAATCACCTAAGGGGGTGGAGGAAAGCTCT

ATTGGGAAAGTGTGTAGAACATTACTAGCAAAATCTGTATTCAACAGCCTATATGCATCT

CCACAACTTGAGGGGTTTTCAGCTGAGTCGAGAAAGTTACTTCTCATTGTTCAGGCATTT

AGGGACAACCTGGAACCTGGGACCTTCGATCTTGGGGGGCTATATGAAGCAATTGAGGAG

TGCCTGATTAATGATCCCTGGGTTTTGCTTAATGCATCTTGGTTCAACTCCTTCCTTACA

CATGCACTGAAATAGTTGTGGCAATGCTACTA---TTTGCTATCCATACTGTCCAAAAAA

GTACCTTGTTTCTACT

>A_duck_Nigeria_SK28T_19VIR8424-2_2019_EPI1777111

AGCGAAAGCAGGTACTGATCCAAAATGGAAGACTTTGTGCGACAATGCTTCAATCCAATG

ATTGTCGAGCTTGCGGAAAAGGCAATGAAAGAATATGGGGAAGATCCGAAAATCGAAACA

AATAAATTTGCCGCAATATGCACGCACTTAGAAGTCTGTTTCATGTATTCAGACTTCCAC

TTCATTGATGAACGAGGCGAATCAATAATTGTAGAATCTGGCGATCCGAACGCATTATTG

AAACACCGATTTGAAATAATTGAAGGGAGAGACCGAACAATTGCCTGGACGGTGGTGAAC

AGTATCTGCAACACCACAGGAGTCGAAAAGCCCAAATTCCTCCCTGATTTGTATGACTAC

AAAGAGAACCGATTCATTGAAATTGGAATAACACGAAGGGAAGTTCACATATACTATCTA

GAAAAAGCCAACAAGATAAAATCAGAAAAGACACACATTCACATATTCTCATTCACTGGA

GAGGAGATGGCCACCAGGGCGGACTACACCCTTGATGAAGAGAGCAGGGCAAGAATAAAA

ACCAGGCTGTTCACTATAAGACAAGAAATGGCCAGCAGGGGTCTATGGGATTCCTTTCGT

CAGTCCGAGAGAGGCGAAGAGACAATTGAAGAAAGATTTGAAATCACAGGAACCATGCGT

AGGCTTGCCGACCAAAGTCTCCCACCGAACTTCTCCAGCCTTGAAAACTTTAGAGCCTAT

GTGGATGGATTCAAACCGAACGGCTGCATTGAGGGCAAGCTTTCTCAAATGTCAAAAGAA

GTAAATGCCAGAATTGAGCCATTTCTGAAGACAACACCACGCCCTCTCAGATTACCTGAT

GGACCTCTCTGTTATCAGCGGTCGAAGTTCTTGCTGATGGATGCCCTTAAATTAAGCATC

GAGGACCCAAGCCATGAGGGAGAGGNTATACCGCTGTATGATGCAATCAAATGCATGAAG

ACATTTTTTGNCTGGAAAGAGCCCAACATCGTAAAACCACATGAAAAAGGCATAAACCCT

AATTACCTCCTGGCTTGGAAGCAGGTACTAGCAGAACTCCAAGACATTGAAAATGAGGAG

AAAATTCCAAAAACAAAGAACATGAAGAAAACAAGCCAATTGAAGTGGGCACTTGGTGAG

AACATGGCACCAGAGAAAGTGGACTTTGAGGACTGTAAAGATGTTAGCGATCTAAGACAG

TACGACAGTGGCGAACCAGAGTCCAGATCACTAGCAAACTGGATCCAGAGTGAATTCAAC

AAGGCATGCGAATTGACAGATTCGAGTTGGATTGAACTTGATGAAATAGGGGAGGACGTT

GCTCCAATTGAACACATTGCGAGTATGAGGAGGAACTATTTCACAGCGGAGGTATCCCAC

TGCAGGGCTACTGAATACATAATGAAGGGAGTATACGTAAACACAGCACTACTGAATGCG

TCCTGTGCAGCCATGGACGACTTCCAACTGATTCCAATGATAAGCAAATGCAGAACCAAA

GAAGGAAGAAGGAAGACAAATCTGTATGGATTCATTATAAAGGGAAGATCCCATTTGAGG

AATGACACCGATGTGGTAAATTTTGTGAGCATGGAATTCTCTCTCACTGACCCGAGGCTG

GAGCCACACAAATGGGAAAAGTACTGTGTTCTAGAAATAGGAGACATGCTCCTACGGACT

GCAATAGGCCAAGTGTCAAGGCCCATGTTCCTGTATGTGAGAACCAATGGGACTTCCAAG

ATCAAAATGAAATGGGGCATGGAGATGAGGCGATGCCTTCTTCAGTCCCTTCAACAAATT

GAGAGCATGATCGAGGCCGAGTCTTCTGTCAAAGAGAAGGACATGACCAAGGAATTCTTT

GAAAACAAATCAGAAACATGGCCAATTGGGGGATCACCCAAAGGGGTGGAGGAAGGCTCC

ATTGGGAAGGTGTGCAGAACATTGTTAGCAAAGTCTGTGTTCAACAGCCTATATGCATCT

CCACAACTCGAGGGGTTTTCAGCTGAATCAAGAAAATTGCTTCTCATTGTTCAGGCGCTT

AGAGACAATCTGGAACCTGGGACCTTCGATCTTGGGGGNCTATATGAAGCAATTGAGGAG

TGCCTGATTAACGATCCCTGGGTTTTGCTTAATGCGTCTTGGTTCAACTCCTTCCTCACA

CATGCACTGAAATAGTTGTGGCAATGCTACTA---TTTGCTATCCATACTGTCCAAAAAN

GTACCTTGTTTCTACT

>A_mute_swan_Kazakhstan_1-267-20-B_2020_EPI1811583

------------------------ATGGAAGACTTTGTGCGACAATGCTTCAATCCAATG

ATTGTCGAGCTTGCGGAAAAGGCAATGAAAGAATATGGGGAAGATCCTAAGATCGAAACA

AACAAGTTTGCCGCAATATGTACACACTTAGAAGTCTGCTTCATGTATTCGGATTTCCAT

TTCATTGATGAACGAGGCGAATCAATAATCGTAGAATCTGGCGACCCGAATGCATTATTG

AAGCACCGATTTGAGATAATTGAAGGGAGAGACCGAACAATGGCCTGGACAGTGGTGAAT

AGTATATGCAACACTACAGGAGTCGAAAAGCCCAAGTTCCTTCCTGATTTGTATGACTAC

AGAGAAAACCGATTCATTGAAATTGGAGTAACGCGAAGGGAAGTTCACATATACTATCTT

GAAAAAGCCAACAAGATAAAATCAGAGAAAACACACATTCACATATTCTCATTCACTGGG

GAGGAAATGGCCACCAAGGCAGATTACACTCTTGATGAAGAGAGCAGAGCAAGAATAAAA

ACCAGGCTATTCACTATAAGACAAGAGATGGCCAATAGGGGTCTATGGGATTCCTTTCGT

CAGTCCGAGAGAGGCGAAGAGACAATTGAAGAAAGATTTGAAATCACAGGAACCATGCGC

AGGCTTGCTGACCAAAGTCTCCCACCGAACTTCGCCAGCCTTGAAAACTTTAGAGCCTAT

GTGGATGGATTTGAACCGAACGGCTGCATTGAGGGCAAGCTTTCTCAAATGTCAAAAGAA

GTGAATGCCAGAATTGAGCCATTTCTGAAGACAACACCACGCCCTCTCAGATTACCTGCT

GGACCTCCCTGTTCCCAGCGGTCGAAATTCTTGCTAATGGATGCCCTTAAATTGAGCATT

GAAGACCCGAGCCATGAGGGGGAGGGTATACCGCTGTACGATGCAACCAAATGCATGAAG

ACATTTTTTGGCTGGAAGGAGCCCAACATCGTGAAACCACATGAAAAGGGCATAAACCCT

AATTACCTCCTGGCTTGGAAGCAGGTGCTATCAGAACTCCAAGATATTGAAAACGAGGAG

AAAATCCCAAAAACAAAGAACATGAAGAAGACGAGCCAACTGAAGTGGGCACTTGGTGAA

AACATGGCACCAGAGAAAGTGGACTTTGAGGACTGTAAAGATGTTAGCGATCTAAAACAG

TACGACAGTGACGAACCAGAATCTAGATCACTAGCAAGTTGGATCCAGAGTGAATTTAAC

AAGGCTTGCGAACTGACAGATTCGAGTTGGATTGAACTTGATGAAATAGGGGAAGACGTT

GCTCCAATTGAACACATTGCGAGTATGAGGAGGAACTATTTCACAGCGGAAGTATCCCAT

TGCAGGGCTACAGAATACATAATGAAAGGAGTATACATAAACACAGCTTTATTGAATGCA

TCCTGTGCAGCCATGGATGACTTCCAACTGATTCCAATGATAAGCAAATGCAGAACCAAA

GAAGGGAGACGGAAGACAAATCTGTATGGCTTCATTATAAAAGGAAGATCCCATTTGAGG

AATGACACCGATGTGGTAAACTTTGTGAGCATGGAATTCTCTCTCACTGACCCAAGGCTG

GAGCCACACAAATGGGAAAAATACTGTGTGCTTGAGGTAGGAGACATGCTCCTACGGACT

GCAATAGGCCAAGTGTCAAGGCCCATGTTCCTGTATGTGAGAACCAATGGGACTTCTAAG

ATCAAAATGAAATGGGGTATGGAGATGAGGCGATGCCTTCTTCAATCCCTTCAACAAATT

GAGAGCATGATTGAAGCCGAGTCTTTTGTCAAAGAAAAAGACATGACCAAGGAATTCTTT

GAAAACAGATCAGAAACATGGCCAATTGGAGAGTCACCCAACGGAGTGGAGGAAGGCTCC

ATTGGGAAGGTGTGCAGAACATTGCTAGCAAAGTCTGTGTTCAACAGCCTATATGCATCT

CCACAACTCGAGGGGTTTTCAGCTGAATCAAGAAAGTTGCTTCTCATTGTTCAGGCACTT

AGGGACAACCTGGAACCTGGAACCTTCGATCTTGGGGGGCTATATGAAGCAATTGAGGAG

TGCCTGATTAACGATCCCTGGGTTTTGCTTAATGCGTCTTGGTTCAACTCCTTCCTCACA

CATGCACTGAAATAG---------------------------------------------

----------------

>A_domestic_goose_Kazakhstan_1-248_2-20-B_2020_EPI1811600

-GCGAAAGCAGGTACTGATCCAAAATGGAAGACTTTGTGCGACAATGCTTCAATCCAATG

ATTGTCGAGCTTGCGGAAAAGGCAATGAAAGAATATGGGGAAGATCCTAAGATCGAAACA

AACAAGTTTGCCGCAATATGTACACACTTAGAAGTCTGCTTCATGTATTCGGATTTCCAT

TTCATTGATGAACGAGGCGAATCAATAATCGTAGAATCTGGCGACCCGAATGCATTATTG

AAGCACCGATTTGAGATAATTGAAGGGAGAGACCGAACAATGGCCTGGACAGTGGTGAAT

AGTATATGCAACACTACAAGAGTCGAAAAGCCCAAGTTCCTTCCTGATTTGTATGACTAC

AGAGAAAACCGATTCATTGAAATTGGAGTAACGCGAAGGGAAGTTCACATATACTATCTT

GAAAAAGCCAACAAGATAAAATCAGAGAAAACACACATTCACATATTCTCATTCACTGGG

GAGGAAATGGCCACCAAGGCAGATTACACTCTTGATGAAGAGAGCAGAGCAAGAATAAAA

ACCAGGCTATTCACTATAAGACAAGAGATGGCCAATAGGGGTCTATGGGATTCCTTTCGT

CAGTCCGAGAGAGGCGAAGAGACAATTGAAGAAAGATTTGAAATCACAGGAACCATGCGC

AGGCTTGCTGACCAAAGTCTCCCACCGAACTTCGCCAGCCTTGAAAACTTTAGAGCCTAT

GTGGATGGATTTGAACCGAACGGCTGCATTGAGGGCAAGCTTTCTCAAATGTCAAAAGAA

GTGAATGCCAGAATTGAGCCATTTCTGAAGACAACACCACGCCCTCTCAGATTACCTGAT

GGACCTCCCTGTTCCCAGCGGTCGAAGTTCTTGCTAATGGATGCCCTTAAATTGAGCATT

GAAGACCCGAGCCATGAGGGGGAGGGTATACCGCTGTACGATGCAATCAAATGCATGAAG

ACATTTTTTGGCTGGAAGGAGCCCAACATCGTGAAACCACATGAAAAGGGCATAAACCCT

AATTACCTCCTGGCTTGGAAGCAGGTGCTATCAGAACTCCAAGATATTGAAAACGAGGAG

AAAATCCCAAAAACAAAGAACATGAAGAAGACGAGCCAACTGAAGTGGGCACTTGGTGAA

AACATGGCACCAGAGAAAGTGGACTTTGAGGACTGTAAAGATGTTAGCGATCTAAAACAG

TACGACAGTGACGAACCAGAATCTAGATCACTAGCAAGTTGGATCCAGAGTGAATTTAAC

AAGGCTTGCGAACTGACAGATTCGAGTTGGATTGAACTTGATGAAATAGGGGAAGACGTT

GCTCCAATTGAACACATTGCGAGTATGAGGAGGAACTATTTCACAGCGGAAGTATCCCAT

TGCAGGGCTACAGAATACATAATGAAAGGAGTATACATAAACACAGCTTTATTGAATGCA

TCCTGTGCAGCCATGGATGACTTCCAACTGATTCCAATGATAAGCAAATGCAGAACCAAA

GAAGGGAGACGGAAGACAAATCTGTATGGCTTCATTATAAAAGGAAGATCCCATTTGAGG

AATGACACCGATGTGGTAAACTTTGTGAGCATGGAATTCTCTCTCACTGACCCAAGGCTG

GAGCCACACAAATGGGAAAAGTACTGTGTGCTTGAGGTAGGAGACATGCTCCTACGGACT

GCAATAGGCCAAGTGTCAAGGCCCATGTTCCTGTATGTGAGAACCAATGGGACTTCTAAG

ATCAAAATGAAATGGGGTATGGAGATGAGGCGATGCCTTCTTCAATCCCTTCAACAAATT

GAGAGCATGATTGAAGCCGAGTCTTCTGTCAAAGAAAAAGACATGACCAAGGAATTCTTT

GAAAACAGATCAGAAACATGGCCAATCGGAGAGTCACCCAAAGGAGTGGAGGAAGGCTCC

ATTGGGAAGGTGTGCAGAACATTGCTAGCAAAGTCTGTGTTCAACAGCCTATATGCATCT

CCACAACTCGAGGGGTTTTCAGCTGAATCAAGAAAGTTGCTTCTCATTGTTCAGGCACTT

AGGGACAACCTGGAACCTGGAACCTTCGATCTTGGGGGGCTATATGAAGCAATTGAGGAG

TGCCTGATTAACGATCCCTGGGTTTTGCTTAATGCGTCTTGGTTCAACTCCTTCCTCACA

CATGCACTGAAATAGTCGTGGCAATGCTACTA---TTTGCTATCCATACTGTCCAAAAAA

----------------

>A_domestic_duck_Kazakhstan_1-274-20-B_2020_EPI1811610

---------------TGATCCAAAATGGAAGACTTTGTGCGACAATGCTTCAATCCAATG

ATTATCGAGCTTGCGGAAAAGGCAATGAAAGAATATGGGGAAGATCCTAAGATCGAAACA

AACAAGTTTGCCGCAATATGTACACACTTAGAAGTCTGCTTCATGTATTCGGATTTCCAT

TTCATTGATGAACGAGGCGAATCAATAATCGTAGAATCTGGCGACCCGAATGCATTATTG

AAGCACCGATTTGAGATAATTGAAGGGAGAGACCGAACAATGGCCTGGACAGTGGTGAAT

AGTATATGCAACACTACAGGAGTCGAAAAGCCCAAGTTCCTTCCTGATTTGTATGACTAC

AGAGAAAACCGATTCATTGAAATTGGAGTAACGCGAAGGGAAGTTCACATATACTATCTT

GAAAAAGCCAACAAGATAAAATCAGAGAAAACACACATTCACATATTCTCATTCACTGGG

GAGGAAATGGCCACCAAGGCAGATTACACTCTTGATGAAGAGAGCAGAGCAAGAATAAAA

ACCAGGCTATTCACTATAAGACAAGAGATGGCCAATAGGGGTCTATGGGATTCCTTTCGT

CAGTCCGAGAGAGGCGAAGAGACAATTGAAGAAAGATTTGAAATCACAGGAACCATGCGC

AGGCTTGCTGACCAAAGTCTCCCACCGAACTTCGCCAGCCTTGAAAACTTTAGAGCCTAT

GTGGATGGATTTGAACCGAACGGCTGCATTGAGGGCAAGCTTTCTCAAATGTCAAAAGAA

GTGAATGCCAGAATTGAGCCATTTCTGAAGACAACACCACGCCCTCTCAGATTACCTGAT

GGACCTCCCTGTTCCCAGCGGTCGAAGTTCTTGCTAATGGATGCCCTTAAATTGAGCATT

GAAGACCCGAGCCATGAGGGGGAGGGTATACCGCTGTACGATGCAACCAAATGCATGAAG

ACATTTTTTGGCTGGAAGGAGCCCAACATCGTGAAACCACATGAAAAGGGCATAAACCCT

AATTACCTCCTGGCTTGGAAGCAGGTGCTATCAGAACTCCAAGATATTGAAAACGAGGAG

AAAATCCCAAAAACAAAGAACATGAAGAAGACGAGCCAACTGAAGTGGGCACTTGGTGAA

AACATGGCACCAGAGAAAGTGGACTTTGAGGACTGTAAAGATGTTAGCGATCTAAGACAG

TACGACAGTGACGAACCAGAATCTAGATCACTAGCAAGTTGGATCCAGAGTGAATTTAAC

AAGGCTTGCGAACTGACAGATTCGAGTTGGATTGAACTTGATGAAATAGGGGAAGACGTT

GCTCCGATTGAACACATTGCGAGTATGAGGAGGAACTATTTCACAGCGGAAGTATCCCAT

TGCAGGGCTACAGAATACATAATGAAAGGAGTATACATAAACACAGCTTTATTGAATGCA

TCCTGTGCAGCCATGGATGACTTCCAACTGATTCCAATGATAAGCAAATGCAGAACCAAA

GAAGGGAGACGGAAGACAAATCTGTATGGCTTCATTATAAAAGGAAGATCCCATTTGAGG

AATGACACCGATGTGGTAAACTTTGTGAGCATGGAATTCTCTCTCACTGACCCAAGGCTG

GAGCCACACAAATGGGAAAAGTACTGTGTGCTTGAGGTAGGAGACATGCTCCTACGGACT

GCAATAGGCCAAGTGTCAAGGCCCATGTTCCTGTATGTGAGAACCAATGGGACTTCTAAG

ATCAAAATGAAATGGGGTATGGAGATGAGGCGATGCCTTCTTCAATCCCTTCAACAAATT

GAGAGCATGATTGAAGCCGAGTCTTCTGTCAAAGAAAAAGACATGACCAAGGAATTCTTT

GAAAACAGATCAGAAACATGGCCAATTGGAGAGTCACCCAAAGGAGTGGAGGAAGGCTCC

ATTGGGAAGGTGTGCAGAACATTGCTAGCAAAGTCTGTGTTCAACAGCCTATATGCATCT

CCACAACTCGAGGGGTTTTCAGCTGAATCAAGAAAGTTGCTTCTCATTGTTCAGGCACTT

AGGGACAACCTGGAACCTGGGACCTTCGATCTTGGGGGGCTATATGAAGCAATTGAGGAG

TGCCTGATTAACGATCCCTGGGTTTTGCTTAATGCGTCTTGGTTCAACTCCTTCCTCACA

CATGCACTGAAATAGTCGTGGCAATGCTACTA---TTTGCTATCCATACTGTCCAAAA--

----------------

>A_domestic_goose_Kazakhstan_1-242_2-20-B_2020_EPI1811618

----------------------------------------------GCTTCAATCCAATG

ATTGTCGAGCTTGCGGAAAAGGCAATGAAAGAATATGGGGAAGATCCTAAGATCGAAACA

AACAAGTTTGCCGCAATATGTACACACTTAGAAGTCTGCTTCATGTATTCGGATTTACAT

TTCATTGATGAACGAGGCGAATCAATAATCGTAGAATCTGGCGACCCGAATGCATTATTG

AAGCACCGATTTGAGATAATTGAAGGGAGAGACCGAACAATGGCCTGGACAGTGGTGAAT

AGTATATGCAACACTACAGGGGTCGAAAAGCCCAAGTTCCTTCCTGATTTGTATGACTAC

AGAGAAAACCGATTCATTGAAATTGGAGTAACGCGAAGGGAAGTTCACATATACTATCTT

GAAAAAGCCAACAAGATAAAATCAGAGAAAACACACATTCACATATTCTCATTCACTGGG

GAGGAAATGGCCACCAAGGCAGATTACACTCTTGATGAAGAGAGCAGAGCAAGAATAAAA

ACCAGGCTATTCACTATAAGACAAGAGATGGCCAATAGGGGTCTATGGGATTCCTTTCGT

CAGTCCGAGAGAGGCGAAGAGACAATTGAAGAAAGATTTGAAATCACAGGAACCATGCGC

AGGCTTGCTGACCAAAGTCTCCCACCGAACTTCGCCAGCCTTGAAAACTTTAGAGCCTAT

GTGGATGGATTTGAACCGAACGGCTGCATTGAGGGCAAGCTTTCTCAAATGTCAAAAGAA

GTGAATGCCAGAATTGAGCCATTTCTGAAGACAACACCACGCCCTCTCAGATTACCTGAT

GGACCTCCCTGTTCCCAGCGGTCGAAGTTCTTGCTAATGGATGCCCTTAAATTGAGCATT

GAAGACCCGAGCCATGAGGGGGAGGGTATACCGCTGTACGATGCAACCAAATGCATGAAG

ACATTTTTTGGCTGGAAGGAGCCCAACATCGTGAAACCACATGAAAAGGGCATAAACCCT

AATTACCTCCTGGCTTGGAAGCAGGTGCTATCAGAACTCCAAGATATTGAAAACGAGGAG

AAAATCCCAAAAACAAAGAACATGAAGAAGACGAGCCAACTGAAGTGGGCACTTGGTGAA

AACATGGCACCAGAGAAAGTGGACTTTGAGGACTGTAAAGATGTTAGCGATCTAAGACAG

TACGACAGTGACGAACCAGAATCTAGATCACTAGCAAGTTGGATCCAGAGTGAATTTAAC

AAGGCTTGCGAACTGACAGATTCGAGTTGGATTGAACTTGATGAAATAGGGGAAGACGTT

GCTCCGATTGAACACATTGCGAGTATGAGGAGGAACTATTTCACAGCGGAAGTATCCCAT

TGCAGGGCTACAGAATACATAATGAAAGGAGTATACATAAACACAGCTTTATTGAATGCA

TCCTGTGCAGCCATGGATGACTTCCAACTGATTCCAATGATAAGCAAATGCAGAACCAAA

GAAGGGAGACGGAAGACAAATCTGTATGGCTTCATTATAAAAGGAAGATCCCATTTGAGG

AATGACACCGATGTGGTAAACTTTGTGAGCATGGAATTCTCTCTCACTGACCCAAGGCTG

GAGCCACACAAATGGGAAAAGTACTGTGTGCTTGAGGTAGGAGACATGCTCCTACGGACT

GCAATAGGCCAAGTATCAAGGCCCATGTTCCTGTATGTGAGAACCAATGGGACTTCTAAG

ATCAAAATGAAATGGGGTATGGAGATGAGGCGATGCCTTCTTCAATCCCTTCAACAAATT

GAGAGCATGATTGAAGCCGAGTCTTCTGTCAAAGAAAAAGACATGACCAAGGAATTCTTT

GAAAACAGATCAGAAACATGGCCAATTGGAGAGTCACCCAAAGGAGTGGAGGAAGGCTCC

ATTGGGAAGGTGTGCAGAACATTGCTAGCAAAGTCTGTGTTCAACAGCCTATATGCATCT

CCACAACTCGAGGGGTTTTCAGCTGAATCAAGAAAGTTGCTTCTCATTGTTCAGGCACTT

AGGGACAACCTGGAACCTGGGACCTTCGATCTTGGGGGGCTATATGAAGCAATTGAGGAG

TGCCTGATTAACGATCCCTGGGTTTTGCTTAATGCGTCTTGGTTCAACTCCTTCCTCACA

CATGCACTGAAATAGTCGTGGCAATGCTACTA---TTTGCTATCCATACTGTCCAAAA--

----------------

>A_chicken_Iraq_1_2020_EPI1811627

---GAAAGCAGGTACTGATCCAAAATGGAAGACTTTGTGCGACAATGCTTCAATCCAATG

ATTGTCGAGCTTGCGGAAAAGGCAATGAAAGAATATGGGGAAGATCCTAAGATCGAAACA

AACAAGTTTGCCGCAATATGTACACACTTAGAAGTCTGCTTCATGTATTCGGATTTCCAT

TTCATTGATGAACGAGGCGAATCAATAATCGTAGAATCGGGCGACCCGAATGCATTATTG

AAGCACCGATTTGAGATAATTGAAGGGAGAGACCGAACAATGGCCTGGACAGTGGTGAAT

AGTATATGTAACACTACAGGAGTCGAAAAGCCCAAGTTCCTTCCTGATTTGTATGACTAC

AGAGAAAACCGATTCATTGAAATTGGAGTAACGCGAAGGGAAGTTCACATATACTATCTT

GAAAAAGCCAACAAGATAAAATCAGAGAAAACACACATTCACATATTCTCATTCACTGGG

GAGGAAATGGCCACCAAGGCAGATTACACTCTTGATGAAGAGAGCAGAGCAAGAATAAAA

ACCAGGCTATTCACTATAAGACAAGAGATGGCCAATAGGGGTCTATGGGATTCCTTTCGT

CAGTCCGAGAGAGGCGAAGAGACAATTGAAGAAAGATTTGAAATCACAGGAACCATGCGC

AGGCTTGCTGACCAAAGTCTCCCACCGAACTTCGCCAGCTTTGAAAACTTTAGAGCCTAT

GTGGATGGATTTGAACCGAACGGCTGCATTGAGGGCAAGCTTTCTCAAATGTCAAAAGAA

GTGAATGCCAGAATTGAGCCATTTCTGAAGACAACACCACGCCCTCTCAGATTACCTGAT

GGACCTCCCTGTTCCCAGCGGTCGAAGTTCTTGCTAATGGATGCCCTTAAATTGAGCATT

GAAGACCCGAGCCATGAGGGGGAGGGTATACCGCTGTATGATGCAACCAAATGCATGAAG

ACATTTTTTGGCTGGAAAGAGCCCAACATCGTGAAACCACATGAAAAGGGCATAAACCCT

AATTACCTCCTGGCTTGGAAGCAGGTGCTATCAGAACTCCAAGATATTGAAAACGAGGAG

AAAATCCCAAAAACAAAGAACATGAAGAAGACAAGCCAACTGAAGTGGGCACTTGGTGAA

AACATGGCACCAGAGAAAGTGGACTTTGAGGACTGTAAAGATGTTAGCGATCTAAGACAG

TACGACAGTGACGGACCAGAATCTAGATCACTAGCAAGTTGGATCCAGAGTGAATTTAAC

AAGGCTTGCGAACTGACAGATTCGAGTTGGATTGAACTTGATGAAATAGGGGAAGACGTT

GCTCCAATTGAACACATTGCGAGTATGAGGAGGAACTATTTCACAGCGGAAGTGTCCCAT

TGCAGGGCTACAGAATACATAATGAAAGGAGTATACATAAACACAGCTTTATTGAATGCA

TCCTGTGCAGCCATGGATGACTTCCAACTGATTCCAATGATAAGCAAATGCAGAACCAAA

GAAGGGAGACGGAAGACAAATTTGTATGGCTTCATTATAAAAGGAAGATCCCATTTGAGG

AATGACACCGATGTGGTAAACTTTGTGAGCATGGAGTTCTCTCTCACTGACCCAAGGCTG

GAGCCACACAAATGGGAAAAGTACTGTGTGCTTGAGGTAGGAGACATGCTCCTACGGACT

GCAATAGGCCAAGTGTCAAGGCCCATGTTCCTGTATGTGAGAACCAATGGGACTTCTAAG

ATCAAAATGAAATGGGGTATGGAGATGAGGCGATGCCTTCTTCAATCCCTTCAACAAATT

GAGAGCATGATTGAAGCCGAGTCTTCTGTCAAAGAAAAAGACATGACCAAGGAATTCTTT

GAAAACAGATCAGAAACATGGCCAATTGGAGAGTCACCCAAAGGTGTGGAGGAAGGCTCC

ATTGGGAAGGTGTGCAGAACATTGCTAGCAAAGTCTGTGTTCAACAGCCTATATGCATCT

CCACAACTCGAGGGGTTTTCAGCTGAATCAAGAAAGTTGCTTCTCATTGTTCAGGCACTT

AGGGACAACCTGGAACCTGGAACCTTCGATCTTGGGGGGCTATATGAAGCAATTGAGGAG

TGCCTGATTAACGATCCCTGGGTTTTGCTTAATGCGTCTTGGTTCAACTCCTTCCTCACA

CATGCACTGAAATAGTCGTGGCAATGCTACTA---TTTGCTATCCATACTGTCCAAAAAA

GTACCTTG--------

>A_whooper_swan_Inner_Mongolia_w1-1_2020_EPI1811643

------------------------ATGGAAGACTTTGTGCGACAATGCTTCAATCCAATG

ATTGTCGAGCTTGCGGAAAAGGCAATGAAAGAATATGGGGAAGATCCTAAGATCGAAACA

AACAAGTTTGCCGCAATATGTACACACTTAGAAGTCTGCTTCATGTATTCGGATTTCCAT

TTCATTGATGAACGAGGCGAATCAATAATCGTAGAATCTGGCGACCCGAATGCATTATTG

AAGCACCGATTTGAGATAATTGAAGGGAGAGACCGAACAATGGCCTGGACAGTGGTGAAT

AGTATATGCAACACTACAGGAGTCGAAAAGCCCAAGTTCCTTCCTGATTTGTATGACTAC

AGAGAAAACCGATTCATTGAAATTGGAGTAACGCGAAGGGAAGTTCACATATACTATCTT

GAAAAAGCCAACAAGATAAAATCAGAGAAAACACACATTCACATATTCTCATTCACTGGG

GAGGAAATGGCCACCAAGGCAGATTACACTCTTGATGAAGAGAGCAGAGCAAGAATAAAA

ACCAGGCTATTCACTATAAGACAAGAGATGGCCAATAGGGGTCTATGGGATTCCTTTCGT

CAGTCCGAGAGAGGCGAAGAGACAATTGAAGAAAGATTTGAAATCACAGGAACCATGCGC

AGGCTTGCTGACCAAAGTCTCCCACCGAACTTCGCCAGCCTTGAAAACTTTAGAGCCTAT

GTGGATGGATTTGAACCGAACGGCTGCATTGAGGGCAAGCTTTCTCAAATGTCAAAAGAA

GTGAATGCCAGAATTGAGCCATTTCTGAAGACAACACCACGCCCTCTCAGATTACCTGAT

GGACCTCCCTGTTCCCAGCGGTCGAAGTTCTTGCTAATGGATGCCCTTAAATTGAGCATT

GAAGACCCGAGCCATGAGGGGGAGGGTATACCGCTGTACGATGCAACCAAATGCATGAAG

ACATTTTTTGGCTGGAAGGAGCCCAACATCGTGAAACCACATGAAAAGGGCATAAACCCT

AATTACCTCCTGGCTTGGAAGCAGGTGCTATCAGAACTCCAAGATATTGAAAACGAGGAG

AAAATCCCAAAAACAAAGAACATGAAGAAGACGAGCCAACTGAAGTGGGCACTTGGTGAA

AACATGGCACCAGAGAAAGTGGACTTTGAGGACTGTAAAGATGTTAGCGATCTAAGACAG

TACGACAGTGACGAACCAGAATCTAGATCACTAGCAAGTTGGATCCAGAGTGAATTTAAC

AAGGCTTGCGAACTGACAGATTCGAGTTGGATTGAACTTGATGAAATAGGGGAAGACGTT

GCTCCGATTGAACACATTGCGAGTATGAGGAGGAACTATTTCACAGCGGAAGTATCCCAT

TGCAGGGCTACAGAATACATAATGAAAGGAGTATACATAAACACAGCTTTATTGAATGCA

TCCTGTGCAGCCATGGATGACTTCCAACTGATTCCAATGATAAGCAAATGCAGAACCAAA

GAAGGGAGACGGAAGACAAATCTGTATGGCTTCATTATAAAAGGAAGATCCCATTTGAGG

AATGACACCGATGTGGTAAACTTTGTGAGCATGGAATTCTCTCTCACTGACCCAAGGCTG

GAGCCACACAAATGGGAAAAGTACTGTGTGCTTGAGGTAGGAGACATGCTCCTACGGACT

GCAATAGGCCAAGTGTCAAGGCCCATGTTCCTGTATGTGAGAACCAATGGGACTTCTAAG

ATCAAAATGAAATGGGGTATGGAGATGAGGCGATGCCTTCTTCAATCCCTTCAACAAATT

GAGAGCATGATTGAAGCCGAGTCTTCTGTCAAAGAAAAAGACATGACCAAGGAATTCTTT

GAAAACAGATCAGAAACATGGCCAATTGGAGAGTCACCCAAAGGAGTGGAGGAAGGCTCC

ATTGGGAAGGTGTGCAGAACATTGCTAGCAAAGTCTGTGTTCAACAGCCTATATGCATCT

CCACAACTCGAGGGGTTTTCAGCTGAATCAAGAAAGTTGCTTCTCATTGTTCAGGCACTT

AGGGACAACCTGGAACCTGGGACCTTCGATCTTGGGGGGCTATATGAAGCAATTGAGGAG

TGCCTGATTAACGATCCCTGGGTTTTGCTTAATGCGTCTTGGTTCAACTCCTTCCTCACA

CATGCACTGAAATAG---------------------------------------------

----------------

>A_mute_swan_Inner_Mongolia_w2-1_2020_EPI1811651

------------------------ATGGAAGACTTTGTGCGACAATGCTTCAATACCATG

ATTGTCGAGCTTGCGGAAAAGGCAATGAAAGAATATGGGGAAGATCCTAAGATCGAAACA

AACAAGTTTGCCGCAATATGTACACACTTAGAAGTCTGCTTCATGTATTCGGATTTCCAT

TTCATTGATGAACGAGGCGAATCAATAATCGTAGAATCTGGCGACCCGAATGCATTATTG

AAGCACCGATTTGAGATAATTGAAGGGAGAGACCGAACAATGGCCTGGACAGTGGTGAAT

AGTATATGCAACACTACAGGAGTCGAAAAGCCCAAGTTCCTTCCTGATTTGTATGACTAC

AGAGAAAACCGATTCATTGAAATTGGAGTAACGCGAAGGGAAGTTCACATATACTATCTT

GAAAAAGCCAACAAGATAAAATCAGAGAAAACACACATTCACATATTCTCATTCACTGGG

GAGGAAATGGCCACCAAGGCAGATTACACTCTTGATGAAGAGAGCAGAGCAAGAATAAAA

ACCAGGCTATTCACTATAAGACAAGAGATGGCCAATAGGGGTCTATGGGATTCCTTTCGT

CAGTCCGAGAGAGGCGAAGAGACAATTGAAGAAAGATTTGAAATCACAGGAACCATGCGC

AGGCTTGCTGACCAAAGTCTCCCACCGAACTTCGCCAGCCTTGAAAACTTTAGAGCCTAT

GTGGATGGATTTGAACCGAACGGCTGCATTGAGGGCAAGCTTTCTCAAATGTCAAAAGAA

GTGAATGCCAGAATTGAGCCATTTCTGAAGACAACACCACGCCCTCTCAGATTACCTGAT

GGACCTCCCTGTTCCCAGCGGTCGAAGTTCTTGCTAATGGATGCCCTTAAATTGAGCATT

GAAGACCCGAGCCATGAGGGGGAGGGTATACCGCTGTACGATGCAACCAAATGCATGAAG

ACATTTTTTGGCTGGAAGGAGCCCAACATCGTGAAACCACATGAAAAGGGCATAAACCCT

AATTACCTCCTGGCTTGGAAGCAGGTGCTATCAGAACTCCAAGATATTGAAAACGAGGAG

AAAATCCCAAAAACAAAGAACATGAAGAAGACGAGCCAACTGAAGTGGGCACTTGGTGAA

AACATGGCACCAGAGAAAGTGGACTTTGAGGACTGTAAAGATGTTAGCGATCTAAGACAG

TACGACAGTGACGAACCAGAATCTAGATCACTAGCAAGTTGGATCCAGAGTGAATTTAAC

AAGGCTTGCGAACTGACAGATTCGAGTTGGATTGAACTTGATGAAATAGGGGAAGACGTT

GCTCCGATTGAACACATTGCGAGTATGAGGAGGAACTATTTCACAGCGGAAGTATCCCAT

TGCAGGGCTACAGAATACATAATGAAAGGAGTATACATAAACACAGCTTTATTGAATGCA

TCCTGTGCAGCCATGGATGACTTCCAACTGATTCCAATGATAAGCAAATGCAGAACCAAA

GAAGGGAGACGGAAGACAAATCTGTATGGCTTCATTATAAAAGGAAGATCCCATTTGAGG

AATGACACCGATGTGGTAAACTTTGTGAGCATGGAATTCTCTCTCACTGACCCAAGGCTG

GAGCCACACAAATGGGAAAAGTACTGTGTGCTTGAGGTAGGAGACATGCTCCTACGGACT

GCAATAGGCCAAGTGTCAAGGCCCATGTTCCTGTATGTGAGAACCAATGGGACTTCTAAG

ATCAAAATGAAATGGGGTATGGAGATGAGGCGATGCCTTCTTCAATCCCTTCAACAAATT

GAGAGCATGATTGAAGCCGAGTCTTCTGTCAAAGAAAAAGACATGACCAAGGAATTCTTT

GAAAACAGATCAGAAACATGGCCAATTGGAGAGTCACCCAAAGGAGTGGAGGAAGGCTCC

ATTGGGAAGGTGTGCAGAACATTGCTAGCAAAGTCTGTGTTCAACAGCCTATATGCATCT

CCACAACTCGAGGGGTTTTCAGCTGAATCAAGAAAGTTGCTTCTCATTGTTCAGGCACTT

AGGGACAACCTGGAACCTGGGACCTTCGATCTTGGGGGGCTATATGAAGCAATTGAGGAG

TGCCTGATTAACGATCCCTGGGTTTTGCTTAATGCGTCTTGGTTCAACTCCTTCCTCACA

CATGCACTGAAATAG---------------------------------------------

----------------

>A_goose_Russian_Federation_Kurgan_1345-25_2020_EPI1811684

------------TACTGATCCAAAATGGAAGACTTTGTGCGACAATGCTTCAATCCAATG

ATTGTCGAGCTTGCGGAAAAGGCAATGAAAGAATATGGGGAAGATCCTAAGATCGAAACA

AACAAGTTTGCCGCAATATGTACACACTTAGAAGTCTGCTTCATGTATTCGGATTTCCAT

TTCATTGATGAACGAGGCGAATCAATAATCGTAGAATCTGGCGACCCGAATGCATTATTG

AAGCACCGATTTGAGATAATTGAAGGGAGAGACCGAACAATGGCCTGGACAGTGGTGAAT

AGTATATGCAACACTACAGGAGTCGAAAAGCCCAAGTTCCTTCCTGATTTGTATGACTAC

AGAGAAAACCGATTCATTGAAATTGGAGTAACGCGAAGGGAAGTTCACATATACTATCTT

GAAAAAGCCAACAAGATAAAATCAGAGAAAACACACATTCACATATTCTCATTCACTGGG

GAGGAAATGGCCACCAAGGCAGATTACACTCTTGATGAAGAGAGCAGAGCAAGAATAAAA

ACCAGGCTATTCACTATAAGACAAGAGATGGCCAATAGGGGTCTATGGGATTCCTTTCGT

CAGTCCGAGAGAGGCGAAGAGACAATTGAAGAAAGATTTGAAATCACAGGAACCATGCGC

AGGCTTGCTGACCAGAGTCTCCCACCGAACTTCGCCAGCCTTGAAAACTTTAGAGCCTAT

GTGGATGGATTTGAACCGAACGGCTGCATTGAGGGCAAGCTTTCTCAAATGTCAAAAGAA

GTGAATGCCAGAATTGAGCCATTTCTGAAGACGACACCACGCCCTCTCAGATTACCTGAT

GGACCTCCCTGTTCCCAGCGGTCGAAGTTCTTGCTAATGGATGCCCTTAAATTGAGCATT

GAAGACCCGAGCCATGAGGGGGAGGGTATACCGCTGTACGATGCAACCAAATGCATGAAG

ACATTTTTTGGCTGGAAGGAGCCCAACATCGTGAAACCACATGAAAAGGGCATAAACCCT

AATTACCTCCTGGCTTGGAAGCAGGTGCTATCAGAACTCCAAGATATTGAAAACGAGGAG

AAAATCCCAAAAACAAAGAACATGAAGAAGACGAGCCAACTGAAGTGGGCACTTGGTGAA

AACATGGCACCAGAGAAAGTGGACTTTGAGGACTGTAAAGATGTTAGCGATCTAAGACAG

TACGACAGTGACGAACCAGAGTCTAGATCACTAGCAAGTTGGATCCAGAGTGAATTTAAC

AAGGCTTGCGAACTGACAGATTCGAGTTGGATTGAACTTGATGAAATAGGGGAAGACGTT

GCTCCAATTGAACACATTGCGAGTATGAGGAGGAACTATTTCACAGCGGAAGTATCCCAT

TGCAGGGCTACAGAATACATAATGAAAGGAGTATACATAAACACAGCTTTATTGAATGCA

TCCTGTGCAGCCATGGATGACTTCCAACTGATTCCAATGATAAGCAAATGCAGAACCAAA

GAAGGGAGACGGAAGACAAATCTGTATGGCTTCATTATAAAAGGAAGATCCCATTTGAGG

AATGACACCGATGTGGTAAACTTTGTGAGCATGGAATTCTCTCTCACTGACCCAAGGCTG

GAGCCACACAAATGGGAAAAGTACTGTGTGCTTGAGGTAGGAGACATGCTCCTACGGACT

GCAATAGGCCAAGTGTCAAGGCCCATGTTCCTGTATGTGAGAACCAATGGGACTTCTAAG

ATCAAAATGAAATGGGGTATGGAGATGAGGCGATGCCTTCTTCAATCCCTTCAACAAATT

GAGAGCATGATTGAAGCCGAGTCTTCTGTCAAAGAAAAAGACATGACCAAGGAATTCTTT

GAAAACAGATCAGAAACATGGCCAATTGGAGAGTCACCCAAAGGAGTGGAGGAAGGCTCC

ATTGGGAAGGTGTGCAGAACATTGCTAGCAAAGTCTGTGTTCAACAGCCTATATGCATCT

CCACAACTCGAGGGGTTTTCAGCTGAATCAAGAAAGTTGCTTCTCATTGTTCAGGCACTT

AGGGACAACCTGGAACCTGGAACCTTCGATCTTGGGGGGCTATATGAAGCAATTGAGGAG

TGCCTGATTAACGATCCCTGGGTTTTGCTTAATGCGTCTTGGTTCAACTCCTTCCTCACA

CATGCACTGAAATAGTCGTGGCAATGCTACTA---TTTGCTATCCATACTGTCCAAAAA-

GTA-------------

>A_duck_Chelyabinsk_1207-1_2020_EPI1812532

------------TACTGATCCAAAATGGAAGACTTTGTGCGACAATGCTTCAATCCAATG

ATTGTCGAGCTTGCGGAAAAGGCAATGAAAGAATATGGGGAAGATCCTAAGATCGAAACA

AACAAGTTTGCCGCAATATGTACACACTTAGAAGTCTGCTTCATGTATTCGGATTTCCAT

TTCATTGATGAACGAGGCGAATCAATAATCGTAGAATCTGGCGACCCGAATGCATTATTG

AAGCACCGATTTGAGATAATTGAAGGGAGAGACCGAACAATGGCCTGGACAGTGGTGAAT

AGTATATGCAACACTACAGGAGTCGAAAAGCCCAAGTTCCTTCCTGATTTGTATGACTAC

AGAGAAAACCGATTCATTGAAATTGGAGTAACGCGAAGGGAAGTTCACATATACTATCTT

GAAAAAGCCAACAAGATAAAATCAGAGAAAACACACATTCACATATTCTCATTCACTGGG

GAGGAAATGGCCACCAAGGCAGATTACACTCTTGATGAAGAGAGCAGAGCAAGAATAAAA

ACCAGGCTATTCACTATAAGACAAGAGATGGCCAATAGGGGTCTATGGGATTCCTTTCGT

CAGTCCGAGAGAGGCGAAGAGACAATTGAAGAAAGATTTGAAATCACAGGAACCATGCGC

AGGCTTGCTGACCAAAGTCTCCCACCGAACTTCGCCAGCCTTGAAAACTTTAGAGCCTAT

GTGGATGGATTTGAACCGAACGGCTGCATTGAGGGCAAGCTTTCTCAAATGTCAAAAGAA

GTGAATGCCAGAATTGAGCCATTTCTGAAGACAACACCACGCCCTCTCAGATTACCTGAT

GGACCTCCCTGTTCCCAGCGGTCGAAGTTCTTGCTAATGGATGCCCTTAAATTGAGCATT

GAAGACCCGAGCCATGAGGGGGAGGGTATACCGCTGTACGATGCAACCAAATGCATGAAG

ACATTTTTTGGCTGGAAGGAGCCCAACATCGTGAAACCACATGAAAAGGGCATAAACCCT

AATTACCTCCTTGCTTGGAAGCAGGTGCTATCAGAACTCCAAGATATTGAAAACGAGGAG

AAAATCCCAAAAACAAAGAACATGAAGAAGACGAGCCAACTGAAGTGGGCACTTGGTGAA

AACATGGCACCAGAGAAAGTGGACTTTGAGGACTGTAAAGATGTTAGCGATCTAAGACAG

TACGACAGTGACGAACCAGAATCTAGATCACTAGCAAGTTGGATCCAGAGTGAATTTAAC

AAGGCTTGCGAACTGACAGATTCGAGTTGGATTGAACTTGATGAAATAGGGGAAGACGTT

GCTCCAATTGAACACATTGCGAGTATGAGGAGGAACTATTTCACAGCGGAAGTATCCCAT

TGCAGGGCTACAGAATACATAATGAAAGGAGTATACATAAACACAGCTTTATTGAATGCA

TCCTGTGCAGCCATGGATGACTTCCAACTGATTCCAATGATAAGCAAATGCAGAACCAAA

GAAGGGAGACGGAAGACAAATCTGTATGGCTTCATTATAAAAGGAAGATCCCATTTGAGG

AATGACACCGATGTGGTAAACTTTGTGAGCATGGAATTCTCTCTCACTGACCCAAGGCTG

GAGCCACACAAATGGGAAAAGTACTGTGTGCTTGAGGTAGGAGACATGCTCCTACGGACT

GCAATAGGCCAAGTGTCAAGGCCCATGTTCCTGTATGTGAGAACCAATGGGACTTCTAAG

ATCAAAATGAAATGGGGTATGGAGATGAGGCGATGCCTTCTCCAATCCCTTCAACAAATT

GAGAGCATGATTGAAGCCGAGTCTTCTGTCAAAGAAAAAGACATGACCAAGGAATTCTTT

GAAAACAGATCAGAAACATGGCCAATTGGAGAGTCACCCAAAGGAGTGGAGGAAGGCTCC

ATTGGGAAGGTGTGCAGAACATTGCTAGCAAAGTCTGTGTTCAACAGCCTATATGCATCT

CCACAACTCGAGGGGTTTTCAGCTGAATCAAGAAAGTTGCTTCTCATTGTTCAGGCACTT

AGGGACAACCTGGAACCTGGAACCTTCGATCTTGGGGGGCTATATGAAGCAATTGAGGAG

TGCCTGATTAACGATCCCTGGGTTTTGCTTAATGCGTCTTGGTTCAACTCCTTCCTCACA

CATGCACTGAAATAGTCGTGGCAATGCTACTA---TTTGCTATCCATACTGTCCAAAAA-

GTA-------------

>A_goose_Omsk_0002_2020_EPI1813117

AGCAAAAGCAGGTACTGATCCAAAATGGAAGACTTTGTGCGACAATGCTTCAATCCAATG

ATTGTCGAGCTTGCGGAAAAGGCAATGAAAGAATATGGGGAAGATCCTAAGATCGAAACA

AACAAGTTTGCCGCAATATGTACACACTTAGAAGTCTGCTTCATGTATTCGGATTTCCAT

TTCATTGATGAACGAGGCGAATCAATAATCGTAGAATCTGGCGACCCGAATGCATTATTG

AAGCACCGATTTGAGATAATTGAAGGGAGAGACCGAACAATGGCCTGGACAGTGGTGAAT

AGTATATGCAACACTACAGGAGTCGAAAAGCCCAAGTTCCTTCCTGATTTGTATGACTAC

AGAGAAAACCGATTCATTGAAATTGGAGTAACGCGAAGGGAAGTTCACATATACTATCTT

GAAAAAGCCAACAAGATAAAATCAGAGAAAACACACATTCACATATTCTCATTCACTGGG

GAGGAAATGGCCACCAAGGCAGATTACACTCTTGATGAAGAGAGCAGAGCAAGAATAAAA

ACCAGGCTATTCACTATAAGACAAGAGATGGCCAATAGGGGTCTATGGGATTCCTTTCGT

AAGTCCGAGAGAGGCGAAGAGACAATTGAAGAAAGATTTGAAATCACAGGAACCATGCGC

AGGCTTGCTGACCAAAGTCTCCCACCGAACTTCGCCAGCCTTGAAAACTTTAGAGCCTAT

GTGGATGGATTTGAACCGAACGGCTGCATTGAGGGCAAGCTTTCTCAAATGTCAAAAGAA

GTGAATGCCAGAATTGAGCCATTTCTGAAGACAACACCACGCCCTCTCAGATTACCTGAT

GGACCTCCCTGTTCCCAGCGGTCGAAATTCTTGCTAATGGATGCCCTTAAATTGAGCATT

GAAGACCCGAGCCATGAGGGGGAGGGTATACCGCTGTACGATGCAACCAAATGCATGAAG

ACATTTTTTGGCTGGAAGGAGCCCAACATCGTGAAACCACATGAAAAGGGCATAAACCCT

AATTACCTCCTGGCTTGGAAGCAGGTGCTATCAGAACTCCAAGATATTGAAAACGAGGAG

AAAATCCCAAAAACAAAGAACATGAAGAAGACGAGCCAACTGAAGTGGGCACTTGGTGAA

AACATGGCACCAGAGAAAGTGGACTTTGAGGACTGTAAAGATGTTAGCGATCTAAAACAG

TACGACAGTGACGAACCAGAATCTAGATCACTAGCAAGTTGGATCCAGAGTGAATTTAAC

AAGGCTTGCGAACTGACAGATTCGAGTTGGATTGAACTTGATGAAATAGGGGAAGACGTT

GCTCCAATTGAACACATTGCGAGTATGAGGAGGAACTATTTCACAGCGGAAGTATCCCAT

TGCAGGGCTACAGAATACATAATGAAAGGAGTATACATAAACACAGCTTTATTGAATGCA

TCCTGTGCAGCCATGGATGACTTCCAACTGATTCCAATGATAAGCAAATGCAGAACCAAA

GAAGGGAGACGGAAGACAAATCTGTATGGCTTCATTATAAAAGGAAGATCCCATTTGAGG

AATGACACCGATGTGGTAAACTTTGTGAGCATGGAATTCTCTCTCACTGACCCAAGGCTG

GAGCCACACAAATGGGAAAAGTACTGTGTGCTTGAGGTAGGAGACATGCTCCTACGGACT

GCAATAGGCCAAGTGTCAAGGCCCATGTTCCTGTATGTGAGAACCAATGGGACTTCTAAG

ATCAAAATGAAATGGGGTATGGAGATGAGGCGATGCCTTCTTCAATCCCTTCAACAAATT

GAGAGCATGATTGAAGCCGAGTCTTCTGTCAAAGAAAAAGACATGACCAAGGAATTCTTT

GAAAACAGATCAGAAACATGGCCAATTGGAGAGTCACCCAAAGGAGTGGAGGAAGGCTCC

ATTGGGAAGGTGTGCAGAACATTGCTAGCAAAGTCTGTGTTCAACAGCCTATATGCATCT

CCACAACTCGAGGGGTTTTCAGCTGAATCAAGAAAGTTGCTTCTCATTGTTCAGGCACTT

AGGGACAACCTGGAACCTGGAACCTTCGATCTTGGGGGGCTATATGAAGCAATTGAGGAG

TGCCTGATTAACGATCCCTGGGTTTTGCTTAATGCGTCTTGGTTCAACTCCTTCCTCACA

CATGCACTGAAATAGTCGTGGCAATGCTACTA---TTTGCTATCCATACTGTCCAAAAAA

GTACCTTGTTTCTACT

>A_goose_Omsk_01171_2020_EPI1813197

AGCAAAAGCAGGTACTGATCCAAAATGGAAGACTTTGTGCGACAATGCTTCAATCCAATG

ATTGTCGAGCTTGCGGAAAAGGCAATGAAAGAATATGGGGAAGATCCTAAGATCGAAACA

AACAAGTTTGCCGCAATATGTACACACTTAGAAGTCTGCTTCATGTATTCGGATTTCCAT

TTCATTGATGAACGAGGCGAATCAATAATCGTAGAATCTGGCGACCCGAATGCATTATTG

AAGCACCGATTTGAGATAATTGAAGGGAGAGACCGAACAATGGCCTGGACAGTGGTGAAT

AGTATATGCAACACTACAGGAGTCGAAAAGCCCAAGTTCCTTCCTGATTTGTATGACTAC

AGAGAAAACCGATTCATTGAAATTGGAGTAACGCGAAGGGAAGTTCACATATACTATCTT

GAAAAAGCCAACAAGATAAAATCAGAGAAAACACACATTCACATATTCTCATTCACTGGG

GAGGAAATGGCCACCAAGGCAGATTACACTCTTGATGAAGAGAGCAGAGCAAGAATAAAA

ACCAGGCTATTCACTATAAGACAAGAGATGGCCAATAGGGGTCTATGGGATTCCTTTCGT

CAGTCCGAGAGAGGCGAAGAGACAATTGAAGAAAGATTTGAAATCACAGGAACCATGCGC

AGGCTTGCTGACCAAAGTCTCCCACCGAACTTCGCCAGCCTTGAAAACTTTAGAGCCTAT

GTGGATGGATTTGAACCGAACGGCTGCATTGAGGGCAAGCTTTCTCAAATGTCAAAAGAA

GTGAATGCCAGAATTGAGCCATTTCTGAAGACAACACCACGCCCTCTCAGATTACCTGAT

GGACCTCCCTGTTCCCAGCGGTCGAAGTTCTTGCTAATGGATGCCCTTAAATTGAGCATT

GAAGACCCGAGCCATGAGGGGGAGGGTATACCGCTGTACGATGCAACCAAATGCATGAAG

ACATTTTTTGGCTGGAAGGAGCCCAACATCGTGAAACCACATGAAAAGGGCATAAACCCT

AATTACCTCCTGGCTTGGAAGCAGGTGCTATCAGAACTCCAAGATATTGAAAACGAGGAG

AAAATCCCAAAAACAAAGAACATGAAGAAGACGAGCCAACTGAAGTGGGCACTTGGTGAA

AACATGGCACCAGAGAAAGTGGACTTTGAGGACTGTAAAGATGTTAGCGATCTAAGACAG

TACGACAGTGACGAACCAGAATCTAGATCACTAGCAAGTTGGATCCAGAGTGAATTTAAC

AAGGCTTGCGAACTGACAGATTCGAGTTGGATTGAACTTGATGAAATAGGGGAAGACGTT

GCTCCGATTGAACACATTGCGAGTATGAGGAGGAACTATTTCACAGCGGAAGTATCCCAT

TGCAGGGCTACAGAATACATAATGAAAGGAGTATACATAAACACAGCTTTATTGAATGCA

TCCTGTGCAGCCATGGATGACTTCCAACTGATTCCAATGATAAGCAAATGCAGAACCAAA

GAAGGGAGACGGAAGACAAATCTGTATGGCTTCATTATAAAAGGAAGATCCCATTTGAGG

AATGACACCGATGTGGTAAACTTTGTGAGCATGGAATTCTCTCTCACTGACCCAAGGCTG

GAGCCACACAAATGGGAAAAGTACTGTGTGCTTGAGGTAGGAGACATGCTCCTACGGACT

GCAATAGGCCAAGTGTCAAGGCCCATGTTCCTGTATGTGAGAACCAATGGGACTTCTAAG

ATCAAAATGAAATGGGGTATGGAGATGAGGCGATGCCTTCTTCAATCCCTTCAACAAATT

GAGAGCATGATTGAAGCCGAGTCTTCTGTCAAAGAAAAAGACATGACCAAGGAATTCTTT

GAAAACAGATCAGAAACATGGCCAATTGGAGAGTCACCCAAAGGAGTGGAGGAAGGCTCC

ATTGGGAAGGTGTGCAGAACATTGCTAGCAAAGTCTGTGTTCAACAGCCTATATGCATCT

CCACAACTCGAGGGGTTTTCAGCTGAATCAAGAAAGTTGCTTCTCATTGTTCAGGCACTT

AGGGACAACCTGGAACCTGGGACCTTCGATCTTGGGGGGCTATATGAAGCAATTGAGGAG

TGCCTGATTAACGATCCCTGGGTTTTGCTTAATGCGTCTTGGTTCAACTCCTTCCTCACA

CATGCACTGAAATAGTCGTGGCAATGCTACTA---TTTGCTATCCATACTGTCCAAAAAA

GTACCTTGTTTCTACT

>A_duck_Omsk_0075_2020_EPI1813277

AGCAAAAGCAGGTACTGATCCAAAATGGAAGACTTTGTGCGACAATGCTTCAATCCAATG

ATTGTCGAGCTTGCGGAAAAGGCAATGAAAGAATATGGGGAAGATCCTAAGATCGAAACA

AACAAGTTTGCCGCAATATGTACACACTTAGAAGTCTGCTTCATGTATTCGGATTTCCAT

TTCATTGATGAACGAGGCGAATCAATAATCGTAGAATCTGGCGACCCGAATGCATTATTG

AAGCACCGATTTGAGATAATTGAAGGGAGAGACCGAACAATGGCCTGGACAGTGGTGAAT

AGTATATGCAACACTACAGGAGTCGAAAAGCCCAAGTTCCTTCCTGATTTGTATGACTAC

AGAGAAAACCGATTCATTGAAATTGGAGTAACGCGAAGGGAAGTTCACATATACTATCTT

GAAAAAGCCAACAAGATAAAATCAGAGAAAACACACATTCACATATTCTCATTCACTGGG

GAGGAAATGGCCACCAAGGCAGATTACACTCTTGATGAAGAGAGCAGAGCAAGAATAAAA

ACCAGGCTATTCACTATAAGACAAGAGATGGCCAATAGGGGTCTATGGGATTCCTTTCGT

CAGTCCGAGAGAGGCGAAGAGACAATTGAAGAAAGATTTGAAATCACAGGAACCATGCGC

AGGCTTGCTGACCAAAGTCTCCCACCGAACTTCGCCAGCCTTGAAAACTTTAGAGCCTAT

GTGGATGGATTTGAACCGAACGGCTGCATTGAGGGCAAGCTTTCTCAAATGTCAAAAGAA

GTGAATGCCAGAATTGAGCCATTTCTGAAGACAACACCACGCCCTCTCAGATTACCTGAT

GGACCTCCCTGTTCCCAGCGGTCGAAGTTCTTGCTAATGGATGCCCTTAAATTGAGCATT

GAAGACCCGAGCCATGAGGGGGAGGGTATACCGCTGTACGATGCAACCAAATGCATGAAG

ACATTTTTTGGCTGGAAGGAGCCCAACATCGTGAAACCACATGAAAAGGGCATAAACCCT

AATTACCTCCTGGCTTGGAAGCAGGTGCTATCAGAACTCCAAGATATTGAAAACGAGGAG

AAAATCCCAAAAACAAAGAACATGAAGAAGACGAGCCAACTGAAGTGGGCACTTGGTGAA

AACATGGCACCAGAGAAAGTGGACTTTGAGGAATGTAAAGATGTTAGCGATCTAAGACAG

TACGACAGTGACGAACCAGAATCTAGATCACTAGCAAGTTGGATCCAGAGTGAATTTAAC

AAGGCTTGCGAACTGACAGATTCGAGTTGGATTGAACTTGATGAAATAGGGGAAGACGTT

GCTCCAATTGAACACATTGCGAGTATGAGGAGGAACTATTTCACAGCGGAAGTATCCCAT

TGCAGGGCTACAGAATACATAATGAAAGGAGTATACATAAACACAGCTTTATTGAATGCA

TCCTGTGCAGCCATGGATGACTTCCAACTGATTCCAATGATAAGCAAATGCAGAACCAAA

GAAGGGAGACGGAAGACAAATCTGTATGGCTTCATTATAAAAGGAAGATCCCATTTGAGG

AATGACACCGATGTGGTAAACTTTGTGAGCATGGAATTCTCTCTCACTGACCCAAGGCTG

GAGCCACACAAATGGGAAAAGTACTGTGTGCTTGAGGTAGGAGACATGCTCCTACGGACT

GCAATAGGCCAAGTGTCAAGGCCCATGTTCCTGTATGTGAGAACCAATGGGACTTCTAAG

ATCAAAATGAAATGGGGTATGGAGATGAGGCGATGCCTTCTTCAATCCCTTCAACAAATT

GAGAGCATGATTGAAGCCGAGTCTTCTGTCAAAGAAAAAGACATGACCAAGGAATTCTTT

GAAAACAGATCAGAAAAATGGCCAATTGGAGAGTCACCCAAAGGAGTGGAGGAAGGCTCC

ATTGGGAAGGTGTGCAGAACATTGCTAGCAAAGTCTGTGTTCAACAGCCTATATGCATCT

CCACAACTCGAGGGGTTTTCAGCTGAATCAAGAAAGTTGCTTCTCATTGTTCAGGCACTT

AGGGACAACCTGGAACCTGGAACCTTCGATCTTGGGGGGCTATATGAAGCAATTGAGGAG

TGCCTGATTAACGATCCCTGGGTTTTGCTTAATGCGTCTTGGTTCAACTCCTTCCTCACA

CATGCACTGAAATAGTCGTGGCAATGCTACTA---TTTGCTATCCATACTGTCCAAAAAA

GTACCTTGTTTCTACT

>A_duck_Omsk_0004_2020_EPI1813333

AGCAAAAGCAGGTACTGATCCAAAATGGAAGACTTTGTGCGACAATGCTTCAATCCAATG

ATTGTCGAGCTTGCGGAAAAGGCAATGAAAGAATATGGGGAAGATCCTAAGATCGAAACA

AACAAGTTTGCCGCAATATGTACACACTTAGAAGTCTGCTTCATGTATTCGGATTTCCAT

TTCATTGATGAACGAGGCGAATCAATAATCGTAGAATCTGGAGACCCGAATGCATTATTG

AAGCACCGATTTGAGATAATTGAAGGGAGAGACCGAACAATGGCCTGGACAGTGGTGAAT

AGTATATGCAACACTACAGGAGTCGAAAAGCCCAAGTTCCTTCCTGATTTGTATGACTAC

AGAGAAAACCGATTCATTGAAATTGGAGTAACGCGAAGGGAAGTTCACATATACTATCTT

GAAAAAGCCAACAAGATAAAATCAGAGAAAACACACATTCACATATTCTCATTCACTGGG

GAGGAAATGGCCACCAAGGCAGATTACACTCTTGATGAAGAGAGCAGAGCAAGAATAAAA

ACCAGGCTATTCACTATAAGACAAGAGATGGCCAATAGGGGTCTATGGGATTCCTTTCGT

CAGTCCGAGAGAGGCGAAGAGACAATTGAAGAAAGGTTTGAAATCACAGGAACAATGCGC

AGGCTTGCTGACCAAAGTCTCCCACCGAACTTCGCCAGCCTTGAAAACTTTAGAGCCTAT

GTGGATGGATTTGAACCGAACGGCTGCATTGAGGGCAAGCTTTCTCAAATGTCAAAAGAA

GTGAATGCCAGAATTGAGCCATTTCTGAAGACAACACCACGCCCTCTCAGATTACCTGAT

GGACCTCCCTGTTCTCAGCGGTCGAAGTTCTTGCTAATGGATGCCCTTAAATTGAGCATT

GAAGACCCGAGCCATGAGGGGGAGGGTATACCGCTGTACGATGCAACCAAATGCATGAAG

ACATTTTTTGGCTGGAAGGAGCCCAACATCGTGAAACCACATGAAAAGGGCATAAACCCT

AATTACCTCCTGACTTGGAAGCAGGTGCTATCAGAACTCCAAGATATTGAAAACGAGGAG

AAAATCCCAAAAACAAAGAACATGAAGAAGACGAGCCAACTGAAGTGGGCACTTGGTGAA

AACATGGCACCAGAGAAAGTGGACTTTGAGGACTGTAAAGATGTTAGCGATCTAAGACAG

TACGACAGTGACGAACCAGAATCTAGATCACTAGCAAGTTGGATCCAGAGTGAATTTAAC

AAGGCTTGCGAACTGACAGATTCGAGTTGGATTGAACTTGATGAAATAGGGGAAGACGTT

GCTCCAATTGAACACATTGCGAGTATGAGGAGGAACTATTTCACAGCGGAAGTATCCCAT

TGCAGGGCTACAGAATACATAATGAAAGGAGTATACATAAACACAGCTTTATTGAATGCA

TCCTGTGCAGCCATGGATGACTTCCAACTGATTCCAATGATAAGCAAATGCAGAACCAAA

GAAGGGAGACGGAAGACAAATCTGTATGGCTTCATTATAAAAGGAAGATCCCATTTGAGG

AATGACACCGATGTGGTAAACTTTGTGAGCATGGAATTCTCTCTCACTGACCCAAGGCTG

GAGCCACACAAATGGGAAAAGTACTGTGTGCTTGAGGTAGGAGACATGCTCCTACGGACT

GCAATAGGCCAAGTGTCAAGGCCCATGTTCCTGTATGTGAGAACCAATGGGACTTCTAAG

ATCAAAATGAAATGGGGTATGGAGATGAGGCGATGCCTTCTTCAATCCCTTCAACAAATT

GAGAGCATGATTGAAGCCGAGTCTTCTGTCAAAGAAAAAGACATGACCAAGGAATTCTTT

GAAAACAGATCAGAAACATGGCCAATTGGAGAGTCACCCAAAGGAGTGGAGGAAGGCTCC

ATTGGGAAGGTGTGCAGAACATTGCTAGCAAAGTCTGTGTTCAACAGCCTATATGCATCT

CCACAACTCGAGGGGTTTTCAGCTGAATCAAGAAAGTTGCTTCTCATTGTTCAGGCACTT

AGGGACAACCTGGAACCTGGAACCTTCGATCTTGGGGGGCTATATGAAGCAATTGAGGAG

TGCCTGATTAACGATCCCTGGGTTTTGCTTAATGCGTCTTGGTTCAACTCCTTCCTCACA

CATGCACTGAAATAGTCGTGGCAATGCTACTA---TTTGCTATCCATACTGTCCAAAAAA

GTACCTTGTTTCTACT

>A_chicken_Omsk_0112_2020_EPI1813341

AGCAAAAGCAGGTACTGATCCAAAATGGAAGACTTTGTGCGACAATGCTTCAATCCAATG

ATTGTCGAGCTTGCGGAAAAGGCAATGAAAGAATATGGGGAAGATCCGAAAATCGAAACA

AACAAATTTGCCGCAATATGCACACACTTAGAAGTCTGTTTCATGTATTCGGATTTCCAC

TTTATTGATGAACGGGGCGAATCAATAATTGTAGAATCTGGCGATCCGAATGCATTATTG

AAACACCGATTTGAGATAATTGAGGGGAGAGACCGAACTATGGCCTGGACGGTGGTGAAT

AGTATCTGCAACACCACAGGAGTCGAAAAGCCCAAATTCCTCCCTGATTTGTATGACTAC

AAAGAGAACCGATTCATTGAAATTGGAGTAACGCGAAGGGAAGTTCACATATACTATCTA

GAAAAAGCCAACAAGATAAAATCAGAGAAGACACACATTCACATATTCTCATTCACTGGA

GAGGAAATGGCCACCAAGGCTGACTACACCCTTGATGAAGAGAGCAGAGCAAGAATAAAA

ACCAGGCTGTTCACTATAAGACAAGAAATGGCCAGTAGGGGTCTATGGGATTCCTTTCGT

CAGTCCGAGAGAGGCGAAGAGACAATTGAAGAAAGATTTGAAATCACAGGAACCATGCGC

AGGCTTGCCGACCAAAGTCTCCCACCGAACTTCTCCAGCCTTGAAAACTTTAGAGCCTAT

GTGGATGGATTCGAACCGAACGGCTGCATTGAGGGCAAGCTTTCTCAAATGTCAAAAGAA

GTGAATGCCAGAATTGAGCCATTTCTGAAGACAACACCACGCCCTCTCAGATTACCTGAT

GGGCCTCCCTGTTCTCAGCGGTCGAAGTTTTTGCTGATGGATGCCCTCAAATTGAGCATC

GAAGACCCGAGCCATGAGGGGGAGGGTATACCGCTGTATGATGCAATCAAATGCATGAAG

ACATTTTTTGGCTGGAAAGAGCCCAACATCGTAAAACCACATGAAAAAGGCATAAACCCT

AATTACCTCCTGGCTTGGAAGCAGGTGCTGGCAGAACTCCAAGATATTGAAAATGAGGAG

AAAATCCCAAAAACAAAGAACATGAAGAAAACAAGCCAATTGAAGTGGGCACTTGGTGAG

AACATGGCACCAGAGAAAGTGGACTTTGAGGACTGTAAAGATGTTAGCGATCTAAGACAG

TACGACAGTGATGAGCCAGAGTCTAGATCACTAGCAAGCTGGATCCAGAGTGAGTTCAAC

AAGGCATGCGAATTGACAGATTCGAGTTGGATTGAACTTGATGAAATAGGGGAAGACATT

GCTCCAATTGAACACATTGCGAGCATGAGGAGGAACTATTTCACAGCGGAAGTGTCCCAT

TGCAGGGCTACTGAATACATAATGAAGGGAGTATACATAAACACAGCCCTATTGAATGCA

TCCTGTGCAGCCATGGATGACTTCCAACTGATTCCAATGATAAGCAAGTGCAGAACCAAA

GAAGGAAGACGAAAGACAAATCTGTATGGATTCATCATAAAGGGAAGATCCCATTTGAGG

AATGACACCGATGTGGTAAACTTTGTGAGCATGGAATTCTCTCTCACTGACCCGAGGCTG

GAGCCACACAAATGGGAAAAGTACTGTGTTCTTGAGATAGGAGACATGCTCCTACGGACT

GCAATAGGCCAAGTGTCAAGGCCCATGTTCCTGTATGTGAGAACAAACGGGACTTCCAAG

ATCAAAATGAAATGGGGCATGGAGATGAGGCGATGTCTTCTTCAGTCCCTTCAACAAATT

GAGAGCATGATTGAGGCCGAGTCTTCTGTCAAAGAGAAGGACATGACCAAGGAATTCTTT

GAAAATAAATCAGAAACATGGCCAATTGGGGAATCACCCAAAGGGGTGGAGGAAGGCTCC

ATTGGGAAGGTATGCAGAACATTGCTAGCAAAGTCTGTGTTCAACAGCCTATATGCATCT

CCACAACTCGAGGGGTTTTCAGCTGAATCAAGAAAATTGCTTCTCATTGTTCAGGCACTT

AGGGACAACCTGGAACCTGGAACTTTCGATCTTGGGGGGCTATATGAAGCAATTGAGGAG

TGCCTGATTAACGATCCCTGGGTTTTGCTTAATGCGTCTTGGTTCAACTCCTTCCTCACA

CATGCACTGAAATAGTTGTGGCAATGCTACTA---TTTGCTATCCATACTGTCCAAAAAA

GTACCTTGTTTCTACT

>A_duck_Saratov_29804_2020_EPI1814261

AGCAAAAGCAGGTACTGATCCAAAATGGAAGACTTTGTGCGACAATGCTTCAATCCAATG

ATTGTCGAGCTTGCGGAAAAGGCAATGAAAGAATATGGGGAAGATCCTAAGATCGAAACA

AACAAGTTTGCCGCAATATGTACACACTTAGAAGTCTGCTTCATGTATTCGGATTTCCAT

TTCATTGATGAACGAGGCGAATCAATAATCGTAGAATCTGGCGACCCGAATGCATTATTG

AAGCACCGATTTGAGATAATTGAAGGGAGAGACCGAACAATGGCCTGGACAGTGGTGAAT

AGTATATGCAACACTACAGGAGTCGAAAAGCCCAAGTTCCTTCCTGATTTGTATGACTAC

AGAGAAAACCGATTCATTGAAATTGGAGTAACGCGAAGGGAAGTTCACATATACTATCTT

GAAAAAGCCAACAAGATAAAATCAGAGAAAACACACATTCACATATTCTCATTCACTGGG

GAGGAAATGGCCACCAAGGCAGATTACACTCTTGATGAAGAGAGCAGAGCAAGAATAAAA

ACCAGGCTATTCACTATAAGACAAGAGATGGCCAATAGGGGTCTATGGGACTCCTTTCGT

CAGTCCGAGAGAGGCGAAGAAACAATTGAAGAAAGATTTGAAATCACAGGAACCATGCGC

AGGCTTGCTGACCAAAGTCTCCCACCGAACTTCGCCAGCCTTGAAAACTTTAGAGCCTAT

GTGGATGGATTTGAACCGAACGGCTGCATTGAGGGCAAGCTTTCTCAAATGTCAAAAGAA

GTGAATGCCAGAATTGAGCCATTTCTGAAGACAACACCACGCCCTCTCAGATTGCCTGAT

GGACCTCCCTGTTCCCAGCGGTCGAAGTTCTTGCTAATGGATGCCCTTAAATTGAGCATT

GAAGACCCGAGCCATGAGGGGGAGGGTATACCGCTGTACGATGCAACCAAATGCATGAAG

ACATTTTTTGGCTGGAAGGAGCCCAACATCGTGAAACCACATGAAAAGGGCATAAACCCT

AATTACCTCCTGGCTTGGAAGCAGGTGCTATCAGAACTCCAAGATATTGAAAACGAGGAG

AAAATCCCAAAGACAAAGAACATGAAGAAGACGAGCCAACTGAAGTGGGCACTTGGTGAA

AACATGGCACCAGAGAAAGTGGACTTTGAGGACTGTAAAGATGTTAGCGATCTAAAACAG

TACGACAGTGACGAACCAGAATCTAGATCACTAGCAAGTTGGATCCAGAGTGAATTTAAC

AAGGCTTGCGAACTGACAGATTCGAGTTGGATTGAACTTGATGAAATAGGGGAAGACGTT

GCTCCAATTGAACACATTGCGAGTATGAGGAGGAACTATTTCACAGCGGAAGTATCCCAT

TGCAGGGCTACAGAATACATAATGAAAGGAGTATACATAAACACAGCTTTATTGAATGCA

TCCTGTGCAGCCATGGATGACTTCCAACTGATTCCAATGATAAGCAAATGCAGAACCAAA

GAAGGGAGACGGAAGACAAATCTGTATGGCTTCATTATAAAAGGAAGATCCCATTTGAGG

AATGACACCGATGTGGTAAACTTTGTGAGCATGGAATTCTCTCTCACTGACCCAAGGCTG

GAGCCACACAAATGGGAAAAGTACTGTGTGCTTGAGGTAGGAGACATGCTCCTACGGACT

GCAATAGGCCAAGTGTCAAGGCCCATGTTCCTGTATGTGAGAACCAATGGGACTTCTAAG

ATCAAAATGAAATGGGGTATGGAGATGAGGCGATGCCTTCTTCAATCCCTTCAACAAATT

GAGAGCATGATTGAAGCCGAGTCTTCTGTCAAAGAAAAAGACATGACCAAGGAATTCTTT

GAAAACAGATCAGAAACATGGCCAATTGGAGAGTCACCCAAAGGAGTGGAGGAAGGCTCC

ATTGGGAAGGTGTGCAGAACATTGCTAGCAAAGTCTGTGTTCAACAGCCTATATGCATCT

CCACAACTCGAGGGGTTTTCAGCTGAATCAAGAAAGTTGCTTCTCATTGTTCAGGCACTT

AGGGACAACCTGGAACCTGGAACCTTCGATCTTGGGGGGCTATATGAAGCAATTGAGGAG

TGCCTGATTAACGATCCCTGGGTTTTGCTTAATGCGTCTTGGTTCAACTCCTTCCTCACA

CATGCACTGAAATAGTCGTGGCAATGCTACTA---TTTGCTATCCATACTGTCCAAAAAG

TACCTTGTTTCTACT-

>A_goose_Omsk_30001_2020_EPI1814277

AGCAAAAGCAGGTACTGATCCAAAATGGAAGACTTTGTGCGACAATGCTTCAATCCAATG

ATTGTCGAGCTTGCGGAAAAGGCAATGAAAGAATATGGGGAAGATCCTAAGATCGAAACA

AACAAGTTTGCCGCAATATGTACACACTTAGAAGTCTGCTTCATGTATTCGGATTTCCAT

TTCATTGATGAACGAGGCGAATCAATAATCGTAGAATCTGGCGACCCGAATGCATTATTG

AAGCACCGATTTGAGATAATTGAAGGGAGAGACCGAACAATGGCCTGGACAATGGTGAAT

AGTATATGCAACACTACAGGAGTCGAAAAGCCCAAGTTCCTTCCTGATTTGTATGACTAC

AGAGAAAACCGATTCATTGAAATTGGAGTAACGCGAAGGGAAGTTCACATATACTATCTT

GAAAAAGCCAACAAGATAAAATCAGAGAAAACACACATTCACATATTCTCATTCACTGGG

GAGGAAATGGCCACCAAGGCAGATTACACTCTTGATGAAGAGAGCAGAGCAAGGATAAAA

ACCAGGCTATTCACTATAAGACAAGAGATGGCCAATAGGGGTCTATGGGATTCCTTTCGT

CAGTCCGAGAGAGGCGAAGAGACAATTGAAGAAAGATTTGAAATCACAGGAACCATGCGC

AGGCTTGCTGACCAAAGTCTCCCACCGAATTTCGCCAGCCTTGAAAACTTTAGAGCCTAT

GTGGATGGATTTGAACCGAACGGCTGCATTGAGGGCAAGCTTTCTCAAATGTCAAAAGAA

GTGAATGCCAGAATTGAGCCATTTCTGAAGACAACACCACGCCCTCTCAGATTACCTGAT

GGACCTCCCTGTTCCCAGCGGTCGAAGTTCTTGCTAATGGATGCCCTTAAATTGAGCATT

GAAGACCCGAGCCATGAGGGGGAGGGTATACCGCTGTACGATGCAACCAAATGCATGAAG

ACATTTTTTGGCTGGAAGGAGCCCAACATCGTGAAACCACATGAAAAGGGCATAAACCCT

AATTACCTCCTGGCTTGGAAGCAGGTGCTATCAGAACTCCAAGATATTGAAAACGAGGAG

AAAATCCCAAAAACAAAGAACATGAAGAAGACGAGCCAACTGAAGTGGGCACTTGGTGAA

AACATGGCACCAGAGAAAGTGGACTTTGAGGACTGTAAAGATGTTAGCGATCTAAGACAG

TACGACAGTGACGAACCAGAATCTAGATCACTAGCAAGTTGGATCCAGAGTGAATTTAAC

AAGGCTTGCGAACTGACAGATTCGAGTTGGATTGAACTTGATGAAATAGGGGAAGACGTT

GCTCCAATTGAACACATTGCGAGTATGAGGAGGAACTATTTCACAGCGGAAGTATCCCAT

TGCAGGGCTACAGAATACATAATGAAAGGAGTATACATAAACACAGCTTTATTGAATGCA

TCCTGTGCAGCCATGGATGACTTCCAACTGATTCCAATGATAAGCAAATGCAGAACCAAA

GAAGGGAGACGGAAGACAAATCTGTATGGCTTCATTATAAAAGGAAGATCCCATTTGAGG

AATGACACCGATGTGGTAAACTTTGTGAGCATGGAATTCTCTCTCACTGACCCAAGGCTG

GAGCCACACAAATGGGAAAAGTACTGTGTGCTTGAGGTAGGAGACATGCTCCTACGGACT

GCAATAGGCCAAGTGTCAAGGCCCATGTTCCTGTATGTGAGAACCAATGGGACTTCTAAG

ATCAAAATGAAATGGGGTATGGAGATGAGGCGATGCCTTCTTCAATCCCTTCAACAAATT

GAGAGCATGATTGAAGCCGAGTCTTCTGTCAAAGAAAAAGACATGACCAAGGAATTCTTT

GAAAACAGATCAGAAACATGGCCAATTGGAGAGTCACCCAAAGGAGTGGAGGAAGGCTCC

ATTGGGAAGGTGTGCAGAACATTGCTAGCAAAGTCTGTATTCAACAGCCTATATGCATCT

CCACAACTCGAGGGGTTTTCAGCTGAATCAAGAAAGTTGCTTCTCATTGTTCAGGCACTT

AGGGACAACCTGGAACCTGGAACCTTCGATCTTGGGGGGCTATATGAAGCAATTGAGGAG

TGCCTGATTAACGATCCCTGGGTTTTGCTTAATGCGTCTTGGTTCAACTCCTTCCTCACA

CATGCACTGAAATAGTCGTGGCAATGCTACTA---TTTGCTATCCATACTGTCCAAAAAA

GTACCTTGTTTCTACT

>A_goose_Omsk_30003_2020_EPI1814285

AGCAAAAGCAGGTACTGATCCAAAATGGAAGACTTTGTGCGACAATGCTTCAATCCAATG

ATTGTCGAGCTTGCGGAAAAGGCAATGAAAGAATATGGGGAAGATCCTAAGATCGAAACA

AACAAGTTTGCCGCAATATGTACACACTTAGAAGTCTGCTTCATGTATTCGGATTTCCAT

TTCATTGATGAACGAGGCGAATCAATAATCGTAGAGTCTGGCGACCCGAATGCATTATTG

AAGCACCGATTTGAGATAATTGAAGGGAGAGACCGAACAATGGCCTGGACAGTGGTGAAT

AGTATATGCAACACTACAGGAGTCGAAAAGCCCAAGTTCCTTCCTGATTTGTATGACTAC

AGAGAAAACCGATTCATTGAAATTGGAGTAACGCGAAGGGAAGTTCACATATACTATCTT

GAAAAAGCCAACAAGATAAAATCAGAGAAAACACACATTCACATATTCTCATTCACTGGG

GAGGAAATGGCCACCAAGGCAGATTACACTCTTGATGAAGAGAGCAGAGCAAGAATAAAA

ACCAGGCTATTCACTATAAGACAAGAGATGGCCAATAGGGGTCTATGGGATTCCTTTCGT

CAGTCCGAGAGAGGCGAAGAGACAATTGAAGAAAGATTTGAAATCACAGGAACCATGCGC

AGGCTTGCTGACCAAAGCCTCCCACCGAACTTCGCCAGCCTTGAAAACTTTAGAGCCTAT

GTGGATGGATTTGAACCGAACGGCTGCATTGAGGGCAAGCTTTCTCAAATGTCAAAAGAA

GTGAATGCCAGAATTGAGCCATTTCTGAAGACAACACCACGCCCTCTCAGATTACCTGAT

GGACCTCCCTGTTCCCAGCGGTCGAAGTTCTTGCTAATGGATGCCCTTAAATTGAGCATT

GAAGACCCGAGCCATGAGGGGGAGGGTATACCGCTGTACGATGCAACCAAATGCATGAAG

ACATTTTTTGGCTGGAAGGAGCCCAACATCGTGAAACCACATGAAAAGGGCATAAACCCT

AATTACCTCCTGGCTTGGAAGCAGGTGCTATCAGAACTCCAAGATATTGAAAACGAGGAG

AAAATCCCAAAAACAAAGAACATGAAGAAGACGAGCCAACTGAAGTGGGCACTTGGTGAA

AACATGGCACCAGAGAAAGTGGACTTTGAGGACTGTAAAGATGTTAGCGATCTAAAACAG

TACGACAGTGACGAACCAGAATCTAGATCACTAGCAAGTTGGATCCAGAGTGAATTTAAC

AAGGCTTGCGAACTGACAGATTCGAGTTGGATTGAACTTGATGAAATAGGGGAAGACGTT

GCTCCAATTGAACACATTGCGAGTATGAGGAGGAACTATTTCACAGCGGAAGTATCCCAT

TGCAGGGCTACAGAATACATAATGAAAGGAGTATACATAAACACAGCTTTATTGAATGCA

TCCTGTGCAGCCATGGATGACTTCCAACTGATTCCAATGATAAGCAAATGCAGAACCAAA

GAAGGGAGACGGAAGACAAATCTGTATGGCTTCATTATAAAAGGAAGATCCCATTTGAGG

AATGACACCGATGTGGTAAACTTTGTGAGCATGGAATTCTCTCTCACTGACCCAAGGCTG

GAGCCACACAAATGGGAAAAGTACTGTGTGCTTGAGGTAGGAGACATGCTCCTACGGACT

GCAATAGGCCAAGTGTCAAGGCCCATGTTCCTGTATGTGAGAACCAATGGGACTTCTAAG

ATCAAAATGAAATGGGGTATGGAGATGAGGCGATGCCTTCTTCAATCCCTTCAACAGATT

GAGAGCATGATTGAAGCCGAGTCTTCTGTCAAAGAAAAAGACATGACCAAGGAATTCTTT

GAAAACAGATCAGAAACATGGCCAATTGGAGAGTCACCCAAAGGAGTGGAGGAAGGCTCC

ATTGGGAAGGTGTGCAGAACATTGCTAGCAAAGTCTGTGTTCAACAGCCTATATGCATCT

CCACAACTCGAGGGGTTTTCAGCTGAATCAAGAAAGTTGCTTCTCATTGTTCAGGCACTT

AGGGACAACCTGGAACCTGGAACCTTCGATCTTGGGGGGCTATATGAAGCAATTGAGGAG

TGCCTGATTAACGATCCCTGGGTTTTGCTTAATGCGTCTTGGTTCAACTCCTTCCTCACA

CATGCACTGAAATAGTCGTGGCAATGCTACTA---TTTGCTATCCATACTGTCCAAAAA-

GTACCTTGTTTCTACT

>A_swan_Tumen_1479-2_2020_EPI1814683

------------TACTGATCCAAAATGGAAGACTTTGTGCGACAATGCTTCAATCCAATG

ATTGTCGAGCTTGCGGAAAAGGCAATGAAAGAATATGGGGAAGATCCTAAGATCGAAACA

AACAAGTTTGCCGCAATATGTACACACTTAGAAGTCTGCTTCATGTATTCGGATTTCCAT

TTCATTGATGAACGAGGCGAATCAATAATCGTAGAATCTGGCGACCCGAATGCATTATTG

AAGCACCGATTTGAGATAATTGAAGGGAGAGACCGAACAATGGCCTGGACAGTGGTGAAT

AGTATATGCAACACTACAGGAGTCGAAAAGCCCAAGTTCCTTCCTGATTTGTATGACTAC

AGAGAAAACCGATTCATTGAAATTGGAGTAACGCGAAGGGAAGTTCACATATACTATCTT

GAAAAAGCCAACAAGATAAAATCAGAGAAAACACACATTCACATATTCTCATTCACTGGG

GAGGAAATGGCCACCAAGGCAGATTACACTCTTGATGAAGAGAGCAGAGCAAGAATAAAA

ACCAGGCTATTCACTATAAGACAAGAGATGGCCAATAGGGGTCTATGGGATTCCTTTCGT

CAGTCCGAGAGAGGCGAAGAGACAATTGAAGAAAGATTTGAAATCACAGGAACCATGCGC

AGGCTTGCTGACCAAAGTCTCCCACCGAACTTCGCCAGCCTTGAAAACTTTAGAGCCTAT

GTGGATGGATTTGAACCGAACGGCTGCATTGAGGGCAAGCTTTCTCAAATGTCAAAAGAA

GTGAATGCCAGAATTGAGCCATTTCTGAAGACAACACCACGCCCTCTCAGATTGCCTGAT

GGACCTCCCTGTTCCCAGCGGTCGAAGTTCTTGCTAATGGATGCCCTTAAATTGAGCATT

GAAGACCCGAGCCATGAGGGGGAGGGTATACCGCTGTACGATGCAACCAAATGCATGAAG

ACATTTTTTGGCTGGAAGGAGCCCAACATTGTGAAACCACATGAAAAGGGCATAAACCCT

AATTACCTCCTGGCTTGGAAGCAGGTGCTATCAGAACTCCAAGATATTGAAAACGAGGAG

AAAATCCCAAAAACAAAGAACATGAAGAAGACGAGCCAACTGAAGTGGGCACTTGGTGAA

AACATGGCACCAGAGAAAGTGGACTTTGAGGACTGTAAAGATGTTAGCGATCTAAAACAG

TACGACAGTGACGAACCAGAATCTAGATCATTAGCAAGTTGGATCCAGAGTGAATTTAAC

AAGGCTTGCGAACTGACAGATTCGAGTTGGATTGAACTTGATGAAATAGGGGAAGACGTT

GCTCCAATTGAACACATTGCGAGTATGAGGAGGAACTATTTCACAGCGGAAGTATCCCAT

TGCAGGGCTACAGAATACATAATGAAAGGAGTATACATAAACACAGCTTTATTGAATGCA

TCATGTGCAGCCATGGATGACTTCCAACTGATTCCAATGATAAGCAAATGCAGAACCAAA

GAAGGGAGACGGAAGACAAATCTGTATGGCTTCATTATAAAAGGAAGATCCCATTTGAGG

AATGACACCGATGTGGTAAACTTTGTGAGCATGGAATTCTCTCTCACTGACCCAAGGCTG

GAGCCACACAAATGGGAAAAGTACTGTGTGCTTGAGGTAGGAGACATGCTCCTACGGACT

GCAATAGGCCAAGTGTCAAGGCCCATGTTCCTGTATGTGAGAACCAATGGGACTTCTAAG

ATCAAAATGAAATGGGGTATAGAGATGAGGCGATGCCTTCTTCAATCCCTTCAACAAATT

GAGAGCATGATTGAAGCCGAGTCTTCTGTCAAAGAAAAAGACATGACCAAGGAATTCTTT

GAAAACAGATCAGAAACATGGCCAATTGGGGAGTCACCCAAAGGAGTGGAGGAAGGCTCC

ATTGGGAAGGTGTGCAGAACATTGTTAGCAAAGTCTGTGTTCAACAGCCTATATGCATCT

CCACAACTCGAGGGGTTTTCAGCTGAATCAAGAAAGTTGCTTCTCATTGTTCAGGCACTT

AGGGATAACCTGGAACCTGGAACCTTCGATCTTGGGGGGCTATATGAAGCAATTGAGGAG

TGCCTGATTAACGATCCCTGGGTTTTGCTTAATGCGTCTTGGTTCAACTCCTTCCTCACA

CATGCACTGAAATAGTCGTGGCAATGCTACTA---TTTGCTATCCATACTGTCCAAAAAG

TA--------------

>A_Whooper_swan_Mongolia_24_2020_EPI1831869

AGCGAAAGCAGGTACTGATTCAAAATGGAGGACTTTGTGCGACAATGCTTCAATCCAATG

ATCGTCGAGCTTGCGGAAAAGACAATGAAAGAATATGGGGAAAGTCCGAAAATCGAGACG

AACAAATTTGCCGCAATATGCACACACCTAGAAGTCTGCTTCATGTACTCGGACTTCCAC

TTTATAGATGAACGAGGCGAATCAATAATTGCAGAGTCTGGCGATCCGAACGCATTATTG

AAACACCGATTTGAGATAATTGAAGGGAGGGACCGAACAATGGCTTGGACAGTGGTAAAC

AGTATCTGCAACACCACAGGGGTCGATAAGCCTAAATTCCTCCCAGATTTATATGACTAC

AAAGAGAACCGGTTCATTGAAATTGGAGTGACACGGAGGGAAGTCCACATATATTACCTA

GAAAAAGCAAATAAGATAAAATCAGAGAGGACACACATACACATATTCTCATTCACTGGA

GAGGAAATGGCCACCAAAGCCGACTATACTCTTGATGAGGAGAGCAGAGCAAGAATTAAA

ACCCGGCTGTTCACTATAAGACAAGAAATGGCCAGTAGGGGCCTATGGGATTCCTTTCGT

CAATCCGAGAGAGGCGAAGAGACAATTGAAGAAAGGTTTGAAATCACAGGAACCATGCGC

AGGCTTGCTGACCAAAGCCTCCCACCGAACTTCTCCAGCCTTGAAAATTTTAGAGCCTAT

GTGGATGGATTCGAACCGAACGGCTGCATTGAGGGCAAGCTTTCTCAAATGTCAAAGGAA

GTGAACGCCAGAATTGAGCCATTTATGAAGACGACACCACGCCCTCTCAGACTACCTGAT

GGGCCTCCCTGCTCCCAGCGGTCGAAGTTCTTACTGATGGATGCCCTTAAACTAAGCATT

GAAGATCCGAGCCATGAGGGGGAAGGTATCCCATTATATGATGCAATCAAATGCATGAAG

ACGTTTTTCGGCTGGAAAGAGCCCAATATAGTAAAACCACATGACAAGGGCATAAATCCC

AATTACCTCCTGGCTTGGAAGCAGGTGCTGGCAGAACTCCAAGACATTGAAAATGAAGAT

AAAATCCCAAGAACAAAGAACATGAAGAAAACAAGCCAATTAAAGTGGGCACTTGGTGAG

AATATGGCACCTGAAAAAGTCGACTTTGAGGACTGCAAGGATGTTAGTGATCTAAAGCAG

TATGACAGTGATGAACCAGAGCCTAGATCGTTATCGAGCTGGGTTCAGAGCGAATTTAAC

AAGGCATGCGAATTGACAGATTCGAGTTGGATTGAACTTGATGAAATAGGAGAAGATGTT

GCTCCAATTGAGCACATTGCAAGTATGAGAAGAAACTATTTCACAGCGGAAGTGTCTCAT

TGCAGGGCTACTGAATACATAATGAAGGGAGTGTATATAAATACAGCCCTATTGAATGCA

TCCTGTGCAGCCATGGATGACTTCCAATTGATCCCAATGATAAGCAAGTGCAGAACCAGA

GAAGGAAGACGGAAGACAAATCTGTATGGGTTCATTATAAAAGGAAGATCCCATTTGAGG

AATGACACCGATGTGGTGAACTTTGTGAGCATGGAATTCTCTCTTACTGACCCGAGGCTA

GAACCTCACAAGTGGGTAAAGTACTGTGTTCTTGAAATAGGAGACATGCTCTTACGGACG

GCAATAGGTCAAGTGTCAAGACCCATGTTTTTATATGTGAGAACCAATGGGACTTCCAAG

ATTAAGATGAAATGGGGCATGGAGATGAGGAGATGCCTTCTTCAATCCCTTCAACAAATT

GAGAGCATGATTGAAGCCGAGTCCTCTGTCAAAGAGAAAGACATGACCAAAGAGTTCTTT

GAGACCAAATCAGAAACGTGGCCAATTGGGGAGTCACCCAAGGGGGTGGAGGAAAGCTCC

ATTGGGAAAGTGTGCAGAACACTACTAGCAAAATCTGTCTTCAACAGCCTATATGCCTCT

CCACAACTTGAGGGGTTTTCAGCTGAATCAAGAAAACTGCTTCTCATTGTTCAGGCACTT

AGGGACAACCTGGAACCTGGGACCTTCGATCTTGGGGGGCTATATGACGCAATTGAGGAG

TGCCTGATTAATGATCCCTGGGTTTTGCTTAATGCATCTTGGTTCAACTCCTTCCTCACA

CATGCACTGAAATAGTTGTGGCAATGCTACTA---TTCGCTATCCATACTGTCCAAAAAA

GTACCTTGTTTCTACT

>A_Whooper_swan_Mongolia_25_2020_EPI1831877

AGCGAAAGCAGGTACTGATTCAAAATGGAGGACTTTGTGCGACAATGCTTCAATCCAATG

ATCGTCGAGCTTGCGGAAAAGACAATGAAAGAATATGGGGAAAGTCCGAAAATCGAGACG

AACAAATTTGCCGCAGTATGCACACACCTAGAAGTCTGCTTCATGTACTCGGACTTCCAC

TTTATAGATGAACGAGGCGAATCAATAATTGCAGAGTCTGGCGATCCGAACGCATTATTG

AAACACCGATTTGAGATAATTGAAGGGAGGGACCGAACAATGGCTTGGACAGTGGTAAAC

AGTATCTGCAACACCACAGGGGTCGATAAGCCTAAATTCCTCCCAGATTTATATGACTAC

AAAGAGAACCGGTTCATTGAAATTGGAGTGACACGGAGGGAAGTCCACATATATTACCTA

GAAAAAGCAAATAAGATAAAATCAGAGAGGACACACATACACATATTCTCATTCACTGGA

GAGGAAATGGCCACCAAAGCCGACTATACTCTTGATGAGGAGAGCAGAGCAAGAATTAAA

ACCCGGCTGTTCACTATAAGACAAGAAATGGCCAGTAGGGGCCTATGGGATTCCTTTCGT

CAATCCGAGAGAGGCGAAGAGACAATTGAAGAAAGGTTTGAAATCACAGGAACCATGCGC

AGGCTTGCTGACCAAAGCCTCCCACCGAACTTCTCCAGCCTTGAAAATTTTAGAGCCTAT

GTGGATGGATTCGAACCGAACGGCTGCATTGAGGGCAAGCTTTCTCAAATGTCAAAGGAA

GTGAACGCCAGAATTGAGCCATTTATGAAGACGACACCACGCCCTCTCAGACTACCTGAT

GGGCCTCCCTGCTCCCAGCGGTCGAAGTTCTTACTGATGGATGCCCTTAAACTAAGCATT

GAAGATCCGAGCCATGAGGGGGAAGGTATCCCATTATATGATGCAATCAAATGCATGAAG

ACGTTTTTCGGCTGGAAAGAGCCCAATATAGTAAAACCACATGACAAGGGCATAAATCCC

AATTACCTCCTGGCTTGGAAGCAGGTGCTGGCAGAACTCCAAGACATTGAAAATGAAGAT

AAAATCCCAAGAACAAAGAACATGAAGAAAACAAGCCAATTAAAGTGGGCACTTGGTGAG

AATATGGCACCTGAAAAAGTCGACTTTGAGGACTGCAAGGATGTTAGTGATCTAAAGCAG

TATGACAGTGATGAACCAGAGCCTAGATCGTTATCGAGCTGGGTTCAGAGCGAATTTAAC

AAGGCATGCGAATTGACAGATTCGAGTTGGATTGAACTTGATGAAATAGGAGAAGATGTT

GCTCCAATTGAGCACATTGCAAGTATGAGAAGAAACTATTTCACAGCGGAAGTGTCTCAT

TGCAGGGCTACTGAATACATAATGAAGGGAGTGTATATAAATACAGCCCTATTGAATGCA

TCCTGTGCAGCCATGGATGACTTCCAATTGATCCCAATGATAAGCAAGTGCAGAACCAGA

GAAGGAAGACGGAAGACAAATCTGTATGGGTTCATTATAAAAGGAAGATCCCATTTGAGG

AATGACACCGATGTGGTGAACTTTGTGAGCATGGAATTCTCCCTTACTGACCCGAGGCTG

GAACCTCACAAGTGGGTAAAGTACTGTGTTCTTGAAATAGGAGACATGCTCTTACGGACG

GCAATAGGTCAAGTGTCAAGACCCATGTTTTTATATGTGAGAACCAATGGGACTTCCAAG

ATTAAGATGAAATGGGGCATGGAGATGAGGAGATGCCTTCTTCAATCCCTTCAACAAATT

GAGAGCATGATTGAAGCCGAGTCCTCTGTCAAAGAGAAAGACATGACCAAAGAGTTCTTT

GAGACCAAATCAGAAACGTGGCCAATTGGGGAGTCACCCAAGGGGGTGGAGGAAAGCTCC

ATTGGGAAAGTGTGCAGAACACTACTAGCAAAATCTGTCTTCAACAGCCTATATGCTTCT

CCACAACTTGAGGGGTTTTCAGCTGAATCAAGAAAACTGCTTCTCATTGTTCAGGCACTT

AGGGACAACCTGGAACCTGGGACCTTCGATCTTGGGGGGCTATATGACGCAATTGAGGAG

TGCCTGATTAATGATCCCTGGGTTTTGCTTAATGCATCTTGGTTCAACTCCTTCCTCACA

CATGCACTGAAATAGTTGTGGCAATGCTACTA---TTCGCTATCCATACTGTCCAAAAAA

GTACCTTGTTTCTACT

>A_chicken_Kazakhstan_Kn-3_2020_EPI1839257

------------------------ATGGAAGACTTTGTGCGACAATGCTTCAATCCAATG

ATTGTCGAGCTTGCGGAAAAGGCAATGAAAGAATATGGGGAAGATCCTAAGATCGAAACA

AACAAGTTTGCCGCAATATGTACACACTTAGAAGTCTGCTTCATGTATTCGGATTTCCAT

TTCATTGATGAACGAGGCGAATCAATAATCGTAGAATCTGGCGACCCGAATGCATTATTG

AAGCACCGATTTGAGATAATTGAAGGGAGAGACCGAACAATGGCCTGGACAGTGGTGAAT

AGTATATGCAACACTACAGGAGTCGAAAAGCCCAAGTTCCTTCCTGATTTGTATGACTAC

AGAGAAAACCGATTCATTGAAATTGGAGTAACGCGAAGGGAAGTTCACATATACTATCTT

GAAAAAGCCAACAAGATAAAATCAGAGAAAACACACATTCACATATTCTCATTCACTGGG

GAGGAAATGGCCACCAAGGCAGATTACACTCTTGATGAAGAGAGCAGAGCAAGAATAAAA

ACCAGGCTATTCACTATAAGACAAGAGATGGCCAATAGGGGTCTATGGGATTCCTTTCGT

CAGTCCGAGAGAGGCGAAGAGACAATTGAAGAAAGATTTGAAATCACAGGAACCATGCGC

AGGCTTGCTGACCAAAGTCTCCCACCGAACTTCGCCAGCCTTGAAAACTTTAGAGCCTAT

GTGGATGGATTTGAACCGAACGGCTGCATTGAGGGCAAGCTTTCTCAAATGTCAAAAGAA

GTGAATGCCAGAATTGAGCCATTTCTGAAGACAACACCACGCCCTCTCAGATTGCCTGAT

GGACCTCCCTGTTCCCAGCGGTCGAAGTTCTTGCTAATGGATGCCCTTAAATTGAGCATT

GAAGACCCGAGCCATGAGGGGGAGGGTATACCGCTGTACGATGCAACCAAATGCATGAAG

ACATTTTTTGGCTGGAAGGAGCCCAACATCGTGAAACCACATGAAAAGGGCATAAACCCT

AATTACCTCCTGGCTTGGAAGCAGGTGCTATCAGAACTCCAAGATATTGAAAACGAGGAG

AAAATCCCAAAAACAAAGAACATGAAGAAGACGAGCCAACTGAAGTGGGCACTTGGTGAA

AACATGGCACCAGAGAAAGTGGACTTTGAGGACTGTAAAGATGTTAGCGATCTAAAACAG

TACGACAGTGACGAACCAGAATCTAGATCACTAGCAAGTTGGATCCAGAGTGAATTTAAC

AAGGCTTGCGAACTGACAGATTCGAGTTGGATTGAACTTGATGAAATAGGGGAAGACGTT

GCTCCAATTGAACACATTGCGAGTATGAGGAGGAACTATTTCACAGCGGAAGTATCCCAT

TGCAGGGCTACAGAATACATAATGAAAGGAGTATACATAAACACAGCTTTATTGAATGCA

TCCTGTGCAGCCATGGATGACTTCCAACTGATTCCAATGATAAGCAAATGCAGAACCAAA

GAAGGGAGACGGAAGACAAATCTGTATGGCTTCATTATAAAAGGAAGATCCCATTTGAGG

AATGACACCGATGTGGTAAACTTTGTGAGCATGGAATTCTCTCTCACTGACCCAAGGCTG

GAGCCACACAAATGGGAAAAGTACTGTGTGCTTGAGGTAGGAGACATGCTCCTACGGACT

GCAATAGGCCAAGTGTCAAGGCCCATGTTCCTGTATGTGAGAACCAATGGGACTTCTAAG

ATCAAAATGAAATGGGGTATGGAGATGAGGCGATGCCTTCTTCAATCCCTTCAACAAATT

GAGAGCATGATTGAAGCCGAGTCTTCTGTCAAAGAAAAAGACATGACCAAGGAATTCTTT

GAAAACAGATCAGAAACATGGCCAATTGGAGAGTCACCCAAAGGAGTGGAGGAAGGCTCC

ATTGGGAAGGTGTGCAGAACATTGCTAGCAAAGTCTGTGTTCAACAGCCTATATGCATCT

CCACAACTCGAGGGGTTTTCAGCTGAATCAAGAAAGTTGCTTCTCATTGTTCAGGCACTT

AGGGACAACCTGGAACCTGGAACCTTCGATCTTGGGGGGCTATATGAAGCAATTGAGGAG

TGCCTGATTAACGATCCCTGGGTTTTGCTTAATGCGTCTTGGTTCAACTCCTTCCTCACA

CATGCACTGAAA------------------------------------------------

----------------

>A_chicken_Kazakhstan_Kn-6_2020_EPI1839265

------------------------ATGGAAGACTTTGTGCGACAATGCTTCAATCCAATG

ATTGTCGAGCTTGCGGAAAAGGCAATGAAAGAATATGGGGAAGATCCTAAGATCGAAACA

AACAAGTTTGCCGCAATATGTACACACTTAGAAGTCTGCTTCATGTATTCGGATTTCCAT

TTCATTGATGAACGAGGCGAATCAATAATCGTAGAATCTGGCGACCCGAATGCATTATTG

AAGCACCGATTTGAGATAATTGAAGGGAGAGACAGAACAATGGCCTGGACAGTGGTGAAT

AGTATATGCAACACTACAGGAGTCGAAAAGCCCAAGTTCCTTCCTGATTTGTATGACTAC

AGAGAAAACCGATTCATTGAAATTGGAGTAACGCGAAGGGAAGTTCACATATACTATCTT

GAAAAAGCCAACAAGATAAAATCAGAGAAAACACACATTCACATATTCTCATTCACTGGG

GAGGAAATGGCCACCAAGGCAGATTACACTCTTGATGAAGAGAGCAGAGCAAGAATAAAA

ACCAGGCTATTCACTATAAGACAAGAGATGGCCAATAGGGGTCTATGGGATTCCTTTCGT

CAGTCCGAGAGAGGCGAAGAGACAATTGAAGAAAGATTTGAAATCACAGGAACCATGCGC

AGGCTTGCTGACCAAAGTCTCCCACCGAACTTCGCCAGCCTTGAAAACTTTAGAGCCTAT

GTGGATGGATTTGAACCGAACGGCTGCATTGAGGGCAAGCTTTCTCAAATGTCAAAAGAA

GTGAATGCCAGAATTGAGCCATTTCTGAAGACAACACCACGCCCTCTCAGATTGCCTGAT

GGACCTCCCTGTTCCCAGCGGTCGAAGTTCTTGCTAATGGATGCCCTTAAATTGAGCATT

GAAGACCCGAGCCATGAGGGGGAGGGTATACCGCTGTACGATGCAACCAAATGCATGAAG

ACATTTTTTGGCTGGAAGGAGCCCAACATCGTGAAACCACATGAAAAGGGCATAAACCCT

AATTACCTCCTGGCTTGGAAGCAGGTGCTATCAGAACTCCAAGATATTGAAAACGAGGAG

AAAATCCCAAAAACAAAGAACATGAAGAAGACGAGCCAACTGAAGTGGGCACTTGGTGAA

AACATGGCACCAGAGAAAGTGGACTTTGAGGACTGTAAAGATGTTAGCGATCTAAAACAG

TACGACAGTGACGAACCAGAATCTAGATCACTAGCAAGTTGGATCCAGAGTGAATTTAAC

AAGGCTTGCGAACTGACAGATTCGAGTTGGATTGAACTTGATGAAATAGGGGAAGACGTT

GCTCCAATTGAACACATTGCGAGTATGAGGAGGAACTATTTCACAGCGGAAGTATCCCAT

TGCAGGGCTACAGAATACATAATGAAAGGAGTATACATAAACACAGCTTTATTGAATGCA

TCCTGTGCAGCCATGGATGACTTCCAACTGATTCCAATGATAAGCAAATGCAGAACCAAA

GAAGGGAGACGGAAGACAAATCTGTATGGCTTCATTATAAAAGGAAGATCCCATTTGAGG

AATGACACCGATGTGGTAAACTTTGTGAGCATGGAATTCTCTCTCACTGACCCAAGGCTG

GAGCCACACAAATGGGAAAAGTACTGTGTGCTTGAGGTAGGAGACATGCTCCTACGGACT

GCAATAGGCCAAGTGTCAAGGCCCATGTTCCTGTATGTGAGAACCAATGGGACTTCTAAG

ATCAAAATGAAATGGGGTATGGAGATGAGGCGATGCCTTCTTCAATCCCTTCAACAAATT

GAGAGCATGATTGAAGCCGAGTCTTCTGTCAAAGAAAAAGACATGACCAAGGAATTCTTT

GAAAACAGATCAGAAACATGGCCAATTGGAGAGTCACCCAAAGGAGTGGAGGAAGGCTCC

ATTGGGAAGGTGTGCAGAACATTGCTAGCAAAGTCTGTGTTCAACAGCCTATATGCATCT

CCACAACTCGAGGGGTTTTCAGCTGAATCAAGAAAGTTGCTTCTCATTGTTCAGGCACTT

AGGGACAACCTGGAACCTGGAACCTTCGATCTTGGGGGGCTATATGAAGCAATTGAGGAG

TGCCTGATTAACGATCCCTGGGTTTTGCTTAATGCGTCTTGGTTCAACTCCTTCCTCACA

CATGCACTGAAA------------------------------------------------

----------------

>A_Muscovy_duck_China_FJFZ21_H5N6_2020_EPI1841915

------------------------ATGGGGGCTTTTGTGCGACAATGCTTCAATCCAATG

ATCGTCGAGCTTGCGGAAAAGACAATGAAAGAATATGGGGAAAGCCCGAAAATCGAGACG

AACAAATTTGCCGCAATATGCACACACCTAGAAGTCTGCTTCATGTACTCGGACTTCCAC

TTTATAGATGAACGAGGCGAATCAATAATTGCAGAGTCTGGTGATCCGAACGCATTATTG

AAACACCGATTTGAGATAATTGAAGGGAGGGACCGAACAATGGCTTGGACAGTGGTAAAC

AGTATCTGCAACACCACAGGGGTCGATAAGCCTAAATTCCTCCCAGATTTATATGACTAC

AAAGAGAACCGGTTTATTGAAATTGGAGTGACACGGAGGGAAGTCCACATATACTACCTA

GAAAAAGCAAATAAGATAAAATCAGAGAGGACACACATCCACATATTCTCATTCACTGGA

GAGGAAATGGCCACCAAAGCTGACTATACTCTTGATGAGGAGAGCAGAGCAAGAATTAAA

ACCAGGCTGTTCACTATAAGACAAGAAATGGCCAGTAGGGGTCTATGGGATTCCTTTCGT

CAATCCGAGAGAGGCGAAGGAACAATTGAAGAAAGGTTTGAAATCACAGGAACCATGCGC

AGGCTTGCTGACCAAAGTCTCCCACCGAATTTCTCCAGCCTTGAAAATTTTAGAGCCTAT

GTGGATGGATTCGAACCGAACGGCTGCATTGAGGGCAAGCTTTCTCAAATGTCAAAGGAA

GTGAACGCTAGAATTGAGCCATTTATGAAGACAACACCACGTCCTCTTAGACTACCTGAT

GGACCTCCCTGCTTCCAGAGGTCGAAGTTCTTACTGATGGATGCCCTTAAACTAAGCATT

GAAGATCCGAGCCATGAGGGGGAAGGTATCCCATTATATGATGCAATCAAATGCATGAAG

ACATTTTTCGGATGGAAAGAGCCCAATATAGTAAAACCACATGACAAGGGCATAAATCCT

AATTACCTCCTGGCTTGGAAGCAGGTGCTGGCAGAACTCCAAGACATTGAAAATGGAGAT

AAAATCCCAAAAACAAAGAACCTGAAGAAAACAAGCCAATTAAAGTGGGCACTTGGTGAG

AATATGGCACCTGAAAAAATAGACTTTGAGGACTGCAAAGATGTTAGTGATCTAAAGCAG

TACGACAGTGATGAACCAGAGCCTAGATCGTTATCGAGCTGGGTTCAGAGCGAATTTAAC

AAGGCATGCGAATTGACAGATTCGAGTTGGATTGAACTTGATGAAATAGGAGAAGATGTT

GCTCCAATTGAGCACATTGCAAGTATGAGAAGAAACTATTTCACAGCGGAAGTGTCTCAT

TGCAGGGCTACTGAATACATAATGAAGGGAGTGTATATAAATACAGCCCTATTGAATGCA

TCCTGTGCAGCCATGGATGACTTCCAATTGATTCCAATGATAAGCAAGTGCAGAACCAGA

GAAGGAAGACGGAAGACAAATCTGTATGGGTTCATTATAAAAGGAAGATCCCATTTGAGG

AATGACACCGATGTGGTGAACTTTGTGAGCATGGAATTCTCTCTTACTGACCCGAGGCTG

GAACCGCACAAGTGGGTAAAGTACTGTGTTCTTGAAATAGGAGACATGCTCTTACGGACG

GCAATAGGTCAAGTGTCAAGACCCATGTTCTTGTATGTGAGAACCAATGGGACTTCCAAG

ATTAAGATGAAATGGGGCATGGAGATGAGGCGATGCCTTCTTCAATCCCTTCAACAAATT

GAGAGCATGATTGAAGCCGAGTCCTCTGTCAAAGAGAAAGACATGACCAAAGAGTTCTTT

GAGACCAAATCAGAAACGTGGCCAATTGGGGAATCACCTAAGGGGGTGGAGGAAAGCTCC

ATTGGGAAGGTGTGCAGAACACTACTAGCAAAATCTGTCTTCAACAGCCTATATGCTTCT

CCACAACTTGAGGGGTTTTCAGCTGAATCAAGAAAATTGCTTCTCATTGTTCAGGCACTT

AGGGACAACCTGGAACCTGGGACCTTCGATCTTGGGGGGCTATATGACGCAATTGAGGAG

TGCCTGATTAATGATCCCTGGGTTTTGCTTAATGCATCTTGGTTCAACTCCTTCCTCACA

CATGCACTGAAATAG---------------------------------------------

----------------

>A_mute_swan_Czech_Republic_1410-2_2021_EPI1843605

------------------------ATGGAAGACTTTGTGCGACAATGCTTCAATCCAATG

ATTGTCGAGCTTGCGGAAAAGGCAATGAAAGAATATGGGGAAGATCCTAAGATCGAAACA

AACAAGTTTGCCGCAATATGTACACACTTAGAAGTCTGCTTCATGTATTCGGATTTCCAT

TTCATTGATGAACGAGGCGAATCAATAATCGTAGAATCTGGCGACCCGAATGCATTATTG

AAGCACCGATTTGAGATAATTGAAGGGAGAGACCGAACAATGGCCTGGACAGTGGTGAAT

AGTATATGCAACACTACAGGAGTCGAAAAGCCCAAGTTCCTTCCTGATTTGTATGACTAC

AGAGAAAACCGATTCATTGAAATTGGAGTAACGCGAAGGGAAGTTCACATATACTATCTT

GAAAAAGCCAACAAGATAAAGTCAGAGAAAACACACATTCACATATTCTCATTCACTGGG

GAGGAAATGGCCACCAAGGCAGATTACACTCTTGATGAAGAGAGCAGAGCAAGAATAAAA

ACCAGGCTATTCACTATAAGACAAGAGATGGCCAATAGGGGTCTATGGGATTCCTTTCGT

CAGTCCGAGAGAGGCGAAGAGACAATTGAAGAAAGATTTGAAATCACAGGAACCATGCGC

AGGCTTGCTGACCAAAGTCTCCCACCGAACTTCGCCAGCCTTGAAAACTTTAGAGCCTAT

GTGGATGGATTTGAACCGAACGGCTGCATTGAGGGCAAGCTTTCTCAAATGTCAAAAGAA

GTGAATGCCAGAATTGAGCCATTTCTGAAGACAACACCACGCCCTCTCAGATTACCTGAT

GGACCTCCCTGTTCCCAGCGGTCGAAGTTCTTGCTAATGGATGCCCTTAAATTGAGCATT

GAAGACCCGAGCCATGAGGGGGAGGGTATACCGCTGTACGATGCAACCAAATGCATGAAG

ACATTTTTTGGCTGGAAGGAGCCCAACATCGTGAAACCACATGAAAAGGGCATAAACCCT

AATTACCTCCTGGCTTGGAAGCAGGTGCTATCAGAACTCCAAGATATTGAAAACGAGGAG

AAAATCCCTAAAACAAAGAACATGAAGAAGACGAGCCAACTGAAGTGGGCACTTGGTGAA

AACATGGCACCAGAGAAAGTGGACTTTGAGGACTGTAAAGATGTTAGCGATCTAAAACAG

TACGACAGTGACGAACCAGAATCTAGATCACTAGCAAGTTGGATCCAGAGTGAATTTAAC

AAGGCTTGCGAACTGACAGATTCGAGTTGGATTGAACTTGATGAAATAGGGGAAGACGTT

GCTCCAATTGAACACATTGCGAGTATGAGGAGGAACTATTTCACAGCGGAAGTATCCCAT

TGCAGGGCTACAGAATACATAATGAAAGGAGTATACATAAACACAGCTTTATTGAATGCA

TCCTGTGCAGCCATGGATGACTTCCAACTGATTCCAATGATAAGCAAATGCAGAACCAAA

GAAGGGAGACGGAAGACAAATCTGTATGGCTTCATTATAAAAGGAAGATCCCATTTGAGG

AATGACACCGATGTGGTAAACTTTGTGAGCATGGAATTCTCTCTCACTGACCCAAGGCTG

GAGCCACACAAATGGGAAAAGTACTGTGTGCTTGAGGTAGGAGACATGCTCCTACGGACT

GCAATAGGCCAAGTGTCAAGGCCCATGTTCCTGTATGTGAGAACCAATGGGACTTCTAAG

ATCAAAATGAAATGGGGTATGGAGATGAGGCGATGCCTTCTTCAATCCCTTCAACAAATT

GAGAGCATGATTGAAGCCGAGTCTTCTGTCAAAGAAAAAGACATGACCAAGGAATTCTTT

GAAAACAGATCAGAAACATGGCCAATTGGAGAGTCACCCAAAGGAGTGGAGGAAGGCTCC

ATTGGGAAGGTGTGCAGAACATTGCTAGCAAAGTCTGTGTTCAACAGCCTATATGCATCT

CCACAACTCGAGGGGTTTTCAGCTGAATCAAGAAAGTTGCTTCTCATTGTTCAGGCACTT

AGGGACAATCTGGAACCTGGAACCTTCGATCTTGGGGGGCTATATGAAGCAATTGAGGAG

TGCCTGATTAACGATCCCTGGGTTTTGCTTAATGCGTCTTGGTTCAACTCCTTCCTCACA

CATGCACTGAAATAGTCGTGGCAATGCTACTA---TTTGCTATCCATACTGTCCAAA---

----------------

>A_chicken_Czech_Republic_1566-1_2021_EPI1844082

------------------------ATGGAAGACTTTGTGCGACAATGCTTCAATCCAATG

ATTGTCGAGCTTGCGGAAAAGGCAATGAAAGAATATGGGGAAGATCCTAAGATCGAAACA

AACAAGTTTGCCGCAATATGTACGCACTTAGAAGTCTGCTTCATGTATTCGGATTTCCAT

TTCATTGATGAACGAGGCGAATCAATAATCGTAGAATCTGGCGACCCGAATGCATTATTG

AAGCACCGATTTGAGATAATTGAAGGGAGAGACCGAACAATGGCCTGGACAGTGGTGAAT

AGTATATGCAACACTACAGGAGTCGAAAAGCCCAAGTTCCTTCCTGATTTGTATGACTAC

AGAGAAAACCGATTCATTGAAATTGGAGTAACGCGAAGGGAAGTTCACATATACTATCTT

GAAAAAGCCAACAAGATAAAATCAGAGAAAACACACATTCACATATTCTCATTCACTGGG

GAGGAAATGGCCACCAAGGCAGATTACACTCTTGATGAAGAGAGCAGAGCAAGAATAAAA

ACCAGGCTATTCACTATAAGACAAGAGATGGCCAATAGGGGTCTATGGGATTCCTTTCGT

CAGTCCGAGAGAGGCGAAGAGACAATTGAAGAAAGATTTGAAATCACAGGAACCATGCGC

AGGCTTGCTGACCAAAGTCTCCCACCGAACTTCGCCAGCCTTGAAAACTTTAGAGCCTAT

GTGGATGGATTTGAACCGAACGGCTGCATTGAGGGCAAGCTTTCTCAAATGTCAAAAGAA

GTGAATGCCAGAATTGAGCCATTTCTGAAGACAACACCACGCCCTCTCAGATTACCTGAT

GGACCTCCCTGTTCCCAGCGGTCGAAGTTCTTGCTAATGGATGCCCTTAAATTGAGCATT

GAAGACCCGAGCCATGAGGGGGAGGGTATACCGCTGTACGATGCAACCAAATGCATGAAG

ACATTTTTTGGCTGGAAGGAGCCCAACATCGTGAAACCACATGAAAAGGGCATAAACCCT

AATTACCTCCTGGCTTGGAAGCAGGTGCTATCAGAACTCCAAGATATTGAAAACGAGGAG

AAAATCCCAAAAACAAAGAACATGAAGAAGACGAGCCAACTGAAGTGGGCACTTGGTGAA

AACATGGCACCAGAGAAAGTGGACTTTGAGGACTGTAAAGATGTTAGCGATCTAAGACAG

TACGACAGTGACGAACCAGAATCTAGATCACTAGCAAGTTGGATCCAGAGTGAATTTAAC

AAGGCTTGCGAACTGACAGATTCGAGTTGGATTGAACTTGATGAAATAGGGGAAGACGTT

GCTCCGATTGAACACATTGCGAGTATGAGGAGGAACTATTTCACAGCGGAAGTATCCCAT

TGCAGGGCTACAGAATACATAATGAAAGGAGTATACATAAACACAGCTTTATTGAATGCA

TCCTGTGCTGCCATGGATGACTTCCAACTGATTCCAATGATAAGCAAATGCAGAACCAAA

GAAGGAAGACGGAAGACAAATCTGTATGGCTTCATTATAAAAGGAAGATCCCATTTGAGG

AATGACACCGATGTGGTAAACTTTGTGAGCATGGAATTCTCTCTCACTGACCCAAGGCTG

GAGCCACACAAATGGGAAAAGTACTGTGTGCTTGAGGTAGGAGACATGCTCCTACGGACT

GCAATAGGCCAAGTGTCAAGGCCCATGTTCCTGTATGTGAGAACCAATGGGACTTCTAAG

ATCAAAATGAAATGGGGTATGGAGATGAGGCGATGCCTTCTTCAATCCCTTCAACAAATT

GAGAGCATGATTGAAGCCGAGTCTTCTGTCAAAGAAAAAGACATGACCAAGGAATTCTTT

GAAAACAGATCAGAAACATGGCCAATTGGAGAGTCACCCAAAGGAGTGGAGGAAGGCTCC

ATTGGGAAGGTGTGCAGAACATTGCTAGCAAAGTCTGTGTTCAACAGCCTATATGCATCT

CCACAACTCGAGGGGTTTTCAGCTGAATCAAGAAAGTTGCTTCTCATTGTTCAGGCACTT

AGGGACAACCTGGAACCTGGGACCTTCGATCTTGGGGGGCTATATGAAGCAATTGAGGAG

TGCCTGATTAACGATCCCTGGGTTTTGCTTAATGCGTCTTGGTTCAACTCCTTCCTCACA

CATGCACTGAAATAGTCGTGGCAATGCTACTA---TTTGCTATCCATACTGTCCAAA---

----------------

>A_chicken_Korea_H008_2021_EPI1846534

------------------------ATGGAAGACTTTGTGCGACAATGCTTCAATCCAATG

ATTGTCGAGCTTGCGGAAAAGGCAATGAAAGAATATGGGGAAGATCCTAAGATCGAAACA

AACAAGTTTGCCGCAATATGTACACACTTAGAAGTCTGCTTCATGTATTCGGATTTCCAT

TTCATTGATGAACGAGGCGAATCAATAATCGTAGAATCTGGCGACCCGAATGCATTATTG

AAGCACCGATTTGAGATAATTGAAGGGAGAGACCGAACAATGGCCTGGACAGTGGTGAAT

AGTATATGCAACACTACAGGAGTCGAAAAGCCCAAGTTCCTTCCTGATTTGTATGACTAC

AGAGAAAACCGATTCATTGAAATTGGAGTAACGCGAAGGGAAGTTCACATATACTATCTT

GAAAAAGCCAACAAGATAAAATCAGAGAAAACACACATTCACATATTCTCATTCACTGGG

GAGGAAATGGCCACCAAGGCAGATTACACTCTTGATGAAGAGAGCAGAGCAAGAATAAAA

ACCAGGCTATTCACTATAAGACAAGAGATGGCCAATAGGGGTCTATGGGATTCCTTTCGT

CAGTCCGAGAGAGGCGAAGAGACAATTGAAGAAAGATTTGAAATCACAGGAACCATGCGC

AGGCTTGCTGACCAGAGTCTCCCACCGAACTTCGCCAGCCTTGAAAACTTTAGAGCCTAT

GTGGATGGATTTGAACCGAACGGCTGCATTGAGGGCAAGCTTTCTCAAATGTCAAAAGAA

GTGAATGCCAGAATTGAGCCATTTCTGAAGACAACACCACGCCCTCTCAGATTACCTGAT

GGACCTCCCTGTTCCCAGCGGTCGAAGTTCTTGCTAATGGATGCCCTTAAATTGAGCATT

GAAGACCCGAGCCATGAGGGGGAGGGTATACCGCTGTACGATGCAACCAAATGCATGAAG

ACATTTTTTGGCTGGAAGGAGCCCAACATCGTGAAACCACATGAAAAGGGCATAAACCCT

AATTACCTCCTGGCTTGGAAGCAGGTGCTATCAGAACTCCAAGATATTGAAAACGAGGAG

AAAATCCCAAAAACAAAGAACATGAAGAAGACGAGCCAACTGAAGTGGGCACTTGGTGAA

AACATGGCACCAGAGAAAGTGGACTTTGAGGACTGTAAAGATGTTAGCGATCTAAGACAG

TACGACAGTGACGAACCAGAATCTAGATCACTAGCAAGTTGGATCCAGAGTGAATTTAAC

AAGGCTTGCGAACTGACAGATTCGAGTTGGATTGAACTTGATGAAATAGGGGAAGACGTT

GCTCCGATTGAACACATTGCGAGTATGAGGAGGAACTATTTCACAGCGGAAGTATCCCAT

TGCAGGGCTACAGAATACATAATGAAAGGAGTATACATAAACACAGCTTTATTGAATGCA

TCCTGTGCAGCCATGGATGACTTCCAACTGATTCCAATGATAAGCAAATGCAGAACCAAA

GAAGGGAGACGGAAGACAAATCTGTATGGCTTCATTATAAAAGGAAGATCCCATTTGAGG

AATGACACCGATGTGGTAAACTTTGTGAGCATGGAATTCTCTCTCACTGACCCAAGGCTG

GAGCCACACAAATGGGAAAAGTACTGTGTGCTTGAGGTAGGAGACATGCTCCTACGGACT

GCAATAGGCCAAGTGTCAAGGCCCATGTTCCTGTATGTGAGAACCAATGGGACTTCTAAG

ATCAAAATGAAATGGGGTATGGAGATGAGGCGATGCCTTCTTCAATCCCTTCAACAAATT

GAGAGCATGATTGAAGCCGAGTCTTCTGTCAAAGAAAAAGACATGACCAAGGAATTCTTT

GAAAACAGATCAGAAACATGGCCAATTGGAGAGTCACCCAAAGGAGTGGAGGAAGGCTCC

ATTGGGAAGGTGTGCAGAACATTGCTAGCAAAGTCTGTGTTCAACAGCCTATATGCATCT

CCACAACTCGAGGGGTTTTCAGCTGAATCAAGAAAGTTGCTTCTCATTGTTCAGGCACTT

AGGGACAACCTGGAACCTGGGACCTTCGATCTTGGGGGGCTATATGAAGCAATTGAGGAG

TGCCTGATTAACGATCCCTGGGTTTTGCTTAATGCGTCTTGGTTCAACTCCTTCCTCACA

CATGCACTGAAATAG---------------------------------------------

----------------

>A_mallard_Korea_WA820_2020_EPI1846598

------------------------ATGGAAGACTTTGTGCGACAATGCTTCAATCCAATG

ATTGTCGAGCTTGCGGAAAAGGCAATGAAAGAATATGGGGAAGATCCTAAGATCGAAACA

AACAAGTTTGCCGCAATATGTACACACTTAGAAGTCTGCTTCATGTATTCGGATTTCCAT

TTCATTGATGAACGAGGCGAATCAATAATCGTAGAATCTGGCGACCCGAATGCATTATTG

AAGCACCGATTTGAGATAATTGAAGGGAGAGACCGAACAATGGCCTGGACAGTGGTGAAT

AGTATATGCAACACTACAGGAGTCGAAAAGCCCAAGTTCCTTCCTGATTTGTATGACTAC

AGAGAAAACCGATTCATTGAAATTGGAGTAACGCGAAGGGAAGTTCACATATACTATCTT

GAAAAAGCCAACAAGATAAAATCAGAGAAAACACACATTCACATATTCTCATTCACTGGG

GAGGAAATGGCCACCAAGGCAGATTACACTCTTGATGAAGAGAGCAGAGCAAGAATAAAA

ACCAGGCTATTCACTATAAGACAAGAGATGGCCAATAGGGGTCTATGGGATTCCTTTCGT

CAGTCCGAGAGAGGCGAAGAGACAATTGAAGAAAGATTTGAAATCACAGGAACCATGCGC

AGGCTTGCTGACCAAAGTCTCCCACCGAACTTCGCCAGCCTTGAAAACTTTAGAGCCTAT

GTGGATGGATTTGAACCGAACGGCTGCATTGAGGGCAAGCTTTCTCAAATGTCAAAAGAA

GTGAATGCCAGAATTGAGCCATTTCTGAAGACAACACCACGCCCTCTCAGATTACCTGAT

GGACCTCCCTGTTCCCAGCGGTCGAAGTTCTTGCTAATGGATGCCCTTAAATTGAGCATT

GAAGACCCGAGCCATGAGGGGGAGGGTATACCGCTGTACGACGCAACCAAATGCATGAAG

ACATTTTTTGGCTGGAAGGAGCCCAACATCGTGAAACCACATGAAAAGGGCATAAACCCT

AATTACCTCCTGGCTTGGAAGCAGGTGCTATCAGAACTCCAAGATATTGAAAACGAGGAG

AAAATCCCAAAAACAAAGAACATGAAGAAGACGAGCCAACTGAAGTGGGCACTTGGTGAA

AACATGGCACCAGAGAAAGTGGACTTTGAGGACTGTAAAGATGTTAGCGATCTAAGACAG

TACGACAGTGACGAACCAGAATCTAGATCACTAGCAAGTTGGATCCAGAGTGAATTTAAC

AAGGCTTGCGAACTGACAGATTCGAGTTGGATTGAACTTGATGAAATAGGGGAAGACGTT

GCTCCGATTGAACACATTGCGAGTATGAGGAGGAACTATTTCACAGCGGAAGTATCCCAT

TGCAGGGCTACAGAATACATAATGAAAGGAGTATACATAAACACAGCTTTATTGAATGCA

TCCTGTGCGGCCATGGATGACTTCCAACTGATTCCAATGATAAGCAAATGCAGAACCAAA

GAAGGGAGACGGAAGACAAATCTGTATGGCTTCATTATAAAAGGAAGATCCCATTTGAGG

AATGACACCGATGTGGTAAACTTTGTGAGCATGGAATTCTCTCTCACTGACCCAAGGCTG

GAGCCACACAAATGGGAAAAGTACTGTGTGCTTGAGGTAGGAGACATGCTCCTACGGACT

GCAATAGGCCAAGTGTCAAGGCCCATGTTCCTGTATGTGAGAACCAATGGGACTTCTAAG

ATCAAAATGAAATGGGGTATGGAGATGAGGCGATGCCTTCTTCAATCCCTTCAACAAATT

GAGAGCATGATTGAAGCCGAGTCTTCTGTCAAAGAAAAAGACATGACCAAGGAATTCTTT

GAAAACAGATCAGAAACATGGCCAATTGGAGAGTCACCCAAAGGAGTGGAGGAAGGCTCC

ATTGGGAAGGTGTGCAGAACATTGCTAGCAAAGTCTGTGTTCAACAGCCTATATGCATCT

CCACAACTCGAGGGGTTTTCAGCTGAATCAAGAAAGTTGCTTCTCATTGTTCAGGCACTT

AGGGACAACCTGGAACCTGGGACCTTCGATCTTGGGGGGCTATATGAAGCAATTGAGGAG

TGCCTGATTAACGATCCCTGGGTTTTGCTTAATGCGTCTTGGTTCAACTCCTTCCTCACA

CATGCACTGAAATAG---------------------------------------------

----------------

>A_duck_Korea_H016_2021_EPI1846702

------------------------ATGGAAGACTTTGTGCGACAATGCTTCAATCCAATG

ATTGTCGAGCTTGCGGAAAAGGCAATGAAAGAATATGGGGAAGATCCTAAGATCGAAACA

AACAAGTTTGCCGCAATATGTACACACTTAGAAGTCTGCTTCATGTATTCGGATTTCCAT

TTCATTGATGAACGAGGCGAATCAATAATCGTAGAATCTGGCGACCCGAATGCATTATTG

AAGCACCGATTTGAGATAATTGAAGGGAGAGACCGAACAATGGCCTGGACAGTGGTGAAT

AGTATATGCAACACTACAGGAGTCGAAAAGCCCAAGTTCCTTCCTGATTTGTATGACTAC

AGAGAAAACCGATTCATTGAAATTGGAGTAACGCGAAGGGAAGTTCACATATACTATCTT

GAAAAAGCCAACAAGATAAAATCAGAGAAAACACACATTCACATATTCTCATTCACTGGG

GAGGAAATGGCCACCAAGGCAGATTACACTCTTGATGAAGAGAGCAGAGCAAGAATAAAA

ACCAGGCTATTCACTATAAGACAAGAGATGGCCAATAGGGGTCTATGGGATTCCTTTCGT

CAGTCCGAGAGAGGCGAAGAGACAATTGAAGAAAGATTTGAAATCACAGGAACCATGCGC

AGGCTTGCTGACCAAAGTCTCCCACCGAACTTCGCCAGCCTTGAAAACTTTAGAGCCTAT

GTGGATGGATTTGAACCGAACGGCTGCATTGAGGGCAAGCTTTCTCAAATGTCAAAAGAA

GTGAATGCCAGAATTGAGCCATTTCTGAAGACAACACCACGCCCTCTCAGATTACCTGAT

GGACCTCCCTGTTCCCAGCGGTCGAAGTTCTTGCTAATGGATGCCCTTAAATTGAGCATT

GAAGACCCGAGCCATGAGGGGGAGGGTATACCGCTGTACGACGCAACCAAATGCATGAAG

ACATTTTTTGGCTGGAAGGAGCCCAACATCGTGAAACCACATGAAAAGGGCATAAACCCT

AATTACCTCCTGGCTTGGAAGCAGGTGCTATCAGAACTCCAAGATATTGAAAACGAGGAG

AAAATCCCAAAAACAAAGAACATGAAGAAGACGAGCCAACTGAAGTGGGCACTTGGTGAA

AACATGGCACCAGAGAAAGTGGACTTTGAGGACTGTAAAGATGTTAGCGATCTAAGACAG

TACGACAGTGACGAACCAGAATCTAGATCACTAGCAAGTTGGATCCAGAGTGAATTTAAC

AAGGCTTGCGAACTGACAGATTCGAGTTGGATTGAACTTGATGAAATAGGGGAAGACGTT

GCTCCGATTGAACACATTGCGAGTATGAGGAGGAACTATTTCACAGCGGAAGTATCCCAT

TGCAGGGCTACAGAATACATAATGAAAGGAGTATACATAAACACAGCTTTATTGAATGCA

TCCTGTGCGGCCATGGATGACTTCCAACTGATTCCAATGATAAGCAAATGCAGAACCAAA

GAAGGGAGACGGAAGACAAATCTGTATGGCTTCATTATAAAAGGAAGATCCCATTTGAGG

AATGACACCGATGTGGTAAACTTTGTGAGCATGGAATTCTCTCTCACTGACCCAAGGCTG

GAGCCACACAAATGGGAAAAGTACTGTGTGCTTGAGGTAGGAGACATGCTCCTACGGACT

GCAATAGGCCAAGTGTCAAGGCCCATGTTCCTGTATGTGAGAACCAATGGGACTTCTAAG

ATCAAAATGAAATGGGGTATGGAGATGAGGCGATGCCTTCTTCAATCCCTTCAACAAATT

GAGAGCATGATTGAAGCCGAGTCTTCTGTCAAAGAAAAAGACATGACCAAGGAATTCTTT

GAAAACAGATCAGAAACATGGCCAATTGGAGAGTCACCCAAAGGAGTGGAGGAAGGCTCC

ATTGGGAAGGTGTGCAGAACATTGCTAGCAAAGTCTGTGTTCAACAGCCTATATGCATCT

CCACAACTCGAGGGGTTTTCAGCTGAATCAAGAAAGTTGCTTCTCATTGTTCAGGCACTT

AGGGACAACCTGGAACCTGGGACCTTCGATCTTGGGGGGCTATATGAAGCAATTGAGGAG

TGCCTGATTAACGATCCCTGGGTTTTGCTTAATGCGTCTTGGTTCAACTCCTTCCTCACA

CATGCACTGAAATAG---------------------------------------------

----------------

>A_chicken_Astrakhan_321-01_2020_EPI1846968

AGCAAAAGCAGGTACTGATCCAAAATGGAAGACTTTGTGCGACAATGCTTCAATCCAATG

ATTGTCGAGCTTGCGGAAAAGGCAATGAAAGAATATGGGGAAGATCCTAAGATCGAAACA

AACAAGTTTGCCGCAATATGTACACACTTAGAAGTCTGCTTCATGTATTCGGATTTCCAT

TTCATTGATGAACGAGGCGAATCAATAATCGTAGAATCTGGCGACCCGAATGCATTATTG

AAGCACCGATTTGAGATAATTGAAGGGAGAGACCGAACAATGGCCTGGACAGTGGTGAAT

AGTATATGCAACACTACAGGAGTCGAAAAGCCCAAGTTCCTTCCTGATTTGTATGACTAC

AGAGAAGACCGATTCATTGAAATTGGAGTAACGCGAAGGGAAGTTCACATATACTATCTT

GAAAAAGCCAACAAGATAAAATCAGAGAAAACACACATTCACATATTCTCATTCACTGGG

GAGGAAATGGCCACCAAGGCAGATTACACTCTTGATGAAGAGAGCAGAGCAAGAATAAAA

ACCAGGCTATTCACTATAAGACAAGAGATGGCCAATAGGGGTCTATGGGATTCCTTTCGT

CAGTCCGAGAGAGGCGAAGAGACAATTGAAGAAAGATTTGAAATCACAGGAACCATGCGC

AGGCTTGCTGACCAAAGTCTCCCACCGAACTTCGCCAGCCTTGAAAACTTTAGAGCCTAT

GTGGATGGATTTGAACCGAACGGCTGCATTGAGGGCAAGCTTTCTCAAATGTCAAAAGAA

GTGAATGCCAGAATTGAGCCATTTCTGAAGACAACACCACGCCCTCTCAGATTACCTGAT

GGACCTCCCTGTTCCCAGCGGTCGAAGTTCTTGCTAATGGATGCCCTTAAATTGAGCATT

GAAGACCCGAGCCATGAGGGGGAGGGTATACCGCTGTACGATGCAACCAAATGCATGAAG

ACATTTTTTGGCTGGAAGGAGCCCAACATCGTGAAACCACATGAAAAGGGCATAAACCCT

AATTACCTCCTGGCTTGGAAGCAGGTGCTATCAGAACTCCAAGATATTGAAAACGAGGAG

AAAATCCCAAAAACAAAGAACATGAAGAAGACGAGCCAACTGAAGTGGGCACTTGGTGAA

AACATGGCACCAGAGAAAGTGGACTTTGAGGACTGTAAAGATGTTAGCGATCTAAGACAG

TACGACAGTGACGAACCAGAATCTAGATCACTAGCAAGTTGGATCCAGAGTGAATTTAAC

AAGGCTTGCGAACTGACAGATTCGAGTTGGATTGAACTTGATGAAATAGGGGAAGACGTT

GCTCCGATTGAACACATTGCGAGTATGAGGAGGAACTATTTCACAGCGGAAGTATCCCAT

TGCAGGGCTACAGAATACATAATGAAAGGAGTATACATAAACACAGCTTTATTGAATGCA

TCCTGTGCAGCCATGGATGACTTCCAACTGATTCCAATGATAAGCAAATGCAGAACCAAA

GAAGGGAGACGGAAGACAAATCTGTATGGCTTCATTATAAAAGGAAGATCCCATTTGAGG

AATGACACCGATGTGGTAAACTTTGTGAGCATGGAATTCTCTCTCACTGACCCAAGGCTG

GAGCCACACAAATGGGAAAAGTACTGTGTGCTTGAGGTAGGAGACATGCTCCTACGGACT

GCAATAGGCCAAGTGTCAAGGCCCATGTTCCTGTATGTGAGAACCAATGGGACTTCTAAG

ATCAAAATGAAATGGGGTATGGAGATGAGGCGATGCCTTCTTCAATCCCTTCAACAAATT

GAGAGCATGATTGAAGCCGAGTCTTCTGTCAAAGAAAAAGACATGACCAAGGAATTCTTT

GAAAACAGATCAGAAACCTGGCCAATTGGAGAGTCACCCAAAGGAGTGGAGGAAGGCTCC

ATTGGGAAGGTGTGCAGAACATTGCTAGCAAAGTCTGTGTTCAACAGCCTATATGCATCT

CCACAACTCGAGGGGTTTTCAGCTGAATCAAGAAAGTTGCTTCTCATTGTTCAGGCACTT

AGGGACAACCTGGAACCTGGGACCTTCGATCTTGGGGGGCTATATGAAGCAATTGAGGAG

TGCCTGATTAACGATCCCTGGGTTTTGCTTAATGCGTCTTGGTTCAACTCCTTCCTCACA

CATGCACTGAAATAGTCGTGGCAATGCTACTA---TTTGCTATCCATACTGTCCAAAAAA

GTACCTTGTTTCTACT

>A_crane_Kagoshima_KU-93_2021_EPI1848526

------------------------ATGGAAGACTTTGTGCGACAATGCTTCAATCCAATG

ATTGTCGAGCTTGCGGAAAAGACAATGAAAGAATATGGGGAAGATCCTAAGATCGAAACA

AACAAGTTTGCCGCAATATGTACACACTTAGAAGTCTGCTTCATGTATTCGGATTTCCAT

TTCATTGATGAACGAGGAGAATCAATAATCGTAGAATCTGGCGACCCGAATGCATTATTG

AAGCACCGATTTGAGATAATTGAAGGGAGAGACCGAACAATGGCCTGGACAGTGGTGAAT

AGTATATGCAACACTACAGGAGCCGAAAAGCCCAAGTTCCTTCCTGATTTGTATGACTAC

AGAGAAAACCGATTCATTGAAATTGGAGTAACGCGAAGGGAAGTTCACATATACTATCTT

GAAAAAGCCAACAAGATAAAATCAGAGAAAACACACATTCACATATTCTCATTCACTGGG

GAGGAAATGGCCACCAAGGCAGATTACACTCTTGATGAAGAGAGCAGAGCAAGAATAAAA

ACCAGGCTATTCACTATAAGACAAGAGATGGCCAATAGGGGTCTATGGGATTCCTTTCGT

CAGTCCGAGAGAGGCGAAGAGACAATTGAAGAAAGATTTGAAATCACAGGAACCATGCGC

AGGCTTGCTGACCAGAGTCTCCCACCGAACTTCGCCAGCCTTGAAAACTTTAGAGCCTAT

GTGGATGGATTTGAACCGAACGGCTGCATTGAGGGCAAGCTTTCTCAAATGTCAAAAGAA

GTGAATGCCAGAATTGAGCCATTTCTGAAGACAACACCACGCCCTCTCAGATTACCTGAT

GGACCTCCCTGTTCCCAGCGGTCGAAGTTCTTGCTAATGGATGCCCTTAAATTGAGCATT

GAAGACCCGAGCCATGARGGGGAGGGTATACCGCTGTACGATGCAACCAAATGCATGAAG

ACATTTTTTGGCTGGAAGGAGCCCAACATCGTGAAACCACATGAAAAGGGCATAAACCCT

AATTACCTCCTGGCTTGGAAGCAGGTGCTATCAGAACTCCAAGATATTGAAAACGAGGAG

AAAATCCCAAAAACAAAGAACATGAAGAAGACGAGCCAACTGAAGTGGGCACTTGGTGAA

AACATGGCACCAGAGAAAGTGGACTTTGAGGACTGTAAAGATGTTAGCGATCTAAGACAG

TACGACAGTGACGAACCAGAATCTAGATCACTAGCAAGTTGGATCCAGAGTGAATTTAAC

AAGGCTTGCGAACTGACAGATTCGAGTTGGATTGAACTTGATGAAATAGGGGAAGACGTT

GCTCCGATTGAACACATTGCGAGTATGAGGAGGAACTATTTCACAGCGGAAGTATCCCAT

TGCAGGGCTACAGAATACATAATGAAAGGAGTATACATAAACACAGCTTTATTGAATGCA

TCCTGTGCAGCCATGGATGACTTCCAACTGATTCCAATGATAAGCAAATGYAGAACCAAA

GAAGGGAGACGGAAGACAAATCTGTATGGCTTCATTATAAAAGGAAGATCCCATTTGAGG

AATGACACCGATGTGGTAAACTTTGTGAGCATGGAATTCTCTCTCACTGACCCAAGGCTG

GAGCCACACAAATGGGAAAAGTACTGTGTGCTTGAGGTAGGAGACATGCTCCTACGGACT

GCAATAGGCCAAGTGTCAAGGCCCATGTTCCTGTATGTGAGGACCAATGGGACTTCTAAG

ATCAAAATGAAATGGGGTATGGAGATGAGGCGATGCCTTCTTCAATCCCTTCAACAAATT

GAGAGCATGATTGAAGCCGAGTCTTCTGTCAAAGAAAAAGACATGACCAAGGAATTCTTT

GAAAACAGATCAGAAACATGGCCAATTGGAGAGTCACCCAAAGGGGTGGAGGAAGGCTCC

ATTGGGAAGGTGTGCAGAACATTGCTAGCAAAGTCTGTGTTCAACAGCCTATATGCATCT

CCACAACTCGAGGGGTTTTCAGCTGAATCAAGAAAGTTGCTTCTCATTGTTCAGGCACTT

AGGGACAACCTGGAACCTGGGACCTTCGATCTTGGGGGGCTATATGAAGCAATTGAGGAG

TGCCTGATTAACGATCCCTGGGTTTTGCTTAATGCGTCTTGGTTCAACTCCTTCCTCACA

CATGCACGGAAATAG---------------------------------------------

----------------

>A_mallard_Kagoshima_KU-d89_2021_EPI1848537

------------------------ATGGAAGACTTTGTGCGACAATGCTTCAATCCAATG

ATTGTCGAGCTTGCGGAAAAGGCAATGAAAGAATATGGGGAAGATCCTAAGATCGAAACA

AACAAGTTTGCCGCAATATGTACACACTTAGAAGTCTGCTTCATGTATTCGGATTTCCAT

TTCATTGATGAACGAGGCGAATCAATAATCGTAGAATCTGGCGACCCGAATGCATTATTG

AAGCACCGATTTGAGATAATTGAAGGGAGAGACCGAACAATGGCCTGGACAGTGGTGAAT

AGTATATGCAACACTACAGGAGCCGAAAAGCCCAAGTTCCTTCCTGATTTGTATGACTAC

AGAGAAAACCGATTCATTGAAATTGGAGTAACGCGAAGGGAAGTTCACATATACTATCTT

GAAAAAGCCAACAAGATAAAATCAGAGAAAACACACATTCACATATTCTCATTCACTGGG

GAGGAAATGGCCACCAAGGCAGATTACACTCTTGATGAAGAGAGCAGAGCAAGAATAAAA

ACCAGGCTATTCACTATAAGACAAGAGATGGCCAATAGGGGTCTATGGGATTCCTTTCGT

CAGTCCGAGAGAGGCGAAGAGACAATTGAAGAAAGATTTGAAATCACAGGAACCATGCGC

AGGCTTGCTGACCAGAGTCTCCCACCGAACTTCGCCAGCCTTGAAAACTTTAGAGCCTAT

GTGGATGGATTTGAACCGAACGGCTGCATTGAGGGCAAGCTTTCTCAAATGTCAAAAGAA

GTGAATGCCAGAATTGAGCCATTTCTGAAGACAACACCACGCCCTCTCAGATTACCTGAT

GGACCTCCCTGTTCCCAGCGGTCGAAGTTCTTGCTAATGGATGCCCTTAAATTGAGCATT

GAAGACCCGAGCCATGAGGGGGAGGGTATACCGCTGTACGATGCAACCAAATGCATGAAG

ACATTTTTTGGCTGGAAGGAGCCCAACATCGTGAAACCACATGAAAAGGGCATAAACCCT

AATTACCTCCTGGCTTGGAAGCAGGTGCTATCAGAACTCCAAGATATTGAAAACGAGGAG

AAAATCCCAAAAACAAAGAACATGAAGAAGACGAGCCAACTGAAGTGGGCACTTGGTGAA

AACATGGCACCAGAGAAAGTGGACTTTGAGGACTGTAAAGATGTTAGCGATCTAAGACAG

TACGACAGTGACGAACCAGAATCTAGATCACTAGCAAGTTGGATCCAGAGTGAATTTAAC

AAGGCTTGCGAACTGACAGATTCGAGTTGGATTGAACTTGATGAAATAGGGGAAGACGTT

GCTCCGATTGAACACATTGCGAGTATGAGGAGGAACTATTTCACAGCGGAAGTATCCCAT

TGCAGGGCTACAGAATACATAATGAAAGGAGTATACATAAACACAGCTTTATTGAATGCA

TCCTGTGCAGCCATGGATGACTTCCAACTGATTCCAATGATAAGCAAATGCAGAACCAAA

GAAGGAAGACGGAAGACAAATCTGTATGGCTTCATTATAAAAGGAAGATCCCATTTGAGG

AATGACACCGATGTGGTAAACTTTGTGAGCATGGAATTCTCTCTCACTGACCCAAGGCTG

GAGCCACACAAATGGGAAAAGTACTGTGTGCTTGAGGTAGGAGACATGCTCCTACGGACT

GCAATAGGCCAAGTGTCAAGGCCCATGTTCCTGTATGTGAGAACCAATGGGACTTCTAAG

ATCAAAATGAAATGGGGTATGGAGATGAGGCGATGCCTTCTTCAATCCCTTCAACAAATT

GAGAGCATGATTGAAGCCGAGTCTTCTGTCAAAGAAAAAGACATGACCAAGGAATTCTTT

GAAAACAGATCAGAAACATGGCCAATCGGAGAGTCACCCAAAGGAGTGGAGGAAGGCTCC

ATTGGGAAGGTGTGCAGAACATTGCTAGCAAAGTCTGTGTTCAACAGCCTATATGCATCT

CCACAACTCGAGGGGTTTTCAGCTGAATCAAGAAAGTTGCTTCTCATTGTTCAGGCACTT

AGGGACAACCTGGAACCTGGGACCTTCGATCTTGGGGGGCTATATGAAGCAATTGAGGAG

TGCCTGATTAACGATCCCTGGGTTTTGCTTAATGCGTCTTGGTTCAACTCCTTCCTCACA

CATGCACTGAAATAG---------------------------------------------

----------------

>A_chicken_Kostroma_304-06_2020_EPI1848642

AGCAAAAGCAGGTACTGATCCAAAATGGAAGACTTTGTGCGACAATGCTTCAATCCAATG

ATTGTCGAGCTTGCGGAAAAGGCAATGAAAGAATATGGGGAAGATCCTAAGATCGAAACA

AACAAGTTTGCCGCAATATGTACACACTTAGAAGTCTGCTTCATGTATTCGGATTTCCAT

TTCATTGATGAACGAGGCGAATCAATAATCGTAGAATCTGGCGACCCGAATGCATTATTG

AAGCACCGATTTGAGATAATTGAAGGGAGAGACCGAACAATGGCCTGGACAGTGGTGAAT

AGTATATGCAACACTACAGGAATCGAAAAGCCCAAGTTCCTTCCTGATTTGTATGACTAC

AGAGAAAACCGATTCATTGAAATTGGAGTAACGCGAAGGGAAGTTCACATATACTATCTT

GAAAAAGCCAACAAGATAAAATCAGAGAAAACACACATTCACATATTCTCATTCACTGGG

GAGGAAATGGCCACCAAGGCAGATTACACTCTTGATGAAGAGAGCAGAGCAAGAATAAAA

ACCAGGCTATTCACTATAAGACAAGAGATGGCCAATAGGGGTCTATGGGATTCCTTTCGT

CAGTCCGAGAGAGGCGAAGAGACAATTGAAGAAAGATTTGAAATCACAGGAACCATGCGC

AGGCTTGCTGACCAAAGTCTCCCACCGAACTTCGCCAGCCTTGAAAACTTTAGAGCCTAT

GTGGATGGATTTGAACCGAACGGCTGCATTGAGGGCAAGCTTTCTCAAATGTCAAAAGAA

GTGAATGCCAGAATTGAGCCATTTCTGAAGACAACACCACGCCCTCTCAGATTACCTGAT

GGACCTCCCTGTTCCCAGCGGTCGAAGTTCTTGCTAATGGATGCCCTTAAATTGAGCATT

GAAGACCCGAGCCATGAGGGGGAGGGTATACCGCTGTACGATGCAACCAAATGCATGAAG

ACATTTTTTGGCTGGAAGGAGCCCAACATCGTGAAACCACATGAAAAGGGCATAAACCCT

AATTACCTCCTGGCTTGGAAGCAGGTGCTATCAGAACTCCAAGATATTGAAAACGAGGAG

AAAATCCCAAAAACAAAGAACATGAAGAAGACGAGCCAACTGAAGTGGGCACTTGGTGAA

AACATGGCACCAGAGAAAGTGGACTTTGAGGACTGTAAAGATGTTAGCGATCTAAGACAG

TACGACAGTGACGAACCAGAATCTAGATCACTAGCAAGTTGGATCCAGAGTGAATTTAAC

AAGGCTTGCGAACTGACAGATTCGAGTTGGATTGAACTTGATGAAATAGGGGAAGACGTT

GCTCCGATTGAACACATTGCGAGTATGAGGAGGAACTATTTCACAGCGGAAGTATCCCAT

TGCAGGGCTACAGAATACATAATGAAAGGAGTATACATAAACACAGCTTTATTGAATGCA

TCCTGTGCAGCCATGGATGACTTCCAACTGATTCCAATGATAAGCAAATGCAGAACCAAA

GAAGGGAGACGGAAGACAAATCTGTATGGCTTCATTATAAAAGGAAGATCCCATTTGAGG

AATGACACCGATGTGGTAAACTTTGTGAGCATGGAATTCTCTCTCACTGACCCAAGGCTG

GAGCCACACAAATGGGAAAAGTACTGTGTGCTTGAGGTAGGAGACATGCTCCTACGGACT

GCAATAGGCCAAGTGTCAAGGCCTATGTTCCTGTATGTGAGAACCAATGGGACTTCTAAG

ATCAAAATGAAATGGGGTATGGAGATGAGGCGATGCCTTCTTCAATCCCTTCAACAAATT

GAGAGCATGATTGAAGCCGAGTCTTCTGTCAAAGAAAAAGACATGACCAAGGAATTCTTT

GAAAACAGATCAGAAACATGGCCAATTGGAGAGTCACCCAAAGGAGTGGAGGAAGGCTCC

ATTGGGAAGGTGTGCAGAACATTGCTAGCAAAGTCTGTGTTCAACAGCCTATATGCATCT

CCACAACTCGAGGGGTTTTCAGCTGAATCAAGAAAGTTGCTTCTCATTGTTCAGGCACTT

AGGGACAACCTGGAACCTGGGACCTTCGATCTTGGGGGGCTATATGAAGCAATTGAGGAG

TGCCTGATTAACGATCCCTGGGTTTTGCTTAATGCGTCTTGGTTCAACTCCTTCCTCACA

CATGCACTGAAATAGTCGTGGCAATGCTACTA---TTTGCTATCCATACTGTCCAAAAA-

GTACCTTGTTTCTACT

>A_chicken_Rostov-on-Don_308-02_2020_EPI1848666

AGCAAAAGCAGGTACTGATCCAAAATGGAAGACTTTGTGCGACAATGCTTCAATCCAATG

ATTGTCGAGCTTGCGGAAAAGGCAATGAAAGAATATGGGGAAGATCCTAAGATCGAAACA

AACAAGTTTGCCGCAATATGTACACACTTAGAAGTCTGCTTCATGTATTCGGATTTCCAT

TTCATTGATGAACGAGGCGAATCAATAATCGTAGAATCTGGCGACCCGAATGCATTATTG

AAGCACCGATTTGAGATAATTGAAGGGAGAGACCGAACAATGGCCTGGACAGTGGTGAAT

AGTATATGCAACACTACAGGAGTCGAAAAGCCCAAGTTCCTTCCTGATTTGTATGACTAC

AGAGAAAACCGATTCATTGAAATTGGAGTAACGCGAAGGGAAGTTCACATATACTATCTT

GAAAAAGCCAACAAGATAAAATCAGAGAAAACACACATTCACATATTCTCATTCACTGGG

GAGGAAATGGCCACCAAGGCCGATTACACTCTTGATGAAGAGAGCAGAGCAAGAATAAAA

ACCAGGCTATTCACTATAAGACAAGAGATGGCCAATAGGGGTCTATGGGATTCCTTTCGT

CAGTCCGAGAGAGGCGAAGAGACAATTGAAGAAAGATTTGAAATCACAGGAACCATGCGC

AGGCTTGCTGACCAAAGTCTCCCACCGAACTTCGCAAGCCTTGAAAACTTTAGAGCCTAT

GTGGATGGATTTGAACCGAACGGCTGCATTGAGGGCAAGCTTTCTCAAATGTCAAAAGAA

GTGAATGCCAGAATTGAGCCATTTCTGAAGACGACACCACGCCCTCTCAGATTACCTGAT

GGACCTCCCTGTTCCCAGCGGTCGAAGTTCTTGCTAATGGATGCCCTTAAATTAAGCATT

GAAGACCCGAGCCATGAGGGGGAGGGTATACCGCTGTACGATGCAACCAAATGCATGAGG

ACATTTTTTGGCTGGAAGGAGCCCAACATCGTGAAACCACATGAAAAGGGCATAAACCCT

AATTACCTCCTGGCTTGGAAGCAGGTGCTATCAGAACTCCAAGATATTGAAAACGAGGAG

AAAATCCCAAAAACAAAGAACATGAAAAAGACGAGCCAACTGAAGTGGGCACTTGGTGAA

AACATGGCACCAGAGAAAGTGGACTTTGAGGACTGTAAAGATGTTAGCGATCTAAGACAG

TACGACAGTGACGAACCAGAGTCTAGATCACTAGCAAGTTGGATCCAGAGTGAATTTAAC

AAGGCTTGCGAACTGACAGATTCGAGTTGGATTGAACTTGATGAAATAGGGGAAGACGTT

GCTCCAATTGAACACATTGCGAGTATGAGGAGGAACTATTTCACAGCGGAAGTATCCCAT

TGCAGGGCTACAGAATACATAATGAAAGGAGTATACATAAACACAGCTTTATTGAATGCA

TCCTGTGCAGCCATGGATGACTTCCAACTGATTCCAATGATAAGCAAATGCAGAACCAAA

GAAGGGAGACGGAAGACAAATCTGTATGGCTTCATTATAAAAGGAAGATCCCATTTGAGG

AATGACACCGATGTGGTAAACTTTGTGAGCATGGAATTCTCTCTCACTGACCCAAGGCTG

GAGCCACACAAATGGGAAAAGTACTGTGTGCTTGAGGTAGGAGACATGCTCCTACGGACT

GCAATAGGCCAAGTGTCAAGGCCCATGTTCCTGTATGTGAGAACCAATGGGACTTCTAAG

ATCAAAATGAAATGGGGTATGGAGATGAGGCGATGCCTTCTTCAATCCCTTCAACAAATT

GAGAGCATGATTGAAGCCGAGTCTTCTGTCAAAGAAAAAGACATGACCAAGGAATTCTTT

GAAAACAGATCAGAAACATGGCCAATTGGAGAGTCACCCAAAGGAGTGGAGGAAGGCTCC

ATTGGGAAGGTGTGCAGAACATTGCTAGCAAAGTCTGTGTTCAACAGCCTATATGCATCT

CCACAACTCGAGGGGTTTTCAGCTGAATCAAGAAAGTTGCTTCTCATTGTTCAGGCACTT

AGGGACAACCTGGAACCTGGAACCTTCGATCTTGGGGGGCTATATGAAGCAATTGAGGAG

TGCCTGATTAACGATCCCTGGGTTTTGCTTAATGCGTCTTGGTTCAACTCCTTCCTCACA

CATGCACTGAAATAGTCGTGGCAATGCTACTA---TTTGCTATCCATACTGTCCAAAAA-

GTACCTTGTTTCTACT

>A_turkey_Stavropol_320-02_2020_EPI1848698

AGCAAAAGCAGGTACTGATCCAAAATGGAAGACTTTGTGCGACAATGCTTCAATCCAATG

ATTGTCGAGCTTGCGGAAAAGGCAATGAAAGAGTATGGGGAAGATCCTAAGATCGAAACA

AACAAGTTTGCCGCAATATGTACACACTTAGAAGTCTGCTTCATGTATTCGGATTTCCAT

TTCATTGATGAACGAGGCGAATCAATAATCGTAGAATCTGGCGACCCGAATGCATTATTG

AAGCACCGATTTGAGATCATTGAAGGGAGAGACCGAACAATGGCCTGGACAGTGGTGAAT

AGCATATGCAACACTACAGGAGTCGAAAAGCCCAAGTTCCTTCCTGATTTGTATGACTAC

AGAGAAAACCGATTCATTGAAATTGGAGTAACGCGAAGGGAAGTTCACATATACTATCTT

GAAAAAGCCAACAAGATAAAATCAGAGAAAACACACATTCACATATTCTCATTCACTGGG

GAGGAAATGGCCACCAAGGCAGATTACACTCTTGATGAAGAGAGCAGAGCAAGAATAAAA

ACCAGGCTATTCACTATAAGACAAGAGATGGCCAATAGGGGTCTATGGGATTCCTTTCGT

CAGTCCGAGAGAGGCGAAGAGACAATTGAAGAAAGATTTGAAATCACAGGAACCATGCGC

AGGCTTGCTGACCAGAGTCTCCCACCGAACTTCGCCAGCCTTGAAAACTTTAGAGCCTAT

GTGGATGGATTTGAACCGAACGGCTGCATTGAGGGCAAGCTTTCTCAAATGTCAAAAGAA

GTGAATGCCAGAATTGAGCCATTTCTGAAGACGACACCACGCCCTCTCAGATTACCTGAT

GGACCTCCCTGTTCCCAGCGGTCGAAGTTCTTGCTAATGGATGCCCTTAAATTGAGCATT

GAAGACCCGAGCCATGAGGGGGAGGGTATACCGCTGTATGATGCAACCAAATGCATGAAG

ACATTTTTTGGCTGGAAGGAGCCCAACATCGTGAAACCACATGAAAAGGGCATAAACCCT

AATTACCTCCTGGCTTGGAAGCAGGTGCTATCAGAACTCCAAGATATTGAAAACGAGGAG

AAAATCCCAAAAACAAAGAACATGAAGAAGACGAGCCAACTGAAGTGGGCACTTGGTGAA

AACATGGCACCAGAGAAAGTGGACTTTGAGGACTGTAAAGATGTTAGCGATCTAAGACAG

TACGACAGTGACGAACCAGAGTCTAGATCACTAGCAAGTTGGATCCAGAGTGAATTTAAC

AAGGCTTGCGAACTGACAGATTCGAGTTGGATTGAACTTGATGAAATAGGGGAAGACGTT

GCTCCAATTGAACACATTGCGAGTATGAGGAGGAACTATTTCACAGCGGAAGTATCCCAT

TGCAGGGCTACAGAATACATAATGAAAGGAGTATACATAAACACAGCTTTATTGAATGCA

TCCTGTGCAGCCATGGATGACTTCCAACTGATTCCAATGATAAGCAAATGCAGAACCAAA

GAAGGGAGACGAAAGACAAATCTGTATGGCTTCATTATAAAAGGAAGATCCCATTTGAGG

AATGACACCGATGTGGTAAACTTTGTGAGCATGGAATTCTCTCTCACTGACCCAAGGCTG

GAGCCACACAAATGGGAAAAGTACTGTGTGCTTGAGGTAGGAGACATGCTCCTACGGACT

GCAATAGGCCAAGTGTCAAGGCCCATGTTCCTGTATGTGAGAACCAATGGGACTTCTAAG

ATCAAAATGAAATGGGGTATGGAGATGAGGCGATGCCTTCTTCAATCCCTTCAACAAATT

GAGAGCATGATTGAAGCCGAGTCTTCTGTCAAAGAAAAAGACATGACCAAGGAATTCTTT

GAAAACAGATCAGAAACATGGCCAATTGGAGAGTCACCCAAAGGAGTGGAGGAAGGCTCC

ATTGGGAAGGTGTGCAGAACATTGCTAGCAAAGTCTGTGTTCAACAGCCTATATGCATCT

CCACAACTCGAGGGGTTTTCAGCTGAATCAAGAAAGTTGCTTCTCATTGTTCAGGCACTT

AGGGACAACCTGGAACCTGGAACCTTCGATCTTGGGGGGCTATATGAAGCAATTGAGGAG

TGCCTGATTAACGATCCCTGGGTTTTGCTTAATGCGTCTTGGTTCAACTCCTTCCTCACA

CATGCACTGAAATAGTCGTGGCAATGCTACTA---TTTGCTATCCATACTGTCCAAAAA-

GTACCTTGTTTCTACT

>A_mute_swan_North_Ossetia-Alania_325-03_2020_EPI1848730

AGCAAAAGCAGGTACTGATCCAAAATGGAAGACTTTGTGCGACAATGCTTCAATCCAATG

ATTGTCGAGCTTGCGGAAAAGGCAATGAAAGAATATGGGGAAGATCCTAAGATCGAAACA

AACAAGTTTGCCGCAATATGTACGCACTTAGAAGTCTGCTTCATGTATTCGGATTTCCAT

TTCATTGATGAACGAGGCGAATCAATAATCGTAGAATCTGGCGACCCGAATGCATTATTG

AAGCACCGATTTGAGATAATTGAAGGGAGAGACCGAACAATGGCCTGGACAGTGGTGAAT

AGTATATGCAACACTACAGGAGTCGAAAAGCCCAAGTTCCTTCCTGATTTGTATGACTAC

AGAGAAAACCGATTCATTGAAATTGGAGTAACGCGAAGGGAAGTTCACATATACTATCTT

GAAAAAGCCAACAAGATAAAATCAGAGAAAACACACATTCACATATTCTCATTCACTGGG

GAGGAAATGGCCACCAAGGCAGATTACACTCTTGATGAAGAGAGCAGAGCAAGAATAAAA

ACCAGGCTATTCACTATAAGACAAGAGATGGCCAATAGGGGTCTATGGGATTCCTTTCGT

CAGTCCGAGAGAGGCGAAGAGACAATTGAAGAAAGATTTGAAATCACAGGAACCATGCGC

AGGCTTGCTGACCAAAGTCTCCCACCGAACTTCGCCAGCCTTGAAAACTTTAGAGCCTAT

GTGGATGGATTTGAACCGAACGGCTGCATTGAGGGCAAGCTTTCTCAAATGTCAAAAGAA

GTGAATGCCAGAATTGAGCCATTTCTGAAGACAACACCACGCCCTCTCAGATTACCTGAT

GGACCTCCCTGTTCCCAGCGGTCGAAGTTCTTGCTAATGGATGCCCTTAAATTGAGCATT

GAAGACCCGAGCCATGAGGGGGAGGGTATACCGCTGTACGATGCAACCAAATGCATGAAG

ACATTTTTTGGCTGGAAGGAGCCCAACATCGTGAAACCACATGAAAAGGGCATAAACCCT

AATTACCTCCTGGCTTGGAAGCAGGTGCTATCAGAACTCCAAGATATTGAAAACGAGGAG

AAAATCCCAAAAACAAAGAACATGAAGAAGACGAGCCAACTGAAGTGGGCACTTGGTGAA

AACATGGCACCAGAGAAAGTGGACTTTGAGGACTGTAAAGATGTTAGCGATCTAAGACAG

TACGACAGTGACGAACCAGAATCTAGATCACTAGCAAGTTGGATCCAGAGTGAATTTAAC

AAGGCTTGCGAACTGACAGATTCGAGTTGGATTGAACTTGATGAAATAGGGGAAGACATT

GCTCCGATTGAACACATTGCGAGTATGAGGAGGAACTATTTCACAGCGGAAGTATCCCAT

TGCAGGGCTACAGAATACATAATGAAAGGAGTATACATAAACACAGCTTTATTGAATGCA

TCCTGTGCAGCCATGGATGACTTCCAACTGATTCCAATGATAAGCAAATGCAGAACCAAA

GAAGGGAGACGGAAGACAAATCTGTATGGCTTCATTATAAAAGGAAGATCCCATTTGAGG

AATGACACCGATGTGGTAAACTTTGTGAGCATGGAATTCTCTCTCACTGACCCAAGGCTG

GAGCCACACAAATGGGAAAAGTACTGTGTGCTTGAGGTAGGAGACATGCTCCTACGGACT

GCAATAGGCCAAGTGTCAAGGCCCATGTTCCTGTATGTGAGAACCAATGGGACTTCTAAG

ATTAAAATGAAATGGGGTATGGAGATGAGGCGATGCCTTCTTCAATCCCTTCAGCAAATT

GAGAGCATGATTGAAGCCGAGTCTTCTGTCAAAGAAAAAGACATGACCAAGGAATTCTTT

GAAAACAGATCAGAAACATGGCCAATTGGAGAGTCACCCAAAGGAGTGGAGGAAGGCTCC

ATTGGGAAGGTGTGCAGAACATTGCTAGCAAAGTCTGTGTTCAACAGCCTATATGCATCT

CCACAACTCGAGGGGTTTTCAGCTGAATCAAGAAAGTTGCTTCTCATTGTTCAGGCACTT

AGGGACAACCTGGAACCTGGGACCTTCGATCTTGGGGGGCTATATGAAGCAATTGAGGAG

TGCCTGATTAACGATCCCTGGGTTTTGCTTAATGCGTCTTGGTTCAACTCCTTCCTCACA

CATGCACTGAAATAGTCATGGCAATGCTACTA---TTTGCTATCCATACTGTCCAAAAAA

GTACCTTGTTTCTACT

>A_turkey_Rostov-on-Don_332-09_2021_EPI1848754

AGCAAAAGCAGGTACTGATTCAAAATGGAAGACTTTGTGCGACAATGCTTCAATCCAATG

ATTGTCGAGCTTGCGGAAAAGGCAATGAAAGAATATGGGGAAGATCCTAAGATCGAAACA

AACAAGTTTGCCGCAATATGTACACACTTAGAAGTCTGCTTCATGTATTCGGATTTCCAT

TTCATTGATGAACGAGGCGAATCAATAATCGTAGAATCTGGCGACCCGAATGCATTGTTG

AAGCACCGATTTGAGATAATTGAAGGGAGAGACCGAACAATGGCCTGGACAGTGGTGAAT

AGTATATGCAACACTACAGGAGTCGAAAAGCCCAAGTTCCTTCCTGATTTGTATGACTAC

AGAGAAAACCGATTCATTGAAATTGGAGTAACGCGAAGGGAAGTTCACATATACTATCTT

GAAAAAGCTAACAAGATAAAATCAGAGAAAACACACATTCACATATTCTCATTCACTGGG

GAGGAAATGGCCACCAAGGCAGATTACACTCTTGATGAAGAGAGCAGAGCAAGAATAAAA

ACCAGGCTATTCACTATAAGACAAGAGATGGCCAATAGGGGTCTATGGGATTCCTTTCGT

CAGTCCGAGAGAGGCGAAGAGACAATTGAAGAAAGATTTGAAATCACAGGAACCATGCGC

AGGCTTGCTGACCAAAGTCTCCCACCGAACTTCGCCAGCCTTGAAAACTTTAGAGCCTAT

GTGGATGGATTTGAACCGAACGGCTGCATTGAGGGCAAGCTTTCTCAAATGTCAAAAGAA

GTGAATGCCAGAATTGAGCCATTTCTGAAGACAACACCACGCCCTCTCAGATTACCTGAT

GGACCTCCCTGTTCCCAGCGGTCGAAGTTCTTGCTAATGGATGCCCTTAAATTGAGCATT

GAAGACCCGAGCCATGAGGGGGAGGGTATACCGCTGTACGATGCAACCAAATGCATGAAG

ACATTTTTTGGCTGGAAGGAGCCCAACATCGTGAAACCACATGAAAAGGGCATAAACCCT

AATTACCTCCTGGCTTGGAAGCAGGTGCTATCAGAACTCCAAGATATTGAAAACGAGGAG

AAAATCCCAAAAACAAAGAACATGAAGAAGACGAGCCAACTGAAGTGGGCACTTGGTGAA

AACATGGCACCAGAGAAAGTGGACTTTGAGGACTGTAAAGATGTTAGCGATCTAAGACAG

TACGACAGTGACGAACCAGAATCTAGATCACTAGCAAGTTGGATCCAGAGTGAATTTAAC

AAGGCTTGCGAACTGACAGATTCGAGTTGGATTGAACTTGATGAAATAGGGGAAGACGTT

GCTCCGATTGAACACATTGCGAGTATGAGGAGGAACTATTTCACAGCGGAAGTATCCCAT

TGCAGGGCTACAGAATACATAATGAAAGGAGTATACATAAACACAGCTTTATTGAATGCC

TCCTGTGCAGCCATGGATGACTTCCAACTGATTCCAATGATAAGCAAATGCAGAACCAAA

GAAGGGAGACGGAAGACAAATCTGTATGGCTTCATTATAAAAGGAAGATCCCATTTGAGG

AATGACACCGATGTGGTAAACTTTGTGAGCATGGAATTCTCTCTCACTGACCCAAGGCTG

GAGCCACACAAATGGGAAAAGTACTGTGTGCTTGAGGTAGGAGACATGCTCCTACGGACT

GCAATAGGCCAAGTGTCAAGGCCCATGTTCCTGTATGTGAGAACCAATGGGACTTCTAAG

ATCAAAATGAAATGGGGTATGGAGATGAGGCGATGCCTTCTTCAATCCCTTCAACAAATT

GAGAGCATGATTGAAGCCGAGTCTTCTGTCAAAGAAAAAGACATGACCAAGGAATTCTTT

GAAAACAGATCAGAAACATGGCCAATTGGAGAGTCACCCAAAGGAGTGGAGGAAGGCTCC

ATTGGGAAGGTGTGCAGAACATTGCTAGCAAAGTCTGTGTTCAACAGCCTATATGCATCT

CCACAACTCGAGGGGTTTTCAGCTGAATCAAGAAAGTTGCTTCTCATTGTTCAGGCACTT

AGGGACAACCTGGAACCTGGGACCTTCGATCTTGGGGGGCTATATGAAGCAATTGAGGAG

TGCCTGATTAACGATCCCTGGGTTTTGCTTAATGCGTCTTGGTTCAACTCCTTCCTCACA

CATGCACTGAAATAGTCGTGGCAATGCTACTA---TTTGCTATCCATACTGTCCAAAAA-

GTACCTTGTTTCTACT

>A_chicken_Krasnodar_334-03_2021_EPI1848802

AGCAAAAGCAGGTACTGATCCAAAATGGAAGACTTTGTGCGACAATGCTTCAATCCAATG

ATTGTCGAGCTTGCGGAAAAGGCAATGAAAGAATATGGGGAAGATCCTAAGATCGAAACA

AACAAGTTTGCCGCAATATGTACACACTTAGAAGTCTGCTTCATGTATTCGGATTTCCAT

TTCATTGATGAACGAGGCGAATCAATAATCGTAGAATCTGGCGACCCGAATGCATTATTG

AAGCACCGATTTGAGATAATTGAAGGGAGAGACCGAACAATGGCCTGGACAGTGGTGAAT

AGTATATGCAACACTACAGGAGTCGAAAAGCCCAAGTTCCTTCCTGATTTGTATGACTAC

AGAGAAAACCGATTCATTGAAATTGGAGTAACGCGAAGGGAAGTTCACATATACTATCTT

GAAAAAGCCAACAAGATAAAATCAGAGAAAACACACATTCACATATTCTCATTCACTGGG

GAGGAAATGGCCACCAAGGCAGATTACACTCTTGATGAAGAGAGCAGAGCAAGAATAAAA

ACCAGGCTATTCACTATAAGACAAGAGATGGCCAATAGGGGTCTATGGGATTCCTTTCGT

CAGTCCGAGAGAGGCGAAGAGACAATTGAAGAAAGATTTGAAATCACAGGAACCATGCGC

AGGCTTGCTGACCAAAGTCTCCCACCGAACTTCGCCAGCCTTGAAAACTTTAGAGCCTAT

GTGGATGGATTTGAACCGAACGGCTGCATTGAGGGCAAGCTTTCTCAAATGTCAAAAGAA

GTGAATGCCAGAATTGAGCCATTTCTGAAGACGACACCACGCCCTCTCAGATTACCTGAT

GGACCTCCCTGTTCCCAGCGGTCGAAGTTCTTGCTAATGGATGCCCTTAAATTGAGCATT

GAAGACCCGAGCCATGAGGGGGAGGGTATACCGCTGTACGATGCAACCAAATGCATGAAG

ACATTTTTTGGCTGGAAGGAGCCCAACATCGTGAAACCACATGAAAAGGGCATAAACCCT

AATTACCTCCTGGCTTGGAAGCAGGTGCTATCAGAACTCCAAGATATTGAAAACGAGGAG

AAAATCCCAAAAACAAAGAACATGAAGAAGACGAGCCAACTGAAGTGGGCACTTGGTGAA

AACATGGCACCAGAGAAATTGGACTTTGAGGACTGTAAAGATGTTAGCGATCTAAGACAG

TACGACAGTGACGAACCAGAGTCTAGATCACTAGCAAGTTGGATCCAGAGTGAATTTAAC

AAGGCTTGCGAACTGACAGATTCGAGTTGGATCGAACTTGATGAAATAGGGGAAGACGTT

GCTCCAATTGAACACATTGCGAGTATGAGGAGGAACTATTTCACAGCGGAAGTATCCCAT

TGCAGGGCTACAGAATACATAATGAAAGGAGTATACATAAACACAGCTTTATTGAATGCA

TCCTGTGCAGCCATGGATGACTTCCAACTGATTCCAATGATAAGCAAATGCAGAACCAAA

GAAGGGAGACGGAAGACAAATCTGTATGGCTTCATTATAAAAGGAAGATCCCATTTGAGG

AATGACACCGATGTGGTAAACTTTGTGAGCATGGAATTCTCTCTCACTGACCCAAGGCTG

GAGCCACACAAATGGGAAAAGTACTGTGTACTTGAGGTAGGAGACATGCTCCTACGGACT

GCAATAGGCCAAGTGTCAAGGCCCATGTTCCTGTATGTGAGAACCAATGGGACTTCTAAG

ATCAAAATGAAATGGGGTATGGAGATGAGGCGATGCCTTCTTCAATCCCTTCAACAAATT

GAGAGCATGATTGAAGCCGAGTCTTCTGTCAAAGAAAAAGACATGACCAAGGAATTCTTT

GAAAACAGATCAGAAACATGGCCAATTGGAGAGTCACCCAAAGGAGTGGAGGAAGGCTCC

ATTGGGAAGGTGTGCAGAACATTGCTAGCAAAGTCTGTGTTCAACAGCCTATATGCATCT

CCACAACTCGAGGGGTTTTCAGCTGAATCAAGAAAGTTGCTTCTCATTGTTCAGGCACTT

AGGGACAACCTGGAACCTGGAACCTTCGATCTTGGGGGGCTATATGAAGCAATTGAGGAG

TGCCTGATTAACGATCCCTGGGTTTTGCTTAATGCGTCTTGGTTCAACTCCTTCCTCACA

CATGCACTGAAATAGTCGTGGCAATGCTACTA---TTTGCTATCCATACTGTCCAAAAAA

GTACCTTGTTTCTACT

>A_pheasant_Wales_000252_2021_EPI1848882

AGCGAAAGCAGGTACTGATCCAAAATGGAAGACTTTGTGCGACAATGCTTCAATCCAATG

ATTGTCGAGCTTGCGGAAAAGGCAATGAAAGAATATGGGGAAGATCCTAAGATCGAAACA

AACAAGTTTGCCGCAATATGTACACACTTAGAAGTCTGCTTCATGTATTCGGATTTCCAT

TTCATTGATGAACGAGGCGAATCAATAATCGTAGAATCTGGCGACCCGAATGCATTATTG

AAGCACCGATTTGAGATAATTGAAGGGAGAGACCGAACAATGGCCTGGACAGTGGTGAAT

AGTATATGCAACACTACAGGAGTCGAAAAGCCCAAGTTCCTTCCTGATTTGTATGACTAC

AGAGAAAACCGATTCATTGAAATTGGAGTAACGCGAAGGGAAGTTCACATATACTATCTT

GAAAAAGCCAACAAGATAAAATCAGAGAAAACACACATTCACATATTCTCATTCACTGGG

GAGGAAATGGCCACCAAGGCCGATTACACTCTTGATGAAGAGAGCAGAGCAAGAATAAAA

ACCAGGCTATTCACTATAAGACAAGAGATGGCCAATAGGGGTCTATGGGATTCCTTTCGT

CAGTCCGAGAGAGGCGAAGAGACAATTGAAGAAAGATTTGAAATCACAGGAACCATGCGC

AGGCTTGCTGACCAAAGTCTCCCACCGAACTTCGCCAGCCTTGAAAACTTTAGAGCCTAT

GTGGATGGATTTGAACCGAACGGCTGCATTGAGGGCAAGCTTTCTCAAATGTCAAAAGAA

GTGAATGCCAGAATTGAGCCATTTCTGAAGACGACACCACGCCCTCTCAGATTACCTGAT

GGACCTCCCTGTTCCCAGCGGTCGAAGTTCTTGCTAATGGATGCCCTTAAATTGAGCATT

GAAGACCCTAGCCATGAGGGGGAGGGTATACCGCTGTACGATGCAACCAAATGCATGAAG

ACATTTTTTGGCTGGAAGGAGCCCAACATCGTGAAACCACATGAAAAGGGCATAAACCCT

AATTACCTCCTGGCTTGGAAGCAGGTGCTATCAGAACTCCATGATATTGAAAACGAGGAG

AAAATCCCAAAAACAAAGAACATGAAGAAGACGAGCCAACTGAAGTGGGCACTTGGTGAA

AACATGGCACCAGAGAAAGTGGACTTTGAGGACTGTAAAGATGTTAGCGATCTAAGACAG

TACGACAGTGACGAACCAGAGTCTAGATCACTAGCAAGTTGGATCCAGAGTGAATTTAAC

AAGGCTTGCGAACTGACAGATTCGAGTTGGATTGAACTTGATGAAATAGGGGAAGACGTT

GCTCCAATTGAACACATTGCGAGTATGAGGAGGAACTATTTCACAGCGGAAGTATCCCAT

TGCAGGGCTACAGAATACATAATGAAAGGAGTATACATAAACACAGCTTTATTGAATGCA

TCCTGTGCAGCCATGGATGACTTCCAACTGATTCCAATGATAAGCAAATGCAGAACCAAA

GAAGGGAGACGGAAGACAAATCTGTATGGCTTCATTATAAAAGGAAGATCCCATTTGAGG

AATGACACCGATGTGGTAAACTTTGTGAGCATGGAATTCTCTCTCACTGACCCAAGGCTG

GAGCCACACAAATGGGAAAAGTACTGTGTGCTTGAGGTAGGAGACATGCTCCTACGGACT

GCAATAGGCCAAGTGTCAAGGCCCATGTTCCTGTATGTGAGAACCAATGGGACTTCTAAG

ATCAAAATGAAATGGGGTATGGAGATGAGGCGATGCCTTCTTCAATCCCTTCAACAAATT

GAGAGCATGATTGAAGCCGAGTCTTCTGTCAAAGAAAAAGACATGACCAAGGAGTTCTTT

GAAAACAGATCAGAAACATGGCCAATTGGAGAGTCACCCAAAGGAGTGGAGGAAGGCTCC

ATTGGGAAGGTGTGCAGAACATTGCTAGCAAAGTCTGTGTTCAACAGCCTATATGCATCT

CCACAACTCGAGGGGTTTTCAGCTGAATCAAGAAAGTTGCTTCTCATTGTTCAGGCACTT

AGGGACAACCTGGAACCTGGAACCTTCGATCTTGGGGGGCTATATGAAGCAATTGAGGAG

TGCCTGATTAACGATCCCTGGGTTTTGCTTAATGCGTCTTGGTTCAACTCCTTCCTCACA

CATGCACTGAAATAGTCGTGGCAATGCTACTA---TTTGCTATCCATACTGTCCAAAAAA

GTACCTTGTTTCTACT

>A_mute_swan_Czech_Republic_1656-1_2021_EPI1850127

------------------------ATGGAAGACTTTGTGCGACAATGCTTCAATCCAATG

ATTGTCGAGCTTGCGGAAAAGGCAATGAAAGAATATGGGGAAGATCCTAAGATCGAAACA

AACAAGTTTGCCGCAATATGTACACACTTAGAAGTCTGCTTCATGTATTCGGATTTCCAT

TTCATTGATGAACGAGGCGAATCAATAATCGTAGAATCTGGCGACCCGAATGCATTATTG

AAGCACCGATTTGAGATAATTGAAGGGAGAGACCGAACAATGGCCTGGACAGTGGTGAAT

AGTATATGCAACACTACAGGAGTCGAAAAGCCCAAGTTCCTTCCTGATTTGTATGACTAC

AGAGAAAACCGATTCATTGAAATTGGAGTAACGCGAAGGGAAGTTCACATATACTATCTT

GAAAAAGCCAACAAAATAAAATCAGAGAAAACACACATTCACATATTCTCATTCACTGGG

GAGGAAATGGCCACCAAGGCAGATTACACTCTTGATGAAGAGAGCAGAGCAAGAATAAAA

ACCAGGCTATTCACTATAAGACAAGAGATGGCCAATAGGGGTCTATGGGATTCCTTTCGT

CAGTCCGAGAGAGGCGAAGAGACAATTGAAGAAAGATTTGAAATCACAGGAACCATGCGC

AGGCTTGCTGACCAAAGTCTCCCACCGAACTTCGCCAGCCTTGAAAATTTTAGAGCCTAT

GTGGATGGATTTGAACCGAACGGCTGCATTGAGGGCAAGCTTTCTCAAATGTCAAAAGAA

GTGAATGCCAGAATTGAGCCATTTCTGAAGAATACACCACGTCCTCTCAGATTGCCTGAT

GGACCTCCCTGTTCCCAGCGGTCGAAGTTCTTGCTAATGGATGCCCTTAAATTAAGCATT

GAAGACCCGAGCCATGAGGGGGAGGGTATACCGCTGTACGATGCAACCAAATGCATGAAG

ACATTTTTTGGCTGGAAGGAGCCCAACATCGTGAAACCACATGAAAAGGGCATAAACCCT

AATTACCTCCTGGCTTGGAAGCAGGTGCTATCAGAACTCCAAGATATTGAAAACGAGGAG

AAAATCCCAAAAACAAAGAACATGAAGAAGACGAGCCAACTGAAGTGGGCACTTGGTGAA

AATATGGCACCAGAGAAAGTGGACTTTGAGGACTGTAAAGATGTTAGCGATCTAAAACAG

TACGACAGTGACGAACCAGAATCCAGATCACTAGCAAGTTGGATCCAGAGTGAATTTAAC

AAGGCTTGCGAACTGACAGATTCGAGTTGGATTGAACTTGATGAAATAGGGGAAGACGTT

GCTCCAATTGAACACATTGCGAGTATGAGGAGGAACTATTTCACAGCGGAAGTATCCCAT

TGCAGGGCTACAGAATACATAATGAAAGGAGTATACATAAACACAGCTTTATTGAATGCA

TCCTGTGCAGCCATGGATGACTTCCAACTGATTCCAATGATAAGCAAATGCAGAACCAAA

GAAGGGAGACGGAAGACAAATCTGTATGGCTTCATTATAAAAGGAAGATCCCATTTGAGG

AATGACACCGATGTGGTAAACTTTGTGAGCATGGAATTCTCTCTCACTGACCCAAGGCTG

GAGCCACACAAATGGGAAAAGTACTGTGTGCTTGAGGTAGGAGACATGCTCCTACGGACT

GCAATAGGCCAAGTGTCAAGGCCCATGTTCCTGTATGTGAGAACCAATGGGACTTCTAAG

ATCAAAATGAAATGGGGTATGGAGATGAGGCGATGCCTTCTTCAATCCCTTCAACAAATT

GAGAGCATGATTGAAGCCGAGTCTTCTGTCAAAGAAAAAGACATGACCAAGGAATTCTTT

GAAAACAGATCAGAAACATGGCCAATTGGAGAGTCACCCAAAGGAGTGGAGGAAGGCTCC

ATTGGGAAGGTGTGCAGAACATTGCTAGCAAAGTCTGTGTTCAATAGCCTATATGCATCT

CCACAACTCGAGGGGTTTTCAGCTGAATCAAGAAAGTTGCTTCTCATTGTTCAGGCACTT

AGGGACAACCTGGAACCTGGAACCTTCGATCTTGGGGGGCTATATGAAGCAATTGAGGAG

TGCCTGATTAACGATCCCTGGGTTTTGCTTAATGCGTCTTGGTTCAACTCCTTCCTCACA

CATGCACTGAAATAGTCGTGGCAATGCTACTA---TTTGCTATCTATACTGTCCAAA---

----------------

>A_mute_swan_Croatia_14_2021_EPI1850961

-------------------CCAAAATGGAAGACTTTGTGCGACAATGCTTCAATCCAATG

ATTGTCGAGCTTGCGGAAAAGGCAATGAAAGAATATGGGGAAGATCCTAAGATCGAAACA

AACAAGTTTGCCGCAATATGTACACACTTAGAAGTCTGCTTCATGTATTCGGATTTCCAT

TTCATTGATGAACGAGGCGAATCAATAATCGTAGAATCTGGCGACCCGAATGCATTATTG

AAGCACCGATTTGAGATAATTGAAGGGAGAGACCGAACAATGGCCTGGACAGTGGTGAAT

AGTATATGCAACACTACAGGAGTCGAAAAGCCCAAGTTCCTTCCTGATTTGTATGACTAC

AGAGAAAACCGATTCATTGAAATTGGAGTAACGCGAAGGGAAGTTCACATATACTATCTT

GAAAAAGCCAACAAGATAAAATCAGAGAAAACACACATTCACATATTCTCATTCACTGGG

GAGGAAATGGCCACCAAGGCAGATTACACTCTTGATGAAGAGAGCAGAGCAAGAATAAAA

ACCAGGCTATTCACTATAAGACAAGAGATGGCCAATAGGGGTCTATGGGATTCCTTTCGT

CAGTCCGAGAGAGGCGAAGAGACAATTGAGGAAAGATTTGAAATCACAGGAACCATGCGC

AGGCTTGCTGACCAAAGTCTCCCACCGAACTTCGCCAGCCTTGAAAACTTTAGAGCCTAT

GTGGATGGATTTGAACCGAACGGCTGCATTGAGGGCAAGCTTTCTCAAATGTCAAAAGAA

GTGAATGCCAGAATTGAGCCATTTCTGAAGACGACACCACGCCCTCTCAGATTACCTGAT

GGACCTCCCTGTTCCCAGCGGTCGAAGTTCTTGCTAATGGATGCCCTTAAATTGAGCATT

GAAGACCCGAGCCATGAGGGGGAGGGTATACCGCTGTACGATGCAACCAAATGCATGAAG

ACATTTTTTGGCTGGAAGGAGCCCAATATCGTGAAACCACATGAAAAGGGCATAAACCCT

AATTACCTCCTGGCTTGGAAGCAGGTGCTATCAGAACTCCAAGATATTGAAAACGAGGAG

AAAATCCCAAAAACAAAGAACATGAAGAAGACGAGCCAACTGAAGTGGGCACTTGGTGAA

AACATGGCACCAGAGAAAGTGGACTTTGAGGACTGTAAAGATGTTAGCGATCTAAGACAG

TACGACAGTGACGAACCAGAGTCTAGATCACTAGCAAGTTGGATCCAGAGTGAATTTAAC

AAGGCTTGCGAATTGACAGATTCGAGTTGGATCGAACTTGATGAAATAGGGGAAGACGTT

GCTCCAATTGAACACATTGCGAGTATGAGGAGGAACTACTTCACAGCGGAAGTATCCCAT

TGCAGGGCTACAGAATACATAATGAAAGGAGTATACATAAACACAGCTTTATTGAATGCA

TCCTGTGCAGCCATGGATGACTTCCAACTGATTCCAATGATAAGCAAATGCAGAACCAAA

GAAGGGAGACGGAAGACAAATCTGTATGGCTTCATTATAAAAGGAAGATCCCATTTGAGG

AATGACACCGATGTGGTAAACTTTGTGAGCATGGAATTCTCTCTCACTGACCCAAGGCTG

GAGCCACACAAATGGGAAAAGTACTGTGTACTTGAGGTAGGAGACATGCTCCTACGGACT

GCAATAGGCCAAGTGTCGAGGCCCATGTTCCTGTATGTGAGAACCAATGGGACTTCTAAG

ATCAAAATGAAATGGGGCATGGAGATGAGGCGATGCCTTCTTCAATCCCTTCAACAAATT

GAGAGCATGATTGAAGCCGAGTCTTCTGTCAAAGAAAAAGACATGACCAAGGAATTCTTT

GAAAACAGATCAGAAACATGGCCAATTGGAGAGTCACCCAAAGGAGTGGAGGAAGGCTCC

ATTGGGAAGGTGTGCAGAACATTGCTAGCAAAGTCTGTGTTCAACAGCCTATATGCATCT

CCACAACTCGAGGGGTTTTCAGCTGAATCAAGAAAGTTGCTTCTCATTGTTCAGGCACTT

AGGGACAACCTGGAACCTGGAACCTTCGATCTTGGGGGGCTATATGAAGCAATTGAGGAG

TGCCTGATTAACGATCCCTGGGTTTTGCTTAATGCGTCTTGGTTCAACTCCTTCCTCACA

CATGCACTGAAATAGTCGTGGCAATGCTACTA---TTTGCTATCCATACTGTCCAAAAAA

GTA-------------

>A_chicken_Czech_Republic_3531-1_2021_EPI1854239

------------------------ATGGAAGACTTTGTGCGACAATGCTTCAATCCAATG

ATTGTCGAGCTTGCGGAAAAGGCAATGAAAGAATATGGGGAAGATCCTAAGATCGAAACA

AACAAGTTTGCCGCAATATGTACACACTTAGAAGTCTGCTTCATGTATTCGGATTTCCAT

TTCATTGATGAACGAGGCGAATCAATAATCGTAGAATCTGGCGACCCGAATGCATTATTG

AAGCACCGATTTGAGATAATTGAAGGGAGAGACCGAACAATGGCCTGGACAGTGGTGAAT

AGTATATGCAACACTACAGGAGTCGAAAAGCCCAAGTTCCTTCCTGATTTGTATGACTAC

AGAGAAAACCGATTCATTGAAATTGGAGTAACGCGAAGGGAAGTTCACATATACTATCTT

GAAAAAGCCAACAAGATAAAATCAGAGAAAACACACATTCACATATTCTCATTCACTGGG

GAGGAAATGGCCACCAAGGCCGATTACACTCTTGATGAAGAGAGCAGAGCAAGAATAAAA

ACCAGGCTATTCACTATAAGACAAGAGATGGCCAATAGGGGTCTATGGGATTCCTTTCGT

CAGTCCGAGAGAGGCGAAGAGACAATTGAAGAAAGATTTGAAATCACAGGAACCATGCGC

AGGCTTGCTGACCAAAGTCTCCCACCGAACTTCGCCAGCCTTGAAAACTTTAGAGCCTAT

GTGGATGGATTTGAACCGAACGGCTGCATTGAGGGCAAGCTTTCTCAAATGTCAAAAGAA

GTGAATGCCAGAATTGAGCCATTTCTGAAGACGACACCACGCCCTCTCAGATTACCTGAT

GGACCTCCCTGTTCCCAGCGGTCGAAGTTCTTGCTAATGGATGCCCTTAAATTAAGCATT

GAAGACCCGAGCCATGAGGGGGAGGGTATACCGCTGTACGATGCAACCAAATGCATGAAG

ACATTTTTTGGCTGGAAGGAGCCCAACATCGTGAAACCACATGAAAAGGGCATAAACCCT

AATTACCTCCTGGCTTGGAAGCAGGTGCTATCAGAACTCCAAGATATTGAAAACGAGGAG

AAAATCCCAAAAACAAAGAACATGAAGAAGACGAGCCAACTGAAGTGGGCACTTGGTGAA

AACATGGCACCAGAGAAAGTGGACTTTGAGGACTGTAAAGATGTTAGCGATCTAAGACAG

TACGACAGTGACGAACCAGAGTCTAGATCACTAGCAAGTTGGATCCAGAGTGAATTTAAC

AAGGCTTGCGAACTGACAGATTCGAGTTGGATTGAACTTGATGAAATAGGGGAAGACGTT

GCTCCAATTGAACACATTGCGAGTATGAGGAGGAACTATTTCACAGCGGAAGTATCCCAT

TGCAGGGCTACAGAATACATAATGAAAGGAGTATACATAAACACAGCTTTATTGAATGCA

TCCTGTGCAGCCATGGATGACTTCCAACTGATTCCAATGATAAGCAAATGCAGAACCAAA

GAAGGGAGACGGAAGACAAATCTGTATGGCTTCATTATAAAAGGAAGATCCCATTTGAGG

AATGACACCGATGTGGTAAACTTTGTGAGCATGGAATTCTCTCTCACTGACCCAAGGCTG

GAGCCACACAAATGGGAAAAGTACTGTGTGCTTGAGGTAGGAGACATGCTCCTACGGACT

GCAATAGGCCAAGTGTCAAGGCCCATGTTCCTGTATGTGAGAACCAATGGGACTTCTAAG

ATCAAAATGAAATGGGGTATGGAGATGAGGCGATGCCTTCTTCAATCCCTTCAACAAATT

GAGAGCATGATTGAAGCCGAGTCTTCTGTCAAAGAAAAAGACATGACCAAGGAATTCTTT

GAAAACAGATCAGAAACATGGCCAATTGGAGAGTCACCCAAAGGAGTGGAGGAAGGCTCC

ATTGGGAAGGTGTGCAGAACATTGCTAGCAAAGTCTGTGTTCAACAGCCTATATGCATCT

CCACAACTCGAGGGGTTTTCAGCTGAATCAAGAAAGTTGCTTCTCATTGTTCAGGCACTT

AGGGACAACCTGGAACCTGGAACCTTCGATCTTGGGGGGCTATATGAAGCAATTGAGGAG

TGCCTGATTAACGATCCCTGGGTTTTGCTTAATGCGTCTTGGTTCAACTCCTTCCTCACA

CATGCACTGAAATAGTCGTGGCAATGCTACTA---TTTGCTATCCATACTGTCCAAA---

----------------

>A_wigeon_Latvia_23903_2021_EPI1855974

---------------TGATCCAAAATGGAAGACTTTGTGCGACAATGCTTCAATCCAATG

ATTGTCGAGCTTGCGGAAAAGGCAATGAAAGAATATGGGGAAGATCCTAAGATCGAAACA

AACAAGTTTGCTGCAATATGTACACACTTAGAAGTCTGCTTCATGTATTCGGATTTCCAT

TTCATTGATGAACGAGGCGAATCAATAATCGTAGAATCTGGCGACCCGAATGCATTATTG

AAGCACCGATTTGAGATAATTGAAGGGAGAGACCGAACAATGGCCTGGACAGTGGTGAAT

AGTATATGCAACACTACAGGAGTCGAAAAGCCCAAGTTCCTTCCTGATTTGTATGACTAC

AGAGAAAACCGATTCATTGAAATTGGAGTAACGCGAAGGGAAGTTCACATATACTATCTT

GAAAAAGCCAACAAAATAAAATCAGAGAAAACACACATTCACATATTCTCATTCACTGGG

GAGGAAATGGCCACCAAGGCAGATTACACTCTTGATGAAGAGAGCAGAGCAAGAATAAAA

ACCAGGCTATTCACTATAAGACAAGAGATGGCCAATAGGGGTCTATGGGATTCCTTTCGT

CAGTCCGAGAGAGGCGAAGAGACAATTGAAGAAAGATTTGAAATCACAGGAACCATGCGC

AGGCTTGCTGACCAAAGTCTCCCACCGAACTTCGCCAGCCTTGAAAATTTTAGAGCCTAT

GTGGATGGATTTGAACCGAACGGCTGCATTGAGGGCAAGCTTTCTCAAATGTCAAAAGAA

GTGAATGCCAGAATTGAGCCATTTCTGAAGAGTACACCACGCCCTCTCAGATTGCCTGAT

GGACCTCCCTGTTCCCAGCGGTCGAAGTTCTTGCTAATGGATGCCCTTAAATTAAGCATT

GAAGACCCGAGCCATGAGGGGGAGGGTATACCGCTGTACGATGCAACCAAATGCATGAAG

ACATTTTTTGGCTGGAAGGAGCCCAACATCGTGAAACCACATGAAAAGGGCATAAACCCT

AATTACCTCCTGGCTTGGAAGCAGGTGCTATCAGAACTCCAAGATATTGAAAACGAGGAG

AAAATCCCAAAAACAAAGAACATGAAGAAGACGAGCCAACTGAAGTGGGCACTTGGTGAA

AATATGGCACCAGAGAAAGTGGACTTTGAGGACTGTAAAGATGTTAGCGATCTAAAACAG

TACGACAGTGACGAACCAGAATCCAGATCACTAGCAAGTTGGATCCAGAGTGAATTTAAC

AAGGCTTGCGAACTGACAGATTCGAGTTGGATTGAACTTGATGAAATAGGGGAAGACGTT

GCTCCAATTGAACACATTGCGAGTATGAGGAGGAACTATTTCACAGCGGAAGTATCCCAT

TGCAGGGCTACAGAATACATAATGAAAGGAGTATACATAAACACAGCTTTATTGAATGCA

TCCTGTGCAGCCATGGATGACTTCCAACTGATTCCAATGATAAGCAAATGCAGAACCAAA

GAAGGGAGACGGAAGACAAATCTGTATGGCTTCATTATAAAAGGAARATCCCATTTGAGG

AATGACACCGATGTGGTAAACTTTGTGAGCATGGAATTCTCTCTCACTGACCCAAGGCTG

GAGCCACACAAATGGGAAAAGTACTGTGTGCTTGAGGTAGGAGACATGCTCCTACGGACT

GCAATAGGCCAAGTGTCAAGGCCCATGTTCCTGTATGTGAGAACCAATGGGACTTCTAAG

ATCAAAATGAAATGGGGTATGGAGATGAGGCGATGCCTTCTTCAATCCCTTCAACAAATT

GAGAGCATGATTGAAGCCGAGTCTTCTGTCAAAGAAAAAGACATGACTAAGGAATTCTTT

GAAAACAGATCAGAAACATGGCCAATTGGAGAGTCACCCAAAGGAGTGGAGGAAGGCTCC

ATTGGGAAGGTGTGCAGAACATTGCTAGCAAAGTCTGTGTTCAATAGCCTATATGCATCT

CCACAACTCGAGGGGTTTTCAGCTGAATCAAGAAAGTTGCTTCTCATTGTTCAGGCACTT

AGGGACAACCTGGAACCTGGAACCTTCGATCTTGGGGGGCTATATGAAGCAATTGAGGAG

TGCCTGATTAACGATCCCTGGGTTTTGCTTAATGCGTCTTGGTTCAACTCCTTCCTCACA

CATGCACTGAAATAGTCGTGGCAATGCTACTA---TTTGCTATCCATACTGTCCAAAA--

----------------

>A_chicken_Czech_Republic_4980_2021_EPI1858496

------------------------ATGGAAGACTTTGTGCGACAATGCTTCAATCCAATG

ATTGTCGAGCTTGCGGAGAAGGCAATGAAAGAATATGGGGAAGATCCTAAGATCGAAACA

AACAAGTTTGCCGCAATATGTACACACTTAGAAGTCTGCTTCATGTATTCGGATTTCCAT

TTCATTGATGAACGAGGCGAATCAATAATCGTAGAATCTGGCGACCCGAATGCATTATTG

AAGCACCGATTTGAGATAATTGAAGGGAGAGACCGAACAATGGCCTGGACAGTGGTGAAT

AGTATATGCAACACTACAGGAGTCGAAAAGCCCAAGTTCCTTCCTGATTTGTATGACTAC

CGAGAAAACCGATTCATTGAAATTGGAGTAACGCGAAGGGAAGTTCACATATACTATCTT

GAAAAAGCCAACAAGATAAAATCAGAGAAAACACACATTCACATATTCTCATTCACTGGG

GAGGAAATGGCCACCAAGGCAGATTACACTCTTGATGAAGAGAGCAGAGCAAGAATAAAA

ACCAGGCTATTCACTATAAGACAAGAGATGGCCAATAGGGGTCTATGGGATTCCTTTCGT

CAGTCCGAGAGAGGCGAAGAGACAATTGAAGAAAGATTTGAAATCACAGGAACCATGCGC

AGGCTTGCTGACCAAAGTCTCCCACCGAACTTCGCCAGCCTTGAAAACTTTAGAGCCTAT

GTGGATGGATTTGAACCGAACGGCTGCATTGAGGGCAAGCTTTCTCAAATGTCAAAAGAA

GTGAATGCCAGAATTGAGCCATTTCTGAAGACGACACCACGCCCTCTCAGATTACCTGAT

GGACCTCCCTGTTCCCAGCGGTCGAAGTTCTTGCTAATGGATGCCCTTAAATTGAGCATT

GAAGACCCGAGCCATGAGGGGGAGGGTATACCGCTGTACGATGCAACCAAATGCATGAAG

ACATTTTTTGGCTGGAAGGAGCCCAACATCGTGAAACCACATGAAAAGGGCATAAACCCT

AATTACCTCCTGGCTTGGAAGCAGGTGCTATCGGAACTCCAAGATATTGAAAACGAGGAG

AAAATCCCAAAAACAAAGAACATGAAGAAGACGAGCCAACTGAAGTGGGCACTTGGTGAA

AACATGGCACCAGAGAAAGTGGACTTTGAGGACTGTAAAGATGTTAGCGATCTAAGACAG

TACGACAGTGACGAACCAGAGTCTAGATCACTAGCAAGTTGGATCCAGAGTGAATTTAAC

AAGGCTTGCGAACTGACAGATTCGAGTTGGATTGAACTTGATGAAATAGGGGAAGACGTT

GCTCCAATTGAACACATTGCGAGTATGAGGAGGAACTATTTCACAGCGGAAGTATCCCAT

TGCAGGGCTACAGAATACATAATGAAAGGAGTATACATAAACACAGCTTTATTGAATGCA

TCCTGTGCAGCCATGGATGACTTCCAACTGATTCCAATGATAAGCAAATGCAGAACCAAA

GAAGGGAGACGGAAGACAAATCTGTATGGCTTCATTATAAAAGGAAGATCCCATTTGAGG

AATGACACCGATGTGGTAAACTTTGTGAGCATGGAATTCTCTCTCACTGACCCAAGGCTG

GAGCCACACAAATGGGAAAAGTATTGTGTACTTGAGGTAGGAGACATGCTCCTACGGACT

GCAATAGGCCAAGTGTCAAGGCCCATGTTCCTGTATGTGAGAACCAATGGGACTTCTAAG

ATCAAAATGAAATGGGGTATGGAGATGAGGCGATGCCTTCTTCAATCCCTTCAACAAATT

GAGAGCATGATTGAAGCCGAGTCTTCTGTCAAAGAAAAAGACATGACCAAGGAATTCTTT

GAAAACAGATCAGAAACATGGCCAATTGGAGAATCACCCAAAGGAGTGGAGGAAGGCTCC

ATTGGGAAGGTGTGCAGAACATTGCTAGCAAAGTCTGTGTTCAACAGCCTATATGCATCT

CCACAACTCGAGGGGTTTTCAGCTGAATCAAGAAAGTTGCTTCTCATTGTTCAGGCACTT

AGGGACAACCTGGAACCTGGAACCTTCGATCTTGGGGGGCTATATGAAGCAATTGAGGAG

TGCCTGATTAACGATCCCTGGGTTTTGCTTAATGCGTCTTGGTTCAACTCCTTCCTCACA

CATGCACTGAAATAGTCGTGGCAATGCTACTA---TTTGCTATCCATACTGTCCAAA---

----------------

>A_swan_Lithuania_1258PG1_21VIR2606-2_2021_EPI1858570

AGCRAAAGCAGGTACTGATCCAAAATGGAAGACTTTGTGCGACAATGCTTCAATCCAATG

ATTGTCGAGCTTGCGGAAAAGGCAATGAAAGAATATGGGGAAGATCCTAAGATCGAAACA

AACAAGTTTGCCGCAATATGTACACACTTAGAAGTCTGCTTCATGTATTCGGATTTCCAT

TTCATTGATGAACGAGGCGAATCAATAATCGTAGAATCTGGCGACCCGAATGCATTATTG

AAGCACCGATTTGAGATAATTGAAGGGAGAGACCGAACAATGGCCTGGACAGTGGTGAAT

AGTATATGCAACACTACAGGAGTCGAAAAGCCCAAGTTCCTTCCTGATTTGTATGACTAC

CGAGAAAACCGATTCATTGAAATTGGAGTAACGCGAAGGGAAGTTCACATATACTATCTT

GAAAAAGCCAACAAGATAAAATCAGAGAAAACACACATTCACATATTCTCATTCACTGGG

GAGGAAATGGCCACCAAGGCAGATTACACTCTTGATGAAGAGAGCAGAGCAAGAATAAAA

ACCAGGCTATTCACTATAAGACAAGAGATGGCCAATAGGGGTCTATGGGATTCCTTTCGT

CAGTCCGAGAGAGGCGAAGAGACAATTGAAGAAAGATTTGAAATCACAGGAACCATGCGC

AGGCTTGCTGACCAAAGTCTCCCACCGAACTTCGCCAGCCTTGAAAACTTTAGAGCCTAT

GTGGATGGATTTGAACCGAACGGCTGCATTGAGGGCAAGCTTTCTCAAATGTCAAAAGAA

GTGAATGCCAGAATTGAGTCATTTCTGAAGACGACACCACGCCCTCTCAGATTACCTGAT

GGACCTCCCTGTTCCCAGCGGTCGAAGTTCTTGCTAATGGATGCCCTTAAATTGAGCATT

GAAGACCCGAGCCATGAGGGGGAGGGTATACCGCTGTACGATGCAACCAAATGCATGAAG

ACATTTTTTGGCTGGAAGGAGCCCAACATCGTGAAACCACATGAAAAGGGCATAAACCCT

AATTACCTCCTGGCTTGGAAGCAGGTGCTATCAGAACTCCAAGATATTGAAAACGAGGAG

AAAATCCCAAAAACAAAGAACATGAAGAAGACGAGCCAACTGAAGTGGGCACTTGGTGAA

AACATGGCACCAGAGAAAGTGGACTTTGAGGACTGTAAAGATGTTAGCGATCTAAGACAG

TACGACAGTGACGAACCAGAGTCTAGATCACTAGCAAGTTGGATCCAGAGTGAATTTAAC

AAGGCTTGCGAACTGACAGATTCGAGTTGGATTGAACTTGATGAAATAGGGGAAGACGTT

GCTCCAATTGAACACATTGCGAGTATGAGGAGGAACTATTTCACAGCGGAAGTATCCCAT

TGCAGGGCTACAGAATACATAATGAAAGGAGTATACATAAACACCGCTTTATTGAATGCA

TCCTGTGCAGCCATGGATGACTTCCAACTGATTCCAATGATAAGCAAATGCAGAACCAAA

GAAGGGAGACGGAAGACAAATCTGTATGGCTTCATTATAAAAGGAAGATCCCATTTGAGG

AATGACACCGATGTGGTAAACTTTGTGAGCATGGAATTCTCTCTCACTGACCCAAGGTTG

GAGCCACACAAATGGGAAAAGTACTGTGTACTTGAGGTAGGAGACATGCTCCTACGGACT

GCAATAGGCCAAGTGTCAAGGCCCATGTTCCTGTATGTGAGAACCAATGGGACTTCTAAG

ATCAAAATGAAATGGGGTATGGAGATGAGGCGATGCCTTCTTCAATCCCTTCAACAAATT

GAGAGCATGATTGAAGCCGAGTCTTCTGTCAAAGAAAAAGACATGACCAAGGAATTCTTT

GAAAACAGATCAGAAACATGGCCAATTGGAGAATCACCCAAAGGAGTGGAGGAAGGCTCC

ATTGGGAAGGTGTGCAGAACATTGCTAGCAAAGTCTGTGTTCAACAGCCTATATGCATCT

CCACAACTCGAGGGGTTTTCAGCTGAATCAAGAAAGTTGCTTCTCATTGTTCAGGCACTT

AGGGACAACCTGGAACCTGGAACCTTCGATCTTGGGGGGCTATATGAAGCAATTGAGGAG

TGCCTGATTAACGATCCCTGGGTTTTGCTTAATGCGTCTTGGTTCAACTCCTTCCTCACA

CATGCACTGAAATAGTCGTGGCAATGCTACTA---TTTGCTATCCATACTGTCCAAAAAG

TACC-TTGTTTCTACT

>A_swan_Lithuania_1298PG1_21VIR2606-3_2021_EPI1858578

AGCRAAAGCAGGTACTGATCCAAAATGGAAGACTTTGTGCGACAATGCTTCAATCCAATG

ATTGTCGAGCTTGCGGAAAAGGCAATGAAAGAATATGGGGAAGATCCTAAGATCGAAACA

AACAAGTTTGCCGCAATATGTACACACTTAGAAGTCTGCTTCATGTATTCGGATTTCCAT

TTCATTGATGAACGAGGCGAATCAATAATCGTAGAATCTGGCGACCCGAATGCATTATTG

AAGCACCGATTTGAGATAATTGAAGGGAGAGACCGAACAATGGCCTGGACAGTGGTGAAT

AGTATATGCAACACTACAGGAGTCGAAAAGCCCAAGTTCCTTCCTGATTTGTATGACTAC

AGAGAAAACCGATTCATTGAAATTGGAGTAACGCGAAGGGAAGTTCACATATACTATCTT

GAAAAAGCCAACAAAATAAAATCAGAGAAAACACACATTCACATATTCTCATTCACTGGG

GAGGAAATGGCCACCAAGGCAGATTACACTCTTGATGAAGAGAGCAGAGCAAGAATAAAA

ACCAGGCTATTCACTATAAGACAAGAGATGGCCAATAGGGGTCTATGGGATTCCTTTCGT

CAGTCCGAGAGAGGCGAAGAGACAATTGAAGAAAGATTTGAAATCACAGGAACCATGCGC

AGGCTTGCTGACCAAAGTCTCCCACCGAACTTCGCCAGCCTTGAAAATTTTAGAGCCTAT

GTGGATGGATTTGAACCGAACGGCTGCATTGAGGGCAAGCTTTCTCAAATGTCAAAAGAA

GTGAATGCCAGAATTGAGCCATTTCTGAAGACTACACCACGCCCTCTCAGATTGCCTGAT

GGACCTCCCTGTTCCCAGCGGTCGAAGTTCTTGCTAATGGATGCCCTTAAATTAAGCATT

GAAGACCCGAGCCATGAGGGGGAGGGTATACCGCTGTACGATGCAACCAAATGCATGAAG

ACATTTTTTGGCTGGAAGGAGCCCAACATCGTGAAACCACATGAAAAGGGCATAAACCCT

AATTACCTCCTGGCTTGGAAGCAGGTGCTATCAGAACTCCAAGATATTGAAAACGAGGAG

AAAATCCCAAAAACAAAGAACATGAAGAAGACGAGCCAACTGAAGTGGGCACTTGGTGAA

AATATGGCACCAGAGAAAGTGGACTTTGAGGACTGTAAAGATGTTAGCGATCTAAAACAG

TACGACAGTGACGAACCAGAATCCAGATCACTAGCAAGTTGGATCCAGAGTGAATTTAAC

AAGGCTTGCGAACTGACAGATTCGAGTTGGATTGAACTTGATGAAATAGGGGAAGACGTT

GCTCCAATTGAACACATTGCGAGTATGAGGAGGAACTATTTCACAGCGGAAGTATCCCAT

TGCAGGGCTACAGAATACATAATGAAAGGAGTATACATAAACACAGCTTTATTGAATGCA

TCCTGTGCAGCCATGGATGACTTCCAACTGATTCCAATGATAAGCAAATGCAGAACCAAA

GAAGGGAGACGGAAGACAAATCTGTATGGCTTCATTATAAAAGGAAGATCCCATTTGAGG

AATGACACCGATGTGGTAAACTTTGTGAGCATGGAATTCTCTCTCACTGACCCAAGGCTG

GAGCCACACAAATGGGAAAAGTACTGTGTGCTTGAGGTGGGAGACATGCTCCTACGGACT

GCAATAGGCCAAGTGTCAAGGCCCATGTTCCTGTATGTGAGAACCAATGGGACTTCTAAG

ATCAAAATGAAATGGGGTATGGAGATGAGGCGATGCCTTCTTCAATCCCTTCAACAAATT

GAGAGCATGATTGAAGCCGAGTCTTCTGTCAAAGAAAAAGACATGACCAAGGAATTCTTT

GAAAACAGATCAGAAACATGGCCAATTGGAGAGTCACCCAAAGGAGTGGAGGAAGGCTCC

ATTGGGAAGGTGTGCAGAACATTGCTAGCAAAGTCTGTGTTCAATAGCCTATATGCATCT

CCACAACTCGAGGGGTTTTCAGCTGAATCAAGAAAGTTGCTTCTCATTGTTCAGGCACTT

AGGGACAACCTGGAACCTGGAACCTTCGATCTTGGAGGGCTATATGAAGCAATTGAGGAG

TGCCTGATTAACGATCCCTGGGTTTTGCTTAATGCGTCTTGGTTCAACTCCTTCCTCACA

CATGCACTGAAATAGTCGTGGCAATGCTGCTA---TTTGCTATCCATATTGTCCAAAAAA

GTACCTTGTTTCTACN

>A_chicken_Bulgaria_50-1_21VIR1454-9_2021_EPI1858618

AGCRAAAGCAGGTACTGATCCAAAATGGAAGACTTTGTGCGACAATGCTTCAATCCAATG

ATTGTCGAGCTTGCGGAAAAGGCAATGAAAGAATATGGGGAAGATCCTAAGATCGAAACA

AACAAGTTTGCCGCAATATGTACACACTTAGAAGTCTGCTTCATGTATTCGGATTTCCAT

TTCATTGATGAACGAGGCGAATCAATAATCATAGAATCTGGCGACCCGAATGCATTATTG

AAGCACCGATTTGAGATAATTGAAGGGAGAGACCGAACAATGGCCTGGACAGTGGTGAAT

AGTATATGCAACACTACAGGAGTCGAAAAGCCCAAGTTCCTTCCTGATTTGTATGACTAC

AGAGAAAACCGATTCATTGAAATTGGAGTAACGCGAAGGGAAGTTCACATATACTATCTT

GAAAAAGCCAACAAGATAAAATCAGAGAAAACACACATTCACATATTCTCATTCACTGGG

GAGGAAATGGCCACCAAGGCAGATTACACTCTTGATGAAGAGAGCAGAGCAAGAATAAAA

ACCAGGCTATTCACTATAAGACAAGAGATGGCCAATAGGGGTCTATGGGATTCCTTTCGT

CAGTCCGAGAGAGGCGAAGAGACAATTGAAGAAAGATTTGAAATCACAGGAACCATGCGC

AGGCTTGCTGACCAAAGTCTCCCACCGAACTTCGCCAGCCTTGAAAACTTTAGAGCCTAT

GTGGATGGATTTGAACCGAACGGCTGCATTGAGGGCAAGCTTTCTCAAATGTCAAAAGAA

GTGAATGCCAGAATTGAGCCATTTCTGAAGACGACACCACGCCCTCTCAGATTACCTGAT

GGACCTCCCTGTTCCCAGCGGTCGAAGTTCTTGCTAATGGATGCCCTTAAATTGAGCATT

GAAGACCCGAGCCATGAAGGGGAGGGTATACCGCTGTACGATGCAACCAAATGCATGAAG

ACATTTTTTGGCTGGAAGGAGCCCAACATCGTGAAACCACATGAAAAGGGCATAAACCCT

AATTACCTCCTGGCTTGGAAGCAGGTTCTATCAGAACTCCAAGATATTGAAAACGAGGAG

AAAATCCCAAAAACAAAGAACATGAAGAAGACGAGCCAACTGAAGTGGGCACTTGGTGAA

AACATGGCACCAGAGAGAGTGGACTTTGAGGACTGTAAAGATGTTAGCGATCTAAGACAG

TACGACAGTGACGAACCAGAGCCTAGATCACTAGCAAGTTGGATCCAGAGTGAATTTAAC

AAGGCTTGCGAACTGACAGATTCGAGTTGGATTGAACTTGATGAAATAGGGGAAGACGTT

GCTCCAATTGAACACATTGCGAGTATGAGGAGGAACTATTTCACAGCGGAAGTATCCCAT

TGCAGGGCTACAGAATACATAATGAAAGGAGTATACATAAACACAGCTTTATTGAATGCA

TCCTGTGCAGCCATGGATGACTTCCAACTGATTCCAATGATAAGCAAATGCAGAACCAAA

GAAGGGAGACGGAAGACAAATCTGTATGGCTTCATTATAAAAGGAAGATCCCATTTGAGG

AATGACACCGATGTGGTAAACTTTGTGAGCATGGAATTCTCTCTCACTGACCCAAGGCTG

GAGCCACACAAATGGGAAAAGTACTGTGTACTTGAGGTAGGAGACATGCTCCTACGGACT

GCAATAGGCCAAGTGTCAAGGCCCATGTTCCTGTATGTGAGAACCAATGGGACTTCTAAG

ATCAAAATGAAATGGGGTATGGAGATGAGGCGATGCCTTCTTCAATCCCTTCAACAAATT

GAGAGCATGATTGAAGCCGAGTCTTCTGTCAAAGAAAAAGACATGACCAAGGAATTCTTT

GAAAACAGATCAGAAACATGGCCAATTGGAGAGTCACCCAAAGGAGTGGAGGAAGGCTCC

ATTGGGAAGGTGTGCAGAACATTGCTAGCAAAGTCTGTGTTCAACAGCCTATATGCATCT

CCACAACTCGAGGGGTTTTCAGCTGAATCAAGAAAGTTGCTTCTCATTGTTCAGGCACTT

AGGGACAACCTGGAACCTGGAACCTTCGATCTTGGGGGGCTATATGAAGCAATTGAGGAG

TGCCTGATTAACGATCCCTGGGTTTTGCTTAATGCGTCTTGGTTCAACTCCTTCCTCACA

CATGCACTGAAATAGTCGTGGCAATGCTACTA---TTTGCTATCCATACTGTCCAAAAAA

GTACCTTGTTTCTACT

>A_mute_swan_Poland_MB189_2021_EPI1859654

-----------GTACTGATCCAAAATGGAAGACTTTGTGCGACAATGCTTCAATCCAATG

ATTGTCGAGCTTGCGGAAAAGGCAATGAAAGAATATGGGGAAGATCCTAAGATCGAAACA

AACAAGTTTGCCGCAATATGTACACACTTAGAAGTCTGCTTCATGTATTCGGATTTCCAT

TTCATTGATGAACGAGGCGAATCAATAATCGTAGAATCTGGCGACCCGAATGCATTATTG

AAGCACCGATTTGAGATAATTGAAGGGAGAGACCGAACAATGGCCTGGACAGTGGTGAAT

AGTATATGCAACACTACAGGAGTCGAAAAGCCCAAGTTCCTTCCTGATTTGTATGACTAC

AGAGAAAACCGATTCATTGAAATTGGAGTAACGCGAAGGGAAGTTCACATATACTATCTT

GAAAAAGCCAACAAGATAAAGTCAGAGAAAACACACATTCACATATTCTCATTCACTGGG

GAGGAAATGGCCACCAAGGCAGATTACACTCTTGATGAAGAGAGCAGAGCAAGAATAAAA

ACCAGGCTATTCACTATAAGACAAGAGATGGCCAATAGGGGTCTATGGGATTCCTTTCGT

CAGTCCGAGAGAGGCGAAGAGACAATTGAAGAAAGATTTGAAATCACAGGAACCATGCGC

AGGCTTGCTGACCAAAGTCTTCCACCGAACTTCGCCAGCCTTGAAAACTTTAGAGCCTAT

GTGGATGGATTTGAACCGAACGGCTGCATTGAGGGCAAGCTTTCTCAAATGTCAAAAGAA

GTGAATGCCAGAATTGAGCCATTTCTGAAGACAACACCACGCCCTCTCAGATTACCTGAT

GGACCTCCCTGTTCCCAGCGGTCGAAGTTCTTGCTAATGGATGCCCTTAAATTGAGCATT

GAAGACCCGAGCCATGAGGGGGAGGGTATACCGCTGTACGATGCAACCAAATGCATGAAG

ACATTTTTTGGCTGGAAGGAGCCCAACATCGTGAAACCACATGAAAAGGGCATAAACCCT

AATTACCTCCTGGCTTGGAAGCAGGTACTATCAGAACTCCAAGATATTGAAAACGAGGAG

AAAATCCCAAAAACAAAGAACATGAAGAAGACGAGCCAACTGAAGTGGGCACTTGGTGAA

AACATGGCACCAGAGAAAGTGGACTTTGAGGACTGTAAAGATGTTAGCGATCTAAAACAG

TACGACAGTGACGAACCAGAATCTAGATCACTAGCAAGTTGGATCCAGAGTGAATTTAAC

AAGGCTTGCGAACTGACAGATTCGAGTTGGATTGAACTTGATGAAATAGGGGAAGACGTT

GCTCCAATTGAACACATTGCGAGTATGAGGAGGAACTATTTCACAGCGGAAGTATCCCAT

TGCAGGGCTACAGAATACATAATGAAAGGAGTATACATAAACACAGCTTTATTGAATGCA

TCCTGTGCAGCCATGGATGACTTCCAACTGATTCCAATGATAAGCAAATGCAGAACCAAA

GAAGGGAGACGGAAGACAAATCTGTATGGCTTCATTATAAAAGGAAGATCCCATTTGAGG

AATGACACCGATGTGGTAAACTTTGTGAGCATGGAATTCTCTCTCACTGACCCAAGGCTG

GAGCCACACAAATGGGAAAAGTACTGTGTGCTTGAGGTAGGAGACATGCTCCTACGGACT

GCAATAGGCCAAGTGTCAAGGCCCATGTTTCTGTATGTGAGAACCAATGGGACTTCTAAG

ATCAAAATGAAATGGGGTATGGAGATGAGGCGATGCCTTCTTCAATCCCTTCAACAAATT

GAGAGCATGATTGAAGCCGAGTCTTCTGTCAAAGAAAAAGACATGACCAAGGAATTCTTT

GAAAACAGATCAGAAACATGGCCAATTGGAGAGTCACCCAAAGGAGTGGAGGAAGGCTCC

ATTGGGAAGGTGTGCAGAACATTGCTAGCAAAGTCTGTGTTCAACAGCCTATATGCATCT

CCACAACTCGAGGGGTTTTCAGCTGAATCAAGAAAGTTGCTTCTCATTGTTCAGGCACTT

AGGGACAACCTGGAACCTGGAACCTTCGATCTTGGGGGGCTATATGAAGCAATTGAGGAG

TGCCTGATTAACGATCCCTGGGTTTTGCTTAATGCGTCTTGGTTCAACTCCTTCCTCACA

CATGCACTGAAATAGTCGTGGCAATGCTACTA---TTTGCTATCCATACTGTCCAAAAAA

GTA-------------

>A_mute_swan_Poland_MB272_2021_EPI1859670

-----------GTACTGATCCAAAATGGAAGACTTTGTGCGACAATGCTTCAATCCAATG

ATTGTCGAGCTTGCGGAAAAGGCAATGAAAGAATATGGGGAAGATCCTAAGATCGAAACA

AACAAGTTTGCCGCAATATGTACACACTTAGAAGTCTGCTTCATGTATTCGGATTTCCAT

TTCATTGATGAACGAGGCGAATCAATAATCGTAGAATCTGGCGACCCGAATGCATTATTG

AAGCACCGATTTGAGATAATTGAAGGGAGAGACCGAACAATGGCCTGGACAGTGGTGAAT

AGTATATGCAACACTACAGGAGTCGAAAAGCCCAAGTTCCTTCCTGATTTGTATGACTAC

CGAGAAAACCGATTCATTGAAATTGGAGTAACGCGAAGGGAAGTTCACATATACTATCTT

GAAAAAGCCAACAAGATAAAATCAGAGAAAACACACATTCACATATTCTCATTCACTGGG

GAGGAAATGGCCACCAAGGCAGATTACACTCTTGATGAAGAGAGCAGAGCAAGAATAAAA

ACCAGGCTATTCACTATAAGACAAGAGATGGCCAATAGGGGTCTATGGGATTCCTTTCGT

CAGTCCGAGAGAGGCGAAGAGACAATTGAAGAAAGATTTGAAATCACAGGAACCATGCGC

AGGCTTGCTGACCAAAGTCTCCCACCGAACTTCGCCAGCCTTGAAAACTTTAGAGCCTAT

GTGGATGGATTTGAACCGAACGGCTGCATTGAGGGCAAGCTTTCTCAAATGTCAAAAGAA

GTGAATGCCAGAATTGAGCCATTTCTGAAGACGACACCACGCCCTCTCAGATTACCTGAT

GGACCTCCCTGTTCCCAGCGGTCGAAGTTCTTGCTAATGGATGCCCTTAAATTGAGCATT

GAAGACCCGAGCCATGAGGGGGAGGGTATACCGCTGTACGATGCAACCAAATGCATGAAG

ACATTTTTTGGCTGGAAGGAGCCCAACATCGTGAAACCACATGAAAAGGGCATAAACCCT

AATTACCTCCTGGCTTGGAAGCAGGTGCTATCAGAACTCCAAGATATTGAAAACGAGGAG

AAAATCCCAAAAACAAAGAACATGAAGAAGACGAGCCAACTGAAGTGGGCACTTGGTGAA

AACATGGCACCAGAGAAAGTGGACTTTGAGGACTGTAAAGATGTTAGCGATCTAAGACAG

TACGACAGTGACGAACCAGAGTCTAGATCACTAGCAAGTTGGATCCAGAGTGAATTTAAC

AAGGCTTGCGAACTGACAGATTCGAGTTGGATTGAACTTGATGAAATAGGGGAAGACGTT

GCTCCAATTGAACACATTGCGAGTATGAGGAGGAACTATTTCACAGCGGAAGTATCCCAT

TGCAGGGCTACAGAATACATAATGAAAGGAGTATACATAAACACAGCTTTATTGAATGCA

TCCTGTGCAGCCATGGATGACTTCCAACTGATTCCAATGATAAGCAAATGCAGAACCAAA

GAAGGGAGACGGAAGACAAATCTGTATGGCTTCATTATAAAAGGAAGATCCCATTTGAGG

AATGACACCGATGTGGTAAACTTTGTGAGCATGGAATTCTCTCTCACTGACCCAAGGCTG

GAGCCACACAAATGGGAAAAGTACTGTGTACTTGAGGTAGGAGACATGCTCCTACGGACT

GCAATAGGCCAAGTGTCAAGGCCCATGTTCCTGTATGTGAGAACCAATGGGACTTCTAAG

ATCAAAATGAAATGGGGTATGGAGATGAGGCGATGCCTTCTTCAATCCCTTCAACAAATT

GAGAGCATGATTGAAGCCGAGTCTTCTGTCAAAGAAAAAGACATGACCAAGGAATTCTTT

GAAAACAGATCAGAAACATGGCCAATTGGAGAATCACCCAAAGGAGTGGAGGAAGGCTCC

ATTGGGAAGGTGTGCAGAACATTGCTAGCAAAGTCTGTGTTCAACAGCCTATATGCATCT

CCACAACTCGAGGGGTTTTCAGCTGAATCAAGAAAGTTGCTTCTCATTGTTCAGGCACTT

AGGGACAACCTGGAACCTGGAACCTTCGATCTTGGGGGGCTATATGAAGCAATTGAGGAG

TGCCTGATTAACGATCCCTGGGTTTTGCTTAATGCGTCTTGGTTCAACTCCTTCCTCACA

CATGCACTGAAATAGTTGTGGCAATGCTACTA---TTTGCTATCCATACTGTCCAAAAAA

GTA-------------

>A_anser_anser_Spain_297-1_21VIR1230-5_2021_EPI1860067

---------------TGATCCAAAATGGAAGACTTTGTGCGACAATGCTTCAATCCAATG

ATTGTCGAGCTTGCGGAAAAGGCAATGAAAGAATATGGGGAAGATCCTAAGATCGAAACA

AACAAGTTTGCCGCAATATGCACACACTTAGAAGTCTGCTTCATGTATTCGGATTTCCAT

TTCATTGATGAACGAGGCGAATCAATAATCGTAGAATCTGGCGACCCGAATGCATTATTG

AAGCACCGATTTGAGATAATTGAAGGGAGAGACCGAACAATGGCCTGGACAGTGGTGAAT

AGTATATGCAACACTACAGGAGTCGAAAAGCCCAAGTTCCTTCCTGATTTGTATGACTAC

AGAGAAAACCGATTCATTGAAATTGGAGTAACGCGAAGGGAAGTTCACATATACTATCTT

GAAAAAGCCAACAAGATAAAATCAGAGAAAACACACATTCACATATTCTCATTCACTGGG

GAGGAAATGGCCACCAAGGCCGATTACACTCTTGATGAAGAGAGCAGAGCAAGAATAAAA

ACCAGGCTATTCACTATAAGACAAGAGATGGCCAATAGGGGTCTATGGGATTCCTTTCGT

CAGTCCGAGAGAGGCGAAGAGACAATTGAAGAAAGATTTGAAATCACAGGAACCATGCGC

AGGCTTGCTGACCAAAGTCTCCCACCGAACTTCGCCAGCCTTGAAAACTTTAGAGCCTAT

GTGGATGGATTTGAACCGAACGGCTGCATTGAGGGCAAGCTTTCTCAAATGTCAAAAGAA

GTGAATGCCAGAATTGAGCCATTTCTGAAGACGACACCACGCCCTCTCAGATTACCTGAT

GGACCTCCCTGTTCCCAGCGGTCGAAGTTCTTGCTAATGGATGCCCTTAAATTGAGCATT

GAAGACCCGAGCCATGAGGGGGAGGGTATACCGCTGTACGATGCAACCAAATGCATGAAG

ACATTTTTTGGCTGGAAGGAGCCCAACATCGTGAAACCACATGAAAAGGGCATAAACCCT

AATTACCTCCTGGCTTGGAAGCAGGTGCTATCAGAACTCCATGATATTGAAAACGAGGAG

AAAATCCCAAAAACAAAGAACATGAAGAAGACGAGCCAACTGAAGTGGGCACTTGGTGAA

AACATGGCACCAGAGAAAGTGGACTTTGAAGACTGTAAAGATGTTAGCGATCTAAGACAG

TACGACAGTGACGAACCAGAGTCTAGATCACTAGCAAGTTGGATCCAGAGTGAATTTAAC

AAGGCTTGCGAACTGACAGATTCGAGTTGGATTGAACTTGATGAAATAGGGGAAGACGTT

GCTCCAATTGAACACATTGCGAGTATGAGGAGGAACTATTTCACAGCGGAAGTATCCCAT

TGCAGGGCTACAGAATATATAATGAAAGGAGTATACATAAACACAGCTTTATTGAATGCA

TCCTGTGCAGCCATGGATGACTTCCAACTGATTCCAATGATAAGCAAATGCAGAACCAAA

GAAGGGAGACGGAAGACAAATCTGTATGGCTTCATTATAAAAGGAAGATCCCATTTGAGG

AATGACACCGATGTGGTAAACTTTGTGAGCATGGAATTCTCTCTCACTGACCCAAGGCTG

GAGCCACACAAATGGGAAAAGTACTGTGTGCTTGAGGTAGGAGACATGCTCCTACGGACT

GCAATAGGCCAAGTGTCAAGGCCCATGTTCCTGTATGTGAGAACCAATGGGACTTCTAAG

ATCAAAATGAAATGGGGTATGGAGATGAGGCGATGCCTTCTTCAATCCCTTCAACAAATT

GAGAGCATGATTGAAGCCGAGTCTTCTGTCAAAGAAAAAGACATGACCAAGGAATTCTTT

GAAAACAGATCAGAAACATGGCCAATTGGAGAGTCACCCAAAGGAGTGGAGGAAGGCTCC

ATTGGGAAGGTGTGCAGAACATTGCTAGCAAAGTCTGTGTTCAACAGCCTATATGCATCT

CCACAACTCGAGGGGTTTTCAGCTGAATCAAGAAAGTTGCTTCTCATTGTTCAGGCACTT

AGGGACAACCTGGAACCTGGAACCTTCGATCTTGGGGGGCTATATGAAGCAATTGAGGAG

TGCCTGATTAACGATCCCTGGGTTTTGCTTAATGCGTCTTGGTTCAACTCCTTCCTCACA

CATGCACTGAAATAGTCGGGGCAATGCTACTA---TTTGCTATCCATACTGTCCAAAA--

----------------

>A_ciconia_ciconia_Spain_102-1_21VIR1230-2_2021_EPI1860075

---------------TGATCCAAAATGGAAGACTTTGTGCGACAATGCTTCAATCCAATG

ATTGTCGAGCTTGCGGAAAAGGCAATGAAAGAATATGGGGAAGATCCTAAGATCGAAACA

AACAAGTTTGCCGCAATATGTACACACTTAGAAGTCTGCTTCATGTATTCGGATTTCCAT

TTCATTGATGAACGAGGCGAATCAATAATCGTAGAATCTGGCGACCCGAATGCATTATTG

AAGCACCGATTTGAGATAATTGAAGGGAGAGACCGAACAATGGCCTGGACAGTGGTGAAT

AGTATATGCAACACTACAGGAGTCGAAAAGCCCAAGTTCCTTCCTGATTTGTATGACTAC

AGAGAAAACCGATTCATTGAAATTGGAGTAACGCGAAGGGAAGTTCACATATACTATCTT

GAAAAAGCCAACAAGATAAAGTCAGAGAAAACACACATTCACATATTCTCATTCACTGGG

GAGGAAATGGCCACCAAGGCAGATTACACTCTTGATGAAGAGAGCAGAGCAAGAATAAAA

ACCAGGCTATTCACTATAAGACAAGAGATGGCCAATAGGGGTCTATGGGATTCCTTTCGT

CAGTCCGAGAGAGGCGAAGAGACAATTGAAGAAAGATTTGAAATCACAGGAACCATGCGC

AGGCTTGCTGACCAAAGTCTCCCACCGAACTTCGCCAGCCTTGAAAACTTTAGAGCCTAT

GTGGATGGATTTGAACCGAACGGCTGCATTGAGGGCAAGCTTTCTCAAATGTCAAAAGAA

GTGAATGCCAGAATTGAGCCATTTCTGAAGACGACACCACGCCCTCTCAGATTACCTGAT

GGACCTCCCTGTTCCCAGCGGTCGAAGTTCTTGCTAATGGATGCCCTTAAATTAAGCATT

GAAGACCCGAGCCATGAGGGGGAGGGTATACCGCTGTACGATGCAACCAAATGCATGAAG

ACATTTTTTGGCTGGAAGGAGCCCAACATCGTGAAACCACATGAAAAGGGCATAAACCCT

AATTACCTCCTGGCTTGGAAGCAGGTGCTATCAGAACTCCAAGATATTGAAAACGAGGAG

AAAATCCCAAAAACAAAGAACATGAAGAAGACGAGCCAACTGAAGTGGGCACTTGGTGAA

AACATGGCACCAGAGAAAGTGGACTTTGAGGACTGTAAAGATGTTAGCGATCTAAGACAG

TACGACAGTGACGAACCAGAGTCTAGATCACTAGCAAGTTGGATCCAGAGTGAATTTAAC

AAGGCTTGCGAACTGACAGATTCGAGTTGGATTGAACTTGATGAAATAGGGGAAGACGTT

GCTCCAATTGAACACATTGCGAGTATGAGGAGGAACTATTTCACAGCGGAAGTATCCCAT

TGCAGGGCTACAGAATACATAATGAAAGGAGTATACATAAACACAGCTTTATTGAATGCA

TCCTGTGCAGCCATGGATGACTTCCAACTGATTCCAATGATAAGCAAATGCAGAACCAAA

GAAGGGAGACGGAAGACAAATCTGTATGGCTTCATTATAAAAGGAAGATCCCATTTGAGG

AATGACACCGATGTGGTAAACTTTGTGAGCATGGAATTCTCTCTCACTGACCCAAGGCTG

GAGCCACACAAATGGGAAAAGTACTGTGTACTTGAGGTAGGAGACATGCTCCTACGGACT

GCAATAGGCCAAGTGTCAAGGCCCATGTTCCTGTATGTGAGAACCAATGGGACTTCTAAG

ATCAAAATGAAATGGGGTATGGAGATGAGGCGATGCCTTCTTCAATCCCTTCAACAAATT

GAGAGCATGATTGAAGCCGAGTCTTCTGTCAAAGAAAAAGACATGACCAAGGAATTCTTT

GAAAACAGATCAGAAACATGGCCAATTGGAGAGTCACCCAAAGGAGTGGAGGAAGGCTCC

ATTGGGAAGGTGTGCAGAACATTGCTAGCAAAGTCTGTGTTCAACAGCCTATATGCATCT

CCACAACTCGAGGGGTTTTCAGCTGAATCAAGAAAGTTGCTTCTCATTGTTCAGGCACTT

AGGGACAACCTGGAACCTGGAACCTTCGATCTTGGGGGGCTATATGAAGCAATTGAGGAG

TGCCTGATTAACGATCCCTGGGTTTTGCTTAATGCGTCTTGGTTCAACTCCTTCCTCACA

CATGCACTGAAATAGTCGTGGCAATGCTACTA---TTTGCTATCCATACTGTCCAAAA--

----------------

>A_duck_Jiangsu_k1203_2010_EPI442016

------------------------------------------------------CCAATG

ATCGTCGAGCTTGCGGAAAAGGCAATGAAAGAATATGGGGAAAATCCAAAAATCGAAACG

AACAAATTCGCAGCAATATGCACTCACTTAGAAGTCTGTTTCATGTATTCGGATTTCCAC

TTTATTGATGAACGAGGTGAATCAATGATTGTAGAATCTGGCGATCCGAATGCATTATTG

AAACACCGATTTGAGATAATTGAAGGGAGAGACCGAACAATGGCTTGGACAGTGGTAAAT

AGTATCTGCAACACCACAGGAGTCGATAAGCCTAAATTCCTCCCAGATTTGTATGATTAC

AAAGAGAACCGATTCATTGAAATTGGAGTGACAAGGAGGGAAGTTCACACATACTACCTA

GAAAAGGCAAATAAGATAAAATCAGAGAAGACACACATTCACATATTCTCATTCACTGGA

GAGGAGATGGCCACCAAAGCTGACTATACCCTTGATGAAGAGAGCAGAGCAAGGATCAAA

ACCAGGCTGTTCACTATCAGGCAAGAAATGGCCAATAGGGGTCTATGGGATTCCTTTCGT

CAATCCGAGAGAGGCGAAGAGACAATTGAAGAAAGATTTGAAATCACAGGAACCATGCGC

AGGCTTGCTGACCAAAGTCTCCCGCCGAATTTCTCCAGCCTTGAAAATTTTAGAGCCTAT

GTGGATGGATTCAAACCGAACGGCTGCATTGAGGGCAAGCTTTCTCAAATGTCAAAAGAA

GTGAACGCCAGAATTGAGCCATTTATGAAGACAACACCACGCCCTCTCAGATTACCTGAT

GGGCCTCCTTGTTCTCAGCGGTCGAAATTCTTACTGATGGATGCCCTTAAATTAAGCATC

GAAGATCCAAGCCATGAGGGAGAAGGTATACCGCTATATGATGCAATCAAATGCATGAAG

ACATTTTTTGGTTGGAAAGAGCCCAACATCGTAAAACCACATGTAAAAGGCATAAATCCC

AACTACCTCCTGGCTTGGAAGCAGGTGCTGGCAGAACTCCAAGACATTGAAAATGAGGAG

AAAATCCCAAAAACAAAGAACATGAAGAAAACAAGCCAATTGAAGTGGGCACTTGGTGAG

AACATGGCACCTGAAAAGGTGGACTTTGATGACTGCAGAGATGTTAGCGATCTAAGACAG

TATGACAGTGATGAACCAGAGCCCAGATCATTATCAAGCTGGATCCAGAGCGAATTCAAC

AAAGCATGCGAATTGACAGATTCAAGTTGGATTGAACTTGATGAAATAGGAGAAGATGTT

GCTCCAATTGAGCACATTGCGAGTATGAGACGAAACTATTTCACAGCGGAAGTGTCTCAT

TGCAGGGCTACTGAGTATATAATGAAAGGAGTGTATGCAAATACAGCTCTGTTGAATGCA

TCCTGTGCAGCCATGGATGATTTCCAATTGATTCCAATGATAAGCAAGTGCAGAACCAAA

GAAGGAAGACGGAAGACAAATCTATATGGATTCATTATAAAAGGAAGATCCCATTTGAGG

AATGACACCGATGTGGTAAACTTTGTGAGCATGGAGTTTTCTCTTACTGACCCGAGGCTG

GAACCACACAAGTGGGAGAAGTACTGTGTTCTCGAGATAGGAGACATGCTCCTACGAACT

GCAATAGGCCAAGTGTCAAGACCCATGTTTCTATATGTGAGAACCAATGGGACTTCCAAG

ATCAAGATGAAATGGGGCATGGAGATGAGGCGATGCCTTCTTCAATCCCTTCAACAAATT

GAGAGCATGATTGAGGCAGAGTCTTCTGTCAAAGAGAAAGACATGACCAAGGAATTCTTT

GAGAACAAATCAGAAACTTGGCCAATTGGGGAATCACCTAAGGGGGTGGAGGAAAGCTCC

ATTGGGAAGGTGTGCAGAACATTACTAGCAAAATCTGTATTCAACAGCCTATATGCGTCT

CCACAACTTGAGGGGTTTTCAGCTGAATCAAGAAAATTACTTCTCATTGTTCAGGCATTT

AGGGACAACCTGGAACCTGGGACCTTCGATCTTGGGGGGCTATATGAAGCAATTGAGGAG

TGCCTGATTAATGATCCCTGGGTTTTGCTTAATGCGTCTTGGTTCAACTCCTTCCTCACA

CATGCACTGAAATAGTTGTGGCAATGC---------------------------------

----------------

>A_Turkey_Egypt_AR550_2018_EPI1420341

------------TACTGATCAAAAATGGAAGACTTTGTGCGACAATGCTTCAATCCAATG

ATTGTCGAGCTTGCGAAAAAGGCAATGAAAGAATATGGGGAAGATCCAAAAATCGAAACA

AACAAGTTTGCTGCAATATGCACACACTTAGAAGTCTGCTTCATGTATTCGGATTTCCAC

TTTATTGATGAACGAGGCGAATCAATAATCGTAGAATCTGGCGATCCGAATGCATTATTG

AAACACCGATTTGAGATAATTGAAGGGAGAGACCGAACAATGGCCTGGACAGTGGTGAAT

AGTATATGCAACACTACAGGAGTCGAAAAACCCAAGTTCCTCCCTGATTTGTATGACTAC

AGAGAAAACCGATTCATTGAAATTGGAGTAACGCGAAGGGAAGTTCACATATACTATCTA

GAAAAAGCCAACAAGATAAAATCAGAGAAAACACACATTCACATATTCTCATTCACTGGG

GAGGAAATGGCCACCAAGGCAGATTACACTCTTGATGAAGAGAGCAGAGCAAGAATAAAA

ACCAGGCTATTCACTATAAGACAAGAGATGGCCAATAGGGGTCTATGGGATTCCTTTCGT

CAGTCCGAGAGAGGCGAAGAGACAATTGAAGAAAGATTTGAAATCACAGGAACCATGCGC

AGGCTTGCCGACCAAAGTCTCCCACCGAACTTCTCCAGCCTTGAAAACTTTAGAGCCTAT

GTGGATGGATTCGAACCGAACGGCTGCATTGAGGGCAAGCTTTCTCAAATGTCAAAAGAA

GTGAATGCCAGAATTGAGCCATTCCTGAAGACAACACCACGCCCTCTCAGATTACCTGAT

GGGCCTCCCTGTTCCCAGCGGTCGAAGTTCTTGCTAATGGATGCCCTTAAATTGAGCATT

GAAGACCCGAGCCATGAGGGGGAGGGTATACCGCTGTATGATGCAACCAAATGCATGAAG

ACATTTTTTGGCTGGAAAGAGCCCAACATCGTGAAACCACATGAAAAGGGCATAAACCCT

AATTACCTCCTGGCTTGGAAGCAGGTGCTAGCAGAACTCCAAGATATTGAAAACGAGGAG

AAAATCCCAAAAACAAAGAACATGAAGAAGACAAGCCAACTGAAGTGGGCACTTGGTGAA

AACATGGCACCAGAGAAAGTGGACTTTGAGGACTGTAAAGATGTTAGCGATCTAAGACAG

TACGACAGTGACGAACCAGAGTCTAGATCACTAGCAAGTTGGATCCAGAGTGAATTTAAC

AAGGCTTGCGAATTGACAGATTCAAGTTGGATTGAACTTGATGAAATAGGGGAAGACGTT

GCTCCAATTGAACACATTGCGAGTATGAGGAGGAACTATTTCACAGCGGAAGTATCCCAT

TGCAGGGCTACAGAATACATAATGAAAGGAGTATACATAAACACAGCCTTATTGAATGCA

TCCTGTGCAGCCATGGATGACTTCCAACTGATTCCAATGATAAGCAAATGCAGAACCAAA

GAAGGGAGACGGAAGACAAATCTGTATGGCTTCATTATAAAAGGAAGATCCCATTTGAGG

AATGACACCGATGTGGTAAACTTTGTGAGCATGGAATTCTCTCTAACTGACCCAAGGCTG

GAGCCATACAAATGGGAAAAATACTGTGTTCTTGAGATAGGAGACATGCTCCTACGGACT

GCAATAGGCCAAGTGTCAAGGCCCATGTTCCTGTATGTGAGAACCAATGGGACTTCCAAG

ATCAAAATGAAATGGGGCATGGAGATGAGGCGATGCCTTCTTCAATCCCTTCAACAAATT

GAGAGCATGATTGAAGCCGAGTCTTCTGTCAAAGAAAAGGACATGACCAAGGAATTCTTT

GAAAACAGATCGGAAACATGGCCAATTGGAGAATCACCCAAAGGAGTGGAGGAAGGCTCC

ATTGGGAAGGTGTGCAGAACGTTGCTAGCAAAGTCTGTGTTCAACAGCCTATATGCATCT

CCACAACTCGAGGGGTTTTCAGCTGAATCAAGAAAGTTGCTTCTCATTGTCCAGGCACTT

AGGGACAACCTGGAACCTGGAACCTTCGATCTTGGGGGGCTATATGAAGCAATTGAGGAG

TGCCTGATTAACGATCCCTGGGTTTTGCTTAATGCGTCTTGGTTCAACTCCTTCCTCACA

CATGCACTGAAATAGTTGTGGCAATGCTACTA---TTTGCTATCCATACTGTCCAAAAAA

GTA-------------

>A_Chicken_Egypt_AI20286_2019_EPI1638784

------------------------ATGGAAGACTTTGTGCGACAATGCTTCAATCCAATG

ATTGTCGAGCTTGCGGAAAAGGCAATGAAAGAATATGGGGAAGATCCAAAAATCGAAACA

AACAAGTTTGCCGCAATATGCACACACTTAGAAGTCTGCTTCATGTATTCGGATTTCCAC

TTTATTGATGAACGAGGCGAATCAATAATCGTAGAATCTGGCGATCCGAATGCATTATTG

AAACACCGATTTGAGATAATTGAAGGGAGAGACCGAACAGTGGCCTGGACAGTGGTGAAT

AGCATATGCAACACTACGGGAGTCGAAAAGCCCAAGTTCCTTCCTGATTTGTATGACTAC

AGAGAGAACCGATTCATTGAAGTTGGAGTAACGCGAAGGGAAGTTCACATATACTATCTA

GAAAAAGCCAACAAGATAAAATCAGAGAAAACACACATTCACATATTCTCATTCACTGGG

GAGGAAATGGCCACCAAGGCAGATTACACTCTTGATGAAGAGAGCAGAGCAAGGATAAAA

ACCAGGCTATTCACTATAAGACAAGAGATGGCCAATAGGGGTCTATGGGATTCCTTTCGT

CAGTCCGAGAGAGGCGAAGAGACAATTGAAGAAAGATTTGAAATCACAGGAACCATGCGC

AGGCTTGCCGACCAAAGTCTCCCACCGAACTTCTCCAGCCTTGAAAACTTTAGAGCCTAT

GTGGATGGATTCGAACCGAACGGCTGCATTGAGGGCAAGCTTTCTCAAATGTCAAAAGAA

GTGAATGCCAGAATTGAGCCATTTCTGAAGACAACACCACGCCCTCTCAGATTACCTGAT

GGGCCTCCCTGTTCCCAGCGGTCGAAGTTCTTGCTAATGGATGCCCTTAAATTGAGCATT

GAAGACCCGAGCCATGAGGGGGAGGGTATACCGCTGTATGATGCAACCAAATGCATGAAG

ACATTTTTTGGCTGGAAAGAGCCCAACATCGTGAAACCACATGAAAAGGGCATAAATCCT

AATTACCTCTTAGCTTGGAAGCAAGTGCTAGCAGAACTCCAAGATATTGAAAACGAGGAG

AAAATCCCAAAAACAAAGAACATGAAAAAGACAAGCCAACTGAAGTGGGCACTTGGTGAA

AACATGGCACCAGAGAAAGTGGACTTTGAGGACTGTAAAGATGTTAGCGACCTAAGACAG

TACGACAGTGACGAACCAGAGTCTAGATCACTAGCAAGTTGGATCCAGAGTGAATTTAAT

AAGGCTTGCGAATTGACAGATTCGAGTTGGATTGAACTTGATGAAATAGGGGAAGACGTT

GCTCCAATTGAACACATCGCGAGTATGAGGAGGAACTATTTCACAGCGGAAGTATCCCAT

TGCAGGGCTACAGAATACATAATGAAAGGAGTATACATAAATACAGCCTTATTGAATGCA

TCCTGTGCAGCCATGGATGACTTCCAACTGATTCCAATGATAAGCAAATGCAGAACCAAA

GAAGGGAGACGGAAGACAAATCTGTATGGCTTCATTATAAAAGGAAGATCCCATTTGAGG

AATGACACCGATGTGGTAAACTTTGTGAGCATGGAATTCTCTCTCACTGACCCAAGGCTG

GAGCCACACAAATGGGAGAAATACTGTGTTCTTGAGATAGGAGACATGCTCCTACGGACT

GCAATAGGCCAAGTGTCGAGGCCCATGTTCCTGTATGTGAGAACCAATGGGACTTCCAAG

ATCAAAATGAAATGGGGCATGGAGATGAGGCGATGCCTTCTTCAATCCCTTCAACAAATT

GAGAGCATGATTGAAGCCGAGTCTTCTGTCAAAGAAAAGGACATGACCAAGGAATTCTTT

GAAAACAGATCAGAAACATGGCCAATTGGAGAATCACCCAAAGGAGTGGAGGAAGGCTCC

ATTGGGAAGGTGTGCAGAACATTGCTAGCAAAGTCTGTGTTCAACAGCCTATATGCATCT

CCACAACTCGAGGGGTTTTCAGCTGAATCAAGAAAGTTGCTTCTCATTGTTCAGGCACTT

AGGGACAACCTGGAACCTGGAACCTTCGATCTTGGGGGGCTATATGAAGCAATTGAGGAG

TGCCTGATTAACGATCCCTGGGTTTTGCTTAATGCGTCTTGGTTCAACTCCTTCCTCACA

CATGCACTGAAATAG---------------------------------------------

----------------

>A_Turkey_Egypt_AI20285_2019_EPI1638795

------------------------ATGGAAGACTTTGTGCGACAATGCTTCAATCCAATG

ATTGTCGAGCTTGCGGAAAAGGCAATGAAAGAATATGGGGAAGATCCAAAAATCGAAACA

AACAAGTTTGCCGCAATATGCACACACTTAGAAGTCTGCTTCATGTATTCGGATTTCCAC

TTTATTGATGAACGAGGCGAATCAATAATCGTAGAATCTGGCGATCCGAATGCATTATTG

AAACACAGATTTGAGATAATTGAAGGGAGAGACCGAACAATGGCCTGGACAGTGGTGAAT

AGCATATGCAACACTACAGGAGTCGAAAAGCCCAAGTTCCTTCCTGATTTGTATGACTAC

AGAGAAAACCGGTTCATTGAAGTTGGAGTAACGCGAAGGGAAGTTCACATATACTATCTA

GAAAAAGTCAACAAGATAAAATCAGAGAAAACACACATTCACATATTCTCATTCACTGGG

GAGGAAATGGCCACCAAGGCAGATTACACTCTTGATGAAGAGAGCAGAGCAAGAATAAAA

ACTAGGCTGTTCACTATAAGACAAGAGATGGCCAATAGGGGTCTATGGGATTCCTTTCGT

CAGTCCGAGAGAGGCGAAGAGACAATTGAAGAAAGATTTGAAATCACAGGAACCATGCGC

AGGCTTGCCGACCAAAGTCTCCCACCGAACTTCTCCAGCCTTGAAAACTTTAGAGCCTAT

GTGGATGGATTCGAACCGAACGGCTGCATTGAGGGCAAGCTTTCTCAAATGTCAAAAGAA

GTGAATGCCAGAATCGAGCCATTTCTGAAAACAACACCACGCCCTCTCAGATTACCTGAT

GGGCCTCCCTGTTCCCAGCGGTCGAAGTTCTTGCTAATGGATGCCCTTAAATTGAGCATT

GAAGACCCGAGCCATGAGGGGGAGGGTATACCGCTGTATGATGCAACCAAATGCATGAAG

ACATTTTTTGGCTGGAAAGAGCCCAACATCGTGAAACCACATGAAAAGGGCATAAATCCT

AATTACCTCTTAGCTTGGAAGCAAGTGCTAGCAGAACTCCAAGATATTGAAAACGAGGAG

AAAATCCCAAAAACAAAGAACATGAAAAAGACAAGCCAACTGAAGTGGGCACTTGGTGAA

AACATGGCACCAGAGAAAGTGGACTTTGAGGACTGTAAAGATGTTAGCGACCTAAGACAG

TACGACAGTGACGAACCAGAGTCTAGATCACTAGCAAGTTGGATCCAGAGTGAATTTAAT

AAGGCTTGCGAATTGACAGATTCGAGTTGGATTGAACTTGATGAAATAGGGGAAGACGTT

GCTCCAATTGAACACATCGCGAGTATGAGGAGGAACTATTTCACAGCGGAAGTATCCCAT

TGCAGGGCTACAGAATACATAATGAAAGGAGTATACATAAATACAGCCTTATTGAATGCA

TCCTGTGCAGCCATGGATGACTTCCAACTGATTCCAATGATAAGCAAATGCAGAACCAAA

GAAGGGAGACGGAAGACAAATCTGTATGGCTTCATTATAAAAGGAAGATCCCATTTGAGG

AATGACACCGATGTGGTAAACTTTGTGAGCATGGAATTCTCTCTCACTGACCCAAGGCTG

GAGCCACACAAATGGGAGAAATACTGTGTTCTTGAGATAGGAGACATGCTCCTACGGACT

GCAATAGGCCAAGTGTCGAGGCCCATGTTCCTGTATGTGAGAACCAATGGGACTTCCAAG

ATCAAAATGAAATGGGGCATGGAGATGAGGCGATGCCTTCTTCAATCCCTTCAACAAATT

GAGAGCATGATTGAAGCCGAGTCTTCTGTCAAAGAAAAGGACATGACCAAGGAATTCTTT

GAAAACAGATCAGAAACATGGCCAATTGGAGAATCACCCAAAGGAGTGGAGGAAGGCTCC

ATTGGGAAGGTGTGCAGAACATTGCTAGCAAAGTCTGTGTTCAACAGCCTATATGCATCT

CCACAACTCGAGGGGTTTTCAGCTGAATCAAGAAAGTTGCTTCTCATTGTTCAGGCACTT

AGGGACAACCTGGAACCTGGAACCTTCGATCTTGGGGGGCTATATGAAGCAATTGAGGAG

TGCCTGATTAACGATCCCTGGGTTTTGCTTAATGCGTCTTGGTTCAACTCCTTCCTCACA

CATGCACTGAAATAG---------------------------------------------

----------------

>A_Chicken_Egypt_AR553_2018_EPI1639001

------------------------ATGGAAGACTTTGTGCGACAATGCTTCAATCCAATG

ATTGTCGAGCTTGCGGAAAAGGCAATGAAAGAATATGGGGAAGATCCAAAAATCGAAACA

AACAAGTTTGCCGCAATATGCACACACTTAGAAGTCTGCTTCATGTATTCGGATTTCCAC

TTTATTGATGAACGAGGCGAATCAATAATCGTAGAATCTGGCGATCCGAATGCATTATTG

AAACACCGATTTGAGATAATTGAAGGGAGAGACCGAACAATGGCCTGGACAGTGGTGAAT

AGTATATGCAACACTACAGGAGTCGAAAAGCCCAAGTTCCTTCCTGATTTGTATGACTAC

AGAGAAAACCGATTCATTGAAATTGGAGTAACGCGAAGGGAAGTTCACATATACTATCTA

GAAAAAGCCAACAAGATAAAATCAGAGAAAACACACATTCACATATTCTCATTCACTGGG

GAGGAAATGGCCACCAAGGCAGATTACACTCTTGATGAAGAGAGCAGAGCAAGAATAAAA

ACCAGGCTATTCACTATAAGACAAGAGATGGCCAGTAGGGGTCTATGGGATTCCTTTCGT

CAGTCCGAGAGAGGCGAAGAGACAATTGAAGAAAGATTTGAAATCACAGGAACCATGCGC

AGGCTTGCCGACCAAAGTCTCCCACCGAACTTCTCCAGCCTTGAAAACTTTAGAGCCTAT

GTGGATGGATTCGAACCGAACGGCTGCATTGAGGGCAAGCTTTCTCAAATGTCAAAAGAA

GTGAATGCCAGAATTGAGCCATTCATGAAGACAACACCACGCCCTCTCAGATTACCTGAT

GGGCCTCCCTGTTCCCAGCGGTCGAAGTTCTTGCTAATGGATGCCCTTAAATTGAGCATT

GAAGACCCGAGCCCTGAGGGGGAGGGTATACCGCTGTATGATGCAACCAAATGCATAAAG

ACATTTTTTGGCTGGAAAGAGCCCAACATCGTAAAACCACACGAAAAGGGCATAAACCCT

AATTACCTCCTGGCTTGGAAGCAAGTGCTGGCAGAACTCCAAGATATTGAAAACGAGGAG

AAAGTCCCAAAAACAAAGAACATGAAGAAGACAAGCCAACTGAAGTGGGTACTTGGTGAA

AACATGGCACCAGAGAAAGTGGACTTTGAGGACTGTAAAGATGTTAGCGATCTAAGACAG

TACGACAGTGACGAACCAGAGTCTAGATCACTAGCAAGTTGGATCCAGAGTGAATTTAAC

AAGGCTTGCGAATTGACAGATTCGAGTTGGATTGAACTTGATGAAATAGGGGAAGACGTT

GCTCCAATTGAACACATTGCGAGTATGAGGAGGAACTATTTCACAGAGGAAGTATCCCAT

TGCAGGGCTACAGAATACATAATGAAAGGATTATACATAACCACAGCCTTATTGAATGCA

TCCTGTGCAGCCCTGTATGAAATGCAATATATTCCAATGATAAGCAAATGCAGAACCAAA

GAAGGGAGACGGAAGACAAATCTGTATGGCTTCATTATAAAAGGAAGATCCCATTTGAGG

AATGACACCGATGTGGTAAACTTTGTGAGCATGGAATTCTCTCTAACTGACCCAAGGCTG

GAGCCATACAAATGGGAAAAATACTGTGTTCTTGAGATAGGAGACATGCTCCTACGGACT

GCAATAGGCCAAGTGTCAAGGCCCATGTTCCTGTATGTGAGAACCAATGGGACTTCCAAG

ATCAAAATGAAATGGGGCATGGAGATGAGGCGATGCCTTCTTCAATCCCTTCAACAAATT

GAGAGCATGATTGAAGCCGAGTCTTCTGTCAAAGAAAAGGACATGACCAAGGAATTCTTT

GAAAACAGATCGGAAACATGGCCAATTGGAGAATCACCCAAAGGAGTGGAGGAAGGCTCC

ATTGGGAAGGTGTGCAGAACGTTGCTAGCAAAGTCTGTGTTCAACAGCCTATATGCATCT

CCACAACTCGAGGGGTTTTCAGCTGAATCAAGAAAGTTGCTTCTCATTGTCCAGGCACTT

AGGGACAACCTGGAACCTGGAACCTTCGATCTTGGGGGGCTATATGAAGCAATTGAGGAG

TGCCTGATTAACGATCCCTGGGTTTTGCTTAATGCGTCTTGGTTCAACTCCTTCCTCACA

CATGCACTGAAATAG---------------------------------------------

----------------

>A_goose_Omsk_0111_2020_EPI1813141

AGCAAAAGCAGGTACTGATCCAAAATGGAAGACTTTGTGCGACAATGCTTCAATCCAATG

ATTGTCGAGCTTGCGGAAAAGGCAATGAAAGAATATGGGGAAGATCCGAAAATCGAAACA

AACAAATTTGCCGCAATATGCACACACTTAGAAGTCTGTTTCATGTATTCGGATTTCCAC

TTTATTGATGAACGGGGCGAATCAATAATTGTAGAATCTGGCGATCCGAATGCATTATTG

AAACACCGATTTGAGATAATTGAGGGGAGAGACCGAACTATGGCCTGGACGGTGGTGAAT

AGTATCTGCAACACCACAGGAGTCGAAAAGCCCAAATTCCTCCCTGATTTGTATGACTAC

AAAGAGAACCGATTCATTGAAATTGGAGTAACGCGAAGGGAAGTTCACATATACTATCTA

GAAAAAGCCAACAAGATAAAATCAGAGAAGACACACATTCACATATTCTCATTCACTGGA

GAGGAAATGGCCACCAAGGCTGACTACACCCTTGATGAAGAGAGCAGAGCAAGAATAAAA

ACCAGGCTGTTCACTATAAGACAAGAAATGGCCAGTAGGGGTCTATGGGATTCCTTTCGT

CAGTCCGAGAGAGGCGAAGAGACAATTGAAGAAAGATTTGAAATCACAGGAACCATGCGC

AGGCTTGCCGACCAAAGTCTCCCACCGAACTTCTCCAGCCTTGAAAACTTTAGAGCCTAT

GTGGATGGATTCGAACCGAACGGCTGCATTGAGGGCAAGCTTTCTCAAATGTCAAAAGAA

GTGAATGCCAGAATTGAGCCATTTCTGAAGACAACACCACGCCCTCTCAGATTACCTGAT

GGGCCTCCCTGTTCTCAGCGGTCGAAGTTTTTGCTGATGGATGCCCTCAAATTGAGCATC

GAAGACCCGAGCCATGAGGGGGAGGGTATACCGCTGTATGATGCAATCAAATGCATGAAG

ACATTTTTTGGCTGGAAAGAGCCCAACATCGTAAAACCACATGAAAAAGGCATAAACCCT

AATTACCTCCTGGCTTGGAAGCAGGTGCTGGCAGAACTCCAAGATATTGAAAATGAGGAG

AAAATCCCAAAAACAAAGAACATGAAGAAAACAAGCCAATTGAAGTGGGCACTTGGTGAG

AACATGGCACCAGAGAAAGTGGACTTTGAGGACTGTAAAGATGTTAGCGATCTAAGACAG

TACGACAGTGATGAGCCAGAGTCTAGATCACTAGCAAGCTGGATCCAGAGTGAGTTCAAC

AAGGCATGCGAATTGACAGATTCGAGTTGGATTGAACTTGATGAAATAGGGGAAGACATT

GCTCCAATTGAACACATTGCGAGCATGAGGAGGAACTATTTCACAGCGGAAGTGTCCCAT

TGCAGGGCTACTGAATACATAATGAAGGGAGTATACATAAACACAGCCCTATTGAATGCA

TCCTGTGCAGCCATGGATGACTTCCAACTGATTCCAATGATAAGCAAGTGCAGAACCAAA

GAAGGAAGACGAAAGACAAATCTGTATGGATTCATCATAAAGGGAAGATCCCATTTGAGG

AATGACACCGATGTGGTAAACTTTGTGAGCATGGAATTCTCTCTCACTGACCCGAGGCTG

GAGCCACACAAATGGGAAAAGTACTGTGTTCTTGAGATAGGAGACATGCTCCTACGGACT

GCAATAGGCCAAGTGTCAAGGCCCATGTTCCTGTATGTGAGAACAAACGGGACTTCCAAG

ATCAAAATGAAATGGGGCATGGAGATGAGGCGATGTCTTCTTCAGTCCCTTCAACAAATT

GAGAGCATGATTGAGGCCGAGTCTTCTGTCAAAGAGAAGGACATGACCAAGGAATTCTTT

GAAAATAAATCAGAAACATGGCCAATTGGGGAATCACCCAAAGGGGTGGAGGAAGGCTCC

ATTGGGAAGGTATGCAGAACATTGCTAGCAAAGTCTGTGTTCAACAGCCTATATGCATCT

CCACAACTCGAGGGGTTTTCAGCTGAATCAAGAAAATTGCTTCTCATTGTTCAGGCACTT

AGGGACAACCTGGAACCTGGAACTTTCGATCTTGGGGGGCTATATGAAGCAATTGAGGAG

TGCCTGATTAACGATCCCTGGGTTTTGCTTAATGCGTCTTGGTTCAACTCCTTCCTCACA

CATGCACTGAAATAGTTGTGGCAATGCTACTA---TTTGCTATCCATACTGTCCAAAAAA

GTACCTTGTTTCTACT

>A_goose_Omsk_01161_2020_EPI1813357

AGCAAAAGCAGGTACTGATCCAAAATGGAAGACTTTGTGCGACAATGCTTCAATCCAATG

ATTGTCGAGCTTGCGGAAAAGGCAATGAAAGAATATGGGGAAGATCCTAAGATCGAAACA

AACAAGTTTGCCGCAATATGTACACACTTAGAAGTCTGCTTCATGTATTCGGATTTCCAT

TTCATTGATGAACGAGGCGAATCAATAATCGTAGAATCTGGCGACCCGAATGCATTATTG

AAGCACCGATTTGAGATAATTGAAGGGAGAGACCGAACAATGGCCTGGACAGTGGTGAAT

AGTATATGCAACACTACAGGAGTCGAAAAGCCCAAGTTCCTTCCTGATTTGTATGACTAC

AGAGAAAACCGATTCATTGAAATTGGAGTAACGCGAAGGGAAGTTCACATATACTATCTT

GAAAAAGCCAACAAGATAAAATCAGAGAAAACACACATTCACATATTCTCATTCACTGGG

GAGGAAATGGCCACCAAGGCAGATTACACTCTTGATGAAGAGAGCAGAGCAAGAATAAAA

ACCAGGCTATTCACTATAAGACAAGAGATGGCCAATAGGGGTCTATGGGATTCCTTTCGT

CAGTCCGAGAGAGGCGAAGAGACAATTGAAGAAAGATTTGAAATCACAGGAACCATGCGC

AGGCTTGCTGACCAAAGTCTCCCACCGAACTTCGCCAGCCTTGAAAACTTTAGAGCCTAT

GTGGATGGATTTGAACCGAACGGCTGCATTGAGGGCAAGCTTTCTCAAATGTCAAAAGAA

GTGAATGCCAGAATTGAGCCATTTCTGAAGACAACACCACGCCCTCTCAGATTACCTGAT

GGACCTCCCTGTTCCCAGCGGTCGAAGTTCTTGCTAATGGATGCCCTTAAATTGAGCATT

GAAGACCCGAGCCATGAGGGGGAGGGTATACCGCTGTACGATGCAACCAAATGCATGAAG

ACATTTTTTGGCTGGAAGGAGCCCAACATCGTGAAACCACATGAAAAGGGCATAAACCCT

AATTACCTCCTGGCTTGGAAGCAGGTGCTATCAGAACTCCAAGATATTGAAAACGAGGAG

AAAATCCCAAAAACAAAGAACATGAAGAAGACGAGCCAACTGAAGTGGGCACTTGGTGAA

AACATGGCACCAGAGAAAGTGGACTTTGAGGACTGTAAAGATGTTAGCGATCTAAAACAG

TACGACAGTGACGAACCAGAATCTAGATCACTAGCAAGTTGGATCCAGAGTGAATTTAAC

AAGGCTTGCGAACTGACAGATTCGAGTTGGATTGAACTTGATGAAATAGGGGAAGACGTT

GCTCCAATTGAACACATTGCGAGTATGAGGAGGAACTATTTCACAGCGGAAGTATCCCAT

TGCAGGGCTACAGAATACATAATGAAAGGAGTATACATAAACACAGCTTTATTGAATGCA

TCCTGTGCAGCCATGGATGACTTCCAACTGATTCCAATGATAAGCAAATGCAGAACCAAA

GAAGGGAGACGGAAGACAAATCTGTATGGCTTCATTATAAAAGGAAGATCCCATTTGAGG

AATGACACCGATGTGGTAAACTTTGTGAGCATGGAATTCTCTCTCACTGACCCAAGGCTG

GAGCCACACAAATGGGAAAAGTACTGTGTGCTTGAGGTAGGAGACATGCTCCTACGGACT

GCAATAGGCCAAGTGTCAAGGCCCATGTTCCTGTATGTGAGAACCAATGGGACTTCTAAG

ATCAAAATGAAATGGGGTATGGAGATGAGGCGATGCCTTCTTCAATCCCTTCAACAGATT

GAGAGCATGATTGAAGCCGAGTCTTCTGTCAAAGAAAAAGACATGACCAAGGAATTCTTT

GAAAACAGATCAGAAACATGGCCAATTGGAGAGTCACCCAAAGGAGTGGAGGAAGGCTCC

ATTGGGAAGGTGTGCAGAACATTGCTAGCAAAGTCTGTGTTCAACAGCCTATATGCATCT

CCACAACTCGAGGGGTTTTCAGCTGAATCAAGAAAGTTGCTTCTCATTGTTCAGGCACTT

AGGGACAACCTGGAACCTGGAACCTTCGATCTTGGGGGGCTATATGAAGCAATTGAGGAG

TGCCTGATTAACGATCCCTGGGTTTTGCTTAATGCGTCTTGGTTCAACTCCTTCCTCACA

CATGCACTGAAATAGTCGTGGCAATGCTACTA---TTTGCTATCCATACTGTCCAAAAAA

GTACCTTGTTTCTACT

>A_chicken_Omsk_0118_2020_EPI1813373

AGCAAAAGCAGGTACTGATCCAAAATGGAAGACTTTGTGCGACAATGCTTCAATCCAATG

ATTGTCGAGCTTGCGGAAAAGGCAATGAAAGAATATGGGGAAGATCCTAAGATCGAAACA

AACAAGTTTGCCGCAATATGTACACACTTAGAAGTCTGCTTCATGTATTCGGATTTCCAT

TTCATTGATGAACGAGGCGAATCAATAATCGTAGAATCTGGCGACCCGAATGCATTATTG

AAGCACCGATTTGAGATAATTGAAGGGAGAGACCGAACAATGGCCTGGACAGTGGTGAAT

AGTATATGCAACACTACAGGAGTCGAAAAGCCCAAGTTCCTTCCTGATTTGTATGACTAC

AGAGAAAACCGATTCATTGAAATTGGAGTAACGCGAAGGGAAGTTCACATATACTATCTT

GAAAAAGCCAACAAGATAAAATCAGAGAAAACACACATTCACATATTCTCATTCACTGGG

GAGGAAATGGCCACCAAGGCAGATTACACTCTTGATGAAGAGAGCAGAGCAAGAATAAAA

ACCAGGCTATTCACTATAAGACAAGAGATGGCCAATAGGGGTCTATGGGATTCCTTTCGT

CAGTCCGAGAGAGGCGAAGAGACAATTGAAGAAAGATTTGAAATCACAGGAACCATGCGC

AGGCTTGCTGACCAAAGTCTCCCACCGAACTTCGCCAGCCTTGAAAACTTTAGAGCCTAT

GTGGATGGATTTGAACCGAACGGCTGCATTGAGGGCAAGCTTTCTCAAATGTCAAAAGAA

GTGAATGCCAGAATTGAGCCATTTCTGAAGACAACACCACGCCCTCTCAGATTACCTGAT

GGACCTCCCTGTTCCCAGCGGTCGAAGTTCTTGCTAATGGATGCCCTTAAATTGAGCATT

GAAGACCCGAGCCATGAGGGGGAGGGTATACCGCTGTACGATGCAACCAAATGCATGAAG

ACATTTTTTGGCTGGAAGGAGCCCAACATCGTGAAACCACATGAAAAGGGCATAAACCCT

AATTACCTCCTGGCTTGGAAGCAGGTGCTATCAGAACTCCAAGATATTGAAAACGAGGAG

AAAATCCCAAAAACAAAGAACATGAAGAAGACGAGCCAACTGAAGTGGGCACTTGGTGAA

AACATGGCACCAGAGAAAGTGGACTTTGAGGACTGTAAAGATGTTAGCGATCTAAGACAG

TACGACAGTGACGAACCAGAATCTAGATCACTAGCAAGTTGGATCCAGAGTGAATTTAAC

AAGGCTTGCGAACTGACAGATTCGAGTTGGATTGAACTTGATGAAATAGGGGAAGACGTT

GCTCCGATTGAACACATTGCGAGTATGAGGAGGAACTATTTCACAGCGGAAGTATCCCAT

TGCAGGGCCACAGAATACATAATGAAAGGAGTATACATAAACACAGCTTTATTGAATGCA

TCCTGTGCAGCCATGGATGACTTCCAACTGATTCCAATGATAAGCAAATGCAGAACCAAA

GAAGGGAGACGGAAGACAAATCTGTATGGCTTCATTATAAAAGGAAGATCCCATTTGAGG

AATGACACCGATGTGGTAAACTTTGTGAGCATGGAATTCTCTCTCACTGACCCAAGGCTG

GAGCCACACAAATGGGAAAAGTACTGTGTGCTTGAGGTAGGAGACATGCTCCTACGGACT

GCAATAGGCCAAGTGTCAAGGCCCATGTTCCTGTATGTGAGAACCAATGGGACTTCTAAG

ATCAAAATGAAATGGGGTATGGAGATGAGGCGATGCCTTCTTCAATCCCTTCAACAAATT

GAGAGCATGATTGAAGCCGAGTCTTCTGTCAAAGAAAAAGACATGACCAAGGAATTCTTT

GAAAACAGATCAGAAACATGGCCAATTGGAGAGTCACCCAAAGGAGTGGAGGAAGGCTCC

ATTGGGAAGGTGTGCAGAACATTGCTAGCAAAGTCTGTGTTCAACAGCCTATATGCATCT

CCACAACTCGAGGGGTTTTCAGCTGAATCAAGAAAGTTGCTTCTCATTGTTCAGGCACTT

AGGGACAACCTGGAACCTGGGACCTTCGATCTTGGGGGGCTATATGAAGCAATTGAGGAG

TGCCTGATTAACGATCCCTGGGTTTTGCTTAATGCGTCTTGGTTCAACTCCTTCCTCACA

CATGCACTGAAATAGTCGTGGCAATGCTACTA---TTTGCTATCCATACTGTCCAAAAAA

GTACCTTGTTTCTACT

>A_chicken_Omsk_0119_2020_EPI1813381

AGCAAAAGCAGGTACTGATCCAAAATGGAAGACTTTGTGCGACAATGCTTCAATCCAATG

ATTGTCGAGCTTGCGGAAAAGGCAATGAAAGAATATGGGGAAGATCCTAAGATCGAAACA

AACAAGTTTGCCGCAATATGTACACACTTAGAAGTCTGCTTCATGTATTCGGATTTCCAT

TTCATTGATGAACGAGGCGAATCAATAATCGTAGAATCTGGCGACCCGAATGCATTATTG

AAGCACCGATTTGAGATAATTGAAGGGAGAGACCGAACAATGGCCTGGACAGTGGTGAAT

AGTATATGCAACACTACAGGAGTCGAAAAGCCCAAGTTCCTTCCTGATTTGTATGACTAC

AGAGAAAACCGATTCATTGAAATTGGAGTAACGCGAAGGGAAGTTCACATATACTATCTT

GAAAAAGCCAACAAGATAAAATCAGAGAAAACACACATTCACATATTCTCATTCACTGGG

GAGGAAATGGCCACCAAGGCAGATTACACTCTTGATGAAGAGAGCAGAGCAAGAATAAAA

ACCAGGCTATTCACTATAAGACAAGAGATGGCCAATAGGGGTCTATGGGATTCCTTTCGT

CAGTCCGAGAGAGGCGAAGAGACAATTGAAGAAAGATTTGAAATCACAGGAACCATGCGC

AGGCTTGCTGACCAAAGTCTCCCACCGAACTTCGCCAGCCTTGAAAACTTTAGAGCCTAT

GTGGATGGATTTGAACCGAACGGCTGCATTGAGGGCAAGCTTTCTCAAATGTCAAAAGAA

GTGAATGCCAGAATTGAGCCATTTCTGAAGACAACACCACGCCCTCTCAGATTACCTGAT

GGACCTCCCTGTTCCCAGCGGTCGAAGTTCTTGCTAATGGATGCCCTTAAATTGAGCATT

GAAGACCCGAGCCATGAGGGGGAGGGTATACCGCTGTACGATGCAACCAAATGCATGAAG

ACATTTTTTGGCTGGAAGGAGCCCAACATCGTGAAACCACATGAAAAGGGCATAAACCCT

AATTACCTCCTGGCTTGGAAGCAGGTGCTATCAGAACTCCAAGATATTGAAAACGAGGAG

AAAATCCCAAAAACAAAGAACATGAAGAAGACGAGCCAACTGAAGTGGGCACTTGGTGAA

AACATGGCACCAGAGAAAGTGGACTTTGAGGACTGTAAAGATGTTAGCGATCTAAGACAG

TACGACAGTGACGAACCAGAATCTAGATCACTAGCAAGTTGGATCCAGAGTGAATTTAAC

AAGGCTTGCGAACTGACAGATTCGAGTTGGATTGAACTTGATGAAATAGGGGAAGACGTT

GCTCCGATTGAACACATTGCGAGTATGAGGAGGAACTATTTCACAGCGGAAGTATCCCAT

TGCAGGGCTACAGAATACATAATGAAAGGAGTATACATAAACACAGCTTTATTGAATGCA

TCCTGTGCAGCCATGGATGACTTCCAACTGATTCCAATGATAAGCAAATGCAGAACCAAA

GAAGGGAGACGGAAGACAAATCTGTATGGCTTCATTATAAAAGGAAGATCCCATTTGAGG

AATGACACCGATGTGGTAAACTTTGTGAGCATGGAATTCTCTCTCACTGACCCAAGGCTG

GAGCCACACAAATGGGAAAAGTACTGTGTGCTTGAGGTAGGAGACATGCTCCTACGGACT

GCAATAGGCCAAGTGTCAAGGCCCATGTTCCTGTATGTGAGAACCAATGGGACTTCTAAG

ATCAAAATGAAATGGGGTATGGAGATGAGGCGATGCCTTCTTCAATCCCTTCAACAAATT

GAGAGCATGATTGAAGCCGAGTCTTCTGTCAAAGAAAAAGACATGACCAAGGAATTCTTT

GAAAACAGATCAGAAACATGGCCAATTGGAGAGTCACCCAAAGGAGTGGAGGAAGGCTCC

ATTGGGAAGGTGTGCAGAACATTGCTAGCAAAGTCTGTGTTCAACAGCCTATATGCATCT

CCACAACTCGAGGGGTTTTCAGCTGAATCAAGAAAGTTGCTTCTCATTGTTCAGGCACTT

AGGGACAACCTGGAACCTGGGACCTTCGATCTTGGGGGGCTATATGAAGCAATTGAGGAG

TGCCTGATTAACGATCCCTGGGTTTTGCTTAATGCGTCTTGGTTCAACTCCTTCCTCACA

CATGCACTGAAATAGTCGTGGCAATGCTACTA---TTTGCTATCCATACTGTCCAAAAAA

GTACCTTGTTTCTACT

>A_chicken_Omsk_0073_2020_EPI1813405

AGCAAAAGCAGGTACTGATCCAAAATGGAAGACTTTGTGCGACAATGCTTCAATCCAATG

ATTGTCGAGCTTGCGGAAAAGGCAATGAAAGAATATGGGGAAGATCCTAAGATCGAAACA

AACAAGTTTGCCGCAATATGTACACACTTAGAAGTCTGCTTCATGTATTCGGATTTCCAT

TTCATTGATGAACGAGGCGAATCAATAATCGTAGAATCTGGCGACCCGAATGCATTATTG

AAGCACCGATTTGAGATAATTGAAGGGAGAGACCGAACAATGGCCTGGACAATGGTGAAT

AGTATATGCAACACTACAGGAGTCGAAAAGCCCAAGTTCCTTCCTGATTTGTATGACTAC

AGAGAAAACCGATTCATTGAAATTGGAGTAACGCGAAGGGAAGTTCACATATACTATCTT

GAAAAAGCCAACAAGATAAAATCAGAGAAAACACACATTCACATATTCTCATTCACTGGG

GAGGAAATGGCCACCAAGGCAGATTACACTCTTGATGAAGAGAGCAGAGCAAGAATAAAA

ACCAGGCTATTCACTATAAGACAAGAGATGGCCAATAGGGGTCTATGGGATTCCTTTCGT

CAGTCCGAGAGAGGCGAAGAGACAATTGAAGAAAGATTTGAAATCACAGGAACCATGCGC

AGGCTTGCTGACCAAAGTCTCCCACCGAACTTCGCCAGCCTTGAAAACTTTAGAGCCTAT

GTGGATGGATTTGAACCGAACGGCTGCATTGAGGGCAAGCTTTCTCAAATGTCAAAAGAA

GTGAATGCCAGAATTGAGCCATTTCTGAAGACAACACCACGCCCTCTCAGATTACCTGAT

GGACCTCCCTGTTCCCAGCGGTCGAAGTTCTTGCTAATGGATGCCCTTAAATTGAGCATT

GAAGACCCGAGCCATGAGGGGGAGGGTATACCGCTGTACGATGCAACCAAATGCATGAAG

ACATTTTTTGGCTGGAAGGAGCCCAACATCGTGAAACCACATGAAAAGGGCATAAACCCT

AATTACCTCCTGGCTTGGAAGCAGGTGCTATCAGAACTCCAAGATATTGAAAACGAGGAG

AAAATCCCAAAAACAAAGAACATGAAGAAGACGAGCCAACTGAAGTGGGCACTTGGTGAA

AACATGGCACCAGAGAAAGTGGACTTTGAGGACTGTAAAGATGTTAGCGATCTAAGACAG

TACGACAGTGACGAACCAGAATCTAGATCACTAGCAAGTTGGATCCAGAGTGAATTTAAC

AAGGCTTGCGAACTGACAGATTCGAGTTGGATTGAACTTGATGAAATAGGGGAAGACGTT

GCTCCAATTGAACACATTGCGAGTATGAGGAGGAACTATTTCACAGCGGAAGTATCCCAT

TGCAGGGCTACAGAATACATAATGAAAGGAGTATACATAAACACAGCTTTATTGAATGCA

TCCTGTGCAGCCATGGATGACTTCCAACTGATTCCAATGATAAGCAAATGCAGAACCAAA

GAAGGGAGACGGAAGACAAATCTGTATGGCTTCATTATAAAAGGAAGATCCCATTTGAGG

AATGACACCGATGTGGTAAACTTTGTGAGCATGGAATTCTCTCTCACTGACCCAAGGCTG

GAGCCACACAAATGGGAAAAGTACTGTGTGCTTGAGGTAGGAGACATGCTCCTACGGACT

GCAATAGGCCAAGTGTCAAGGCCCATGTTCCTGTATGTGAGAACCAATGGGACTTCTAAG

ATCAAAATGAAATGGGGTATGGAGATGAGGCGATGCCTTCTTCAATCCCTTCAACAAATT

GAGAGCATGATTGAAGCCGAGTCTTCTGTCAAAGAAAAAGACATGACCAAGGAATTCTTT

GAAAACAGATCAGAAACATGGCCAATTGGAGAGTCACCCAAAGGAGTGGAGGAAGGCTCC

ATTGGGAAGGTGTGCAGAACATTGCTAGCAAAGTCTGTATTCAACAGCCTATATGCATCT

CCACAACTCGAGGGGTTTTCAGCTGAATCAAGAAAGTTGCTTCTCATTGTTCAGGCACTT

AGGGACAACCTGGAACCTGGAACCTTCGATCTTGGGGGGCTATATGAAGCAATTGAGGAG

TGCCTGATTAACGATCCCTGGGTTTTGCTTAATGCGTCTTGGTTCAACTCCTTCCTCACA

CATGCACTGAAATAGTCGTGGCAATGCTACTA---TTTGCTATCCATACTGTCCAAAAAA

GTACCTTGTTTCTACT

>A_chicken_Omsk_30007_2020_EPI1814309

AGCAAAAGCAGGTACTGATCCAAAATGGAAGACTTTGTGCGACAATGCTTCAATCCAATG

ATTGTCGAGCTTGCGGAAAAGGCAATGAAAGAATATGGGGAAGATCCTAAGATCGAAACA

AACAAGTTTGCCGCAATATGTACACACTTAGAAGTCTGCTTCATGTATTCGGATTTCCAT

TTCATTGATGAACGAGGCGAATCAATAATCGTAGAATCTGGCGACCCGAATGCATTATTG

AAGCACCGATTTGAGATAATTGAAGGGAGAGACCGAACAATGGCCTGGACAGTGGTGAAT

AGTATACACAACACTACAGGAGTCGAAAAGCCCAAGTTCCTTCCTGATTTGTATGACTAC

AGAGAAAACCGATTCATTGAAATTGGAGTAACGCGAAGGGAAGTTCACATATACTATCTT

GAAAAAGCCAACAAGATAAAATCAGAGAAAACACACATTCACATATTCTCGTTCACTGGG

GAGGAAATGGCCACCAAGGCAGATTACACTCTTGATGAAGAGAGCAGAGCAAGAATAAAA

ACCAGGCTATTCACTATAAGACAAGAGATGGCCAATAGGGGTCTATGGGATTCCTTTCGT

CAGTCCGAGAGAGGCGAAGAGACAATTGAAGAAAGATTTGAAATCACAGGAACCATGCGC

AGGCTTGCTGACCAAAGTCTCCCACCGAACTTCGCCAGCCTTGAAAACTTTAGAGCCTAT

GTGGATGGATTTGAACCGAACGGCTGCATTGAGGGCAAGCTTTCTCAAATGTCAAAAGAA

GTGAATGCCAGAATTGAGCCATTTCTGAAGACAACACCACGCCCTCTCAGATTACCTGAT

GGACCTCCCTGTTCCCAGCGGTCGAAATTCTTGCTAATGGATGCCCTTAAATTAAGCATT

GAAGACCCGAGCCATGAGGGGGAGGGTATACCGCTGTACGATGCAACCAAATGCATGAAG

ACATTTTTTGGCTGGAAGGAGCCCAACATCGTGAAACCACATGAAAAGGGCATAAACCCT

AATTACCTCCTGGCTTGGAAGCAGGTGCTATCAGAACTCCAAGATATTGAAAACGAGGAG

AAAATCCCAAAAACAAAGAACATGAAGAAGACGAGCCAACTGAAGTGGGCACTTGGTGAA

AACATGGCACCAGAGAAAGTGGACTTTGAGGACTGTAAAGATGTTAGCGATCTAAAACAG

TACGACAGTGACGAACCAGAATCTAGATCACTAGCAAGTTGGATCCAGAGTGAATTTAAC

AAGGCTTGCGAACTGACAGATTCGAGTTGGATTGAACTTGATGAAATAGGGGAAGACGTT

GCTCCAATTGAACACATTGCGAGTATGAGGAGGAACTATTTCACAGCGGAAGTATCCCAT

TGCAGGGCTACAGAATACATAATGAAAGGAGTATACATAAACACAGCTTTATTGAATGCA

TCCTGTGCAGCCATGGATGACTTCCAACTGATTCCAATGATAAGCAAATGCAGAACCAAA

GAAGGGAGACGGAAGACAAATCTGTATGGCTTCATTATAAAAGGAAGATCCCATTTGAGG

AATGACACCGATGTGGTAAACTTTGTGAGCATGGAATTCTCTCTCACTGACCCAAGGCTG

GAGCCACACAAATGGGAAAAGTACTGTGTGCTTGAGGTAGGAGACATGCTCCTACGGACT

GCAATAGGCCAAGTGTCAAGGCCCATGTTCCTGTATGTGAGAACCAATGGGACTTCTAAG

ATCAAAATGAAATGGGGTATGGAGATGAGGCGATGCCTTCTTCAATCCCTTCAACAAATT

GAGAGCATGATTGAAGCCGAGTCTTCTGTCAAAGAAAAAGACATGACCAAGGAATTCTTT

GAAAACAGATCAGAAACATGGCCAATTGGAGAGTCACCCAAAGGAGTGGAGGAAGGCTCC

ATTGGGAAGGTGTGCAGAACATTGCTAGCAAAGTCTGTGTTCAACAGCCTATATGCATCT

CCACAACTCGAGGGGTTTTCAGCTGAATCAAGAAAGTTGCTTCTCATTGTTCAGGCACTT

AGGGACAACCTGGAACCTGGAACCTTCGATCTTGGGGGGCTATATGAAGCAATTGAGGAG

TGCCTGATTAACGATCCCTGGGTTTTGCTTAATGCGTCTTGGTTCAACTCCTTCCTCACA

CATGCACTGAAATAGTCGTGGCAATGCTACTA---TTTGCTATCCATACTGTCCAAAAA-

GTACCTTGTTTCTACT

>A_goose_Omsk_30009_2020_EPI1814317

AGCAAAAGCAGGTACTGATCCAAAATGGAAGACTTTGTGCGACAATGCTTCAATCCAATG

ATTGTCGAGCTTGCGGAAAAGGCAATGAAAGAATATGGGGAAGATCCTAAGATCGAAACA

AACAAGTTTGCCGCAATATGTACACACTTAGAAGTCTGCTTCATGTATTCGGATTTCCAT

TTCATTGATGAACGAGGCGAATCAACAATCGTAGAATCTGGCGACCCGAATGCATTATTG

AAGCACCGATTTGAGATAATTGAAGGGAGAGACCGAACAATGGCCTGGACAGTGGTGAAT

AGTATATGCAACACTACAGGAGTCGAAAAGCCCAAGTTCCTTCCTGATTTGTATGACTAC

AGAGAAAACCGATTCATTGAAATTGGAGTAACGCGAAGGGAAGTTCACATATACTATCTT

GAAAAAGCCAACAAGATAAAATCAGAGAAAACACACATTCACATATTCTCATTCACTGGG

GAGGAAATGGCCACCAAGGCAGATTACACTCTTGATGAAGAGAGCAGAGCAAGAATAAAA

ACCAGGCTATTCACTATAAGACAAGAGATGGCCAATAGGGGTCTATGGGATTCCTTTCGT

CAGTCCGAGAGAGGCGAAGAGACAATTGAAGAAAGATTTGAAATCACAGGAACCATGCGC

AGGCTTGCTGACCAAAGTCTCCCACCGAACTTCGCCAGCCTTGAAAACTTTAGAGCCTAT

GTGGATGGATTTGAACCGAACGGCTGCATTGAGGGCAAGCTTTCTCAAATGTCAAAAGAA

GTGAATGCCAGAATTGAGCCATTTCTGAAGACAACACCACGCCCTCTCAGATTACCTGAT

GGACCTCCCTGTTCCCAGCGGTCGAAATTCTTGCTAATGGATGCCCTTAAATTGAGCATT

GAAGACCCGAGCCATGAGGGGGAGGGTATACCGCTGTACGATGCAACCAAATGCATGAAG

ACATTTTTTGGCTGGAAGGAGCCCAACATCGTGAAACCACATGAAAAGGGCATAAACCCT

AATTACCTCCTGGCTTGGAAGCAGGTGCTATCAGAACTCCAAGATATTGAAAACGAGGAG

AAAATCCCAAAAACAAAGAACATGAAGAAGACGAGCCAACTGAAGTGGGCACTTGGTGAA

AACATGGCACCAGAGAAAGTGGACTTTGAGGACTGTAAAGATGTTAGCGATCTAAAACAG

TACGACAGTGACGAACCAGAATCTAGATCACTAGCAAGTTGGATCCAGAGTGAATTTAAC

AAGGCTTGCGAACTGACAGATTCGAGTTGGATTGAACTTGATGAAATAGGGGAAGACGTT

GCTCCAATTGAACACATTGCGAGTATGAGGAGGAACTATTTCACAGCGGAAGTATCCCAT

TGCAGGGCTACAGAATACATAATGAAAGGAGTATACATAAACACAGCTTTATTGAATGCA

TCCTGTGCAGCCATGGATGACTTCCAACTGATTCCAATGATAAGCAAATGCAGAACCAAA

GAAGGGAGACGGAAGACAAATCTGTATGGCTTCATTATAAAAGGAAGATCCCATTTGAGG

AATGACACCGATGTGGTAAACTTTGTGAGCATGGAATTCTCTCTCACTGACCCAAGGCTG

GAGCCACACAAATGGGAAAAGTACTGTGTGCTTGAGGTAGGAGACATGCTCCTACGGACT

GCAATAGGCCAAGTGTCAAGGCCCATGTTCCTGTATGTGAGAACCAATGGGACTTCTAAG

ATCAAAATGAAATGGGGTATGGAGATGAGGCGATGCCTTCTTCAATCCCTTCAACAAATT

GAGAGCATGATTGAAGCCGAGTCTTCTGTCAAAGAAAAAGACATGACCAAGGAATTCTTT

GAAAACAGATCAGAAACATGGCCAATTGGAGAGTCACCCAAAGGAGTGGAGGAAGGCTCC

ATTGGGAAGGTGTGCAGAACATTGCTAGCAAAGTCTGTGTTCAACAGCCTATATGCATCT

CCACAACTCGAGGGGTTTTCAGCTGAATCAAGAAAGTTGCTTCTCATTGTTCAGGCACTT

AGGGACAACCTGGAACCTGGAACCTTCGATCTTGGGGGGCTATATGAAGCAATTGAGGAG

TGCCTGATTAACGATCCCTGGGTTTTGCTTAATGCGTCTTGGTTCAACTCCTTCCTCACA

CATGCACTGAAATAGTCGTGGCAATGCTACTA---TTTGCTATCCATACTGTCCAAAAA-

GTACCTTGTTTCTACT

>A_chicken_Chelyabinsk_201_2020_EPI1814333

AGCAAAAGCAGGTACTGATCCAAAATGGAAGACTTTGTGCGACAATGCTTCAATCCAATG

ATTGTCGAGCTTGCGGAAAAGGCAATGAAAGAATATGGGGAAGATCCTAAGATCGAAACA

AACAAGTTTGCCGCAATATGTACACACTTAGAAGTCTGCTTCATGTATTCGGATTTCCAT

TTCATTGATGAACGAGGCGAATCAATAATCGTAGAATCTGGCGACCCGAATGCATTATTG

AAGCACCGATTTGAGATAATTGAAGGGAGAGACCGAACAATGGCCTGGACAGTGGTGAAT

AGTATATGCAACACTACAGGAGTCGAAAAGCCCAAGTTCCTTCCTGATTTGTATGACTAC

AGAGAAAACCGATTCATTGAAATTGGAGTAACGCGAAGGGAAGTTCACATATACTATCTT

GAAAAAGCCAACAAGATAAAATCAGAGAAAACACACATTCACATATTCTCATTCACTGGG

GAGGAAATGGCCACCAAGGCAGATTACACTCTTGATGAAGAGAGCAGAGCAAGAATAAAA

ACCAGGCTATTCACTATAAGACAAGAGATGGCCAATAGGGGTCTATGGGATTCCTTTCGT

CAGTCCGAGAGAGGCGAAGAGACAATTGAAGAAAGATTTGAAATCACAGGAACCATGCGC

AGGCTTGCTGACCAAAGTCTCCCACCGAACTTCGCCAGCCTTGAAAACTTTAGAGCCTAT

GTGGATGGATTTGAACCGAACGGCTGCATTGAGGGCAAGCTTTCTCAAATGTCAAAAGAA

GTGAATGCCAGAATTGAGCCATTTCTGAAGACAACACCACGCCCTCTCAGATTACCTGAT

GGACCTCCCTGTTCCCAGCGGTCGAAGTTCTTGCTAATGGATGCCCTTAAATTGAGCATT

GAAGACCCGAGCCATGAGGGGGAGGGTATACCGCTGTACGATGCAACCAAATGCATGAAG

ACATTTTTTGGCTGGAAGGAGCCCAACATCGTGAAACCACATGAAAAGGGCATAAACCCT

AATTACCTCCTGGCTTGGAAGCAGGTGCTATCAGAACTCCAAGATATTGAAAACGAGGAG

AAAATCCCAAAAACAAAGAACATGAAGAAGACGAGCCAACTGAAGTGGGCACTTGGTGAA

AACATGGCACCAGAGAAAGTGGACTTTGAGGACTGTAAAGATGTTAGCGATCTACGACAG

TACGACAGTGACGAACCAGAATCTAGATCACTAGCAAGTTGGATCCAGAGTGAATTTAAC

AAGGCTTGCGAACTGACAGATTCGAGTTGGATTGAACTTGATGAAATAGGGGAAGACGTT

GCTCCAATTGAACACATTGCGAGTATGAGGAGGAACTATTTCACAGCGGAAGTATCCCAT

TGCAGGGCTACAGAATACATAATGAAAGGAGTATACATAAACACAGCTTTATTGAATGCA

TCCTGTGCAGCCATGGATGACTTCCAACTGATTCCAATGATAAGCAAATGCAGAACCAAA

GAAGGGAGACGGAAGACAAATCTGTATGGCTTCATTATAAAAGGAAGATCCCATTTGAGG

AATGACACCGATGTGGTAAACTTTGTGAGCATGGAATTCTCTCTCACTGACCCAAGGCTG

GAGCCACACAAATGGGAAAAGTACTGTGTGCTTGAGGTAGGAGACATGCTCCTACGGACT

GCAATAGGCCAAGTGTCAAGGCCCATGTTCCTGTATGTGAGAACCAATGGGACTTCTAAG

ATCAAAATGAAATGGGGTATGGAGATGAGGCGATGCCTTCTCCAATCCCTTCAACAAATT

GAGAGCATGATTGAAGCCGAGTCTTCTGTCAAAGAAAAAGACATGACCAAGGAATTCTTT

GAAAACAGATCAGAAACATGGCCAATTGGAGAGTCACCCAAAGGAGTGGAGGAAGGCTCC

ATTGGGAAGGTGTGCAGAACATTGCTAGCAAAGTCTGTGTTCAACAGCCTATATGCATCT

CCACAACTCGAGGGGTTTTCAGCTGAATCAAGAAAGTTGCTTCTCATTGTTCAGGCACTT

AGGGACAACCTGGAACCTGGAACCTTCGATCTTGGGGGGCTATATGAAGCAATTGAGGAG

TGCCTGATTAACGATCCCTGGGTTTTGCTTAATGCGTCTTGGTTCAACTCCTTCCTCACA

CATGCACTGAAATAGTCGTGGCAATGCTACTA---TTTGCTATCCATACTGTCCAAAAA-

GTACCTTGTTTCTACT

>A_chicken_Kurgan_1005_2020_EPI1814365

AGCAAAAGCAGGTACTGATCCAAAATGGAAGACTTTGTGCGACAATGCTTCAATCCAATG

ATTGTCGAGCTTGCGGAAAAGGCAATGAAAGAATATGGGGAAGATCCTAAGATCGAAACA

AACAAGTTTGCCGCAATATGTACACACTTAGAAGTCTGCTTCATGTATTCGGATTTCCAT

TTCATTGATGAACGAGGCGAATCAATAATCGTAGAATCTGGCGACCCGAATGCATTATTG

AAGCACCGATTTGAGATAATTGAAGGGAGAGACCGAACAATGGCCTGGACAGTGGTGAAT

AGTATATGCAACACTACAGGAGTCGAAAAGCCCAAATTCCTTCCTGATTTGTATGACTAC

AGAGAAAACCGATTCATTGAAATTGGAGTAACGCGAAGGGAAGTTCACATATACTATCTT

GAAAAAGCCAACAAGATAAAATCAGAGAAAACACACATTCACATATTCTCATTCACTGGG

GAGGAAATGGCCACCAAGGCAGATTACACTCTTGATGAAGAGAGCAGAGCAAGAATAAAA

ACCAGGCTATTCACTATAAGACAAGAGATGGCCAATAGGGGTCTATGGGATTCCTTTCGT

CAGTCCGAGAGAGGCGAAGAGACAATTGAAGAAAGATTTGAAATCACAGGAACCATGCGC

AGGCTTGCTGACCAAAGTCTCCCACCGAACTTCGCCAGCCTTGAAAACTTTAGAGCCTAT

GTGGATGGATTTGAACCGAACGGCTGCATTGAGGGCAAGCTTTCTCAAATGTCAAAAGAA

GTGAATGCCAGAATTGAGCCATTTCTGAAGACAACACCACGCCCTCTCAGATTACCTGAT

GGACCTCCCTGTTCCCAGCGGTCGAAGTTCTTGCTAATGGATGCCCTTAAATTGAGCATT

GAAGACCCGAGCCATGAGGGGGAGGGTATACCGCTGTACGATGCAACCAAATGCATGAAG

ACATTTTTTGGCTGGAAGGAGCCCAACATCGTGAAACCACATGAAAAGGGCATAAACCCT

AATTACCTCCTGGCTTGGAAGCAGGTGCTATCAGAACTCCAAGATATTGAAAACGAGGAG

AAAATCCCAAAAACAAAGAACATGAAGAAGACGAGCCAACTGAAGTGGGCACTTGGTGAA

AACATGGCACCAGAGAAAGTGGACTTTGAGGACTGTAAAGATGTTAGCGATCTAAGACAG

TACGACAGTGACGAACCAGAATCTAGATCACTAGCAAGTTGGATCCAGAGTGAATTTAAC

AAGGCTTGCGAACTGACAGATTCGAGTTGGATTGAACTTGATGAAATAGGGGAAGACGTT

GCTCCAATTGAACACATTGCGAGTATGAGGAGGAACTATTTCACAGCGGAAGTATCCCAT

TGCAGGGCTACAGAATACATAATGAAAGGAGTATACATAAACACAGCTTTATTGAATGCA

TCCTGTGCAGCCATGGATGACTTCCAACTGATTCCAATGATAAGCAAATGCAGAACCAAA

GAAGGGAGACGGAAGACAAATCTGTATGGCTTCATTATAAAAGGAAGATCCCATTTGAGG

AATGACACCGATGTGGTAAACTTTGTGAGCATGGAATTCTCTCTCACTGACCCAAGGCTG

GAGCCACACAAGTGGGAAAAGTACTGTGTGCTTGAGGTAGGAGACATGCTCCTACGGACT

GCAATAGGCCAAGTGTCAAGGCCCATGTTCCTGTATGTGAGAACCAATGGGACTTCTAAG

ATCAAAATGAAATGGGGTATGGAGATGAGGCGATGCCTTCTCCAATCCCTTCAACAAATT

GAGAGCATGATTGAAGCCGAGTCTTCTGTCAAAGAAAAAGACATGACCAAGGAATTCTTT

GAAAACAGATCAGAAACATGGCCAATTGGAGAGTCACCCAAAGGAGTGGAGGAAGGCTCC

ATTGGGAAGGTGTGCAGAACATTGCTAGCAAAGTCTGTGTTCAACAGCCTATATGCATCT

CCACAACTCGAGGGGTTTTCAGCTGAATCAAGAAAGTTGCTTCTCATTGTTCAGGCACTT

AGGGACAACCTGGAACCTGGAACCTTCGATCTTGGGGGGCTATATGAAGCAATTGAGGAG

TGCCTGATTAACGATCCCTGGGTTTTGCTTAATGCGTCTTGGTTCAACTCCTTCCTCACA

CATGCACTGAAATAGTCGTGGCAATGCTACTA---TTTGATATCCATACTGTCCAAAAA-

GTACCTTGTTTCTACT

>A_turkey_Poland_464_2020_EPI1841309

-----------GTACTGATCCAAAATGGAAGACTTTGTGCGACAATGCTTCAATCCAATG

ATTGTCGAGCTTGCGGAAAAGGCAATGAAAGAATATGGGGAAGATCCTAAGATCGAAACA

AACAAGTTTGCCGCAATATGTACGCACTTAGAAGTCTGCTTCATGTATTCGGATTTCCAT

TTCATTGATGAACGAGGCGAATCAATAATCGTAGAATCTGGCGACCCGAATGCATTATTG

AAGCACCGATTTGAGATAATTGAAGGGAGAGACCGAACAATGGCCTGGACAGTGGTGAAT

AGTATATGCAACACTACAGGAGTCGAAAAGCCCAAGTTCCTTCCTGATTTGTATGACTAC

AGAGAAAACCGATTCATTGAAATTGGAGTAACGCGAAGGGAAGTTCACATATACTATCTT

GAAAAAGCCAACAAGATAAAATCAGAGAAAACACACATTCACATATTCTCATTCACTGGG

GAGGAAATGGCCACCAAGGCAGATTACACTCTTGATGAAGAGAGCAGAGCAAGAATAAAA

ACCAGGCTATTCACTATAAGACAAGAGATGGCCAATAGGGGTCTATGGGATTCCTTTCGT

CAGTCCGAGAGAGGCGAAGAGACAATTGAAGAAAGATTTGAAATCACAGGAACCATGCGC

AGGCTTGCTGACCAAAGTCTCCCACCGAACTTCGCCAGCCTTGAAAACTTTAGAGCCTAT

GTGGATGGATTTGAACCGAACGGCTGCATTGAGGGCAAGCTTTCTCAAATGTCAAAAGAA

GTGAATGCCAGAATTGAGCCATTTCTGAAGACAACACCACGCCCTCTCAGATTACCTGAT

GGACCTCCCTGTTCCCAGCGGTCGAAGTTCTTGCTAATGGATGCCCTTAAATTGAGCATT

GAAGACCCGAGCCATGAGGGGGAGGGTATACCGCTGTACGATGCAACYAAATGCATGAAG

ACATTTTTTGGCTGGAAGGAGCCCAACATCGTGAAACCACATGAAAAGGGCATAAACCCT

AATTACCTCCTGGCTTGGAAGCAGGTGCTATCAGACCTCCAAGATATTGAAAACGAGGAG

AAAATCCCAAAAACAAAGAACATGAAGAAGACGAGCCAACTGAAGTGGGCACTTGGTGAA

AACATGGCACCAGAGAAAGTGGACTTTGAGGACTGTAAAGATGTTAGCGATCTAAGACAG

TACGACAGTGACGAACCAGAATCTAGATCACTAGCAAGTTGGATCCAGAGTGAATTTAAC

AAGGCTTGCGAACTGACAGATTCGAGTTGGATTGAACTTGATGAAATAGGGGAAGACGTT

GCTCCGATTGAACACATTGCGAGTATGAGGAGGAACTATTTCACAGCGGAAGTATCCCAT

TGCAGGGCTACAGAATACATAATGAAAGGAGTATACATAAACACAGCTTTATTGAATGCA

TCCTGTGCTGCCATGGATGACTTCCAACTGATTCCAATGATAAGCAAATGCAGAACCAAA

GAAGGGAGACGGAAGACAAATCTGTATGGCTTCATTATAAAAGGAAGATCCCATTTGAGG

AATGACACCGATGTGGTAAACTTTGTGAGCATGGAATTCTCTCTCACTGACCCAAGGCTG

GAGCCACACAAATGGGAAAAGTACTGTGTGCTTGAGGTAGGAGACATGCTCCTACGGACT

GCAATAGGCCAAGTGTCAAGGCCCATGTTCCTGTATGTGAGAACCAATGGGACTTCTAAG

ATCAAAATGAAATGGGGTATGGAGATGAGGCGATGCCTTCTTCAATCCCTTCAACAAATT

GAGAGCATGATTGAAGCCGAGTCTTCTGTCAAAGAAAAAGACATGACCAAGGAATTCTTT

GAAAACAGATCAGAAACATGGCCAATTGGAGAGTCACCCAAAGGAGTGGAGGAAGGCTCC

ATTGGGAAGGTGTGCAGAACATTGCTAGCAAAGTCTGTGTTCAACAGCCTATATGCATCT

CCACAACTCGAGGGGTTTTCAGCTGAATCAAGAAAGTTGCTTCTCATTGTTCAGGCACTT

AGGGACAACCTGGAACCTGGGACCTTCGATCTTGGGGGGCTATATGAAGCAATTGAGGAG

TGCCTGATTAACGATCCCTGGGTTTTGCTTAATGCGTCTTGGTTCAACTCCTTCCTCACA

CATGCACTGAAATAGTCGTGGCAATGCTACTA---TTTGCTATCCATACTGTCCAAAAAA

GTA-------------

>A_duck_Northern_China_ZGL_2020_EPI1844090

------------------------ATGGAAGACTTTGTGCGACAATGCTTCAATCCAATG

ATTGTCGAGCTTGCGGAAAAGGCAATGAAAGAATATGGGGAAGATCCTAAGATCGAAACA

AACAAGTTTGCCGCAATATGTACACACTTAGAAGTCTGCTTCATGTATTCGGATTTCCAT

TTCATTGATGAACGAGGCGAATCAATAATCGTAGAATCTGGCGACCCGAATGCATTATTG

AAGCACCGATTTGAGATAATTGAAGGGAGAGACCGAACAATGGCCTGGACAGTGGTGAAT

AGTATATGCAACACTACTGGAGTCGAAAAGCCCAAGTTCCTTCCTGATTTGTATGACTAC

AGAGAAAACCGATTCATTGAAATTGGAGTAACGCGAAGGGAAGTTCACATATACTATCTT

GAAAAAGCTAACAAGATAAAATCAGAGAAAACACACATTCACATATTCTCATTCACTGGG

GAGGAAATGGCCACCAAGGCAGATTACACTCTTGATGAAGAGAGCAGAGCAAGAATAAAA

ACCAGGCTATTCACTATAAGACAAGAGATGGCCAATAGGGGTCTATGGGATTCCTTTCGT

CAGTCCGAGAGAGGCGAAGATACAATTGAAGAAAGATTTGAAATCACAGGAACCATGCGC

AGGCTTGCTGACCAAAGTCTCCCACCGAACTTCGCCAGCCTTGAAAACTTTAGAGCCTAT

GTGGATGGATTTGAACCGAACGGCTGCATTGAGGGCAAGCTTTCTCAAATGTCAAAAGAA

GTGAATGCCAGAATTGAGCCATTTCTGAAGACAACACCACGCCCTCTCAGATTACCTGAT

GGACCTCCCTGTTCCCAGCGGTCGAAGTTCTTGCTAATGGATGCCCTTAAATTGAGCATT

GAAGACCCGAGCCATGAGGGGGAGGGTATACCGCTGTACGATGCAACCAAATGCATGAAG

ACATTTTTTGGCTGGAAGGAGCCCAACATCGTGAAACCACATGAAAAGGGCATAAACCCT

AATTACCTCCTGGCTTGGAAGCAGGTGCTATCAGAACTCCAAGATATTGAAAACGAGGAG

AAAATCCCAAAAACAAAGAACATGAAGAAGACGAGCCAACTGAAGTGGGCACTTGGTGAA

AACATGGCACCAGAGAAAGTGGACTTTGAGGACTGTAAAGATGTTAGCGATCTAAGACAG

TACGACAGTGACGAACCAGAATCTAGATCACTAGCAAGTTGGATCCAGAGTGAATTTAAC

AAGGCTTGCGAACTGACAGATTCGAGTTGGATTGAACTTGATGAAATAGGGGAAGACGTT

GCTCCGATTGAACACATTGCGAGTATGAGGAGGAACTATTTCACAGCGGAAGTATCCCAT

TGCAGGGCTACAGAATACATAATGAAAGGAGTATACATAAACACAGCTTTATTGAATGCA

TCCTGTGCAGCCATGGATGACTTCCAACTGATTCCAATGATAAGCAAATGCAGAACCAAA

GAAGGGAGACGGAAGACAAATCTGTATGGCTTCATTATAAAAGGAAGATCCCATTTGAGG

AATGACACCGATGTGGTAAACTTTGTGAGCATGGAATTCTCTCTCACTGATCCAAGGCTG

GAGCCACACAAATGGGAAAAGTACTGTGTGCTTGAGGTAGGAGACATGCTCCTACGGACT

GCAATAGGCCAAGTGTCAAGGCCCATGTTCCTGTATGTGAGGACCAATGGGACTTCTAAG

ATCAAAATGAAATGGGGTATGGAGATGAGGCGATGCCTTCTTCAATCCCTTCAACAAATT

GAGAGCATGATTGAAGCCGAGTCTTCTGTCAAAGAAAAAGACATGACCAAAGAATTCTTT

GAAAACAGATCAGAAACATGGCCAATTGGAGAGTCACCCAAAGGAGTGGAGGAAGGCTCC

ATTGGGAAGGTGTGCAGAACATTGCTAGCAAAGTCTGTGTTCAACAGCCTATATGCATCT

CCACAACTCGAGGGGTTTTCAGCTGAATCAAGAAAGTTGCTTCTCATTGTTCAGGCACTT

AGGGACAACCTGGAACCTGGGACCTTCGATCTTGGGGGGCTATATGAAGCAATTGAGGAG

TGCCTGATTAACGATCCCTGGGTTTTGCTTAATGCGTCTTGGTTCAACTCCTTCCTCACA

CATGCACTGAAATAG---------------------------------------------

----------------

>A_duck_Southwestern_China_B1904_2020_EPI1844098

------------------------ATGGAAGACTTTGTGCGACAATGCTTCAATCCAATG

ATTGTCGAGCTTGCGGAAAAGGCAATGAAAGAATATGGGGAAGATCCTAAGATCGAAACA

AACAAGTTTGCCGCAATATGTACACACTTAGAAGTCTGCTTCATGTATTCGGATTTCCAT

TTCATTGATGAACGAGGCGAATCAATAATCGTAGAATCTGGCGACCCGAATGCATTATTG

AAGCACCGATTTGAGATAATTGAAGGGAGAGACCGAACAATGGCCTGGACAGTGGTGAAT

AGTATATGCAACACTACAGGAGTCGAAAAGCCCAAGTTCCTTCCTGATTTGTATGACTAC

AGAGAAAACCGATTCATTGAAATTGGAGTAACGCGAAGGGAAGTTCACATATACTATCTT

GAAAAAGCCAACAAGATAAAATCAGAGAGAACACACATTCACATATTCTCATTCACAGGG

GAGGAAATGGCCACCAAGGCAGATTACACTCTTGATGAAGAGAGCAGAGCAAGAATAAAA

ACCAGGCTATTCACTATAAGACAAGAGATGGCCAATAGGGGTCTATGGGATTCCTTTCGT

CAGTCCGAGAGAGGCGAAGAGACAATTGAAGAAAGATTTGAAATCACAGGAACCATGCGC

AGGCTTGCTGACCAAAGTCTCCCACCGAACTTCGCCAGCCTTGAAAACTTTAGAGCCTAT

GTGGATGGATTTGAACCGAACGGCTGCATTGAGGGCAAGCTTTCTCAAATGTCAAAAGAA

GTGAATGCCAGAATTGAGCCATTTCTGAAGACAACACCACGCCCTCTCAGATTACCTGAT

GGACCTCCCTGTTCCCAGCGGTCGAAGTTCTTGCTAATGGATGCCCTTAAATTGAGCATT

GAAGACCCGAGCCATGAGGGGGAGGGTATACCGCTGTACGATGCAACCAAATGCATGAAG

ACATTTTTTGGCTGGAAGGAGCCCAACATCGTGAAACCACATGAAAAGGGCATAAACCCT

AATTACCTCCTGGCTTGGAAGCAGGTGCTATCAGAACTCCAAGATATTGAAAACGAGGAG

AAAATCCCAAAAACAAAGAACATGAAGAAGACGAGCCAACTGAAGTGGGCACTTGGTGAA

AACATGGCACCAGAGAAAGTGGAYTTTGAGGACTGTAAAGATGTTAGCGATCTAAGACAG

TACGACAGTGACGAACCAGAATCTAGATCACTAGCAAGTTGGATCCAGAGTGAATTTAAC

AAGGCTTGCGAACTGACAGATTCGAGTTGGATTGAACTTGATGAAATAGGGGAAGACGTT

GCTCCGATTGAACACATTGCGAGTATGAGGAGGAACTATTTCACAGCGGAAGTATCCCAT

TGCAGGGCTACAGAATACATAATGAAAGGAGTATACATAAACACAGCTTTATTGAATGCA

TCCTGTGCAGCCATGGATGACTTCCAACTGATTCCAATGATAAGCAAATGCAGAACCAAA

GAAGGGAGACGGAAGACAAATCTGTATGGCTTCATTATAAAAGGAAGATCCCATTTGAGG

AATGACACCGATGTGGTAAACTTTGTGAGCATGGAATTCTCTCTCACTGACCCAAGGCTG

GAGCCACACAAATGGGAAAAGTACTGTGTGCTTGAGGTAGGAGACATGCTCCTACGGACT

GCAATAGGCCAAGTGTCAAGGCCCATGTTCCTGTATGTGAGAACCAATGGGACTTCTAAG

ATCAAAATGAAATGGGGTATGGAGATGAGGCGATGCCTTCTTCAATCCCTTCAACAAATT

GAGAGCATGATTGAAGCCGAGTCTTCTGTCAAAGAAAAAGACATGACCAAGGAATTCTTT

GAAAACAGATCAGAAACATGGCCAATTGGAGAGTCACCCAAAGGAGTGGAGGAAGGCTCC

ATTGGGAAGGTGTGCAGAACATTGCTAGCAAAGTCTGTGTTCAACAGCCTATATGCATCT

CCACAACTCGAGGGGTTTTCAGCTGAATCAAGAAAGTTGCTTCTCATTGTTCAGGCACTT

AGGGACAACCTGGAACCTGGGACCTTCGATCTTGGGGGGCTATATGAAGCAATTGAGGAG

TGCCTGATTAACGATCCCTGGGTTTTGCTTAATGCGTCTTGGTTCAACTCCTTCCTCACA

CATGCACTGAAATAG---------------------------------------------

----------------

>A_duck_Korea_H411_2020_EPI1845933

------------------------ATGGAAGACTTTGTGCGACAATGCTTCAATCCAATG

ATTGTCGAGCTTGCGGAAAAGGCAATGAAAGAATATGGGGAAGATCCTAAGATCGAAACA

AACAAGTTTGCCGCAATATGTACACACTTAGAAGTCTGCTTCATGTATTCGGATTTCCAT

TTCATTGATGAACGGGGCGAATCAATAATCGTAGAATCTGGCGACCCGAATGCATTATTG

AAGCACCGATTTGAGATAATTGAAGGGAGAGACCGAACAATGGCCTGGACAGTGGTGAAT

AGTATATGCAACACTACAGGAGCCGAAAAGCCCAAGTTCCTTCCTGATTTGTATGACTAC

AGAGAAAACCGATTCATTGAAATTGGAGTAACGCGAAGGGAAGTTCACATATACTATCTT

GAAAAAGCCAACAAGATAAAATCAGAGAAAACACACATTCACATATTCTCATTCACTGGG

GAGGAAATGGCCACCAAGGCAGATTACACTCTTGATGAAGAGAGCAGAGCAAGAATAAAA

ACCAGGCTATTCACTATAAGACAAGAGATGGCCAATAGGGGTCTATGGGATTCCTTTCGT

CAGTCCGAGAGAGGCGAAGAGACAATTGAAGAAAGATTTGAAATCACAGGAACCATGCGC

AGGCTTGCTGACCAGAGTCTCCCACCGAACTTCGCCAGCCTTGAAAACTTTAGAGCCTAT

GTGGATGGATTTGAACCGAACGGCTGCATTGAGGGCAAGCTTTCTCAAATGTCAAAAGAA

GTGAATGCCAGAATTGAGCCATTTCTGAAGACAACACCACGCCCTCTCAGATTACCTGAT

GGACCTCCCTGTTCCCAGCGGTCGAAGTTCTTGCTAATGGATGCCCTTAAATTGAGCATT

GAAGACCCGAGCCATGAGGGGGAGGGTATACCGCTGTACGATGCAACCAAATGCATGAAG

ACATTTTTTGGCTGGAAGGAGCCCAACATCGTGAAACCACATGAAAAGGGCATAAACCCT

AATTACCTCCTGGCTTGGAAGCAGGTGCTATCAGAACTCCAAGATATTGAAAACGAGGAG

AAAATCCCAAAAACAAAGAACATGAAGAAGACGAGCCAACTGAAGTGGGCACTTGGTGAA

AACATGGCACCAGAGAAAGTGGACTTTGAGGACTGTAAAGATGTTAGCGATCTAAGACAG

TACGACAGTGACGAACCAGAATCTAGATCACTAGCAAGTTGGATCCAGAGTGAATTTAAC

AAGGCTTGCGAACTGACAGATTCGAGTTGGATTGAACTTGATGAAATAGGGGAAGACGTT

GCTCCGATTGAACACATTGCGAGTATGAGGAGGAACTATTTCACAGCGGAAGTATCCCAT

TGCAGGGCTACAGAATACATAATGAAAGGAGTATACATAAACACAGCTTTATTGAATGCA

TCCTGTGCAGCCATGGATGACTTCCAACTGATTCCAATGATAAGCAAATGCAGAACCAAA

GAAGGGAGACGGAAGACAAATCTGTATGGCTTCATTATAAAAGGAAGATCCCATTTGAGG

AATGACACCGATGTGGTAAACTTTGTGAGCATGGAATTCTCTCTCACTGACCCAAGGCTG

GAGCCACACAAATGGGAAAAGTACTGTGTGCTTGAGGTAGGAGACATGCTCCTACGGACT

GCAATAGGCCAAGTGTCAAGGCCCATGTTCCTGTATGTGAGAACCAATGGGACTTCTAAG

ATCAAAATGAAATGGGGTATGGAGATGAGGCGATGCCTTCTTCAATCCCTTCAACAAATT

GAGAGCATGATTGAAGCCGAGTCTTCTGTCAAAGAAAAAGACATGACCAAGGAATTCTTT

GAAAACAGATCAGAAACATGGCCAATTGGAGAGTCACCCAAAGGAGTGGAGGAAGGCTCC

ATTGGGAAGGTGTGCAGAACATTGCTAGCAAAGTCTGTGTTCAACAGCCTATATGCATCT

CCACAACTCGAGGGGTTTTCAGCTGAATCAAGAAAGTTGCTTCTCATTGTTCAGGCACTT

AGGGACAACCTGGAACCTGGGACCTTCGATCTTGGGGGGCTATATGAAGCAATTGAGGAG

TGCCTGATTAACGATCCCTGGGTTTTGCTTAATGCGTCTTGGTTCAACTCCTTCCTCACA

CATGCACTGAAATAG---------------------------------------------

----------------

>A_duck_Korea_H431_2020_EPI1845957

------------------------ATGGAAGACTTTGTGCGACAATGCTTCAATCCAATG

ATTGTCGAGCTTGCGGAAAAGGCAATGAAAGAATATGGGGAAGATCCTAAGATCGAAACA

AACAAGTTTGCCGCAATATGTACACACTTAGAAGTCTGCTTCATGTATTCGGATTTCCAT

TTCATTGATGAACGAGGCGAATCAATAATCGTAGAATCTGGCGACCCGAATGCATTATTG

AAGCACCGATTTGAGATAATTGAAGGGAGAGACCGAACAATGGCCTGGACAGTGGTGAAT

AGTATATGCAACACTACAGGAGCCGAAAAGCCCAAGTTCCTTCCTGATTTGTATGACTAC

AGAGAAAACCGATTCATTGAAATTGGAGTAACGCGAAGGGAAGTTCACATATACTATCTT

GAAAAAGCCAACAAGATAAAATCAGAGAAAACACACATTCACATATTCTCATTCACTGGG

GAGGAAATGGCCACCAAGGCAGATTACACTCTTGATGAAGAGAGCAGAGCAAGAATAAAA

ACCAGGCTATTCACTATAAGACAAGAGATGGCCAATAGGGGTCTATGGGATTCCTTTCGT

CAGTCCGAGAGAGGCGAAGAGACAATTGAAGAAAGATTTGAAATCACAGGAACCATGCGC

AGGCTTGCTGACCAGAGTCTCCCACCGAACTTCGCCAGCCTTGAAAACTTTAGAGCCTAT

GTGGATGGATTTGAACCGAACGGCTGCATTGAGGGCAAGCTTTCTCAAATGTCAAAAGAA

GTGAATGCCAGAATTGAGCCATTTCTGAAGACAACACCACGCCCTCTCAGATTACCTGAT

GGACCTCCCTGTTCCCAGCGGTCGAAGTTCTTGCTAATGGATGCCCTTAAATTGAGCATT

GAAGACCCGAGCCATGAGGGGGAGGGTATACCGCTGTACGATGCAACCAAATGCATGAAG

ACATTTTTTGGCTGGAAGGAGCCCAACATCGTGAAACCACATGAAAAGGGCATAAACCCT

AATTACCTCCTGGCTTGGAAGCAGGTGCTATCAGAACTCCAAGATATTGAAAACGAGGAG

AAAATCCCAAAAACAAAGAACATGAAGAAGACGAGCCAACTGAAGTGGGCACTTGGTGAA

AACATGGCACCAGAGAAAGTGGACTTTGAGGACTGTAAAGATGTTAGCGATCTAAGACAG

TACGACAGTGACGAACCAGAATCTAGATCACTAGCAAGTTGGATCCAGAGTGAATTTAAC

AAGGCTTGCGAACTGACAGATTCGAGTTGGATTGAACTTGATGAAATAGGGGAAGACGTT

GCTCCGATTGAACACGTTGCGAGTATGAGGAGGAACTATTTCACAGCGGAAGTATCCCAT

TGCAGGGCTACAGAATACATAATGAAAGGAGTATACATAAACACAGCTTTATTGAATGCA

TCCTGTGCAGCCATGGATGACTTCCAACTGATTCCAATGATAAGCAAATGCAGAACCAAA

GAAGGGAGACGGAAGACAAATCTGTATGGCTTCATTATAAAAGGAAGATCCCATTTGAGG

AATGACACCGATGTGGTAAACTTTGTGAGCATGGAATTCTCTCTCACTGACCCAAGGCTG

GAGCCACACAAATGGGAAAAGTACTGTGTGCTTGAGGTAGGAGACATGCTCCTACGGACT

GCAATAGGCCAAGTGTCAAGGCCCATGTTCCTGTATGTGAGAACCAATGGGACTTCTAAG

ATCAAAATGAAATGGGGTATGGAGATGAGGCGATGCCTTCTTCAATCCCTTCAACAAATT

GAGAGCATGATTGAAGCCGAGTCTTCTGTCAAAGAAAAAGACATGACCAAGGAATTCTTT

GAAAACAGATCAGAAACATGGCCAATTGGAGAGTCACCCAAAGGAGTGGAGGAAGGCTCC

ATTGGGAAGGTGTGCAGAACATTGCTAGCAAAGTCTGTGTTCAACAGCCTATATGCATCT

CCACAACTCGAGGGGTTTTCAGCTGAATCAAGAAAGTTGCTTCTCATTGTTCAGGCACTT

AGGGACAACCTGGAACCTGGGACCTTCGATCTTGGGGGGCTATATGAAGCAATTGAGGAG

TGCCTGATTAACGATCCCTGGGTTTTGCTTAATGCGTCTTGGTTCAACTCCTTCCTCACA

CATGCACTGAAATAG---------------------------------------------

----------------

>A_duck_Korea_H471_2020_EPI1846029

------------------------ATGGAAGACTTTGTGCGACAATGCTTCAATCCAATG

ATTGTCGAGCTTGCGGAAAAGGCAATGAAAGAATATGGGGAAGATCCTAAGATCGAAACA

AACAAGTTTGCCGCAATATGTACACACTTAGAAGTCTGCTTCATGTATTCGGATTTCCAT

TTCATTGATGAACGAGGCGAATCAATAATCGTAGAATCTGGCGACCCGAATGCATTATTG

AAGCACCGATTTGAGATAATTGAAGGGAGAGACCGAACAATGGCCTGGACAGTGGTGAAT

AGTATATGCAACACTACAGGAGCCGAAAAGCCCAAGTTCCTTCCTGATTTGTATGACTAC

AGAGAAAACCGATTCATTGAAATTGGAGTAACGCGAAGGGAAGTTCACATATACTATCTT

GAAAAAGCCAACAAGATAAAATCAGAGAAAACACACATTCACATATTCTCATTCACTGGG

GAGGAAATGGCCACCAAGGCAGATTACACTCTTGATGAAGAGAGCAGAGCAAGAATAAAA

ACCAGGCTATTCACTATAAGACAAGAGATGGCCAATAGGGGTCTATGGGATTCCTTTCGT

CAGTCCGAGAGAGGCGAAGAGACAATTGAAGAAAGATTTGAAATCACAGGAACCATGCGC

AGGCTTGCTGACCAGAGTCTCCCACCGAACTTCGCCAGCCTTGAAAACTTTAGAGCCTAT

GTGGATGGATTTGAACCGAACGGCTGCATTGAGGGCAAGCTTTCTCAAATGTCAAAAGAA

GTGAATGCCAGAATTGAGCCATTTCTGAAGACAACACCACGCCCTCTCAGATTACCTGAT

GGACCTCCCTGTTCCCAGCGGTCGAAGTTCTTGCTAATGGATGCCCTTAAATTGAGCATT

GAAGACCCGAGCCATGAGGGGGAGGGTATACCGCTGTACGATGCAACCAAATGCATGAAG

ACATTTTTTGGCTGGAAGGAGCCCAACATCGTGAAACCACATGAAAAGGGCATAAACCCT

AATTACCTCCTGGCTTGGAAGCAGGTGCTATCAGAACTCCAAGATATTGAAAACGAGGAG

AAAATCCCAAAAACAAAGAACATGAAGAAGACGAGCCAACTGAAGTGGGCACTTGGTGAA

AACATGGCACCAGAGAAAGTGGACTTTGAGGACTGTAAAGATGTTAGCGATCTAAGACAG

TACGACAGTGACGAACCAGAATCTAGATCACTAGCAAGTTGGATCCAGAGTGAATTTAAC

AAGGCTTGCGAACTGACAGATTCGAGTTGGATTGAACTTGATGAAATAGGGGAAGACGTT

GCTCCGATTGAACACATTGCGAGTATGAGGAGGAACTATTTCACAGCGGAAGTATCCCAT

TGCAGGGCTACAGAATACATAATGAAAGGAGTATACATAAACACAGCTTTATTGAATGCA

TCCTGTGCAGCCATGGATGACTTCCAACTGATTCCAATGATAAGCAAATGCAGAACCAAA

GAAGGGAGACGGAAGACAAATCTGTATGGCTTCATTATAAAAGGAAGATCCCATTTGAGG

AATGACACCGATGTGGTAAACTTTGTGAGCATGGAATTCTCTCTCACTGACCCAAGGCTG

GAGCCACACAAATGGGAAAAGTACTGTGTGCTTGAGGTAGGAGACATGCTCCTACGGACT

GCAATAGGCCAAGTGTCAAGGCCCATGTTCCTGTATGTGAGAACCAATGGGACTTCTAAG

ATCAAAATGAAATGGGGTATGGAGATGAGGCGATGCCTTCTTCAATCCCTTCAACAAATT

GAGAGCATGATTGAAGCCGAGTCTTCTGTCAAAGAAAAAGACATGACCAAGGAATTCTTT

GAAAACAGATCAGAAACATGGCCAATTGGAGAGTCACCCAAAGGAGTGGAGGAAGGCTCC

ATTGGGAAGGTGTGCAGAACATTGCTGGCAAAGTCTGTGTTCAACAGCCTATATGCATCT

CCACAACTCGAGGGGTTTTCAGCTGAATCAAGAAAGTTGCTTCTCATTGTTCAGGCACTT

AGGGACAACCTGGAACCTGGGACCTTCGATCTTGGGGGGCTATATGAAGCAATTGAGGAG

TGCCTGATTAACGATCCCTGGGTTTTGCTTAATGCGTCTTGGTTCAACTCCTTCCTCACA

CATGCACTGAAATAG---------------------------------------------

----------------

>A_chicken_Korea_H510_2020_EPI1846053

------------------------ATGGAAGACTTTGTGCGACAATGCTTCAATCCAATG

ATTGTCGAGCTTGCGGAAAAGGCAATGAAAGAATATGGGGAAGATCCTAAGATCGAAACA

AACAAGTTTGCCGCAATATGTACACACTTAGAAGTCTGCTTCATGTATTCGGATTTCCAT

TTCATTGATGAACGAGGCGAATCAATAATCGTAGAATCTGGCGACCCGAATGCATTATTG

AAGCACCGATTTGAGATAATTGAAGGGAGAGACCGAACAATGGCCTGGACAGTGGTGAAT

AGTATATGCAACACTACAGGAGTCGAAAAGCCCAAGTTCCTTCCTGATTTGTATGACTAC

AGAGAAAACCGATTCATTGAAATTGGAGTAACGCGAAGGGAAGTTCACATATACTATCTT

GAAAAAGCCAACAAGATAAAATCAGAGAAAACACACATTCACATATTCTCATTCACTGGG

GAGGAAATGGCCACCAAGGCAGATTACACTCTTGATGAAGAGAGCAGAGCAAGAATAAAA

ACCAGGCTATTCACTATAAGACAAGAGATGGCCAATAGGGGTCTATGGGATTCCTTTCGT

CAGTCCGAGAGAGGCGAAGAGACAATTGAAGAAAGATTTGAAATCACAGGAACCATGCGC

AGGCTTGCTGACCAGAGTCTCCCACCGAACTTCGCCAGCCTTGAAAACTTTAGAGCCTAT

GTGGATGGATTTGAACCGAACGGCTGCATTGAGGGCAAGCTTTCTCAAATGTCAAAAGAA

GTGAATGCCAGAATTGAGCCATTTCTGAAGACAACACCACGCCCTCTCAGATTACCTGAT

GGACCTCCCTGTTCCCAGCGGTCGAAGTTCTTGCTAATGGATGCCCTTAAATTGAGCATT

GAAGACCCGAGCCATGAGGGGGAGGGTATACCGCTGTACGATGCAACCAAATGCATGAAG

ACATTTTTTGGCTGGAAGGAGCCCAACATCGTGAAACCACATGAAAAGGGCATAAACCCT

AACTACCTCCTGGCTTGGAAGCAGGTGCTATCAGAACTCCAAGATATTGAAAACGAGGAG

AAAATCCCAAAAACAAAGAACATGAAGAAGACGAGCCAACTGAAGTGGGCACTTGGTGAA

AACATGGCACCAGAGAAAGTGGACTTTGAGGACTGTAAAGATGTTAGCGATCTAAGACAG

TACGACAGTGACGAACCAGAATCTAGATCACTAGCAAGTTGGATCCAGAGTGAATTTAAC

AAGGCTTGCGAACTGACAGATTCGAGTTGGATTGAACTTGATGAAATAGGGGAAGACGTT

GCTCCGATTGAACACATTGCGAGTATGAGGAGGAACTATTTCACAGCGGAAGTATCCCAT

TGCAGGGCTACAGAATACATAATGAAAGGAGTATACATAAACACAGCTTTATTGAATGCA

TCCTGTGCAGCCATGGATGACTTCCAACTGATTCCAATGATAAGCAAATGCAGAACCAAA

GAAGGGAGACGGAAGACAAATCTGTATGGCTTCATTATAAAAGGAAGATCCCATTTGAGG

AATGACACCGATGTGGTAAACTTTGTGAGCATGGAATTCTCTCTCACTGACCCAAGGCTG

GAGCCACACAAATGGGAAAAGTACTGTGTGCTTGAGGTAGGAGACATGCTCCTACGGACT

GCAATAGGCCAAGTGTCAAGGCCCATGTTCCTGTATGTGAGAACCAATGGAACTTCTAAG

ATCAAAATGAAATGGGGTATGGAGATGAGGCGATGCCTTCTTCAATCCCTTCAACAAATT

GAGAGCATGATTGAAGCCGAGTCTTCTGTCAAAGAAAAAGACATGACCAAGGAATTCTTT

GAAAACAGATCAGAAACATGGCCAATTGGAGAGTCACCCAAAGGAGTGGAGGAAGGCTCC

ATTGGGAAGGTGTGCAGAACATTGCTAGCAAAGTCTGTGTTCAACAGCCTATATGCATCT

CCACAACTCGAGGGGTTTTCAGCTGAATCAAGAAAGTTGCTTCTCATTGTTCAGGCACTT

AGGGACAACCTGGAACCTGGGACCTTCGATCTTGGGGGGCTATATGAAGCAATTGAGGAG

TGCCTGATTAACGATCCCTGGGTTTTGCTTAATGCGTCTTGGTTCAACTCCTTCCTCACA

CATGCACTGAAATAG---------------------------------------------

----------------

>A_duck_Korea_H538_2020_EPI1846149

------------------------ATGGAAGACTTTGTGCGACAATGCTTCAATCCAATG

ATTGTCGAGCTTGCGGAAAAGGCAATGAAAGAATATGGGGAAGATCCTAAGATCGAAACA

AACAAGTTTGCCGCAATATGTACACACTTAGAAGTCTGCTTCATGTATTCGGATTTCCAT

TTCATTGATGAACGAGGCGAATCAATAATCGTAGAATCTGGCGACCCGAATGCATTATTG

AAGCACCGATTTGAGATAATTGAAGGGAGAGACCGAACAATGGCCTGGACAGTGGTGAAT

AGTATATGCAACACTACAGGAGCCGAAAAGCCCAAGTTCCTTCCTGATTTGTATGACTAC

AGAGAAAACCGATTCATTGAAATTGGAGTAACGCGAAGGGAAGTTCACATATACTATCTT

GAAAAAGCCAACAAGATAAAATCAGAGAAAACACACATTCACATATTCTCATTCACTGGG

GAGGAAATGGCCACCAAGGCAGATTACACTCTTGATGAAGAGAGCAGAGCAAGAATAAAA

ACCAGGCTATTCACTATAAGACAAGAGATGGCCAATAGGGGTCTATGGGATTCCTTTCGT

CAGTCCGAGAGAGGCGAAGAGACAATTGAAGAAAGATTTGAAATCACAGGAACCATGCGC

AGGCTTGCTGACCAGAGTCTCCCACCGAACTTCGCCAGCCTTGAAAACTTTAGAGCCTAT

GTGGATGGATTTGAACCGAACGGCTGCATTGAGGGCAAGCTTTCTCAAATGTCAAAAGAA

GTGAATGCCAGAATTGAGCCATTTCTGAAGACAACACCACGCCCTCTCAGATTACCTGAT

GGACCTCCCTGTTCCCAGCGGTCGAAGTTCTTGCTAATGGATGCCCTTAAATTGAGCATT

GAAGACCCGAGCCATGAGGGGGAGGGTATACCGCTGTACGATGCAACCAAATGCATGAAG

ACATTTTTTGGCTGGAAGGAGCCCAACATCGTGAAACCACATGAAAAGGGCATAAACCCT

AATTACCTCCTGGCTTGGAAGCAGGTGCTATCAGAACTCCAAGATATTGAAAACGAGGAG

AAAATCCCAAAAACAAAGAACATGAAGAAGACGAGCCAACTGAAGTGGGCACTTGGTGAA

AACATGGCACCAGAGAAAGTGGACTTTGAGGACTGTAAAGATGTTAGCGATCTAAGACAG

TACGACAGTGACGAACCAGAATCTAGATCACTAGCAAGTTGGATCCAGAGTGAATTTAAC

AAGGCTTGCGAACTGACAGATTCGAGTTGGATTGAACTTGATGAAATAGGGGAAGACGTT

GCTCCGATTGAACACATTGCGAGTATGAGGAGGAACTATTTCACAGCGGAAGTATCCCAT

TGCAGGGCTACAGAATACATAATGAAAGGAGTATACATAAACACAGCTTTATTGAATGCA

TCCTGTGCAGCCATGGATGACTTCCAACTGATTCCAATGATAAGCAAATGCAGAACCAAA

GAAGGGAGACGGAAGACAAATCTGTATGGCTTCATTATAAAAGGAAGATCCCATTTGAGG

AATGACACCGATGTGGTAAACTTTGTGAGCATGGAATTCTCTCTCACTGACCCAAGGCTG

GAGCCACACAAATGGGAAAAGTACTGTGTGCTTGAGGTAGGAGACATGCTCCTACGGACT

GCAATAGGCCAAGTGTCAAGGCCCATGTTCCTGTATGTGAGAACCAATGGGACTTCTAAG

ATCAAAATGAAATGGGGTATGGAGATGAGGCGATGCCTTCTTCAATCCCTTCAACAAATT

GAGAGCATGATTGAAGCCGAGTCTTCTGTCAAAGAAAAAGACATGACCAAGGAATTCTTT

GAAAACAGATCAGAAACATGGCCAATTGGAGAGTCACCCAAAGGAGTGGAGGAAGGCTCC

ATTGGGAAGGTGTGCAGAACATTGCTAGCAAAGTCTGTGTTCAACAGCCTATATGCATCT

CCACAACTCGAGGGGTTTTCAGCTGAATCAAGAAAGTTGCTTCTCATTGTTCAGGCACTT

AGGGACAACCTGGAACCTGGGACCTTCGATCTTGGGGGGCTATATGAAGCAATTGAGGAG

TGCCTGATTAACGATCCCTGGGTTTTGCTTAATGCGTCTTGGTTCAACTCCTTCCTCACA

CATGCACTGAAATAG---------------------------------------------

----------------

>A_chicken_Tyumen_302-01_2020_EPI1848602

AGCAAAAGCAGGTACTGATCCAAAATGGAAGACTTTGTGCGACAATGCTTCAATCCAATG

ATTGTCGAGCTTGCGGAAAAGGCAATGAAAGAATATGGGGAAGATCCTAAGATCGAAACA

AACAAGTTTGCCGCAATATGTACACACTTAGAAGTCTGCTTCATGTATTCGGATTTCCAT

TTCATTGATGAACGAGGCGAATCAATAATCGTAGAATCTGGCGACCCGAATGCATTATTG

AAGCACCGATTTGAGATAATTGAAGGGAGAGACCGAACAATGGCCTGGACAGTGGTGAAT

AGTATATGCAACACTACAGGAGTCGAAAAGCCCAAGTTCCTTCCTGATTTGTATGACTAC

AGAGAAAACCGATTCATTGAAATTGGAGTAACGCGAAGGGAAGTTCACATATACTATCTT

GAAAAAGCCAACAAGATAAAATCAGAGAAAACACACATTCACATATTCTCATTCACTGGG

GAGGAAATGGCCACCAAGGCAGATTACACTCTTGATGAAGAGAGCAGAGCAAGAATAAAA

ACCAGGCTATTCACTATAAGACAAGAGATGGCCAATAGGGGTCTATGGGATTCCTTTCGT

CAGTCCGAGAGAGGCGAAGAGACAATTGAAGAAAGATTTGAAATCACAGGAACCATGCGC

AGGCTTGCTGACCAAAGTCTCCCACCGAACTTCGCCAGCCTTGAAAACTTTAGAGCCTAT

GTGGATGGATTTGAACCGAACGGCTGCATTGAGGGCAAGCTTTCTCAAATGTCAAAAGAA

GTGAATGCCAGAATTGAGCCATTTCTGAAGACAACACCACGCCCTCTCAGATTACCTGAT

GGACCTCCCTGTTCCCAGCGGTCGAAGTTCTTGCTAATGGATGCCCTTAAATTGAGCATT

GAAGACCCGAGCCATGAGGGGGAGGGTATACCGCTGTACGATGCAACCAAATGCATGAAG

ACATTTTTTGGCTGGAAGGAGCCCAACATCGTGAAACCACATGAAAAGGGCATAAACCCT

AATTACCTCCTGGCTTGGAAGCAGGTGCTATCAGAACTCCAAGATATTGAAAACGAGGAG

AAAATCCCAAAAACAAAGAACATGAAGAAGACGAGCCAACTGAAGTGGGCACTTGGTGAA

AACATGGCACCAGAGAAAGTGGACTTTGAGGACTGTAAAGATGTTAGCGATCTAAGACAG

TACGACAGTGACGAACCAGAATCTAGATCACTAGCAAGTTGGATCCAGAGTGAATTTAAC

AAGGCTTGCGAACTGACAGATTCGAGTTGGATTGAACTTGATGAAATAGGGGAAGACGTT

GCTCCAATTGAACACATTGCGAGTATGAGGAGGAACTATTTCACAGCGGAAGTATCCCAT

TGCAGGGCTACAGAATACATAATGAAAGGAGTATACATAAACACAGCTTTATTGAATGCA

TCCTGTGCAGCCATGGATGACTTCCAACTGATTCCAATGATAAGCAAATGCAGAACCAAA

GAAGGGAGACGGAAGACAAATCTGTATGGCTTCATTATAAAAGGAAGATCCCATTTGAGG

AACGACACCGATGTGGTAAACTTTGTGAGCATGGAATTCTCTCTCACTGACCCAAGGCTG

GAGCCACACAAATGGGAAAAGTACTGTGTGCTTGAGGTAGGAGACATGCTCCTACGGACT

GCAATAGGCCAAGTGTCAAGGCCCATGTTCCTGTATGTGAGAACCAATGGGACTTCTAAG

ATCAAAATGAAATGGGGTATGGAGATGAGGCGATGCCTTCTTCAATCCCTTCAACAAATT

GAGAGCATGATTGAAGCCGAGTCTTCTGTCAAAGAAAAAGACATGACCAAGGAATTCTTT

GAAAACAGATCAGAAACATGGCCAATTGGAGAGTCACCCAAAGGAGTGGAGGAAGGCTCC

ATTGGGAAGGTGTGCAGAACATTGCTAGCAAAGTCTGTGTTCAACAGCCTATATGCATCT

CCACAACTCGAGGGGTTTTCAGCTGAATCAAGAAAGTTGCTTCTCATTGTTCAGGCACTT

AGGGACAACCTGGAACCTGGAACCTTCGATCTTGGGGGGCTATATGAAGCAATTGAGGAG

TGCCTGATTAACGATCCCTGGGTTTTGCTTAATGCGTCTTGGTTCAACTCCTTCCTCACA

CATGCACTGAAATAGTCGTGGCAATGCTACTA---TTTGCTATCCATACTGTCCAAAAA-

GTACCTTGTTTCTACT

>A_chicken_Tyumen_302-02_2020_EPI1848610

AGCAAAAGCAGGTACTGATCCAAAATGGAAGACTTTGTGCGACAATGCTTCAATCCAATG

ATTGTCGAGCTTGCGGAAAAGGCAATGAAAGAATATGGGGAAGATCCTAAGATCGAAACA

AACAAGTTTGCCGCAATATGTACACACTTAGAAGTCTGCTTCATGTATTCGGATTTCCAT

TTCATTGATGAACGAGGCGAATCAATAATCGTAGAATCTGGCGACCCGAATGCATTATTG

AAGCACCGATTTGAGATAATTGAAGGGAGAGACCGAACAATGGCCTGGACAGTGGTGAAT

AGTATATGCAACACTACAGGAGTCGAAAAGCCCAAGTTCCTTCCTGATTTGTATGACTAC

AGAGAAAACCGATTCATTGAAATTGGAGTAACGCGAAGGGAAGTTCACATATACTATCTT

GAAAAAGCCAACAAGATAAAATCAGAGAAAACACACATTCACATATTCTCATTCACTGGG

GAGGAAATGGCCACCAAGGCAGATTACACTCTTGATGAAGAGAGCAGAGCAAGAATAAAA

ACCAGGCTATTCACTATAAGACAAGAGATGGCCAATAGGGGTCTATGGGATTCCTTTCGT

CAGTCCGAGAGAGGCGAAGAGACAATTGAAGAAAGATTTGAAATCACAGGAACCATGCGC

AGGCTTGCTGACCAAAGTCTCCCACCGAACTTCGCCAGCCTTGAAAACTTTAGAGCCTAT

GTGGATGGATTTGAACCGAACGGCTGCATTGAGGGCAAGCTTTCTCAAATGTCAAAAGAA

GTGAATGCCAGAATTGAGCCATTTCTGAAGACAACACCACGCCCTCTCAGATTACCTGAT

GGACCTCCCTGTTCCCAGCGGTCGAAGTTCTTGCTAATGGATGCCCTTAAATTGAGCATT

GAAGACCCGAGCCATGAGGGGGAGGGTATACCGCTGTACGATGCAACCAAATGCATGAAG

ACATTTTTTGGCTGGAAGGAGCCCAACATCGTGAAACCACATGAAAAGGGCATAAACCCT

AATTACCTCCTGGCTTGGAAGCAGGTGCTATCAGAACTCCAAGATATTGAAAACGAGGAG

AAAATCCCAAAAACAAAGAACATGAAGAAGACGAGCCAACTGAAGTGGGCACTTGGTGAA

AACATGGCACCAGAGAAAGTGGACTTTGAGGACTGTAAAGATGTTAGCGATCTAAGACAG

TACGACAGTGACGAACCAGAATCTAGATCACTAGCAAGTTGGATCCAGAGTGAATTTAAC

AAGGCTTGCGAACTGACAGATTCGAGTTGGATTGAACTTGATGAAATAGGGGAAGACGTT

GCTCCAATTGAACACATTGCGAGTATGAGGAGGAACTATTTCACAGCGGAAGTATCCCAT

TGCAGGGCTACAGAATACATAATGAAAGGAGTATACATAAACACAGCTTTATTGAATGCA

TCCTGTGCAGCCATGGATGACTTCCAACTGATTCCAATGATAAGCAAATGCAGAACCAAA

GAAGGGAGACGGAAGACAAATCTGTATGGCTTCATTATAAAAGGAAGATCCCATTTGAGG

AACGACACCGATGTGGTAAACTTTGTGAGCATGGAATTCTCTCTCACTGACCCAAGGCTG

GAGCCACACAAATGGGAAAAGTACTGTGTGCTTGAGGTAGGAGACATGCTCCTGCGGACT

GCAATAGGCCAAGTGTCAAGGCCCATGTTCCTGTATGTGAGAACCAATGGGACTTCTAAG

ATCAAAATGAAATGGGGTATGGAGATGAGGCGATGCCTTCTTCAATCCCTTCAACAAATT

GAGAGCATGATTGAAGCCGAGTCTTCTGTCAAAGAAAAAGACATGACCAAGGAATTCTTT

GAAAACAGATCAGAAACATGGCCAATTGGAGAGTCACCCAAAGGAGTGGAGGAAGGCTCC

ATTGGGAAGGTGTGCAGAACATTGCTAGCAAAGTCTGTGTTCAACAGCCTATATGCATCT

CCACAACTCGAGGGGTTTTCAGCTGAATCAAGAAAGTTGCTTCTCATTGTTCAGGCACTT

AGGGACAACCTGGAACCTGGAACCTTCGATCTTGGGGGGCTATATGAAGCAATTGAGGAG

TGCCTGATTAACGATCCCTGGGTTTTGCTTAATGCGTCTTGGTTCAACTCCTTCCTCACA

CATGCACTGAAATAGTCGTGGCAATGCTACTA---TTTGCTATCCATACTGTCCAAAAA-

GTACCTTGTTTCTACT

>A_chicken_Poland_474_2020_EPI1850192

-----------GTACTGATCCAAAATGGAAGACTTTGTGCGACAATGCTTCAATCCAATG

ATTGTCGAGCTTGCGGAAAAGGCAATGAAAGAATATGGGGAAGATCCTAAGATCGAAACA

AACAAGTTTGCCGCAATATGTACACACTTAGAAGTCTGCTTCATGTATTCGGATTTCCAT

TTCATTGATGAACGAGGCGAATCAATAATCGTAGAATCTGGCGACCCGAATGCATTATTG

AAGCACCGATTTGAGATAATTGAAGGGAGAGACCGAACAATGGCCTGGACAGTGGTGAAT

AGTATATGCAACACTACAGGAGTCGAAAAGCCCAAGTTCCTTCCTGATTTGTATGACTAC

AGAGAAAACCGATTCATTGAAATTGGAGTAACGCGAAGGGAAGTTCACATATACTATCTT

GAAAAAGCCAACAAAATAAAATCAGAGAAAACACACATTCACATATTCTCATTCACTGGG

GAGGAAATGGCCACCAAGGCAGATTACACTCTTGATGAAGAGAGCAGAGCAAGAATAAAA

ACCAGGCTATTCACTATAAGACAAGAGATGGCCAATAGGGGTCTATGGGATTCCTTTCGT

CAGTCCGAGAGAGGCGAAGAGACAATTGAAGAAAGATTTGAAATCACAGGAACCATGCGC

AGGCTTGCTGACCAAAGTCTCCCACCGAACTTCGCCAGCCTTGAAAATTTTAGAGCCTAT

GTGGATGGATTTGAACCGAACGGCTGCATTGAGGGCAAGCTTTCTCAAATGTCAAAAGAA

GTGAATGCCAGAATTGAGCCATTTCTGAAGAATACACCACGCCCTCTCAGATTGCCTGAT

GGACCTCCCTGTTCCCAGCGGTCGAAGTTCTTGCTAATGGATGCCCTTAAATTAAGCATT

GAAGACCCGAGCCATGAGGGGGAGGGTATACCGCTGTACGATGCAACCAAATGCATGAAG

ACATTTTTTGGCTGGAAGGAGCCCAACATCGTGAAACCACATGAAAAGGGCATAAACCCT

AATTACCTCCTGGCTTGGAAGCAGGTGCTATCAGAACTCCAAGATATTGAAAACGAGGAG

AAAATCCCAAAAACAAAGAACATGAAGAAGACGAGCCAACTGAAGTGGGCACTTGGTGAA

AATATGGCACCAGAGAAAGTGGACTTTGAGGACTGTAAAGATGTTAGCGATCTAAAACAG

TACGACAGTGACGAACCAGAATCCAGATCACTAGCAAGTTGGATCCAGAGTGAATTTAAC

AAGGCTTGCGAACTGACAGATTCGAGTTGGATTGAACTTGATGAAATAGGGGAAGACGTT

GCTCCAATTGAACACATTGCGAGTATGAGGAGGAACTATTTCACAGCGGAAGTATCCCAT

TGCAGGGCTACAGAATACATAATGAAAGGAGTATACATAAACACAGCTTTATTGAATGCA

TCCTGTGCAGCCATGGATGACTTCCAACTGATTCCAATGATAAGCAAATGCAGAACCAAA

GAAGGGAGACGGAAGACAAATCTGTATGGCTTCATTATAAAAGGAAGATCCCATTTGAGG

AATGACACCGATGTGGTAAACTTTGTGAGCATGGAATTCTCTCTCACTGACCCAAGGCTG

GAGCCACACAAATGGGAAAAGTACTGTGTGCTTGAGGTAGGAGACATGCTCCTACGGACT

GCAATAGGCCAAGTGTCAAGGCCCATGTTCCTGTATGTGAGAACCAATGGGACTTCTAAG

ATCAAAATGAAATGGGGTATGGAGATGAGGCGATGCCTTCTTCAATCCCTTCAACAAATT

GAGAGCATGATTGAAGCCGAGTCTTCTGTCAAAGAAAAAGACATGACCAAGGAATTCTTT

GAAAACAGATCAGAAACATGGCCAATTGGAGAGTCACCCAAAGGAGTGGAAGAAGGCTCC

ATTGGGAAGGTGTGCAGAACATTGCTAGCAAAGTCTGTGTTCAATAGCCTATATGCATCT

CCACAACTCGAGGGGTTTTCAGCTGAATCAAGAAAGTTGCTTCTCATTGTTCAGGCACTT

AGGGACAACCTGGAACCTGGAACCTTCGATCTTGGGGGGCTATATGAAGCAATTGAGGAG

TGCCTGATTAACGATCCCTGGGTTTTGCTTAATGCGTCTTGGTTCAACTCCTTCCTCACA

CATGCACTGAAATAGTCGTGGCAATGCTACTA---TTTGCTATCCATACTGTCCAAAAAA

GTA-------------

>A_swan_Poland_MB141_2020_EPI1850215

-----------GTACTGATCCAAAATGGAAGACTTTGTGCGACAATGCTTCAATCCAATG

ATTGTCGAGCTTGCGGAAAAGGCAATGAAAGAATATGGGGAAGATCCTAAGATCGAAACA

AACAAGTTTGCCGCAATATGTACACACTTAGAAGTCTGCTTCATGTATTCGGATTTCCAT

TTCATTGATGAACGAGGCGAATCAATAATCGTAGAATCTGGCGACCCGAATGCATTATTG

AAGCACCGATTTGAGATAATTGAAGGGAGAGACCGAACAATGGCCTGGACAGTGGTGAAT

AGTATATGCAACACTACAGGAGTCGAAAAGCCCAAGTTCCTTCCTGATTTGTATGACTAC

AGAGAAAACCGATTCATTGAAATTGGAGTAACGCGAAGGGAAGTTCACATATACTATCTT

GAAAAAGCCAACAAAATAAAATCAGAGAAAACACACATTCACATATTCTCATTCACTGGG

GAGGAAATGGCCACCAAGGCAGATTACACTCTTGATGAAGAGAGCAGAGCAAGAATAAAA

ACCAGGCTATTCACTATAAGACAAGAGATGGCCAATAGGGGTCTATGGGATTCCTTTCGT

CAGTCCGAGAGAGGCGAAGAGACAATTGAAGAAAGATTTGAAATCACAGGAACCATGCGC

AGGCTTGCTGACCAAAGTCTCCCACCGAACTTCGCCAGCCTTGAAAATTTTAGAGCCTAT

GTGGATGGATTTGAACCGAACGGCTGCATTGAGGGCAAGCTTTCTCAAATGTCAAAAGAA

GTGAATGCCAGAATTGAGCCATTTCTGAAGACTACACCACGCCCTCTCAGATTGCCTGAT

GGACCTCCCTGTTCCCAGCGGTCGAAGTTCTTGCTAATGGATGCCCTTAAATTAAGCATT

GAAGACCCGAGCCATGAGGGGGAGGGTATACCGCTGTACGATGCAACCAAATGCATGAAG

ACATTTTTTGGCTGGAAGGAGCCCAACATCGTGAAACCACATGAAAAGGGCATAAACCCT

AATTACCTCCTGGCTTGGAAGCAGGTGCTATCAGAACTCCAAGATATTGAAAACGAGGAG

AAAATCCCAAAAACAAAGAACATGAAGAAGACGAGCCAACTGAAGTGGGCACTTGGTGAA

AATATGGCACCAGAGAAAGTGGACTTTGAGGACTGTAAAGATGTTAGCGATCTAAAACAG

TACGACAGTGACGAACCAGAATCCAGATCACTAGCAAGTTGGATCCAGAGTGAATTTAAC

AAGGCTTGCGAACTGACAGATTCGAGTTGGATTGAACTTGATGAAATAGGGGAAGACGTT

GCTCCAATTGAACACATTGCGAGTATGAGGAGGAACTATTTCACAGCGGAAGTATCCCAT

TGCAGGGCTACAGAATACATAATGAAAGGAGTATACATAAACACAGCTTTATTGAATGCA

TCCTGTGCAGCCATGGATGACTTCCAACTGATTCCAATGATAAGCAAATGCAGAACCAAA

GAAGGGAGACGGAAGACAAATCTGTATGGCTTCATTATAAAAGGAAGATCCCATTTGAGG

AATGACACCGATGTGGTAAACTTTGTGAGCATGGAATTCTCTCTCACTGACCCAAGGCTG

GAGCCACACAAATGGGAAAAGTACTGTGTGCTTGAGGTAGGAGACATGCTCCTACGGACT

GCAATAGGCCAAGTGTCAAGGCCCATGTTCCTGTATGTGAGAACCAATGGGACTTCTAAG

ATCAAAATGAAATGGGGTATGGAGATGAGGCGATGCCTTCTTCAATCCCTTCAACAAATT

GAGAGCATGATTGAAGCCGAGTCTTCTGTCAAAGAAAAAGACATGACCAAGGAATTCTTT

GAAAACAGATCAGAAACATGGCCAATTGGAGAGTCACCCAAAGGAGTGGAGGAAGGCTCC

ATTGGGAAGGTGTGCAGAACATTGCTAGCAAAGTCTGTGTTCAATAGCCTATATGCATCT

CCACAACTCGAGGGGTTTTCAGCTGAATCAAGAAAGTTGCTTCTCATTGTTCAGGCACTT

AGGGACAACCTGGAACCTGGAACCTTCGATCTTGGGGGGCTATATGAAGCAATTGAGGAG

TGCCTGATTAACGATCCCTGGGTTTTGCTTAATGCGTCTTGGTTCAACTCCTTCCTCACA

CATGCACTGAAATAGTCGTGGCAATGCTACTA---TTTGCTATCCATACTGTCCAAAAAA

GTA-------------

>A_muscovy_duck_Slovakia_Pah1_21VIR1086-1_2021_EPI1858240

------------------------ATGGAAGACTTTGTGCGACAATGCTTCAATCCAATG

ATTGTCGAGCTTGCGGAAAAGGCAATGAAAGAATATGGGGAAGATCCTAAGATCGAAACA

AACAAGTTTGCCGCAATATGTACACACTTAGAAGTCTGCTTCATGTATTCGGATTTCCAT

TTCATTGATGAACGAGGCGAATCAATAATCGTAGAATCTGGCGACCCGAATGCATTATTG

AAGCACCGATTTGAGATAATTGAAGGGAGAGACCGAACAATGGCCTGGACAGTGGTGAAT

AGTATATGCAACACTACAGGAGTCGAAAAGCCCAAGTTCCTTCCTGATTTGTATGACTAC

AGAGAAAACCGATTCATTGAAATTGGAGTAACGCGAAGGGAAGTTCACATATACTATCTT

GAAAAAGCCAACAAGATAAAATCAGAGAAAACACACATTCACATATTCTCATTCACTGGG

GAGGAAATGGCCACCAAGGCAGATTACACTCTTGATGAAGAGAGCAGAGCAAGAATAAAA

ACCAGGCTATTCACTATAAGACAAGAGATGGCCAATAGGGGTATATGGGATTCCTTTCGT

CAGTCCGAGAGAGGCGAAGAGACAATTGAAGAAAGATTTGAAATCACAGGAACCATGCGC

AGGCTTGCTGACCAAAGTCTCCCACCGAACTTCGCAAGCCTTGAAAACTTTAGAGCCTAT

GTGGATGGATTTGAACCGAACGGCTGCATTGAGGGCAAGCTTTCTCAAATGTCAAAAGAA

GTGAATGCCAGAATTGAGCCATTTCTAAAGACAACACCACGCCCTCTCAGATTACCTGAT

GGACCTCCCTGTTCCCAGCGGTCGAAGTTCTTGCTAATGGATGCCCTTAAATTGAGCATT

GAAGACCCGAGCCATGAGGGGGAGGGTATACCGCTGTACGATGCAACCAAATGCATGAAG

ACATTTTTTGGCTGGAAGGAGCCCAACATCGTGAAACCACATGAAAAGGGCATAAACCCT

AATTACCTCCTGGCTTGGAAGCAGGTGCTATCAGAACTCCAAGATATTGAAAACGAGGAG

AAAATCCCAAAAACAAAGAACATGAAGAAGACGAGCCAACTGAAGTGGGCACTTGGTGAA

AACATGGCACCAGAGAAAGTGGACTTTGAGGACTGTAAAGATGTTAGCGATCTAAGACAG

TACGACAGTGACGAACCAGAATCTAGATCACTAGCAAGTTGGATCCAGAGTGAATTTAAC

AAGGCTTGCGAACTGACAGATTCGAGTTGGATTGAACTTGATGAAATAGGGGAAGACGTT

GCTCCAATTGAACACATTGCGAGTATGAGGAGGAACTATTTCACAGCGGAAGTATCCCAT

TGCAGGGCTACAGAATACATAATGAAAGGAGTATACATAAACACAGCTTTATTGAATGCA

TCCTGTGCAGCCATGGATGACTTCCAACTGATTCCAATGATAAGCAAATGCAGAACCAAA

GAAGGGAGACGGAAGACAAATCTGTATGGCTTCATTATAAAAGGAAGATCCCATTTGAGG

AATGACACCGATGTGGTAAACTTTGTGAGCATGGAATTCTCTCTCACTGACCCAAGGCTG

GAGCCACACAAATGGGAAAAGTACTGTGTGCTTGAGGTAGGAGACATGCTCCTACGGACT

GCAATAGGCCAAGTGTCAAGGCCCATGTTCCTGTATGTGAGAACCAATGGGACTTCTAAG

ATCAAAATGAAATGGGGTATGGAGATGAGGCGATGCCTTCTTCAATCCCTTCAACAAATT

GAGAGCATGATTGAAGCCGAGTCATCTGTCAAAGAAAAAGACATGACCAAGGAATTCTTT

GAAAACAGATCAGAAACATGGCCAATTGGAGAGTCACCCAAAGGAGTGGAGGAAGGCTCC

ATTGGGAAGGTGTGCAGAACATTGCTAGCAAAGTCTGTGTTCAACAGCCTATATGCATCT

CCACAACTCGAGGGGTTTTCAGCTGAATCAAGAAAGTTGCTTCTCATTGTTCAGGCACTT

AGGGACAACCTGGAACCTGGAACCTTCGATCTTGGGGGGCTATATGAAGCAATTGAGGAG

TGCCTGATTAACGATCCCTGGGTTTTGCTTAATGCGTCTTGGTTCAACTCCTTCCTCACA

CATGCACTGAAATAG---------------------------------------------

----------------

>A_mute_swan_Slovenia_1639-20_21VIR959-1_2020_EPI1858296

------------------------ATGGAAGACTTTGTGCGACAATGCTTCAATCCAATG

ATTGTCGAGCTTGCGGAAAAGGCAATGAAAGAATATGGGGAAGATCCTAAGATCGAAACA

AACAAGTTTGCCGCAATATGTACGCACTTAGAAGTCTGCTTCATGTATTCGGATTTCCAT

TTCATTGATGAACGAGGCGAATCAATAATCGTAGAATCTGGCGACCCGAATGCATTATTG

AAGCACCGATTTGAGATAATTGAAGGGAGAGACCGAACAATGGCCTGGACAGTGGTGAAT

AGTATATGCAACACTACAGGAGTCGAAAAGCCCAAGTTCCTTCCTGATTTGTATGACTAC

AGAGAAAACCGATTCATTGAAATTGGAGTAACGCGAAGGGAAGTTCACATATACTATCTT

GAAAAAGCCAACAAGATAAAATCAGAGAAAACACACATTCACATATTCTCATTCACTGGG

GAGGAAATGGCCACCAAGGCAGATTACACTCTTGATGAAGAGAGCAGAGCAAGAATAAAA

ACCAGGCTATTCACTATAAGACAAGAGATGGCCAATAGGGGTCTATGGGATTCCTTTCGT

CAGTCCGAGAGAGGCGAAGAAACAATTGAAGAAAGATTTGAAATCACAGGAACCATGCGC

AGGCTTGCTGACCAAAGTCTCCCACCGAACTTCGCCAGCCTTGAAAACTTTAGAGCCTAT

GTGGATGGATTTGAACCGAACGGCTGCATTGAGGGCAAGCTTTCTCAAATGTCAAAAGAA

GTGAATGCCAGAATTGAGCCATTTCTGAAGACAACACCACGCCCTCTCAGATTACCTGAT

GGACCTCCCTGTTCCCAGCGGTCGAAGTTCTTGCTAATGGATGCCCTTAAATTGAGCATT

GAAGACCCGAGCCATGAGGGGGAGGGTATACCGCTGTACGATGCAACCAAATGCATGAAG

ACATTTTTTGGCTGGAAGGAGCCCAACATCGTGAAACCACATGAAAAGGGCATAAACCCT

AATTACCTCCTGGCTTGGAAGCAGGTGCTATCAGAACTCCAAGATATTGAAAACGAGGAG

AAAATCCCAAAAACAAAGAACATGAAGAAGACGAGCCAACTGAAGTGGGCACTTGGTGAA

AACATGGCACCAGAGAAAGTGGACTTTGAGGACTGTAAAGATGTTAGCGATCTAAGACAG

TACGACAGTGACGAACCAGAATCTAGATCACTAGCAAGTTGGATCCAGAGTGAATTTAAC

AAGGCTTGCGAACTGACAGATTCAAGTTGGATTGAACTTGATGAAATAGGGGAAGACGTT

GCTCCGATTGAACACATTGCGAGTATGAGGAGGAACTATTTCACAGCGGAAGTATCCCAT

TGCAGGGCTACAGAATACATAATGAAAGGAGTATACATAAACACAGCTTTATTGAATGCA

TCCTGTGCAGCCATGGATGACTTCCAACTGATTCCAATGATAAGCAAATGCAGAACCAAA

GAAGGGAGACGGAAGACAAATCTGTATGGCTTCATTATAAAAGGAAGATCCCATTTGAGG

AATGACACCGATGTGGTAAACTTTGTGAGCATGGAATTCTCTCTCACTGACCCAAGGCTG

GAGCCACACAAATGGGAAAAGTACTGTGTGCTTGAGGTAGGAGACATGCTCCTACGGACT

GCAATAGGCCAAGTGTCAAGGCCCATGTTCCTGTATGTGAGAACCAATGGGACTTCTAAG

ATCAAAATGAAATGGGGTATGGAGATGAGGCGATGCCTTCTTCAATCCCTTCAACAAATT

GAGAGCATGATTGAAGCCGAGTCTTCTGTCAAAGAAAAAGACATGACCAAGGAATTCTTT

GAAAACAGATCAGAAACATGGCCAATTGGAGAGTCACCCAAAGGAGTGGAGGAAGGCTCC

ATTGGGAAGGTGTGCAGAACATTGCTAGCAAAGTCTGTGTTCAACAGCCTATATGCATCT

CCACAACTCGAGGGGTTTTCAGCTGAATCAAGAAAGTTGCTTCTCATTGTTCAGGCACTT

AGGGACAACCTGGAACCTGGGACCTTCGATCTTGGGGGGCTATATGAAGCAATTGAGGAG

TGCCTGATTAACGATCCCTGGGTTTTGCTTAATGCGTCTTGGTTCAACTCCTTCCTCACA

CATGCACTGAAATAG---------------------------------------------

----------------

>A_tundra_swan_Niigata_5112007_2016_EPI1184361

AGCGAAAGCAGGTACTGATCCAAAATGGAGGACTTTGTGCGACAATGCTTCAATCCAATG

ATCGTCGAGCTTGCGGAAAAGACAATGAAAGAATATGGGGAAAGTCCGAAAATAGAGACG

AACAAATTTGCCGCAATATGCACACACCTAGAAGTCTGCTTCATGTATTCAGACTTCCAC

TTTATAGATGAACGAGGCGAATCAATAATTGCAGAGTCTGGCGATCCGAACGCATTATTG

AAACACCGATTTGAGATAATTGAAGGGAGGGACCGAACAATGGCTTGGACAGTGGTAAAT

AGTATCTGCAACACCACAGGGGTTGATAAGCCTAAATTTCTCCCAGATTTGTATGACTAC

AAAGAGAACCGATTCATTGAAATTGGCGTGACACGGAGGGAAGTTCACATATACTACCTA

GAAAAAGCAAATAAGATAAAATCAGAGAGGACACACATTCACATATTCTCATTCACTGGA

GAGGAAATGGCCACCAAAGCTGACTATACTCTTGATGAGGAGAGCAGAGCAAGAATTAAA

ACCCGGCTGTTCACTATAAGACAAGAACTGGCCAGTAGGGGTCTATGGGATTCCTTTCGT

CAATCCGAGAGAGGCGAAGAGACAATTGAAGAAAGGTTTGAAATCACAGGAACCATGCGA

AGGCTCGCTGACCAAAGTCTCCCACCGAACTTCTCCAGCCTTGAAAATTTTAGAGCCTAT

GTGGATGGATTCAAACCGAACAGCTGCATTGAGGGCAAGCTTTCTCAAATGTCAAAGGAA

GTGAACGCCAGAATTGAGCCATTTATGAAGACAACACCACGCCCTCTCAGACTACCTGAT

GGGCCTCCCTGCTCCCAGCGGTCGAAGTTCTTGCTGATGGATGCTCTTAAACTAAGCATT

GAAGATCCGAGCCATGAGGGGGAAGGTATACCACTATATGATGCAATCAAATGCATGAAG

ACATTTTTCGGCTGGAAAGAGCCCAACATCGTAAAACCACATGACAAAGGCATAAATCCC

AATTACCTCCTGGCTTGGAAGCAGGTGCTGGCAGAACTCCAAGACATTGAAAATGAAGAT

AAAATCCCAAAGACAAAGAACATGAAGAAAACAAGCCAATTAAAGTGGGCACTTGGTGAG

AATATGGCACCTGAAAAAGTAGACTTTGAGGACTGCAAAGATGTTAGTGATCTAAAGCAG

TATGACAGTGATGAACCAGAGCCTAGATCGTTATCAAGCTGGGTTCAGAGCGAATTCAAC

AAGGCATGCGAATTGACAGATTCGAGTTGGATTGAACTTGATGAAATAGGAGAAGATGTT

GCTCCAATCGAGCACATTGCAAGTATGAGAAGAAACTACTTCACAGCAGAAGTGTCTCAT

TGCAGGGCTACTGAATACATAATGAAGGGGGTGTATATAAATACAGCCCTATTGAATGCA

TCCTGTGCAGCCATGGATGACTTCCAATTGATTCCAATGATAAGCAAGTGCAGAACCAAA

GAAGGAAGACGGAGGACAAATCTGTATGGATTCATTATAAAAGGAAGGTCCCATTTGAGG

AATGACACCGATGTGGTAAACTTTGTGAGCATGGAATTCTCTCTTACTGACCCGAGGCTG

GAACCACACAAGTGGGTAAAGTACTGTGTTCTTGAAATAGGAGACATGCTCTTACGAACG

GCAATAGGCCAAGTGTCAAGACCCATGTTTTTGTATGTGAGAACCAATGGGACTTCCAAG

ATTAAGATGAAATGGGGCATGGAGATGAGGCGATGCCTTCTCCAATCCCTTCAACAAATT

GAGAGCATGATTGAAGCTGAGTCTTCTGTCAAAGAGAAAGACATGACCAAAGAGTTCTTT

GAGACCAAATCAGAAATGTGGCCAATTGGGGAATCACCTAAGGGGGTGGAAGAAAGCTCC

ATTGGGAAGGTGTGCAGAACACTATTAGCAAAATCTGTCTTCAACAGCCTATATGCATCT

CCACAACTTGAGGGGTTTTCAGCTGAATCAAGAAAATTGCTTCTCATTGTTCAGGCACTT

AGGGACAACCTGGAACCTGGGACTTTCGATCTTGGGGGGCTATATGATGCAATTGAGGAG

TGCCTGATTAATGACCCCTGGGTTTTGCTTAATGCATCTTGGTTCAACTCCTTCCTCACG

CATGCACTGAAATAGTTATGGCAATGCTACTA---TTCGCTATCCATACTGTCC------

----------------

>A_Sichuan_26221_2014_EPI533587

------------------------ATGGAAGACTTTGTGCGACAATGCTTCAATCCAATG

ATCGTCGAGCTTGCGGAAAAGACAATGAAAGAATATGGGGAAAGTCCGAAAATCGAGACG

AACAAATTTGCCGCAATATGCACACACCTAGAAGTCTGCTTCATGTATTCGGACTTCCAC

TTTATAGATGAACGAGGCGAATCAATAATTGCAGAATCTGGCGATCCGAATGCATTATTG

AAACACCGATTTGAGATAATTGAAGGGAGAGACCGAACAATGGCTTGGACAGTGGTAAAT

AGTATCTGCAACACCACAGGGGTCGATAAGCCTAAGTTCCTCCCAGATTTGTATGACTAC

AAAGAGAACCGATTCATTGAAATTGGAGTGACACGGAGGGAAGTTCACATATACTACCTA

GAAAAAGCAAATAAGATAAAATCAGAGAGGACACACATTCACATATTCTCATTCACTGGA

GAGGAAATGGCCACCAAAGCTGACTATACTCTTGATGAGGAGAGCAGAGCAAGAATTAAA

ACCAGGCTGTTCACCATAAGACAAGAAATGGCCAGTAGAGGTCTATGGGATTCCTTTCGT

CAATCCGAGAGAGGCGAAGAGACAATTGAAGAAAGGTTTGAAATCACAGGAACCATGCGC

AGGCTTGCTGACCAAAGTCTCCCACCGAACTTCTCCGGCCTTGAAAATTTTAGAGCCTAT

GTGGATGGATTCGAACCGAACGGCTGCATTGAGGGCAAGCTTTCTCAAATGTCAAAGGAA

GTGAACGCCAGAATTGAGCCATTTATGAAGACAACACCACGCCCTCTCAAACTACCTGAG

GGGCCTCCCTGCTCCCAGCGGTCGAAGTTCTTACTGATGGATGCCCTTAAATTAAGCATC

GAAGACCCGAGCCATGAGGGGGAAGGTATACCACTATATGATGCAATCAAATGCATGAAG

ACATTTTTCGGCTGGAAAGAGCCCAACATCGTAAAACCACATGACAAGGGCATAAATCCC

AATTACCTCCTGGCTTGGAAGCAGGTGCTGGCGGAACTCCAAGACATTGAAAATGAAGAT

AAAATCCCAAAAACAAAGAACATGAAGAAAACAAGCCAATTAAAGTGGGCACTTGGTGAG

AATATGGCACCTGAAAAAGTAGACTTTGAGGACTGCAAAGATGTTAGTGATCTAAAGCAG

TACGACAGTGATGAACCAGAGCCTAGATCATTATCAAGCTGGGTTCAGAGCGAATTCAAC

AAGGCATGCGAATTGACAGATTCGAGTTGGATTGAACTTGATGAAATAGGAGAAGATGCT

GCTCCAATTGAGCACATTGCAAGTATGAGAAGAAACTATTTCACAGCGGAAGTGTCTCAT

TGCAGGGCTACTGAATACATAATGAAGGGGGTGTATATAAATACAGCCCTACTGAATGCA

TCCTGTGCAGCCATGGATGACTTCCAATTGATTCCAATGATAAGCAAGTGCAGAACCAAA

GAAGGAAGACGGAAGACAAATCTGTATGGATTCATTATAAAAGGAAGGTCCCATTTGAGG

AACGACACCGATGTGGTAAACTTTGTGAGCATGGAATTCTCTCTTACTGACCCAAGGCTG

GAACCACACAAGTGGGTAAAGTACTGTGTTCTTGAAATAGGAGAAATGCTCTTACGAACG

GCAATAGGCCAAGTGTCAAGACCCATGTTCTTGTATGTGAGAACCAATGGGACTTCCAAG

ATTAAGATGAAATGGGGCATGGAGATGAGGCGATGCCTTCTTCAATCTCTTCAACAAATT

GAGAGCATGATTGAAGCCGAGTCTTCTGTCAAAGAGAAAGACATGACCAAAGAATTCTTT

GAAACCAAATCAGAAACGTGGCCAATTGGGGAATCACCCAAGGGGGTGGAGGAAAGTTCC

ATTGGAAAGGTGTGCAGAACACTACTAGCAAAATCTGTCTTCAACAGCCTATATGCATCT

CCGCAACTTGAGGGGTTTTCAGCTGAATCAAGAAAATTGCTCCTCATTGTTCAGGCACTT

AGGGACAACCTGGAACCTGGGACCTTCGATCTTGGGGGGCTATATGAAGCAATTGAGGAG

TGCCTGATTAATGATCCTTGGGTTTTGCTTAATGCATCTTGGTTCAACTCCTTCCTCACA

CATGCACTGAAATAG---------------------------------------------

----------------

>A_duck_Sichuan_NCXJ16_2014_EPI590831

------------------------ATGGAAGACTTTGTGCGACAATGCTTCAATCCAATG

ATCGTCGAGCTTGCGGAAAAGACAATGAAAGAATATGGGGAAAGTCCGAAAATCGAGACG

AACAAATTTGCCGCAATATGCACACACCTAGAAGTCTGCTTCATGTATTCGGACTTCCAC

TTTATAGATGAACGAGGCGAATCAATAATTGCAGAATCTGGCGATCCGAATGCATTATTG

AAACACCGATTTGAGATAATTGAAGGGAGAGACCGAACAATGGCTTGGACAGTGGTAAAT

AGTATCTGCAACACCACAGGGGTCGATAAGCCTAAGTTCCTCCCAGATTTGTATGACTAC

AAAGAGAACCGATTCATTGAAATTGGAGTGACACGGAGGGAAGTTCACATATACTACCTA

GAAAAAGCAAATAAGATAAAATCAGAGAGGACACACATTCACATATTCTCATTCACTGGA

GAGGAAATGGCCACCAAAGCTGACTATACTCTTGATGAGGAGAGCAGAGCAAGAATTAAA

ACCAGGCTGTTCACTATAAGACAAGAAATGGCCAGTAGGGGTCTATGGGATTCCTTTCGT

CAATCCGAGAGAGGCGAAGAGACAATTGAAGAAAGGTTTGAAATCACAGGAACCATGCGC

AGGCTTGCTGACCAAAGTCTCCCACCGAACTTCTCCAGCCTTGAAAATTTTAGAGCCTAT

GTGGATGGATTCGAACCGAACGGCTGCATTGAGGGCAAGCTTTCTCAAATGTCAAAGGAA

GTGAACGCCAGAATTGAGCCATTTATGAAGACAACACCACGCCCTCTCAAACTACCTGAG

GGGCCTCCCTGCTCCCAGCGGTCGAAGTTCTTACTGATGGATGCCCTTAAATTAAGCATC

GAAGACCCGAGCCATGAGGGGGAAGGTATACCACTATATGATGCAATCAAATGCATGAAG

ACATTTTTCGGCTGGAAAGAGCCCAACATCGTAAAACCACATGACAAGGGCATAAATCCC

AATTACCTCCTGGCTTGGAAGCAGGTGCTGGCGGAACTCCAAGACATTGAAAATGAAGAT

AAAATCCCAAAAACAAAGAACATGAAGAAAACAAGCCAATTAAAGTGGGCACTTGGTGAG

AATATGGCACCTGAAAAAGTAGACTTTGAGGACTGCAAAGATGTTAGTGATCTAAAGCAG

TACGACAGTGATGAACCAGAGCCTAGATCATTATCAAGCTGGGTTCAGAGCGAATTCAAC

AAGGCATGCGAATTGACAGATTCGAGTTGGATTGAACTTGATGAAATAGGAGAAGATGCT

GCTCCAATTGAGCACATTGCAAGTATGAGAAGAAACTATTTCACAGCGGAAGTGTCTCAT

TGCAGGGCTACTGAATACATAATGAAGGGGGTGTATATAAATACAGCCCTACTGAATGCA

TCCTGTGCAGCCATGGATGACTTCCAATTGATTCCAATGATAAGCAAGTGCAGAACCAAA

GAAGGAAGACGGAAGACAAATCTGTATGGATTCATTATAAAAGGAAGGTCCCATTTGAGG

AACGACACCGATGTGGTAAACTTTGTGAGCATGGAATTCTCTCTTACTGACCCAAGGCTG

GAACCACACAAGTGGGTAAAGTACTGTGTTCTTGAAATAGGAGAAATGCTCTTACGAACG

GCAATAGGCCAAGTGTCAAGACCCATGTTCTTGTATGTGAGAACCAATGGGACTTCCAAG

ATTAAGATGAAATGGGGCATGGAGATGAGGCGATGCCTTCTTCAATCTCTTCAACAAATT

GAGAGCATGATTGAAGCCGAGTCTTCTGTCAAAGAGAAAGACATGACCAAAGAATTCTTT

GAAACCAAATCAGAAACGTGGCCAATTGGGGAATCACCCAAGGGGGTGGAGGAAAGTTCC

ATTGGAAAGGTGTGCAGAACACTACTAGCAAAATCTGTCTTCAACAGCCTATATGCATCT

CCGCAACTTGAGGGGTTTTCAGCTGAATCAAGAAAATTGCTCCTCATTGTTCAGGCACTT

AGGGACAACCTGGAACCTGGGACCTTCGATCTCGGGGGGCTATATGAAGCAATTGAGGAG

TGCCTGATTAATGATCCTTGGGTTTTGCTTAATGCATCTTGGTTCAACTCCTTCCTCACA

CATGCACTGAAATAG---------------------------------------------

----------------

>A_Fujian-Sanyuan_21099_2017_x_PR8_CNIC_1369972

----AAAGCAGGTACTGATCCAAAATGGAAGATTTTGTGCGACAATGCTTCAATCCGATG

ATTGTCGAGCTTGCGGAAAAAACAATGAAAGAGTATGGGGAGGACCTGAAAATCGAAACA

AACAAATTTGCAGCAATATGCACTCACTTGGAAGTATGCTTCATGTATTCAGATTTTCAC

TTCATCAATGAGCAAGGCGAGTCAATAATCGTAGAACTTGGTGATCCAAATGCACTTTTG

AAGCACAGATTTGAAATAATCGAGGGAAGAGATCGCACAATGGCCTGGACAGTAGTAAAC

AGTATTTGCAACACTACAGGGGCTGAGAAACCAAAGTTTCTACCAGATTTGTATGATTAC

AAGGAGAATAGATTCATCGAAATTGGAGTAACAAGGAGAGAAGTTCACATATACTATCTG

GAAAAGGCCAATAAAATTAAATCTGAGAAAACACACATCCACATTTTCTCGTTCACTGGG

GAAGAAATGGCCACAAAGGCAGACTACACTCTCGATGAAGAAAGCAGGGCTAGGATCAAA

ACCAGACTATTCACCATAAGACAAGAAATGGCCAGCAGAGGCCTCTGGGATTCCTTTCGT

CAGTCCGAGAGAGGAGAAGAGACAATTGAAGAAAGGTTTGAAATCACAGGAACAATGCGT

AAGCTTGCCGACCAAAGTCTCCCGCCGAACTTCTCCAGCCTTGAAAATTTTAGAGCCTAT

GTGGATGGATTCGAACCGAACGGCTACATTGAGGGCAAGCTGTCTCAAATGTCCAAAGAA

GTAAATGCTAGAATTGAACCTTTTTTGAAAACAACACCACGACCACTTAGACTTCCGAAT

GGGCCTCCCTGTTCTCAGCGGTCCAAATTCCTGCTGATGGATGCCTTAAAATTAAGCATT

GAGGACCCAAGTCATGAAGGAGAGGGAATACCGCTATATGATGCAATCAAATGCATGAGA

ACATTCTTTGGATGGAAGGAACCCAATGTTGTTAAACCACACGAAAAGGGAATAAATCCA

AATTATCTTCTGTCATGGAAGCAAGTACTGGCAGAACTGCAGGACATTGAGAATGAGGAG

AAAATTCCAAAGACTAAAAATATGAAGAAAACAAGTCAGCTAAAGTGGGCACTTGGTGAG

AACATGGCACCAGAAAAGGTAGACTTTGACGACTGTAAAGATGTAGGTGATTTGAAGCAA

TATGATAGTGATGAACCAGAATTGAGGTCGCTTGCAAGTTGGATTCAGAATGAGTTTAAC

AAGGCATGCGAACTGACAGATTCAAGCTGGATAGAGCTCGATGAGATTGGAGAAGATGTG

GCTCCAATTGAACACATTGCAAGCATGAGAAGGAATTATTTCACATCAGAGGTGTCTCAC

TGCAGAGCCACAGAATACATAATGAAGGGAGTGTACATCAATACTGCCTTGCTTAATGCA

TCTTGTGCAGCAATGGATGATTTCCAATTAATTCCAATGATAAGCAAGTGTAGAACTAAG

GAGGGAAGGCGAAAGACCAACTTGTATGGTTTCATCATAAAAGGAAGATCCCACTTAAGG

AATGACACCGACGTGGTAAACTTTGTGAGCATGGAGTTTTCTCTCACTGACCCAAGACTT

GAACCACATAAATGGGAGAAGTACTGTGTTCTTGAGATAGGAGATATGCTTATAAGAAGT

GCCATAGGCCAGGTTTCAAGGCCCATGTTCTTGTATGTGAGAACAAATGGAACCTCAAAA

ATTAAAATGAAATGGGGAATGGAGATGAGGCGTTGCCTCCTCCAGTCACTTCAACAAATT

GAGAGTATGATTGAAGCTGAGTCCTCTGTCAAAGAGAAAGACATGACCAAAGAGTTCTTT

GAGAACAAATCAGAAACATGGCCCATTGGAGAGTCCCCCAAAGGAGTGGAGGAAAGTTCC

ATTGGGAAGGTCTGCAGGACTTTATTAGCAAAGTCGGTATTCAACAGCTTGTATGCATCT

CCACAACTAGAAGGATTTTCAGCTGAATCAAGAAAACTGCTTCTTATCGTTCAGGCTCTT

AGGGACAACCTGGAACCTGGGACCTTTGATCTTGGGGGGCTATATGAAGCAATTGAGGAG

TGCCTGATTAATGATCCCTGGGTTTTGCTTAATGCTTCTTGGTTCAACTCCTTCCTTACA

CATGCATTGAGTTAGTTGTGGCAGTGCTACTA---TTTGCTATCCATACTGTCCAAAAAA

GTACCTTGTTTCTACT

>A_Perigrine_falcon_Netherlands_18003274_1327124

------------TACTGATCCAAAATGGAAGACTTTGTGCGACAATGCTTCAATCCAATG

ATTGTCGAGCTTGCGGAAAAGGCAATGAAAGAATATGGGGAAGATCCGAAAATCGAAACA

AACAAATTTGCCGCAATATGCACGCACTTAGAAGTCTGTTTCATGTATTCAGACTTCCAC

TTCATTGATGAACGAGGCGAATCAATAATTGTAGAATCTGGCGATCCGAACGCATTATTG

AAACACCGATTTGAAATAATTGAAGGGAGAGACCGAACAATGGCCTGGACGGTGGTGAAT

AGTATCTGCAACACCACAGGAGTCGAAAAGCCCAAATTCCTCCCTGATTTGTATGACTAC

AAAGAGAACCGATTCATTGAAATTGGAATAACGCGAAGGGAAGTTCACATATACTATCTA

GAAAAAGCCAACAAGATAAAATCAGAAAAGACACACATTCACATATTCTCATTCATTGGA

GAGGAAATGGCCACCAGGGCGGACTACACCCTTGATGAAGAGAGCAGGGCAAGAATAAAA

ACCAGGCTGTTCACTATAAGACAAGAAATGGCCAGCAGGGGTCTATGGGATTCCTTTCGT

CAGTCCGAGAGAGGCGAAGAGACAATTGAAGAAAGATTTGAAATCACAGGAACCGTGCGT

AGGCTTGCCGACCAAAGTCTCCCACCGAACTTCTCCAGCCTTGAAAACTTTAGAGCCTAT

GTGGATGGATTCGAACCGAACGGCTGCATTGAGGGCAAGCTTTCTCAAATGTCAAAAGAA

GTGAATGCCAGAATTGAGCCATTTCTGAAGACAACACCACGCCCTCTCAGATTACCTGAT

GGACCTCCCTGTTCTCAGCGGTCGAAGTTCTTGCTGATGGATGCCCTTAAATTGAGCATC

GAGGACCCAAGCCATGAGGGGGAGGGTATACCACTGTATGATGCAATCAAATGCATGAAG

ACATTTTTTGGCTGGAAAGAGCCCAACATTGTAAAACCACATGAAAAAGGCATAAACCCT

AATTACCTCCTGGCTTGGAAGCAGGTGCTGGCAGAACTCCAAGATATTGAAAATGAGGAG

AAAATTCCAAAAACAAAGAACATGAAGAAAACAAGCCAATTGAAGTGGGCACTTGGTGAG

AACATGGCACCAGAGAAAGTGGACTTTGAGGACTGTAAAGATGTTAGCGATCTAAGACAG

TACGACAGTGACGAACCAGAGTCCAGATCACTAGCAAGCTGGATCCAGAGTGAATTCAAC

AAGGCATGCGAATTGACAGATTCGAGTTGGATTGAACTTGATGAAATAGGGGAGGACGTT

GCTCCAATTGAACACATTGCGAGTATGAGGAGGAACTATTTCACAGCGGAGGTATCCCAT

TGCAGGGCTACTGAATACATAATGAAGGGAGTATACATAAACACAGCACTATTGAATGCG

TCCTGTGCAGCCATGGATGACTTCCAACTGATTCCAATGATAAGCAAATGCAGAACCAAA

GAAGGAAGAAGGAAGACAAATCTGTATGGATTCATTATAAAGGGAAGATCCCATTTGAGG

AATGACACCGATGTGGTAAATTTCGTGAGCATGGAATTCTCTCTCACTGACCCGAGGCTG

GAGCCACACAAATGGGAAAAGTACTGTGTTCTCGAAATAGGAGACATGCTCCTACGGACT

GCAATAGGCCAAGTGTCAAGGCCCATGTTCCTGTATGTGAGAACCAATGGGACTTCCAAG

ATCAAAATGAAATGGGGCATGGAGATGAGGCGATGCCTTCTTCAGTCCCTTCAACAAATT

GAGAGCATGATCGAGGCCGAGTCTTCTGTCAAAGAGAAGGACATGACCAAGGAATTCTTT

GAAAACAAATCAGAAACATGGCCAATTGGGGAATCACCCAAAGGGGTGGAGGAAGGCTCC

ATTGGGAAGGTGTGCAGAACATTGCTAGCAAAGTCTGTGTTCAACAGCCTATATGCATCT

CCACAACTCGAGGGGTTTTCAGCTGAATCAAGAAAATTGCTTCTCATTGTTCAGGCGCTT

AGGGACAATCTGGAACCTGGGACCTTCGATCTTGGGGGGCTATATGAAGCAATTGAGGAG

TGCCTGATTAACGATCCCTGGGTTTTGCTTAATGCGTCTTGGTTCAACTCCTTCCTCACA

CATGCACTGAAATAGTTGTGGCAATGCTACTA---TTTGCTATCCATACTGTCCAAAAAA

GTA-------------

>A_chicken_Washington_3490-18_2015_EPI590470

------------------------ATGGAAGACTTTGTGCGACAATGCTTCAATCCAATG

ATCGTCGAGCTTGCGGAAAAGACAATGAAAGAATATGGGGAGAATCCAAAAATCGAAACG

AACAAATTCGCTGCAATATGCACTCACTTAGAGGTCTGTTTCATGTATTCGGATTTCCAC

TTTATTGATGAACGAGGCAAATCAATAATTGTAGAATCTGGCGATCCGAATGCATTATTG

AAACACCGATTTGAGATAATTGAAGGGAGAGACCGAACGATGGCTTGGACAGTGGTAAAT

AGTATCTGCAACACCACAGGAGTCGATAAGCCTAAATTCCTCCCAGATTTGTATGATTAC

AAGGAGAACCGATTCATTGAAATTGGAGTGACAAGGAGGGAAGTTCACACATACTACCTA

GAAAAGGCAAATAAGATAAAATCAGAGAAGACACACATTCACATATTCTCATTCACTGGG

GAGGAGATGGCCACCAAGGCTGACTATACCCTTGATGAAGAGAGCAGAGCAAGGATCAAA

ACCAGGTTGTTCACTATCAGGCAAGAAATGGCCAATAGGGGTCTGTGGGATTCCTTTCGT

CAATCTGAGAGAGGCGAAGAGACAATTGAAGAAAGGTTTGAAATCACAGGAACCATGCGC

AGGCTTGCAGACCAAAGCCTCCCACCGAATTTCTCCAGCCTTGAAAATTTTAGAGCCTAT

GTGGATGGATTCAAACCGAACGGATGCCTTGAGGGCAAGCTTTCTCAAATGTCAAAAGAA

GTGAACGCCAGAATTGAGCCATTCATGAAGACAACACCACGCCCTCTCAGATTACCTGAT

GGTCCTCCTTGCTCTCAGCGATCGAAATTCTTACTGATGGATTCCCTTAAATTGAGCATC

GAAGACCCAAGCCATGAAGGAGAAGGTATACCGCTATATGATGCAATCAAATGCATGAAG

ACGTTTTTTGGTTGGAAAGAGCCCAACATTGTAAAACCACATGTAAAAGGCATAAATCCC

AACTATCTCTTGGCTTGGAAGCAGGTGCTGGCAGAACTCCAAGACATTGAAAATGAAGAG

AAAATCCCAAAAACAAAAAACATGAAGAAAACAAGCCAACTAAAGTGGGCACTCGGTGAG

AATATGGCACCTGAAAAAGTGGACTTTGAGGACTGCAGAGATGTTAGCGATCTAAGACAG

TATGACAGTGATGAACCAGAGCCCAGATCATTATCAAGCTGGATCCAGAGCGAATTCAAC

AAAGCATGCGAATTGACAGATTCGAGTTGGATTGAACTTGATGAAATAGGAGAAGATGTT

GCTCCAATTGAGCACATTGCGAGTATGAGAAGAAACTACTTCACAGCGGAAGTGTCTCAT

TGCAGGGCTACTGAATATATAATGAAAGGAGTTTATATAAATACAGCCCTGTTGAATTCA

TCCTGCGCAGCCATGGATGACTTCCAATTGATTCCAATGATAAGCAAGTGCAGAACCAAA

GAAGGAAGACGGAAGACAAATTTATATGGGTTCATTATAAAAGGAAGATCCCATTTGAGG

AATGATACCGATGTGGTAAATTTTGTGAGCATGGAGTTCTCCCTCACTGACCCGAGGCTG

GAACCACACAAGTGGGAAAAGTACTGTGTTCTCGAAATAGGAGACATGCTCCTACGAACT

GCAATAGGCCAAGTATCAAGATCCATGTTTCTTTATGTAAGAACCAATGGGACTTCCAAG

ATCAAGATGAAATGGGGCATGGAGATGAGGCGATGCCTTCTTCAATCCCTCCAACAAATT

GAGAGCATGATTGAGGCAGAGTCTTCTGTCAAAGAGAAAGACATGACCAAGGAATTCTTT

GAAAATAAATCAGAAACGTGGCCAATTGGGGAATCACCTAAGGGGGTGGAGGAAAGCTCT

ATTGGGAAGGTGTGTAGAACATTACTAGCAAAATCTGTATTCAACAGCCTATATGCATCT

CCACAACTTGAGGGGTTTTCAGCTGAGTCGAGAAAGTTACTTCTCATTGTTCAGGCATTT

AGGGACAACCTGGAACCTGGGACCTTCGATCTTGGGGGGCTATATGAAGCAATTGAGGAG

TGCCTGATTAATGATCCCTGGGTTTTGCTTAATGCATCTTGGTTCAACTCCTTCCTTACA

CATGCACTGAAATAG---------------------------------------------

----------------

>A_gyrfalcon_Washington_41088-6_2014_EPI569389

------------------------ATGGAAGACTTTGTGCGACAATGCTTCAATCCAATG

ATCGTCGAGCTTGCGGAAAAGACAATGAAAGAATATGGGGAAAATCCAAAAATCGAAACG

AACAAATTCGCTGCAATATGCACTCACTTAGAGGTCTGTTTCATGTATTCGGATTTCCAC

TTTATTGATGAACGAGGCAAATCAATAATTGTAGAATCTGGCGATCCGAATGCATTATTG

AAACACCGATTTGAGATAATTGAAGGGAGAGACCGAACGATGGCTTGGACAGTGGTAAAT

AGTATCTGCAACACCACAGGAGTCGATAAGCCTAAATTCCTCCCAGATTTGTATGATTAC

AAGGAGAACCGATTCATTGAAATTGGAGTGACAAGGAGGGAAGTTCACACATACTACCTA

GAAAAGGCAAATAAGATAAAATCAGAGAAGACACACATTCACATATTCTCATTCACTGGG

GAGGAGATGGCCACCAAAGCTGACTATATCCTTGATGAAGAGAGCAGAGCAAGGATCAAA

ACCAGGTTGTTCACTATCAGGCAAGAAATGGCCAATAGGGGTCTGTGGGATTCCTTTCGT

CAATCTGAGAGAGGCGAAGAGACAATTGAAGAAAGGTTTGAAATCACAGGAACCATGCGC

AGGCTTGCAGACCAAAGCCTCCCACCGAATTTCTCCAGCCTTGAAAATTTTAGAGCCTAT

GTGGATGGATTCAAACCGAACGGATGCCTTGAGGGCAAGCTTTCTCAAATGTCAAAAGAA

GTGAACGCCAGAATTGAGCCATTCATGAAGACAACACCACGCCCTCTCAGATTACCTGAT

GGTCCTCCTTGCTCTCAGCGATCAAAATTCTTACTGATGGATTCCCTTAAATTGAGCATC

GAAGACCCAAGCCATGAGGGAGAAGGTATACCGCTATATGATGCAATCAAATGCATGAAG

ACGTTTTTTGGTTGGAAAGAGCCCAACATTGTAAAACCACATGTAAAAGGCATAAATCCC

AACTATCTCTTGGCTTGGAAGCAGGTGCTGGCAGAACTCCAAGACATTGAAAATGAAGAG

AAAATCCCAAAAACAAAAAACATGAAGAAAACAAGCCAACTAAAGTGGGCACTCGGTGAG

AATATGGCACCTGAAAAAGTGGACTTTGAGGACTGCAGAGATGTTAGCGATCTAAGACAG

TATGACAGTGATGAACCAGAGCCCAGATCATTATCAAGCTGGATCCAGAGCGAATTCAAC

AAAGCATGCGAATTGACAGATTCGAGTTGGATTGAACTTGATGAAATAGGAGAAGATGTT

GCTCCAATTGAGCACATTGCGAGTATGAGAAGAAACTACTTCACAGCGGAAGTGTCTCAT

TGCAGGGCTACTGAATATATAATGAAAGGAGTTTATATAAATACAGCCCTGTTGAATTCA

TCCTGTGCAGCCATGGATGACTTCCAATTGATTCCAATGATAAGCAAGTGCAGAACCAAA

GAAGGAAGACGGAAGACAAATTTATATGGGTTCATTATAAAAGGAAGATCCCATTTGAGG

AATGATACCGATGTGGTAAATTTTGTGAGCATGGAGTTCTCCCTCACTGACCCGAGGCTG

GAACCACACAAATGGGAAAAGTACTGTGTTCTCGAAATAGGAGACATGCTCCTACGAACT

GCAATAGGCCAAGTATCAAGATCCATGTTTCTTTATGTAAGAACCAATGGGACTTCCAAG

ATCAAGATGAAATGGGGCATGGAGATGAGGCGATGCCTTCTTCAATCCCTCCAACAAATT

GAGAGCATGATTGAGGCAGAGTCTTCTGTCAAAGAGAAAGACATGACCAAGGAATTCTTT

GAAAATAAATCAGAAACGTGGCCAATTGGGGAATCACCTAAGGGGGTGGAGGAAAGCTCT

ATTGGGAAGGTGTGTAGAACATTACTAGCAAAATCTGTATTCAACAGCCTATATGCATCT

CCACAACTTGAGGGGTTTTCAGCTGAGTCGAGAAAGTTACTTCTTATTGTTCAGGCATTT

AGGGACAACCTGGAACCTGGGACCTTCGATCTTGGGGGGCTATATGAAGCAATTGAGGAG

TGCCTGATTAATGATCCCTGGGTTTTGCTTAATGCATCTTGGTTCAACTCCTTCCTTACA

CATGCACTGAAATAGTTGTGGCAATGCTACTA------TTTGCTATCCATAC--------

----------------

>A_chicken_Hubei_ZYSJF38_2016_EPI895190

------------------------ATGGAAGACTTTGTGCGACAGTGCTTCAATCCAATG

ATCGTCGAGCTTGCGGAAAAGGCAATGAAAGAATATGGGGAAGATCCGAAAATCGAAACA

AACAAATTTGCATCAATATGCACACACTTAGAAGTCTGCTTCATGTACTCTGATTTCCAC

TTCATCGACGAACGAGGCGAATCAACTATAATAGAATCTGGCGATCCAAATGTGCTGCTG

AAACACCGATTTGAAATAATCGAAGGGAGAGACCGAACAATGGCCTGGACAGTGGTGAAT

AGTATCTGCAACACCACAGGAGCCGAAAAACCCAAATTTCTCCCGGATTTGTATGACTAC

AAGGAAAACCGTTTCATTGAAATTGGAGTGACGAGGAGGGAAGTCCACATATATTACCTA

GAGAAAGCCAATAAAATAAAATCCGAGAAGACACACATCCATATTTTTTCATTCACTGGA

GAAGAGATGGCCACCAAAGCAGATTACACTCTTGACGAAGAAAGCAGGGCAAGAATCAAG

ACCAGGCTGTTCATCATAAGGCAGGAAATGGCCAGCAGGGGTCTATGGGATTCCTTTCGT

CAGTCCGAAAGAGGCGAAGAAACAATTGAAGAAAGATTTGAAATCACAGGAACCATGCGC

AGGCTTGCCGACCAAAGTCTCCCACCGAACTTCTCCAGCCTTGAAAACTTTAGAGCCTAT

GTGGATGGATTCGAACCGAACGGCTGCATTGAGGGCAAGCTTTCTCAGATGTCAAAAGAA

GTGAACGCCAGAATTGAACCATTTCTAAGAACAACACCACGCCCTCTCAGATTGCCTAAT

GGGCCTCCCTGCTCTCAGCGGTCGAAATTCTTGCTGATGGATGCTCTGAAATTAAGCATT

GAGGACCCGAGCCACGAGGGGGAGGGGATACCGCTATATGATGCGATCAAATGCATGAAA

ACGTTCTTCGGGTGGAAAGAGCCCAACATTGTCAAACCACATGAGAAAGGCATAAACCCC

AATTATCTCCTGGCTTGGAAGCAGGTGCTAGCAGAACTTCAGGACATTGAAAATGAAGAG

AAGATTCCAAGGACAAAGAACATGAAGAAGACAAGCCAATTAAAGTGGGCACTCGGTGAG

AACATGGCACCGGAGAAGGTGGACTTTGAGGATTGCAAAGATGTCAACGACTTGAAACAG

TACAACAGTGATGAGCCAGAGCCCAAATCACTAGCATGTTGGATCCAGAATGAATTCAAC

AAGGCGTGTGAACTGACTGACTCAAGCTGGATGGAACTTGATGAAATAGGGGAAGATGTT

GCCCCAATCGAACACATTGCAAGCATGAGACGGAACTATTTTACAGCAGAGGTGTCCCAC

TGCAGGGCTACTGAATATATAATGAAGGGAGTGTACATAAACACAGCTTTGCTCAATGCA

TCTTGCGCAGCCATGGATGACTTTCAACTGATTCCAATGATAAGTAAGTGTAGAACTAAA

GAAGGAAGACGGAAAACAAACCTGTATGGATTCATTATAAAAGGAAGATCTCATTTGAGA

AATGACACCGACGTGGTAAACTTTGTAAGTATGGAATTTTCCCTTACCGACCCAAGGTTG

GAACCACATAAATGGGAAAAGTATTGTGTTCTTGAAATAGGGGACATGCTCCTGCGAACT

GCAGTAGGCCAAGTATCAAGACCCATGTTTCTGTATGTGAGAACCAATGGGACCTCCAAG

ATCAAGATGAAATGGGGTATGGAAATGAGACGCTGCCTCCTTCAATCTCTCCAACAGATT

GAGAGCATGATTGAAGCTGAATCCTCCGTCAAAGAGAAAGACCTGACCAAAGAATTCTTT

GAAAACAAATCAGAAACATGGCCAATTGGAGAGTCACCTAGAGGAGTGGAGGAAGGTTCC

ATCGGGAAGGTGTGCAGAACTTTACTAGCAAAATCTGTATTCAACAGCCTATACGCATCT

CCGCAACTCGAGGGGTTCTCAGCTGAATCGAGAAAACTGCTACTCATTGTTCAGGCACTT

AGGGATAACCTGGAACCTGGAACCTTTGATCTTGAGGGGCTATATGAAGCAATCGAGGAG

TGCCTGATTAATGATCCCTGGGTTTTGCTTAATGCATCTTGGTTCAACTCCTTCCTCACA

CATGCACTAAGATAGTTGTGGCAATGCTACTACTATTTGCTATCCATACTGTCCAAAAA-

----------------

>A_Hubei_29578_2016_x_PR8_CNIC-HB29578_1369964

-----AAGCAGGTACTGATCCAAAATGGAAGATTTTGTGCGACAATGCTTCAATCCGATG

ATTGTCGAGCTTGCGGAAAAAACAATGAAAGAGTATGGGGAGGACCTGAAAATCGAAACA

AACAAATTTGCAGCAATATGCACTCACTTGGAAGTATGCTTCATGTATTCAGATTTTCAC

TTCATCAATGAGCAAGGCGAGTCAATAATCGTAGAACTTGGTGATCCAAATGCACTTTTG

AAGCACAGATTTGAAATAATCGAGGGAAGAGATCGCACAATGGCCTGGACAGTAGTAAAC

AGTATTTGCAACACTACAGGGGCTGAGAAACCAAAGTTTCTACCAGATTTGTATGATTAC

AAGGAGAATAGATTCATCGAAATTGGAGTAACAAGGAGAGAAGTTCACATATACTATCTG

GAAAAGGCCAATAAAATTAAATCTGAGAAAACACACATCCACATTTTCTCGTTCACTGGG

GAAGAAATGGCCACAAAGGCAGACTACACTCTCGATGAAGAAAGCAGGGCTAGGATCAAA

ACCAGACTATTCACCATAAGACAAGAAATGGCCAGCAGAGGCCTCTGGGATTCCTTTCGT

CAGTCCGAGAGAGGAGAAGAGACAATTGAAGAAAGGTTTGAAATCACAGGAACAATGCGT

AAGCTTGCCGACCAAAGTCTCCCGCCGAACTTCTCCAGCCTTGAAAATTTTAGAGCCTAT

GTGGATGGATTCGAACCGAACGGCTACATTGAGGGCAAGCTGTCTCAAATGTCCAAAGAA

GTAAATGCTAGAATTGAACCTTTTTTGAAAACAACACCACGACCACTTAGACTTCCGAAT

GGGCCTCCCTGTTCTCAGCGGTCCAAATTCCTGCTGATGGATGCCTTAAAATTAAGCATT

GAGGACCCAAGTCATGAAGGAGAGGGAATACCGCTATATGATGCAATCAAATGCATGAGA

ACATTCTTTGGATGGAAGGAACCCAATGTTGTTAAACCACACGAAAAGGGAATAAATCCA

AATTATCTTCTGTCATGGAAGCAAGTACTGGCAGAACTGCAGGACATTGAGAATGAGGAG

AAAATTCCAAAGACTAAAAATATGAAGAAAACAAGTCAGCTAAAGTGGGCACTTGGTGAG

AACATGGCACCAGAAAAGGTAGACTTTGACGACTGTAAAGATGTAGGTGATTTGAAGCAA

TATGATAGTGATGAACCAGAATTGAGGTCGCTTGCAAGTTGGATTCAGAATGAGTTTAAC

AAGGCATGCGAACTGACAGATTCAAGCTGGATAGAGCTCGATGAGATTGGAGAAGATGTG

GCTCCAATTGAACACATTGCAAGCATGAGAAGGAATTATTTCACATCAGAGGTGTCTCAC

TGCAGAGCCACAGAATACATAATGAAGGGAGTGTACATCAATACTGCCTTGCTTAATGCA

TCTTGTGCAGCAATGGATGATTTCCAATTAATTCCAATGATAAGCAAGTGTAGAACTAAG

GAGGGAAGGCGAAAGACCAACTTGTATGGTTTCATCATAAAAGGAAGATCCCACTTAAGG

AATGACACCGACGTGGTAAACTTTGTGAGCATGGAGTTTTCTCTCACTGACCCAAGACTT

GAACCACATAAATGGGAGAAGTACTGTGTTCTTGAGATAGGAGATATGCTTATAAGAAGT

GCCATAGGCCAGGTTTCAAGGCCCATGTTCTTGTATGTGAGAACAAATGGAACCTCAAAA

ATTAAAATGAAATGGGGAATGGAGATGAGGCGTTGCCTCCTCCAGTCACTTCAACAAATT

GAGAGTATGATTGAAGCTGAGTCCTCTGTCAAAGAGAAAGACATGACCAAAGAGTTCTTT

GAGAACAAATCAGAAACATGGCCCATTGGAGAATCCCCCAAAGGAGTGGAGGAAAGTTCC

ATTGGGAAGGTCTGCAGGACTTTATTAGCAAAGTCGGTATTCAACAGCTTGTATGCATCT

CCACAACTAGAAGGATTTTCAGCTGAATCAAGAAAACTGCTTCTTATCGTTCAGGCTCTT

AGGGACAACCTGGAACCTGGGACCTTTGATCTTGGGGGGCTATATGAAGCAATTGAGGAG

TGCCTGATTAATGATCCCTGGGTTTTGCTTAATGCTTCTTGGTTCAACTCCTTCCTTACA

CATGCATTGAGTTAGTTGTGGCAGTGCTACTA---TTTGCTATCCATACTGTCCAAAAAA

GTA-------------

>A_duck_Hyogo_1_2016_EPI866707

------------------------ATGGAGGACTTTGTGCGACAGTGCTTCAATCCAATG

ATCGTCGAGCTTGCGGAAAAGACAATGAAAGAATATGGGGAAAGTCCGAAAATCGAGACG

AACAAATTTGCCGCAATATGCACACACCTAGAAGTCTGCTTCATGTATTCAGACTTCCAC

TTTATAGATGAACGAGGCGAATCAATAATTGCAGAGTCTGGCGATCCGAACGCATTATTG

AAACACCGATTTGAGATAATTGAAGGGAGGGACCGAACAATGGCTTGGACAGTGGTAAAT

AGTATCTGCAACACCACAGGGGTCGATAAGCCTAAATTTCTCCCAGATTTGTATGACTAC

AAAGAGAACCGATTCATTGAAATTGGCGTGACACGGAGGGAAGTTCACATATACTACCTA

GAAAAAGCAAATAAGATAAAATCAGAGAGGACACACATTCACATATTCTCATTCACTGGA

GAGGAAATGGCCACCAAAGCTGACTATACTCTTGATGAGGAGAGCAGAGCAAGAATTAAA

ACCCGGCTGTTCACTATAAGACAAGAACTGGCCAGTAGGGGTCTATGGGATTCCTTTCGT

CAATCCGAGAGAGGCGAAGAGACAATTGAAGAAAGGTTTGAAATCACAGGAACCATGCGA

AGGCTCGCTGACCAAAGTCTCCCACCGAACTTCTCCAGCCTTGAAAATTTTAGAGCCTAT

GTGGATGGATTCAAACCGAACAGCTGCATTGAGGGCAAGCTTTCTCAAATGTCAAAGGAA

GTGAACGCCAGAATTGAGCCATTTATGAAGACAACACCACGCCCTCTCAGACTACCTGAT

GGGCCTCCCTGCTCCCAGCGGTCGAAGTTCTTGCTGATGGATGCTCTTAAACTAAGCATT

GAAGATCCGAGCCATGAGGGGGAAGGTATACCACTATATGATGCAATCAAATGCATGAAG

ACATTTTTCGGCTGGAAAGAGCCCAACATCGTAAAACCACATGACAAAGGCATAAATCCC

AATTACCTCCTGGCTTGGAAGCAGGTGCTGGCAGAACTCCAAGACATTGAAAATGAAGAT

AAAATCCCAAAGACAAAGAACATGAAGAAAACAAGCCAATTAAAGTGGGCACTTGGTGAG

AATATGGCACCTGAAAAAGTAGACTTTGAGGACTGCAAAGATGTTAGTGATCTAAAGCAG

TATGACAGTGATGAACCAGAGCCTAGATCGTTATCAAGCTGGGTTCAGAGCGAATTCAAC

AAGGCATGCGAATTGACAGATTCGAGTTGGATTGAACTTGATGAAATAGGAGAAGATGTT

GCTCCAATCGAGCACATTGCAAGTATGAGAAGAAACTACTTCACAGCAGAAGTGTCTCAT

TGCAGGGCTACTGAATACATAATGAAGGGAGTGTATATAAATACAGCCCTATTGAATGCA

TCCTGTGCAGCCATGGATGACTTCCAATTGATTCCAATGATAAGCAAGTGCAGAACCAAA

GAAGGAAGACGGAAGACAAATCTGTATGGATTCATTATAAAAGGAAGGTCCCATTTGAGG

AATGACACCGATGTGGTAAACTTTGTGAGCATGGAATTCTCTCTTACTGACCCGAGGCTG

GAACCACACAAGTGGGTAAAGTACTGTGTTCTTGAAATAGGAGACATGCTCTTACGAACG

GCAATAGGCCAAGTGTCAAGACCCATGTTTTTGTATGTGAGAACCAATGGGACTTCCAAG

ATTAAGATGAAATGGGGCATGGAGATGAGGCGATGCCTTCTTCAATCCCTTCAACAAATT

GAGAGCATGATTGAAGCCGAGTCTTCTGTCAAAGAGAAAGACATGACCAAAGAGTTCTTT

GAGACCAAATCAGAAATGTGGCCAATTGGGGAATCACCTAAGGGGGTGGAAGAAAGCTCC

ATTGGGAAGGTGTGCAGAACACTATTAGCAAAATCTGTCTTCAACAGCCTATATGCATCT

CCACAACTTGAGGGGTTTTCAGCTGAATCAAGAAAATTGCTTCTCATTGTTCAGGCACTT

AGGGACAACCTGGAACCTGGGACTTTCGATCTTGGGGGGCTATATGATGCAATTGAGGAG

TGCCTGATTAATGACCCCTGGGTTTTGCTTAATGCATCTTGGTTCAACTCCTTCCTCACG

CATGCACTGAAATAG---------------------------------------------

----------------

>A_chicken_Vietnam_NCVD-15A55_2015_EPI895045

------------TACTGATCCAAAATGGAGGACTTTGTGCGACAATGCTTCAATCCAATG

ATCGTCGAGCTTGCGGAAAAGACAATGAAAGAATATGGGGAAAGTCCGAAAATCGAGACG

AACAAATTTGCCGCAATATGCACACACCTAGAAGTCTGCTTCATGTATTCGGACTTCCAC

TTTATAGATGAACGAGGCGAATCAATAATTGCAGAGTCTGGCGATCCGAATGCATTATTG

AAACACCGATTTGAGATAATTGAAGGGAGGGACCGAACGATGGCTTGGACAGTGGTAAAT

AGTATCTGCAACACCACAGGGGTCGATAAGCCCAAGTTCCTCCCAGATTTGTATGACTAC

AAAGAGAACCGATTCATTGAAATTGGAGTGACACGGAGGGAAGTTCACATATACTACCTA

GAAAAAGCAAATAAGATAAAATCAGAGAGGACACACATTCACATATTCTCATTCACTGGA

GAGGAAATGGCCACCAAAGCTGACTATACTCTTGATGAGGAGAGCAGAGCAAGAATTAAG

ACCCGGCTGTTCACTATAAGACAAGAAATGGCCAGTAGGGGTCTATGGGATTCCTTTCGT

CAATCCGAGAGAGGCGAAGAGACAATTGAAGAAAGGTTTGAAATCACAGGAACCATGCGC

AGGCTTGCTGACCAAAGTCTCCCACCGAACTTCTCCAGCCTTGAAAATTTTAGAGCCTAT

GTGGATGGATTCGAACCGAACGGCTGCATTGAGGGCAAGCTTTCTCAAATGTCAAAGGAA

GTGAACGCCAGAATTGAGCCATTTATGAAGGCAACACCACGCCCTCTCAGACTGCCTGAT

GGGCCTCCCTGCTCCCAGCGGTCGAAGTTCTTACTGATGGATGCCCTTAAATTAAGCATT

GAAGATCCGAGCCATGAGGGGGAAGGTATACCATTATATGATGCAATCAAATGTATGAAG

ACATTTTTCGGCTGGAAAGAGCCCAACATCGTAAAACCACATGACAAGGGCATAAATCCC

AATTACCTCCTGGCTTGGAAGCAGGTGCTGGCAGAACTCCAAGACATTGAAAATGAAGAT

AAAATCCCAAAAACAAAGAACATGAAGAAAACAAGCCAATTAAAGTGGGCACTTGGTGAG

AATATGGCACCTGAAAAAGTAGACTTTGAGGATTGCAAAGATGTTAGTGATCTAAAGCAG

TATAACAGTGATGAACCAGAGCCTAGATCGTTATCAAGCTGGGTTCAGAGCGAATTCAAC

AAGGCATGCGAATTGACAGATTCGAGTTGGATTGAACTTGATGAAATAGGAGAAGATGTT

GCTCCAATTGAGCACATTGCAAGTATGAGAAGAAACTATTTCACAGCGGAAGTGTCTCAT

TGCAGGGCTACTGAATACATAATGAAGGGAGTGTATATAAATACAGCTCTATTGAATGCA

TCCTGTGCAGCCATGGATGACTTCCAATTGATTCCAATGATAAGCAAGTGCAGAACCAAA

GAAGGAAGACGGAAGACAAATCTGTATGGATTCATTATAAAAGGAAGGTCCCATTTGAGG

AATGACACCGATGTGGTAAATTTTGTGAGCATGGAATTCTCTCTTACTGACCCGAGGCTG

GAACCACACAAGTGGGTAAAGTACTGTGTTCTTGAAATAGGGGACATGCTTTTACGAACG

GCAATAGGCCAAGTGTCAAGACCCATGTTCTTGTATGTGAGAACCAATGGGACTTCCAAG

ATTAAGATGAAATGGGGCATGGAGATGAGGCGATGCCTTCTTCAATCCCTTCAACAAATT

GAGAGCATGATTGAAGCCGAGTCTTCTGTCAAAGAGAAAGACATGACCAAAGAGTTCTTT

GAGACCAAATCAGAAACGTGGCCAATTGGGGAATCACCTAAGGGGGTGGAGGAAAGCTCC

ATTGGGAAGGTGTGCAGAACACTACTAGCAAAATCTGTCTTCAACAGCCTATATGCATCT

CCACAACTTGAGGGATTTTCAGCTGAATCAAGAAAATTGCTTCTCATTGTTCAGGCACTT

AGGGACAACCTGGAACCTGGGACCTTCGATCTTGGGGGGCTATATGACGCAATTGAGGAG

TGCCTGATTAATGATCCCTGGGTTTTGCTTAATGCATCTTGGTTCAACTCCTTCCTCACA

CATGCACTGAAATAGTTGTGGCAATGCTACTA---TTCGCTATCCATACTGTCCAAAAA-

----------------

>A_chicken_Vietnam_NCVD-15A59_2015_EPI895062

------------TACTGATCCAAAATGGAGGACTTTGTGCGACAATGCTTCAATCCAATG

ATCGTCGAGCTTGCGGAAAAGACAATGAAAGAATATGGGGAAAATCCGAAAATCGAGACG

AACAAATTTGCCGCAATATGCACACACCTAGAAGTCTGCTTCATGTACTCGGACTTCCAC

TTTATAGATGAACGAGGCGAATCAGTAATTGCAGAGTCGGGCGATCCGAATGCATTATTG

AAACACCGATTTGAGATAATTGAAGGGAGGGACCGAACAATGGCTTGGACAGTGGTAAAT

AGTATCTGCAACACCACAGGGGTCGATAAGCCTAAATTCCTCCCAGATTTGTATGACTAC

AAAGAGAACCGGTTCATTGAAATTGGAGTGACACGGAGGGAAGTTCACTTATACTACCTA

GAAAAAGCGAATAAGATAAAATCAGAGAGGACACACATTCACATATTCTCATTCACTGGA

GAGGAGATGGCCACCAAAGCTGACTATACTCTTGATGAGGAGAGCAGAGCAAGAATTAAA

ACCCGGTTGTTCACTATAAGACAAGAAATGGCCAGTAGGGGTCTGTGGGATTCCTTTCGT

CAATCCGAGAGAGGCGAAGAGACAATTGAAGAAAGGTTTGAAATCACAGGAACCATGCGC

AGGCTTGCTGACCAAAGTCTCCCACCGAACTTCTCCAGCCTTGAAAATTTTAGAGCCTAT

GTGGATGGATTCGAACCGAACGGATGCATTGAGGGCAAGCTTTCTCAAATGTCAAAGGAA

GTGAACGCCAGAATTGAGCCGTTTATGAAGACAACACCACGTCCTCTCAGACTACCTGAT

GGGCCTCCCTGCTCCCAGCGGTCGAAGTTCTTACTGATGGATGCCCTTAAACTAAGCATT

GAAGACCCGAGCCATGAGGGGGAAGGTATACCACTATATGATGCAATCAAATGCATGAAG

ACATTTTTCGGCTGGAAAGAGCCCAATATCGTAAAACCACATGACAAGGGCATAAATCCC

AATTACCTCCTGGCTTGGAAGCAGGTGCTGGCAGAACTCCAAGACATTGAAAATGAAGAT

AAAATCCCAAAAACAAAGAACATGAAGAAAACAAGCCAATTAAAGTGGGCACTTGGTGAG

AATATGGCACCTGAAAAAGTAGACTTTGAGGACTGCAAAGATGTTAGTGATCTAAAGCAG

TATGACAGTGATGAACCAAAGCCTAGGTCGTTATCAAGCTGGGTTCAGAGCGAATTCAAC

AAGGCTTGCGAATTGACAGATTCGAGTTGGATTGAACTTGATGAAATAGGAGAAGATGTT

GCTCCAATTGAGCACATTGCAAGTATGAGAAGAAACTATTTCACAGCGGAAGTGTCTCAT

TGCAGGGCTACTGAATACATAATGAAGGGAGTGTATATAAATACAGCCCTATTGAATGCA

TCCTGTGCAGCCATGGATGACTTCCAATTGATTCCAATGATAAGCAAGTGCAGAACCAGA

GAAGGAAGACGGAAGACAAATCTGTATGGATTCATTATAAAAGGAAGGTCCCATTTGAGG

AATGACACCGATGTGGTAAACTTTGTGAGCATGGAATTCTCTCTTACTGACCCGAGGCTG

GAACCGCACAAGTGGGTAAAGTACTGTGTTCTTGAAATAGGAGACATGCTCTTACGAACG

GCAATAGGCCAAGTGTCAAGACCCATGTTCTTGTATGTGAGAACCAATGGGACTTCCAAG

ATTAAGATGAAATGGGGCATGGAGATGAGGCGATGTCTTCTTCAATCCCTTCAGCAAATT

GAGAGCATGATTGAAGCCGAGTCCTCTGTCAAAGAGAAAGACATGACCAAAGAGTTCTTT

GAGACCAAATCAGAAATGTGGCCAATTGGGGAATCACCTAAGGGAGTGGAGGAAAGCTCC

ATTGGGAAAGTGTGCAGAACACTACTAGCAAAATCTGTCTTCAACAGCCTATATGCTTCT

CCACAACTTGAGGGGTTTTCAGCTGAATCAAGAAAATTGCTTCTCATTGTTCAGGCACTT

AGGGACAACCTGGAACCTGGGACCTTCGATCTTGGGGGGCTATATGACGCAATTGAGGAG

TGTCTGATTAATGATCCCTGGGTTTTGCTTAATGCATCTTGGTTCAACTCCTTCCTCACA

CATGCACTGAAATAGTTATGGCAATGCTACTA---TTCGCTATCCATACTGTCCAAAAA-

----------------

>A_duck_Wuhan_JXYFB22_2015_EPI683231

-------------------CCAAAATGGAGGACTTTGTGCGACAATGCTTCAATCCAATG

ATCGTCGAGCTTGCGGAAAAGACAATGAAAGAGTATGGGGAAAGTCCGAAAATCGAGACG

AACAAATTTGCCGCATTATGCACACACCTAGAAGTCTGCTTCATGTATTCGGACTTCCAC

TTTATAGATGAACGAGGCGAATCAATAATTGCAGAATCTGGCGATCCGAATGCATTATTG

AAACACCGATTTGAGATAATTGAAGGGAGGGACCGAACAATGGCTTGGACAGTGGTAAAT

AGTATCTGCAACACCACAGGGGTCGATAAGCCTAAATTCCTCCCAGATTTGTATGACTAC

AAAGAGAACCGATTCATTGAAATTGGAGTGACACGGAGGGAAGTTCACATATACTACCTA

GAAAAAGCAAATAAGATAAAGTCAGAGAGGACACACATTCACATATTCTCATTCACTGGA

GAGGAAATGGCCACCAAAGCTGACTATACTCTTGATGAGGAGAGCAGAGCAAGAATTAAA

ACCCGGCTGTTCACTATAAGACAGGAAATGGCCAGTAGGGGTCTATGGGATTCCTTTCGT

CAATCCGAGAGAGGCGAAGAGACAATTGAAGAAAGGTTTGAAATCACAGGAACCATGCGC

AGGCTTGCTGACCAAAGTCTCCCACCGAACTTCTCCAGCCTTGAAAATTTTAGAGCCTAT

GTGGATGGATTCGAACCGAACGGCTGCATTGAGGGCAAGCTTTCTCAAATGTCAAAGGAA

GTGAACGCCAGAATTGAGCCATTTATGAAGACAACACCACGCCCTCTCAGACTACCTGAT

GGGCCTCCCTGCTCCCAGCGGTCGAAGTTCTTACTGATGGATGCCCTTAAATTAAGCATT

GAAGATCCGAGCCATGAGGGGGAAGGTATACCACTATATGATGCAATCAAATGCATGAAG

ACATTTTTCGGCTGGAAAGAGCCCAACATCGTAAAACCACATGACAAGGGCATAAATCCC

AATTACCTCCTGGCTTGGAAGCAGGTGCTGGCAGAACTCCAAGACATTGAAAATGAAGAT

AAAATCCCAAAAACAAAGAACATGAAGAAAACAAGCCAATTAAAGTGGGCACTTGGTGAG

AATATGGCACCTGAAAAAGTAGACTTTGAGGACTGCAAAGATGTTAGTGATCTAAAGCAG

TATGACAGTGATGAACCAGAGCCTAGATCATTATCAAGCTGGGTTCAGAGCGAATTCAAC

AAGGCATGCGAATTGACAGATTCGAGTTGGATTGAACTTGATGAAATAGGAGAAGATGTT

GCTCCAATTGAGCACATTGCAAGTATGAGAAGAAACTATTTCACAGCGGAAGTGTCTCAT

TGCAGGGCTACTGAATACATAATGAAGGGAGTGTATATAAATACGGCCCTATTGAATGCA

TCCTGTGCAGCCATGGATGACTTCCAATTGATTCCAATGATAAGCAAGTGCAGAACCAAA

GAAGGGAGACGGAAAACAAATCTGTATGGATTCATTATAAAAGGAAGGTCCCATTTGAGG

AATGACACCGATGTGGTAAACTTTGTGAGCATGGAATTCTCTCTTACTGACCCGAGGCTG

GAACCACACAAGTGGGTAAAGTACTGTGTTCTTGAAATAGGAGACATGCTCTTACGAACG

GCAATAGGCCAAGTGTCAAGACCCATGTTTTTGTATGTGAGAACCAATGGGACTTCCAAG

ATTAAGATGAAATGGGGCATGGAGATGAGGCGATGCCTTCTTCAATCCCTTCAACAAATT

GAGAGCATGATTGAAGCCGAGTCTTCTGTCAAAGAGAAAGACATGACCAAAGAATTCTTT

GAGACCAAATCAGAAACGTGGCCAATTGGGGAATCACCTAAGGGGGTGGAGGAAAGCTCC

ATTGGGAAGGTGTGCAGAACACTACTAGCAAAATCTGTCTTCAACAGCCTTTATGCATCT

CCACAACTTGAGGGGTTTTCAGCTGAATCAAGAAAATTGCTTCTCATTGTTCAGGCACTT

AGGGACAACCTGGAACCTGGGACCTTCGATCTTGGGGGGCTATATGACGCAATTGAGGAG

TGCCTGATTAATGATCCCTGGGTTTTGCTTAATGCATCTTGGTTCAACTCCTTCCTCACA

CATGCACTGAAATAGTTGTGGCAATGCTACTA----------------------------

----------------

>A_goose_Hunan_116_2014_EPI958631

------------------------ATGGAGGACTTTGTGCGACAATGCTTCAATCCAATG

ATCGTCGAGCTTGCGGAAAAGACAATGAAAGAATATGGGGAAAGTCCGAAAATCGAGACG

AACAAATTTGCCGCAATATGCACACACCTAGAAGTCTGCTTCATGTATTCGGACTTCCAC

TTTATAGATGAACGAGGCGAATCAATAATTGCAGAATCTGGCGATCCGAATGCATTATTG

AAGCACCGATTTGAGATAATTGAAGGGAGGGACCGAACAATGGCTTGGACAGTGGTAAAT

AGTATCTGCAACACCACAGGGGTCGATAAGCCTAAATTCCTCCCAGATTTATATGACTAC

AAAGAGAACCGATTCATTGAAATTGGAGTGACACGGAGGGAAGTTCACATATACTACCTA

GAAAAAGCAAATAAGATAAAATCAGAGAGGACACACATTCACATATTCTCATTCACTGGA

GAGGAAATGGCTACCAAAGCTGACTATACTCTTGATGAGGAGAGCAGAGCAAGAATTAAA

ACCAGGCTGTTCACTATAAGACAAGAAATGGCCAGTAGGGGTCTATGGGATTCCTTTCGT

CAATCCGAGAGAGGCGAAGAGACAATTGAAGAAAGGTTTGAAATCACAGGAACCATGCGC

AGGCTTGCTGACCAAAGTCTCCCACCGAACTTCTCCAGCCTTGAAAATTTTAGAGCCTAT

GTGGATGGATTCGAACCGAACGGCTGCATTGAGGGCAAGCTTTCTCAAATGTCAAAGGAA

GTGAACGCCAGAATTGAGCCATTTATGAAGACAACACCACGCCCTCTCAGACTACCTGAG

GGGCCTCCCTGCTCCCAGCGGTCGAAGTTCTTACTAATGGATGCCCTTAAATTAAGCATT

GAAGATCCGAGCCATGAAGGGGAAGGTATACCACTATATGATGCAATCAAATGCATGAAG

ACATTCTTCGGCTGGAAAGAGCCCAACATCGTAAAACCACATGACAAGGGCATAAATCCC

AATTACCTCCTGGCTTGGAAACAGGTGCTGGCAGAACTCCAAGACATTGAAAATGAAGAT

AAAATCCCAAAAACAAAGAACATGAAGAAAACAAGCCAATTAAAGTGGGCACTTGGTGAG

AATATGGCACCTGAAAAAGTAGACTTTGAGGACTGCAAAGATGTTAGTGATCTAAAGCAG

TATGACAGTGATGAACCAGAGCCTAGATCATTATCAAGCTGGGTTCAGAGCGAATTCAAC

AAGGCATGCGAATTGACAGATTCGAGTTGGATTGAACTTGATGAAATAGGAGAAGATGTT

GCTCCAATTGAGCACATTGCAAGTATGAGAAGAAACTATTTCACAGCGGAAGTGTCTCAT

TGCAGGGCTACTGAATACATAATGAAGGGAGTGTATATAAATACAGCCCTATTGAATGCA

TCCTGTGCAGCCATGGATGACTTCCAATTGATTCCAATGATAAGCAAGTGCAGAACCAAA

GAAGGAAGACGGAAGACAAATCTGTATGGATTCATTATAAAAGGAAGGTCCCATTTGAGG

AATGACACCGATGTGGTGAACTTTGTGAGCATGGAGTTCTCTCTTACTGACCCGAGGCTG

GAACCACACAAGTGGGTAAAGTACTGTGTTCTTGAAATAGGAGACATGCTCTTACGAACG

GCAATAGGCCAAGTGTCAAGACCTATGTTCTTGTATGTGAGAACCAATGGGACTTCCAAG

ATTAAGATGAAATGGGGCATGGAGATGAGGCGATGCCTTCTTCAATCTCTTCAACAAATT

GAGAGCATGATTGAAGCCGAGTCTTCTGTCAAAGAGAAAGACATGACCAAAGAATTCTTT

GAGACCAAATCAGAAACGTGGCCAATTGGGGAATCACCTAAGGGGGTGGAGGAAAGCTCC

ATTGGGAAGGTGTGCAGAACACTACTAGCAAAATCTGTCTTCAACAGCCTATATGCATCT

CCACAACTTGAGGGGTTTTCAGCTGAATCAAGAAAATTGCTTCTCATTGTTCAGGCACTT

AGGGACAACCTGGAACCTGGGACCTTCGATCTTGGGGGGCTATATGACGCAATTGAGGAG

TGCCTGATTAATGATCCCTGGGTTTTGCTTAATGCATCTTGGTTCAACTCCTTCCTCACA

CATGCACTGAAATAG---------------------------------------------

----------------

>A_Guangdong_18SF020_2018_EPI1352809

------------------------ATGGAGGACTTTGTGCGACAATGCTTCAATCCAATG

ATCGTCGAGCTTGCGGAAAAGACAATGAAAGAATATGGGGAAAGTCCGAAAATCGAGACG

AACAAATTTGCCGCAATATGCACACACCTAGAAGTCTGCTTCATGTACTCGGACTTCCAC

TTTATAGATGAACGAGGCGAATCAATAATTGCAGAGTCTGGCGATCCGAACGCATTATTG

AAACACCGATTTGAGATAATTGAAGGGAGGGACCGAACAATGGCTTGGACAGTGGTAAAC

AGTATCTGCAACACCACAGGGGTCGATAAGCCTAAATTCCTCCCAGATTTATATGACTAC

AAAGAGAACCGGTTTATTGAAATTGGAGTGACACGGAGGGAAGTCCACATATACTACCTA

GAAAAAGCAAATAAGATAAAATCAGAGAGAACACACATCCACATATTCTCATTCACTGGA

GAGGAAATGGCCACCAAAGCTGATTATACTCTTGATGAGGAGAGCAGAGCAAGAATTAAA

ACCCGGCTGTTCACTATAAGACAAGAAATGGCCAGTAGGGGTCTATGGGATTCCTTTCGT

CAATCCGAGAGAGGCGAAGAGACAATTGAAGAAAGGTTTGAAATCACAGGAACCATGCGC

AGGCTTGCTGACCAAAGTCTCCCACCGAACTTCTCCAGCCTTGAAAATTTTAGAGCCTAT

GTGGATGGATTCGAACCGAACGGCTGCATTGAGGGCAAGCTTTCTCAAATGTCAAAGGAA

GTGAATGCCAGAATTGAGCCATTTATGAAGACGACACCACGTCCTCTTAGACTACCTGAT

GGGCCTCCCTGCTCCCAACGGTCGAAGTTCTTGCTGATGGATGCCCTTAAACTAAGCATT

GAAGATCCGAGCCATGAGGGGGAAGGTATCCCATTATATGATGCAATCAAATGCATGAAG

ACATTTTTCGGCTGGAAAGAGCCCAATATAGTAAAACCACATGACAAGGGCATAAATCCC

AATTACCTCCTGGCTTGGAAGCAGGTGCTGGCAGAATTCCAAGACATTGAAAATGAAGAT

AAAATCCCAAAAACAAAGAACCTGAAGAAAACAAGCCAATTAAAGTGGGCACTTGGTGAG

AATATGGCACCTGAAAAAGTAGACTTTGAGGACTGCAAAGATGTTAGTGATCTAAAGCAG

TATGACAGTGATGAACCAGAGCCTAGATCATTATCGAGCTGGGTTCAGAGCGAATTTAAC

AAGGCATGCGAATTGACAGATTCGAGTTGGATTGAACTTGATGAAATAGGAGAAGATGTT

GCTCCAATTGAGCACATTGCAAGTATGAGAAGAAACTATTTCACAGCGGARGTGTCCCAT

TGCAGGGCTACTGAATACATAATGAAGGGGGTGTATATAAATACAGCCCTATTGAATGCA

TCCTGTGCAGCCATGGATGACTTCCAACTGATTCCAATGATAAGCAAGTGCAGAACCAGA

GAAGGAAGACGGAAGACAAATCTGTATGGGTTCATTATAAAAGGAAGATCCCATTTGAGG

AATGACACCGATGTGGTGAACTTTGTGAGCATGGAATTCTCTCTTACTGACCCGAGGCTG

GAACCACACAAGTGGGTAAAGTACTGTGTTCTTGAAATAGGAGACATGCTCTTACGGACG

GCAATAGGTCAAGTGTCAAGACCCATGTTTTTGTATGTGAGAACCAATGGGACTTCCAAG

ATTAAGATGAAATGGGGCATGGAGATGAGGAGATGCCTTCTTCAATCCCTTCAACAAATT

GAGAGCATGATTGAAGCCGAGTCCTCTGTCAAAGAAAAAGACATGACCAAAGAGTTCTTT

GAGACCAAATCAGAAACGTGGCCAATTGGGGAGTCACCTAAGGGGGTGGAGGAAAGCTCC

ATTGGGAAGGTGTGCAGAACACTACTAGCAAAATCTGTCTTCAACAGCCTATATGCTTCT

CCACAACTTGAGGGGTTTTCAGCTGAATCAAGAAAATTGCTTCTCATTGTTCAGGCACTT

AGGGACAACCTGGAACCTGGGACCTTCGATCTTGGGGGGCTATATGAAGCAATCGAGGAG

TGCCTGATTAATGATCCCTGGGTTTTGCTTAATGCATCTTGGTTCAACTCCTTCCTCACA

CATGCACTGAGATAG---------------------------------------------

----------------

>A_duck_Bangladesh_43127_2020_EPI1902990

------------------------ATGGAGGACTTTGTGCGACAATGCTTCAATCCAATG

ATCGTCGAGCTTGCGGAAAAGACAATGAAAGAATATGGGGAAAGTCCGAAAATCGAGACG

AACAAATTTGCAGCAATATGCACACACCTAGAAGTCTGCTTCATGTACTCGGACTTCCAC

TTTATAGATGAACGAGGCGAATCAATAATTGCAGAGTCTGGCGATCCGAACGCATTATTG

AAACACCGATTTGAGATAATTGAAGGGAGGGACCGAACAATGGCTTGGACAGTGGTAAAC

AGTATCTGCAACACCACAGGGGTCGATAAGCCTAAATTCCTCCCAGATTTATATGACTAC

AAAGAGAACCGGTTCATTGAAATTGGAGTGACACGGAGGGAAGTCCACATATATTACCTA

GAAAAAGCAAATAAGATAAAATCAGAGAGGACACACATACACATATTCTCATTCACTGGA

GAGGAAATGGCCACCAAAGCCGACTATACTCTTGATGAGGAGAGCAGAGCAAGAATTAAA

ACCCGGCTGTTCACTATAAGACAAGAAATGGCCAGTAGGGGCCTATGGGATTCCTTTCGT

CAATCCGAGAGAGGCGAAGAGACAATTGAAGAAAGGTTTGAAATCACAGGAACCATGCGC

AGGCTTGCTGACCAAAGCCTCCCACCGAACTTCTCCAGCCTTGAAAATTTTAGAGCCTAT

GTGGATGGATTCGAACCGAACGGCTGCATTGAGGGCAAGCTTTCTCAAATGTCAAAGGAA

GTGAACGCCAGAATTGAGCCATTTATGAAGACGACACCACGCCCTCTCAGACTACCTGAT

GGGCCTCCCTGCTCCCAGCGGTCGAAGTTCTTACTGATGGATGCCCTTAAACTAAGCATT

GAAGATCCGAGCCATGAGGGGGAAGGTATCCCATTATATGATGCAATCAAATGCATGAAG

ACGTTTTTCGGCTGGAAAGAGCCCAATATAGTAAAACCACATGACAAGGGCATAAATCCC

AATTACCTCCTGGCTTGGAAGCAGGTGCTGGCAGAACTCCAAGACATTGAAAATGAAGAT

AAAATCCCAAGAACAAAGAACATGAAGAAAACAAGCCAATTAAAGTGGGCACTTGGTGAG

AACATGGCACCTGAAAAAGTCGACTTTGAGGACTGCAAGGATGTTAGTGATCTAAAGCAG

TATGACAGTGATGAACCAGAGCCTAGATCGTTATCGAGCTGGGTTCAGAGCGAATTTAAC

AAGGCATGCGAATTGACAGATTCGAGTTGGATTGAACTTGATGAAATAGGAGAAGATGTT

GCTCCAATTGAGCACATTGCAAGTATGAGAAGAAACTATTTCACAGCGGAAGTGTCTCAT

TGCAGGGCTACTGAATACATAATGAAGGGAGTGTATATAAATACAGCCCTATTGAATGCA

TCCTGTGCAGCCATGGATGACTTCCAATTGATCCCAATGATAAGCAAGTGCAGAACCAGA

GAAGGAAGACGGAAGACAAATCTGTATGGGTTCATTATAAAAGGAAGATCCCATTTGAGG

AATGACACCGATGTGGTGAACTTTGTGAGCATGGAATTCTCTCTTACTGACCCGAGGCTG

GAACCTCACAAGTGGGTAAAGTACTGTGTTCTTGAAATAGGAGACATGCTCTTACGGACG

GCAATAGGTCAAGTGTCAAGACCCATGTTTTTGTATGTGAGAACCAATGGGACTTCCAAG

ATTAAGATGAAATGGGGCATGGAGATGAGGAGATGCCTTCTTCAATCCCTTCAACAAATT

GAGAGCATGATTGAAGCCGAGTCCTCTGTCAAAGAGAAAGACATGACCAGAGAGTTCTTT

GAGACCAAATCAGAAACGTGGCCAATTGGGGAGTCACCCAAGGGGGTGGAGGAAAGCTCC

ATTGGGAAAGTGTGCAGAACACTACTAGCAAAATCTGTCTTCAACAGCCTATATGCTTCT

CCACAACTTGAGGGGTTTTCAGCTGAATCAAGAAAACTGCTTCTCATTGTTCAGGCACTT

AGGGACAACCTGGAACCTGGGACCTTCGATCTTGGGGGGCTATATGACGCAATTGAGGAG

TGCCTGATTAATGATCCCTGGGTTTTGCTTAATGCATCTTGGTTCAACTCCTTCCTCACA

CATGCACTGAAATAGTTGTGGCAATGCTACTA---TTCGCTAT-----------------

----------------
