# Supplementary material for: Highly pathogenic avian influenza virus of the A/H5N8 subtype, clade 2.3.4.4b, caused outbreaks in Kazakhstan in 2020
Source: PeerJ. 2022 Mar 2;10:e13038. doi: 10.7717/peerj.13038 (PMC8898005; doi:10.7717/peerj.13038)
Supplement: Figure S6 [file peerj-10-13038-s007.docx]

**Fig.S6** Alignment of the nucleotide sequences for the NP segment used in this study

>A_goose_Kazakhstan_4-190-20-B-H5N8-1_2020_EPI1927651

AGCAAAAGCAGGGTAGATAATCACTCACTGAGTGACATCAACATCATGGCGTCTCAAGGC

ACCAAACGATCTTATGAACAGATGGAAACTGGTGGGGAGCGCCAGAATGCCACTGAGATT

AGAGCATCGGTTGGAAGAATGGTTGGTGGAATTGGGAGGTTCTACATACAGATGTGCACT

GAGCTCAAACTCAGCGACTATGAAGGAAGACTGATCCAGAACAGCATAACAATAGAGAGA

ATGGTTCTCTCCGCATTTGATGAAAGGAGAAACAAATATCTGGAAGAACATCCCAGTGCG

GGGAAAGACCCGAAGAAAACAGGAGGTCCAATTTATCGAAGGAGAGATGGGAAATGGGTG

AGAGAGCTGATCCTGTATGACAAAGAGGAGATCAGGAGGATCTGGCGTCAAGCGAATAAT

GGAGAAGACGCAACTGCTGGTCTCACTCACCTGATGATCTGGCATTCCAATCTAAATGAT

GCCACATACCAGAGGACAAGAGCTCTCGTGCGTACCGGTATGGACCCCAGGATGTGCTCC

CTCATGCAAGGGTCAACTCTCCCAAGGAGGTCTGGAGCTGCTGGTGCAGCAGTGAAGGGA

GTTGGAACGATGGTGATGGAACTAATTCGGATGATAAAGCGAGGAGTTAATGATCGAAAC

TTCTGGAGAGGCGAGAATGGACGGAGGACAAGGATTGCATTTGAGAGAATGTGCAATATT

CTCAAAGGGAAATTCCAAACAGCAGCACAAAGAGCTATGATGGATCAAGTGCGTGAAAGC

AGGAATCCTGGCAATGCTGAAATTGAAGATCTCATTTTTCTGGCGCGGTCTGCTCTCATT

CTGAGAGGATCAGTGGCCCATAAGTCCTGCTTGCCCGCTTGTGTATACGGGCTCGCTGTA

GCCAGTGGATACGAATTTGAAAGAGAAGGGTACTCTCTAGTTGGAATAGACCCTTTCCGA

CTGCTTCAAAACAGCCAGGTCTTTAGTCTCATTAGACCAAATGAGAATCCAGCACACAAG

AGTCAATTGGTGTGGATGGCATGTCATTCTGCAGCATTCGAGGATCTGAGAGTCTCAAGT

TTCATCAGAGGAACAAGAGTAGTCCCAAGAGGACAACTATCCACTAGAGGAGTTCAAATT

GCTTCAAATGAGAACATAGAAACAATGGACTCCAGCACTCTTGAACTGAGAAGCAGATAT

TGGGCTATAAGAACCAGGAGTGGAGGAAACACCAACCAACAGAGAGCATCTGCAGGACAA

ATCAGTGTACAGCCCACTTTCTCGGTACAGAGAAACCTTCCCTTCGAAAGAGCGACCATT

ATGGCGGCATTCACAGGGAACACTGAAGGCAGAACGTCCGACATGAGGACTGAAATCATA

AGAATGATGGAAAGTGCCAGACCAGAAGATGTGTCTTTCCAGGGGCGGGGAGTCTTCGAG

CTCTCGGACGAAAAGGCAACGAACCCGATCGTGCCTTCCTTTGACATGAGTAATGAAGGA

TCTTATTTCTTCGGAGACAATGCAGAGGAGTATGACAATTAAAGAAAAATACCCTTGTTT

CTACT-------------------------------------------------

>A_chicken_Kazakhstan_220-B-2-H5N8-4_2020_EPI1927657

AGCAAAAGCAGGGTAGATAATCACTCACTGAGTGACATCAACATCATGGCGTCTCAAGGC

ACCAAACGATCTTATGAACAGATGGAAACTGGTGGGGAGCGCCAGAATGCCACTGAGATT

AGAGCATCGGTTGGAAGAATGGTTGGTGGAATTGGGAGGTTCTACATACAGATGTGCACT

GAGCTCAAACTCAGCGACTATGAAGGAAGACTGATCCAGAACAGCATAACAATAGAGAGA

ATGGTTCTCTCCGCATTTGATGAAAGGAGAAACAAATATCTGGAAGAACATCCCAGTGCG

GGGAAAGACCCGAAGAAAACAGGAGGTCCAATTTATCGAAGGAGAGATGGGAAATGGGTG

AGAGAGCTGATCCTGTATGACAAAGAGGAGATCAGGAGGATCTGGCGTCAAGCGAATAAT

GGAGAAGACGCAACTGCTGGTCTCACTCACCTGATGATCTGGCATTCCAATCTAAATGAT

GCCACATACCAGAGGACAAGAGCTCTCGTGCGTACCGGTATGGACCCCAGGATGTGCTCC

CTCATGCAAGGGTCAACTCTCCCAAGGAGGTCTGGAGCTGCTGGTGCAGCAGTGAAGGGA

GTTGGAACGATGGTGATGGAACTAATTCGGATGATAAAGCGAGGAGTTAATGATCGAAAC

TTCTGGAGAGGCGAGAATGGACGGAGGACAAGGATTGCATTTGAGAGAATGTGCAATATT

CTCAAAGGGAAATTCCAAACAGCAGCACAAAGAGCTATGATGGATCAAGTGCGTGAAAGC

AGGAATCCTGGCAATGCTGAAATTGAAGATCTCATTTTTCTGGCGCGGTCTGCTCTCATT

CTGAGAGGATCAGTGGCCCATAAGTCCTGCTTGCCCGCTTGTGTATACGGGCTCGCTGTA

GCCAGTGGATACGAATTTGAAAGAGAAGGGTACTCTCTAGTTGGAATAGACCCTTTCCGA

CTGCTTCAAAACAGCCAGGTCTTTAGTCTCATTAGACCAAATGAGAATCCAGCACACAAG

AGTCAATTGGTGTGGATGGCATGTCATTCTGCAGCATTCGAGGATCTGAGAGTCTCAAGT

TTCATCAGAGGAACAAGAGTAGTCCCAAGAGGACAACTATCCACTAGAGGAGTTCAAATT

GCTTCAAATGAGAACATAGAAACAATGGACTCCAGCACTCTTGAACTGAGAAGCAGATAT

TGGGCTATAAGAACCAGGAGTGGAGGAAACACCAACCAACAGAGAGCATCTGCAGGACAA

ATCAGTGTACAGCCCACTTTCTCGGTACAGAGAAACCTTCCCTTCGAAAGAGCGACCATT

ATGGCGGCATTCACAGGGAACACTGAAGGCAGAACGTCCGACATGAGGACTGAAATCATA

AGAATGATGGAAAGTGCCAGACCAGAAGATGTGTCTTTCCAGGGGCGGGGAGTCTTCGAG

CTCTCGGACGAAAAGGCAACGAACCCGATCGTGCCTTCCTTTGACATGAGTAATGAAGGA

TCTTATTTCTTCGGAGACAATGCAGAGGAGTATGACAATTAAAGAAAAATACCCTTGTTT

CTACT-------------------------------------------------

>A_duck_Kazakhstan_12-20-B-Talg-11_2020_EPI1927663

AGCAAAAGCAGGGTAGATAATCACTCACTGAGTGACATCAACATCATGGCGTCTCAAGGC

ACCAAACGATCTTATGAACAGATGGAAACTGGTGGGGAGCGCCAGAATGCCACTGAGATT

AGAGCATCGGTTGGAAGAATGGTTGGTGGAATTGGGAGGTTCTACATACAGATGTGCACT

GAGCTCAAACTCAGCGACTATGAAGGAAGACTGATCCAGAACAGCATAACAATAGAGAGA

ATGGTTCTCTCCGCATTTGATGAAAGGAGAAACAAATATCTGGAAGAACATCCCAGTGCG

GGGAAAGACCCGAAGAAAACAGGAGGTCCAATTTATCGAAGGAGAGATGGGAAATGGATG

AGAGAGCTGATCCTGTATGACAAAGAGGAGATCAGGAGGATCTGGCGTCAAGCGAATAAT

GGAGAAGACGCAACTGCTGGTCTCACTCACCTGATGATCTGGCATTCCAATCTAAATGAT

GCCACATACCAGAGGACAAGAGCTCTCGTGCGTACCGGTATGGACCCCAGGATGTGCTCC

CTCATGCAAGGGTCAACTCTCCCAAGGAGGTCTGGAGCTGCTGGTGCAGCAGTGAAGGGA

GTTGGAACGATGGTGATGGAACTAATTCGGATGATAAAGCGAGGAGTTAATGATCGAAAC

TTCTGGAGAGGCGAGAATGGACGGAGGACAAGGATTGCATTTGAGAGAATGTGCAATATT

CTCAAAGGGAAATTCCAAACAGCAGCACAAAGAGCAATGATGGATCAAGTGCGTGAAAGC

AGGAATCCTGGCAATGCTGAAATTGAAGATCTCATTTTTCTGGCGCGGTCTGCTCTCATT

CTGAGAGGATCAGTGGCCCATAAGTCCTGCTTGCCCGCTTGTGTATACGGGCTCGCTGTA

GCCAGTGGATACGACTTTGAGAGAGAAGGGTACTCTCTAGTTGGAATAGACCCTTTCCGA

CTGCTTCAAAACAGCCAGGTCTTTAGTCTCATTAGACCAAATGAGAATCCAGCACACAAG

AGTCAATTGGTGTGGATGGCATGTCATTCTGCAGCATTCGAGGATCTGAGAGTCTCAAGT

TTCATCAGAGGAACAAGAGTAGTCCCAAGAGGACAACTATCCACTAGAGGAGTTCAAATT

GCTTCAAATGAGAACATAGAAACAATGGACTCCAGCACTCTTGAACTGAGAAGCAGATAT

TGGGCTATAAGAACCAGGAGTGGAGGAAACACCAACCAACAGAGAGCATCTGCAGGACAA

ATCAGTGTACAGCCCACTTTCTCGGTACAGAGAAACCTTCCCTTCGAAAGAGCGACCATT

ATGGCGGCATTCACAGGGAACACTGAAGGCAGAACGTCCGACATGAGGACTGAAATCATA

AGAATGATGGAAAGTGCCAGACCAGAAGATGTGTCTTTCCAGGGGCGGGGAGTCTTCGAG

CTCTCGGACGAAAAGGCAACGAACCCGATCGTGCCTTCCTTTGACATGAGTAATGAAGGA

TCTTATTTCTTCGGAGACAATGCAGAGGAGTATGACAATTAAAGAAAAATACCCTTGTTT

CTACT-------------------------------------------------

>A_goose_Kazakhstan_7-20-B-Talg-12_2020_EPI1927669

AGCAAAAGCAGGGTAGATAATCACTCACTGAGTGACATCAACATCATGGCGTCTCAAGGC

ACCAAACGATCTTATGAACAGATGGAAACTGGTGGGGAGCGCCAGAATGCCACTGAGATT

AGAGCATCGGTTGGAAGAATGGTTGGTGGAATTGGGAGGTTCTACATACAGATGTGCACT

GAGCTCAAACTCAGCGACTATGAAGGAAGACTGATCCAGAACAGCATAACAATAGAGAGA

ATGGTTCTCTCCGCATTTGATGAAAGGAGAAACAAATATCTGGAAGAACATCCCAGTGCG

GGGAAAGACCCGAAGAAAACAGGAGGTCCAATTTATCGAAGGAGAGATGGGAAATGGATG

AGAGAGCTGATCCTGTATGACAAAGAGGAGATCAGGAGGATCTGGCGTCAAGCGAATAAT

GGAGAAGACGCAACTGCTGGTCTCACTCACCTGATGATCTGGCATTCCAATCTAAATGAT

GCCACATACCAGAGGACAAGAGCTCTCGTGCGTACCGGTATGGACCCCAGGATGTGCTCC

CTCATGCAAGGGTCAACTCTCCCAAGGAGGTCTGGAGCTGCTGGTGCAGCAGTGAAGGGA

GTTGGAACGATGGTGATGGAACTAATTCGGATGATAAAGCGAGGAGTTAATGATCGAAAC

TTCTGGAGAGGCGAGAATGGACGGAGGACAAGGATTGCATTTGAGAGAATGTGCAATATT

CTCAAAGGGAAATTCCAAACAGCAGCACAAAGAGCAATGATGGATCAAGTGCGTGAAAGC

AGGAATCCTGGCAATGCTGAAATTGAAGATCTCATTTTTCTGGCGCGGTCTGCTCTCATT

CTGAGAGGATCAGTGGCCCATAAGTCCTGCTTGCCCGCTTGTGTATACGGGCTCGCTGTA

GCCAGTGGATACGACTTTGAGAGAGAAGGGTACTCTCTAGTTGGAATAGACCCTTTCCGA

CTGCTTCAAAACAGCCAGGTCTTTAGTCTCATTAGACCAAATGAGAATCCAGCACACAAG

AGTCAATTGGTGTGGATGGCATGTCATTCTGCAGCATTCGAGGATCTGAGAGTCTCAAGT

TTCATCAGAGGAACAAGAGTAGTCCCAAGAGGACAACTATCCACTAGAGGAGTTCAAATT

GCTTCAAATGAGAACATAGAAACAATGGACTCCAGCACTCTTGAACTGAGAAGCAGATAT

TGGGCTATAAGAACCAGGAGTGGAGGAAACACCAACCAACAGAGAGCATCTGCAGGACAA

ATCAGTGTACAGCCCACTTTCTCGGTACAGAGAAACCTTCCCTTCGAAAGAGCGACCATT

ATGGCGGCATTCACAGGGAACACTGAAGGCAGAACGTCCGACATGAGGACTGAAATCATA

AGAATGATGGAAAGTGCCAGACCAGAAGATGTGTCTTTCCAGGGGCGGGGAGTCTTCGAG

CTCTCGGACGAAAAGGCAACGAACCCGATCGTGCCTTCCTTTGACATGAGTAATGAAGGA

TCTTATTTCTTCGGAGACAATGCAGAGGAGTATGACAATTAAAGAAAAATACCCTTGTTT

CTACT-------------------------------------------------

>A_swan_Kazakhstan_9-20-B-Talg-39_2020_EPI1927697

AGCAAAAGCAGGGTAGATAATCACTCACTGAGTGACATCAACATCATGGCGTCCCAAGGC

ACCAAACGATCCCATGAACAGATGGAAACTGGTGGGGAGCGCCAGAATGCCACTGAGATT

AGAGCATCGGTTGGAAGAATGGTTGGTGGAATTGGGAGGTTCTACATACAGATGTGCACT

GAGCTCAAACTCAGCGACTATGAAGGAAGACTGATCCAGAACAGCATAACAATAGAGAGA

ATGGTTCTCTCCGCATTTGATGAAAGGAGAAACAAATATCTGGAAGAACATCCCAGTGCG

GGGAAAGACCCGAAGAAAACAGGAGGTCCAATTTATCGAAGGAGAGATGGGAAATGGGTG

AGAGAGCTGATCCTGTATGACAAAGAGGAGATCAGGAGGATCTGGCGTCAAGCGAATAAT

GGAGAAGACGCAACTGCTGGTCTCACTCACCTAATGATCTGGCATTCCAATCTAAATGAT

GCCACATACCAGAGGACAAGAGCTCTCGTGCGTACCGGTATGGACCCCAGGATGTGCTCC

CTCATGCAAGGGTCAACTCTCCCAAGGAGGTCTGGAGCTGCTGGTGCAGCAGTGAAGGGA

GTTGGAACGATGGTGATGGAACTAATTCGGATGATAAAGCGAGGAGTTAATGATCGAAAC

TTCTGGAGAGGCGAGAATGGACGGAGGACAAGGATTGCATTTGAGAGAATGTGCAATATT

CTCAAAGGGAAATTCCAAACAGCAGCACAAAGAGCAATGATGGATCAAGTGCGTGAAAGC

AGGAATCCTGGCAATGCTGAAATTGAAGATCTCATTTTTCTGGCGCGGTCTGCTCTCATT

CTGAGAGGATCAGTGGCCCATAAGTCCTGCTTGCCCGCTTGTGTATACGGGCTCGCTGTA

GCCAGTGGATACGACTTTGAGAGAGAAGGGTACTCTCTAGTTGGAATAGACCCTTTCCGA

CTGCTTCAAAACAGCCAGGTCTTTAGTCTCATTAGACCAAATGAGAATCCAGCACACAAG

AGTCAATTGGTGTGGATGGCATGTCATTCTGCAGCATTCGAGGATCTGAGAGTCTCAAGT

TTCATCAGAGGAACAAGAGTAGTCCCAAGAGGACAACTATCCACTAGAGGAGTTCAAATT

GCTTCAAATGAGAACATAGAAACAATGGACTCCAGCACTCTTGAACTGAGAAGCAGATAT

TGGGCTATAAGAACCAGGAGTGGAGGAAACACCAACCAACAGAGAGCATCTGCAGGACAA

ATCAGCGTACAGCCCACTTTCTCAGTACAGAGAAACCTTCCCTTCGAAAGAGCGACCATT

ATGGCGGCATTCACAGGGAACACTGAAGGCAGAACGTCCGACATGAGGACTGAAATCATA

AGAATGATGGAAAGTGCCAGACCAGAAGATGTGTCTTTCCAGGGGCGGGGAGTCTTCGAG

CTCTCGGACGAAAAGGCAACGAACCCGATCGTGCCTTCCTTTGACATGAGTAATGAAGGA

TCTTATTTCTTCGGAGACAATGCAGAGGAGTATGACAATTAAAGAAAAATACCCTTGTTT

CTACT-------------------------------------------------

>A_chicken_Kazakhstan_12-20-B-Talg-45_2020_EPI1927703

AGCAAAAGCAGGGTAGATAATCACTCACTGAGTGACATCAACATCATGGCGTCTCAAGGC

ACCAAACGATCTTATGAACAGATGGAAACTAGTGGGGAGCGCCAGAATGCCACTGAGATT

AGAGCATCTGTTGGAAGAATGGTTGGTGGAATTGGGAGGTTCTACATACAGATGTGCACT

GAGCTCAAACTCAGCGACTATGAAGGAAGACTGATCCAGAACAGCATAACAATAGAGAGA

ATGGTTCTCTCCGCATTTGATGAAAGGAGAAACAAATATCTGGAAGAACATCCCAGTGCG

GGGAAAGACCCGAAGAAAACAGGAGGTCCAATTTATCGAAGGAGAGATGGGAAATGGGTG

AGAGAGCTGATCCTGTATGACAAAGAGGAGATCAGGAGGATCTGGCGTCAAGCGAATAAT

GGAGAAGACGCAACTGCTGGTCTCACTCACCTGATGATCTGGCATTCCAATCTAAATGAT

GCCACATACCAGAGGACAAGAGCTCTCGTGCGTACCGGTATGGACCCCAGGATGTGCTCC

CTCATGCAAGGGTCAACTCTCCCAAGGAGATCTGGAGCTGCTGGTGCAGCAGTGAAGGGA

GTTGGAACGATGGTGATGGAACTAATTCGGATGATAAAGCGAGGAGTTAATGATCGAAAC

TTCTGGAGAGGCGAGAATGGACGGAGGACAAGGATTGCATTTGAGAGAATGTGCAATATT

CTCAAAGGGAAATTCCAAACAGCAGCACAAAGAGCAATGATGGATCAAGTGCGTGAAAGC

AGGAATCCTGGCAATGCTGAAATTGAAGATCTCATTTTTCTGGCGCGGTCTGCTCTCATT

CTGAGAGGATCAGTGGCCCATAAGTCCTGCTTGCCCGCTTGTGTATACGGGCTCGCTGTA

GCCAGTGGATACGACTTTGAGAGAGAAGGGTACTCTCTAGTTGGAATAGACCCTTTCCGA

CTGCTTCAAAACAGCCAGGTCTTTAGTCTCATTAGACCAAATGAGAATCCAGCACACAAG

AGTCAATTGGTGTGGATGGCATGTCATTCTGCAGCATTCGAGGATCTGAGAGTCTCAAGT

TTCATCAGAGGAACAAGAGTAGTCCCAAGAGGACAACTATCCACTAGAGGAGTTCAAATT

GCTTCAAATGAGAACATAGAAACAATGGACTCCAGCACTCTTGAACTGAGAAGCAGATAT

TGGGCTATAAGAACCAGGAGTGGAGGAAACACCAACCAACAGAGAGCATCTGCAGGACAA

ATCAGTGTACAGCCCACTTTCTCGGTACAGAGAAACCTTCCCTTCGAAAGAGCGACCATT

ATGGCGGCATTCACAGGGAACACTGAAGGCAGAACGTCCGACATGAGGACTGAAATCATA

AGAATGATGGAAAGTGCCAGACCAGAAGATGTGTCTTTCCAGGGGCGGGGAGTCTTCGAG

CTCTCGGACGAAAAGGCAACGAACCCGATCGTGCCTTCCTTTGACATGAGTAATGAAGGA

TCTTATTTCTTCGGAGACAATGCAGAGGAGTATGACAATTAAAGAAAAATACCCTTGTTT

CTACT-------------------------------------------------

>A_crow_Kazakhstan_15-20-B-Talg-4_2020_EPI1927709

AGCAAAAGCAGGGTAGATAATCACTCACTGAGTGACATCAACATCATGGCGTCTCAAGGC

ACCAAACGATCTTATGAACAGATGGAAACTGGTGGGGAGCGCCAGAATGCCACTGAGATT

AGAGCATCGGTTGGAAGAATGGTTGGTGGAATTGGGAGGTTCTACATACAGATGTGCACT

GAGCTCAAACTCAGCGACTATGAAGGAAGACTGATCCAGAACAGCATAACAATAGAGAGA

ATGGTTCTCTCCGCATTTGATGAAAGGAGAAACAAATATCTGGAAGAACATCCCAGTGCG

GGGAAAGACCCGAAGAAAACAGGAGGTCCAATTTATCGAAGGAGAGATGGGAAATGGATG

AGAGAGCTGATCCTGTATGACAAAGAGGAGATCAGGAGGATCTGGCGTCAAGCGAATAAT

GGAGAAGACGCAACTGCTGGTCTCACTCACCTGATGATCTGGCATTCCAATCTAAATGAT

GCCACATACCAGAGGACAAGAGCTCTCGTGCGTACCGGTATGGACCCCAGGATGTGCTCC

CTCATGCAAGGGTCAACTCTCCCAAGGAGGTCTGGAGCTGCTGGTGCAGCAGTGAAGGGA

GTTGGAACGATGGTGATGGAACTAATTCGGATGATAAAGCGAGGAGTTAATGATCGAAAC

TTCTGGAGAGGCGAGAATGGACGGAGGACAAGGATTGCATTTGAGAGAATGTGCAATATT

CTCAAAGGGAAATTCCAAACAGCAGCACAAAGAGCAATGATGGATCAAGTGCGTGAAAGC

AGGAATCCTGGCAATGCTGAAATTGAAGATCTCATTTTTCTGGCGCGGTCTGCTCTCATT

CTGAGAGGATCAGTGGCCCATAAGTCCTGCTTGCCCGCTTGTGTATACGGGCTCGCTGTA

GCCAGTGGATACGACTTTGAGAGAGAAGGGTACTCTCTAGTTGGAATAGACCCTTTCCGA

CTGCTTCAAAACAGCCAGGTCTTTAGTCTCATTAGACCAAATGAGAATCCAGCACACAAG

AGTCAATTGGTGTGGATGGCATGTCATTCTGCAGCATTCGAGGATCTGAGAGTCTCAAGT

TTCATCAGAGGAACAAGAGTAGTCCCAAGAGGACAACTATCCACTAGAGGAGTTCAAATT

GCTTCAAATGAGAACATAGAAACAATGGACTCCAGCACTCTTGAACTGAGAAGCAGATAT

TGGGCTATAAGAACCAGGAGTGGAGGAAACACCAACCAACAGAGAGCATCTGCAGGACAA

ATCAGTGTACAGCCCACTTTCTCGGTACAGAGAAACCTTCCCTTCGAAAGAGCGACCATT

ATGGCGGCATTCACAGGGAACACTGAAGGCAGAACGTCCGACATGAGGACTGAAATCATA

AGAATGATGGAAAGTGCCAGACCAGAAGATGTGTCTTTCCAGGGGCGGGGAGTCTTCGAG

CTCTCGGACGAAAAGGCAACGAACCCGATCGTGCCTTCCTTTGACATGAGTAATGAAGGA

TCTTATTTCTTCGGAGACAATGCAGAGGAGTATGACAATTAAAGAAAAATACCCTTGTTT

CTACT-------------------------------------------------

>A_swan_Kazakhstan_1-267-20-B-Talg-52_2020_EPI1927715

AGCAAAAGCAGGGTAGATAATCACTCACTGAGTGACATCAACATCATGGCGTCTCAAGGC

ACCAAACGATCTTATGAACAGATGGAAACTAGTGGGGAGCGCCAGAATGCCACTGAGATT

AGAGCATCGGTTGGAAGAATGGTTGGTGGAATTGGGAGGTTCTACATACAGATGTGCACT

GAGCTCAAACTCAGCGACTATGAAGGAAGACTGATCCAGAACAGCATAACAATAGAGAGA

ATGGTTCTCTCCGCATTTGATGAAAGGAGAAACAAATATCTGGAAGAACATCCCAGTGCG

GGGAAAGACCCGAAGAAAACAGGAGGTCCAATTTATCGAAGGAGAGATGGGAAATGGGTG

AGAGAGCTGATCCTGTATGACAAAGAGGAGATCAGGAGGATCTGGCGTCAAGCGAATAAT

GGAGAAGACGCAACTGCTGGTCTCACTCACCTGATGATCTGGCATTCCAATCTAAATGAT

GCCACATACCAGAGGACAAGAGCTCTCGTGCGTACCGGTATGGACCCCAGGATGTGCTCC

CTCATGCAAGGGTCAACTCTCCCAAGGAGGTCTGGAGCTGCTGGTGCAGCAGTGAAGGGA

GTTGGAACGATGGTGATGGAACTAATTCGGATGATAAAGCGAGGAGTTAATGATCGAAAC

TTCTGGAGAGGCGAGAATGGACGGAGGACAAGGATTGCATTTGAGAGAATGTGCAATATT

CTCAAAGGGAAATTCCAAACAGCAGCACAAAGAGCAATGATGGATCAAGTGCGTGAAAGC

AGGAATCCTGGCAATGCTGAAATTGAAGATCTCATTTTTCTGGCGCGGTCTGCTCTCATT

CTGAGAGGATCAGTGGCCCATAAGTCCTGCTTGCCCGCTTGTGTATACGGGCTCGCTGTA

GCCAGTGGATACGACTTTGAGAGAGAAGGGTACTCTCTAGTTGGAATAGACCCTTTCCGA

CTGCTTCAAAACAGCCAGGTCTTTAGTCTCATTAGACCAAATGAGAATCCAGCACACAAG

AGTCAATTGGTGTGGATGGCATGTCATTCTGCAGCATTCGAGGATCTGAGAGTCTCAAGT

TTCATCAGAGGAACAAGAGTAGTCCCAAGAGGACAACTATCCACTAGAGGAGTTCAAATT

GCTTCAAATGAGAACATAGAAACAATGGACTCCAGCACTCTTGAACTGAGAAGCAGATAT

TGGGCTATAAGAACCAGGAGTGGAGGAAACACCAACCAACAGAGAGCATCTGCAGGACAA

ATCAGTGTACAGCCCACTTTCTCGGTACAGAGAAACCTTCCCTTTGAAAGAGCGACCATT

ATGGCGGCATTCACAGGGAACACTGAAGGCAGAACGTCCGACATGAGGACTGAAATCATA

AGAATGATGGAAAGTGCCAGACCAGAAGATGTGTCTTTCCAGGGGCGGGGAGTCTTCGAG

CTCTCGGACGAAAAGGCAACGAACCCGATCGTGCCTTCCTTTGACATGAGTAATGAAGGA

TCTTATTTCTTCGGAGACAATGCAGAGGAGTATGACAATTAAAGAAAAATACCCTTGTTT

CTACT-------------------------------------------------

>A_pigeon_Kazakhstan_15-20-B-Talg-5_2020_EPI1927721

AGCAAAAGCAGGGTAGATAATCACTCACTGAGTGACATCAACATCATGGCGTCTCAAGGC

ACCAAACGATCTTATGAACAGATGGAAACTGGTGGGGAGCGCCAGAATGCCACTGAGATT

AGAGCATCGGTTGGAAGAATGGTTGGTGGAATTGGGAGGTTCTACATACAGATGTGCACT

GAGCTCAAACTCAGCGACTATGAAGGAAGACTGATCCAGAACAGCATAACAATAGAGAGA

ATGGTTCTCTCCGCATTTGATGAAAGGAGAAACAAATATCTGGAAGAACATCCCAGTGCG

GGGAAAGACCCGAAGAAAACAGGAGGTCCAATTTATCGAAGGAGAGATGGGAAATGGATG

AGAGAGCTGATCCTGTATGACAAAGAGGAGATCAGGAGGATCTGGCGTCAAGCGAATAAT

GGAGAAGACGCAACTGCTGGTCTCACTCACCTGATGATCTGGCATTCCAATCTAAATGAT

GCCACATACCAGAGGACAAGAGCTCTCGTGCGTACCGGTATGGACCCCAGGATGTGCTCC

CTCATGCAAGGGTCAACTCTCCCAAGGAGGTCTGGAGCTGCTGGTGCAGCAGTGAAGGGA

GTTGGAACGATGGTGATGGAACTAATTCGGATGATAAAGCGAGGAGTTAATGATCGAAAC

TTCTGGAGAGGCGAGAATGGACGGAGGACAAGGATTGCATTTGAGAGAATGTGCAATATT

CTCAAAGGGAAATTCCAAACAGCAGCACAAAGAGCAATGATGGATCAAGTGCGTGAAAGC

AGGAATCCTGGCAATGCTGAAATTGAAGATCTCATTTTTCTGGCGCGGTCTGCTCTCATT

CTGAGAGGATCAGTGGCCCATAAGTCCTGCTTGCCCGCTTGTGTATACGGGCTCGCTGTA

GCCAGTGGATACGACTTTGAGAGAGAAGGGTACTCTCTAGTTGGAATAGACCCTTTCCGA

CTGCTTCAAAACAGCCAGGTCTTTAGTCTCATTAGACCAAATGAGAATCCAGCACACAAG

AGTCAATTGGTGTGGATGGCATGTCATTCTGCAGCATTCGAGGATCTGAGAGTCTCAAGT

TTCATCAGAGGAACAAGAGTAGTCCCAAGAGGACAACTATCCACTAGAGGAGTTCAAATT

GCTTCAAATGAGAACATAGAAACAATGGACTCCAGCACTCTTGAACTGAGAAGCAGATAT

TGGGCTATAAGAACCAGGAGTGGAGGAAACACCAACCAACAGAGAGCATCTGCAGGACAA

ATCAGTGTACAGCCCACTTTCTCGGTACAGAGAAACCTTCCCTTCGAAAGAGCGACCATT

ATGGCGGCATTCACAGGGAACACTGAAGGCAGAACGTCCGACATGAGGACTGAAATCATA

AGAATGATGGAAAGTGCCAGACCAGAAGATGTGTCTTTCCAGGGGCGGGGAGTCTTCGAG

CTCTCGGACGAAAAGGCAACGAACCCGATCGTGCCTTCCTTTGACATGAGTAATGAAGGA

TCTTATTTCTTCGGAGACAATGCAGAGGAGTATGACAATTAAAGAAAAATACCCTTGTTT

CTACT-------------------------------------------------

>A_chicken_Kazakhstan_1-20-B-Talg-67_2020_EPI1927727

AGCAAAAGCAGGGTAGATAATCACTCACTGAGTGACATCAACATCATGGCGTCTCAAGGC

ACCAAACGATCTTATGAACAGATGGAAACTGGTGGGGAGCGCCAGAATGCCACTGAGATT

AGAGCATCGGTTGGAAGAATGGTTGGTGGAATTGGGAGGTTCTACATACAGATGTGCACT

GAGCTCAAACTCAGCGACTATGAAGGAAGACTGATCCAGAACAGCATAACAATAGAGAGA

ATGGTTCTCTCCGCATTTGATGAAAGGAGAAACAAATATCTGGAAGAACATCCCAGTGCG

GGGAAAGACCCGAAGAAAACAGGAGGTCCAATTTATCGAAGGAGAGATGGGAAATGGGTG

AGAGAGCTGATCCTGTATGACAAAGAGGAGATCAGGAGGATCTGGCGTCAAGCGAATAAT

GGAGAAGACGCAACTGCTGGTCTCACTCACCTGATGATCTGGCATTCCAATCTAAATGAT

GCCACATACCAGAGGACAAGAGCTCTCGTGCGTACCGGTATGGACCCCAGGATGTGCTCC

CTCATGCAAGGGTCAACTCTCCCAAGGAGGTCTGGAGCTGCTGGTGCAGCAGTGAAGGGA

GTTGGAACGATGGTGATGGAACTAATTCGGATGATAAAGCGAGGAGTTAATGATCGAAAC

TTCTGGAGAGGCGAGAATGGACGGAGGACAAGGATTGCATTTGAGAGAATGTGCAATATT

CTCAAAGGGAAATTCCAAACAGCAGCACAAAGAGCAATGATGGATCAAGTGCGTGAAAGC

AGGAATCCTGGCAATGCTGAAATTGAAGATCTCATTTTTCTGGCGCGGTCTGCTCTCATT

CTGAGAGGATCAGTGGCCCATAAGTCCTGCTTGCCCGCTTGTGTATACGGGCTCGCTGTA

GCCAGTGGATACGACTTTGAGAGAGAAGGGTACTCTCTAGTTGGAATAGACCCTTTCCGA

CTGCTTCAAAACAGCCAGGTCTTTAGTCTCATTAGACCAAATGAGAATCCAGCACACAAG

AGTCAATTGGTGTGGATGGCATGTCATTCTGCAGCATTCGAGGATCTGAGAGTCTCAAGT

TTCATCAGAGGAACAAGAGTAGTCCCAAGAGGACAACTATCCACTAGAGGAGTTCAAATT

GCTTCAAATGAGAACATAGAAACAATGGACTCCAGCACTCTTGAACTGAGAAGCAGATAT

TGGGCTATAAGAACCAGGAGTGGAGGAAACACCAACCAACAGAGAGCATCTGCAGGACAA

ATCAGTGTACAGCCCACTTTCTCGGTACAGAGAAACCTTCCCTTCGAAAGAGCGACCATT

ATGGCGGCATTCACAGGGAACACTGAAGGCAGAACGTCCGACATGAGGACTGAAATCATA

AGAATGATGGAAAGTGCCAGACCAGAAGATGTGTCTTTCCAGGGGCGGGGAGTCTTCGAG

CTCTCGGACGAAAAGGCAACGAACCCGATCGTGCCTTCCTTTGACATGAGTAATGAAGGA

TCTTATTTCTTCGGAGACAATGCAGAGGAGTATGACAATTAAAGAAAAATACCCTTGTTT

CTACT-------------------------------------------------

>A_duck_Lao_961_2010_EPI335154

AGCAAAAGCAGGGTAGATAATCACTCACCGAGTGACATCAACATTATGGCGTCTCAAGGC

ACCAAACGATCTTATGAACAAATGGAAACTGGTGGAGAGCGCCAGAATGCTACTGAGATC

AGGGCATCTGTTGGAAGAATGGTTAGTGGCATTGGGAGGTTCTACATACAGATGTGCACA

GAACTCAAACTCAGTGACTATGAAGGGAGACTGATCCAGAACAGCATAACAATAGAGAGG

ATGGTACTTTCTGCATTTGATGAAAGAAGGAACAGGTACCTGGAAGAACACCCCAGTGCG

GGGAAGGATCCGAAGAAAACTGGAGGCCCAATTTATCGGAGGAGAGACGTAAAATGGGTG

AGGGAGCTGATTCTGTACGACAAAGAGGAGATCAGGAGGATTTGGCGTCAAGCGAACAAT

GGAGAGGACGCAACTGCTGGTCTTACCCACCTGATGATATGGCATTCTAATCTAAATGAT

GCCACATATCAGAGAACGAGAGCTCTCGTGCGTACTGGGATGGACCCCAGGATGTGCTCT

CTGATGCAAGGGTCAACTCTCCCGAGGAGATCTGGAGCTGCCGGTGCAGCAGTGAAGGGG

GTAGGGACAATGGTGATGGAGCTGATTCGGATGATAAAACGAGGGGTCAACGACCGGAAT

TTCTGGAGAGGCGAAAATGGAAGAAGAACTAGGATTGCATATGAGAGAATGTGCAACATC

CTCAAAGGGAAATTCCAAACAGCGGCACAAAGAGCAATGATGGATCAAGTGCGAGAGAGC

AGAAATCCTGGGAATGCTGAAATTGAAGATCTCATTTTTCTGGCACGGTCTGCACTCATC

CTGAGAGGATCAGTGGCCCATAAGTCCTGCTTGCCTGCTTGTGTATACGGACTTGCAGTG

GCCAGTGGATATGACTTTGAGAGAGAAGGGTACTCTCTGGTTGGAATAGATCCTTTCCGT

CTGCTTCAAAACAGCCAGGTCTTTAGTCTCATTAGGCCAAACGAGAATCCAGCACATAAG

AGTCAATTAGTGTGGATGGCATGTCACTCTGCAGCATTTGAGGACCTTAGAGTCTCAAGC

TTCATCAGAGGGACAAGAGTGGTCCCAAGAGGACAGCTATCCACCAGAGGGGTTCAAATT

GCTTCAAATGAGAACATGGAAGCAATGGACTCCAACACTCTTGAACTGAGGAGTAGATAT

TGGGCTATAAGAACCAGAAGCGGGGGAAACACCAACCAGCAGAGGGCATCTGCAGGGCAG

ATCAGCGTTCAGCCCACTTTCTCGGTACAGAGAAACCTTCCCTTCGAAAGAGCGACCATT

ATGGCAGCATTTACAGGGAATGCTGAGGGCAGAACGTCTGACATGAGGACTGAAATCATA

AGAATGATGGAAAGTGCCAGACCAGAAGATGTGTCATTCCAGGGGCGGGGAGTCTTCGAG

CTCTCGGACGAAAAGGCAACGAACCCGATCGTGCCTTCCTTTGACATGAATAATGAAGGA

TCTTATTTCTTCGGAGACAATGCAGAGGAGTATGACAATTAAAGAAAAATACCCTTGTTT

CTACT-------------------------------------------------

>A_duck_Lao_567_2010_EPI335178

AGCAAAAGCAGGGTAGATAATCACTCACCGAGTGACATCAACATCATGGCGTCTCAAGGC

ACCAAACGATCTTATGAACAGATGGAAACTGGTGGAGAACGCCAGAATGCCACTGAGATC

AGGGCATCTGTTGGAAGAATGGTTAGTGGAATTGGGAGGTTCTACATACAGATGTGCACA

GAGCTCAAACTCAGTGACTATGAAGGGAGGCTGATCCAGAACAGCATAACAATAGAGAGA

ATGGTGCTCTCTGCATTTGATGAAAGAAGGAATAGATACCTGGAAGAACACCCCAGTGCG

GGGAAAGACCCGAAGAAAACTGGAGGTCCAATTTATCGGAGGAGAGACGGAAAATGGGTG

CGGGAGCTGATTCTGTACGACAAAGAGGAGATCAGAAGGATTTGGCGTCAGGCGAACAAT

GGAGAGGACGCGACTGCTGGTCTTACCCACCTGATGATATGGCATTCCAATCTAAATGAT

GCCACATATCAGAGAACGAGAGCTCTCGTACGTACTGGAATGGACCCCAGGATGTGCTCT

CTGATGCAAGGGTCTACTCTCCCAAGGAGGTCTGGAGCTGCCGGTGCAGCAGTGAAGGGG

GTAGGAACAATGGTGATGGAGCTGATTAGGATGATAAAACGAGGGATCAACGACCGGAAT

TTCTGGAGAGGCGAAAATGGAAGAAGAACAAGGATTGCATATGAGAGAATGTGCAACATC

CTCAAAGGGAAATTCCAAACAGCAGCACAAAGAGCAATGATGGATCAAGTGCGAGAGAGC

AGAAATCCTGGGAATGCTGAAATTGAAGATCTCATTTTCCTGGCACGGTCTGCACTCATC

CTGAGAGGGTCAGTGGCCCATAAGTCCTGCTTGCCTGCTTGCGTGTATGGACTTGCAGTG

GCAAGTGGATATGACTTTGAGAGAGAAGGGTACTCTCTGGTTGGAATAGATCCTTTCCGT

CTGCTTCAAAACAGCCAGGTCTTTAGTCTCATTAGACCAAATGAGAATCCAGCACATAAG

AGTCAATTAGTGTGGATGGCTTGCCACTCTGCAGCATTTGAGGACCTTAGAGTCTCAAGT

TTTATCAGAGGAACAAGAGTGGTTCCAAGAGGGCAGCTATCCACCAGAGGGGTTCAAATT

GCTTCAAATGAGAACATGGAAACAATGGAGTCCAACACTCTTGAATTGAGAAGTAGATAT

TGGGCAATAAGAACCAGAAGTGGAGGAAACACCAACCAGCAGAGGGCTTCTGCAGGACAG

ATCAGCGTTCAGCCCACTTTCTCGGTACAGAGAAACCTTCCTTTCGAAAGAGCGACCATT

ATGGCAGCATTTGCAGGAAATACTGAGGGCAGAACGTCTGACATGAGGACTGAAATCATA

AAAATGATGGAAAGTGCCAGACCAGAAGATGTGTCATTCCAGGGGCGGGGAGTCTTCGAG

TTCTCGGACGAAAAGGCAACGAACCCGATCGTGCCTTCCTTTGACATGAATAATGAAGGA

TCTTATTTCTTCGGAGACAATGCAGAGGAGTATGACAATTAAAGAAAAATACCCTTGTTT

CTACT-------------------------------------------------

>A_breeder_duck_Korea_Gochang1_2014_EPI509699

---------------GATAATCACTCACTGAGTGACATCAACATCATGGCGTCTCAAGGC

ACCAAACGATCTTATGAACAGATGGAAACTGGTGGAGAACGCCAGAATGCCACTGAAATC

AGAGCATCTGTTGGAAGAATGGTTGGTGGAATTGGGAGGTTTTACATACAGATGTGCACT

GAACTCAAACTCAGCGATTATGAGGGGAGACTGATCCAGAACAGCATAACAATAGAAAGA

ATGGTTCTCTCTGCATTTGATGAAAGGAGGAACAAGTACCTGGAAGAACATCCCAGTGCG

GGGAAGGACCCAAAGAAAACTGGAGGTCCAATCTACAGAAGAAGAGACGGAAAGTGGGTG

AGAGAGCTGATTCTGTATGACAAAGAAGAGATCAGGAGGATCTGGCGTCAAGCAAATAAT

GGAGAAGATGCAACTGCTGGTCTCACTCATCTGATGATCTGGCACTCCAACCTGAATGAT

GCCACATACCAGAGAACAAGAGCTCTCGTGCGCACTGGAATGGATCCCAGAATGTGCTCT

CTGATGCAAGGATCAACTCTCCCAAGGAGGTCTGGAGCTGCTGGTGCAGCAGTAAAAGGG

GTCGGAACAATGGTAATGGAATTGATTCGGATGATAAAGCGAGGGATTAATGATCGGAAT

TTCTGGAGAGGCGAAAATGGACGAAGGACAAGGATTGCCTATGAGAGAATGTGCAACATC

CTCAAAGGAAAATTTCAAACAGCAGCACAAAGAGCAATGATGGATCAAGTGCGAGAAAGC

AGGAATCCTGGGAATGCTGAAATTGAAGATCTCATCTTTCTGGCACGGTCTGCACTCATC

CTGAGAGGATCGGTGGCCCATAAGTCCTGCTTGCCTGCCTGTGTTTACGGACTTGCTGTG

GCCAGTGGATATGACTTTGAGAGAGAAGGGTACTCTCTGGTCGGAATAGACCCTTTCCGT

CTGCTTCAAAACAGCCAGGTCTTCAGTCTCATTAGACCAAATGAAAACCCAGCACATAAA

AGCCAATTGGTATGGATGGCATGCCATTCAGCAGCGTTTGAGGACCTGAGGGTATCAAGT

TTCATCAGAGGGACAAGAGTGGTCCCAAGAGGACAATTATCCACCAGAGGAGTTCAAATT

GCATCTAATGAAAACATGGAAACAATGGACTCCAGCACTCTTGAATTGAGGAGCAGATAC

TGGGCTATAAGAACCAGGAGTGGGGGAACCACCAACCAACAGAGAGCTTCTGCAGGACAA

ATCAGCGTACAACCTACCTTCTCAGTACAGAGAAATCTTCCCTTCGAAAGAGCGACCATC

ATGGCGGCATTTACAGGGAACACTGAAGGCAGGACATCTGACATGAGGACTGAAATCATA

AGAATGATGGAAAGTGCCAAACCAGAAGATGTGTCCTTCCAGGGGCGGGGAGTCTTCGAG

CTCTCGGACGAAAAGGCAACGAACCCGATCGTGCCTTCCTTTGACATGAGTAACGAAGGA

TCTTATTTCTTCGGAGACAATGCAGAGGAGTATGACAATTAAAGAAAAA-----------

------------------------------------------------------

>A_broiler_duck_Korea_Buan2_2014_EPI509705

---------------GATAATCACTCACTGAGTGACATCAACATCATGGCGTCTCAAGGC

ACCAAACGATCTTATGAACAGATGGAAACTGGTGGAGAACGCCAGAATGCCACTGAAATC

AGAGCATCTGTTGGAAGAATGGTTGGTGGAATTGGAAGGTTTTATATACAGATGTGCACT

GAACTCAAACTCAGCGATTATGAGGGGAGACTGATCCAGAACAGCATAACAATAGAAAGA

ATGGTTCTCTCTGCATTTGATGAAAGGAGGAACAAGTACCTGGAAGAACATCCCAGTGCG

GGGAAGGACCCAAAGAAAACTGGAGGTCCAATCTACAGAAGAAGAGACGGAAAGTGGATG

AGGGAGCTGATTCTGTATGACAAAGAAGAGATCAGAAGAATCTGGCGTCAAGCAAATAAT

GGAGAAGATGCAACTGCTGGTCTCACCCATCTGATGATCTGGCACTCCAACCTGAATGAT

GCCACATATCAGAGGACAAGGGCTCTCGTGCGCACTGGAATGGATCCCAGAATGTGCTCT

CTGATGCAAGGATCAACTCTCCCAAGAAGGTCTGGAGCTGCTGGTGCAGCAGTAAAAGGG

GTCGGAACAATGGTAATGGAATTGATTCGAATGATAAAGCGAGGGATTAATGATCGGAAT

TTCTGGAGAGGCGAAAATGGAAGAAGGACAAGGATTGCCTATGAGAGAATGTGCAACATC

CTCAAAGGGAAATTTCAAACAGCAGCACAAAGAGCAATGATGGATCAAGTGCGAGAAAGC

AGGAATCCTGGGAATGCTGAAATTGAAGATCTCATTTTTCTGGCACGGTCTGCACTCATC

CTGAGAGGATCAGTGGCCCACAAGTCTTGTCTGCCTGCTTGTGTTTACGGACTTGCTGTG

GCCAGTGGATATGACTTTGAGAGAGAAGGATACTCTCTGGTTGGAATAGACCCTTTCCGT

CTGCTTCAAAACAGCCAGGTCTTCAGTCTCATTAGACCAAATGAAAACCCAGCACATAAA

AGCCAGTTGGTATGGATGGCATGCCATTCAGCAGCGTTTGAGGACCTGAGGGTATCAAGT

TTCATCAGAGGGACAAGAGTGGTCCCAAGAGGACAACTATCCACCAGAGGAGTTCAAATT

GCATCAAATGAAAACATGGAAACAATGGACTCCAGCACTCTTGAATTGAGAAGCAGATAC

TGGGCTATAAGAACCAGGAGTGGAGGAAACACCAACCAACAGAGAGCTTCTGCAGGACAA

ATCAGCGTACAACCCACCTTCTCAGTACAGAGAAATCTTCCCTTTGAAAGAGCGACCATC

ATGGCGGCATTTACAGGGAACACTGAAGGCAGGACCTCTGACATGAGGACTGAAATCATA

AGAATGATGGAAAGTGCCAAACCAGAAGATGTGTCCTTCCAGGGGCGGGGAGTCTTCGAG

CTCTCGGACGAAAAGGCAACGAACCCGATCGTGCCTTCCTTTGACATGAGCAACGAAGGA

TCTTATTTCTTCGGAGACAGTGCAGAGGAGTATGACAATTAAAG----------------

------------------------------------------------------

>A_goose_Taiwan_TNO15_2015_EPI690745

---------------------------------------------ATGGCGTCTCAAGGC

ACCAAACGATCTTATGAACAAATGGAAACTGGTGGAGAACGCCAGAATGCCACTGAAATC

AGAGCATCTGTTGGAAGGATGGTTGGTGGAATTGGGAGGTTTTACATACAGATGTGCACT

GAACTCAAACTCAGCGACTATGAAGGGAGGCTGATCCAGAACAGCATAACAATAGAGAGA

ATGGTTCTCTCTGCATTTGATGAAAGGAGGAACAAATACCTGGAAGAACATCCCAGTGCT

GGGAAGGATCCGAAGAAAACTGGAGGTCCAATTTATCGAAGAAGGGACGGGAAATGGATG

AGAGAGCTGATTCTGTATGACAAAGAGGAGATCAGGAGAATCTGGCGTCAAGCGAACAAT

GGAGAGGACGCAACTGCTGGTCTCACTCACCTGATGATCTGGCATTCCAATCTAAATGAT

GCCACATACCAGAGAACCAGAGCTCTCGTGCGCACTGGGATGGACCCCAGAATGTGCTCT

CTGATGCAAGGATCAACTCTCCCGAGGAGATCTGGAGCTGCTGGCGCAGCAGTAAAGGGT

GTCGGGACGATGGTGATGGAACTAATTCGGATGATAAAACGAGGGATTAATGATCGGAAT

TTCTGGAGAGGCGAAAATGGACGGAGAACAAGGATTGCATATGAGAGGATGTGCAACATC

CTCAAAGGGAAATTCCAAACAGCAGCACAAAGAGCAATGATGGACCAAGTGCGAGAAAGC

AGAAATCCTGGGAATGCTGAAATTGAAGATCTCATCTTCCTGGCACGGTCAGCACTCATC

CTGAGAGGATCAGTGGCCCATAAGTCTTGCCTTCCTGCTTGTGTGTACGGGCTTGCTGTG

GCCAGTGGATATGACTTTGAGAGAGAAGGGTACTCTCTAGTTGGAATAGATCCTTTCCGT

CTGCTTCAGAACAGCCAGGTCTTCAGTCTCATCAGACCAAATGAGAACCCAGCACACAAG

AGTCAATTGGTGTGGATGGCATGCCATTCTGCAGCATTTGAGGACCTGAGAGTCTCAAGT

TTCATCAGAGGGACAAGAGTGGTCCCAAGAGGACAACTATCCACCAGAGGAGTTCAAATT

GCTTCAAATGAGAACATGGAAACAATGGACTCCAGCACTCTTGAACTGAGGAGCAGGTAT

TGGGCTATAAGGACCAGGAGTGGAGGAAACACCAATCAACAGAGAGCATCTGCAGGACAG

ATCAGTGTACAGCCTACTTTCTCAGTACAGAGAAATCTTCCCTTCGAAAGAGCAACCATC

ATGGCGGCGTTCACAGGAAATACTGAAGGCAGAACATCTGACATGAGGACTGAAATCATA

AGAATGATGGAAAGTGCCAGACCAGAAGATGTGTCCTTCCAGGGGCGGGGAGTCTTCGAG

CTCTCGGACGAAAAGGCAACGAACCCGATCGTGCCTTCCTTTGACATGAGTAATGAAGGA

TCTTATTTCTTCGGAGACAATGCAGAGGAGTATGACAATTAA------------------

------------------------------------------------------

>A_wigeon_Sakha_1_2014_EPI1201485

AGCAAAAGCAGGGTAGATAATCACTCACTGAGTGACATCAACATCATGGCGTCTCAAGGC

ACCAAACGATCTTATGAACAGATGGAAACTGGTGGAGAACGCCAGAATGCCACTGAAATC

AGAGCATCTGTTGGAAGAATGGTTGGTGGAATTGGAAGGTTTTATATACAGATGTGCACT

GAACTCAAACTCAGTAATTATGAGGGGAGACTGATCCAGAACAGCATAACAATAGAAAGA

ATGGTTCTCTCTGCATTTGATGAAAGGAGGAACAAGTACCTGGAAGAACATCCCAGTGCG

GGGAAGGACCCAAAGAAAACTGGAGGTCCAATCTACAGAAGAAGAGACGGAAAGTGGATG

AGGGAGCTGATTCTGTATGACAAAGAAGAGATCAGAAGGATCTGGCGTCAAGCAAATAAT

GGAGAAGATGCAACTGCTGGTCTCACCCATCTGATGATCTGGCACTCCAACCTGAATGAT

GCCACATATCAGAGGACAAGGGCTCTCGTGCGCACTGGAATGGATCCCAGAATGTGCTCT

CTGATGCAAGGATCAACTCTCCCAAGAAGGTCTGGAGCTGCTGGTGCAGCAGTAAAAGGG

GTCGGAACAATGGTAATGGAATTGATTCGAATGATAAAGCGAGGAATTAATGATCGGAAT

TTCTGGAGAGGCGAAAATGGAAGAAGGACAAGGATTGCCTATGAGAGAATGTGCAACATC

CTCAAAGGGAAATTTCAAACAGCAGCACAAAGAGCAATGATGGATCAAGTGCGAGAAAGC

AGGAATCCTGGGAATGCTGAAATTGAAGATCTCATTTTTCTGGCACGGTCTGCACTCATC

CTGAGAGGATCAGTGGCCCACAAGTCTTGTCTGCCTGCTTGTGTTTACGGACTTGCTGTG

GCCAGTGGATATGACTTTGAGAGAGAAGGATACTCTCTGGTTGGAATAGACCCTTTCCGT

CTGCTTCAAAACAGCCAGGTCTTCAGTCTCATTAGACCAAATGAAAACCCAGCACATAAA

AGTCAGTTGGTATGGATGGCATGCCATTCAGCAGCGTTTGAGGACCTGAGGGTATCAAGT

TTCATCAGAGGGACAAGAGTGGTCCCAAGAGGACAACTATCCACCAGAGGAGTTCAAATT

GCATCAAATGAAAACATGGAAACAATGGACTCCAGCACTCTTGAATTGAGAAGCAGATAC

TGGGCTATAAGAACCAGGAGTGGAGGAAACACCAACCAACAGAGAGCTTCTGCAGGACAA

ATCAGCGTACAACCCACCTTCTCAGTACAGAGAAATCTTCCCTTTGAAAGAGCGACCATC

ATGGCGGCATTTACAGGGAACACTGAAGGCAGGACCTCTGACATGAGGACTGAGATCATA

AGAATGATGGAAAGTGCCAAACCAGAAGATGTGTCCTTCCAGGGGCGGGGAGTCTTCGAG

CTCTCGGACGAAAAGGCAACGAACCCGATCGTGCCTTCCTTTGACATGAGCAACGAAGGA

TCTTATTTCTTCGGAGACAGTGCAGAGGAGTATGACAATTAAAGAAAAATACCCTTGTTT

CTACT-------------------------------------------------

>A_duck_Nigeria_SK28T_19VIR8424-2_2019_EPI1777105

AGCAAAAGCAGGGTAGATAATCACTCACTGAGTGACATCAACACCATGGCGTCTCAAGGC

ACCAAACGATCTTATGAACAGATGGAAACTGGTGGAGAGCGCCAGAATGCCACTGAGATC

AGAGCATCTGTTGGAAGAATGGTTAGTGGAATTGGGAGGTTCTACATACAGATGTGCACT

GAGCTCAAACTCAGCGACTATGAAGGAAGGCTGATCCAGAACAGCATAACAATAGAGAGA

ATGATTCTCTCCGCATTTGATGAAAGGAGGAACAAATATCTGGAAGAACATCCCAGTGCG

GGGAAAGACCCGAAGAAAACTGGAGGTCCAATTTATCGAAGGAGAGATGGGAAATGGGTG

AGAGAGCTGATCCTGTATGACAAAGAGGAGATCAGGAGGATCTGGCGTCAAGCGAACAAT

GGAGAAGATGCAACTGCTGGTCTCACTCACCTGATGATCTGGCATTCCAATCTAAATGAT

GCCACATACCAGAGGACAAGAGCTCTCGTGCGTACTGGAATGGACCCTAGGATGTGCTCT

CTCATGCAAGGATCAACTCTCCCAAGGAGGTCTGGAGCTGCTGGTGCAGCAGTAAAGGGA

GTCGGGACGATGGTGATGGAACTAATTCGGATGATAAAGCGAGGAATTAATGATCGAAAC

TTCTGGAGAGGCGAGAATGGACGAAGGACAAGGATTGCATATGAGAGAATGTGCAACATT

CTCAAAGGGAAATTCCAAACAGCAGCACAAAGAGCAATGATGGATCAGGTGCGTGAAAGC

AGGAATCCTGGCAATGCTGAAATTGAAGATCTCATCTTTCTGGCAAGGTCTGCTCTCATC

CTGAGAGGATCAGTGGCCCACAAGTCCTGCTTGCCTGCTTGTGTGTACGGACTCGCTGTG

GCCAGTGGATACGACTTTGAGAGAGAAGGGTACTCTCTAGTTGGAATAGATCCTTTCCGT

CTGCTCCAAAACAGCCAGGTCTTCAGTCTCATTAGACCAAATGAGAATCCAGCACACAAG

AGCCAATTGGTGTGGATGGCATGTCACTCTGCAGCATTCGAGGATCTGAGGGTCTCAAGT

TTCATCAGAGGAACAAGAGTAGTCCCTAGAGGGCAACTATCCACTAGAGGAGTTCAAATT

GCTTCAAATGAGAACATGGAAACAATGGACTCCAGCACTCTTGAACTGAGAAGCAGATAT

TGGGCTATAAGAACCAGGAGTGGAGGAAACACCAACCAACAGAGAGCATCTGCAGGACAA

ATCAGTGTACAGCCCACTTTCTCAGTACAGAGAAACCTTCCCTTCGAAAGAGCGACCATT

ATGGCGGCATTCACAGGGAACACTGAGGGCAGGACGTCCGACATGAGGACTGAAATCATA

AGAATGATGGAAAGTGCCAGACCAGAAGATGTGTCTTTCCAGGGGCGGGGAGTCTTCGAG

CTCTCGGACGAAAAGGCAACGAACCCGATCGTGCCTTCCTTTGACATGAGTAATGAAGGA

TCTTATTTCTTCGGAGACAATGCAGAGGAGTACGACAATTAAAGAAAAATACCCTTGTTT

CTACT-------------------------------------------------

>A_mute_swan_Kazakhstan_1-267-20-B_2020_EPI1811585

---------------------------------------------ATGGCGTCTCAAGGC

ACCAAACGATCTTATGAACAGATGGAAACTGGTGGGGAGCGCCAGAATGCCACTGAGATT

AGAGCATCGGTTGGAAGAATGGTTGGTGGAATTGGGAGGTTCTACATACAGATGTGCACT

GAGCTCAAACTCAGCGACTATGAAGGAAGACTGATCCAGAACAGCATAACAATAGAGAGA

ATGGTTCTCTCCGCATTTGATGAAAGGAGAAACAAATATCTGGAAGAACATCCCAGTGCG

GGGAAAGACCCGAAGAAAACAGGAGGTCCAATTTATCGAAGGAGAGATGGGAAATGGGTG

AGAGAGCTGATCCTGTATGACAAAGAGGAGATCAGGAGGATCTGGCGCCAAGCGAATAAT

GGAGAAGACGCAACTGCTGGTCTCACTCACCTGATGATCTGGCATTCCAATCTAAATGAT

GCCACATACCAGAGGACAAGAGCCCTCGTGCGTACCGGTATGGACCCCAGGATGTGCTCC

CTCATGCAAGGGTCAACTCTCCCAAGGAGGTCTGGAGCTGCTGGTGCAGCAGTGAAGGGA

GTTGGAACGATGGTGATGGAACTAATTCGGATGATAAAGCGAGGAGTTAATGATCGAAAC

TTCTGGAGAGGCGAGAATGGACGGAGGACAAGGATTGCATTTGAGAGAATGTGCAATATT

CTCAAAGGGAAATTCCAAACAGCAGCACAAAGAGCAATGATGGATCAAGTGCGTGAAAGC

AGGAATCCTGGCAATGCTGAAATTGAAGATCTCATTTTTCTGGCGCGGTCTGCTCTCATT

CTGAGAGGATCAGTGGCCCATAAGTCCTGCTTGCCCGCTTGTGTATACGGGCTCGCTGTA

GCCAGTGGATACGACTTTGAGAGAGAAGGGTACTCTCTAGTTGGAATAGACCCTTTCCGA

CTGCTTCAAAACAGCCAGGTCTTTAGTCTCATTAGACCAAATGAGAATCCGGCACACAAG

AGTCAATTGGTGTGGATGGCATGTCATTCTGCAGCATTCGAGGATCTGAGAGTCTCAAGT

TTCATCAGAGGAACAAGAGTAGTCCCAAGAGGACAACTATCCACTAGAGGAGTTCAAATT

GCTTCAAATGAGAACATAGAAACAATGGACTCCAGCACTCTTGAACTGAGAAGCAGATAT

TGGGCTATAAGAACCAGGAGTGGAGGAAACACCAACCAACAGAGAGCATCTGCAGGACAA

ATCAGTGTACAGCCCACTTTCTCGGTACAGAGAAACCTTCCCTTCGAAAGAGCGACCATT

ATGGCGGCATTCACAGGGAACACTGAAGGCAGAACGTCCGACATGAGGACTGAAATCATA

AGAATGATGGAAAGTGCCAGACCAGAAGATGTGTCTTTCCAGGGGCGGGGAGTCTTCGAG

CTCTCGGACGAAAAGGCAACGAACCCGATCGTGCCTTCCTTTGACATGAGTAATGAAGGA

TCTTATTTCTTCGGAGACAATGCAGAGGAGTATGACAATTAA------------------

------------------------------------------------------

>A_domestic_goose_Kazakhstan_1-248_2-20-B_2020_EPI1811602

AGCAAAAGCAGGGTAGATAATCACTCACTGAGTGACATCAACATTATGGCGTCTCAAGGC

ACCAAACGATCTTATGAACAGATGGAAACTGGTGGGGAGCGCCAGAATGCCACTGAGATT

AGAGCATCGGTTGGAAGAATGGTTGGTGGAATTGGGAGGTTCTACATACAGATGTGCACT

GAGCTCAAACTCAGCGACTATGAAGGAAGACTGATCCAGAACAGCATAACAATAGAGAGA

ATGGTTCTCTCCGCATTTGATGAAAGGAGAAACAAATATCTGGAAGAACATCCCAGTGCG

GGGAAAGACCCGAAGAAAACAGGAGGTCCAATTTATCGAAGGAGAGATGGGAAATGGGTG

AGAGAGCTGATCCTGTATGACAAAGAGGAGATCAGGAGGATCTGGCGTCAAGCGAATAAT

GGAGAAGACGCAACTGCTGGTCTCACTCACCTGATGATCTGGCATTCCAATCTAAATGAT

GCCACATACCAGAGGACAAGAGCTCTCGTGCGTACCGGTATGGACCCCAGGATGTGCTCC

CTCATGCAAGGGTCAACTCTCCCAAGGAGGTCTGGAGCTGCTGGTGCAGCAGTGAAGGGA

GTTGGAACGATGGTGATGGAACTAATTCGGATGATAAAGCGAGGAGTTAATGATCGAAAC

TTCTGGAGAGGCGAGAATGGACGGAGGACAAGGATTGCATTTGAGAGAATGTGCAATATT

CTCAAAGGGAAATTCCAAACAGCAGCACAAAGAGCAATGATGGATCAAGTGCGTGAAAGC

AGGAATCCTGGCAATGCTGAAATTGAAGATCTCATTTTTCTGGCGCGGTCTGCTCTCATT

CTGAGAGGATCAGTGGCCCATAAGTCCTGCTTGCCCGCTTGTGTATACGGGCTCGCTGTA

GCCAGTGGATACGACTTTGAGAGAGAAGGGTACTCTCTAGTTGGAATAGACCCTTTCCGA

CTGCTTCAAAACAGCCAGGTCTTTAGTCTCATTAGACCAAATGAGAATCCAGCACACAAG

AGTCAATTGGTGTGGATGGCATGTCATTCTGCAGCATTCGAGGATCTGAGAGTCTCAAGT

TTCATCAGAGGAACAAGAGTAGTCCCAAGAGGACAACTATCCACTAGAGGAGTTCAAATT

GCTTCAAATGAGAACATAGAAACAATGGACTCCAGCACTCTTGAACTGAGAAGCAGATAT

TGGGCTATAAGAACCAGGAGTGGAGGAAACACCAACCAACAGAGAGCATCTGCAGGACAA

ATCAGTGTACAGCCCACTTTCTCGGTACAGAGAAACCTTCCCTTCGAAAGAGCGACCATT

ATGGCGGCATTCACAGGGAACACTGAAGGCAGAACGTCCGACATGAGGACTGAAATCATA

AGAATGATGGAAAGTGCCAGACCAGAAGATGTGTCTTTCCAGGGGCGGGGAGTCTTCGAG

CTCTCGGACGAAAAGGCAACGAACCCGATCGTGCCTTCCTTTGACATGAGTAATGAAGGA

TCTTATTTCTTCGGAGACAATGCAGAGGAGTATGACAATTAAAGAAAAATACCCTT----

------------------------------------------------------

>A_domestic_duck_Kazakhstan_1-274-20-B_2020_EPI1811612

---------------------------------------------ATGGCGTCTCAAGGC

ACCAAACGATCTTATGAACAGATGGAAACTAGTGGGGAGCGCCAGAATGCCACTGAGATT

AGAGCATCTGTTGGAAGAATGGTTGGTGGAATTGGGAGGTTCTACATACAGATGTGCACT

GAGCTCAAACTCAGCGACTATGAAGGAAGACTGATCCAGAACAGCATAACAATAGAGAGA

ATGGTTCTCTCCGCATTTGATGAAAGGAGAAACAAATATCTGGAAGAACATCCCAGTGCG

GGGAAAGACCCGAAGAAAACAGGAGGTCCAATTTATCGAAGGAGAGATGGGAAATGGGTG

AGAGAGCTGATCCTGTATGACAAAGAGGAGATCAGGAGGATCTGGCGTCAAGCAAATAAT

GGAGAAGACGCAACTGCTGGTCTCACTCACCTGATGATCTGGCATTCCAATCTAAATGAT

GCCACATACCAGAGGACAAGAGCTCTCGTGCGTACCGGTATGGACCCCAGGATGTGCTCC

CTCATGCAAGGGTCAACTCTCCCAAGGAGATCTGGAGCTGCTGGTGCAGCAGTGAAAGGA

GTTGGAACGATGGTGATGGAACTAATTCGGATGATAAAGCGAGGAGTTAATGATCGAAAC

TTCTGGAGAGGCGAGAATGGACGGAGGACAAGGATTGCATTTGAGAGAATGTGCAATATT

CTCAAAGGGAAATTCCAAACAGCAGCACAAAGAGCAATGATGGATCAAGTGCGTGAAAGC

AGGAATCCTGGCAATGCTGAAATTGAAGATCTCATTTTTCTGGCGCGGTCTGCTCTCATT

CTGAGAGGATCAGTGGCCCATAAGTCCTGCTTGCCCGCTTGTGTATACGGGCTCGCTGTA

GCCAGTGGATACGACTTTGAGAGAGAAGGGTACTCTCTAGTTGGAATAGACCCTTTCCGA

CTGCTTCAAAACAGCCAGGTCTTTAGTCTCATTAGACCAAATGAGAATCCAGCACACAAG

AGTCAATTGGTGTGGATGGCATGTCATTCTGCAGCATTCGAGGATCTGAGAGTCTCAAGT

TTCATCAGAGGAACAAGAGTAGTCCCAAGAGGACAACTATCCACTAGAGGAGTTCAAATT

GCTTCAAATGAGAACATAGAAACAATGGACTCCAGCACTCTTGAACTGAGAAGCAGATAT

TGGGCTATAAGAACCAGGAGTGGAGGAAACACCAACCAACAGAGAGCATCTGCAGGACAA

ATCAGTGTACAGCCCACTTTCTCGGTACAGAGAAACCTTCCCTTCGAAAGAGCGACCATT

ATGGCGGCATTCACAGGGAACACTGAAGGCAGAACGTCCGACATGAGGACTGAAATCATA

AGAATGATGGAAAGTGCCAGACCAGAAGATGTGTCTTTCCAGGGGCGGGGAGTCTTCGAG

CTCTCGGACGAAAAGGCAACGAACCCGATCGTGCCTTCCTTTGACATGAGTAATGAAGGA

TCTTATTTCTTCGGAGACAATGCAGAGGAGTATGACAATTAA------------------

------------------------------------------------------

>A_domestic_goose_Kazakhstan_1-242_2-20-B_2020_EPI1811620

--CAAAAGCAGGGTAGATAATCACTCACTGAGTGACATCAACATCATGGCGTCTCAAGGC

ACCAAACGATYTTATGAACAGATGGAAAYTAGTGGGGAGYGCCAGAATGCCACTGAGATT

AGAGCATCGGTTGGAAGAATGGTTGGTGGAATTGGGAGGTTCTACATACAGATGTGCACT

GAGCTCAAACTCAGCGACTATGAAGGAAGACTGATCCAGAACAGCATAACAATAGAGAGA

ATGGTTCTCTCCGCATTTGATGAAAGGAGAAACAAATATCTGGAAGAACATCCCAGTGCG

GGGAAAGACCCGAAGAAAACAGGAGGTCCAATTTATCGAAGGAGAGATGGGAAATGGGTG

AGAGAGCTGATCCTGTATGACAAAGAGGAGATCAGGAGGATCTGGCGTCAAGCGAATAAT

GGAGAAGACGCAACTGCTGGTCTCACTCACCTGATGATCTGGCATTCCAATCTAAATGAT

GCCACATACCAGAGGACAAGAGCTCTCGTGCGTACCGGTATGGACCCCAGGATGTGCTCC

CTCATGCAAGGGTCAACTCTCCCAAGGAGGTCTGGAGCTGCTGGTGCAGCAGTGAAGGGA

GTTGGAACGATGGTGATGGAACTAATTCGGATGATAAAGCGAGGAGTTAATGATCGAAAC

TTCTGGAGAGGCGAGAATGGACGGAGGACAAGGATTGCATTTGAGAGAATGTGCAATATT

CTCAAAGGGAAATTCCAAACAGCAGCACAAAGAGCAATGATGGATCAAGTGCGTGAAAGC

AGGAATCCTGGCAATGCTGAAATTGAAGATCTCATTTTTCTGGCGCGGTCTGCTCTCATT

CTGAGAGGATCAGTGGCCCATAAGTCCTGCTTGCCCGCTTGTGTATACGGGCTCGCTGTA

GCCAGTGGATACGACTTTGAGAGAGAAGGGTACTCTCTAGTTGGAATAGACCCTTTCCGA

CTGCTTCAAAACAGCCAGGTCTTTAGTCTCATTAGACCAAATGAGAATCCAGCACACAAG

AGTCAATTGGTGTGGATGGCATGTCATTCTGCAGCATTCGAGGATCTGAGAGTCTCAAGT

TTCATCAGAGGAACAAGAGTAGTCCCAAGAGGACAACTATCCACTAGAGGAGTTCAAATT

GCTTCAAATGAGAACATAGAAACAATGGACTCCAGCACTCTTGAACTGAGAAGCAGATAT

TGGGCTATAAGAACCAGGAGTGGAGGAAACACCAACCAACAGAGAGCATCTGCAGGACAA

ATCAGTGTACAGCCCACTTTCTCGGTACAGAGAAACCTTCCCTTTGAAAGAGCGACCATT

ATGGCGGCATTCACAGGGAACACTGAAGGCAGAACGTCCGACATGAGGACTGAAATCATA

AGAATGATGGAAAGTGCCAGACCAGAAGATGTGTCTTTCCAGGGGCGGGGAGTCTTCGAG

CTCTCGGACGAAAAGGCAACGAACCCGATCGTGCCTTCCTTTGACATGAGTAATGAAGGA

TCTTATTTCTTCGGAGACAATGCAGAGGAGTATGACAATTAAAGAAAAATACCCTT----

------------------------------------------------------

>A_chicken_Iraq_1_2020_EPI1811629

-----------------------------------CATCAACATCATGGCGTCTCAAGGC

ACCAAACGATCTTATGAACAGATGGAAACTGGTGGGGAGCGCCAGAATGCCACTGAGATC

AGAGCATCGGTTGGAAGAATGGTTGGTGGAATTGGGAGGTTCTACATACAGATGTGCACT

GAGCTCAAACTCAGCGACTATGAAGGAAGACTGATCCAGAACAGCATAACAATAGAGAGA

ATGGTTCTCTCTGCATTTGATGAAAGGAGAAACAAATATCTGGAAGAACATCCCAGTGCG

GGGAAAGACCCGAAGAAAACAGGAGGTCCAATTTATCGAAGGAGAGATGGGAAATGGGTG

AGAGAGCTGATCCTGTATGACAAAGAGGAGATCAGGAGGATCTGGCGTCAAGCGAACAAT

GGAGAAGACGCAACTGCTGGTCTCACTCACCTGATGATCTGGCATTCCAATCTAAATGAT

GCCACATACCAGAGGACAAGAGCTCTCGTGCGTACCGGTATGGACCCCAGGATGTGCTCC

CTCATGCAAGGGTCAACTCTCCCAAGAAGGTCTGGAGCTGCTGGTGCAGCAGTGAAGGGA

GTCGGGACGATGGTGATGGAATTAATTCGGATGATAAAGCGAGGAGTTAATGATCGAAAC

TTCTGGAGAGGCGAGAATGGGCGGAGGACAAGGATTGCATTTGAGAGAATGTGCAACATT

CTCAAAGGGAAATTCCAAACAGCAGCACAAAGAGCAATGATGGATCAAGTGCGTGAAAGC

AGGAATCCTGGCAATGCTGAAATTGAAGATCTCATTTTTCTGGCGCGGTCTGCTCTCATT

CTGAGAGGATCAGTGGCCCATAAGTCCTGCTTGCCCGCTTGTGTATACGGGCTCGCTGTA

GCCAGTGGATACGACTTTGAGAGAGAAGGGTACTCTCTAGTTGGAATAGACCCTTTCCGA

CTGCTTCAAAACAGCCAGGTCTTTAGTCTCATTAGACCAAATGAGAATCCAGCACACAAG

AGTCAATTGGTGTGGATGGCATGTCATTCTGCAGCATTCGAGGATCTGAGAGTCTCAAGT

TTCATCAGAGGAACAAGAGTAGTCCCAAGAGGACAACTATCCACTAGAGGAGTTCAAATT

GCTTCAAATGAGAACATGGAAACAATGGACTCCAGCACTCTTGAACTGAGAAGCAGATAT

TGGGCTATAAGAACCAGGAGTGGAGGAAACACCAACCAACAGAGAGCATCTGCAGGACAA

ATCAGTGTACAGCCCACTTTCTCGGTACAGAGAAACCTTCCCTTCGAAAGAGCGACCGTT

ATGGCGGCATTCACAGGGAACACTGAGGGCAGAACGTCCGACATGAGGACTGAAATCATA

AGAATGATGGAAAGTGCCAGACCAGAAGATGTGTCTTTCCAGGGGCGGGGAGTCTTCGAG

CTCTCGGACGAAAAGGCAACGAACCCGATCGTGCCTTCCTTTGACATGAGTAATGAAGGA

TCTTATTTCTTCGGAGACAATGCAGAGGAGTATGACAATTAA------------------

------------------------------------------------------

>A_whooper_swan_Inner_Mongolia_w1-1_2020_EPI1811645

---------------------------------------------ATGGCGTCTCAAGGC

ACCAAACGATCTTATGAACAGATGGAAACTAGTGGGGAGCGCCAGAATGCCACTGAGATT

AGAGCATCGGTTGGAAGAATGGTTGGTGGAATCGGGAGGTTCTACATACAGATGTGCACT

GAGCTCAAACTCAGCGACTATGAAGGAAGACTGATCCAGAACAGCATAACAATAGAGAGA

ATGGTTCTCTCCGCATTTGATGAAAGGAGAAACAAATATCTGGAAGAACATCCCAGTGCG

GGGAAAGACCCGAAGAAAACAGGAGGTCCAATTTATCGAAGGAGAGATGGGAAATGGGTG

AGAGAGCTGATCCTGTATGACAAAGAGGAGATCAGGAGGATCTGGCGTCAAGCGAACAAT

GGAGAAGACGCAACTGCTGGTCTCACTCACCTGATGATCTGGCATTCCAATCTAAATGAT

GCCACATACCAGAGGACAAGAGCTCTCGTGCGTACCGGTATGGACCCCAGGATGTGCTCC

CTCATGCAAGGGTCAACTCTCCCAAGGAGGTCTGGAGCTGCTGGTGCAGCAGTGAAGGGA

GTTGGAACGATGGTGATGGAACTAATTCGGATGATAAAGCGAGGAGTTAATGATCGAAAC

TTCTGGAGAGGCGAGAATGGACGGAGGACAAGGATTGCATTTGAGAGAATGTGCAATATT

CTCAAAGGGAAATTCCAAACAGCAGCACAAAGAGCAATGATGGATCAAGTGCGTGAAAGC

AGGAATCCTGGCAATGCTGAAATTGAAGATCTCATTTTTCTGGCGCGGTCTGCTCTCATT

CTGAGAGGATCAGTGGCCCATAAGTCCTGCTTGCCCGCTTGTGTATACGGGCTCGCTGTA

GCCAGTGGATACGACTTTGAGAGAGAAGGGTACTCTCTAGTTGGAATAGACCCTTTCCGA

CTGCTTCAAAACAGCCAGGTCTTTAGTCTCATTAGACCAAATGAGAATCCAGCACACAAG

AGTCAATTGGTGTGGATGGCATGTCATTCTGCAGCATTCGAGGATCTGAGAGTCTCAAGT

TTCATCAGAGGAACAAGAGTAGTCCCAAGAGGACAACTATCCACTAGAGGAGTTCAAATT

GCTTCAAATGAGAACATAGAAACAATGGACTCCAGCACTCTTGAACTGAGAAGCAGATAC

TGGGCTATAAGAACCAGGAGTGGAGGAAACACCAACCAACAGAGAGCATCTGCAGGACAA

ATCAGTGTACAGCCCACTTTCTCGGTACAGAGAAACCTTCCCTTCGAAAGAGCGACCATT

ATGGCGGCATTCACAGGGAACACTGAAGGCAGAACGTCCGACATGAGGACTGAAATCATA

AGAATGATGGAAAGTGCCAGACCAGAAGATGTGTCTTTCCAGGGGCGGGGAGTCTTCGAG

ATCTCGGACGAAAAGGCAACGAACCCGATCGTGCCTTCCTTTGACATGAGTAATGAAGGA

TCTTATTTCTTCGGAGACAATGCAGAGGAGTATGACAATTAA------------------

------------------------------------------------------

>A_mute_swan_Inner_Mongolia_w2-1_2020_EPI1811653

---------------------------------------------ATGGCGTCTCAAGGC

ACCAAACGATCTTATGAACAGATGGAAACTAGTGGGGAGCGCCAGAATGCCACTGAGATT

AGAGCATCGGTTGGAAGAATGGTTGGTGGAATCGGGAGGTTCTACATACAGATGTGCACT

GAGCTCAAACTCAGCGACTATGAAGGAAGACTGATCCAGAACAGCATAACAATAGAGAGA

ATGGTTCTCTCCGCATTTGATGAAAGGAGAAACAAATATCTGGAAGAACATCCCAGTGCG

GGGAAAGACCCGAAGAAAACAGGAGGTCCAATTTATCGAAGGAGAGATGGGAAATGGGTG

AGAGAGCTGATCCTGTATGACAAAGAGGAGATCAGGAGGATCTGGCGTCAAGCGAACAAT

GGAGAAGACGCAACTGCTGGTCTCACTCACCTGATGATCTGGCATTCCAATCTAAATGAT

GCCACATACCAGAGGACAAGAGCTCTCGTGCGTACCGGTATGGACCCCAGGATGTGCTCC

CTCATGCAAGGGTCAACTCTCCCAAGGAGGTCTGGAGCTGCTGGTGCAGCAGTGAAGGGA

GTTGGAACGATGGTGATGGAACTAATTCGGATGATAAAGCGAGGAGTTAATGATCGAAAC

TTCTGGAGAGGCGAGAATGGACGGAGGACAAGGATTGCATTTGAGAGAATGTGCAATATT

CTCAAAGGGAAATTCCAAACAGCAGCACAAAGAGCAATGATGGATCAAGTGCGTGAAAGC

AGGAATCCTGGCAATGCTGAAATTGAAGATCTCATTTTTCTGGCGCGGTCTGCTCTCATT

CTGAGAGGATCAGTGGCCCATAAGTCCTGCTTGCCCGCTTGTGTATACGGGCTCGCTGTA

GCCAGTGGATACGACTTTGAGAGAGAAGGGTACTCTCTAGTTGGAATAGACCCTTTCCGA

CTGCTTCAAAACAGCCAGGTCTTTAGTCTCATTAGACCAAATGAGAATCCAGCACACAAG

AGTCAATTGGTGTGGATGGCATGTCATTCTGCAGCATTCGAGGATCTGAGAGTCTCAAGT

TTCATCAGAGGAACAAGAGTAGTCCCAAGAGGACAACTATCCACTAGAGGAGTTCAAATT

GCTTCAAATGAGAACATAGAAACAATGGACTCCAGCACTCTTGAACTGAGAAGCAGATAC

TGGGCTATAAGAACCAGGAGTGGAGGAAACACCAACCAACAGAGAGCATCTGCAGGACAA

ATCAGTGTACAGCCCACTTTCTCGGTACAGAGAAACCTTCCCTTCGAAAGAGCGACCATT

ATGGCGGCATTCACAGGGAACACTGAAGGCAGAACGTCCGACATGAGGACTGAAATCATA

AGAATGATGGAAAGTGCCAGACCAGAAGATGTGTCTTTCCAGGGGCGGGGAGTCTTCGAG

ATCTCGGACGAAAAGGCAACGAACCCGATCGTGCCTTCCTTTGACATGAGTAATGAAGGA

TCTTATTTCTTCGGAGACAATGCAGAGGAGTATGACAATTAA------------------

------------------------------------------------------

>A_goose_Russian_Federation_Kurgan_1345-25_2020_EPI1811681

------------GTAGATAATCACTCACTGAGTGACATCAACATCATGGCGTCTCAAGGC

ACCAAACGATCTTATGAACAGATGGAAACTGGTGGGGAGCGCCAGAATGCCACTGAGATT

AGAGCATCGGTTGGAAGAATGGTTGGTGGAATTGGGAGGTTCTACATACAGATGTGCACT

GAGCTCAAACTCAGCGACTATGAAGGAAGACTGATCCAGAACAGCATAACAATAGAGAGA

ATGGTTCTCTCTGCATTTGATGAAAGGAGAAACAAATATCTGGAAGAACATCCCAGTGCG

GGGAAAGACCCGAAGAAAACAGGAGGTCCAATTTATCGAAGGAGAGATGGGAAATGGGTG

AGAGAGCTGATCCTGTATGACAAAGAGGAGATCAGGAGGATCTGGCGTCAAGCGAATAAT

GGAGAAGACGCAACTGCTGGTCTCACTCACCTGATGATCTGGCATTCCAATCTAAATGAT

GCCACATACCAGAGGACAAGAGCTCTCGTACGTACCGGTATGGACCCCAGGATGTGCTCC

CTCATGCAAGGGTCAACTCTCCCAAGGAGGTCTGGAGCTGCTGGTGCAGCAGTGAAGGGA

GTTGGAACGATGGTGATGGAACTAATTCGGATGATAAAGCGAGGAGTTAATGATCGAAAC

TTCTGGAGAGGCGAGAATGGACGGAGGACAAGGATTGCATTTGAGAGAATGTGCAATATT

CTCAAAGGGAAATTCCAAACAGCAGCACAAAGAGCAATGATGGATCAAGTGCGTGAAAGC

AGGAATCCTGGCAATGCTGAAATTGAAGATCTCATTTTTCTGGCGCGGTCTGCTCTCATT

CTGAGAGGATCAGTGGCCCATAAGTCCTGCTTGCCCGCTTGTGTATACGGGCTCGCTGTA

GCCAGTGGATACGACTTTGAGAGAGAAGGGTACTCTCTAGTTGGAATAGACCCTTTCCGA

CTGCTTCAAAACAGCCAGGTCTTTAGTCTCATTAGACCAAATGAGAATCCAGCACACAAG

AGTCAATTGGTGTGGATGGCATGTCATTCTGCAGCATTCGAGGATCTGAGAGTCTCAAGT

TTCATCAGAGGAACAAGAGTAGTCCCAAGAGGACAACTATCCACTAGAGGAGTTCAAATT

GCTTCAAATGAGAACATAGAAACAATGGACTCCAGCACTCTTGAACTGAGAAGCAGATAT

TGGGCTATAAGAACCAGGAGTGGAGGAAACACCAACCAACAGAGAGCATCTGCAGGACAA

ATCAGTGTACAGCCCACTTTCTCGGTACAGAGAAACCTTCCCTTCGAAAGAGCGACCATT

ATGGCGGCATTCACAGGGAACACTGAAGGCAGAACGTCCGACATGAGGACTGAAATCATA

AGAATGATGGAAAGTGCCAAACCAGAAGATGTGTCTTTCCAGGGGCGGGGAGTCTTCGAG

CTCTCGGACGAAAAGGCAACGAACCCGATCGTGCCTTCCTTTGACATGAGTAATGAAGGA

TCTTATTTCTTCGGAGACAATGCAGAGGAGTATGACAATTAAAGAAAAATACCC------

------------------------------------------------------

>A_duck_Chelyabinsk_1207-1_2020_EPI1812534

------------GTAGATAATCACTCACTGAGTGACATCAACATCATGGCGTCTCAAGGC

ACCAAACGATCTTATGAACAGATGGAAACTGGTGGGGAGCGCCAGAATGCCACTGAGATT

AGAGCATCGGTTGGAAGAATGGTTGGTGGAATTGGGAGGTTCTACATACAGATGTGCACT

GAGCTCAAACTCAGCGACTATGAAGGAAGACTGATCCAGAACAGCATAACAATAGAGAGA

ATGGTTCTCTCCGCATTTGATGAAAGGAGAAACAAATATCTGGAAGAACATCCCAGTGCG

GGGAAAGACCCGAAGAAAACAGGAGGTCCAATTTATCGAAGGAGAGATGGGAAATGGGTG

AGAGAGCTGATCCTGTATGACAAAGAGGAGATCAGGAGGATCTGGCGTCAAGCGAATAAT

GGAGAAGACGCAACTGCTGGTCTCACTCACCTGATGATCTGGCATTCCAATCTAAATGAT

GCCACATACCAGAGGACAAGAGCTCTCGTGCGTACCGGTATGGACCCCAGGATGTGCTCC

CTCATGCAAGGGTCAACTCTCCCAAGGAGGTCTGGAGCTGCTGGTGCAGCAGTGAAGGGA

GTTGGAACGATGGTGATGGAACTAATTCGGATGATAAAGCGAGGAGTTAATGATCGAAAC

TTCTGGAGAGGCGAGAATGGACGGAGGACAAGGATTGCATTTGAGAGAATGTGCAATATT

CTCAAAGGGAAATTCCAAACAGCAGCACAAAGAGCAATGATGGATCAAGTGCGTGAAAGC

AGGAATCCTGGCAATGCTGAAATTGAAGATCTCATTTTTCTGGCGCGGTCTGCTCTCATT

CTGAGAGGATCAGTGGCTCATAAGTCCTGCTTGCCCGCTTGTGTATACGGGCTCGCTGTA

GCCAGTGGATACGACTTTGAGAGAGAAGGGTACTCTCTAGTTGGAATAGACCCTTTCCGA

CTGCTTCAAAACAGCCAGGTCTTTAGTCTCATTAGACCAAATGAGAATCCAGCACACAAG

AGTCAATTGGTGTGGATGGCATGTCATTCTGCAGCATTCGAGGATCTGAGAGTCTCAAGT

TTCATCAGAGGAACAAGAGTAGTCCCAAGAGGACAACTATCCACTAGAGGAGTTCAAATT

GCTTCAAATGAGAACATAGAAACAATGGACTCCAGCACTCTTGAACTGAGAAGCAGATAT

TGGGCTATAAGAACCAGGAGTGGAGGAAACACCAACCAACAGAGAGCATCTGCAGGACAA

ATCAGTGTACAGCCCACTTTCTCGGTACAGAGAAACCTTCCCTTCGAAAGAGCGACCATT

ATGGCGGCATTCACAGGGAACACTGAAGGCAGAACGTCCGACATGAGGACTGAAATCATA

AGAATGATGGAAAGTGCCAGACCAGAAGATGTGTCTTTCCAGGGGCGGGGAGTCTTCGAG

CTCTCGGACGAAAAGGCAACGAACCCGATCGTGCCTTCCTTTGACATGAGTAATGAAGGA

TCTTATTTCTTCGGAGACAATGCAGAGGAGTATGACAATTAAAGAAAAATAC--------

------------------------------------------------------

>A_goose_Omsk_0002_2020_EPI1813114

AGCAAAAGCAGGGTAGATAATCACTCACTGAGTGACATCAACATCATGGCGTCTCAAGGC

ACCAAACGATCTTATGAACAGATGGAAACTGGTGGGGAACGCCAGAATGCCACTGAGATT

AGAGCATCGGTTGGAAGAATGGTTGGTGGAATTGGGAGGTTCTACATACAGATGTGCACT

GAGCTCAAACTCAGCGACTATGAAGGAAGACTGATCCAGAACAGCATAACAATAGAGAGA

ATGGTTCTCTCCGCATTTGATGAAAGGAGAAACAAATATCTGGAAGAACATCCCAGTGCG

GGGAAAGACCCGAAGAAAACAGGAGGTCCAATTTATCGAAGGAGAGATGGGAAATGGGTG

AGAGAGCTGATCCTGTATGACAAAGAGGAGATCAGGAGGATCTGGCGCCAAGCGAATAAT

GGAGAAGACGCAACTGCTGGTCTCACTCACCTGATGATCTGGCATTCCAATCTAAATGAT

GCCACATACCAGAGGACAAGAGCTCTCGTGCGTACCGGTATGGACCCCAGGATGTGCTCC

CTCATGCAAGGGTCAACTCTCCCAAGGAGGTCTGGAGCTGCTGGTGCAGCAGTGAAGGGA

GTTGGAACGATGGTGATGGAACTAATTCGGATGATAAAGCGAGGAGTTAATGATCGAAAC

TTCTGGAGAGGCGAGAATGGACGGAGGACAAGGATTGCATTTGAGAGAATGTGCAATATT

CTCAAAGGGAAATTCCAAACAGCAGCACAAAGAGCAATGATGGATCAAGTGCGTGAAAGC

AGGAATCCTGGCAATGCTGAAATTGAAGATCTCATTTTTCTGGCGCGGTCTGCTCTCATT

CTGAGAGGATCAGTGGCCCATAAGTCCTGCTTGCCCGCTTGTGTATACGGGCTCGCTGTA

GCCAGTGGATACGACTTTGAGAGAGAAGGGTACTCTCTAGTTGGAATAGACCCTTTCCGA

CTGCTTCAAAACAGCCAGGTCTTTAGTCTCATTAGACCAAATGAGAATCCGGCACACAAG

AGTCAATTGGTGTGGATGGCATGTCATTCTGCAGCATTCGAGGATCTGAGAGTCTCAAGT

TTCATCAGAGGAACAAGAGTAGTCCCAAGAGGACAACTATCCACTAGAGGAGTTCAAATT

GCTTCAAATGAGAACATAGAAACAATGGACTCCAGCACTCTTGAACTGAGAAGCAGATAT

TGGGCTATAAGAACCAGGAGTGGAGGAAACACCAACCAACAGAGAGCATCTGCAGGACAA

ATCAGTGTACAGCCCACTTTCTCGGTACAGAGAAACCTTCCCTTCGAAAGAGCGACCATT

ATGGCGGCATTCACAGGGAACACTGAAGGCAGAACGTCCGACATGAGGACTGAAATCATA

AGAATGATGGAAAGTGCCAGACCAGAAGATGTGTCTTTCCAGGGGCGGGGAGTCTTCGAG

CTCTCGGACGAAAAGGCAACGAACCCGATCGTGCCTTCCTTTGACATGAGTAATGAAGGA

TCTTATTTCTTCGGAGACAATGCAGAGGAGTATGACAATTAAAGAAAAATACCCTTGTTT

CTACT-------------------------------------------------

>A_goose_Omsk_01171_2020_EPI1813194

AGCAAAAGCAGGGTAGATAATCACTCACTGAGTGACATCAACATCATGGCGTCTCAAGGC

ACCAAACGATCTTATGAACAGATGGAAACTAGTGGGGAGCGCCAGAATGCCACTGAGATT

AGAGCATCTGTTGGAAGAATGGTTGGTGGAATTGGGAGGTTCTACATACAGATGTGCACT

GAGCTCAAACTCAGCGACTATGAAGGAAGACTGATCCAGAACAGCATAACAATAGAGAGA

ATGGTTCTCTCCGCATTTGATGAAAGGAGAAACAAATATCTGGAAGAACATCCCAGTGCG

GGGAAAGACCCGAAGAAAACAGGAGGTCCAATTTATCGAAGGAGAGATGGGAAATGGGTG

AGAGAGCTGATCCTGTATGACAAAGAGGAGATCAGGAGGATCTGGCGTCAAGCGAATAAT

GGAGAAGACGCAACTGCTGGTCTCACTCACCTGATGGTCTGGCATTCCAATCTAAATGAT

GCCACATACCAGAGGACAAGAGCTCTCGTGCGTACCGGTATGGACCCCAGGATGTGCTCC

CTCATGCAAGGGTCAACTCTCCCAAGGAGGTCTGGAGCTGCTGGTGCAGCAGTGAAGGGA

GTTGGAACGATGGTGATGGAACTAATTCGGATGATAAAGCGAGGAGTTAATGATCGAAAC

TTCTGGAGAGGCGAGAATGGACGGAGGACAAGGATTGCATTTGAGAGAATGTGCAATATT

CTCAAAGGGAAATTCCAAACAGCAGCACAAAGAGCAATGATGGATCAAGTGCGTGAAAGC

AGGAATCCTGGCAATGCTGAAATTGAAGATCTCATTTTTCTGGCGCGGTCTGCTCTCATT

CTGAGAGGATCAGTGGCCCATAAGTCCTGCTTGCCCGCTTGTGTATACGGGCTCGCTGTA

GCCAGTGGATACGACTTTGAGAGAGAAGGGTACTCTCTAGTTGGAATAGACCCTTTCCGA

CTGCTTCAAAACAGCCAGGTCTTTAGTCTCATTAGACCAAATGAGAATCCAGCACACAAG

AGTCAATTGGTGTGGATGGCATGTCATTCTGCAGCATTCGAGGATCTGAGAGTCTCAAGT

TTCATCAGAGGAACAAGAGTAGTCCCAAGAGGACAACTATCCACTAGAGGAGTTCAAATT

GCTTCAAATGAGAACATAGAAACAATGGACTCCAGCACTCTTGAACTGAGAAGCAGATAT

TGGGCTATAAGAACCAGGAGTGGAGGAAACACCAACCAACAGAGAGCATCTGCAGGACAA

ATCAGTGTACAGCCCACTTTCTCGGTACAGAGAAACCTTCCCTTCGAAAGAGCGACCATT

ATGGCGGCATTCACAGGGAACACTGAAGGCAGAACGTCCGACATGAGGACTGAAATCATA

AGAATGATGGAAAGTGCCAGACCAGAAGATGTGTCTTTCCAGGGGCGGGGAGTCTTCGAG

CTCTCGGACGAAAAGGCAACGAACCCGATCGTGCCTTCCTTTGACATGAGTAATGAAGGA

TCTTATTTCTTCGGAGACAATGCAGAGGAGTATGACAATTAAAGAAAAATACCCTTGTTT

CTACT-------------------------------------------------

>A_duck_Omsk_0075_2020_EPI1813274

AGCAAAAGCAGGGTAGATAATCACTCACTGAGTGACATCAACATCATGGCGTCTCAAGGC

ACCAAACGATCTTATGAACAGATGGAAACTGGTGGGGAGCGCCAGAATGCCACTGAGATT

AGAGCATCGGTTGGAAGAATGGTTGGTGGAATTGGGAGGTTCTACATACAGATGTGCACT

GAGCTCAAACTCAGCGACTATGAAGGAAGACTGATCCAGAACAGCATAACAATAGAGAGA

ATGGTTCTCTCCGCATTTGATGAAAGGAGAAACAAATATCTGGAAGAACATCCCAGTGCG

GGGAAAGACCCGAAGAAAACAGGAGGTCCAATTTATCGAAGGAGAGATGGGAAATGGGTG

AGAGAGCTGATCCTGTATGACAAAGAGGAGATCAGGAGGATCTGGCGTCAAGCGAATAAT

GGAGAAGACGCAACTGCTGGTCTCACTCACCTGATGATCTGGCATTCCAATCTAAATGAT

GCCACATACCAGAGGACAAGAGCTCTCGTGCGTACCGGTATGGACCCCAGGATGTGCTCC

CTCATGCAAGGGTCAACTCTCCCAAGGAGGTCTGGAGCTGCTGGTGCAGCAGTGAAGGGA

GTTGGAACGATGGTGATGGAACTAATTCGGATGATAAAGCGAGGAGTTAATGATCGAAAC

TTCTGGAGAGGCGAGAATGGACGGAGGACAAGGATTGCATTTGAGAGAATGTGCAATATT

CTCAAAGGGAAATTCCAAACAGCAGCACAAAGAGCAATGATGGATCAAGTGCGTGAAAGC

AGGAATCCTGGCAATGCTGAAATTGAAGATCTCATTTTTCTGGCGCGGTCTGCTCTCATT

CTGAGAGGATCAGTGGCCCATAAGTCCTGCTTGCCCGCTTGTGTATACGGGCTCGCTGTA

GCCAGTGGATACGACTTTGAGAGAGAAGGGTACTCTCTAGTTGGAATAGACCCTTTCCGA

CTGCTTCAAAACAGCCAGGTCTTTAGTCTCATTAGACCAAATGAGAATCCAGCACACAAG

AGTCAATTGGTGTGGATGGCATGTCATTCTGCAGCATTCGAGGATCTGAGAGTCTCAAGT

TTCATCAGAGGAACAAGAGTAGTCCCAAGAGGACAACTATCCACTAGAGGAGTTCAAATT

GCTTCAAATGAGAACATAGAAACAATGGACTCCAGCACTCTTGAACTGAGAAGCAGATAT

TGGGCTATAAGAACCAGGAGTGGAGGAAACACCAACCAACAGAGAGCATCTGCAGGACAA

ATCAGTGTACAGCCCACTTTCTCGGTACAGAGAAACCTTCCCTTCGAAAGAGCGACCATT

ATGGCGGCATTCACAGGGAACACTGAAGGCAGAACGTCCGACATGAGGACTGAAATCATA

AGAATGATGGAAAGTGCCAGACCAGAAGATGTGTCTTTCCAGGGGCGGGGAGTCTTCGAG

CTCTCGGACGAAAAGGCAACGAACCCGATCGTGCCTTCCTTTGACATGAGTAATGAAGGA

TCTTATTTCTTCGGAGACAATGCAGAGGAGTATGACAATTAAAGAAAAATACCCTTGTTT

CTACT-------------------------------------------------

>A_duck_Omsk_0004_2020_EPI1813330

AGCAAAAGCAGGGTAGATAATCACTCACTGAGTGACATCAACATCATGGCGTCTCAAGGC

ACCAAACGATCTTATGAACAGATGGAAACTGGTGGGGAGCGCCAGAATGCCACTGAGATT

AGAGCATCGGTTGGAAGAATGGTTGGTGGAATTGGGAGGTTCTACATACAGATGTGCACT

GAGCTCAAACTCAGCGACTATGAAGGAAGACTGATCCAGAACAGCATAACAATAGAGAGA

ATGGTTCTCTCCGCATTTGATGAAAGGAGAAACAAATATCTGGAAGAACATCCCAGTGCG

GGGAAAGACCCGAAGAAAACAGGAGGTCCAATTTATCGAAGGAGAGATGGGAAATGGGTG

AGAGAGCTGATCCTGTATGACAAAGAGGAGATCAGGAGGATCTGGCGTCAAGCGAATAAT

GGAGAAGACGCAACTGCTGGTCTCACTCACCTGATGATCTGGCATTCCAATCTAAATGAT

GCCACATACCAGAGGACAAGAGCTCTCGTGCGTACCGGTATGGACCCCAGGATGTGCTCC

CTCATGCAAGGGTCAACTCTCCCAAGGAGGTCTGGAGCTGCTGGTGCAGCAGTGAAGGGA

GTTGGAACGATGGTGATGGAACTAATTCGGATGATAAAGCGAGGAGTTAATGATCGAAAC

TTCTGGAGAGGCGAGAATGGACGGAGGACAAGGATTGCATTTGAGAGAATGTGCAATATT

CTCAAAGGGAAATTCCAAACAGCAGCACAAAGAGCAATGATGGATCAAGTGCGTGAAAGC

AGGAATCCTGGCAATGCTGAAATTGAAGATCTCATTTTTCTGGCGCGGTCTGCTCTCATT

CTGAGAGGATCAGTGGCCCATAAGTCCTGCTTGCCCGCTTGTGTATACGGGCTCGCTGTA

GCCAGTGGATACGACTTTGAGAGAGAAGGGTACTCTCTAGTTGGAATAGACCCTTTCCGA

CTGCTTCAAAACAGCCAGGTCTTTAGTCTCATTAGACCAAATGAGAATCCAGCACACAAG

AGTCAATTGGTGTGGATGGCATGTCATTCTGCAGCATTCGAGGATCTGAGAGTCTCAAGT

TTCATCAGAGGAACAAGAGTAGTCCCAAGAGGACAACTATCCACTAGAGGAGTTCAAATT

GCTTCAAATGAGAACATAGAAACAATGGACTCCAGCACTCTTGAACTGAGAAGCAGATAT

TGGGCTATAAGAACCAGGAGTGGAGGAAACACCAACCAACAGAGAGCATCTGCAGGACAA

ATCAGTGTACAGCCCACTTTCTCGGTACAGAGAAACCTTCCCTTCGAAAGAGCGACCATT

ATGGCGGCATTCACAGGGAACACTGAAGGCAGAACGTCCGACATGAGGACTGAAATCATA

AGAATGATGGAAAGTGCCAGACCAGAAGATGTGTCTTTCCAGGGGCGGGGAGTCTTCGAG

CTCTCGGACGAAAAGGCAACGAACCCGATCGTGCCTTCCTTTGACATGAGTAATGAAGGA

TCTTATTTCTTCGGAGACAATGCAGAGGAGTATGACAATTAAAGAAAAATACCCTTGTTT

CTACT-------------------------------------------------

>A_chicken_Omsk_0112_2020_EPI1813338

AGCAAAAGCAGGGTAGATAATCACTCACTGAGTGACATCAACATCATGGCGTCTCAAGGC

ACCAAACGATCTTATGAACAGATGGAAACTAGTGGGGAGCGCCAGAATGCCACTGAGATT

AGAGCATCGGTTGGAAGAATGGTTGGTGGAATTGGGAGGTTCTACATACAGATGTGCACT

GAGCTCAAACTCAGCGACTATGAAGGAAGACTGATCCAGAACAGCATAACAATAGAGAGA

ATGGTTCTCTCCGCATTTGATGAAAGGAGAAACAAATATCTGGAAGAACATCCCAGTGCG

GGGAAAGACCCGAAGAAAACAGGAGGTCCAATTTATCGAAGGAGAGATGGGAAATGGGTG

AGAGAGCTGATCCTGTATGACAAAGAGGAGATCAGGAGGATCTGGCGTCAAGCGAATAAT

GGAGAAGACGCAACTGCTGGTCTCACTCACCTGATGATCTGGCATTCCAATCTAAATGAT

GCCACATACCAGAGGACAAGAGCTCTCGTGCGTACCGGTATGGACCCCAGGATGTGCTCC

CTCATGCAAGGGTCAACTCTCCCAAGGAGGTCTGGAGCTGCTGGTGCAGCAGTGAAGGGA

GTTGGAACGATGGTGATGGAACTAATTCGGATGATAAAGCGAGGAGTTAATGATCGAAAC

TTCTGGAGAGGCGAGAATGGACGGAGGACAAGGATTGCATTTGAGAGAATGTGCAATATT

CTCAAAGGGAAATTCCAAACAGCAGCACAAAGAGCAATGATGGATCAAGTGCGTGAAAGC

AGGAATCCTGGCAATGCTGAAATTGAAGATCTCATTTTTCTGGCGCGGTCTGCTCTCATT

CTGAGAGGATCAGTGGCCCATAAGTCCTGCTTGCCCGCTTGTGTATACGGGCTCGCTGTA

GCCAGTGGATACGACTTTGAGAGAGAAGGGTACTCTCTAGTTGGAATAGACCCTTTCCGA

CTGCTTCAAAACAGCCAGGTCTTTAGTCTCATTAGACCAAATGAGAATCCAGCACACAAG

AGTCAATTGGTGTGGATGGCATGTCATTCTGCAGCATTCGAGGATCTGAGAGTCTCAAGT

TTCATCAGAGGAACAAGAGTAGTCCCAAGAGGACAACTATCCACTAGAGGAGTTCAAATT

GCTTCAAATGAGAACATAGAAACAATGGACTCCAGCACTCTTGAACTGAGAAGCAGATAT

TGGGCTATAAGAACCAGGAGTGGAGGAAACACCAACCAACAGAGAGCATCTGCAGGACAA

ATCAGTGTACAGCCCACTTTCTCGGTACAGAGAAACCTTCCCTTCGAAAGAGCGACCATT

ATGGCGGCATTCACAGGGAACACTGAAGGCAGAACGTCCGACATGAGGACTGAAATCATA

AGAATGATGGAAAGTGCCAGACCAGAAGATGTGTCTTTCCAGGGGCGGGGAGTCTTCGAG

CTCTCGGACGAAAAGGCAACGAACCCGATCGTGCCTTCCTTTGACATGAGTAATGAAGGA

TCTTATTTCTTCGGAGACAATGCAGAGGAGTATGACAATTAAAGAAAAATACCCTTGTTT

CTACT-------------------------------------------------

>A_duck_Saratov_29804_2020_EPI1814258

AGCAAAAGCAGGGTAGATAATCACTCACTGAGTGACATCAACATCATGGCGTCTCAAGGC

ACCAAACGATCTTATGAACAGATGGAAACTGGTGGGGAGCGCCAGAATGCCACTGAGATT

AGAGCATCGGTTGGAAGAATGGTTGGTGGAATTGGGAGGTTCTACATACAGATGTGCACT

GAGCTCAAACTCAGCGACTATGAAGGAAGACTGATCCAGAACAGCATAACAATAGAGAGA

ATGGTTCTCTCCGCATTTGATGAAAGGAGAAACAAATATCTGGAAGAACATCCCAGTGCG

GGGAAAGACCCGAAGAAAACAGGAGGTCCAATTTATCGAAGGAGAGATGGGAAATGGGTG

AGAGAGCTGATCCTGTATGACAAAGAGGAGATCAGGAGAATCTGGCGTCAAGCGAATAAT

GGAGAAGACGCAACTGCTGGTCTCACTCACCTAATGATCTGGCATTCCAATCTAAATGAT

GCCACATACCAGAGGACAAGAGCTCTCGTGCGTACCGGTATGGACCCCAGGATGTGCTCC

CTCATGCAAGGGTCAACTCTCCCAAGGAGGTCTGGAGCTGCTGGTGCAGCAGTGAAGGGA

GTTGGAACGATGGTGATGGAACTAATTCGGATGATAAAGCGAGGAGTTAATGATCGAAAC

TTCTGGAGAGGCGAGAATGGACGGAGGACAAGGATTGCATTTGAGAGAATGTGCAATATT

CTCAAAGGGAAATTCCAAACAGCAGCACAAAGAGCAATGATGGATCAAGTGCGTGAAAGC

AGGAATCCTGGCAATGCTGAAATTGAAGATCTTATTTTTCTGGCGCGGTCTGCTCTCATT

CTGAGAGGATCAGTGGCCCATAAGTCCTGCTTGCCCGCTTGTGTATACGGGCTCGCTGTA

GCCAGTGGATACGACTTTGAGAGAGAAGGGTACTCTCTAGTTGGAATAGACCCTTTCCGA

CTGCTTCAAAACAGCCAGGTCTTTAGTCTCATTAGACCAAATGAGAATCCAGCACACAAG

AGTCAATTGGTGTGGATGGCATGTCATTCTGCAGCATTCGAGGATCTGAGAGTCTCAAGT

TTCATCAGAGGAACAAGAGTAGTCCCAAGAGGACAACTATCCACTAGAGGAGTTCAAATT

GCTTCAAATGAGAACATAGAAACAATGGACTCCAGCACTCTTGAACTGAGAAGCAGATAT

TGGGCTATAAGAACCAGGAGTGGAGGAAACACCAACCAACAGAGAGCATCTGCAGGACAA

ATCAGTGTACAGCCCACTTTCTCGGTACAGAGAAACCTTCCCTTCGAAAGAGCGACCATT

ATGGCGGCATTCACAGGGAACACTGAAGGCAGAACGTCCGACATGAGGACTGAAATCATA

AGAATGATGGAAAGTGCCAGACCAGAAGATGTGTCTTTCCAGGGGCGGGGAGTCTTCGAG

CTCTCGGACGAAAAGGCAACGAACCCGATCGTGCCTTCCTTTGACATGAGTAATGAAGGA

TCTTATTTCTTCGGAGACAATGCAGAGGAGTATGACAATTAAAGAAAAATACCCTTGTTT

CTACT-------------------------------------------------

>A_goose_Omsk_30001_2020_EPI1814274

AGCAAAAGCAGGGTAGATAATCACTCACTGAGTGACATCAACATCATGGCGTCTCAAGGC

ACCAAACGATCTTATGAACAGATGGAAACTGGTGGGGAGCGCCAGAATGCCACTGAGATT

AGAGCATCGGTTGGAAGAATGGTTGGTGGAATTGGGAGGTTCTACATACAGATGTGCACT

GAGCTCAAACTCAGCGACTATGAAGGAAGACTGATCCAGAACAGCATAACAATAGAGAGA

ATGGTTCTCTCCGCATTTGATGAAAGGAGAAACAAATATCTGGAAGAACATCCCAGTGCG

GGGAAAGACCCGAAGAAAACAGGAGGTCCAATTTATCGAAGGAGAGATGGGAAATGGGTG

AGAGAGCTGATCCTGTATGACAAAGAGGAGATCAGGAGGATCTGGCGTCAAGCGAATAAT

GGAGAAGACGCAACTGCTGGTCTCACTCACCTGATGATCTGGCATTCCAATCTAAATGAT

GCCACATACCAGAGGACAAGAGCTCTCGTGCGTACCGGTATGGACCCCAGGATGTGCTCC

CTCATGCAAGGGTCAACTCTCCCAAGGAGGTCTGGAGCTGCTGGTGCAGCAGTGAAGGGA

GTTGGAACGATGGTGATGGAACTAATTCGGATGATAAAGCGAGGAGTTAATGATCGAAAC

TTCTGGAGAGGTGAGAATGGACGGAGGACAAGGATTGCATTTGAGAGAATGTGCAATATT

CTCAAAGGGAAATTCCAAACAGCAGCACAAAGAGCAATGATGGATCAAGTGCGTGAAAGC

AGGAATCCTGGCAATGCTGAAATTGAAGATCTCATTTTTCTGGCGCGGTCTGCTCTCATT

CTGAGAGGATCAGTGGCCCATAAGTCCTGCTTGCCCGCTTGTGTATACGGGCTCGCTGTA

GCCAGTGGATACGACTTTGAGAGAGAAGGGTACTCTCTAGTTGGAATAGACCCTTTCCGA

CTGCTTCAAAACAGCCAGGTCTTTAGTCTCATTAGACCAAATGAGAATCCAGCACACAAG

AGTCAATTGGTGTGGATGGCATGTCATTCTGCAGCATTCGAGGATCTGAGAGTCTCAAGT

TTCATCAGAGGAACAAGAGTAGTCCCAAGAGGACAACTATCCACTAGAGGAGTTCAAATT

GCTTCAAATGAGAACATAGAAACAATGGACTCCAGCACTCTTGAACTGAGAAGCAGATAT

TGGGCTATAAGAACCAGGAGTGGAGGAAACACCAACCAACAGAGAGCATCTGCAGGACAA

ATCAGTGTACAGCCCACTTTCTCGGTACAGAGAAACCTTCCCTTCGAAAGAGCGACCATT

ATGGCAGCATTCACAGGGAACACTGAAGGCAGAACGTCCGACATGAGGACTGAAATCATA

AGAATGATGGAAAGTGCCAGACCAGAAGATGTGTCTTTCCAGGGGCGGGGAGTCTTCGAG

CTCTCGGACGAAAAGGCAACGAACCCGATCGTGCCTTCCTTTGACATGAGTAATGAAGGA

TCTTATTTCTTCGGAGACAATGCAGAGGAGTATGACAATTAAAGAAAAATACCCTTGTTT

CTACT-------------------------------------------------

>A_goose_Omsk_30003_2020_EPI1814282

AGCAAAAGCAGGGTAGATAATCACTCACTGAGTGACATCAACATCATGGCGTCTCAAGGC

ACCAAACGATCTTATGAACAGATGGAAACTGGTGGGGAGCGCCAGAATGCCACTGAGATT

AGAGCATCGGTTGGAAGAATGGTTGGTGGAATTGGGAGGTTCTACATACAGATGTGCACT

GAGCTCAAACTCAGCGACTATGAAGGAAGACTGATCCAGAACAGCATAACAATAGAGAGA

ATGGTTCTCTCCGCATTTGATGAAAGGAGAAACAAATATCTGGAAGAACATCCCAGTGCG

GGGAAAGACCCGAAGAAAACAGGAGGTCCAATTTATCGAAGGAGAGATGGGAAATGGGTG

AGAGAGCTGATCCTGTATGACAAAGAGGAGATCAGGAGGATCTGGCGTCAAGCGAATAAT

GGAGAAGACGCAACTGCTGGTCTCACTCACCTGATGATCTGGCATTCCAATCTAAATGAT

GCCACATACCAGAGGACAAGAGCTCTCGTGCGTACCGGTATGGACCCCAGGATGTGCTCC

CTCATGCAAGGGTCAACTCTCCCAAGGAGGTCTGGAGCTGCTGGTGCAGCAGTGAAGGGA

GTTGGAACGATGGTGATGGAACTAATTCGGATGATAAAGCGAGGAGTTAATGATCGAAAC

TTCTGGAGAGGCGAGAATGGACGGAGGACAAGGATTGCATTTGAGAGAATGTGCAATATT

CTCAAAGGGAAATTCCAAACAGCAGCACAAAGAGCAATGATGGATCAAGTGCGTGAAAGC

AGGAATCCTGGCAATGCTGAAATTGAAGATCTCATTTTTCTGGCGCGGTCTGCTCTCATT

CTGAGAGGATCAGTGGCCCATAAGTCCTGCTTGCCCGCTTGTGTATACGGGCTCGCTGTA

GCCAGTGGATACGACTTTGAGAGAGAAGGGTACTCTCTAGTTGGAATAGACCCTTTCCGA

CTGCTTCAAAACAGCCAGGTCTTTAGTCTCATTAGACCAAATGAGAATCCAGCACACAAG

AGTCAATTGGTGTGGATGGCATGTCATTCTGCAGCATTCGAGGATCTGAGAGTCTCAAGT

TTCATCAGAGGAACAAGAGTAGTCCCAAGAGGACAACTATCCACTAGAGGAGTTCAAATT

GCTTCAAATGAGAACATAGAAACAATGGACTCCAGCACTCTTGAACTGAGAAGCAGATAT

TGGGCTATAAGAACCAGGAGTGGAGGAAACACCAACCAACAGAGAGCATCTGCAGGACAA

ATCAGTGTACAGCCCACTTTCTCGGTACAGAGAAACCTTCCCTTCGAAAGAGCGACCATT

ATGGCGGCATTCACAGGGAACACTGAAGGCAGAACGTCCGACATGAGGACTGAAATCATA

AGAATGATGGAAAGTGCCAGACCAGAAGATGTGTCTTTCCAGGGGCGGGGAGTCTTCGAG

CTCTCGGACGAAAAGGCAACGAACCCGATCGTGCCTTCCTTTGACATGAGTAATGAAGGA

TCTTATTTCTTCGGAGACAATGCAGAGGAGTATGACAATTAAAGAAAAATACCCTTGTTT

CTACT-------------------------------------------------

>A_swan_Tumen_1479-2_2020_EPI1814685

------------GTAGATAATCACTCACTGAGTGACATCAACATCATGGCGTCTCAAGGC

ACCAAACGATCTTATGAACAGATGGAAACTGGTGGGGAGCGCCAGAATGCCACTGAGATT

AGAGCATCGGTTGGAAGAATGGTTGGTGGAATTGGGAGGTTCTACATACAGATGTGCACT

GAGCTCAAACTCAGCGACTATGAAGGAAGACTGATCCAGAACAGCATAACAATAGAGAGA

ATGGTTCTCTCCGCATTTGATGAAAGGAGAAACAAATATCTGGAAGAACATCCCAGTGCG

GGGAAAGACCCGAAGAAAACAGGAGGTCCAATTTATCGAAGGAGAGATGGGAAATGGGTG

AGAGAGCTGATCCTGTATGACAAAGAGGAGATCAGGAGGATCTGGCGTCAAGCGAATAAT

GGAGAAGACGCAACTGCTGGTCTCACTCACCTAATGATCTGGCATTCCAATCTAAATGAT

GCCACATACCAGAGGACAAGAGCTCTCGTGCGTACCGGTATGGACCCCAGGATGTGCTCC

CTCATGCAAGGGTCAACTCTCCCAAGGAGGTCTGGAGCTGCTGGTGCAGCAGTGAAGGGA

GTTGGAACGATGGTGATGGAACTAATTCGGATGATAAAGCGAGGAGTTAATGATCGAAAC

TTCTGGAGAGGCGAGAATGGACGGAGGACAAGGATTGCATTTGAGAGAATGTGCAATATT

CTCAAAGGGAAATTCCAAACAGCAGCACAAAGAGCAATGATGGATCAAGTGCGTGAAAGC

AGGAATCCTGGCAATGCTGAAATTGAAGATCTCATTTTTCTGGCGCGGTCTGCTCTCATT

CTGAGAGGATCAGTGGCCCATAAGTCCTGCTTGCCCGCTTGTGTATACGGGCTCGCTGTA

GCCAGTGGATACGACTTTGAGAGAGAAGGGTACTCTCTAGTTGGAATAGACCCTTTCCGA

CTGCTTCAAAACAGCCAGGTCTTTAGTCTCATTAGACCAAATGAGAATCCAGCACACAAG

AGTCAATTGGTGTGGATGGCATGTCATTCTGCAGCATTCGAGGATCTGAGAGTCTCAAGT

TTCATCAGAGGAACAAGAGTAGTCCCAAGAGGACAACTATCCACTAGAGGAGTTCAAATT

GCTTCAAATGAGAACATAGAAACAATGGACTCCAGCACTCTTGAACTGAGAAGCAGGTAT

TGGGCTATAAGAACCAGGAGTGGAGGAAACACCAACCAACAGAGAGCATCTGCAGGACAA

ATCAGTGTACAGCCCACTTTCTCGGTACAGAGAAACCTTCCCTTCGAAAGAGCGACCATT

ATGGCGGCATTCACAGGGAACACTGAAGGCAGAACGTCCGACATGAGGACTGAAATCATA

AGAATGATGGAAAGTGCCAGACCAGAAGATGTGTCTTTCCAGGGGCGGGGAGTCTTCGAG

CTCTCGGACGAAAAGGCAACGAACCCGATCGTGCCTTCCTTTGACATGAGTAATGAAGGA

TCTTATTTCTTCGGAGACAATGCAGAGGAGTATGACAATTAAAGAAAAATAC--------

------------------------------------------------------

>A_Whooper_swan_Mongolia_24_2020_EPI1831867

AGCAAAAGCAGGGTAGATAATCACTCACCGAGTGACATCAACATCATGGCGTCTCAAGGC

ACCAAACGATCCTATGAACAGATGGAAACTGGTGGAGAGCGCCAGAATGCTACTGAGATC

AGGGCCTCTGTTGGAAGAATGGTTGGTGGCATTGGGAGGTTCTACATACAGATGTGCACA

GAACTCAAACTCAGCGACCATGAGGGGAGACTTATCCAGAACAGCATAACAATAGAGAGA

ATGGTACTTTCTGCATTTGACGAAAGAAGGAACAGGTATTTGGAAGAGCACCCCAGTGCA

GGGAAGGACCCTAAGAAAACTGGAGGTCCAATCTATCGGAGGAGAGACGGGAAATGGATT

AGAGAGCTGATTTTGTACGACAAAGAAGAAATCAGGAGGATTTGGCGCCAAGCGAACAAC

GGAGAGGACGCAACTGCTGGTCTTACCCACCTGATGATATGGCACTCCAATCTTAATGAT

GCCACATATCAGAGAACAAGAGCTCTCGTGCGTACCGGAATGGACCCCAGGATGTGCTCC

CTAATGCAGGGATCAACTCTCCCGAGAAGATCTGGAGCTGCTGGTGCAGCAGTGAAGGGG

GTAGGAACAATGGTGATGGAGCTGATCCGAATGATAAAACGAGGGATTAACGACCGGAAT

TTCTGGAGAGGCGAAAATGGGCGGAGAACAAGGATTGCATATGAGAGAATGTGCAACATC

CTCAAAGGGAAATTCCAAACAGCTGCACAAAGAGCAATGATGGATCAAGTGCGAGAGAGC

AGAAATCCTGGGAATGCTGAAATTGAGGATCTTATTTTTCTGGCACGGTCTGCACTCATC

CTGAGAGGATCAGTGGCCCATAAGTCCTGCTTGCCTGCTTGTGTGTACGGACTTGCAGTG

GCCAGTGGGTATGATTTCGAGAGAGAAGGATACTCTCTGGTTGGGATAGATCCTTTCCGT

CTGCTTCAAAACAGCCAGGTCTTTAGTCTCATTAGGCCAAATGAAAACCCAGCACATAAG

AGTCAGTTAGTGTGGATGGCATGCCATTCTGCAGCATTTGAGGACCTCAGAGTATCAAGT

TTCATCAGAGGGACAAGAGTTGTCCCAAGAGGGCAGTTATCCACTAGAGGGGTTCAAATT

GCTTCAAATGAGAACATGGAAACAATGGACTCCAACACACTTGAGCTGAGAAGTAGGTAT

TGGGCCATAAGAACCAGGAGCGGAGGGAATACTAACCAGCAGAGGGCGTCTGCAGGGCAA

ATTAGTGTTCAACCCACTTTCTCAGTGCAGAGAAACCTTCCCTTCGAAAGAACGACCATT

ATGGCAGCATTTGCAGGAAATACTGAAGGCAGAACGTCCGATATGAGGACAGAAATCATA

AGAATGATGGAAAATGCCAAACCAGAAGATGTGTCATTCCAGGGGCGGGGAGTCTTCGAG

CTCTCGGACGAAAAGGCAACGAACCCGATCGTGCCTTCCTTTGACATGAATAATGAAGGA

TCTTATTTCTTCGGAGACAATGCAGAGGAGTATGACAATTAAAGAAAAATACCCTTGTTT

CTACT-------------------------------------------------

>A_Whooper_swan_Mongolia_25_2020_EPI1831875

AGCAAAAGCAGGGTAGATAATCACTCACCGAGTGACATCAACATCATGGCGTCTCAAGGC

ACCAAACGATCCTATGAACAGATGGAAACTGGTGGAGAGCGCCAGAATGCTACTGAGATC

AGGGCCTCTGTTGGAAGAATGGTTGGTGGCATTGGGAGGTTCTACATACAGATGTGCACA

GAACTCAAACTCAGCGACCATGAGGGGAGACTTATCCAGAACAGCATAACAATAGAGAGA

ATGGTACTTTCTGCATTTGACGAAAGAAGGAACAGGTATTTGGAAGAGCACCCCAGTGCA

GGGAAGGACCCTAAGAAAACTGGAGGTCCAATCTATCGGAGGAGAGACGGGAAATGGATT

AGAGAGCTGATTTTGTACGACAAAGAAGAAATCAGGAGGATTTGGCGCCAAGCGAACAAC

GGAGAGGACGCAACTGCTGGTCTTACCCACCTGATGATATGGCACTCCAATCTGAATGAT

GCCACATATCAGAGAACAAGAGCTCTCGTGCGTACCGGAATGGACCCCAGGATGTGCTCC

CTAATGCAGGGATCAACTCTCCCGAGAAGATCTGGAGCTGCTGGTGCAGCAGTGAAGGGG

GTAGGAACAATGGTGATGGAGCTGATCCGAATGATAAAACGAGGGATTAACGACCGGAAT

TTCTGGAGAGGCGAAAATGGGCGGAGAACAAGGATTGCATATGAGAGAATGTGCAACATC

CTCAAAGGGAAATTCCAAACAGCTGCACAAAGAGCAATGATGGATCAAGTGCGAGAGAGC

AGAAATCCTGGGAATGCTGAAATTGAGGATCTTATTTTTCTGGCACGGTCTGCACTCATC

CTGAGAGGATCAGTGGCCCATAAGTCCTGCTTGCCTGCTTGTGTGTACGGACTTGCAGTG

GCCAGTGGGTATGATTTCGAGAGAGAAGGATACTCTCTGGTTGGGATAGATCCTTTCCGT

CTGCTTCAAAACAGCCAGGTCTTTAGTCTCATTAGGCCAAATGAAAACCCAGCACATAAG

AGTCAGTTAGTATGGATGGCATGCCACTCTGCAGCATTTGAGGACCTCAGAGTATCAAGT

TTCATCAGAGGGACAAGAGTTGTCCCAAGAGGGCAGTTATCCACTAGAGGGGTTCAAATT

GCTTCAAATGAGAACATGGAAACAATGGACTCCAACACACTTGAGCTGAGAAGTAGGTAT

TGGGCCATAAGAACCAGGAGCGGAGGGAATACTAACCAGCAGAGGGCGTCTGCAGGGCAA

ATTAGTGTTCAACCCACTTTCTCAGTGCAGAGAAACCTTCCCTTCGAAAGAACGACCATT

ATGGCAGCATTTGCAGGAAATACTGAAGGCAGAACGTCCGATATGAGGACAGAAATCATA

AGAATGATGGAAAATGCCAAACCAGAAGATGTGTCATTCCAGGGGCGGGGAGTCTTCGAG

CTCTCGGACGAAAAGGCAACGAACCCGATCGTGCCTTCCTTTGACATGAATAATGAAGGA

TCTTATTTCTTCGGAGACAATGCAGAGGAGTATGACAATTAAAGAAAAATACCCTTGTTT

CTACT-------------------------------------------------

>A_chicken_Kazakhstan_Kn-3_2020_EPI1839254

---------------------------------------------ATGGCGTCTCAAGGC

ACCAAACGATCTTATGAACAGATGGAAACTGGTGGGGAGCGCCAGAATGCCACTGAGATT

AGAGCATCGGTTGGAAGAATGGTTGGTGGAATTGGGAGGTTCTACATACAGATGTGCACT

GAGCTCAAACTCAGCGACTATGAAGGAAGACTGATCCAGAACAGCATAACAATAGAGAGA

ATGGTTCTCTCCGCATTTGATGAAAGGAGAAACAAATATCTGGAAGAACATCCCAGTGCG

GGGAAAGACCCGAAGAAAACAGGAGGTCCAATTTATCGAAGGAGAGATGGGAAATGGGTG

AGAGAGCTGATCCTGTATGACAAAGAGGAGATCAGGAGGATCTGGCGTCAAGCGAATAAT

GGAGAAGACGCAACTGCTGGTCTCACTCACCTAATGATCTGGCATTCCAATCTAAATGAT

GCCACATACCAGAGGACAAGAGCTCTCGTGCGTACCGGTATGGACCCCAGGATGTGCTCC

CTCATGCAAGGGTCAACTCTCCCAAGGAGGTCTGGAGCTGCTGGTGCAGCAGTGAAGGGA

GTTGGAACGATGGTGATGGAACTAATTCGGATGATAAAGCGAGGAGTTAATGATCGAAAC

TTCTGGAGAGGCGAGAATGGACGGAGGACAAGGATTGCATTTGAGAGAATGTGCAATATT

CTCAAAGGGAAATTCCAAACAGCAGCACAAAGAGCAATGATGGATCAAGTGCGTGAAAGC

AGGAATCCTGGCAATGCTGAAATTGAAGATCTCATTTTTCTGGCGCGGTCTGCTCTCATT

CTGAGAGGATCAGTGGCCCATAAGTCCTGCTTGCCCGCTTGTGTATACGGGCTCGCTGTA

GCCAGTGGATACGACTTTGAGAGAGAAGGGTACTCTCTAGTTGGAATAGACCCTTTCCGA

CTGCTTCAAAACAGCCAGGTCTTTAGTCTCATTAGACCAAATGAGAATCCAGCACACAAG

AGTCAATTGGTGTGGATGGCATGTCATTCTGCAGCATTCGAGGATCTGAGAGTCTCAAGT

TTCATCAGAGGAACAAGAGTAGTCCCAAGAGGACAACTATCCACTAGAGGAGTTCAAATT

GCTTCAAATGAGAACATAGAAACAATGGACTCCAGCACTCTTGAACTGAGAAGCAGATAT

TGGGCTATAAGAACCAGGAGTGGAGGAAACACCAACCAACAGAGAGCATCTGCAGGACAA

ATCAGTGTACAGCCCACTTTCTCGGTACAGAGAAACCTTCCCTTCGAAAGAGCGACCATT

ATGGCGGCATTCACAGGGAACACTGAAGGCAGAACGTCCGACATGAGGACTGAAATCATA

AGAATGATGGAAAGTGCCAGACCAGAAGATGTGTCTTTCCAGGGGCGGGGAGTCTTCGAG

CTCTCGGACGAAAAGGCAACGAACCCGATCGTGCCTTCCTTTGACATGAGTAATGAAGGA

TCTTATTTCTTCGGAGACAATGCAGAGGAGTATGACAAT---------------------

------------------------------------------------------

>A_chicken_Kazakhstan_Kn-6_2020_EPI1839262

---------------------------------------------ATGGCGTCTCAAGGC

ACCAAACGATCTTATGAACAGATGGAAACTGGTGGGGAGCGCCAGAGTGCCACTGAGATT

AGAGCATCGGTTGGAAGAATGGTTGGTGGAATTGGGAGGTTCTACATACAGATGTGCACT

GAGCTCAAACTCAGCGACTATGAAGGAAGACTGATCCAGAACAGCATAACAATAGAGAGA

ATGGTTCTCTCCGCATTTGATGAAAGGAGAAACAAATATCTGGAAGAACATCCCAGTGCG

GGGAAAGACCCGAAGAAAACAGGAGGTCCAATTTATCGAAGGAGAGATGGGAAATGGGTG

AGAGAGCTGATCCTGTATGACAAAGAGGAGATCAGGAGGATCTGGCGTCAAGCGAATAAT

GGAGAAGACGCAACTGCTGGTCTCACTCACCTAATGATCTGGCATTCCAATCTAAATGAT

GCCACATACCAGAGGACAAGAGCTCTCGTGCGTACCGGTATGGACCCCAGGATGTGCTCC

CTCATGCAAGGGTCAACTCTCCCAAGGAGGTCTGGAGCTGCTGGTGCAGCAGTGAAGGGA

GTTGGAACGATGGTGATGGAACTAATTCGGATGATAAAGCGAGGAGTTAATGATCGAAAC

TTCTGGAGAGGCGAGAATGGACGGAGGACAAGGATTGCATTTGAGAGAATGTGCAATATT

CTCAAAGGGAAATTCCAAACAGCAGCACAAAGAGCAATGATGGATCAAGTGCGTGAAAGC

AGGAATCCTGGCAATGCTGAAATTGAAGATCTCATTTTTCTGGCGCGGTCTGCTCTCATT

CTGAGAGGATCAGTGGCCCATAAGTCCTGCTTGCCCGCTTGTGTATACGGGCTCGCTGTA

GCCAGTGGATACGACTTTGAGAGAGAAGGGTACTCTCTAGTTGGAATAGACCCTTTCCGA

CTGCTTCAAAACAGCCAGGTCTTTAGTCTCATTAGACCAAATGAGAATCCAGCACACAAG

AGTCAATTGGTGTGGATGGCATGTCATTCTGCAGCATTCGAGGATCTGAGAGTCTCAAGT

TTCATCAGAGGAACAAGAGTAGTCCCAAGAGGACAACTATCCACTAGAGGAGTTCAAATT

GCTTCAAATGAGAACATAGAAACAATGGACTCCAGCACTCTTGAACTGAGAAGCAGATAT

TGGGCTATAAGAACCAGGAGTGGAGGAAACACCAACCAACAGAGAGCATCTGCAGGACAA

ATCAGTGTACAGCCCACTTTCTCGGTACAGAGAAACCTTCCCTTCGAAAGAGCGACCATT

ATGGCGGCATTCACAGGGAACACTGAAGGCAGAACGTCCGACATGAGGACTGAAATCATA

AGAATGATGGAAAGTGCCAGACCAGAAGATGTGTCTTTCCAGGGGCGGGGAGTCTTCGAG

CTCTCGGACGAAAAGGCAACGAACCCGATCGTGCCTTCCTTTGACATGAGTAATGAAGGA

TCTTATTTCTTCGGAGACAATGCAGAGGAGTATGACAAT---------------------

------------------------------------------------------

>A_Muscovy_duck_China_FJFZ21_H5N6_2020_EPI1841917

---------------------------------------------ATGGCGTCTCAAGGC

ACCAAACGATCTTATGAACAGATGGAGACTGGTGGAGAGCGCCAGAATGCTACTGAGATC

AGGGCCTCTGTTGGAAGAATGGTTGGTGGTATTGGAAGGTTCTACATACAGATGTGTACA

GAACTCAAACTCAGCGACTATGAAGGGAGACTGATCCAGAACAGCATAACAATAGAGAGA

ATGGTACTTTCTGCATTTGATGAAAGAAGGAACAGGTATCTGGAAGAGCACCCCAGTGCA

GGAAAGGACCCTAAGAAAACTGGAGGTCCAATTTATCGGAGGAGAGACGGGAAATGGATT

AGAGAGCTGATTTTGTACGACAAAGAAGAGATCAGGAGGATTTGGCGCCAAGCAAACAAC

GGAGAGGACGCAACTGCTGGTCTTACCCATCTGATGATATGGCACTCCAATCTGAATGAT

GCCACATATCACAGAACAAGAGCTCTCGTTCGTACCGGAATGGACCCCAGGATGTGCTCC

CTAATGCAGGGATCAACTCTCCCGAGAAGATCTGGAGCTGCTGGTGCAGCAGTGAAGGGG

GTAGGAACAATGGTGATGGAGCTGATTCGAATGATAAAACGAGGGATTAACGACCGGAAT

TTCTGGAGAGGCGAAAATGGACGGAGAACAAGGATTGCATATGAGAGAATGTGCAACATC

CTCAAAGGGAAATTCCAAACAGCTGCACAACGAGCAATGATGGATCAAGTGCGAGAGAGC

AGGAATCCTGGGAATGCTGAGATTGAAGATCTTATTTTTCTGGCACGGTCTGCACTCATC

CTGAGAGGATCAGTGGCCCATAAGTCCTGCTTGCCTGCTTGTGTGTACGGACTTGCAGTG

GCCAGTGGGTATGATTTCGAGAGAGAAGGATACTCTCTGGTTGGGATAGATCCTTTCCGT

TTGCTTCAAAACAGCCAGGTCTTTAGTCTCATTAGGCCAAATGAAAACCCAGCACATAAG

AGTCAATTAGTGTGGATGGCATGCCACTCTGCAGCATTTGAGGACCTCAGAGTCTCAAGT

TTCATCAGAGGGACAAGAGTTGTCCCAAGAGGGCAGCTATCCACTAGAGGGGTTCAAATT

GCTTCAAATGAGAACATGGAAACAATGGACTCCAACACACTTGAACTGAGAAGTAGATAT

TGGGCTATAAGAACCAGGAGCGGAGGGAATACCAACCAGCAGAGGGCATCTGCAGGGCAG

ATTAGTGTTCAACCCACTTTCTCGGTGCAGAGAAACCTTCCCTTCGAAAGAGCGACCATT

ATGGCGGCATTTGCAGGAAATTCTGAAGGCAGAACGTCCGACATGAGGACAGAAATCATA

AGAATGATGGAAAATGCCAAACCAGAAGATGTGTCATTCCAGGGGCGGGGAGTCTTCGAG

CTCTCGGACGAAAAGGCAACGAGCCCGATCGTGCCTTCCTTTGACATGAATAATGAAGGA

TCTTATTTCTTCGGAGACAATGCAGAGGAGTATGACAATTAA------------------

------------------------------------------------------

>A_mute_swan_Czech_Republic_1410-2_2021_EPI1843607

------------------------TCACTGAGTGACATCAACATCATGGCGTCTCAAGGC

ACCAAACGATCTTATGAACAGATGGAAACTGGTGGGGAGCGCCAGAATGCCACTGAGATT

AGAGCATCGGTTGGAAGAATGGTTGGTGGAATCGGGAGGTTCTACATACAGATGTGCACT

GAGCTCAAACTCAGCGACTATGAAGGAAGACTGATCCAGAACAGCATAACAATAGAGAGA

ATGGTTCTCTCCGCATTTGATGAAAGGAGAAACAAATATCTGGAAGAACATCCCAGTGCG

GGGAAAGACCCGAAGAAAACAGGAGGTCCAATTTATCGAAGGAGAGATGGGAAATGGGTG

AGAGAGCTGATCCTGTATGACAAAGAGGAGATCAGGAGGATCTGGCGTCAAGCGAATAAT

GGAGAAGACGCAACTGCTGGTCTCACTCACCTGATGATCTGGCATTCCAATCTAAATGAT

GCCACATACCAGAGGACAAGAGCTCTCGTGCGTACCGGTATGGACCCCAGGATGTGCTCC

CTCATGCAAGGGTCAACTCTCCCAAGGAGGTCTGGAGCTGCTGGTGCAGCAGTGAAGGGA

GTTGGAACGATGGTGATGGAACTAATTCGGATGATAAAGCGAGGAGTTAATGATCGAAAC

TTCTGGAGAGGCGAGAATGGACGGAGGACAAGGATTGCATTTGAGAGAATGTGCAATATT

CTCAAAGGGAAATTCCAAACAGCAGCACAAAGAGCAATGATGGATCAAGTGCGTGAAAGC

AGGAATCCTGGCAATGCTGAAATTGAAGATCTCATTTTTCTGGCGCGGTCTGCTCTCGTT

CTGAGAGGATCAGTGGCCCATAAGTCCTGCTTGCCCGCTTGTGTATACGGGCTCGCTGTA

GCCAGTGGATACGACTTTGAGAGAGAAGGGTACTCTCTAGTTGGAATAGACCCTTTCCGA

CTGCTTCAAAACAGCCAGGTCTTTAGTCTCATTAGACCAAATGAGAATCCAGCACACAAG

AGTCAATTGGTGTGGATGGCATGTCATTCTGCAGCATTCGAGGATCTGAGAGTCTCAAGT

TTCATCAGAGGAACAAGAGTAGTCCCAAGAGGACAACTATCCACTAGAGGAGTTCAAATT

GCTTCAAATGAGAACATAGAAACAATGGACTCCAGCACTCTTGAACTGAGAAGCAGATAT

TGGGCTATAAGAACCAGGAGTGGAGGAAACACCAACCAACAGAGAGCATCTGCAGGACAA

ATCAGTGTACAGCCCACTTTCTCGGTACAGAGAAACCTTCCCTTCGAAAGAGCGACCATT

ATGGCGGCATTCACAGGGAACACTGAAGGCAGAACGTCCGACATGAGAACTGAAATCATA

AGAATGATGGAAAGTGCCAGACCAGAAGATGTGTCTTTCCAGGGGCGGGGAGTCTTCGAG

CTCTCGGACGAAAAGGCAACGAACCCGATCGTGCCTTCCTTTGACATGAGTAATGAAGGA

TCTTATTTCTTCGGAGACAATGCAGAGGAGTATGACA-----------------------

------------------------------------------------------

>A_chicken_Czech_Republic_1566-1_2021_EPI1844084

------------------------TCACTGAGTGACATCAACATCATGGCGTCTCAAGGC

ACCAAACGATCTTATGAACAGATGGAAACTAGTGGGGAGCGCCAGAATGCCACTGAGATT

AGAGCATCGGTTGGAAGAATGGTTGGTGGAATTGGGAGGTTCTACATACAGATGTGCACT

GAGCTCAAACTCAGCGACTATGAAGGAAGACTGATCCAGAACAGCATAACAATAGAGAGA

ATGGTTCTCTCCGCATTTGATGAAAGGAGAAACAAATATCTAGAAGAACATCCCAGTGCG

GGGAAAGACCCGAAGAAAACAGGAGGTCCAATTTATCGAAGGAGAGATGGGAAATGGGTG

AGAGAACTGATCCTGTATGACAAAGAGGAGATCAGGAGGATCTGGCGTCAAGCGAATAAT

GGAGAAGACGCAACTGCTGGTCTCACTCACCTGATGATCTGGCATTCCAATCTAAATGAT

GCCACATACCAGAGGACAAGAGCTCTCGTGCGTACCGGTATGGACCCCAGGATGTGCTCC

CTCATGCAAGGGTCAACTCTCCCAAGGAGGTCTGGAGCTGCTGGTGCAGCAGTGAAGGGA

GTTGGAACGATGGTGATGGAACTAATTCGGATGATAAAGCGAGGAGTTAATGATCGAAAC

TTCTGGAGAGGCGAGAATGGACGGAGGACAAGGATTGCATTTGAGAGAATGTGCAATATT

CTCAAAGGGAAATTCCAAACAGCAGCACAAAGAGCAATGATGGATCAAGTGCGTGAAAGC

AGGAATCCTGGCAATGCTGAAATTGAAGATCTCATTTTTCTGGCGCGGTCTGCTCTCATT

CTGAGAGGATCAGTGGCCCATAAGTCCTGCTTGCCCGCTTGTGTATACGGGCTCGCTGTA

GCCAGTGGATACGACTTTGAGAGAGAAGGGTACTCTCTAGTTGGAATAGACCCTTTCCGA

CTGCTTCAAAACAGCCAGGTCTTTAGTCTCATTAGACCAAATGAGAATCCAGCACACAAG

AGCCAATTGGTGTGGATGGCATGTCATTCTGCAGCATTCGAAGATCTGAGAGTCTCAAGT

TTCATCAGAGGAACAAGAGTAGTCCCAAGAGGACAACTATCCACTAGAGGAGTTCAAATT

GCTTCAAATGAGAACATAGAAACAATGGACTCCAGCACTCTTGAACTGAGAAGCAGATAT

TGGGCTATAAGAACCAGGAGTGGAGGAAACACCAACCAACAGAGAGCATCTGCAGGACAA

ATCAGTGTACAGCCCACTTTCTCGGTACAGAGAAACCTTCCCTTCGAAAGAGCGACCATT

ATGGCGGCATTCACAGGGAACACTGAAGGCAGAACGTCCGACATGAGGACTGAAATCATA

AGAATGATGGAAAGTGCCAGACCAGAAGATGTGTCTTTCCAGGGGCGGGGAGTCTTCGAG

CTCTCGGACGAAAAGGCAACGAACCCGATCGTGCCTTCCTTTGACATGAGTAATGAAGGA

TCTTATTTCTTCGGAGACAATGCAGAGGAGTATGACA-----------------------

------------------------------------------------------

>A_chicken_Korea_H008_2021_EPI1846531

---------------------------------------------ATGGCGTCTCAAGGC

ACCAAACGATCTTATGAACAGATGGAAACTAGTGGGGAGCGCCAGAATGCCACTGAGATT

AGAGCATCGGTTGGAAGAATGGTTGGTGGAATTGGGAGGTTCTACATACAGATGTGCACT

GAGCTCAAACTCAGCGACTATGAAGGAAGACTGATCCAGAACAGCATAACAATAGAGAGA

ATGGTTCTCTCCGCATTTGATGAAAGGAGAAACAAATATCTGGAAGAACATCCCAGTGCG

GGGAAAGACCCGAAGAAAACAGGAGGTCCAATTTATCGAAGGAGAGATGGGAAATGGGTG

AGAGAGCTGATCCTGTATGACAAAGAGGAGATCAGGAGGATCTGGCGTCAAGCGAACAAT

GGAGAAGACGCAACTGCTGGTCTCACTCATCTGATGATCTGGCATTCCAATCTAAATGAT

GCCACATACCAGAGGACAAGAGCTCTCGTGCGTACCGGTATGGACCCCAGGATGTGCTCC

CTCATGCAAGGGTCAACTCTCCCAAGGAGGTCTGGAGCTGCTGGTGCAGCAGTGAAGGGA

GTTGGAACGATGGTGATGGAACTAATTCGGATGATAAAGCGAGGAGTTAATGATCGAAAC

TTCTGGAGAGGCGAGAATGGACGGAGGACAAGGATTGCATTTGAGAGAATGTGCAATATT

CTCAAAGGGAAATTCCAAACAGCAGCACAAAGAGCAATGATGGATCAAGTGCGTGAAAGC

AGGAATCCTGGCAATGCTGAAATTGAAGATCTCATTTTTCTGGCGCGGTCTGCTCTCATT

CTGAGAGGATCAGTGGCCCATAAGTCCTGCTTGCCCGCTTGTGTATACGGGCTCGCTGTA

GCCAGTGGATACGACTTTGAGAGAGAAGGGTACTCTCTAGTTGGAATAGACCCTTTCCGA

CTGCTTCAAAACAGCCAGGTCTTTAGTCTCATTAGACCAAATGAGAATCCAGCACACAAG

AGTCAATTGGTGTGGATGGCATGTCATTCTGCAGCATTCGAGGATCTGAGAGTCTCAAGT

TTCATCAGAGGAACAAGAGTAGTCCCAAGAGGACAACTATCCACTAGAGGAGTTCAAATT

GCTTCAAATGAGAACATAGAAACAATGGACTCCAGCACTCTTGAACTGAGAAGCAGATAC

TGGGCTATAAGAACCAGGAGTGGAGGAAACACCAACCAACAGAGAGCATCTGCAGGACAA

ATCAGTGTACAGCCCACTTTCTCGGTACAGAGAAACCTTCCCTTCGAAAGAGCGACCATT

ATGGCGGCATTCACAGGGAACACTGAAGGCAGAACGTCCGACATGAGGACTGAAATCATA

AGAATGATGGAAAGTGCCAGACCAGAAGATGTGTCTTTCCAGGGGCGGGGAGTCTTCGAG

ATCTCGGACGAAAAGGCAACGAACCCGATCGTGCCTTCCTTTGACATGAGTAATGAAGGA

TCTTATTTCTTCGGAGACAATGCAGAGGAGTATGACAATTAA------------------

------------------------------------------------------

>A_mallard_Korea_WA820_2020_EPI1846595

---------------------------------------------ATGGCGTCTCAAGGC

ACCAAACGATCTTATGAACAGATGGAAACTAGTGGGGAGCGCCAGAATGCCACTGAGATT

AGAGCATCGGTTGGAAGAATGGTTGGTGGAATCGGGAGGTTCTACATACAGATGTGCACT

GAGCTCAAACTCAGCGACTATGAAGGAAGACTGATCCAGAACAGCATAACAATAGAGAGA

ATGGTTCTCTCCGCATTTGATGAAAGGAGAAACAAATATCTGGAAGAACATCCCAGTGCG

GGGAAAGACCCGAAGAAAACAGGAGGTCCAATTTATCGAAGGAGAGATGGGAAATGGGTG

AGAGAGCTGATCCTGTATGACAAAGAGGAGATCAGGAGGATCTGGCGTCAAGCGAACAAT

GGAGAAGACGCAACTGCTGGTCTCACTCACCTGATGATCTGGCATTCCAATCTAAATGAT

GCCACATACCAGAGGACAAGAGCTCTCGTGCGTACCGGTATGGACCCCAGGATGTGCTCC

CTCATGCAAGGGTCAACTCTCCCAAGGAGGTCTGGAGCTGCTGGTGCAGCAGTGAAGGGA

GTTGGAACGATGGTGATGGAAATAATTCGGATGATAAAGCGAGGAGTTAATGATCGAAAC

TTCTGGAGAGGCGAGAATGGACGGAGGACAAGGATTGCATTTGAGAGAATGTGCAATATT

CTCAAAGGGAAATTCCAAACAGCAGCACAAAGAGCAATGATGGATCAAGTGCGTGAAAGC

AGGAATCCTGGCAATGCTGAAATTGAAGATCTCATTTTTCTGGCGCGGTCTGCTCTCATT

CTGAGAGGATCAGTGGCCCATAAGTCCTGCTTGCCCGCTTGTGTATACGGGCTCGCTGTA

GCCAGTGGATACGACTTTGAGAGAGAAGGGTACTCTCTAGTTGGAATAGACCCTTTCCGA

CTGCTTCAAAACAGCCAGGTCTTTAGTCTCATTAGACCAAATGAGAATCCAGCACACAAG

AGTCAATTGGTGTGGATGGCATGTCATTCTGCAGCATTCGAGGATCTGAGAGTCTCAAGT

TTCATCAGAGGAACAAGAGTAGTCCCAAGAGGACAACTATCCACTAGAGGAGTTCAAATT

GCTTCAAATGAGAACATAGAAACAATGGACTCCAGCACTCTTGAACTGAGAAGCAGATAC

TGGGCTATAAGAACCAGGAGTGGAGGAAACACCAACCAACAGAGAGCATCTGCAGGACAA

ATCAGTGTACAGCCCACTTTCTCGGTACAGAGAAACCTTCCCTTCGAAAGAGCGACCATT

ATGGCGGCATTCACAGGGAACACTGAAGGCAGAACGTCCGACATGAGGACTGAAATCATA

AGAATGATGGAAAGTGCCAGACCAGAAGATGTGTCTTTCCAGGGGCGGGGAGTCTTCGAG

ATCTCGGACGAAAAGGCAACGAACCCGATCGTGCCTTCCTTTGACATGAGTAATGAAGGA

TCTTATTTCTTCGGAGACAATGCAGAGGAGTATGACAATTAA------------------

------------------------------------------------------

>A_duck_Korea_H016_2021_EPI1846699

---------------------------------------------ATGGCGTCTCAAGGC

ACCAAACGATCTTATGAACAGATGGAAACTAGTGGGGAGCGCCAAAATGCCACTGAGATT

AGAGCATCGGTTGGAAGAATGGTTGGTGGAATCGGGAGGTTCTACATACAGATGTGCACT

GAGCTCAAACTCAGCGACTATGAAGGAAGACTGATCCAGAACAGCATAACAATAGAGAGA

ATGGTTCTCTCCGCATTTGATGAAAGGAGAAACAAATATCTGGAAGAACATCCCAGTGCG

GGGAAAGACCCGAAGAAAACAGGAGGTCCAATTTATCGAAGGAGAGATGGGAAATGGGTG

AGAGAGCTGATCCTGTATGACAAAGAGGAGATCAGGAGGATCTGGCGTCAAGCGAACAAT

GGAGAAGACGCAACTGCTGGTCTCACTCACCTGATGATCTGGCATTCCAATCTAAATGAT

GCCACATACCAGAGGACAAGAGCTCTCGTGCGTACCGGTATGGACCCCAGGATGTGCTCC

CTCATGCAAGGGTCAACTCTCCCAAGGAGGTCTGGAGCTGCTGGTGCAGCAGTGAAGGGA

GTTGGAACGATGGTGATGGAAATAATTCGGATGATAAAGCGAGGAGTTAATGATCGAAAC

TTCTGGAGAGGCGAGAATGGACGGAGGACAAGGATTGCATTTGAGAGAATGTGCAATATT

CTCAAAGGGAAATTCCAAACAGCAGCACAAAGAGCAATGATGGATCAAGTGCGTGAAAGC

AGGAATCCTGGCAATGCTGAAATTGAAGATCTCATTTTTCTGGCGCGGTCTGCTCTCATT

CTGAGAGGATCAGTGGCCCATAAGTCCTGCTTGCCCGCTTGTGTATACGGGCTCGCTGTA

GCCAGTGGATACGACTTTGAGAGAGAAGGGTACTCTCTAGTTGGAATAGACCCTTTCCGA

CTGCTTCAAAACAGCCAGGTCTTTAGTCTCATTAGACCAAATGAGAATCCAGCACACAAG

AGTCAATTGGTGTGGATGGCATGTCATTCTGCAGCATTCGAGGATCTGAGAGTCTCAAGT

TTCATCAGAGGAACAAGAGTAGTCCCAAGAGGACAACTATCCACTAGAGGAGTTCAAATT

GCTTCAAATGAGAACATAGAAACAATGGACTCCAGCACTCTTGAACTGAGAAGCAGATAC

TGGGCTATAAGAACCAGGAGTGGAGGAAACACCAACCAACAGAGAGCATCTGCAGGACAA

ATCAGTGTACAGCCCACTTTCTCGGTACAGAGAAACCTTCCCTTCGAAAGAGCGACCATT

ATGGCGGCATTCACAGGGAACACTGAAGGCAGAACGTCCGACATGAGGACTGAAATCATA

AGAATGATGGAAAGTGCCAGACCAGAAGATGTGTCTTTCCAGGGGCGGGGAGTCTTCGAG

ATCTCGGACGAAAAGGCAACGAACCCGATCGTGCCTTCCTTTGACATGAGTAATGAAGGA

TCTTATTTCTTCGGAGACAATGCAGAGGAGTATGACAATTAA------------------

------------------------------------------------------

>A_chicken_Astrakhan_321-01_2020_EPI1846970

AGCAAAAGCAGGGTAGATAATCACTCACTGAGTGACATCAACATCATGGCGTCTCAAGGC

ACCAAACGATCTTATGAACAGATGGAAACTAGTGGGGAGCGCCAGAATGCCACTGAGATT

AGAGCATCTGTTGGAAGAATGGTTGGTGGAATTGGGAGGTTCTACATACAGATGTGCACT

GAGCTCAAACTCAGCGACTATGAAGGAAGACTGATCCAGAACAGCATAACAATAGAGAGA

ATGGTTCTCTCCGCATTTGATGAAAGGAGAAACAAATATCTGGAAGAACATCCCAGTGCG

GGGAAAGACCCGAAGAAAACAGGAGGTCCAATTTATCGAAGGAGAGATGGGAAATGGGTG

AGAGAGCTGATCCTGTATGACAAAGAGGAGATCAGGAGGATCTGGCGTCAAGCGAATAAT

GGAGAAGACTCAACTGCTGGTCTCACTCACCTGATGATCTGGCATTCCAATCTAAATGAT

GCCACATACCAGAGGACAAGAGCTCTCGTGCGTACCGGTATGGACCCCAGGATGTGCTCC

CTCATGCAAGGGTCAACTCTCCCAAGGAGGTCTGGAGCTGCTGGTGCAGCAGTGAAGGGA

GTAGGAACGATGGTGATGGAACTAATTCGGATGATAAAGCGAGGAGTTAATGATCGAAAC

TTCTGGAGAGGCGAGAATGGACGGAGGACAAGGATTGCATTTGAGAGAATGTGCAATATT

CTCAAAGGGAAATTCCAAACAGCAGCACAAAGAGCAATGATGGATCAAGTGCGTGAAAGC

AGGAATCCTGGCAATGCTGAAATTGAAGATCTCATTTTTCTGGCGCGGTCTGCTCTCATT

CTGAGAGGATCAGTGGCCCATAAGTCCTGCTTGCCCGCTTGTGTATACGGGCTCGCTGTA

GCCAGTGGATACGACTTTGAGAGAGAAGGGTACTCTCTAGTTGGAATAGACCCTTTCCGA

CTGCTTCAAAACAGCCAGGTCTTTAGTCTCATTAGACCAAATGAGAATCCAGCACACAAG

AGTCAATTGGTGTGGATGGCATGTCATTCTGCAGCATTCGAGGATCTGAGAGTCTCAAGT

TTCATCAGAGGAACAAGAGTAGTCCCAAGAGGACAACTATCCACTAGAGGAGTTCAAATT

GCTTCAAATGAGAACATAGAAACAATGGACTCCAGCACTCTTGAACTGAGAAGTAGATAT

TGGGCTATAAGAACCAGGAGTGGAGGAAACACCAACCAACAGAGAGCATCTGCAGGACAA

ATCAGTGTACAGCCCACTTTCTCGGTACAGAGAAACCTTCCCTTCGAAAGAGCGACCATT

ATGGCGGCATTCACAGGGAACACTGAAGGCAGAACGTCCGACATGAGGACTGAAATCATA

AGAATGATGGAAAGTGCCAGACCAGAAGATGTGTCTTTCCAGGGGCGGGGAGTCTTCGAG

CTCTCAGACGAAAAGGCAACGAACCCGATCGTGCCTTCCTTTGACATGAGTAATGAAGGA

TCTTATTTCTTCGGAGACAATGCAGAGGAGTATGACAATTAAAGAAAAATACCCTTGTTT

CTACT-------------------------------------------------

>A_crane_Kagoshima_KU-93_2021_EPI1848528

---------------------------------------------ATGGCGTCTCAAGGC

ACCAAACGATCTTATGAACAGATGGAAACTAGTGGGGAGCGCCAGAATGCCACTGAGATT

AGAGCATCGGTTGGAAGAATGGTTGGTGGAATTGGGAGGTTCTACATACAGATGTGCACT

GAGCTCAAACTCAGCGACTATGAAGGAAGACTGATCCAGAACAGCATAACAATAGAGAGA

ATGGTTCTCTCCGCATTTGATGAAAGGAGAAACAAATATCTGGAAGAACATCCCAGTGCG

GGGAAAGACCCGAAGAAAACAGGAGGTCCAATTTATCGAAGGAGAGATGGGAAATGGGTG

AGAGAGCTGATCCTGTATGACAAAGAGGAGATCAGGAGGATCTGGCGTCAAGCGAACAAT

GGAGAAGACGCAACTGCTGGTCTCACTCATCTGATGATCTGGCATTCCAATCTAAATGAT

GCCACATACCAGAGGACAAGAGCTCTCGTGCGTACCGGTATGGACCCCAGGATGTGCTCC

CTCATGCAAGGGTCAACTCTCCCAAGGAGGTCTGGAGCTGCTGGTGCAGCAGTGAAGGGA

GTTGGAACGATGGTGATGGAACTAATTCGGATGATAAAGCGAGGAGTTAATGATCGAAAC

TTCTGGAGAGGCGAGAATGGACGGAGGACAAGGATTGCATTTGAGAGAATGTGCAATATT

CTCAAAGGGAAATTCCAAACAGCAGCACAAAGAGCAATGATGGATCAAGTGCGTGAAAGC

AGGAATCCTGGCAATGCTGAAATTGAAGATCTCATYTTTCTGGCRCGGTCTGCTCTCATY

CTGAGAGGATCAGTGGCCCATAAGTCCTGCTTGCCCGCTTGTGTATACGGGCTCGCTGTA

GCCAGTGGATACGACTTTGAGAGAGAAGGGTACTCTCTAGTTGGAATAGAYCCTTTCCGA

CTGCTTCAAAACAGCCAGGTCTTTAGTCTCATTAGACCAAATGAGAACCCAGCACACAAG

AGTCAATTGGTGTGGATGGCATGYCATTCTGCAGCATTYGAGGATCTGAGAGTCTCAAGT

TTCATCAGAGGAACAAGAGTRGTCCCAAGAGGACAACTATCCACYAGAGGAGTTCAAATT

GCTTCAAATGAGAACATRGAAACAATGGACTCCAGCACTCTTGAACTGAGRAGCAGATAY

TGGGCTATAAGAACCAGGAGTGGAGGAAACACYAACCAACAGAGAGCATCTGCAGGACAR

ATCAGTGTACARCCYACTTTCTCGGTACAGAGAAATCTYCCCTTCGAAAGAGCRACCATT

ATGGCGGCRTTCACAGGRAAYACTGARGGCAGAACRTCYGACATGAGGACTGAAATCATA

AGAATGATGGAAAGTGCCAGACCAGAAGATGTGTCYTTCCAGGGGCGGGGAGTCTTCGAG

MTCTCGGACGAAAAGGCAACGAACCCGATCGTGCCTTCCTTTGACATGAGTAATGAAGGA

TCTTATTTCTTCGGAGACAATGCAGAGGAGTATGACAATTAA------------------

------------------------------------------------------

>A_mallard_Kagoshima_KU-d89_2021_EPI1848539

---------------------------------------------ATGGCGTCTCAAGGC

ACCAAACGATCTTATGAACAGATGGAAACTAGTGGGGAGCGCCAGAATGCCACTGAGATT

AGAGCATCGGTTGGAAGAATGGTTGGTGGAATTGGGAGGTTCTACATACAGATGTGCACT

GAGCTCAAACTCAGCGACTATGAAGGAAGACTGATCCAGAACAGCATAACAATAGAGAGA

ATGGTTCTCTCCGCATTTGATGAAAGGAGAAACAAATATCTGGAAGAACATCCCAGTGCG

GGGAAAGACCCGAAGAAAACAGGAGGTCCAATTTATCGAAGGAGAGATGGGAAATGGGTG

AGAGAGCTGATCCTGTATGACAAAGAGGAGATCAGGAGGATCTGGCGTCAAGCGAACAAT

GGAGAAGACGCAACTGCTGGTCTCACTCATCTGATGATCTGGCATTCCAATCTAAATGAT

GCCACATACCAGAGGACAAGAGCTCTCGTGCGTACCGGTATGGACCCCAGGATGTGCTCC

CTCATGCAAGGGTCAACTCTCCCAAGGAGGTCTGGAGCTGCTGGTGCAGCAGTGAAGGGA

GTTGGAACGATGGTGATGGAACTAATTCGGATGATAAAGCGAGGAGTTAATGATCGAAAC

TTCTGGAGAGGCGAGAATGGACGGAGGACAAGGATTGCATTTGAGAGAATGTGCAATATT

CTCAAAGGGAAATTCCAAACAGCAGCACAAAGAGCAATGATGGATCAAGTGCGTGAAAGC

AGGAATCCTGGCAATGCTGAAATTGAAGATCTCATTTTTCTGGCGCGGTCTGCTCTCATT

CTGAGAGGATCAGTGGCCCATAAGTCCTGCTTGCCCGCTTGTGTATACGGGCTCGCTGTA

GCCAGTGGATACGACTTTGAGAGAGAAGGGTACTCTCTAGTTGGAATAGACCCTTTCCGA

CTGCTTCAAAACAGCCAGGTCTTTAGTCTCATTAGACCAAATGAGAATCCAGCACACAAG

AGTCAATTGGTGTGGATGGCATGTCATTCTGCAGCATTCGAGGATCTGAGAGTCTCAAGT

TTCATCAGAGGAACAAGAGTAGTCCCAAGAGGACAACTATCCACTAGAGGAGTTCAAATT

GCTTCAAATGAGAACATAGAAACAATGGACTCCAGCACTCTTGAACTGAGAAGCAGATAC

TGGGCTATAAGAACCAGGAGTGGAGGAAACACCAACCAACAGAGAGCATCTGCAGGACAA

ATCAGTGTACAGCCCACTTTCTCGGTACAGAGAAACCTTCCCTTCGAAAGAGCGACCATT

ATGGCGGCATTCACAGGGAACACTGAAGGCAGAACGTCCGACATGAGGACTGAAATCATA

AGAATGATGGAAAGTGCCAGACCAGAAGATGTGTCTTTCCAGGGGCGGGGAGTCTTCGAG

ATCTCGGACGAAAAGGCAACGAACCCGATCGTGCCTTCCTTTGACATGAGTAATGAAGGA

TCTTATTTCTTCGGAGACAATGCAGAGGAGTATGACAATTAA------------------

------------------------------------------------------

>A_chicken_Kostroma_304-06_2020_EPI1848639

AGCAAAAGCAGGGTAGATAATCACTCACTGAGTGACATCAACATCATGGCGTCTCAAGGC

ACCAAACGATCTTATGAACAGATGGAAACTAGTGGGGAGCGCCAGAATGCCACTGAGATT

AGAGCATCGGTTGGAAGAATGGTTGGTGGAATTGGGAGGTTCTACATACAGATGTGCACT

GAGCTCAAACTCAGCGACTATGAAGGAAGACTGATCCAGAACAGCATAACAATAGAGAGA

ATGGTTCTCTCCGCATTTGATGAAAGGAGAAACAAATATCTGGAAGAGCATCCCAGTGCG

GGGAAAGACCCGAAGAAAACAGGAGGTCCAATTTATCGAAGGAGAGATGGGAAATGGGTG

AGAGAGCTGATCCTGTATGACAAAGAAGAGATCAGGAGGATCTGGCGTCAAGCGAACAAT

GGAGAAGACGCAACTGCTGGTCTCACTCACCTGATGATCTGGCATTCCAATCTAAATGAT

GCCACATACCAGAGGACAAGAGCTCTCGTGCGTACCGGTATGGACCCCAGGATGTGCTCC

CTCATGCAAGGGTCAACTCTCCCAAGGAGGTCTGGAGCTGCTGGTGCAGCAGTGAAGGGA

GTTGGAACGATGGTGATGGAACTAATTCGGATGATAAAGCGAGGAGTTAATGATCGAAAC

TTCTGGAGAGGCGAGAATGGACGGAGGACAAGGATTGCATTTGAGAGAATGTGCAATATT

CTCAAAGGGAAATTCCAAACAGCAGCACAAAGAGCAATGATGGATCAAGTGCGTGAAAGC

AGGAATCCTGGCAATGCTGAAATTGAAGATCTCATTTTTCTGGCGCGGTCTGCTCTCATT

CTGAGAGGATCAGTGGCCCATAAGTCCTGCTTGCCCGCTTGTGTATACGGGCTCGCTGTA

GCCAGTGGATACGACTTTGAGAGAGAAGGGTACTCTCTAGTTGGAATAGACCCTTTCCGA

CTGCTTCAAAACAGCCAGGTCTTTAGTCTCATTAGACCAAATGAGAATCCAGCACACAAG

AGTCAATTGGTGTGGATGGCATGTCATTCTGCAGCATTCGAGGATCTGAGAGTCTCAAGT

TTCATCAGAGGAACAAGAGTAGTCCCAAGAGGACAACTATCCACTAGAGGAGTTCAAATT

GCTTCAAATGAGAACATAGAAACAATGGACTCCAGCACTCTTGAACTGAGAAGCAGATAT

TGGGCTATAAGAACCAGGAGTGGAGGAAACACCAACCAACAGAGAGCATCTGCAGGACAA

ATCAGTGTACAGCCCACTTTCTCGGTACAGAGAAACCTTCCCTTCGAAAGAGCGACCATT

ATGGCGGCATTCACAGGGAACACTGAAGGCAGAACGTCCGACATGAGGACTGAAATCATA

AGAATGATGGAAAGTGCCAGACCAGAAGATGTGTCTTTCCAGGGGCGGGGAGTCTTCGAG

ATCTCGGACGAAAAGGCAACGAACCCGATCGTGCCTTCCTTTGACATGAGTAATGAAGGA

TCTTATTTCTTCGGAGACAATGCAGAGGAGTATGACAATTAAAGAAAAATACCCTTGTTT

CTACT-------------------------------------------------

>A_chicken_Rostov-on-Don_308-02_2020_EPI1848663

AGCAAAAGCAGGGTAGATAATCACTCACTGAGTGACATCAACATCATGGCGTCTCAAGGC

ACCAAACGATCTTATGAACAGATGGAAACTGGTGGGGAGCGCCAGAATGCCACTGAGATT

AGAGCATCGGTTGGAAGAATGGTTGGTGGAATTGGGAGGTTCTACATACAGATGTGCACT

GAGCTCAAACTCAGCGACTATGAAGGAAGACTGATCCAGAACAGCATAACAATAGAGAGA

ATGGTTCTCTCCGCATTTGATGAAAGGAGAAACAAATATCTGGAAGAACATCCCAGTGCG

GGGAAAGACCCGAAGAAAACAGGAGGTCCAATTTATCGAAGGAGAGATGGGAAATGGGTG

AGAGAGCTGATCCTGTATGACAAAGAGGAGATCAGGAGGATCTGGCGTCAAGCGAATAAT

GGAGAAGACGCAACTGCTGGTCTCACTCACCTGATGATCTGGCATTCCAATCTAAATGAT

GCCACATACCAGAGGACAAGAGCTCTCGTACGTACCGGTATGGACCCCAGGATGTGCTCC

CTCATGCAAGGGTCAACTCTCCCAAGGAGGTCTGGAGCTGCTGGTGCAGCAGTGAAGGGA

GTTGGAACGATGGTGATGGAACTAATTCGGATGATAAAGCGAGGAGTTAATGATCGAAAC

TTCTGGAGAGGCGAGAATGGACGGAGGACAAGGATTGCATTTGAGAGAATGTGCAATATT

CTCAAAGGGAAATTCCAAACAGCAGCACAAAGAGCAATGATGGATCAAGTGCGTGAAAGC

AGGAATCCTGGCAATGCTGAAATTGAAGATCTCATTTTTCTGGCGCGGTCTGCTCTCATT

CTGAGAGGATCAGTGGCCCATAAGTCCTGCTTGCCCGCTTGTGTATACGGGCTCGCTGTA

GCCAGTGGATACGACTTTGAGAGAGAAGGGTACTCTCTAGTTGGAATAGACCCTTTCCGA

CTGCTTCAAAACAGCCAGGTCTTTAGTCTCATTAGACCAAATGAGAATCCAGCACACAAG

AGTCAATTGGTGTGGATGGCATGTCATTCTGCAGCATTCGAGGATCTGAGAGTCTCAAGT

TTCATCAGAGGAACAAGAGTAGTCCCAAGAGGACAACTATCCACTAGAGGAGTTCAAATT

GCTTCAAATGAGAACATAGAAACAATGGACTCCAGCACTCTTGAACTGAGAAGCAGATAT

TGGGCTATAAGAACCAGGAGTGGAGGAAACACCAACCAACAGAGAGCATCTGCAGGACAA

ATCAGTGTACAGCCCACTTTCTCGGTACAGAGAAACCTTCCCTTCGAAAGAGCGACCATT

ATGGCGGCATTCACAGGGAACACTGAAGGCAGAACGTCCGACATGAGGACTGAAATAATA

AGAATGATGGAAAGTGCCAAACCAGAAGATGTGTCTTTCCAGGGGCGGGGAGTCTTCGAG

CTCTCGGACGAAAAGGCAACGAACCCGATCGTGCCTTCCTTTGACATGAGTAATGAAGGA

TCTTATTTCTTCGGAGACAATGCAGAGGAGTATGACAATTAAAGAAAAATACCCTTGTTT

CTACT-------------------------------------------------

>A_turkey_Stavropol_320-02_2020_EPI1848695

AGCAAAAGCAGGGTAGATAATCACTCACTGAGTGACATCAACATCATGGCGTCTCAAGGC

ACCAAACGATCTTATGAACAGATGGAAACTGGTGGGGAGCGCCAGAATGCCACTGAGATT

AGAGCATCGGTTGGAAGAATGGTTGGTGGAATTGGGAGGTTCTACATACAGATGTGCACT

GAGCTCAAACTCAGCGACTATGAAGGAAGACTGATCCAGAACAGCATAACAATAGAGAGA

ATGGTTCTCTCCGCATTTGATGAAAGGAGAAACAAATATCTGGAAGAACATCCCAGTGCG

GGGAAAGACCCGAAGAAAACAGGAGGTCCAATTTATCGAAGGAGAGATGGGAAATGGGTG

AGAGAGCTGATCCTGTATGACAAAGAGGAGATCAGGAGGATCTGGCGTCAAGCGAATAAT

GGAGAAGACGCAACTGCTGGTCTCACTCACCTGATGATCTGGCATTCCAATCTAAATGAT

GCCACATACCAGAGAACAAGAGCTCTCGTACGTACCGGTATGGACCCCAGGATGTGCTCC

CTCATGCAAGGGTCAACTCTCCCAAGGAGGTCTGGAGCTGCTGGTGCAGCAGTGAAGGGA

GTTGGAACGATGGTGATGGAACTAATTCGGATGATAAAGCGAGGAGTTAATGATCGAAAC

TTCTGGAGAGGCGAGAATGGACGGAGGACAAGGATTGCATTTGAGAGAATGTGCAATATT

CTCAAAGGGAAATTCCAAACAGCAGCACAAAGAGCAATGATGGATCAAGTGCGTGAAAGC

AGGAATCCTGGCAATGCTGAAATTGAAGATCTCATTTTTCTGGCGCGGTCTGCTCTCATT

CTGAGAGGATCAGTGGCCCATAAGTCCTGCTTGCCCGCTTGTGTATACGGGCTCGCTGTA

GCCAGTGGATACGACTTTGAGAGAGAAGGGTACTCTCTAGTTGGAATAGACCCTTTCCGA

CTGCTTCAAAACAGCCAGGTCTTTAGTCTCATTAGACCAAATGAGAATCCAGCACACAAG

AGTCAATTGGTGTGGATGGCATGTCATTCTGCAGCATTCGAGGATCTGAGAGTCTCAAGT

TTCATCAGAGGAACAAGAGTAGTCCCAAGAGGACAACTATCCACTAGAGGAGTTCAAATT

GCTTCAAATGAGAACATAGAAACAATGGACTCCAGCACTCTTGAACTGAGAAGCAGATAT

TGGGCTATAAGAACCAGGAGTGGAGGAAACACCAACCAACAGAGAGCATCTGCAGGACAA

ATCAGTGTACAGCCCACTTTCTCGGTACAGAGAAACCTTCCCTTCGAAAGAGCGACCATT

ATGGCGGCATTCACAGGGAACACTGAAGGCAGAACGTCCGACATGAGGACTGAAATCATA

AGAATGATGGAAAGTGCCAAACCAGAAGATGTGTCTTTCCAGGGGCGGGGAGTCTTCGAG

CTCTCGGACGAAAAGGCAACGAACCCGATCGTGCCTTCCTTTGACATGAGTAATGAAGGA

TCTTATTTCTTCGGAGACAATGCAGAGGAGTATGACAATTAAAGAAAAATACCCTTGTTT

CTACT-------------------------------------------------

>A_mute_swan_North_Ossetia-Alania_325-03_2020_EPI1848727

AGCAAAAGCAGGGTAGATAATCACTCACTGAGTGACATCAACATCATGGCGTCTCAAGGC

ACCAAACGATCTTATGAACAGATGGAAACTAGTGGGGAGCGCCAGAATGCCACTGAGATT

AGAGCATCGGTTGGAAGAATGGTTGGTGGAATTGGGAGGTTCTACATACAGATGTGCACT

GAGCTCAAACTCAGCGACTATGAAGGAAGACTGATCCAGAACAGCATAACAATAGAGAGA

ATGGTTCTCTCCGCATTTGATGAAAGGAGAAACAAATATCTGGAAGAACATCCCAGTGCG

GGGAAAGACCCGAAGAAAACAGGAGGTCCAATTTATCGAAGGAGAGATGGGAAATGGGTG

AGAGAGCTGATCCTGTATGACAAAGAGGAGATCAGGAGGATCTGGCGTCAAGCGAATAAT

GGAGAAGACGCAACTGCTGGTCTCACTCACCTGATGATCTGGCATTCCAATCTAAATGAT

GCCACATACCAGAGGACAAGAGCTCTCGTGCGTACCGGTATGGACCCCAGGATGTGCTCC

CTCATGCAAGGGTCAACTCTCCCAAGGAGGTCTGGAGCTGCTGGTGCAGCAGTGAAGGGA

GTTGGAACGATGGTGATGGAACTAATTCGGATGATAAAGCGAGGAGTTAATGATCGAAAC

TTCTGGAGAGGCGAGAATGGACGGAGGACAAGGATTGCATTTGAGAGAATGTGCAATATT

CTCAAAGGGAAATTCCAAACAGCAGCACAAAGAGCAATGATGGATCAAGTGCGTGAAAGC

AGGAATCCTGGCAATGCTGAAATTGAAGATCTCATTTTTCTGGCGCGGTCTGCTCTCATT

CTGAGAGGATCAGTGGCCCATAAGTCCTGCTTGCCCGCTTGTGTATACGGGCTCGCTGTA

GCCAGTGGATACGACTTTGAGAGAGAAGGGTACTCTCTAGTTGGAATAGACCCTTTCCGA

CTGCTTCAAAACAGCCAGGTCTTTAGTCTCATTAGACCAAATGAGAATCCAGCACACAAG

AGTCAATTGGTGTGGATGGCATGTCATTCTGCAGCATTCGAGGATCTGAGAGTCTCAAGT

TTCATCAGAGGAACAAGAGTAGTCCCAAGAGGACAACTATCCACTAGAGGAGTTCAAATT

GCTTCAAATGAGAACATAGAAACAATGGACTCCAGCACTCTTGAACTGAGAAGCAGATAT

TGGGCTATAAGAACCAGGAGTGGAGGAAACACCAACCAACAGAGAGCATCTGCAGGACAA

ATCAGTGTACAGCCCACTTTCTCGGTACAGAGAAACCTTCCCTTCGAAAGAGCGACCATT

ATGGCGGCATTCACAGGGAACACTGAAGGCAGAACGTCCGACATGAGGACTGAAATCATA

AGAATGATGGAAAGTGCCAGACCAGAAGATGTGTCTTTCCAGGGGCGGGGAGTCTTCGAG

CTCTCGGACGAAAAGGCAACGAACCCGATCGTGCCTTCCTTTGACATGAGTAATGAAGGA

TCTTATTTCTTCGGAGACAATGCAGAGGAGTATGACAATTAAAGAAAAATACCCTTGTTT

CTACT-------------------------------------------------

>A_turkey_Rostov-on-Don_332-09_2021_EPI1848751

AGCAAAAGCAGGGTAGATAATCACTCACTGAGTGACATCAACATCATGGCGTCTCAAGGC

ACCAAACGATCTTATGAACAGATGGAAACTAGTGGGGAGCGCCAGAATGCCACTGAGATT

AGAGCATCGGTTGGAAGAATGGTTGGTGGAATTGGGAGGTTCTACATACAGATGTGCACT

GAGCTCAAACTCAGCGACTATGAAGGAAGACTGATCCAGAACAGCATAACAATAGAGAGA

ATGGTTCTCTCCGCATTTGATGAAAGGAGAAACAAATATCTGGAAGAACATCCCAGTGCG

GGGAAAGACCCGAAGAAAACAGGAGGTCCAATTTATCGAAGGAGAGATGGGAAATGGGTA

AGAGAGCTGATCCTGTATGACAAAGAGGAGATCAGGAGGATCTGGCGTCAAGCGAACAAT

GGAGAAGACGCAACTGCTGGTCTCACTCACCTGATGATCTGGCATTCCAATCTAAATGAT

GCCACATACCAGAGGACAAGAGCTCTCGTGCGTACCGGTATGGACCCCAGGATGTGCTCC

CTCATGCAAGGGTCAACTCTCCCAAGGAGGTCTGGAGCTGCTGGTGCAGCAGTGAAGGGA

GTTGGAACGATGGTGATGGAACTAATTCGGATGATAAAGCGAGGAGTTAATGATCGAAAC

TTCTGGAGAGGCGAGAATGGACGGAGGACAAGGATTGCATTTGAGAGAATGTGCAATATT

CTCAAAGGGAAATTCCAAACAGCAGCACAAAGAGCAATGATGGATCAAGTGCGTGAAAGC

AGGAATCCTGGCAATGCTGAAATTGAAGATCTCATTTTTCTGGCGCGGTCTGCTCTCATT

CTGAGAGGATCAGTGGCCCATAAGTCCTGCTTGCCCGCTTGTGTATACGGGCTCGCTGTA

GCCAGTGGATACGACTTTGAGAGAGAAGGGTACTCTCTAGTTGGAATAGACCCTTTCCGG

CTGCTTCAAAACAGCCAGGTCTTTAGTCTCATTAGACCAAATGAGAATCCAGCACACAAG

AGTCAATTGGTGTGGATGGCATGTCATTCTGCAGCATTCGAGGATCTGAGAGTCTCAAGT

TTCATCAGAGGAACAAGAGTAGTCCCAAGAGGACAACTATCCACTAGAGGAGTTCAAATT

GCTTCAAATGAGAACATAGAAACAATGGACTCCAGCACTCTTGAACTGAGAAGCAGATAC

TGGGCTATAAGAACCAGGAGTGGGGGAAACACCAACCAACAGAGAGCATCTGCAGGACAA

ATCAGTGTACAGCCCACTTTCTCGGTACAGAGAAACCTTCCCTTCGAAAGAGCGACCATT

ATGGCGGCATTCACAGGGAACACTGAAGGCAGAACGTCCGACATGAGGACTGAAATCATA

AGAATGATGGAAAGTGCCAGACCAGAAGATGTGTCTTTCCAGGGGCGGGGAGTCTTCGAG

ATCTCGGACGAAAAGGCAACGAACCCGATCGTGCCTTCCTTTGACATGAGTAATGAAGGA

TCTTATTTCTTCGGAGACAATGCAGAGGAGTATGACAATTAAAGAAAAATACCCTTGTTT

CTACT-------------------------------------------------

>A_chicken_Krasnodar_334-03_2021_EPI1848799

AGCAAAAGCAGGGTAGATAATCACTCACTGAGTGACATCAACATCATGGCGTCTCAAGGC

ACCAAACGATCTTATGAACAGATGGAAACTGGTGGGGAGCGCCAGAATGCCACTGAGATT

AAAGCATCGGTTGGAAGAATGGTTGGTGGAATTGGGAGGTTCTACATACAGATGTGCACT

GAGCTCAAACTCAGCGACTATGAAGGAAGACTGATCCAGAACAGCATAACAATAGAGAGA

ATGGTTCTCTCCGCATTTGATGAAAGGAGAAACAAATATCTGGAAGAACATCCCAGTGCG

GGGAAAGACCCGAAGAAAACAGGAGGTCCAATTTATCGAAGGAGAGATGGGAAATGGGTG

AGAGAGCTGATCCTGTATGACAAAGAGGAGATCAGGAGGATCTGGCGTCAAGCGAATAAT

GGAGAAGACGCAACTGCTGGTCTCACTCACCTGATGATCTGGCACTCCAATCTAAATGAT

GCCACATACCAGAGGACAAGAGCTCTCGTACGTACCGGTATGGACCCCAGAATGTGCTCC

CTCATGCAAGGGTCAACTCTCCCAAGGAGGTCTGGAGCTGCTGGTGCAGCAGTGAAGGGA

GTTGGAACGATGGTAATGGAACTAATTCGGATGATAAAGCGAGGAGTTAATGATCGAAAC

TTCTGGAGAGGCGAGAATGGACGGAGGACAAGGATTGCATTTGAGAGAATGTGCAATATT

CTCAAAGGGAAATTCCAAACAGCAGCACAAAGAGCAATGATGGATCAAGTGCGTGAAAGC

AGGAATCCTGGCAATGCTGAAATTGAAGATCTCATTTTTCTGGCGCGGTCTGCTCTCATT

TTGAGAGGATCAGTGGCCCATAAGTCCTGCTTGCCCGCTTGTGTATACGGGCTCGCTGTA

GCCAGTGGATACGACTTTGAGAGAGAAGGGTACTCTCTAGTTGGAATAGATCCTTTCCGA

CTGCTTCAAAACAGCCAGGTCTTTAGTCTCATTAGACCAAATGAGAATCCAGCACACAAG

AGTCAATTGGTGTGGATGGCATGTCATTCTGCAGCATTCGAGGATCTGAGAGTCTCAAGT

TTCATCAGAGGAACAAGAGTAGTCCCAAGAGGACAACTATCCACTAGAGGAGTTCAAATT

GCTTCAAATGAGAACATAGAAACAATGGACTCCAGCACTCTTGAACTGAGAAGCAGATAT

TGGGCTATAAGAACCAGGAGTGGAGGAAACACCAACCAACAGAGAGCATCTGCAGGACAA

ATCAGTGTACAGCCCACTTTCTCGGTACAGAGAAACCTTCCCTTCGAAAGAGCGACCATT

ATGGCGGCATTCACAGGGAACACTGAAGGCAGAACGTCCGACATGAGGACTGAAATCATA

AGAATGATGGAAAGTGCCAAACCAGAAGATGTGTCTTTCCAGGGGCGAGGAGTCTTCGAG

CTCTCGGACGAAAAGGCAACGAACCCGATCGTGCCTTCCTTTGACATGAGTAATGAAGGA

TCTTATTTCTTCGGAGACAATGCAGAGGAGTATGACAATTAAAGAAAAATACCCTTGTTT

CTACT-------------------------------------------------

>A_pheasant_Wales_000252_2021_EPI1848879

---------------------------------------------ATGGCGTCTCAAGGC

ACCAAACGATCTTATGAACAGATGGAAACTGGTGGGGAGCGCCAGAATGCCACTGAGATT

AGAGCATCGGTTGGAAGAATGGTTGGTGGAATTGGGAGGTTCTACATACAGATGTGCACT

GAGCTCAAACTCAGCGAATATGAAGGAAGACTGATCCAGAACAGCATAACAATAGAGAGA

ATGGTTCTCTCCGCATTTGATGAAAGGAGAAACAAATATCTGGAAGAACATCCCAGTGCG

GGGAAAGACCCGAAGAAAACAGGAGGTCCAATTTATCGAAGGAGAGATGGGAAATGGGTG

AGAGAGCTGATCCTGTATGACAAAGAGGAGATCAGGAGGATCTGGCGTCAAGCGAATAAT

GGAGAAGACGCAACTGCTGGTCTCACTCACCTGATGATCTGGCATTCCAATCTAAATGAT

GCCACATACCAGAGGACGAGAGCTCTCGTACGTACTGGTATGGACCCCAGGATGTGCTCC

CTCATGCAAGGGTCAACTCTCCCAAGGAGGTCTGGAGCTGCTGGTGCAGCAGTGAAGGGA

GTTGGAACGATGGTGATGGAACTAATTCGGATGATAAAGCGAGGAGTTAATGATCGAAAC

TTCTGGAGAGGCGAGAATGGACGGAGGACAAGGATTGCATTTGAGAGAATGTGCAATATT

CTCAAAGGGAAATTCCAAACAGCAGCACAAAGAGCAATGATGGATCAAGTGCGTGAAAGC

AGGAATCCTGGCAATGCTGAAATTGAAGATCTCATTTTTCTGGCGCGGTCTGCTCTCATT

CTGAGAGGATCAGTGGCCCATAAGTCCTGCTTGCCCGCTTGTGTATACGGGCTCGCTGTA

GCCAATGGATACGACTTTGAGAGAGAAGGGTACTCTCTAGTTGGAATAGACCCTTTCCGA

CTGCTTCAAAACAGCCAGGTCTTTAGTCTCATTAGACCAAATGAGAATCCAGCACACAAG

AGTCAATTGGTGTGGATGGCATGTCATTCTGCAGCATTCGAGGATCTGAGAGTCTCAAGT

TTCATCAGAGGAACAAGAGTAGTCCCAAGAGGACAACTATCCACTAGAGGAGTTCAAATT

GCTTCAAATGAGAACATAGAAACAATGGACTCTAGTACTCTTGAACTGAGAAGCAGATAT

TGGGCTATAAGAACCAGGAGTGGAGGAAACACCAACCAACAGAGAGCATCTGCAGGACAA

ATCAGTGTACAGCCCACTTTCTCGGTACAGAGAAACCTTCCCTTCGAAAGAGCGACCATT

ATGGCGGCATTCACCGGGAACACTGAAGGCAGAACGTCCGACATGAGGACTGAAATCATA

AGAATGATGGAAAGTGCCAAACCAGAAGATGTGTCTTTCCAGGGGCGGGGAGTCTTCGAG

CTCTCGGACGAAAAGGCAACGAACCCGATCGTGCCTTCCTTTGACATGAGTAATGAAGGA

TCTTATTTCTTCGGAGACAATGCAGAGGAGTATGACAATTAA------------------

------------------------------------------------------

>A_mute_swan_Czech_Republic_1656-1_2021_EPI1850129

------------------------TCACTGAGTGACATCAACATCATGGCGTCTCAAGGC

ACCAAACGATCTTATGAACAGATGGAAACTGGTGGGGAGCGCCAGAATGCCACTGAGATT

AGAGCATCGGTTGGAAGAATGGTTGGTGGAATTGGGAGGTTCTACATACAGATGTGCACT

GAGCTCAAACTCAGCGACTATGAAGGAAGACTGATCCAGAACAGCATAACAATAGAGAGA

ATGGTTCTCTCCGCATTTGATGAAAGGAGAAACAAATATCTGGAAGAACATCCCAGTGCG

GGGAAAGACCCGAAGAAAACAGGAGGTCCAATTTATCGAAGGAGAGATGGGAAATGGGTG

AGAGAGCTGATCCTGTATGACAAAGAGGAGATCAGGAGGATCTGGCGTCAAGCGAATAAT

GGAGAAGACGCAACTGCTGGTCTCACTCACCTAATGATCTGGCATTCCAATCTAAATGAT

GCCACATACCAGAGGACAAGAGCTCTCGTGCGTACCGGTATGGACCCCAGGATGTGCTCC

CTCATGCAAGGGTCAACTCTCCCAAGGAGGTCTGGAGCTGCTGGTGCAGCAGTGAAGGGA

GTTGGAACGATGGTGATGGAACTAATTCGGATGATAAAGCGAGGAGTTAATGATCGAAAC

TTCTGGAGAGGCGAGAATGGACGGAGGACAAGGATTGCATTTGAGAGAATGTGCAATATT

CTCAAAGGGAAATTCCAAACAGCAGCACAAAGAGCAATGATGGATCAAGTGCGTGAAAGC

AGGAATCCTGGCAATGCTGAAATTGAAGATCTCATTTTTCTGGCGCGGTCTGCTCTCATT

CTGAGAGGATCAGTGGCCCATAAGTCCTGCTTGCCCGCTTGTGTATACGGGCTCGCTGTA

GTCAGTGGATACGACTTTGAGAGAGAAGGGTACTCTCTAGTTGGAATAGACCCTTTCCGA

CTGCTTCAAAACAGCCAGGTCTTTAGTCTCATTAGACCAAATGAGAATCCAGCACACAAG

AGTCAATTGGTGTGGATGGCATGTCATTCTGCAGCATTCGAGGATCTGAGAGTCTCAAGT

TTCATCAGAGGAACAAGAGTAGTCCCAAGAGGACAACTATCCACTAGAGGAGTTCAAATT

GCTTCAAATGAGAACATAGAAACAATGGACTCCAGCACTCTTGAACTAAGAAGCAGATAT

TGGGCTATAAGAACCAGGAGTGGAGGAAACACCAACCAACAGAGAGCATCTGCAGGACAA

ATCAGTGTACAGCCCACTTTCTCGGTACAGAGAAACCTTCCCTTCGAAAGAGCGACCATT

ATGGCGGCATTCACAGGGAACACTGAAGGCAGAACGTCCGACATGAGGACTGAAATCATA

AGAATGATGGAAAGTGCCAGACCAGAAGATGTGTCTTTCCAGGGGCGGGGAGTCTTCGAG

CTCTCGGACGAAAAGGCAACGAACCCGATCGTGCCTTCCTTTGACATGAGTAATGAAGGA

TCTTATTTCTTCGGAGACAATGCAGAGGAGTATGACA-----------------------

------------------------------------------------------

>A_mute_swan_Croatia_14_2021_EPI1850963

------------GTAGATAATCACTCACTGAGTGACATCAACATCATGGCGTCTCAAGGC

ACCAAACGATCTTATGAACAGATGGAAACTGGTGGGGAGCGCCAGAATGCCACTGAGATT

AGAGCATCGGTTGGAAGAATGGTTGGTGGAATTGGGAGGTTCTACATACAGATGTGCACT

GAGCTCAAACTCAGTGACTATGAAGGAAGACTGATCCAGAACAGCATAACAATAGAGAGA

ATGGTTCTCTCCGCATTTGATGAAAGGAGAAACAAATATCTGGAAGAACATCCCAGTGCG

GGGAAAGACCCGAAGAAAACAGGAGGTCCAATTTATCGAAGGAGAGATGGGAAATGGGTG

AGAGAGCTGATCCTGTATGACAAAGAGGAGATCAGGAGGATCTGGCGCCAAGCGAATAAT

GGAGAAGACGCAACTGCTGGTCTCACTCACCTGATGATCTGGCACTCCAATCTAAATGAT

GCCACATACCAGAGGACAAGAGCTCTCGTACGTACCGGTATGGACCCCAGGATGTGCTCC

CTCATGCAAGGGTCAACTCTCCCAAGGAGGTCTGGAGCTGCTGGTGCAGCAGTGAAGGGA

GTTGGAACGATGGTAATGGAACTAATTCGGATGATAAAGCGAGGAGTTAATGATCGAAAC

TTCTGGAGAGGCGAGAATGGACGGAGGACAAGGATTGCATTTGAGAGAATGTGCAATATT

CTCAAAGGGAAATTCCAAACAGCAGCACAAAGAGCAATGATGGATCAAGTGCGTGAAAGC

AGGAATCCTGGCAATGCTGAAATTGAAGATCTCATTTTTCTGGCGCGGTCTGCTCTCATT

CTGAGAGGATCAGTGGCCCATAAGTCCTGCTTGCCCGCTTGTGTATACGGGCTCGCTGTA

GCCAGTGGATACGACTTTGAGAGAGAAGGGTACTCTCTAGTTGGAATAGACCCTTTCCGA

CTGCTTCAAAACAGCCAGGTCTTTAGTCTCATTAGACCAAATGAGAATCCAGCACACAAG

AGTCAATTGGTGTGGATGGCATGTCATTCTGCAGCATTCGAGGATCTGAGAGTCTCAAGT

TTCATCAGAGGAACAAGAGTAGTCCCAAGAGGACAACTATCCACTAGAGGAGTTCAAATT

GCTTCAAATGAGAACATAGAAACAATGGACTCCAGCACTCTTGAACTGAGAAGCAGATAT

TGGGCTATAAGAACCAGGAGTGGAGGAAACACCAACCAACAGAGAGCATCTGCAGGACAA

ATCAGTGTACAGCCCACTTTCTCGGTACAGAGAAACCTTCCCTTCGAAAGAGCGACCATT

ATGGCGGCATTCACAGGAAACACTGAAGGCAGAACGTCCGACATGAGGACTGAAATCATA

AGAATGATGGAAAGTGCCAAACCAGAAGATGTGTCTTTCCAGGGGCGGGGAGTCTTCGAG

CTCTCGGACGAAAAGGCAACGAACCCGATCGTGCCTTCCTTTGACATGAGTAATGAAGGA

TCTTATTTCTTCGGAGACAATGCAGAGGAGTATGACAATTAAAGAAAAATAC--------

------------------------------------------------------

>A_chicken_Vietnam_Raho4-Cd-20-421_2020_EPI1853932

------------GTAGATAATCACTCACCGAGTGACATCAACATCATGGCGTCTCAAGGC

ACCAAACGATCCTATGAACAGATGGAAACTGGTGGAGAGCGCCAGAATGCTACTGAAATC

AGGGCCTCTGTTGGAAGGATGGTTGGTGGCATTGGGAGGTTCTACATACAGATGTGCACA

GAACTCAAACTCAGTGACTATGAAGGGAGGCTGATCCAGAACAGCATAACGATGGAGAGA

ATGGTACTTTCTGCCTTTGACGAGAGAAGGAACAGGTATCTGGAAGAGCACCCCAGTGCA

GGGAAGGACCCTAAGAAAACTGGAGGTCCAATTTATCGGAGAAGAGACGGGAAATGGATT

AGAGAGCTGATTTTGTACGACAAAGAAGAGATTAGGAGGATTTGGCGCCAAGCGAACAAT

GGAGAAGACGCAACCGCTGGTCTTACCCACCTGATGATATGGCACTCCAATCTGAATGAT

GCCACATATCAGAGAACAAGAGCTCTCGTGCGTACCGGAATGGACCCCAGGATGTGCTCC

CTAATGCAGGGATCAACTCTCCCGAGAAGGTCTGGAGCTGCTGGTGCAGCAGTGAAGGGG

GTAGGAACAATGGTGATGGAGCTGATTCGAATGATAAAACGAGGGATTAATGACCGGAAT

TTCTGGAGAGGCGAAAATGGAAGAAGAACAAGGATTGCATATGAGAGAATGTGTAACATC

CTCAAAGGGAAATTCCAAACAGCTGCACAAAGAGCAATGATGGATCAGGTGAGAGAGAGC

AGAAATCCTGGGAATGCTGAAATTGAAGATCTCATTTTTCTGGCACGGTCTGCACTCATC

CTGAGAGGATCAGTGGCCCATAAGTCCTGTTTGCCTGCTTGTGTGTACGGACTTGCAGTA

GCCAGTGGGTATGACTTCGAGAGAGAAGGATACTCCCTGGTTGGGATAGATCCTTTCCGT

CTACTTCAAAACAGCCAGGTCTTTAGTCTCATTAGGCCAAATGAAAACCCAGCACATAAG

AGTCAATTAGTATGGATGGCATGCCACTCTGCAGCATTTGAGGACCTCAGAGTTTCAAGT

TTCATCAGAGGAACAAGAGTGGTACCACGAGGGCAGCTATCCACTAGAGGGGTTCAAATT

GCCTCAAATGAGAACATGGAAACAATGGACTCAAACACACTTGAGCTGAGAAGTAGATAC

TGGGCTATAAGAACCAGGAGCGGAGGAAATACCAACCAGCAGAGGGCATCTGCAGGGCAG

ATCAGCGTTCAACCCACTTTCTCGGTGCAGAGGAACCTTCCCTTCGAAAGAGCGACCATT

ATGGCAGCATTTGCAGGAAATACTGAAGGCAGAACGTCCGACATGAGGACAGAAATCATA

AGAATGATGGAGAATGCCAAACCAGAAGATGTGTCATTCCAAGGGCGGGGAGTCTTCGAG

CTTTCGGACGAAAAGGCAACGAACCCGATCGTGCCTTCCTTTGACATGAACAATGAAGGA

TCTTATTTCTTCGGAGACAATGCAGAGGAGTATGACAATTAAAGAAAAATAC--------

------------------------------------------------------

>A_chicken_Czech_Republic_3531-1_2021_EPI1854236

------------------------TCACTGAGTGACATCAACATCATGGCGTCTCAAGGC

ACCAAACGATCTTATGAACAGATGGAAACTGGTGGGGAGCGCCAGAATGCCACTGAGATT

AGAGCATCGGTTGGAAGAATGGTTGGTGGAATTGGGAGGTTCTACATACAGATGTGCACT

GAGCTCAAACTCAGCGACTATGAAGGAAGACTGATCCAGAACAGCATAACAATAGAGAGA

ATGGTTCTCTCAGCATTTGATGAAAGGAGAAACAAATATCTGGAAGAACATCCCAGTGCG

GGGAAAGACCCGAAGAAAACAGGAGGTCCAATTTATCGAAGGAGAGATGGGAAATGGGTG

AGAGAGCTGATCCTGTATGACAAAGAGGAGATCAGGAGGATCTGGCGTCAAGCGAATAAT

GGAGAAGACGCAACTGCTGGTCTCACTCACCTGATGATCTGGCATTCCAATCTAAATGAT

GCCACATACCAGAGGACAAGAGCTCTCGTACGTACCGGTATGGACCCCAGGATGTGCTCC

CTCATGCAAGGGTCAACTCTCCCAAGGAGGTCTGGAGCTGCTGGTGCAGCAGTGAAGGGA

GTTGGAACGATGGTGATGGAACTAATTCGGATGATAAAGCGAGGAGTTAATGATCGAAAC

TTCTGGAGAGGCGAGAATGGACGGAGGACAAGGATTGCATTTGAGAGAATGTGCAATATT

CTCAAAGGGAAATTCCAAACAGCAGCACAAAGAGCAATGATGGATCAAGTGCGTGAAAGC

AGGAATCCTGGCAATGCTGAAATTGAAGATCTCATTTTTCTGGCGCGGTCTGCTCTCATT

CTGAGAGGATCAGTGGCCCATAAGTCCTGCTTGCCCGCTTGTGTATACGGGCTCGCTGTA

GCCAGTGGATACGACTTTGAGAGAGAAGGGTACTCTCTAGTTGGAATAGACCCTTTCCGA

CTGCTTCAAAACAGCCAGGTCTTTAGTCTCATTAGACCAAATGAGAATCCAGCACACAAG

AGTCAATTGGTGTGGATGGCATGTCATTCTGCAGCATTCGAGGATCTGAGAATCTCAAGT

TTCATCAGAGGAACAAGAGTAGTCCCAAGAGGACAACTATCCACTAGAGGAGTTCAAATT

GCTTCAAATGAGAACATAGAAACAATGGACTCCAGCACTCTTGAACTGAGAAGCAGATAT

TGGGCTATAAGAACCAGGAGTGGAGGAAACACCAACCAACAGAGAGCATCTGCAGGACAA

ATCAGTGTACAGCCCACTTTCTCGGTACAGAGAAACCTTCCCTTCGAAAGAGCGACCATT

ATGGCGGCATTCACAGGGAACACTGAAGGCAGAACGTCCGACATGAGGACTGAAATCATA

AGAATGATGGAAAGTGCCAAACCAGAAGATGTGTCTTTCCAGGGGCGGGGAGTCTTCGAG

CTCTCGGACGAAAAGGCAGCGAACCCGATCGTGCCTTCCTTTGACATGAGTAATGAAGGA

TCTTATTTCTTCGGAGACAATGCAGAGGAGTATGACA-----------------------

------------------------------------------------------

>A_wigeon_Latvia_23903_2021_EPI1855976

---------------GATAATCACTCACTGAGTGACATCAACATCATGGCGTCTCAAGGC

ACCAAACGATCTTATGAACAGATGGAAACTGGTGGGGAGCGCCAGAATGCCACTGAGATT

AGAGCATCGGTTGGAAGAATGGTTGGTGGAATTGGGAGGTTCTACATACAGATGTGCACT

GAGCTCAAACTCAGCGACTATGAAGGAAGACTGATCCAGAACAGCATAACAATAGAGAGA

ATGGTTCTCTCCGCATTTGATGAAAGGAGAAACAAATACCTGGAAGAACACCCCAGTGCG

GGGAAAGACCCGAAGAAAACAGGAGGTCCAATTTATCGAAGGAGAGATGGGAAATGGGTG

AGAGAGCTGATCCTGTATGACAAAGAGGAGATCAGGAGGATCTGGCGTCAAGCGAATAAT

GGAGARGACGCAACTGCTGGTCTCACTCACCTAATGATCTGGCATTCCAATCTAAATGAT

GCCACATACCAGAGGACAAGAGCTCTCGTGCGTACCGGTATGGACCCCAGGATGTGCTCC

CTCATGCAAGGGTCAACTCTCCCAAGGAGGTCTGGAGCTGCTGGTGCAGCAGTGAAGGGA

GTTGGAACGATGGTGATGGAACTAATTCGGATGATAAAGCGAGGAGTTAATGATCGAAAC

TTCTGGAGAGGCGAGAATGGACGGAGGACAAGGATTGCATTTGAGAGAATGTGCAATATT

CTCAAAGGGAAATTCCAAACAGCAGCACAAAGAGCAATGATGGATCAAGTGCGTGAAAGC

AGGAATCCTGGCAATGCTGAAATTGAAGATCTCATTTTTCTGGCGCGGTCTGCTCTCATT

CTGAGAGGATCAGTGGCCCATAAGTCCTGCTTGCCCGCTTGTGTATACGGGCTCGCTGTA

GTCAGTGGATACGACTTTGAGAGAGAAGGGTACTCTCTAGTTGGAATAGACCCTTTCCGA

CTGCTTCAAAACAGCCAGGTCTTTAGTCTCATTAGACCAAATGAGAATCCAGCACACAAG

AGTCAATTGGTGTGGATGGCATGTCATTCTGCAGCATTCGAGGATCTGAGAGTCTCAAGT

TTCATCAGAGGAACAAGAGTAGTCCCAAGAGGACAACTATCCACTAGAGGAGTTCAAATT

GCTTCAAATGAGAACATAGAAACAATGGACTCCAGCACTCTTGAACTAAGAAGCAGATAT

TGGGCTATAAGAACCAGGAGTGGAGGAAACACCAACCAACAGAGAGCATCTGCAGGACAA

ATCAGTGTACAGCCCACTTTCTCTGTACAGAGAAACCTTCCCTTCGAAAGAGCGACCATT

ATGGCGGCATTCACAGGGAACACTGAAGGCAGAACGTCCGACATGAGGACTGAAATCATA

AGAATGATGGAAAGTGCCAGACCAGAAGATGTGTCTTTCCAGGGGCGGGGAGTCTTCGAG

CTCTCGGACGAAAAGGCAACGAACCCGATCGTGCCTTCCTTTGACATGAGTAATGAAGGA

TCTTATTTCTTCGGAGACAATGCAGAGGAGTATGACAATTAAAG----------------

------------------------------------------------------

>A_chicken_Czech_Republic_4980_2021_EPI1858493

------------------------TCACTGAGTGACATCAACATCATGGCGTCTCAAGGC

ACCAAACGATCTTATGAACAGATGGAAACTGGTGGGGAGCGCCAGAATGCCACTGAGATT

AGAGCATCGGTTGGAAGAATGGTTGGTGGAATTGGGAGGTTCTACATACAGATGTGCACT

GAGCTCAAACTCAGCGACTATGAAGGAAGACTGATCCAGAACAGCATAACAATAGAGAGA

ATGGTTCTCTCCGCATTTGATGAAAGGAGAAACAAATATCTGGAAGAACATCCCAGTGCG

GGGAAAGACCCGAAGAAAACAGGAGGTCCAATTTATCGAAGGAGAGATGGGAAATGGGTG

AGAGAGCTGATCCTGTATGACAAAGAGGAGATCAGGAGGATCTGGCGTCAAGCGAATAAT

GGAGAAGACGCAACTGCTGGTCTCACTCACCTGATGATCTGGCATTCCAATCTAAATGAT

GCCACATACCAGAGGACAAGAGCTCTCGTRCGTACCGGCATGGACCCCAGGATGTGCTCC

CTCATGCAAGGGTCAACTCTCCCAAGGAGGTCTGGAGCTGCTGGTGCAGCAGTGAAGGGA

GTTGGAACGATGGTAATGGAACTAATTCGGATGATAAAGCGAGGAGTTAATGATCGAAAC

TTCTGGAGAGGCGAGAATGGACGGAGGACAAGGATTGCATTTGAGAGAATGTGCAATATT

CTCAAAGGGAAATTCCAAACAGCAGCACAAAGAGCAATGATGGATCAAGTGCGTGAAAGC

AGGAATCCTGGCAATGCTGAAATTGAAGATCTCATTTTTCTGGCGCGGTCTGCTCTCATT

CTGAGAGGATCAGTGGCCCATAAGTCCTGCTTGCCCGCTTGTGTATACGGGCTCGCTGTG

GCCAGTGGATACGACTTTGAGAGAGAAGGGTACTCTCTAGTTGGAATAGACCCTTTCCGA

CTGCTTCAAAACAGCCAGGTCTTTAGTCTCATTAGACCAAATGAGAATCCAGCACACAAG

AGTCAATTGGTGTGGATGGCATGTCATTCTGCAGCATTCGAGGATCTGAGAGTCTCAAGT

TTCATCAGAGGAACAAGAGTAGTCCCAAGAGGACAACTATCCACTAGAGGAGTTCAAATT

GCTTCAAATGAGAACATAGAAACAATGGACTCCAGCACTCTTGAACTGAGAAGCAGATAT

TGGGCTATAAGAACCAGGAGTGGAGGAAACACCAACCAACAGAGAGCATCTGCAGGACAA

ATCAGTGTACAGCCCACTTTCTCGGTACAGAGAAACCTTCCCTTCGAAAGAGCGACCATT

ATGGCGGCATTCACAGGGAACACTGAAGGCAGAACGTCCGACATGAGGACTGAAATCATA

AGAATGATGGAAAGTGCCAAACCAGAAGATGTGTCTTTCCAGGGGCGGGGAGTCTTCGAG

CTCTCGGACGAAAAGGCAACGAACCCGATCGTGCCTTCCTTTGACATGAGTAATGAAGGA

TCTTATTTCTTCGGAGACAATGCAGAGGAGTATGACA-----------------------

------------------------------------------------------

>A_swan_Lithuania_1258PG1_21VIR2606-2_2021_EPI1858567

AGCRAAAGCAGGGTAGATAATCACTCACTGAGTGACATCAACATCATGGCGTCTCAAGGC

ACCAAACGATCTTATGAACAGATGGAAACTGGTGGGGAGCGCCAGAATGCCACTGAGATT

AGAGCATCGGTTGGAAGAATGGTTGGTGGAATTGGGAGGTTCTACATACAGATGTGCACT

GAGCTCAAACTCAGCGACTACGAAGGAAGACTGATCCAGAACAGCATAACAATAGAGAGA

ATGGTTCTCTCCGCATTTGATGAAAGGAGAAACAAATATCTGGAAGAACATCCCAGTGCG

GGGAAAGACCCGAAGAAAACAGGAGGTCCAATTTATCGAAGGAGAGATGGGAAATGGGTG

AGAGAGCTGATCCTGTATGACAAAGAGGAGATCAGGAGGATCTGGCGTCAAGCGAATAAT

GGAGAAGACGCAACTGCTGGTCTCACTCACCTGATGATCTGGCATTCCAATCTAAATGAT

GCCACATACCAGAGGACAAGAGCTCTCGTACGTACCGGTATGGACCCCAGGATGTGCTCC

CTCATGCAAGGGTCAACTCTCCCAAGGAGGTCTGGAGCTGCTGGTGCAGCAGTGAAGGGA

GTTGGAACGATGGTAATGGAACTAATTCGGATGATAAAGCGAGGAGTTAATGATCGAAAC

TTCTGGAGAGGCGAGAATGGACGGAGGACAAGGATTGCATTTGAGAGAATGTGCAATATT

CTCAAAGGGAAATTCCAAACAGCAGCACAAAGAGCAATGATGGATCAAGTGCGTGAAAGC

AGGAATCCTGGCAATGCTGAAATTGAAGATCTCATTTTTTTGGCGCGGTCTGCTCTCATT

CTGAGAGGATCAGTGGCCCATAAGTCCTGCTTGCCCGCTTGTGTATACGGGCTCGCTGTA

GCCAGTGGATACGACTTTGAGAGAGAAGGGTACTCTCTAGTTGGAATAGACCCTTTCCGA

CTGCTTCAAAACAGCCAGGTCTTTAGTCTCATTAGACCAAATGAGAATCCAGCACACAAG

AGTCAATTGGTGTGGATGGCATGTCATTCTGCAGCATTCGAGGATCTGAGAGTCTCAAGT

TTCATCAGAGGAACAAGAGTAGTCCCAAGAGGACAACTATCCACTAGAGGAGTTCAAATT

GCTTCAAATGAGAACATAGAAACAATGGACTCCAGCACTCTTGAACTGAGAAGCAGATAT

TGGGCTATAAGAACCAGGAGTGGAGGAAACACCAACCAACAGAGAGCATCTGCAGGACAA

ATCAGTGTACAGCCCACTTTCTCGGTACAGAGAAACCTTCCCTTCGAAAGAGCGACCATT

ATGGCGGCATTCACAGGGAACACTGAAGGCAGAACGTCCGACATGAGGACTGAAATCATA

AGAATGATGGAAAGTGCCAAACCAGAAGATGTGTCTTTCCAGGGGCGGGGAGTCTTCGAG

CTCTCGGACGAAAAGGCAACGAACCCGATCGTGCCTTCCTTTGACATGAGTAATGAAGGA

TCTTATTTCTTCGGAGACAATGCAGAGGAGTATGACAATTAAAGAAAAATACCCTTGTTT

CTACT-------------------------------------------------

>A_swan_Lithuania_1298PG1_21VIR2606-3_2021_EPI1858575

AGCRAAAGCAGGGTAGATAATCACTCACTGAGTGACATCAACATCATGGCGTCTCAAGGC

ACCAAACGATCTTATGAACAGATGGAAACTGGTGGGGAGCGCCAGAATGCCACTGAGATT

AGAGCATCGGTTGGAAGAATGGTTGGTGGAATTGGGAGGTTCTACATACAGATGTGCACT

GAGCTCAAACTCAGCGACTATGAAGGAAGACTGATCCAGAACAGCATAACAATAGAGAGA

ATGGTTCTCTCCGCATTTGATGAAAGGAGAAACAAATATCTGGAAGAACATCCCAGTGCG

GGGAAAGACCCGAAGAAAACAGGAGGTCCAATTTATCGAAGGAGAGATGGGAAATGGGTG

AGAGAGCTGATCCTGTATGACAAAGAGGAGATCAGGAGGATCTGGCGTCAAGCGAATAAT

GGAGAAGACGCAACTGCTGGTCTCACTCACCTAATGATCTGGCATTCCAATCTAAATGAT

GCCACATACCAGAGGACAAGAGCTCTCGTGCGTACCGGTATGGACCCCAGGATGTGCTCC

CTCATGCAAGGGTCAACTCTCCCAAGGAGGTCTGGAGCTGCTGGTGCAGCAGTGAAGGGA

GTTGGAACGATGGTGATGGAACTAATTCGGATGATAAAGCGAGGAGTTAATGATCGAAAC

TTCTGGAGAGGCGAGAATGGACGGAGGACAAGGATTGCATTTGAGAGAATGTGCAATATT

CTCAAAGGGAAATTCCAAACAGCAGCACAAAGAGCAATGATGGATCAAGTGCGTGAAAGC

AGGAATCCTGGCAATGCTGAAATTGAAGATCTCATTTTTCTGGCGCGGTCTGCTCTCATT

CTGAGAGGATCAGTGGCCCATAAGTCCTGCTTGCCCGCTTGTGTATACGGGCTCGCTGTA

GCCAGTGGATACGACTTTGAGAGAGAAGGGTACTCTCTAGTTGGAATAGACCCTTTCCGA

CTGCTTCAAAACAGCCAGGTCTTTAGTCTCATTAGACCAAATGAGAATCCAGCACACAAG

AGTCAATTGGTGTGGATGGCATGTCATTCTGCAGCATTCGAGGATCTGAGAGTCTCAAGT

TTCATCAGAGGAACAAGAGTAGTCCCAAGAGGGCAACTATCCACTAGAGGAGTTCAAATT

GCTTCAAATGAGAACATAGAAACAATGGACTCCAGCACTCTTGAACTAAGAAGCAGATAT

TGGGCTATAAGAACCAGGAGTGGAGGAAACACCAACCAACAGAGAGCATCTGCAGGACAA

ATCAGTGTACAGCCCACTTTCTCGGTACAGAGAAACCTTCCCTTCGAAAGAGCGACCATT

ATGGCGGCATTCACAGGGAACACTGAAGGCAGAACGTCCGACATGAGGACTGAAATCATA

AGAATGATGGAAAGTGCCAGACCAGAAGATGTGTCTTTCCAGGGGCGGGGAGTCTTCGAG

CTCTCGGACGAAAAGGCAACGAACCCGATCGTGCCTTCCTTTGACATGAGTAATGAAGGA

TCTTATTTCTTCGGAGACAATGCAGAGGAGTATGACAATTAAAGAAAAATACCCTTGTTT

CTACT-------------------------------------------------

>A_chicken_Bulgaria_50-1_21VIR1454-9_2021_EPI1858615

AGCAAAAGCAGGGTAGATAATCACTCACTGAGTGACATCAACATCATGGCGTCTCAAGGC

ACCAAACGATCTTATGAACAGATGGAAACTGGTGGGGAGCGCCAGAATGCCACTGAGATT

AGAGCATCGGTTGGAAGAATGGTTGGTGGAATTGGGAGGTTCTACATACAGATGTGCACT

GAGCTCAAACTCAGCGACTATGAAGGAAGACTGATCCAGAACAGCATAACAATAGAGAGA

ATGGTTCTCTCCGCATTTGATGAAAGGAGAAACAAATATCTGGAAGAACATCCCAGTGCG

GGGAAAGACCCGAAGAAAACAGGAGGTCCAATTTATCGAAGGAGAGATGGGAAATGGGTG

AGAGAGCTGATCCTGTATGACAAAGAGGAGATCAGGAGGATCTGGCGTCAAGCGAATAAT

GGGGAAGACGCAACTGCTGGTCTCACTCACCTGATGATCTGGCACTCCAATCTAAATGAT

GCCACATACCAGAGGACAAGAGCTCTCGTACGTACCGGTATGGACCCCAGGATGTGCTCC

CTCATGCAAGGGTCAACTCTCCCAAGGAGGTCTGGAGCTGCTGGTGCTGCAGTGAAGGGA

GTTGGAACGATGGTAATGGAACTAATTCGGATGATAAAGCGAGGAGTTAATGATCGAAAC

TTCTGGAGAGGCGAGAATGGACGGAGGACAAGGATTGCATTTGAGAGAATGTGCAATATT

CTCAAAGGGAAATTCCAAACAGCAGCACAAAGAGCAATGATGGATCAAGTGCGTGAAAGC

AGGAATCCTGGCAATGCTGAAATTGAAGATCTCATTTTTCTGGCGCGGTCTGCTCTCATT

CTGAGAGGATCAGTGGCCCATAAGTCCTGCTTGCCCGCTTGTGTATATGGGCTCGCTGTA

GCCAGTGGATACGACTTTGAGAGAGAAGGGTACTCTCTAGTTGGAATAGACCCTTTCCGA

CTGCTTCAAAACAGCCAGGTCTTTAGTCTCATTAGACCAAATGAGAATCCAGCACACAAG

AGTCAGTTGGTGTGGATGGCATGTCATTCTGCAGCATTCGAGGATCTGAGAGTCTCAAGT

TTCATCAGAGGAACAAGAGTAGTCCCAAGAGGACAACTATCCACTAGAGGAGTTCAAATT

GCTTCAAATGAGAACATAGAAACAATGGACTCCAGCACTCTTGAACTGAGAAGCAGATAT

TGGGCTATAAGAACCAGGAGTGGAGGAAACACCAACCAACAGAGAGCATCTGCAGGACAA

ATCAGTGTACAGCCCACTTTCTCGGTACAGAGAAACCTTCCCTTCGAAAGAGCGACCATT

ATGGCGGCATTCACAGGGAACACTGAAGGCAGAACGTCCGACATGAGGACTGAAATCATA

AGAATGATGGAAAGTGCCAAACCAGAAGATGTGTCTTTCCAGGGGCGGGGAGTCTTCGAG

CTCTCGGACGAAAAGGCAACGAACCCGATCGTGCCTTCCTTTGACATGAGTAATGAAGGA

TCTTATTTCTTCGGAGACAATGCAGAGGAGTATGACAATTAAAGAAAAATACCCTTGTTT

CTACT-------------------------------------------------

>A_mute_swan_Poland_MB189_2021_EPI1859656

-------------TAGATAATCACTCACTGAGTGACATCAACATCATGGCGTCTCAAGGC

ACCAAACGATCTTATGAACAGATGGAAACTGGTGGGGAGCGCCAGAATGCCACTGAGATT

AGAGCATCGGTTGGAAGAATGGTTGGTGGAATTGGGAGGTTCTACATACAGATGTGCACT

GAGCTCAAACTCAGCGACTATGAAGGAAGACTGATCCAGAACAGCATAACAATAGAGAGA

ATGGTTCTCTCCGCATTTGATGAAAGGAGAAACAAATATCTGGAAGAACATCCCAGTGCG

GGGAAAGACCCGAAGAAAACAGGAGGTCCAATTTATCGAAGGAGAGATGGGAAATGGGTG

AGAGAGCTGATCCTGTATGACAAAGAGGAGATCAGGAGGATCTGGCGTCAAGCGAATAAT

GGAGAAGACGCAACTGCTGGTCTCACTCACCTGATGATCTGGCATTCCAATCTAAATGAT

GCCACATACCAGAGGACAAGAGCTCTCGTGCGTACCGGTATGGACCCCAGGATGTGCTCC

CTCATGCAAGGGTCAACTCTCCCAAGGAGGTCTGGAGCTGCTGGTGCAGCAGTGAAGGGA

GTTGGAACGATGGTGATGGAACTAATTCGGATGATAAAGCGAGGAGTTAATGATCGAAAC

TTCTGGAGAGGCGAGAATGGACGGAGGACAAGGATTGCATTTGAGAGAATGTGCAATATT

CTCAAAGGGAAATTCCAAACAGCAGCACAAAGAGCAATGATGGATCAAGTGCGTGAAAGC

AGGAATCCTGGCAATGCTGAAATTGAAGATCTCATTTTTCTGGCGCGGTCTGCTCTCGTT

CTGAGAGGATCAGTGGCCCATAAGTCCTGCTTGCCCGCTTGTGTATACGGGCTCGCTGTA

GCCAGTGGATACGACTTTGAGAGAGAAGGGTACTCTCTAGTTGGAATAGACCCTTTCCGA

CTGCTTCAAAACAGCCAGGTCTTTAGTCTCATTAGACCAAATGAGAATCCAGCACACAAG

AGTCAATTGGTGTGGATGGCATGTCATTCTGCAGCATTCGAGGATCTGAGAGTCTCAAGT

TTCATCAGAGGAACAAGAGTAGTCCCAAGAGGACAACTATCCACTAGAGGAGTTCAAATT

GCTTCAAATGAGAACATAGAAACAATGGACTCCAGCACTCTTGAACTGAGAAGCAGATAT

TGGGCTATAAGAACCAGGAGTGGAGGAAACACCAACCAACAGAGAGCATCTGCAGGACAA

ATCAGTGTACAGCCCACTTTCTCGGTACAGAGAAACCTTCCCTTCGAAAGAGCAACCATT

ATGGCGGCATTCACAGGGAACACTGAAGGTAGAACGTCCGATATGAGGACTGAAATCATA

AGAATGATGGAAAGTGCCAGACCAGAAGATGTGTCTTTCCAGGGGCGGGGGGTCTTCGAG

CTCTCGGACGAAAAGGCAACGAACCCGATCGTGCCTTCCTTTGACATGAGTAATGAAGGA

TCTTATTTCTTCGGAGACAATGCAGAGGAGTATGACAATTAAAGAAAAATAC--------

------------------------------------------------------

>A_mute_swan_Poland_MB272_2021_EPI1859672

-------------TAGATAATCACTCACTGAGTGACATCAACATCATGGCGTCCCAAGGC

ACCAAACGATCTTATGAACAGATGGAAACTGGTGGGGAGCGCCAGAATGCCACTGAGATT

AGAGCATCGGTTGGAAGAATGGTTGGTGGAATTGGGAGGTTCTACATACAGATGTGCACT

GAGCTCAAACTCAGCGACTATGAAGGAAGACTGATCCAGAACAGCATAACAATAGAGAGA

ATGGTTCTCTCCGCATTTGATGAAAGGAGAAACAAATATCTGGAAGAACATCCCAGTGCG

GGGAAAGACCCGAAGAAAACAGGAGGTCCAATTTATCGAAGGAGAGATGGGAAATGGGTG

AGAGAGCTGATCCTGTATGACAAAGAGGAGATCAGGAGGATCTGGCGTCAAGCGAATAAT

GGAGAAGACGCAACTGCTGGTCTCACTCACCTGATGATCTGGCATTCCAATCTAAATGAT

GCCACATACCAGAGGACAAGAGCTCTCGTACGTACCGGTATGGACCCCAGGATGTGCTCC

CTCATGCAAGGGTCAACTCTCCCAAGGAGGTCTGGAGCTGCTGGTGCAGCAGTGAAGGGA

GTTGGAACGATGGTAATGGAACTAATTCGGATGATAAAGCGAGGAGTTAATGATCGAAAC

TTCTGGAGAGGCGAGAATGGACGGAGGACAAGGATTGCATTTGAGAGAATGTGCAATATT

CTCAAAGGGAAATTCCAAACAGCAGCACAAAGAGCAATGATGGATCAAGTGCGTGAAAGC

AGGAATCCTGGCAATGCTGAAATTGAAGATCTCATTTTTCTGGCGCGGTCTGCTCTCATT

CTGAGAGGATCAGTGGCCCATAAGTCCTGCTTGCCCGCTTGTGTATACGGGCTCGCTGTA

GCCAGTGGATACGACTTTGAGAGAGAAGGGTACTCTCTAGTTGGAATAGACCCTTTCCGA

CTGCTTCAAAACAGCCAGGTCTTTAGTCTCATTAGACCAAATGAGAATCCAGCACACAAG

AGTCAATTGGTGTGGATGGCATGTCATTCTGCAGCATTCGAGGATCTGAGAGTCTCAAGT

TTCATCAGAGGAACAAGAGTAGTCCCAAGAGGACAACTATCCACTAGAGGAGTTCAAATT

GCTTCAAATGAGAACATAGAAACAATGGACTCCAGCACTCTTGAACTGAGAAGCAGATAT

TGGGCTATAAGAACCAGGAGTGGAGGAAACACCAACCAACAGAGAGCATCTGCAGGACAA

ATCAGTGTACAGCCCACTTTCTCGGTACAGAGAAACCTTCCCTTCGAAAGAGCGACCATT

ATGGCGGCATTCACAGGGAACACTGAAGGCAGAACGTCCGACATGAGGACTGAAATCATA

AGAATGATGGAAAGTGCCAAACCAGAAGATGTGTCTTTCCAGGGGCGGGGAGTCTTCGAG

CTCTCGGACGAAAAGGCAACGAACCCGATCGTGCCTTCCTTTGACATGAGTAATGAAGGA

TCTTATTTCTTCGGAGACAATGCAGAGGAGTATGACAATTAAAGAAAAATAC--------

------------------------------------------------------

>A_anser_anser_Spain_297-1_21VIR1230-5_2021_EPI1860064

-------GCAGGGTAGATAATCACTCACTGAGTGACATCAACATCATGGCGTCTCAAGGC

ACCAAACGATCTTATGAACAGATGGAAACTGGTGGGGAGCGCCAGAATGCCACTGAGATT

AGAGCATCGGTTGGAAGAATGGTTGGTGGAATTGGGAGGTTCTACATACAGATGTGCACT

GAGCTCAAACTCAGCGAATATGAAGGAAGACTGATCCAGAACAGCATAACAATAGAGAGA

ATGGTTCTCTCCGCATTTGATGAAAGGAGAAACAAATATCTGGAAGAACATCCCAGTGCG

GGGAAAGACCCGAAGAAAACAGGAGGTCCAATTTATCGAAGGAGAGATGGGAAATGGGTG

AGAGAGCTGATCCTGTATGACAAAGAGGAGATCAGGAGGATCTGGCGTCAAGCGAATAAT

GGAGAAGACGCAACTGCTGGTCTCACTCACCTGATGATCTGGCATTCCAATCTAAATGAT

GCCACATACCAGAGGACGAGAGCTCTCGTACGTACTGGTATGGACCCCAGGATGTGCTCC

CTCATGCAAGGGTCAACTCTCCCAAGGAGGTCTGGAGCTGCTGGTGCAGCAGTGAAGGGA

GTTGGAACGATGGTGATGGAACTAATTCGGATGATAAAGCGAGGAGTTAATGATCGAAAC

TTCTGGAGAGGCGAGAATGGACGGAGGACAAGGATTGCATTTGAGAGAATGTGCAATATT

CTCAAAGGGAAATTCCAAACAGCAGCACAAAGAGCAATGATGGATCAAGTGCGTGAAAGC

AGGAATCCTGGCAATGCTGAAATTGAAGATCTCATTTTTCTGGCGCGGTCTGCTCTCATT

CTGAGAGGATCAGTGGCCCATAAGTCCTGCTTGCCCGCTTGTGTATACGGGCTCGCTGTA

GCCAATGGATACGACTTTGAGAGAGAAGGGTACTCTCTAGTTGGAATAGACCCTTTCCGA

CTGCTTCAAAACAGTCAGGTCTTTAGTCTCATTAGACCAAATGAGAATCCAGCACACAAG

AGTCAATTGGTGTGGATGGCATGTCATTCTGCAGCATTCGAGGATCTGAGAGTCTCAAGT

TTCATCAGAGGAACAAGAGTAGTCCCAAGAGGACAACTATCCACTAGAGGAGTTCAAATT

GCTTCAAATGAGAACATAGAAACAATGGACTCTAGTACTCTTGAACTGAGAAGCAGATAT

TGGGCTATAAGAACCAGGAGTGGAGGAAACACCAACCAACAGAGAGCATCTGCAGGACAA

ATCAGTGTACAGCCCACTTTCTCGGTACAGAGAAACCTTCCCTTCGAAAGAGCGACCATT

ATGGCGGCATTCACCGGGAACACTGAAGGCAGAACGTCCGACATGAGGACTGAAATCATA

AGAATGATGGAAAGTGCCAAACCAGAAGATGTGTCTTTCCAGGGGCGGGGAGTCTTCGAG

CTCTCGGACGAAAAGGCAACGAACCCGATCGTGCCTTCCTTTGACATGAGTAATGAAGGA

TCTTATTTCTTCGGAGACAATGCAGAGGAGTATGACAATTAAAGAAAAATACCCTTGTTT

CTACT-------------------------------------------------

>A_ciconia_ciconia_Spain_102-1_21VIR1230-2_2021_EPI1860072

-------GCAGGGTAGATAATCACTCACTGAGTGACATCAACATCATGGCGTCTCAAGGC

ACCAAACGATCTTATGAACAAATGGAAACTGGTGGGGAGCGCCAGAATGCCACTGAGATT

AGAGCATCGGTTGGAAGAATGGTTGGTGGAATTGGGAGGTTCTACATACAGATGTGCACT

GAGCTCAAACTCAGCAACTATGAAGGAAGACTGATCCAGAACAGCATAACAATAGAGAGA

ATGGTTCTCTCCGCATTTGATGAAAGGAGAAACAAATATCTGGAAGAACATCCCAGTGCG

GGGAAAGACCCGAAGAAAACAGGAGGTCCAATTTATCGAAGGAGAGATGGGAAATGGGTG

AGAGAGCTGATCCTGTATGACAAAGAGGAGATCAGGAGGATCTGGCGTCAAGCGAATAAT

GGAGAAGACGCAACTGCTGGTCTCACTCACCTGATGATCTGGCATTCCAATCTAAATGAT

GCCACATACCAGAGGACAAGAGCTCTCGTACGTACCGGTATGGACCCCAGGATGTGCTCC

CTCATGCAAGGGTCAACTCTCCCAAGGAGGTCTGGAGCTGCTGGTGCAGCAGTGAAGGGA

GTTGGAACGATGGTAATGGAACTAATTCGGATGATAAAGCGAGGAGTTAATGATCGAAAC

TTCTGGAGAGGCGAGAATGGACGGAGGACAAGGATTGCATTTGAGAGAATGTGCAATATT

CTCAAAGGGAAATTCCAAACAGCAGCACAAAGAGCAATGATGGATCAAGTGCGTGAAAGC

AGGAATCCTGGCAATGCTGAAATTGAAGATCTCATTTTTCTGGCGCGGTCTGCTCTCATT

CTGAGAGGATCAGTGGCCCATAAGTCCTGCTTGCCCGCTTGTGTATACGGGCTCGCTGTA

GCCAGTGGATACGACTTTGAGAGAGAAGGGTACTCTCTAGTTGGAATAGACCCTTTCCGA

CTGCTTCAAAACAGCCAGGTCTTTAGTCTCATTAGACCAAATGAGAATCCAGCACACAAG

AGTCAATTGGTGTGGATGGCATGTCATTCTGCAGCATTCGAGGATCTGAGAGTCTCAAGT

TTCATCAGAGGAACAAGAGTAGTCCCAAGAGGACAACTATCCACCAGAGGAGTTCAAATT

GCTTCAAATGAGAACATAGAAACAATGGACTCCAGCACTCTTGAACTGAGAAGCAGATAT

TGGGCTATAAGAACCAGGAGTGGAGGAAACACCAACCAACAGAGAGCATCTGCAGGACAA

ATCAGTGTACAGCCCACTTTCTCGGTACAGAGAAACCTTCCCTTCGAAAGAGCGACCATT

ATGGCGGCATTCACAGGGAACACTGAAGGCAGAACGTCCGACATGAGGACTGAAATCATA

AGAATGATGGAAAGTGCCAAACCAGAAGATGTGTCTTTCCAGGGGCGGGGAGTCTTCGAG

CTCTCGGACGAAAAGGCAACGAACCCGATCGTGCCTTCCTTTGACATGAGTAATGAAGGA

TCTTATTTCTTCGGAGACAATGCAGAGGAGTATGACAATTAAAGAAAAATACCCTTGTTT

CTACT-------------------------------------------------

>A_duck_Jiangsu_k1203_2010_EPI442018

------------------------------------------TCAATGGCGTCTCAAGGC

ACCAAACGATCTTATGAACAGATGGAAACTGGTGGAGAACGCCAGAATGCCACTGAAATC

AGAGCATCTGTTGGAAGAATGGTTGGTGGAATTGGGAGGTTTTACATACAGATGTGCACT

GAACTCAAACTCAGCGATTATGAGGGGAGACTGATCCAGAACAGCATAACAATAGAAAGA

ATGGTTCTCTCTGCATTTGATGAAAGGAGGAACAAGTACCTGGAAGAACATCCCAGTGCG

GGGAAGGACCCAAAGAAAACTGGAGGTCCAATCTACAGAAGAAGAGACGGAAAGTGGATG

AGGGAGCTGATTCTGTATGACAAAGAAGAGATCAGAAGGATCTGGCGTCAAGCAAATAAT

GGAGAAGATGCAACTGCTGGTCTCACCCATCTGATGATCTGGCACTCCAACCTGAATGAT

GCCACATACCAGAGAACAAGAGCTCTCGTGCGCACTGGAATGGATCCCAGAATGTGCTCT

CTGATGCAAGGATCAACTCTCCCAAGGAGGTCTGGAGCTGCTGGTGCAGCAGTAAAAGGG

GTCGGAACAATGGTAATGGAATTGATTCGGATGATAAAGCGAGGGATTAATGATCGGAAT

TTCTGGAGAGGCGAAAATGGACGAAGGACAAGGATTGCCTATGAGAGAATGTGCAACATC

CTCAAAGGGAAATTTCAAACAGCAGCACAAAGAGCAATGATGGATCAAGTGCGAGAAAGC

AGGAATCCTGGGAATGCTGAAATTGAAGATCTCATCTTTCTGGCACGGTCTGCACTCATC

CTGAGAGGATCAGTGGCCCATAAGTCCTGCCTGCCTGCTTGTGTTTACGGACTTGCTGTG

GCCAGTGGATATGACTTTGAGAGAGAAGGATACTCTCTGGTTGGAATAGACCCTTTCCGT

CTGCTTCAAAACAGCCAGGTCTTCAGTCTCATTAGACCAAATGAAAACCCAGCACATAAA

AGCCAGTTGGTATGGATGGCATGCCATTCAGCAGCGTTTGAGGACCTGAGGGTATCAAGT

TTCATCAGAGGGACAAGAGTGGTCCCAAGAGGACAATTATCCACCAGAGGAGTTCAAATT

GCATCAAATGAAAACATGGAAACAATGGACTCCAGCACTCTTGAATTGAGGAGCAGATAC

TGGGCTATAAGAACCAGGAGTGGAGGAAACACCAACCAACAGAGAGCTTCTGCAGGACAA

ATCAGCGTACAACCTACCTTCTCAGTACAGAGAAATCTTCCCTTCGAAAGAGCGACCATC

ATGGCGGCATTTACAGGGAACACTGAAGGCAGGACATCTGACATGAGGACTGAAATCATA

AGAATGATGGAAAGTGCCAAACCAGAAGATGTGTCCTTCCAGGGGCGGGGAGTCTTCGAG

CTCTCGGACGAAAAGGCAACGAACCCGATCGTGCCTTCCTTTGACATGAGTAACGAAGGA

TCTTATTTCTTCGGAGACAATGCA------------------------------------

------------------------------------------------------

>A_Turkey_Egypt_AR550_2018_EPI1420339

------AAGCAGGTAGATAATCACTCACTGAGTGACATCAACATCATGGCGTCTCAAGGC

ACCAAACGATCTTATGAACAGATGGAAACTGGTGGGGAGCGCCAGAATGCTACTGAGATC

AGAGCATCTGTTGGAAGAATGGTTGGTGGAATTGGGAGGTTCTACATACAGATGTGCACT

GAGCTCAAACTCAGCGACTATGAAGGAAGGCTGATCCAGAACAGCATAACAATAGAGAGA

ATGGTTCTCTCCGCATTTGATGAAAGGAGAAACAAATATCTGGAAGAACATCCCAGTGCG

GGGAAAGACCCGAAGAAAACTGGAGGTCCAATTTATCGAAGGAGAGATGGGAAATGGGTG

AGAGAGCTAATCCTGTATGACAAAGAGGAGATCAGGAGGATCTGGCGTCAAGCGAACAAT

GGGGAAGACGCAACTGCTGGTCTCACTCACCTGATGATCTGGCATTCCAATCTAAATGAT

GCCACATACCAGAGGACAAGAGCTCTCGTGCGTACTGGGATGGACCCCAGGATGTGCTCT

CTCATGCAAGGATCAACTCTCCCAAGGAGGTCTGGAGCTGCTGGTGCAGCAGTAAAGGGA

ATCGGGACGATGGTGATGGAACTAATTCGGATGATAAAGCGAGGAATTAATGATCGAAAC

TTCTGGAGAGGCGAGAATGGACGGAGGACAAGGATTGCATATGAGAGAATGTGCAACATC

CTCAAAGGGAAATTCCAAACAGCAGCACAAAGAGCAATGATGGATCAAGTGCGTGAAAGC

AGGAATCCTGGCAATGCTGAAATTGAAGATCTCATCTTTCTGGCACGGTCTGCTCTCATC

CTGAGAGGATCAGTGGCCCATAAGTCCTGCTTGCCTGCTTGTGTGTACGGACTCGCTGTG

GCCAGTGGATATGACTTTGAGAGAGAAGGGTACTCTCTAGTTGGAATAGATCCTTTCCGT

CTGCTTCAAAACAGCCAGGTCTTCAGTCTCATTAGACCAAATGAGAATCCAGCACACAAG

AGTCAATTGGTATGGATGGCATGTCATTCTGCAGCATTCGAGGATCTGAGAGTCTCAAGT

TTCATCAGAGGAACAAGAGTAGTCCCAAGAGGACAATTATCCACCAGAGGAGTTCAAATT

GCTTCAAATGAGAACATGGAAACAATGGACTCCAGCACTCTTGAACTGAGAAGCAGATAT

TGGGCTATAAGAACCAGGAGCGGAGGAAACACCAACCAACAGAGAGCATCTGCAGGACAA

ATCAGTGTACAGCCCACTTTCTCGGTACAGAGAAACCTTCCCTTCGAAAGAGTGACCATT

ATGGCGGCATTCACAGGGAACACTGAGGGCAGAACGTCCGACATGAGGACTGAAATCATA

AGAATGATGGAAAGTGCCAGACCAGAAGATGTGTCTTTCCAAGGGCGGGGAGTCTTCGAG

CTCTCGGACGAAAAGGCAACGAACCCGATCGTGCCTTCCTTTGACATGAGTAATGAAGGA

TCTTATTTCTTCGGAGACAATGCAGAGGAGTATGACAATTAAAGAAAAATACC-------

------------------------------------------------------

>A_Chicken_Egypt_AI20286_2019_EPI1638785

---------------------------------------------ATGGCGTCTCAAGGC

ACCAAACGATCTTATGAACAGATGGAAACTGGTGGGGAGCGCCAGAATGCCACTGAGATC

AGAGCATCTGTTGGAAGAATGATTGGTGGAATTGGGAGGTTCTACATACAGATGTGCACT

GAGCTCAAACTCAGCGACTATGAAGGAAGGCTGATCCAGAACAGCATAACAATAGAGAGA

ATGGTTCTCTCCGCATTTGATGAAAGGAGAAACAAATATCTGGAAGAACATCCCAGTGCG

GGGAAAGACCCGAAGAAAACTGGAGGTCCAATTTATCGAAGGAGAGATGGGAAATGGGTG

AGAGAGCTGATCCTGTATGACAAAGAGGAGATCAGGAGGATCTGGCGTCAAGCGAACAAT

GGAGAAGACGCAACTGCTGGTCTCACTCACCTGATGATCTGGCATTCCAATCTAAATGAT

GCCACATACCAGAGGACAAGAGCTCTCGTGCGTACTGGGATGGACCCCAGGATGTGCTCT

CTCATGCAAGGATCAACTCTCCCAAGGAGGTCTGGAGCAGCTGGTGCAGCGGTAAAGGGA

GTCGGGACGATGGTGATGGAACTAATCCGGATGATAAAGCGAGGTATTAATGATCGAAAC

TTCTGGAGAGGCGAGAATGGACGGAGGACAAGGATTGCATATGAGAGAATGTGCAACATC

CTCAAAGGGAAATTCCAAACAGCAGCACAAAGAGCAATGATGGATCAGGTGCGTGAGAGC

AGAAATCCTGGCAATGCTGAAATTGAAGATCTCATCTTTCTGGCACGGTCTGCTCTCATC

CTGAGAGGATCAGTGGCCCATAAGTCCTGCTTGCCTGCTTGTGTGTACGGACTCGCTGTG

GCTAGTGGATACGACTTTGAGAGAGAAGGGTACTCTCTAGTTGGAATAGACCCTTTCCGT

CTGCTTCAAAACAGCCAGGTCTTCAGTCTCATTAGACCAAATGAGAATCCAGCACACAAG

AGTCAATTGGTGTGGATGGCATGTCATTCTGCAGCATTCGAGGATCTGAGAGTCTCAAGT

TTCATCAGAGGAACAAGAGTAGTCCCAAGAGGACAACTATCCACCAGAGGAGTTCAAATT

GCTTCAAATGAGAACATGGAAACAATGGACTCCAGCACTCTTGAACTGAGAAGCAGATAT

TGGGCUAUAAGAACCAGGAGTGGAGGAAACACCAACCAACAGAGAGCATCTGCAGGACAA

AUCAGUGUACAGCCCACTTTCTCGGTACAGAGAAACCTTCCCTTCGAAAGAGCGACCATT

ATGGCGGCATTCACAGGGAACACTGAGGGCAGAACGTCCGACATGAGGACTGAAATCATA

AGAATGATGGAAAGTGCCAGACCAGAAGATGTGTCTTTCCAGGGGCGGGGAGTCTTCGAG

CTCTCGGACGAAAAGGCAACGAACCCGATCGTGCCTTCCTTTGACATGAGTAATGAAGGA

TCTTATTTCTTCGGAGACAATGCAGAGGAGTATGACAATTA-------------------

------------------------------------------------------

>A_Turkey_Egypt_AI20285_2019_EPI1638798

------------------------------------------------GCGTCTCAAGGC

ACCAAACGATCTTATGAACAGATGGAAACTGGTGGGGAGCGCCAGAATGCCACTGAAATC

AGAGCATCTGTTGGAAGAATGGTTGGTGGAATTGGGAGGTTCTACATACAGATGTGCACT

GAGCTCAAACTCAGCGACTATGAAGGAAGGCTGATCCAGAACAGCATAACAATAGAGAGA

ATGGTTCTCTCCGCATTTGATGAAAGGAGAAACAAATATCTGGAAGAACATCCCAGTGCG

GGGAAAGACCCGAAGAAAACTGGAGGTCCAATTTACCGAAGGAGAGATGGGAAATGGGTG

AGAGAGCTGATCCTGTATGACAAAGAGGAGATCAGGAGGATCTGGCGTCAAGCGAACAAT

GGAGAAGACGCAACTGCTGGTCTCACTCACCTGATGATCTGGCATTCCAATCTAAATGAT

GCCACATACCAGAGGACAAGGGCTCTCGTGCGTACTGGGATGGACCCCAGGATGTGCTCT

CTCATGCAAGGATCAACTCTCCCAAGGAGGTCTGGAGCTGCTGGTGCAGCAGTAAAGGGG

GTCGGGACAATGGTGATGGAACTAATTCGGATGATAAAGCGAGGAATTAATGATCGAAAC

TTCTGGAGAGGCGAGAATGGACGGAGGACAAGGATTGCATATGAGAGAATGTGCAACATC

CTCAAAGGGAAATTCCAAACAGCAGCACAAAGAGCAATGATGGATCAGGTGCGTGAAAGC

AGGAATCCTGGTAATGCTGAAATTGAAGATCTCATCTTTCTGGCACGGTCTGCTCTCATC

CTGAGAGGATCAGTGGCCCATAAGTCCTGCTTGCCTGCTTGTGTGTACGGACTCGCTGTG

GCCAGTGGATACGACTTTGAGAGAGAAGGGTACTCTCTAGTTGGAATAGACCCTTTCCGT

CTGCTTCAAAACAGCCAGGTCTTCAGTCTCATTAGACCAAATGAGAATCCAGCACACAAG

AGTCAATTGGTGTGGATGGCATGTCATTCTGCAGCATTCGAGGATCTGAGAGTCTCAAGT

TTCATCAGAGGAACAAGAGTAGTCCCAAGAGGACAACTATCCACCAGAGGAGTTCAAATT

GCTTCAAATGAGAACATGGAAACAATGGACTCCAGCACTCTTGAACTGAGAAGCAGATAT

TGGGCUAUAAGAACCAGGAGTGGAGGAAACACCAACCAACAGAGAGCATCTGCAGGACAA

AUCAGUGUACAGCCCACTTTCTCGGTACAGAGAAACCTTCCCTTCGAAAGAGCGACCATT

ATGGCGGCATTCACAGGGAACACTGAGGGCAGAACGTCCGACATGAGGACTGAAATCATA

AGAATGATGGAAAGTGCCAGACCAGAAGATGTGTCTTTCCAGGGGCGGGGAGTCTTCGAG

CTCTCGGACGAAAAGGCAACGAACCCGATCGTGCCTTCCTTTGACATGAGTAATGAAGGA

TCTTATTTCTTCGGAGACAATGCAGAGGAGTATGACAATTAAA-----------------

------------------------------------------------------

>A_Chicken_Egypt_AR553_2018_EPI1638802

---------------------------------------------ATGGCGTCTCAAGGC

ACCAAACGATCTTATGAACAGATGGAAACTGGTGGGGAGCGCCAGAATGCTACTGAGATC

AGAGCATCTGTTGGAAGAATGGTTGGTGGAATTGGGAGGTTCTACATACAGATGTGCACT

GAGCTCAAACTCAGCGACTATGAAGGAAGGCTGATCCAGAACAGCATAACAATAGAGAGA

ATGGTTCTCTCCGCATTTGATGAAAGGAGAAACAAATATCTGGAAGAACATCCCAGTGCG

GGGAAAGACCCGAAGAAAACTGGAGGTCCAATTTATCGAAGGAGAGATGGGAAATGGGTG

AGAGAGCTAATCCTGTATGACAAAGAGGAGATCAGGAGGATCTGGCGTCAAGCGAACAAT

GGGGAAGACGCAACTGCTGGTCTCACTCACCTGATGATCTGGCATTCCAATCTAAATGAT

GCCACATACCAGAGGACAAGAGCTCTCGTGCGTACTGGGATGGACCCCAGGATGTGCTCT

CTCATGCAAGGATCAACTCTCCCAAGGAGGTCTGGAGCTGCTGGTGCAGCAGTAAAGGGA

ATCGGGACGATGGTGATGGAACTAATTCGGATGATAAAGCGAGGAATTAATGATCGAAAC

TTCTGGAGAGGCGAGAATGGACGGAGGACAAGGATTGCATATGAGAGAATGTGCAACATC

CTCAAAGGGAAATTCCAAACAGCAGCACAAAGAGCAATGATGGATCAAGTGCGTGAAAGC

AGGAATCCTGGCAATGCTGAAATTGAAGATCTCATCTTTCTGGCACGGTCTGCTCTCATC

CTGAGAGGATCAGTGGCCCATAAGTCCTGCTTGCCTGCTTGTGTGTACGGACTCGCTGTG

GCCCGTGGATACGACTTTGAGAGAGAAGGGTACTCTCTAGTTGGAATAGATCCTTTCCGT

CTGCTTCAAAACAGCCAGGTCTTCAGTCTCATTAGACCAAATGAGAATCCAGCACACAAG

AGTCAATTGGTGTGGATGGCATGTCATTCTGCAGCATTCGAGGATCTGAGAGTCTCAAGT

TTCATCAGAGGAACAAGAGTAGTCCCAAGAGGACAACTATCCACCAGAGGAGTTCAAATT

GCTTCAAATGAGAACATGGAAACAATGGACTCCAGCACTCTTGAACTGAGAAGCAGATAT

TGGGCTATAAGAACCAGGAGTGGAGGAAACACCAACCAACAGAGAGCATCTGCAGGACAA

ATCAGTGTACAGCCCACTTTCTCGGTACAGAGAAACCTTCCCTTCGAAAGAGCGACCATT

ATGGCGGCATTCACAGGGAACACTGAGGGCAGAACGTCCGACATGAGGACTGAAATCATA

AGAATGATGGAAAGTGCCAGACCAGAAGATGTGTCTTTCCAAGGGCGGGGAGTCTTCGAG

CTCTCGGACGAAAAGGCAACGAACCCGATCGTGCCTTCCTTTGACATGAGTAATGAAGGA

TCTTATTTCTTCGGAGACAATGCAGAGGAGTATGACAATTA-------------------

------------------------------------------------------

>A_goose_Omsk_0111_2020_EPI1813138

AGCAAAAGCAGGGTAGATAATCACTCACTGAGTGACATCAACATCATGGCGTCTCAAGGC

ACCAAACGATCTTATGAACAGATGGAAACTAGTGGGGAGCGCCAGAATGCCACTGAGATT

AGAGCATCGGTTGGAAGAATGGTTGGTGGAATTGGGAGGTTCTACATACAGATGTGCACT

GAGCTCAAACTCAGCGACTATGAAGGAAGACTGATCCAGAACAGCATAACAATAGAGAGA

ATGGTTCTCTCCGCATTTGATGAAAGGAGAAACAAATATCTGGAAGAACATCCCAGTGCG

GGGAAAGACCCGAAGAAAACAGGAGGTCCAATTTATCGAAGGAGAGATGGGAAATGGGTG

AGAGAGCTGATCCTGTATGACAAAGAGGAGATCAGGAGGATCTGGCGTCAAGCGAATAAT

GGAGAAGACGCAACTGCTGGTCTCACTCACCTGATGATCTGGCATTCCAATCTAAATGAT

GCCACATACCAGAGGACAAGAGCTCTCGTGCGTACCGGTATGGACCCCAGGATGTGCTCC

CTCATGCAAGGGTCAACTCTCCCAAGGAGGTCTGGAGCTGCTGGTGCAGCAGTGAAGGGA

GTTGGAACGATGGTGATGGAACTAATTCGGATGATAAAGCGAGGAGTTAATGATCGAAAC

TTCTGGAGAGGCGAGAATGGACGGAGGACAAGGATTGCATTTGAGAGAATGTGCAATATT

CTCAAAGGGAAATTCCAAACAGCAGCACAAAGAGCAATGATGGATCAAGTGCGTGAAAGC

AGGAATCCTGGCAATGCTGAAATTGAAGATCTCATTTTTCTGGCGCGGTCTGCTCTCATT

CTGAGAGGATCAGTGGCCCATAAGTCCTGCTTGCCCGCTTGTGTATACGGGCTCGCTGTA

GCCAGTGGATACGACTTTGAGAGAGAAGGGTACTCTCTAGTTGGAATAGACCCTTTCCGA

CTGCTTCAAAACAGCCAGGTCTTTAGTCTCATTAGACCAAATGAGAATCCAGCACACAAG

AGTCAATTGGTGTGGATGGCATGTCATTCTGCAGCATTCGAGGATCTGAGAGTCTCAAGT

TTCATCAGAGGAACAAGAGTAGTCCCAAGAGGACAACTATCCACTAGAGGAGTTCAAATT

GCTTCAAATGAGAACATAGAAACAATGGACTCCAGCACTCTTGAACTGAGAAGCAGATAT

TGGGCTATAAGAACCAGGAGTGGAGGAAACACCAACCAACAGAGAGCATCTGCAGGACAA

ATCAGTGTACAGCCCACTTTCTCGGTACAGAGAAACCTTCCCTTCGAAAGAGCGACCATT

ATGGCGGCATTCACAGGGAACACTGAAGGCAGAACGTCCGACATGAGGACTGAAATCATA

AGAATGATGGAAAGTGCCAGACCAGAAGATGTGTCTTTCCAGGGGCGGGGAGTCTTCGAG

CTCTCGGACGAAAAGGCAACGAACCCGATCGTGCCTTCCTTTGACATGAGTAATGAAGGA

TCTTATTTCTTCGGAGACAATGCAGAGGAGTATGACAATTAAAGAAAAATACCCTTGTTT

CTACT-------------------------------------------------

>A_goose_Omsk_01161_2020_EPI1813354

AGCAAAAGCAGGGTAGATAATCACTCACTGAGTGACATCAACATCATGGCGTCTCAAGGC

ACCAAACGATCTTATGAACAGATGGAAACTGGTGGGGAGCGCCAGAATGCCACTGAGATT

AGAGCATCGGTTGGAAGAATGGTTGGTGGAATTGGGAGGTTCTACATACAGATGTGCACT

GAGCTCAAACTCAGCGACTATGAAGGAAGACTGATCCAGAACAGCATAACAATAGAGAGA

ATGGTTCTCTCCGCATTTGATGAAAGGAGAAACAAATATCTGGAAGAACATCCCAGTGCG

GGGAAAGACCCGAAGAAAACAGGAGGTCCAATTTATCGAAGGAGAGATGGGAAATGGGTG

AGAGAGCTGATCCTGTATGACAAAGAGGAGATCAGGAGGATCTGGCGTCAAGCGAATAAT

GGAGAAGACGCAACTGCTGGTCTCACTCACCTGATGATCTGGCATTCCAATCTAAATGAT

GCCACATACCAGAGGACAAGAGCTCTCGTGCGTACCGGTATGGACCCCAGGATGTGCTCC

CTCATGCAAGGGTCAACTCTCCCAAGGAGGTCTGGAGCTGCTGGTGCAGCAGTGAAGGGA

GTTGGAACGATGGTGATGGAACTAATTCGGATGATAAAGCGAGGAGTTAATGATCGAAAC

TTCTGGAGAGGCGAGAATGGACGGAGGACAAGGATTGCATTTGAGAGAATGTGCAATATT

CTCAAAGGGAAATTCCAAACAGCAGCACAAAGAGCAATGATGGATCAAGTGCGTGAAAGC

AGGAATCCTGGCAATGCTGAAATTGAAGATCTCATTTTTCTGGCGCGGTCTGCTCTCATT

CTGAGAGGATCAGTGGCCCATAAGTCCTGCTTGCCCGCTTGTGTATACGGGCTCGCTGTA

GCCAGTGGATACGACTTTGAGAGAGAAGGGTACTCTCTAGTTGGAATAGACCCTTTCCGA

CTGCTTCAAAACAGCCAGGTCTTTAGTCTCATTAGACCAAATGAGAATCCAGCACACAAG

AGTCAATTGGTGTGGATGGCATGTCATTCTGCAGCATTCGAGGATCTGAGAGTCTCAAGT

TTCATCAGAGGAACAAGAGTAGTCCCAAGAGGACAACTATCCACTAGAGGAGTTCAAATT

GCTTCAAATGAGAACATAGAAACAATGGACTCCAGCACTCTTGAACTGAGAAGCAGATAT

TGGGCTATAAGAACCAGGAGTGGAGGAAACACCAACCAACAGAGAGCATCTGCAGGACAA

ATCAGTGTACAGCCCACTTTCTCGGTACAGAGAAACCTTCCCTTCGAAAGAGCGACCATT

ATGGCGGCATTCACAGGGAACACTGAAGGCAGGACGTCCGACATGAGGACTGAAATCATA

AGAATGATGGAAAGTGCCAGACCAGAAGATGTGTCTTTCCAGGGGCGGGGAGTCTTCGAG

CTCTCGGACGAAAAGGCAACGAACCCGATCGTGCCTTCCTTTGACATGAGTAATGAAGGA

TCTTATTTCTTCGGAGACAATGCAGAGGAGTATGACAATTAAAGAAAAATACCCTTGTTT

CTACT-------------------------------------------------

>A_chicken_Omsk_0118_2020_EPI1813370

AGCAAAAGCAGGGTAGATAATCACTCACTGAGTGACATCAACATCATGGCGTCTCAAGGC

ACCAAACGATCTTATGAACAGATGGAAACTAGTGGGGAGCGCCAGAATGCCACTGAGATT

AGAGCATCTGTTGGAAGAATGGTTGGTGGAATTGGGAGGTTCTACATACAGATGTGCACT

GAGCTCAAACTCAGCGACTATGAAGGAAGACTGATCCAGAACAGCATAACAATAGAGAGA

ATGGTTCTCTCCGCATTTGATGAAAGGAGAAACAAATATCTGGAAGAACATCCCAGTGCG

GGGAAAGACCCGAAGAAAACAGGAGGTCCAATTTATCGAAGGAGAGATGGGAAATGGGTG

AGAGAGCTGATCCTGTATGACAAAGAGGAGATCAGGAGGATCTGGCGTCAAGCGAATAAT

GGAGAAGACGCAACTGCTGGTCTCACTCACCTGATGATCTGGCATTCCAATCTAAATGAT

GCCACATACCAGAGGACAAGAGCTCTCGTGCGTACCGGTATGGACCCCAGGATGTGCTCC

CTCATGCAAGGGTCAACTCTCCCAAGGAGGTCTGGAGCTGCTGGTGCAGCAGTGAAGGGA

GTTGGAACGATGGTGATGGAACTAATTCGGATGATAAAGCGAGGAGTTAATGATCGAAAC

TTCTGGAGAGGCGAGAATGGACGGAGGACAAGGATTGCATTTGAGAGAATGTGCAATATT

CTCAAAGGGAAATTCCAAACAGCAGCACAAAGAGCAATGATGGATCAAGTGCGTGAAAGC

AGGAATCCTGGCAATGCTGAAATTGAAGATCTCATTTTTCTGGCGCGGTCTGCTCTCATT

CTGAGAGGATCAGTGGCCCATAAGTCCTGCTTGCCCGCTTGTGTATACGGGCTCGCTGTA

GCCAGTGGATACGACTTTGAGAGAGAAGGATACTCTCTAGTTGGAATAGACCCTTTCCGA

CTGCTTCAAAACAGCCAGGTCTTTAGTCTCATTAGACCAAATGAGAATCCAGCACACAAG

AGTCAATTGGTGTGGATGGCATGTCATTCTGCAGCATTCGAGGATCTGAGAGTCTCAAGT

TTCATCAGAGGAACAAGAGTAGTCCCAAGAGGACAACTATCCACTAGAGGAGTTCAAATT

GCTTCAAATGAGAACATAGAAACAATGGACTCCAGCACTCTTGAACTGAGAAGCAGATAT

TGGGCTATAAGAACCAGGAGTGGAGGAAACACCAACCAACAGAGAGCATCTGCAGGACAA

ATCAGTGTACAGCCCACTTTCTCGGTACAGAGAAACCTTCCCTTCGAAAGAGCGACCATT

ATGGCGGCATTCACAGGGAACACTGAAGGCAGAACGTCCGACATGAGGACTGAAATCATA

AGAATGATGGAAAGTGCCAGACCAGAAGATGTGTCTTTCCAGGGGCGGGGAGTCTTCGAG

CTCTCGGACGAAAAGGCAACGAACCCGATCGTGCCTTCCTTTGACATGAGTAATGAAGGA

TCTTATTTCTTCGGAGACAATGCAGAGGAGTATGACAATTAAAGAAAAATACCCTTGTTT

CTACT-------------------------------------------------

>A_chicken_Omsk_0119_2020_EPI1813378

AGCAAAAGCAGGGTAGATAATCACTCACTGAGTGACATCAACATCATGGCGTCTCAAGGC

ACCAAACGATCTTATGAACAGATGGAAACTAGTGGGGAGCGCCAGAATGCCACTGAGATT

AGAGCATCTGTTGGAAGAATGGTTGGTGGAATTGGGAGGTTCTACATACAGATGTGCACT

GAGCTCAAACTCAGCGACTATGAAGGAAGACTGATCCAGAACAGCATAACAATAGAGAGA

ATGGTTCTCTCCGCATTTGATGAAAGGAGAAACAAATATCTGGAAGAACATCCCAGTGCG

GGGAAAGACCCGAAGAAAACAGGAGGTCCAATTTATCGAAGGAGAGATGGGAAATGGGTG

AGAGAGCTGATCCTGTATGACAAAGAGGAGATCAGGAGGATCTGGCGTCAAGCGAATAAT

GGAGAAGACGCAACTGCTGGTCTCACTCACCTGATGATCTGGCATTCCAATCTAAATGAT

GCCACATACCAGAGGACAAGAGCTCTCGTGCGTACCGGTATGGACCCCAGGATGTGCTCC

CTCATGCAAGGGTCAACTCTCCCAAGGAGATCTGGAGCTGCTGGTGCAGCAGTGAAGGGA

GTTGGAACGATGGTGATGGAACTAATTCGGATGATAAAGCGAGGAGTTAATGATCGAAAC

TTCTGGAGAGGCGAGAATGGACGGAGGACAAGGATTGCATTTGAGAGAATGTGCAATATT

CTCAAAGGGAAATTCCAAACAGCAGCACAAAGAGCAATGATGGATCAAGTGCGTGAAAGC

AGGAATCCTGGCAATGCTGAAATTGAAGATCTCATTTTTCTGGCGCGGTCTGCTCTCATT

CTGAGAGGATCAGTGGCCCATAAGTCCTGCTTGCCCGCTTGTGTATACGGGCTCGCTGTA

GCCAGTGGATACGACTTTGAGAGAGAAGGGTACTCTCTAGTTGGAATAGACCCTTTCCGA

CTGCTTCAAAACAGCCAGGTCTTTAGTCTCATTAGACCAAATGAGAATCCAGCACACAAG

AGTCAATTGGTGTGGATGGCATGTCATTCTGCAGCATTCGAGGATCTGAGAGTCTCAAGT

TTCATCAGAGGAACAAGAGTAGTCCCAAGAGGACAACTATCCACTAGAGGAGTTCAAATT

GCTTCAAATGAGAACATAGAAACAATGGACTCCAGCACTCTTGAACTGAGAAGCAGATAT

TGGGCTATAAGAACCAGGAGTGGAGGAAACACCAACCAACAGAGAGCATCTGCAGGACAA

ATCAGTGTACAGCCCACTTTCTCGGTACAGAGAAACCTTCCCTTCGAAAGAGCGACCATT

ATGGCGGCATTCACAGGGAACACTGAAGGCAGAACGTCCGACATGAGGACTGAAATCATA

AGAATGATGGAAAGTGCCAGACCAGAAGATGTGTCTTTCCAGGGGCGGGGAGTCTTCGAG

CTCTCGGACGAAAAGGCAACGAGCCCGATCGTGCCTTCCTTTGACATGAGTAATGAAGGA

TCTTATTTCTTCGGAGACAATGCAGAGGAGTATGACAATTAAAGAAAAATACCCTTGTTT

CTACT-------------------------------------------------

>A_chicken_Omsk_0073_2020_EPI1813402

AGCAAAAGCAGGGTAGATAATCACTCACTGAGTGACACCAACATCATGGCGTCTCAAGGC

ACCAAACGATCTTATGAACAGATGGAAACTGGTGGGGAGCGCCAGAATGCCACTGAGATT

AGAGCATCGGTTGGAAGAATGGTTGGTGGAATTGGGAGGTTCTACATACAGATGTGCACT

GAGCTCAAACTCAGCGACTATGAAGGAAGACTGATCCAGAACAGCATAACAATAGAGAGA

ATGGTTCTCTCCGCATTTGATGAAAGGAGAAACAAATATCTAGAAGAACATCCCAGTGCG

GGGAAAGACCCGAAGAAAACAGGAGGTCCAATTTATCGAAGGAGAGATGGGAAATGGGTG

AGAGAGCTGATCCTGTATGACAAAGAGGAGATCAGGAGGATCTGGCGTCAAGCGAATAAT

GGAGAAGACGCAACTGCTGGTCTCACTCACCTGATGATCTGGCATTCCAATCTAAATGAT

GCCACATACCAGAGGACAAGAGCTCTCGTGCGTACCGGTATGGACCCCAGGATGTGCTCC

CTCATGCAAGGGTCAACTCTCCCAAGGAGGTCTGGAGCTGCTGGTGCAGCAGTGAAGGGA

GTTGGAACGATGGTGATGGAACTAATTCGGATGATAAAGCGAGGAGTTAATGATCGAAAC

TTCTGGAGAGGCGAGAATGGACGGAGGACAAGGATTGCATTTGAGAGAATGTGCAATATT

CTCAAAGGGAAATTCCAAACAGCAGCACAAAGAGCAATGATGGATCAAGTGCGTGAAAGC

AGGAATCCTGGCAATGCTGAAATTGAAGATCTCATTTTTCTGGCGCGGTCTGCTCTCATT

CTGAGAGGATCAGTGGCCCATAAGTCCTGCTTGCCCGCTTGTGTATACGGGCTCGCTGTA

GCCAGTGGATACGACTTTGAGAGAGAAGGGTACTCTCTAGTTGGAATAGACCCTTTCCGA

CTGCTTCAAAACAGCCAGGTCTTTAGTCTCATTAGACCAAATGAGAATCCAGCACACAAG

AGTCAATTGGTGTGGATGGCATGTCACTCTGCAGCATTCGAGGATCTGAGAGTCTCAAGT

TTCATCAGAGGAACAAGAGTAGTCCCAAGAGGACAACTATCCACTAGAGGAGTTCAAATT

GCTTCAAATGAGAACATAGAAACAATGGACTCCAGCACTCTTGAACTGAGAAGCAGATAT

TGGGCTATAAGAACCAGGAGTGGAGGAAACACCAACCAACAGAGAGCATCTGCAGGACAA

ATCAGTGTACAGCCCACTTTCTCGGTACAGAGAAACCTTCCCTTCGAAAGAGCGACCATT

ATGGCAGCATTCACAGGGAACACTGAAGGCAGAACGTCCGACATGAGGACTGAAATCATA

AGAATGATGGAAAGTGCCAGACCAGAAGATGTGTCTTTCCAGGGGCGGGGAGTCTTCGAG

CTCTCGGACGAAAAGGCAACGAACCCGATCGTGCCTTCCTTTGACATGAGTAATGAAGGA

TCTTATTTCTTCGGAGACAATGCAGAGGAGTATGACAATTAAAGAAAAATACCCTTGTTT

CTACT-------------------------------------------------

>A_chicken_Omsk_30007_2020_EPI1814306

AGCAAAAGCAGGGTAGATAATCACTCACTGAGTGACATCAACATCATGGCGTCTCAAGGC

ACCAAACGATCTTATGAACAGATGGAAACTGGTGGGGAGCGCCAGAATGCCACTGAGATT

AGAGCATCGGTTGGAAGAATGGTTGGTGGAATTGGGAGGTTCTACATACAGATGTGCACT

GAGCTCAAACTCAGCGACTATGAAGGAAGACTGATCCAGAACAGCATAACAATAGAGAGA

ATGGTTCTCTCCGCATTTGATGAAAGGAGAAACAAATATCTGGAAGAACATCCCAGTGCG

GGGAAAGACCCGAAGAAAACAGGAGGTCCAATTTATCGAAGGAGAGATGGGAAATGGGTG

AGAGAGCTGATCCTGTATGACAAAGAGGAGATCAGGAGGATCTGGCGCCAAGCGAATAAT

GGAGAAGACGCAACTGCTGGTCTCACTCACCTGATGATCTGGCATTCCAATCTAAATGAT

GCCACATACCAGAGGACAAGAGCTCTCGTGCGTACCGGTATGGACCCCAGGATGTGCTCC

CTCATGCAAGGGTCAACTCTCCCAAGGAGGTCTGGAGCTGCTGGTGCAGCAGTGAAGGGA

GTTGGAACGATGGTGATGGAACTAATTCGGATGATAAAGCGAGGAGTTAATGATCGAAAC

TTCTGGAGAGGCGAGAATGGACGGAGGACAAGGATTGCATTTGAGAGAATGTGCAATATT

CTCAAAGGGAAATTCCAAACAGCAGCACAAAGAGCAATGATGGATCAAGTGCGTGAAAGC

AGGAATCCTGGCAATGCTGAAATTGAAGATCTCATTTTTCTGGCGCGGTCTGCTCTCATT

CTGAGAGGATCAGTGGCCCATAAGTCCTGCTTGCCCGCTTGTGTATACGGGCTCGCTGTA

GCCAGTGGATACGACTTTGAGAGAGAAGGGTACTCTCTAGTTGGAATAGACCCTTTCCGA

CTGCTTCAAAACAGCCAGGTCTTTAGTCTCATTAGACCAAATGAGAATCCGGCACACAAG

AGTCAATTGGTGTGGATGGCATGTCATTCTGCAGCATTCGAGGATCTGAGAGTCTCAAGT

TTCATCAGAGGAACAAGAGTAGTCCCAAGAGGACAACTATCCACTAGAGGAGTTCAAATT

GCTTCAAATGAGAACATAGAAACAATGGACTCCAGCACTCTTGAACTGAGAAGCAGATAT

TGGGCTATAAGAACCAGGAGTGGAGGAAACACCAACCAACAGAGAGCATCTGCAGGACAA

ATCAGTGTACAGCCCACTTTCTCGGTACAGAGAAACCTTCCCTTCGAAAGAGCGACCATT

ATGGCGGCATTCACAGGGAACACTGAAGGCAGAACGTCCGACATGAGGACTGAAATCATA

AGAATGATGGAAAGTGCCAGACCAGAAGATGTGTCTTTCCAGGGGCGGGGAGTCTTCGAG

CTCTCGGACGAAAAGGCAACGAACCCGATCGTGCCTTCCTTTGACATGAGTAATGAAGGA

TCTTATTTCTTCGGAGACAATGCAGAGGAGTATGACAATTAAAGAAAAATACCCTTGTTT

CTACT-------------------------------------------------

>A_goose_Omsk_30009_2020_EPI1814314

AGCAAAAGCAGGGTAGATAATCACTCACTGAGTGACATCAACATCATGGCGTCTCAAGGC

ACCAAACGATCTTATGAACAGATGGAAACTGGTGGGGAGCGCCAGAATGCCACTGAGATT

AGAGCATCGGTTGGAAGAATGGTTGGTGGAATTGGGAGGTTCTACATACAGATGTGCACT

GAGCTCAAACTCAGCGACCATGAAGGAAGACTGATCCAGAACAGCATAACAATAGAGAGA

ATGGTTCTCTCCGCATTTGATGAAAGGAGAAACAAATATCTGGAAGAACATCCCAGTGCG

GGGAAAGACCCGAAGAAAACAGGAGGTCCAATTTATCGAAGGAGAGATGGGAAATGGGTG

AGAGAGCTGATCCTGTATGACAAAGAGGAGATCAGGAGGATCTGGCGCCAAGCGAATAAT

GGAGAAGACGCAACTGCTGGTCTCACTCACCTGATGATCTGGCATTCCAATCTAAATGAT

GCCACATACCAGAGGACAAGAGCTCTCGTACGTACCGGTATGGACCCCAGGATGTGCTCC

CTCATGCAAGGGTCAACTCTCCCAAGGAGGTCTGGAGCTGCTGGTGCAGCAGTGAAGGGA

GTTGGAACGATGGTGATGGAACTAATTCGGATGATAAAGCGAGGAGTTAATGATCGAAAC

TTCTGGAGAGGCGAGAATGGACGGAGGACAAGGATTGCATTTGAGAGAATGTGCAATATT

CTCAAAGGGAAATTCCAAACAGCAGCACAAAGAGCAATGATGGATCAAGTGCGTGAAAGC

AGGAATCCTGGCAATGCTGAAATTGAAGATCTCATTTTTCTGGCGCGGTCTGCTCTCATT

CTGAGAGGATCAGTGGCCCATAAGTCCTGCTTGCCCGCTTGTGTATACGGGCTCGCTGTA

GCCAGTGGATACGACTTTGAGAGAGAAGGGTACTCTCTAGTTGGAATAGACCCTTTCCGA

CTGCTTCAAAACAGCCAGGTCTTTAGTCTCATTAGACCAAATGAGAATCCGGCACACAAG

AGTCAATTGGTGTGGATGGCATGTCATTCTGCAGCATTCGAGGATCTGAGAGTCTCAAGT

TTCATCAGAGGAACAAGAGTAGTCCCAAGAGGACAACTATCCACTAGAGGAGTTCAAATT

GCTTCAAATGAGAACATAGAAACAATGGACTCCAGCACTCTTGAACTGAGAAGCAGATAT

TGGGCTATAAGAACCAGGAGTGGAGGAAACACCAACCAACAGAGAGCATCTGCAGGACAA

ATCAGTGTACAGCCCACTTTCTCGGTACAGAGAAACCTTCCCTTCGAAAGAGCGACCATT

ATGGCGGCATTCACAGGGAACACTGAAGGCAGAACGTCCGACATGAGGACTGAAATCATA

AGAATGATGGAAAGTGCCAGACCAGAAGATGTGTCTTTCCAGGGGCGGGGAGTCTTCGAG

CTCTCGGACGAAAAGGCAACGAACCCGATCGTGCCTTCCTTTGACATGAGTAATGAAGGA

TCTTATTTCTTCGGAGACAATGCAGAGGAGTATGACAATTAAAGAAAAATACCCTTGTTT

CTACT-------------------------------------------------

>A_chicken_Chelyabinsk_201_2020_EPI1814330

AGCAAAAGCAGGGTAGATAATCACTCACTGAGTGACATCAACATCATGGCGTCTCAAGGC

ACCAAACGATCTTATGAACAGATGGAAACTGGTGGGGAGCGCCAGAATGCCACTGAGATT

AGAGCATCGGTTGGAAGAATGGTTGGTGGAATTGGGAGGTTCTACATACAGATGTGCACT

GAGCTCAAACTCAGCGACTATGAAGGAAGACTGATCCAGAACAGCATAACAATAGAGAGA

ATGGTTCTCTCCGCATTTGATGAAAGGAGAAACAAATATCTGGAAGAACATCCCAGTGCG

GGGAAAGACCCGAAGAAAACAGGAGGTCCAATTTATCGAAGGAGAGATGGGAAATGGGTG

AGAGAGCTGATCCTGTATGACAAAGAGGAGATCAGGAGGATCTGGCGTCAAGCGAATAAT

GGAGAAGACGCAACTGCTGGTCTCACTCACCTGATGATCTGGCATTCCAATCTAAATGAT

GCCACATACCAGAGGACAAGAGCTCTCGTGCGTACCGGTATGGACCCCAGGATGTGCTCC

CTCATGCAAGGGTCAACTCTCCCAAGGAGGTCTGGAGCTGCTGGTGCAGCAGTGAAGGGA

GTTGGAACGATGGTGATGGAACTAATTCGGATGATAAAGCGAGGAGTTAATGATCGAAAC

TTCTGGAGAGGCGAGAATGGACGGAGGACAAGGATTGCATTTGAGAGAATGTGCAATATT

CTCAAAGGGAAATTCCAAACAGCAGCACAAAGAGCAATGATGGATCAAGTGCGTGAAAGC

AGGAATCCTGGCAATGCTGAAATTGAAGATCTCATTTTTCTTGCGCGGTCTGCTCTCATT

CTGAGAGGATCAGTGGCTCATAAGTCCTGCTTGCCCGCTTGTGTATACGGGCTCGCTGTA

GCCAGTGGATACGACTTTGAGAGAGAAGGGTACTCTCTAGTTGGAATAGACCCTTTCCGA

CTGCTTCAAAACAGCCAGGTCTTTAGTCTCATTAGACCAAATGAGAATCCAGCACACAAG

AGTCAATTGGTGTGGATGGCATGTCATTCTGCAGCATTCGAGGATCTGAGAGTCTCAAGT

TTCATCAGAGGAACAAGAGTAGTCCCAAGAGGACAACTATCCACTAGAGGAGTTCAAATT

GCTTCAAATGAGAACATAGAAACAATGGACTCCAGCACTCTTGAACTGAGAAGCAGATAT

TGGGCTATAAGAACCAGGAGTGGAGGAAACACCAACCAACAGAGAGCATCTGCAGGACAA

ATCAGTGTACAGCCCACTTTCTCGGTACAGAGAAACCTTCCCTTCGAAAGAGCGACCATT

ATGGCGGCATTCACAGGGAACACTGAAGGCAGAACGTCCGACATGAGGACTGAAATCATA

AGAATGATGGAAAGTGCCAGACCAGAAGATGTGTCTTTCCAGGGGCGGGGAGTCTTCGAG

CTCTCGGACGAAAAGGCAACGAACCCGATCGTGCCTTCCTTTGACATGAGTAATGAAGGA

TCTTATTTCTTCGGAGACAATGCAGAGGAGTATGACAATTAAAGAAAAATACCCTTGTTT

CTACT-------------------------------------------------

>A_chicken_Kurgan_1005_2020_EPI1814362

AGCAAAAGCAGGGTAGATAATCACTCACTGAGTGACATCAACATCATGGCGTCTCAAGGC

ACCAAACGATCTTATGAACAGATGGAAACTGGTGGGGAGCGCCAGAATGCCACTGAGATT

AGAGCATCGGTTGGAAGAATGGTTGGTGGAATTGGGAGGTTCTACATACAGATGTGCACT

GAGCTCAAACTCAGCGACTATGAAGGAAGACTGATCCAGAACAGCATAACAATAGAGAGA

ATGGTTCTCTCCGCATTTGATGAAAGGAGAAACAAATATCTGGAAGAACATCCCAGTGCG

GGGAAAGACCCGAAGAAAACAGGAGGTCCAATTTATCGAAGGAGAGATGGGAAATGGGTG

AGAGAGCTGATCCTGTATGACAAAGAGGAGATCAGGAGGATCTGGCGTCAAGCGAATAAT

GGAGAAGACGCAACTGCTGGTCTCACTCACCTGATGATCTGGCATTCCAATCTAAATGAT

GCCACATACCAGAGGACAAGAGCTCTCGTGCGTACCGGTATGGACCCCAGGATGTGCTCC

CTCATGCAAGGGTCAACTCTCCCAAGGAGGTCTGGAGCTGCTGGTGCAGCAGTGAAGGGA

GTTGGAACGATGGTGATGGAACTAATTCGGATGATAAAGCGAGGAGTTAATGATCGAAAC

TTCTGGAGAGGCGAGAATGGACGGAGGACAAGGATTGCATTTGAGAGAATGTGCAATATT

CTCAAAGGGAAATTCCAAACAGCAGCACAAAGAGCAATGATGGATCAAGTGCGTGAAAGC

AGGAATCCTGGCAATGCTGAAATTGAAGATCTCATTTTTCTGGCGCGGTCTGCTCTCATT

CTGAGAGGATCAGTGGCCCATAAGTCCTGCTTGCCCGCTTGTGTATACGGGCTCGCTGTA

GCCAGTGGATACGACTTTGAGAGAGAAGGGTACTCTCTAGTTGGAATAGACCCTTTCCGA

CTGCTTCAAAACAGCCAGGTCTTTAGTCTCATTAGACCAAATGAGAATCCAGCACACAAG

AGTCAATTGGTGTGGATGGCATGTCATTCTGCAGCATTCGAGGATCTGAGAGTCTCAAGT

TTCATCAGAGGAACAAGAGTAGTCCCAAGAGGACAACTATCCACTAGAGGAGTTCAAATT

GCTTCAAATGAGAACATAGAAACAATGGACTCCAGCACTCTTGAACTGAGAAGCAGATAT

TGGGCTATAAGAACCAGGAGTGGAGGAAACACCAACCAACAGAGAGCATCTGCAGGACAA

ATCAGTGTACAGCCCACTTTCTCGGTACAGAGAAACCTTCCCTTCGAAAGAGCGACCATT

ATGGCGGCATTCACAGGGAACACTGAAGGCAGAACGTCCGACATGAGGACTGAAATCATA

AGAATGATGGAAAGTGCCAGACCAGAAGATGTGTCTTTCCAGGGGCGGGGAGTCTTCGAG

CTCTCGGACGAAAAGGCAACGAACCCGATCGTGCCTTCCTTTGACATGAGTAATGAAGGA

TCTTATTTCTTCGGAGACAATGCAGAGGAGTATGACAATTAAAGAAAAATACCCTTGTTT

CTACT-------------------------------------------------

>A_turkey_Poland_464_2020_EPI1841311

-------------TAGATAATCACTCACTGAGTGACATCAACATCATGGCGTCTCAAGGC

ACCAAACGATCTTATGAACAGATGGAAACTAGTGGGGAGCGCCAGAATGCCACTGAGATT

AGAGCATCGGTTGGAAGAATGGTTGGTGGAATTGGGAGGTTCTACATACAGATGTGCACT

GAGCTCAAACTCAGCGACTATGAAGGAAGACTGATCCAGAACAGCATAACAATAGAGAGA

ATGGTTCTCTCCGCATTTGATGAAAGGAGAAACAAATATCTGGAAGAACATCCCAGTGCG

GGGAAAGACCCGAAGAAAACAGGAGGTCCAATTTATCGAAGGAGAGATGGGAAATGGGTG

AGAGAGCTGATCCTGTATGACAAAGAGGAGATCAGGAGGATCTGGCGTCAAGCGAATAAT

GGAGAAGACGCAACTGCTGGTCTCACTCACCTGATGATCTGGCATTCCAATCTAAATGAT

GCCACATACCAGAGGACAAGAGCTCTCGTGCGTACCGGTATGGACCCCAGGATGTGCTCC

CTCATGCAAGGGTCAACTCTCCCAAGGAGGTCTGGAGCTGCTGGTGCAGCAGTGAAGGGA

GTTGGAACGATGGTGATGGAACTAATTCGGATGATAAAGCGAGGAGTTAATGATCGAAAC

TTCTGGAGAGGCGAGAATGGACGGAGGACAAGGATTGCATTTGAGAGAATGTGCAATATT

CTCAAAGGGAAATTCCAAACAGCAGCACAAAGAGCAATGATGGATCAAGTGCGTGAAAGC

AGGAATCCTGGCAATGCTGAAATTGAAGATCTCATTTTTCTGGCGCGGTCTGCTCTCATT

CTGAGAGGATCAGTGGCCCATAAGTCCTGCTTGCCCGCTTGTGTATACGGGCTCGCTGTA

GCCAGTGGATACGACTTTGAAAGAGAAGGGTACTCTCTAGTTGGAATAGACCCTTTCCGA

TTGCTTCAAAACAGCCAGGTCTTTAGTCTCATTAGACCAAATGAGAATCCAGCACACAAG

AGCCAATTGGTGTGGATGGCATGTCATTCTGCAGCATTCGAAGATCTGAGAGTCTCAAGT

TTCATCAGAGGAACAAGAGTAGTCCCAAGAGGACAACTATCCACTAGAGGAGTTCAAATT

GCTTCAAATGAGAACATAGAAACAATGGACTCCAGCACTCTTGAACTGAGAAGCAGATAT

TGGGCTATAAGAACCAGGAGTGGAGGAAACACCAACCAACAGAGAGCATCTGCAGGACAA

ATCAGTGTACAGCCCACTTTCTCGGTACAGAGAAACCTTCCCTTCGAAAGAGCGACCATT

ATGGCGGCATTCACAGGGAACACTGAAGGCAGAACGTCCGACATGAGGACTGAAATCATA

AGAATGATGGAAAGTGCCAGACCAGAAGATGTGTCTTTCCAGGGGCGGGGAGTCTTCGAG

CTCTCGGACGAAAAGGCAACGAACCCGATCGTGCCTTCCTTTGACATGAGTAATGAAGGA

TCTTATTTCTTCGGAGACAATGCAGAGGAGTATGACAATTAAAGAAAAATAC--------

------------------------------------------------------

>A_duck_Northern_China_ZGL_2020_EPI1844091

---------------------------------------------ATGGCGTCTCAAGGC

ACCAAACGATCTTATGAACAGATGGAAACTAGTGGGGAGCGCCAGAATGCCACTGAGATT

AGAGCATCGGTTGGAAGAATGGTTGGTGGAATTGGGAGGTTCTACATACAGATGTGCACT

GAGCTCAAACTCAGCGACTATGAAGGAAGACTGATCCAGAACAGCATAACAATAGAGAGA

ATGGTTCTCTCCGCATTTGATGAAAGGAGAAACAAATATCTGGAAGAACATCCCAGTGCG

GGGAAAGACCCGAAGAAAACAGGAGGTCCAATTTATCGAAGGAGAGATGGGAAATGGGTG

AGAGAGCTGATCCTGTATGACAAAGAGGAGATCAGGAGGATCTGGCGTCAAGCGAACAAT

GGAGAAGACGCAACTGCTGGTCTCACTCACCTGATGATCTGGCATTCCAATCTAAATGAT

GCCACATACCAGAGGACAAGAGCTCTCGTGCGTACCGGTATGGACCCCAGGATGTGCTCC

CTCATGCAAGGGTCAACTCTCCCAAGGAGGTCTGGAGCTGCTGGTGCAGCAGTGAAGGGA

GTTGGAACGATGGTGATGGAACTAATTCGGATGATAAAGCGAGGAGTTAATGATCGAAAC

TTCTGGAGAGGCGAGAATGGACGGAGGACAAGGATTGCATTTGAGAGAATGTGCAATATT

CTCAAAGGGAAATTCCAAACAGCAGCACAAAGAGCAATGATGGATCAAGTGCGTGAAAGC

AGGAATCCTGGCAATGCTGAGATTGAAGATCTCATTTTTCTGGCGCGGTCTGCTCTCATT

CTGAGAGGATCAGTGGCCCATAAGTCCTGCTTGCCCGCTTGTGTATACGGGCTCGCTGTA

GCCAGTGGATACGACTTTGAGAGAGAAGGGTACTCTCTAGTTGGAATAGACCCTTTCCGA

CTGCTTCAAAACAGCCAGGTCTTTAGTCTCATTAGACCAAATGAGAATCCAGCACACAAG

AGTCAATTGGTGTGGATGGCATGTCATTCTGCAGCATTCGAGGATCTGAGAGTCTCAAGT

TTCATCAGAGGAACAAGAGTAGTCCCAAGAGGACAACTATCCACTAGAGGAGTTCAAATT

GCTTCAAATGAGAACATAGAAACAATGGACTCCAGCACTCTTGAACTGAGAAGCAGATAC

TGGGCTATAAGAACCAGGAGTGGAGGAAACACCAACCAACAGAGAGCATCTGCAGGACAA

ATCAGTGTACAGCCCACTTTCTCGGTACAGAGAAACCTTCCCTTCGAAAGAGCGACCATT

ATGGCGGCATTCACAGGGAACACTGAAGGCAGAACGTCCGACATGAGGACTGAAATCATA

AGAATGATGGAAAGTGCCAGACCAGAAGATGTGTCTTTCCAGGGGCGGGGAGTCTTCGAG

ATCTCGGACGAAAAGGCAACGAACCCGATCGTGCCTTCCTTTGACATGAGTAATGAAGGA

TCTTATTTCTTCGGAGACAATGCAGAGGAGTATGACAATTAA------------------

------------------------------------------------------

>A_duck_Southwestern_China_B1904_2020_EPI1844100

---------------------------------------------ATGGCGTCTCAAGGC

ACCAAACGATCTTATGAACAGATGGAAACTAGTGGGGAGCGCCAGAATGCCACTGAGATT

AGAGCATCGGTTGGAAGAATGGTTGGTGGAATCGGGAGGTTCTACATACAGATGTGCACT

GAGCTCAAACTCAGCGACTATGAAGGAAGACTGATCCAGAACAGCATAACAATAGAGAGA

ATGGTTCTCTCCGCATTTGATGAAAGGAGAAACAAATATCTGGAAGAACATCCCAGTGCG

GGGAAAGACCCGAAGAAAACAGGAGGTCCAATTTATCGGAGGAGAGATGGGAAATGGGTG

AGAGAGCTGATCCTGTATGACAAAGAGGAGATCAGGAGGATCTGGCGTCAAGCGAACAAT

GGAGAAGACGCAACTGCTGGTCTCACTCACCTGATGATCTGGCATTCCAATCTAAATGAT

GCCACATACCAGAGGACAAGAGCTCTCGTGCGTACCGGTATGGACCCCAGGATGTGCTCC

CTCATGCAAGGGTCAACTCTCCCAAGGAGGTCTGGAGCTGCTGGTGCAGCAGTGAAGGGA

GTTGGAACGATGGTGATGGAACTAATTCGGATGATAAAGCGAGGAGTTAATGATCGAAAC

TTCTGGAGAGGCGAGAATGGACGGAGGACAAGGATTGCATTTGAGAGAATGTGCAATATT

CTCAAAGGGAAATTCCAAACAGCAGCACAAAGAGCAATGATGGATCAAGTGCGTGAAAGC

AGGAATCCTGGCAATGCTGAAATTGAAGATCTCATTTTTCTGGCGCGGTCTGCTCTCATT

CTGAGAGGATCAGTGGCCCATAAGTCCTGCTTGCCCGCTTGTGTATACGGGCTCGCTGTC

GCCAGTGGATACGACTTTGAGAGAGAAGGGTACTCTCTAGTTGGAATAGACCCTTTCCGA

CTGCTTCAAAACAGCCAGGTCTTTAGTCTCATTAGACCAAATGAGAATCCAGCACACAAG

AGTCAATTGGTGTGGATGGCATGTCATTCTGCAGCATTCGAGGATCTGAGAGTCTCAAGT

TTCATCAGAGGAACAAGAGTAGTCCCAAGAGGACAACTATCCACTAGAGGAGTTCAAATT

GCTTCAAATGAGAACATAGAAACAATGGACTCCAGCACTCTTGAACTGAGAAGCAGATAC

TGGGCTATAAGAACCAGGAGTGGAGGAAACACCAACCAACAGAGAGCATCTGCAGGACAA

ATCAGTGTACAGCCCACTTTCTCGGTACAGAGAAACCTTCCCTTCGAAAGAGCGACCATT

ATGGCGGCATTCACAGGGAACACTGAAGGCAGAACGTCCGACATGAGGACTGAAATCATA

AGAATGATGGAAAGTGCCAGACCAGAAGATGTGTCTTTCCAGGGGCGGGGAGTCTTCGAG

ATCTCGGACGAAAAGGCAACGAACCCGATCGTGCCTTCCTTTGACATGAGTAATGAAGGA

TCTTATTTCTTCGGAGACAATGCAGAGGAGTATGACAATTAA------------------

------------------------------------------------------

>A_duck_Korea_H411_2020_EPI1845930

---------------------------------------------ATGGCGTCTCAAGGC

ACCAAACGATCTTATGAACAGATGGAAACTAGTGGGGAGCGCCAGAATGCCACTGAGATT

AGAGCATCGGTTGGAAGAATGGTTGGTGGAATTGGGAGGTTCTACATACAGATGTGCACT

GAGCTCAAACTCAGCGACTATGAAGGAAGACTGATCCAGAACAGCATAACAATAGAGAGA

ATGGTTCTCTCCGCATTTGATGAAAGGAGAAACAAATATCTGGAAGAACATCCCAGTGCG

GGGAAAGACCCGAAGAAAACAGGAGGTCCAATTTATCGAAGGAGAGATGGGAAATGGGTG

AGAGAGCTGATCCTGTATGACAAAGAGGAGATCAGGAGGATCTGGCGTCAAGCGAACAAT

GGAGAAGACGCAACTGCTGGTCTCACTCATCTGATGATCTGGCATTCCAATCTAAATGAT

GCCACATACCAGAGGACAAGAGCTCTCGTGCGTACCGGTATGGACCCCAGGATGTGCTCC

CTCATGCAAGGGTCAACTCTCCCAAGGAGGTCTGGAGCTGCTGGTGCAGCAGTGAAGGGA

GTTGGAACGATGGTGATGGAACTAATTCGGATGATAAAGCGAGGAGTTAATGATCGAAAC

TTCTGGAGAGGCGAGAATGGACGGAGGACAAGGATTGCATTTGAGAGAATGTGCAATATT

CTCAAAGGGAAATTCCAAACAGCAGCACAAAGAGCAATGATGGATCAAGTGCGTGAAAGC

AGGAATCCTGGCAATGCTGAAATTGAAGATCTCATTTTTCTGGCGCGGTCTGCTCTCATT

CTGAGAGGATCAGTGGCCCATAAGTCCTGCTTGCCCGCTTGTGTATACGGGCTCGCTGTA

GCCAGTGGATACGACTTTGAGAGAGAAGGGTACTCTCTAGTTGGAATAGACCCTTTCCGA

CTGCTTCAAAACAGCCAGGTCTTTAGTCTCATTAGACCAAATGAGAATCCAGCACACAAG

AGTCAATTGGTGTGGATGGCATGTCATTCTGCAGCATTCGAGGATCTGAGAGTCTCAAGT

TTCATCAGAGGAACAAGAGTAGTCCCAAGAGGACAACTATCCACTAGAGGAGTTCAAATT

GCTTCAAATGAGAACATAGAAACAATGGACTCCAGCACTCTTGAACTGAGAAGCAGATAC

TGGGCTATAAGAACCAGGAGTGGAGGAAACACCAACCAACAGAGAGCATCTGCAGGACAA

ATCAGTGTACAGCCCACTTTCTCGGTACAGAGAAACCTTCCCTTCGAAAGAGCGACCATT

ATGGCGGCATTCACAGGGAACACTGAAGGCAGAACGTCCGACATGAGGACTGAAATCATA

AGAATGATGGAAAGTGCCAGACCAGAAGATGTGTCTTTCCAGGGGCGGGGAGTCTTCGAG

ATCTCGGACGAAAAGGCAACGAACCCGATCGTGCCTTCCTTTGACATGAGTAATGAAGGA

TCTTATTTCTTCGGAGACAATGCAGAGGAGTATGACAATTAA------------------

------------------------------------------------------

>A_duck_Korea_H431_2020_EPI1845954

---------------------------------------------ATGGCGTCTCAAGGC

ACCAAACGATCTTATGAACAGATGGAAACTAGTGGGGAGCGCCAGAATGCCACTGAGATT

AGAGCATCGGTTGGAAGAATGGTTGGTGGAATTGGGAGGTTCTACATACAGATGTGCACT

GAGCTCAAACTCAGCGACTATGAAGGAAGACTGATCCAGAACAGCATAACAATAGAGAGA

ATGGTTCTCTCCGCATTTGATGAAAGGAGAAACAAATATCTGGAAGAACATCCCAGTGCG

GGGAAAGACCCGAAGAAAACAGGAGGTCCAATTTATCGAAGGAGAGATGGGAAATGGGTG

AGAGAGCTGATCCTGTATGACAAAGAGGAGATCAGGAGGATCTGGCGTCAAGCGAACAAT

GGAGAAGACGCAACTGCTGGTCTCACTCATCTGATGATCTGGCATTCCAATCTAAATGAT

GCCACATACCAGAGGACAAGAGCTCTCGTGCGTACCGGTATGGACCCCAGGATGTGCTCC

CTCATGCAAGGGTCAACTCTCCCAAGGAGGTCTGGAGCTGCTGGTGCAGCAGTGAAGGGA

GTTGGAACGATGGTGATGGAACTAATTCGGATGATAAAGCGAGGAGTTAATGATCGAAAC

TTCTGGAGAGGCGAGAATGGACGGAGGACAAGGATTGCATTTGAGAGAATGTGCAATATT

CTCAAAGGGAAATTCCAAACAGCAGCACAAAGAGCAATGATGGATCAAGTGCGTGAAAGC

AGGAATCCTGGCAATGCTGAAATTGAAGATCTCATTTTTCTGGCGCGGTCTGCTCTCATT

CTGAGAGGATCAGTGGCCCATAAGTCCTGCTTGCCCGCTTGTGTATACGGGCTCGCTGTA

GCCAGTGGATACGACTTTGAGAGAGAAGGGTACTCTCTAGTTGGAATAGACCCTTTCCGA

CTGCTTCAAAACAGCCAGGTCTTTAGTCTCATTAGACCAAATGAGAATCCAGCACACAAG

AGTCAATTGGTGTGGATGGCATGTCATTCTGCAGCATTCGAGGATCTGAGAGTCTCAAGT

TTCATCAGAGGAACAAGAGTAGTCCCAAGAGGACAACTATCCACTAGAGGAGTTCAAATT

GCTTCAAATGAGAACATAGAAACAATGGACTCCAGCACTCTTGAACTGAGAAGCAGATAC

TGGGCTATAAGAACCAGGAGTGGAGGAAACACCAACCAACAGAGAGCATCTGCAGGACAA

ATCAGTGTACAGCCCACTTTCTCGGTACAGAGAAACCTTCCCTTCGAAAGAGCGACCATT

ATGGCGGCATTCACAGGGAACACTGAAGGCAGAACGTCCGACATGAGGACTGAAATCATA

AGAATGATGGAAAGTGCCAGACCAGAAGATGTGTCTTTCCAGGGGCGGGGAGTCTTCGAG

ATCTCGGACGAAAAGGCAACGAACCCGATCGTGCCTTCCTTTGACATGAGTAATGAAGGA

TCTTATTTCTTCGGAGACAATGCAGAGGAGTATGACAATTAA------------------

------------------------------------------------------

>A_duck_Korea_H471_2020_EPI1846026

---------------------------------------------ATGGCGTCTCAAGGC

ACCAAACGATCTTATGAACAGATGGAAACTAGTGGGGAGCGCCAGAATGCCACTGAGATT

AGAGCATCGGTTGGAAGAATGGTTGGTGGAATTGGGAGGTTCTACATACAGATGTGCACT

GAGCTCAAACTCAGCGACTATGAAGGAAGACTGATCCAGAACAGCATAACAATAGAGAGA

ATGGTTCTCTCCGCATTTGATGAAAGGAGAAACAAATATCTGGAAGAACATCCCAGTGCG

GGGAAAGACCCGAAGAAAACAGGAGGTCCAATTTATCGAAGGAGAGATGGGAAATGGGTG

AGAGAGCTGATCCTGTATGACAAAGAGGAGATCAGGAGGATCTGGCGTCAAGCGAACAAT

GGAGAAGACGCAACTGCTGGTCTCACTCATCTGATGATCTGGCATTCCAATCTAAATGAT

GCCACATACCAGAGGACAAGAGCTCTCGTGCGTACCGGTATGGACCCCAGGATGTGCTCC

CTCATGCAAGGGTCAACTCTCCCAAGGAGGTCTGGAGCTGCTGGTGCAGCAGTGAAGGGA

GTTGGAACGATGGTGATGGAACTAATTCGGATGATAAAGCGAGGAGTTAATGATCGAAAC

TTCTGGAGAGGCGAGAATGGGCGGAGGACAAGGATTGCATTTGAGAGAATGTGCAATATT

CTCAAAGGGAAATTCCAAACAGCAGCACAAAGAGCAATGATGGATCAAGTGCGTGAAAGC

AGGAATCCTGGCAATGCTGAAATTGAAGATCTCATTTTTCTGGCGCGGTCTGCTCTCATT

CTGAGAGGATCAGTGGCCCATAAGTCCTGCTTGCCCGCTTGTGTATACGGGCTCGCTGTA

GCCAGTGGATACGACTTTGAGAGAGAAGGGTACTCTCTAGTTGGAATAGACCCTTTCCGA

CTGCTTCAAAACAGCCAGGTCTTTAGTCTCATTAGACCAAATGAGAATCCAGCACACAAG

AGTCAATTGGTGTGGATGGCATGTCATTCTGCAGCATTCGAGGATCTGAGAGTCTCAAGT

TTCATCAGAGGAACAAGAGTAGTCCCAAGAGGACAACTATCCACTAGAGGAGTTCAAATT

GCTTCAAATGAGAACATAGAAACAATGGACTCCAGCACTCTTGAACTGAGAAGCAGATAC

TGGGCTATAAGAACCAGGAGTGGAGGAAACACAAACCAACAGAGAGCATCTGCAGGACAA

ATCAGTGTACAGCCCACTTTCTCGGTACAGAGAAACCTTCCCTTCGAAAGAGCGACCATT

ATGGCGGCATTCACAGGGAACACTGAAGGCAGAACGTCCGACATGAGGACTGAAATCATA

AGAATGATGGAAAGTGCCAGACCAGAAGATGTGTCTTTCCAGGGGCGGGGAGTCTTCGAG

ATCTCGGACGAAAAGGCAACGAACCCGATCGTGCCTTCCTTTGACATGAGTAATGAAGGA

TCTTATTTCTTCGGAGACAATGCAGAGGAGTATGACAATTAA------------------

------------------------------------------------------

>A_chicken_Korea_H510_2020_EPI1846050

---------------------------------------------ATGGCGTCTCAAGGC

ACCAAACGATCTTATGAACAGATGGAAACTAGTGGGGAGCGCCAGAATGCCACTGAGATT

AGAGCATCGGTTGGAAGAATGGTTGGTGGAATTGGGAGGTTCTACATACAGATGTGCACT

GAGCTCAAACTCAGCGACTATGAAGGAAGACTGATCCAGAACAGCATAACAATAGAGAGA

ATGGTTCTCTCCGCATTTGATGAAAGGAGAAACAAATATCTGGAAGAACATCCCAGTGCG

GGGAAAGACCCGAAGAAAACAGGAGGTCCAATTTATCGAAGGAGAGATGGGAAATGGGTG

AGAGAGCTGATCCTGTATGACAAAGAGGAGATCAGGAGGATCTGGCGTCAAGCGAACAAT

GGAGAAGACGCAACTGCTGGTCTCACTCATCTGATGATCTGGCATTCCAATCTAAATGAT

GCCACATACCAGAGGACAAGAGCTCTCGTGCGTACCGGTATGGACCCCAGGATGTGCTCC

CTCATGCAAGGGTCAACTCTCCCAAGGAGGTCTGGAGCTGCTGGTGCAGCAGTGAAGGGA

GTTGGAACGATGGTGATGGAACTAATTCGGATGATAAAGCGAGGAGTTAATGATCGAAAC

TTCTGGAGAGGCGAGAATGGACGGAGGACAAGGATTGCATTTGAGAGAATGTGCAATATT

CTCAAAGGGAAATTCCAAACAGCAGCACAAAGAGCAATGATGGATCAAGTGCGTGAAAGC

AGGAATCCTGGCAATGCTGAAATTGAAGATCTCATTTTTCTGGCGCGGTCTGCTCTCATT

CTGAGAGGATCAGTGGCCCATAAGTCCTGCTTGCCCGCTTGTGTATACGGGCTCGCTGTA

GCCAGTGGATACGACTTTGAGAGAGAAGGGTACTCTCTAGTTGGAATAGACCCTTTCCGA

CTGCTTCAAAACAGCCAGGTCTTTAGTCTCATTAGACCAAATGAGAATCCAGCACACAAG

AGTCAATTGGTGTGGATGGCATGTCATTCTGCAGCATTCGAGGATCTGAGAGTCTCAAGT

TTCATCAGAGGAACAAGAGTAGTCCCAAGAGGACAACTATCCACTAGAGGAGTTCAAATT

GCTTCAAATGAGAACATAGAAACAATGGACTCCAGCACTCTTGAACTGAGAAGCAGATAC

TGGGCTATAAGAACCAGGAGTGGAGGAAACACCAACCAACAGAGAGCATCTGCAGGACAA

ATCAGTGTACAGCCCACTTTCTCGGTACAGAGAAACCTTCCCTTCGAAAGAGCGACCATT

ATGGCGGCATTCACAGGGAACACTGAAGGCAGAACGTCCGACATGAGGACTGAAATCATA

AGAATGATGGAAAGTGCCAGACCAGAAGATGTGTCTTTCCAGGGGCGGGGAGTCTTCGAG

ATCTCGGACGAAAAGGCAACGAACCCGATCGTGCCTTCCTTTGACATGAGTAATGAAGGA

TCTTATTTCTTCGGAGACAATGCAGAGGAGTATGACAATTAA------------------

------------------------------------------------------

>A_duck_Korea_H538_2020_EPI1846146

---------------------------------------------ATGGCGTCTCAAGGC

ACCAAACGATCTTATGAACAGATGGAAACTAGTGGGGAGCGCCAGAATGCCACTGAGATT

AGAGCATCGGTTGGAAGAATGGTTGGTGGAATTGGGAGGTTCTACATACAGATGTGCACT

GAGCTCAAACTCAGCGACTATGAAGGAAGACTGATCCAGAACAGCATAACAATAGAGAGA

ATGGTTCTCTCCGCATTTGATGAAAGGAGAAACAAATATCTGGAAGAACATCCCAGTGCG

GGGAAAGACCCGAAGAAAACAGGAGGTCCAATTTATCGAAGGAGAGATGGGAAATGGGTG

AGAGAGCTGATCCTGTATGACAAAGAGGAGATCAGGAGGATCTGGCGTCAAGCGAACAAT

GGAGAAGACGCAACTGCTGGTCTCACTCATCTGATGATCTGGCATTCCAATCTAAATGAT

GCCACATACCAGAGGACAAGAGCTCTCGTGCGTACCGGTATGGACCCCAGGATGTGCTCC

CTCATGCAAGGGTCAACTCTCCCAAGGAGGTCTGGAGCTGCTGGTGCAGCAGTGAAGGGA

GTTGGAACGATGGTGATGGAACTAATTCGGATGATAAAGCGAGGAGTTAATGATCGAAAC

TTCTGGAGAGGCGAGAATGGACGGAGGACAAGGATTGCATTTGAGAGAATGTGCAATATT

CTCAAAGGGAAATTCCAAACAGCAGCACAAAGAGCAATGATGGATCAAGTGCGTGAAAGC

AGGAATCCTGGCAATGCTGAAATTGAAGATCTCATTTTTCTGGCGCGGTCTGCTCTCATT

CTGAGAGGATCAGTGGCCCATAAGTCCTGCTTGCCCGCTTGTGTATACGGGCTCGCTGTA

GCCAGTGGATACGACTTTGAGAGAGAAGGGTACTCTCTAGTTGGAATAGACCCTTTCCGA

CTGCTTCAAAACAGCCAGGTCTTTAGTCTCATTAGACCAAATGAGAATCCAGCACACAAG

AGTCAATTGGTGTGGATGGCATGTCATTCTGCAGCATTCGAGGATCTGAGAGTCTCAAGT

TTCATCAGAGGAACAAGAGTAGTCCCAAGAGGACAACTATCCACTAGAGGAGTTCAAATT

GCTTCAAATGAGAACATAGAAACAATGGACTCCAGCACTCTTGAACTGAGAAGCAGATAC

TGGGCTATAAGAACCAGGAGTGGAGGAAACACCAACCAACAGAGAGCATCTGCAGGACAA

ATCAGTGTACAGCCCACTTTCTCGGTACAGAGAAACCTTCCCTTCGAAAGAGCGACCATT

ATGGCGGCATTCACAGGGAACACTGAAGGCAGAACGTCCGACATGAGGACTGAAATCATA

AGAATGATGGAAAGTGCCAGACCAGAAGATGTGTCTTTCCAGGGGCGGGGAGTCTTCGAG

ATCTCGGACGAAAAGGCAACGAACCCGATCGTGCCTTCCTTTGACATGAGTAATGAAGGA

TCTTATTTCTTCGGAGACAATGCAGAGGAGTATGACAATTAA------------------

------------------------------------------------------

>A_chicken_Tyumen_302-01_2020_EPI1848599

AGCAAAAGCAGGGTAGATAATCACTCACTGAGTGACATCAACATCATGGCGTCTCAAGGC

ACCAAACGATCTTATGAACAGATGGAAACTGGTGGGGAGCGCCAGAATGCCACTGAGATT

AGAGCATCGGTTGGAAGAATGGTTGGTGGAATTGGGAGGTTCTACATACAGATGTGCACT

GAGCTCAAACTCAGCGACTATGAAGGAAGACTGATCCAGAACAGCATAACAATAGAGAGA

ATGGTTCTCTCCGCATTTGATGAAAGGAGAAACAAATATCTGGAAGAACATCCCAGTGCG

GGGAAAGACCCGAAGAAAACAGGAGGTCCAATTTATCGAAGGAGAGATGGGAAATGGGTG

AGAGAGCTGATCCTGTATGACAAAGAGGAGATCAGGAGGATCTGGCGTCAAGCGAATAAT

GGAGAAGACGCAACTGCTGGTCTCACTCACCTGATGATCTGGCATTCCAATCTAAATGAT

GCCACATACCAGAGGACAAGAGCTCTCGTGCGTACCGGTATGGACCCCAGGATGTGCTCC

CTCATGCAAGGGTCAACTCTCCCAAGGAGGTCTGGAGCTGCTGGTGCAGCAGTGAAGGGA

GTTGGAACGATGGTGATGGAACTAATTCGGATGATAAAGCGAGGAGTTAATGATCGAAAC

TTCTGGAGAGGCGAGAATGGACGGAGGACAAGGATTGCATTTGAGAGAATGTGCAATATT

CTCAAAGGGAAATTCCAAACAGCAGCACAAAGAGCAATGATGGATCAAGTGCGTGAAAGC

AGGAATCCTGGCAATGCTGAAATTGAAGATCTCATTTTTCTGGCGCGGTCTGCTCTCATT

CTGAGAGGATCAGTGGCCCATAAGTCCTGCTTGCCCGCTTGTGTATACGGGCTCGCTGTA

GCCAGTGGATACGACTTTGAGAGAGAAGGGTACTCTCTAGTTGGAATAGACCCTTTCCGA

CTGCTTCAAAACAGCCAGGTCTTTAGTCTCATTAGACCAAATGAGAATCCAGCACACAAG

AGTCAATTGGTGTGGATGGCATGTCATTCTGCAGCATTCGAGGATCTGAGAGTCTCAAGT

TTCATCAGAGGAACAAGAGTAGTCCCAAGAGGACAACTATCCACTAGAGGAGTTCAAATT

GCTTCAAATGAGAACATAGAAACAATGGACTCCAGCACTCTTGAACTGAGAAGCAGATAT

TGGGCTATAAGAACCAGGAGTGGAGGAAACACCAACCAACAGAGAGCATCTGCAGGACAA

ATCAGTGTACAGCCCACTTTCTCGGTACAGAGAAACCTTCCCTTCGAAAGAGCGACCATT

ATGGCGGCATTCACAGGGAACACTGAAGGCAGAACGTCCGACATGAGGACTGAAATCATA

AGAATGATGGAAAGTGCCAGACCAGAAGATGTGTCTTTCCAGGGGCGGGGAGTCTTCGAG

CTCTCGGACGAAAAGGCAACGAACCCGATCGTGCCTTCCTTTGACATGAGTAATGAAGGA

TCTTATTTCTTCGGAGACAATGCAGAGGAGTATGACAATTAAAGAAAAATACCCTTGTTT

CTACT-------------------------------------------------

>A_chicken_Tyumen_302-02_2020_EPI1848607

AGCAAAAGCAGGGTAGATAATCACTCACTGAGTGACATCAACATCATGGCGTCTCAAGGC

ACCAAACGATCTTATGAACAGATGGAAACTGGTGGGGAGCGCCAGAATGCCACTGAGATT

AGAGCATCGGTTGGAAGAATGGTTGGTGGAATTGGGAGGTTCTACATACAGATGTGCACT

GAGCTCAAACTCAGCGACTATGAAGGAAGACTGATCCAGAACAGCATAACAATAGAGAGA

ATGGTTCTCTCCGCATTTGATGAAAGGAGAAACAAATATCTGGAAGAACATCCCAGTGCG

GGGAAAGACCCGAAGAAAACAGGAGGTCCAATTTATCGAAGGAGAGATGGGAAATGGGTG

AGAGAGCTGATCCTGTATGACAAAGAGGAGATCAGGAGGATCTGGCGTCAAGCGAATAAT

GGAGAAGACGCAACTGCTGGTCTCACTCACCTGATGATCTGGCATTCCAATCTAAATGAT

GCCACATACCAGAGGACAAGAGCTCTCGTGCGTACCGGTATGGACCCCAGGATGTGCTCC

CTCATGCAAGGGTCAACTCTCCCAAGGAGGTCTGGAGCTGCTGGTGCAGCAGTGAAGGGA

GTTGGAACGATGGTGATGGAACTAATTCGGATGATAAAGCGAGGAGTTAATGATCGAAAC

TTCTGGAGAGGCGAGAATGGACGGAGGACAAGGATTGCATTTGAGAGAATGTGCAATATT

CTCAAAGGGAAATTCCAAACAGCAGCACAAAGAGCAATGATGGATCAAGTGCGTGAAAGC

AGGAATCCTGGCAATGCTGAAATTGAAGATCTCATTTTTCTGGCGCGGTCTGCTCTCATT

CTGAGAGGATCAGTGGCCCATAAGTCCTGCTTGCCCGCTTGTGTATACGGGCTCGCTGTA

GCCAGTGGATACGACTTTGAGAGAGAAGGGTACTCTCTAGTTGGAATAGACCCTTTCCGA

CTGCTTCAAAACAGCCAGGTCTTTAGTCTCATTAGACCAAATGAGAATCCAGCACACAAG

AGTCAATTGGTGTGGATGGCATGTCATTCTGCAGCATTCGAGGATCTGAGAGTCTCAAGT

TTCATCAGAGGAACAAGAGTAGTCCCAAGAGGACAACTATCCACTAGAGGAGTTCAAATT

GCTTCAAATGAGAACATAGAAACAATGGACTCCAGCACTCTTGAACTGAGAAGCAGATAT

TGGGCTATAAGAACCAGGAGTGGAGGAAACACCAACCAACAGAGAGCATCTGCAGGACAA

ATCAGTGTACAGCCCACTTTCTCGGTACAGAGAAACCTTCCCTTCGAAAGAGCGACCATT

ATGGCGGCATTCACAGGGAACACTGAAGGCAGAACGTCCGACATGAGGACTGAAATCATA

AGAATGATGGAAAGTGCCAGACCAGAAGATGTGTCTTTCCAGGGGCGGGGAGTCTTCGAG

CTCTCGGACGAAAAGGCAACGAACCCGATCGTGCCTTCCTTTGACATGAGTAATGAAGGA

TCTTATTTCTTCGGAGACAATGCAGAGGAGTATGACAATTAAAGAAAAATACCCTTGTTT

CTACT-------------------------------------------------

>A_chicken_Poland_474_2020_EPI1850193

-------------TAGATAATCACTCACTGAGTGACATCAACATCATGGCGTCTCAAGGC

ACCAAACGATCTTATGAACAGATGGAAACTGGTGGGGAGCGCCAGAATGCCACTGAGATT

AGAGCATCGGTTGGAAGAATGGTTGGTGGAATTGGGAGGTTCTACATACAGATGTGCACT

GAGCTCAAACTCAGCGACTATGAAGGAAGACTGATCCAGAACAGCATAACAATAGAGAGA

ATGGTTCTCTCCGCATTTGATGAAAGGAGAAACAAATATCTGGAAGAACATCCCAGTGCG

GGGAAAGACCCGAAGAAAACAGGAGGTCCAATTTATCGAAGGAGAGATGGGAAATGGGTG

AGAGAGCTGATCCTGTATGACAAAGAGGAGATCAGGAGGATCTGGCGTCAAGCGAATAAT

GGAGAAGACGCAACTGCTGGTCTCACTCACCTAATGATCTGGCATTCCAATCTAAATGAT

GCCACATACCAGAGGACAAGAGCTCTCGTGCGTACCGGTATGGACCCCAGGATGTGCTCC

CTCATGCAAGGGTCAACTCTCCCAAGGAGGTCTGGAGCTGCTGGTGCAGCAGTGAAGGGA

GTTGGAACGATGGTGATGGAACTAATTCGGATGATAAAGCGAGGAGTTAATGATCGAAAC

TTCTGGAGAGGCGAGAATGGACGGAGGACAAGGATTGCATTTGAGAGAATGTGCAATATT

CTCAAAGGGAAATTCCAAACAGCAGCACAAAGAGCAATGATGGATCAAGTGCGTGAAAGC

AGGAATCCTGGCAATGCTGAAATTGAAGATCTCATTTTTCTGGCGCGGTCTGCTCTCATT

CTGAGAGGATCAGTGGCCCATAAGTCCTGCTTGCCCGCTTGTGTATACGGGCTCGCTGTA

GTCAGTGGATACGACTTTGAGAGAGAAGGGTACTCTCTAGTTGGAATAGACCCTTTCCGA

CTGCTTCAAAACAGCCAGGTCTTTAGTCTCATTAGACCAAATGAGAATCCAGCACACAAG

AGTCAATTGGTGTGGATGGCATGTCATTCTGCAGCATTCGAGGATCTGAGAGTCTCAAGT

TTCATCAGAGGAACAAGAGTAGTCCCAAGAGGACAACTATCCACTAGAGGAGTTCAAATT

GCTTCAAATGAGAACATAGAAACAATGGACTCCAGCACTCTTGAACTAAGAAGCAGATAT

TGGGCTATAAGAACCAGGAGTGGAGGAAACACCAACCAACAGAGAGCATCTGCAGGACAA

ATCAGTGTACAGCCCACTTTCTCGGTACAGAGAAACCTTCCCTTCGAAAGAGCGACCATT

ATGGCGGCATTCACAGGGAACACTGAAGGCAGAACGTCCGACATGAGGACTGAAATCATA

AGAATGATGGAAAGTGCCAGACCAGAAGATGTGTCTTTCCAGGGGCGGGGAGTCTTCGAG

CTCTCGGACGAAAAGGCAACGAACCCGATCGTGCCTTCCTTTGACATGAGTAATGAAGGA

TCTTATTTCTTCGGAGACAATGCAGAGGAGTATGACAATTAAAGAAAAATAC--------

------------------------------------------------------

>A_swan_Poland_MB141_2020_EPI1850210

-------------TAGATAATCACTCACTGAGTGACATCAACATCATGGCGTCTCAAGGC

ACCAAACGATCTTATGAACAGATGGAAACTGGTGGGGAGCGCCAAAATGCCACTGAGATT

AGAGCATCGGTTGGAAGAATGGTTGGTGGAATTGGGAGGTTCTACATACAGATGTGCACT

GAGCTCAAACTCAGCGACTATGAAGGAAGACTGATCCAGAACAGCATAACAATAGAGAGA

ATGGTTCTCTCCGCATTTGATGAAAGGAGAAACAAATATCTGGAAGAACATCCCAGTGCG

GGGAAAGACCCGAAGAAAACAGGAGGTCCAATTTATCGAAGGAGAGATGGGAAATGGGTG

AGAGAGCTGATCCTGTATGACAAAGAGGAGATCAGGAGGATCTGGCGTCAAGCGAATAAT

GGAGAAGACGCAACTGCTGGTCTCACTCACCTAATGATCTGGCATTCCAATCTAAATGAT

GCCACATACCAGAGGACAAGAGCTCTCGTGCGTACCGGTATGGACCCCAGGATGTGCTCC

CTCATGCAAGGGTCAACTCTCCCAAGGAGGTCTGGAGCTGCTGGTGCAGCAGTGAAGGGA

GTTGGAACGATGGTGATGGAACTAATTCGGATGATAAAGCGAGGAGTTAATGATCGAAAC

TTCTGGAGAGGCGAGAATGGACGGAGGACAAGGATTGCATTTGAGAGAATGTGCAATATT

CTCAAAGGGAAATTCCAAACAGCAGCACAAAGAGCAATGATGGATCAAGTGCGTGAAAGC

AGGAATCCTGGCAATGCTGAAATTGAAGATCTCATTTTTCTGGCGCGGTCTGCTCTCATT

CTGAGAGGATCAGTGGCCCATAAGTCCTGCTTGCCCGCTTGTGTATACGGGCTCGCTGTA

GTCAGTGGATACGACTTTGAGAGAGAAGGGTACTCTCTAGTTGGAATAGACCCTTTCCGA

CTGCTTCAAAACAGCCAGGTCTTTAGTCTCATTAGACCAAATGAGAATCCAGCACACAAG

AGTCAATTGGTGTGGATGGCATGTCATTCTGCAGCATTCGAGGATCTGAGAGTCTCAAGT

TTCATCAGAGGAACAAGAGTAGTCCCAAGAGGACAACTATCCACTAGAGGAGTTCAAATT

GCTTCAAATGAGAACATAGAAACAATGGACTCCAGCACTCTTGAACTAAGAAGCAGATAT

TGGGCTATAAGAACCAGGAGTGGAGGAAACACCAACCAACAGAGAGCATCTGCAGGACAA

ATCAGTGTACAGCCCACTTTCTCGGTACAGAGAAACCTTCCCTTCGAAAGAGCGACCATT

ATGGCGGCATTCACAGGGAACACTGAAGGCAGAACGTCCGACATGAGGACTGAAATCATA

AGAATGATGGAAAGTGCCAGACCAGAAGATGTGTCTTTCCAGGGGCGGGGAGTCTTCGAG

CTCTCGGACGAAAAGGCAACGAACCCGATCGTGCCTTCCTTTGACATGAGTAATGAAGGA

TCTTATTTCTTCGGAGACAATGCAGAGGAGTATGACAATTAAAGAAAAATAC--------

------------------------------------------------------

>A_muscovy_duck_Slovakia_Pah1_21VIR1086-1_2021_EPI1858237

---------------------------------------------ATGGCGTCTCAAGGC

ACCAAACGATCTTATGAACAGATGGAAACTGGTGGGGAGCGCCAGAATGCCACTGAGATT

AGAGCATCRGTTGGAAGAATGGTTGGTGGAATTGGGAGGTTCTACATACAGATGTGCACT

GAGCTCAAACTCAGCAACTATGAAGGAAGACTGATCCAGAACAGCATAACAATAGAGAGA

ATGGTTCTCTCCGCATTTGATGAAAGGAGAAACAAATATCTGGAAGAACATCCCAGTGCG

GGGAAAGACCCGAAGAAAACAGGAGGTCCAATTTATCGAAGGAGAGATGGGAAATGGGTG

AGAGAGCTGATCCTGTATGACAAAGAGGAAATCAGGAGGATCTGGCGTCAAGCGAATAAT

GGAGAAGACGCAACTGCTGGTCTCACTCACCTGATGATCTGGCATTCCAATCTAAATGAT

GCCACATACCAGAGGACAAGAGCTCTCGTGCGTACCGGTATGGACCCCAGGATGTGCTCC

CTCATGCAAGGGTCAACTCTCCCAAGGAGGTCTGGAGCTGCTGGTGCAGCAGTGAAGGGA

GTTGGAACGATGGTGATGGAACTAATTCGGATGATAAAGCGAGGAGTTAATGATCGAAAC

TTCTGGAGAGGCGAGAATGGACGGAGGACAAGGATTGCATTTGAGAGAATGTGCAATATT

CTCAAAGGGAAATTCCAAACAGCAGCACAAAGAGCAATGATGGATCAAGTGCGTGAAAGC

AGGAATCCTGGCAATGCTGAAATTGAAGATCTCATTTTTCTGGCGCGGTCTGCTCTCATT

CTGAGAGGATCAGTGGCCCATAAGTCCTGCTTGCCCGCTTGTGTATACGGGCTCGCTGTA

GCCAGTGGATACGACTTTGAGAGAGAAGGGTACTCTCTAGTTGGAATAGACCCTTTCCGA

CTGCTTCAAAACAGCCAGGTCTTTAGTCTCATTAGACCAAATGAGAATCCAGCACACAAG

AGTCAATTGGTGTGGATGGCATGTCATTCTGCAGCATTCGAGGATCTGAGAGTCTCAAGT

TTCATCAGAGGAACAAGAGTAGTCCCAAGAGGACAACTATCCACTAGAGGAGTTCAAATT

GCTTCAAATGAGAACATGGAAACAATGGACTCCAGCACTCTTGAACTGAGAAGCAGATAT

TGGGCTATAAGAACCAGGAGTGGAGGAAACACCAACCAACAGAGAGCATCTGCAGGACAA

ATCAGTGTACAGCCCACTTTCTCAGTACAGAGAAACCTTCCCTTCGAAAGAGCGACCATT

ATGGCGGCATTCACAGGGAACACTGAAGGCAGAACGTCTGACATGAGGACTGAAATCATA

AGAATGATGGAAAGTGCCAGACCAGAAGATGTGTCTTTCCAGGGGCGGGGAGTCTTCGAG

CTCTCGGACGAAAAGGCAACGAACCCGATCGTGCCTTCCTTTGACATGAGTAATGAAGGG

TCTTATTTCTTCGGAGACAATGCAGAGGAGTATGACAATTAA------------------

------------------------------------------------------

>A_mute_swan_Slovenia_1639-20_21VIR959-1_2020_EPI1858293

---------------------------------------------ATGGCGTCTCAAGGC

ACCAAACGATCTTATGAACAGATGGAAACTAGTGGGGAGCGCCAGAATGCCACTGAGATT

AGAGCATCGGTTGGAAGAATGGTTGGTGGAATTGGGAGGTTCTACATACAGATGTGCACT

GAGCTCAAACTCAGCGACTATGAAGGAAGACTGATCCAGAACAGCATAACAATAGAGAGA

ATGGTTCTCTCCGCATTTGATGAAAGGAGAAACAAATATCTGGAAGAACATCCCAGTGCG

GGGAAAGACCCGAAGAAAACAGGAGGTCCAATTTATCGAAGGAGAGATGGGAAATGGGTG

AGAGAGCTGATCCTGTATGATAAAGAGGAGATCAGGAGGATCTGGCGTCAAGCGAATAAT

GGAGAAGACGCAACTGCTGGTCTCACTCACCTGATGATCTGGCATTCCAATCTAAATGAT

GCCACATACCAGAGGACAAGAGCTCTCGTGCGTACCGGTATGGACCCCAGGATGTGCTCC

CTCATGCAAGGGTCAACTCTCCCAAGGAGGTCTGGAGCTGCTGGTGCAGCAGTGAAGGGA

GTTGGAACGATGGTGATGGAACTAATTCGGATGATAAAGCGAGGAGTTAATGATCGAAAC

TTCTGGAGAGGCGAGAATGGACGGAGGACAAGGATTGCATTTGAGAGAATGTGCAATATT

CTCAAAGGGAAATTCCAAACAGCAGCACAAAGAGCAATGATGGATCAAGTGCGTGAAAGC

AGGAATCCTGGCAATGCTGAAATTGAAGACCTCATTTTTCTGGCGCGGTCTGCTCTCATT

CTGAGAGGATCAGTGGCCCATAAGTCCTGCTTGCCCGCTTGTGTATACGGGCTCGCTGTA

GTCAGTGGATACGACTTTGAGAGAGAAGGGTACTCTCTAGTTGGAATAGACCCTTTCCGA

CTGCTTCAAAACAGCCAGGTCTTTAGTCTCATTAGACCAAATGAGAATCCAGCACACAAG

AGTCAATTGGTGTGGATGGCATGTCATTCTGCAGCATTCGAGGATCTGAGAGTCTCAAGT

TTCATCAGAGGAACAAGAGTAGTCCCAAGAGGACAACTATCCACTAGAGGAGTTCAAATT

GCTTCAAATGAGAACATAGAAACAATGGACTCCAGCACTCTTGAACTGAGAAGCAGATAT

TGGGCTATAAGAACCAGGAGTGGAGGAAACACCAACCAACAGAGAGCATCTGCAGGACAA

ATCAGTGTACAGCCCACTTTCTCGGTACAGAGAAACCTTCCCTTCGAAAGAGCGACCATT

ATGGCGGCATTCACAGGGAACACTGAAGGCAGAACGTCTGACATGAGGACTGAAATCATA

AGAATGATGGAAAGTGCCAGACCAGAAGATGTGTCTTTCCAGGGGCGGGGAGTCTTCGAG

CTCTCGGACGAAAAGGCAACGAACCCGATCGTGCCTTCCTTTGACATGAGTAATGAAGGA

TCTTATTTCTTCGGAGACAATGCAGAGGAGTATGACA-----------------------

------------------------------------------------------

>A_duck_Bangladesh_43127_2020_EPI1902991

------------GTAGATAATCACTCACCGAGTGACATCAACATCATGGCGTCTCAAGGC

ACCAAACGATCCTATGAACAGATGGAAACTGGTGGAGAGCGCCAGAATGCTACTGAGATC

AGGGCCTCTGTTGGAAGAATGGTTGGTGGCATTGGGAGGTTCTACATACAGATGTGCACA

GAACTCAAACTCAGCGACCATGAGGGGAGACTTATCCAGAACAGCATAACAATAGAGAGA

ATGGTACTTTCTGCATTTGACGAAAGAAGGAACAGGTATTTGGAAGAGCACCCCAGTGCA

GGGAAGGACCCTAAGAAAACTGGAGGTCCAATCTATCGGAGGAGAGACGGGAAATGGATT

AGAGAGCTGATTTTGTACGACAAAGAAGAAATCAGGAGGATTTGGCGCCAAGCGAACAAC

GGAGAGGACGCAACTGCTGGTCTTACCCACCTGATGATATGGCACTCCAATCTGAATGAT

GCCACATATCAGAGAACAAGAGCTCTCGTGCGTACCGGAATGGACCCCAGGATGTGCTCC

CTAATGCAGGGATCAACTCTCCCGAGAAGATCTGGAGCTGCTGGTGCAGCAGTGAAGGGG

GTAGGAACAATGGTGATGGAGCTGATCCGAATGATAAAACGAGGGATTAACGACCGGAAT

TTCTGGAGAGGCGAAAATGGGCGGAGAACAAGGATTGCATATGAGAGAATGTGCAACATC

CTCAAAGGGAAATTCCAAACAGCTGCACAAAGAGCAATGATGGATCAAGTGCGAGAGAGC

AGAAATCCTGGGAATGCTGAAATTGAGGATCTTATTTTTCTGGCACGGTCTGCACTCATC

CTGAGAGGATCAGTGGCCCATAAGTCCTGCTTGCCTGCTTGTGTGTACGGACTTGCAGTG

GCCAGTGGGTATGATTTCGAGAGAGAAGGATACTCTCTGGTTGGGATAGATCCTTTCCGT

CTGCTTCAAAACAGCCAGGTCTTTAGTCTCATTAGGCCAAATGAAAACCCAGCACATAAG

AGTCAGTTAGTGTGGATGGCATGCCACTCTGCAGCATTTGAGGACCTCAGAGTATCAAGT

TTCATCAGAGGGACAAGAGTTGTCCCAAGAGGGCAGTTATCCACTAGAGGGGTTCAAATT

GCTTCAAATGAGAACATGGAAACAATGGACTCCAACACACTTGAGCTGAGAAGTAGGTAT

TGGGCCATAAGAACCAGGAGCGGAGGGAATACTAACCAGCAGAGGGCGTCTGCAGGGCAA

ATTAGTGTTCAACCCACTTTCTCAGTGCAGAGAAACCTTCCCTTCGAAAGAACGACCATT

ATGGCAGCATTTGCAGGAAATACTGAAGGCAGAACGTCCGATATGAGGACAGAAATCATA

AGAATGATGGAAAATGCCAAACCAGAAGATGTGTCATTCCAGGGGCGGGGAGTCTTCGAG

CTCTCGGACGAAAAGGCAACGAACCCGATCGTGCCTTCCTTTGACATGAATAATGAAGGA

TCTTATTTCTTCGGAGACAATGCAGAGGAGTATGACAATTAAAGAAAAATAC--------

------------------------------------------------------

>A_Guangdong_18SF020_2018_EPI1352806

AGCAAAAGCAGGGTAGATAATCACTCACCGAGTGACATCAACATCATGGCGTCTCAAGGC

ACCAAACGATCTTATGAACAGATGGAAACTGGTGGAGAGCGCCAGAATGCTACTGAGATC

AGGGCCTCTGTTGGAAGAATGGTTGGTGGCATTGGGAGGTTCTACATACAGATGTGCACA

GAACTCAAACTCAGCGACTATGAAGGGAGACTGATCCAGAACAGCATAACAATAGAGAGA

ATGGTACTTTCTGCATTTGATGAAAGAAGGAACAGGTATCTGGAAGAGCACCCCAGTGCA

GGGAAGGACCCTAAGAAAACTGGAGGTCCAATTTATCGGAGGAGAGACGGGAAATGGATT

AGAGAGCTGATTTTGTACGACAAAGAAGAGATCAGGAGGATTTGGCGCCAAGCAAACAAC

GGAGAGGACGCAACTGCTGGTCTTACCCACCTGATGATATGGCACTCCAATCTGAATGAT

GCCACATATCAGAGAACAAGAGCTCTCGTGCGTACCGGAATGGACCCCAGGATGTGCTCC

CTAATGCAGGGATCGACTCTTCCGAGAAGATCTGGAGCTGCTGGTGCAGCAGTGAAGGGG

GTAGGAACAATGGTGATGGAGCTGGTTCGAATGATAAAACGAGGGATTAACGACCGGAAT

TTCTGGAGAGGCGAAAATGGACGGAGAACAAGGATTGCATATGAGAGAATGTGCAACATC

CTCAAAGGGAAATTCCAAACAGCTGCACAAAGAGCAATGATGGATCAAGTGCGAGAGAGC

AGGAATCCTGGGAATGCTGAAATTGAAGATCTTATTTTTCTGGCACGGTCTGCACTCATC

CTGAGAGGATCAGTGGCCCATAAGTCCTGCTTGCCTGCTTGTGTGTACGGACTTGCAGTG

GCCAGTGGGTATGATTTCGAGAGAGAAGGATACTCTCTGGTTGGGATAGATCCTTTCCGT

TTGCTTCAAAACAGCCAGGTCTTTAGTCTCATTAGGCCAAATGAAAACCCAGCACATAAG

AGTCAATTAGTGTGGATGGCATGCCACTCTGCAGCATTTGAGGATCTCAGAGTCTCAAGT

TTCATCAGAGGGACAAGAGTTGTCCCAAGAGGGCAGCTATCCACTAGAGGGGTTCAAATT

GCTTCAAATGAGAACATGGAAACAATGGACTCCAACACACTTGAGCTAAGAAGTAGATAT

TGGGCTATAAGAACCAGGAGCGGAGGGAATACCAACCAGCAGAGGGCATCTGCAGGGCAG

ATTAGTGTTCAACCCACTTTCTCGGTGCAGAGAAACCTTCCCTTCGAAAGAGCGACCATT

ATGGCGGCATTTGCAGGAAATACTGAGGGCAGAACGTCCGACATGAGGACAGAAATCATA

AGAATGATGGAAAATGCCAAACCAGAAGATGTGTCATTCCAGGGGCGGGGAGTCTTCGAG

CTCTCGGACGAAAAGGCAACGAACCCGATCGTGCCTTCCTTTGACATGAATAATGAAGGA

TCTTATTTCTTCGGAGACAATGCAGAGGAGTATGACAATTAAAGAAAAATACCCTTGTTT

CTACT-------------------------------------------------

>A_goose_Hunan_116_2014_EPI958633

---------------------------------------------ATGGCGTCTCAAGGC

ACCAAACGATCTTATGAACAGATGGAAACTGGTGGAGAGCGCCAGAATGCTACTGAGATC

AGAGCCTCTGTTGGAAGAATGGTTAGTGGCATTGGGAGGTTCTACATACAGATGTGCACA

GAACTCAAACTCAGTGACCATGAAGGGAGGCTGATCCAGAACAGCATAACAATAGAGAGA

ATGGTACTTTCTGCATTTGATGAAAGAAGGAACAGGTATCTGGAAGAGCACCCCAGTGCG

GGGAAGGACCCTAAGAAAACTGGAGGTCCAATTTATCGGAGGAGAGACGGAAAATGGATG

AGAGAGCTGATTTTGTACGACAAAGAAGAGATCAGGAGGATTTGGCGCCAAGCGAACAAT

GGAGAGGACGCAACTGCTGGTCTTACCCACCTGATGATATGGCACTCCAATCTGAATGAT

GCCACATATCAGAGAACAAGAGCTCTCGTGCGTACCGGAATGGACCCCAGGATGTGCTCC

CTAATGCAGGGATCAACTCTCCCGAGAAGATCTGGAGCTGCTGGTGCAGCAGTGAAGGGG

GTAGGAACAATGGTGATGGAGCTGATTCGAATGATAAAACGAGGAATTAACGACCGGAAT

TTCTGGAGAGGCGAACATGGAAGAAGAACAAGGATTGCATATGATAGAATGTGCAACATC

CTCAAAGGGAAATTCCAAACAGCTGCACAAAGAGCAATGATGGATCAAGTGCGAGAGAGC

AGAAATCCTGGGAATGCTGAAATTGAAGATCTTATTTTTCTGGCACGGCCTGCACTCATC

CTGAGAGGATCAGTGGCCCATAAGTCCTGCTTGCCTGCTTGTGTGTACGGACTTGCAGTG

GCCAGTGGGTATGACTTCGAGAGAGAAGGATACTCTCTGGTTGGGATAGATCCTTTCCGT

CTGCTTCAAAACAGCCAGGTCTTTAGTCTCATTAGGCCAAATGAAAACCCAGCACATAAG

AGTCAATTAGTGTGGATGGCATGCCACTCTGCAGCATTTGAGGACCTCAGAGTCTCAAGT

TTCATCAGAGGAACAAGAGTGGTCCCAAGAGGGCAGCTATCTACTAGAGGGGTTCAAATT

GCTTCAAATGAGAATATGGAAACAATGGACTCCAACACACTTGAGCTGAGAAGTAGATAT

TGGGCTATAAGAACCAGGAGCGGAGGGAATACCAACCAGCAGAGGGCATCTGCAGGGCAG

ATCAGCGTTCAACCCACTTTCTCGGTGCAGAGAAACCTTCCCTTCGAGAGAGTGACCATT

ATGGCAGCATTTGCAGGAAATACTGAAGGCAGAACGTCCGACATGAGGACAGAAATCATA

AGAATGATGGAAAGTGCCAAACCAGAAGATGTGTCATTCCAGGGGCGGGGAGTCTTCGAG

CTCTCGGACGAAAAGGCAACGAACCCGATCGTGCCTTCCTTTGACATGAATAATGAAGGA

TCTTATTTCTTCGGAGACAATGCAGAGGAGTATGACAATTAA------------------

------------------------------------------------------

>A_duck_Wuhan_JXYFB22_2015_EPI683061

---------------------------------------------ATGGCGTCTCAAGGC

ACCAAACGATCTTATGAACAGATGGAAACTGGTGGAGAGCGCCAGAATGCTACTGAGATC

AGGGCCTCTGTTGGAAGAATGGTTAGTGGCATTGGGAGGTTCTACATACAGATGTGCACA

GAACTCAAACTCAGTGACCATGAAGGGAGACTGATCCAGAACAGCATAACAATAGAGAGA

ATGGTACTTTCTGCATTTGATGAAAGAAGGAACAGGTATCTGGAAGAGCACCCCAGTGCG

GGGAAGGACCCTAAGAAAACTGGAGGTCCAATTTATCGGAGGAGAGACGGGAAATGGATT

AGAGAGCTGATTTTGTACGACAAAGAAGAGATCAGGAGGATTTGGCGCCAAGCGAACAAT

GGAGAGGACGCAACTGCTGGTCTTACCCACCTGATGATATGGCACTCCAATCTGAATGAT

GCCACATATCAGAGAACAAGAGCTCTCGTGCGTACCGGAATGGACCCCAGGATGTGCTCC

CTAATGCAGGGATCAACTCTCCCGAGAAGATCTGGAGCTGCTGGTGCAGCAGTGAAGGGG

GTAGGAACAATGGTGATGGAGCTGATTCGAATGATAAAACGAGGGATTAACGACCGGAAT

TTCTGGAGAGGCGAAAATGGAAGAAGAACAAGGATTGCATATGAGAGAATGTGCAACATC

CTCAAAGGGAAATTCCAAACAGCTGCACAAAGAGCAATGATGGATCAAGTGCGAGAGAGC

AGAAATCCTGGGAATGCTGAAATTGAAGATCTTATTTTTCTGGCACGGTCTGCACTCATC

CTGAGAGGATCAGTGGCCCATAAATCCTGCTTGCCTGCTTGTGTGTACGGACTTGCAGTG

GCCAGTGGGTATGACTTCGAGAGAGAAGGATACTCTCTGGTTGGGATAGATCCTTTCCGT

CTGCTTCAAAACAGCCAGGTCTTTAGTCTCATTAGGCCAAATGAAAACCCAGCACATAAG

AGTCAATTAGTGTGGATGGCATGCCACTCTGCAGCATTTGAGGACCTCAGAGTCTCAAGT

TTCATCAGAGGAACAAGAGTGGTTCCAAGAGGGCAGCTATCTACTAGAGGGGTTCAAATT

GCTTCAAATGAGAACATGGAAACAATGGACTCCAACACACTTGAGCTGAGAAGTAGATAT

TGGGCTATAAGAACCAGGAGCGGAGGGAATACCAACCAGCAGAGGGCATCTGCAGGGCAG

ATCAGCGTTCAACCCACTTTCTCGGTGCAGAGAAACCTTCCCTTCGAAAGAGTGACCATT

ATGGCAGCATTTGCAGGAAATACTGAAGGCAGAACGTCCGACATGAGGACAGAAATCATA

AGAATGATGGAAAGTGCCAAACCAGAAGATGTGTCATTCCAGGGGCGGGGAGTCTTCGAG

CTCTCGGACGAAAAGGCAACGAACCCGATCGTGCCTTCCTTTGACATGAATAATGAAGGA

TCTTATTTCTTCGGAGACAATGCAGAGGAGTATGACAATTAA------------------

------------------------------------------------------

>A_chicken_Vietnam_NCVD-15A59_2015_EPI895064

------------GTAGATAATCACTCACCGAGTGACATCAACATTATGGCGTCTCAAGGC

ACCAAACGATCTTATGAACAGATGGAAACTGGTGGAGAGCGCCAGAATGCTACTGAGATC

AGGGCCTCTGTTGGAAGAATGGTTAGTGGCATTGGGAGGTTCTACATACAGATGTGCACA

GAACTCAAACTCAGTGACCATGAAGGGAGACTGATCCAGAACAGCATAACAATAGAGAGA

ATGGTACTTTCTGCATTTGATGAAAGAAGGAACAGGTATCTGGAAGAGAACCCCAGTGCG

GGGAAGGACCCTAAGAAAACTGGAGGTCCAATTTATCGGAGGAGAGACGGGAAATGGATT

AGAGAGCTGATTTTGTACGACAAAGAGGAGATCAGGAGGATTTGGCGCCAAGCGAACAAT

GGAGAGGACGCAACTGCTGGTCTTACCCACCTGATGATATGGCACTCCAATCTGAATGAT

GCCACATATCAGAGAACAAGAGCTCTCGTGCGTACCGGAATGGACCCCAGGATGTGCTCC

CTAATGCAGGGATCAACTCTCCCGAGAAGATCTGGAGCTGCTGGTGCAGCAGTGAAGGGG

GTAGGAACAATGGTGATGGAGCTGATTCGAATGATAAAACGAGGGATTAATGACCGGAAT

TTCTGGAGAGGCGAAAATGGAAGAAGAACAAGGATTGCATATGAGAGAATGTGCAACATC

CTCAAAGGGAAATTCCAAACAGCTGCACAAAGAGCAATGATGGATCAAGTGCGAGAGAGC

AGAAATCCTGGAAATGCTGAAATTGAAGATCTTATTTTTCTGGCACGGTCTGCACTCATC

CTGAGAGGATCAGTGGCCCATAAGTCCTGCTTGCCTGCTTGTGTGTACGGACTTGCAGTG

GCCAGTGGGTATGACTTCGAGAGAGAAGGATACTCTCTGGTTGGGATAGATCCTTTCCGT

TTGCTTCAAAACAGCCAGGTCTTTAGTCTCATCAGGCCAAATGAAAACCCAGCACATAAG

AGTCAATTAGTGTGGATGGCATGCCACTCTGCAGCATTTGAGGACCTCAGAGTCTCAAGT

TTCATCAGAGGAACAAGAGTGGTCCCAAGAGGGCAGCTATCCACTAGAGGGGTTCAAATT

GCTTCAAATGAGAACATGGAAACAATGGACTCCAACACACTTGAGCTGAGAAGTAGATAT

TGGGCTATAAGAACCAGGAGCGGAGGAAATACCAACCAGCAGAGGGCATCTGCAGGGCAG

ATCAGCGTTCAACCCACTTTCTCGGTGCAGAGAAACCTTCCCTTCGAAAGAGCGACCATT

ATGGCAGCATTTGCAGGAAATACTGAAGGTAGAACGTCTGACATGAGGACAGAAATCATA

AGAATGATGGAAAATGCCAAACCAGAAGATGTGTCATTCCAGGGGCGGGGAGTCTTCGAG

CTCTCGGACGAAAAGGCAACGAACCCGATCGTGCCTTCCTTTGACATGAATAATGAAGGA

TCTTATTTCTTCGGAGACAATGCAGAGGAGTATGACAATTAAAGAAAAATAC--------

------------------------------------------------------

>A_chicken_Vietnam_NCVD-15A55_2015_EPI895047

------------GTAGATAATCACTCACCGAGTGACATCAACACTATGGCGTCTCAAGGC

ACCAAACGATCTTATGAACAGATGGAAACTGGTGGAGAGCGCCAGAATGCTACTGAGATC

AGGGCCTCTGTTGGAAGAATGGTTAGTGGCATTGGGAGGTTCTACATACAGATGTGCACA

GAACTCAAACTCAGTGACCATGAAGGGAGACTGATCCAGAACAGCATAACAATAGAGAGA

ATGGTACTTTCTGCATTTGATGAAAGAAGGAACAGGTATCTGGAAGAGCACCCCAGTGCG

GGGAAGGACCCTAAGAAAACTGGAGGTCCAATTTATCGGAGGAGAGACGGGAAATGGATT

AGAGAGCTGATTTTGTACGACAAAGAAGAGATCAGGAGGATTTGGCGCCAAGCGAACAAT

GGAGAGGACGCAACTGCTGGTCTTACCCACCTGATGATATGGCACTCCAATCTGAATGAT

GCCACATATCAGAGAACAAGAGCTCTCGTGCGTACCGGAATGGACCCCAGGATGTGCTCC

CTAATGCAGGGATCAACTCTCCCGAGAAGATCTGGAGCTGCTGGTGCAGCAGTGAAGGGG

GTAGGAACAATGGTGATGGAGCTGATTCGAATGATAAAACGAGGGATTAACGACCGGAAT

TTCTGGAGAGGCGAAAATGGAAGAAGAACAAGGATTGCATATGAGAGAATGTGCAACATC

CTCAAAGGGAAATTCCAAACAGCTGCACAAAGAGCAATGATGGATCAAGTGCGAGAGAGC

AGAAATCCTGGGAATGCTGAAATTGAAGATCTCATTTTTCTGGCACGGTCTGCACTCATC

CTGAGAGGATCCGTGGCCCATAAGTCCTGCTTGCCTGCTTGTGTGTACGGACTTGCAGTG

GCCAGTGGGTATGACTTCGAGAGAGAAGGATACTCTCTGGTTGGGATAGATCCTTTCCGT

CTGCTTCAAAACAGCCAGGTCTTTAGTCTCATTAGGCCAAATGAAAACCCAGCACATAAG

AGTCAATTAGTGTGGATGGCATGCCACTCTGCAGCGTTTGAGGACCTCAGAGTCTCAAGT

TTCATCAGAGGAACAAGAGTGGTCCCAAGAGGGCAGCTATCCACTAGAGGGGTTCAAATT

GCTTCAAATGAGAACATGGAAACAATGGACTCCAACACACTTGAGCTGAGAAGTAGATAT

TGGGCTATAAGAACCAGGAGCGGAGGGAATACCAACCAGCAGAGGGCATCTGCAGGGCAG

ATCAGCGTTCAACCCACTTTCTCGGTGCAGAGAAACCTTCCCTTCGAACGAGCGACCATT

ATGGCAGCATTTGCAGGAAATACTGAAGGCAGAACGTCCGACATGAGGACAGAAATCATA

AGAATGATGGAAAATGCCAAACCAGAAGATGTGTCATTCCAGGGGCGGGGAGTCTTCGAG

CTCTCGGACGAAAAGGCAACGAACCCGATCGTGCCTTCCTTTGACATGAATAATGAAGGA

TCTTATTTCTTCGGAGACAATGCAGAGGAGTATGACAACTAAAGAAAAATACCCTTGTTT

CTAC--------------------------------------------------

>A_tundra_swan_Niigata_5112007_2016_EPI1184363

-------------------------CACCGAGTGACATCAACATCATGGCGTCTCAAGGC

ACCAAACGATCTTATGAACAGATGGAAACTGGTGGAGAGCGCCAGAATGCTACTGAAATC

AGGGCCTCTGTTGGAAGAATGATTGGTGGCATTGGGAGGTTCTACATACAGATGTGCACA

GAACTCAAACTCAGTGACCATGAAGGGAGGCTGATCCAGAACAGCATAACGATAGAGAGA

ATGGTACTTTCTGCCTTTGATGAGAGAAGGAACAGGTATCTGGAAGAGCACCCCAGTGCG

GGGAAGGACCCTAAGAAAACTGGAGGTCCAATTTATCGGAGGAGAGACGGGAAATGGATT

AGAGAGCTGATTTTGTACGACAAAGAAGAGATCAGGAGGATTTGGCGCCAAGCGAACAAT

GGAGAAGACGCAACTGCTGGTCTTACCCACCTGATGATATGGCACTCCAATCTGAATGAT

GCCACATATCAGAGAACAAGAGCTCTCGTGCGTACCGGAATGGACCCCAGGATGTGCTCC

CTAATGCAGGGATCAACTCTCCCGAGAAGATCTGGAGCTGCTGGTGCAGCAGTGAAGGGG

GTAGGAACAATGGTGATGGAGCTGATTCGAATGATAAAACGAGGGATTAACGACCGGAAT

TTCTGGAGAGGCGAAAATGGAAGAAGAACAAGGATTGCATATGAGAGAATGTGCAACATC

CTCAAAGGGAAATTCCAAACAGCTGCACAAAGAGCAATGATGGATCAAGTGCGAGAGAGC

AGAAATCCTGGGAATGCTGAAATTGAAGATCTCATTTTTCTGGCACGGTCTGCACTCATC

CTGAGAGGATCAGTGGCCCATAAGTCCTGCTTGCCTGCTTGTGTGTACGGACTTGCAGTG

GCCAGTGGGTATGACTTCGAGAGAGAAGGATACTCTCTGGTTGGGATAGATCCTTTCCGT

CTGCTTCAAAACAGCCAGGTCTTTAGTCTCATTAGGCCAAATGAAAGCCCAGCACATAAG

AGTCAATTAGTGTGGATGGCATGCCACTCTGCAGCATTTGAGGACCTCAGAGTCTCAAGT

TTCATCAGAGGAACAAGAGTGGTCCCACGAGGGCAGCTATCCACTAGAGGGGTTCAAATT

GCCTCAAATGAGAACATGGAAACAATGGACTCCAACACACTTGAGCTGAGAAGTAGATAC

TGGGCTATAAGAACCAGGAGCGGAGGGAATACCAACCAGCAGAGGGCATTTGCAGGGCAG

ATCAGCGTTCAACCCACTTTCTCGGTGCAGAGGAACCTTCCCTTCGAAAGAACGACCATT

ATGGCAGCATTTGCAGGAAATACTGAAGGCAGAACGTCCGACATGAGGACAGAAATCATA

AGAATGATGGAAAATGCCAAACCAGAAGATGTGTCATTCCAGGGGCGGGGAGTCTTCGAG

CTTTCGGACGAAAAGGCAACGAACCCGATCGTGCCTTCCTTTGACATGAATAATGAAGGA

TCTTATTTCTTCGGAGACAATGCAGAGGAGTATGACAATTAAAG----------------

------------------------------------------------------

>A_duck_Hyogo_1_2016_EPI866709

---------------------------------------------ATGGCGTCTCAAGGC

ACCAAACGATCTTATGAACAGATGGAAACTGGTGGAGAGCGCCAGAATGCTACTGAGATC

AGGGCCTCTGTTGGAAGAATGGTTGGTGGCATTGGGAGGTTCTACATACAGATGTGCACA

GAACTCAAACTCAGTGACCATGAAGGGAGGCTGATCCAGAACAGCATAACGATAGAGAGA

ATGGTACTTTCTGCCTTCGATGAGAGAAGGAACAGGTATCTGGAAGAGCACCCCAGTGCG

GGGAAGGACCCTAAGAAAACTGGAGGTCCAATTTATCGGAGGAGAGACGGGAAATGGATT

AGAGAGCTGATTTTGTACGACAAAGAAGAGATCAGGAGGATTTGGCGCCAAGCGAACAAT

GGAGAAGACGCAACTGCTGGTCTTACCCACCTGATGATATGGCACTCCAATCTGAATGAT

GCCACATATCAGAGAACAAGAGCTCTCGTGCGTACCGGAATGGACCCCAGGATGTGCTCC

CTAATGCAGGGATCAACTCTCCCGAGAAGATCTGGAGCTGCTGGTGCAGCAGTGAAGGGG

GTAGGAACAATGGTGATGGAGCTGATTCGAATGATAAAACGAGGGATTAACGACCGGAAT

TTCTGGAGAGGCGAAAATGGAAGAAGAACAAGGATTGCATATGAGAGAATGTGCAACATC

CTCAAAGGGAAATTCCAAACAGCTGCACAAAGAGCAATGATGGATCAAGTGCGAGAGAGC

AGAAATCCTGGGAATGCTGAAATTGAAGATCTCATTTTTCTGGCACGGTCTGCACTCATC

CTGAGAGGATCAGTGGCCCATAAGTCCTGCTTGCCTGCTTGTGTGTACGGACTTGCAGTG

GCCAGTGGGTATGACTTCGAGAGAGAAGGATACTCTCTGGTTGGGATAGATCCTTTCCGT

CTGCTTCAAAACAGCCAGGTCTTTAGTCTCATTAGGCCAAATGAAAGCCCAGCACATAAG

AGTCAATTAGTGTGGATGGCATGCCACTCTGCAGCATTTGAGGACCTCAGAGTCTCAAGT

TTCATCAGAGGAACAAGAGTGGTCCCACGAGGGCAGCTATCCACTAGAGGGGTTCAAATT

GCCTCAAATGAGAACATGGAAACAATGGACTCCAACACACTTGAGCTGAGAAGTAGATAC

TGGGCTATAAGAACCAGGAGCGGAGGGAATACCAACCAGCAGAGGGCATCTGCAGGGCAG

ATCAGTGTTCAACCCACTTTCTCGGTGCAGAGGAACCTTCCCTTCGAAAGGGCGACCATT

ATGGCAGCATTTGCAGGAAATACTGAAGGCAGAACGTCCGACATGAGGACAGAAATCATA

AGAATGATGGAAAATGCCAAACCAGAAGATGTGTCATTCCAGGGGCGGGGAGTCTTCGAG

CTTTCGGACGAAAAGGCAACGAACCCGATCGTGCCTTCCTTTGACATGAATAATGAAGGA

TCTTATTTCTTCGGAGACAATGCAGAGGAGTATGACAATTAA------------------

------------------------------------------------------

>A_Hubei_29578_2016_x_PR8_CNIC-HB29578_1369966

AGCAAAAGCAGGGTAGATAATCACTCACTGAGTGACATCAAAATCATGGCGTCTCAAGGC

ACCAAACGATCTTACGAACAGATGGAGACTGATGGAGAACGCCAGAATGCCACTGAAATC

AGAGCATCCGTCGGAAAAATGATTGGTGGAATTGGACGATTCTACATCCAAATGTGCACC

GAACTCAAACTCAGTGATTATGAGGGACGGTTGATCCAAAACAGCTTAACAATAGAGAGA

ATGGTGCTCTCTGCTTTTGACGAAAGGAGAAATAAATACCTTGAAGAACATCCCAGTGCG

GGGAAAGATCCTAAGAAAACTGGAGGACCTATATACAGGAGAGTAAACGGAAAGTGGATG

AGAGAACTCATCCTTTATGACAAAGAAGAAATAAGGCGAATCTGGCGCCAAGCTAATAAT

GGTGACGATGCAACGGCTGGTCTGACTCACATGATGATCTGGCATTCCAATTTGAATGAT

GCAACTTATCAGAGGACAAGAGCTCTTGTTCGCACCGGAATGGATCCCAGGATGTGCTCT

CTGATGCAAGGTTCAACTCTCCCTAGGAGGTCTGGAGCCGCAGGTGCTGCAGTCAAAGGA

GTTGGAACAATGGTGATGGAATTGGTCAGAATGATCAAACGTGGGATCAATGATCGGAAC

TTCTGGAGGGGTGAGAATGGACGAAAAACAAGAATTGCTTATGAAAGAATGTGCAACATT

CTCAAAGGGAAATTTCAAACTGCTGCACAAAAAGCAATGATGGATCAAGTGAGAGAGAGC

CGGAACCCAGGGAATGCTGAGTTCGAAGATCTCACTTTTCTAGCACGGTCTGCACTCATA

TTGAGAGGGTCGGTTGCTCACAAGTCCTGCCTGCCTGCCTGTGTGTATGGACCTGCCGTA

GCCAGTGGGTACGACTTTGAAAGGGAGGGATACTCTCTAGTCGGAATAGACCCTTTCAGA

CTGCTTCAAAACAGCCAAGTGTACAGCCTAATCAGACCAAATGAGAATCCAGCACACAAG

AGTCAACTGGTGTGGATGGCATGCCATTCTGCCGCATTTGAAGATCTAAGAGTATTAAGC

TTCATCAAAGGGACGAAGGTGCTCCCAAGAGGGAAGCTTTCCACTAGAGGAGTTCAAATT

GCTTCCAATGAAAATATGGAGACTATGGAATCAAGTACACTTGAACTGAGAAGCAGGTAC

TGGGCCATAAGGACCAGAAGTGGAGGAAACACCAATCAACAGAGGGCATCTGCGGGCCAA

ATCAGCATACAACCTACGTTCTCAGTACAGAGAAATCTCCCTTTTGACAGAACAACCATT

ATGGCAGCATTCAATGGGAATACAGAGGGGAGAACATCTGACATGAGGACCGAAATCATA

AGGATGATGGAAAGTGCAAGACCAGAAGATGTGTCTTTCCAGGGGCGGGGAGTCTTCGAG

CTCTCGGACGAAAAGGCAGCGAGCCCGATCGTGCCTTCCTTTGACATGAGTAATGAAGGA

TCTTATTTCTTCGGAGACAATGCAGAGGAGTACGACAATTAAAGAAAAATACCCTTGTTT

CTACTA------------------------------------------------

>A_chicken_Hubei_ZYSJF38_2016_EPI895176

--------------GGGTTATCACTCACTGAGTGACATCAACATCATGGCGTCTCAAGGC

ACCAAACGATCCTATGAACAGATGGAAACTGGTGGGGAACGCCAGAATGCTACTGAGATC

AGGGCATCTGTTGGAAGAATGGTTAGCGGCATTGGGAGATTCTACATACAGATGTGTACA

GAACTCAAGCTCAGTGACAATGAAGGGAGGCTGATTCAGAACAGTATAACAATAGAGAGA

ATGGTACTCTCTGCCTTTGATGAAAGAAGGAACAGATATCTGGAAGAGCACCCCAGTGCA

GGAAAGGACCCTAAGAAAACTGGAGGTCCAATTTACAGGAGAAGAAACGGAAAATGGGTC

AGAGAGCTGATCCTATATGACAAAGAGGAAATCAGGAGAATTTGGCGACAAGCGAACAAT

GGAGAAGATGCAACTGCTGGTCTTACCCATCTGATGATATGGCATTCCAACCTGAATGAT

GCTACCTATCAGAGAACAAGAGCTCTAGTGCGTACTGGAATGGATCCCCGGATGTGCTCT

CTGATGCAAGGATCAACTCTCCCGAGGAGATCTGGAGCTGCAGGTGCAGCAGTGAAGGGG

ATAGGGACAATGGTAATGGAACTGATTCGGATGATAAAACGAGGGATCAACGACCGGAAT

TTCTGGAGAGGCGAAAATGGAAGAAGGACAAGAATTGCATATGAGAGAATGTGCAACATC

CTCAAAGGGAAATTCCAAACAGCAGCACAAAGGGCAATGATGGATCAAGTACGAGAGAGC

AGAAATCCTGGGAATGCTGAAATAGAAGATCTCATTTTTCTGGCAAGGTCTGCACTCATC

CTGAGAGGATCAGTGGCTCATAAATCCTGCTTGCCTGCTTGTGTGTACGGACTTGCAGTG

GCTAGTGGATATGACTTTGAGAGAGAAGGGTACTCCTTGGTTGGAATAGATCCTTTCCGT

CTGCTTCAAAACAGCCAGGTCTTTAGTCTCATTAGACCAAATGAGAACCCAGCACATAAG

AGCCAACTAGTGTGGATGGCATGCCACTCTGCAGCGTTTGAGGACCTTAGGGTATCAAGT

TTCATTAGAGGGACAAGAATGGTCCCAAGAGGTCAGCTATCCACTAGAGGGGTTCAAATT

GCCTCAAATGAGAACATGGAAGCAATGGACTCCAATACTCTTGAACTGAGAAGTAGATAT

TGGGCTATAAGAACCAGAAGCGGAGGGAACACCAACCAACAGAGGGCATCTGCAGGACAG

GTCAGCGTTCAACCCACTTTCTCAGTACAGAGAAACCTTCCTTTCGAAAGAGCAACCATT

ATGGCAGCATTTACAGGAAATACTGAGGGTAGAACATCTGACATGAGGACTGAAATCATA

AGAATGATGGAAAGTGCCAGACCAGAAGATGTGTCATTCCAGGGGCGGGGAGTCTTCGAG

CTCTCGGACGAAAAGGCAACGAACCCGATCGTGCCTTCCTTTGACATGAATAATGAAGGA

TCTTATTTCTTCGGAGACAATGCAGAGGAGTATGACAATTAAAGAAAAATACCCTTGTTT

CTACA-------------------------------------------------

>A_gyrfalcon_Washington_41088-6_2014_EPI569391

--------------------TCACTCACTGAGTGACATCAACATCATGGCGTCTCAAGGC

ACCAAACGATCTTATGAACAGATGGAAACTGGTGGAGAACGCCAGAATGCCACTGAAATC

AGAGCATCTGTTGGAAGAATGGTTGGTGGAATTGGAAGGTTTTATATACAGATGTGCACT

GAACTCAAACTCAGCGATTATGAGGGGAGACTGATCCAGAACAGCATAACAATAGAGAGA

ATGGTTCTCTCTGCATTTGATGAAAGGAGGAACAAGTACCTGGAAGAACATCCCAGTGCG

GGAAAGGACCCAAAGAAAACTGGAGGTCCAATCTACAGAAGAAGAGACGGAAAGTGGATG

AGGGAGCTGATTCTGTATGACAAAGAAGAGATCAGAAGGATCTGGCGTCAAGCGAATAAT

GGAGAAGATGCAACTGCTGGTCTCACCCATCTGATGATCTGGCACTCCAACCTGAATGAT

GCCACATATCAGAGAACAAGGGCTCTCGTGCGCACTGGAATGGATCCCAGAATGTGCTCT

CTGATGCAAGGATCAACTCTCCCAAGAAGGTCTGGAGCTGCTGGTGCAGCAGTAAAAGGG

GTCGGAACAATGGTAATGGAATTGATTCGAATGATAAAGCGAGGGATTAATGATCGGAAT

TTCTGGAGAGGCGAAAATGGAAGAAGGACAAGGATTGCCTATGAGAGAATGTGCAACATC

CTCAAAGGGAAATTTCAAACAGCAGCACAAAGAGCAATGATGGATCAAGTGCGAGAAAGC

AGGAATCCTGGGAATGCTGAAATTGAAGATCTCATTTTTCTGGCACGGTCTGCACTCATC

CTGAGAGGATCAGTGGCCCACAAGTCTTGTCTGCCTGCTTGTGTTTACGGACTTGCTGTG

GTCAGTGGATACGACTTTGAGAGAGAAGGATACTCTCTGGTCGGAATAGACCCTTTCCGT

CTACTTCAAAACAGCCAGGTCTTCAGTCTCATTAGACCAAATGAAAACCCAGCACATAAA

AGCCAATTGGTATGGATGGCATGCCATTCAGCAGCGTTTGAGGACCTGAGGGTATCAAGT

TTCATCAGAGGGACAAGAGTGGTCCCAAGAGGACAACTATCCACCAGAGGAGTTCAAATT

GCATCAAATGAAAACATGGAAACAATGGACTCCAGCACTCTTGAATTGAGAAGCAGATAC

TGGGCTATAAGAACCAGGAGTGGAGGAAACACCAACCAACAGAGAGCTTCTGCAGGACAA

ATCAGCGTACAACCCACCTTCTCAGTACAGAGAAATCTTCCCTTTGAAAGAGCGACCATC

ATGGCGGCATTTACAGGGAACACTGAAGGCAGGACCTCTGACATGAGGACTGAAATCATA

AGAATGATGGAAAGTGCCAAACCAGAAGATGTGTCCTTCCAGGGGCGGGGAGTCTTCGAG

CTCTCGGACGAAAAGGCAACGAACCCGATCGTGCCTTCCTTTGACATGAGCAACGAAGGA

TCTTATTTCTTCGGAGACAGTGCAGAGGAGTATGACAATTAAAG----------------

------------------------------------------------------

>A_chicken_Washington_3490-18_2015_EPI590693

---------------------------------------------ATGGCGTCTCAAGGC

ACCAAACGATCTTATGAGCAAATGGAAACTGGTGGAGAACGCCAGAATGCCACTGAAATC

AGAGCATCTGTTGGGAGAATGGTTGGTGGAATCGGAAGATTCTACATACAGATGTGTACT

GAACTCAAGCTCAGTGACTATGAAGGAAGGCTGATCCAAAACAGCATAACCATAGAGAGA

ATGGTTCTCTCGGCATTTGATGAGAGGAGAAATAAATATCTGGAAGAGCATCCCAGTGCT

GGGAAAGACCCCAAGAAGACTGGAGGCCCAATCTACAGAAGAAGAGATGGAAAATGGATG

AGAGAGTTGATCCTATATGACAAAGAAGAAATCAGGAGGATTTGGCGTCAAGCAAATAAC

GGAGAAGACGCAACTGCTGGTCTCACCCATTTGATGATTTGGCATTCCAATCTGAATGAT

GCCACATACCAGAGAACAAGGGCACTTGTGCGTACTGGGATGGATCCCAGGATGTGCTCC

CTTATGCAAGGCTCAACTCTTCCAAGAAGATCTGGGGCTGCTGGAGCAGCAGTGAAAGGG

GTTGGAACAATGGTGATGGAACTGATCCGGATGATCAAGCGAGGTATCAATGATAGGAAT

TTCTGGAGAGGCGAAAATGGACGGAGGACGAGAATTGCTTATGAAAGAATGTGCAACATC

CTCAAGGGGAAATTCCAAACAGCAGCACAACGAGCAATGATGGATCAGGTGAGAGAAAGC

CGGAATCCTGGGAATGCTGAAATTGAAGATCTCATCTTCCTTGCACGGTCTGCTCTCATT

CTGAGAGGATCAGTAGCTCATAAGTCCTGCCTGCCTGCTTGTGTGTATGGGCTTGCAGTG

GCCAGTGGATACGACTTTGAGAGAGAGGGATATTCCCTAGTCGGAATTGATCCTTTCCGT

CTGCTCCAAAACAGTCAAGTCTTCAGCCTCATCAGACCAAACGAAAATCCAGCGCATAAG

AGTCAGCTGGTATGGATGGCATGTCACTCTGCTGCATTTGAAGATTTGAGAGTGTCAAGC

TTCATCAGAGGAACAAGAGTAGTCCCAAGAGGACAACTATCCACCAGAGGAGTTCAGATT

GCTTCAAATGAGAACATGGAGACAATGGACTCCAGCACTCTTGAACTGAGGAGCAGATAC

TGGGCTATAAGGACAAGAAGCGGAGGGAACACTAACCAGCAGAGAGCATCTGCAGGGCAA

ATCAGCGTGCAGCCCACCTTCTCTGTGCAGAGAAACCTCCCATTCGAGAGGGCAACCATC

ATGGCGGCATTTACAGGAAATACCGAGGGTAGAACTTCAGACATGAGGACTGAGATCATA

AGGATGATGGAAAATGCCAGACCTGAAGATGTGTCTTTCCAGGGGCGGGGAGTCTTCGAG

CTCTCGGACGAAAAGGCAACGAACCCGATCGTGCCTTCCTTTGACATGAGTAATGAAGGA

TCTTATTTCTTCGGAGACAATGCAGAGGAGTATGACAATTAA------------------

------------------------------------------------------

>A_Perigrine_falcon_Netherlands_18003274_1327121

------------GTAGATAATCACTCACTGAGTGACATCAACATCATGGCGTCTCAAGGC

ACCAAACGATCTTATGAACAGATGGAAACTGCTGGAGAGCGCCAGAATGCCACTGAGATC

AGAGCATCTGTTGGAAGAATGGTTGGTGGAATTGGGAGGTTCTACATACAGATGTGCACT

GAGCTCAAACTCAGCGACTATGAAGGAAGGCTGATCCAGAACAGCATAACAATAGAGAGA

ATGGTTCTCTCCGCATTTGATGAAAGGAGGAACAAATATCTGGAAGAACATCCCAGTGCG

GGGAAAGACCCGAAGAAAACTGGAGGTCCAATTTATCGAAGGAGAGATGGGAAATGGGTG

AGAGAGCTGATCCTGTATGACAAAGAGGAGATCAGGAGGATCTGGCGTCAAGCGAACAAT

GGAGAAGACGCAACCGCTGGTCTCACTCACCTGATGATCTGGCATTCCAATCTAAATGAT

GCCACATACCAGAGGACAAGAGCTCTCGTGCGCACTGGGATGGACCCTAGGATGTGCTCT

CTCATGCAAGGATCAACTCTCCCAAGGAGGTCTGGAGCTGCTGGTGCAGCAGTAAAGGGA

GTCGGGACGATGGTGATGGAACTAATTCGGATGATAAAGCGAGGAATTAATGATCGAAAC

TTCTGGAGAGGCGAGAATGGACGAAGGACAAGGATTGCATATGAGAGAATGTGCAACATC

CTCAAAGGGAAATTCCAAACAGCAGCACAAAGAGCAATGATGGATCAGGTGCGTGAAAGC

AGGAATCCTGGCAATGCTGAAATTGAAGATCTCATCTTTCTGGCAAGGTCTGCTCTCATC

CTGAGAGGATCAGTGGCCCATAAGTCCTGCTTGCCTGCTTGTGTGTACGGACTCGCTGTG

GTCAGTGGATACGACTTTGAGAGAGAAGGGTACTCTCTAGTTGGAATAGATCCTTTCCGT

CTGCTTCAAAACAGCCAGGTCTTCAGTCTCATTAGACCAAATGAGAATCCAGCACACAAG

AGTCAATTGGTGTGGATGGCATGTCATTCTGCAGCATTCGAGGATCTGAGGGTCTCAAGT

TTCATCAGAGGAACAAGAGTAGTCCCAAGAGGACAACTATCCACCAGAGGAGTTCAAATT

GCTTCAAATGAGAACATGGAAACAATGGGCTCCAGCACTCTTGAACTGAGAAGCAGATAT

TGGGCTATAAGAACCAGGAGTGGAGGAAACACCAACCAACAGAGAGCATCTGCAGGACAA

ATCAGTGTACAGCCCACTTTCTCAGTACAGAGAAACCTTCCCTTCGAAAGAGCGACCATT

ATGGCAGCATTCACAGGGAACACTGAGGGCAGGACGTCCGACATGAGGACTGAAATCATA

AGAATGATGGAAAGTGCCAGACCAGAAGATGTGTCTTTCCAGGGGCGGGGAGTCTTCGAG

CTCTCGGACGAAAAGGCAACGAACCCGATCGTGCCTTCCTTTGACATGAGTAATGAAGGA

TCTTATTTCTTCGGAGACAATGCAGAGGAGTACGACAATTAAAGAAAAATAC--------

------------------------------------------------------

>A_Fujian-Sanyuan_21099_2017_x_PR8_CNIC_1369974

AGCAAAAGCAGGGTAGATAATCACTCACTGAGTGACATCAAAATCATGGCGTCTCAAGGC

ACCAAACGATCTTACGAACAGATGGAGACTGATGGAGAACGCCAGAATGCCACTGAAATC

AGAGCATCCGTCGGAAAAATGATTGGTGGAATTGGACGATTCTACATCCAAATGTGCACC

GAACTCAAACTCAGTGATTATGAGGGACGGTTGATCCAAAACAGCTTAACAATAGAGAGA

ATGGTGCTCTCTGCTTTTGACGAAAGGAGAAATAAATACCTTGAAGAACATCCCAGTGCG

GGGAAAGATCCTAAGAAAACTGGAGGACCTATATACAGGAGAGTAAACGGAAAGTGGATG

AGAGAACTCATCCTTTATGACAAAGAAGAAATAAGGCGAATCTGGCGCCAAGCTAATAAT

GGTGACGATGCAACGGCTGGTCTGACTCACATGATGATCTGGCATTCCAATTTGAATGAT

GCAACTTATCAGAGGACAAGAGCTCTTGTTCGCACCGGAATGGATCCCAGGATGTGCTCT

CTGATGCAAGGTTCAACTCTCCCTAGGAGGTCTGGAGCCGCAGGTGCTGCAGTCAAAGGA

GTTGGAACAATGGTGATGGAATTGGTCAGAATGATCAAACGTGGGATCAATGATCGGAAC

TTCTGGAGGGGTGAGAATGGACGAAAAACAAGAATTGCTTATGAAAGAATGTGCAACATT

CTCAAAGGGAAATTTCAAACTGCTGCACAAAAAGCAATGATGGATCAAGTGAGAGAGAGC

CGGAACCCAGGGAATGCTGAGTTCGAAGATCTCACTTTTCTAGCACGGTCTGCACTCATA

TTGAGAGGGTCGGTTGCTCACAAGTCCTGCCTGCCTGCCTGTGTGTATGGACCTGCCGTA

GCCAGTGGGTACGACTTTGAAAGGGAGGGATACTCTCTAGTCGGAATAGACCCTTTCAGA

CTGCTTCAAAACAGCCAAGTGTACAGCCTAATCAGACCAAATGAGAATCCAGCACACAAG

AGTCAACTGGTGTGGATGGCATGCCATTCTGCCGCATTTGAAGATCTAAGAGTATTAAGC

TTCATCAAAGGGACGAAGGTGCTCCCAAGAGGGAAGCTTTCCACTAGAGGAGTTCAAATT

GCTTCCAATGAAAATATGGAGACTATGGAATCAAGTACACTTGAACTGAGAAGCAGGTAC

TGGGCCATAAGGACCAGAAGTGGAGGAAACACCAATCAACAGAGGGCATCTGCGGGCCAA

ATCAGCATACAACCTACGTTCTCAGTACAGAGAAATCTCCCTTTTGACAGAACAACCATT

ATGGCAGCATTCAATGGGAATACAGAGGGGAGAACATCTGACATGAGGACCGAAATCATA

AGGATGATGGAAAGTGCAAGACCAGAAGATGTGTCTTTCCAGGGGCGGGGAGTCTTCGAG

CTCTCGGACGAAAAGGCAGCGAGCCCGATCGTGCCTTCCTTTGACATGAGTAATGAAGGA

TCTTATTTCTTCGGAGACAATGCAGAGGAGTACGACAATTAAAGAAAAATACCCTTGTTT

CTACTA------------------------------------------------

>A_duck_Sichuan_NCXJ16_2014_EPI590873

---------------------------------------------ATGGCGTCTCAAGGC

ACCAAACGATCTTATGAACAGATGGAAACTGGTGGAGAGCGCCAGAATGCTACTGAAATC

AGGGCATCTGTTGGAAGAATGATTAGTGGCATTGGGAGGTTCTACATACAGATGTGCACA

GAACTCAAACTCGGTGACCATGAAGGGAGACTGATCCAGAACAGCATAACAATAGAGAGA

ATGGTACTTTCTGCATTTGATGAAAGAAGGAACAGGTACCTGGAAGAGCACCCCAGTGCG

GGGAAGGACCCTAAGAAAACTGGAGGTCCAATTTATCGGAGGAGAGACGGGAAATGGATG

AGAGAGCTGATTTTGTACGACAAAGAAGAGATTAGGAGGATTTGGCGCCAAGCGAACAAT

GGAGAGGACGCAACTGCTGGTCTTACCCACCTGATGATATGGCACTCCAACCTGAATGAT

GCCACATATCAGAGAACAAGAGCTCTCGTGCGTACCGGAATGGACCCCAGGATGTGCTCT

CTAATGCAGGGATCAACTCTCCCGAGGAGATCTGGAGCTGCTGGTGCAGCGGTGAAAGGG

GTAGGAACAATGGTGATGGAGCTGATTCGAATGATAAAACGAGGGATTAACGACCGGAAT

TTCTGGAGAGGCGAAAATGGAAGAAGAACAAGGATTGCATATGAGAGAATGTGCAACATC

CTCAAAGGGAAATTCCAAACAGCTGCACAAAGAGCAATGATGGATCAAGTGCGAGAGAGC

AGAAATCCTGGGAATGCTGAAATCGAAGATCTTATTTTTCTGGCACGGTCGGCACTCATC

CTGAGAGGATCAGTGGCCCATAAGTCCTGCTTGCCTGCTTGTGTGTACGGACTTGCAGTG

GCCAGTGGGTATGACTTCGAGAGAGAAGGATACTCTCTGGTTGGGATAGATCCATTCCGT

CTGCTTCAAAACAGCCAGGTCTTTAGTCTCATTAGACCAAATGAAAACCCAGCACATAAG

AGTCAGTTAGTGTGGATGGCATGCCACTCTGCAGCATTTGAAGACCTCAGAGTCTCAAGT

TTCATCAGAGGAACAAGAGTGGTCCCAAGAGGGCAGCTATCCACTAGAGGGGTTCAAATT

GCTTCAAATGAGAACATGGAAACAATGGACTCCAACACACTTGAACTGAGAAGCAGATAT

TGGGCTATAAGAACCAGGAGCGGAGGGAACACCAACCAGCAGAGGGCATCTGCAGGGCAG

ATCAGCGTTCAACCCACTTTCTCGGTGCAGAGAAACCTTCCCTTCGAAAGAGCGACCATT

ATGGCAGCATTTGCAGGAAATACTGAGGGCAGAACATCCGACATGAGGACAGAAATCATA

AGAATGATGGAAAGTGCCAAACCAGAAGATGTGTCATTCCAGGGGCGGGGAGTCTTCGAG

CTCTCGGACGAAAAGGCAACGAACCCGATCGTGCCTTCCTTTGACATGAATAATGAAGGA

TCTTATTTCTTCGGAGACAATGCAGAGGAGTATGACAATTAA------------------

------------------------------------------------------

>A_Sichuan_26221_2014_EPI533588

---------------------------------------------ATGGCGTCTCAAGGC

ACCAAACGATCTTATGAACAGATGGAAACTGGTGGAGAGCGCCAGAATGCTACTGAAATC

AGGGCATCTGTTGGAAGAATGATTAGTGGCATTGGGAGGTTCTACATACAGATGTGCACA

GAACTCAAACTCGGTGACCATGAAGGGAGACTGATCCAGAACAGCATAACAATAGAGAGA

ATGGTACTTTCTGCATTTGATGAAAGAAGGAACAGGTACCTGGAAGAGCACCCCAGTGCG

GGGAAGGACCCTAAGAAAACTGGAGGTCCAATTTATCGGAGGAGAGACGGGAAATGGATG

AGAGAGCTGATTTTGTACGACAAAGAAGAGATTAGGAGGATTTGGCGCCAAGCGAACAAT

GGAGAGGACGCAACTGCTGGTCTTACCCACCTGATGATATGGCACTCCAACCTGAATGAT

GCCACATATCAGAGAACAAGAGCTCTCGTGCGTACCGGAATGGACCCCAGGATGTGCTCT

CTAATGCAGGGATCAACTCTCCCGAGGAGATCTGGAGCTGCTGGTGCAGCGGTGAAAGGG

GTAGGAACAATGGTGATGGAGCTGATTCGAATGATAAAACGAGGGATTAACGACCGGAAT

TTCTGGAGAGGCGAAAATGGAAGAAGAACAAGGATTGCATATGAGAGAATGTGCAACATC

CTCAAAGGGAAATTCCAAACAGCTGCACAAAGAGCAATGATGGATCAAGTGCGAGAGAGC

AGAAATCCTGGGAATGCTGAAATTGAAGATCTTATTTTTCTGGCACGGTCGGCACTCATC

CTGAGAGGATCAGTGGCCCATAAGTCCTGCTTGCCTGCTTGTGTGTACGGACTTGCAGTG

GCCAGTGGGTATGACTTCGAGAGAGAAGGATACTCTCTGGTTGGGATAGATCCATTCCGT

CTGCTTCAAAACAGCCAGGTCTTTAGTCTCATTAGACCAAATGAAAACCCAGCACATAAG

AGTCAGTTAGTGTGGATGGCATGCCACTCTGCAGCATTTGAAGACCTCAGAGTCTCAAGT

TTCATCAGAGGAACAAGAGTGGTCCCAAGAGGGCAGCTATCCACTAGAGGGGTTCAAATT

GCTTCAAATGAGAACATGGAAACAATGGACTCCAACACACTTGAACTGAGAAGCAGATAT

TGGGCTATAAGAACCAGGAGCGGAGGGAACACCAACCAGCAGAGGGCATCTGCAGGGCAG

ATCAGCGTTCAACCCACTTTCTCGGTGCAGAGAAACCTTCCCTTCGAAAGAGCGACCATT

ATGGCAGCATTTGCAGGAAATACTGAGGGCAGAACATCCGACATGAGGACAGAAATCATA

AGAATGATGGAAAGTGCCAAACCAGAAGATGTGTCATTCCAGGGGCGGGGAGTCTTCGAG

CTCTCGGACGAAAAGGCAACGAACCCGATCGTGCCTTCCTTTGACATGAATAATGAAGGA

TCTTATTTCTTCGGAGACAATGCAGAGGAGTATGACAATTAA------------------

------------------------------------------------------
