# Supplementary material for: Highly pathogenic avian influenza virus of the A/H5N8 subtype, clade 2.3.4.4b, caused outbreaks in Kazakhstan in 2020
Source: PeerJ. 2022 Mar 2;10:e13038. doi: 10.7717/peerj.13038 (PMC8898005; doi:10.7717/peerj.13038)
Supplement: Figure S7 [file peerj-10-13038-s008.docx]

**Fig.S7** Alignment of the nucleotide sequences for the MP segment used in this study

>A_duck_Lao_961_2010_EPI335152

--AGCAAAAGCAGGTAGATGTTGAAAGATGAGTCTTCTAACTGAGGTCGAAACGTACGTT

CTCTCTATCATCCCGTCAGGCCCCCTCAAAGCCGAGATAGCGCAGAAACTTGAGGATGTC

TTTGCAGGAAAGAACACCGATCTCGAAGCTCTCATGGAGTGGCTAAAGACAAGACCAATC

CTGTCACCTCTGACTAAAGGGATCTTGGGATTTGTATTCACGCTCACCGTGCCCAGTGAG

CGAGGACTGCAGCGTAGACGCTTTGTCCAGAATGCCCTAAATGGAAATGGAGATCCAAAT

AATATGGATAGAGCAGTTAAGCTATATAAGAAGCTGAAAAGAGAAATTACATTCCATGGG

GCTAAGGAAGTCGCACTCAGCTACTCAACCGGTGCACTTGCCAGTTGCATGGGTCTCATA

TACAACAGGATGGGAACGGTGACTACGGAAGTGGCTTTTGGCCTAGTGTGTGCCACTTGT

GAGCAGATTGCAGATTCACAGCATCGGTCTCACAGACAGATGGCAACCATCACCAACCCA

CTAATCAGGCATGAAAACAGAATGGTGCTGGCTAGCACTACAGCTAAGGCTATGGAGCAG

ATGGCGGGATCAAGCGAGCAGGCAGCGGAAGCCATGGAGATTGCTAATCAGGCTAGGCAG

ATGGTGCAGGCAATGAGGACAATTGGGACTCATCCTAACTCTAGTGCTGGTCTGAGAGAT

AATCTTCTTGAAAATTTGCAGGCATACCAGAAACGAATGGGAGTGCAGATGCAGCGATTC

AAGTGATCCTCTTGTTGTTGCTGCAAGTATCATTGGGATCTTGCACTTGATATTGTGGAT

TCTTGATCGTCTTTTCTTCAAATGCATTTATCGTCGCCTTAAATACGGTTTGAAAAGAGG

GCCTTCTACGGAAGGAGTACCTGAGTCTATGAGGGAAGAGTACCGGCAGGAACAGCAGAG

TGCTGTGGATGTTGACGATGGTCATTTTGTCAACATAGAATTGGAGTAAAAAACTACCTT

GTTTCTACT----------------------------------

>A_duck_Lao_567_2010_EPI335176

--AGCAAAAGCAGGTAGATGTTGAAAGATGAGTCTTCTAACTGAGGTCGAAACGTACGTT

CTCTCTATCATCCCGTCAGGCCCCCTCAAAGCCGAGATAGCGCAGAAACTTGAGGATGTC

TTTGCAGGAAAGAACACCGATCTCGAGGCTCTCATGGAGTGGCTAAAGACAAGACCAATC

CTGTCACCTCTGACTAAAGGGATATTGGGATTTGTATTCACGCTCACCGTGCCCAGTGAG

CGAGGACTGCAGCGTAGACGCTTTGTCCAGAATGCCCTAAATGGGAATGGAGATCCAAAC

AACATGGACAGAGCGGTCAAACTGTACAGGAAGCTGAAAAGGGAAATAACATTCCATGGG

GCTAAGGAGGTCGCACTCAGCTACTCAACCGGTGCACTTGCCAGTTGCATGGGTCTCATA

TACAACAGGATGGGAACGGTGACTACGGAAGTGGCTTTCGGCCTAGTGTGTGCCACTTGT

GAGCAGATTGCAGATTCACAGCATCGGTCTCACAGACAGATGGCAACCATCACCAACCCA

CTAATCAGGCATGAGAACAGAATGGTACTGGCCAGTACTACGGCTAAAGCCATGGAGCAA

ATGGCAGGATCAAGTGAGCAAGCAGCGGAAGCCATGGAGGTCGCTAATCAGGCTAGGCAG

ATGGTGCAGGCAATGAGGACAATTGGGACTCATCCCAACTCTAGTGCTGGTCTGAGAGAT

AACCTTCTTGAAAATTTGCAGGCCTACCAGAAACGAATGGGAGTGCAGATGCAACGATTC

AAGTGATCCTCTTGTTGTTGCCGCAAGTATCATTGGAATCTTGCACTTGATATTGTGGAT

TCTTGATCGTCTTTTCTTCAAATGCATTTATCGTCGCCTTAAATACGGTTTGAAAAGAGG

GCCTTCTACGGAAGGAGTGCCTGAGTCTATGAGGGAAGAGTACCGGCAGGAACAGCAGAG

TGCTGTGGATGTTGACGATGGTCATTTTGTCAACATAGAATTGGAGTAAAAAACTACCTT

GTTTCTACT----------------------------------

>A_breeder_duck_Korea_Gochang1_2014_EPI509701

-----------------ATATTGAAAGATGAGTCTTCTAACCGAGGTCGAAACGTACGTT

CTCTCTATCATCCCGTCAGGCCCCCTCAAAGCCGAGATCGCGCAGAAACTTGAAGATGTC

TTTGCAGGGAAGAACACCGATCTCGAGGCTCTCATGGAGTGGCTAAAGACAAGACCAATC

CTGTCACCTCTGACTAAAGGGATTTTGGGATTTGTATTCACGCTCACCGTGCCCAGTGAG

CGAGGACTGCAGCGTAGACGCTTTGTCCAGAACGCCCTAAATGGAAATGGAGATCCAAAT

AATATGGATAGGGCAGTTAAGCTATATAAGAAGCTGAAAAGGGAAATAACATTCCATGGG

GCTAAGGAGGTCGCACTTAGTTACTCAACCGGTGCGCTTGCCAGTTGCATGGGTCTCATA

TACAATAGGATGGGAACGGTGACTACAGAAGTGGCTTTTGGCCTAGTGTGTGCCACTTGT

GAGCAGATTGCAGATTCACAGCATCGGTCTCACAGACAGATGGCAACCATCACCAACCCA

TTAATCAGGCATGAGAACAGAATGGTGCTGGCCAGCACTACAGCTAAGGCCATGGAGCAG

ATGGCAGGATCAAGTGAGCAGGCAGCGGAAGCCATGGAAGTAGCTAATCAGGCTAGGCAG

ATGGTACAGGCAATGAGGACAATTGGGACTCATCCTAACTCCAGTGCTGGTCTGAGAGAT

AATCTTCTTGAAAATTTGCAGGCCTACCAGAACCGAATGGGAGTGCAGATGCAGCGATTC

AAGTGATCCTCTTGTTGTTGCCGCAAGTATCATTGGGATCTTGCACTTGATATTGTGGAT

TCTTGATCGTCTTTTCTTCAAATGCATTTACCGTTGCCTTAAACACGGTTTGAAAATAGG

GCCTTCTACGGAAGGGGTACCTGAGTCTATGAGGGAAGAATACCGGCAGGAACAGCAGAA

TGCTGTGGATGTTGACGATGGTCATTTTGTCAACATAGAATTGGAGTAA-----------

-------------------------------------------

>A_broiler_duck_Korea_Buan2_2014_EPI509711

-----------------ATATTGAAAGATGAGTCTTCTAACCGAGGTCGAAACGTACGTT

CTCTCTATCATCCCGTCAGGCCCCCTCAAAGCCGAGATCGCGCAGAGACTTGAAGATGTC

TTTGCAGGGAAAAACACCGATCTCGAGGCTCTCATGGAGTGGCTAAAGACAAGACCAATC

CTGTCACCTCTGACTAAAGGGATTTTGGGATTTGTGTTCACGCTCACCGTGCCCAGTGAG

CGAGGACTGCAGCGTAGACGCTTCGTCCAGAATGCCCTAAATGGAAATGGGGATCCAAAT

AATATGGATAAGGCAGTTAAGCTATATAAGAAGCTGAAAAGAGAGATAACATTCCATGGG

GCTAAGGAGGTCGCACTTAGCTACTCAACCGGTGCACTTGCCAGCTGCATGGGTCTCATA

TACAACAGGATGGGAACGGTGACTACAGAAGTGGCTTTTGGCCTAGTGTGTGCCACTTGT

GAGCAGATTGCAGATTCACAGCATCGGTCCCACAGACAGATGGCAACCATCACCAACCCA

TTAATCAGACATGAGAACAGAATGGTGCTGGCCAGCACTACAGCTAAGGCCATGGAGCAG

ATGGCAGGATCAAGCGAGCAGGCATCAGAAGCCATGGAGGTTGCTAATCAGGCCAGGCAG

ATGGTACAGGCAATGAGGACAATTGGGACTCATCCTAACTCTAGTGCTGGTCTGAGAGAT

AATCTTCTTGAAAATTTGCAGGCCTACCAGAACCGAATGGGAGTGCAGATGCAGCGATTC

AAGTGATCCTCTTGTTGTTGCCGCAAATATCATTGGGATCCTGCACTTGATATTGTGGAT

CCTTGATCGTCTTTTCTTCAAATGCATTTATCGTCGCCTTAAATACGGTTTGAAAATAGG

GCCTTCTACGGAAGGGGTACCTGAGTCTATGAGGGAAGAGTACCGGCAGGAACAGCAGAG

TGCTGTGGATGTTGACGATGGTCATTTTGTCAACATAGAATTGGAGTAA-----------

-------------------------------------------

>A_goose_Taiwan_TNO15_2015_EPI690747

---------------------------ATGAGTCTTCTAACCGAGGTCGAAACGTACGTT

CTCTCTATCATCCCGTCAGGCCCCCTCAAAGCCGAGATCGCGCAGAGACTTGAAGATGTC

TTTGCAGGGAAAAACACCGATCTCGAGGCTCTCATGGAGTGGCTAAAGACAAGACCAATC

CTGTCACCTCTGACTAAAGGGATTTTGGGATTTGTGTTCACGCTCACCGTGCCCAGTGAG

CGAGGACTGCAGCGTAGACGCTTCGTCCAGAATGCCCTAAATGGAAATGGGGATCCAAAT

AATATGGATAAGGCAGTTAAGCTATATAAGAAGCTGAAAAGAGAGATAACATTCCATGGG

GCTAAGGAGGTCGCACTTAGCTACTCAACCGGTGCACTTGCCAGCTGCATGGGTCTCATA

TACAACAGGATGGGAACGGTGACTACAGAAGTGGCTTTTGGCCTAGTGTGTGCCACTTGT

GAGCAGATTGCAGATTCACAGCATCGGTCCCACAGACAGATGGCAACCATCACCAACCCA

TTAATCAGACATGAGAACAGAATGGTGCTGGCCAGCACTACAGCTAAGGCCATGGAGCAG

ATGGCAGGATCAAGCGAGCAGGCATCAGAAGCCATGGAGGTTGCTAATCAGGCCAGGCAG

ATGGTACAGGCAATGAGGACAATTGGGACTCATCCTAATTCTAGTGCTGGTCTGAGAGAT

AATCTTCTTGAAAATTTGCAGGCCTACCAGAACCGAATGGGAGTGCAGATGCAGCGATTC

AAGTGATCCTCTTGTTGTTGCCGCAAATATCATTGGGATCCTGCACTTGATATTGTGGAT

CCTTGATCGTCTTTTCTTCAAATGCATTTATCGTCGCCTTAAATACGGTTTGAAAATAGG

GCCTTCTACGGAAGGGGTACCTGAGTCTATGAGGGAAGAGTACCGGCAGGAACAGCAGAG

TGCTGTGGATGTTGACGATGGTCATTTTGTCAACATAGAATTGGAGTAA-----------

-------------------------------------------

>A_wigeon_Sakha_1_2014_EPI1201482

--AGCAAAAGCAGGTAGATATTGAAAGATGAGTCTTCTAACCGAGGTCGAAACGTACGTT

CTCTCTATCATCCCGTCAGGCCCCCTCAAAGCCGAGATCGCGCAGAGACTTGAAGATGTC

TTTGCAGGGAAAAACACCGATCTCGAGGCTCTCATGGAGTGGCTAAAGACAAGACCAATC

CTGTCACCTCTGACTAAAGGGATTTTGGGATTTGTGTTCACGCTCACCGTGCCCAGTGAG

CGAGGACTGCAGCGTAGACGCTTCGTCCAGAATGCCCTAAATGGAAACGGGGATCCAAAT

AATATGGATAAGGCAGTTAAGCTATATAAGAAGCTGAAAAGAGAGATAACATTCCATGGG

GCTAAGGAGGTCGCACTTAGCTACTCAACCGGTGCACTTGCCAGCTGCATGGGTCTCATA

TACAACAGGATGGGAACGGTGACTACAGAAGTGGCTTTTGGCCTAGTGTGTGCCACTTGT

GAGCAGATTGCAGATTCACAGCATCGGTCCCACAGACAGATGGCAACCATCACCAACCCA

TTAATCAGACATGAGAACAGAATGGTGCTGGCCAGCACTACAGCTAAGGCCATGGAGCAG

ATGGCAGGATCAAGCGAGCAGGCATCAGAAGCCATGGAGGTTGCTAATCAGGCCAGGCAG

ATGGTACAGGCAATGAGGACAATTGGGACTCATCCTAACTCTAGTGCTGGTCTGAGAGAT

AATCTTCTTGAAAATTTGCAGGCCTACCAGAACCGAATGGGAGTGCAGATGCAGCGATTC

AAGTGATCCTCTTGTTGTTGCCGCAAATATCATTGGGATCCTGCACTTGATATTGTGGAT

CCTTGATCGTCTTTTCTTCAAATGCATTTATCGTCGCCTTAAATACGGGTTGAAAATAGG

GCCTTCTACGGAAGGGGTACCTGAGTCTATGAGGGAAGAGTACCGGCAGGAACAGCAGAG

TGCTGTGGATGTTGACGATGGTCATTTTGTCAACATAGAATTGGAGTAA-----------

-------------------------------------------

>A_duck_Nigeria_SK28T_19VIR8424-2_2019_EPI1777108

--AGCAAAAGCAGGTAGATATTGAAAGATGAGCCTTCTAACCGAGGTCGAAACGTACGTT

CTCTCTATTGTCCCGTCAGGCCCCCTCAAAGCCGAGATCGCGCAGAGACTTGAAGATGTC

TTTGTAGGGAAGAACACCGATCTTGAGGCTCTCATGGAATGGCTAAAGACAAGACCAATC

CTGTCACCTCTGACAAAGGGGATTTTAGGAGTTGTGTTCACGCTCACCGTGCCCAGTGAG

CGAGGACTGCAGCGTAGACGATTTGTCCAAAATGCCCTAAATGGAAATGGAGACCCAAAC

AATATAGACAGGGCAGTCAAACTGTACAGGAAATTAAAGAGAGAGATAACATTCCATGGG

GCTAAAGAAGTTGCACTCAGTTATTCAACCGGTGCACTTGCCTGTTGTATGGGTCTCATA

TACAACAGGATGGGGACGGTGACCACAGAAGTGGCGTTGGGCCTAGTATGTGCCACCTGT

GAGCAGATTGCTGATTCACAGCATCGGTCTCACAGACAAATAGCAACCACCACCAACCCA

CTAATCAGACATGAAAACAGAATGGTGCTGGCCAGTACTACAGCTAAGGCTATGGAGCAG

ATGGCTGGGTCGAGTGAGCAAGCAGCGGAAGCCATGGAGGTTGCTAGTCAGGCTAGGCAG

ATGGTGCAGGCGATGAGGACCATTGGAACTCACCCTAGCTCCAGTGCCGGTCTGAGAGAT

GATCTCCTTGAAAATTTGCAGGCCTACCAGAAACGGATGGGAGTGCAAATGCAGCGATTC

AAGTGATCCTCTCGTTATTGCCGCAAGTATCGTTGGGATCTTGCACTTGATATTGTGGAT

TCTTGATCGTCTTTTTCTCAAATGCGTTTATCGTCGCCTTAAATACGGTTTGAAAAGAGG

GCCTTCTACGGAAGGAGTGCCTGAGTCTATGAGGGAAGAGTATCGGCAGGAACAGCAGAG

TGCTGTGGATGTTGACTATGGTCATTTTGTCAACATAGAGCTGGAGTAAAAANCTACCTT

GTTTCTACT----------------------------------

>A_mute_swan_Kazakhstan_1-267-20-B_2020_EPI1811587

--------------------TTGAAAGATGAGTCTTCTAACCGAGGYCGAAACGTACGTT

CTCTCTATCGTCCCGTCAGGCCCCCTCAAAGCCGAGATCGCGCAGAGACTTGAAGATGTC

TTTGCAGGGAAGAACACCGATCTTGAGGCTCTCATGGAATGGCTAAAGACAAGACCAATC

CTGTCACCTCTGACTAAGGGGATTTTGGGATTTGTGTTCACGCTCACCGTGCCCAGTGAG

CGAGGACTGCAGCGTAGACGCTTTGTCCAAAATGCTCTAAATGGAAATGGAGACCCAAAC

AACATGGACAGGGCAGTCAAACTGTACAGGAAATTGAAGAGAGAGATAACATTCCATGGG

GCTAAAGAAGTTGCACTCAGTTACTCAACCGGCGCACTTGCCAGTTGTATGGGTCTCATA

TACAACAGGATGGGGACGGTGACCGCAGAAGTGGCATTGGGCCTAGTGTGTGCCACCTGT

GAGCAGATTGCTGATTCACAGCATCGGTCTCACAGACAGATAGCAACCACCACCAACCCA

CTAATCAGACATGAAAACAGAATGGTGTTGGCCAGTACTACAGCTAAGGCTATGGAGCAG

ATGGCTGGATCGAGTGAGCAAGCAGCGGAAGCCATGGAGGTTGCTAGTCAGGCTAGGCAG

ATGGTGCAGGCGATGAGGACCATTGGAACTCATCCTAGCTCCAGTGCCGGTCTGAGAGAT

GATCTCCTTGAAAATTTGCAGGCCTACCAAAAACGGATGGGAGTGCAACTGCAGCGATTC

AAGTGATCCTCTCGTTATTGCCGCAAGTATCATTGGGATCTTGCACTTGATATTGTGGAT

TCTTGATCGCCTTTTCTTCAAATGCGTTTATCGTCGCCTTAAATACGGTTTGAAAAGAGG

GCCTTCTACGGAAGGAGTACCTGAGTCCATGAGGGAAGAGTACCGGCAGGAACAGCAGAG

TGCTGTGGATGTTGACGATGGTCATTTTGTCAACATAGAGCTGGAGTAAAAAACTA----

-------------------------------------------

>A_domestic_goose_Kazakhstan_1-248_2-20-B_2020_EPI1811604

--AGCAAAAGCAGGTAGATATTGAAAGATGAGTCTTCTAACCGAGGTCGAAACGTACGTT

CTCTCTATCGTCCCGTCAGGCCCCCTCAAAGCCGAGATCGCGCAGAGACTTGAAGATGTC

TTTGCAGGGAAGAACACCGATCTTGAGGCTCTCATGGAATGGCTAAAGACAAGACCAATC

CTGTCACCTCTGACTAAGGGGATTTTGGGATTTGTGTTCACGCTCACCGTGCCCAGTGAG

CGAGGACTGCAGCGTAGACGCTTTGTCCAAAATGCTCTAAATGGAAATGGAGACCCAAAC

AACATGGACAGGGCAGTCAAACTGTACAGGAAATTGAAGAGAGAGATAACATTCCATGGG

GCTAAAGAAGTTGCACTCAGTTACTCAACCGGTGCACTTGCCAGTTGTATGGGTCTCATA

TACAACAGGATGGGGACGGTGACCGCAGAAGTGGCATTGGGCCTAGTGTGTGCCACCTGT

GAGCAGATTGCTGATTCACAGCATCGGTCTCACAGACAGATAGCAACCACCACCAACCCA

CTAATCAGACATGAAAACAGAATGGTGTTGGCCAGTACTACAGCTAAGGCTATGGAGCAG

ATGGCTGGATCGAGTGAGCAAGCAGCGGAAGCCATGGAGGTTGCTAGTCAGGCTAGGCAG

ATGGTGCAGGCGATGAGGACCATTGGAACTCATCCTAGCTCCAGTGCCGGTCTGAGAGAT

GATCTCCTTGAAAATTTGCAGGCCTACCAAAAACGGATGGGAGTGCAACTGCAGCGATTC

AAGTGATCCTCTCGTTATTGCCGCAAGTATCATTGGGATCTTGCACTTGATATTGTGGAT

TCTTGATCGCCTTTTCTTCAAATGCGTTTATCGTCGCCTTAAATACGGTTTGAAAAGAGG

GCCTTCTACGGAAGGAGTACCTGAGTCCATGAGGGAAGAGTACCGGCAGGAACAGCAGAG

TGCTGTGGATGTTGACGATGGTCATTTTGTCAACATAGAGCTGGAGTAAAAAA-------

-------------------------------------------

>A_domestic_duck_Kazakhstan_1-274-20-B_2020_EPI1811614

--AGCAAAAGCAGGTAGATATTGAAAGATGAGTCTTCTAACCGAGGTCGAAACGTACGTT

CTCTCTATCGTCCCGTCAGGCCCCCTCAAAGCCGAGATCGCGCAGAGACTTGAAGATGTC

TTTGCAGGGAAGAACACCGATCTTGAGGCTCTCATGGAATGGCTAAAGACAAGACCAATY

CTGTCACCTCTGACTAAGGGGATTTTGGGATTTGTGTTCACGCTCACCGTGCCCAGTGAG

CGAGGACTGCAGCGTAGACGCTTTGTCCAAAATGCTCTAAATGGAAATGGAGACCCAAAC

AACATGGACAGGGCAGTCAAACTGTACAGGAAATTGAAGAGAGAGATAACATTCCATGGG

GCTAAAGAAGTTGCACTCAGTTACTCAACCGGTGCACTTGCCAGTTGTATGGGTCTCATA

TACAACAGGATGGGGACGGTGACCGCAGAAGTGGCATTGGGCCTAGTGTGTGCCACCTGT

GAGCAGATTGCTGATTCACAGCATCGGTCTCACAGACAGATAGCAACCACCACCAACCCA

CTAATCAGACATGAAAACAGAATGGTGTTGGCCAGTACTACAGCTAAGGCTATGGAGCAG

ATGGCTGGATCGAGTGAGCAAGCAGCGGAAGCCATGGAGGTTGCCAGTCAGGCTAGGCAG

ATGGTGCAGGCGATGAGGGCCATTGGAACTCATCCTAGCTCCAGTGCCGGTCTGAGAGAT

GATCTCCTTGAAAATTTGCAGGCCTACCAAAAACGGATGGGAGTGCAACTGCAGCGATTC

AAGTGATCCTCTCGTTATTGCCGCAAGTATCATTGGGATCTTGCACTTGATATTGTGGAT

TCTTGATCGCCTTTTCTTCAAATGCGTTTATCGTCGCCTTAAATACGGTTTGAAAAGAGG

GCCTTCTACGGAAGGAGTACCTGAGTCCATGAGGGAAGAGTACCGGCAGGAACAGCAGAG

TGCTGTGGATGTTGACGATGGTCATTTTGTCAACATAGAGCTGGAGTAAAAAACTAC---

-------------------------------------------

>A_domestic_goose_Kazakhstan_1-242_2-20-B_2020_EPI1811622

--------------------------GATGAGTCTTCTAACCGAGGTCGAAACGTACGTT

CTCTCTATCGTCCCGTCAGGCCCCCTCAAAGCCGAGATCGCGCAGAGACTTGAAGATGTC

TTTGCAGGGAAGAACACCGATCTTGAGGCTCTCATGGAATGGCTAAAGACAAGACCAATC

CTGTCACCTCTGACTAAGGGGATTTTGGGATTTGTGTTCACGCTCACCGTGCCCAGTGAG

CGAGGACTGCAGCGTAGACGCTTTGTCCAAAATGTTCTAAGTGGAAATGGAGACCCAAAC

AACATGGACAGGGCAGTCAAACTGTACAGGAAATTGAAGAGAGAGATAACATTCCATGGG

GCTAAAGAAGTTGCACTCAGTTACTCAACCGGTGCACTTGCCAGTTGTATGGGTCTCATA

TACAACAGGATGGGGACGGTGACCGCAGAAGTGGCATTGGGCCTAGTGTGTGCCACCTGT

GAGCAGATTGCTGATTCACAGCATCGGTCTCACAGACAGATAGCAACCACCACCAACCCA

CTAATCAGACATGAAAACAGAATGGTGTTGGCCAGTACTACAGCTAAGGCTATGGAGCAG

ATGGCTGGATCGAGTGAGCAAGCAGCGGAAGCCATGGAGGTTGCCAGTCAGGCTAGGCAG

ATGGTGCAGGCGATGAGGACCATTGGAACTCATCCTAGCTCCAGTGCCGGTCTGAGAGAT

GATCTCCTTGAAAATTTGCAGGCCTACCAAAAACGGATGGGAGTGCAACTGCAGCGATTC

AAGTGATCCTCTCGTTATTGCCGCAAGTATCATTGGGATCTTGCACTTGATATTGTGGAT

TCTTGATCGCCTTTTCTTCAAATGCGTTTATCGTCGCCTTAAATACGGTTTTAAAAGAGG

GCCTTCTACGGAAGGAGTACCTGAGTCCATGAGGGAAGAGTACCGGCAGGAACAGCAGAG

TGCTGTGGATGTTGACGATGGTCATTTTGTCAACATAGAGCTGGAGTAAAAA-CTAC-TT

GTTTCTA------------------------------------

>A_chicken_Iraq_1_2020_EPI1811631

---GCAAAAGCAGGTAGATATTGAAAGATGAGTCTTCTAACCGAGGTCGAAACGTACGTT

CTCTCTATCGTCCCGTCAGGCCCCCTCAAAGCCGAGATCGCGCAGAGACTTGAAGATGTC

TTTGCAGGGAAGAACACCGATCTTGAGGCTCTCATGGAATGGCTAAAGACAAGACCAATC

CTGTCACCTCTGACTAAGGGGATTTTGGGATTTGTGTTTACGCTCACCGTGCCCAGTGAG

CGAGGACTGCAGCGTAGACGCTTTGTCCAAAATGCTCTAAATGGAAATGGAGACCCAAAC

AACATGGACAGGGCAGTCAAACTGTACAGGAAATTGAAGAGAGAGATAACATTCCATGGG

GCTAAAGAAGTTGCACTCAGTTACTCAACCGGTGCACTTGCCAGTTGTATGGGTCTCATA

TACAACAGGATGGGGACGGTGACCGCAGAAGTGGCATTGGGCCTAGTGTGTGCCACCTGT

GAGCAGATTGCTGATTCACAGCATCGGTCTCACAGACAGATAGCAACCACCACCAACCCA

CTAATCAGACATGAAAACAGAATGGTGTTGGCCAGTACTACAGCTAAGGCTATGGAGCAG

ATGGCTGGATCGAGTGAGCAAGCAGCGGAAGCCATGGAGGTTGCTAGTCAGGCTAGGCAG

ATGGTGCAGGCGATGAGGACCATTGGAACTCATCCTAGCTCCAGTGCCGGTCTGAGAGAT

GATCTCCTTGAAAATTTGCAGGCCTACCAAAAACGGATGGGAGTGCAACTGCAGCGATTC

AAGTGATCCTCTCGTTATTGCCGCAAGTATCATTGGGATCTTGCACTTGATATTGTGGAT

CCTTGATCGCCTTTTCTTCAAATGCGTTTATCGTCGCCTTAAATACGGTTTGAAAAGAGG

GCCTTCTACGGAAGGAGTACCTGAGTCCATGAGGGAAGAGTACCGGCAGGAACAGCAGAG

TGCTGTGGATGTTGACGATGGTCATTTTGTCAACATAGAGCTGGAGTAAAAAACTAC---

-------------------------------------------

>A_whooper_swan_Inner_Mongolia_w1-1_2020_EPI1811647

-GAAAAAAAAACAGTAGATATTGAAAGATGAGTCTTCTAACCGAGGTCGAAACGTACGTT

CTCTCTATCGTCCCGTCAGGCCCCCTCAAAGCCGAGATCGCGCAGAGACTTGAAGATGTC

TTTGCAGGGAAGAACACCGATCTTGAGGCTCTCATGGAATGGCTAAAGACAAGACCAATC

CTGTCACCTCTGACTAAGGGGATTTTGGGGTTTGTGTTCACGCTCACCGTGCCCAGTGAG

CGAGGACTGCAGCGTAGACGCTTTGTCCAAAATGCTCTAAATGGAAATGGAGACCCAAAC

AACATGGACAGGGCAGTCAAACTGTACAGGAAACTGAAGAGAGAGATAACATTCCATGGG

GCTAAAGAAGTTGCACTCAGTTACTCAACCGGTGCACTTGCCAGTTGTATGGGTCTCATA

TACAACAGGATGGGGACGGTGACCGCAGAAGTGGCATTGGGCCTAGTGTGTGCCACCTGT

GAGCAGATTGCTGATTCACAGCATCGGTCTCACAGACAGATAGCAACCACCACCAACCCA

CTAATCAGACATGAAAACAGAATGGTGTTGGCCAGTACTACAGCTAAGGCTATGGAGCAG

ATGGCTGGATCGAGTGAGCAAGCAGCGGAAGCCATGGAGGTTGCCAGTCAGGCTAGGCAG

ATGGTGCAGGCGATGAGGACCATTGGAACTCATCCTAGCTCCAGTGCCGGTCTGAGAGAT

GATCTCCTTGAAAATTTGCAGGCCTACCAAAAACGGATGGGAGTGCAACTGCAGCGATTC

AAGTGATCCTCTCGTTATTGCCGCAAGTATCATTGGGATCTTGCACTTGATATTGTGGAT

TCTTGATCGCCTTTTCTTCAAATGCGTTTATCGTCGCCTTAAATACGGTTTGAAAAGAGG

GCCTTCTACGGAAGGAGTACCTGAGTCCATGAGGGAAGAGTACCGGCAGGAACAGCAGAG

TGCTGTGGATGTTGACGATGGTCATTTTGTCAACATAGAGCTGGAGTAACAC--------

-------------------------------------------

>A_mute_swan_Inner_Mongolia_w2-1_2020_EPI1811655

ACGAAAAAGGACAGTAGATAT-GAAAGATGAGTCTTCTAACCGAGGTCGAAACGTACGTT

CTCTCTATCGTCCCGTCAGGCCCCCTCAAAGCCGAGATCGCGCAGAGACTTGAAGATGTC

TTTGCAGGGAAGAACACCGATCTTGAGGCTCTCATGGAATGGCTAAAGACAAGACCAATC

CTGTCACCTCTGACTAAGGGGATTTTGGGGTTTGTGTTCACGCTCACCGTGCCCAGTGAG

CGAGGACTGCAGCGTAGACGCTTTGTCCAAAATGCTCTAAATGGAAATGGAGACCCAAAC

AACATGGACAGGGCAGTCAAACTGTACAGGAAACTGAAGAGAGAGATAACATTCCATGGG

GCTAAAGAAGTTGCACTCAGTTACTCAACCGGTGCACTTGCCAGTTGTATGGGTCTCATA

TACAACAGGATGGGGACGGTGACCGCAGAAGTGGCATTGGGCCTAGTGTGTGCCACCTGT

GAGCAGATTGCTGATTCACAGCATCGGTCTCACAGACAGATAGCAACCACCACCAACCCA

CTAATCAGACATGAAAACAGAATGGTGTTGGCCAGTACTACAGCTAAGGCTATGGAGCAG

ATGGCTGGATCGAGTGAGCAAGCAGCGGAAGCCATGGAGGTTGCCAGTCAGGCTAGGCAG

ATGGTGCAGGCGATGAGGACCATTGGAACTCATCCTAGCTCCAGTGCCGGTCTGAGAGAT

GATCTCCTTGAAAATTTGCAGGCCTACCAAAAACGGATGGGAGTGCAACTGCAGCGATTC

AAGTGATCCTCTCGTTATTGCCGCAAGTATCATTGGGATCTTGCACTTGATATTGTGGAT

TCTTGATCGCCTTTTCTTCAAATGCGTTTATCGTCGCCTTAAATACGGTTTGAAAAGAGG

GCCTTCTACGGAAGGAGTACCTGAGTCCATGAGGGAAGAGTACCGGCAGGAACAGCAGAG

TGCTGTGGATGTTGACGATGGTCATTTTGTCAACATAGAGCTGGAGTAA-----------

-------------------------------------------

>A_goose_Russian_Federation_Kurgan_1345-25_2020_EPI1811683

--------------TAGATATTGAAAGATGAGTCTTCTAACCGAGGTCGAAACGTACGTT

CTCTCTATCGTCCCGTCAGGCCCCCTCAAAGCCGAGATCGCGCAGAGACTTGAAGATGTC

TTTGCAGGGAAGAACACCGATCTTGAGGCTCTCATGGAATGGCTAAAGACAAGACCAATC

CTGTCACCTCTGACTAAGGGGATTTTGGGATTTGTGTTCACGCTCACCGTGCCCAGTGAG

CGAGGACTGCAGCGTAGACGCTTTGTCCAAAATGCTCTAAATGGAAATGGAGACCCAAAC

AACATGGACAGGGCAGTCAAACTGTACAGGAAATTGAAGAGAGAGATAACATTCCATGGG

GCTAAAGAAGTTGCACTCAGTTACTCAACCGGTGCACTTGCCAGTTGTATGGGTCTCATA

TACAACAGGATGGGGACGGTGACCGCAGAAGTGGCATTGGGCCTAGTGTGTGCCACCTGT

GAGCAGATTGCTGATTCACAGCATCGGTCTCACAGACAGATAGCAACCACCACCAACCCA

CTAATCAGACATGAAAACAGAATGGTGTTGGCCAGTACTACAGCTAAGGCTATGGAGCAG

ATGGCTGGATCGAGTGAGCAAGCAGCGGAAGCCATGGAGGTTGCTAGTCAGGCTAGGCAG

ATGGTGCAGGCGATGAGGACCATTGGAACTCATCCTAGCTCCAGTGCCGGTCTGAGAGAT

GATCTCCTTGAAAATTTGCAGGCCTACCAAAAACGGATGGGAGTGCAACTGCAGCGATTC

AAGTGATCCTCTCGTTATTGCCGCAAGTATCATTGGGATCTTGCACTTGATATTGTGGAT

TCTTGATCGCCTTTTCTTCAAATGCGTTTATCGTCGCCTTAAATACGGTTTGAAAAGAGG

GCCTTCTACGGAAGGAGTACCTGAGTCCATGAGGGAAGAGTACCGGCAGGAACAGCAGAG

TGCTGTGGATGTTGACGATGGTCATTTTGTCAACATAGAGCTGGAGTAAAAAACTA----

-------------------------------------------

>A_duck_Chelyabinsk_1207-1_2020_EPI1812536

--------------TAGATATTGAAAGATGAGTCTTCTAACCGAGGTCGAAACGTACGTT

CTCTCTATCGTCCCGTCAGGCCCCCTCAAAGCCGAGATCGCGCAGAGACTTGAAGATGTC

TTTGCAGGGAAGAACACCGATCTTGAGGCTCTCATGGAATGGCTAAAGACAAGACCAATC

CTGTCACCTCTGACTAAGGGGATTTTGGGATTTGTGTTCACGCTCACCGTGCCCAGTGAG

CGAGGACTGCAGCGTAGACGCTTTGTCCAAAATGCTCTAAATGGAAATGGAGACCCGAAC

AACATGGACAGGGCAGTCAAACTGTACAGGAAATTGAAGAGAGAGATAACATTCCATGGG

GCTAAAGAAGTTGCACTCAGTTACTCAACCGGTGCACTTGCCAGTTGTATGGGTCTCATA

TACAACAGGATGGGGACGGTGACCGCAGAAGTGGCATTGGGCCTAGTGTGTGCCACCTGT

GAGCAGATTGCTGATTCACAGCATCGGTCTCACAGACAGATAGCAACCACCACCAACCCA

CTAATCAGACATGAAAACAGAATGGTGTTGGCCAGTACTACAGCTAAGGCTATGGAGCAG

ATGGCTGGATCGAGTGAGCAAGCAGCGGAAGCCATGGAGGTTGCTAGTCAGGCTAGGCAG

ATGGTGCAGGCGATGAGGACCATTGGAACTCATCCTAGCTCCAGTGCCGGTCTGAGAGAT

GATCTCCTTGAAAATTTGCAGGCCTACCAAAAACGGATGGGAGTGCAACTGCAGCGATTC

AAGTGATCCTCTCGTTATTGCCGCAAGTATCATTGGGATCTTGCACTTGATATTGTGGAT

TCTTGATCGCCTTTTCTTCAAATGCGTTTATCGTCGCCTTAAATACGGTTTGAAAAGAGG

GCCTTCTACGGAAGGAGTACCTGAGTCCATGAGGGAAGAGTACCGGCAGGAACAGCAGAG

TGCTGTGGATGTTGACGATGGTCATTTTGTCAACATAGAGCTGGAGTAAAAAACTA----

-------------------------------------------

>A_goose_Omsk_0002_2020_EPI1813116

--AGCAAAAGCAGGTAGATGTTGAAAGATGAGTCTTCTAACCGAGGTCGAAACGTACGTT

CTCTCTATCGTCCCGTCAGGCCCCCTCAAAGCCGAGATCGCGCAGAGACTTGAAGATGTC

TTTGCAGGGAAGAACACCGATCTTGAGGCTCTCATGGAATGGCTAAAGACAAGACCAATC

CTGTCACCTCTGACTAAGGGGATTTTGGGATTTGTGTTCACGCTCACCGTGCCCAGTGAG

CGAGGACTGCAGCGTAGACGCTTTGTCCAAAATGCTCTAAATGGAAATGGAGACCCAAAC

AACATGGACAGGGCAGTCAAACTGTACAGGAAATTGAAGAGAGAGATAACATTCCATGGG

GCTAAAGAAGTTGCACTCAGTTACTCAACCGGCGCACTTGCCAGTTGTATGGGTCTCATA

TACAACAGGATGGGGACGGTGACCGCAGAAGTGGCATTGGGCCTAGTGTGTGCCACCTGT

GAGCAGATTGCTGATTCACAGCATCGGTCTCACAGACAGATAGCAACCACCACCAACCCA

CTAATCAGACATGAAAACAGAATGGTGTTGGCCAGTACTACAGCTAAGGCTATGGAGCAG

ATGGCTGGATCGAGTGAGCAAGCAGCGGAAGCCATGGAGGTTGCTAGTCAGGCTAGGCAG

ATGGTGCAGGCGATGAGGACCATTGGAACTCACCCTAGCTCCAGTGCCGGTCTGAGAGAT

GATCTCCTTGAAAATTTGCAGGCCTACCAAAAACGGATGGGAGTGCAACTGCAGCGATTC

AAGTGATCCTCTCGTTATTGCCGCAAGTATCATTGGGATCTTGCACTTGATATTGTGGAT

TCTTGATCGCCTTTTCTTCAAATGCGTTTATCGTCGCCTTAAATACGGTTTGAAAAGAGG

GCCTTCTACGGAAGGAGTACCTGAGTCCATGAGGGAAGAGTACCGGCAGGAACAGCAGAG

TGCTGTGGATGTTGACGATGGTCATTTTGTCAACATAGAGCTGGAGTAAAAAACTACCTT

GTTTCTACT----------------------------------

>A_goose_Omsk_01171_2020_EPI1813196

--AGCAAAAGCAGGTAGATATTGAAAGATGAGTCTTCTAACCGAGGTCGAAACGTACGTT

CTCTCTATCGTCCCGTCAGGCCCCCTCAAAGCCGAGATCGCGCAGAGACTTGAAGATGTC

TTTGCAGGGAAGAACACCGATCTTGAGGCTCTCATGGAATGGCTAAAGACAAGACCAATC

CTGTCACCTCTGACTAAGGGGATTTTGGGATTTGTGTTCACGCTCACCGTGCCCAGTGAG

CGAGGACTGCAGCGTAGACGCTTTGTCCAAAATGCTCTAAATGGAAATGGAGACCCAAAC

AACATGGACAGGGCAGTCAAACTGTACAGGAAATTGAAGAGAGAGATAACATTCCATGGG

GCTAAAGAAGTTGCACTCAGTTACTCAACCGGTGCACTTGCCAGTTGTATGGGTCTCATA

TACAACAGGATGGGGACGGTGACCGCAGAAGTGGCATTGGGCCTAGTGTGTGCCACCTGT

GAGCAGATTGCTGATTCACAGCATCGGTCTCACAGACAGATAGCAACCACCACCAACCCA

CTAATCAGACATGAAAACAGAATGGTGTTGGCCAGTACTACAGCTAAGGCTATGGAGCAG

ATGGCTGGATCGAGTGAGCAAGCAGCGGAAGCCATGGAGGTTGCCAGTCAGGCTAGGCAG

ATGGTGCAGGCGATGAGGACCATTGGAACTCATCCTAGCTCCAGTGCCGGTCTGAGAGAT

GATCTCCTTGAAAATTTGCAGGCCTACCAAAAACGGATGGGAGTGCAACTGCAGCGATTC

AAGTGATCCTCTCGTTATTGCCGCAAGTATCATTGGGATCTTGCACTTGATATTGTGGAT

TCTTGATCGCCTTTTCTTCAAATGCGTTTATCGTCGCCTTAAATACGGTTTGAAAAGAGG

GCCTTCTACGGAAGGAGTACCTGAGTCCATGAGGGAAGAGTACCGGCAGGAACAGCAGAG

TGCTGTGGATGTTGACGATGGTCATTTTGTCAACATAGAGCTGGAGTAAAAAACTACCTT

GTTTCTACT----------------------------------

>A_duck_Omsk_0075_2020_EPI1813276

--AGCAAAAGCAGGTAGATATTGAAAGATGAGTCTTCTAACCGAGGTAGAAACGTACGTT

CTCTCTATCGTCCCGTCAGGCCCCCTCAAAGCCGAGATCGCGCAGAGACTTGAAGATGTC

TTTGCAGGGAAGAACACCGATCTTGAGGCTCTCATGGAATGGCTAAAGACAAGACCAATC

CTGTCACCTCTGACTAAGGGGATTTTGGGATTTGTGTTCACGCTCACCGTGCCCAGTGAG

CGAGGACTGCAGCGTAGACGCTTTGTCCAAAATGCTCTAAATGGGAATGGAGACCCAAAC

AACATGGACAGGGCAGTCAAACTGTACAGGAAATTGAAGAGAGAGATAACATTCCATGGG

GCTAAAGAAGTTGCACTCAGTTACTCAACCGGTGCACTTGCCAGTTGTATGGGTCTCATA

TACAACAGGATGGGGACGGTGACCGCAGAAGTGGCATTGGGCCTAGTGTGTGCCACCTGT

GAGCAGATTGCTGATTCACAGCACCGGTCTCACAGACAGATAGCAACCACCACCAACCCA

CTAATCAGACATGAAAACAGAATGGTGTTGGCCAGTACTACAGCTAAGGCTATGGAGCAG

ATGGCTGGATCGAGTGAGCAAGCAGCGGAAGCCATGGAGGTTGCTAGTCAGGCTAGGCAG

ATGGTGCAGGCGATGAGGACCATTGGAACTCATCCTAGCTCCAGTGCCGGTCTGAGAGAT

GATCTCCTTGAAAATTTGCAGGCCTACCAAAAACGGATGGGAGTGCAACTGCAGCGATTC

AAGTGATCCTCTCGTTATTGTCGCAAGTATCATTGGGATCTTGCACTTGATATTGTGGAT

TCTTGATCGCCTTTTCTTCAAATGCGTTTATCGTCGCCTTAAATACGGTTTGAAAAGAGG

GCCTTCTACGGAAGGAGTACCTGAGTCCATGAGGGAAGAGTACCGGCAGGAACAGCAGAG

TGCTGTGGATGTTGACGATGGTCATTTTGTCAACATAGAGCTGGAGTAAAAAACTACCTT

GTTTCTACT----------------------------------

>A_duck_Omsk_0004_2020_EPI1813332

--AGCAAAAGCAGGTAGATATTGAAAGATGAGTCTTCTAACCGAGGTCGAAACGTACGTT

CTCTCTATCGTCCCGTCAGGCCCCCTCAAAGCCGAGATCGCGCAGAGACTTGAAGATGTC

TTTGCAGGGAAGAACACCGATCTTGAGGCTCTCATGGAATGGCTAAAGACAAGACCAATC

CTGTCACCTCTGACTAAGGGGATTTTGGGATTTGTGTTCACGCTCACCGTGCCCAGTGAG

CGAGGACTGCAGCGTAGACGCTTTGTCCAAAATGCTCTAAATGGAAATGGAGACCCAAAC

AACATGGACAGGGCAGTCAAACTGTACAGGAAATTGAAGAGAGAGATAACATTCCATGGG

GCTAAAGAAGTTGCACTCAGTTACTCAACCGGTGCACTTGCCAGTTGCATGGGTCTCATA

TACAACAGGATGGGGACGGTGACCGCAGAAGTGGCATTGGGCCTAGTGTGTGCCACCTGT

GAGCAGATTGCTGATTCACAGCATCGGTCTCACAGACAGATAGCAACCACCACCAACCCA

CTAATCAGACATGAAAACAGAATGGTGTTGGCCAGTACTACAGCTAAGGCTATGGAGCAG

ATGGCTGGGTCGAGTGAGCAAGCAGCGGAAGCCATGGAGGTTGCTAGTCAGGCTAGGCAG

ATGGTGCAGGCGATGAGGACCATTGGAACTCATCCTAGCTCCAGTGCCGGTCTGAGAGAT

GATCTCCTTGAAAATTTGCAGGCCTACCAAAAACGGATGGGAGTGCAACTGCAGCGATTC

AAGTGATCCTCTCGTTATTGCCGCAAGTATCATTGGGATCTTGCACTTGATATTGTGGAT

TCTTGATCGCCTTTTCTTCAAATGCGTTTATCGTCGCCTTAAATACGGTTTGAAAAGAGG

GCCTTCTACGGAAGGAGTACCTGAGTCCATGAGGGAAGAGTACCGGCAGGAACAGCAGAG

CGCTGTGGATGTTGACGATGGTCATTTTGTCAACATAGAGCTGGAGTAAAAAACTACCTT

GTTTCTACT----------------------------------

>A_chicken_Omsk_0112_2020_EPI1813340

--AGCAAAAGCAGGTAGATATTGAAAGATGAGTCTTCTAACCGAGGTCGAAACGTACGTT

CTCTCTATCGTCCCGTCAGGCCCCCTCAAAGCCGAGATCGCGCAGAGACTTGAAGATGTC

TTTGCAGGGAAGAACACCGATCTTGAGGCTCTCATGGAATGGCTAAAGACAAGACCAATC

CTGTCACCTCTGACTAAGGGGATTTTGGGATTTGTGTTCACGCTCACCGTGCCCAGTGAG

CGAGGACTGCAGCGTAGACGCTTTGTCCAAAATGCTCTAAATGGAAATGGAGACCCAAAC

AACATGGACAGGGCAGTCAAACTGTACAGGAAATTGAAGAGAGAGATAACATTCCATGGG

GCTAAAGAAGTTGCACTCAGTTACTCAACCGGTGCACTTGCCAGTTGTATGGGTCTCATA

TACAACAGGATGGGGACGGTGACCGCAGAAGTGGCATTGGGCCTAGTGTGTGCCACCTGT

GAGCAGATTGCTGATTCACAGCATCGGTCTCACAGACAGATAGCAACCACCACCAACCCA

CTAATCAGACATGAAAACAGAATGGTGTTGGCCAGTACTACAGCTAAGGCTATGGAGCAG

ATGGCTGGATCGAGTGAGCAAGCAGCGGAAGCCATGGAGGTTGCCAGTCAGGCTAGGCAG

ATGGTGCAGGCGATGAGGACCATTGGAACTCATCCTAGCTCCAGTGCCGGTCTGAGAGAT

GATCTCCTTGAAAATTTGCAGGCCTACCAAAAACGGATGGGAGTGCAACTGCAGCGATTC

AAGTGATCCTCTCGTTATTGCCGCAAGTATCATTGGGATCTTGCACTTGATATTGTGGAT

TCTTGATCGCCTTTTCTTCAAATGCGTTTATCGTCGCCTTAAATACGGTTTGAAAAGAGG

GCCTTCTACGGAAGGAGTACCTGAGTCCATGAGGGAAGAGTACCGGCAGGAACAGCAGAG

TGCTGTGGATGTTGACGATGGTCATTTTGTCAACATAGAGCTGGAGTAAAAAACTACCTT

GTTTCTACT----------------------------------

>A_duck_Saratov_29804_2020_EPI1814260

--AGCAAAAGCAGGTAGATATTGAAAGATGAGTCTTCTAACCGAGGTCGAAACGTACGTT

CTCTCTATCGTCCCGTCAGGCCCCCTCAAAGCCGAGATCGCGCAGAGACTTGAAGATGTC

TTTGCAGGGAAGAACACCGATCTTGAGGCTCTCATGGAATGGCTAAAGACAAGACCAATC

CTGTCACCTCTGACTAAGGGGATTTTGGGATTTGTGTTCACGCTCACCGTGCCCAGTGAG

CGAGGACTGCAGCGTAGACGCTTTGTCCAAAATGCTCTAAATGGAAATGGAGACCCAAAC

AACATGGACAGGGCAGTCAAACTGTACAGGAAATTGAAGAGAGAGATAACATTCCATGGG

GCTAAAGAAGTTGCACTCAGTTACTCAACCGGTGCACTTGCCAGTTGTATGGGTCTCATA

TACAACAGGATGGGGACGGTGACCGCAGAAGTGGCATTGGGCCTAGTGTGTGCCACCTGT

GAGCAGATTGCTGATTCACAGCATCGGTCTCACAGACAGATAGCAACCACCACCAACCCA

CTAATCAGACATGAAAACAGAATGGTGTTGGCCAGTACTACAGCTAAGGCTATGGAGCAG

ATGGCTGGATCGAGCGAGCAAGCAGCGGAAGCCATGGAGGTTGCTAGTCAGGCTAGGCAG

ATGGTGCAGGCGATGAGGACCATTGGAACTCATCCTAGCTCCAGTGCCGGTCTGAGAGAT

GATCTCCTTGAAAATTTGCAGGCCTACCAAAAACGGATGGGAGTGCAACTGCAGCGATTC

AAGTGATCCTCTCGTTATTGCCGCAAGTATCATTGGGATCTTGCACTTGATATTGTGGAT

TCTTGATCGCCTTTTCTTCAAATGCGTTTATCGTCGCCTTAAATACGGTTTGAAAAGAGG

GCCTTCTACGGAAGGAGTACCTGAGTCCATGAGGGAAGAGTACCGGCAGGAACAGCAGAG

TGCTGTGGATGTTGACGATGGTCATTTTGTCAACATAGAGCTGGAGTAAAAAACTACCTT

GTTTCTACT----------------------------------

>A_goose_Omsk_30001_2020_EPI1814276

--AGCAAAAGCAGGTAGATATTGAAAGATGAGTCTTCTAACCGAGGTCGAAACGTACGTT

CTCTCTATCGTCCCGTCAGGCCCCCTCAAAGCCGAGATCGCGCAGAGACTTGAAGATGTC

TTTGCAGGGAAGAACACCGATCTTGAGGCTCTCATGGAATGGCTAAAGACAAGACCAATC

CTGTCACCTCTGACTAAGGGGATTTTGGGATTTGTGTTCACGCTCACCGTGCCCAGTGAG

CGAGGACTGCAGCGTAGACGCTTTGTCCAAAATGCTCTAAATGGAAATGGAGACCCAAAC

AACATGGACAGGGCAGTCAAACTGTACAGGAAATTGAAGAGAGAGATAACATTCCATGGA

GCTAAAGAAGTTGCACTCAGTTACTCAACCGGTGCACTTGCCAGTTGTATGGGTCTCATA

TACAACAGGATGGGGACGGTGACCGCAGAAGTGGCATTGGGCCTAGTGTGTGCCACCTGT

GAGCAGATTGCTGATTCACAGCATCGGTCTCACAGACAGATAGTAACCACCACCAACCCA

CTAATCAGACATGAAAACAGAATGGTGTTGGCCAGTACTACAGCTAAGGCTATGGAGCAG

ATGGCTGGATCGAGTGAGCAAGCAGCGGAAGCCATGGAGGTTGCTAGTCAGGCTAGGCAG

ATGGTGCAGGCGATGAGAACCATTGGAACTCATCCTAGCTCCAGTGCCGGTCTGAGAGAT

GATCTCCTTGAAAATTTGCAGGCCTACCAAAAACGGATGGGAGTGCAACTGCAGCGATTC

AAGTGATCCTCTCGTTATTGCCGCAAGTATCATTGGGATCTTGCACTTGATATTGTGGAT

TCTTGATCGCCTTTTCTTCAAATGCGTTTATCGTCGCCTTAAATACGGTTTGAAAAGAGG

GCCTTCTACGGAAGGAGTACCTGAGTCCTTGAGGGAAGAGTACCGGCAGGAACAGCAGAG

TGCTGTGGATGTTGACGATGGTCATTTTGTCAACATAGAGCTGGAGTAAAAAACTACCTT

GTTTCTACT----------------------------------

>A_goose_Omsk_30003_2020_EPI1814284

--AGCAAAAGCAGGTAGATATTGAAAGATGAGTCTTCTAACCGAGGTCGAAACGTACGTT

CTCTCTATCGTCCCGTCAGGCCCCCTCAAAGCCGAGATCGCGCAGAGACTTGAAGATGTC

TTTGCAGGGAAGAACACCGATCTTGAGGCTCTCATGGAATGGCTAAAGACAAGACCAATC

CTGTCACCTCTGACTAAGGGGATTTTGGGATTTGTGTTCACGCTCACCGTGCCCAGTGAG

CGAGGACTGCAGCGTAGACGCTTTGTCCAAAATGCTCTAAATGGAAATGGAGACCCAAAC

AACATGGACAGGGCAGTCAAACTGTACAGGAAATTGAAGAGAGAGATAACATTCCATGGG

GCTAAAGAAGTTGCACTCAGTTACTCAACCGGTGCACTTGCCAGTTGTATGGGTCTCATA

TACAACAGGATGGGGACGGTGACCGCAGAAGTGGCATTGGGCCTAGTGTGTGCCACCTGT

GAGCAGATTGCTGATTCACAGCATCGGTCTCACAGACAGATAGCAACCACCACCAACCCA

CTAATCAGACATGAAAACAGAATGGTGTTGGCCAGTACTACAGCTAAGGCTATGGAGCAG

ATGGCTGGATCGAGTGAGCAAGCAGCGGAAGCCATGGAGGTTGCTAGTCAGGCTAGGCAG

ATGGTGCAGGCGATGAGGACCATTGGAACTCATCCTAGCTCCAGTGCCGGTCTGAGAGAT

GATCTCCTTGAAAATTTGCAGGCCTACCAAAAACGGATGGGAGTGCAACTGCAGCGATTC

AAGTGATCCTCTCGTTATTGCCGCAAGTATCATTGGGATCTTGCACTTGATATTGTGGAT

TCTTGATCGCCTTTTCTTCAAATGCGTTTATCGTCGCCTTAAATACGGTTTGAAAAGAGG

GCCTTCTACGGAAGGAGTACCTGAGTCCATGAGGGAAGAGTACCGGCAGGAACAGCAGAG

TGCTGTGGATGTTGACGATGGTCATTTTGTCAACATAGAGCTGGAGTAAAAAACTACCTT

GTTTCTACT----------------------------------

>A_swan_Tumen_1479-2_2020_EPI1814688

--------------TAGATATTGAAAGATGAGTCTTCTAACCGAGGTCGAAACGTACGTT

CTCTCTATCGTCCCGTCAGGCCCCCTCAAAGCCGAGATCGCGCAGAGACTTGAAGATGTC

TTTGCAGGGAAGAACACCGATCTTGAGGCTCTCATGGAATGGCTAAAGACAAGACCAATC

CTGTCACCTCTGACTAAGGGGATTTTGGGATTTGTGTTCACGCTCACCGTGCCCAGTGAG

CGAGGACTGCAGCGTAGACGCTTTGTCCAAAATGCTCTAAATGGAAATGGAGACCCAAAC

AACATGGACAGGGCAGTCAAACTGTACAGGAAATTGAAGAGAGAGATAACATTCCATGGG

GCTAAAGAAGTTGCACTCAGTTACTCAACCGGTGCACTTGCCAGTTGTATGGGTCTCATA

TACAACAGGATGGGGACGGTGACCGCAGAAGTGGCATTGGGCCTAGTGTGTGCCACCTGT

GAGCAGATTGCTGATTCACAGCATCGGTCTCACAGACAGATAGCAACCACCACCAACCCA

CTAATCAGACATGAAAACAGAATGGTGTTGGCCAGTACTACAGCTAAGGCTATGGAGCAG

ATGGCTGGATCGAGTGAGCAAGCAGCGGAAGCCATGGAGGTTGCTAGTCAGGCTAGGCAG

ATGGTGCAGGCGATGAGGACCATTGGAACTCATCCTAGCTCCAGTGCCGGTCTGAGAGAT

GATCTCCTTGAAAATTTGCAGGCCTACCAAAAACGGATGGGAGTGCAACTGCAGCGATTC

AAGTGATCCTCTCGTTATTGCCGCAAGTATCATTGGAATCTTGCACTTGATATTGTGGAT

TCTTGATCGCCTTTTCTTCAAATGCGTTTATCGTCGCCTTAAATACGGTTTGAAAAGAGG

GCCTTCTACGGAAGGAGTACCTGAGTCCATGAGGGAAGAGTACCGGCAGGAACAGCAGAG

TGCTGTGGATGTTGACGATGGTCATTTTGTCAACATAGAGCTGGAGTAAAAAACTA----

-------------------------------------------

>A_Whooper_swan_Mongolia_24_2020_EPI1831865

--AGCGAAAGCAGGTAGATATTGAAAGATGAGTCTTCTAACCGAGGTCGAAACGTACGTT

CTCTCTATCATTCCATCAGGCCCCCTCAAAGCCGAGATCGCGCAGAAACTTGAGGATGTG

TTTGCAGGAAAGAACGCCGATCTCGAGGCTCTCATGGAGTGGCTAAAGACAAGACCAATC

CTGTCACCTCTGACTAAGGGAATTTTGGGATTTATATTCACGCTCACCGTGCCCAGTGAG

CGAGGACTGCAGCGTAGACGGTTTGTCCAGAATGCCCTAAATGGAAATGGAGATCCAAAT

AATATGGATAGGGCAGTTAAGCTATATAAGAAGCTGAAAAGAGAAATAACATTCCATGGA

GCTAAGGAGGTCGCACTCAGTTACTCAACTGGTGCACTTGCCAGTTGCATGGGTCTCATA

TACAACAGAATGGGAACAGTGACTACAGAAGTGGCTTTTGGTCTAGTGTGTGCCACTTGT

GAGCAGATTGCGGATTCACAGCATCGGTCTCACAGACAGATGGCAACCATCACCAACCCA

CTAATCAGACATGAGAACAGAATGGTGCTGGCCAGCACTACAGCTAAGGCTATGGAGCAG

ATGGCGGGATCAAGTGAGCAGGCAGCAGAAGCCATGGAGGTCGCCAATCAGGCTAGACAG

ATGGTGCAGGCAATGAGAACAATTGGGACTCATCCTAATTCTAGTACTGGTCTGAGAGAC

AATCTTCTTGAAAATTTGCAGGCCTACCAGAAACGGATGGGAGTGCAGATGCAGCGATTC

AAGTGATCCTCTTGTTGTTGCCGCAAGTATCATTGGGATCTTGCACTTGATATTGTGGAT

TCTTGATCGTCTTTTCTTCAAATGCATTTATCGTCGCTTTAAATACGGTTTGAAAAGAGG

GCCTTCTACGGAAGGAGTACCGGAGTCTATGAGGGAAGAGTACCGGCAGGAACAGCAAAA

TGCTGTAGATGTTGACGATGGTCATTTTGTCAACATAGAGTTGGAGTAAAAAACTACCTT

GTTTCTACT----------------------------------

>A_Whooper_swan_Mongolia_25_2020_EPI1831873

--AGCGAAAGCAGGTAGATATTGAAAGATGAGTCTTCTAACCGAGGTCGAAACGTACGTT

CTCTCTATCATTCCATCAGGCCCCCTCAAAGCCGAGATCGCGCAGAAACTTGAGGATGTG

TTTGCAGGAAAGAACGCCGATCTCGAGGCTCTCATGGAGTGGCTAAAGACAAGACCAATC

CTGTCACCTCTGACTAAGGGAATTTTGGGATTTATATTCACGCTCACCGTGCCCAGTGAG

CGAGGACTGCAGCGTAGACGGTTTGTCCAGAATGCCCTAAATGGAAATGGAGATCCAAAT

AATATGGATAGGGCAGTTAAGCTATATAAGAAGCTGAAAAGAGAAATAACATTCCATGGA

GCTAAGGAGGTCGCACTCAGTTACTCAACTGGTGCACTTGCCAGTTGCATGGGTCTCATA

TACAACAGAATGGGAACAGTGACTACAGAAGTGGCTTTTGGTCTAGTGTGTGCCACTTGT

GAGCAGATTGCGGATTCACAGCATCGGTCTCACAGACAGATGGCAACCATCACCAACCCA

CTAATCAGACATGAGAACAGAATGGTGCTGGCCAGCACTACAGCTAAGGCTATGGAGCAG

ATGGCGGGATCAAGTGAGCAGGCAGCAGAAGCCATGGAGGTCGCCAATCAGGCTAGACAG

ATGGTGCAGGCAATGAGAACAATTGGGACTCATCCTAATTCTAGTACTGGTCTGAGAGAC

AATCTTCTTGAAAATTTGCAGGCCTACCAGAAACGGATGGGAGTGCAGATGCAGCGATTC

AAGTGATCCTCTTGTTGTTGCCGCAAGTATCATTGGGATCTTGCACTTGATATTGTGGAT

TCTTGATCGTCTTTTCTTCAAATGCATTTATCGTCGCTTTAAATACGGTTTGAAAAGAGG

GCCTTCTACGGAAGGAGTACCGGAGTCTATGAGGGAAGAGTACCGGCAGGAACAGCAAAA

TGCTGTAGATGTTGACGATGGTCATTTTGTCAACATAGAGTTGGAGTAAAAAACTACCTT

GTTTCTACT----------------------------------

>A_chicken_Kazakhstan_Kn-3_2020_EPI1839256

---------------------------ATGAGTCTTCTAACCGAGGTCGAAACGTACGTT

CTCTCTATCGTCCCGTCAGGCCCCCTCAAAGCCGAGATCGCGCAGAGACTTGAAGATGTC

TTTGCAGGGAAGAACACCGATCTTGAGGCTCTCATGGAATGGCTAAAGACAAGACCAATC

CTGTCACCTCTGACTAAGGGGATTTTGGGATTTGTGTTCACGCTCACCGTGCCCAGTGAG

CGAGGACTGCAGCGTAGACGCTTTGTCCAAAATGCTCTAAATGGAAATGGAGACCCAAAC

AACATGGACAGGGCAGTCAAACTGTACAGGAAATTGAAGAGAGAGATAACATTCCATGGG

GCTAAAGAAGTTGCACTCAGTTACTCAACCGGTGCACTTGCCAGTTGTATGGGTCTCATA

TACAACAGGATGGGGACGGTGACCGCAGAAGTGGCATTGGGCCTAGTGTGTGCCACCTGT

GAGCAGATTGCTGATTCACAGCATCGGTCTCACAGACAGATAGCAACCACCACCAACCCA

CTAATCAGACATGAAAACAGAATGGTGTTGGCCAGTACTACAGCTAAGGCTATGGAGCAG

ATGGCTGGATCGAGTGAGCAAGCAGCGGAAGCCATGGAGGTTGCTAGTCAAGCTAGGCAG

ATGGTGCAGGCGATGAGGACCATTGGAACTCATCCTAGCTCCAGTGCCGGTCTGAGAGAT

GATCTCCTTGAAAATTTGCAGGCCTACCAAAAACGGATGGGAGTGCAACTGCAGCGATTC

AAGTGATCCTCTCGTTATTGCCGCAAGTATCATTGGGATCTTGCACTTGATATTGTGGAT

TCTTGATCGCCTTTTCTTCAAATGCGTTTATCGTCGCCTTAAATACGGTTTGAAAAGAGG

GCCTTCTACGGAAGGAGTACCTGAGTCCATGAGGGAAGAGTACCGGCAGGAACAGCAGAG

TGCTGTGGATGTTGACGATGGTCATTTTGTCAACATAGAGCTGGAG--------------

-------------------------------------------

>A_chicken_Kazakhstan_Kn-6_2020_EPI1839264

---------------------------ATGAGTCTTCTAACCGAGGTCGAAACGTACGTT

CTCTCTATCGTCCCGTCAGGCCCCCTCAAAGCCGAGATCGCGCAGAGACTTGAAGATGTC

TTTGCAGGGAAGAACACCGATCTTGAGGCTCTCATGGAATGGCTAAAGACAAGACCAATC

CTGTCACCTCTGACTAAGGGGATTTTGGGATTTGTGTTCACGCTCACCGTGCCCAGTGAG

CGAGGACTGCAGCGTAGACGCTTTGTCCAAAATGCTCTAAATGGAAATGGAGACCCAAAC

AACATGGACAGGGCAGTCAAACTGTACAGGAAATTGAAGAGAGAGATAACATTCCATGGG

GCTAAAGAAGTTGCACTCAGTTACTCAACCGGTGCACTTGCCAGTTGTATGGGTCTCATA

TACAACAGGATGGGGACGGTGACCGCAGAAGTGGCATTGGGCCTAGTGTGTGCCACCTGT

GAGCAGATTGCTGATTCACAGCATCGGTCTCACAGACAGATAGCAACCACCACCAACCCA

CTAATCAGACATGAAAACAGAATGGTGTTGGCCAGTACTACAGCTAAGGCTATGGAGCAG

ATGGCTGGATCGAGTGAGCAAGCAGCGGAAGCCATGGAGGTTGCTAGTCAAGCTAGGCAG

ATGGTGCAGGCGATGAGGACCATTGGAACTCATCCTAGCTCCAGTGCCGGTCTGAGAGAT

GATCTCCTTGAAAATTTGCAGGCCTACCAAAAACGGATGGGAGTGCAACTGCAGCGATTC

AAGTGATCCTCTCGTTATTGCCGCAAGTATCATTGGGATCTTGCACTTGATATTGTGGAT

TCTTGATCGCCTTTTCTTCAAATGCGTTTATCGTCGCCTTAAATACGGTTTGAAAAGAGG

GCCTTCTACGGAAGGAGTACCTGAGTCCATGAGGGAAGAGTACCGGCAGGAACAGCAGAG

TGCTGTGGATGTTGACGATGGTCATTTTGTCAACATAGAGCTGGAG--------------

-------------------------------------------

>A_Muscovy_duck_China_FJFZ21_H5N6_2020_EPI1841920

---------------------------ATGAGTCTTCTAACCGAGGTCGAAACGTACGTT

CTCTCTATCATCCCATCAGGCCCCCTCAAAGCCGAGATCGCGCAGAAACTTGAGGATGTG

TTTGCAGGAAAGAACGCTGATCTCGAGGCTCTCATGGAGTGGCTAAAGACAAGACCAATC

CTGTCACCTCTGACTAAAGGAATTTTGGGATTTGTATTCACGCTCACCGTGCCCAGTGAG

CGAGGACTGCAGCGTAGACGGTTTGTCCAGAATGCCCTAAATGGAAATGGAGATCCAAAT

AATATGGATAGGGCAGTTAAGCTATATAAGAAGCTGAAAAGAGAAATAACATTCCATGGA

GCTAAGGAGGTCGCACTCAGTTACTCAACTGGTGCACTTGCCAGTTGCATGGGTCTCATA

TACAACAGAATGGGAACAGTGACTACAGAAGTGGCTTTTGGCCTAGTGTGTGCCACTTGT

GAGCAGATTGCGGATTCACAGCATCGGTCTCACAGACAGATGGCAACCATCACCAATCCA

CTAATCAGACATGAGAACAGAATGGTGCTGGCCAGCACTACAGCTAAGGCTATGGAGCAG

ATGGCGGGATCAAGTGAGCAGGCAGCAGAAGCCATGGAGGTCGCCAATCAGGCTAGACAG

ATGGTGCAGGCAATGAGAACAATTGGGACTCATCCTAATTCTAGTACTGGTCTGAGAGAC

AATCTTCTTGAAAATTTGCAGGCCTACCAGAAACGGATGGGAGTGCAGATGCAGCGATTC

AAGTGATCCTCTTATTGTTGCCTCAAGTATCATTGGGATCTTGCACTTGATATTGTGGAT

TCTTGATCGTCTTTTCTTCAAATGCATTTATCGTCGCCTTAAATACGGTTTGAAAAGAGG

GCCTTCTACGGAAGGAGTACCGGAGTCTATGAGGGAAGAGTACCGGCAGGAACAGCAGAA

TGCTGTAGATGTTGACGATGGTCATTTTGTCAACATAGAGTTGGAGTAA-----------

-------------------------------------------

>A_mute_swan_Czech_Republic_1410-2_2021_EPI1843609

--------------------------GATGAGTCTTCTAACCGAGGTCGAAACGTACGTT

CTCTCTATCGTCCCGTCAGGCCCCCTCAAAGCCGAGATCGCGCAGAGACTTGAAGATGTC

TTTGCAGGGAAGAACACCGATCTTGAGGCTCTCATGGAATGGCTAAAGACAAGACCAATC

CTGTCACCTCTGACTAAGGGGATTTTGGGATTTGTGTTCACGCTCACCGTGCCCAGTGAG

CGAGGACTGCAGCGTAGACGCTTTGTCCAAAATGCTCTAAATGGAAATGGAGACCCAAAC

AACATGGACAGGGCAGTCAAACTGTACAGGAAATTGAAGAGAGAGATAACATTCCATGGG

GCTAAAGAAGTTGCACTCAGTTATTCAACCGGTGCACTTGCCAGTTGTATGGGTCTCATA

TACAACAGGATGGGGACGGTGACCGCAGAAGTGGCATTGGGCCTAGTGTGTGCCACCTGT

GAGCAGATTGCTGATTCACAGCATCGGTCTCACAGACAGATAGCAACCACCACCAACCCA

CTAATCAGACATGAAAACAGAATGGTGTTGGCCAGTACTACAGCTAAGGCTATGGAGCAG

ATGGCTGGATCGAGTGAACAGGCAGCGGAAGCCATGGAGGTTGCTAGTCAGGCTAGGCAG

ATGGTGCAGGCAATGAGGACCATTGGAACTCATCCTAGCTCCAGTGCCGGTCTGAGAGAT

GATCTCCTTGAAAATTTGCAGGCCTACCAAAAACGGATGGGAGTGCAACTGCAGCGATTC

AAGTGATCCTCTCGTTATTGCCGCAAGTATCATTGGGATCTTGCACTTGATATTGTGGAT

TCTTGATCGCCTTTTCTTCAAATGCGTTTATCGTCGCCTTAAATACGGTTTGAAAAGAGG

GCCTTCTACGGAAGGGGTACCTGAGTCCATGAGGGAAGAGTACCGGCAGGAACAGCAGAG

TGCTGTGGATGTTGACGATGGTCATTTTGTCAACATAGAGCTGGAGTA------------

-------------------------------------------

>A_chicken_Czech_Republic_1566-1_2021_EPI1844086

--------------------------GATGAGCCTTCTAACCGAGGTCGAAACGTACGTT

CTCTCTATCGTCCCGTCAGGCCCCCTCAAAGCCGAGATCGCGCAGAGACTTGAAGATGTC

TTTGTAGGGAAGAACACCGATCTTGAGGCTCTCATGGAATGGCTAAAGACAAGACCAATC

CTGTCACCTCTGACTAAGGGGATTTTAGGATTTGTGTTCACGCTCACCGTGCCCAGTGAG

CGAGGACTGCAGCGTAGACGCTTTGTCCAAAATGCTCTAAATGGAAATGGAGACCCAAAC

AACATGGACAGGGCAGTCAAACTGTACAGGAAATTGAAGAGAGAGATAACATTCCATGGG

GCTAAAGAAGTTGCACTCAGTTACTCAACCGGTGCACTTGCCAGTTGTATGGGTCTCATA

TACAACAGGATGGGGACGGTTACCGCAGAAGTGGCATTGGGCCTAGTGTGTGCCACCTGT

GAGCAGATTGCTGATTCACAGCATCGGTCTCACAGACAAATAGCAACCACCACAAACCCA

CTAATCAGACATGAAAACAGAATGGTATTGGCCAGTACTACAGCTAAGGCTATGGAGCAG

ATGGCTGGATCGAGTGAGCAAGCAGCGGAAGCCATGGAGGTTGCCAGTCAGGCTAGGCAG

ATGGTGCAGGCGATGAGGACCATTGGAACTCATCCTAGCTCCAGTGCCGGTCTGAGAGAT

GATCTCCTTGAAAATTTGCAGGCCTACCAAAAACGGATGGGAGTGCAACTGCAGCGATTC

AAGTGATCCTCTCGTTATTGCCGCAAGTATCATTGGGATCTTGCACTTGATATTGTGGAT

TCTTGATCGCCTTTTCTTCAAATGCGTTTATCGTCGCCTTAAATACGGTTTGAAAAGAGG

GCCTTCTACGGAAGGAGTACCTGAGTCCATGAGGGAAGAGTACCGGCAGAAACAGCAGAG

TGCTGTGGATGTTGACGATGGTCATTTTGTCAACATAGAGCTGGAGTA------------

-------------------------------------------

>A_chicken_Korea_H008_2021_EPI1846533

---------------------------ATGAGTCTTCTAACCGAGGTCGAAACGTACGTT

CTCTCTATCGTCCCGTCAGGCCCCCTCAAAGCCGAGATCGCGCAGAGACTTGAAGATGTC

TTTGCAGGGAAGAACACCGATCTTGAGGCTCTCATGGAATGGCTAAAGACAAGACCAATC

CTGTCACCTCTGACTAAGGGGATTTTGGGATTTGTGTTCACGCTCACCGTGCCCAGTGAG

CGAGGACTGCAGCGTAGACGCTTTGTCCAAAATGCTCTAAATGGAAATGGGGACCCAAAC

AACATGGACAGGGCAGTCAAACTGTACAGGAAACTGAAGAGAGAGATAACATTCCATGGG

GCTAAAGAAGTTGCACTCAGTTACTCAACCGGTGCACTTGCCAGTTGTATGGGTCTCATA

TACAACAGGATGGGGACGGTGACCGCAGAAGTGGCATTGGGCCTAGTGTGTGCCACCTGT

GAGCAGATTGCTGATTCACAGCATCGGTCTCACAGACAGATAGCAACCACCACCAACCCA

CTAATCAGACATGAAAACAGAATGGTGTTGGCCAGTACTACAGCTAAGGCTATGGAGCAG

ATGGCTGGATCGAGTGAGCAAGCAGCGGAAGCCATGGAGGTTGCCAGTCAGGCTAGGCAG

ATGGTGCAGGCGATGAGGACCATTGGAACTCATCCTAGCTCCAGTGCCGGTCTGAGAGAT

GATCTCCTTGAAAATTTGCAGGCCTACCAAAAACGGATGGGAGTGCAACTGCAGCGATTC

AAGTGATCCTCTCGTTATTGCCGCAAGTATCATTGGGATCTTGCACTTGATATTGTGGAT

TCTTGATCGCTTTTTCTTCAAATGCGTTTATCGTCGCCTTAAATACGGTTTGAAAAGAGG

GCCTTCTACGGAAGGAGTACCTGAGTCCATGAGGGAAGAGTACCGGCAGGAACAGCAGAG

TGCTGTGGATGTTGACGATGGTCATTTTGTCAACATAGAGCTGGAGTAA-----------

-------------------------------------------

>A_duck_Korea_H016_2021_EPI1846701

---------------------------ATGAGCCTTCTAACCGAGGTCGAAACGTACGTT

CTCTCTATCGTCCCGTCAGGCCCCCTCAAAGCCGAGATCGCGCAGAGACTTGAAGATGTC

TTTGCAGGGAAGAACACCGATCTTGAGGCTCTCATGGAATGGCTAAAGACAAGACCAATC

CTGTCACCTCTGACTAAGGGGATTTTGGGATTTGTGTTCACGCTCACCGTGCCCAGTGAG

CGAGGACTGCAGCGTAGACGCTTTGTCCAAAATGCTCTAAATGGAAATGGAGACCCAAAC

AACATGGACAGGGCAGTCAAACTGTACAGGAAACTGAAGAGAGAGATAACATTCCATGGG

GCTAAAGAAGTTGCACTCAGTTACTCAACCGGTGCACTTGCCAGTTGTATGGGTCTCATA

TACAACAGGATGGGGACGGTGACCGCAGAAGTGGCATTGGGCCTAGTGTGTGCCACCTGT

GAGCAGATTGCTGATTCACAGCATCGGTCTCACAGACAGATAGCAACCACCACCAACCCA

CTAATCAGACATGAAAACAGAATGGTGTTGGCAAGTACTACAGCTAAGGCTATGGAGCAG

ATGGCTGGATCGAGTGAGCAAGCAGCGGAAGCCATGGAGGTTGCCAGTCAGGCTAGGCAG

ATGGTGCAGGCGATGAGGACCATTGGAACTCATCCTAGCTCCAGTGCCGGTCTGAGAGAT

GATCTCCTTGAAAATTTGCAGGCCTACCAAAAACGGATGGGAGTGCAACTGCAGCGATTC

AAGTGATCCTCTCGTTATTGCCGCAAGTATCATTGGGATCTTGCACTTGATATTGTGGAT

TCTTGATCGCCTTTTCTTCAAATGCGTTTATCGTCGCCTTAAATACGGTTTGAAAAGAGG

GCCTTCTACGGAAGGAGTACCTGAGTCCATGAGGGAAGAGTACCGGCAGGAACAGCAGAG

TGCTGTGGATGTTGACGATGGTCATTTTGTCAACATAGAGCTGGAGTAA-----------

-------------------------------------------

>A_chicken_Astrakhan_321-01_2020_EPI1846972

--AGCAAAAGCAGGTAGATATTGAAAGATGAGTCTTCTAACCGAGGTCGAAACGTACGTT

CTCTCTATCGTCCCGTCAGGCCCCCTCAAAGCCGAGATCGCGCAGAGACTTGAAGATGTC

TTTGCAGGGAAGAACACCGATCTTGAGGCTCTCATGGAATGGCTAAAGACAAGACCAATC

CTGTCACCTCTGACTAAGGGGATTTTGGGATTTGTGTTCACGCTCACCGTGCCCAGTGAG

CGAGGACTGCAGCGTAGACGCTTTGTCCAAAATGCTCTAAATGGAAATGGAGACCCAAAC

AACATGGACAGGGCAGTCAAACTGTACAGGAAATTGAAGAGAGAGATAACATTCCATGGG

GCTAAAGAAGTTGCACTCAGTTACTCAACCGGTGCACTTGCCAGTTGTATGGGTCTCATA

TACAACAGGATGGGGACGGTGACCGCAGAAGTGGCATTGGGCCTAGTGTGTGCCACCTGT

GAGCAGATTGCTGATTCACAGCATCGGTCTCACAGACAGATAGCAACCACCACCAACCCA

CTAATCAGACATGAAAACAGAATGGTGTTGGCCAGTACTACAGCTAAGGCTATGGAGCAG

ATGGCTGGATCGAGTGAGCAAGCAGCGGAAGCCATGGAGGTTGCCAGTCAGGCTAGGCAG

ATGGTGCAGGCGATGAGGACCATTGGAACTCATCCTAGCTCCAGTGCCGGTCTGAGAGAT

GATCTCCTTGAAAATTTGCAGGCCTACCAAAAACGGATGGGAGTGCAACTGCAGCGATTC

AAGTGATCCTCTCGTTATTGCCGCAAGTATCATTGGGATCTTGCACTTGATATTGTGGAT

TCTTGATCGCCTTTTCTTCAAATGCGTTTATCGTCGCCTTAAATACGGTTTGAAAAGAGG

GCCTTCTACGGAAGGAGTACCTGAGTCCATGAGGGAAGAGTACCGGCAGGAACAGCAGAG

TGCTGTGGATGTTGACGATGGTCATTTTGTCAACATAGAGCTGGAGTAAAAAACTACCTT

GTTTCTACT----------------------------------

>A_crane_Kagoshima_KU-93_2021_EPI1848530

---------------------------ATGAGTCTTCTAACCGAGGTCGAAACGTACGTT

CTCTCTATCGTCCCGTCAGGCCCCCTCAAAGCCGAGATCGCGCAGAGACTTGAAGATGTC

TTTGCAGGGAAGAACACCGATCTTGAGGCTCTCATGGAATGGCTAAAGACAAGACCAATC

CTGTCACCTCTGACTAAGGGGATTTTGGGGTTTGTGTTCACGCTCACCGTGCCCAGTGAG

CGAGGACTGCAGCGTAGACGCTTTGTCCAAAATGCTCTAAATGGAAATGGAGACCCAAAC

AACATGGACAGGGCAGTCAAACTGTACAGGAAACTGAAGAGAGAGATAACATTCCATGGG

GCTAAAGAAGTTGCACTCAGTTACTCAACCGGTGCACTTGCCAGTTGTATGGGTCTCATA

TACAACAGGATGGGGACGGTGACCGCAGAAGTGGCATTGGGCCTAGTGTGTGCCACCTGT

GAGCAGATTGCTGATTCACAGCATCGGTCTCACAGACAGATAGCAACCACCACCAACCCA

CTAATCAGACATGAAAACAGAATGGTGTTGGCCAGTACTACAGCTAAGGCTATGGAGCAG

ATGGCTGGATCGAGTGAGCAAGCAGCGGAAGCCATGGAGGTTGCCAGTCAGGCTAGGCAG

ATGGTGCAGGCGATGAGGACCATTGGAACTCATCCTAGCTCCAGTGCCGGTCTGAGAGAT

GATCTCCTTGAAAATTTGCAGGCCTACCAAAAACGGATGGGAGTGCAACTGCAGCGATTC

AAGTGATCCTCTCGTTATTGCCGCAAGTATCATTGGGATCTTGCACTTGATATTGTGGAT

TCTTGATCGCCTTTTCTTCAAATGCGTTTATCGTCGCCTTAAATACGGTTTGAAAAGAGG

GCCTTCTACGGAAGGAGTACCTGAGTCCATGAGGGAAGAGTACCGGCAGGAACAGCAGAG

TGCTGTGGATGTTGACGATGGTCATTTTGTCAACATAGAGCTGGAGTAA-----------

-------------------------------------------

>A_mallard_Kagoshima_KU-d89_2021_EPI1848541

---------------------------ATGAGTCTTCTAACCGAGGTCGAAACGTACGTT

CTCTCTATCGTCCCGTCAGGCCCCCTCAAAGCCGAGATCGCGCAGAGACTTGAAGATGTC

TTTGCAGGGAAGAACACCGATCTTGAGGCTCTCATGGAATGGCTAAAGACAAGACCAATC

CTGTCACCTCTGACTAAGGGGATTTTGGGATTTGTGTTCACGCTCACCGTGCCCAGTGAG

CGAGGACTGCAGCGTAGACGCTTTGTCCAAAATGCTCTAAATGGAAATGGAGACCCAAAC

AACATGGACAGGGCAGTCAAACTGTACAGGAAACTGAAGAGAGAGATAACATTCCATGGG

GCTAAAGAAGTTGCACTCAGTTACTCAACCGGTGCACTTGCCAGTTGTATGGGTCTCATA

TACAACAGGATGGGGACGGTGACCGCAGAAGTGGCATTGGGCCTAGTGTGTGCCACCTGT

GAGCAGATTGCTGATTCACAGCATCGGTCTCACAGACAGATAGCAACCACCACCAACCCA

CTAATCAGACATGAAAACAGAATGGTGTTGGCCAGTACTACAGCTAAGGCTATGGAGCAG

ATGGCTGGATCGAGTGAGCAAGCAGCGGAAGCCATGGAGGTTGCCAGTCAGGCTAGGCAG

ATGGTGCAGGCGATGAGGACCATTGGAACTCATCCTAGCTCCAGTGCCGGTCTGAGAGAT

GATCTCCTTGAAAATTTGCAGGCCTACCAAAAACGGATGGGAGTGCAACTGCAGCGATTC

AAGTGATCCTCTCGTTATTGCCGCAAGTATCATTGGGATCTTGCACTTGATATTGTGGAT

TCTTGATCGCCTTTTCTTCAAATGCGTTTATCGTCGCCTTAAATACGGTTTGAAAAGAGG

GCCTTCTACGGAAGGAGTACCTGAGTCCATGAGGGAAGAGTACCGGCAGGAACAGCAGAG

TGCTGTGGATGTTGACGATGGTCATTTTGTCAACATAGAGCTGGAGTAA-----------

-------------------------------------------

>A_chicken_Kostroma_304-06_2020_EPI1848641

--AGCAAAAGCAGGTAGATATTGAAAGATGAGTCTTCTAACCGAGGTCGAAACGTACGTT

CTCTCTATCGTCCCGTCAGGCCCCCTCAAAGCCGAGATCGCGCAGAGACTTGAAGATGTC

TTTGCAGGGAAGAACACCGATCTTGAGGCTCTCATGGAATGGCTAAAGACAAGACCAATC

CTGTCACCTCTGACTAAGGGGATTTTGGGATTTGTGTTCACGCTCACCGTGCCCAGTGAG

CGAGGACTGCAGCGTAGACGCTTTGTCCAAAATGCTCTAAATGGAAATGGAGACCCAAAC

AACATGGACAGGGCAGTCAAACTGTACAGGAAACTGAAGAGAGAGATAACATTCCATGGG

GCTAAAGAAGTTGCACTCAGTTACTCAACCGGTGCACTTGCCAGTTGTATGGGTCTCATA

TACAACAGGATGGGGACGGTGACCGCAGAAGTGGCATTGGGCCTAGTGTGTGCCACCTGT

GAGCAGATTGCTGATTCACAGCATCGGTCTCACAGACAGATAGCAACCACCACCAACCCA

CTAATCAGACATGAAAACAGAATGGTGTTGGCCAGTACTACAGCTAAGGCTATGGAGCAG

ATGGCTGGATCGAGTGAGCAAGCAGCGGAAGCCATGGAGGTTGCCAGTCAGGCTAGGCAG

ATGGTGCAGGCGATGAGGACCATTGGAACTCATCCTAGCTCCAGTGCCGGTCTGAGAGAT

GATCTCCTTGAAAATTTGCAGGCCTACCAAAAACGGATGGGAGTGCAACTGCAGCGATTC

AAGTGATCCTCTCGTTATTGCCGCAAGTATCATTGGGATCTTGCACTTGATATTGTGGAT

TCTTGATCGCCTTTTCTTCAAATGCGTTTATCGTCGCCTTAAATACGGTTTGAAAAGAGG

GCCTTCTACGGAAGGAGTACCTGAGTCCATGAGGGAAGAGTACCGGCAGGAACAGCAGAG

TGCTGTGGATGTTGACGATGGTCATTTTGTCAACATAGAGCTGGAGTAAAAAACTACCTT

GTTTCTACT----------------------------------

>A_chicken_Rostov-on-Don_308-02_2020_EPI1848665

--AGCAAAAGCAGGTAGATATTGAAAGATGAGTCTTCTAACCGAGGTCGAAACGTACGTT

CTCTCTATCGTCCCGTCAGGCCCCCTCAAAGCCGAGATCGCGCAGAGACTTGAAGATGTC

TTTGCAGGGAAGAACACCGATCTTGAGGCTCTCATGGAATGGCTAAAGACAAGACCAATC

CTGTCACCTCTGACTAAGGGGATTTTGGGATTTGTGTTCACGCTCACCGTGCCCAGTGAG

CGAGGACTGCAGCGTAGACGCTTTGTCCAAAATGCTCTAAATGGAAATGGAGACCCAAAC

AACATGGACAGGGCAGTCAAACTGTACAGGAAATTGAAGAGAGAGATAACATTCCATGGG

GCTAAAGAAGTTGCACTCAGTTACTCAACCGGTGCACTTGCCAGTTGTATGGGTCTCATA

TACAACAGGATGGGGACGGTGACCGCAGAAGTGGCATTGGGCCTAGTGTGTGCCACCTGT

GAGCAGATTGCTGATTCACAGCATCGGTCTCACAGACAGATAGCAACCACCACCAACCCA

CTAATCAGACATGAAAACAGAATGGTGTTGGCCAGTACTACAGCTAAGGCTATGGAGCAG

ATGGCTGGATCGAGTGAGCAAGCAGCGGAAGCCATGGAGGTTGCTAGTCAGGCTAGGCAG

ATGGTGCAGGCGATGAGGACCATTGGAACTCATCCTAGCTCCAGTGCCGGTCTGAGAGAT

GATCTCCTTGAAAATTTGCAGGCCTACCAAAAACGGATGGGAGTGCAACTGCAGCGATTC

AAGTGATCCTCTCGTTATTGCCGCAAGTATCATTGGGATCTTGCACTTGATATTGTGGAT

TCTTGATCGCCTTTTCTTCAAATGCGTTTATCGTCGCCTTAAATACGGTTTGAAAAGAGG

GCCTTCTACGGAAGGGGTACCTGAGTCCATGAGGGAAGAGTACCGGCAGGAACAGCAGAG

TGCTGTGGATGTTGACGATGGTCATTTTGTCAACATAGAGCTGGAGTAAAAAACTACCTT

GTTTCTACT----------------------------------

>A_turkey_Stavropol_320-02_2020_EPI1848697

--AGCAAAAGCAGGTAGATATTGAAAGATGAGTCTTCTAACCGAGGTCGAAACGTACGTT

CTCTCTATCGTCCCGTCAGGCCCCCTCAAAGCCGAGATCGCGCAGAGACTTGAAGATGTC

TTTGCAGGGAAGAACACCGATCTTGAGGCTCTCATGGAATGGCTAAAGACAAGACCAATC

CTGTCACCTCTGACTAAGGGGATTTTGGGATTTGTGTTCACGCTCACCGTGCCCAGTGAG

CGAGGACTGCAGCGTAGACGCTTTGTCCAAAATGCTCTAAATGGAAATGGAGACCCAAAC

AACATGGACAGGGCAGTCAAACTGTACAGGAAATTGAAGAGAGAGATAACATTCCATGGG

GCTAAAGAAGTTGCACTCAGTTACTCAACCGGTGCACTTGCCAGTTGTATGGGTCTCATA

TACAACAGGATGGGGACGGTGACCGCAGAAGTGGCATTGGGCCTAGTGTGTGCCACCTGT

GAGCAGATTGCTGATTCACAGCATCGGTCTCACAGACAGATAGCAACCACCACCAACCCA

CTAATCAGACATGAAAACAGAATGGTGTTGGCCAGTACTACAGCTAAGGCCATGGAGCAG

ATGGCTGGATCGAGTGAGCAAGCAGCGGAAGCCATGGAGGTTGCTAGTCAGGCTAGGCAG

ATGGTGCAGGCGATGAGGACCATTGGAACTCATCCTAGCTCCAGTGCCGGTCTGAGAGAT

GATCTCCTTGAAAATTTGCAGGCCTACCAAAAACGGATGGGAGTGCAACTGCAGCGATTC

AAGTGATCCTCTCGTTATTGCCGCAAGTATCATTGGGATCTTGCACTTGATATTGTGGAT

TCTTGATCGTCTTTTCTTCAAATGCGTTTATCGTCGCCTTAAATACGGTTTGAAAAGAGG

GCCTTCTACGGAAGGAGTACCTGAGTCCATGAGGGAAGAGTACCGGCAGGAACAGCAGAG

TGCTGTGGATGTTGACGATGGTCATTTTGTCAACATAGAGCTGGAGTAAAAAACTACCTT

GTTTCTACT----------------------------------

>A_mute_swan_North_Ossetia-Alania_325-03_2020_EPI1848729

--AGCAAAAGCAGGTAGATATTGAAAGATGAGTCTTCTAACCGAGGTCGAAACGTACGTT

CTCTCTATCGTCCCGTCAGGCCCCCTCAAAGCCGAGATCGCGCAGAGACTTGAAGATGTC

TTTGCAGGGAAGAACACCGATCTTGAGGCTCTCATGGAATGGCTAAAGACAAGACCAATC

CTGTCACCTCTGACTAAGGGGATTTTGGGATTTGTGTTCACGCTCACCGTGCCCAGTGAG

CGGGGACTGCAGCGTAGACGCTTTGTCCAAAATGCTCTAAATGGAAATGGAGACCCAAAC

AACATGGACAGGGCAGTCAAACTGTACAGGAAATTGAAGAGAGAGATAACATTCCATGGG

GCTAAAGAAGTTGCACTCAGTTACTCAACCGGTGCACTTGCCAGTTGTATGGGTCTCATA

TACAACAGGATGGGGACGGTGACCGCAGAAGTGGCATTGGGCCTAGTGTGTGCCACCTGT

GAGCAGATTGCTGATTCACAGCATCGGTCTCACAGACAAATAGCAACCACCACCAACCCA

CTAATCAGACATGAAAACAGAATGGTGTTGGCCAGTACTACAGCTAAGGCTATGGAGCAG

ATGGCTGGATCGAGTGAGCAAGCAGCGGAAGCCATGGAGGTTGCCAGTCAGGCTAGGCAG

ATGGTGCAGGCGATGAGGACCATTGGAACTCATCCTAGCTCCAGTGCCGGTCTGAGAGAT

GATCTCCTTGAAAATTTGCAGGCCTACCAAAAACGGATGGGAGTGCAACTGCAGCGATTC

AAGTGATCCTCTCGTTATTGCCGCAAGTATCATTGGGATCTTGCACTTGATATTGTGGAT

TCTTGATCGCCTTTTCTTCAAATGCGTTTATCGTCGCCTTAAATACGGTTTGAAAAGAGG

GCCTTCTACGGAAGGAGTACCTGAGTCCATGAGGGAAGAGTACCGGCAGGAACAGCAGAG

TGCTGTGGATGTTGACGATGGTCATTTTGTCAACATAGAGCTGGAGTAAAAAACTACCTT

GTTTCTACT----------------------------------

>A_turkey_Rostov-on-Don_332-09_2021_EPI1848753

--AGCAAAAGCAGGTAGATATTGAAAGATGAGTCTTCTAACCGAGGTCGAAACGTACGTT

CTCTCTATCGTCCCGTCAGGCCCCCTCAAAGCCGAGATCGCGCAGAGACTTGAAGATGTC

TTTGCAGGGAAAAACACCGATCTTGAGGCTCTCATGGAATGGCTAAAGACAAGACCAATC

CTGTCACCTCTGACTAAGGGGATTTTGGGATTTGTGTTCACGCTCACCGTGCCCAGTGAG

CGAGGACTGCAGCGTAGACGCTTTGTCCAAAATGCTCTAAATGGAAATGGAGACCCAAAC

AACATGGACAGGGCAGTCAAACTGTACAGGAAACTGAAGAGAGAGATAACATTCCATGGG

GCTAAAGAAGTTGCACTCAGTTACTCAACCGGTGCACTTGCCAGTTGTATGGGTCTCATA

TACAACAGGATGGGGACGGTGACCGCAGAAGTGGCATTGGGCCTAGTGTGTGCCACCTGT

GAGCAGATTGCTGATTCACAGCATCGGTCTCACAGACAGATAGCAACCACCACCAACCCA

CTAATCAGACATGAAAACAGAATGGTGTTGGCCAGTACTACAGCTAAGGCTATGGAGCAG

ATGGCTGGGTCGAGTGAGCAAGCAGCGGAAGCCATGGAGGTTGCCAGTCAGGCTAGGCAG

ATGGTGCAGGCGATGAGGACCATTGGAACTCATCCTAGCTCCAGTGCCGGTCTGAGAGAT

GATCTCCTTGAAAATTTGCAGGCCTACCAAAAACGGATGGGAGTGCAACTGCAGCGATTC

AAGTGATCCTCTCGTTATTGCCGCAAGTATCATTGGGATCTTGCACTTGATATTGTGGAT

TCTTGATCGCCTTTTCTTCAAATGCGTTTATCGTCGCCTTAAATACGGTTTGAAAAGAGG

GCCTTCTACGGAAGGAGTACCTGAGTCCATGAGGGAAGAGTACCGGCAGAAACAGCAGAG

TGCTGTGGATGTTGACGATGGTCATTTTGTCAACATAGAGCTGGAGTAAAAAACTACCTT

GTTTCTACT----------------------------------

>A_chicken_Krasnodar_334-03_2021_EPI1848801

--AGCAAAAGCAGGTAGATATTGAAAGATGAGTCTTCTAACCGAGGTCGAAACGTACGTT

CTCTCTATCGTCCCGTCAGGCCCCCTCAAAGCCGAGATCGCGCAGAGACTTGAAGATGTC

TTTGCAGGGAAGAACACCGATCTTGAGGCTCTCATGGAATGGCTAAAGACAAGACCAATC

CTGTCACCTCTGACTAAGGGGATTTTGGGATTTGTGTTCACGCTCACCGTGCCCAGTGAG

CGAGGACTGCAGCGTAGACGCTTTGTCCAAAATGCTCTAAATGGAAATGGAGACCCAAAC

AACATGGACAGGGCAGTCAAACTGTACAGGAAATTGAAGAGAGAGATAACATTCCATGGG

GCTAAAGAAGTTGCACTCAGTTACTCAACCGGTGCACTTGCCAGTTGTATGGGTCTCATA

TACAACAGGATGGGGACGGTGACCGCAGAAGTGGCATTGGGCCTAGTGTGTGCCACCTGT

GAGCAGATTGCTGATTCACAGCATCGGTCTCACAGACAGATAGCAACCACCACCAACCCA

CTAATCAGACATGAAAACAGAATGGTGTTGGCCAGTACTACAGCTAAGGCTATGGAGCAG

ATGGCTGGATCGAGTGAGCAAGCAGCGGAAGCCATGGAGGTTGCTAGTCAGGCTAGGCAG

ATGGTGCAGGCGATGAGGACCATTGGAACTCATCCTAGCTCCAGTGCCGGTCTGAGAGAT

GATCTCCTTGAAAATTTGCAGGCCTACCAAAAACGGATGGGAGTGCAACTGCAGCGATTC

AAGTGATCCTCTCGTTATTGCCGCAAGTATCATTGGGATCTTGCACTTGATATTGTGGAT

TCTTGATCGCCTTTTCTTCAAATGCGTTTATCGTCGCCTTAAATACGGTTTGAAAAGAGG

GCCTTCTACGGAAGGAGTACCTGAGTCCATGAGGGAAGAGTACCGGCAGGAACAGCAGAG

TGCTGTGGATGTTGACGATGGTCATTTTGTCAACATAGAGCTGGAGTAAAAAACTACCTT

GTTTCTACT----------------------------------

>A_pheasant_Wales_000252_2021_EPI1848881

---------------------------ATGAGTCTTCTAACCGAGGTCGAAACGTACGTT

CTCTCTATCGTCCCGTCAGGCCCCCTCAAAGCCGAGATCGCGCAGAGACTTGAAGATGTC

TTTGCAGGGAAGAACACCGATCTTGAGGCTCTCATGGAATGGCTAAAGACAAGACCAATC

CTGTCACCTCTGACTAAGGGGATTTTGGGATTTGTGTTCACGCTCACCGTGCCCAGTGAG

CGAGGACTGCAGCGTAGACGCTTTGTCCAAAATGCTCTAAATGGAAATGGAGACCCAAAC

AACATGGACAGGGCAGTCAAACTGTACAGGAAATTGAAGAGAGAGATAACATTCCATGGG

GCTAAAGAAGTTGCACTCAGTTACTCAACCGGTGCACTTGCCAGTTGTATGGGTCTCATA

TACAACAGGATGGGGACGGTGACCGCAGAAGTGGCATTGGGCCTAGTGTGTGCCACCTGT

GAGCAGATTGCTGATTCACAGCATCGGTCTCACAGACAGATAGCAACCACCACCAACCCA

CTAATCAGACATGAAAACAGAATGGTGTTGGCCAGTACTACAGCTAAGGCTATGGAGCAG

ATGGCTGGATCGAGTGAGCAAGCAGCGGAAGCCATGGAGGTTGCTAGTCAGGCTAGGCAG

ATGGTGCAGGCGATGAGGACCATTGGAACTCATCCTAGCTCCAGTGCCGGTCTGAGAGAT

GATCTCCTTGAAAATTTGCAGGCCTACCAAAAACGGATGGGAGTGCAACTGCAGCGATTC

AAGTGATCCTCTCGTTATTGCCGCAAGTATCATTGGGATCTTGCACTTGATATTGTGGAT

TCTTGATCGCCTTTTCTTCAAATGCGTTTATCGTCGCCTTAAATACGGTTTGAAAAGAGG

GCCTTCTACGGAAGGAGTACCTGAGTCCATGAGGGAAGAGTACCGGCAGGAACAGCAGAG

TGCTGTGGATGTTGACGATGGTCATTTTGTCAACATAGAGCTGGAGTAA-----------

-------------------------------------------

>A_mute_swan_Czech_Republic_1656-1_2021_EPI1850131

--------------------------GATGAGTCTTCTAACCGAGGTCGAAACGTACGTT

CTCTCTATCGTCCCGTCAGGCCCCCTCAAAGCCGAGATCGCGCAGAGACTTGAAGATGTC

TTTGCAGGGAAGAACACCGATCTTGAGGCTCTCATGGAATGGCTAAAGACAAGACCAATC

CTGTCACCTCTGACTAAGGGGATTTTGGGATTTGTGTTCACGCTCACCGTGCCCAGTGAG

CGAGGACTGCAGCGTAGACGCTTTGTCCAAAATGCTCTAAATGGAAATGGAGACCCAAAC

AACATGGACAGGGCAGTCAAACTGTACAGGAAATTGAAGAGAGAGATAACATTCCATGGG

GCTAAAGAAGTTGCACTCAGTTACTCAACCGGTGCACTTGCCAGTTGTATGGGTCTCATA

TACAACAGGATGGGGACRGTGACCGCAGAAGTGGCATTGGGCCTAGTGTGTGCCACCTGT

GAGCAGATTGCTGATTCACAGCATCGGTCTCACAGACAGATAGCAACCACCACCAACCCA

CTAATCAGACATGAAAACAGAATGGTGTTGGCCAGTACTACAGCTAAGGCTATGGAGCAG

ATGGCTGGATCGAGTGAGCAAGCAGCGGAAGCCATGGAGGTTGCTAGTCAGGCTAGGCAG

ATGGTGCAGGCGATGAGGACCATTGGAACTCATCCTAGCTCCAGTGCCGGTCTGAGAGAT

GATCTCCTTGAAAATTTGCAGGCCTACCAAAAACGGATGGGAGTGCAACTGCAGCGATTC

AAGTGATCCTCTCGTTATTGCCGCAAGTATCATTGGGATCTTGCACTTGATATTGTGGAT

TCTTGATCGCCTTTTCTTCAAATGCGTTTATCGTCGCCTTAAATACGGTTTGAAAAGAGG

GCCTTCTACGGAAGGAGTACCTGAGTCCATGAGGGAAGAGTACCGGCAGGAACAGCAGAG

TGCTGTGGATGTTGACGATGGTCATTTTGTCAACATAGAGCTGGAGTA------------

-------------------------------------------

>A_mallard_Korea_WA820_2020_EPI1850671

---------------------------ATGAGTCTTCTAACCGAGGTCGAAACGTACGTT

CTCTCTATCGTCCCGTCAGGCCCCCTCAAAGCCGAGATCGCGCAGAGACTTGAAGATGTC

TTTGCAGGGAAGAACACCGATCTTGAGGCTCTCATGGAATGGCTAAAGACAAGACCAATC

CTGTCACCTCTGACTAAGGGGATTTTGGGATTTGTGTTCACGCTCACCGTGCCCAGTGAG

CGAGGACTGCAGCGTAGACGCTTTGTCCAAAATGCTCTAAATGGAAATGGAGACCCAAAC

AACATGGACAGGGCAGTCAAACTGTACAGGAAACTGAAGAGAGAGATAACATTCCATGGG

GCTAAAGAAGTTGCACTCAGTTACTCAACCGGTGCACTTGCCAGTTGTATGGGTCTCATA

TACAACAGGATGGGGACGGTGACCGCAGAAGTGGCATTGGGCCTAGTGTGTGCCACCTGT

GAGCAGATTGCTGATTCACAGCATCGGTCTCACAGACAGATAGCAACCACCACCAACCCA

CTAATCAGACATGAAAACAGAATGGTGTTGGCAAGTACTACAGCTAAGGCTATGGAGCAG

ATGGCTGGATCGAGTGAGCAAGCAGCGGAAGCCATGGAGGTTGCCAGTCAGGCTAGGCAG

ATGGTGCAGGCGATGAGGACCATTGGAACTCATCCTAGCTCCAGTGCCGGTCTGAGAGAT

GATCTCCTTGAAAATTTGCAGGCCTACCAAAAACGGATGGGAGTGCAACTGCAGCGATTC

AAGTGATCCTCTCGTTATTGCCGCAAGTATCATTGGGATCTTGCACTTGATATTGTGGAT

TCTTGATCGCCTTTTCTTCAAATGCGTTTATCGTCGCCTTAAATACGGTTTGAAAAGAGG

GCCTTCTACGGAAGGAGTACCTGAGTCCATGAGGGAAGAGTACCGGCAGGAACAGCAGAG

TGCTGTGGATGTTGACGATGGTCATTTTGTCAACATAGAGCTGGAGTAA-----------

-------------------------------------------

>A_mute_swan_Croatia_14_2021_EPI1850965

--------------TAGATATTGAAAGATGAGTCTTCTAACCGAGGTCGAAACGTACGTT

CTCTCTATCGTCCCGTCAGGCCCCCTCAAAGCCGAGATCGCGCAGAGACTTGAAGATGTC

TTTGCAGGGAAGAACACCGATCTTGAGGCTCTCATGGAATGGCTAAAGACAAGACCAATC

CTGTCACCTCTGACTAAGGGGATTTTGGGATTTGTGTTCACGCTCACCGTGCCCAGTGAG

CGAGGACTGCAGCGTAGACGCTTTGTCCAAAATGCTCTAAATGGAAATGGAGACCCAAAC

AACATGGACAGGGCAGTCAAACTGTACAGGAAATTGAAGAGAGAGATAACATTCCATGGG

GCTAAAGAAGTTGCACTCAGTTACTCAACCGGTGCACTTGCCAGTTGTATGGGTCTCATA

TACAACAGGATGGGGACGGTGACCGCAGAAGTGGCATTGGGCCTAGTGTGTGCAACCTGT

GAGCAGATTGCTGATTCACAGCATCGGTCTCACAGACAGATAGCAACCACCACCAACCCA

CTAATCAGACATGAAAACAGAATGGTGTTGGCCAGTACAACAGCTAAGGCTATGGAGCAG

ATGGCTGGATCGAGTGAGCAAGCAGCGGAAGCCATGGAGGTTGCTAGTCAGGCTAGGCAG

ATGGTGCAGGCGATGAGGACCATTGGAACTCATCCTAGCTCCAGTGCCGGTCTGAGAGAT

GATCTCCTTGAAAATTTGCAGGCCTACCAAAAACGGATGGGAGTGCAACTGCAGCGATTC

AAGTGATCCTCTCGTTATTGCCGCAAGTATCATTGGGATCTTGCACTTGATATTGTGGAT

TCTTGATCGCCTTTTCTTCAAATGCGTTTATCGTCGCCTTAAATACGGTTTGAAAAGAGG

GCCTTCTACGGAAGGAGTACCTGAGTCCATGAGGGAAGAGTACCGGCAGGAACAGCAGAG

TGCTGTGGATGTTGACGATGGTCATTTTGTCAACATAGAGCTGGAGTAAAAAACTA----

-------------------------------------------

>A_chicken_Vietnam_Raho4-Cd-20-421_2020_EPI1853934

--------------TAGATGTTGAAAGATGAGTCTTCTAACCGAGGTCGAAACGTACGTT

CTCTCTATCATCCCATCAGGCCCCCTCAAAGCCGAGATCGCACAGAAACTTGAGGATGTG

TTTGCAGGAAAGAACGCTGATCTCGAGGCTCTCATGGAGTGGCTAAAGACAAGACCAATC

CTGTCACCTCTGACTAAAGGGATTTTGGGATTTGTATTCACGCTCACCGTGCCCAGTGAG

CGAGGACTGCAACGTAGACGTTTTGTCCAAAATGCCCTAAATGGAAATGGAGATCCAAAT

AACATGGATAGGGCAGTTAAGCTATATAAGAAGCTGAAAAGAGAAATAACATTCCATGGA

GCTAAGGAGGTCGCACTCAGTTACTCAACCGGTGCACTTGCCAGTTGCATGGGTCTCATA

TACAACAGAATGGGAACGGTGACTACAGAAGTGGCTTTTGGCCTAGTGTGTGCCACTTGT

GAGCAGATTGCAGATTCACAGCATCGGTCTCACAGACAGATGGCAACCATCACCAATCCA

CTAATCAGGCATGAGAACAGAATGGTGCTGGCCAGCACTACAGCTAAGGCTATGGAACAG

ATGGCGGGATCAAGTGAGCAGGCAGCAGAAGCAATGGAGGTCGCCAATCAGGCTAGACAG

ATGGTGCAGGCAATGAGAACAATTGGGACTCATCCTAATTCTAGTGCTGGTCTGAGAGAC

AATCTTCTTGAAAATTTGCAGGCCTACCAGAAACGAATGGGAGTGCAGATGCAGCGATTC

AAGTGATCCTCTTGTTGTTGCCGCAAGTATCATTGGGATCTTGCACTTGATATTGTGGAT

TCTTGATCGTCTTTTCTTCAAATACATTTATCGTCGCCTTAAATACGGTTTGAAAAGAGG

GCCTTCTACGGAAGGAGTACCGGAGTCTATGAGGGAGGAGTACCGGCAGGAACAGCAGAA

TGCTGTAGATGTTGACAATGACCATTTTGTCAACATAGAATTGGAGTAAAAAACTA----

-------------------------------------------

>A_chicken_Czech_Republic_3531-1_2021_EPI1854238

--------------------------GATGAGTCTTCTAACCGAGGTCGAAACGTACGTT

CTCTCTATCGTCCCGTCAGGCCCCCTCAAAGCCGAGATCGCGCAGAGACTTGAAGATGTC

TTTGCAGGGAAGAACACCGATCTTGAGGCTCTCATGGAATGGCTAAAGACAAGACCAATC

CTGTCACCTCTGACTAAGGGGATTTTGGGATTTGTGTTCACGCTCACCGTGCCCAGTGAG

CGAGGACTGCAGCGTAGACGCTTTGTCCAAAATGCTCTAAATGGAAATGGAGACCCAAAC

AACATGGACAGGGCAGTCAAACTGTACAGGAAATTGAAGAGAGAGATAACATTCCATGGG

GCTAAAGAAGTTGCACTCAGTTACTCAACCGGTGCACTTGCCAGTTGTATGGGTCTCATA

TACAACAGGATGGGGACGGTGACCGCAGAAGTGGCATTGGGCCTAGTGTGTGCCACCTGT

GAGCAGATTGCTGATTCACAGCATCGGTCTCACAGACAGATAGCAACCACCACCAACCCA

CTAATCAGACATGAAAACAGAATGGTGTTGGCCAGTACTACAGCTAAGGCTATGGAGCAG

ATGGCTGGATCGAGTGAGCAAGCAGCGGAAGCCATGGAGGTTGCTAGTCAGGCTAGGCAG

ATGGTGCAGGCGATGAGGACCATTGGAACTCATCCTAGCTCCAGTGCCGGTCTGAGAGAT

GATCTCCTTGAAAATTTGCAGGCCTACCAAAAACGGATGGGAGTGCAACTGCAGCGATTC

AAGTGATCCTCTCGTTATTGCCGCAAGTATCATTGGAATCTTGCACTTGATATTGTGGAT

TCTTGATCGCCTTTTCTTCAAATGCGTTTATCGTCGCCTTAAATACGGTTTGAAAAGAGG

GCCTTCTACGGAAGGAGTACCTGAGTCCATGAGGGAAGAGTACCGGCAGGAACAGCAGAG

TGCTGTGGATGTTGACGATGGTCATTTTGTCAACATAGAGCTGGAGTA------------

-------------------------------------------

>A_wigeon_Latvia_23903_2021_EPI1855978

--------AGCAGGTAGATATTGAAAGATGAGTCTTCTAACCGAGGTCGAAACGTACGTT

CTCTCTATCGTCCCGTCAGGCCCCCTCAAAGCCGAGATCGCGCAGAGACTTGAAGATGTC

TTTGCAGGGAAGAACACCGATCTTGAGGCTCTCATGGAATGGCTAAAGACAAGACCAATC

CTGTCACCTCTGACTAAGGGGATTTTGGGATTTGTGTTCACGCTCACCGTGCCCAGTGAG

CGAGGACTGCAGCGTAGACGCTTTGTCCAAAATGCTCTAAATGGAAATGGAGACCCAAAC

AACATGGACAGGGCAGTCAAACTGTACAGGAAATTGAAGAGAGAGATAACATTCCATGGG

GCTAAAGAAGTTGCACTCAGTTACTCAACCGGTGCACTTGCCAGTTGTATGGGTCTCATA

TACAACAGGATGGGGACGGTGACCGCAGAAGTGGCATTGGGCCTAGTGTGTGCCACCTGT

GAGCAGATTGCTGATTCACAGCATCGGTCTCACAGACAGATAGCAACCACCACCAACCCA

CTAATCAGACATGAAAACAGAATGGTGTTGGCCAGTACTACAGCTAAGGCTATGGAGCAG

ATGGCTGGATCGAGTGAGCAAGCAGCGGAAGCCATGGAGGTTGCTAGTCAGGCTAGGCAG

ATGGTGCAGGCGATGAGGACCATTGGAACTCATCCTAGCTCCAGTGCCGGTCTGAGAGAT

GATCTCCTTGAAAATTTGCAGGCCTACCAAAAACGGATGGGAGTGCAACTGCAGCGATTC

AAGTGATCCTCTCGTTATTGCCGCAAGTATCATTGGGATCTTGCACTTGATATTGTGGAT

TCTTGATCGCCTTTTCTTCAAATGCGTTTATCGTCGCCTTAAATACGGTTTGAAAAGAGG

GCCTTCTACGGAAGGAGTACCTGAGTCCATGAGGGAAGAGTACCGGCAGGAACAGCAGAG

TGCTGTGGATGTTGACGATGGTCATTTTGTCAACATAGAGCTGGAGTAAAAA--------

-------------------------------------------

>A_chicken_Czech_Republic_4980_2021_EPI1858495

--------------------------GATGAGTCTTCTAAACGAGGTCGAAACGTACGTT

CTCTCTATCGTCCCGTCAGGCCCCCTCAAAGCCGAAATCGCGCAGAGACTTGAAGATGTC

TTTGCAGGGAAGAACACCGATCTTGAGGCTCTCATGGAATGGCTAAAGACAAGACCAATC

CTGTCACCTCTGACTAAGGGGATTTTGGGATTTGTGTTCACGCTCACCGTGCCCAGTGAG

CGAGGACTGCAGCGTAGACGCTTTGTCCAAAATGCTCTAAATGGAAATGGAGACCCAAAC

AACATGGACAGGGCAGTCAAACTGTACAGGAAATTGAAGAGAGAGATAACATTCCATGGG

GCTAAAGAAGTTGCACTCAGTTACTCAACCGGTGCACTTGCCAGTTGTATGGGTCTCATA

TACAACAGGATGGGGACGGTGACCGCAGAAGTGGCATTGGGCCTAGTGTGTGCCACCTGT

GAGCAGATTGCTGATTCACAGCATCGGTCTCACAGACAGATAGCAACCACCACCAACCCA

CTAATCAGACATGAAAACAGAATGGTGTTGGCCAGTACTACAGCTAAGGCTATGGAGCAG

ATGGCTGGATCGAGTGAGCAAGCAGCGGAAGCCATGGAGGTTGCTAGTCAGGCTAGGCAG

ATGGTGCAGGCGATGAGGACCATTGGAACTCATCCTAGCTCCAGTGCCGGTCTGAGAGAT

GATCTCCTTGAAAATTTGCAGGCTTACCAAAAACGGATGGGAGTGCAACTGCAGCGATTC

AAGTGATCCTCTCGTTATTGCCGCAAGTATCATTGGGATCTTGCACTTGATATTGTGGAT

TCTTGATCGCCTTTTCTTCAAATGCGTTTATCGTCGCCTTAAATACGGTTTGAAAAGAGG

GCCTTCTACGGAAGGAGTACCTGAGTCCATGAGGGAAGAGTACCGGCAGGAACAGCAGAG

TGCTGTGGATGTTGACGATGGTCATTTTGTCAACATAGAGCTGGAGTA------------

-------------------------------------------

>A_swan_Lithuania_1258PG1_21VIR2606-2_2021_EPI1858569

--AGCRAAAGCAGGTAGATATTGAAAGATGAGTCTTCTAAACGAGGTCGAAACGTACGTT

CTCTCTATCGTCCCGTCAGGCCCCCTCAAAGCCGAGATCGCGCAGAGACTTGAAGATGTC

TTTGCAGGGAAGAACACCGATCTTGAGGCTCTCATGGAATGGCTAAAGACAAGACCAATC

CTGTCACCTCTGACTAAGGGGATTTTGGGATTTGTGTTCACGCTCACCGTGCCCAGTGAG

CGAGGACTGCAGCGTAGACGCTTTGTCCAAAATGCTCTAAATGGAAATGGAGACCCAAAC

AACATGGACAGGGCAGTCAAACTGTACAGGAAATTGAAGAGAGAGATAACATTCCATGGG

GCTAAAGAAGTTGCACTCAGTTACTCAACCGGTGCACTTGCCAGTTGTATGGGTCTCATA

TACAACAGGATGGGGACGGTGACCGCAGAAGTGGCATTGGGCCTAGTGTGTGCCACCTGT

GAGCAGATTGCTGATTCACAGCATCGGTCTCACAGACAGATAGCAACCACCACCAACCCA

CTAATCAGACATGAAAACAGAATGGTGTTGGCCAGTACTACAGCTAAGGCTATGGAGCAG

ATGGCTGGATCGAGTGAGCAAGCAGCGGAAGCCATGGAGGTTGCTAGTCAGGCTAGGCAG

ATGGTGCAGGCGATGAGGACCATTGGAACTCATCCTAGCTCCAGTGCCGGTCTGAGAGAT

GATCTCCTTGAAAATTTGCAGGCCTACCAAAAACGGATGGGAGTGCAACTGCAGCGATTC

AAGTGATCCTCTCGTTATTGCCCCAAGTATCATTGGGATCTTGCACTTGATATTGTGGAT

TCTTGATCGCCTTTTCTTCAAATGCGTTTATCGTCGCCTTAAATACGGTTTGAAAAGAGG

GCCTTCTACGGAAGGAGTACCTGAGTCCATGAGGGAAGAGTACCGGCAGGAACAGCAGAG

TGCTGTGGATGTTGACGATGGTCATTTTGTCAACATAGAGCTGGAGTAAAAAACTACCTT

GTTTCTACT----------------------------------

>A_swan_Lithuania_1298PG1_21VIR2606-3_2021_EPI1858577

--AGCRAAAGCAGGTAGATATTGAAAGATGAGTCTTCTAACCGAGGTCGAAACGTACGTT

CTCTCTATCGTCCCGTCAGGCCCCCTCAAAGCCGAGATCGCGCAGAGACTTGAAGATGTC

TTTGCAGGGAAGAACACCGATCTTGAGGCTCTCATGGAATGGCTAAAGACAAGACCAATC

CTGTCACCTCTGACTAAGGGGATTTTGGGATTTGTGTTCACGCTCACCGTGCCCAGTGAG

CGAGGACTGCAGCGTAGACGCTTTGTCCAAAATGCTCTAAATGGAAATGGAGACCCAAAC

AACATGGACAGGGCAGTCAAACTGTACAGGAAATTGAAGAGAGAGATAACATTCCATGGG

GCTAAAGAAGTTGCACTCAGTTACTCAACTGGTGCACTTGCCAGTTGTATGGGTCTCATA

TACAACAGGATGGGGACGGTGACCGCAGAAGTGGCATTGGGCCTAGTGTGTGCCACCTGT

GAGCAGATTGCTGATTCACAGCATCGGTCTCACAGACAAATAGCAACCACCACCAACCCA

CTAATCAGACATGAAAACAGAATGGTGTTGGCCAGTACTACAGCTAAGGCTATGGAGCAG

ATGGCTGGATCGAGTGAGCAAGCAGCGGAAGCCATGGAGGTTGCTAGTCAGGCTAGGCAG

ATGGTGCAGGCGATGAGGACCATTGGAACTCATCCTAGCTCCAGTGCCGGTCTGAGAGAT

GATCTCCTTGAAAATTTGCAGGCCTACCAAAAACGGATGGGAGTGCAACTGCAGCGATTC

AAGTGATCCTCTCGTTATTGCCGCAAGTATCATTGGGATCTTGCACTTGATATTGTGGAT

TCTTGATCGCCTTTTCTTCAAATGCGTTTATCGTCGCCTTAAATACGGTTTGAAAAGAGG

GCCTTCTACGGAAGGAGTACCTGAGTCCATGAGGGAAGAGTACCGGCAGGAACAGCAGAG

TGCTGTGGATGTTGACGATGGTCATTTTGTCAACATAGAGCTGGAGTAAAAAACTACCTT

GTTTCTACT----------------------------------

>A_chicken_Bulgaria_50-1_21VIR1454-9_2021_EPI1858617

--AGCAAAAGCAGGTAGATATTGAAAGATGAGTCTTCTAACCGAGGTCGAAACGTACGTT

CTCTCTATCGTCCCGTCAGGCCCCCTCAAAGCCGAGATCGCGCAGAGACTTGAAGATGTC

TTTGCAGGGAAGAACACCGATCTTGAGGCTCTCATGGAATGGCTAAAGACAAGACCAATC

CTGTCACCTCTGACTAAGGGGATTTTGGGATTTGTGTTCACGCTCACCGTGCCCAGTGAG

CGAGGACTGCAGCGTAGACGCTTTGTCCAAAATGCTCTAAATGGAAATGGAGACCCAAAC

AACATGGACAGGGCAGTCAAACTGTACAGGAAATTGAAGAGAGAGATAACATTCCATGGG

GCAAAAGAAGTTGCACTCAGTTACTCAACCGGTGCACTTGCCAGTTGTATGGGTCTCATA

TACAACAGGATGGGGACGGTGACCGCAGAAGTGGCATTGGGCCTAGTGTGTGCCACCTGT

GAGCAGATTGCTGATTCACAGCATCGGTCTCACAGACAGATAGCAACCACCACCAACCCA

CTAATCAGACATGAAAACAGAATGGTGTTGGCCAGTACTACAGCTAAGGCTATGGAGCAG

ATGGCTGGATCGAGTGAGCAAGCAGCGGAAGCCATGGAGGTTGCTAGTCAGGCTAGGCAG

ATGGTGCAGGCGATGAGGACCATTGGAACTCATCCTAGCTCCAGTGCCGGTCTGAGAGAT

GATCTCCTTGAAAATTTGCAGGCCTACCAAAAACGGATGGGAGTGCAACTGCAGCGATTC

AAGTGATCCTCTCGTTATTGCCGCAAGTATCATTGGGATCTTGCACTTGATATTGTGGAT

TCTTGATCGCCTTTTCTTCAAATGCGTTTATCGTCGCCTTAAATACGGTTTGAAAAGAGG

GCCTTCTACGGAAGGAGTACCTGAGTCCATGAGGGAAGAGTACCGGCAGGAACAGCAGAG

TGCTGTGGATGTTGACGATGGTCATTTTGTCAACATAGAGCTGGAGTAAAAAACTACCTT

GTTTCTACT----------------------------------

>A_mute_swan_Poland_MB189_2021_EPI1859658

---------------AGATATTGAAAGATGAGTCTTCTAACCGAGGTCGAAACGTACGTT

CTCTCTATCGTCCCGTCAGGCCCCCTCAAAGCCGAGATCGCGCAGAGACTTGAAGATGTC

TTTGCAGGGAAGAACACCGATCTTGAGGCTCTCATGGAATGGCTAAAGACAAGACCAATC

CTGTCACCTCTGACTAAGGGGATTTTGGGATTTGTGTTCACGCTCACCGTGCCCAGTGAG

CGAGGACTGCAGCGTAGACGCTTTGTCCAAAATGCTCTAAATGGAAATGGAGACCCAAAC

AACATGGACAGGGCAGTCAAACTGTACAGGAAATTGAAGAGAGAGATAACATTCCATGGG

GCTAAAGAAGTTGCACTCAGTTACTCAACCGGTGCACTTGCCAGTTGTATGGGTCTCATA

TACAACAGGATGGGGACGGTGACCGCAGAAGTGGCATTGGGCCTAGTGTGTGCCACCTGT

GAGCAGATTGCTGATTCACAGCACCGGTCTCACAGACAGATAGCAACCACCACCAACCCA

CTAATCAGACATGAAAACAGAATGGTATTGGCCAGTACTACAGCTAAGGCTATGGAGCAG

ATGGCTGGATCGAGTGAGCAGGCAGCAGAAGCCATGGAGGTTGCTAGTCAGGCTAGGCAG

ATGGTGCAGGCAATGAGGACCATTGGAACTCATCCTAGCTCCAGTGCCGGTCTGAGAGAT

GATCTCCTTGAAAATTTGCAGGCCTACCAAAAACGGATGGGAGTGCAACTGCAGCGATTC

AAGTGATCCTCTCGTTATTGCCGCAAGTATCATTGGGATCTTGCACTTGATATTGTGGAT

TCTTGATCGCCTTTTCTTCAAATGCGTTTATCGTCGCCTTAAATACGGTTTGAAAAGAGG

GCCTTCTACGGAAGGGGTACCTGAGTCCATGAGGGAAGAGTACCGGCAGGAACAGCAGAG

TGCTGTGGATGTTGACGATGGTCATTTTGTCAACATAGAGCTGGAGTAAAAAACTA----

-------------------------------------------

>A_mute_swan_Poland_MB272_2021_EPI1859674

---------------AGATATTGAAAGATGAGTCTTCTAAACGAGGTCGAAACGTACGTT

CTCTCTATCGTCCCGTCAGGCCCCCTCAAAGCCGAGATCGCGCAGAGACTTGAAGATGTC

TTTGCAGGGAAGAACACCGATCTTGAGGCTCTCATGGAATGGCTAAAGACAAGACCAATC

CTGTCACCTCTGACTAAGGGGATTTTGGGATTTGTGTTCACGCTCACCGTGCCCAGTGAG

CGAGGACTGCAGCGTAGACGCTTTGTCCAAAATGCTCTAAATGGAAATGGAGACCCAAAC

AACATGGACAGGGCAGTCAAACTGTACAGGAAATTGAAGAGAGAGATAACATTCCATGGG

GCTAAAGAAGTTGCACTCAGTTACTCAACCGGTGCACTTGCCAGTTGTATGGGTCTCATA

TACAACAGGATGGGGACGGTGACCGCAGAAGTGTCATTGGGCCTAGTGTGTGCCACCTGT

GAGCAGATTGCTGATTCACAGCATCGGTCTCACAGACAGATAGCAACCACCACCAACCCA

CTAATCAGACATGAAAACAGAATGGTGTTGGCCAGTACTACAGCTAAGGCTATGGAGCAG

ATGGCTGGATCGAGTGAGCAAGCAGCGGAAGCCATGGAGGTTGCTAGTCAGGCTAGGCAG

ATGGTGCAGGCGATGAGGACCATTGGAACTCATCCTAGCTCCAGTGCCGGTCTGAGAGAT

GATCTCCTTGAAAATTTGCAGGCCTACCAAAAACGGATGGGAGTGCAACTGCAGCGATTC

AAGTGATCCTCTCGTTATTGCCGCAAGTATCATTGGGATCTTGCACTTGATATTGTGGAT

TCTTGATCGCCTTTTCTTCAAATGCGTTTATCGTCGCCTTAAATACGGTTTGAAAAGAGG

GCCTTCTACGGAAGGAGTACCTGAGTCCATGAGGGAAGAGTACCGGCAGGAACAGCAGAG

TGCTGTGGATGTTGACGATGGTCATTTTGTCAACATAGAGCTGGAGTAAAAAACTA----

-------------------------------------------

>A_anser_anser_Spain_297-1_21VIR1230-5_2021_EPI1860066

--AGCAAAAGCAGGTAGATATTGAAAGATGAGTCTTCTAACCGAGGTCGAAACGTACGTT

CTCTCTATCGTCCCGTCAGGCCCCCTCAAAGCCGAGATCGCGCAGAGACTTGAAGATGTC

TTTGCAGGGAAGAACACCGATCTTGAGGCTCTCATGGAATGGCTAAAGACAAGACCAATC

CTGTCACCTCTGACTAAGGGGATTTTGGGATTTGTGTTCACGCTCACCGTGCCCAGTGAG

CGAGGACTGCAGCGTAGACGCTTTGTCCAAAATGCTCTAAATGGAAATGGAGACCCAAAC

AACATGGACAGGGCAGTCAAACTGTACAGGAAATTGAAGAGAGAGATAACATTCCATGGG

GCTAAAGAAGTTGCACTCAGTTACTCAACCGGTGCACTTGCCAGTTGTATGGGTCTCATA

TACAACAGGATGGGGACGGTGACCGCAGAAGTGGCATTGGGCCTAGTGTGTGCCACCTGT

GAGCAGATTGCTGATTCACAGCATCGGTCTCACAGACAGATAGCAACCACCACCAACCCA

CTAATCAGACATGAAAACAGAATGGTGTTGGCCAGTACTACAGCTAAGGCTATGGAGCAG

ATGGCTGGATCGAGTGAGCAAGCAGCGGAAGCCATGGAGGTTGCTAGTCAGGCTAGGCAG

ATGGTGCAGGCGATGAGGACCATTGGAACTCATCCTAGCTCCAGTGCCGGTCTGAGAGAT

GATCTCCTTGAAAATTTGCAGGCCTACCAAAAACGGATGGGAGTGCAACTGCAGCGATTC

AAGTGATCCTCTCGTTATTGCCGCAAGTATCATTGGGATCTTGCACTTGATATTGTGGAT

TCTTGATCGCCTTTTCTTCAAATGCGTTTATCGTCGCCTTAAATACGGTTTGAAAAGAGG

GCCTTCTACGGAAGGAGTACCTGAGTCCATGAGGGAAGAGTACCGGCAGGAACAGCAGAG

TGCTGTGGATGTTGACGATGGTCATTTTGTCAACATAGAGCTGGAGTAAAAAACTACCTT

GTTTCTACT----------------------------------

>A_ciconia_ciconia_Spain_102-1_21VIR1230-2_2021_EPI1860074

--AGCAAAAGCAGGTAGATATTGAAAGATGAGTCTTCTAACCGAGGTCGAAACGTACGTT

CTCTCTATCGTCCCGTCAGGCCCCCTCAAAGCCGAGATCGCGCAGAGACTTGAAGATGTC

TTTGCAGGGAAGAACACCGATCTTGAGGCTCTCATGGAATGGCTAAAGACAAGACCAATC

CTGTCACCTCTGACTAAGGGAATTTTGGGATTTGTGTTCACGCTCACCGTGCCCAGTGAG

CGAGGACTGCAGCGTAGACGCTTTGTCCAAAATTCTCTAAATGGAAATGGAGACCCAAAC

AACATGGACAGGGCAGTCAAACTGTACAGGAAATTGAAGAGAGAGATAACATTCCATGGG

GCTAAAGAAGTTGCACTCAGTTACTCAACCGGTGCACTTGCCAGTTGTATGGGTCTCATA

TACAACAGGATGGGGACGGTGACCGCAGAAGTGGCATTGGGCCTAGTGTGTGCCACCTGT

GAGCAGATTGCTGATTCACAGCATCGGTCTCACAGACAGATAGCAACCACCACCAACCCA

CTAATCAGACATGAAAACAGAATGGTGTTGGCCAGTACTACAGCTAAGGCTATGGAGCAG

ATGGCTGGATCGAGTGAGCAAGCAGCGGAAGCCATGGAGGTTGCTAGTCAGGCTAGGCAG

ATGGTGCAGGCGATGAGGACCATTGGAACTCATCCTAGCTCCAGTGCCGGTCTGAGAGAT

GATCTCCTTGAAAATTTGCAGGCCTACCAAAAACGGATGGGAGTGCAACTGCAGCGATTC

AAGTGATCCTCTCGTTATTGCCGCAAGTATCATTGGGATCTTGCACTTGATATTGTGGAT

TCTTTATCGCCTTTTCTTCAAATGCGTTTATCGTCGCCTTAAATACGGTTTGAAAAGAGG

GCCTTCTACGGAAGGAGTACCTGAGTCCATGAGGGAAGAGTACCGGCAGGAACAGCAGAG

TGCTGTGGATGTTGACGATGGTCATTTTGTCAACATAGAGCTGGAGTAAAAAACTACCTT

GTTTCTACT----------------------------------

>A_duck_Jiangsu_k1203_2010_EPI442020

--AGCAAAAGCAGGTAGATATTGAAAGATGAGTCTTCTAACCGAGGTCGAAACGTACGTT

CTCTCTATCATCCCGTCAGGCCCCCTCAAAGCCGAGATCGCGCAGAAACTTGAAGATGTC

TTTGCAGGGAAGAACACCGATCTCGAGGCTCTCATGGAGTGGCTAAAGACAAGACCAATC

CTGTCACCTCTGACTAAAGGGATTTTGGGATTTGTATTCACGCTCACCGTGCCCAGTGAG

CGAGGACTGCAGCGTAGACGCTTTGTCCAGAATGCCCTAAATGGAAATGGAGATCCAAAT

AATATGGATAGGGCAGTTAAGCTATATAAGAAGCTGAAAAGGGAAATAACATTCCATGGG

GCTAAGGAGGTCGCACTTAGTTACTCAACCGGTGCGCTTGCCAGTTGCATGGGTCTCATA

TACAATAGGATGGGAACGGTGACTACAGAAGTGGCTTTTGGCCTAGTGTGTGCCACTTGT

GAGCAGATTGCAGATTCACAGCATCGGTCTCACAGACAGATGGCAACCATCACCAACCCA

TTAATCAGGCATGAGAACAGAATGGTGCTGGCCAGCACTACAGCTAAGGCCATGGAGCAG

ATGGCGGGATCAAGTGAGCAGGCAGCGGAAGCCATGGAAGTTGCTAATCAGGCTAGGCAG

ATGGTACAGGCAATGAGGACAATTGGGACTCATCCTAACTCTAGTGCTGGTCTGAGAGAT

AATCTTCTTGAAAATTTGCAGGCCTACCAGAACCGAATGGGAGTGCAGATGCAGCGATTC

AAGTGATCCTCTTGTTGTTGCCGCAAATATCATTGGGATCTTGCACTTGATATTGTGGAT

TCTTGATCGTCTTTTCTTCAAATGCATTTACCGTCGCCTTAAACACGGTTTGAAAATAGG

GCCTTCTACGGAAGGGGTACCTGAGTCTATGAGGGAAGAGTACCGGCAGGAACAGCAGAG

TGCTGTGGATGTTGACGATGGTCATTTTGTCAACATAGAATTGGAGTAAAAAACTAC---

-------------------------------------------

>A_Turkey_Egypt_AR550_2018_EPI1420336

-----------AGTTAGATATTGAAAGATGAGTCTTCTAACCGAGGTCGAAACGTACGTT

CTCTCTATCGTCCCGTCAGGCCCCCTCAAAGCCGAGATCGCGCAGAGACTTGAAGATGTC

TTTGTAGGGAAGAACACCGACCTTGAGGCTATCATGGAATGGCTAAAGACAAGACCAATC

CTGTCACCTCTGACTAAGGGGATTTTAGGGTTTGTGTTCACGCTCACCGTGCCCAGTGAG

CGAGGACTGCAGCGTAGACGCTTTGTCCAAAATGCTCTAAATGGAAATGGAGACCCAAAC

AACATGGACAGAGCAGTCAAACTGTACAGGAAATTAAAGAGAGAGATAACATTCCATGGG

GCTAAAGAAGTTGCACTCAGTTACTCAACCGGTGCACTTGCCAGTTGTATGGGTCTCATA

TACAACAAGATGGGGACGGTGACCACAGAAGCGGCGTTGGGCCTAGTGTGTGCCACCTGT

GAGCAGATTGCTGATTCACAGCATCGATCTCACAGACAGATAGCAACCATCACCAACCCA

CTAATCAGACATGAAAACAGAATGGTGCTGGCCAGTACTACAGCTAAGGCTATGGAGCAG

ATGGCTGGGTCGAGTGAGCAGGCAGCGGAAGCCATGGAGGTTGCTAGTCAAGCTAGGCAG

ATGGTGCAGGCTATGAGGACCATTGGAACTCACCCTAGCTCCAGTGCCGGTCTGAGAGAT

GATCTCCTTGAAAATTTACAGGCCTACCAAAAACGGATGGGAGTGCAGATGCAGCGATTC

AAGTGATCCTCTCGTTATTGCCGCAAGTATCATTGGGATCTTGCACTTGATATTGTGGAT

TCTTGATCGTTTTTTCTTCAAATGCGTTTATCGTCGCCTTAAATACGGTTTGAAAAGAGG

GCCTTCTACGGAAGGAGTGCCTGAGTCTATGAGGGAAGAGTATCGGCAGGAACAGCAGAG

TGCTGTGGATGTTGACGATGGTCATTTTGTCAACATAGAGCTGGAGTAAAAAACTACT--

-------------------------------------------

>A_Chicken_Egypt_AI20286_2019_EPI1636740

------------------------------------CTAACCGAGGTCGAAACGTACGTT

CTCTCTATCGTCCCGTCAGGCCCCCTCAAAGCCGAGATCGCGCAGAGACTTGAAGATGTC

TTTGTAGGGAAGAACACCGATCTTGAGGCTATCATGGAATGGCTAAAGACAAGACCAATC

CTGTCACCTCTGACTAAGGGGATTTTAGGGTTTGTGTTCACGCTCACCGTGCCCAGTGAG

CGAGGACTGCAGCGTAGACGCTTTGTCCAAAATGCTCTAAATGGAAATGGAGACCCAAAC

AACATGGACAAAGCAGTCAAACTGTACAGGAAATTAAAGAGAGAGATAACATTCCATGGG

GCTAAAGAAGTTGCGCTCAGTTACTCAACCGGTGCACTTGCAAGTTGTATGGGTCTCATA

TACAACAAGATGGGGACGGTGACCACAGAAGTGGCGTTGGGCCTAGTGTGTGCCACCTGT

GAGCAGATTGCTGATTCACAGCATCGGTCTCACAGACAGATAGCAACCATCACCAACCCA

CTAATCAGACATGAAAACAGGATGGTGCTGGCCAGTACTACAGCTAAGGCTATGGAGCAG

ATGGCTGGGTCGAGTGAGCAGGCAGCGGAAGCCATGGAGGTTGCTAGTCAAGCTAGGCAG

ATGGTGCAGGCGATGAGAACCATTGGAACTCACCCTAGCTCCAGTGCCGGTCTGAGAGAT

GATCTCCTTGAAAATTTACAGGCCTACCAGAAACGGATGGGAGTGCAGATGCAGCGATTC

AAGTGATCCTCTCGTTATTGCCGCAAGTATCATTGGGATCTTGCACTTGATATTGTGGAT

TCTTGATCGTTTTTTCTTCAAATGCGTTTATCGTCGCCTTAAATACGGTTTGAAAAGAGG

GCCTTCTACGGAAGGAGTGCCTGAGTCTATGAGGGAAGAGTATCGGCAGGAACAGCAGAG

TGCTGTGGATGT------------------------------------------------

-------------------------------------------

>A_Chicken_Egypt_AR553_2018_EPI1637085

------------------------------------CTAACCGAGGTCGAAACGTACGTT

CTCTCTATCGTCCCGTCAGGCCCCCTCAAAGCCGAGATCGCGCAGAGACTTGAAGATGTC

TTTGTAGGGAAGAACACCGACCTTGAGGCTATCATGGAATGGCTAAAGACAAGACCAATC

CTGTCACCTCTGACTAAGGGGATTTTAGGGTTTGTGTTCACGCTCACCGTGCCCAGTGAG

CGAGGACTGCAGCGTAGACGCTTTGTCCAAAATGCTCTAAATGGAAATGGAGACCCAAAC

AACATGGACAGAGCAGTCAAACTGTACAGGAAATTAAAGAGAGAGATAACATTCCATGGG

GCTAAAGAAGTTGCACTCAGTTACTCAACCGGTGCACTTGCCAGTTGTATGGGTCTCATA

TACAACAAGATGGGGACGGTGACCACAGAAGCGGCGTTGGGCCTAGTGTGTGCCACCTGT

GAGCAGATTGCTGATTCACAGCATCGATCTCACAGACAGATAGCAACCATCACCAACCCA

CTAATCAGACATGAAAACAGAATGGTGCTGGCCAGTACTACAGCTAAGGCTATGGAGCAG

ATGGCTGGGTCGAGTGAGCAGGCAGCGGAAGCCATGGAGGTTGCTAGTCAAGCTAGGCAG

ATGGTGCAGGCTATGAGGACCATTGGAACTCACCCTAGCTCCAGTGCCGGTCTGAGAGAT

GATCTCCTTGAAAATTTACAGGCCTACCAAAAACGGATGGGAGTGCAGATGCAGCGATTC

AAGTGATCCTCTCGTTATTGCCGCAAGTATCATTGGGATCTTGCACTTGATATTGTGGAT

TCTTGATCGTTTTTTCTTCAAATGCGTTTATCGTCGCCTTAAATACGGTTTGAAAAGAGG

GCCTTCTACGGAAGGAGTGCCTGAGTCTATGAGGGAAGAGTATCGGCAGGAACAGCAGAG

TGCTG-------------------------------------------------------

-------------------------------------------

>A_Turkey_Egypt_AI20285_2019_EPI1638800

------------------------------------CTAACCGAGGTCGAAACGTACGTT

CTCTCTATCGTCCCGTCAGGCCCCCTCAAAGCCGAGATCGCGCAGAGACTTGAAGATGTC

TTTGTAGGGAAGAACACCGATCTTGAGGCTATCATGGAATGGCTAAAGACAAGACCAATC

CTGTCACCTCTGACTAAGGGGATTTTAGGGTTTGTGTTCACGCTCACCGTGCCCAGTGAG

CGAGGACTGCAGCGTAGACGCTTTGTCCAAAATGCTCTAAATGGAAATGGAGATCCAAAC

AACATGGACAGAGCAGTCAAACTGTACAGGAAATTGAAGAGAGAGATAACATTCCATGGG

GCTAAAGAAGTTGCACTCAGTTACTCAACCGGTGCACTTGCCAGTTGTATGGGTCTCATA

TACAACAAAATGGGGACGGTGACCACAGAAGTGGCGTTGGGCCTAGTGTGTGCCACCTGT

GAGCAGATTGCTGATTCACAGCATCGGTCTCACAGACAGATAGCAACCATCACCAACCCA

CTAATCAGACATGAAAACAGAATGGTGCTGGCCAGTACTACAGCTAAGGCTATGGAGCAG

ATGGCTGGGTCGAGTGAGCAGGCAGCGGAAGCCATGGAGGTTGCTAGTCAAGCTAGGCAG

ATGGTGCAGGCGATGAGAACCATTGGAACTCACCCTAGCTCCAGTGCCGGTCTGAGGGAT

GATCTCCTTGAAAATTTACAGGCCTACCAGAAACGGATGGGAGTGCAGATGCAGCGATTC

AAGTGATCCTCTCGTTATTGCCGCAAGTATCATTGGGATCTTGCACTTGATATTGTGGAT

TCTTGATCGTTTTTTCTTCAAATGCGTTTATCGTCGCCTTAAATACGGTTTGAAAAGAGG

GCCTTCTACGGAAGGAGTGCCTGAGTCTATGAGGGAAGAGTATCGGCAGGAACAGCAGAG

TGCTGTGGATGT------------------------------------------------

-------------------------------------------

>A_goose_Omsk_0111_2020_EPI1813140

--AGCAAAAGCAGGTAGATATTGAAAGATGAGTCTTCTAACCGAGGTCGAAACGTACGTT

CTCTCTATCGTCCCGTCAGGCCCCCTCAAAGCCGAGATCGCGCAGAGACTTGAAGATGTC

TTTGCAGGGAAGAACACCGATCTTGAGGCTCTCATGGAATGGCTAAAGACAAGACCAATC

CTGTCACCTCTGACTAAGGGGATTTTGGGATTTGTGTTCACGCTCACCGTGCCCAGTGAG

CGAGGACTGCAGCGTAGACGCTTTGTCCAAAATGCTCTAAATGGAAATGGAGACCCAAAC

AACATGGACAGGGCAGTCAAACTGTACAGGAAATTGAAGAGAGAGATAACATTCCATGGG

GCTAAAGAAGTTGCACTCAGTTACTCAACCGGTGCACTTGCCAGTTGTATGGGTCTCATA

TACAACAGGATGGGGACGGTGACCGCAGAAGTGGCATTGGGCCTAGTGTGTGCCACCTGT

GAGCAGATTGCTGATTCACAGCATCGGTCTCACAGACAGATAGCAACCACCACCAACCCA

CTAATCAGACATGAAAACAGAATGGTGTTGGCCAGTACTACAGCTAAGGCTATGGAGCAG

ATGGCTGGATCGAGTGAGCAAGCAGCGGAAGCCATGGAGGTTGCCAGTCAGGCTAGGCAG

ATGGTGCAGGCGATGAGGACCATTGGAACTCATCCTAGCTCCAGTGCCGGTCTGAGAGAT

GATCTCCTTGAAAATTTGCAGGCCTACCAAAAACGGATGGGAGTGCAACTGCAGCGATTC

AAGTGATCCTCTCGTTATTGCCGCAAGTATCATTGGGATCTTGCACTTGATATTGTGGAT

TCTTGATCGCCTTTTCTTCAAATGCGTTTATCGTCGCCTTAAATACGGTTTGAAAAGAGG

GCCTTCTACGGAAGGAGTACCTGAGTCCATGAGGGAAGAGTACCGGCAGGAACAGCAGAG

TGCTGTGGATGTTGACGATGGTCATTTTGTCAACATAGAGCTGGAGTAAAAAACTACCTT

GTTTCTACT----------------------------------

>A_goose_Omsk_01161_2020_EPI1813356

--AGCAAAAGCAGGTAGATATTGAAAGATGAGTCTTCTAACCGAGGTCGAAACGTACGTT

CTCTCTATCGTCCCGTCAGGCCCCCTCAAAGCCGAGATCGCGCAGAGACTTGAAGATGTC

TTTGCAGGGAAGAACACCGATCTTGAGGCTCTCATGGAATGGCTAAAGACAAGACCAATC

CTGTCACCTCTGACTAAGGGGATTTTGGGATTTGTGTTCACGCTCACCGTGCCCAGTGAG

CGAGGACTGCAGCGTAGACGCTTTGTCCAAAATGCTCTAAATGGGAATGGAGACCCAAAC

AACATGGACAGGGCAGTCAAACTGTACAGGAAATTGAAGAGAGAGATAACATTCCATGGG

GCTAAAGAAGTTGCACTCAGTTACTCAACCGGTGCACTTGCCAGTTGTATGGGTCTCATA

TACAACAGGATGGGGACGGTGACCGCAGAAGTGGCATTGGGCCTAGTGTGTGCCACCTGT

GAGCAGATTGCTGATTCACAGCATCGGTCTCACAGACAGATAGCAACCACCACCAACCCA

CTAATCAGACATGAAAACAGAATGGTGTTGGCCAGTACTACAGCTAAGGCTATGGAGCAG

ATGGCTGGATCGAGTGAGCAAGCAGCGGAAGCCATGGAGGTTGCTAGTCAGGCTAGGCAG

ATGGTGCAGGCGATGAGGACCATTGGAACTCATCCTAGCTCCAGTGCCGGTCTGAGAGAT

GATCTCCTTGAAAATTTGCAGGCCTACCAAAAACGGATGGGAGTGCAACTGCAGCGATTC

AAGTGATCCTCTCGTTATTGCCGCAAGTATCATTGGGATCTTGCACTTGATATTGTGGAT

TCTTGATCGCCTTTTCTTCAAATGCGTTTATCGTCGCCTTAAATACGGTTTGAAAAGAGG

GCCTTCTACGGAAGGAGTACCTGAGTCCATGAGGGAAGAGTACCGGCAGGAACAGCAGAG

TGCTGTGGATGTTGACGATGGTCATTTTGTCAACATAGAGCTGGAGTAAAAAACTACCTT

GTTTCTACT----------------------------------

>A_chicken_Omsk_0118_2020_EPI1813372

--AGCAAAAGCAGGTAGATATTGAAAGATGAGTCTTCTAACCGAGGTCGAAACGTACGTT

CTCTCTATCGTCCCGTCAGGCCCCCTCAAAGCCGAGATCGCGCAGAGACTTGAAGATGTC

TTTGCAGGGAAGAACACCGATCTTGAGGCTCTCATGGAATGGCTAAAGACAAGACCAATC

CTGTCACCTCTGACTAAGGGGATTTTGGGATTTGTGTTCACGCTCACCGTGCCCAGTGAG

CGAGGACTGCAGCGTAGACGCTTTGTCCAAAATGCTCTAAATGGAAATGGAGACCCAAAC

AACATGGACAGGGCAGTCAAACTGTACAGGAAATTGAAGAGAGAGATAACATTCCATGGG

GCTAAAGAAGTTGCACTCAGTTACTCAACCGGTGCACTTGCCAGTTGTATGGGTCTCATA

TACAACAGGATGGGGACGGTGACCGCAGAAGTGGCATTGGGCCTAGTGTGTGCCACCTGT

GAGCAGATTGCTGATTCACAGCATCGGTCTCACAGACAGATAGCAACCACCACCAACCCA

CTAATCAGACATGAAAACAGAATGGTGTTGGCCAGTACTACAGCTAAGGCTATGGAGCAG

ATGGCTGGATCGAGTGAGCAAGCAGCGGAAGCCATGGAGGTTGCCAGTCAGGCTAGGCAG

ATGGTGCAGGCGATGAGGACCATTGGAACTCATCCTAGCTCCAGTGCCGGTCTGAGAGAT

GATCTCCTTGAAAATTTGCAGGCCTACCAAAAACGGATGGGAGTGCAACTGCAGCGATTC

AAGTGATCCTCTCGTTATTGCCGCAAGTATCATTGGGATCTTGCACTTGATATTGTGGAT

TCTTGATCGCCTTTTCTTCAAATGCGTTTATCGTCGCCTTAAATACGGTTTGAAAAGAGG

GCCTTCTACGGAAGGAGTACCTGAGTCCATGAGGGAAGAGTACCGGCAGGAACAGCAGAG

TGCTGTGGATGTTGACGATGGTCATTTTGTCAACATAGAGCTGGAGTAAAAAACTACCTT

GTTTCTACT----------------------------------

>A_chicken_Omsk_0119_2020_EPI1813380

--AGCAAAAGCAGGTAGATATTGAAAGATGAGTCTTCTAACCGAGGTCGAAACGTACGTT

CTCTCTATCGTCCCGTCAGGCCCCCTCAAAGCCGAGATCGCGCAGAGACTTGAAGATGTC

TTTGCAGGGAAGAACACCGATCTTGAGGCTCTCATGGAATGGCTAAAGACAAGACCAATC

CTGTCACCTCTGACTAAGGGGATTTTGGGATTTGTGTTCACGCTCACCGTGCCCAGTGAG

CGAGGACTGCAGCGTAGACGCTTTGTCCAAAATGCTCTAAATGGAAATGGAGACCCAAAC

AACATGGACAGGGCAGTCAAACTGTACAGGAAATTGAAGAGAGAGATAACATTCCATGGG

GCTAAAGAAGTTGCACTCAGTTACTCAACCGGTGCACTTGCCAGTTGTATGGGTCTCATA

TACAACAGGATGGGGACGGTGACCGCAGAAGTGGCATTGGGCCTAGTGTGTGCCACCTGT

GAGCAGATTGCTGATTCACAGCATCGGTCTCACAGACAGATAGCAACCACCACCAACCCA

CTAATCAGACATGAAAACAGAATGGTGTTGGCCAGTACTACAGCTAAGGCTATGGAGCAG

ATGGCTGGATCGAGTGAGCAAGCAGCGGAAGCCATGGAGGTTGCCAGTCAGGCTAGGCAG

ATGGTGCAGGCGATGAGGACCATTGGAACTCATCCTAGCTCCAGTGCCGGTCTGAGAGAT

GATCTCCTTGAAAATTTGCAGGCCTACCAAAAACGGATGGGAGTGCAACTGCAGCGATTC

AAGTGATCCTCTCGTTATTGCCGCAAGTATCATTGGGATCTTGCACTTGATATTGTGGAT

TCTTGATCGCCTTTTCTTCAAATGCGTTTATCGTCGCCTTAAATACGGTTTGAAAAGAGG

GCCTTCTACGGAAGGAGTACCTGAGTCCATGAGGGAAGAGTACCGGCAGGAACAGCAGAG

TGCTGTGGATGTTGACGATGGTCATTTTGTCAACATAGAGCTGGAGTAAAAAACTACCTT

GTTTCTACT----------------------------------

>A_chicken_Omsk_0073_2020_EPI1813404

--AGCAAAAGCAGGTAGATATTGAAAGATGAGTCTTCTAACCGAGGTCGAAACGTACGTT

CTCTCTATCGTCCCGTCAGGCCCCCTCAAAGCCGAGATCGCGCAGAGACTTGAAGATGTC

TTTGCAGGGAAGAACACCGATCTTGAGGCTCTCATGGAATGGCTAAAGACAAGACCAATC

CTGTCACCTCTGACTAAGGGGATTTTGGGATTTGTGTTCACGCTCACCGTGCCCAGTGAG

CGAGGACTGCAGCGTAGACGCTTTGTCCAAAATGCTCTAAATGGAAATGGAGACCCAAAC

AACATGGACAGGGCAGTCAAACTGTACAGGAAATTGAAGAGAGAGATAACATTCCATGGA

GCTAAAGAAGTTGCACTCAGTTACTCAACCGGTGCACTTGCCAGTTGTATGGGTCTCATA

TACAACAGGATGGGGACGGTGACCGCAGAAGTGGCATTGGGCCTAGTGTGTGCCACCTGT

GAGCAGATTGCTGATTCACAGCATCGGTCTCACAGACAGATAGCAACCACCACCAACCCA

CTAATCAGACATGAAAACAGAATGGTGTTGGCCAGTACTACAGCTAAGGCTATGGAGCAG

ATGGCTGGATCGAGTGAGCAAGCAGCGGAAGCCATGGAGGTTGCTAGTCAGGCTAGGCAG

ATGGTGCAGGCGATGAGAACCATTGGAACTCATCCTAGCTCCAGTGCCGGTCTGAGAGAT

GATCTCCTTGAAAATTTGCAGGCCTACCAAAAGCGGATGGGAGTGCAACTGCAGCGATTC

AAGTGATCCTCTCGTTATTGCCGCAAGTATCATTGGGATCTTGCACTTGATATTGTGGAT

TCTTGATCGCCTTTTCTTCAAATGCGTTTATCGTCGCCTTAAATACGGTTTGAAAAGAGG

GCCTTCTACGGAAGGAGTACCTGAGTCCATGAGGGAAGAGTACCGGCAGGAACAGCAGAG

TGCTGTGGATGTTGACGATGGTCATTTTGTCAACATAGAGCTGGAGTAAAAAACTACCTT

GTTTCTACT----------------------------------

>A_chicken_Omsk_30007_2020_EPI1814308

--AGCAAAAGCAGGTAGATGTTGAAAGATGAGTCTTCTAACCGAGGTCGAAACGTACGTT

CTCTCTATCGTCCCGTCAGGCCCCCTCAAAGCCGAGATCGCGCAGAGACTTGAAGATGTC

TTTGCAGGGAAGAACACCGATCTTGAGGCTCTCATGGAATGGCTAAAGACAAGACCAATC

CTGTCACCTCTGACTAAGGGGATTTTGGGATTTGTGTTCACGCTCACCGTGCCCAGTGAG

CGAGGACTGCAGCGTAGACGCTTTGTCCAAAATGCTCTAAATGGAAATGGAGACCCAAAC

AACATGGACAGGGCAGTCAAACTGTACAGGAAATTGAAGAGAGAGATAACATTCCATGGG

GCTAAAGAAGTTGCACTCAGTTACTCAACCGGTGCACTTGCCAGTTGTATGGGTCTCATA

TACAACAGGATGGGGACGGTGACCGCAGAAGTGGCATTGGGCCTAGTGTGTGCCACCTGT

GAGCAGATTGCTGATTCACAGCATCGGTCTCACAGACAGATAGCAACCACCACCAACCCA

CTAATCAGACATGAAAACAGAATGGTGTTGGCCAGTACTACAGCCAAGGCTATGGAGCAG

ATGGCTGGATCGAGTGAGCAAGCAGCGGAAGCCATGGAGGTTGCTAGTCAGGCTAGGCAG

ATGGTGCAGGCGATGAGGACCATTGGAACTCATCCTAGCTCCAGTGCCGGTCTGAGAGAT

GATCTCCTTGAAAATTTACAGGCCTACCAAAAACGGATGGGAGTGCAACTGCAGCGATTC

AAGTGATCCTCTCGTTATTGCCGCAAGTATCATTGGGATCTTGCACTTGATATTGTGGAT

TCTTGATCGCCTTTTCTTCAAATGCGTTTATCGTCGCCTTAAATACGGTTTGAAAAGAGG

GCCTTCTACGGAAGGAGTACCTGAGTCCATGAGGGAAGAGTACCGGCAGGAACAGCAGAG

TGCTGTGGATGTTGACGATGGTCATTTTGTCAACATAGAGCTGGAGTAAAAAACTACCTT

GTTTCTACT----------------------------------

>A_goose_Omsk_30009_2020_EPI1814316

--AGCAAAAGCAGGTAGATGTTGAAAGATGAGTCTTCTAACCGAGGTCGAAACGTACGTT

CTCTCTATCGTCCCGTCAGGCCCCCTCAAAGCCGAGATCGCGCAGAGACTTGAAGATGTC

TTTGCAGGGAAGAACACCGATCTTGAGGCTCTCATGGAATGGCTAAAGACAAGACCAATC

CTGTCACCTCTGACTAAGGGGATTTTGGGATTTGTGTTCACGCTCACCGTGCCCAGTGAG

CGAGGACTGCAGCGTAGACGCTTTGTCCAAAATGCTCTAAATGGAAATGGAGACCCAAAC

AACATGGACAGGGCAGTCAAACTGTACAGGAAATTGAAGAGAGAGATAACATTCCATGGG

GCTAAAGAAGTTGCACTCAGTTACTCAACCGGTGCACTTGCCAGTTGTATGGGTCTCATA

TACAACAGGATGGGGACGGTGACCGCAGAAGTGGCATTGGGCCTAGTGTGTGCCACCTGT

GAGCAGATTGCTGATTCACAGCATCGGTCTCACAGGCAGATAGCAACCACCACCAACCCA

CTAATCAGACATGAAAACAGAATGGTGTTGGCCAGTACTACAGCTAAGGCTATGGAGCAG

ATGGCTGGATCGAGTGAGCAAGCAGCGGAAGCCATGGAGGTTGCTAGTCAGGCTAGGCAG

ATGGTGCAGGCGATGAGGACCATTGGAACTCATCCTAGCTCCAGTGCCGGTCTGAGAGAT

GATCTCCTTGAAAATTTGCAGGCCTACCAAAAACGGATGGGAGTGCAACTGCAGCGATTC

AAGTGATCCTCTCGTTATTGCCGCAAGTATCATTGGGATCTTGCACTTGATATTGTGGAT

TCTTGATCGCCTTTTCTTCAAATGCGTTTATCGTCGCCTTAAATACGGTTTGAAAAGAGG

GCCTTCTACGGAAGGAGTACCTGAATCCATGAGGGAAGAGTACCGGCAGGAACAGCAGAG

TGCTGTGGATGTTGACGATGGTCATTTTGTCAACATAGAGCTGGAGTAAAAAACTACCTT

GTTTCTACT----------------------------------

>A_chicken_Chelyabinsk_201_2020_EPI1814332

--AGCAAAAGCAGGTAGATATTGAAAGATGAGTCTTCTAACCGAGGTCGAAACGTACGTT

CTCTCTATCGTCCCGTCAGGCCCCCTCAAAGCCGAGATCGCGCAGAGACTTGAAGATGTC

TTTGCAGGGAAGAACACCGATCTTGAGGCTCTCATGGAATGGCTAAAGACAAGACCAATC

CTGTCACCTCTGACTAAGGGGATTTTGGGATTTGTGTTCACGCTCACCGTGCCCAGTGAG

CGAGGACTGCAGCGTAGACGCTTTGTCCAAAATGCTCTAAATGGAAATGGAGACCCGAAC

AACATGGACAGGGCAGTCAAACTGTACAGGAAATTGAAGAGAGAGATAACATTCCATGGG

GCTAAAGAAGTTGCACTCAGTTACTCAACCGGTGCACTTGCCAGTTGTATGGGTCTCATA

TACAACAGGATGGGGACGGTGACCGCAGAAGTGGCATTGGGCCTAGTGTGTGCCACCTGT

GAGCAGATTGCTGATTCACAGCATCGGTCTCACAGACAGATAGCAACCACCACCAACCCA

CTAATCAGACATGAAAACAGAATGGTGTTGGCCAGTACTACAGCTAAGGCTATGGAGCAG

ATGGCTGGATCGAGTGAGCAAGCAGCGGAAGCCATGGAGGTTGCTAGTCAGGCTAGGCAG

ATGGTGCAGGCGATGAGGACCATTGGAACTCATCCTAGCTCCAGTGCCGGTCTGAGAGAT

GATCTCCTTGAAAATTTGCAGGCCTACCAAAAACGGATGGGAGTGCAACTGCAGCGATTC

AAGTGATCCTCTCGTTATTGCCGCAAGTATCATTGGGATCTTGCACTTGATATTGTGGAT

TCTTGATCGCCTTTTCTTCAAATGCGTTTATCGTCGCCTTAAATACGGTTTGAAAAGAGG

GCCTTCTACGGAAGGAGTACCTGAGTCCATGAGGGAAGAGTACCGGCAGGAACAGCAGAG

TGCTGTGGATGTTGACGATGGTCATTTTGTCAACATAGAGCTGGAGTAAAAAACTACCTT

GTTTCTACT----------------------------------

>A_chicken_Kurgan_1005_2020_EPI1814364

--AGCAAAAGCAGGTAGATATTGAAAGATGAGTCTTCTAACCGAGGTCGAAACGTACGTT

CTCTCTATCGTCCCGTCAGGCCCCCTCAAAGCCGAGATCGCGCAGAGACTTGAAGATGTC

TTTGCAGGGAAGAACACCGATCTTGAGGCTCTCATGGAATGGCTAAAGACAAGACCAATC

CTGTCACCTCTGACTAAGGGGATTTTGGGATTTGTGTTCACGCTCACCGTGCCCAGTGAG

CGAGGACTGCAGCGTAGACGCTTTGTCCAAAATGCTCTAAATGGAAATGGAGACCCAAAC

AACATGGACAGGGCAGTCAAACTGTACAGGAAATTGAAGAGAGAGATAACATTCCATGGG

GCTAAAGAAGTTGCACTCAGTTACTCAACCGGTGCACTTGCCAGTTGTATGGGTCTCATA

TACAACAGGATGGGGACGGTGACCGCAGAAGTGGCATTGGGCCTAGTGTGTGCCACCTGT

GAGCAGATTGCTGATTCACAGCATCGGTCTCACAGACAGATAGCAACCACCACCAACCCA

CTAATCAGACATGAAAACAGAATGGTGTTGGCCAGTACTACAGCTAAGGCTATGGAGCAG

ATGGCTGGATCGAGTGAGCAAGCAGCGGAAGCCATGGAGGTTGCTAGTCAGGCTAGGCAG

ATGGTGCAGGCGATGAGGACCATTGGAACTCATCCTAGCTCCAGTGCCGGTCTGAGAGAT

GATCTCCTTGAAAATTTGCAGGCCTACCAAAAACGGATGGGAGTGCAACTGCAGCGATTC

AAGTGATCCTCTCGTTATTGCCGCAAGTATCATTGGGATCTTGCACTTGATATTGTGGAT

TCTTGATCGCCTTTTCTTCAAATGCGTTTATCGTCGCCTTAAATACGGTTTGAAAAGAGG

GCCTTCTACGGAAGGAGTACCTGAGTCCATGAGGGAAGAGTACCGGCAGGAACAGCAGAG

TGCTGTGGATGTTGACGATGGTCATTTTGTCAACATAGAGCTGGAGTAAAAAACTACCTT

GTTTCTACT----------------------------------

>A_turkey_Poland_464_2020_EPI1841313

---------------AGATATTGAAAGATGAGTCTTCTAACCGAGGTCGAAACGTACGTT

CTCTCTATCGTCCCGTCAGGCCCCCTCAAAGCCGAGATCGCGCAGAGACTTGAAGATGTC

TTTGCAGGGAAGAACACCGATCTTGAGGCTCTCATGGAATGGCTAAAGACAAGACCAATC

CTGTCACCTCTGACTAAGGGGATTTTAGGATTTGTGTTCACGCTCACCGTGCCCAGTGAG

CGAGGACTGCAGCGTAGACGCTTTGTCCAAAATGCTCTAAATGGAAATGGAGACCCAAAC

AACATGGACAGGGCAGTCAGACTGTACAGGAAATTGAAGAGAGAGATAACATTCCATGGG

GCTAAAGAAGTTGCACTCAGTTACTCAACCGGTGCACTTGCCAGTTGTATGGGTCTCATA

TACAACAGGATGGGGACGGTGACCGCAGAAGTGGCATTGGGCCTAGTGTGTGCCACCTGT

GAGCAGATTGCTGATTCACAGCATCGGTCTCACAGACAAATAGCAACCACCACCAACCCA

CTAATCAGACATGAAAACAGAATGGTATTGGCCAGTACTACAGCTAAGGCTATGGAGCAG

ATGGCTGGATCGAGTGAGCAAGCAGCGGAAGCCATGGAGGTTGCCAGTCAGGCTAGGCAG

ATGGTGCAGGCGATGAGGACCATTGGAACTCATCCTAGCTCCAGTGCCGGTCTGAGAGAT

GATCTCCTTGAAAATTTGCAGGCCTACCAAAAACGGATGGGAGTGCAACTGCAGCGATTC

AAGTGATCCTCTCGTTATTGCCGCAAGTATCATTGGGATCTTGCACTTGATATTGTGGAT

TCTTGATCGCCTTTTCTTCAAATGCGTTTATCGTCGCCTTAAATACGGTTTGAAAAGAGG

GCCTTCTACGGAAGGAGTACCTGAGTCCATGAGGGAAGAGTACCGGCAGAGACAGCAGAG

TGCTGTGGATGTTGACGATGGTCATTTTGTCAACATAGAGCTGGAGTAAAAAACTA----

-------------------------------------------

>A_duck_Northern_China_ZGL_2020_EPI1844093

--AGCAAAAGCAGGTAGATATTGAAAGATGAGTCTTCTAACCGAGGTCGAAACGTACGTT

CTCTCTATCGTCCCGTCAGGCCCCCTCAAAGCCGAGATCGCGCAGAGACTTGAAGATGTC

TTTGCAGGGAAGAACACCGATCTTGAGGCTCTCATGGAATGGCTAAAGACAAGACCAATC

CTGTCACCTCTGACTAAGGGGATTTTGGGATTTGTGTTCACGCTCACCGTGCCCAGTGAG

CGAGGACTGCAGCGTAGACGCTTTGTCCAAAATGCTCTAAATGGAAATGGAGACCCAAAC

AACATGGACAGGGCAGTCAAACTGTACAGGAAACTGAAGAGAGAGATAACATTCCATGGG

GCTAAAGAAGTTGCACTCAGTTACTCAACCGGTGCACTTGCCAGTTGTATGGGTCTCATA

TACAACAGGATGGGGACGGTGACCGCAGAAGTGGCATTGGGCCTAGTGTGTGCCACCTGT

GAGCAGATTGCTGATTCACAGCATCGGTCTCACAGACAGATAGCAACCACCACCAACCCA

CTAATCAGACATGAAAACAGAATGGTGTTGGCCAGTACTACAGCTAAGGCCATGGAGCAG

ATGGCTGGATCGAGTGAGCAAGCAGCGGAAGCCATGGAGGTTGCCAGTCAGGCTAGGCAG

ATGGTGCAGGCGATGAGGACCATTGGAACTCATCCTAGCTCCAGTGCCGGTCTGAGAGAT

GATCTCCTTGAAAATTTGCAGGCCTACCAAAAACGGATGGGAGTGCAACTGCAGCGATTC

AAGTGATCCTCTCGTTATTGCCGCAAGTATCATTGGGATCTTGCACTTGATATTGTGGAT

TCTTGATCGCCTTTTCTTCAAATGCGTTTATCGTCGCCTTAAATACGGTTTGAAAAGAGG

GCCTTCTACGGAAGGAGTACCTGAGTCCATGAGGGAAGAGTACCGGCAGGAACAGCAGAA

TGCTGTGGATGTTGACGATGGTCATTTTGTCAACATAGAGCTGGAGTAAAAAACTACCTT

GTTTCTACT----------------------------------

>A_duck_Southwestern_China_B1904_2020_EPI1844104

--AGCAAAAGCAGGTAGATATTGAAAGATGAGTCTTCTAACCGAGGTCGAAACGTACGTT

CTCTCCATCGTCCCGTCAGGCCCCCTCAAAGCCGAGATCGCGCAGAGACTTGAAGATGTC

TTTGCAGGGAAGAACACCGATCTTGAGGCTCTCATGGAATGGCTAAAGACAAGACCAATC

CTGTCACCTCTGACTAAGGGGATTTTGGGATTTGTGTTCACGCTCACCGTGCCCAGTGAG

CGAGGACTGCAGCGTAGACGCTTTGTCCAAAATGCTCTAAATGGAAATGGAGACCCAAAC

AACATGGACAGGGCAGTCAAACTGTACAGGAAACTGAAGAGAGAGATAACATTCCATGGG

GCTAAAGAAGTTGCACTCAGTTACTCAACCGGTGCACTTGCCAGTTGTATGGGTCTCATA

TACAACAGGATGGGGACGGTGACCGCAGAAGTGGCATTGGGCCTAGTGTGTGCCACCTGT

GAGCAGATTGCTGATTCACAGCATCGGTCTCACAGACAGATAGCAACCACCACCAACCCA

CTAATCAGACATGAAAACAGAATGGTGTTGGCCAGTACTACAGCTAAGGCTATGGAGCAG

ATGGCTGGATCGAGTGAGCAAGCAGCGGAAGCCATGGAGGTTGCCAGTCAGGCTAGGCAG

ATGGTGCAGGCGATGAGGACCATTGGAACTCATCCTAGCTCCAGTGCCGGTCTGAGAGAT

GATCTCCTTGAAAATTTGCAGGCCTACCAAAAACGGATGGGAGTGCAACTGCAGCGATTC

AAGTGATCCTCTCGTTATTGCCGCAAGTATCATTGGGATCTTGCACTTGATATTGTGGAT

TCTTGATCGCCTTTTCTTCAAATGCGTTTATCGTCGCCTTAAATACGGTTTGAAAAGAGG

GCCTTCTACGGAAGGAGTACCTGAGTCCATGAGGGAAGAGTACCGGCAGGAACAGCAGAG

TGCTGTGGATGTTGACGATGGTCATTTTGTCAACATAGAGCTGGAGTAAAAAACTACCTT

GTTTCTACT----------------------------------

>A_duck_Korea_H411_2020_EPI1845932

---------------------------ATGAGTCTTCTAACCGAGGTCGAAACGTACGTT

CTCTCTATCGTCCCGTCAGGCCCCCTCAAAGCCGAGATCGCGCAGAGACTTGAAGATGTC

TTTGCAGGGAAGAACACCGATCTTGAGGCTCTCATGGAATGGCTAAAGACAAGACCAATC

CTGTCACCTCTGACTAAGGGGATTTTGGGATTTGTGTTCACGCTCACCGTGCCCAGTGAG

CGAGGACTGCAGCGTAGACGCTTTGTCCAAAATGCTCTAAATGGAAATGGAGACCCAAAC

AACATGGACAGGGCAGTCAAACTGTACAGGAAACTGAAGAGAGAGATAACATTCCATGGG

GCTAAAGAAGTTGCACTCAGTTACTCAACCGGTGCACTTGCCAGTTGTATGGGTCTCATA

TACAACAGGATGGGGACGGTGACCGCAGAAGTGGCATTGGGCCTAGTGTGTGCCACCTGT

GAGCAGATTGCTGATTCACAGCATCGGTCTCACAGACAGATAGCAACCACCACCAACCCA

CTAATCAGACATGAAAACAGAATGGTGTTGGCCAGTACTACAGCTAAGGCTATGGAGCAG

ATGGCTGGATCGAGTGAGCAAGCAGCGGAAGCCATGGAGGTTGCCAGTCAGGCTAGGCAG

ATGGTGCAGGCGATGAGGACCATTGGAACTCATCCTAGCTCCAGTGCCGGTCTGAGAGAT

GATCTCCTTGAAAATTTGCAGGCCTACCAAAAACGGATGGGAGTGCAACTGCAGCGATTC

AAGTGATCCTCTCGTTATTGCCGCAAGTATCATTGGGATCTTGCACTTGATATTGTGGAT

TCTTGATCGCCTTTTCTTCAAATGCGTTTATCGTCGCCTTAAATACGGTTTGAAAAGAGG

GCCTTCTACGGAAGGAGTACCTGAGTCCATGAGGGAAGAGTACCGGCAGGAACAGCAGAG

TGCTGTGGATGTTGACGATGGTCATTTTGTCAACATAGAGCTGGAGTAA-----------

-------------------------------------------

>A_duck_Korea_H431_2020_EPI1845956

---------------------------ATGAGTCTTCTAACCGAGGTCGAAACGTACGTT

CTCTCTATCGTCCCGTCAGGCCCCCTCAAAGCCGAGATCGCGCAGAGACTTGAAGATGTC

TTTGCAGGGAAGAACACCGATCTTGAGGCTCTCATGGAATGGCTAAAGACAAGACCAATC

CTGTCACCTCTGACTAAGGGGATTTTGGGATTTGTGTTCACGCTCACCGTGCCCAGTGAG

CGAGGACTGCAGCGTAGACGCTTTGTCCAAAATGCTCTAAATGGAAATGGAGACCCAAAC

AACATGGACAGGGCAGTCAAACTGTACAGGAAACTGAAGAGAGAGATAACATTCCATGGG

GCTAAAGAAGTTGCACTCAGTTACTCAACCGGTGCACTTGCCAGTTGTATGGGTCTCATA

TACAACAGGATGGGGACGGTGACCGCAGAAGTGGCATTGGGCCTAGTGTGTGCCACCTGT

GAGCAGATTGCTGATTCACAGCATCGGTCTCACAGACAGATAGCAACCACCACCAACCCA

CTAATCAGACATGAAAACAGAATGGTGTTGGCCAGTACTACAGCTAAGGCTATGGAGCAG

ATGGCTGGATCGAGTGAGCAAGCAGCGGAAGCCATGGAGGTTGCCAGTCAGGCTAGGCAG

ATGGTGCAGGCGATGAGAACCATTGGAACTCATCCTAGCTCCAGTGCCGGTCTGAGAGAT

GATCTCCTTGAAAATTTGCAGGCCTACCAAAAACGGATGGGAGTGCAACTGCAGCGATTC

AAGTGATCCTCTCGTTATTGCCGCAAGTATCATTGGGATCTTGCACTTGATATTGTGGAT

TCTTGATCGCCTTTTCTTCAAATGCGTTTATCGTCGCCTTAAATACGGTTTGAAAAGAGG

GCCTTCTACGGAAGGAGTACCTGAGTCCATGAGGGAAGAGTACCGGCAGGAACAGCAGAG

TGCTGTGGATGTTGACGATGGTCATTTTGTCAACATAGAGCTGGAGTAA-----------

-------------------------------------------

>A_duck_Korea_H471_2020_EPI1846028

---------------------------ATGAGTCTTCTAACCGAGGTCGAAACGTACGTT

CTCTCTATCGTCCCGTCAGGCCCCCTCAAAGCCGAGATCGCGCAGAGACTTGAAGATGTC

TTTGCAGGGAAGAACACCGATCTTGAGGCTCTCATGGAATGGCTAAAGACAAGACCAATC

CTGTCACCTCTGACTAAGGGGATTTTGGGATTTGTGTTCACGCTCACCGTGCCCAGTGAG

CGAGGACTGCAGCGTAGACGCTTTGTCCAAAATGCTCTAAATGGAAATGGAGACCCAAAC

AACATGGACAGGGCAGTCAAACTGTACAGGAAACTGAAGAGAGAGATAACATTCCATGGG

GCTAAAGAAGTTGCACTCAGTTACTCAACCGGTGCACTTGCCAGTTGTATGGGTCTCATA

TACAACAGGATGGGGACGGTGACCGCAGAAGTGGCATTGGGCCTAGTGTGTGCCACCTGT

GAGCAGATTGCTGATTCACAGCATCGGTCTCACAGACAGATAGCAACCACCACCAACCCA

CTAATCAGACATGAAAACAGAATGGTGTTGGCCAGTACTACAGCTAAGGCTATGGAGCAG

ATGGCTGGATCGAGTGAGCAAGCAGCGGAAGCCATGGAGGTTGCCAGTCAGGCTAGGCAG

ATGGTGCAGGCGATGAGAACCATTGGAACTCATCCTAGCTCCAGTGCCGGTCTGAGAGAT

GATCTCCTTGAAAATTTGCAGGCCTACCAAAAACGGATGGGAGTGCAACTGCAGCGATTC

AAGTGATCCTCTCGTTATTGCCGCAAGTATCATTGGGATCTTGCACTTGATATTGTGGAT

TCTTGATCGCCTTTTCTTCAAATGCGTTTATCGTCGCCTTAAATACGGTTTGAAAAGAGG

GCCTTCTACGGAAGGAGTACCTGAGTCCATGAGGGAAGAGTACCGGCAGGAACAGCAGAG

TGCTGTGGATGTTGACGATGGTCATTTTGTCAACATAGAGCTGGAGTAA-----------

-------------------------------------------

>A_chicken_Korea_H510_2020_EPI1846052

---------------------------ATGAGTCTTCTAACCGAGGTCGAAACGTACGTT

CTCTCTATCGTCCCGTCAGGCCCCCTCAAAGCCGAGATCGCGCAGAGACTTGAAGATGTC

TTTGCAGGGAAGAACACCGATCTTGAGGCTCTCATGGAATGGCTAAAGACAAGACCAATC

CTGTCACCTCTGACTAAGGGGATTTTGGGATTTGTGTTCACGCTCACCGTGCCCAGTGAG

CGAGGACTGCAGCGTAGACGCTTTGTCCAAAATGCTCTAAATGGAAATGGAGACCCAAAC

AACATGGACAGGGCAGTCAAACTGTACAGGAAACTGAAGAGAGAGATAACATTCCATGGG

GCTAAAGAAGTTGCACTCAGTTACTCAACCGGTGCACTTGCCAGTTGTATGGGTCTCATA

TACAACAGGATGGGGACGGTGACCGCAGAAGTGGCATTGGGCCTAGTGTGTGCCACCTGT

GAGCAGATTGCTGATTCACAGCATCGGTCTCACAGACAGATAGCAACCACCACCAACCCA

CTAATCAGACATGAAAACAGAATGGTGTTGGCCAGTACTACAGCTAAGGCTATGGAGCAG

ATGGCTGGATCGAGTGAGCAAGCAGCGGAAGCCATGGAGGTTGCCAGTCAGGCTAGGCAG

ATGGTGCAGGCGATGAGGACCATTGGAACTCATCCTAGCTCCAGTGCCGGTCTGAGAGAT

GATCTCCTTGAAAATTTGCAGGCCTACCAAAAACGGATGGGAGTGCAACTGCAGCGATTC

AAGTGATCCTCTCGTTATTGCCGCAAGTATCATTGGGATCTTGCACTTGATATTGTGGAT

TCTTGATCGCCTTTTCTTCAAATGCGTTTATCGTCGCCTTAAATACGGTTTGAAAAGAGG

GCCTTCTACGGAAGGAGTACCTGAGTCCATGAGGGAAGAGTACCGGCAGGAACAGCAGAG

TGCTGTGGATGTTGACGATGGTCATTTTGTCAACATAGAGCTGGAGTAA-----------

-------------------------------------------

>A_duck_Korea_H538_2020_EPI1846148

---------------------------ATGAGTCTTCTAACCGAGGTCGAAACGTACGTT

CTCTCTATCGTCCCGTCAGGCCCCCTCAAAGCCGAGATCGCGCAGAGACTTGAAGATGTC

TTTGCAGGGAAGAACACCGATCTTGAGGCTCTCATGGAATGGCTAAAGACAAGACCAATC

CTGTCACCTCTGACTAAGGGGATTTTGGGATTTGTGTTCACGCTCACCGTGCCCAGTGAG

CGAGGACTGCAGCGTAGACGCTTTGTCCAAAATGCTCTAAATGGAAATGGAGACCCAAAC

AACATGGACAGGGCAGTCAAACTGTACAGGAAACTGAAGAGAGAGATAACATTCCATGGG

GCTAAAGAAGTTGCACTCAGTTACTCAACCGGTGCACTTGCCAGTTGTATGGGTCTCATA

TACAACAGGATGGGGACGGTGACCGCAGAAGTGGCATTGGGCCTAGTGTGTGCCACCTGT

GAGCAGATTGCTGATTCACAGCATCGGTCTCACAGACAGATAGCAACCACCACCAACCCA

CTAATCAGACATGAAAACAGAATGGTGTTGGCCAGTACTACAGCTAAGGCTATGGAGCAG

ATGGCTGGATCGAGTGAGCAAGCAGCGGAAGCCATGGAGGTTGCCAGTCAGGCTAGGCAG

ATGGTGCAGGCGATGAGAACCATTGGAACTCATCCTAGCTCCAGTGCCGGTCTGAGAGAT

GATCTCCTTGAAAATTTGCAGGCCTACCAAAAACGGATGGGAGTGCAACTGCAGCGATTC

AAGTGATCCTCTCGTTATTGCCGCAAGTATCATTGGGATCTTGCACTTGATATTGTGGAT

TCTTGATCGCCTTTTCTTCAAATGCGTTTATCGTCGCCTTAAATACGGTTTGAAAAGAGG

GCCTTCTACGGAAGGAGTACCTGAGTCCATGAGGGAAGAGTACCGGCAGGAACAGCAGAG

TGCTGTGGATGTTGACGATGGTCATTTTGTCAACATAGAGCTGGAGTAA-----------

-------------------------------------------

>A_chicken_Tyumen_302-01_2020_EPI1848601

--AGCAAAAGCAGGTAGATATTGAAAGATGAGTCTTCTAACCGAGGTCGAAACGTACGTT

CTCTCTATCGTCCCGTCAGGCCCCCTCAAAGCCGAGATCGCGCAGAGACTTGAAGATGTC

TTTGCAGGGAAGAACACCGATCTTGAGGCTCTCATGGAATGGCTAAAGACAAGACCAATC

CTGTCACCTCTGACTAAGGGGATTTTGGGATTTGTGTTCACGCTCACCGTGCCCAGTGAG

CGAGGACTGCAGCGTAGACGCTTTGTCCAAAATGCTCTAAATGGAAATGGAGACCCAAAC

AACATGGACAGGGCAGTCAAACTGTACAGGAAATTGAAGAGAGAGATAACATTCCATGGG

GCTAAAGAAGTTGCACTCAGTTACTCAACCGGTGCACTTGCCAGTTGTATGGGTCTCATA

TACAACAGGATGGGGACGGTGACCGCAGAAGTGGCATTGGGCCTAGTGTGTGCCACCTGT

GAACAGATTGCTGATTCACAGCATCGGTCTCACAGACAGATAGCAACCACCACCAACCCA

CTAATCAGACATGAAAACAGAATGGTGTTGGCCAGTACTACAGCTAAGGCTATGGAGCAG

ATGGCTGGATCGAGTGAGCAAGCAGCGGAAGCCATGGAGGTTGCTAGTCAGGCTAGGCAG

ATGGTGCAGGCGATGAGGACCATTGGAACTCATCCTAGCTCCAGTGCCGGTCTGAGAGAT

GATCTCCTTGAAAATTTGCAGGCCTACCAAAAACGGATGGGAGTGCAACTGCAGCGATTC

AAGTGATCCTCTCGTTATTGCCGCAAGTATCATTGGGATCTTGCACTTGATATTGTGGAT

TCTTGATCGCCTTTTCTTCAGATGCGTTTATCGTCGCCTTAAATACGGTTTGAAAAGAGG

GCCTTCTACGGAAGGAGTACCTGAGTCCATGAGGGAAGAGTACCGGCAGGAACAGCAGAG

TGCTGTGGATGTTGACGATGGTCATTTTGTCAACATAGAGCTGGAGTAAAAAACTACCTT

GTTTCTACT----------------------------------

>A_chicken_Tyumen_302-02_2020_EPI1848609

--AGCAAAAGCAGGTAGATATTGAAAGATGAGTCTTCTAACCGAGGTCGAAACGTACGTT

CTCTCTATCGTCCCGTCAGGCCCCCTCAAAGCCGAGATCGCGCAGAGACTTGAAGATGTC

TTTGCAGGGAAGAACACCGATCTTGAGGCTCTCATGGAATGGCTAAAGACAAGACCAATC

CTGTCACCTCTGACTAAGGGGATTTTGGGATTTGTGTTCACGCTCACCGTGCCCAGTGAG

CGAGGACTGCAGCGTAGACGCTTTGTCCAAAATGCTCTAAATGGAAATGGAGACCCAAAC

AACATGGACAGGGCAGTCAAACTGTACAGGAAATTGAAGAGAGAGATAACATTCCATGGG

GCTAAAGAAGTTGCACTCAGTTACTCAACCGGTGCACTTGCCAGTTGTATGGGTCTCATA

TACAACAGGATGGGGACGGTGACCGCAGAAGTGGCATTGGGCCTAGTGTGTGCCACCTGT

GAACAGATTGCTGATTCACAGCATCGGTCTCACAGACAGATAGCAACCACCACCAACCCA

CTAATCAGACATGAAAACAGAATGGTGTTGGCCAGTACTACAGCTAAGGCTATGGAGCAG

ATGGCTGGATCGAGTGAGCAAGCAGCGGAAGCCATGGAGGTTGCTAGTCAGGCTAGGCAG

ATGGTGCAGGCGATGAGGACCATTGGAACTCATCCTAGCTCCAGTGCCGGTCTGAGAGAT

GATCTCCTTGAAAATTTGCAGGCCTACCAAAAACGGATGGGAGTGCAACTGCAGCGATTC

AAGTGATCCTCTCGTTATTGCCGCAAGTATCATTGGGATCTTGCACTTGATATTGTGGAT

TCTTGATCGCCTTTTCTTCAGATGCGTTTATCGTCGCCTTAAATACGGTTTGAAAAGAGG

GCCTTCTACGGAAGGAGTACCTGAGTCCATGAGGGAAGAGTACCGGCAGGAACAGCAGAG

TGCTGTGGATGTTGACGATGGTCATTTTGTCAACATAGAGCTGGAGTAAAAAACTACCTT

GTTTCTACT----------------------------------

>A_chicken_Poland_474_2020_EPI1850194

---------------AGATATTGAAAGATGAGTCTTCTAACCGAGGTCGAAACGTACGTT

CTCTCTATCGTCCCGTCAGGCCCCCTCAAAGCCGAGATCGCGCAGAGACTTGAAGATGTC

TTTGCAGGGAAGAACACCGATCTTGAGGCTCTCATGGAATGGCTAAAGACAAGACCAATC

CTGTCACCTCTGACTAAGGGGATTTTGGGATTTGTGTTCACGCTCACCGTGCCCAGTGAG

CGAGGACTGCAGCGTAGACGCTTTGTCCAAAATGCTCTAAATGGAAATGGAGACCCAAAC

AACATGGACAGGGCAGTCAAACTGTACAGGAAATTGAAGAGAGAGATAACATTCCATGGG

GCTAAAGAAGTTGCACTCAGTTACTCAACCGGTGCACTTGCCAGTTGTATGGGTCTCATA

TACAACAGGATGGGGACGGTGACCGCAGAAGTGGCATTGGGCCTAGTGTGTGCCACCTGT

GAGCAGATTGCTGATTCACAGCATCGGTCTCACAGACAGATAGCAACCACCACCAACCCA

CTAATCAGACATGAAAACAGAATGGTGTTGGCCAGTACTACAGCTAAGGCTATGGAGCAG

ATGGCTGGATCGAGTGAGCAAGCAGCGGAAGCCATGGAGGTTGCTAGTCAGGCTAGGCAG

ATGGTGCAGGCGATGAGGACCATTGGAACTCATCCTAGCTCCAGTGCCGGTCTGAGAGAT

GATCTCCTTGAAAATTTGCAGGCCTACCAAAAACGGATGGGAGTGCAACTGCAGCGATTC

AAGTGATCCTCTCGTTATTGCCGCAAGTATCATTGGGATCTTGCACTTGATATTGTGGAT

TCTTGATCGCCTTTTCTTCAAATGCGTTTATCGTCGCCTTAAATACGGTTTGAAAAGAGG

GCCTTCTACGGAAGGAGTACCTGAGTCCATGAGGGAAGAGTACCGGCAGGAACAGCAGAG

TGCTGTGGATGTTGACGATGGTCATTTTGTCAACATAGAGCTGGAGTAAAAAACTA----

-------------------------------------------

>A_swan_Poland_MB141_2020_EPI1850212

---------------AGATATTGAAAGATGAGTCTTCTAACCGAGGTCGAAACGTACGTT

CTCTCTATCGTCCCGTCAGGCCCCCTCAAAGCCGAGATCGCGCAGAGACTTGAAGATGTC

TTTGCAGGGAAGAACACCGATCTTGAGGCTCTCATGGAATGGCTAAAGACAAGACCAATC

CTGTCACCTCTGACTAAGGGGATTTTGGGATTTGTGTTCACGCTCACCGTGCCCAGTGAG

CGAGGACTGCAGCGTAGACGCTTTGTCCAAAATGCTCTAAATGGAAATGGAGACCCAAAC

AACATGGACAGGGCAGTCAAACTGTACAGGAAATTGAAGAGAGAGATAACATTCCATGGG

GCTAAAGAAGTTGCACTCAGTTACTCAACCGGTGCACTTGCCAGTTGTATGGGTCTCATA

TACAACAGGATGGGGACGGTGACCGCAGAAGTGGCATTGGGCCTAGTGTGTGCCACCTGT

GAGCAGATTGCTGATTCACAGCATCGGTCTCACAGACAGATAGCAACCACCACCAACCCA

CTAATCAGACATGAAAACAGAATGGTGTTGGCCAGTACTACAGCCAAGGCTATGGAGCAG

ATGGCTGGATCGAGTGAGCAAGCAGCGGAAGCCATGGAGGTTGCTAGTCAGGCTAGGCAG

ATGGTGCAGGCGATGAGGACCATTGGAACTCATCCTAGCTCCAGTGCCGGTCTGAGAGAT

GATCTCCTTGAAAATTTGCAGGCCTACCAAAAACGGATGGGAGTGCAACTGCAGCGATTC

AAGTGATCCTCTCGTTATTGCCGCAAGTATCATTGGGATCTTGCACTTGATATTGTGGAT

TCTTGATCGCCTTTTCTTCAAATGCGTTTATCGTCGCCTTAAATACGGTTTGAAAAGAGG

GCCTTCTACGGAAGGAGTACCTGAGTCCATGAGGGAAGAGTACCGGCAGGAACAGCAGAG

TGCTGTGGATGTTGACGATGGTCATTTTGTCAACATAGAGCTGGAGTAAAAAACTA----

-------------------------------------------

>A_muscovy_duck_Slovakia_Pah1_21VIR1086-1_2021_EPI1858239

---------------------------ATGAGTCTTCTAACCGAGGTCGAAACGTACGTT

CTCTCTATCGTCCCGTCAGGCCCCCTCAAAGCCGAGATCGCGCAGAGACTTGAAGATGTC

TTTGCAGGGAAGAACACCGATCTTGAGGCTCTCATGGAATGGCTAAAGACAAGACCAATC

CTGTCACCTCTGACTAAGGGGATTTTGGGATTTGTGTTCACGCTCACCGTGCCCAGTGAG

CGAGGACTGCAGCGTAGACGCTTTGTCCAAAATGCTCTAAATGGAAATGGAGACCCAAAC

AACATGGACAGGGCAGTCAAACTGTACAGGAAATTGAAGAGAGAGATAACATTCCATGGG

GCTAAAGAAGTTGCACTCAGTTACTCAACCGGTGCACTTGCCAGTTGTATGGGTCTCATA

TACAACAGGATGGGGACGGTGACCGCAGAAGTGGCATTGGGCCTAGTGTGTGCCACCTGT

GAGCAGATTGCTGATTCACAGCATCGGTCTCACAGACAGATAGCAACCACCACCAACCCA

CTAATCAGACATGAAAACAGAATGGTGTTGGCCAGTACTACAGCTAAGGCTATGGAGCAG

ATGGCTGGATCGAGTGAGCAAGCAGCGGAAGCCATGGAGGTTGCTAGTCAGGCTAGGCAG

ATGGTGCAGGCGATGAGAACCATTGGAACTCATCCTAGCTCCAGTGCCGGTCTGAGAGAT

GATCTCCTTGAAAATTTGCAGGCCTACCAAAAACGGATGGGAGTGCAACTGCAGCGATTC

AAGTGATCCTCTCGCTATTGCCGCAAGCATCATTGGGATCTTGCACTTGATATTGTGGAT

TCTTGATCGCCTTTTCTTCAAATGCGTTTATCGTCGCCTTAAATACGGTTTGAAAAGAGG

GCCTTCTACGGAAGGAGTACCTGAGTCCATGAGGGAAGAGTACCGGCAGGAACAGCAGAG

TGCTGTGGATGTTGACGATGGTCATTTTGTCAACATAGAGCTGGAGTAA-----------

-------------------------------------------

>A_mute_swan_Slovenia_1639-20_21VIR959-1_2020_EPI1858295

---------------------------ATGAGTCTTCTAACCGAGGTCGAAACGTACGTT

CTCTCTATCGTCCCGTCAGGCCCCCTCAAAGCCGAGATCGCGCAGAGACTTGAAGATGTC

TTTGCAGGGAAGAACACCGATCTTGAGGCTCTCATGGAATGGCTAAAGACAAGACCAATC

CTGTCACCTCTGACTAAGGGGATTTTGGGATTTGTGTTCACGCTCACCGTGCCCAGTGAG

CGAGGACTGCAGCGTAGACGCTTTGTCCAAAATGCTCTAAATGGAAATGGAGACCCAAAC

AACATGGACAGGGCAGTCAAACTGTACAGGAAATTGAAGAGAGAGATAACATTCCATGGG

GCTAAAGAAGTTGCACTCAGTTACTCAACCGGTGCACTTGCCAGTTGTATGGGTCTCATA

TACAACAGGATGGGGACGGTGACCGCAGAAGTGGCATTGGGCCTAGTGTGTGCCACCTGT

GAGCAGATTGCTGATTCACAGCATCGGTCTCACAGACAAATAGCAACCACCACCAACCCA

CTAATCAGACATGAAAACAGAATGGTGTTGGCCAGTACTACAGCTAAGGCTATGGAGCAG

ATGGCTGGATCGAGTGAGCAAGCAGCGGAAGCCATGGAGGTTGCCAGTCAGGCTAGGCAG

ATGGTGCAGGCAATGAGGACCATTGGAACTCATCCTAGCTCCAGTGCCGGTCTGAGAGAT

GATCTCCTTGAAAATTTGCAGGCCTACCAAAAACGGATGGGAGTGCAACTGCAGCGATTC

AAGTGATCCTCTCGTTATTGCCGCAAGTATCATTGGGATCTTGCACTTGATATTGTGGAT

TCTTGATCGCCTTTTCTTCAAATGCGTTTATCGTCGCCTTAAATACGGTTTGAAAAGAGG

GCCTTCTACGGAAGGAGTACCTGAGTCCATGAGGGAAGAGTACCGGCAGGAACAGCAGAG

TGCTGTGGATGTTGACGATGGTCATTTTGTCAACATAGAGCTGGAGTAA-----------

-------------------------------------------

>A_goose_Kazakhstan_4-190-20-B-H5N8-1_2020_EPI1927652

--AGCAAAAGCAGGTAGATATTGAAAGATGAGTCTTCTAACCGAGGTCGAAACGTACGTT

CTCTCTATCGTCCCGTCAGGCCCCCTCAAAGCCGAGATCGCGCAGAGACTTGAAGATGTC

TTTGCAGGGAAGAACACCGATCTTGAGGCTCTCATGGAATGGCTAAAGACAAGACCAATC

CTGTCACCTCTGACTAAGGGGATTTTGGGATTTGTGTTCACGCTCACCGTGCCCAGTGAG

CGAGGACTGCAGCGTAGACGCTTTGTCCAAAATGCTCTAAATGGAAATGGAGACCCAAAC

AACATGGACAGGGCAGTCAAACTGTACAGGAAATTGAAGAGAGAGATAACATTCCATGGG

GCTAAAGAAGTTGCACTCAGTTACTCAACCGGTGCACTTGCCAGTTGTATGGGTCTCATA

TACAACAGGATGGGGACGGTGACCGCAGAAGTGGCATTGGGCCTAGTGTGTGCCACCTGT

GAGCAGATTGCTGACTCACAGCATCGGTCTCACAGACAGATAGCAACCACCACCAACCCA

CTAATCAGACATGAAAACAGAATGGTGTTGGCCAGTACTACAGCTAAGGCTATGGAGCAG

ATGGCTGGATCGAGTGAGCAAGCAGCAGAAGCCATGGAGGTTGCTAGTCAGGCTAGGCAG

ATGGTGCAGGCGATGAGGACCATTGGAACTCATCCTAGCTCCAGTGCCGGTCTGAGAGAT

GATCTCCTTGAAAATTTGCAGGCCTACCAAAAACGGATGGGAGTGCAACTGCAGCGATTC

AAGTGATCCTCTCGTTATTGCCGCAAGTATCATTGGGATCTTGCACTTGATATTGTGGAT

TCTTGATCGCCTTTTCTTCAAATGCGTTTATCGTCGCCTTAAATACGGTTTGAAAAGAGG

GCCTTCTACGGAAGGAGTACCTGAGTCCATGAGGGAAGAGTACCGGCAGGAACAGCAGAG

TGCTGTGGATGTTGACGATGGTCATTTTGTCAACATAGAGCTGGAGTAAAAAACTACCTT

GTTTCTACT----------------------------------

>A_chicken_Kazakhstan_220-B-2-H5N8-4_2020_EPI1927658

--AGCAAAAGCAGGTAGATATTGAAAGATGAGTCTTCTAACCGAGGTCGAAACGTACGTT

CTCTCTATCGTCCCGTCAGGCCCCCTCAAAGCCGAGATCGCGCAGAGACTTGAAGATGTC

TTTGCAGGGAAGAACACCGATCTTGAGGCTCTCATGGAATGGCTAAAGACAAGACCAATC

CTGTCACCTCTGACTAAGGGGATTTTGGGATTTGTGTTCACGCTCACCGTGCCCAGTGAG

CGAGGACTGCAGCGTAGACGCTTTGTCCAAAATGCTCTAAATGGAAATGGAGACCCAAAC

AACATGGACAGGGCAGTCAAACTGTACAGGAAATTGAAGAGAGAGATAACATTCCATGGG

GCTAAAGAAGTTGCACTCAGTTACTCAACCGGTGCACTTGCCAGTTGTATGGGTCTCATA

TACAACAGGATGGGGACGGTGACCGCAGAAGTGGCATTGGGCCTAGTGTGTGCCACCTGT

GAGCAGATTGCTGACTCACAGCATCGGTCTCACAGACAGATAGCAACCACCACCAACCCA

CTAATCAGACATGAAAACAGAATGGTGTTGGCCAGTACTACAGCTAAGGCTATGGAGCAG

ATGGCTGGATCGAGTGAGCAAGCAGCAGAAGCCATGGAGGTTGCTAGTCAGGCTAGGCAG

ATGGTGCAGGCGATGAGGACCATTGGAACTCATCCTAGCTCCAGTGCCGGTCTGAGAGAT

GATCTCCTTGAAAATTTGCAGGCCTACCAAAAACGGATGGGAGTGCAACTGCAGCGATTC

AAGTGATCCTCTCGTTATTGCCGCAAGTATCATTGGGATCTTGCACTTGATATTGTGGAT

TCTTGATCGCCTTTTCTTCAAATGCGTTTATCGTCGCCTTAAATACGGTTTGAAAAGAGG

GCCTTCTACGGAAGGAGTACCTGAGTCCATGAGGGAAGAGTACCGGCAGGAACAGCAGAG

TGCTGTGGATGTTGACGATGGTCATTTTGTCAACATAGAGCTGGAGTAAAAAACTACCTT

GTTTCTACT----------------------------------

>A_duck_Kazakhstan_12-20-B-Talg-11_2020_EPI1927664

--AGCAAAAGCAGGTAGATATTGAAAGATGAGTCTTCTAACCGAGGTCGAAACGTACGTT

CTCTCTATCGTCCCGTCAGGCCCCCTCAAAGCCGAGATCGCGCAGAGACTTGAAGATGTC

TTTGCAGGGAAGAACACCGATCTTGAGGCTCTCATGGAATGGCTAAAGACAAGACCAATC

CTGTCACCTCTGACTAAGGGGATTTTGGGATTTGTGTTCACGCTCACCGTGCCCAGTGAG

CGAGGACTGCAGCGTAGACGCTTTGTCCAAAATGCTCTAAATGGAAATGGAGACCCAAAC

AACATGGACAGGGCAGTCAAATTGTACAGGAAATTGAAGAGAGAGATAACATTCCATGGG

GCTAAAGAAGTTGCACTCAGTTACTCAACCGGTGCACTTGCCAGTTGTATGGGTCTCATA

TACAACAGGATGGGGACGGTGACCGCAGAAGTGGCATTGGGCCTAGTGTGTGCCACCTGT

GAGCAGATTGCTGATTCACAGCATCGGTCTCACAGACAGATTGCAACCACCACCAATCCA

CTAATCAGACATGAAAACAGAATGGTGTTGGCCAGTACTACAGCTAAGGCTATGGAGCAG

ATGGCTGGATCGAGTGAGCAAGCAGCGGAAGCCATGGAGGTTGCTAGTCAGGCTAGGCAG

ATGGTGCAGGCGATGAGGACCATTGGAACTCATCCTAGCTCCAGTGCCGGTCTGAGAGAT

GATCTCCTTGAAAATTTGCAGGCCTACCAAAAACGGATGGGAGTGCAACTGCAGCGATTC

AAGTGATCCTCTCGTTATTGCCGCAAGTATCATTGGGATCTTGCACTTGATATTGTGGAT

TCTTGATCGCCTTTTCTTCAAATGCGTTTATCGTCGCCTTAAATACGGTTTGAAAAGAGG

GCCTTCTACGGAAGGAGTACCTGAGTCCATGAGGGAAGAGTACCGGCAGGAACAGCAGAG

TGCTGTGGATGTTGACGATGGTCATTTTGTCAACATAGAGCTGGAGTAAAAAACTACCTT

GTTTCTACT----------------------------------

>A_goose_Kazakhstan_7-20-B-Talg-12_2020_EPI1927670

--AGCAAAAGCAGGTAGATATTGAAAGATGAGTCTTCTAACCGAGGTCGAAACGTACGTT

CTCTCTATCGTCCCGTCAGGCCCCCTCAAAGCCGAGATCGCGCAGAGACTTGAAGATGTC

TTTGCAGGGAAGAACACCGATCTTGAGGCTCTCATGGAATGGCTAAAGACAAGACCAATC

CTGTCACCTCTGACTAAGGGGATTTTGGGATTTGTGTTCACGCTCACCGTGCCCAGTGAG

CGAGGACTGCAGCGTAGACGCTTTGTCCAAAATGCTCTAAATGGAAATGGAGACCCAAAC

AACATGGACAGGGCAGTCAAATTGTACAGGAAATTGAAGAGAGAGATAACATTCCATGGG

GCTAAAGAAGTTGCACTCAGTTACTCAACCGGTGCACTTGCCAGTTGTATGGGTCTCATA

TACAACAGGATGGGGACGGTGACCGCAGAAGTGGCATTGGGCCTAGTGTGTGCCACCTGT

GAGCAGATTGCTGATTCACAGCATCGGTCTCACAGACAGATTGCAACCACCACCAATCCA

CTAATCAGACATGAAAACAGAATGGTGTTGGCCAGTACTACAGCTAAGGCTATGGAGCAG

ATGGCTGGATCGAGTGAGCAAGCAGCGGAAGCCATGGAGGTTGCTAGTCAGGCTAGGCAG

ATGGTGCAGGCGATGAGGACCATTGGAACTCATCCTAGCTCCAGTGCCGGTCTGAGAGAT

GATCTCCTTGAAAATTTGCAGGCCTACCAAAAACGGATGGGAGTGCAACTGCAGCGATTC

AAGTGATCCTCTCGTTATTGCCGCAAGTATCATTGGGATCTTGCACTTGATATTGTGGAT

TCTTGATCGCCTTTTCTTCAAATGCGTTTATCGTCGCCTTAAATACGGTTTGAAAAGAGG

GCCTTCTACGGAAGGAGTACCTGAGTCCATGAGGGAAGAGTACCGGCAGGAACAGCAGAG

TGCTGTGGATGTTGACGATGGTCATTTTGTCAACATAGAGCTGGAGTAAAAAACTACCTT

GTTTCTACT----------------------------------

>A_swan_Kazakhstan_9-20-B-Talg-39_2020_EPI1927698

--AGCAAAAGCAGGTAGATATTGAAAGATGAGTCTTCTAACCGAGGTCGAAACGTACGTT

CTCTCTATCGTCCCGTCAGGCCCCCTCAAAGCCGAGATCGCGCAGAGACTTGAAGATGTC

TTTGCAGGGAAGAACACCGATCTTGAGGCTCTCATGGAATGGCTAAAGACAAGACCAATC

CTGTCACCTCTGACTAAGGGGATTTTGGGATTTGTGTTCACGCTCACCGTGCCCAGTGAG

CGAGGACTGCAGCGTAGACGCTTTGTCCAAAATGCTCTAAATGGAAATGGAGACCCAAAC

AACATGGACAGGGCAGTCAAACTGTACAGGAAATTGAAGAGAGAGATAACATTCCATGGG

GCTAAAGAAGTTGCACTCAGTTACTCAACCGGTGCACTTGCCAGTTGTATGGGTCTCATA

TACAACAGGATGGGGACGGTGACCGCAGAAGTGGCATTGGGCCTAGTGTGTGCCACCTGT

GAGCAGATTGCTGATTCACAGCATCGGTCTCACAGACAGATAGCAACCACCACCAACCCA

CTAATCAGACATGAAAACAGAATGGTGTTGGCCAGTACTACAGCTAAGGCTATGGAGCAG

ATGGCTGGATCGAGTGAGCAAGCAGCGGAAGCCATGGAGGTTGCTAGTCAGGCTAGGCAG

ATGGTGCAGGCGATGAGGACCATTGGAACTCATCCTAGCTCCAGTGCCGGTCTGAGAGAT

GATCTCCTTGAAAATTTGCAGGCCTACCAAAAACGGATGGGAGTGCAACTGCAGCGATTC

AAGTGATCCTCTCGTTATTGCCGCAAGTATCATTGGGATCTTGCACTTGATATTGTGGAT

TCTTGATCGCCTTTTCTTCAAATGCGTTTATCGTCGCCTTAAATACGGTTTAAAAAGAGG

GCCTTCTACGGAAGGAGTACCTGAGTCCATGAGGGAAGAGTACCGGCAGGAACAGCAGAG

TGCTGTGGATGTTGACGATGGTCATTTTGTCAACATAGAGCTGGAGTAAAAAACTACCTT

GTTTCTACT----------------------------------

>A_chicken_Kazakhstan_12-20-B-Talg-45_2020_EPI1927704

--AGCAAAAGCAGGTAGATATTGAAAGATGAGTCTTCTAACCGAGGTCGAAACGTACGTT

CTCTCTATCGTCCCGTCAGGCCCCCTCAAAGCCGAGATCGCGCAGAGACTTGAAGATGTC

TTTGCAGGGAAGAACACCGATCTTGAGGCTCTCATGGAATGGCTAAAGACAAGACCAATC

CTGTCACCTCTGACTAAGGGGATTTTGGGATTTGTGTTCACGCTCACCGTGCCCAGTGAG

CGAGGACTGCAGCGTAGACGCTTTGTCCAAAATGCTCTAAATGGAAATGGAGACCCAAAC

AACATGGACAGGGCAGTCAAACTGTACAGGAAATTGAAGAGAGAGATAACATTCCATGGG

GCTAAAGAAGTTGCACTCAGTTACTCAACCGGTGCACTTGCCAGTTGTATGGGTCTCATA

TACAACAGGATGGGGACGGTGACCGCAGAAGTGGCATTGGGCCTAGTGTGTGCCACCTGT

GAGCAGATTGCTGATTCACAGCATCGGTCTCACAGACAGATAGCAACCACCACCAACCCA

CTAATCAGACATGAAAACAGAATGGTGTTGGCCAGTACTACAGCTAAGGCTATGGAGCAG

ATGGCTGGATCGAGTGAGCAAGCAGCGGAAGCCATGGAGGTTGCCAGTCAGGCTAGGCAG

ATGGTGCAGGCGATGAGGACCATTGGAACTCATCCTAGCTCCAGTGCCGGTCTGAGAGAT

GATCTCCTTGAAAATTTGCAGGCCTACCAAAAACGGATGGGAGTGCAACTGCAGCGATTC

AAGTGATCCTCTCGTTATTGCCGCAAGTATCATTGGGATCTTGCACTTGATATTGTGGAT

TCTTGATCGCCTTTTCTTCAAATGCGTTTATCGTCGCCTTAAATACGGTTTGAAAAGAGG

GCCTTCTACGGAAGGAGTACCTGAGTCCATGAGGGAAGAGTACCGGCAGGAACAGCAGAG

TGCTGTGGATGTTGACGATGGTCATTTTGTCAACATAGAGCTGGAGTAAAAAACTACCTT

GTTTCTACT----------------------------------

>A_crow_Kazakhstan_15-20-B-Talg-4_2020_EPI1927710

--AGCAAAAGCAGGTAGATATTGAAAGATGAGTCTTCTAACCGAGGTCGAAACGTACGTT

CTCTCTATCGTCCCGTCAGGCCCCCTCAAAGCCGAGATCGCGCAGAGACTTGAAGATGTC

TTTGCAGGGAAGAACACCGATCTTGAGGCTCTCATGGAATGGCTAAAGACAAGACCAATC

CTGTCACCTCTGACTAAGGGGATTTTGGGATTTGTGTTCACGCTCACCGTGCCCAGTGAG

CGAGGACTGCAGCGTAGACGCTTTGTCCAAAATGCTCTAAATGGAAATGGAGACCCAAAC

AACATGGACAGGGCAGTCAAATTGTACAGGAAATTGAAGAGAGAGATAACATTCCATGGG

GCTAAAGAAGTTGCACTCAGTTACTCAACCGGTGCACTTGCCAGTTGTATGGGTCTCATA

TACAACAGGATGGGGACGGTGACCGCAGAAGTGGCATTGGGCCTAGTGTGTGCCACCTGT

GAGCAGATTGCTGATTCACAGCATCGGTCTCACAGACAGATTGCAACCACCACCAATCCA

CTAATCAGACATGAAAACAGAATGGTGTTGGCCAGTACTACAGCTAAGGCTATGGAGCAG

ATGGCTGGATCGAGTGAGCAAGCAGCGGAAGCCATGGAGGTTGCTAGTCAGGCTAGGCAG

ATGGTGCAGGCGATGAGGACCATTGGAACTCATCCTAGCTCCAGTGCCGGTCTGAGAGAT

GATCTCCTTGAAAATTTGCAGGCCTACCAAAAACGGATGGGAGTGCAACTGCAGCGATTC

AAGTGATCCTCTCGTTATTGCCGCAAGTATCATTGGGATCTTGCACTTGATATTGTGGAT

TCTTGATCGCCTTTTCTTCAAATGCGTTTATCGTCGCCTTAAATACGGTTTGAAAAGAGG

GCCTTCTACGGAAGGAGTACCTGAGTCCATGAGGGAAGAGTACCGGCAGGAACAGCAGAG

TGCTGTGGATGTTGACGATGGTCATTTTGTCAACATAGAGCTGGAGTAAAAAACTACCTT

GTTTCTACT----------------------------------

>A_swan_Kazakhstan_1-267-20-B-Talg-52_2020_EPI1927716

--AGCAAAAGCAGGTAGATATTGAAAGATGAGTCTTCTAACCGAGGTCGAAACGTACGTT

CTCTCTATCGTCCCGTCAGGCCCCCTCAAAGCCGAGATCGCGCAGAGACTTGAAGATGTC

TTTGCAGGGAAGAACACCGATCTTGAGGCTCTCATGGAATGGCTAAAGACAAGACCAATC

CTGTCACCTCTGACTAAGGGGATTTTGGGATTTGTGTTCACGCTCACCGTGCCCAGTGAG

CGAGGACTGCAGCGTAGACGCTTTGTCCAAAATGTTCTAAGTGGAAATGGAGACCCAAAC

AACATGGACAGGGCAGTCAAACTGTACAGGAAATTGAAGAGAGAGATAACATTCCATGGG

GCTAAAGAAGTTGCACTCAGTTACTCAACCGGTGCACTTGCCAGTTGTATGGGTCTCATA

TACAACAGGATGGGGACGGTGACCGCAGAAGTGGCATTGGGCCTAGTGTGTGCCACCTGT

GAGCAGATTGCTGATTCACAGCATCGGTCTCACAGACAGATAGCAACCACCACCAACCCA

CTAATCAGACATGAAAACAGAATGGTGTTGGCCAGTACTACAGCTAAGGCTATGGAGCAG

ATGGCTGGATCGAGTGAGCAAGCAGCGGAAGCCATGGAGGTTGCCAGTCAGGCTAGGCAG

ATGGTGCAGGCGATGAGGACCATTGGAACTCATCCTAGCTCCAGTGCCGGTCTGAGAGAT

GATCTCCTTGAAAATTTGCAGGCCTACCAAAAACGGATGGGAGTGCAACTGCAGCGATTC

AAGTGATCCTCTCGTTATTGCCGCAAGTATCATTGGGATCTTGCACTTGATATTGTGGAT

TCTTGATCGCCTTTTCTTCAAATGCGTTTATCGTCGCCTTAAATACGGTTTTAAAAGAGG

GCCTTCTACGGAAGGAGTACCTGAGTCCATGAGGGAAGAGTACCGGCAGGAACAGCAGAG

TGCTGTGGATGTTGACGATGGTCATTTTGTCAACATAGAGCTGGAGTAAAAAACTACCTT

GTTTCTACT----------------------------------

>A_pigeon_Kazakhstan_15-20-B-Talg-5_2020_EPI1927722

--AGCAAAAGCAGGTAGATATTGAAAGATGAGTCTTCTAACCGAGGTCGAAACGTACGTT

CTCTCTATCGTCCCGTCAGGCCCCCTCAAAGCCGAGATCGCGCAGAGACTTGAAGATGTC

TTTGCAGGGAAGAACACCGATCTTGAGGCTCTCATGGAATGGCTAAAGACAAGACCAATC

CTGTCACCTCTGACTAAGGGGATTTTGGGATTTGTGTTCACGCTCACCGTGCCCAGTGAG

CGAGGACTGCAGCGTAGACGCTTTGTCCAAAATGCTCTAAATGGAAATGGAGACCCAAAC

AACATGGACAGGGCAGTCAAATTGTACAGGAAATTGAAGAGAGAGATAACATTCCATGGG

GCTAAAGAAGTTGCACTCAGTTACTCAACCGGTGCACTTGCCAGTTGTATGGGTCTCATA

TACAACAGGATGGGGACGGTGACCGCAGAAGTGGCATTGGGCCTAGTGTGTGCCACCTGT

GAGCAGATTGCTGATTCACAGCATCGGTCTCACAGACAGATTGCAACCACCACCAATCCA

CTAATCAGACATGAAAACAGAATGGTGTTGGCCAGTACTACAGCTAAGGCTATGGAGCAG

ATGGCTGGATCGAGTGAGCAAGCAGCGGAAGCCATGGAGGTTGCTAGTCAGGCTAGGCAG

ATGGTGCAGGCGATGAGGACCATTGGAACTCATCCTAGCTCCAGTGCCGGTCTGAGAGAT

GATCTCCTTGAAAATTTGCAGGCCTACCAAAAACGGATGGGAGTGCAACTGCAGCGATTC

AAGTGATCCTCTCGTTATTGCCGCAAGTATCATTGGGATCTTGCACTTGATATTGTGGAT

TCTTGATCGCCTTTTCTTCAAATGCGTTTATCGTCGCCTTAAATACGGTTTGAAAAGAGG

GCCTTCTACGGAAGGAGTACCTGAGTCCATGAGGGAAGAGTACCGGCAGGAACAGCAGAG

TGCTGTGGATGTTGACGATGGTCATTTTGTCAACATAGAGCTGGAGTAAAAAACTACCTT

GTTTCTACT----------------------------------

>A_chicken_Kazakhstan_1-20-B-Talg-67_2020_EPI1927728

--AGCAAAAGCAGGTAGATATTGAAAGATGAGTCTTCTAACCGAGGTCGAAACGTACGTT

CTCTCTATCGTCCCGTCAGGCCCCCTCAAAGCCGAGATCGCGCAGAGACTTGAAGATGTC

TTTGCAGGGAAGAACACCGATCTTGAGGCTCTCATGGAATGGCTAAAGACAAGACCAATC

CTGTCACCTCTGACTAAGGGGATTTTGGGATTTGTGTTCACGCTCACCGTGCCCAGTGAG

CGAGGACTGCAGCGTAGACGCTTTGTCCAAAATGCTCTAAATGGAAATGGAGACCCAAAC

AACATGGACAGGGCAGTCAAACTGTACAGGAAATTGAAGAGAGAGATAACATTCCATGGG

GCTAAAGAAGTTGCACTCAGTTACTCAACCGGTGCACTTGCCAGTTGTATGGGTCTCATA

TACAACAGGATGGGGACGGTGACCGCAGAAGTGGCATTGGGCCTAGTGTGTGCCACCTGT

GAGCAGATTGCTGATTCACAGCATCGGTCTCACAGACAGATTGCAACCACCACCAATCCA

CTAATCAGACATGAAAACAGAATGGTGTTGGCCAGTACTACAGCTAAGGCTATGGAGCAG

ATGGCTGGATCGAGTGAGCAAGCAGCGGAAGCCATGGAGGTTGCTAGTCAGGCTAGGCAG

ATGGTGCAGGCGATGAGGACCATTGGAACTCATCCTAGCTCCAGTGCCGGTCTGAGAGAT

GATCTCCTTGAAAATTTGCAGGCCTACCAAAAACGGATGGGAGTGCAACTGCAGCGATTC

AAGTGATCCTCTCGTTATTGCCGCAAGTATCATTGGGATCTTGCACTTGATATTGTGGAT

TCTTGATCGCCTTTTCTTCAAATGCGTTTATCGTCGCCTTAAATACGGTTTGAAAAGAGG

GCCTTCTACGGAAGGAGTACCTGAGTCCATGAGGGAAGAGTACCGGCAGGAACAGCAGAG

TGCTGTGGATGTTGACGATGGTCATTTTGTCAACATAGAGCTGGAGTAAAAAACTACCTT

GTTTCTACT----------------------------------

>A_tundra_swan_Niigata_5112007_2016_EPI1184381

-----------------ATGTTGAAAGATGAGTCTTCTAACCGAGGTCGAAACGTACGTT

CTCTCTATCATCCCATCAGGCCCCCTCAAAGCCGAGATCGCACAGAAACTTGAGGATGTG

TTTGCAGGAAAGAACGCTGATCTCGAGGCTCTCATGGAGTGGCTAAAGACAAGACCAATC

CTGTCACCTCTGACTAAAGGGATCTTGGGATTTGTATTCACGCTCACCGTGCCCAGTGAG

CGAGGACTGCAGCGTAGACGTTTTGTCCAGAATGCCCTAAATGGAAATGGAGATCCAAAT

AATATGGATAGGGCAGTTAAGCTATATAAGAAGCTGAAAAGAGAAATAACATTCCATGGA

GCTAAGGAGGTCGCACTCAGTTACTCAACCGGTGCACTTGCCAGTTGCATGGGCCTCATA

TACAACAGAATGGGAACGGTGACTACAGAAGTGGCTTTTGGCCTAGTGTGTGCCACTTGT

GAGCAGATTGCTGATTCACAGCATCGGTCTCACAGACAGATGGCAACCATCACCAACCCA

CTAATCAGGCATGAGAACAGAATGGTGCTGGCCAGCACTACAGCTAAGGCTATGGAGCAG

ATGGCGGGATCAAGTGAGCAGGCAGCAGAAGCCATGGAGGTCGCCAATCAGGCTAGACAG

ATGGTGCAGGCAATGAGGACAATTGGGACTCATCCTAATTCTAGTACTGGTCTGAGAGAC

AATCTTCTTGAAAATTTGCAGGCCTACCAGAAACGAATGGGAGTGCAGATGCAGCGATTC

AAGTGATCCTCTTGTTGTTGCCGCAAGTATCATTGGGATCTTGCACTTGATATTGTGGAT

TCTTGATCGTCTTTTCTTCAAATGCATTTATCGTCGCCTTAAATACGGTTTGAAAAGAGG

GCCTTCTACGGAAGGAGTACCGGAGTCTATGAGGGAAGAGTACCGACAGGAACAGCAGAA

TGCTGTAGATGTTGACGATGGTCATTTTGTCAACATAGAATTGGAGTAA-----------

-------------------------------------------

>A_chicken_Vietnam_NCVD-15A55_2015_EPI895049

--------------TAGATGTTGAAAGATGAGTCTTCTAACCGAGGTCGAAACGTACGTT

CTCTCTATCATCCCATCAGGCCCCCTCAAAGCCGAGATCGCGCAGAAACTTGAGGATGTG

TTTGCAGGAAAGAACGCTGATCTCGAGGCTCTCATGGAGTGGCTAAAGACAAGACCAATC

CTGTCACCTCTGACTAAAGGGATTTTGGGATTTGTATTCACGCTCACCGTGCCCAGTGAG

CGAGGACTGCAGCGTAGACGTTTTGTCCAGAATGCCCTAAATGGAAATGGAGATCCAAAT

AATATGGATAGGGCAGTTAAGCTATATAAGAAGCTGAAAAGAGAAATAACATTCCATGGA

GCTAAGGAGGTCGCACTCAGTTACTCAACCGGTGCACTTGCCAGTTGCATGGGTCTCATA

TACAACAGAATGGGAACGGTGACTACAGAAGTGGCTTTTGGCCTAGTGTGTGCCACTTGT

GAGCAGATTGCAGATTCACAGCATCGGTCTCACAGACAGATGGCAACCATCACCAACCCA

CTAATCAGGCATGAGAACAGAATGGTGCTGGCCAGCACTACAGCTAAGGCTATGGAACAG

ATGGCGGGATCAAGTGAGCAGGCAGCAGAAGCCATGGAGGTCGCCAATCAGGCTAGACAG

ATGGTGCAGGCAATGAGAACAATTGGGACTCATCCTAATTCTAGTGCTGGTCTGAGAGAC

AATCTTCTTGAAAATTTGCAGGCCTACCAGAAACGAATGGGAGTGCAGATGCAGCGATTC

AAGTGATCCTCTTGTTGTTGCCGCAAGTATCATTGGGATCTTGCACTTGATATTGTGGAT

TCTTGATCGTCTTTTCTTCAAATACATTTATCGTCGCCTTAAATACGGTTTGAAAAGAGG

GCCTTCTACGGAAGGAGTACCGGAGTCTATGAGGGAAGAGTACCGGCAGGAACAGCAGAA

TGCTGTAGATGTTGACGATGATCATTTTGTCAACATAGAATTGGAGTAAAAAACTA----

-------------------------------------------

>A_chicken_Vietnam_NCVD-15A59_2015_EPI895066

--------------TAGATGTTGAAAGATGAGTCTTCTAACCGAGGTCGAAACGTACGTT

CTCTCTATCATCCCATCAGGCCCCCTCAAAGCCGAGATCGCGCAGAAACTTGAGGATGTG

TTTGCAGGAAAGAACGCTGATCTCGAGGCTCTCATGGAGTGGCTAAAGACAAGACCAATC

CTGTCACCTCTGACTAAAGGGATTTTGGGATTTGTATTCACGCTCACCGTGCCCAGTGAG

CGAGGACTGCAGCGTAGACGTTTTGTCCAGAATGCCCTAAATGGAAATGGAGATCCAAAT

AATATGGATAGGGCAGTTAAGCTATATAAGAAGCTGAAAAGAGAGATAACATTCCATGGA

GCTAAGGAGGTCGCACTCAGTTACTCAACCGGTGCACTTGCCAGTTGCATGGGTCTCATA

TACAACAGAATGGGAACGGTGACTACAGAAGTGGCTTTTGGCCTAGTGTGTGCCACTTGT

GAGCAGATTGCAGATTCACAGCATCGGTCTCACAGACAGATGGCAACTATCACCAACCCA

CTAATCAGGCATGAGAACAGAATGGTGCTGGCCAGCACTACAGCTAAGGCTATGGAGCAG

ATGGCGGGATCAAGTGAGCAGGCAGCAGAAGCCATGGAGGTCGCCAATCAGGCTAGACAG

ATGGTGCAGGCAATGAGGACAATTGGGACTCATCCTAATTCTAGTGCTGGTCTGAGAGAC

AATCTTCTTGAAAATTTGCAGGCCTACCAGAAACGAATGGGAGTGCAGATGCAGCGATTC

AAGTGATCCTCTTGTTGTTGCCGCAAGTATCATTGGGATCTTGCACTTGATATTGTGGAT

TCTTGATCGTCTTTTCTTCAAATACATTTATCGTCGCCTTAAATACGGTTTGAAAAGAGG

GCCTTCTACGGAAGGAGTACCGGAGTCTATGAGGGAAGAGTACCGGCAGGAACAGCAGAA

TGCTGTAGATGTTGACGATGGTCATTTTGTCAACATAGAATTGGAGTAAAAAACTA----

-------------------------------------------

>A_duck_Wuhan_JXYFB22_2015_EPI682973

---------------------TGAAAGATGAGTCTTCTAACCGAGGTCGAAACGTACGTT

CTCTCTATCATCCCATCAGGCCCCCTCAAAGCCGAGATCGCGCAGAAACTTGAGGATGTG

TTTGCAGGAAAGAACGCTGATCTCGAGGCTCTCATGGAGTGGCTAAAGACAAGACCAATC

CTGTCACCTCTGACTAAAGGGATCTTGGGATTTGTATTCACGCTCACCGTGCCCAGTGAG

CGAGGACTGCAGCGTAGACGTTTTGTCCAGAATGCCCTAAATGGAAATGGAGATCCAAAT

AATATGGATAGGGCAGTTAAGCTATATAAGAAGCTGAAAAGAGAAATAACATTCCATGGA

GCTAAGGAGGTCGCACTCAGTTACTCAACCGGTGCACTTGCCAGTTGCATGGGTCTCATA

TACAACAGAATGGGAACGGTGACTACAGAAGTGGCTTTTGGTCTAGTGTGTGCCACTTGT

GAGCAGATTGCAGATTCACAGCATCGGTCTCACAGACAGATGGCAACCATCACCAACCCA

CTAATCAGGCATGAGAATAGAATGGTGCTGGCCAGCACTACAGCTAAGGCTATGGAGCAG

ATGGCGGGATCAAGTGAGCAGGCAGCAGAAGCCATGGAGGTCGCCAATCAGGCTAGACAG

ATGGTGCAGGCAATGAGGACAATTGGGACTCATCCTAATTCTAGTGCTGGTCTGAGAGAT

AATCTTCTTGAAAATTTGCAGGCCTACCAGAAACGAATGGGAGTGCAGATGCAGCGATTC

AAGTGATCCTCTTGTTGTTGCCGCAAGTATCATTGGGATCTTGCACTTGATATTGTGGAT

TCTTGATCGTCTTTTCTTCAAATACATTTATCGTCGCCTTAAATACGGTTTGAAAAGAGG

GCCTTCTACGGAAGGGGTACCGGAGTCTATGAGGGAAGAGTACCGGCAGGAACAGCAGAA

TGCTGTAGATGTTGACGATGGTCATTTTGTCAACATAGAATTGGAGTAAAAAACT-----

-------------------------------------------

>A_goose_Hunan_116_2014_EPI958635

---------------------------ATGAGTCTTCTAACCGAGGTCGAAACGTACGTT

CTCTCTATCATCCCATCAGGCCCCCTCAAAGCCGAGATCGCGCAGAAACTTGAGGATGTG

TTTGCAGGAAAGAACGCTGATCTCGAGGCTCTCATGGAGTGGCTAAAGACAAGACCAATC

CTGTCACCTCTGACTAAAGGGATCTTGGGATTTGTATTCACGCTCACCGTGCCCAGTGAG

CGAGGACTGCAGCGTAGACGTTTTGTCCAGAATGCCCTAAATGGAAATGGAGATCCAAAT

AATATGGATAGGGCAGTTAAGCTATATAAGAAGCTGAAAAGAGAAATAACATTCCATGGA

GCTAAGGAGGTCGCACTCAGTTACTCAACCGGTGCACTTGCCAGTTGCATGGGTCTCATA

TACAACAGAATGGGAACGGTGACTACAGAAGTGGCTTTTGGTCTAGTGTGTGCCACTTGT

GAGCAGATTGCAGATTCACAGCATCGGTCTCACAGACAGATGGCAACCATCACCAACCCA

CTAATCAGGCATGAGAACAGAATGGTGCTGGCCAGCACTACAGCTAAGGCTATGGAGCAG

ATGGCGGGATCAAGTGAGCAGGCAGCAGAAGCCATGGAGGTCGCCAATCAGGCTAGACAG

ATGGTGCAGGCAATGAGGACAATTGGGACTCATCCTAATTCTAGTGCTGGTCTGAGAGAT

AATCTTCTTGAAAATTTGCAGGCCTACCAGAAACGAATGGGAGTGCAGATGCAGCGATTC

AAGTGATCCTCTTGTTGTTGCCGCAAGTATCATTGGGATCTTGCACTTGATATTGTGGAT

TCTTGATCGTCTTTTCTTCAAATACATTTATCGTCGCCTTAAATACGGTTTGAAAAGAGG

GCCTTCTACGGAAGGGGTACCGGAGTCTATGAGGGAAGAGTACCGGCAGGAACAGCAGAA

TGCTGTAGATGTTGACGATGGTCATTTTGTCAACATAGAATTGGAGTAA-----------

-------------------------------------------

>A_Guangdong_18SF020_2018_EPI1352808

--AGCAAAAGCAGGTAGATGTTGAAAGATGAGTCTTCTAACCGAGGTCGAAACGTACGTT

CTCTCTATCATCCCATCAGGCCCCCTCAAAGCCGAGATCGCGCAGAAACTTGAGGATGTG

TTTGCAGGAAAGAACGCTGATCTCGAGGCTCTCATGGAGTGGCTAAAGACAAGACCAATC

CTGTCACCTCTGACTAAAGGAATTTTGGGATTTGTATTCACGCTCACCGTGCCCAGTGAG

CGAGGACTGCAGCGTAGACGGTTTGTCCAGAATGCCCTAAATGGAAATGGAGATCCAAAT

AATATGGATAGGGCAGTTAAGCTATATAAGAAGCTGAAAAGAGAAATAACATTTCATGGA

GCTAAGGAGGTCGCACTCAGTTACTCAACTGGTGCACTTGCCAGTTGCATGGGTCTCATA

TACAACAGAATGGGAACAGTGACTACAGAAGTGGCTTTTGGCCTAGTGTGTGCCACTTGT

GAGCAGATTGCGGATTCACAGCATCGGTCTCACAGACAGATGGCAACCATCACCAACCCA

CTAATCAGGCATGAGAACAGAATGGTGCTGGCCAGCACTACAGCTAAGGCTATGGAGCAG

ATGGCGGGATCAAGTGAGCAGGCAGCAGAAGCCATGGAGGTCGCCAATCAGGCTAGACAG

ATGGTGCAGGCAATGAGAACAATTGGGACTCATCCTAATTCTAGTACTGGTCTGAGAGAC

AATCTTCTTGAAAATTTGCAGACCTACCAGAAACGGATGGGAGTGCAGATGCAGCGATTC

AAGTGATCCTCTTGTTGTTGCCGCAAGTATCATTGGGATCTTGCACTTGATATTGTGGAT

TCTTGATCGTCTTTTCTTCAAATGCATTTATCGTCGCCTTAAATACGGTTTGAAAAGAGG

GCCTTCTACGGAAGGAGTACCGGAGTCTATGAGGGAAGAGTACCGGCAGGAACAGCAGAA

TGCTGTAGATGTTGACGATAGTCATTTTGTCAACATAGAGTTGGAGTAAAAAACTACCTT

GTTTCTACT----------------------------------

>A_duck_Bangladesh_43127_2020_EPI1902989

--------------TAGATGTTGAAAGATGAGTCTTCTAACCGAGGTCGAAACGTACGTT

CTCTCTATCATTCCATCAGGCCCCCTCAAAGCCGAGATCGCGCAGAAACTTGAGGATGTG

TTTGCAGGAAAGAACGCCGATCTCGAGGCTCTCATGGAGTGGCTAAAGACAAGACCAATC

CTGTCACCTCTGACTAAGGGAATTTTGGGATTTATATTCACGCTCACCGTGCCCAGTGAG

CGAGGACTGCAGCGTAGACGGTTTGTCCAGAATGCCCTAAATGGAAATGGAGATCCAAAT

AATATGGATAGGGCAGTTAAGCTATATAAGAAGCTGAAAAGAGAAATAACATTCCATGGA

GCTAAGGAGGTCGCACTCAGTTACTCAACTGGTGCACTTGCCAGTTGCATGGGTCTCATA

TACAACAGAATGGGAACAGTGACTACAGAAGTGGCTTTTGGTCTAGTGTGTGCCACTTGT

GAGCAGATTGCGGATTCACAGCATCGGTCTCACAGACAGATGGCAACCATCACCAACCCA

CTAATCAGACATGAGAACAGAATGGTGCTGGCCAGCACTACAGCTAAGGCTATGGAGCAG

ATGGCGGGATCAAGTGAGCAGGCAGCAGAAGCCATGGAGGTCGCCAATCAGGCTAGACAG

ATGGTGCAGGCAATGAGAACAATTGGGACTCATCCTAATTCTAGTACTGGTCTGAGAGAC

AATCTTCTTGAAAATTTGCAGGCCTACCAGAAACGGATGGGAGTGCAGATGCAGCGATTC

AAGTGATCCTCTTGTTGTTGCCGCAAGTATCATTGGGATCTTGCACTTGATATTGTGGAT

TCTTGATCGTCTTTTCTTCAAATGCATTTATCGTCGCCTTAAATACGGTTTGAAAAGAGG

GCCTTCTACGGAAGGAGTACCGGAGTCTATGAGGGAAGAGTACCGGCAGGAACAGCAAAA

TGCTGTAGATGTTGACGATGGTCATTTTGTCAACATAGAGTTGGAGTAAAAAACTA----

-------------------------------------------

>A_duck_Hyogo_1_2016_EPI866711

---------------------------ATGAGTCTTCTAACCGAGGTCGAAACGTACGTT

CTCTCTATCATCCCATCAGGCCCCCTCAAAGCCGAGATCGCGCAGAAACTTGAGGATGTG

TTTGCAGGAAAGAACGCTGATCTCGAGGCTCTCATGGAGTGGCTAAAGACAAGACCAATC

CTGTCACCTCTGACTAAAGGGATCTTGGGATTTGTATTCACGCTCACCGTGCCCAGTGAG

CGAGGACTGCAGCGTAGACGTTTTGTCCAGAATGCCCTAAATGGAAATGGAGATCCAAAT

AATATGGATAGGGCAGTTAAGCTATATAAGAAGCTGAAAAGAGAAATAACATTCCATGGA

GCTAAGGAGGTCGCACTCAGTTACTCAACCGGTGCACTTGCCAGTTGCATGGGCCTCATA

TACAACAGAATGGGAACGGTGACTGCAGAAGTGGCTTTTGGCCTAGTGTGTGCCACTTGT

GAGCAGATTGCTGATTCACAGCATCGGTCTCACAGACAGATGGCAACCATCACTAACCCA

CTAATCAGGCATGAGAACAGAATGGTGCTGGCCAGCACTACAGCTAAGGCTATGGAGCAG

ATGGCGGGATCAAGTGAGCAGGCAGCAGAAGCCATGGAGGTCGCCAATCAGGCTAGACAG

ATGGTGCAGGCAATGAGGACAATTGGGACTCATCCTAATTCTAGTACTGGTCTGAGAGAC

AATCTTCTTGAAAATTTGCAGGCCTACCAGAAACGAATGGGAGTGCAGATGCAGCGATTC

AAGTGATCCTCTTGTTGTTGCCGCAAGTATCATTGGGATCTTGCACTTGATATTGTGGAT

TCTTGATCGTCTTTTCTTCAAATGCATTTATCGTCGCCTTAAATACGGTTTGAAAAGAGG

GCCTTCTACGGAAGGAGTACCGGAGTCTATGAGGGAAGAGTACCGACAGGAACAGCAGAA

TGCTGTAGATGTTGACGATGGTCATTTTGTCAACATAGAATTGGAGTAA-----------

-------------------------------------------

>A_Hubei_29578_2016_x_PR8_CNIC-HB29578_2016_1369968

--AGCAAAAGCAGGTAGATATTGAAAGATGAGTCTTCTAACCGAGGTCGAAACGTACGTA

CTCTCTATCATCCCGTCAGGCCCCCTCAAAGCCGAGATCGCACAGAGACTTGAAGATGTC

TTTGCAGGGAAGAACACCGATCTTGAGGTTCTCATGGAATGGCTAAAGACAAGACCAATC

CTGTCACCTCTGACTAAGGGGATTTTAGGATTTGTGTTCACGCTCACCGTGCCCAGTGAG

CGAGGACTGCAGCGTAGACGCTTTGTCCAAAATGCCCTTAATGGGAACGGGGATCCAAAT

AACATGGACAAAGCAGTTAAACTGTATAGGAAGCTCAAGAGGGAGATAACATTCCATGGG

GCCAAAGAAATCTCACTCAGTTATTCTGCTGGTGCACTTGCCAGTTGTATGGGCCTCATA

TACAACAGGATGGGGGCTGTGACCACTGAAGTGGCATTTGGCCTGGTATGTGCAACCTGT

GAACAGATTGCTGACTCCCAGCATCGGTCTCATAGGCAAATGGTGACAACAACCAATCCA

CTAATCAGACATGAGAACAGAATGGTTTTAGCCAGCACTACAGCTAAGGCTATGGAGCAA

ATGGCTGGATCGAGTGAGCAAGCAGCAGAGGCCATGGAGGTTGCTAGTCAGGCTAGACAA

ATGGTGCAAGCGATGAGAACCATTGGGACTCATCCTAGCTCCAGTGCTGGTCTGAAAAAT

GATCTTCTTGAAAATTTGCAGGCCTATCAGAAACGAATGGGGGTGCAGATGCAACGGTTC

AAGTGATCCTCTCACTATTGCCGCAAATATCATTGGGATCTTGCACTTGACATTGTGGAT

TCTTGATCGTCTTTTTTTCAAATGCATTTACCGTCGCTTTAAATACGGACTGAAAGGAGG

GCCTTCTACGGAAGGAGTGCCAAAGTCTATGAGGGAAGAATATCGAAAGGAACAGCAGAG

TGCTGTGGATGCTGACGATGGTCATTTTGTCAGCATAGAGCTGGAGTAAAAAACTACCTT

GTTTCTACT----------------------------------

>A_chicken_Hubei_ZYSJF38_2016_EPI895148

--AGCAAAAGCAGGTAGATGTTTAAAGATGAGTCTTCTAACCGAGGTCGAAACGTACGTT

CTCTCTATCATTCCATCAGGCCCCCTCAAAGCCGAAATCGCGCAGAGACTTGAGGATGTT

TTTGCAGGGAAGAACGCGGATCTCGAGGCTCTCATGGAGTGGATAAAGACAAGACCAATC

TTGTCACCTCTGACTAAGGGGATTTTAGGGTTTGTGTTCACGCTCACCGTGCCCAGTGAG

CGAGGACTGCAGCGTAGACGTTTTGTCCAAAACGCCCTAAATGGGAATGGAGACCCAAAC

AACATGGACAAGGCAGTTAAATTGTACAAGAAACTGAAGAGAGAAATGACATTTCATGGA

GCAAAGGAAGTTGCACTCAGTTACTCAACTGGTGCGCTTGCCAGCTGCATGGGTCTCATA

TACAACAGGATGGGGACAGTAACAGCAGAAGGGGCTCTTGGACTAGTATGTGCCACTTGT

GAGCAGATTGCTGACGCACAACATCGGTCCCACAGGCAGATGGCGACTACTACCAACCCA

CTAATTAGGCATGAGAATAGAATGGTACTAGCCAGCACTACAGCTAAGGCTATGGAGCAG

ATGGCTGGATCAAGTGAACAGGCAGCGGAAGCCATGGAAGTCGCAAGTCAGGCCAGGCAA

ATGGTGCAGGCTATGAGAACAGTCGGGACACACCCTAACTCCAGTACAGGTCTAAAGGAT

GATCTTATTGAAAATTTGCAGGCTTACCAAAACCGGATGGGAGTGCAACTGCAGCGGTTC

AAGTGATCCCCTCGTTGTTGCAGCTAACATTATTGGGATATTGCACTTGATATTGTGGAT

TCTTGATCGTCTTTTCTTCAAATGCATTTATCGTCGCTTTAAATACGGTTTGAAAAGAGG

GCCTTCTACGGAAGGGATACCTGAGTCTATGAGGGAAGAATATCGGCAGGAACAGCAGAA

TGCTGTGGATGTTGACGATGGTCATTTTGTCAACATAGAGCTGAAGTAAAAAACTACCTT

GTTTCTACT----------------------------------

>A_gyrfalcon_Washington_41088-6_2014_EPI569393

----------------------GAAAGATGAGTCTTCTAACCGAGGTCGAAACGTACGTT

CTCTCTATCATCCCGTCAGGCCCCCTCAAAGCCGAGATCGCGCAGAGACTTGAAGATGTC

TTTGCAGGGAAAAACACCGATCTCGAGGCTCTCATGGAGTGGCTAAAGACAAGACCAATC

CTGTCACCTCTGACTAAAGGGATTTTGGGATTTGTGTTCACGCTCACCGTGCCCAGTGAG

CGAGGACTGCAGCGTAGACGCTTCGTCCAGAATGCCCTAAATGGAAATGGGGATCCAAAT

AATATGGATAAGGCAGTTAAGCTATATAAGAAGCTGAAAAGAGAGATAACATTTCATGGG

GCTAAGGAGGTCGCACTTAGCTACTCAACCGGTGCACTTGCCAGCTGCATGGGTCTCATA

TACAACAGGATGGGAACGGTGACTACAGAAGTGGCTTTTGGCCTAGTGTGTGCCACTTGT

GAGCAGATTGCAGATTCACAGCATCGGTCCCACAGACAGATGGCAACCATCACCAACCCA

TTAATCAGACATGAGAACAGAATGGTGCTGGCCAGCACTACAGCTAAGGCCATGGAGCAG

ATGGCAGGATCAAGCGAGCAGGCATCAGAAGCCATGGAGGTTGCTAATCAGGCCAGGCAG

ATGGTACAGGCAATGAGGACAATTGGGACTCATCCTAACTCTAGTGCTGGTCTGAGAGAT

AATCTTCTTGAAAATTTGCAGGCCTACCAGAACCGAATGGGAGTGCAGATGCAGCGATTC

AAGTGATCCTCTTGTTGTTGCCGCAAATATCATTGGGATCCTGCACTTGATATTGTGGAT

CCTTGATCGTCTTTTCTTCAAATGCATTTATCGTCGCCTTAAATACGGTTTGAAAATAGG

GCCTTCTACGGAAGGGGTACCTGAGTCTATGAGGGAAGAGTACCGGCAGGAACAGCAGAG

TGCTGTGGATGTTGACGATGGTCATTTTGTCAACATAGAATTGGAGTAA-----------

-------------------------------------------

>A_chicken_Washington_3490-18_2015_EPI590695

---------------------------ATGAGTCTTCTAACCGAGGTCGAAACGTACGTT

CTCTCTATCATCCCGTCAGGCCCCCTCAAAGCCGAGATCGCGCAGAGACTTGAAGATGTC

TTTGCAGGGAAAAACACCGATCTCGAGGCTCTCATGGAGTGGCTAAAGACAAGACCAATC

CTGTCACCTCTGACTAAAGGGATTTTGGGATTTGTGTTCACGCTCACCGTGCCCAGTGAG

CGAGGACTGCAGCGTAGACGCTTCGTCCAGAATGCCCTAAATGGAAATGGGGATCCAAAT

AATATGGATAAGGCAGTTAAGCTATATAAGAAGCTGAAAAGAGAGATAACATTCCATGGG

GCTAAGGAGGTCGCACTTAGCTACTCAACCGGTGCACTTGCCAGCTGCATGGGTCTCATA

TACAACAGGATGGGAACGGTGACTACAGAAGTGGCTTTTGGCCTAGTGTGTGCCACTTGT

GAGCAGATTGCAGATTCACAGCATCGGTCCCACAGACAGATGGCAACCATCACCAACCCA

TTAATCAGACATGAGAACAGAATGGTGCTGGCCAGCACTACAGCTAAGGCCATGGAGCAG

ATGGCAGGATCAAGCGAGCAGGCATCAGAAGCCATGGAGGTTGCTAATCAGGCCAGGCAG

ATGGTACAGGCAATGAGGACAATTGGGACTCATCCTAACTCTAGTGCTGGTCTGAGAGAT

AATCTTCTTGAAAATTTGCAGGCCTACCAGAACCGAATGGGAGTGCAGATGCAGCGATTC

AAGTGATCCTCTTGTTGTTGCCGCAAATATCATTGGGATCCTGCACTTGATATTGTGGAT

CCTTGATCGTCTTTTCTTCAAATGCATTTATCGTCGCCTTAAATACGGTTTGAAAATGGG

GCCTTCTACGGAAGGGGTACCTGAGTCTATGAGGGAAGAGTACCGGCAGGAACAGCAGAG

TGCTGTGGATGTTGACGATGGTCATTTTGTCAACATAGAATTGGAGTAA-----------

-------------------------------------------

>A_Perigrine_falcon_Netherlands_18003274_1327123

--------------TAGATATTGAAAGATGAGTCTTCTAACCGAGGTCGAAACGTACGTT

CTCTCTATTGTCCCGTCAGGCCCCCTCAAAGCCGAGATCGCGCAGAGACTTGAAGATGTC

TTTGTAGGGAAGAACACCGATCTTGAGGCTCTCATGGAATGGCTAAAGACAAGACCAATC

CTGTCACCTCTGACTAAGGGGCTTTTAGGAGTTGTGTTCACGCTCACCGTGCCCAGTGAG

CGAGGACTGCAGCGTAGACGCTTTGTCCAAAATGCCCTAAATGGAAATGGAGACCCAAAC

AATATGGACAGGGCAGTCAAACTGTACAAGAAATTAAAGAGAGAGATAACATTCCATGGG

GCTAAAGAAGTTGCACTCAGTTATTCAACCGGTGCACTTGCCTGTTGTATGGGTCTCATA

TACAACAGGATGGGGACGGTGACCACAGAAGTGGCGTTGGGCCTAGTGTGTGCCACCTGT

GAGCAGATTGCTGATTCACAGCATCGGTCTCACAGACAAATAGCAACCACCACCAACCCA

CTAATCAGACATGAAAACAGAATGGTGCTGGCCAGTACTACAGCTAAGGCTATGGAGCAG

ATGGCTGGGTCGAGTGAGCAAGCAGCGGAAGCCATGGAGGTTGCTAGTCAGGCTAGGCAG

ATGGTGCAGGCGATGAGGACCATTGGAACTCACCCTAGCTCCAGTGCCGGTCTGAGAGAT

GATCTCCTTGAAAATTTGCAGGCCTACCAGAAACGAATGGGAGTGCAGATGCAGCGATTC

AAGTGATCCTCTCGTTATTGCCGCAAGTATCATTGGGATCTTGCACTTGATATTGTGGAT

TCTTGATCGTCTTTTCCTCAAATGCGTTTATCGTCGCCTTAAATACGGTTTGAAAAGAGG

GCCTTCTACGGAAGGAGTGCCTGAGTCTATGAGGGAAGAGTATCGGCAGGAACAGCAGAG

TGCTGTGGATGTTGACTATGGTCATTTTGTCAACATAGAGCTGGAGTAAAAAACTA----

-------------------------------------------

>A_Fujian-Sanyuan_21099_2017_x_PR8_CNIC_1369976

--AGCAAAAGCAGGTAGATATTGAAAGATGAGTCTTCTAACCGAGGTCGAAACGTACGTA

CTCTCTATCATCCCGTCAGGCCCCCTCAAAGCCGAGATCGCACAGAGACTTGAAGATGTC

TTTGCAGGGAAGAACACCGATCTTGAGGTTCTCATGGAATGGCTAAAGACAAGACCAATC

CTGTCACCTCTGACTAAGGGGATTTTAGGATTTGTGTTCACGCTCACCGTGCCCAGTGAG

CGAGGACTGCAGCGTAGACGCTTTGTCCAAAATGCCCTTAATGGGAACGGGGATCCAAAT

AACATGGACAAAGCAGTTAAACTGTATAGGAAGCTCAAGAGGGAGATAACATTCCATGGG

GCCAAAGAAATCTCACTCAGTTATTCTGCTGGTGCACTTGCCAGTTGTATGGGCCTCATA

TACAACAGGATGGGGGCTGTGACCACTGAAGTGGCATTTGGCCTGGTATGTGCAACCTGT

GAACAGATTGCTGACTCCCAGCATCGGTCTCATAGGCAAATGGTGACAACAACCAATCCA

CTAATCAGACATGAGAACAGAATGGTTTTAGCCAGCACTACAGCTAAGGCTATGGAGCAA

ATGGCTGGATCGAGTGAGCAAGCAGCAGAGGCCATGGAGGTTGCTAGTCAGGCTAGACAA

ATGGTGCAAGCGATGAGAACCATTGGGACTCATCCTAGCTCCAGTGCTGGTCTGAAAAAT

GATCTTCTTGAAAATTTGCAGGCCTATCAGAAACGAATGGGGGTGCAGATGCAACGGTTC

AAGTGATCCTCTCACTATTGCCGCAAATATCATTGGGATCTTGCACTTGACATTGTGGAT

TCTTGATCGTCTTTTTTTCAAATGCATTTACCGTCGCTTTAAATACGGACTGAAAGGAGG

GCCTTCTACGGAAGGAGTGCCAAAGTCTATGAGGGAAGAATATCGAAAGGAACAGCAGAG

TGCTGTGGATGCTGACGATGGTCATTTTGTCAGCATAGAGCTGGAGTAAAAAACTACCTT

GTTTCTACT----------------------------------

>A_duck_Sichuan_NCXJ16_2014_EPI590903

---------------------------ATGAGTCTTCTAACCGAGGTCGAAACGTACGTT

CTCTCTATCATCCCATCAGGCCCCCTCAAAGCCGAGATCGCGCAGAAACTTGAGGATGTG

TTTGCAGGGAAGAACACTGATCTCGAGGCTCTCATGGAGTGGCTAAAGACAAGACCAATC

CTGTCACCTCTGACCAAAGGGATCTTGGGATTTGTATTCACGCTCACCGTGCCCAGTGAG

CGAGGACTGCAGCGTAGACGTTTTGTCCAGAATGCCCTAAATGGAAATGGAGATCCAAAT

AATATGGATAGGGCAGTTAAGCTATATAAGAAGCTGAAAAGAGAAATAACATTCCATGGA

GCTAAGGAGGTCTCACTCAGTTACTCAACCGGTGCACTTGCCAGTTGCATGGGTCTCATA

TACAACAGGATGGGAACGGTGACTGCAGAAGTGGCTTTTGGCCTAGTGTGTGCCACTTGT

GAGCAGATTGCAGATTCACAGCATCGGTCTCACAGACAGATGGCAACCATCACTAACCCA

CTAATCAGGCATGAGAACAGAATGGTGCTGGCCAGCACTACAGCTAAGGCTATGGAGCAG

ATGGCGGGATCAAGTGAGCAGGCAGCAGAAGCAATGGAGGTCGCCAATCAGGCTAGACAG

ATGGTGCAGGCAATGAGGACAATTGGGACTCATCCTAACTCTAGTGCTGGTCTGAGAGAT

AATCTTCTTGAAAATTTGCAGGCCTACCAGAAAAGAATGGGAGTGCAGATGCAGCGATTC

AAGTGATCCTCTTGTTGTTGCCGCAAGTATCATTGGGATCTTGCACTTGATATTGTGGAT

TCTTGATCGTCTTTTCTTCAAATGCATTTATCGTCGCCTTAAATACGGTTTGAAAAGAGG

GCCTTCTACGGAAGGGGTACCGGAGTCTATGAGGGAAGAGTACCGGCAGGAACAGCAGAA

TGCTGTGGATGTTGACGATAGTCATTTTGTCAACATAGAATTGGAGTAA-----------

-------------------------------------------

>A_Sichuan_26221_2014_EPI533589

---------------------------ATGAGTCTTCTAACCGAGGTCGAAACGTACGTT

CTCTCTATCATCCCATCAGGCCCCCTCAAAGCCGAGATCGCGCAGAAACTTGAGGATGTG

TTTGCAGGGAAGAACACTGATCTCGAGGCTCTCATGGAGTGGCTAAAGACAAGACCAATC

CTGTCACCTCTGACCAAAGGGATCTTGGGATTTGTATTCACGCTCACCGTGCCCAGTGAG

CGAGGACTGCAGCGTAGACGTTTTGTCCAGAATGCCCTAAATGGAAATGGAGATCCAAAT

AATATGGATAGGGCAGTTAAGCTATATAAGAAGCTGAAAAGAGAAATAACATTCCATGGA

GCTAAGGAGGTCTCACTCAGTTACTCAACCGGTGCACTTGCCAGTTGCATGGGTCTCATA

TACAACAGGATGGGAACGGTGACTACAGAAGTGGCTTTTGGCCTAGTGTGTGCCACTTGT

GAGCAGATTGCAGATTCACAGCATCGGTCTCACAGACAGATGGCAACCATCACTAACCCA

CTAATCAGGCATGAGAACAGAATGGTGCTGGCCAGCACTACAGCTAAGGCTATGGAGCAG

ATGGCGGGATCAAGTGAGCAGGCAGCAGAAGCAATGGAGGTCGCCAATCAGGCTAGACAG

ATGGTGCAGGCAATGAGGACAATTGGGACTCATCCTAACTCTAGTGCTGGTCTGAGAGAT

AATCTTCTTGAAAATTTGCAGGCCTACCAGAAAAGAATGGGAGTGCAGATGCAGCGATTC

AAGTGATCCTCTTGTTGTTGCCGCAAGTATCATTGGGATCTTGCACTTGATATTGTGGAT

TCTTGATCGTCTTTTCTTCAAATGCATTTATCGTCGCCTTAAATACGGTTTGAAAAGAGG

GCCTTCTACGGAAGGGGTACCGGAGTCTATGAGGGAAGAGTACCGGCAGGAACAGCAGAA

TGCTGTGGATGTTGACGATAGTCATTTTGTCAACATAGAATTGGAGTAA-----------

-------------------------------------------
