# Supplementary material for: Highly pathogenic avian influenza virus of the A/H5N8 subtype, clade 2.3.4.4b, caused outbreaks in Kazakhstan in 2020
Source: PeerJ. 2022 Mar 2;10:e13038. doi: 10.7717/peerj.13038 (PMC8898005; doi:10.7717/peerj.13038)
Supplement: Figure S8 [file peerj-10-13038-s009.docx]

**Fig.S8** Alignment of the nucleotide sequences for the NS segment used in this study

>A_goose_Kazakhstan_4-190-20-B-H5N8-1_2020_EPI1927653

----AGCAAAAGCAGGGTGACAAAAACATAATGGATTCCAACACTATGTTAAGCTTTCAG

GTAGACTGTTTTCTTTGGTATGTCCGCAAACGATTCGCAGACCAAGAACTGGGTGATGCC

CCTTTCCTTGACCGGCTTCGCCGAGATCAGAAGTCTTTAAGAGGAAGAGGCACCACTCTT

GGTCTGAGCATCGAAGCAGCTACTCGTGAGGGAAAGCAGATAGTGAAGCGAATTCTGAAG

GAAGAGTCTGATGAGGCACTTAAAATGACTGTTGCTTCAGGTCCGTCTTCACGCTACCTA

ACTGATATGACTCTTGGAGAAATGTCAAGGGACTGGTTCATGCTCATGCCCAAACGGAAA

GTGGCAGGTCCACTTTGCATCAAAATGGACCAGGCAATAATGGATAAAAACATCATATTG

AAAGCAAACTTCAGTGTAATTTTCAACCGGCTGGAAGCTCTAATACTACTTCGAGCTTTC

ACAGAAGAAGGAGCAATTGTGGGAGAAATCTCACCGTTACCTTCTTTTCCAGGACATACT

GATGAGGATGTCAAAAATGCAATTGGAGTCCTCATCGGAGGGCTTGAATGGAATAATAAC

ACAGTTCGGGTCTCTGAAACTCTACAGAGATTCGCTTGGAGAAACAGTGATGAGGATGGG

AGACCTTCACTCCCTCCAAAGTAGAAACGGAAAATGGCGAGAACAATTGGGTCAGAAGTT

TGAAGAAATAAGATGGCTGATTGAAGAAGTGCGACATAGACTGAAGATTACAGAAAATAG

CTTCGAACAGATAACATTTATGCAAGCCTTACAACTATTGCTTGAAGTGGAACAAGAGAT

AAGAACTTTCTCGTTTCAGCTTATTTGATGATAAAAAACACCCTTGTTTCTACT------

-----------------------------------

>A_chicken_Kazakhstan_220-B-2-H5N8-4_2020_EPI1927659

----AGCAAAAGCAGGGTGACAAAAACATAATGGATTCCAACACTATGTTAAGCTTTCAG

GTAGACTGTTTTCTTTGGTATGTCCGCAAACGATTCGCAGACCAAGAACTGGGTGATGCC

CCTTTCCTTGACCGGCTTCGCCGAGATCAGAAGTCTTTAAGAGGAAGAGGCACCACTCTT

GGTCTGAGCATCGAAGCAGCTACTCGTGAGGGAAAGCAGATAGTGAAGCGAATTCTGAAG

GAAGAGTCTGATGAGGCACTTAAAATGACTGTTGCTTCAGGTCCGTCTTCACGCTACCTA

ACTGATATGACTCTTGGAGAAATGTCAAGGGACTGGTTCATGCTCATGCCCAAACGGAAA

GTGGCAGGTCCACTTTGCATCAAAATGGACCAGGCAATAATGGATAAAAACATCATATTG

AAAGCAAACTTCAGTGTAATTTTCAACCGGCTGGAAGCTCTAATACTACTTCGAGCTTTC

ACAGAAGAAGGAGCAATTGTGGGAGAAATCTCACCGTTACCTTCTTTTCCAGGACATACT

GATGAGGATGTCAAAAATGCAATTGGAGTCCTCATCGGAGGGCTTGAATGGAATAATAAC

ACAGTTCGGGTCTCTGAAACTCTACAGAGATTCGCTTGGAGAAACAGTGATGAGGATGGG

AGACCTTCACTCCCTCCAAAGTAGAAACGGAAAATGGCGAGAACAATTGGGTCAGAAGTT

TGAAGAAATAAGATGGCTGATTGAAGAAGTGCGACATAGACTGAAGATTACAGAAAATAG

CTTCGAACAGATAACATTTATGCAAGCCTTACAACTATTGCTTGAAGTGGAACAAGAGAT

AAGAACTTTCTCGTTTCAGCTTATTTGATGATAAAAAACACCCTTGTTTCTACT------

-----------------------------------

>A_duck_Kazakhstan_12-20-B-Talg-11_2020_EPI1927665

----AGCAAAAGCAGGGTGACAAAAACATAATGGATCCCAACACTATGTTAAGCTTTCAG

GTAGACTGTTTTCTTTGGTATGTCCGCAAACGATTCGCAGACCAAGAACTGGGTGATGCC

CCTTTCCTTGACCGGCTTCGCCGAGATCAGAAGTCTTTAAGAGGAAGAGGCACCACTCTT

GGTCTGAGCATCGAAACAGCTACTCGTGAGGGAAAGCAGATAGTGAAGCGAATTCTGAAG

GAAGAGTCTGATGAGGCACTTAAAATGACTGTTGCTTCAGGTCCGTCTTCACGCTACCTA

ACTGATATGACTCTTGAAGAAATGTCAAGGGACTGGTTCATGCTCATGCCCAAACGGAAA

GTGGCAGGTCCACTTTGCATCAAAATGGACCAGGCAGTAATGGATAAAAACATCATATTG

AAAGCAAACTTCAGTGTAATTTTCAACCGGCTGGAAGCTCTAATACTACTTCGAGCTTTC

ACAGAAGAAGGAGCAATTGTGGGAGAAATCTCACCGTTACCTTCTTTTCCAGGACATACT

GATGAGGATGTCAAAAATGCAATTGGGGTCCTCATCGGAGGGCTTGAATGGAATAATAAC

ACAGTTCGGGTCTCTGAAACTCTACAGAGATTCGCTTGGAGAAACAGTGATGAGGATGGG

AGACCTTCACTCCCTCCAAAGTAGAAACGGAAAATGGCGAGAACAATTGGGTCAGAAGTT

TGAAGAAATAAGATGGCTGATTGAAGAAGTGCGACATAGACTGAAGATTACAGAAAATAG

CTTCGAACAGATAACGTTTATGCAAGCCTTACAACTATTGCTTGAAGTGGAACAAGAGAT

AAGAACTTTCTCGTTTCAGCTTATTTGATGATAAAAAACACCCTTGTTTCTACT------

-----------------------------------

>A_goose_Kazakhstan_7-20-B-Talg-12_2020_EPI1927671

----AGCAAAAGCAGGGTGACAAAAACATAATGGATCCCAACACTATGTTAAGCTTTCAG

GTAGACTGTTTTCTTTGGTATGTCCGCAAACGATTCGCAGACCAAGAACTGGGTGATGCC

CCTTTCCTTGACCGGCTTCGCCGAGATCAGAAGTCTTTAAGAGGAAGAGGCACCACTCTT

GGTCTGAGCATCGAAACAGCTACTCGTGAGGGAAAGCAGATAGTGAAGCGAATTCTGAAG

GAAGAGTCTGATGAGGCACTTAAAATGACTGTTGCTTCAGGTCCGTCTTCACGCTACCTA

ACTGATATGACTCTTGAAGAAATGTCAAGGGACTGGTTCATGCTCATGCCCAAACGGAAA

GTGGCAGGTCCACTTTGCATCAAAATGGACCAGGCAGTAATGGATAAAAACATCATATTG

AAAGCAAACTTCAGTGTAATTTTCAACCGGCTGGAAGCTCTAATACTACTTCGAGCTTTC

ACAGAAGAAGGAGCAATTGTGGGAGAAATCTCACCGTTACCTTCTTTTCCAGGACATACT

GATGAGGATGTCAAAAATGCAATTGGGGTCCTCATCGGAGGGCTTGAATGGAATAATAAC

ACAGTTCGGGTCTCTGAAACTCTACAGAGATTCGCTTGGAGAAACAGTGATGAGGATGGG

AGACCTTCACTCCCTCCAAAGTAGAAACGGAAAATGGCGAGAACAATTGGGTCAGAAGTT

TGAAGAAATAAGATGGCTGATTGAAGAAGTGCGACATAGACTGAAGATTACAGAAAATAG

CTTCGAACAGATAACGTTTATGCAAGCCTTACAACTATTGCTTGAAGTGGAACAAGAGAT

AAGAACTTTCTCGTTTCAGCTTATTTGATGATAAAAAACACCCTTGTTTCTACT------

-----------------------------------

>A_swan_Kazakhstan_9-20-B-Talg-39_2020_EPI1927699

----AGCAAAAGCAGGGTGACAAAAACATAATGGATTCCAACACTATGTTAAGCTTTCAG

GTAGACTGTTTTCTTTGGTATGTCCGCAAACGATTCGCAGACCAAGAACTGGGTGATGCC

CCTTTCCTTGACCGGCTTCGCCGAGATCAGAAGTCTTTAAGAGGAAGAGGCACCACTCTT

GGTCTGAGCATCGAAGCAGCTACTCGTGAGGGAAAGCAGATAGTGAAGCGAATTCTGAAG

GAAGAGTCTGATGAGGCACTTAAAATGACTGTTGCTTCAGGTCCGTCTTCACGCTACCTA

ACTGATATGACTCTTGAAGAAATGTCAAGGGACTGGTTCATGCTCATGCCCAAACGGAAA

GTGGCAGGTCCACTTTGCATCAAAATGGACCAGGCAATAATGGATAAAAACATCATATTG

AAAGCAAACTTCAGTGTAATTTTCAACCGGCTGGAAGCTCTAATACTACTTCGAGCTTTC

ACAGAAGAAGGAGCAATTGTGGGAGAAATTTCACCGTTACCTTCTTTTCCAGGACATACT

GATGAGGATGTCAAAAATGCAATTGGGGTCCTCATCGGAGGGCTTGAATGGAATAATAAC

ACAGTTCGGGTCTCTGAAACTCTACAGAGATTCGCTTGGAGAAACAGTGATGAGGATGGG

AGACCTTCACTCCCTCCAAAGTAGAAACGAAAAATGGCGAGAACAATTGGGTCAGAAGTT

TGAAGAAATAAGATGGCTGATTGAAGAAGTGCGACATAGACTGAAGATTACAGAAAATAG

CTTCGAACAGATAACGTTTATGCAAGCCTTACAACTATTGCTTGAAGTTGAACAAGAGAT

AAGAACTTTCTCGTTTCAGCTTATTTGATGATAAAAAACACCCTTGTTTCTACT------

-----------------------------------

>A_chicken_Kazakhstan_12-20-B-Talg-45_2020_EPI1927705

----AGCAAAAGCAGGGTGACAAAAACATAATGGATCCCAACACTATGTTAAGCTTTCAG

GTAGACTGTTTTCTTTGGTATGTCCGCAAACGATTCGCAGACCAAGAACTGGGTGATGCC

CCTTTCCTTGACCGGCTTCGCCGAGATCAGAAGTCTTTAAGAGGAAGAGGCACCACTCTT

GGTCTGAGCATCGAAGCAGCTACTCGTGAGGGAAAGCAGATAGTGAAGCGAATTCTGAAG

GAAGAGTCTGATGAGGCACTTAAAATGACTGTTGCTTCAGGTCCGTCTTCACGCTACCTA

ACTGATATGACTCTTGAAGAAATGTCAAGGGACTGGTTCATGCTCATGCCCAAACGGAAA

GTGGCAGGTCCACTTTGCATCAAAATGGACCAGGCAATAATGGATAAAAACATCATATTG

AAAGCAAACTTCAGTGTAATTTTCAACCGGCTGGAAGCTCTAATACTACTTCGAGCTTTC

ACAGAAGAAGGAGCAATTGTGGGAGAAATCTCACCGTTACCTTCTTTTCCAGGACATACT

GATGAGGATGTCAAAAATGCAATTGGGGTCCTCATCGGAGGGCTTGAATGGAATAATAAC

ACAGTTCGGGTCTCTGAAACTCTACAGAGATTCGCTTGGAGAAACAGTGATGAGGATGGG

AGACCTTCACTCCCTCCAAAGTAGAAACGGAAAATGGCGAGAACAATTGGGTCAGAAGTT

TGAAGAAATAAGATGGCTGATTGAAGAAGTGCGACATAGACTGAAGATTACAGAAAATAG

CTTCGAACAGATAACGTTTATGCAAGCCTTACAACTATTGCTTGAAGTGGAGCAAGAGAT

AAGAACTTTCTCGTTTCAGCTTATTTGATGATAAAAAACACCCTTGTTTCTACT------

-----------------------------------

>A_crow_Kazakhstan_15-20-B-Talg-4_2020_EPI1927711

----AGCAAAAGCAGGGTGACAAAAACATAATGGATCCCAACACTATGTTAAGCTTTCAG

GTAGACTGTTTTCTTTGGTATGTCCGCAAACGATTCGCAGACCAAGAACTGGGTGATGCC

CCTTTCCTTGACCGGCTTCGCCGAGATCAGAAGTCTTTAAGAGGAAGAGGCACCACTCTT

GGTCTGAGCATCGAAACAGCTACTCGTGAGGGAAAGCAGATAGTGAAGCGAATTCTGAAG

GAAGAGTCTGATGAGGCACTTAAAATGACTGTTGCTTCAGGTCCGTCTTCACGCTACCTA

ACTGATATGACTCTTGAAGAAATGTCAAGGGACTGGTTCATGCTCATGCCCAAACGGAAA

GTGGCAGGTCCACTTTGCATCAAAATGGACCAGGCAGTAATGGATAAAAACATCATATTG

AAAGCAAACTTCAGTGTAATTTTCAACCGGCTGGAAGCTCTAATACTACTTCGAGCTTTC

ACAGAAGAAGGAGCAATTGTGGGAGAAATCTCACCGTTACCTTCTTTTCCAGGACATACT

GATGAGGATGTCAAAAATGCAATTGGGGTCCTCATCGGAGGGCTTGAATGGAATAATAAC

ACAGTTCGGGTCTCTGAAACTCTACAGAGATTCGCTTGGAGAAACAGTGATGAGGATGGG

AGACCTTCACTCCCTCCAAAGTAGAAACGGAAAATGGCGAGAACAATTGGGTCAGAAGTT

TGAAGAAATAAGATGGCTGATTGAAGAAGTGCGACATAGACTGAAGATTACAGAAAATAG

CTTCGAACAGATAACGTTTATGCAAGCCTTACAACTATTGCTTGAAGTGGAACAAGAGAT

AAGAACTTTCTCGTTTCAGCTTATTTGATGATAAAAAACACCCTTGTTTCTACT------

-----------------------------------

>A_swan_Kazakhstan_1-267-20-B-Talg-52_2020_EPI1927717

----AGCAAAAGCAGGGTGACAAAAACATAATGGATCCCAACACTATGTTAAGCTTTCAG

GTAGACTGTTTTCTTTGGTATGTCCGCAAACGATTCGCAGACCAAGAACTGGGTGATGCC

CCCTTCCTTGACCGGCTTCGCCGAGATCAGAAGTCTTTAAGAGGAAGAGGCACCACTCTT

GGTCTGAGCATCGAAGCAGCCACTCGTGAGGGAAAGCAGATAGTGAAGCGAATTCTGAAG

GAAGAGTCTGATGAGGCACTTAAAATGACTGTTGCTTCAGGTCCGTCTTCACGCTACCTA

ACTGATATGACTCTTGAAGAAATGTCAAGGGACTGGTTCATGCTCATGCCCAAACGGAAA

GTGGCAGGTCCACTTTGCATCAAAATGGACCAGGCAATAATGGATAAAAACATCATATTG

AAAGCAAACTTCAGTGTAATTTTCAACCGGCTGGAAGCTCTAATACTACTTCGAGCTTTC

ACAGAAGAAGGAGCAATTGTGGGAGAAATCTCACCGTTACCTTCTTTTCCAGGACATACT

GATGAGGATGTCAAAAATGCAATTGGGGTCCTCATCGGAGGGCTTGAATGGAATAATAAC

ACAGTTCGGGTCTCTGAAACTCTACAGAGATTCGCTTGGAGAAACAGTGATGAGGATGGG

AGACCTTCACTCCCTCCAAAGTAGAAACGGAAAATGGCGAGAACAATTGGGTCAGAAGTT

TGAAGAAATAAGATGGCTGATTGAAGAAGTGCGACATAGACTGAAGATTACAGAAAATAG

CTTCGAACAGATAACGTTTATGCAAGCCTTACAACTATTGCTTGAAGTGGAGCAAGAGAT

AAGAACTTTCTCGTTTCAGCTTATTTGATGATAAAAAACACCCTTGTTTCTACT------

-----------------------------------

>A_pigeon_Kazakhstan_15-20-B-Talg-5_2020_EPI1927723

----AGCAAAAGCAGGGTGACAAAAACATAATGGATCCCAACACTATGTTAAGCTTTCAG

GTAGACTGTTTTCTTTGGTATGTCCGCAAACGATTCGCAGACCAAGAACTGGGTGATGCC

CCTTTCCTTGACCGGCTTCGCCGAGATCAGAAGTCTTTAAGAGGAAGAGGCACCACTCTT

GGTCTGAGCATCGAAACAGCTACTCGTGAGGGAAAGCAGATAGTGAAGCGAATTCTGAAG

GAAGAGTCTGATGAGGCACTTAAAATGACTGTTGCTTCAGGTCCGTCTTCACGCTACCTA

ACTGATATGACTCTTGAAGAAATGTCAAGGGACTGGTTCATGCTCATGCCCAAACGGAAA

GTGGCAGGTCCACTTTGCATCAAAATGGACCAGGCAGTAATGGATAAAAACATCATATTG

AAAGCAAACTTCAGTGTAATTTTCAACCGGCTGGAAGCTCTAATACTACTTCGAGCTTTC

ACAGAAGAAGGAGCAATTGTGGGAGAAATCTCACCGTTACCTTCTTTTCCAGGACATACT

GATGAGGATGTCAAAAATGCAATTGGGGTCCTCATCGGAGGGCTTGAATGGAATAATAAC

ACAGTTCGGGTCTCTGAAACTCTACAGAGATTCGCTTGGAGAAACAGTGATGAGGATGGG

AGACCTTCACTCCCTCCAAAGTAGAAACGGAAAATGGCGAGAACAATTGGGTCAGAAGTT

TGAAGAAATAAGATGGCTGATTGAAGAAGTGCGACATAGACTGAAGATTACAGAAAATAG

CTTCGAACAGATAACGTTTATGCAAGCCTTACAACTATTGCTTGAAGTGGAACAAGAGAT

AAGAACTTTCTCGTTTCAGCTTATTTGATGATAAAAAACACCCTTGTTTCTACT------

-----------------------------------

>A_chicken_Kazakhstan_1-20-B-Talg-67_2020_EPI1927729

----AGCAAAAGCAGGGTGACAAAAACATAATGGATCCCAACACTATGTTAAGCTTTCAG

GTAGACTGTTTTCTTTGGTATGTCCGCAAACGATTCGCAGACCAAGAACTGGGTGATGCC

CCTTTCCTTGACCGGCTTCGCCGAGATCAGAAGTCTTTAAGAGGAAGAGGCACCACTCTT

GGTCTGAGCATCGAAGCAGCTACTCGTGAGGGAAAGCAGATAGTGAAGCGAATTCTGAAG

GAAGAGTCTGATGAGGCACTTAAAATGACTGTTGCTTCAGGTCCGTCTTCACGCTACCTA

ACTGATATGACTCTTGAAGAAATGTCAAGGGACTGGTTCATGCTCATGCCAAAACGGAAA

GCGGCAGGTCCACTTTGCATCAAAATGGACCAGGCAGTAATGGATAAAAACATCATATTG

AAAGCAAACTTCAGTGTAATTTTCAACCGGCTGGAAGCTCTAATACTACTTCGAGCTTTC

ACAGAAGAAGGAGCAATTGTGGGAGAAATCTCACCGTTACCTTCTTTTCCAGGACATACT

GATGAGGATGTCAAAAATGCAATTGGGGTCCTCATCGGAGGGCTTGAATGGAATAATAAC

ACAGTTCGGGTCTCTGAAACTCTACAGAGATTCGCTTGGAGAAACAGTGATGAGGATGGG

AGACCTTCACTCCCTCCAAAGTAGAAACGGAAAATGGCGAGAACAATTGGGTCAGAAGTT

TGAAGAAATAAGATGGCTGATTGAAGAAGTGCGACATAGACTGAAGATTACAGAAAATAG

CTTCGAACAGATAACGTTTATGCAAGCCTTACAACTATTGCTTGAAGTGGAACAAGAGAT

AAGAACTTTCTCGTTTCAGCTTATTTGATGATAAAAAACACCCTTGTTTCTACT------

-----------------------------------

>A_duck_Lao_961_2010_EPI335151

------------------------------ATGGATCCCAACACTGTGTCAAGCTTTCAG

GTAGACTGCTTTCTTTGGCATGTCCGCAAACGATTTGCAGACCAAGAACTGGGTGATGCC

CCATTCCTTGACCGGCTTCGCCGAGATCAGAAGTCCCTAAGAGGAAGAGGCAACACTCTT

GGTCTGGACATCAAAACAGCTACCCGTGCGGGGAAACAGATAGTTGAGCGGATTCTGGAG

GAGGAATCTGATGAGGCACTTAAAATGGCGANNCTTAAAATGCCGACTTCACGCTACCTA

ACTGACATGACTCTCGAAGAAATGTCAAGGGACTGGTTCATGCTCATGCCCAAACAGAAA

GTGGCAGGTTCCCTTTGCATCAAAATGGACCAGGCAATAATGGATAAAACCATCATATTG

AAAGCAAACTTCAGTGTGATTTTTGACCGGTTAGACACCCTAATACTACTTAGAGCTTTC

ACAGAAGAAGGAGCAATCGTGGGAGAAATCTCACCATTACCTTCTCTTCCAGGACATACT

AGTGAGGATGTCAAAAATGCAATTGGCGTCCTCATCGGAGGACTTGAATGGAATGATAAC

ACAGTTCGGGTCTCTGAAACTATACAGAGATTCGCTTGGAGAAGCAGTAATGAGGATGGG

AGACTTCTACTCCCTCCAAATCAGAAACGGTAAATGGCGAGAACAATTGAGTCAGAAGTT

TGAAGAAATAAGGTGGCTGATTGAAGAAGTACGACATAGATTGAAAAGCACAGAAAACAG

CTTCGAACAGATAACGTTTATGCAAGCCTTACAACTACTGCTTGAAGTGGAGCAAGAGAT

AAGAGCCTTCTCGTTTCAGCTTATTTAA--------------------------------

-----------------------------------

>A_duck_Lao_567_2010_EPI335175

------------------------------ATGGATTCCAACACTGTGTCAAGTTTTCAG

GTAGACTGCTTTCTTTGGCATGTCCGCAAACGATTTGCAGACCAAGAAATGGGTGATGCC

CCATTCCTTGACCGGCTTCGCCGAGATCAGAAGTCCCTAAGAGGAAGAGGCAACACTCTT

GGTCTGGACATCGAAACAGCTACTCGTGCGGGAAAACAGATAGTGGAGCGGATTCTGGAG

GAGGAATCTGATGAGGCACTTAAAATGACTATTGCTTCTGTGCCTGCTTCACGCTACCTA

ACTGACATGACTCTCGAAGAAATGTCAAGGGACTGGTTCATGCTCATGCCCAAGCAGAAA

GTGGCGGGTTCCCTTTGCATCAAAATGGACCAGGCCATAATGGATAAAACCATCATATTG

AAAGCAAACTTCAGCGTGATTTGTGACCGGTTAGAAACCCTAATACTGCTTAGAGCTTTC

ACAAAAGAAGGAGCAATCGTGGGAGAAATCTCACCATTACCTTCTCTTCCAGGACATACT

GGTGAGGATGTCAAAAATGCAATTGGCGTCCTCATCGGAGGACTTGAATGGAATGATAAC

ACAGTTCGGGTCTCTGAAACTATACAGAGATTCGCTTGGAGAAGCAGTGATGAGGGTGGG

AGACTTCCACTCCCTCCAAATCAGAAACGGAAAATGGCGAGAACAATTGAGTCAGAAGTT

TGAAGAAATAAGGTGGCTGATTGAAGAAGTACGACATAGATTGAAAATTACAGAAAACAG

CTTCGAACAGATAACGTTTATGCAAGCCTTACAACTACTGCTTGAAGTGGAGCAAGAGAT

AAGAACTTTCTCGTTTCAGCTTATTTAA--------------------------------

-----------------------------------

>A_breeder_duck_Korea_Gochang1_2014_EPI509710

-------------------ACAAAAACATAATGGATTCCAACACTATGTTAAGCTTTCAG

GTAGACTGCTTTCTTTGGTATGTCCGCAAACGATTTGCAGACCAAGAACTGGGTGATGCC

CCGTTCCTTGACCGGCTTCGCCGAGATCAGAAGTCTTTAAGAGGAAGAGGCAGCACTCTT

GGTCTGGACATCGAAACGGCTAATCGTGCGGGAAAGCAGATAGTGGAGCGAATTCTGGAG

GAAGAATCTGATGAGGCACTTAAAATGACTATTGCTTCAGTGCCGTCTTCACGCTACCTA

ACTGACATGACTCTTGAAGAAATGTCAAGGGACTGGTTCATGCTCATGCCCAAACAGAAA

GTGGCAGGTTCTCTTTGCATCAAAATGGACCAGGCAATAATGGATAAAAACATCATATTG

AAAGCAAATTTCAGTGTAATTTTTAACCGGCTGGAAGCTCTAATACTACTTAGAGCTTTC

ACAGAAGAAGGAGCAATTGTGGGAGAAATCTCACCGTTACCTTCTTTTCCAGGACATACT

GATGAGGATGTCAAAAATGCAATTGGGGTCCTCATCGGAGGACTTGAATGGAATGATAAC

ACAGTTCGGGTCTCTGAAACTCTACAGAGATTCGCTTGGAGAAGCAGTAATGAGGATGGG

AGACCTTCACTCCCTCCAAAGCAGAAACGGAAAATGGCGAGAACAATTGAGTCAGAAGTT

TGAAGAAATAAGATGGCTGATTGAAGAAGTGCGACATAGATTGAAGATTACAGAAAATAG

CTTCGAACAGATAACGTTTATGCAAGCCTTACAACTATTGCTTGAAGTGGAGCAAGAGAT

AAGAACTTTCTCGTTTCAGCTTATTTAATGATAA--------------------------

-----------------------------------

>A_broiler_duck_Korea_Buan2_2014_EPI509712

-------------------ACAAAAACATAATGGACTCCAACACTGTGTCAAGCTTTCAG

GTAGACTGCTTTCTTTGGCATGTCCGCAAACGATTTGCAGACCAAGAACTGGGTGATGCC

CCATTCCTTGACCGGCTTCGCCGAGACCAGAAGTCCCTAAGAGGAAGAGGCAGCACTCTT

GGTCTGGACATCGAGACAGCTACTCGTGCGGGAAAGCAAATAGTGGAGCGGATTCTGGGG

GAAGAATCTGATGAAGCACTTAAAATGAATATTGCTTCTGTACCGACTTCACGCTACCTA

ACTGACATGACTCTTGAAGAAATGTCAAGAGACTGGTTCATGCTCATGCCCAAGCAGAAA

GTAGCAGGTTCTCTCTGCATCAAAATGGACCAGGCAATAATGGATAAAACCATCATACTG

AAAGCAAACTTCAGTGTGATTTTTGATCGGCTGGAAACCCTAATATTACTTAGAGCTTTC

ACAGAAGAAGGAGCAATTGTGGGAGAAATCTCACCATTACCTTCTCTTCCAGGACATACT

GATGAGGATGTCAAAATTGCAATTGGGGTCCTCATCGGAGGGCTTGAATGGAATGATAAC

ACAGTTCGAGTCTCTGAAACTCTACAGAGATTCACTTGGAGAAGCAGTAATGAGGATGGG

AGACCTTCACTCCCTTCAAAACAGAAACGGAAAATGGCGAGAACAATTGAGTCAGAAGTT

CGAGGAAATAAGATGGCTGATTGAGGAAATGCGACATAGATTGAAGATCACAGAGAACAG

CTTCGAACAAATAACGTTTATGCAAGCTTTACAACTATTGCTTGAAGTGGAGCAAGAGAT

AAGAACCTTCTCGTTTCAGCTTATTTAATGATAA--------------------------

-----------------------------------

>A_wigeon_Sakha_1_2014_EPI553471

----AGCAAAAGCAGGGTGACAAAAACATAATGGACTCCAACACTGTGTCAAGCTTTCAG

GTAGACTGCTTTCTTTGGCATGTCCGCAAACGATTTGCAGACCAAGAACTGGGTGATGCC

CCATTCCTTGACCGGCTTCGCCGAGACCAGAAGTCCCTAAGAGGAAGAGGCAGCACTCTT

GGTCTGGACATCGAGACAGCTACTCGTGCGGGAAAGCAAATATTGGAGCGGATTCTGGGG

GAAGAATCTGATGAAGCACTTAAAATGAATATTGCTTCTGTACCGACTTCACGCTACCTA

ACTGACATGACTCTTGAAGAAATGTCAAGAGACTGGTTCATGCTCATGCCCAAGCAGAAA

GTAGCAGGTTCTCTCTGCATCAAAATGGACCAGGCAATAATGGATAAAACCATCATACTG

AAAGCAAACTTCAGTGTGATTTTTGATCGGCTGGAAACCCTAATATTACTTAGAGCTTTC

ACAGAAGAAGGAGCAATTGTGGGAGAAATCTCACCATTACCTTCTCTTCCAGGACATACT

GATGAGGATGTCAAAATTGCAATTGGGGTCCTCATCGGAGGGCTTGAATGGAATGATAAC

ACAGTTCGAGTCTCTGAAACTCTACAGAGATTCACTTGGAGAAGCAGTAATGAGGATGGG

AGACCTTCACTCCCTTCAAAACAGAAACGGAAAATGGCGAGAACAATTGAGTCAGAAGTT

CGAGGAAATAAGATGGCTGATTGAGGAAATGCGACATAGATTGAAGATCACAGAGAACAG

CTTCGAACAAATAACGTTTATGCAAGCTTTACAACTATTGCTTGAAGTGGAGCAAGAGAT

AAGAACCTTCTCGTTTCAGCTTATTTAATGATAAAAAACACCCTTGTTTCTACT------

-----------------------------------

>A_goose_Taiwan_TNO15_2015_EPI690748

------------------------------ATGGACTCCAACACTGTGTCAAGCTTTCAG

GTAGACTGCTTTCTTTGGCATGTCCGCAAACGATTTGCAGACCAAGAACTGGGTGATGCC

CCATTCCTTGACCGGCTTCGCCGAGACCAGAAGTCCCTAAGAGGAAGAGGCAGCACTCTT

GGTCTGGACATCGAGACAGCTACTCGTGCGGGAAAGCAAATAGTGGAGCGGATTCTGGGG

GAAGAATCTGATGAAGCACTTAAAATGAATATTGCTTCTGTACCGACTTCACGCTACCTA

ACTGACATGACTCTTGAAGAAATGTCAAGAGACTGGTTCATGCTCATGCCCAAGCAGAAA

GTAGCAGGTTCTCTCTGCATCAAAATGGACCAGGCAATAATGGATAAAACCATCATACTG

AAAGCAAATTTCAGTGTGATTTTTGATCGGCTGGAAACCCTAATATTACTTAGAGCTTTC

ACAGAAGAAGGAGCAATTGTGGGAGAAATCTCACCATTACCTTCTCTTCCAGGACATACT

GATGAGGATGTCAAAATTGCAATTGGGGTCCTCATCGGAGGGCTTGAATGGAATGATAAC

ACAGTTCGAGTCTCTGAAACTCTACAGAGATTCACTTGGAGAAGCAGTAATGAGGATGGG

AGACCTTCACTCCCTTCAAAACAGAAACGGAAAATGGCGAGAACAATTGAGTCAGAAGTT

CGAGGAAATAAGATGGCTGATTGAGGAAATGCGACATAGATTGAAGACCACAGAGAACAG

CTTCGAACAAATAACGTTTATGCAAGCTTTACAACTATTGCTTGAAGTGGAGCAAGAGAT

AAGAACCTTCTCGTTTCAGCTTATTTAA--------------------------------

-----------------------------------

>A_duck_Nigeria_SK28T_19VIR8424-2_2019_EPI1777110

----AGCAAAAGCAGGGTGGCAAAAACATAATGGATTCCAACACTATGTTAAGCTTTCAG

GTAGATTGCTTTCTTTGGTATGTCCGCAAACGATTCGCAGACCAAGAACTGGGTGATGCC

CCGTTCCTTGACCGGCTTCGCCGAGATCAGAAGTCTTTAAGAGGAAGAGGCAACACTCTT

GGTCTGGGCATTGAAACAGCTACTCGTGCGGGAAAGCAGATAGTGGAGCGAATTCTGGAG

GAAGAATCTGATGAGGCACTTAAAATCACTGTTTCTTCAAGTCCGTCTTCACACTACCTA

ACTGACATGACTCTTGAAGAAATGTCAAGGGACTGGTTCATGCTCATGCCCAAACAGAAA

GTGGCAGGTTCACTTTGCATCAAAATGGACCAGGCAATAATGGATAAAAACATCATATTG

AAAGCAAACTTCAGTGTAATTTTTAACCGGCTGGAAGCTCTAATATTACTTAGAGCTTTT

ACAGAAGACGGAGCAATTGTGGGAGAAATCTCACCGTTACCTTTTTTTCCAGGACATACT

GATGAGGATGTCAAAAATGCAATTGGAGTCCTCATCGGAGGACTTGAGTGGAATGATAAC

ACAGTTCGAGTCTCTGAGACTCTACAGAGATTCGCTTGGAGAAACAGTAATGAGAGTGGG

AGACCTCCACTCCCTCCAAAGTAGAAACGGAAAATGGAGAGAACAATTGGGTCAGAAGTT

TGAAGAAATAAGATGGCTGATTGAAGAAGTGCGACATAGATTAAAGATTACAGAAAATAG

CTTCGAACAGATAACGTTTATGCAAGCCTTACAACTATTGCTTGAAGTGGAACAAGAGAT

AAGAACTTTCTCGTTTCAGCTTATTTGATAATAAAAANCACCCTTGTTTCTACT------

-----------------------------------

>A_mute_swan_Kazakhstan_1-267-20-B_2020_EPI1811588

--------------GGGTGACAAAAACATAATGGATCCCAACACTATGTTAAGCTTTCAG

GTAGACTGTTTTCTTTGGTATGTCCGCAAACGATTCGCAGACCAAGAACTGGGTGATGCC

CCTTTCCTTGACCGGCTTCGCCGAGATCAGAAGTCTTTAAGAGGAAGAGGCACCACTCTT

GGTCTGAGCATCGAAGCAGCTACTCGTGAGGGAAAGCAGATAGTGAAGCGAATTCTGAAG

GAAGAGTCTGATGAGGCACTTAAAATGACTGTTGCTTCAGGTCCGTCTTCACGCTACCTA

ACTGATATGACTCTTGAAGAAATGTCAAGGGACTGGTTCATGCTCATGCCCAAACGGAAA

GTGGCAGGTCCACTTTGCATCAAAATGGACCAGGCAATAATGGATAAAAACATCATATTG

AAAGCAAACTTCAGTGTAATTTTCAACCGGCTGGAAGCTCTAATACTACTTCGAGCTTTC

ACAGAAGAAGGAGCAATTGTGGGAGAAATCTCACCGTTACCTTCTTTTCCAGGACATACT

GATGAGGATGTCAAAAATGCAATTGGGGTCCTCATCGGAGGGCTTGAATGGAATAATAAC

ACAGTTCGGGTCTCTGAAACTCTACAGAGATTCGCTTGGAGAAACAGTGATGAGGATGGG

AGACCTTCACTCCCTCCAAAGTAGAAACGGAAAATGGCGAGAACAATTGGGTCAGAAGTT

TGAAGAAATAAGATGGCTGATTGAAGAAGTGCGACATAGACTGAAGATTACAGAAAATAG

CTTCGAACAGATAACGTTTATGCAAGCCTTACAACTATTGCTTGAAGTGGAACAAGAGAT

AAGAACTTTCTCGTTTCAGCTTATTTGATGATAAAAAACAGTCTATGTT-----------

-----------------------------------

>A_domestic_goose_Kazakhstan_1-248_2-20-B_2020_EPI1811605

CTGGAGGATAAGCAGGGTGACAAAAACATAATGGATTCCAACACTATGTTAAGCTTTCAG

GTAGACTGTTTTCTTTGGTATGTCCGCAAACGATTCGCAGACCAAGAACTGGGTGATGCC

CCTTTCCTTGACCGGCTTCGCCGAGATCAGAAGTCTTTAAGAGGAAGAGGCAACACTCTT

GGTCTGAGCATCGAAGCAGCTACTCGTGAGGGAAAGCAGATAGTGAAGCGAATTCTGAAG

GAAGAGTCTGATGAGGCACTTAAAATGACTGTTGCTTCAGGTCCGTCTTCACGCTACCTA

ACTGATATGACTCTTGAAGAAATGTCAAGGGACTGGTTCATGCTCATGCCCAAACGGAAA

GTGGCAGGTCCACTTTGCATCAAAATGGACCAGGCAATAATGGATAAAAACATCATATTG

AAAGCAAACTTCAGTGTAATTTTCAACCGGCTGGAAGCTCTAATACTACTTCGAGCTTTC

ACAGAAGAAGGAGCAATTGTGGGAGAAATCTCACCGTTACCTTCTTTTCCAGGACATACT

GATGAGGATGTCAAAAATGCAATTGGGGTCCTCATCGGAGGGCTTGAATGGAATAATAAC

ACAGTTCGGGTCTCTGAAACTCTACAGAGATTCGCTTGGAGAAACAGTGATGAGAATGGG

AGACCTTCACTCCCTCCAAAGTAGAAACGGAAAATGGCGAGAACAATTGGGTCAGAAGTT

TGAAGAAATAAGATGGCTGATTGAAGAAGTGCGACATAGACTGAAGATTACAGAAAATAG

CTTCGAACAGATAACATTTATGCAAGCCTTACAACTATTGCTTGAAGTGGAACAAGAGAT

AAGAACTTTCTCGTTTCAGCTTATTTGATGATAA--------------------------

-----------------------------------

>A_domestic_duck_Kazakhstan_1-274-20-B_2020_EPI1811615

----AGCAAAAGCAGGGTGACAAAAACATAATGGATCCCAACACTATGTTAAGCTTTCAG

GTAGACTGTTTTCTTTGGTATGTCCGCAAACGATTCGCAGACCAAGAACTGGGTGATGCC

CCTTTCCTTGACCGGCTTCGCCGAGATCAGAAGTCTTTAAGAGGAAGAGGCACCACTCTT

GGTCTGAGCATCGAAGCAGCTACTCGTGAGGGAAAGCAGGTAGTGAAGCGAATTCTGAAG

GAAGAGTCTGATGAGGCACTTAAAATGACTGTTGCTTCAGGTCCGTCTTCACGCTACCTA

ACTGATATGACTCTTGAAGAAATGTCAAGGGACTGGTTCATGCTCATGCCCAAACGGAAA

GTGGCAGGTCCACTTTGCATCAAAATGGACCAGGCAATAATGGATAAAAACATCATATTG

AAAGCAAACTTCAGTGTAATTTTCAACCGGCTGGAAGCTCTAATACTACTTCGAGCTTTC

ACAGAAGAAGGAGCAATTGTGGGAGAAATCTCACCGTTACCTTCTTTTCCAGGACATACT

GATGAGGATGTCAAAAATGCAATTGGGGTCCTCATCGGAGGGCTTGAATGGAATAATAAC

ACAGTTCGGGTCTCTGAAACTCTACAGAGATTCGCTTGGAGAAACAGTGATGAGGATGGG

AGACCTTCACTCCCTCCAAAGTAGAAACGGAAAATGGCGAGAACAATTGGGTCAGAAGTT

TGAAGAAATAAGATGGCTGATTGAAGAAGTGCGACATAGACTGAAGATTACAGAAAATAG

CTTCGAACAGATAACGTTTATGCAAGCCTTACAACTATTGCTTGAAGTGGAGCAAGAGAT

AAGAACTTTCTCGTTTCAGCTTATTTGATGATAAAAAACA--------------------

-----------------------------------

>A_domestic_goose_Kazakhstan_1-242_2-20-B_2020_EPI1811623

-------------------------ACATAATGGATCCCAACACTATGTTAAGCTTTCAG

GTAGACTGTTTTCTTTGGTATGTCCGCAAACGATTCGCAGACCAAGAACTGGGTGATGCC

CCCTTCCTTGACCGGCTTCGCCGAGATCAGAAGTCTTTAAGAGGAAGAGGCACCACTCTT

GGTCTGAGCATCGAAGCAGCCACTCGTGAGGGAAAGCAGATAGTGAAGCGAATTCTGAAG

GAAGAGTCTGATGAGGCACTTAAAATGACTGTTGCTTCAGGTCCGTCTTCACGCTACCTA

ACTGATATGACTCTTGAAGAAATGTCAAGGGACTGGTTCATGCTCATGCCCAAACGGAAA

GTGGCAGGTCCACTTTGCATCAAAATGGACCAGGCAATAATGGATAAAAACATCATATTG

AAAGCAAACTTCAGTGTAATTTTCAACCGGCTGGAAGCTCTAATACTACTTCGAGCTTTC

ACAGAAGAAGGAGCAATTGTGGGAGAAATCTCACCGTTACCTTCTTTTCCAGGACATACT

GATGAGGATGTCAAAAATGCAATTGGGGTCCTCATCGGAGGGCTTGAATGGAATAATAAC

ACAGTTCGGGTCTCTGAAACTCTACAGAGATTCGCTTGGAGAAACAGTGATGAGGATGGG

AGACCTTCACTCCCTCCAAAGTAGAAACGGAAAATGGCGAGAACAATTGGGTCAGAAGTT

TGAAGAAATAAGATGGCTGATTGAAGAAGTGCGACATAGACTGAAGATTACAGAAAATAG

CTTCGAACAGATAACGTTTATGCAAGCCTTACAACTATTGCTTGAAGTGGAGCAAGAGAT

AAGAACTTTCTCGTTTCAGCTTATTTGATGATAAAAAACACCCTTGTT------------

-----------------------------------

>A_chicken_Iraq_1_2020_EPI1811632

----AGCAAAAGCAGGGTGACAAAAACATAATGGATCCCAACACTATGTTAAGCTTTCAG

GTAGACTGTTTTCTTTGGTATGTCCGCAAACGATTCGCAGACCAAGAACTGGGTGATGCC

CCTTTCCTTGACCGGCTTCGCCGAGATCAGAAGTCTTTAAGAGGAAGAGGCACCACTCTT

GGTCTGAGCATCGAAGCAGCTACTCGTGCGGGAAAGCAGATAGTGAAGCGAATTCTGAAG

GAAGAGTCTGATGAGGCACTTAAAATGACTGTTGCTTCAGGTCCGCCTTTACGCTACCTA

ACTGATATGACTCTTGAAGAAATGTCAAGGGACTGGTTCATGCTCATGCCCAAACAGAAA

GTGGCAGGTCCACTTTGCATCAAAATGGACCAGGCAATAATGGATAAAAACATCATATTG

AAAGCAAACTTCAGTGTAATTTTCAACCGGCTGGAAGCTCTAATACTACTTCGAGCTTTC

ACAGAAGAAGGAGCAATTGTGGGAGAAATCTCACCGTTACCTTCTTTTCCAGGACATACT

GATGAGGATGTCAAAAATGCAATTGGGGTCCTCATCGGAGGGCTTGAATGGAATAATAAC

ACAGTTCGGGTCTCTGAAACTCTACAGAGATTCGCTTGGAGAAACAGTGATGAGGATGGG

AGACCTTCACTCCCTCCAAAGTAGAAACGGAAAATGGCGAGAACAATTGGGTCAGAAGTT

TGAAGAAATAAGATGGCTGATTGAAGAAGTGCGACATAGACTGAAGATTACAGAAAATAG

CTTCGAACAGATAACGTTTATGCAAGCCTTGCAACTATTGCTTGAAGTGGAGCAAGAGAT

AAGAACTTTCTCGTTTCAGCTTATTTGATAATAAAAAACA--------------------

-----------------------------------

>A_whooper_swan_Inner_Mongolia_w1-1_2020_EPI1811648

-----------GCAGGGTGACAAAAACATAATGGATCCCAACACTATGCTAAGCTTTCAG

GTAGACTGTTTTCTTTGGTATGTCCGCAAACGATTCGCAGACCAAGAACTGGGTGATGCC

CCTTTCCTTGACCGGCTTCGCCGAGATCAGAAGTCTTTAAGAGGAAGAGGCACCACTCTT

GGTCTGAGCATCGAAGCAGCTACTCGTGAGGGAAAGCAGATAGTGAAGCGAATTCTGAAG

GAAGAGTCTGATGAGGCACTTAAAATGACTGTTGCTTCAGGTCCGTCTTCACGCTACCTA

ACTGATATGACTCTTGAAGAGATGTCAAGGGACTGGTTCATGCTCATGCCCAAACGGAAA

GTGGCAGGTCCACTTTGCATCAAAATGGACCAGGCAATAATGGATAAAAACATCATATTG

AAAGCAAACTTCAGTGTAATTTTCAACCGGCTGGAAGCTCTAATACTACTTCGAGCTTTC

ACAGAAGAAGGAGCAATTGTGGGAGAAATCTCACCGTTACCTTCTTTTCCAGGACATACT

GATGAGGATGTCAAAAATGCAATTGGGGTCCTCATCGGAGGGCTTGAATGGAATAATAAC

ACAGTTCGGGTCTCTGAAACTCTACAGAGATTCGCTTGGAGAAACAGTGATGAGGATGGG

AGACCTTCACTCCCTCCAAAGTAGAAACGGAAAATGGCGAGAACAATTGGGTCAGAAGTT

TGAAGAAATAAGATGGCTGATTGAAGAAGTGCGACATAGACTGAAGATTACAGAAAATAG

CTTCGAACAGATAACGTTTATGCAAGCCTTACAACTATTGCTTGAAGTGGAACAAGAGAT

AAGAACTTTCTCGTTTCAGCTTATTTGATGATAAAACACACCCTTGTTTC----------

-----------------------------------

>A_mute_swan_Inner_Mongolia_w2-1_2020_EPI1811656

-----------GCAGGGTGACAAAAACATAATGGATTCCAACACTATGCTAAGCTTTCAG

GTAGACTGTTTTCTTTGGTATGTCCGCAAACGATTCGCAGACCAAGAACTGGGTGATGCC

CCTTTCCTTGACCGGCTTCGCCGAGATCAGAAGTCTTTAAGAGGAAGAGGCACCACTCTT

GGTCTGAGCATCGAAGCAGCTACTCGTGAGGGAAAGCAGATAGTGAAGCGAATTCTGAAG

GAAGAGTCTGATGAGGCACTTAAAATGACTGTTGCTTCAGGTCCGTCTTCACGCTACCTA

ACTGATATGACTCTTGAAGAGATGTCAAGGGACTGGTTCATGCTCATGCCCAAACGGAAA

GTGGCAGGTCCACTTTGCATCAAAATGGACCAGGCAATAATGGATAAAAACATCATATTG

AAAGCAAACTTCAGTGTAATTTTCAACCGGCTGGAAGCTCTAATACTACTTCGAGCTTTC

ACAGAAGAAGGAGCAATTGTGGGAGAAATCTCACCGTTACCTTCTTTTCCAGGACATACT

GATGAGGATGTCAAAAATGCAATTGGGGTCCTCATCGGAGGGCTTGAATGGAATAATAAC

ACAGTTCGGGTCTCTGAAACTCTACAGAGATTCGCTTGGAGAAACAGTGATGAGGATGGG

AGACCTTCACTCCCTCCAAAGTAGAAACGGAAAATGGCGAGAACAATTGGGTCAGAAGTT

TGAAGAAATAAGATGGCTGATTGAAGAAGTGCGACATAGACTGAAGATTACAGAAAATAG

CTTCGAACAGATAACGTTTATGCAAGCCTTACAACTATTGCTTGAAGTGGAACAAGAGAT

AAGAACTTTCTCGTTTCAGCTTATTTGATGATAAAAAACACCCTTGTTTC----------

-----------------------------------

>A_goose_Russian_Federation_Kurgan_1345-25_2020_EPI1811682

----------------GTGACAAAAACATAATGGATCCCAACACTATGTTAAGCTTTCAG

GTAGACTGTTTTCTTTGGTATGTCCGCAAACGATTCGCAGACCAAGAACTGGGTGATGCC

CCTTTCCTTGACCGGCTTCGCCGAGATCAGAAGTCTTTAAGAGGAAGAGGCACCACTCTT

GGTCTGAGCATCGAAGCAGCTACTCGTGAGGGAAAGCAGATAGTGAAGCGAATTCTGAAG

GAAGAGTCTGATGAGGCACTTAAAATGACTGTTGCTTCAGGTCCGTCTTCACGCTACCTA

ACTGATATGACTCTTGAAGAAATGTCAAGGGACTGGTTCATGCTCATGCCCAAACGGAAA

GTGGCAGGTCCACTTTGCATCAAAATGGACCAGGCAATAATGGATAAAAACATCATATTG

AAAGCAAACTTCAGTGTAATTTTCAACCGGCTGGAAGCTCTAATACTACTTCGAGCTTTC

ACAGAAGAAGGAGCAATTGTGGGAGAAATCTCACCGTTACCTTCTTTTCCAGGACATACT

GATGAGGATGTCAAAAATGCAATTGGGGTCCTCATCGGAGGGCTTGAATGGAATAATAAC

ACAGTTCGGGTCTCTGAAACTCTACAGAGATTCGCTTGGAGAAACAGTGATGAGGATGGG

AGACCTTCACTCCCTCCAAAGTAGAAACGGAAAATGGCGAGAACAATTGGGTCAGAAGTT

TGAAGAAATAAGATGGCTGATTGAAGAAATGCGACATAGACTGAAGATTACAGAAAATAG

CTTCGAACAGATAACGTTTATGCAAGCCTTACAACTATTGCTTGAAGTGGAACAAGAGAT

AAGAACTTTCTCGTTTCAGCTTATTTGATGATAAAAAACAC-------------------

-----------------------------------

>A_duck_Chelyabinsk_1207-1_2020_EPI1812537

----------------GTGACAAAAACATAATGGATCCCAACACTATGTTAAGCTTTCAG

GTAGACTGTTTCCTTTGGTATGTCCGCAAACGATTCGCAGACCAAGAACTGGGTGATGCC

CCTTTCCTTGACCGGCTTCGCCGAGATCAGAAGTCTTTAAGAGGAAGAGGCACCACTCTT

GGTCTGAGCATCGAAGCAGCTACTCGTGAGGGAAAGCAGATAGTGAAGCGAATTCTGAAG

GAAGAGTCTGATGAGGCACTTAAAATGACTGTTGCTTCAGGTCCGTCTTCACGCTACCTA

ACTGATATGACTCTTGAAGAAATGTCAAGGGACTGGTTCATGCTCATGCCCAAACGGAAA

GTGGCAGGTCCACTTTGCATCAAAATGGACCAGGCAATAATGGATAAAAACATCATATTG

AAAGCAAACTTCAGTGTAATTTTCAACCGGCTGGAAGCTCTAATACTACTTCGAGCTTTC

ACAGAAGAAGGAGCAATTGTGGGAGAAATCTCACCGTTACCTTCTTTTCCAGGACATACT

GATGAGGATGTCAAAAATGCAATTGGGGTCCTCATCGGAGGGCTTGAATGGAATAATAAC

ACAGTTCGGGTCTCTGAAACTCTACAGAGATTCGCTTGGAGAAACAGTGATGAGGATGGG

AGACCTTCACTCCCTCCAAAGTAGAAACGGAAAATGGCGAGAACAATTGGGTCAGAAGTT

TGAAGAAATAAGATGGCTGATTGAAGAAGTGCGACATAGACTGAAGATTACAGAAAATAG

CTTCGAACAGATAACGTTTATGCAAGCCTTACAACTATTGCTTGAAGTGGAACAAGAGAT

AAGAACTTTCTCGTTTCAGCTTATTTGATGATAAAAAACAC-------------------

-----------------------------------

>A_goose_Omsk_0002_2020_EPI1813115

----AGCAAAAGCAGGGTGACAAAAACATAATGGATCCCAACACTATGTTAAGCTTTCAG

GTAGACTGTTTTCTTTGGTATGTCCGCAAACGATTCGCAGACCAAGAACTGGGTGATGCC

CCTTTCCTTGACCGGCTTCGCCGAGATCAGAAGTCTTTAAGAGGAAGAGGCACCACTCTT

GGTCTGAGCATCGAAGCAGCTACTCGTGAGGGAAAGCAGATAGTGAAGCGAATTCTGAAG

GAAGAGTCTGATGAGGCACTTAAAATGACTGTTGCTTCAGGTCCGTCTTCACGCTACCTA

ACTGATATGACTCTTGAAGAAATGTCAAGGGACTGGTTCATGCTCATGCCCAAACGGAAA

GTGGCAGGTCCACTTTGCATCAAAATGGACCAGGCAATAATGGATAAAAACATCATATTG

AAAGCAAACTTCAGTGTAATTTTCAACCGGCTGGAAGCTCTAATACTACTTCGAGCTTTC

ACAGAAGAAGGAGCAATTGTGGGAGAAATCTCACCGTTACCTTCTTTTCCAGGACATACT

GATGAGGATGTCAAAAATGCAATTGGGGTCCTCATCGGAGGGCTTGAATGGAATAATAAC

ACAGTTCGGGTCTCTGAAACTCTACAGAGATTCGCTTGGAGAAACAGTGATGAGGATGGG

AGACCTTCACTCCCTCCAAAGTAGAAACGGAAAATGGCGAGAACAATTGGGTCAGAAGTT

TGAAGAAATAAGATGGCTGATTGAAGAAGTGCGACATAGACTGAAGATTACAGAAAATAG

CTTCGAACAGATAACGTTTATGCAAGCCTTACAACTATTGCTTGAAGTGGAACAAGAGAT

AAGAACTTTCTCGTTTCAGCTTATTTGATGATAAAAAACACCCTTGTTTCTACT------

-----------------------------------

>A_goose_Omsk_01171_2020_EPI1813195

----AGCAAAAGCAGGGTGACAAAAACATAATGGATCCCAACACTATGTTAAGCTTTCAG

GTAGACTGTTTTCTTTGGTATGTCCGCAAACGATTCGCAGACCAAGAACTGGGTGATGCC

CCTTTCCTTGACCGGCTTCGCCGAGATCAGAAGTCTTTAAGAGGAAGAGGCACCACTCTT

GGTCTGAGCATCGAAGCAGCTACTCGTGAGGGAAAGCAGATAGTGAAGCGAATTCTGAAG

GAAGAGTCTGATGAGGCACTTAAAATGACTGTTGCTTCAGGTCCGTCTTCACGCTACCTA

ACTGATATGACTCTTGAAGAAATGTCAAGGGACTGGTTCATGCTCATGCCCAAACGGAAA

GTGGCAGGTCCACTTTGCATCAAAATGGACCAGGCAATAATGGATAAAAACATCATATTG

AAAGCAAACTTCAGTGTAATTTTCAACCGGCTGGAAGCTCTAATACTACTTCGAGCTTTC

ACAGAAGAAGGAGCAATTGTGGGAGAAATCTCACCGTTACCTTCTTTTCCAGGACATACT

GATGAGGATGTCAAAAATGCAATTGGGGTCCTCATCGGAGGGCTTGAATGGAATAATAAC

ACAGTTCGGGTCTCTGAAACTCTACAGAGATTCGCTTGGAGAAACAGTGATGAGGATGGG

AGACCTTCACTCCCTCCAAAGTAGAAACGGAAAATGGCGAGAACAATTGGGTCAGAAGTT

TGAAGAAATAAGATGGCTGATTGAAGAAGTGCGACATAGACTGAAGATTACAGAAAATAG

CTTCGAACAGATAACGTTTATGCAAGCCTTACAACTATTGCTTGAAGTGGAGCAAGAGAT

AAGAACTTTCTCGTTTCAGCTTATTTGATGATAAAAAACACCCTTGTTTCTACT------

-----------------------------------

>A_duck_Omsk_0075_2020_EPI1813275

----AGCAAAAGCAGGGTGACAAAAACATAATGGATCCCAACACTATGTTAAGCTTTCAG

GTAGACTGTTTCCTTTGGTATGTCCGCAAACGATTCGCAGACCAAGAACTGGGTGATGCC

CCTTTCCTTGACCGGCTTCGCCGAGATCAGAAGTCTTTAAGAGGAAGAGGCACCACTCTT

GGTCTGAGCATCGAAGCAGCTACTCGTGAGGGAAAGCAGATAGTGAAGCGAATTCTGAAG

GAAGAGTCTGATGAGGCACTTAAAATGACTGTTGCTTCAGGTCCGTCTTCACGCTACCTA

ACTGATATGACTCTTGAAGAAATGTCAAGGGACTGGTTCATGCTCATGCCCAAACGGAAA

GTGGCAGGTCCACTTTGCATCAAAATGGACCAGGCAATAATGGATAAAAACATCATATTG

AAAGCAAACTTCAGTGTAATTTTCAACCGGCTGGAAGCTCTAATACTACTTCGAGCTTTC

ACAGAAGAAGGAGCAATTGTGGGAGAAATCTCACCGTTACCTTCTTTTCCAGGACATACT

GATGAGGATGTCAAAAATGCAATTGGGGTCCTCATCGGAGGGCTTGAATGGAATAATAAC

ACAGTTCGGGTCTCTGAAACTCTACAGAGATTCGCTTGGAGAAACAGTGATGAGGATGGG

AGACCTTCACTCCCTCCAAAGTAGAAACGGAAAATGGCGAGAACAATTGGGTCAGAAGTT

TGAAGAAATAAGATGGCTGATTGAAGAAGTGCGACATAGACTGAAGATTACAGAAAATAG

CTTCGAACAGATAACGTTTATGCAAGCCTTACAACTATTGCTTGAAGTGGAACAAGAGAT

AAGAACTTTCTCGTTTCAGCTTATTTGATGATAAAAAACACCCTTGTTTCTACT------

-----------------------------------

>A_duck_Omsk_0004_2020_EPI1813331

----AGCAAAAGCAGGGTGACAAAAACATAATGGATCCCAACACTATGTTAAGCTTTCAG

GTAGACTGTTTCCTTTGGTATGTCCGCAAACGATTCGCAGACCAAGAACTGGGTGATGCC

CCTTTCCTTGACCGGCTTCGCCGAGATCAGAAGTCTTTAAGAGGAAGAGGCACCACTCTT

GGTCTGAGCATCGAAGCAGCTACTCGTGAGGGAAAGCAGATAGTGAAGCGGATTCTGAAG

GAAGAGTCTGATGAGGCACTTAAAATGACTGTTGCTTCAGGTCCGTCTTCACGCTACCTA

ACTGATATGACTCTTGAAGAAATGTCAAGGGACTGGTTCATGCTCATGCCCAAACGGAAA

GTGGCAGGTCCACTTTGCATCAAAATGGACCAGGCAATAATGGATAAAAACATCATATTG

AAAGCAAACTTCAGTGTAATTTTCAACCGGCTGGAAGCTCTAATACTACTTCGAGCTTTC

ACAGAAGAAGGAGCAATTGTGGGAGAAATCTCACCGTTACCTTCTTTTCCAGGACATACT

GATGAGGATGTCAAAAATGCAATTGGGGTCCTCATCGGAGGGCTTGAATGGAATAATAAC

ACAGTTCGGGTCTCTGAAACTCTACAGAGATTCGCTTGGAGAAACAGTGATGAGGATGGG

AGACCTTCACTCCCTCCAAAGTAGAAACGGAAAATGGCGAGAACAATTGGGTCAGAAGTT

TGAAGAAATAAGATGGCTGATTGAAGAAGTGCGACATAGACTGAAGATTACAGAAAATAG

CTTCGAACAGATAACGTTTATGCAAGCCTTACAACTATTGCTTGAAGTGGAACAAGAGAT

AAGAACTTTCTCGTTTCAGCTTATTTGATGATAAAAAACACCCTTGTTTCTACT------

-----------------------------------

>A_chicken_Omsk_0112_2020_EPI1813339

----AGCAAAAGCAGGGTGACAAAAACATAATGGATCCCAACACTATGTTAAGCTTTCAG

GTAGACTGTTTTCTTTGGTATGTCCGCAAACGATTCGCAGACCAAGAACTGGGTGATGCC

CCTTTCCTTGACCGGCTTCGCCGAGATCAGAAGTCTTTAAGAGGAAGAGGCACCACTCTT

GGTCTGAGCATCGAAGCAGCTACTCGTGAGGGAAAGCAGATAGTGAAGCGAATTCTGAAG

GAAGAGTCTGATGAGGCACTTAAAATGACTGTTGCTTCAGGTCCGTCTTCACGCTACCTA

ACTGATATGACTCTTGAAGAAATGTCAAGGGACTGGTTCATGCTCATGCCCAAACGGAAA

GTGGCAGGTCCACTTTGCATCAAAATGGACCAGGCAATAATGGATAAAAACATCATATTG

AAAGCAAACTTCAGTGTAATTTTCAACCGGCTGGAAGCTCTAATACTACTTCGAGCTTTC

ACAGAAGAAGGAGCAATTGTGGGAGAAATCTCACCGTTACCTTCTTTTCCAGGACATACT

GATGAGGATGTCAAAAATGCAATTGGGGTCCTCATCGGAGGGCTTGAATGGAATAATAAC

ACAGTTCGGGTCTCTGAAACTCTACAGAGATTCGCTTGGAGAAACAGTGATGAGGATGGG

AGACCTTCACTCCCTCCAAAGTAGAAACGGAAAATGGCGAGAACAATTGGGTCAGAAGTT

TGAAGAAATAAGATGGCTGATTGAAGAAGTGCGACATAGACTGAAGATTACAGAAAATAG

CTTCGAACAGATAACGTTTATGCAAGCCTTACAACTATTGCTTGAAGTGGAGCAAGAGAT

AAGAACTTTCTCGTTTCAGCTTATTTGATGATAAAAAACACCCTTGTTTCTACT------

-----------------------------------

>A_duck_Saratov_29804_2020_EPI1814259

----AGCAAAAGCAGGGTGACAAAAACATAATGGATTCCAACACTATGTTAAGCTTTCAG

GTAGACTGTTTTCTTTGGTATGTCCGCAAACGATTCGCAGACCAAGAACTGGGTGATGCC

CCTTTCCTTGACCGGCTTCGCCGAGATCAGAAGTCTTTAAGAGGAAGAGGCACCACTCTT

GGTCTGAGCATCGAAGCAGCTACTCGTGAGGGAAAGCAGATAGTGAAGCGAATTCTGAAG

GAAGAGTCTGATGAGGCACTTAAAATGACTGTTGCTTCAGGTCCGTCTTCACGCTACCTA

ACTGACATGACTCTTGAAGAAATGTCAAGGGACTGGTTCATGCTCATGCCCAAACGGAAA

GTGGCAGGTCCACTTTGCATCAAAATGGACCAGGCAATAATGGATAAAAACATCATATTG

AAAGCAAACTTCAGTGTAATTTTCAACCGGCTGGAAGCTCTAATACTACTTCGAGCTTTC

ACAGAAGAAGGAGCAATTGTGGGAGAAATCTCACCGTTACCTTCTTTTCCAGGACATACT

GATGAGGATGTCAAAAATGCAATTGGGGTCCTCATCGGAGGGCTTGAATGGAATAATAAC

ACAGTTCGGGTCTCTGAAACTCTACAGAGATTCGCTTGGAGAAACAGTGATGAGGATGGG

AGACCTTCACTCCCTCCAAAGTAGAAACGGAAAATGGCGAGAACAATTGGGTCAGAAGTT

TGAAGAAATAAGATGGCTGATTGAAGAAGTGCGACATAGACTGAAGATTACAGAAAATAG

CTTCGAACAGATAACGTTTATGCAAGCATTACAACTATTGCTTGAAGTGGAACAAGAGAT

AAGAACTTTCTCGTTTCAGCTTATTTGATGATAAAAAACACCCTTGTTTCTACT------

-----------------------------------

>A_goose_Omsk_30001_2020_EPI1814275

----AGCAAAAGCAGGGTGACAAAAACATAATGGATCCCAACACTATGTTAAGCTTTCAG

GTAGACTGTTTCCTTTGGTATGTCCGCAAACGATTCGCAGACCAAGAACTGGGTGATGCC

CCTTTCCTTGACCGGCTTCGCCGAGATCAGAAGTCTTTAAGAGGAAGAGGCACCACTCTT

GGTCTGAGCATCGAAACAGCTACTCGTGAGGGAAAGCAGATAGTGAAGCGAATTCTGAAG

GAAGAGTCTGATGAGGCACTTAAAATGACTGTTGCTTCAGGTCCGTCTTCACGCTACCTA

ACTGATATGACTCTTGAAGAAATGTCAAGGGACTGGTTCATGCTCATGCCCAAACGGAAA

GTGGCAGGTCCACTTTGCATCAAAATGGACCAGGCAATAATGGATAAAAACATCATATTG

AAAGCAAACTTCAGTGTAGTTTTCAACCGGCTGGAAGCTCTAATACTACTTCGAGCTTTC

ACAGAAGAAGGAGCAATTGTGGGAGAAATCTCACCGTTACCTTCTTTTCCAGGACATACT

GATGAGGATGTCAAAAATGCAATTGGGGTCCTCATCGGAGGGCTTGAATGGAATAATAAC

ACAGTTCGGGTCTCTGAAACTCTACAGAGATTCGCTTGGAGAAACAGTGATGAGGATGGG

AGACCTTCACTCCCTCCAAAGTAGAAACGGAAAATGGCGAGAACAATTGGGTCAGAAGTT

TGAAGAAATAAGATGGCTGATTGAAGAAGTGCGACATAGACTGAAGATTACAGAAAATAG

CTTCGAACAGATAACGTTTATGCAAGCCTTACAACTATTGCTTGAAGTGGAACAAGAGAT

AAGAACTTTCTCGTTTCAGCTTATTTGATGATAAAAAACACCCTTGTTTCTACT------

-----------------------------------

>A_goose_Omsk_30003_2020_EPI1814283

----AGCAAAAGCAGGGTGACAAAAACATAATGGATCCCAACACTATGTTAAGCTTTCAG

GTAGACTGTTTTCTTTGGTATGTCCGCAAACGATTCGCAGACCAAGAACTGGGTGATGCC

CCTTTCCTTGACCGACTTCGCCGAGATCAGAAGTCTTTAAGAGGAAGAGGCACCACTATT

GGTCTGAGCATCGAAGCAGCTACTCGTGAGGGAAAGCAGATAGTGAAGCGAATTCTGAAG

GAAGAGTCTGATGAGGCACTTAAAATGACTGTTGCTTCAGGTCCGTCTTCACGCTACCTA

ACTGATATGACTCTTGAAGAAATGTCAAGGGACTGGTTCATGCTCATGCCCAAACGGAAA

GTGGCAGGTCCACTTTGCATCAAAATGGACCAGGCAATAATGGATAAAAACATCATATTG

AAAGCAAACTTCAGTGTAATTTTAAACCGGCTGGAAGCTCTAATACTACTTCGAGCTTTC

ACAGAAGAAGGAGCAATTGTGGGAGAAATCTCACCGTTACCTTCTTTTCCAGGACATACT

GATGAGGATGTCAAAAATGCAATTGGGGTCCTCATCGGAGGGCTTGAATGGAATAATAAC

ACAGTTCGGGTCTCTGAAACTCTACAGAGATTCGCTTGGAGAAACAGTGATGAGGATGGG

AGACCTTCACTCCCTCCAAAGTAGAAACGGAAAATGGCGAGAACAATTGGGTCAGAAGTT

TGAAGAAATAAGATGGCTGATTGAAGAAGTGCGACATAGACTGAAGATTACAGAAAATAG

CTTCGAACAGATAACGTTTATGCAAGCCTTACAACTATTGCTTGAAGTGGAACAAGAGAT

AAGAACTTTCTCGTTTCAGCTTATTTGATGATAAAAAACACCCTTGTTTCTACT------

-----------------------------------

>A_swan_Tumen_1479-2_2020_EPI1814689

----------------GTGACAAAAACATAATGGATTCCAACACTATGTTAAGCTTTCAG

GTAGACTGTTTCCTTTGGTATGTCCGCAAACGATTCGCAGACCAAGAACTGGGTGATGCC

CCTTTCCTTGACCGGCTTCGCCGAGATCAGAAGTCTTTAAGAGGAAGAGGCACCACTCTT

GGTCTGAGCATCGAAGCAGCTACTCGTGAGGGAAAGCAGATAGTGAAGCGAATTCTGAAG

GAAGAGTCTGATGAGGCACTTAAAATGACTGTTGCTTCAGGTCCGTCTTCACGCTACCTA

ACTGATATGACTCTTGAAGAAATGTCAAGGGACTGGTTCATGCTCATGCCCAAACGGAAA

GTGGCAGGTCCACTTTGCATCAAAATGGACCAGGCAATAATGGATAAAAACATCATATTG

AAAGCAAACTTCAGTGTAATTTTCAACCGGCTGGAAGCTCTAATACTACTTCGAGCTTTC

ACAGAAGAAGGAGCAATTGTGGGAGAAATCTCACCGTTACCTTCTTTTCCAGGACATACT

GATGAGGATGTCAAAAATGCAATTGGGGTCCTCATCGGAGGGCTTGAATGGAATAATAAC

ACAGTTCGGGTCTCTGAAACTCTACAGAGATTCGCTTGGAGAAACAGTGATGAGGATGGG

AGACCTTCACTCCCTCCAAAGTAGAAACGGAAAATGGCGAGAACAATTGGGTCAGAAGTT

TGAAGAAATAAGATGGCTGATTGAAGAAGTGCGACATAGACTGAAGATTACAGAAAATAG

CTTCGAACAGATAACGTTTATGCAAGCCTTACAACTATTGCTTGAAGTGGAACAAGAGAT

AAGAACTTTCTCGTTTCAGCTTATTTGATGATAAAAAACAC-------------------

-----------------------------------

>A_Whooper_swan_Mongolia_24_2020_EPI1831868

----AGCGAAAGCAGGGTGACAAAGACATAATGGATTCCAACACTGTGTCAAGTTTTCAG

GTAGATTGCTTTCTTTGGCATGTCCGCAAACGATTTGCAGACCAAGAACTGGGTGATGCC

CCATTCCTTGACCGGCTTCGCCGAGATCAGAAGTCCCTAAGAGGAAGAGGCAACACTCTT

GGTCTGGACATCGAAACAGCTACTCGTGCAGGGAAACAAATAGTGGAGCGGATTCTTGAT

GAGGAACCTGATGAAGCACTTAAAATG---------------CCGACTTCACGTTACCTA

ACTGAAATGACTCTTGAAGAAATGTCGAGGGACTGGTTCATGCTCATGCCCAAGCAGAAA

GTGGTGGGTTCCCTTTGCATCAAAATGGACCAGGCAATAATGGATAAAAGCATCATACTG

AAAGCAAATTTCAGTGTGATTTTTGGCCGGTTAGAGACCCTAATACTACTCAGAGCCTTC

ACAGAAGAGGGAGCAATCGTGGGAGAAATCTCACCATTACCTTCTCTTCCAGGACATACT

GGCGAGGATGTCAAAAATGCAATTGGCGTCCTCATCGGAGGACTTGAATGGAATGATAAC

ACAGTTCGGGTCTCTGAAGTTATACAGAGATTCGCTTGGGGAAGCAGTGATGAGGGTAGG

AGACTTCCACTCCCTCCAAATCAGAAACGGAAACTGGCGAGAGCAATTGAGTCAGAAGTT

TGAAGAAATAAGGTGGCTGATTGAAGAAGTACGACATAGATTGAAAATTACAGAAAACAG

CTTCGAACAGATAACTTTTATGCAAGCCTTACAACTACTGCTTGAAGTGGAGCAAGAGAT

AAGAGCCTTCTCGTTTCAGCTTATTTAATGATAAAAAACACCCTTGTTTCTACT------

-----------------------------------

>A_Whooper_swan_Mongolia_25_2020_EPI1831876

----AGCGAAAGCAGGGTGACAAAGACATAATGGATTCCAACACTGTGTCAAGTTTTCAG

GTAGATTGCTTTCTTTGGCATGTCCGCAAACGATTTGCAGACCAAGAACTGGGTGATGCC

CCATTCCTTGACCGGCTTCGCCGAGATCAGAAGTCCCTAAGAGGAAGAGGCAACACTCTT

GGTCTGGACATCGAAACAGCTACTCGTGCAGGGAAACAAATAGTGGAGCGGATTCTTGAT

GAGGAACCTGATGAAGCATTTAAAATG---------------CCGACTTCACGTTACCTA

ACTGAAATGACTCTTGAAGAAATGTCGAGGGACTGGTTCATGCTCATGCCCAAGCAGAAA

GTGGTGGGTTCCCTTTGCATCAAAATGGACCAGGCAATAATGGATAAAAGCATCATACTG

AAAGCAAATTTCAGTGTGATTTTTGGCCGGTTAGAGACCCTAATACTACTCAGAGCTTTC

ACAGAAGAGGGAGCAATCGTGGGAGAAATCTCACCATTACCTTCTCTTCCAGGACATACT

GGCGAGGATGTCAAAAATGCAATTGGCGTCCTCATCGGAGGACTTGAATGGAATGATAAC

ACAGTTCGGGTCTCTGAAGTTATACAGAGATTCGCTTGGGGAAGCAGTGATGAGGGTAGG

AGACTTCCACTCCCTCCAAATCAGAAACGGAAACTGGCGAGAGCAATTGAGTCAGAAGTT

TGAAGAAATAAGGTGGCTGATTGAAGAAGTACGACATAGATTGAAAATTACAGAAAACAG

CTTCGAACAGATAACTTTTATGCAAGCCTTACAACTACTGCTTGAAGTGGAGCAAGAGAT

AAGAGCCTTCTCGTTTCAGCTTATTTAATGATAAAAAACACCCTTGTTTCTACT------

-----------------------------------

>A_chicken_Kazakhstan_Kn-3_2020_EPI1839255

------------------------------ATGGATTCCAACACTATGTTAAGCTTTCAG

GTAGACTGTTTTCTTTGGTATGTCCGCAAACGATTCGCAGACCAAGAACTGGGTGATGCC

CCTTTCCTTGACCGGCTTCGCCGAGATCAGAAGTCTTTAAGAGGAAGAGGCACCACTCTT

GGTCTGAGCATCGAAGCAGCTACTCGTGAGGGAAAGCAGATAGTGAAGCGAATTCTGAAG

GAAGAGTCTGATGAGGCACTTAAAATGACTGTTGCTTCAGGTCCGTCTTCACGCTACCTA

ACTGATATGACTCTTGAAGAAATGTCAAGGGACTGGTTCATGCTCATGCCCAAACGGAAA

GTGGCAGGTCCACTTTGCATCAAAATGGACCAGGCAATAATGGATAAAAACATCATATTG

AAAGCAAACTTCAGTGTAATTTTCAACCGGCTGGAAGCTCTAATACTACTTCGAGCTTTC

ACAGAAGAAGGAGCAATTGTGGGAGAAATCTCACCGTTACCTTCTTTTCCAGGACATACT

GATGAGGATGTCAAAAATGCAATTGGGGTCCTCATCGGAGGGCTTGAATGGAATAATAAC

ACAGTTCGGGTCTCTGAAACTCTACAGAGATTCGCTTGGAGAAACAGTGATGAGGATGGG

AGACCTTCACTCCCTCCAAAGTAGAAACGGAAAATGGCGAGAACAATTGGGTCAGAAGTT

TGAAGAAATAAGATGGCTGATTGAAGAAGTGCGACATAGACTGAAGATTACAGAAAATAG

CTTCGAACAGATAACGTTTATGCAAGCCTTACAACTATTGCTTGAGGTGGAGCAAGAGAT

AAGAACTTTCTCGTTTCAGCTTATT-----------------------------------

-----------------------------------

>A_chicken_Kazakhstan_Kn-6_2020_EPI1839263

------------------------------ATGGATTCCAACACTATGTTAAGCTTTCAG

GTAGACTGTTTTCTTTGGTATGTCCGCAAACGATTCGCAGACCAAGAACTGGGTGATGCC

CCTTTCCTTGACCGGCTTCGCCGAGATCAGAAGTCTTTAAGAGGAAGAGGCACCACTCTT

GGTCTGAGCATCGAAGCAGCTACTCGTGAGGGAAAGCAGATAGTGAAGCGAATTCTGAAG

GAAGAGTCTGATGAGGCACTTAAAATGACTGTTGCTTCAGGTCCGTCTTCACGCTACCTA

ACTGATATGACTCTTGAAGAAATGTCAAGGGACTGGTTCATGCTCATGCCCAAACGGAAA

GTGGCAGGTCCACTTTGCATCAAAATGGACCAGGCAATAATGGATAAAAACATCATATTG

AAAGCAAACTTCAGTGTAATTTTCAACCGGCTGGAAGCTCTAATACTACTTCGAGCTTTC

ACAGAAGAAGGAGCAATTGTGGGAGAAATCTCACCGTTACCTTCTTTTCCAGGACATACT

GATGAGGATGTCAAAAATGCAATTGGGGTCCTCATCGGAGGGCTTGAATGGAATAATAAC

ACAGTTCGGGTCTCTGAAACTCTACAGAGATTCGCTTGGAGAAACAGTGATGAGGATGGG

AGACCTTCACTCCCTCCAAAGTAGAAACGGAAAATGGCGAGAACAATTGGGTCAGAAGTT

TGAAGAAATAAGATGGCTGATTGAAGAAGTGCGACATAGACTGAAGATTACAGAAAATAG

CTTCGAACAGATAACGTTTATGCAAGCCTTACAACTATTGCTTGAGGTGGAACAAGAGAT

AAGAACTTTCTCGTTTCAGCTTATT-----------------------------------

-----------------------------------

>A_Muscovy_duck_China_FJFZ21_H5N6_2020_EPI1841916

------------------------------ATGGACTCCAACACTGTGTCAAGTTTTCAG

GTAGACTGCTTTCTTTGGCATGTCCGCAAACGATTTGCAGACCAAGAACTGGGTGATGCC

CCATTCCTTGACCGGCTTCGCCGAGATCAGAAGTCCCTAAGAGGAAGAGGCAACACTCTT

GGTCTGGACATCGAAACAGCTACTCGTGCGGGAAAGCAAATAGTGGAGCGGATTCTTGAT

GAGGAACCTGATGAAGCACTTAAAATG---------------CCGACTTCACGTTACCTA

ACTGAAATGACTCTCGAAGAAATGTCGAGGGACTGGTTCATGCTCATGCCCAAGCAGAAG

GTGATTGGTTCCCTTTGCATCAAAATGGACCAGGCAATAATGGATAAAAGCATCATACTG

AAAGCAAATTTCAGTGTGATTCTTGGCCGGTTAGAGACCCTAATACTACTCAGAGCTTTC

ACAGAAGAAGGAGCAATCGTGGGAGAAATCTCACCATTACCTTCTCTTCCAGGACATACT

GGCGAGGATGTCAAAAATGCAATTGGCGTCCTCATCGGAGGACTTGAATGGAATGATAAC

ACAGTTCGGGTCTCTGAAGTTATACAGAGATTCGCTTGGAGAAGCAGTGATGAGGGTGGG

AGACTTCCACTCCCTCCAAATCAGAAACGGAAACTGGCGGGAGCAATTAAGTCAGAAGTT

TGA---------------------------------------------------------

------------------------------------------------------------

------------------------------------------------------------

-----------------------------------

>A_mute_swan_Czech_Republic_1410-2_2021_EPI1843610

------------------------AACATAATGGATTCCAACACTATGTTAAGCTTTCAG

GTAGACTGTTTTCTTTGGTATGTCCGCAAACGATTCGCAGACCAAGAACTGGGTGATGCC

CCTTTCCTTGACCGGCTTCGCCGAGATCAGAAGTCTTTAAGAGGAAGAGGCACCACTCTT

GGTCTGAGCATCGAAGCAGCTACTCGTGAGGGAAAGCAGATAGTGAAGCGAATTCTGAAG

GAAGAGTCTGATGAGGCACTTAAAATGACTGTTGCTTCAGGTCCGTCTTCACGCTACCTA

ACTGATATGACTCTTGAAGAAATGTCAAGGGACTGGTTCATGCTCATGCCCAAACGGAAA

GTGGCAGGTCCACTTTGCATCAAAATGGACCAGGCAATAATGGATAAAAACATCATATTG

AAAGCAAACTTCAGTGTAATTTTCAACCGGCTGGAAGCTCTAATACTACTTCGAGCTTTC

ACAGAAGAAGGAGCAATTGTGGGAGAAATCTCACCGTTACCTTCTTTTCCAGGACATACT

GATGAGGATGTCAAAAATGCAATTGGGGTCCTCATCGGAGGGCTTGAATGGAATAATAAC

ACAGTTCGGGTCTCTGAAACTCTACAGAGATTCGCTTGGAGAAACAGTGATGAGGATGGG

AGACCTTCACTCCCTCCAAAGTAGAAACGGAAAATGGCGAGAACAATTGGGTCAGAAGTT

TGAAGAAATAAGATGGCTGATTGAAGAAGTGCGACATAGACTGAAGATTACAGAAAATAG

CTTCGAACAGATAACGTTTATGCAAGCCTTACAACTATTGCTTGAAGTGGAACAAGAGAT

AAGAACTTTCTCGTTTCAGCTTATTTGATGATAA--------------------------

-----------------------------------

>A_chicken_Czech_Republic_1566-1_2021_EPI1844087

------------------------AACATAATGGATCCCAACACTATGTTAAGCTTTCAG

GTAGACTGTTTTCTTTGGTATGTCCGCAAACGATTCGCAGACCAAGAACTGGGTGATGCC

CCTTTCCTTGACCGGCTTCGCCGAGATCAGAAGTCTTTAAGAGGAAGAGGCACCACTCTT

GGTCTGAGCATCGAAGCAGCTACTCGTGAGGGAAAGCAGATAGTGAAGCGAATTCTGAAG

GAAGAGTCTGATGAGGCACTTAAAATGACTGTTGCTTCAGGTCCGTCTTCACGCTACCTA

ACTGATATGACTCTTGAAGAAATGTCAAGGGACTGGTTCATGCTCATGCCCAAACGGAAA

GTGGCAGGTCCACTTTGCATCAAAATGGACCAGGCAATAATGGATAAAAACATCATATTG

AAAGCAAACTTCAGTGTAATTTTCAACCGGCTGGAAGCTCTAATACTACTTCGAGCTTTC

ACAGAAGAAGGAGCAATTGTGGGAGAAATCTCACCGTTACCTTCTTTTCCAGGACATACT

GATGAGGATGTCAAAAATGCAATTGGGGTCCTCATCGGAGGGCTTGAATGGAATAATAAC

ACAGTTCGGGTCTCTGAAACTCTACAGAGATTCACTTGGAGAAACAGTGATGAGGATGGG

AGACCTTCACTCCCTCCAAAGTAGAAACGGAAAATGGCGAGAACAATTGGGTCAGAAGTT

TGAAGAAATAAGATGGCTGATTGAAGAAGTGCGACATAGACTGAAGATTACAGAAAATAG

CTTCGAACAGATAACGTTTATGCAAGCCTTACAACTATTGCTTGAAGTGGAGCAAGAGAT

AAGAACTTTCTCGTTTCAGCTTATTTGATGATAA--------------------------

-----------------------------------

>A_chicken_Korea_H008_2021_EPI1846532

------------------------------ATGGATCCCAACACTATGTTAAGCTTTCAG

GTAGACTGTTTTCTTTGGTATGTCCGCAAACGATTCGCAGACCAAGAACTGGGTGATGCC

CCTTTCCTTGACCGGCTTCGCCGAGATCAGAAGTCTTTAAGAGGAAGAGGCACCACTCTT

GGTCTGAGCATCGAAGCAGCTACTCGTGAGGGAAAGCAGATAGTGAAGCGAATTCTGAAG

GAAGAGTCTGATGAGGCACTTAAAATGACTGCTGCTTCAGGTCCGTCTTCACGCTACCTA

ACTGATATGACTCTTGAAGAGATGTCAAGGGACTGGTTCATGCTCATGCCCAAACGGAAA

GTGGCAGGTCCACTTTGCATCAAAATGGACCAGGCAATAATGGATAAAAACATCATATTG

AAAGCAAATTTCAGTGTAATTTTCAACCGGCTGGAAGCTCTAATACTACTTCGAGCTTTC

ACAGAAGAAGGAGCAATTGTGGGAGAAATCTCACCGTTACCTTCTTTTCCAGGACATACT

GATGAGGATGTCAAAAATGCAATTGGGGTCCTCATCGGAGGGCTTGAATGGAATAATAAC

ACAGTTCGGCTCTCTGAAACTCTACAGAGATTCGCTTGGAGAAACAGTGATGAGGATGGG

AGACCTTCACTCCCTCCAAAGTAGAAACGGAAAATGGCGAGAACAATTGGGTCAGAAGTT

TGAAGAAATAAGATGGCTGATTGAAGAAGTGCGACATAGACTGAAGATTACAGAAAATAG

CTTCGAACAGATAACGTTTATGCAAGCCTTACAACTATTGCTTGAAGTGGAACAAGAGAT

AAGAACTTTCTCGTTTCAGCTTATTTGA--------------------------------

-----------------------------------

>A_mallard_Korea_WA820_2020_EPI1846596

------------------------------ATGGATCCCAACACTATGCTAAGCTTTCAG

GTAGACTGTTTTCTTTGGTATGTCCGCAAACGATTCGCAGACCAAGAACTGGGTGATGCC

CCTTTCCTTGACCGGCTTCGCCGAGATCAGAAGTCTTTAAGAGGAAGAGGCACCACTCTT

GGTCTGAGCATCGAAGCAGCTACTCGTGAGGGAAAGCAGATAGTGAAGCGAATTCTGAAG

GAAGAGTCTGATGAGGCACTTAAAATGACTGTTGCTTCAGGTCCGTCTTCACGCTACCTA

ACTGATATGACTCTTGAAGAGATGTCAAGGGACTGGTTCATGCTCATGCCCAAACGGAAA

GTGGCAGGTCCACTTTGCATCAAAATGGACCAGGCAATAATGGATAAAACCATCATATTG

AAAGCAAACTTCAGTGTAATTTTCAACCGGCTGGAAGCTCTAATACTACTTCGAGCTTTC

ACAGAAGAAGGAGCAATTGTGGGAGAAATCTCACCGTTACCTTCTTTTCCAGGACATACT

GATGAGGATGTCAAAAATGCAATTGGGGTCCTCATCGGAGGGCTTGAATGGAATAATAAC

ACAGTTCGGGTCTCTGAAACTCTACAGAGATTCGCTTGGAGAAACAGTGATGAGGATGGG

AGACCTTCACTCCCTCCAAAATAGAAACGGAAAATGGCGAGAACAATTGGGTCAGAAGTT

TGAAGAAATAAGATGGCTGATTGAAGAAGTGCGACATAGACTGAAGATTACAGAAAATAG

CTTCGAGCAGATAACGTTTATGCAAGCCTTACAACTATTGCTTGAAGTGGAACAAGAGAT

AAGAACTTTCTCGTTTCAGCTTATTTGA--------------------------------

-----------------------------------

>A_duck_Korea_H016_2021_EPI1846700

------------------------------ATGGATCCCAACACTATGCTAAGCTTTCAG

GTAGACTGTTTTCTTTGGTATGTCCGCAAACGATTCGCAGACCAAGAACTGGGTGATGCC

CCTTTCCTTGACCGGCTTCGCCGAGATCAGAAGTCTTTAAGAGGAAGAGGCACCACTCTT

GGTCTGAGCATCGAAGCAGCTACTCGTGAGGGAAAGCAGATAGTGAAGCGAATTCTGAAG

GAAGAGTCTGATGAGGCACTTAAAATGACTGTTGCTTCAGGTCCGTCTTCACGCTACCTA

ACTGATATGACTCTTGAAGAGATGTCAAGGGACTGGTTCATGCTCATGCCCAAACGGAAA

GTGGCAGGTCCACTTTGCATCAAAATGGACCAGGCAATAATGGATAAAACCATCATATTG

AAAGCAAACTTCAGTGTAATTTTCAACCGGCTGGAAGCTCTAATACTACTTCGAGCTTTC

ACAGAAGAAGGAGCAATTGTGGGAGAAATCTCACCGTTACCTTCTTTTCCAGGACATACT

GATGAGGATGTCAAAATTGCAATTGGGGTCCTCATCGGAGGGCTTGAATGGAATAATAAC

ACAGTTCGGGTCTCTGAAACTCTACAGAGATTCGCTTGGAGAAACAGTGATGAGGATGGG

AGACCTTCACTCCCTCCAAAATAGAAACGGAAAATGGCGAGAACAATTGGGTCAGAAGTT

TGAAGAAATAAGATGGCTGATTGAAGAAGTGCGACATAGACTGAAGATTACAGAAAATAG

CTTCGAGCAGATAACGTTTATGCAAGCCTTACAACTATTGCTTGAAGTGGAACAAGAGAT

AAGAACTTTCTCGTTTCAGCTTATTTGA--------------------------------

-----------------------------------

>A_chicken_Astrakhan_321-01_2020_EPI1846973

----AGCAAAAGCAGGGTGACAAAAACATAATGGATCCCAACACTATGTTAAGCTTTCAG

GTAGACTGTTTTCTTTGGTATGTCCGCAAACGATTCGCAGACCAAGAACTGGGTGATGCC

CCTTTCCTTGACCGGCTTCGCCGAGATCAGAAGTCTTTAAGAGGAAGAGGCACCACTCTT

GGTCTGAGCATCGAAGCAGCTACTCGTGAGGGAAAGCAGATAGTGAAGCGAATTCTGAAG

GAAGAGTCTGATGAGGCACTTAAAATGACTGTTGCTTCAGGTCCGTCTTCACGCTACCTA

ACTGATATGACTCTTGAAGAAATGTCAAGGGACTGGTTCATGCTCATGCCCAAACGAAAA

GTGGCAGGTCCACTTTGCATCAAAATGGACCAGGCAATAATGGATAAAAACATCATATTG

AAAGCAAACTTCAGTGTAATTTTCAACCGGCTGGAAGCTCTAATACTACTTCGAGCTTTC

ACAGAAGAAGGAGCAATTGTGGGAGAAATCTCACCGTTACCTTCTTTTCCAGGACATACT

GATGAGGATGTCAAAAATGCAATTGGGGTCCTCATCGGAGGGCTTGAATGGAATAATAAC

ACAGTTCGGGTCTCTGAAACTCTACAGAGATTCGCTTGGAGAAACAGTGATGAGGATGGG

AGACCTTCACTCCCTCCAAAGTAGAAACGGAAAATGGCGAGAACAATTGGGTCAGAAGTT

TGAAGAAATAAGATGGCTGATTGAAGAAGTGCGACATAGACTGAAGATTACAGAAAATAG

CTTCGAACAGATAACGTTTATGCAAGCCTTACAACTATTGCTTGAAGTGGAGCAAGAGAT

AAGAACTTTCTCGTTTCAGCTTATTTGATGATAAAAAACACCCTTGTTTCTACT------

-----------------------------------

>A_crane_Kagoshima_KU-93_2021_EPI1848531

------------------------------ATGGATCCCAACACTATGTTAAGCTTTCAG

GTAGACTGTTTTCTTTGGTATGTCCGCAAACGATTCGCAGACCAAGAACTGGGTGATGCC

CCTTTCCTTGACCGGCTTCGCCGAGATCAGAAGTCTTTAAGAGGAAGAGGCACCACTCTT

GGTCTGAGCATCGAAGCAGCTACTCGTGAGGGAAAGCAGATAGTGAAGCGAATTCTGAAG

GAAGAGTCTGATGAGGCACTTAAAATGACTGTTGCTTCAGGTCCGTCTTCACGCTACCTA

ACTGATATGACTCTTGAAGAGATGTCAAGGGACTGGTTCATGCTCATGCCCAAACGGAAA

GTGGCAGGTCCACTTTGCATCAAAATGGACCAGGCAATAATGGATAAAAACATCATATTG

AAAGCAAATTTCAGTGTAATTTTCAACCGGCTGGAAGCTCTAATACTACTTCGAGCTTTC

ACAGAAGAAGGAGCAATTGTGGGAGAAATCTCACCGTTACCTTCTTTTCCAGGACATACT

GATGAGGATGTCAAAAATGCAATTGGGGTCCTCATCGGAGGGCTTGAATGGAATAATAAC

ACAGTTCGGGTCTCTGAAACTCTACAGAGATTCGCTTGGAGAAACAGTGATGAGGATGGG

AGACCTTCACTCCCTCCAAAGCAGAAACGGAAAATGGCGAGAACAATTGGGTCAGAAGTT

TGAAGAAATAAGATGGCTGATTGAAGAAGTGCGACATAGACTGAAGATTACAGAAAATAG

CTTCGAACAGATAACGTTTATGCAAGCCTTACAACTATTGCTTGAAGTGGAACAAGAGAT

AAGAACTTTCTCGTTTCAGCTTATTTGA--------------------------------

-----------------------------------

>A_mallard_Kagoshima_KU-d89_2021_EPI1848542

------------------------------ATGGATCCCAACACTATGTTAAGCTTTCAG

GTAGACTGTTTTCTTTGGTATGTCCGCAAACGATTCGCAGACCAAGAACTGGGTGATGCC

CCTTTCCTTGACCGGCTTCGCCGAGATCAGAAGTCTTTAAGAGGAAGAGGCACCACTCTT

GGTCTGAGCATCGAAGCAGCTACTCGTGAGGGAAAGCAGATAGTGAAGCGAATTCTGAAG

GAAGAGTCTGATGAGGCACTTAAAATGACTGTTGCTTCAGGTCCGTCTTCACGCTACCTA

ACTGATATGACTCTTGAAGAGATGTCAAGGGACTGGTTCATGCTCATGCCCAAACGGAAA

GTGGCAGGTCCACTTTGCATCAAAATGGACCAGGCAATAATGGATAAAAACATCATATTG

AAAGCAAATTTCAGTGTAATTTTCAACCGGCTGGAAGCTCTAATACTACTTCGAGCTTTC

ACAGAAGAAGGAGCAATTGTGGGAGAAATCTCACCGTTACCTTCTTTTCCAGGACATACT

GATGAGGATGTCAAAAATGCAATTGGGGTCCTCATCGGAGGGCTTGAATGGAATAATAAC

ACAGTTCGGGTCTCTGAAACTCTACAGAGATTCGCTTGGAGAAACAGTGATGAGGATGGG

AGACCTTCACTCCCTCCAAAGTAGAAACGGAAAATGGCGAGAACAATTGGGTCAGAAGTT

TGAAGAAATAAGATGGCTGATTGAAGAAGTGCGACATAGACTGAAGATTACAGAAAATAG

CTTCGAACAGATAACGTTTATGCAAGCCTTACAACTATTGCTTGAAGTGGAACAAGAGAT

AAGAACTTTCTCGTTTCAGCTTATTTGA--------------------------------

-----------------------------------

>A_chicken_Kostroma_304-06_2020_EPI1848640

----AGCAAAAGCAGGGTGACAAAAACATAATGGATCCCAACACTATGTTAAGCTTTCAG

GTAGACTGTTTTCTTTGGTATGTCCGCAAACGATTCGCAGACCAAGAACTGGGTGATGCC

CCTTTCCTTGACCGGCTTCGCCGAGATCAGAAGTCTTTAAGAGGAAGAGGCACCACTCTT

GGTCTGAGCATCGAAGCAGCTACTCATGAGGGGAAGCAGATAGTGAAGCGAATTCTGAAG

GAAGAGTCTGATGAGGCATTTAAAATGACTGTTGCTTCAGGTCCGTCTTCACGCTACCTA

ACTGATATGACTCTTGAAGAGATGTCAAGGGACTGGTTCATGCTCATGCCCAAACGGAAA

GTGGCAGGTCCACTTTGCATCAAAATGGACCAGGCAATAATGGATAAAAACATCATATTG

AAAGCAAACTTCAGTGTAATTTTCAACCGGCTGGAAGCTCTAATACTACTTCGAGCTTTC

ACAGAAGAAGGAGCAATTGTGGGAGAAATCTCACCGTTACCTTCTTTTCCAGGACATACT

GATGAGGATGTCAAAAATGCAATTGGGGTCCTCATCGGAGGGCTTGAATGGAATAATAAC

ACAGTTCGGGTCTCTGAAACTCTACAGAGATTCGCTTGGAGAAACAGTGATGAGGATGGG

AGACCTTCACTCCCTCCAAAGTAGAAACGGAAAATGGCGAGAACAATTGGGTCAGAAGTT

TGAAGAAATAAGATGGCTGATTGAAGAAGTGCGACATAGACTGAAGATTACAGAAAATAG

CTTCGAACAGATAACGTTTATGCAAGCCTTACAACTATTGCTTGAAGTGGAACAAGAGAT

AAGAACTTTCTCGTTTCAGCTTATTTGATGACAAAAAACACCCTTGTTTCTACT------

-----------------------------------

>A_chicken_Rostov-on-Don_308-02_2020_EPI1848664

----AGCAAAAGCAGGGTGACAAAAACATAATGGATCCCAACACTATGTTAAGCTTTCAG

GTAGACTGTTTTCTTTGGTATGTCCGCAAACGATTCGCAGACCAAGAACTGGGTGATGCC

CCTTTCCTTGACCGGCTTCGCCGAGATCAGAAGTCTTTAAGAGGAAGAGGCACCACTCTT

GGTCTGAGCATCGAAGCAGCTACCCGTGAGGGAAAGCAGATAGTGAAGCGAATTCTGAAG

GAAGAGTCTGATGAGGCACTTAAAATGACTGTTGCTTCAGGTCCGTCTTCACGCTACCTA

ACTGATATGACTCTTGAAGAAATGTCAAGGGACTGGTTCATGCTCATGCCCAAACGGAAA

GTGGCAGGTCCACTTTGCATCAAAATGGACCAGGCAATAATGGATAAAAACATCATATTG

AAAGCAAACTTCAGTGTAATTTTCAACCGGCTGGAAGCTCTAATACTACTCCGAGCTTTC

ACAGAAGAAGGAGCAATTGTGGGAGAAATCTCACCGTTACCTTCTTTTCCAGGACATACT

GATGAGGATGTCAAAAATGCAATTGGGGTCCTCATCGGAGGGCTTGAATGGAATAATAAC

ACAGTTCGGGTCTCTGAAACTCTACAGAGATTCGCTTGGAGAAACAGTGATGAGGATGGG

AGACCTTCACTCCCTCCAAAGTAGAAACGGAAAATGGCGAGAACAATTGGGTCAGAAGTT

TGAAGAAATAAGATGGCTGATTGAAGAAATGCGACATAGACTGAAGATTACAGAAAATAG

CTTCGAACAGATAACGTTTATGCAAGCCTTACAACTATTGCTTGAAGTGGAACAAGAGAT

AAGAACTTTCTCGTTTCAGCTTATTTGATGATAAAAAACACCCTTGTTTCTACT------

-----------------------------------

>A_turkey_Stavropol_320-02_2020_EPI1848696

----AGCAAAAGCAGGGTGACAAAAACATAATGGATCCCAACACTATGTTAAGCTTTCAG

GTAGACTGTTTTCTTTGGTATGTCCGCAAACGATTCGCAGACCAAGAACTGGGTGATGCC

CCTTTCCTTGACCGGCTTCGCCGAGATCAGAAGTCTTTAAGAGGAAGAGGCACCACTCTT

GGTCTGAGCATCGAAGCAGCTACTCGTGAGGGAAAGCAGATAGTGAAGCGAATTCTGAAG

GAAGAGTCTGATGAGGCACTTAAAATGACTGTTGCTTCAGGTCCGTCTTCACGCTACCTA

ACTGATATGACTCTTGAAGAAATGTCAAGGGACTGGTTCATGCTCATGCCCAAACGGAAA

GTGGCAGGTCCACTTTGCATCAAAATGGACCAGGCAATAATGGATAAAAACATCATATTG

AAAGCAAACTTCAGTGTAATTTTCAACCGGCTGGAAGCTCTAATACTACTTCGAGCTTTC

ACAGAAGAAGGAGCAATTGTGGGAGAAATCTCACCGTTACCTTCTTTTCCAGGACATACT

GATGAGGATGTCAAAAATGCAATTGGGGTCCTCATCGGAGGGCTTGAATGGAATAATAAC

ACAGTTCGGGTCTCTGAAACTCTACAGAGATTCGCTTGGAGAAACAGTGATGAGGATGGG

AGACCTTCACTCCCTCCAAAATAGAAACGGAAAATGGCGAGAACAATTGGGTCAGAAGTT

TGAAGAAATAAGATGGCTGATTGAAGAAATGCGACATAGACTGAAGATTACAGAAAATAG

CTTCGAACAGATAACGTTTATGCAAGCCTTACAACTATTGCTTGAAGTGGAACAAGAGAT

AAGAACTTTCTCGTTTCAGCTTATTTGATGATAAAAAACACCCTTGTTTCTACT------

-----------------------------------

>A_mute_swan_North_Ossetia-Alania_325-03_2020_EPI1848728

----AGCAAAAGCAGGGTGACAAAAACATAATGGATCCCAACACTATGTTAAGCTTTCAG

GTAGACTGTTTTCTTTGGTATGTCCGCAAACGATTCGCAGACCAAGAACTGGGTGATGCC

CCTTTCCTTGACCGGCTTCGCCGAGATCAGAAGTCTTTAAGAGGAAGAGGCACCACTCTT

GGTCTGAGCATCGAAGCAGCTACTCGTGAGGGAAAGCAGATAGTGAAGCGAATTCTGAAG

GAAGAGTCTGATGAGGCACTTAAAATGACTGCTGCTTCAGGTCCGTCTTCACGCTACCTA

ACTGATATGACTCTTGAAGAAATGTCAAGGGACTGGTTCATGCTCATGCCCAAACGGAAA

GTGGCAGGTCCACTTTGCATCAAAATGGACCAGGCAATAATGGATAAAAACATCATATTG

AAAGCAAACTTCAGTGTAATTTTCAACCGGCTGGGAGCTCTAATACTACTTCGAGCTTTC

ACAGAAGAAGGAGCAATTGTGGGAGAAATCTCACCGTTACCTTCTTTTCCAGGACATACT

GATGAGGATGTCAAAAATGCAATTGGGGTCCTCATCGGAGGGCTTGAATGGAATAATAAC

ACAGTTCGGGTCTCTGAAACTCTACAGAGATTCGCTTGGAGAAACAGTGATGAGGATGGG

AGACCTTCACTCCCTCCAAAGTAGAAACGGAAAATGGCGAGAACAATTGGGTCAGAAGTT

TGAAGAAATAAGATGGCTGATTGAAGAAGTGCGACATAGACTGAAGATTACAGAAAATAG

CTTCGAACAGATAACGTTTATGCAAGCCTTACAACTATTGCTTGAAGTGGAGCAAGAGAT

AAGAACTTTCTCGTTTCAGCTTATTTGATGATAAAAAACACCCTTGTTTCTACT------

-----------------------------------

>A_turkey_Rostov-on-Don_332-09_2021_EPI1848752

----AGCAAAAGCAGGGTGACAAAAACATAATGGATCCCAACACTATGTTAAGCTTTCAG

GTAGACTGTTTTCTTTGGTATGTCCGCAAACGATTCGCAGACCAAGAACTGGGTGATGCC

CCTTTCCTTGACCGGCTTCGCCGAGATCAGAAGTCTTTAAGAGGAAGAGGCACCACTCTT

GGTCTGAGCATCGAAGCAGCTACTCGTGAGGGAAAGCAGATAGTGAAGCGAATTCTGAAG

GAAGAGCCTGATGAGGCACTTAAAATGACTGTTGCTTCAGGTCCGTCTTCACGCTACCTA

ACTGATATGACTCTTGAAGAGATGTCAAGGGACTGGTTCATGCTCATGCCCAAACGGAAA

GTGGCAGGTCCATTTTGCATCAAAATGGACCAGGCAATAATGGATAAAAACATCATATTG

AAAGCAAACTTCAGTGTAATTTTCAACCGGCTGGAAGCTCTAATACTACTTCGAGCTTTC

ACAGAAGAAGGAGCAATTGTGGGAGAAATCTCACCGTTACCTTCTTTTCCAGGACATACT

GATGAGGATGTCAAAAATGCAATTGGGGTCCTCATCGGAGGGCTTGAATGGAATAATAAC

ACAGTTCGGGTCTCTGAAACTCTACAGAGATTCGCTTGGAGAAACAGTGATGAGGATGGG

AGACCTTCACTCCCTCCAAAGTAGAAACGGAAAATGGCGAGAACAATTGGGTCAGAAGTT

TGAAGAAATAAGATGGCTGATTGAAGAAGTGCGACATAGACTGAAGATTACAGAAAATAG

CTTCGAACAGATAACGTTTATGCAAGCCTTACAACTATTGCTTGAAGTGGAACAAGAGAT

AAGAACTTTCTCGTTTCAGCTTATTTGATGATAAAAAACACCCTTGTTTCTACT------

-----------------------------------

>A_chicken_Krasnodar_334-03_2021_EPI1848800

----AGCAAAAGCAGGGTGACAAAAACATAATGGATCCCAACACTATGTTAAGCTTTCAG

GTAGACTGTTTTCTTTGGTATGTCCGCAAACGATTCGCAGACCAAGAACTGGGTGATGCC

CCTTTCCTTGACCGGCTTCGCCGAGATCAGAAGTCTTTAAGAGGAAGAGGCACCACTCTT

GGTCTGAGCATCGAAGCAGCTACTCGTGAGGGAAAGCAGATAGTGAAGCGAATTCTGAAG

GAAGAGTCTGATGAGGCACTTAAAATGACTGTTGCTTCAGGTCCGTCTTCACGCTACCTA

ACTGATATGACTCTTGAAGAAATGTCAAGGGACTGGTTCATGTTCATGCCCAAACGGAAA

GTGGCAGGTCCACTTTGCATCAAAATGGACCAGGCAATAATGGATAAAAACATCATATTG

AAAGCAAACTTCAGTGTAATTTTCAACCGACTGGAAGCTCTAATACTACTTCGAGCTTTC

ACAGAAGAAGGAGCAATTGTGGGAGAAATCTCACCGTTACCTTCTTTTCCAGGACATACT

GATGAGGATGTCAAAAATGCAATTGGGGTCCTCATCGGAGGGCTTGAATGGAATAATAAC

ACAGTTCGGGTCTCTGAAACTCTACAGAGATTCGCTTGGAGAAACAGTGATGAGGATGGG

AGACCTTCATTCCCTCCAAAGTAGAAACGGAAAATGGCGAGAACAATTGGGTCAGAAGTT

TGAAGAAATAAGATGGCTGATTGAAGAAATGCGACATAGACTGAAGATTACAGAAAATAG

CTTCGAACAGATAACGTTTATGCAAGCCTTACAACTATTGCTTGAAGTGGAACAAGAGAT

AAGAACTTTCTCGTTTCAGCTTATTTGATGATAAAAAACACCCTTGTTTCTACT------

-----------------------------------

>A_pheasant_Wales_000252_2021_EPI1848880

----AGCAAAAGCAGGGTGACAAAAACATAATGGATCCCAACACTATGTTAAGCTTTCAG

GTAGACTGTTTTCTTTGGTATGTCCGCAAACGATTCGCAGACCAAGAACTGGGTGATGCC

CCTTTCCTTGACCGGCTTCGCCGAGATCAGAAGTCTTTAAGAGGAAGAGGCACCACTCTT

GGTCTGAGCATCGAAGCAGCTACTCGTGAGGGAAAGCAGATAGTGAAGCGAATTCTGAAG

GAAGAGTCTGATGAGGCACTTAAAATGACTGTTGCTTCAGGTCCGTCTTCACGCTACCTA

ACTGATATGACTCTTGAAGAAATGTCAAGGGACTGGTTCATGCTCATGCCCAAACGGAAA

GTGGCAGGTCCACTTTGCATCAAAATGGACCAGGCAATAATGGATAAAAACATCATATTG

AAAGCAAACTTCAGTGTAATTTTCAACCGGCTGGAAGCTCTAATACTACTTCGAGCTTTC

ACAGAAGAAGGAGCAATTGTGGGAGAAATCTCACCGTTACCTTCTTTTCCAGGACATACT

GATGAGGATGTCAAAAATGCAATTGGGGTCCTCATCGGAGGGCTTGAATGGAATAATAAC

ACAGTTCGGGTCTCTGAAACTCTACAGAGATTCGCTTGGAGAAACAGTGATGAGGATGGG

AGACCTTCACTCCCTCCAAAGTAGAAACGAAAAATGGCGAGAACAATTGGGTCAGAAGTT

TGAAGAAATAAGATGGCTGATTGAAGAAATGCGACATAGACTGAAGATTACAGAAAATAG

CTTCGAACAGATAACGTTTATGCAAGCCTTACAACTATTGCTTGAAGTGGAGCAAGAGAT

AAGAACTTTCTCGTTTCAGCTTATTTGATGATAAAAAACACCCTTGTTTCTACT------

-----------------------------------

>A_mute_swan_Czech_Republic_1656-1_2021_EPI1850132

------------------------AACATAATGGATCCCAACACTATGTTAAGCTTTCAG

GTAGACTGTTTTCTTTGGTATGTCCGCAAACGATTCGCAGACCAAGAACTGGGTGATGCC

CCTTTCCTTGACCGGCTTCGCCGAGATCAGAAGTCTTTAAGAGGAAGAGGCACCACTCTT

GGTCTGAGCATCGAAGCAGCTACTCGTGAGGGAAAGCAGATAGTGAAGCGAATTCTGAAG

GAAGAGTCTGATGAGGCACTTAAAATGACTGTTGCTTCAGGTCCGTCTTCACGCTACCTA

ACTGATATGACTCTTGAAGAAATGTCAAGGGACTGGTTCATGCTCATGCCCAAACGGAAA

GTGGCAGGTCCACTTTGCATCAAAATGGACCAGGCAATAATGGATAAAAACATCATATTG

AAAGCAAACTTCAGTGTAATTTTCAACCGGCTGGAAGCTCTAATACTACTTCGAGCTTTC

ACAGAAGAAGGAGCAATTGTGGGAGAAATCTCACCGTTACCTTCTTTTCCAGGACATACT

GATGAGGATGTCAAAAATGCAATTGGGGTCCTCATCGGAGGGCTTGAATGGAATAATAAC

ACAGTTCGGGTCTCTGAAACTCTACAGAGATTCGCTTGGAGAAACAGTGATGAGGATGGG

AGACCTTCACTCCCTCCAAAGTAGAAACGAAAAATGGCGAGAACAATTGGGTCAGAAGTT

TGAAGAAATAAGATGGCTGATTGAAGAAGTGCGACATAGACTGAAGATTACAGAAAATAG

CTTCGAACAGATAACGTTTATGCAAGCCTTACAACTATTGCTTGAAGTGGAACAAGAGAT

AAGAACTTTCTCGTTTCAGCTTATTTGATGATAA--------------------------

-----------------------------------

>A_mute_swan_Croatia_14_2021_EPI1850966

----------------GTGACAAAAACATAATGGATCCCAACACTATGTTAAGCTTTCAG

GTAGACTGTTTTCTTTGGTATGTCCGCAAACGATTCGCAGACCAAGAACTGGGTGATGCC

CCTTTCCTTGACCGGCTTCGCCGAGATCAGAGGTCTTTAAGAGGAAGAGGCACCACTCTT

GGTCTGAGCATCGAAGCAGCTACTCGTGAGGGAAAGCAGATAGTGAAGCGAATTCTGAAG

GAAGAGTCTGATGAGGCACTTAAAATGACTGTTGCTTCAGGTCCGTCTTCACGCTACTTA

ACTGATATGACTCTTGAAGAAATGTCAAGGGACTGGTTCATGCTCATGCCCAAACGGAAA

GTGGCAGGTCCACTTTGCATCAAAATGGACCAGGCAATAATGGATAAAAACATCATATTG

AAAGCAAACTTCAGTGTAATTTTCAACCGGCTGGAAGCTCTAATACTACTTCGAGCTTTC

ACAGAAGAAGGAGCAATTGTGGGAGAAATCTCACCGTTACCTTCTTTTCCAGGACATACT

GATGAGGATGTCAAAAATGCAATTGGGGTCCTCATCGGAGGGCTTGAATGGAATAATAAC

ACAGTTCGGGTCTCTGAAACTCTACAGAGATTCGCTTGGAGAAACAGTGATGAGGATGGG

AGACCTTCATTCCCTCCAAAGTAGAAACGGAAAATGGCGAGAACAATTGGGTCAGAAGTT

TGAAGAAATAAGATGGCTGATTGAAGAAATGCGACATAGACTGAAGATTACAGAAAATAG

CTTCGAACAGATAACGTTTATGCAAGCCTTACAACTACTGCTTGAAGTGGAACAAGAGAT

AAGAACTTTCTCGTTTCAGCTTATTTGATGATAAAAAACAC-------------------

-----------------------------------

>A_chicken_Vietnam_Raho4-Cd-20-421_2020_EPI1853933

----------------GTGACAAAAACATAATGGATTCCAACACTGTGTCAAGTTTTCAG

GTAGACTGCTTTCTTTGGCATGTCCGCAAACGATTTGCAGACCAAGAACTGGGTGATGCC

CCATTCCTTGACCGGCTTCGCCGAGACCAGAAGTCCCTAAGAGGAAGAGGCAGCACCCTT

GGTCTGGACATCGAAACAGCTACTCGTGCGGGGAAACAAATAGTGGAGCGGATTCTTAAT

GAGGAACCCGATGAGGCACTTAAAGCA---------------TCGACTTCACGTCACCTA

ACTGAAATGACTCTCGAAGAAATGTCGAGAGACTGGTTCATGCTCATGCCCAAGCAGAAA

GTGGTGGGTTCCCTTTGCATCAAAATGGACCAGGCAATAATGGATAAAAGCATCATACTG

AAAGCAAATTTCAGTGTAATTTTTGACCAATTAGAGACCCTAATACTGCTCAGAGCTTTC

ACAGAAGAAGGAGCAATAGTGGGTGAAATCTCACCATTACCTTCTCTTCCAGGACATACT

AGCGAGGATGTCAAAAATGCAATTGGCGTCCTCATCGGAGGACTTGAATGGAATGATAAC

ACAGTTCGGGTCTCTGAGGTTATACAGAGATTCGCTTGGGGAAGCAGTGATGAGGGTGGG

AGACTTCCACTCCCTCCAAATCAGAAACGGAAACTGGCGAGAGCAACTAAGTCAGAAGTT

TGAAGAAATAAGGTGGCTGATTGAAGAAATACGACATAGATTGAAGATTACAGAAAACAG

CTTCGAACAGATAACTTTTATGCAAGCCTTACAACTACTGCTTGAAGTGGAGCAAGAGAT

AAGAGCCTTCTCGTTCCAGCTTATTTAATGATAAAAAACAC-------------------

-----------------------------------

>A_chicken_Czech_Republic_3531-1_2021_EPI1854237

------------------------AACATAATGGATCCCAACACTATGTTAAGCTTTCAG

GTAGACTGTTTTCTTTGGTATGTCCGCAAACGATTCGCAGACCAAGAACTGGGTGATGCC

CCTTTCCTTGACCGGCTTCGCCGAGATCAGAAGTCTTTAAGAGGAAGAGGCACCACTCTT

GGTCTGAGCATCGAAGCAGCTACTCGTGAGGGAAAGCAGATAGTGAAGCGAATTCTGAAG

GAAGAGTCTGATGAGGCACTTAAAATGACTGTTGCTTCAGGTCCGTCTTCACGCTACCTA

ACTGATATGACTCTTGAAGAAATGTCAAGGGACTGGTTCATGCTCATGCCCAAACGGAAA

GTGGCAGGTCCACTTTGCATCAAAATGGACCAGGCAATAATGGATAAAAACATCATATTG

AAAGCAAACTTCAGTGTAATTTTCAACCGGCTGGAAGCTCTAATACTACTTCGAGCTTTC

ACAGAAGAAGGAGCAATTGTGGGAGAAATCTCACCGTTACCTTCTTTTCCAGGACATACT

GATGAGGATGTCAAAAATGCAATTGGGGTCCTCATCGGAGGGCTTGAATGGAATAATAAC

ACAGTTCGGGTCTCTGAAACTCTACAGAGATTCGCTTGGAGAAACAGTGATGAGGATGGG

AGACCTTCACTCCCTCCAAAGTAGAAACGGAAAATGGCGAGAACAATTGGGTCAGAAGTT

TGAAGAAATAAGATGGCTGATTGAAGAAATGCGACATAGACTGAAGATTACAGAAAATAG

CTTCGAACAGATAACGTTTATGCAAGCCTTACAACTATTGCTTGAAGTGGAACAAGAGAT

AAGAACTTTCTCGTTTCAGCTTATTTGATGATAA--------------------------

-----------------------------------

>A_wigeon_Latvia_23903_2021_EPI1855979

-------------------ACAAAAACATAATGGATTCCAACACTATGTTAAGCTTTCAG

GTAGACTGTTTTCTTTGGTATGTCCGCAAACGATTCGCAGACCAAGAACTGGGTGATGCC

CCTTTCCTTGACCGGCTTCGCCGAGACCAGAAGTCTTTAAGAGGAAGAGGCACCACTCTT

GGTCTGAGCATCGAAGCAGCTACTCGTGAGGGAAAGCAGATAGTGAAGCGAATTCTGAAG

GAAGAGTCTGATGAGGCACTTAAAATGACTGTTGCTTCAGGTCCGTCTTCACGCTACCTA

ACTGATATGACTCTTGAAGAAATGTCAAGGGACTGGTTCATGCTCATGCCCAAACGGAAA

GTGGCAGGTCCACTTTGCATCAAAATGGACCAGGCAATAATGGATAAAAACATCATATTG

AAAGCAAACTTCAGTGTAATTTTCAACCGGCTGGAAGCTCTAATACTACTTCGAGCTTTC

ACAGAAGAAGGAGCAATTGTGGGAGAAATCTCACCGTTACCTTCTTTTCCAGGACATACT

GATGAGGATGTCAAAAATGCAATTGGGGTCCTCATCGGAGGGCTTGAATGGAATAATAAC

ACAGTTCGGGTCTCTGAAACTCTACAGAGATTCGCTTGGAGAAACAGTGATGAGGATGGG

AGACCTTCACTCCCTCCAAAGTAGAAACGAAAAATGGCGAGAACAATTGGGTCAGAAGTT

TGAAGAAATAAGATGGCTGATTGAAGAAGTGCGACATAGACTGAAGATTACAGAAAATAG

CTTCGAACAGATAACGTTTATGCAAGCCTTACAACTATTGCTTGAAGTGGAACAAGAGAT

AAGAACTTTCTCGTTTCAGCTTATTTGATGATAA--------------------------

-----------------------------------

>A_chicken_Czech_Republic_4980_2021_EPI1858494

------------------------AACATAATGGATCCCAACACTATGTTAAGCTTTCAG

GTAGACTGTTTTCTTTGGTATGTCCGCAAACGATTCGCAGACCAAGAACTGGGTGATGCC

CCTTTCCTTGACCGGCTTCGCCGAGATCAGAAGTCTTTAAGAGGAAGAGGCACCACTCTT

GGTCTGAGCATCGAAGCAGCTACTCGTGAGGGAAAGCAGATAGTGAAGCGAATTCTGAAG

GAAGAGTCTGATGAGGCACTTAAAATGACTGTTGCTTCAGGTCCGTCTTCACGCTACCTA

ACTGATATGACTCTTGAAGAAATGTCAAGGGACTGGTTCATGCTCATGCCCAAACGGAAA

GTGGCAGGTCCACTTTGCATCAAAATGGACCAGGCAATAATGGATAAAAACATCATATTG

AAAGCAAACTTCAGTGTAATTTTCAACCGGCTGGAAGCTCTAATACTACTTCGAGCTTTC

ACAGAAGAAGGAGCAATTGTGGGAGAAATCTCACCGTTACCTTCTTTTCCAGGACATACT

GATGAGGATGTCAAAAATGCAATTGGGGTCCTCATCGGAGGGCTTGAATGGAATAATAAC

ACAGTTCGGGTCTCTGAAACTCTACAGAGATTCGCTTGGAGAAACAGTGATGAGGATGGG

AGACCTTCATTCCCTCCAAAGTAGAAACGGAAAATGGCGAGAACAATTGGGTCAGAAGTT

TGAAGAAATAAGATGGCTGATTGAAGAAATGCGACATAGACTGAAGATTACAGAAAATAG

CTTCGAACAGATAACGTTTATGCAAGCCTTACAACTATTGCTTGAAGTGGAACAAGAGAT

AAGAACTTTCTCGTTTCAGCTTATTTGATGATAA--------------------------

-----------------------------------

>A_swan_Lithuania_1258PG1_21VIR2606-2_2021_EPI1858568

----AGCRAAAGCAGGGTGACAAAAACATAATGGATCCCAACACTATGTTAAGCTTTCAG

GTAGACTGTTTTCTTTGGTATGTCCGCAAACGATTCGCAGACCAAGAACTGGGTGATGCC

CCTTTCCTTGACCGGCTTCGCCGAGATCAGAAGTCTTTAAGAGGAAGAGGCACCACTCTT

GGTCTGAGCATCGAAGCAGCTACTCGTGAGGGAAAGCAGATAGTGAAGCGAATTCTGAAG

GAAGAGTCTGATGAGGCACTTAAAATGACTGTTGCTTCAGGTCCGTCTTCACGCTACCTA

ACTGATATGACTCTTGAAGAAATGTCAAGGGACTGGTTCATGCTCATGCCCAAACGGAAA

GTGGCAGGTCCACTTTGCATCAAAATGGACCAGGCAATAATGGATAAAAACATCATATTG

AAAGCAAACTTCAGTGTAATTTTCAACCGGCTGGAAGCTCTAATACTACTTCGAGCTTTC

ACAGAAGAAGGAGCAATTGTGGGAGAAATCTCACCGTTACCTTCTTTTCCAGGACATACT

GATGAGGATGTCAAAAATGCAATTGGGGTCCTCATCGGAGGGCTTGAATGGAATAATAAC

ACAGTTCGGGTCTCTGAAACTCTACAGAGATTCGCTTGGAGAAACAGTGATGAGGATGGG

AGACCTTCATTCCCTCCAAAGTAGAAACGGAAAATGGCGAGAACAATTGGGTCAGAAGTT

TGAAGAAATAAGATGGCTGATTGAAGAAATGCGACATAGACTGAAGATTACAGAAAATAG

CTTCGAACAGATAACGTTTATGCAAGCCTTACAACTATTGCTTGAAGTGGAACAAGAGAT

AAGAACTTTCTCGTTTCAGCTTATTTGATGATAAAAAACACCCTTGTTTCTACT------

-----------------------------------

>A_swan_Lithuania_1298PG1_21VIR2606-3_2021_EPI1858576

----AGCRAAAGCAGGGTGACAAAAACATAATGGATTCCAACACTATGTTAAGCTTTCAG

GTAGACTGTTTTCTTTGGTATGTCCGCAAACGATTCGCAGACCAAGAACTGGGTGATGCC

CCTTTCCTTGACCGGCTTCGCCGAGATCAGAAGTCTTTAAGAGGAAGAGGCACCACTCTT

GGTCTGAGCATCGAAGCAGCTACTCGTGAGGGAAAGCAGATAGTGAAGCGAATTCTGAAG

GAAGAGTCTGATGAGGCACTTAAAATGACTGTTGCTTCAGGTCCGTCTTCACGCTACCTA

ACTGATATGACTCTTGAAGAAATGTCAAGGGACTGGTTCATGCTCATGCCCAAACGGAAA

GTGACAGGTCCACTTTGCATCAAAATGGACCAGGCAATAATGGATAAAAACATCATATTG

AAAGCAAACTTCAGTGTAATTTTCAACCGGCTGGAAGCTCTAATACTACTTCGAGCTTTC

ACAGAAGAAGGAGCAATTGTGGGAGAAATCTCACCGTTACCTTCTTTTCCAGGACATACT

GATGAGGATGTCAAAAATGCAATTGGGGTCCTCATCGGAGGGCTTGAATGGAATAATAAC

ACAGTTCGGGTCTCTGAAACTCTACAGAGATTCGCTTGGAGAAACAGTGATGAGGATGGG

AGACCTTCACTCCCTCCAAAGTAGAAACGAAAAATGGCGAGAACAATTGGGTCAGAAGTT

TGAAGAAATAAGATGGCTGATTGAAGAAGTGCGACATAGACTGAAGATTACAGAAAATAG

CTTCGAACAGATAACGTTTATGCAAGCCTTACAACTATTGCTTGAAGTGGAACAAGAGAT

AAGAACTTTCTCGTTTCAGCTTATTTGATGATAAAAAACACCCTTGTTTCTACT------

-----------------------------------

>A_chicken_Bulgaria_50-1_21VIR1454-9_2021_EPI1858616

----AGCAAAAGCAGGGTGACAAAAACATAATGGATCCCAACACTATGTTAAGCTTTCAG

GTAGACTGTTTTCTTTGGTATGTCCGCAAACGATTCGCAGACCAAGAACTGGGTGATGCC

CCTTTCCTTGACCGGCTTCGCCGAGATCAGAAGTCTTTAAGAGGAAGAGGCACCACTCTT

GGTCTGAGCATCGAAGCAGCTACTCGTGAGGGAAAGCAGATAGTGAAGCGAATTCTGAAG

GAAGAGTCTGATGAGGCACTTAAAATGACTGTTGCTTCAGGTCCGTCTTCACGCTACCTA

ACTGATATGACTCTTGAAGAAATGTCAAGGGACTGGTTCATGCTCATGCCCAAACGGAAA

GTGGCAGGTCCACTTTGCATCAAAATGGACCAGGCAATAATGGATAAAAACATCATATTG

AAAGCAAACTTCAGTGTAATTTTCAACCGGCTGGAAGCTCTAATACTACTTCGAGCTTTC

ACAGAAGAAGGAGCAATTGTGGGAGAAATCTCACCGTTACCTTCTTTTCCAGGACATACT

GATGAGGATGTCAAAAATGCAATTGGGGTCCTCATCGGAGGGCTTGAATGGAATAATAAC

ACAGTTCGGGTCTCTGAAACTCTACAGAGATTCGCTTGGAGAAACAGTGATGAGGATGGG

AGACCTTCATTCCCTCCAAAGTAGAAACGGAAAATGGCGAGAACAATTGGGTCAGAAGTT

TGAAGAAATAAGATGGCTGATTGAAGAAATGCGACATAGACTGAAGATTACAGAAAATAG

CTTCGAACAGATAACGTTTATGCAAGCCTTACAACTATTGCTTGAAGTGGAACAAGAGAT

AAGAACTTTCTCGTTTCAGCTTATTTGATGATAAAAAACACCCTTGTTTCTACT------

-----------------------------------

>A_mute_swan_Poland_MB189_2021_EPI1859659

---------------GGTGACAAAAACATAATGGATTCCAACACTATGTTAAGCTTTCAG

GTAGACTGTTTTCTTTGGTATGTCCGCAAACGATTCGCAGACCAAGAACTGGGTGATGCC

CCTTTCCTTGACCGGCTTCGCCGAGATCAGAAGTCTTTAAGAGGAAGAGGCACCACTCTT

GGTCTGAGCATCGAAGCAGCTACTCGTGAGGGAAAGCAGATAGTGAAGCGAATTCTGAAG

GAAGAGTCTGATGAGGCACTTAAAATGACTGTTGCTTCAGGTCCGTCTTCACGCTACCTA

ACTGATATGACCCTTGAAGAAATGTCAAGGGACTGGTTCATGCTCATGCCCAAACGGAAA

GTGGCAGGTCCACTTTGCATCAAAATGGACCAGGCAATAATGGATAAAAACATCATATTG

AAAGCAAACTTCAGTGTAATTTTCAACCGGCTGGAAGCTCTAATACTACTTCGAGCTTTC

ACAGAAGAAGGAGCAATTGTGGGAGAAATCTCACCGTTACCTTCTTTTCCAGGACATACT

GATGAGGATGTCAAAAATGCAATTGGGGTCCTCATCGGAGGGCTTGAATGGAATAATAAC

ACAGTTCGGGTCTCTGAAACTCTACAGAGATTCGCTTGGAGAAACAGTGATGAGGATGGG

AGACCTTCACTCCCTCCAAAGTAGAAACGGAAAATGGCGAGAACAATTGGGTCAGAAGTT

TGAAGAAATAAGATGGCTGATTGAAGAAGTGCGACATAGACTGAAGATTACAGAAAATAG

CTTCGAACAGATAACGTTTATGCAAGCCTTACAACTATTGCTTGAAGTGGAACAAGAGAT

AAGAACTTTCTCGTTTCAGCTTATTTGATGATAAAAAACA--------------------

-----------------------------------

>A_mute_swan_Poland_MB272_2021_EPI1859675

---------------GGTGACAAAAACATAATGGATCCCAACACTATGTTAAGCTTTCAG

GTAGACTGTTTTCTTTGGTATGTCCGCAAACGATTCGCAGACCAAGAACTGGGTGATGCC

CCTTTCCTTGACCGGCTTCGCCGAGATCAGAAGTCTTTAAGAGGAAGAGGCACCACTCTT

GGTCTGAGCATCGAAGCAGCTACTCGTGAGGGAAAGCAGATAGTGAAGCGAATTCTGAAG

GAAGAGTCTGATGTGGCACTTAAAATGACTGTTGCTTCAGGTCCGTCTTCACGCTACCTA

ACTGATATGACTCTTGAAGAAATGTCAAGGGACTGGTTCATGCTCATGCCCAAACGGAAA

GTGGCAGGTCCACTTTGCATCAAAATGGACCAGGCAATAATGGATAAAAACATCATATTG

AAAGCAAACTTCAGTGTAATTTTCAACCGGCTGGAAGCTCTAATACTACTTCGAGCTTTC

ACAGAAGAAGGAGCAATTGTGGGAGAAATCTCACCGTTACCTTCTTTTCCAGGACATACT

GATGAGGATGTCAAAAATGCAATTGGGGTCCTCATCGGAGGGCTTGAATGGAATAATAAC

ACAGTTCGGGTCTCTGAAACTCTACAGAGATTCGCTTGGAGAAACAGTGATGAGGATGGG

AGACCTTCATTCCCTCCAAAGTAGAAATGGAAAATGGCGAGAACAATTGGGTCAGAAGTT

TGAAGAAATAAGATGGCTGATTGAAGAAATGCGACATAGACTGAAGATTACAGAAAATAG

CTTCGAACAGATAACGTTTATGCAAGCCTTACAACTATTGCTTGAAGTGGAACAAGAGAT

AAGAACTTTCTCGTTTCAGCTTATTTGATGATAAAAAACA--------------------

-----------------------------------

>A_anser_anser_Spain_297-1_21VIR1230-5_2021_EPI1860065

------------------GACAAAAACATAATGGATCCCAACACTATGTTAAGCTTTCAG

GTAGACTGTTTTCTTTGGTATGTCCGCAAACGATTCGCAGACCAAGAACTGGGTGATGCC

CCTTTCCTTGACCGGCTTCGCCGAGATCAGAAGTCTTTAAGAGGAAGAGGCACCACTCTT

GGTCTGAGCATCGAAGCAGCTACTCGTGAGGGAAAGCAGATAGTGAAGCGAATTCTGAAG

GAAGAGTCTGATGAGGCACTTAAAATGACTGTTGCTTCAGGTCCGTCTTCACGCTACCTA

ACTGATATGACTCTTGAAGAAATGTCAAAGGACTGGTTCATGCTCATGCCCAAACGGAAA

GTGGCAGGTCCACTTTGCATCAAAATGGACCAGGCAATAATGGATAAAAACATCATATTG

AAAGCAAACTTCAGTGTAACTTTCAACCGGCTGGAAGCTCTAATACTACTTCGAGCTTTC

ACAGAAGAAGGAGCAATTGTGGGAGAAATCTCACCGTTACCTTATTTTCCAGGACATACT

GATGAGGATGTCAAAAATGCAATTGGGGTCCTCATCGGAGGGCTTGAATGGAATAATAAC

ACAGTTCGGGTCTCTGAAACTCTACAGAGATTCGCTTGGAGAAACAGTGATGAGGATGGG

AGACCTTCACTCCCTCCAAAGTAGAAACGGAAAATGGCGAGAACAATTGGGTCAGAAGTT

TGAAGAAATAAGATGGCTGATTGAAGAAATACGACATAGACTGAAGATTACAGAAAATAG

CTTCGAACAGATAACGTTTATGCAAGCCTTACAACTATTGCTTGAAGTGGAGCAAGAGAT

AAGAACTTTCTCGTTTCAGCTTATTTGATGATAA--------------------------

-----------------------------------

>A_ciconia_ciconia_Spain_102-1_21VIR1230-2_2021_EPI1860073

------------------GACAAAAACATAATGGATCCCAACACTATGTTAAGCTTTCAG

GTAGACTGTTTTCTTTGGTATGTCCGCAAACGATTCGCAGACCAAGAACTGGGTGATGCC

CCTTTCCTTGACCGGCTTCGCCGAGATCAGAAGTCTTTAAGAGGAAGAGGCACCACTCTT

GGTCTGAGCATCGAAGCAGCTACTCGTGAGGGAAAGCAGATAGTGAAGCGAATTCTGAAG

GAAGAGTCTGATGAGGCACTTAAAATGACTGTTGCTTCAGGTCCGTCTTCACGCTACCTA

ACTGATATGACTCTTGAAGAAATGTCAAGGGACTGGTTCATGCTCATGCCCAAACGGAAA

GTGGCAGGTCCACTTTGCATCAAAATGGACCAGGCAATAATGGATAAAAACATCATATTG

AAAGCAAACTTCAGTGTAATTTTCAACCGGCTGGAAGCTCTAATACTACTTCGAGCTTTC

ACAGAAGAAGGAGCAATTGTGGGAGAAATCTCACCGTTACCTTCTTTTCCAGGACATACT

GATGAGGATGTCAAAAATGCAATTGGGGTCCTCATCGGAGGGCTTGAATGGAATAATAAC

ACAGTTCGGGTCTCTGAAACTCTACAGAGATTCGCTTGGAGAAACAGTGATGAGGATGGG

AGACCTTCATTCCCTCCAAAGTAGAAACGGAAAATGGCGAGAACAATTGGGTCAGAAGTT

TGAAGAAATAAGATGGCTGATTGAAGAAATGCGACATAGACTGAAGATTACAGAAAATAG

CTTCGAACAGATAACGTTTATGCAAGCCTTACAACTATTGCTTGAAGTGGAACAAGAGAT

AAGAACTTTCTCGTTTCAGCTTATTTGATGATAA--------------------------

-----------------------------------

>A_duck_Jiangsu_k1203_2010_EPI442021

----AGCAAAAGCAGGGTGACAAAAACATAATGGATTCCAACACTGTGTCAAGCTTCCAG

GTAGACTGCTTTCTTTGGCATGTCCGCGAGCGATTTGCAGACCAAGAACTGGGTGATGCC

CCATTCCTTGATCGACTTCGCCGAGATCAGAAGTCCCTAAGAGGAAGAGGCAACACTCTT

GGTCTGGACATCAAAACAGCTACTCGTGCGGGAAAGCAGATCGTGGAGCGGATTCTGGAG

GAGGAGCCTGATGAGGCACTTAAAATG---------------CCGACTTCACGCTACCTA

ACTGACATGACCCTCGAAGAGATGTCAAGGGACTGGTTCATGCTCATGCCTAAGCAGAAA

GTGGCAGGTCCCCTTTGCATTAAAATGGACCAGGCAATAATGGATAAAAACATCACATTG

AAGGCAAACTTCAGTGTGATTTTTGACCGGTTAGAAACCCTAATACTGATTAGAGCCTTC

ACAGAAGAAGGAACAATCGTGGGAGAAATCTCACCATTACCTTCTCTTCCAGGACATACT

GGTGAGGATGTCAAAAATGCAATTGGCGTCCTCATCGGAGGACTTGAATGGAATGATAAC

ACAGTTCGGATCTCTGAAACTATACAGAGATACGCTTGGAGAAGCAGTGATGAGGATGGG

AGACTTCCACTCCTTCCAAATCAGAAATGGAAAATGGCGAGAACAATTGAGCCAGAAGTT

TGAAGAAATAAGGTGGCTGATTGAAGAAGTACGACATAGATTGAAAATTACAGAAAACAG

CTTCGAACAGATAACGTTTATGCAAGCCTTACAACTACTGCTTGAAGTGGAGCAAGAGAT

AAGAGCCTTCTCGTTTCAGCTTATTTAATGATAAAAAACACCCTTGTTTCTACTA-----

-----------------------------------

>A_Turkey_Egypt_AR550_2018_EPI1420340

----------------GTGACAAAAACATAATGGATTCCAACACTATGTTAAGCTTTCAG

GTAGACTGTTTTCTTTGGTATGTCCGCAAACGATTCGCAGACCAAGAACTGGGTGATGCC

CCTTTCCTTGACCGGCTTCGCCGAGATCAGAAGTCTTTAAGAGGAAGGGGCAACACTCTT

GGTCTGGGCATCGAAACAGCTACCCGTGCGGGAAAGCAGATAGTGGAGCGAATTCTGGAG

GAAGAATCTGATGAGGCACTTAAAATGACTGTTGCTCCAAGTCCGTCTTCACGCTACCTA

ACTGACATGACTCTTGAAGAAATGTCAAGGGACTGGTTCATGCTCATGCCCAAACAGAAA

GTGGCAGGTTCACTTTGCATCAAAATGGACCAGGCAATAATGGATAAAAACATCATATTG

AAAGCAAACTTCAGTGTGAATTTTAACCGGCTGGAAGCTCTAATACTACTTCGAGCTTTC

ACAGAAGAAGGAGCAATTGTGGGAGAAATCTCACCGTTACCTTCTTTTCCAGGACATACT

GATGAGGATGTCAAAAATGCAATTGGGGTCCTCATCGGAGGACTTGAATGGAATGATAAC

ACAGTTCGGGTCTCTGAAACTCTACAGAGATTCGCTTGGAGAAACAGTAATGAGGATGGG

AGACCTTCACTCCCTCCAAAGTAGAAACGGACAATGGCGAGAACAATTGGGTCAGAAGTT

TGAAGAAATAAGATGGCTGATTGAAGAAGTGCGACATAGATTGAAGATTACAGAAAATAG

CTTCGAACAGATAACGTTTATGCAAGCCTTACAACTATTGCTTGAAGTGGAACAAGAGAT

AAGAACTTTCTCGTTTCAGCTTATTTGATGATAAAAAACAC-------------------

-----------------------------------

>A_Chicken_Egypt_AI20286_2019_EPI1636954

---------------------------------------------ATGTTAAGCTTTCAG

GTAGACTGTTTTCTTTGGCATGTCCGCAAACGATTCGCAGACCAAGAATTGGGTGATGCC

CCTTTCCTTGACCGGCTTCGCCGAGATCAGAAGTCTTTAAGAGGAAGAGGCAACACTCTT

GGTCTGGGCATCGAAACAGCTACTCGTGCGGGAAAGCAGATAGTGGAGCGAATTCTGGAG

GAAGAATCTGATGAGGCACTTAAAATGACTGTTACTTCAAGTCCGTCTTCACGCTACCTA

ACTGACATGACTCTTGAGGAAATGTCAAGGGACTGGTTCATGCTCATGCCCAAACAGAAA

GTGGCAGGTTCACTTTGCATCAAAATGGACCAGGCAATAATGGATAAAAACATCATATTG

AAAGCAAACTTCAGTGTAATTTTCAACCGGCTGGAAGCTCTAATACTACTTCGAGCTTTC

ACAGAAGAAGGAGCAATTGTGGGAGAAATCTCACCGTTACCTTCTTTTCCAGGACATACT

GATGAGGATGTCAAAAATGCAATTGGGTTCCTCATCGGAGGACTTGAATGGAATGATAAC

ACAGTTCGGGTCTCTGAAACTCTACAGAGATTCGCTTGGAGAAACAGTAATGAGGATGGG

AGACCTTCACTCCCTCCAGAACAGAAACGGAAAATGGCGAGAACAATTGGGTCAGAAGTT

TGAAGAAATAAGATGGCTGATTGAAGAAGTGCGTCATAGGTTGAAGATTACAGAAAATAG

CTTCGAACAGATAACGTTTATGCAAGCCTTACAACTATTGCTTGAAGTGGAACAAGAGAT

AAGAACTTTCTCGTTTCAGCT---------------------------------------

-----------------------------------

>A_Chicken_Egypt_AR553_2018_EPI1637086

----------------------AAAACATAATGGATTCCAACACTATGTTAAGCTTTCAG

GTAGACTGTTTTCTTTGGTATGTCCGCAAACGATTCGCAGACCAAGAACTGGGTGATGCC

CCTTTCCTTGACCGGCTTCGCCGAGATCAGAAGTCTTTAAGAGGAAGGGGCAACACTCTT

GGTCTGGGCATCGAAACAGCTACCCGTGCGGGAAAGCAGATAGTGGAGCGAATTCTGGAG

GAAGAATCTGATGAGGCACTTAAAATGACTGTTGCTCCAAGTCCGTCTTCACGCTACCTA

ACTGACATGACTCTTGAAGAAATGTCAAGGGACTGGTTCATGCTCATGCCCAAACAGAAA

GTGGCAGGTTCACTTTGCATCAAAATGGACCAGGCAATAATGGATAAAAACATCATATTG

AAAGCAAACTTCAGTGTGAATTTTAACCGGCTGGAAGCTCTAATACTACTTCGAGCTTTC

ACAGAAGAAGGAGCAATTGTGGGAGAAATCTCACCGTTACCTTCTTTTCCAGGACATACT

GATGAGGATGTCAAAAATGCAATTGGGGTCCTCATCGGAGGACTTGAATGGAATGATAAC

ACAGTTCGGGTCTCTGAAACTCTACAGAGATTCGCTTGGAGAAACAGTAATGAGGATGGG

AGACCTTCACTCCCTCCAAAGTAGAAACGGACAATGGCGAGAACAATTGGGTCAGAAGTT

TGAAGAAATAAGATGGCTGATTGAAGAAGTGCGACATAGATTGAAGATTACAGAAAATAG

CTTCGAACAGATAACGTTTATGCAAGCCTTACAACTATTGCTTGAAGTGGAACAAGAGAT

AAGAACTTTCTCGTTTCAGCTTATTTGATGATAAAAAACACCCTTGTT------------

-----------------------------------

>A_Turkey_Egypt_AI20285_2019_EPI1638801

------------------------------------------ACTATGTTAAGCTTTCAG

GTAGACTGTTTTCTTTGGTATGTCCGCAAACGATTCGCAGACCAAGAACTGGGTGATGCC

CCTTTCCTTGACCGGCTTCGCCGAGATCAAAAGTCTTTAAGAGGAAGAGGCAACACTCTT

GGTCTGGGCATCGAAACTGCTACTCGTGCGGGGAAGCAGATAGTGGAGCGAATTCTGGAG

GAAGAATCTGATGAAGCACTTAAAATGACTGTTGCTTCAAGTCCGTCTTCACGCTACCTA

ACTGACATGACTCTTGAGGAAATGTCAAGGGACTGGTTCATGCTCATGCCCAAACAGAAA

GTGGCAGGTTCACTTTGCATCAAAATGGACCAGGCAATAATGGATAAAAACATCATATTG

AAAGCAAACTTCAGTGTAAATTTTAACCGGCTGGAAGCCCTAATACTACTTCGAGCTTTC

ACAGAAGACGGAGCAATTGTGGGAGAAATTTCACCGTTACCTTCTCTTCCAGGACATACT

GATAAGGATGTCAAAAATGCAATTGGGGTCCTCATCGGAGGACTTGAATGGAATGATAAC

ACAGTTCGGGTCTCTGAAACTCTACAGAGATTCGCTTGGAGAAACAGTAATGAGGATGGG

AGACCTTCATTCCCTCCAGAGTAGAAACGGAAAATGGCGAGAACAATTGGGTCAGAAGTT

TGAAGAAATAAGATGGCTAATTGAAGAAGTGCGACATAGATTGAAGATTACAGAAAATAG

CTTCGAACAGATAACGTTTATGCAAGCCTTACAACTATTGCTTGAAGTGGAACAAGAGAT

AAGAACTTTCTCGTTTCAGC----------------------------------------

-----------------------------------

>A_goose_Omsk_0111_2020_EPI1813139

----AGCAAAAGCAGGGTGACAAAAACATAATGGATCCCAACACTATGTTAAGCTTTCAG

GTAGACTGTTTTCTTTGGTATGTCCGCAAACGATTCGCAGACCAAGAACTGGGTGATGCC

CCTTTCCTTGACCGGCTTCGCCGAGATCAGAAGTCTTTAAGAGGAAGAGGCACCACTCTT

GGTCTGAGCATCGAAGCAGCTACTCGTGAGGGAAAGCAGATAGTGAAGCGAATTCTGAAG

GAAGAGTCTGATGAGGCACTTAAAATGACTGTTGCTTCAGGTCCGTCTTCACGCTACCTA

ACTGATATGACTCTTGAAGAAATGTCAAGGGACTGGTTCATGCTCATGCCCAAACGGAAA

GTGGCAGGTCCACTTTGCATCAAAATGGACCAGGCAATAATGGATAAAAACATCATATTG

AAAGCAAACTTCAGTGTAATTTTCAACCGGCTGGAAGCTCTAATACTACTTCGAGCTTTC

ACAGAAGAAGGAGCAATTGTGGGAGAAATCTCACCGTTACCTTCTTTTCCAGGACATACT

GATGAGGATGTCAAAAATGCAATTGGGGTCCTCATCGGAGGGCTTGAATGGAATAATAAC

ACAGTTCGGGTCTCTGAAACTCTACAGAGATTCGCTTGGAGAAACAGTGATGAGGATGGG

AGACCTTCACTCCCTCCAAAGTAGAAACGGAAAATGGCGAGAACAATTGGGTCAGAAGTT

TGAAGAAATAAGATGGCTGATTGAAGAAGTGCGACATAGACTGAAGATTACAGAAAATAG

CTTCGAACAGATAACGTTTATGCAAGCCTTACAACTATTGCTTGAAGTGGAGCAAGAGAT

AAGAACTTTCTCGTTTCAGCTTATTTGATGATAAAAAACACCCTTGTTTCTACT------

-----------------------------------

>A_goose_Omsk_01161_2020_EPI1813355

----AGCAAAAGCAGGGTGACAAAAACATAATGGATCCCAACACTATGTTAAGCTTTCAG

GTAGACTGTTTTCTTTGGTATGTCCGCAAACGATTCGCAGACCAAGAACTGGGTGATGCC

CCTTTCCTTGACCGGCTTCGCCGAGATCAGAAGTCTTTAAGAGGAAGAGGCACCACTCTT

GGTCTGAGCATCGAAGCAGCTACTCGTGAGGGAAAGCAGATAGTGAAGCGAATTCTGAAG

GAAGAGTCTGATGAGGCACTTAAAATGACTGTTGCTTCAGGTCCGTCTTCACGCTACCTA

ACTGATATGACTCTTGAAGAAATGTCAAGGGACTGGTTCATGCTCATGCCCAAACGGAAA

GTGGCAGGTCCACTTTGCATCAAAATGGACCAGGCAATAATGGATAAAAACATCATATTG

AAAGCAAACTTCAGTGTAATTTTCAACCGGCTGGAAGCTCTAATACTACTTCGAGCTTTC

ACAGAAGAAGGAGCAATTGTGGGAGAAATCTCACCGTTACCTTCTTTTCCAGGACATACT

GATGAGGATGTCAAAAATGCAATTGGGGTCCTCATCGGAGGGCTTGAATGGAATAATAAC

ACAGTTCGGGTCTCTGAAACTCTACAGAGATTCGCTTGGAGAAACAGTGATGAGGATGGG

AGACCTTCACTCCCTCCAAAGTAGAAACGGAAAATGGCGAGAACAATTGGGTCAGAAGTT

TGAAGAAATAAGATGGCTGATTGAAGAAGTGCGACATAGACTGAAGATTACAGAAAATAG

CTTCGAACAGATAACGTTTATGCAAGCCTTACAACTATTGCTTGAAGTGGAACAAGAGAT

AAGAACTTTCTCGTTTCAGCTTATTTGATGATAAAAAACACCCTTGTTTCTACT------

-----------------------------------

>A_chicken_Omsk_0118_2020_EPI1813371

----AGCAAAAGCAGGGTGACAAAAACATAATGGATCCCAACACTATGTTAAGCTTTCAG

GTAGACTGTTTTCTTTGGTATGTCCGCAAACGATTCGCAGACCAAGAACTGGGTGATGCC

CCTTTCCTTGACCGGCTTCGCCGAGATCAGAAGTCTTTAAGAGGAAGAGGCACCACTCTT

GGTCTGAGCATCGAAGCAGCTACTCGTGAGGGAAAGCAGATAGTGAAGCGAATTCTGAAG

GAAGAGTCTGATGAGGCACTTAAAATGACTGTTGCTTCAGGTCCGTCTTCACGCTACCTA

ACTGATATGACTCTTGAAGAAATGTCAAGGGACTGGTTCATGCTCATGCCCAAACGGAAA

GTGGCAGGTCCACTTTGCATCAAAATGGACCAGGCAATAATGGATAAAAACATCATATTG

AAAGCAAACTTCAGTGTAATTTTCAACCGGCTGGAAGCTCTAATACTACTTCGAGCTTTC

ACAGAAGAAGGAGCAATTGTGGGAGAAATCTCACCGTTACCTTCTTTTCCAGGACATACT

GATGAGGATGTCAAAAATGCAATTGGGGTCCTCATCGGAGGGCTTGAATGGAATAATAAC

ACAGTTCGGGTCTCTGAAACTCTACAGAGATTCGCTTGGAGAAACAGTGATGAGGATGGG

AGACCTTCACTCCCTCCAAAGTAGAAACGGAAAATGGCGAGAACAATTGGGTCAGAAGTT

TGAAGAAATAAGATGGCTGATTGAAGAAGTGCGACATAGACTGAAGATTACAGAAAATAG

CTTCGAACAGATAACGTTTATGCAAGCCTTACAACTATTGCTTGAAGTGGAGCAAGAGAT

AAGAACTTTCTCGTTTCAGCTTATTTGATGATAAAAAACACCCTTGTTTCTACT------

-----------------------------------

>A_chicken_Omsk_0119_2020_EPI1813379

----AGCAAAAGCAGGGTGACAAAAACATAATGGATCCCAACACTATGTTAAGCTTTCAG

GTAGACTGTTTTCTTTGGTATGTCCGCAAACGATTCGCAGACCAAGAACTGGGTGATGCC

CCTTTCCTTGACCGGCTTCGCCGAGATCAGAAGTCTTTAAGAGGAAGAGGCACCACTCTT

GGTCTGAGCATCGAAGCAGCTACTCGTGAGGGAAAGCAGATAGTGAAGCGAATTCTGAAG

GAAGAGTCTGATGAGGCACTTAAAATGACTGTTGCTTCAGGTCCGTCTTCACGCTACCTA

ACTGATATGACTCTTGAAGAAATGTCAAGGGACTGGTTCATGCTCATGCCCAAACGGAAA

GTGGCAGGTCCACTTTGCATCAAAATGGACCAGGCAATAATGGATAAAACCATCATATTG

AAAGCAAACTTCAGTGTAATTTTCAACCGGCTGGAAGCTCTAATACTACTTCGAGCTTTC

ACAGAAGAAGGAGCAATTGTGGGAGAAATCTCACCGTTACCTTCTTTTCCAGGACATACT

GATGAGGATGTCAAAAATGCAATTGGGGTCCTCATCGGAGGGCTTGAATGGAATAATAAC

ACAGTTCGGGTCTCTGAAACTCTACAGAGATTCGCTTGGAGAAACAGTGATGAGGATGGG

AGACCTTCACTCCCTCCAAAGTAGAAACGGAAAATGGCGAGAACAATTGGGTCAGAAGTT

TGAAGAAATAAGATGGCTGATTGAAGAAGTGCGACATAGACTGAAGATTACAGAAAATAG

CTTCGAACAGATAACGTTTATGCAAGCCTTACAACTATTGCTTGAAGTGGAGCAAGAGAT

AAGAACTTTCTCGTTTCAGCTTATTTGATGATAAAAAACACCCTTGTTTCTACT------

-----------------------------------

>A_chicken_Omsk_0073_2020_EPI1813403

----AGCAAAAGCAGGGTGACAAAAACATAATGGATCCCAACACTATGTTAAGCTTTCAG

GTAGACTGTTTCCTTTGGTATGTCCGCAAACGATTCGCAGACCAAGAACTGGGTGATGCC

CCTTTCCTTGACCGGCTTCGCCGAGATCAGAAGTCTTTAAGAGGAAGAGGCACCACTCTT

GGTCTGAGCATCGAAACAGCTACTCGTGAGGGAAAGCAGATAGTGAAGCGAATTCTGAAG

GAAGAGTCTGATGAGGCACTTAAAATGACTGTTGCTTCAGGTCCGTCTTCACGCTACCTA

ACTGATATGACTCTTGAAGAAATGTCAAGGGACTGGTTCATGCTCATGCCCAAACGGAAA

GTGGCAGGTCCACTTTGCATCAAAATGGACCAGGCAATAATGGATAAAAACATCATATTG

AAAGCAAACTTCAGTGTAGTTTTCAACCGGCTGGAAGCTCTAATACTACTTCGAGCTTTC

ACAGAAGAAGGAGCAATTGTGGGAGAAATCTCACCGTTACCTTCTTTTCCAGGACATACT

GATGAGGATGTCAAAAATGCAATTGGGGTCCTCATCGGAGGGCTTGAATGGAATAATAAC

ACAGTTCGGGTCTCTGAAACTCTACAGAGATTCGCTTGGAGAAACAGTGATGAGGATGGG

AGACCTTCACTCCCTCCAAAGTAGAAACGGAAAATGGCGAGAACAATTGGGTCAGAAGTT

TGAAGAAATAAGATGGCTGATTGAAGAAGTGCGACATAGACTGAAGATTACAGAAAATAG

CTTCGAACAGATAACGTTTATGCAAGCCTTACAACTATTGCTTGAAGTGGAACAAGAGAT

AAGAACTTTCTCGTTTCAGCTTATTTGATGATAAAAAACACCCTTGTTTCTACT------

-----------------------------------

>A_chicken_Omsk_30007_2020_EPI1814307

----AGCAAAAGCAGGGTGACAAAAACATAATGGATCCCAACACTATGTTAAGCTTTCAG

GTAGACTGTTTTCTTTGGTATGTCCGCAAACGATTCGCAGACCAAGAACTGGGTGATGCC

CCTTTCCTTGACCGGCTCCGCCGAGATCAGAAGTCTTTAAGAGGAAGAGGCACCACTCTT

GGTCTGAGCATCGAAGCAGCTACTCGTGAGGGAAAGCAGATAGTGAAGCGAATTCTGAAG

GAAGAGTCTGATGAGGCACTTAAAATGACTGTTGCTTCAGGTCCGTCTTCACGCTACCTA

ACTGATATGACTCTTGAAGAAATGTCAAGGGACTGGTTCATGCTCATGCCCAAACGGAAA

GTGGCAGGTCCACTTTGCATCAAAATGGACCAGGCAATAATGGATAAAAACATCATATTG

AAAGCAAACTTCAGTGTAATTTTCAACCGGCTGGAAGCTCTAATACTACTTCGAGCTTTC

ACAGAAGAAGGAGCAATTGTGGGAGAAATCTCACCGTTACCTTCTTTTCCAGGACATACT

GATGAGGATGTCAAAAATGCAATTGGGGTCCTCATCGGAGGGCTTGAATGGAATAATAAC

ACAGTTCGGGTCTCTGAAACTCTACAGAGATTCGCTTGGAGAAACAGTGATGAGGATGGG

AGACCTTCACTCCCTCCAAAGTAGAAACGGAAAATGGAGAGAACAATTGGGTCAGAAGTT

TGAAGAAATAAGATGGCTGATTGAAGAAGTGCGACATAGACTGAAGATTACAGAAAATAG

CTTCGAACAGATAACGTTTATGCAAGCCTTACAACTATTGCTTGAAGTGGAACAAGAGAT

AAGAACTTTCTCGTTTCAGCTTATTTGATGATAAAAAACACCCTTGTTTCTACT------

-----------------------------------

>A_goose_Omsk_30009_2020_EPI1814315

----AGCAAAAGCAGGGTGACAAAAACATAATGGATCCCAACACTATGTTAAGCTTTCAG

GTAGACTGTTTTCTTTGGTATGTCCGCAAACGATTCGCAGACCAAGAACTGGGTGATGCC

CCTTTCCTTGACCGGCTTCGCCGAGATCAGAAGTCTTTAAGAGGAAGAGGCACCACTCTT

GGTCTGAGCATCGAAGCAGCTACTCGTGAGGGAAAGCAGATAGTGAAGCGAATTCTGAAG

GAAGAGTCTGATGAGGCACTTAAAATGACTGTTGCTTCAGGTCCGTCTTCACGCTACCTA

ACTGATATGACTCTTGAAGAAATGTCAAGGGACTGGTTCATGCTCATGCCCAAACGGAAA

GTGGCAGGTCCACTTTGCATCAAAATGGACCAGGCAATAATGGATAAAAACATCATATTG

AAAGCAAACTTCAGTGTAATTTTCAACCGGCTGGAAGCTCTAATACTACTTCGAGCTTTC

ACAGAAGAAGGAGCAATTGTGGGAGAAATCTCACCGTTACCTTCTTTTCCAGGACATACT

GATGAGGATGTCAAAAATGCAATTGGGGTCCTCATCGGAGGGCTTGAATGGAATAATAAC

ACAGTTCGGGTCTCTGAAACTCTACAGAGATTCGCTTGGAGAAACAGTGATGAGGATGGG

AGACCTTCACTCCCTCCAAAGTAGAAACGGAAAATGGCGAGAACAATTGGGTCAGAAGTT

TGAAGAAATAAGATGGCTGATTGAAGAAGTGCGACATAGACTGAAGATTACAGAAAATAG

CTTCGAACAGATAACGTTTATGCAAGCCTTACAACTATTGCTTGAAGTGGAACAAGAGAT

AAGAACTTTCTCGTTTCAGCTTATTTGATGATAAAAAACACCCTTGTTTCTACT------

-----------------------------------

>A_chicken_Chelyabinsk_201_2020_EPI1814331

----AGCAAAAGCAGGGTGACAAAAACATAATGGATCCCAACACTATGTTAAGCTTTCAG

GTAGACTGTTTCCTTTGGTATGTCCGCAAACGATTCGCAGACCAAGAACTGGGTGATGCC

CCTTTCCTTGACCGGCTTCGCCGAGATCAGAAGTCTTTAAGAGGAAGAGGCACCACTCTT

GGTCTGAGCATCGAAGCAGCTACTCGTGAGGGAAAGCAGATAGTGAAGCGAATTCTGAAG

GAAGAGTCTGATGAGGCACTTAAAATGACTGTTGCTTCAGGTCCGTCTTCACGCTACCTA

ACTGATATGACTCTTGAAGAAATGTCAAGGGACTGGTTCATGCTCATGCCCAAACGGAAA

GTGGCAGGTCCACTTTGCATCAAAATGGACCAGGCAATAATGGATAAAAACATCATATTG

AAAGCAAACTTCAGTGTAATTTTCAACCGGCTGGAAGCTCTAATACTACTTCGAGCTTTC

ACAGAAGAAGGAGCAATTGTGGGAGAAATCTCACCGTTACCTTCTTTTCCAGGACATACT

GATGAGGATGTCAAAAATGCAATTGGGGTCCTCATCGGAGGGCTTGAATGGAATAATAAC

ACAGTTCGGGTCTCTGAAACTCTACAGAGATTCGCTTGGAGAAACAGTGATGAGGATGGG

AGACCTTCACTCCCTCCAAAGTAGAAACGGAAAATGGCGAGAACAATTGGGTCAGAAGTT

TGAAGAAATAAGATGGCTGATTGAAGAAGTGCGACATAGACTGAAGATTACAGAAAATAG

CTTCGAACAGATAACGTTTATGCAAGCCTTACAACTATTGCTTGAAGTGGAACAAGAGAT

AAGAACTTTCTCGTTTCAGCTTATTTGATGATAAAAAACACCCTTGTTTCTACT------

-----------------------------------

>A_chicken_Kurgan_1005_2020_EPI1814363

----AGCAAAAGCAGGGTGACAAAAACATAATGGATCCCAACACTATGTTAAGCTTTCAG

GTAGACTGTTTCCTTTGGTATGTCCGCAAACGATTCGCAGACCAAGAACTGGGTGATGCC

CCTTTCCTTGACCGGCTTCGCCGAGATCAGAAGTCTTTAAGAGGAAGAGGCACCACTCTT

GGTCTGAGCATCGAAGCAGCTACTCGTGAGGGAAAGCAGATAGTGAAGCGAATTCTGAAG

GAAGAGTCTGATGAGGCACTTAAAATGACTGTTGCTTCAGGTCCGTCTTCACGCTACCTA

ACTGATATGACTCTTGAAGAAATGTCAAGGGACTGGTTCATGCTCATGCCCAAACGGAAA

GTGGCAGGTCCACTTTGCATCAAAATGGACCAGGCAATAATGGATAAAAACATCATATTG

AAAGCAAACTTCAGTGTAATTTTCAACCGGCTGGAAGCTCTAATACTACTTCGAGCTTTC

ACAGAAGAAGGAGCAATTGTGGGAGAAATCTCACCGTTACCTTCTTTTCCAGGACATACT

GATGAGGATGTCAAAAATGCAATTGGGGTCCTCATCGGAGGGCTTGAATGGAATAATAAC

ACAGTTCGGGTCTCTGAAACTCTACAGAGATTCGCTTGGAGAAACAGTGATGAGGATGGG

AGACCTTCACTCCCTCCAAAGTAGAAACGGAAAATGGCGAGAACAATTGGGTCAGAAGTT

TGAAGAAATAAGATGGCTGATTGAAGAAGTGCGACATAGACTGAAGATTACAGAAAATAG

CTTCGAACAGATAACGTTTATGCAAGCCTTACAACTATTGCTTGAAGTGGAACAAGAGAT

AAGAACTTTCTCGTTTCAGCTTATTTGATGATAAAAAACACCCTTGTTTCTACT------

-----------------------------------

>A_turkey_Poland_464_2020_EPI1841314

---------------GGTGACAAAAACATAATGGATCCCAACACTATGTTAAGCTTTCAG

GTAGACTGTTTTCTTTGGTATGTCCGCAAACGATTCGCAGACCAAGAACTGGGTGATGCC

CCTTTCCTTGACCGGCTTCGCCGAGATCAGAAGTCTTTAAGAGGAAGAGGCACCACTCTT

GGTCTGAGCATCGAAGCAGCTACTCGTGAGGGAAAGCAGATAGTGAAGCGAATTCTGAAG

GAAGAGTCTGATGAGGCACTTAAAATGACTGTTGCTTCAGGTCCGTCTTCACGCTACCTA

ACTGATATGACTCTTGAAGAAATGTCAAGGGACTGGTTCATGCTCATGCCCAAACGGAAA

GTGGCAGGTCCACTTTGCATCAAAATGGACCAGGCAATAATGGATAAAAACATCATATTG

AAAGCAAACTTCAGTGTAATTTTCAACCGGCTGGAAGCTCTAATACTACTTCGAGCTTTC

ACAGAAGAAGGAGCAATTGTGGGAGAAATCTCACCGTTACCTTCTTTTCCAGGACATACT

GATGAGGATGTCAAAAATGCAATTGGGGTCCTCATCGGAGGGCTTGAATGGAATAATAAC

ACAGTTCGGGTCTCTGAAACTCTACAGAGATTCGCTTGGAGAAACAGTGATGAGGATGGG

AGACCTTCACTCCCTCCAAAGTAGAAACGGAAAATGGCGAGAACAATTGRGTCAGAAGTT

TGAAGAAATAAGATGGCTGATTGAAGAAGTGCGACATAGACTGAAGATTACAGAAAATAG

CTTCGAACAGATAACGTTTATGCAAGCCTTACAACTATTGCTTGAAGTGGAGCAAGAGAT

AAGAACTTTCTCGTTTCAGCTTATTTGATGATAAAAAACA--------------------

-----------------------------------

>A_duck_Northern_China_ZGL_2020_EPI1844094

----AGCAAAAGCAGGGTGACAAAAACATAATGGATCCCAACACTATGTTAAGCTTTCAG

GTAGACTGTTTTCTTTGGTATGTCCGCAAACGATTCGCAGACCAAGAACTGGGTGATGCC

CCTTTCCTTGACCGGCTTCGCCGAGATCAGAAGTCTTTAAGAGGAAGAGGCACCACTCTT

GGTCTGAGCATCGAAGCAGCTACTCGTGAGGGAAAGCAGATAGTGAAGCGAATTCTGAAG

GAAGAGTCTGATGAGGCACTTAAAATGACTGTTGCTTCAGGTCCGTCTTCACGCTACCTA

ACTGATATGACTCTTGAAGAGATGTCAAGGGACTGGTTCATGCTCATGCCCAAACGGAAA

GTGGCAGGTCCACTTTGCATCAAAATGGACCAGGCAATAATGGATAAAAACATCATATTG

AAAGCAAACTTCAGTGTAATTTTCAACCGGCTGGAAGCTCTAATACTACTTCGAGCTTTC

ACAGAAGAAGGAGCAATTGTGGGAGAAATCTCACCGTTACCTTCTTTTCCAGGACATACT

GATGAGGATGTCAAAAATGCAATTGGGGTCCTCATCGGAGGGCTTGAATGGAATAATAAC

ACAGTTCGGGTCTCTGAAACTCTACAGAGATTCGCTTGGAGAAACAGTGATGAGGATGGG

AGACCTTCACTCCCTCCAAAGTAGAAACGGAAAATGGCGAGAACAATTGGGTCAGAAGTT

TGAAGAAATAAGATGGCTGATTGAAGAAGTGCGACATAGACTGAAGATTACAGAAAATAG

CTTCGAACAGATAACGTTTATGCAAGCCTTACAACTATTGCTTGAAGTGGAACAAGAGAT

AAGAACTTTCTCGTTTCAGCTTATTTGATGATAAAAAACACCCTTGTTTCTACT------

-----------------------------------

>A_duck_Southwestern_China_B1904_2020_EPI1844105

----AGCAAAAGCAGGGTGACAAAAACATAATGGATCCCAACACTATGCTAAGCTTTCAG

GTAGACTGTTTTCTTTGGTATGTACGCAAACGATTCGCAGACCAAGAACTGGGTGATGCC

CCTTTCCTTGACCGGCTTCGCCGAGATCAGAAGTCTTTAAGAGGAAGAGGCACCACTCTT

GGTCTGAGCATCGAAGCAGCTACTCGTGAGGGAAAGCAGATAGTGAAGCGAATTCTGAAG

GAAGAGTCTGATGAGGCACTTAAAATGACTGTTGCTTCAGGTCCGTCTTCACGCTACCTA

ACTGATATGACTCTTGAAGAGATGTCAAGGGACTGGTTCATGCTCATGCCCAAACGGAAA

GTGGCAGGTCCACTTTGCATCAAAATGGACCAGGCAATAATGGATAAAAACATCATATTG

AAAGCAAACTTCAGTGTAATTTTCAACCGGCTGGAAGCTCTAATACTACTTCGAGCTTTC

ACAGAAGAAGGAGCAATTGTGGGAGAAATCTCACCGTTACCTTCTTTTCCAGGACATACT

GATGAGGATGTCAAAAATGCAATTGGGGTCCTCATCGGAGGGCTTGAATGGAATAATAAC

ACAGTTCGGGTCTCTGAAACTCTACAGAGATTCGCTTGGAGAAACAGTGATGAGGATGGG

AGACCTTCACTCCCTCCAAAGTAGAAACGGAAAATGGCGAGAACAATTGGGTCAGAAGTT

TGAAGAAATAAGATGGCTGATTGAAGAAGTGCGACATAGACTGAAGATTACAGAAAATAG

CTTCGAACAGATAACGTTTATGCAAGCCTTACAACTATTGCTTGAAGTGGAACAAGAGAT

AAGAACTTTCTCGTTTCAGCTTATTTGATGATAAAAAACACCCTTGTTTCTACT------

-----------------------------------

>A_duck_Korea_H411_2020_EPI1845931

------------------------------ATGGATCCCAACACTATGTTAAGCTTTCAG

GTAGACTGTTTTCTTTGGTATGTCCGCAAACGATTCGCAGACCAAGAACTGGGTGATGCC

CCTTTCCTTGACCGGCTTCGCCGAGATCAGAAGTCTTTAAGAGGAAGAGGCACCACTCTT

GGTCTGAGCATCGAAGCAGCTACTCGTGAGGGAAAGCAGATAGTGAAGCGAATTCTGAAG

GAAGAGTCTGATGAGGCACTTAAAATGACTGTTGCTTCAGGTCCGTCTTCACGCTACCTA

ACTGATATGACTCTTGAAGAGATGTCAAGGGACTGGTTCATGCTCATGCCCAAACGGAAA

GTGGCAGGTCCACTTTGCATCAAAATGGACCAGGCAATAATGGATAAAAACATCATATTG

AAAGCAAATTTCAGTGTAATTTTCAACCGGCTGGAAGCTCTAATACTACTTCGAGCTTTC

ACAGAAGAAGGAGCAATTGTGGGAGAAATCTCACCGTTACCTTCTTTTCCAGGACATACT

GATGAGGATGTCAAAAATGCAATTGGGGTCCTCATCGGAGGGCTTGAATGGAATAATAAC

ACAGTTCGGGTCTCTGAAACTCTACAGAGATTCGCTTGGAGAAACAGTGATGAGGATGGG

AGACCTTCACTCCCTCCAAAGTAGAAACGGAAAATGGCGAGAACAATTGGGTCAGAAGTT

TGAAGAAATAAGATGGCTGATTGAAGAAGTGCGACATAGACTGAAGATTACAGAAAATAG

CTTCGAACAGATAACGTTTATGCAAGCCTTACAACTATTGCTTGAAGTGGAACAAGAGAT

AAGAACTTTCTCGTTTCAGCTTATTTGA--------------------------------

-----------------------------------

>A_duck_Korea_H431_2020_EPI1845955

------------------------------ATGGATCCCAACACTATGTTAAGCTTTCAG

GTAGACTGTTTTCTTTGGTATGTCCGCAAACGATTCGCAGACCAAGAACTGGGTGATGCC

CCTTTCCTTGACCGGCTTCGCCGAGATCAGAGGTCTTTAAGAGGAAGAGGCACCACTCTT

GGTCTGAGCATCGAAGCAGCTACTCGTGAGGGAAAGCAGATAGTGAAGCGAATTCTGAAG

GAAGAGTCTGATGAGGCACTTAAAATGACTGTTGCTTCAGGTCCGTCTTCACGCTACCTA

ACTGATATGACTCTTGAAGAGATGTCAAGGGACTGGTTCATGCTCATGCCCAAACGGAAA

GTGGCAGGTCCACTTTGCATCAAAATGGACCAGGCAATAATGGATAAAAACATCATATTG

AAAGCAAATTTCAGTGTAATTTTCAACCGGCTGGAAGCTCTAATACTACTTCGAGCTTTC

ACAGAAGAAGGAGCAATTGTGGGAGAAATCTCACCGTTACCTTCTTTTCCAGGACATACT

GATGAGGATGTCAAAAATGCAATTGGGGTCCTCATCGGAGGGCTTGAATGGAATAATAAC

ACAGTTCGGGTCTCTGAAACTCTACAGAGATTCGCTTGGAGAAACAGTGATGAGGATGGG

AGACCTTCACTCCCTCCAAAGTAGAAACGGAAAATGGCGAGAACAATTGGGTCAGAAGTT

TGAAGAAATAAGATGGCTGATTGAAGAAGTGCGACATAGACTGAAGATTACAGAAAATAG

CTTCGAACAGATAACGTTTATGCAAGCCTTACAACTATTGCTTGAAGTGGAACAAGAGAT

AAGAACTTTCTCGTTTCAGCTTATTTGA--------------------------------

-----------------------------------

>A_duck_Korea_H471_2020_EPI1846027

------------------------------ATGGATCCCAACACTATGTTAAGCTTTCAG

GTAGACTGTTTTCTTTGGTATGTCCGCAAACGATTCGCAGACCAAGAACTGGGTGATGCC

CCTTTCCTTGACCGGCTTCGCCGAGATCAGAAGTCTTTAAGAGGAAGAGGCACCACTCTT

GGTCTGAGCATCGAAGCAGCTACTCGTGAGGGAAAGCAGATAGTGAAGCGAATTCTGAAG

GAAGAGTCTGATGAGGCACTTAAAATGACTGTTGCTTCAGGTCCGTCTTCACGCTACCTA

ACTGATATGACTCTTGAAGAGATGTCAAGGGACTGGTTCATGCTCATGCCCAAACGGAAA

GTGGCAGGTCCACTTTGCATCAAAATGGACCAGGCAATAATGGATAAAAACATCATATTG

AAAGCAAATTTCAGTGTAATTTTCAACCGGCTGGAAGCTCTAATACTACTTCGAGCTTTC

ACAGAAGAAGGAGCAATTGTGGGAGAAATCTCACCGTTACCTTCTTTTCCAGGACATACT

GATGAGGATGTCAAAAATGCAATTGGGGTCCTCATCGGAGGGCTTGAATGGAATAATAAC

ACAGTTCGGGTCTCTGAAACTCTACAGAGATTCGCTTGGAGAAACAGTGATGAGGATGGG

AGACCTTCACTCCCTCCAAAGTAGAAACGGAAAATGGCGAGAACAATTGGGTCAGAAGTT

TGAAGAAATAAGATGGCTGATTGAAGAAGTGCGACATAGACTGAAGATTACAGAAAATAG

CTTCGAACAGATAACGTTTATGCAAGCCTTACAACTATTGCTTGAAGTGGAACAAGAGAT

AAGAACTTTCTCGTTTCAGCTTATTTGA--------------------------------

-----------------------------------

>A_chicken_Korea_H510_2020_EPI1846051

------------------------------ATGGATCCCAACACTATGTTAAGCTTTCAG

GTAGACTGTTTTCTTTGGTATGTCCGCAAACGATTCGCAGACCAAGAACTGGGTGATGCC

CCTTTCCTTGACCGGCTTCGCCGAGATCAGAAGTCTTTAAGAGGAAGAGGCACCACTCTT

GGTCTGAGCATCGAAGCAGCTACTCGTGAGGGAAAGCAGATAGTGAAGCGAATTCTGAAG

GAAGAGTCTGATGAGGCACTTAAAATGACTGTTGCTTCAGGTCCGTCTTCACGCTACCTA

ACTGATATGACTCTTGAAGAGATGTCAAGGGACTGGTTCATGCTCATGCCCAAACGGAAA

GTGGCAGGTCCACTTTGCATCAAAATGGACCAGGCAATAATGGATAAAAACATCATATTG

AAAGCAAATTTCAGTGTAATTTTCAACCGGCTGGAAGCTCTAATACTACTTCGAGCTTTC

ACAGAAGAAGGAGCAATTGTGGGAGAAATCTCACCGTTACCTTCTTTTCCAGGACATACT

GATGAGGATGTCAAAAATGCAATTGGGGTCCTCATCGGAGGGCTTGAATGGAATAATAAC

ACAGTTCGGGTCTCTGAAACTCTACAGAGATTCGCTTGGAGAAACAGTGATGAGGATGGG

AGACCTTCACTCCCTCCAAAGTAGAAACGGAAAATGGCGAGAACAATTGGGTCAGAAGTT

TGAAGAAATAAGATGGCTGATTGAAGAAGTGCGACATAGACTGAAGATTACAGAAAATAG

CTTCGAACAGATAACGTTTATGCAAGCCTTACAACTATTGCTTGAAGTGGAACAAGAGAT

AAGAACTTTCTCGTTTCAGCTTATTTGA--------------------------------

-----------------------------------

>A_duck_Korea_H538_2020_EPI1846147

------------------------------ATGGATCCCAACACTATGTTAAGCTTTCAG

GTAGACTGTTTTCTTTGGTATGTCCGCAAACGATTCGCAGACCAAGAACTGGGTGATGCC

CCTTTCCTTGACCGGCTTCGCCGAGATCAGAAGTCTTTAAGAGGAAGAGGCACCACTCTT

GGTCTGAGCATCGAAGCAGCTACTCGTGAGGGAAAGCAGATAGTGAAGCGAATTCTGAAG

GAAGAGTCTGATGAGGCACTTAAAATGACTGTTGCTTCAGGTCCGTCTTCACGCTACCTA

ACTGATATGACTCTTGAAGAGATGTCAAGGGACTGGTTCATGCTCATGCCCAAACGGAAA

GTGGCAGGTCCACTTTGCGTCAAAATGGACCAGGCAATAATGGATAAAAACATCATATTG

AAAGCAAATTTCAGTGTAATTTTCAACCGGCTGGAAGCTCTAATACTACTTCGAGCTTTC

ACAGAAGAAGGAGCAATTGTGGGAGAAATCTCACCGTTACCTTCTTTTCCAGGACATACT

GATGAGGATGTCAAAAATGCAATTGGGGTCCTCATCGGAGGGCTTGAATGGAATAATAAC

ACAGTTCGGGTCTCTGAAACTCTACAGAGATTCGCTTGGAGAAACAGTGATGAGGATGGG

AGACCTTCACTCCCTCCAAAGTAGAAACGGAAAATGGCGAGAACAATTGGGTCAGAAGTT

TGAAGAAATAAGATGGCTGATTGAAGAAGTGCGACATAGACTGAAGATTACAGAAAATAG

CTTCGAACAGATAACGTTTATGCAAGCCTTACAACTATTGCTTGAAGTGGAACAAGAGAT

AAGAACTTTCTCGTTTCAGCTTATTTGA--------------------------------

-----------------------------------

>A_chicken_Tyumen_302-01_2020_EPI1848600

----AGCAAAAGCAGGGTGACAAAAACATAATGGATCCCAACACTATGTTAAGCTTTCAG

GTAGACTGTTTCCTTTGGTATGTCCGCAAACGATTCGCAGACCAAGAACTGGGTGATGCC

CCTTTCCTTGACCGGCTTCGCCGAGATCAGAAGTCTTTAAGAGGAAGAGGCACCACTCTT

GGTCTGAGCATCGAAGCAGCTACTCGCGAGGGAAAGCAGATAGTGAAGCGAATTCTGAAG

GAAGAGTCTGATGAGGCACTTAAAATGACTGTTGCTTCAGGTCCGTCTTCACGCTACCTA

ACTGATATGACTCTTGAAGAAATGTCAAGGGACTGGTTCATGCTCATGCCCAAACGGAAA

GTGGCAGGTCCACTTTGCATCAAAATGGACCAGGCAATAATGGATAAAAACATCATATTG

AAAGCAAACTTCAGTGTAATTTTCAACCGGCTGGAAGCTCTAATACTACTTCGAGCTTTC

ACAGAAGAAGGAGCAATTGTGGGAGAAATCTCACCGTTACCTTCTTTTCCAGGACATACT

GGTGAGGATGTCAAAAATGCAATTGGGGTCCTCATCGGAGGGCTTGAATGGAATAATAAC

ACAGTTCGGGTCTCTGAAACTCTACAGAGATTCGCTTGGAGAAACAGTGATGAGGATGGG

AGACCTTCACTCCCTCCAAAGTAGAAACGGAAAATGGCGAGAACAATTGGGTCAGAAGTT

TGAAGAAATAAGATGGCTGATTGAAGAAGTGCGACATAGACTGAAGATTACAGAAAATAG

CTTCGAACAGATAACGTTTATGCAAGCCTTACAACTATTGCTTGAAGTGGAACAAGAGAT

AAGAACTTTCTCGTTTCAGCTTATTTGATGATAAAAAACACCCTTGTTTCTACT------

-----------------------------------

>A_chicken_Tyumen_302-02_2020_EPI1848608

----AGCAAAAGCAGGGTGACAAAAACATAATGGATCCCAACACTATGTTAAGCTTTCAG

GTAGACTGTTTCCTTTGGTATGTCCGCAAACGATTCGCAGACCAAGAACTGGGTGATGCC

CCTTTCCTTGACCGGCTTCGCCGAGATCAGAAGTCTTTAAGAGGAAGAGGCACCACTCTT

GGTCTGAGCATCGAAGCAGCTACTCGCGAGGGAAAGCAGATAGTGAAGCGAATTCTGAAG

GAAGAGTCTGATGAGGCACTTAAAATGACTGTTGCTTCAGGTCCGTCTTCACGCTACCTA

ACTGATATGACTCTTGAAGAAATGTCAAGGGACTGGTTCATGCTCATGCCCAAACGGAAA

GTGGCAGGTCCACTTTGCATCAAAATGGACCAGGCAATAATGGATAAAAACATCATATTG

AAAGCAAACTTCAGTGTAATTTTCAACCGGCTGGAAGCTCTAATACTACTTCGAGCTTTC

ACAGAAGAAGGAGCAATTGTGGGAGAAATCTCACCGTTACCTTCTTTTCCAGGACATACT

GGTGAGGATGTCAAAAATGCAATTGGGGTCCTCATCGGAGGGCTTGAATGGAATAATAAC

ACAGTTCGGGTCTCTGAAACTCTACAGAGATTCGCTTGGAGAAACAGTGATGAGGATGGG

AGACCTTCACTCCCTCCAAAGTAGAAACGGAAAATGGCGAGAACAATTGGGTCAGAAGTT

TGAAGAAATAAGATGGCTGATTGAAGAAGTGCGACATAGACTGAAGATTACAGAAAATAG

CTTCGAACAGATAACGTTTATGCAAGCCTTACAACTATTGCTTGAAGTGGAACAAGAGAT

AAGAACTTTCTCGTTTCAGCTTATTTGATGATAAAAAACACCCTTGTTTCTACT------

-----------------------------------

>A_chicken_Poland_474_2020_EPI1850195

---------------GGTGACAAAAACATAATGGATTCCAACACTATGTTAAGCTTTCAG

GTAGACTGTTTTCTTTGGTATGTCCGCAAACGATTCGCAGACCAAGAACTGGGTGATGCC

CCTTTCCTTGACCGGCTTCGCCGAGATCAGAAGTCTTTAAGAGGAAGAGGCACCACTCTT

GGTCTGAGCATCGAAGCAGCTACTCGTGAGGGAAAGCAGATAGTGAAGCGAATTCTGAAG

GAAGAGTCTGATGAGGCACTTAAAATGACTGTTGCTTCAGGTCCGTCTTCACGCTACCTA

ACTGATATGACTCTTGAAGAAATGTCAAGGGACTGGTTCATGCTCATGCCCAAACGGAAA

GTGGCAGGYCCACTTTGCATCAAAATGGACCAGGCAATAATGGATAAAAACATCATATTG

AAAGCAAACTTCAGTGTAATTTTCAACCGGCTGGAAGCTCTAATACTACTTCGAGCTTTC

ACAGAAGAAGGAGCAATTGTGGGAGAAATCTCACCGTTACCTTCTTTTCCAGGACATACT

GATGAGGATGTCAAAAATGCAATTGGGGTCCTCATCGGAGGGCTTGAATGGAATAATAAC

ACAGTTCGGGTCTCTGAAACTCTACAGAGATTCGCTTGGAGAAACAGTGATGAGGATGGG

AGACCTTCACTCCCTCCAAAGTAGAAACGAAAAATGGCGAGAACAATTGGGTCAGAAGTT

TGAAGAAATAAGATGGCTGATTGAAGAAGTGCGACATAGACTGAAGATTACAGAAAATAG

CTTCGAACAGATAACGTTTATGCAAGCCTTACAACTATTGCTTGAAGTGGAACAAGAGAT

AAGAACTTTCTCGTTTCAGCTTATTTGATGATAAAAAACA--------------------

-----------------------------------

>A_swan_Poland_MB141_2020_EPI1850214

--------------TGGTGACAAAAACATAATGGATTCCAACACTATGTTAAGCTTTCAG

GTAGACTGTTTTCTTTGGTATGTCCGCAAACGATTCGCAGACCAAGAACTGGGTGATGCC

CCTTTCCTTGACCGGCTTCGCCGAGATCAGAAGTCTTTAAGAGGAAGAGGCACCACTCTT

GGTCTGAGCATCGAAGCAGCTACTCGTGAGGGAAAGCAGATAGTGAAGCGAATTCTGAAG

GAAGAGTCTGATGAGGCACTTAAAATGACTGTTGCTTCAGGTCCGTCTTCACGCTACCTA

ACTGATATGACTCTTGAAGAAATGTCAAGGGACTGGTTCATGCTCATGCCCAAACGGAAA

GTGGCAGGTCCACTTTGCATCAAAATGGACCAGGCAATAATGGATAAAAACATCATATTG

AAAGCAAACTTCAGTGTAATTTTCAACCGGCTGGAAGCTCTAATACTACTTCGAGCTTTC

ACAGAAGAAGGAGCAATTGTGGGAGAAATCTCACCGTTACCTTCTTTTCCAGGACATACT

GATGAGGATGTCAAAAATGCAATTGGGGTCCTCATCGGAGGGCTTGAATGGAATAATAAC

ACAGTTCGGGTCTCTGAAACTCTACAGAGATTCGCTTGGAGAAACAGTGATGAGGATGGG

AGACCTTCACTCCCTCCAAAGTAGAAACGAAAAATGGCGAGAACAATTGGGTCAGAAGTT

TGAAGAAATAAGATGGCTGATTGAAGAAGTGCGACATAGACTGAAGATTACAGAAAATAG

CTTCGAACAGATAACGTTTATGCAAGCCTTACAACTATTGCTTGAAGTGGAACAAGAGAT

AAGAACTTTCTCGTTTCAGCTTATTTGATGATAAAAAACA--------------------

-----------------------------------

>A_muscovy_duck_Slovakia_Pah1_21VIR1086-1_2021_EPI1858238

------------------------------ATGGATCCCAACACTATGTTAAGCTTTCAG

GTAGACTGTTTCCTTTGGTATGTCCGCAAACGATTCGCAGACCAAGAACTGGGTGATGCC

CCTTTCCTTGACCGGCTTCGCCGAGATCAGAAGTCTTTAAGAGGAAGAGGCACCACTCTT

GGTCTGAGCATCGAAGCAGCTACTCGTGAGGGAAAGCAGATAGTGAAGCGAATTCTGAAG

GAAGAGTCTGATGAGGCACTTAAAATGACTGTTGCTTCAGGTCCGTCTTCACGCTACCTA

ACTGATATGACTCTTGAAGAAATGTCAAGGGACTGGTTCATGCTCATGCCCAAACGGAAA

GTGGCAGGTCCACTTTGCATCAAAATGGACCAGGCAATAATGGATAAAAACATCATATTG

AAAGCAAACTTCAGTGTAATTTTCAACCGGCTGGAAGCTCTAATACTACTTCGAGCTTTC

ACAGAAGAAGGAGCAATTGTGGGAGAAATCTCACCGTTACCTTCTTTTCCAGGACATACT

GATGAGGATGTCAAAAATGCAATTGGGGTCCTCATCGGAGGGCTTGAATGGAATAATAAC

ACAGTTCGGGTCTCTGAAACTCTACAGAGATTCGCTTGGAGAAACAGTGATGAGGATGGG

AGACCTTCACTCCCTCCAAAGTAGAAACGGAAAATGGCGAGAACAATTGGGTCAGAAGTT

TGAAGAAATAAGATGGCTGATTGAAGAAGTGCGACATAGACTGAAGATTACAGAAAATAG

CTTCGAACAGATAACGTTTATGCAAGCCTTACAACTATTGCTTGAAGTGGAACAAGAGAT

AAGAACTTTCTCGTTTCAGCTTATTTGA--------------------------------

-----------------------------------

>A_mute_swan_Slovenia_1639-20_21VIR959-1_2020_EPI1858294

------------------------------ATGGATCCCAACACTATGTTAAGCTTTCAG

GTAGACTGTTTTCTTTGGTATGTCCGCAAACGATTCGCAGACCAAGAACTGGGTGATGCC

CCTTTCCTTGACCGGCTTCGCCGAGATCAGAAGTCTTTAAGAGGAAGAGGCACCACTCTT

GGTCTGAGCATCGAAGCAGCTACTCGTGAGGGAAAGCAGATAGTGAAGCGAATTCTGAAG

GAAGAGTCTGATGAGGCATTTAAAATGACTGTTGCTTCAGGTCCGTCTTCACGCTACCTA

ACTGATATGACTCTTGAAGAAATGTCAAGAGACTGGTTCATGCTCATGCCCAAACGGAAA

GTGGCAGGTCCACTTTGCATCAAAATGGACCAGGCAATAATGGATAAAAACATCATATTG

AAAGCAAACTTCAGTGTAATTTTCAACCGGCTGGAAGCTCTAATACTACTTCGAGCTTTC

ACAGAAGAAGGAGCAATTGTGGGAGAAATCTCACCGTTACCTTCTTTTCCAGGACATACT

GATGAGGATGTCAAAAATGCAATTGGGGTCCTCATCGGAGGGCTTGAATGGAATAATAAC

ACAGTTCGGGTCTCTGAAACTCTACAGAGATTCGCTTGGAGAAACAGTGATGAGGATGGG

AGACCTTCACTCCCTCCAAAGTAGAAACGGAAAATGGCGAGAACAATTGGGTCAGAAGTT

TGAAGAAATAAGATGGCTGATTGAAGAAGTGCGACATAGACTGAAGATTACAGAAAATAG

CTTCGAACAGATAACGTTTATGCAAGCCTTACAACTATTGCTTGAAGTGGAGCAAGAGAT

AAGAACTTTCTCGTTTCAGCTTATTTGA--------------------------------

-----------------------------------

>A_Sichuan_26221_2014_EPI533590

------------------------------ATGGATTCCAATACTGTGTCAAGTTTTCAG

GTAGACTGCTTTCTTTGGCATGTCCGCAAACGATTTGCAGACCAAGAACTGGGTGATGCC

CCATTCCTTGACCGGCTTCGCCGAGATCAGAAGTCCCTAAGAGGAAGAGGCAACACTCTT

GGTCTGGACATCGAAACAGCTACTCGTGCGGGAAAACAAATAGTGGAGCGGATTCTTGAT

GAGGAACCTGATGAGGCACTTAAAATG---------------CCGACTTCACGCTACCTA

ACTGAAATGACTCTCGAAGAAATGTCGAGGGACTGGTTCATGCTCATGCCCAAGCAGAAA

GTGGTGGGTTCCCTTTGCATCAAAATGGACCAGGCAATAATGGATAAAATCATCATACTG

AAAGCAAACTTCAGTGTGATTTTTGACCGGTTAGAGACTCTAATACTACTCAGAGCTTTC

ACAGAAGAAGGAGCAATTGTGGGAGAAATCTCACCATTACCTTCTCTTCCAGGACATACT

GGCGAGGATGTCAAAAATGCAATTGGCGTCCTCATCGGAGGACTTGAATGGAATGATAAC

ACAGTTCGGGTCTCTGAAATTATACAGAGATTCGCTTGGAGAAGCAGTGATGAGGGTGGG

AGACTTCCACTCCCTCCAAATCAGAAACGGAAACTGGCGAGAGCAATTGAGTCAGAAGTT

TGAAGAAATAAGGTGGCTGATTGAAGAAGTACGACATAGATTGAAAATTACAGAAAACAG

CTTCGAACAGATAACGTTTATGCAAGCCTTACAACTACTGCTTGAAGTGGAGCAAGAGAT

AAGAGCCTTCTCGTTTCAGCTTATTTAA--------------------------------

-----------------------------------

>A_duck_Sichuan_NCXJ16_2014_EPI590883

------------------------------ATGGATTCCAATACTGTGTCAAGTTTTCAG

GTAGACTGCTTTCTTTGGCATGTCCGCAAACGATTTGCAGACCAAGAACTGGGTGATGCC

CCATTCCTTGACCGGCTTCGCCGAGATCAGAAGTCCCTAAGAGGAAGAGGCAACACTCTT

GGTCTGGACATCGAAACAGCTACTCGTGCGGGAAAACAAATAGTGGAGCGGATTCTTGAT

GAGGAACCTGATGAGGCACTTAAAATG---------------CCGACTTCACGCTACCTA

ACTGAAATGACTCTCGAAGAAATGTCGAGGGACTGGTTCATGCTCATGCCCAAGCAGAAA

GTGGTGGGTTCCCTTTGCATCAAAATGGACCAGGCAATAATGGATAAAATCATCATACTG

AAAGCAAACTTCAGTGTGATTTTTGACCGATTAGAGACTCTAATACTACTCAGAGCTTTC

ACAGAAGAAGGAGCAATTGTGGGAGAAATCTCACCATTACCTTCTCTTCCAGGACATACT

GGCGAGGATGTCAAAAATGCAATTGGCGTCCTCATCGGAGGACTTGAATGGAATGATAAC

ACAGTTCGGGTCTCTGAAATTATACAGAGATTCGCTTGGAGAAGCAGTGATGAGGGTGGG

AGACTTCCACTCCCTCCAAATCAGAAACGGAAACTGGCGAGAGCAATTGAGTCAGAAGTT

TGAAGAAATAAGGTGGCTGATTGAAGAAGTACGACATAGATTGAAAATTACAGAAAACAG

CTTCGAACAGATAACGTTTATGCAAGCCTTACAACTACTGCTTGAAGTGGAGCAAGAGAT

AAGAGCCTTCTCGTTTCAGCTTATTTAA--------------------------------

-----------------------------------

>A_Fujian-Sanyuan_21099_2017_x_PR8_CNIC_1369977

----TGCAAAAGCAGGGTGACAAAAACATAATGGATCCAAACACTGTGTCAAGCTTTCAG

GTAGATTGCTTTCTTTGGCATGTCCGCAAACGAGTTGCAGACCAAGAACTAGGCGATGCC

CCATTCCTTGATCGGCTTCGCCGAGATCAGAAATCCCTAAGAGGAAGGGGCAGTACTCTC

GGTCTGGACATCAAGACAGCCACACGTGCTGGAAAGCAGATAGTGGAGCGGATTCTGAAA

GAAGAATCCGATGAGGCACTTAAAATGACCATGGCCTCTGTACCTGCGTCGCGTTACCTA

ACTGACATGACTCTTGAGGAAATGTCAAGGGACTGGTCCATGCTCATACCCAAGCAGAAA

GTGGCAGGCCCTCTTTGTATCAGAATGGACCAGGCGATCATGGATAAGAACATCATACTG

AAAGCGAACTTCAGTGTGATTTTTGACCGGCTGGAGACTCTAATATTGCTAAGGGCTTTC

ACCGAAGAGGGAGCAATTGTTGGCGAAATTTCACCATTGCCTTCTCTTCCAGGACATACT

GCTGAGGATGTCAAAAATGCAGTTGGAGTCCTCATCGGAGGACTTGAATGGAATGATAAC

ACAGTTCGAGTCTCTGAAACTCTACAGAGATTCGCTTGGAGAAGCAGTAATGAGAATGGG

AGACCTCCACTCACTCCAAAACAGAAACGAGAAATGGCGGGAACAATTAGGTCAGAAGTT

TGAAGAAATAAGATGGTTGATTGAAGAAGTGAGACACAAACTGAAGATAACAGAGAATAG

TTTTGAGCAAATAACATTTATGCAAGCCTTACATCTATTGCTTGAAGTGGAGCAAGAGAT

AAGAACTTTCTCGTTTCAGCTTATTTAGTACTAAAAAACACCCTTGTTTCTACT------

-----------------------------------

>A_Perigrine_falcon_Netherlands_18003274_1327122

----------------GTGACAAAAACATAATGGATTCCAACACTATGTTAAGCTTTCAG

GTAGATTGCTTTCTTTGGTATGTCCGCAAACGATTCGCAGACCAAGAACTGGGTGATGCC

CCGTTCCTTGACCGGCTTCGCCGAGATCAGAAGTCTTTAAGAGGAAGAGGCAACACTCTT

GGTCTGGGCATCGAAACAGCTACTCGTGCGGGAAAGCAGATAGTGGAGCGAATTCTGGAG

GAAGAATCTGATGAGGCACTTAAAATCACTGTTACTTCAAGTCCGTCTTCACACTACCTA

ACTGACATGACTCTTGAAGAAATGTCAAGGGACTGGTTCATGCTCATGCCCAAAAAGAAA

GTGGCAGGTTCACTTTGCATCAAAATGGACCAGGCAATAATGGATAAAAACATCATATTG

AAAGCAAACTTCAGTGTAATTTTTAACCGGCTGGAAGCTCTAATATTACTTAGAGCTTTC

ACAGAAGACGGAGCAATTGTGGGAGAGATCTCACCGTTACCTTCTTTTCCAGGACATACT

GATGAGGATGTCAAAAATGCAATTGGGGTCCTCATCGGAGGACTTGAATGGAATGATAAC

ACAGTTCGGGTCTCTGAGACTCTACAGAGATTCGCTTGGAGAAACAGTAATGAGGGTGGG

AGACCTCCACTCCCTCCAAAGTAGAAACGGAAAATGGAGAGAACAATTGGGTCAGAAGTT

TGAAGAAATAAGATGGCTGATTGAAGAAGTGCGACATAGATTAAAGATTACAGAAAATAG

CTTCGAACAGATAACGTTTATGCAAGCCTTACAACTATTGCTTGAAGTGGAACAAGAGAT

AAGAACTTTCTCGTTTCAGCTTATTTGATGATAAAAAACAC-------------------

-----------------------------------

>A_chicken_Washington_3490-18_2015_EPI590696

------------------------------ATGGACTCCAACACTGTGTCAAGCTTTCAG

GTAGACTGCTTTCTTTGGCATGTCCGCAAACGATTTGCAGACCAAGAACTGGGTGATGCC

CCATTCCTTGACCGGCTTCGCCGAGACCAGAAGTCCCTAAGAGGAAGAGGCAGCACTCTT

GGTCTGGACATCGAGACAGCTACTCGTGCGGGAAAGCAAATAGTGGAGCGGATTCTGGGG

GAAGAATCTGATGAAGCACTTAAAATGAATATTGCTTCTGTACCGACTTCACGCTACCTA

ACTGACATGACTCTTGAAGAAATGTCAAGAGACTGGTTCATGCTCATGCCCAAGCAGAAA

GTAGCAGGTTCTCTCTGCATCAAAATGGACCAGGCAATAATGGATAAAACCATCATACTG

AAAGCAAACTTCAGTGTGATTTTTGATCGGCTGGAAACCCTAATATTACTTAGAGCTTTC

ACAGAAGAAGGAGCAATTGTGGGAGAAATCTCACCATTACCTTCTCTTCCAGGACATACT

GAAGAGGATGTCAAAATTGCAATTGGGGTCCTCATCGGAGGGCTTGAATGGAATGATAAC

ACAGTTCGAGTCTCTGAAACTCTACAGAGATTCACTTGGAGAAGCAGTAATGAGGATGGG

AGACCTTCACTCCCTTCAAAACAGAAACGGAAAATGGCGAGAACAATTGAGTCAGAAGTT

CGAGGAAATAAGATGGCTGATTGAGGAAATGCGACATAGATTGAAGGTCACAGAGAACAG

CTTCGAACAAATAACGTTTATGCAAGCTTTACAACTATTGCTTGAAGTGGAGCAAGAGAT

AAGAACCTTCTCGTTTCAGCTTATTTAA--------------------------------

-----------------------------------

>A_gyrfalcon_Washington_41088-6_2014_EPI569394

------------------------AACATAATGGACTCCAACACTGTGTCAAGCTTTCAG

GTAGACTGCTTTCTTTGGCATGTCCGCAAACGATTTGCAGACCAAGAACTGGGTGATGCC

CCATTCCTTGACCGGCTTCGCCGAGACCAGAAGTCCCTAAGAGGAAGAGGCAGCACTCTT

GGTCTGGACATCGAGACAGCTACTCGTGCGGGAAAGCAAATAGTGGAGCGGATTCTGGGG

GAAGAATCTGATGAAGCACTTAAAATGAATATTGCTTCTGTACCGACTTCACGCTACCTA

ACTGACATGACTCTTGAAGAAATGTCAAGAGACTGGTTCATGCTCATGCCCAAGCAGAAA

GTAGCAGGTTCTCTCTGCATCAAAATGGACCAGGCAATAATGGATAAAACCATCATACTG

AAAGCAAACTTCAGTGTGATTTTTGATCGGCTGGAAACCCTAATATTACTTAGAGCTTTC

ACAGAAGAAGGAGCAATTGTGGGAGAAATCTCACCATTACCTTCTCTTCCAGGACATACT

GATGAGGATGTCAAAATTGCAATTGGGGTCCTCATCGGAGGGCTTGAATGGAATGATAAC

ACAGTTCGAGTCTCTGAAACTCTACAGAGATTCACTTGGAGAAGCAGTAATGAGGATGGG

AGACCTTCACTCCCTTCAAAACAGAAACGGAAAATGGCGAGAACAATTGAGTCAGAAGTT

CGAGGAAATAAGATGGCTGATTGAGGAAATGCGACATAGATTGAAGATCACAGAGAACAG

CTTCGAACAAATAACGTTTATGCAAGCTTTACAACTATTGCTTGAAGTGGAGCAAGAGAT

AAGAACCTTCTCGTTTCAGCTTATTTAATGATAA--------------------------

-----------------------------------

>A_chicken_Hubei_ZYSJF38_2016_EPI895232

------------------------------ATGGATTCCAACACTGTGTCAAGCTTCCAG

GTAGACTGCTTTCTTTGGCATGTCCGCAAACGGTTTGCAGACCAAGAACTGGGTGATGCC

CCATTTCTAGACCGGCTTCGCCGAGACCAGAAGTCCCTGAGAGGAAGAAGCAGCACTCTT

GGTCTGGACATCAGAACAGCAACTCGTGAAGGAAAGCATATAGTGGAGCGAATTTTGGAG

GAAGAGTCAGACGAAGCATTTAAAATGACTATTGCTTCAGTGCCAGCTCCACGCTATCTA

ACTGACATGACTCTTGAAGAAATGTCAAGAGATTGGTTGATGCTCATACCCAAGCAGAAA

GTGACAGGGTCCCTTTGTATTAAAATGGACCAAGCAATAGTGGACAAAACCATCACATTG

AAAGCAAATTTCAGTGTAATTTTCAATCGATTGGAAGCCCTAATACTACTTAGAGCTTTT

ACGGATGAAGGAGCAATAGTGGGCGAAATCTCACCATTACCTTCTCTTCCAGGACATACT

GACAAGGATGTCAAAAATGCAATTGAGGTCCTCATCGGAGGATTTGAATGGAATGATAAC

ACAGTTCGAGTCTCTGAAACTCTACAGAGATTCGCTTGGAGAAGCAGCGATGAGGATGGG

AGACCTCCACTCTCTCCAAAGTAGGAACGGGAAATGGAGAGAACAATTAAGCCAGAAGTT

CGAAGAAATAAGATGGTTGATTGAAGAAGTACGACATAGGTTAAAGATTACAGAGAATAG

CTTTGAACAAATAACTTTTATGCAAGCCTTACAACTATTGCTTGAAGTGGAGCAAGAGAT

AAGAACTTTCTCGTTTCAGCTTATTTAA--------------------------------

-----------------------------------

>A_Hubei_29578_2016_x_PR8_CNIC-HB29578_1369969

-----GCAAAAGCAGGGTGACAAAAACATAATGGATCCAAACACTGTGTCAAGCTTTCAG

GTAGATTGCTTTCTTTGGCATGTCCGCAAACGAGTTGCAGACCAAGAACTAGGCGATGCC

CCATTCCTTGATCGGCTTCGCCGAGATCAGAAATCCCTAAGAGGAAGGGGCAGTACTCTC

GGTCTGGACATCAAGACAGCCACACGTGCTGGAAAGCAGATAGTGGAGCGGATTCTGAAA

GAAGAATCCGATGAGGCACTTAAAATGACCATGGCCTCTGTACCTGCGTCGCGTTACCTA

ACTGACATGACTCTTGAGGAAATGTCAAGGGACTGGTCCATGCTCATACCCAAGCAGAAA

GTGGCAGGCCCTCTTTGTATCAGAATGGACCAGGCGATCATGGATAAGAACATCATACTG

AAAGCGAACTTCAGTGTGATTTTTGACCGGCTGGAGACTCTAATATTGCTAAGGGCTTTC

ACCGAAGAGGGAGCAATTGTTGGCGAAATTTCACCATTGCCTTCTCTTCCAGGACATACT

GCTGAGGATGTCAAAAATGCAGTTGGAGTCCTCATCGGAGGACTTGAATGGAATGATAAC

ACAGTTCGAGTCTCTGAAACTCTACAGAGATTCGCTTGGAGAAGCAGTAATGAGAATGGG

AGACCTCCACTCACTCCAAAACAGAAACGAGAAATGGCGGGAACAATTAGGTCAGAAGTT

TGAAGAAATAAGATGGTTGATTGAAGAAGTGAGACACAAACTGAAGATAACAGAGAATAG

TTTTGAGCAAATAACATTTATGCAAGCCTTACATCTATTGCTTGAAGTGGAGCAAGAGAT

AAGAACTTTCTCGTTTCAGCTTATTTAGTACTAAAAAACACCCTTGTTTCTACT------

-----------------------------------

>A_duck_Hyogo_1_2016_EPI866712

------------------------------ATGGATTCCAACACTGTGTCAAGTTTTCAG

GTAGACTGCTTTCTTTGGCATGTCCGCAAACGATTTGCAGACCAAGAACTGGGTGATGCC

CCATTCCTTGACCGGCTTCGCCGAGACCAGAAGTCCCTAAGAGGAAGAGGCAACACTCTT

GGTCTGGACATCGAAACAGCTACTCGTGCGGGAAAACAAATAGTGGAGCGGATTCTTGAT

GAGGAACCTGATGAGACACTTAAAATG---------------CCAACCTCACGTTACCTA

ACTGAAATGACTCTCGAAGAAATGTCGAGGGACTGGTTCATGCTAATGCCCAAGCAGAAA

GTGGTGGGTTCCCTTTGCATCAAAATGGACCAGGCAATAATGGATAAAAGCATCATACTG

AAAGCAAATTTCAGTGTGATTTTTAACCGGTTAGAGACCCTAATACTGCTCAGAGCTTTC

ACAGAAGAAGGAGCAATCGTGGGAGAAATCTCACCATTACCTTCTCTTCCAGGACATACT

GGCGAGGATGTCAAAAATGCAATTGGCGTCCTCATCGGAGGACTTGAATGGAATGATAAC

ACAGTTCGGGTCTCTGAAGTTATACAGAGATTCGCTTGGAGAAGCAGTGATGAGGGTGGG

AGATTTCCACTCTCTCCAAATCAGAAACGGAAACTGGCGAGAGCAATTGAGTCAGAAGTT

TGAAGAAATAAGGTGGCTGATTGAAGAAATACGACATAGATTGAAAATTACAGAAAACAG

CTTCGAACAGATAACTTTTATGCAAGCCTTACAACTACTGCTTGAAGTGGAGCAAGAGAT

AAGAGCCTTCTCGTTTCAGCTTATTTAA--------------------------------

-----------------------------------

>A_tundra_swan_Niigata_5112007_2016_EPI1184384

-------------------------ACATAATGGATTCCAACACTGTGTCAAGTTTTCAG

GTAGACTGCTTTCTTTGGCATGTCCGCAAACGATTTGCAGACCAAGAACTGGGTGATGCC

CCATTCCTTGACCGGCTTCGCCGAGATCAGAAGTCCCTAAGAGGAAGAGGCAACACTCTT

GGTCTGGACATCGAAACAGCTACTCGTGCGGGAAAACAAATAGTGGAGCGGATTCTTGAT

GAGGAACCTGATGAGACACTTAAAATG---------------CCGACTTCACGTTACCTA

ACTGAAATGACTCTCGAAGAGATGTCGAGGGACTGGTTCATGCTCATGCCAAAGCAGAAA

GTGGTGGGTTCCCTTTGCATCAAAATGGACCAGGCAATAATGGATAAAACCATCATACTG

AAAGCAAACTTCAGTGTGATTTTTGACCGGTTAGAGACCCTAATACTACTCAGAGCTTTC

ACAGAAGAAGGAGCAATCGTGGGAGAAATCTCACCATTACCTTCTCTTCCAGGACATACT

GATGAGGATGTCAAAAATGCAATTGGCGTCCTCATCGGAGGACTTGAATGGAATGATAAC

ACAGTTCGGGTCTCTGAAACTATACAGAGATTCGCTTGGAGAGGCAGTGATAAGGGTGGG

AGACTTCCATTCCCTCCAAATCAGAAACGGAAAATGGCGAGAGCAATTGAGTCAGAAGTT

TGAAGAAATAAGATGGCTGATTGAAGAAGTACGACATAGATTGAAAATTACAGAAAACAG

CTTCGAACAGATAACGTTTATGCAAGCTTTACAACTACTGCTTGAAGTGGAGCAAGAGAT

AAGAGCCTTCTCGTTTCAGCTTATTTAATAAT----------------------------

-----------------------------------

>A_chicken_Vietnam_NCVD-15A55_2015_EPI895050

----------------GTGACAAAAACATAATGGATTCCAACACTGTGTCAAGTTTTCAG

GTAGACTGCTTTCTTTGGCATGTCCGCAAACGATTTGCAGACCRAGAACTGGGTGATGCC

CCATTCCTTGACCGGCTTCGCCGAGACCAGAAGTCCCTAAGAGGAAGAGGCAGCACTCTT

GGTCTGGACATCGAAACAGCTACTCGTGCGGGAAAACAAATAGTGGAGCGGATTCTTAAT

GAGGAACCTGATGAGGCACTTAAAGTG---------------CCGACTTCACGTTACCTA

ACTGAAATGACTCTCGAAGAAATGTCGAGAGACTGGTTCATGCTCATGCCCAAGCAGAAA

GTGGTGGGTTCCCTTTGCATCAAAATGGACCAGGCAATAATGGATAAAAGCATCATACTG

AAAGCAAATTTCAGTGTGATTTTTGACCGGTTAGAGACCCTAATACTGCTCAGAGCTTTC

ACAGAAGAAGGAGCAATCGTGGGAGAAATCTCACCATTACCTTCTCTTCCAGGACATACT

GGCGAGGATGTCAAAAATGCAATTGGCGTCCTCATCGGAGGACTTGAATGGAATGATAAC

ACAGTTCGGGTCTCTGAAGTTATACAGAGATTCGCTTGGGGAAGCAGTGATGAGGGTGGG

AGACTTCCACTCCCTCCAAATCAGAAACGGAAACTGGCGAGAGCAATTGAGTCAGAAGTT

TGAAGAAATAAGGTGGCTGATTGAAGAAGTACGACATAGATTGAAAATTACAGAAAACAG

CTTCGAACAGATAACTTTTATGCAAGCCTTACAACTACTGCTTGAAGTGGAGCAAGAGAT

AAGAGCCTTCTCGTTTCAGCTTATTTAATGATAAAAAACAC-------------------

-----------------------------------

>A_chicken_Vietnam_NCVD-15A59_2015_EPI895067

----------------GTGACAAAAACATAATGGATTCCAACACTGTGTCAAGTTTTCAG

GTAGACTGCTTTCTTTGGCATGTCCGCAAACGATTTGCAGACCAAGAACTGGGTGATGCC

CCATTCCTTGACCGGCTTCGCCGAGACCAGAAGTCCTTAAGAGGAAGAGGCAACACTCTT

GGTCTGGACATCGAAACAGCTACTCGTGCGGGGAAACAAATAGTGGAGCGGATTCTTGAT

GAGGAACCTGATGAGGCACTTAAAATG---------------CCGACTTCACGTTACCTA

ACTGAAATGACTCTCGAAGAAATGTCGAGGGACTGGTTCATGCTCATGCCCAAGCAGAAA

GTGATGGGTTCCCTTTGCATCAAAATGGACCAGGCAATAATGGATAAAAGCATCATACTG

AAAGCAAATTTCAGTGTGATTTTTAACCGGTTAGAGACCCTAATACTGCTCAGAGCTTTC

ACAGAAGAAGGAGCAATCGTGGGAGAAATCTCACCATTACCTTCTCTTCCAGGACATACT

GGCGAGGATGTCAAAAATGCAATTGGCGTCCTCATCGGAGGGCTTGAATGGAATGATAAC

ACAGTTCGGGTCTCTGAAGTTATACAGAGATTCGCTTGGAGAAGCAGTGATGAGGGTGGG

AGACTTCCACTCCCTCCAAATCAGAAACGGAAACTGGCGAGAGCAATTGAGTCAGAAGTT

TGAAGAAATAAGGTGGCTGATTGAAGAAGTACGACATAGATTGAAAATTACAGAAAACAG

CTTCGAACAGATAACTTTTATGCAAGCCTTACAACTACTGCTTGAAGTGGAGCAAGAGAT

AAGAGCCTTCTCGTTTCAGCTTATTTAATGATAAAAAACAC-------------------

-----------------------------------

>A_duck_Wuhan_JXYFB22_2015_EPI683103

-------------------------ACATAATGGATTCCAATACTGTGTCAAGCTTTCAG

GTAGACTGCTTTCTTTGGCATGTCCGCAAACGATTTGCAGACCAAGAACTGGGTGATGCC

CCATTCCTTGACCGGCTTCGCCGAGATCAGAAGTCCCTAAGAGGAAGAGGCAGCACTCTT

GGTCTGGACATCGAAACAGCTACTCGTGCGGGAAAACAGATAGTGGAGCGGATTCTTGAT

GAGGAACCTGATGAGGAACTTAAAATG---------------CCGACTTCACGTTACCTA

ACTGAAATGACTCTCGAAGAAATGTCGAGGGACTGGTTCATGCTCATGCCCAAGCAGAAA

GTGGTGGGTTCCCTTTGCATCAAAATGGACCAGGCAATAATGGATAAAAGCATCATACTG

AAAGCAAATTTCAGTGTGATTTTTGACCGGTTAGAGACCCTAATACTGCTCAGAGCTTTC

ACAGAAGAAGGAGCAATCGTGGGAGAAATCTCACCATTACCTTCTCTTCCAGGACATACT

GGCGAGGATGTCAAAAATGCAATTGGCGTCCTCATCGGAGGACTTGAATGGAATGATAAC

ACAGTTCGGGTCTCTGAAATTATACAGAGATTCGCTTGGGGAAGCAGTGATGAGGGTGGG

AGACTTCCACTCCCTCCAAATCAGAAATGGAAACTGGCGAGAGCAATTGAGTCAGAAGTT

TGAAGAAATAAGGTGGCTGATTGAAGAAGTACGACATAGATTGAAAATTACAGAAAACAG

CTTCGAACAGATAACTTTTATGCAAGCCTTACAACTACTGCTTGAAGTGGAGCAAGAGAT

AAGAGCCTTCTCGTTTCAGCTTATTTAATGATAAAAAACACCCTTGTTTCTAT-------

-----------------------------------

>A_goose_Hunan_116_2014_EPI958636

------------------------------ATGGATTCCAATACTGTGTCAAGTTTTCAG

GTAGACTGCTTTCTTTGGCATATCCGCAAACGATTTGCAGACCAAGAACTGGGTGATGCC

CCATTCCTTGACCGGCTTCGCCGAGACCAGAAGTCCCTAAGAGGAAGAGGCAACACTCTT

GGTCTGGACATCGAAACAGCTACTCGTGCGGGAAAACAAATAGTAGAGCGGATTCTTGAT

GAGGAACCTGATGAGGCACTTAAAATG---------------CCGACTTCACGTTACCTA

ACTGAAATGACTCTCGAAGAAATGTCGAGGGACTGGTTCATGCTCATGCCCAAGCAGAAA

GTGGTGGGTTCCCTTTGCATCAAAATGGACCAGGCAATAATGGATAAAAGCATCATACTG

AAAGCAAATTTCAGTGTGATTTTTGACCGGTTAGAGACCCTAATACTGCTCAGAGCTTTC

ACAGAAGAAGGAGCAATCGTGGGAGAAATCTCACCATTACCTTCTCTTCCAGGACATACT

GGCGAGGATGTCAAAAATGCAATTGGCGTCCTCATCGGAGGACTTGAATGGAATGATAAC

ACAGTTCAGGTCTCTGAAATTATACAGAGATTCGCTTGGAGAAGCAGTGATGAGGGTGGG

AGACTTCCACTCCCTCCAAATCAGAAACGGAAACTGGCGAGAGCAATTGAAACCGAACTT

TGAAGAAATAAGGTGGCTGATTGAAGAAGTACAACTTAAATTGAAAATTACAGAAAACAG

CTTCGAACAGATAACTTTTATGCAAGCCTTACAACTACTGCTTGAAGTGGAGCAAGAGAT

AAGAGCCTTCTCGTTTCACCTTATTTAA--------------------------------

-----------------------------------

>A_Guangdong_18SF020_2018_EPI1352807

----AGCAAAAGCAGGGTGACAAAAACATAATGGATTCCAACACTGTGTCAAGTTTTCAG

GTAGACTGCTTTCTTTGGCATGTCCGCAAACGATTTGCAGACCAAGAACTGGGTGATGCC

CCATTCCTTGACCGGCTTCGCCGAGATCAGAAGTCCCTAAGAGGAAGAGGCAACACTCTT

GGTCTGGACATCGAAACAGCTACTCGTGCGGGAAAACAAATAGTGGAGCGGATTCTTGAT

GAGGAACCTGATGAAGCACTTAAAATG---------------CCGACTTCACGTTACCTA

ACTGAAATGACTCTCGAGGAAATGTCGAGGGACTGGTTCATGCTCATGCCCAAGCAGAAA

GTGGTGGGTTCCCTTTGCATCAAAATGGACCAGGCAATAATGGATAAAAGCATCATACTG

AAAGCAAATTTCAGTGTGATTTTTGGCCGGTTAGAGACCCTAATACTGCTCAGAGCTTTC

ACAGAAGAAGGAGCAATCGTGGGAGAAATCTCACCATTACCTTCTCTTCCAGGACATACT

GGCGAGGATGTCAAAAATGCAATTGGCGTCCTCATCGGAGGACTTGAATGGAATGATAAC

ACAGTTCGGGTCTCTGAAGTTATACAGAGATTCGCTTGGAGAAGCAGTGATGAGGGTGGG

AGACTTCCACTCCCTCCAAATCAGAAATGGAAACTGGCGAGAGCAATTGAGTCAGAAGTT

TGAAGAAATAAGGTGGCTGATTGAAGAGGTACGACATAGATTGAAAATTACAGAAAACAG

CTTCGAACAGATAACTTTTATGCAAGCCTTACAACTACTGCTTGAAGTGGAGCAAGAGAT

AAGAGCCTTCTCGTTTCAGCTTATTTAATGATAAAAAACACCCTTGTTTCTACT------

-----------------------------------

>A_duck_Bangladesh_43127_2020_EPI1902992

----------------GTGACAAAGACATAATGGATTCCAACACTGTGTCAAGTTTTCAG

GTAGATTGCTTTCTTTGGCATGTCCGCAAACGATTTGCAGACCAAGAACTGGGTGATGCC

CCATTCCTTGACCGGCTTCGCCGAGATCAGAAGTCCCTAAGAGGAAGAGGCAACACTCTT

GGTCTGGACATCGAAACAGCTACTCGTGCAGGGAAACAAATAGTGGAGCGGATTCTTGAT

GAGGAACCTGATGAAGCACTTAAAATG---------------CCGACTTCACGTTACCTA

ACTGAAATGACTCTTGAAGAAATGTCGAGGGACTGGTTCATGCTCATGCCCAAGCAGAAA

GTGGTGGGTTCCCTTTGCATCAAAATGGACCAGGCAATAATGGATAAAAGCATCATACTG

AAAGCAAATTTCAGTGTGATTTTTGGCCGGTTAGAGACCCTAATACTACTCAGAGCTTTC

ACAGAAGAAGGAGCAATCGTGGGAGAAATCTCACCATTACCTTCTCTTCCAGGACATACT

GGCGAGGATGTCAAAAATGCAATTGGCGTCCTCATCGGAGGACTTGAATGGAATGATAAC

ACAGTTCGGGTCTCTGAAGTTATACAGAGATTCGCTTGGGGAAGCAGTGATGAGGGTAGG

AGACTTCCACTCCCTCCAAATCAGAAACGGAAACTGGCGAGAGCAATTGAGTCAGAAGTT

TGAAGAAATAAGGTGGCTGATTGAAGAAGTACGACATAGATTGAAAATTACAGAAAACAG

CTTCGAACAGATAACTTTTATGCAAGCCTTACAACTACTGCTTGAAGTGGAGCAAGAGAT

AAGAGCCTTCTCGTTTCAGCTTATTTAATGATAAAAAACAC-------------------

-----------------------------------
