# Supplementary figures and images for: Highly pathogenic avian influenza virus of the A/H5N8 subtype, clade 2.3.4.4b, caused outbreaks in Kazakhstan in 2020
Source: PeerJ. 2022 Mar 2;10:e13038. doi: 10.7717/peerj.13038 (PMC8898005; doi:10.7717/peerj.13038)

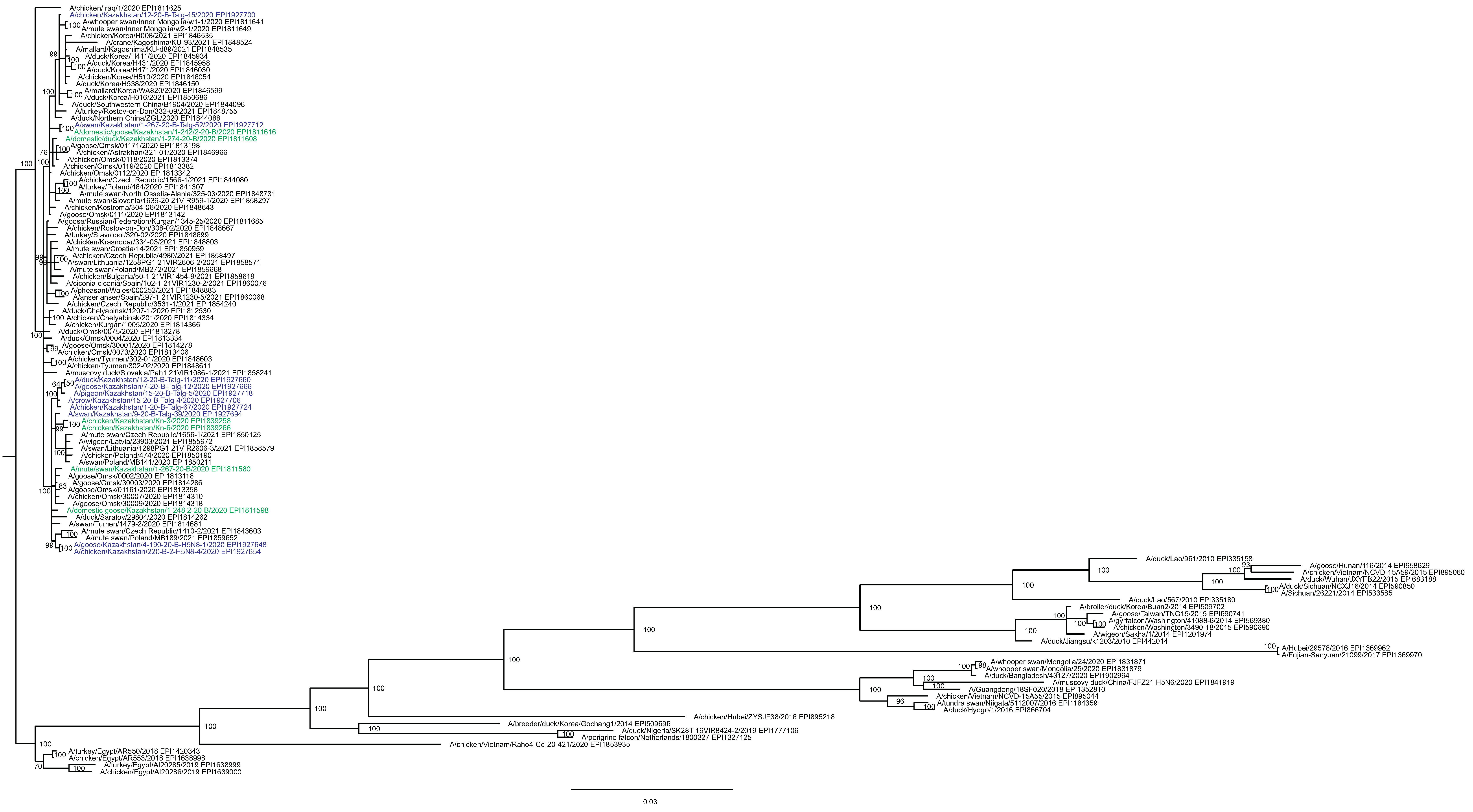

Supplement: Figure S9 [file peerj-10-13038-s010.png]

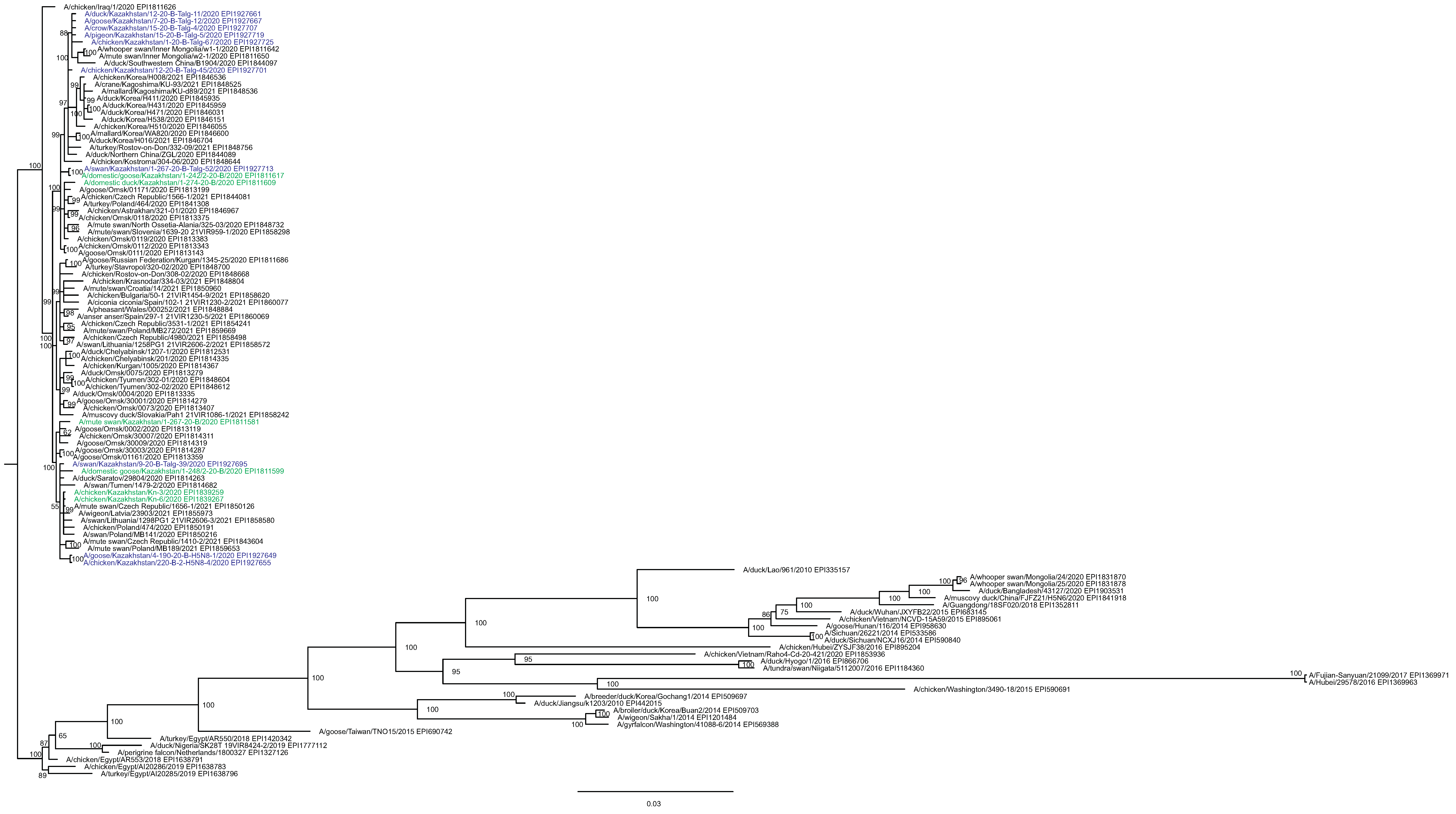

Supplement: Figure S10 [file peerj-10-13038-s011.png]

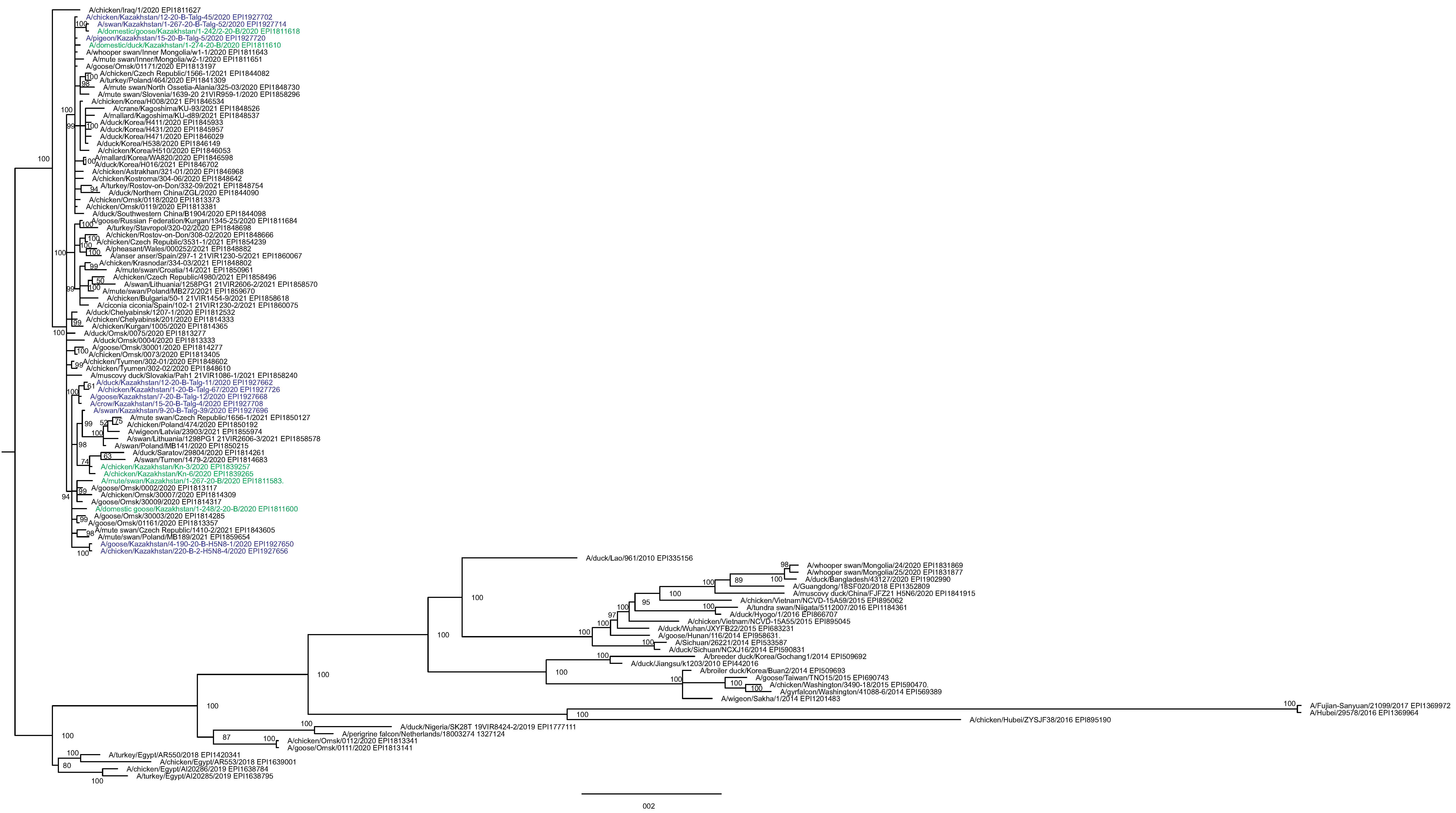

Supplement: Figure S11 [file peerj-10-13038-s012.png]

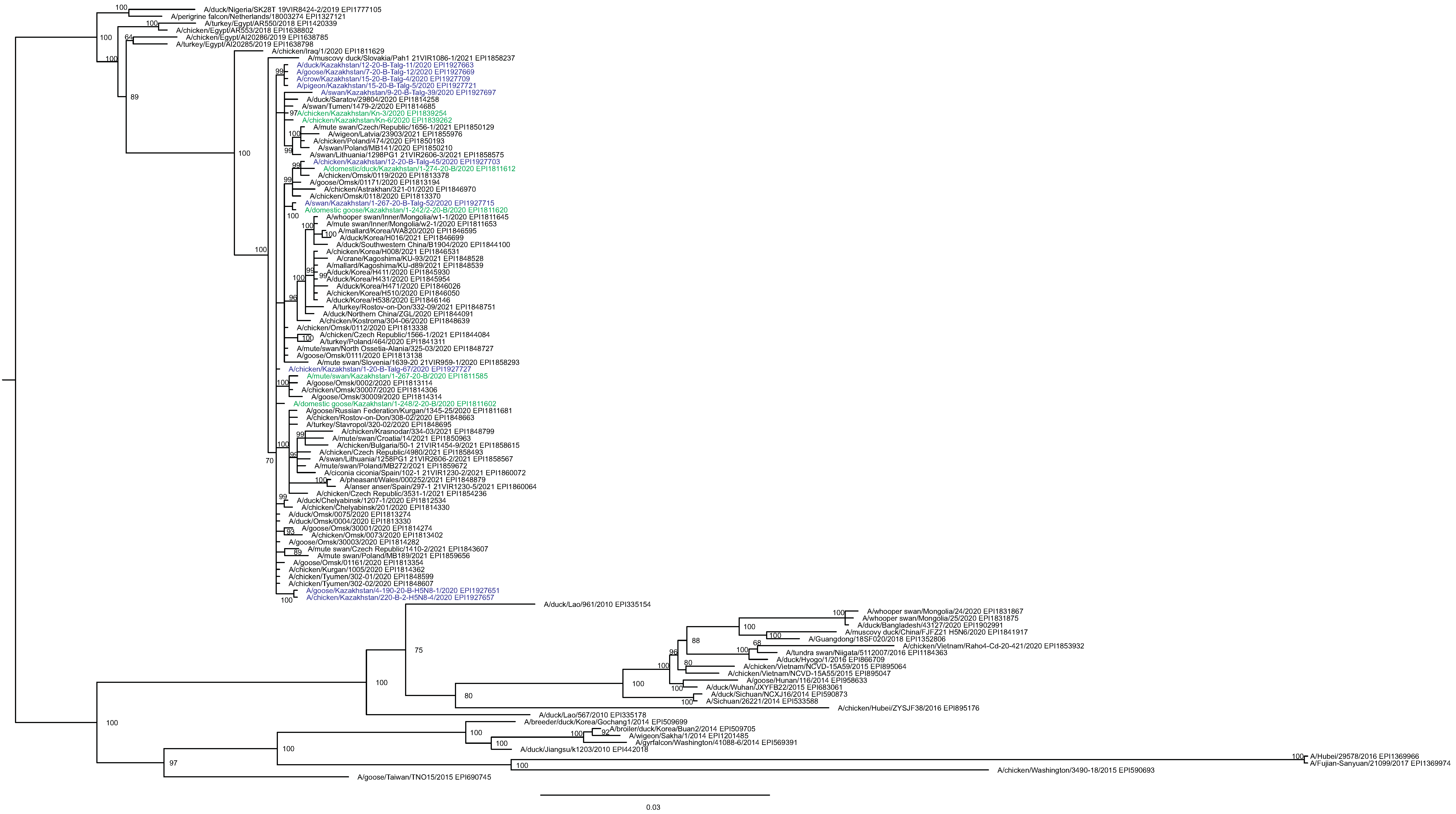

Supplement: Figure S12 [file peerj-10-13038-s013.png]

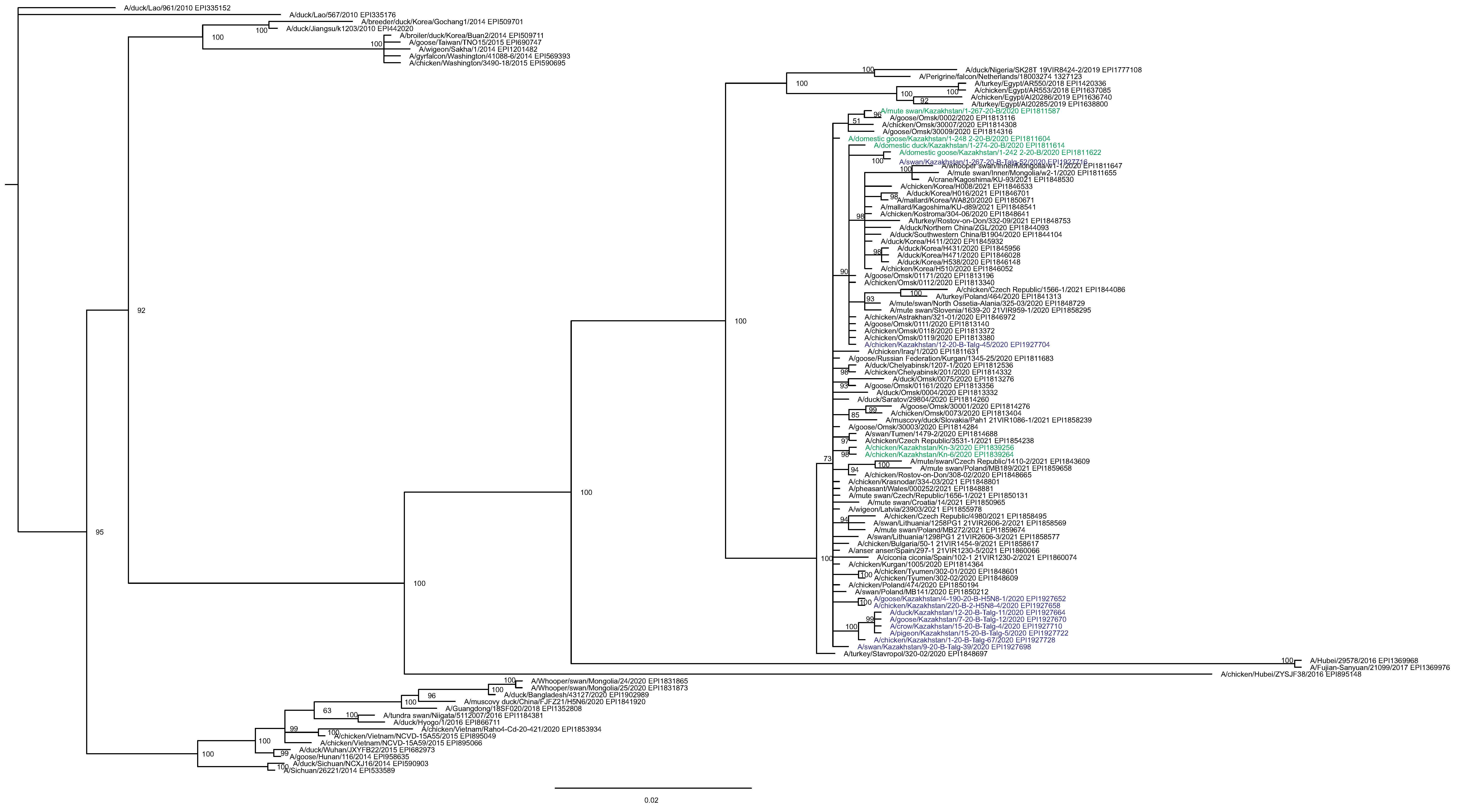

Supplement: Figure S13 [file peerj-10-13038-s014.png]

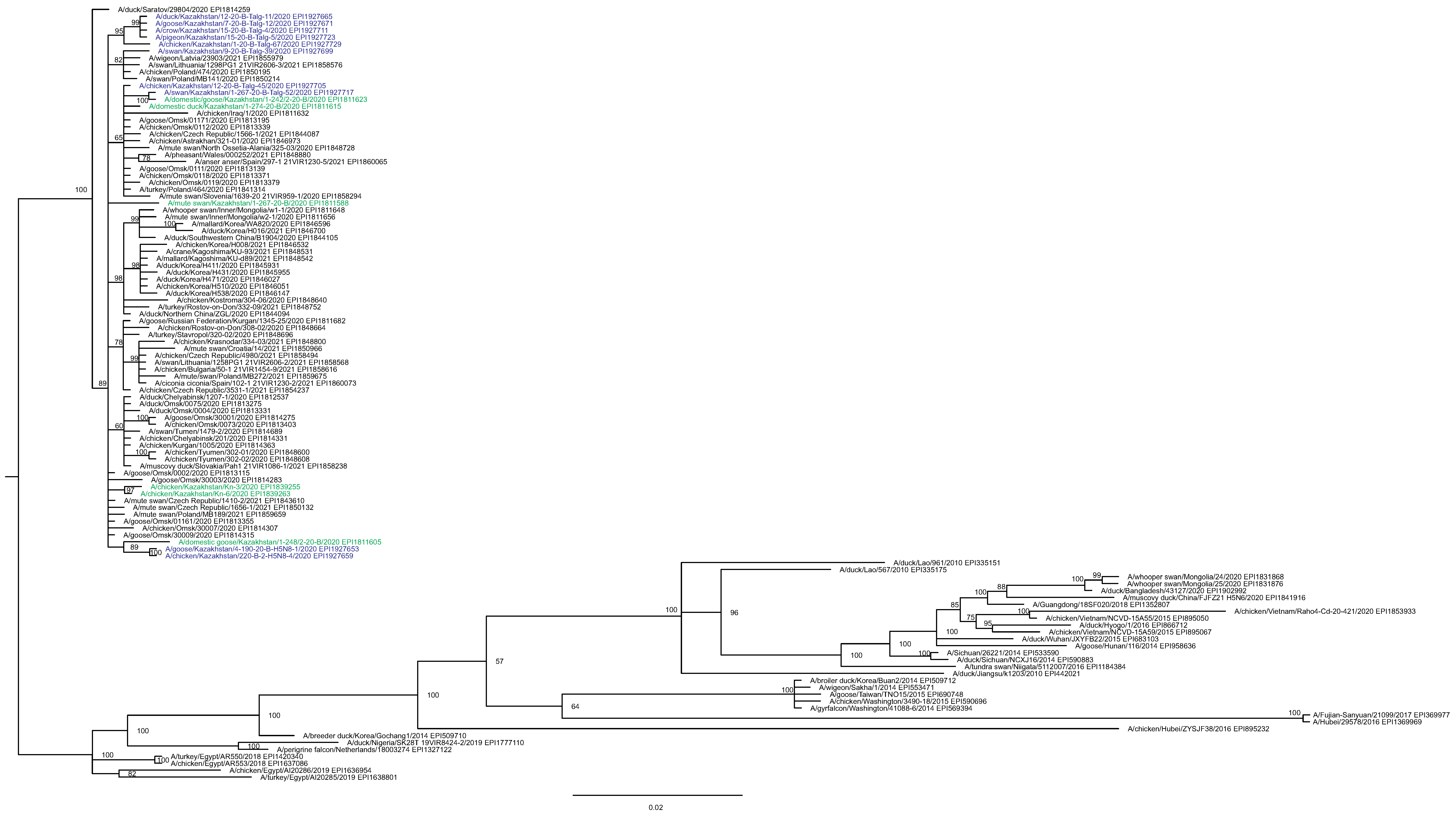

Supplement: Figure S14 [file peerj-10-13038-s015.png]
